# Training Mid-Level Providers to Treat Severe Non-Communicable Diseases in Neno, Malawi through PEN-Plus Strategies

**DOI:** 10.5334/aogh.3750

**Published:** 2022-08-11

**Authors:** Todd Ruderman, Evelyn Chibwe, Chantelle Boudreaux, Enoch Ndarama, Emily B. Wroe, Emilia Connolly, Gene Bukhman

**Affiliations:** 1 Partners In Health, Neno, Malawi; 2 Center for Integration Science, Division of Global Health Equity, Brigham & Women’s Hospital, Boston, MA, USA; 3 Ministry of Health, Neno, Malawi; 4 NCD Synergies Project, Partners In Health, Boston, MA, USA; 5 Program in Global NCDs and Social Change, Department of Global Health and Social Medicine, Harvard Medical School, Boston, MA, USA; 6 Division of Pediatrics, University of Cincinnati College of Medicine, Cincinnati, OH, USA; 7 Division of Hospital Medicine, Cincinnati Children’s Hospital Medical Center, Cincinnati, OH, USA

**Keywords:** NCDs, task shifting, mid-level providers, implementation research, type 1 diabetes, rheumatic heart disease, heart failure, sickle cell disease

## Abstract

**Background:**

Non-communicable diseases (NCDs) are a leading cause of worldwide morbidity and mortality, yet access to care in lower-income countries is limited. Rural communities, where poverty levels are high, feel the greatest burden. In Malawi, as elsewhere in the African region, it is particularly challenging for patients in rural districts to obtain care for locally endemic and severe NCDs such as type 1 diabetes, rheumatic heart disease, and sickle cell disease. The Package of Essential NCD Interventions – Plus (PEN-Plus) is a strategy to decentralize care for these severe conditions by enabling local clinicians at intermediate-care facilities to provide services otherwise available only through specialty clinics at central hospitals.

**Objectives:**

The primary objective of this study was to evaluate the impact of training mid-level providers to treat severe and chronic NCDs in newly established PEN-Plus clinics in Neno, Malawi.

**Methods:**

Our team developed a logic model to describe the anticipated impacts of the intervention on provider knowledge, patient recruitment, and care provision. We applied a retrospective review of routinely collected clinical and administrative data to assess changes along these hypothesized pathways.

**Findings:**

Didactic trainings improved provider test scores immediately following training (25-point improvement; p < 0.01), with demonstrated retention of knowledge after 6 months (21-point improvement, p < 0.01). Over 350 patients were enrolled in the first 18 months of program initiation. The PEN-Plus clinic led to significant improvement in the provision of medications and testing across a range of services.

**Conclusion:**

Mid-level providers can be successfully trained to treat severe NCDs with physician-guided education, mentorship, and supervision. The PEN-Plus clinic improved patient enrollment, the quality of clinical care and access to essential medications and laboratory supplies. These lessons learned can guide decentralization of NCD care to district hospitals in Malawi and expansion of PEN-Plus services in the African region.

## Introduction

Noncommunicable diseases (NCDs) are the leading cause of death worldwide, accounting for 71% of mortality [1]. It is a common misconception that NCDs are primarily a problem for high-income countries and limited to older, richer, urban populations in low-income countries (LICs). Eighty-five percent of NCD deaths between 30–69 years old occur in low- and middle-income countries (LMICs) [1] with a significant burden in younger and rural populations [2]. Despite the high burden of disease, NCDs continue to be severely underrecognized and underfunded [3]. For example, in Malawi, a LIC country in southeastern Africa, funding for NCDs and Injuries accounted for 15.8% of the total health expenditure during the 2014–2015 fiscal year, yet the corresponding disease burden – measured in Disability-Adjusted Life years (DALYs) – was 31.3% [4].

Chronic NCDs and their risk factors are major public health problems in Malawi. A 2017 national survey of adults aged 25–64 found that 19.3% have elevated blood pressure and 1.4% have elevated fasting blood glucose [5]. An increasing burden of obesity, tobacco and alcohol consumption in urban areas and the better-off in Malawi has led to higher rates of cardiovascular disease and diabetes [6]. However, the burden of NCDs in rural settings is significant, and is due to a more heterogeneous set of causes and risk factor. In 2015, 79% of DALYs from NCDs in Malawi were not explained by metabolic or behavioral risk factors [4].

Poverty is also a risk factor affecting NCDs and one of the greatest barriers in providing NCD care in low-income countries [7]. As of 2016, 51.6% of the population of Malawi lived below the international poverty line [8]. For these populations, NCDs can result in catastrophic costs in addition to death and suffering [9].

Countries, such as Rwanda have shown how to decentralize integrated care for less common, but more severe, chronic NCDs such as type 1 diabetes and rheumatic heart disease [10,11]. In 2019, the WHO Regional Office for Africa hosted a regional consultation to discuss Package of Essential NCD Interventions – Plus (PEN-Plus), a strategy to accelerate WHO PEN implementation, and to promote integrated care for severe chronic NCDs at intermediate-care facilities (such as district hospitals) throughout the African region [12]. PEN-Plus was also identified as a promising integrated delivery model by the Lancet NCDI Poverty Commission, and has been promoted as a key initiative of the NCDI Poverty Network working to support implementation of the Commission’s recommendations [3].

A barrier to high-quality, decentralized NCD care in Malawi is the lack of health care professionals trained in treating NCDs. Limited health care professionals in Malawi makes it a challenge to provide basic health services to the population [13]. To combat the lack of physicians, many lower-income countries are task shifting treatment of hypertension, diabetes, and asthma to nurses or clinical officers (often referred to collectively as mid-level providers). This strategy has been found to be successful in bridging this human resources gap [14,15]. In 2018, Partners In Health, a non-governmental organization, began working with the Malawi Ministry of Health to train clinical officers in Neno, Malawi to provide chronic care for patients with severe and chronic NCDs at the district and community hospitals.

The aim of this paper is to describe the implementation of this new model of care, with a particular emphasis on the early experiences, including capacity building, resource considerations, and the lessons learned that may help to inform programmatic efforts across the region.

## Methods

### Design

**Figure 1:**
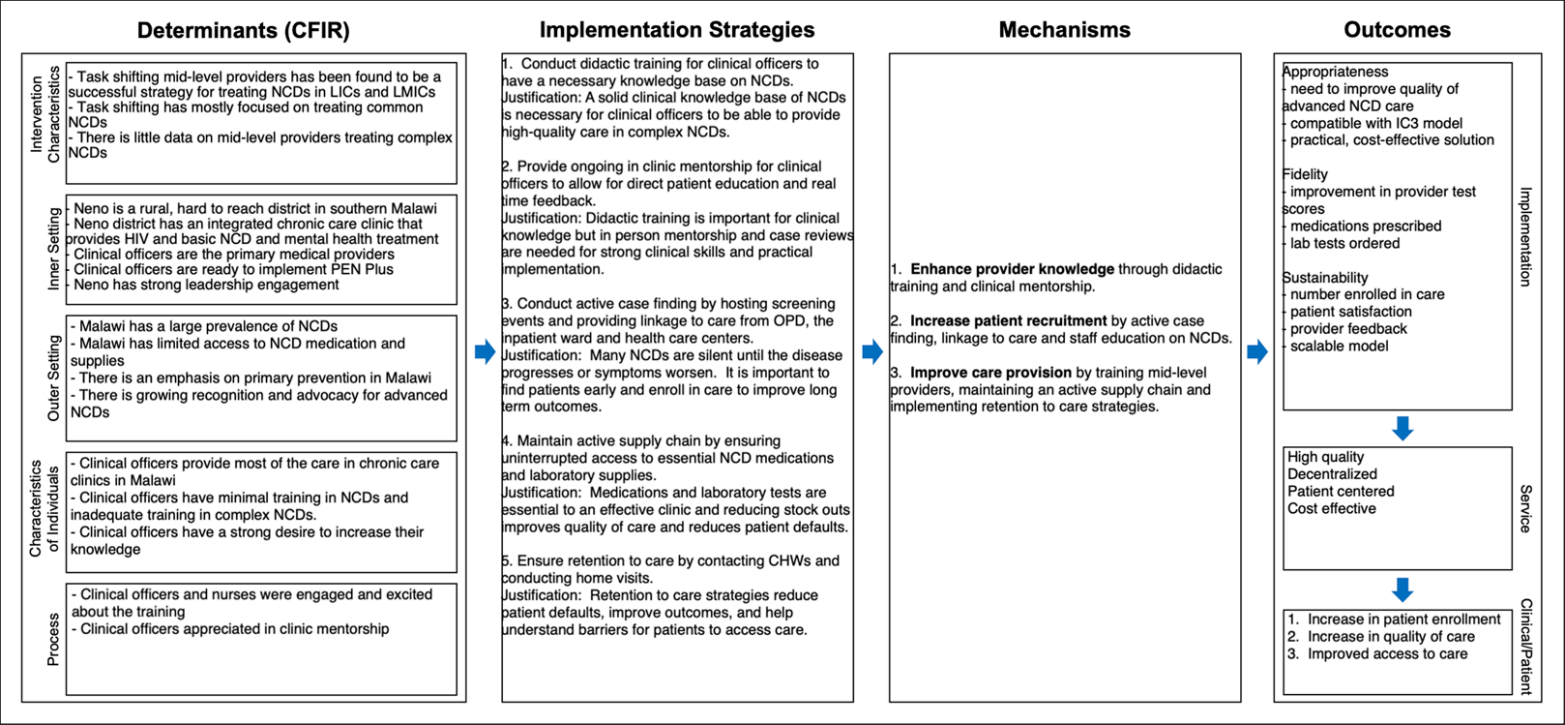
Theory of change, highlighting three mechanisms of action.

This study is a retrospective review of routinely collected clinical and administrative data. Using the Implementation Research Logic Model [16], our team developed a logic model to structure our review (Figure 1). Using available data, we focus on three mechanisms (provider knowledge, patient recruitment, and care provision).

### Local Setting

Neno is a rural district of 138 291 people, located in the southern region of Malawi [17]. The majority of people rely on subsistence farming and less than five percent of the population has electricity [18]. Poor transportation infrastructure and road maintenance makes it difficult for patients to access hospitals and patients typically travel long distances on foot to reach health care centers. Neno’s local health system is mostly public and is comprised of a district hospital in the mountainous western region, a community hospital in the lower, eastern region and 12 health care centers.

It is often difficult for patients in rural districts in Malawi, such as Neno, to obtain adequate care for complex NCDs. Specialized clinics for insulin-dependent diabetes, sickle cell disease and chronic cardiac failure only exist at the 4 central hospitals. Travel to a central hospital from Neno takes hours and is unaffordable for most patients. At the district level, midlevel providers, including clinical officers and nurses, provide most of the treatment at chronic care clinics. However, they are widely not trained or given resources to provide care for complex NCDs and minimal physician support is available.

### The MOH/PIH IC3 and PEN-PLUS Program

Partners In Health (PIH), a non-government organization, has partnered with the Ministry of Health (MOH) in Neno since 2007. PIH combines accompaniment and direct service provision with a goal of overall health systems building. Major areas of support include HIV care, maternal health, community health and non-communicable diseases.

Since 2009, the MOH and PIH have worked to strengthen care for NCDs in the district, first with the introduction of Chronic Care Clinics (CCC) treating patients diagnosed with a variety of NCDs at the 2 hospitals, and then through the Integrated Chronic Care Clinics (IC3). IC3 clinics were decentralized and led by mid-level providers and provide outpatient services for HIV and non-communicable chronic conditions at weekly clinics located at health centers across the district [19,20]. As part of this effort, the team has worked to address gaps in staffing, equipment and medicines.

### Approach

Partners In Health, in conjunction with the MOH, planned on opening two PEN-Plus clinics in October 2018. Weekly clinics were scheduled at Neno District Hospital and Lisungwi Community Hospital. These clinics would provide care for severe NCDs while the Integrated Chronic Care Clinics (IC3) would maintain services for basic NCDs included in the PEN package such as hypertension, type 2 diabetes, chronic respiratory disease, epilepsy, basic mental health conditions, and cervical cancer screening.

In anticipation of opening the clinics, PIH held a series of NCD trainings for clinicians and nurses in Neno District from August 2018 to February 2019. All PIH and MOH clinical officers and nurses in the district were invited to attend the trainings to improve case finding and district-wide treatment of NCDs. However, an essential purpose of the trainings was to prepare the midlevel providers who would work in the PEN-Plus clinics. A total of 4 sessions over 7 days were given in diabetes, cardiovascular disease, pulmonary and renal disease, and gastrointestinal, neurologic and hematologic disease. Trainings were a mix of clinical lectures, case studies and strategies on counseling patients. Materials were developed by internal medicine, cardiology, and pulmonary specialists at an appropriate level for mid-level providers.

Didactic PEN-Plus trainings were supplemented with in-clinic mentorship. At the PEN-Plus clinic, a physician served as a clinical mentor for the NCD clinical officers, and a nurse mentor supervised the NCD nurses in patient care and counseling. The mentors would see patients with staff in the clinic which provided the opportunity for onsite, real-time questions and feedback. Physician led mentorship started weekly, but gradually decreased to bi-weekly and monthly as the clinical officers became more proficient over the first year of the clinic.

The essential staff hired for each clinic include a clinical officer, nurse, clerk, and pharmacy assistant. Stock of some vital NCD medications, such as insulin, ACE-inhibitors, and beta-blockers, were supplemented in IC3 prior to establishment of PEN-Plus clinics, meaning that changes in medication distribution are not attributed to increased availability.

### Data and Analysis

Clinical knowledge was assessed using structured 40-point questionnaires given at 3 points: immediately prior to training, immediately following the initial training, and 6 months post training. Tests were separately developed for each daily NCD session and included an assessment of epidemiology, diagnosis, management, medication, and counseling. We conducted paired t-tests to assess for differences in scores across the 3 time points.

To investigate the care given at the PEN-Plus clinic since inception, we examined process outcomes for selected advanced NCDs. Using electronic medical records, we gathered data on the proportion of patients receiving medical tests and appropriate medications between January 2017 and January 2020 – a period covering 18 months before and after the clinic was established. For all eligible visits, we collected patient-level (age, gender, HIV-comorbidity) and visit-level (lab tests, medication, symptoms) data from the clinic’s routine data system. For the purposes of this work, we focused on insulin-dependent diabetes, chronic kidney disease and chronic heart failure as they are among the most common conditions seen at the PEN-Plus clinics.

Data collected prior to the PEN-Plus clinic was collected from the IC3 clinic in Neno with both clinics using the same protocols for the outcomes considered. Hence, any increase in the distribution of testing or medication use is expected to be attributed to the training, mentorship, dedicated staff, and clinic for complex NCD patients, rather than systematic differences in the patient population. Chi-square tests, univariate, and multivariable logistic regression were used to assess for differences in the provision of tracer drugs between the groups. Multivariable models control for patient-level characteristics (age, gender, and HIV-comorbidity) that are hypothesized to affect the care [21]. To ease interpretability of our results, we use the “margins” command to translate these results into predicted probabilities of correct clinical care with and without training. All analyses were conducted using Stata version 15 [22].

### NCD Clinical Guidelines

Diabetes and chronic kidney disease analysis focused on lab testing per recommendations. Clinical guidelines for patients with insulin-dependent diabetes recommend blood sugar testing during each clinic visit and Hemoglobin A1C (HbA1C) testing once per 6 months. Patients with chronic kidney disease are recommended to receive a urine protein level every 3 months and a blood creatinine level every 6 months.

Chronic heart failure review looked at appropriate medication use. Patients with a diagnosis of New York Heart Association (NYHA) class II or greater are recommended to be on an ACE Inhibitor and Beta Blocker. Additionally, patients with a diagnosis of chronic heart failure and symptoms of shortness of breath, lower extremity edema or fluid overload are recommended to be on furosemide.

### Ethics

This study was reviewed and approved by the Neno District Health Research Committee. The study falls under the PIH umbrella IRB with the Malawi National Health Sciences Research Committee (NHSRC) as regularly collected patient data was used.

## Results

### Provider Knowledge

To assess improvement and retention of clinical knowledge, providers took a pretest at the start of trainings, a posttest immediately after trainings and a 6-month follow-up test. The average pretest score was 63.0%, the average post test score was 88.3% and the average 6-month follow up test score was 84.4%. These test score findings show improvement of clinical knowledge after trainings (p < 0.01) as well as retention of knowledge 6 months after the course (p < 0.01).

### Patient Recruitment

By April 2020, 28 months after the clinic opened, the PEN-Plus clinics in Neno had approximately 350 patients enrolled. Some of these patients were transferred from IC3 clinics while others were diagnosed in the hospital or through active case finding projects. A wide range of diseases were seen in the clinic (Table 1). In addition, the clinic has diagnosed rare diseases, such amyotrophic lateral sclerosis, muscular dystrophy and polyarticular juvenile idiopathic arthritis.

**Table 1:** List of severe NCDs treated at PEN-Plus clinic in Neno, Malawi.


CONDITIONS TREATED	

Cardiovascular	Rheumatic Heart Disease; Heart Failure; complicated Hypertension; Congenital heart disease; Stroke; Deep Venous Thrombosis, Pulmonary Embolism, Myocardial Infarction

Renal	Chronic kidney disease

Gastrointestinal	Liver cirrhosis

Endocrine	Type 1 DM Type 2 DM on insulin

Pulmonary	Severe chronic respiratory disease

Hematology	Sickle cell disease

Neurology	Severe epilepsy


### Care Provision

**Figure 2:**
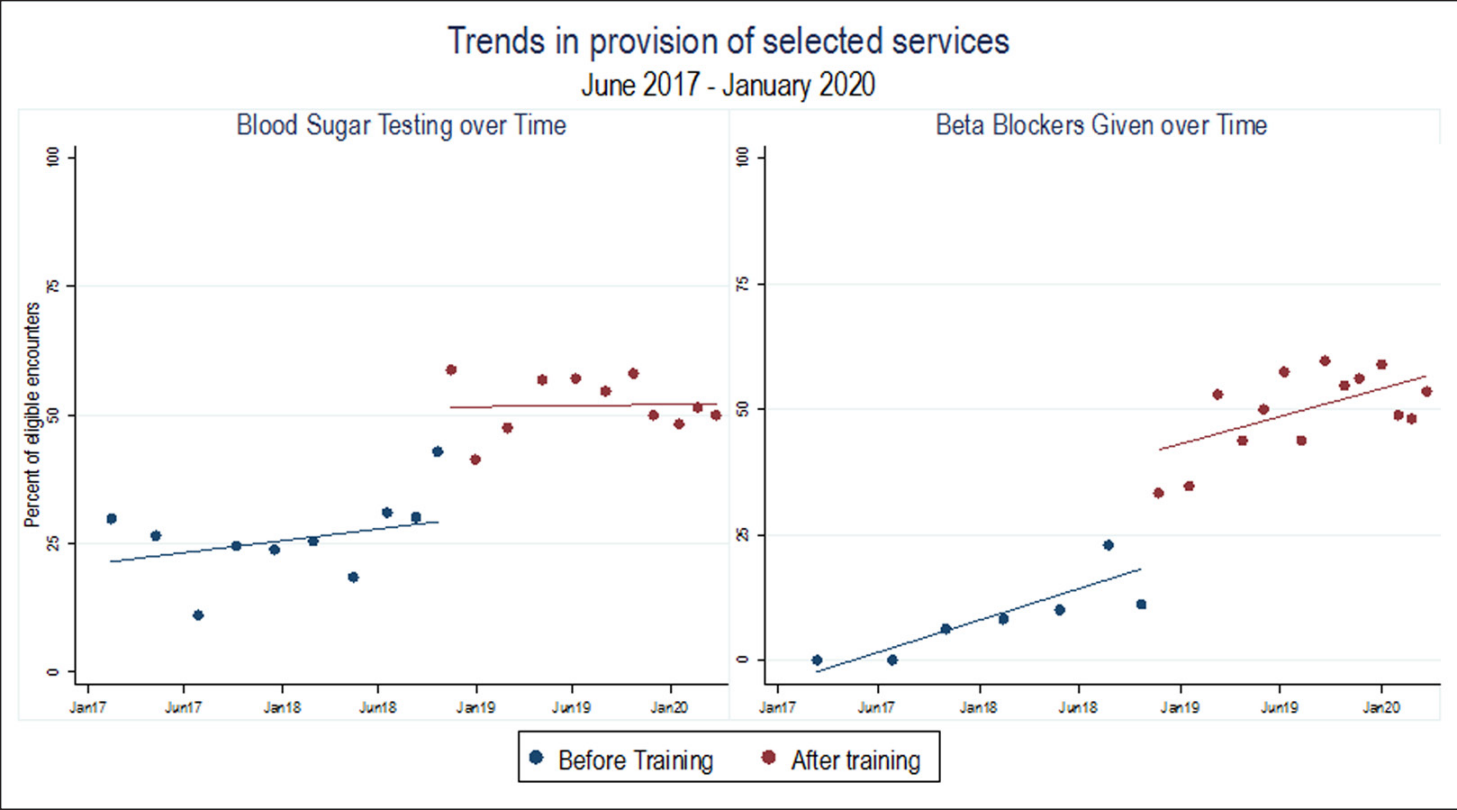
Improvements in Care Provision: Distribution of selected medical tests and medication for NCDs, before and after the launch of the advanced NCD clinic. This figure shows temporal trends in care provision to highlight the increase at the time of the training. On the right is blood sugar testing and on the left is provision of beta blockers. Data indicates the proportion of eligible encounters in which the services was provided for the period January 2017–January 2020. Dates before the initiation of the training are indicated in blue. Dates after the initiation of training are in red. Trend lines before and after the training are also shown.

Data suggest improvement in the provision of medicines and tests across a range of services; baseline coverage rates for several of these can be found in Table 2. Figure 2 focuses on 2 of these services, and presents temporal trends in testing for blood sugar and provision of beta blockers between January 2017 and January 2020. These graphs show that, while clinical care was improving before the launch of PEN-Plus, there is a jump in the proportion of patients receiving these services that aligns with the timing of the training.

**Table 2:** Improvements in care for type 1 diabetes, kidney disease, and heart disease following training.


	TYPE 1 DIABETES		KIDNEY DISEASE		HEART DISEASE		
		
	HBA1C DRAWN	BLOOD SUGAR DRAWN	CREATININE DOCUMENTED	URINE PROTEIN DOCUMENTED	ENALAPRIL GIVEN	BETA BLOCKER GIVEN	FUROSEMIDE GIVEN

Average Baseline Coverage (Jan 2017–Oct 2020)	0.04	0.24	0.08	0.05	0.28	0.08	0.09

Predicted increase in coverage after the launch of PEN-Plus	0.03***	0.22***	0.18***	0.33***	0.24***	0.41***	0.26***

	(0.01)	(0.02)	(0.03)	(0.03)	(0.03)	(0.03)	(0.02)


Standard errors are in parentheses. * p < 0.05, ** p < 0.01, *** p < 0.001. This table presents the predicted increase in probability that a clinical service will be provided at an eligible visit. For example, 24% of patients with type 1 diabetes had their blood sugar drawn at an eligible visits at baseline. The training is associated with a 22% point increase in the predicted probabiliy that this service would occur at a visit. Results reflect the results for the mean patient after a multivariable logistic regression models that control for patient age, HIV status, and gender (results from these underlying models can be found in the supplementary materials).

We additionally modeled this relationship using simple and multivariable logistic regression. Multivariable models control for a number of patient-level factors – sex, age, and HIV comorbidity – that have been associated with care quality in the literature (full results are provided in the supplemental literature). To improve the interpretability of these models, we translated the resulting odds ratios into predicted probability that the mean patient would receive a given drug or test at an eligible visit (Table 2). For patients with type 1 diabetes, training is associated with a 21.4% (p < 0.01) increased probability that blood sugars completed at clinic and a 2.7% (p < 0.01) increase probability that HbA1C tests are completed. For patients with chronic kidney disease, there was an 18.2% (p < 0.01) and a 33.3% (p < 0.01) increased probability that creatinine and urine protein will be completed per protocol, respectively. For patients with chronic heart disease, there was a 23.5% (p < 0.01) increased probability that enalapril will be given, a 40.7% (p < 0.01) increase in beta-blockers, and a 26.3% (p < 0.01) increased predicted probability that furosemide will be prescribed appropriately.

## Discussion

Training mid-level providers to become proficient in treating complex NCDs, combined with the establishment of a new PEN-Plus clinic, led to improvement of service. Many factors contributed to this success. A 7 day long curriculum on the diagnosis, treatment, and counseling for complex NCDs improved provider knowledge, demonstrating that didactics are important in training clinicians (Table 3). Additionally, onsite, longitudinal mentorship helped clinicians develop skills and build confidence in treating complicated patients. Our findings demonstrate that investing in human resources through training mid-level providers is one of the most effective ways to fill the gap in care for patients with severe NCDs in rural Malawi.

**Table 3:** List of didactic lecture topics by category. Pre-tests, post-tests, and case reviews were also conducted. Full materials are available upon request.


TRAINING CURRICULUM	

Diabetes Mellitus	Epidemiology and Pathology Diagnosis Medications Insulin Management Complications Foot care, diet, lifestyle

Cardiovascular	Epidemiology and Pathology Hypertension and Preeclampsia DVT, PE and anticoagulation Chronic Heart Failure Rheumatic Heart Disease Counseling in Cardiovascular Disease

Pulmonology	Epidemiology and Pathology Asthma COPD, Bronchiectasis, Cough

Renal	Epidemiology and Pathology Chronic Kidney Disease Electrolytes

Gastroenterology	Hepatitis Ascites and Cirrhosis

Hematology	Sickle cell disease

Neurology	Epilepsy


Data collected showed improvement of process outcomes with the establishment of the PEN-Plus clinic in Neno. However, the enrollment of 350 patients over the first 18 months shows that the impact was greater than improvements in medications prescribed, and lab tests performed. Most patients were enrolled though active case finding and linkage to care programs.

Active case finding is important for increasing clinic census and finding patients with disease in the community [23]. Previously, these patients were undiagnosed in the community or seen in IC3 where clinicians did not have adequate training to treat their disease. For example, patients with type 1 diabetes need close blood sugar monitoring, titration of insulin and continuous diabetic education. Linkage to care is important in ensuring that patients diagnosed in the outpatient department, inpatient ward and other clinical departments are appropriately enrolled into the NCD clinic. Districts without PEN-Plus or strong NCD clinics are susceptible to losing patients to follow up and providing suboptimal care. The process indicators indicate that combination of didactic training and mentorship quickly and effectively improved the quality of clinical care in Neno District. More studies are necessary to evaluate whether these efforts translate into improved clinical outcomes and patient satisfaction in PEN-Plus clinics.

There are many challenges with developing advanced NCD clinics in rural, low-income settings, such as Neno District. It is important to note that the efforts described here took place in the context of a broader health systems strengthening efforts. Human resource shortages are common in rural districts and clinicians often have many responsibilities. Our prior experience found that, without dedicated time, clinicians struggle to run specialty clinics like PEN-Plus clinics. To respond to this, a clinical officer, nurse, and clerk were hired for each clinic. Early experience also highlighted the outsized impact of supply chain issues on NCD clinics. In the early stages of implementation, NCD drugs were regularly out of stock. These gaps in supply contributed to high rates of loss to follow up of patients. Thus, efforts to improve supply chains were an early component of our work, and medicine supplies were well established prior to the launch of the training programs discussed here.

The COVID-19 pandemic was a new challenge that affected NCD services. During March, April, and May of 2020, patient visits were spaced out and labs were drawn less frequently to reduce patient contact. Maintaining high quality care was difficult during this time. However, the pandemic has highlighted the need for decentralized severe NCD services. Traveling to cities was more challenging during the pandemic and many patients from rural districts had difficulty accessing care. Further, patients with chronic diseases have worse outcomes from COVID-19 which emphasizes the importance of ensuring all patients have access to quality care [24].

The WHO supported Package of NCDs (PEN) program has led to significant improvement in access to care for hypertension, type 2 diabetes, chronic respiratory disease and basic breast and cervical cancer screening. To provide decentralized access to PEN services, many LICs and LMICs are empowering mid-level providers, either nurses or clinical officers, to provide this care [14,15,25,26,27,28]. Subsequently, most of the literature around task shifting of NCD care to mid-level providers focuses on primary prevention and PEN services. While this work is extremely important, a gap remains in management and treatment of more severe NCDs at first level hospitals. Task shifting care to mid-level providers for complex NCDs is a feasible [11] and cost effective [29] way to provide care at first level hospitals. However, there is little literature on providing care for complex NCDs in rural, remote, and poverty-stricken regions of LICs and LMICs. This paper demonstrates that the PEN-Plus model of training mid-level providers is an effective strategy to meet this demand.

One limitation of this study is that it did not assess clinical outcomes of patients in the PEN-Plus clinic. While we document improvement of clinical knowledge and practices, we were unable to assess changes in patient morbidity and mortality. Nonetheless, it is reasonable to extrapolate that the didactics and mentorship, improvement of provider knowledge and increase in lab tests and medications given will ultimately lead to better patient outcomes. Work is ongoing to evaluate clinical outcomes.

This study looked at only two PEN-Plus clinics in Neno district in Malawi. Analysis of additional clinics in multiple districts throughout Malawi would lead to a more robust understanding of mid-level provider led treatment of complex NCDs. As Malawi works to expand PEN Plus services to other rural districts, future analysis should look at a wider range of clinics [30].

Our experience shows that with physician-guided education, mentorship and supervision, mid-level providers can be successfully trained to improve NCD care in rural districts with human resource and medication support. NCDs are globally underfunded, and that burden is felt greatest in countries with high levels of poverty. We hope that the lessons learned from establishing the PEN-Plus clinics in Neno can be applied towards advocacy and funding for creation of similar clinics in other districts and improving NCD treatment throughout Malawi and Sub-Saharan Africa [12].

## Additional Files

The additional files for this article can be found as follows:

10.5334/aogh.3750.s1 Supplementary Material. Supplemental Tables 1 and 2.

10.5334/aogh.3750.s2 Didactic Materials. The supplementary materials contain a suggested didactic training schedule and the PowerPoint presentations used for PEN-Plus training in Neno, Malawi. These materials have been reviewed and accepted by the Malawi Ministry of Health for future PEN-Plus trainings in Malawi.
