# Supplementary material for: Training Mid-Level Providers to Treat Severe Non-Communicable Diseases in Neno, Malawi through PEN-Plus Strategies
Source: Ann Glob Health. 2022 Aug 11;88(1):69. doi: 10.5334/aogh.3750 (PMC9389951; doi:10.5334/aogh.3750)
Supplement: Supplementary Material. — Supplemental Tables 1 and 2. [file agh-88-1-3750-s1.pdf]

Supplementary Material

	Type 1 Diabetes		Kidney Disease		Heart Disease		
	HbA1c Drawn	Blood Sugar Drawn	Creatinine documented	Urine protein documented	Enalapril Given	Beta Blocker Given	Furosemide Given
Training	1.94***	2.83***	3.99***	11.47***	2.46***	10.32***	5.90***
	(0.37)	(0.26)	(0.99)	(3.27)	(0.36)	(2.29)	(1.26)
Number of Visits	2,203	2,203	673	673	1,011	1,011	1,011

Supplemental Table 1: Correlation between training and clinical care. This table presents the results of logistic regression assessing the relationship between the training and clinical care. All results are presented as odds ratios. Standard errors in parentheses. Analyses control for patient age, HIV status, and gender. *p<0.05, **p<0.01, ***p<0.001

	Type 1 Diabetes		Kidney Disease		Heart Disease		
	HbA1c Drawn	Blood Sugar Drawn	Creatinine documented	Urine protein documented	Enalapril Given	Beta Blocker Given	Furosemide Given
Training	1.71***	2.62***	4.31***	12.16***	2.80***	11.62***	5.71***
	(0.33)	(0.25)	(1.08)	(3.52)	(0.44)	(2.65)	(1.25)
Patient Age	0.98***	0.99***	1.01**	1.01	1.02***	1.01*	1.01*
	(0.00)	(0.00)	(0.01)	(0.01)	(0.00)	(0.00)	(0.00)
PLWHIV	0.83	0.73**	0.57*	0.49**	0.49***	1.63**	0.27***
	(0.22)	(0.10)	(0.19)	(0.15)	(0.10)	(0.33)	(0.07)
Gender	0.84	0.91	1.09	0.75	1.12	1.02	0.96
	(0.15)	(0.09)	(0.24)	(0.16)	(0.16)	(0.16)	(0.15)
Number of Visits	2,203	2,203	673	673	1,011	1,011	1,011

Supplemental Table 2. Correlation between training and clinical care, controlling for patient-level variables. This table presents the results of a multivariable logistic regression assessing the relationship between the training and clinical care, controlling for patient age, HIV status, and gender. All results are presented as adjusted odds ratios. Standard errors in parentheses. *p<0.05, **p<0.01, ***p<0.001
