# Supplementary material for: Training Mid-Level Providers to Treat Severe Non-Communicable Diseases in Neno, Malawi through PEN-Plus Strategies
Source: Ann Glob Health. 2022 Aug 11;88(1):69. doi: 10.5334/aogh.3750 (PMC9389951; doi:10.5334/aogh.3750)
Supplement: Didactic Materials. — The supplementary materials contain a suggested didactic training schedule and the PowerPoint presentations used for PEN-Plus training in Neno, Malawi. These materials have been reviewed and accepted by the Malawi Ministry of Health for future PEN-Plus trainings in Malawi. [file agh-88-1-3750-s2.zip › Didactic_Materials/CV_CHF and RHD.pptx]

## Slide 1
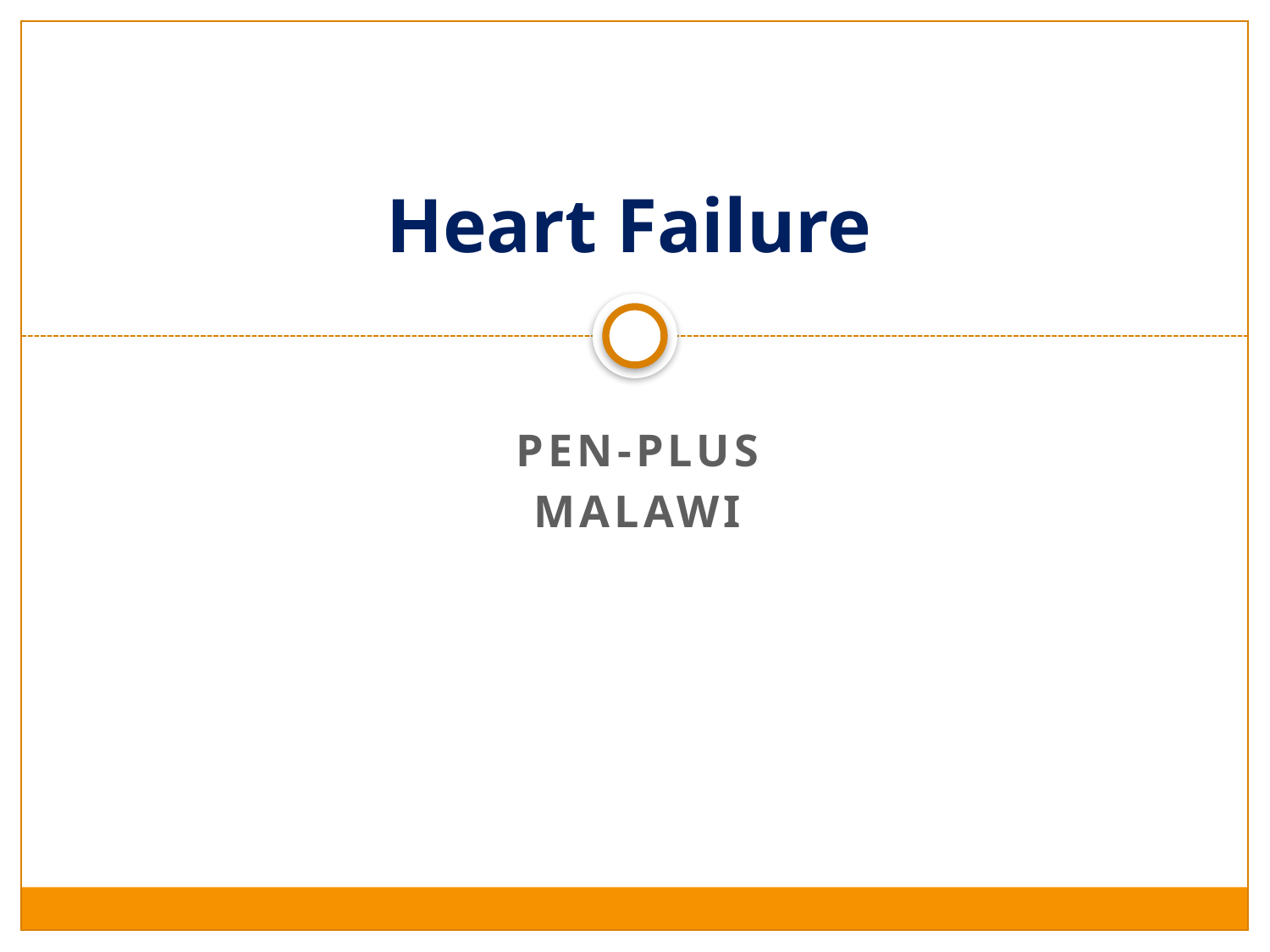

# Heart Failure
PEN-Plus
Malawi

## Slide 2
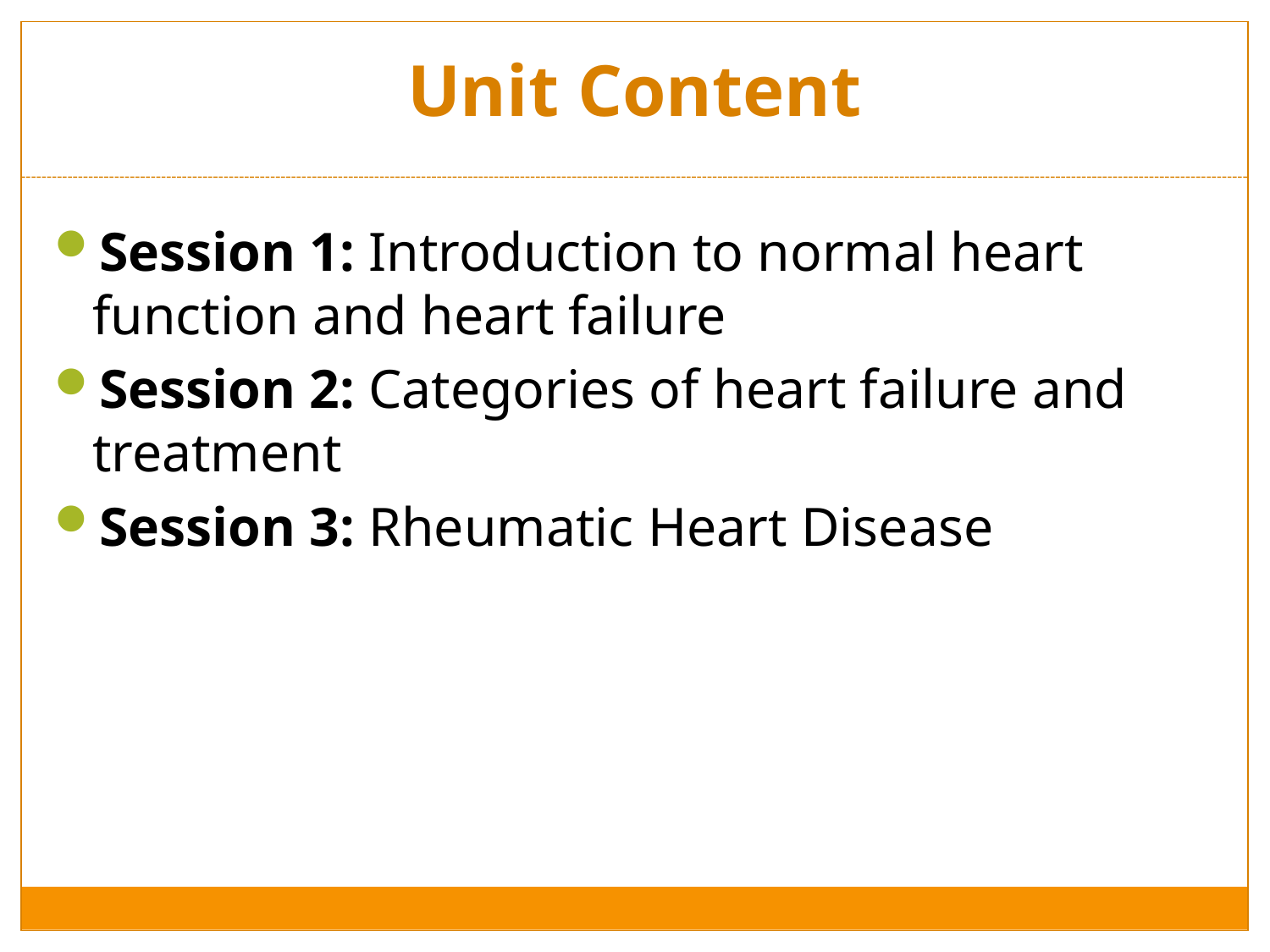

# Unit Content
Session 1: Introduction to normal heart function and heart failure
Session 2: Categories of heart failure and treatment
Session 3: Rheumatic Heart Disease

## Slide 3
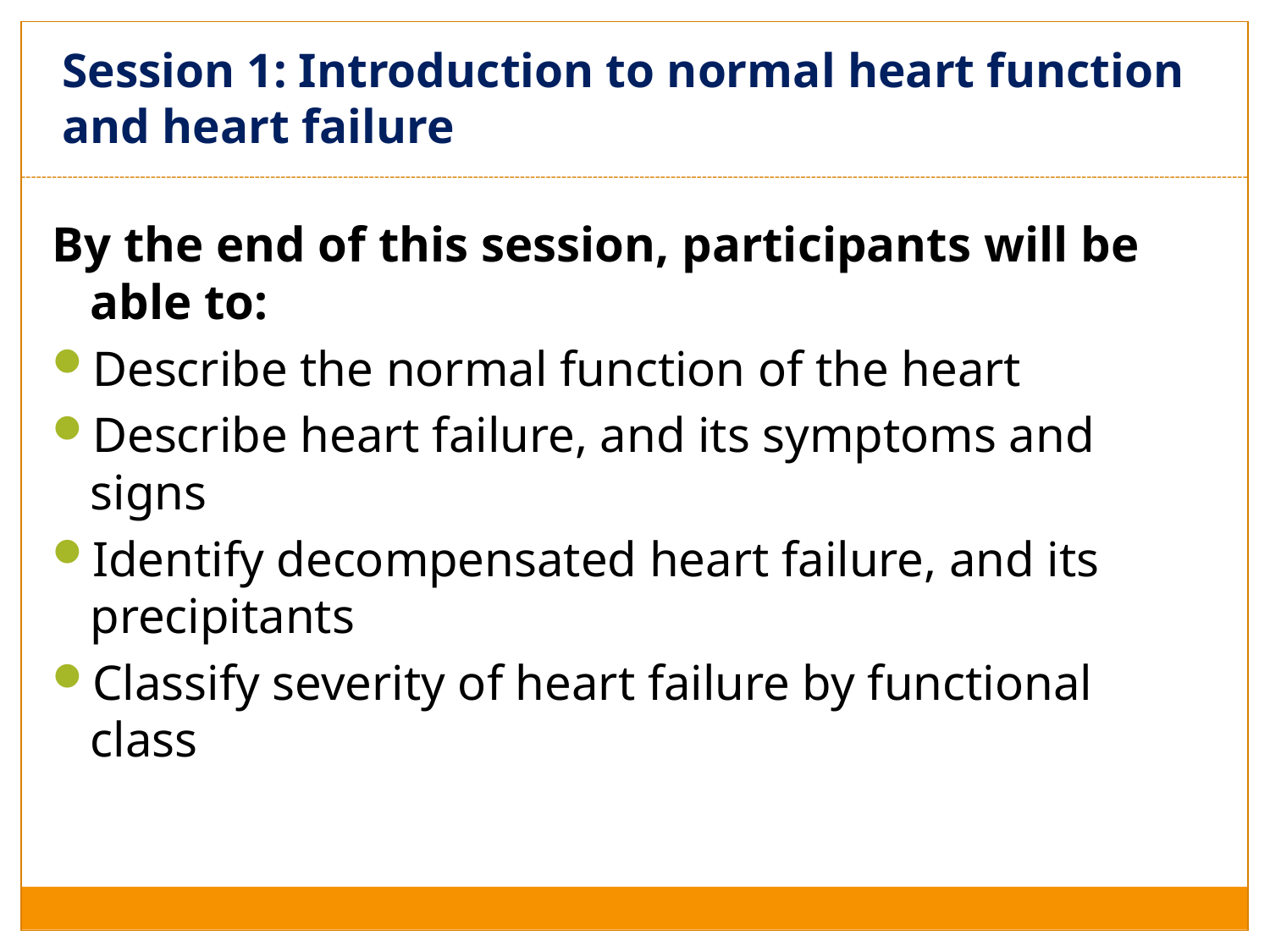

# Session 1: Introduction to normal heart function and heart failure
By the end of this session, participants will be able to:
Describe the normal function of the heart
Describe heart failure, and its symptoms and signs
Identify decompensated heart failure, and its precipitants
Classify severity of heart failure by functional class

## Slide 4
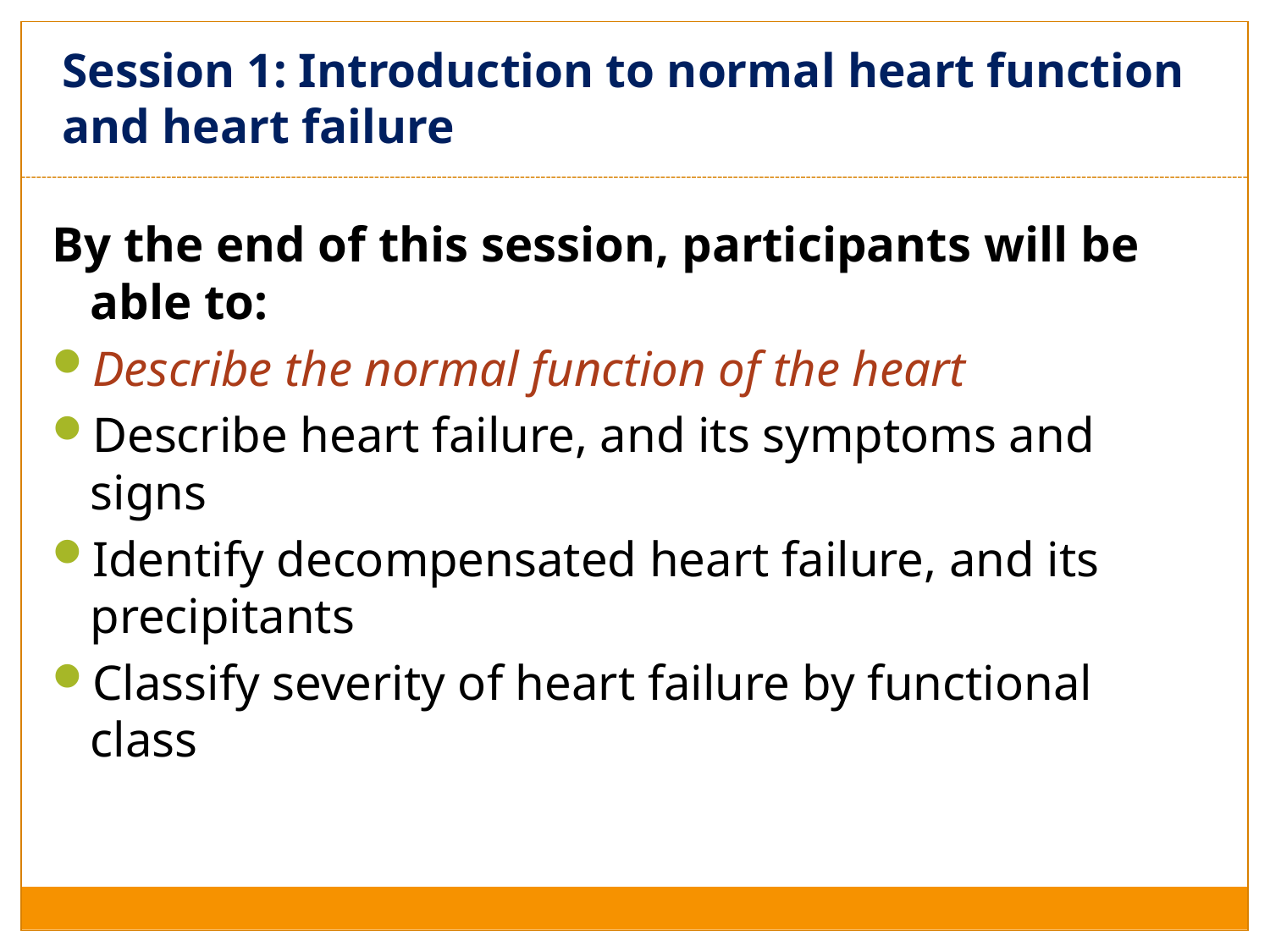

# Session 1: Introduction to normal heart function and heart failure
By the end of this session, participants will be able to:
Describe the normal function of the heart
Describe heart failure, and its symptoms and signs
Identify decompensated heart failure, and its precipitants
Classify severity of heart failure by functional class

## Slide 5
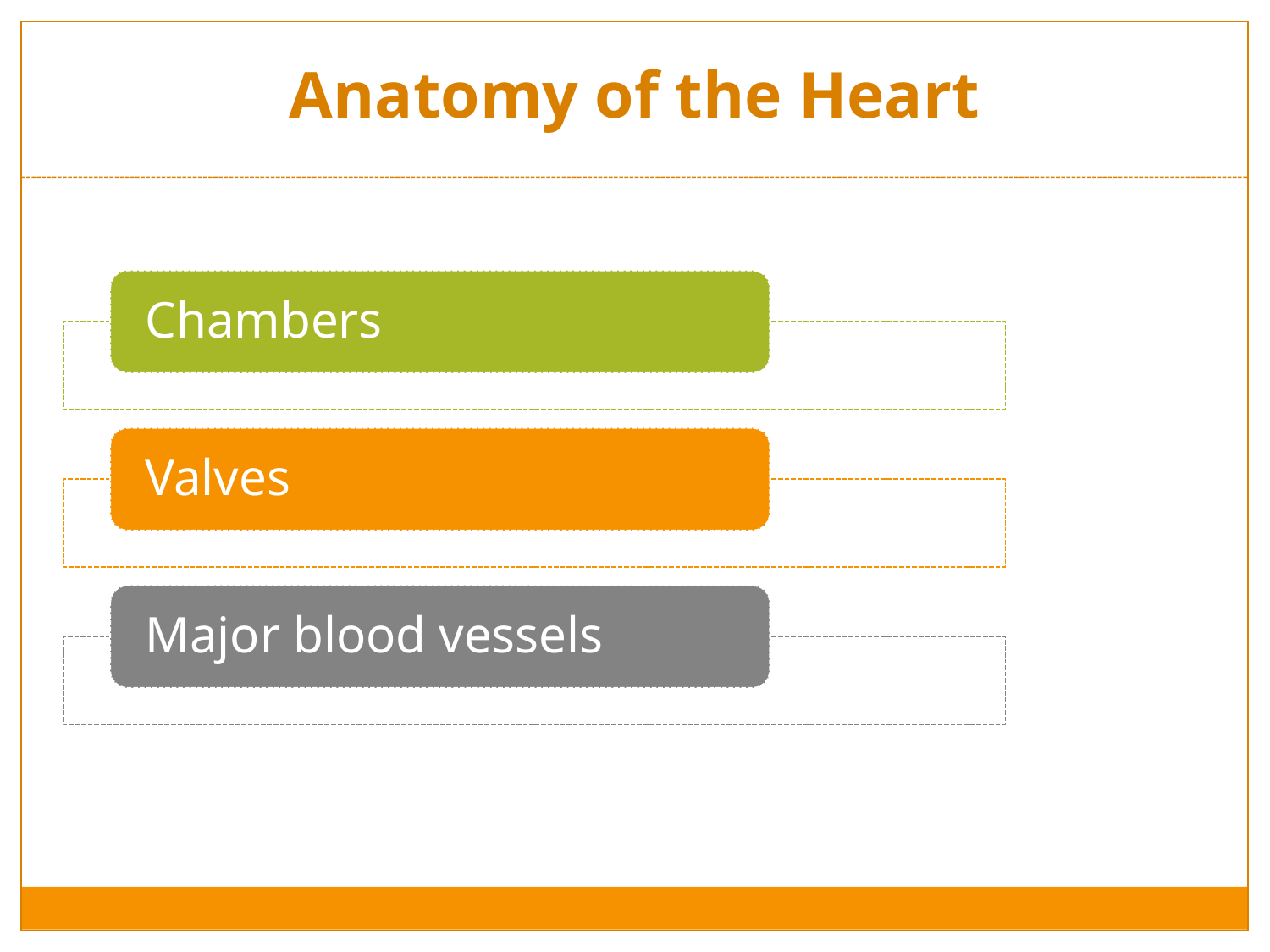

# Anatomy of the Heart

## Slide 6
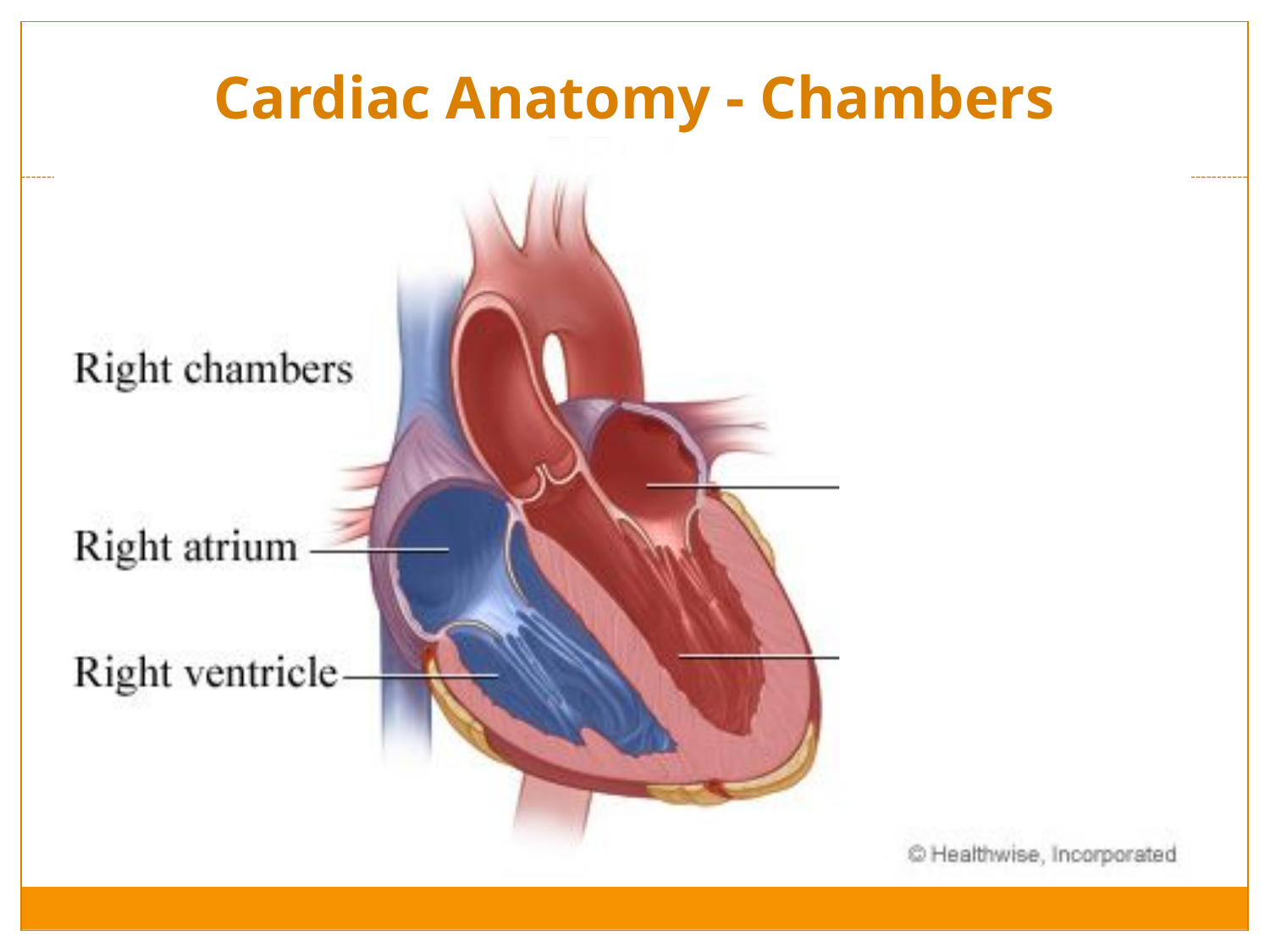

# Cardiac Anatomy - Chambers

## Slide 7
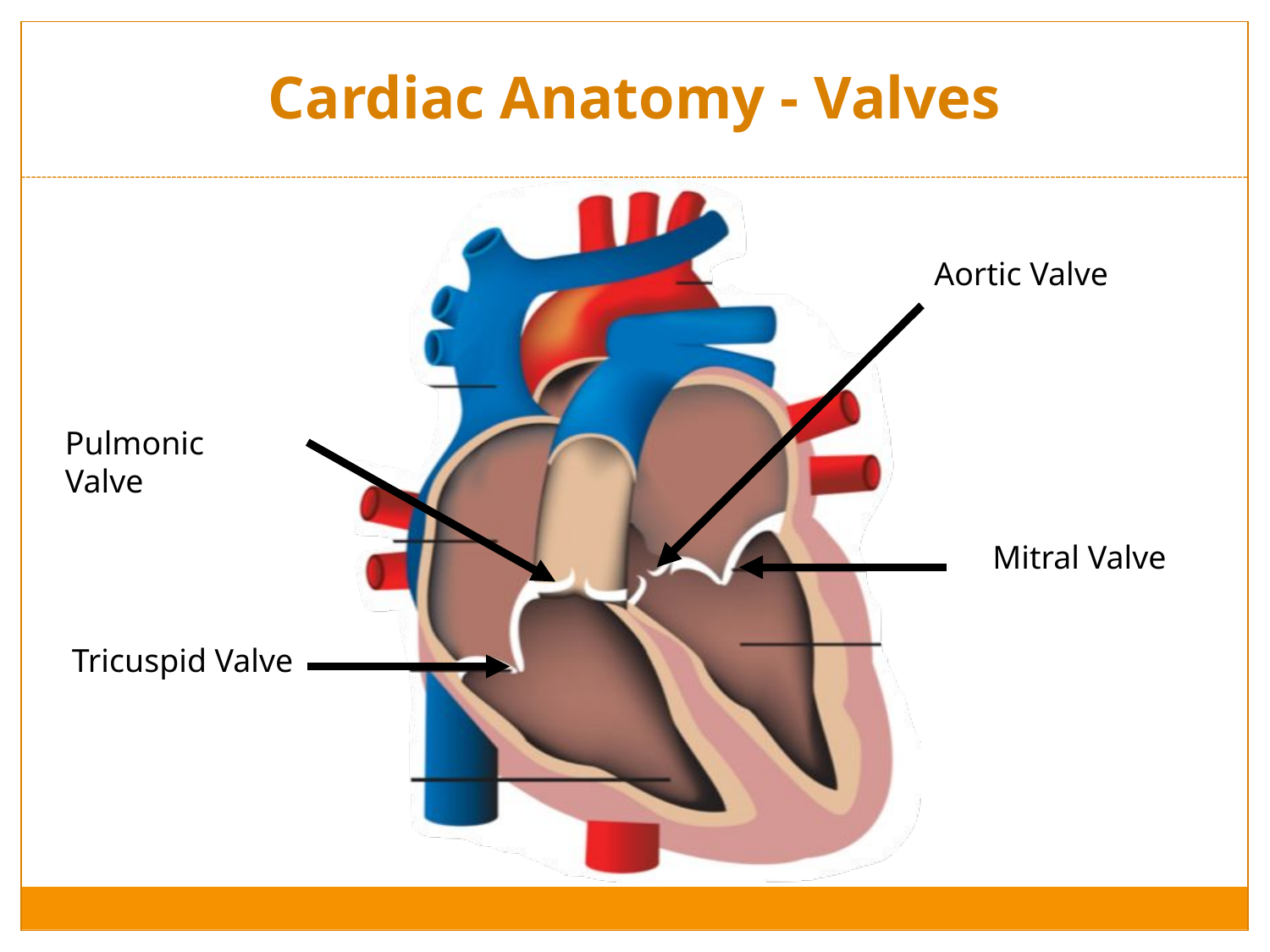

# Cardiac Anatomy - Valves
Aortic Valve
Pulmonic Valve
Mitral Valve
Tricuspid Valve

## Slide 8
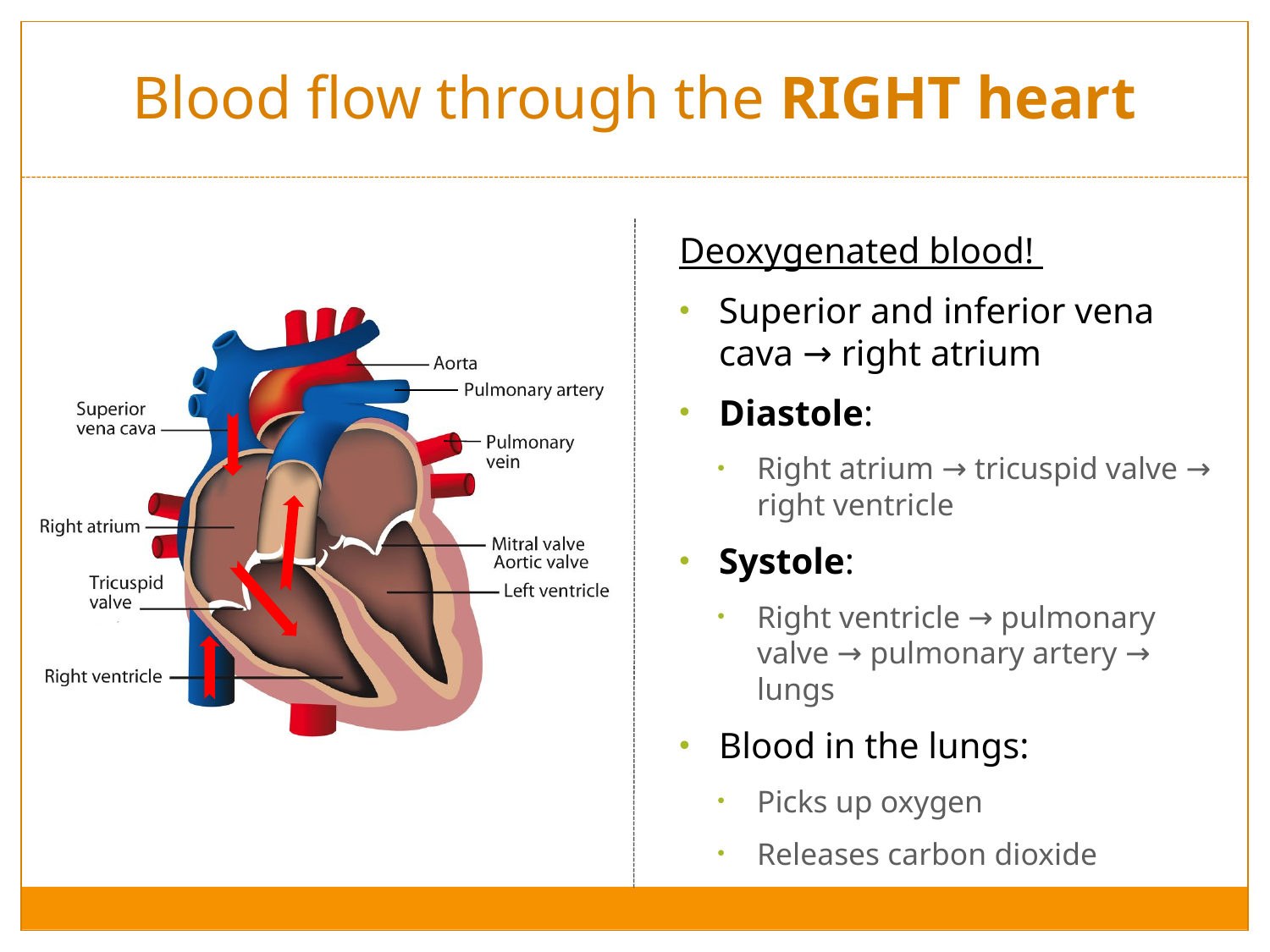

# Blood flow through the RIGHT heart
Deoxygenated blood!
Superior and inferior vena cava → right atrium
Diastole:
Right atrium → tricuspid valve → right ventricle
Systole:
Right ventricle → pulmonary valve → pulmonary artery → lungs
Blood in the lungs:
Picks up oxygen
Releases carbon dioxide

## Slide 9
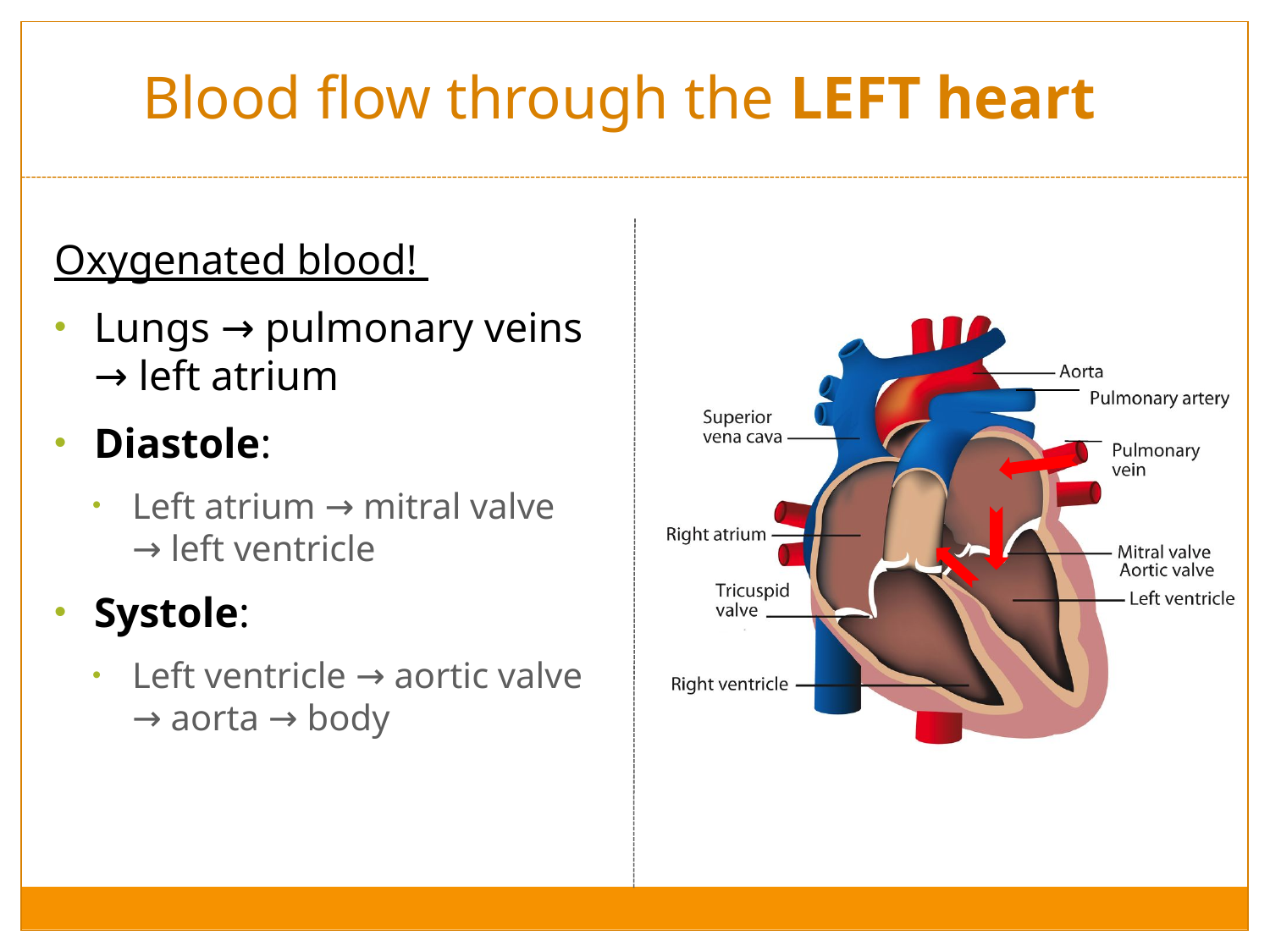

# Blood flow through the LEFT heart
Oxygenated blood!
Lungs → pulmonary veins → left atrium
Diastole:
Left atrium → mitral valve → left ventricle
Systole:
Left ventricle → aortic valve → aorta → body

## Slide 10
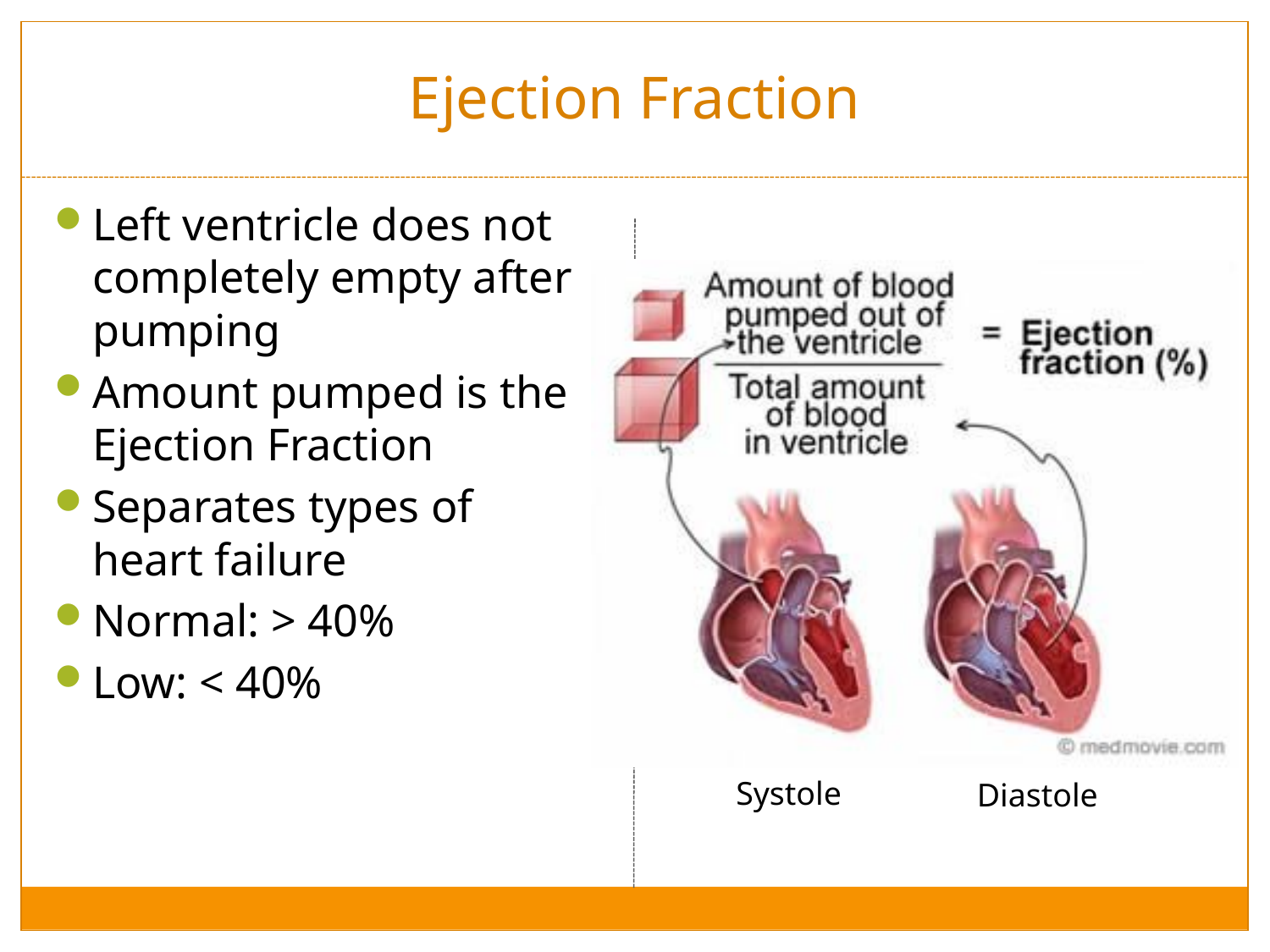

# Ejection Fraction
Left ventricle does not completely empty after pumping
Amount pumped is the Ejection Fraction
Separates types of heart failure
Normal: > 40%
Low: < 40%
Systole
Diastole

## Slide 11
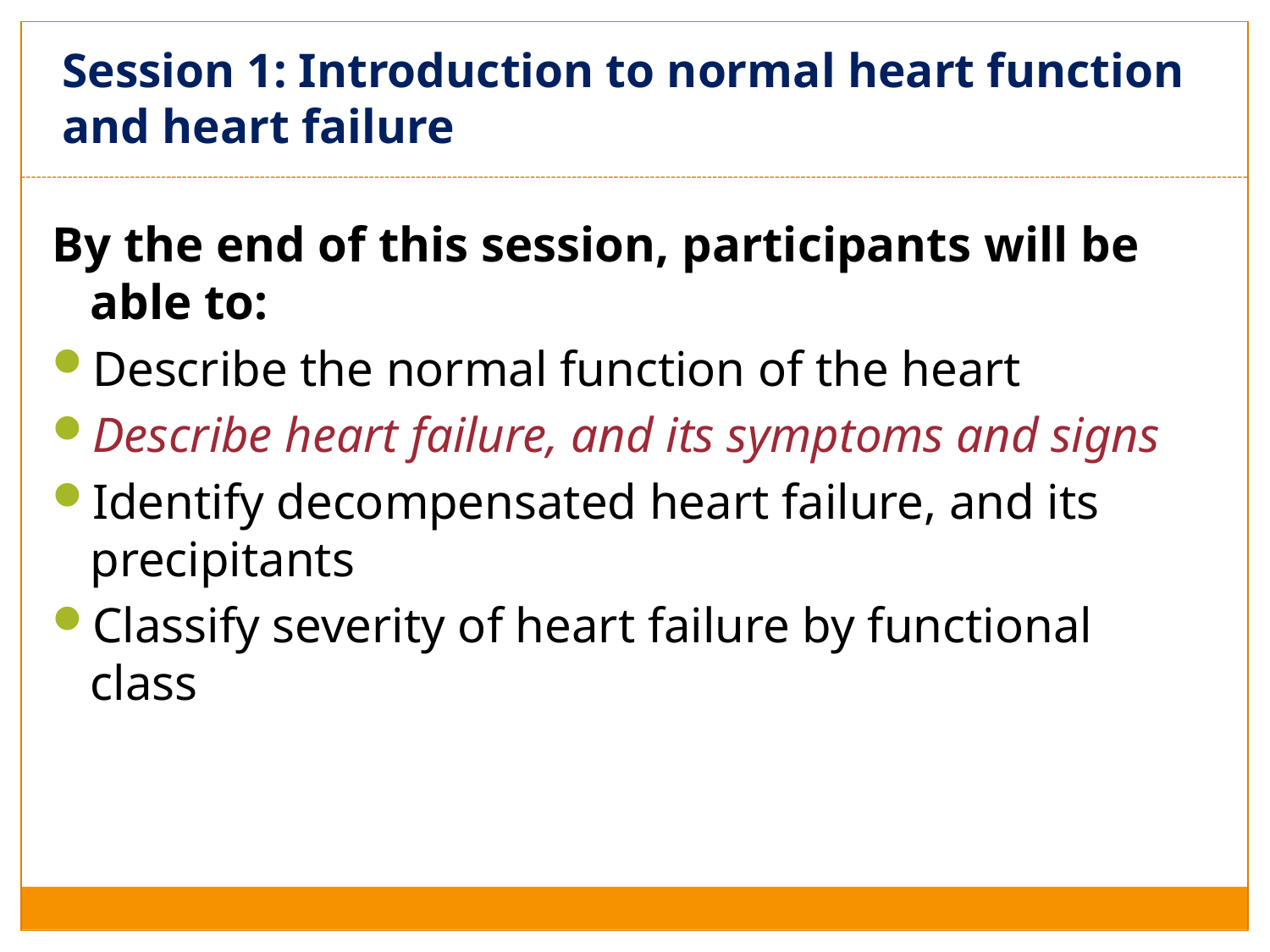

# Session 1: Introduction to normal heart function and heart failure
By the end of this session, participants will be able to:
Describe the normal function of the heart
Describe heart failure, and its symptoms and signs
Identify decompensated heart failure, and its precipitants
Classify severity of heart failure by functional class

## Slide 12
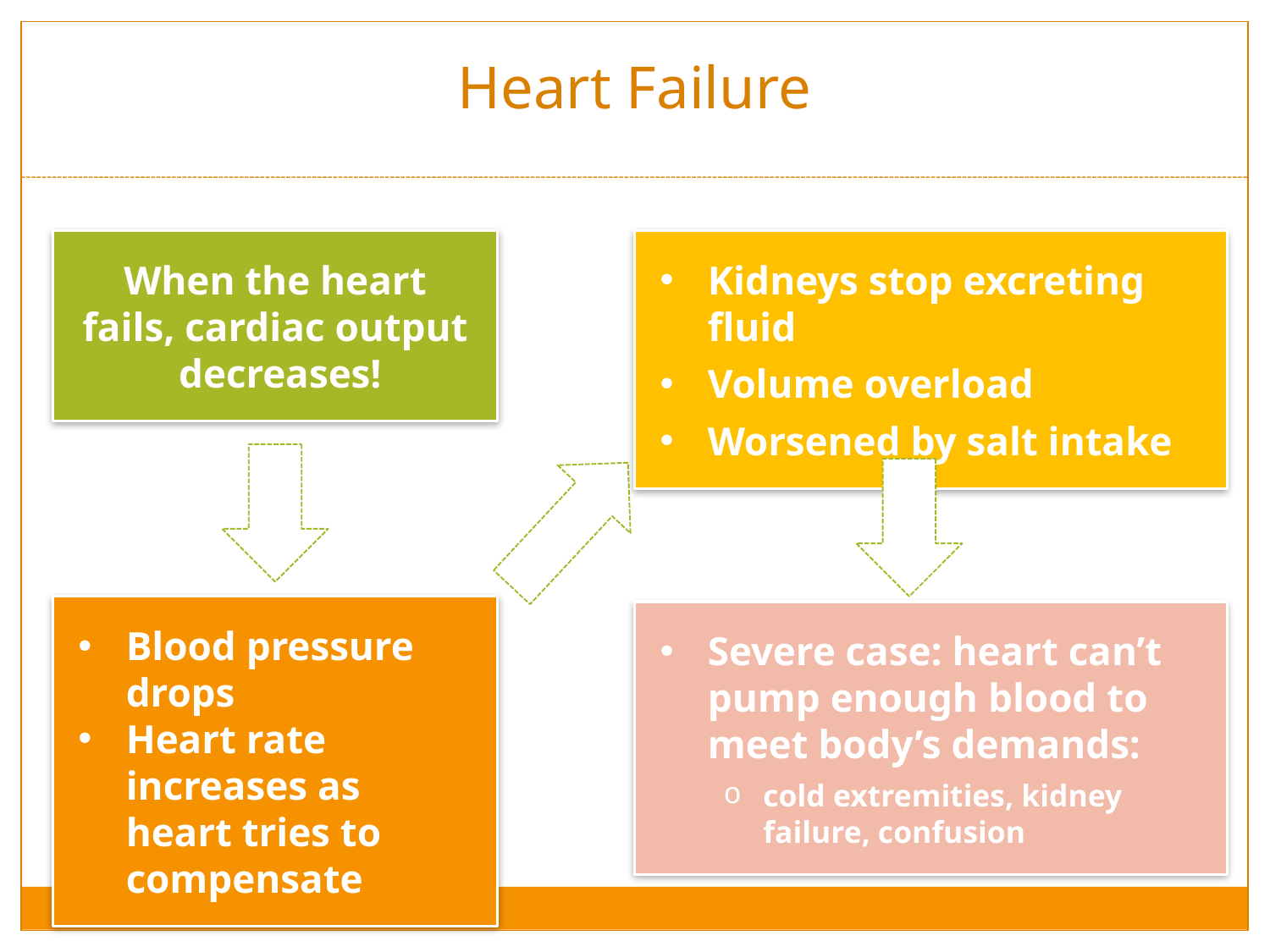

# Heart Failure
Kidneys stop excreting fluid
Volume overload
Worsened by salt intake
When the heart fails, cardiac output decreases!
Blood pressure drops
Heart rate increases as heart tries to compensate
Severe case: heart can’t pump enough blood to meet body’s demands:
cold extremities, kidney failure, confusion

## Slide 13
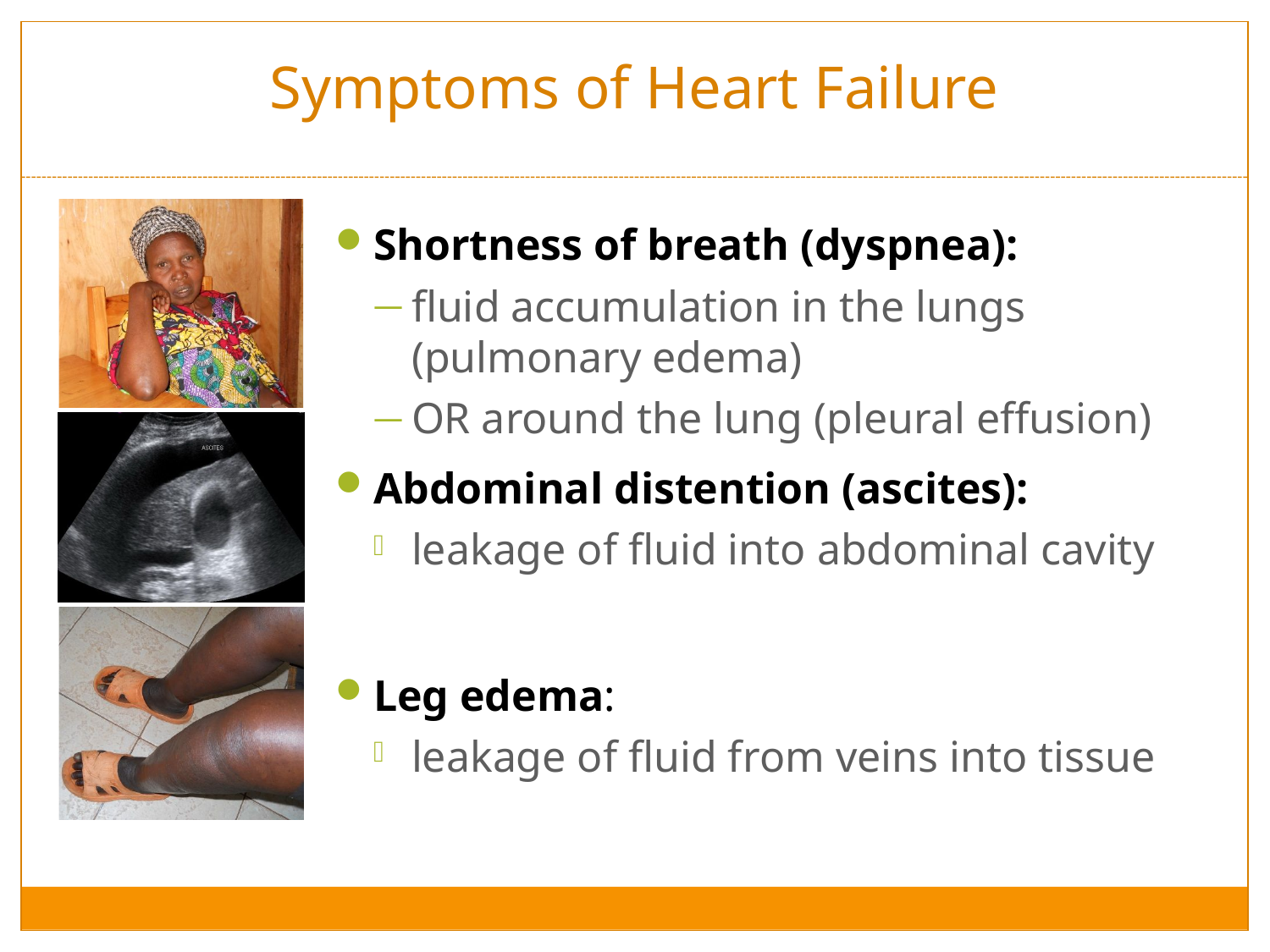

# Symptoms of Heart Failure
Shortness of breath (dyspnea):
fluid accumulation in the lungs (pulmonary edema)
OR around the lung (pleural effusion)
Abdominal distention (ascites):
leakage of fluid into abdominal cavity
Leg edema:
leakage of fluid from veins into tissue

## Slide 14
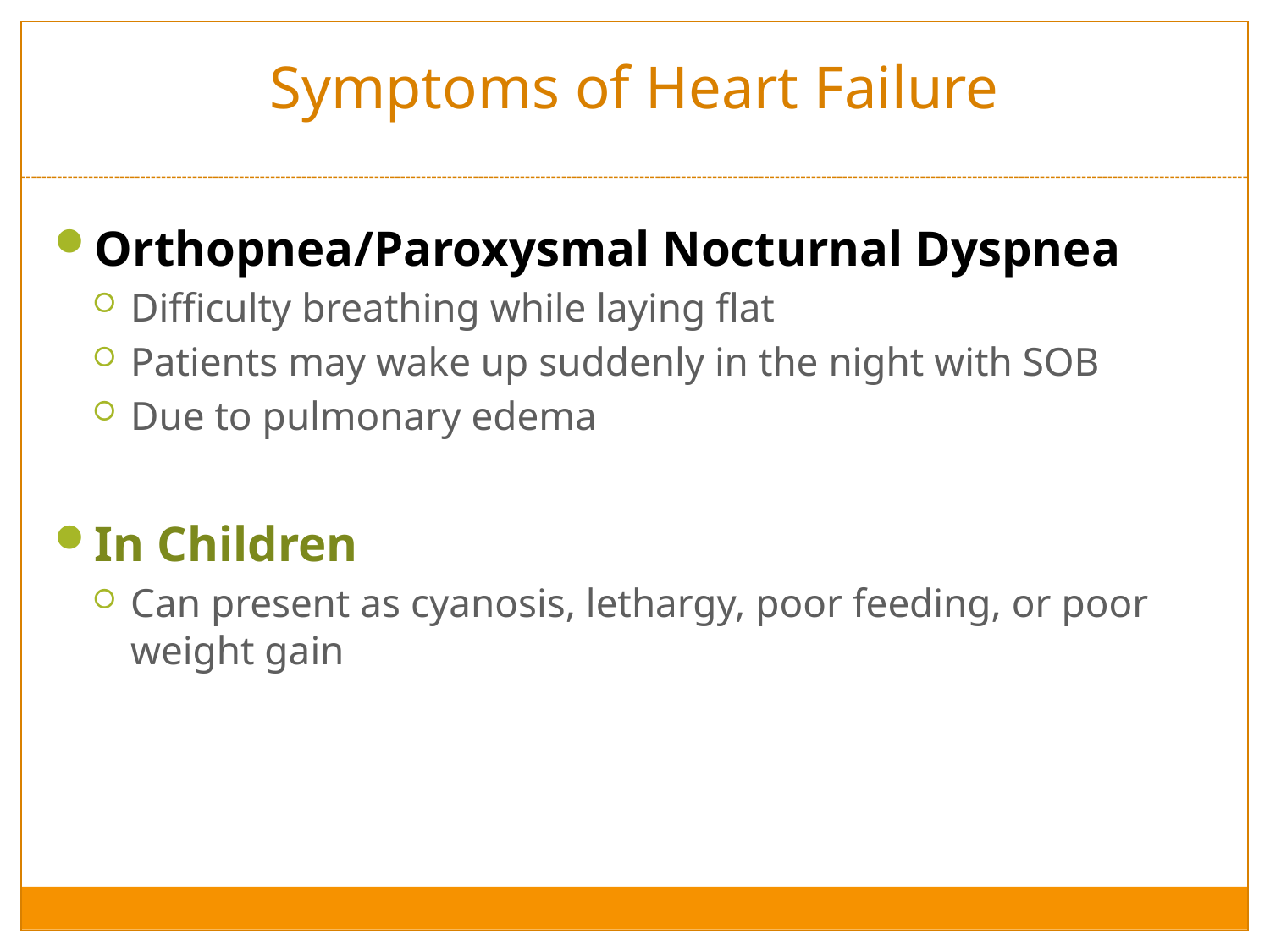

# Symptoms of Heart Failure
Orthopnea/Paroxysmal Nocturnal Dyspnea
Difficulty breathing while laying flat
Patients may wake up suddenly in the night with SOB
Due to pulmonary edema
In Children
Can present as cyanosis, lethargy, poor feeding, or poor weight gain

## Slide 15
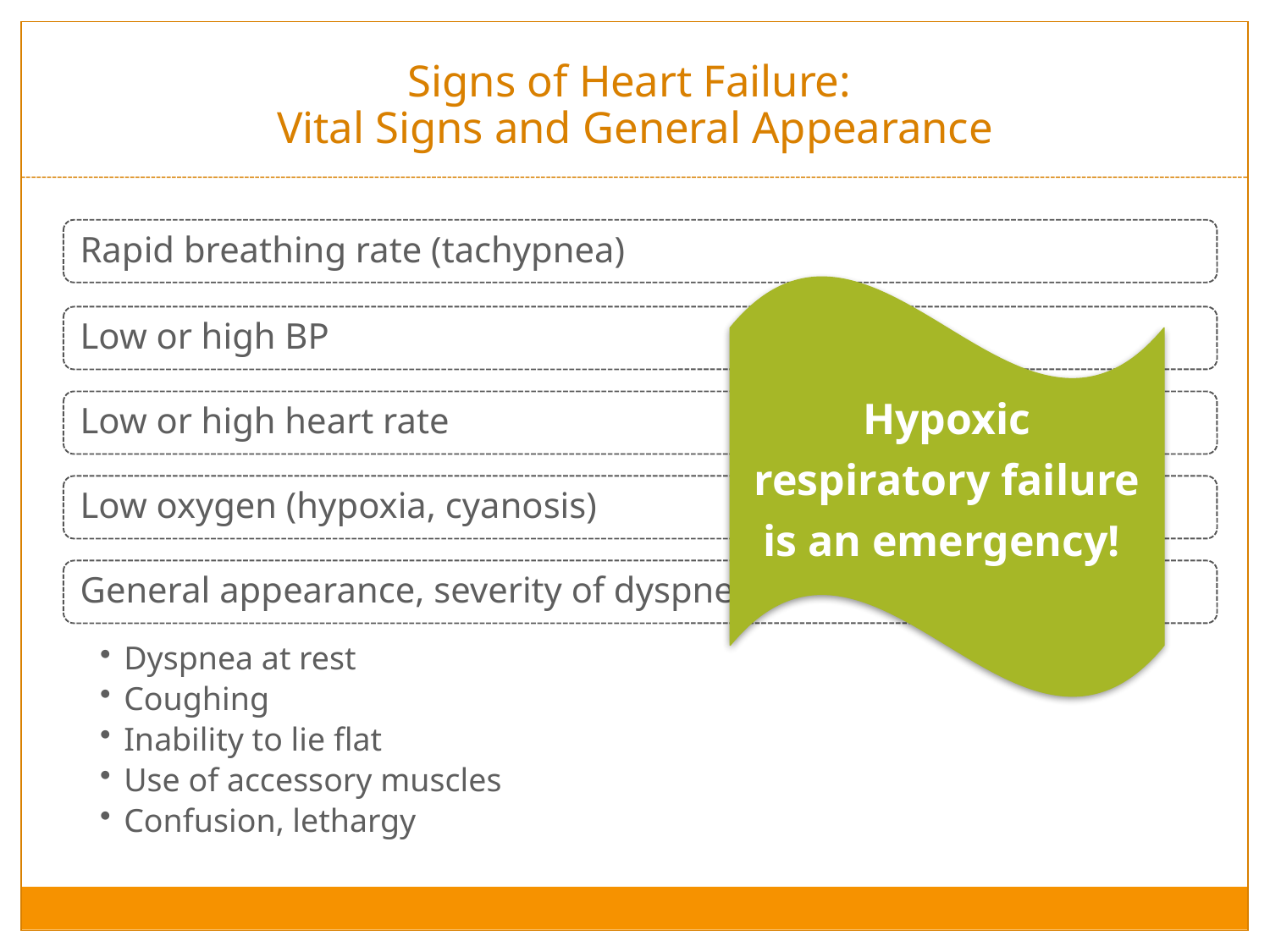

# Signs of Heart Failure: Vital Signs and General Appearance
Hypoxic respiratory failure is an emergency!

## Slide 16
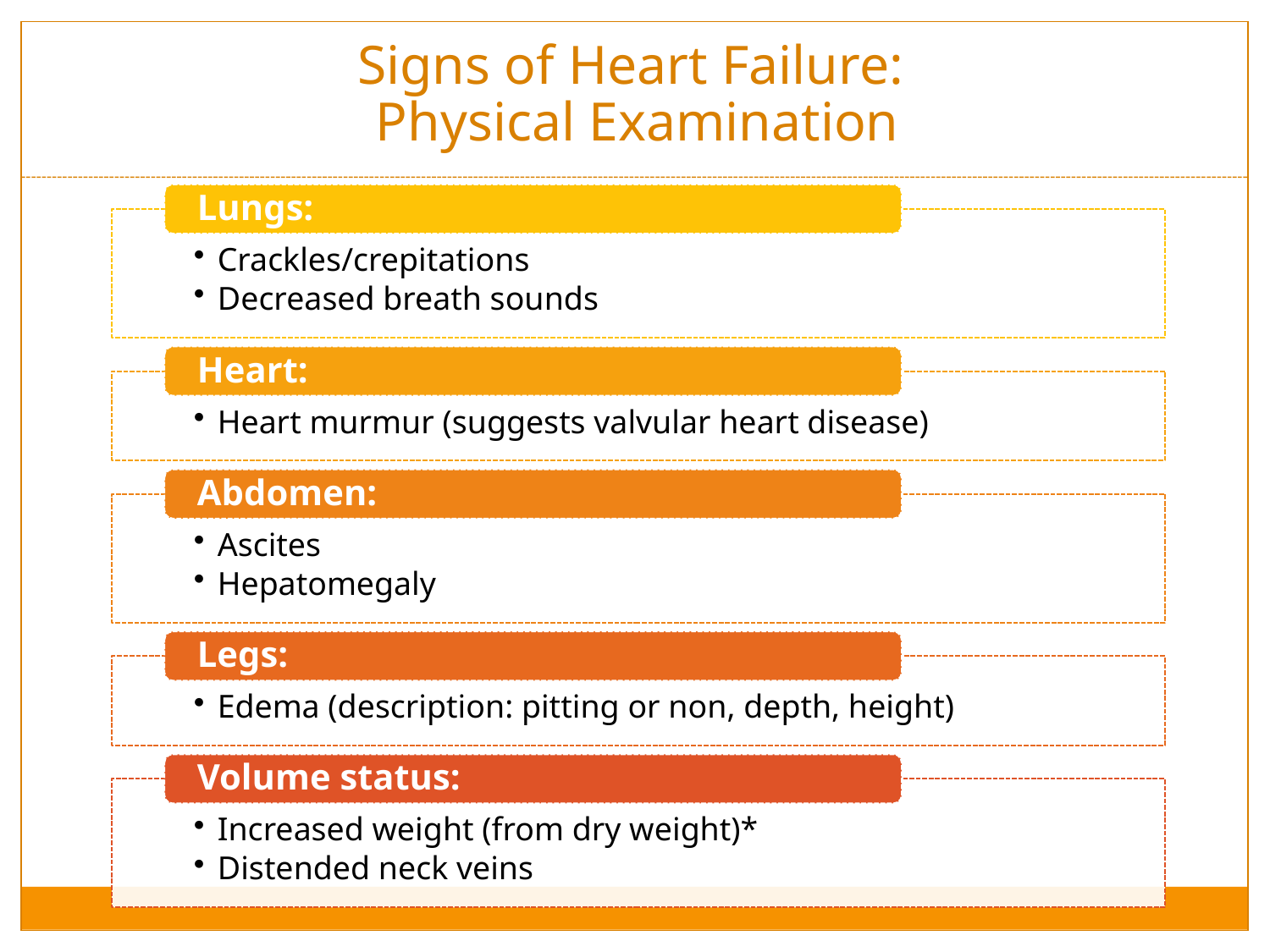

# Signs of Heart Failure: Physical Examination

## Slide 17
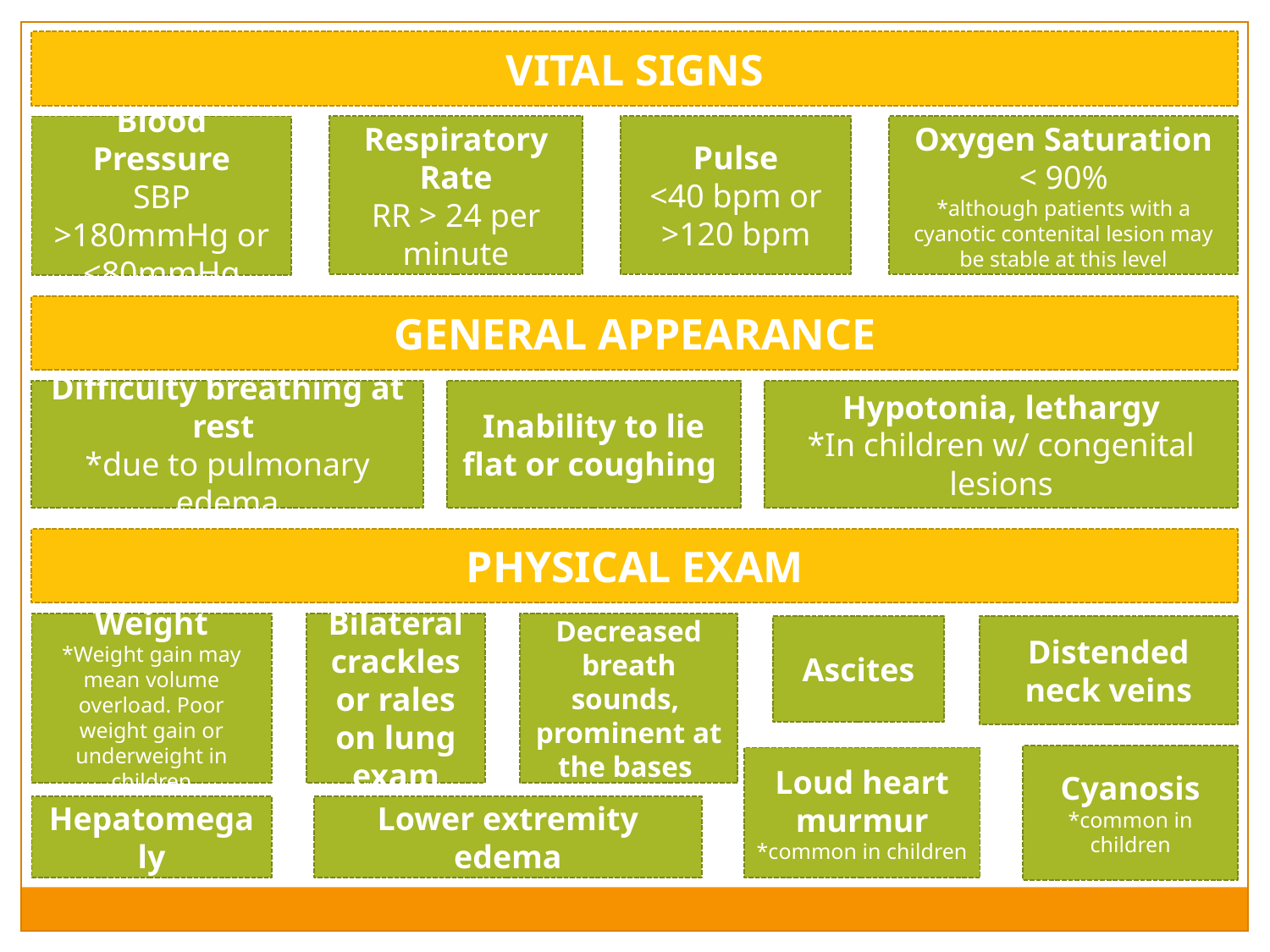

VITAL SIGNS
Blood Pressure
SBP >180mmHg or <80mmHg
Respiratory Rate
RR > 24 per minute
Pulse
<40 bpm or >120 bpm
Oxygen Saturation
< 90%
*although patients with a cyanotic contenital lesion may be stable at this level
GENERAL APPEARANCE
Difficulty breathing at rest
*due to pulmonary edema
Inability to lie flat or coughing
Hypotonia, lethargy
*In children w/ congenital lesions
PHYSICAL EXAM
Weight
*Weight gain may mean volume overload. Poor weight gain or underweight in children
Bilateral crackles or rales on lung exam
Decreased breath sounds, prominent at the bases
Ascites
Distended neck veins
Cyanosis
*common in children
Loud heart murmur
*common in children
Hepatomegaly
Lower extremity edema

## Slide 18
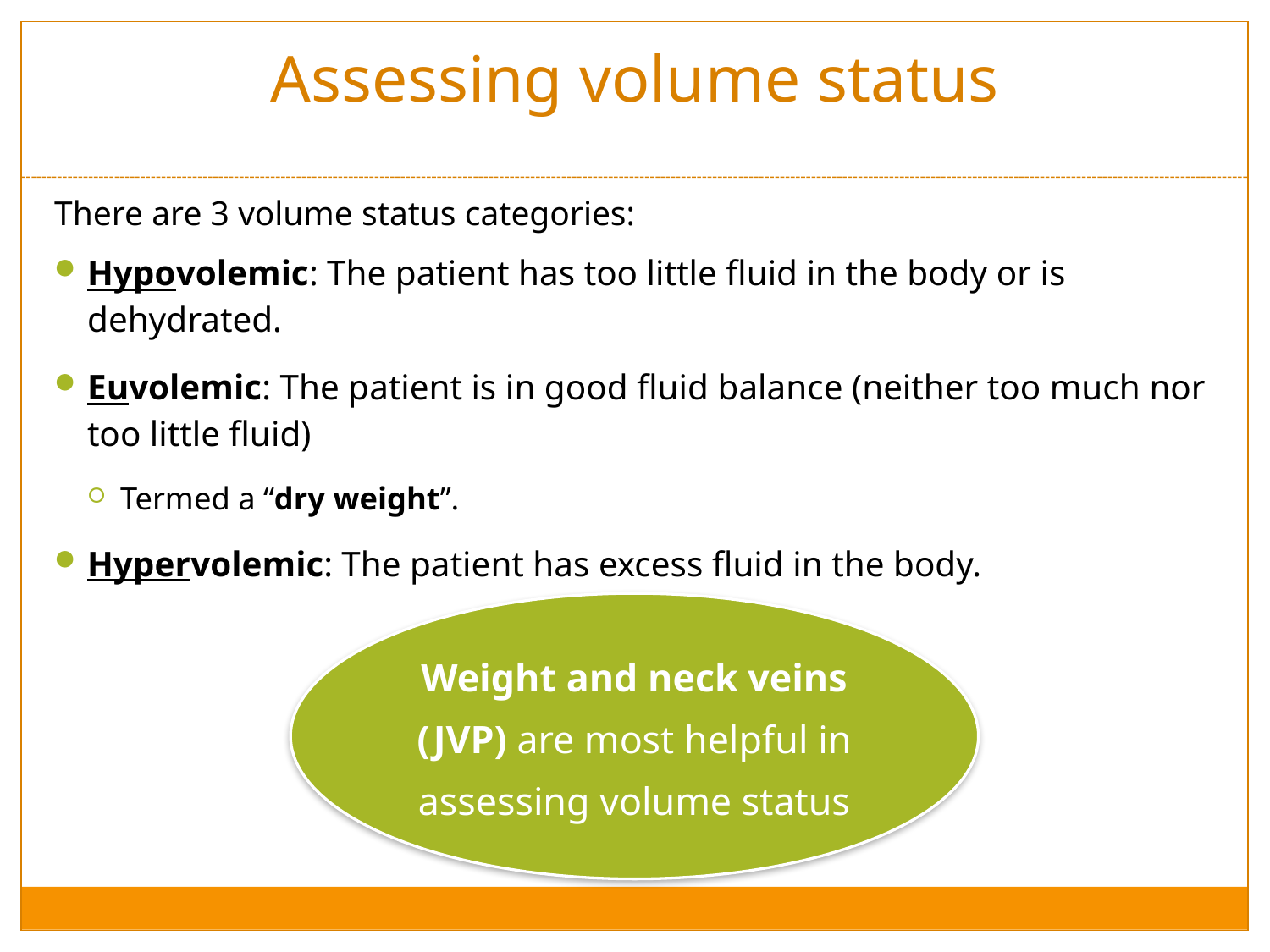

# Assessing volume status
There are 3 volume status categories:
Hypovolemic: The patient has too little fluid in the body or is dehydrated.
Euvolemic: The patient is in good fluid balance (neither too much nor too little fluid)
Termed a “dry weight”.
Hypervolemic: The patient has excess fluid in the body.
Weight and neck veins (JVP) are most helpful in assessing volume status

## Slide 19
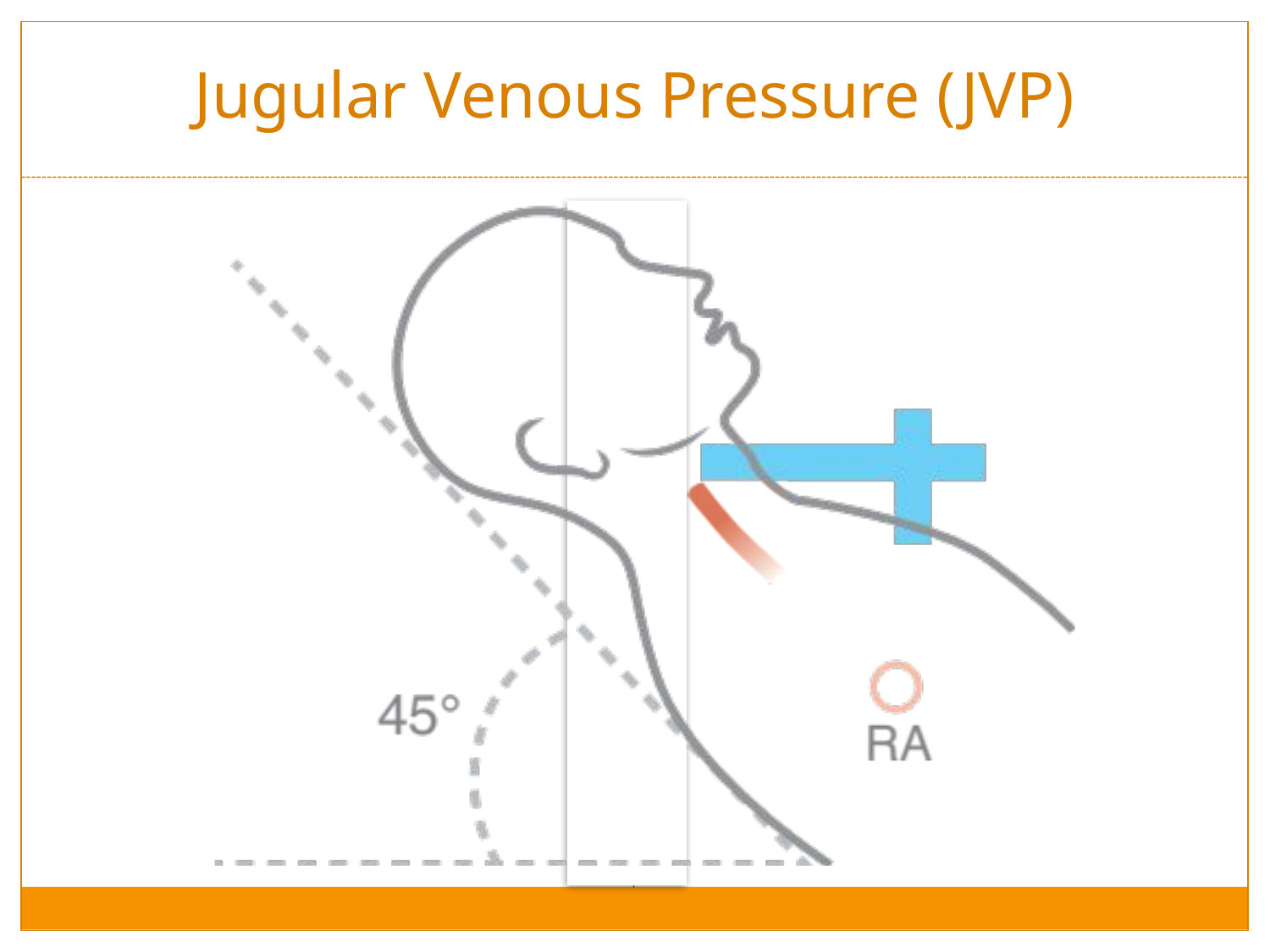

# Jugular Venous Pressure (JVP)

## Slide 20
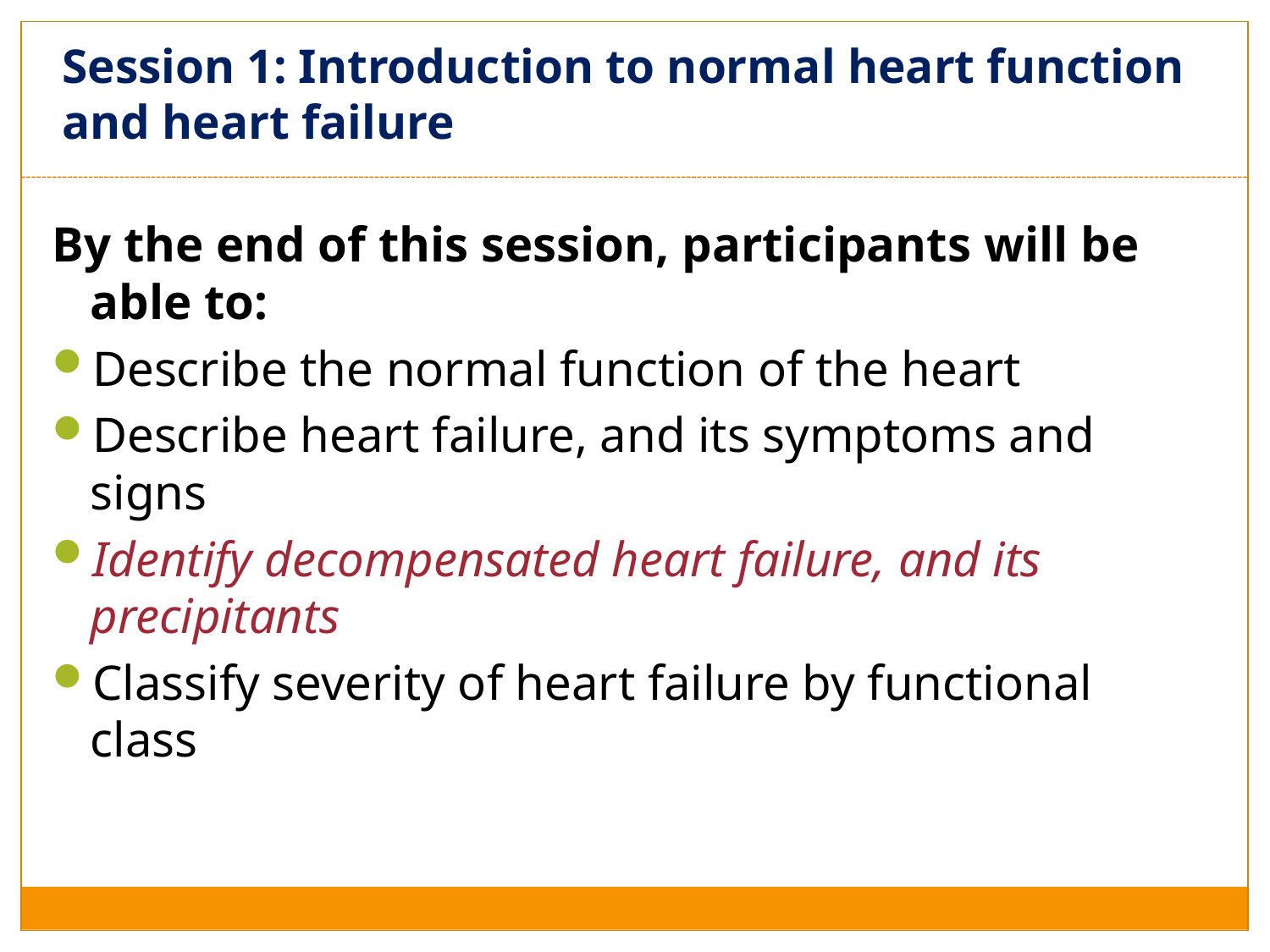

# Session 1: Introduction to normal heart function and heart failure
By the end of this session, participants will be able to:
Describe the normal function of the heart
Describe heart failure, and its symptoms and signs
Identify decompensated heart failure, and its precipitants
Classify severity of heart failure by functional class

## Slide 21
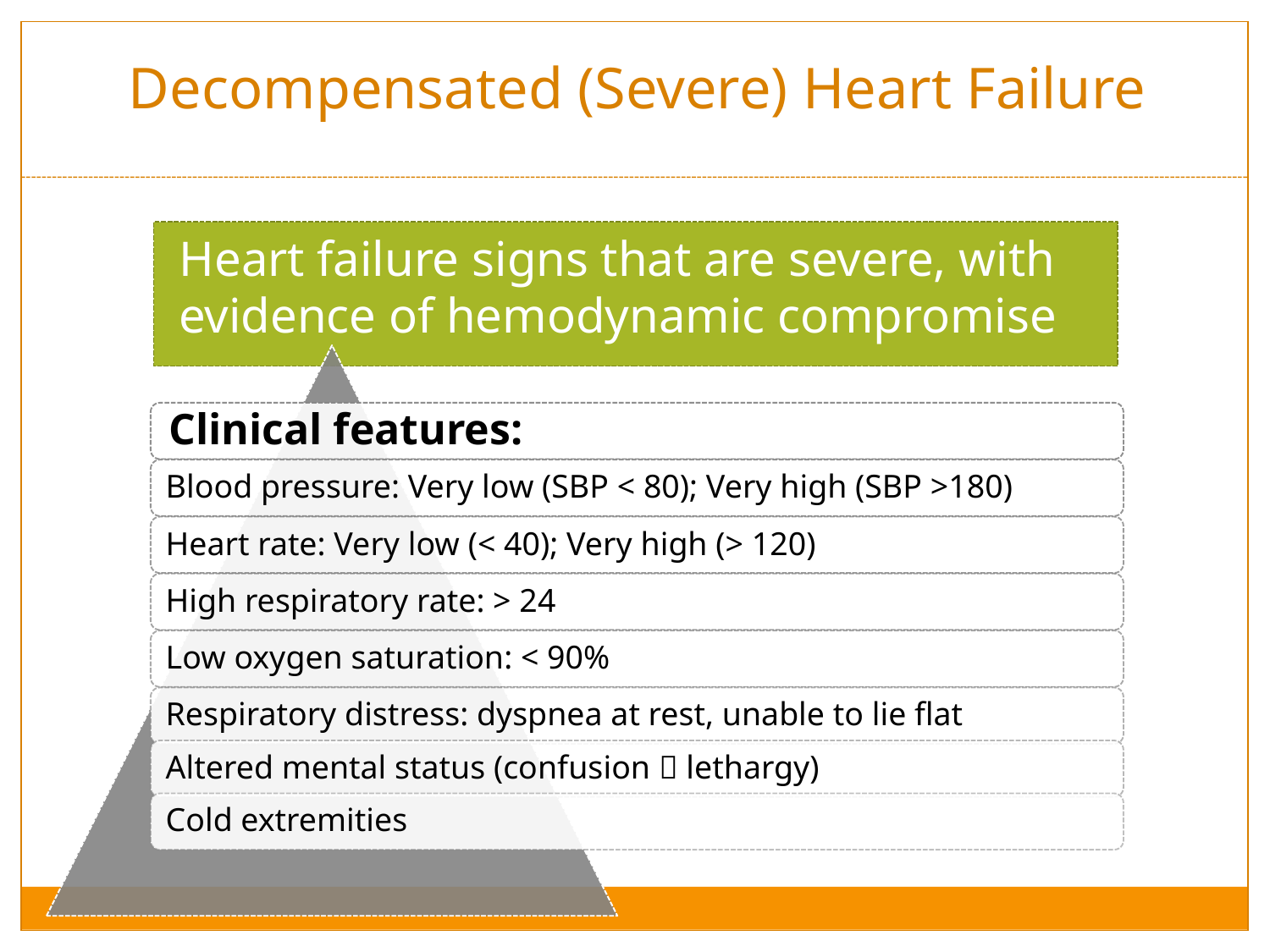

# Decompensated (Severe) Heart Failure
Heart failure signs that are severe, with evidence of hemodynamic compromise

## Slide 22
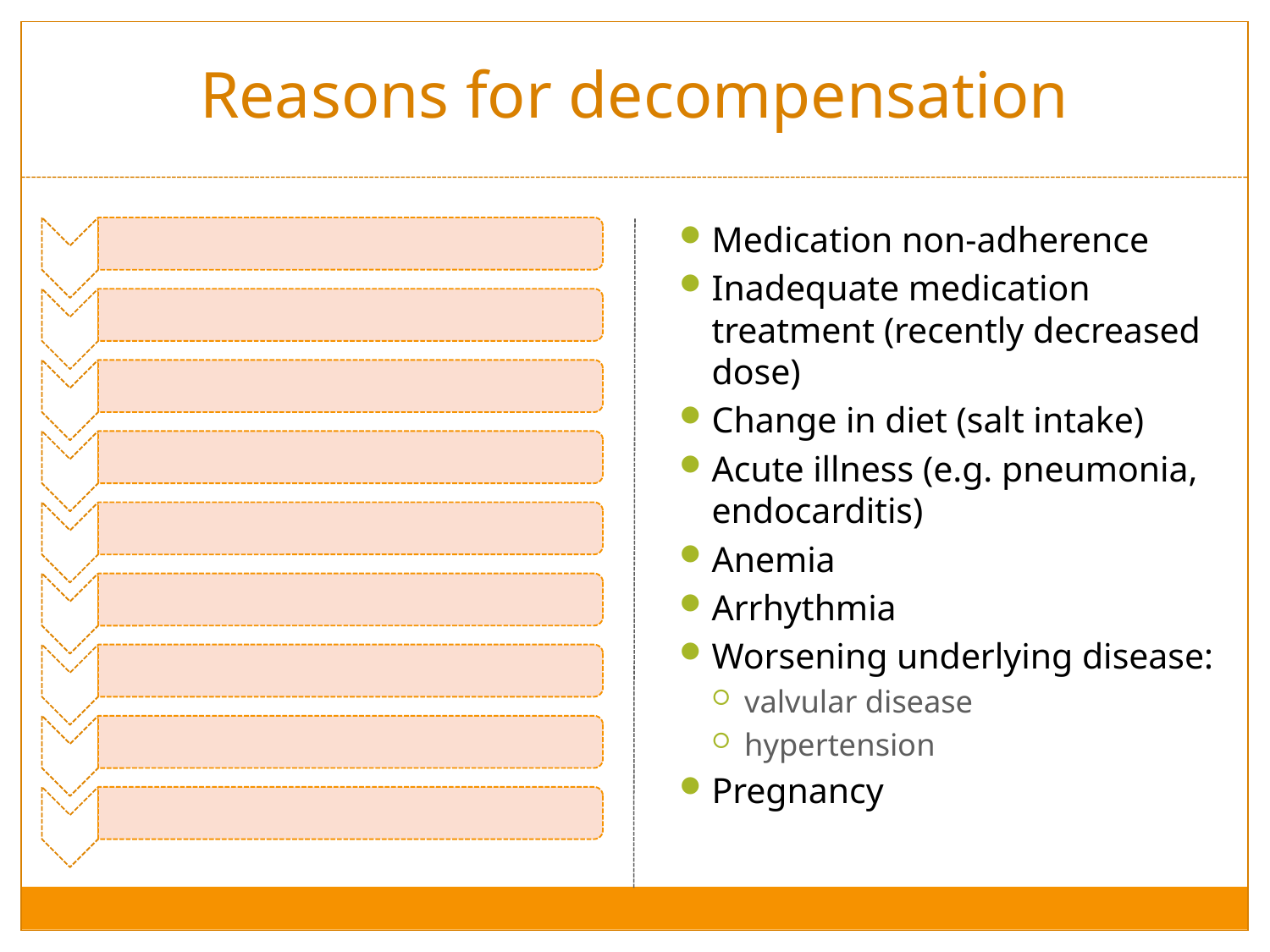

# Reasons for decompensation
Medication non-adherence
Inadequate medication treatment (recently decreased dose)
Change in diet (salt intake)
Acute illness (e.g. pneumonia, endocarditis)
Anemia
Arrhythmia
Worsening underlying disease:
valvular disease
hypertension
Pregnancy

## Slide 23
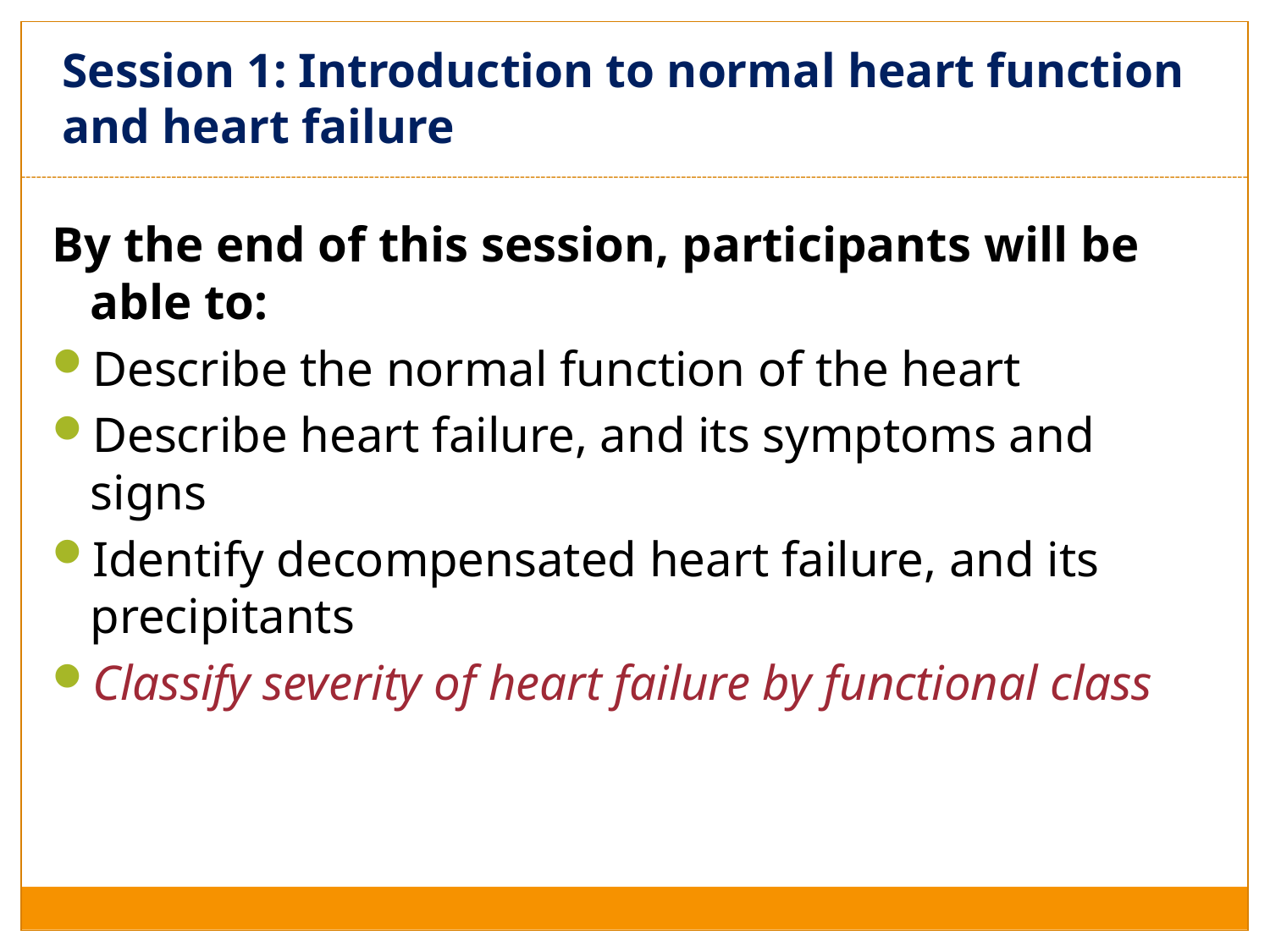

# Session 1: Introduction to normal heart function and heart failure
By the end of this session, participants will be able to:
Describe the normal function of the heart
Describe heart failure, and its symptoms and signs
Identify decompensated heart failure, and its precipitants
Classify severity of heart failure by functional class

## Slide 24
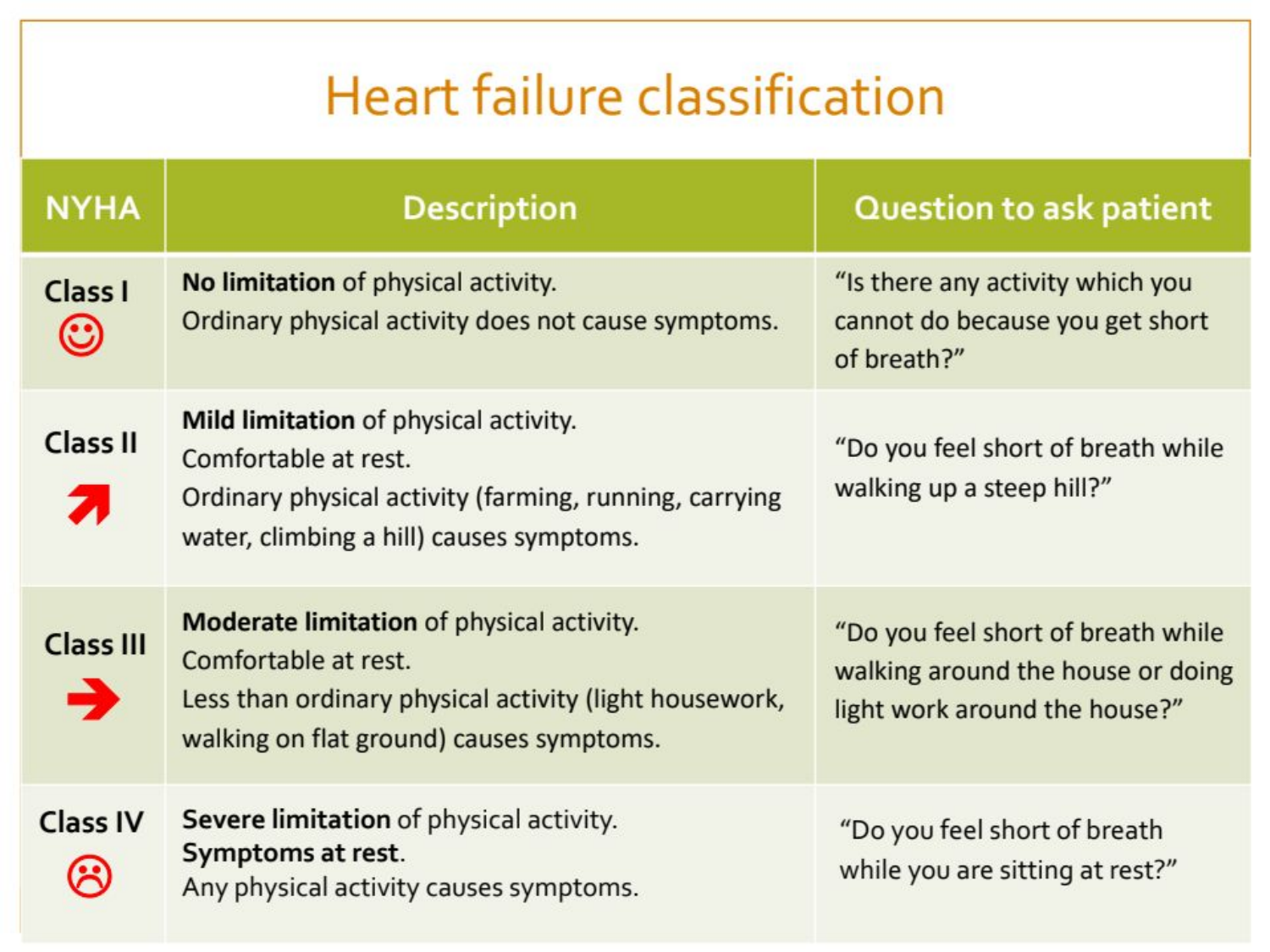

# Heart failure classification
| NYHA | Description | Question to ask patient |
| --- | --- | --- |
| | | |
| | | |
| | | |
| | | |
No limitation of physical activity.
Ordinary physical activity does not cause symptoms.
“Is there any activity which you cannot do because you get short of breath?”
Class I

Mild limitation of physical activity.
Comfortable at rest.
Ordinary physical activity (farming, running, carrying water, climbing a hill) causes symptoms.
Class II
“Do you feel short of breath while walking up a steep hill?”

Moderate limitation of physical activity.
Comfortable at rest.
Less than ordinary physical activity (light housework, walking on flat ground) causes symptoms.
“Do you feel short of breath while walking around the house or doing light work around the house?”
Class III

Class IV
Severe limitation of physical activity.
Symptoms at rest.
Any physical activity causes symptoms.
“Do you feel short of breath while you are sitting at rest?”


## Slide 25
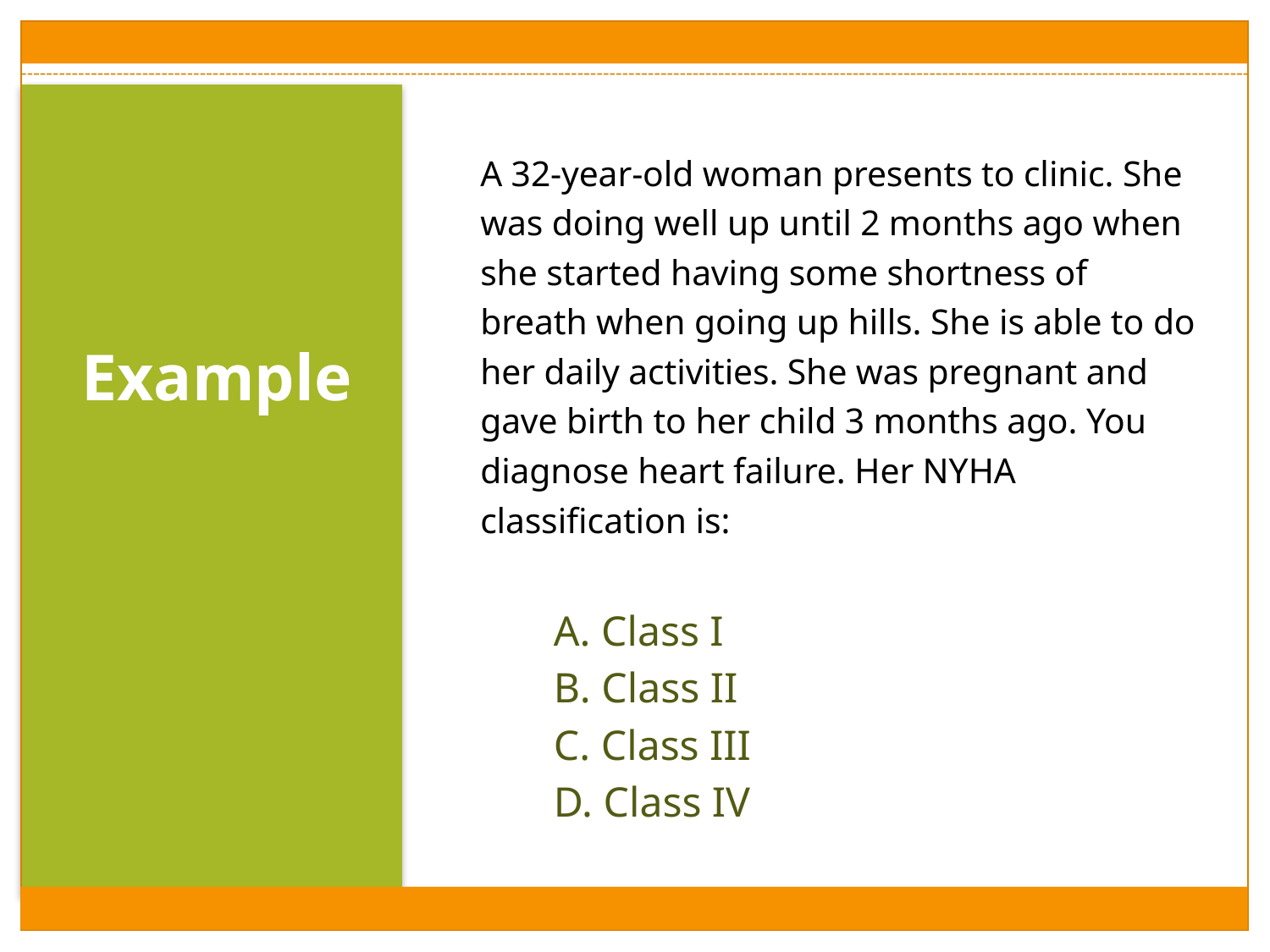

# Example
A 32-year-old woman presents to clinic. She was doing well up until 2 months ago when she started having some shortness of breath when going up hills. She is able to do her daily activities. She was pregnant and gave birth to her child 3 months ago. You diagnose heart failure. Her NYHA classification is:
A. Class I
B. Class II
C. Class III
D. Class IV

## Slide 26
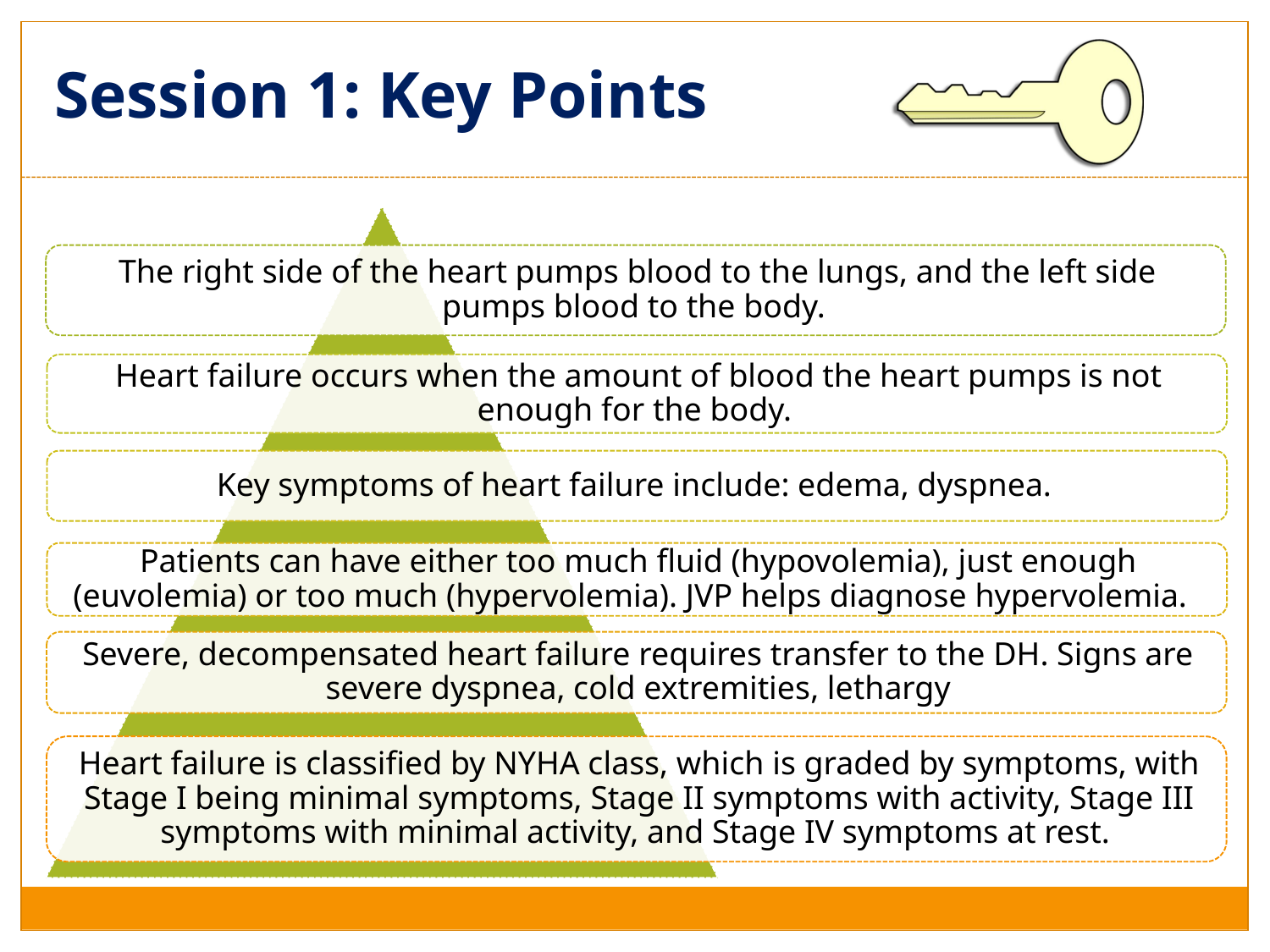

# Session 1: Key Points

## Slide 27
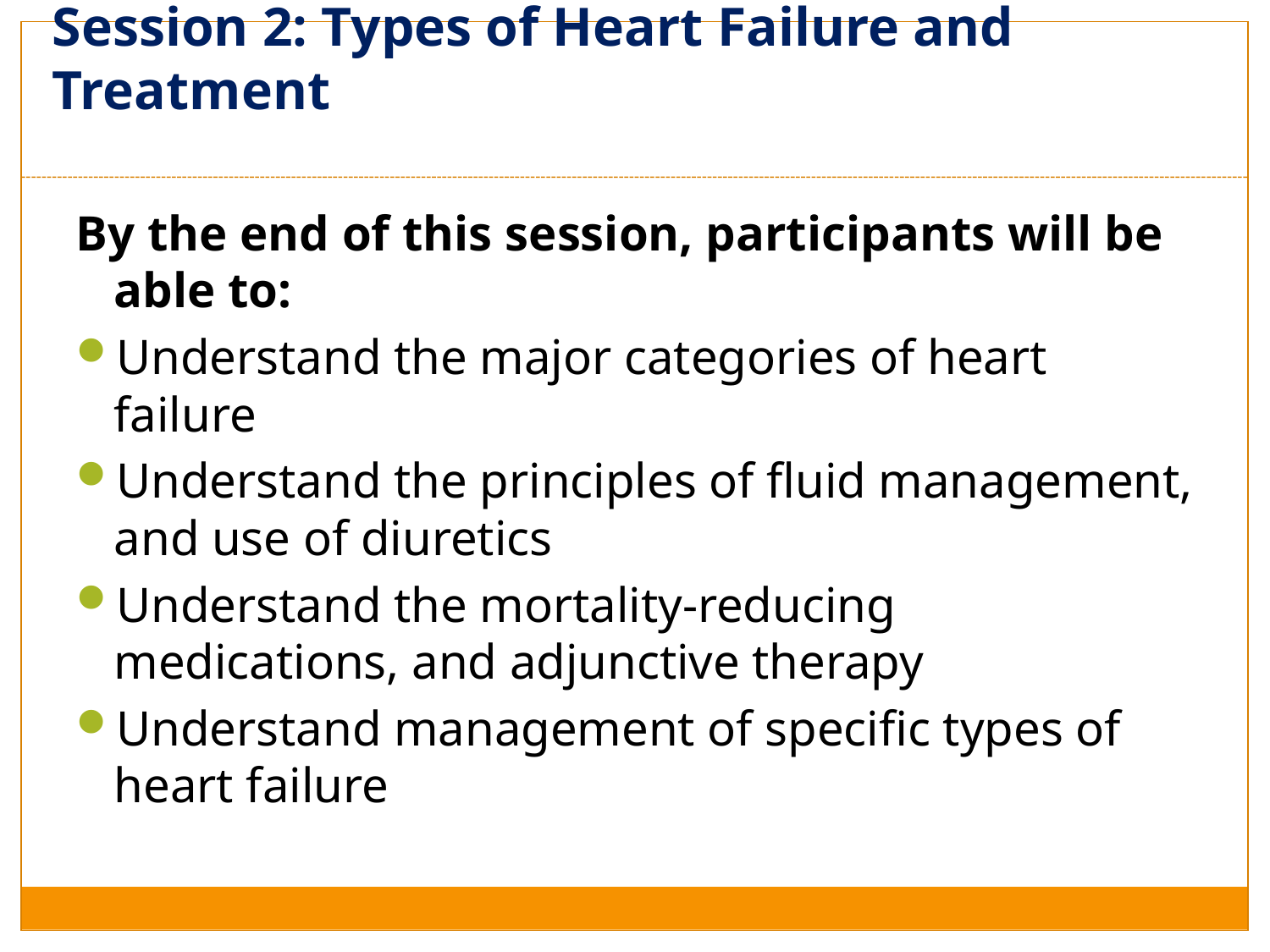

# Session 2: Types of Heart Failure and Treatment
By the end of this session, participants will be able to:
Understand the major categories of heart failure
Understand the principles of fluid management, and use of diuretics
Understand the mortality-reducing medications, and adjunctive therapy
Understand management of specific types of heart failure

## Slide 28
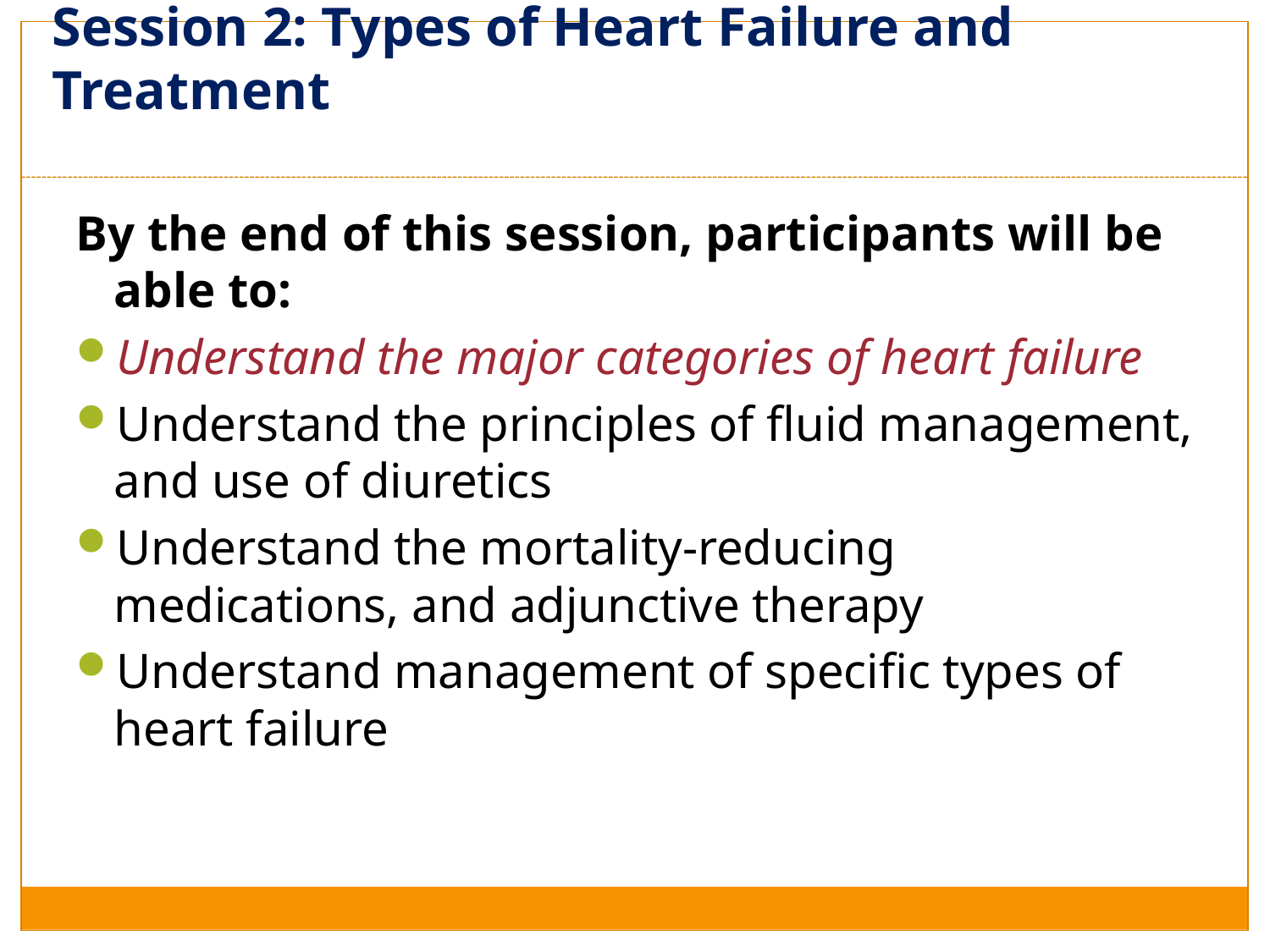

Session 2: Types of Heart Failure and Treatment
By the end of this session, participants will be able to:
Understand the major categories of heart failure
Understand the principles of fluid management, and use of diuretics
Understand the mortality-reducing medications, and adjunctive therapy
Understand management of specific types of heart failure

## Slide 29
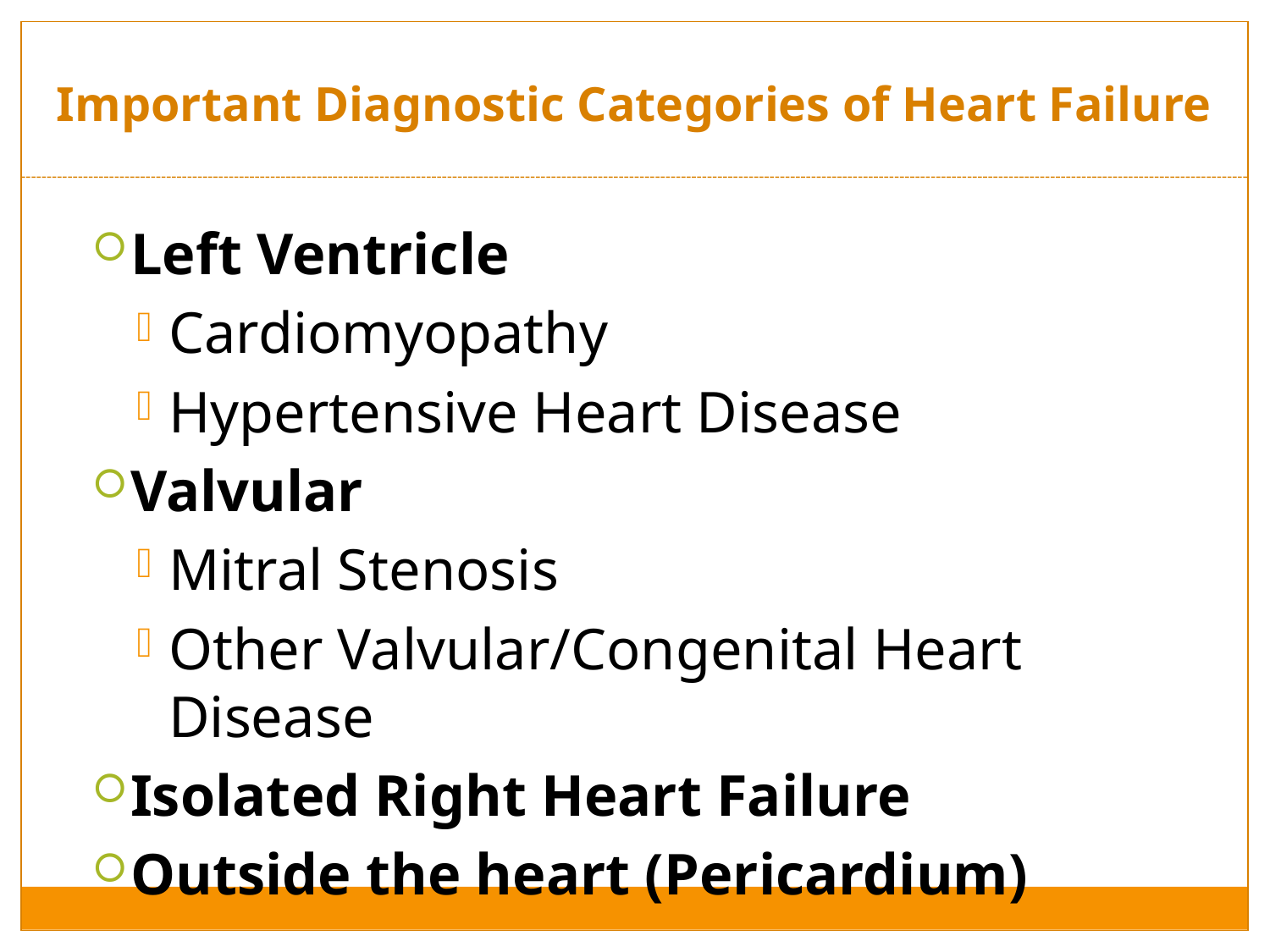

# Important Diagnostic Categories of Heart Failure
Left Ventricle
Cardiomyopathy
Hypertensive Heart Disease
Valvular
Mitral Stenosis
Other Valvular/Congenital Heart Disease
Isolated Right Heart Failure
Outside the heart (Pericardium)

## Slide 30
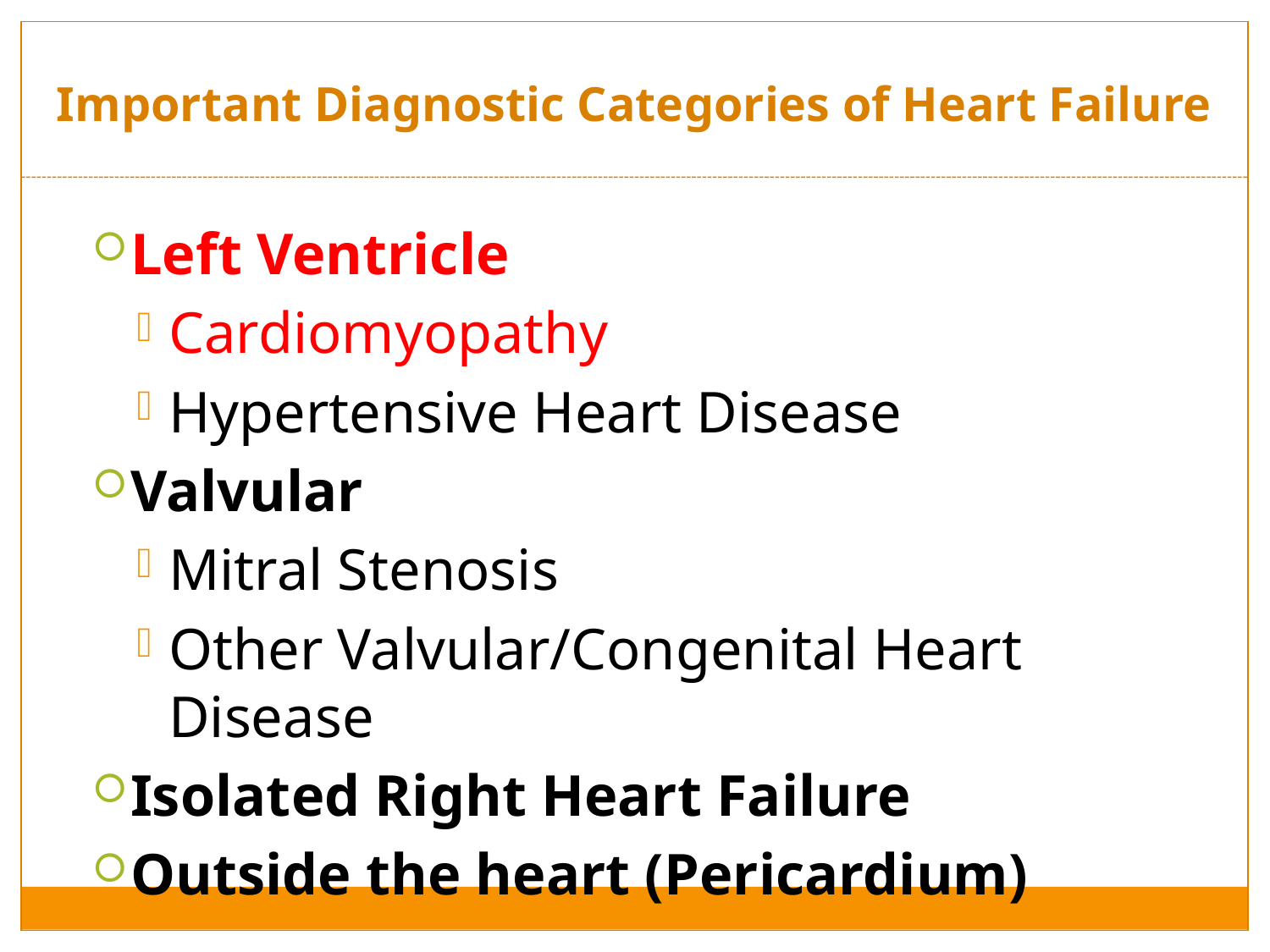

# Important Diagnostic Categories of Heart Failure
Left Ventricle
Cardiomyopathy
Hypertensive Heart Disease
Valvular
Mitral Stenosis
Other Valvular/Congenital Heart Disease
Isolated Right Heart Failure
Outside the heart (Pericardium)

## Slide 31
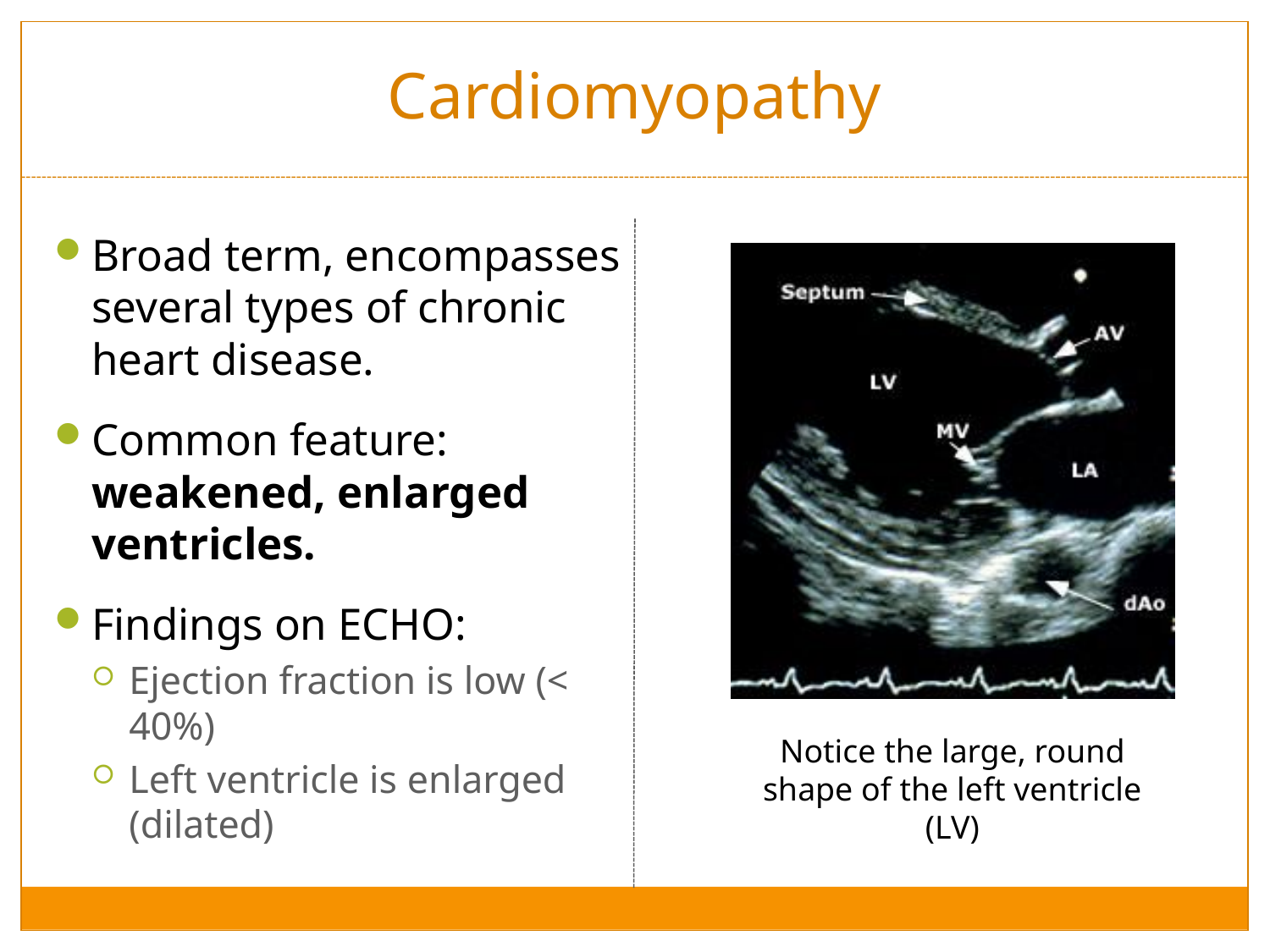

# Cardiomyopathy
Broad term, encompasses several types of chronic heart disease.
Common feature: weakened, enlarged ventricles.
Findings on ECHO:
Ejection fraction is low (< 40%)
Left ventricle is enlarged (dilated)
Notice the large, round shape of the left ventricle (LV)

## Slide 32
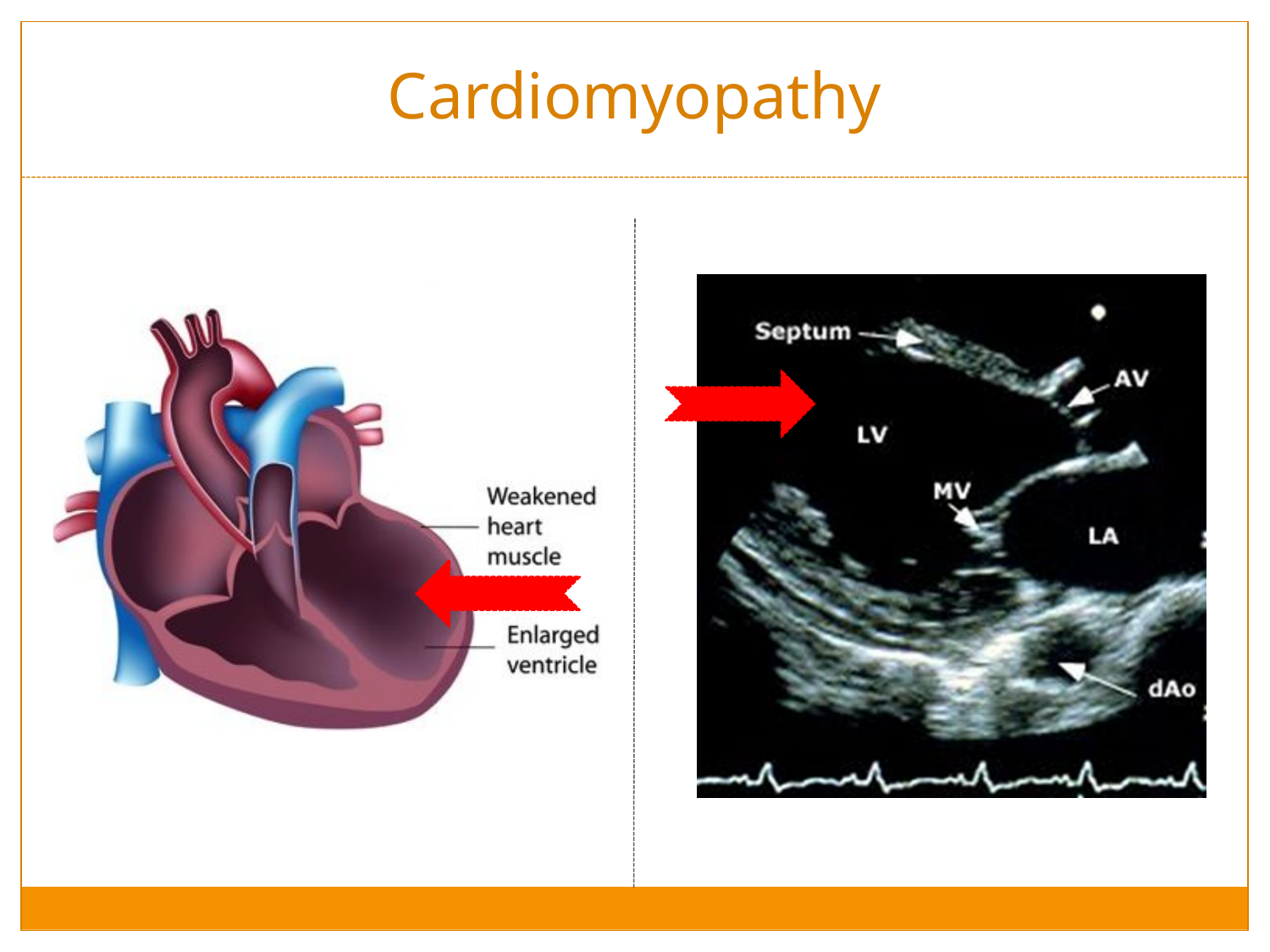

# Cardiomyopathy

## Slide 33
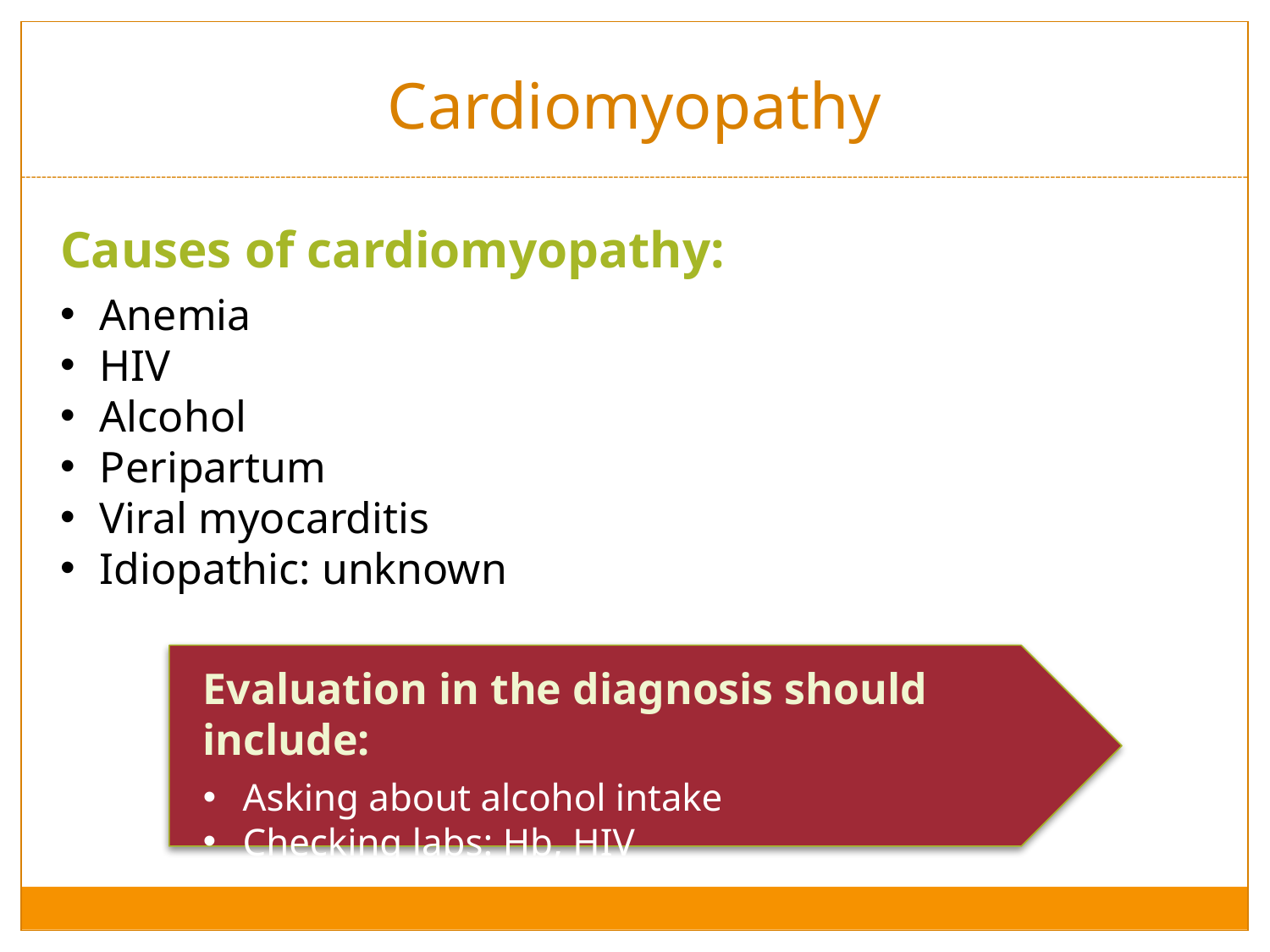

# Cardiomyopathy
Causes of cardiomyopathy:
Anemia
HIV
Alcohol
Peripartum
Viral myocarditis
Idiopathic: unknown
Evaluation in the diagnosis should include:
Asking about alcohol intake
Checking labs: Hb, HIV

## Slide 34
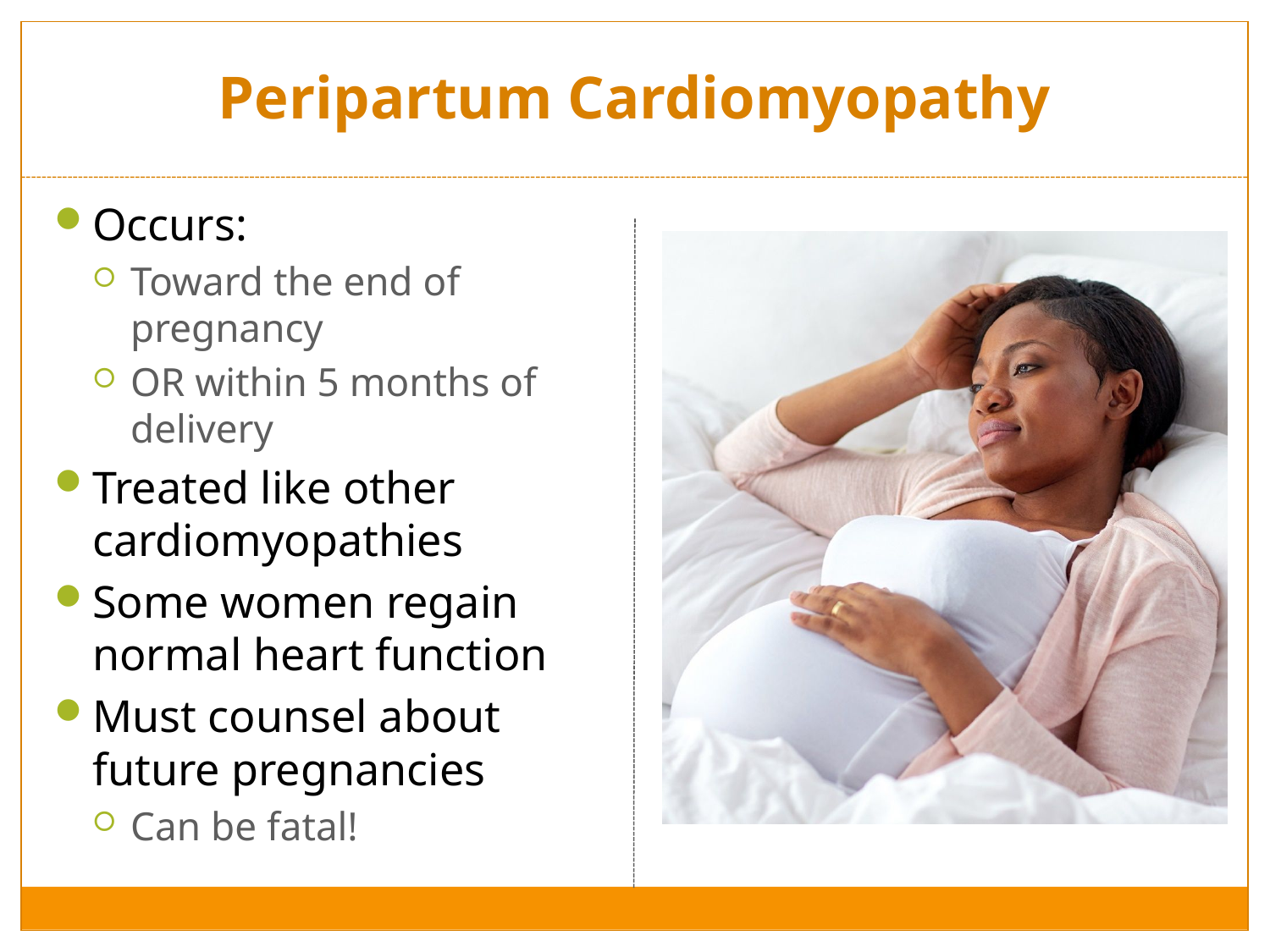

# Peripartum Cardiomyopathy
Occurs:
Toward the end of pregnancy
OR within 5 months of delivery
Treated like other cardiomyopathies
Some women regain normal heart function
Must counsel about future pregnancies
Can be fatal!

## Slide 35
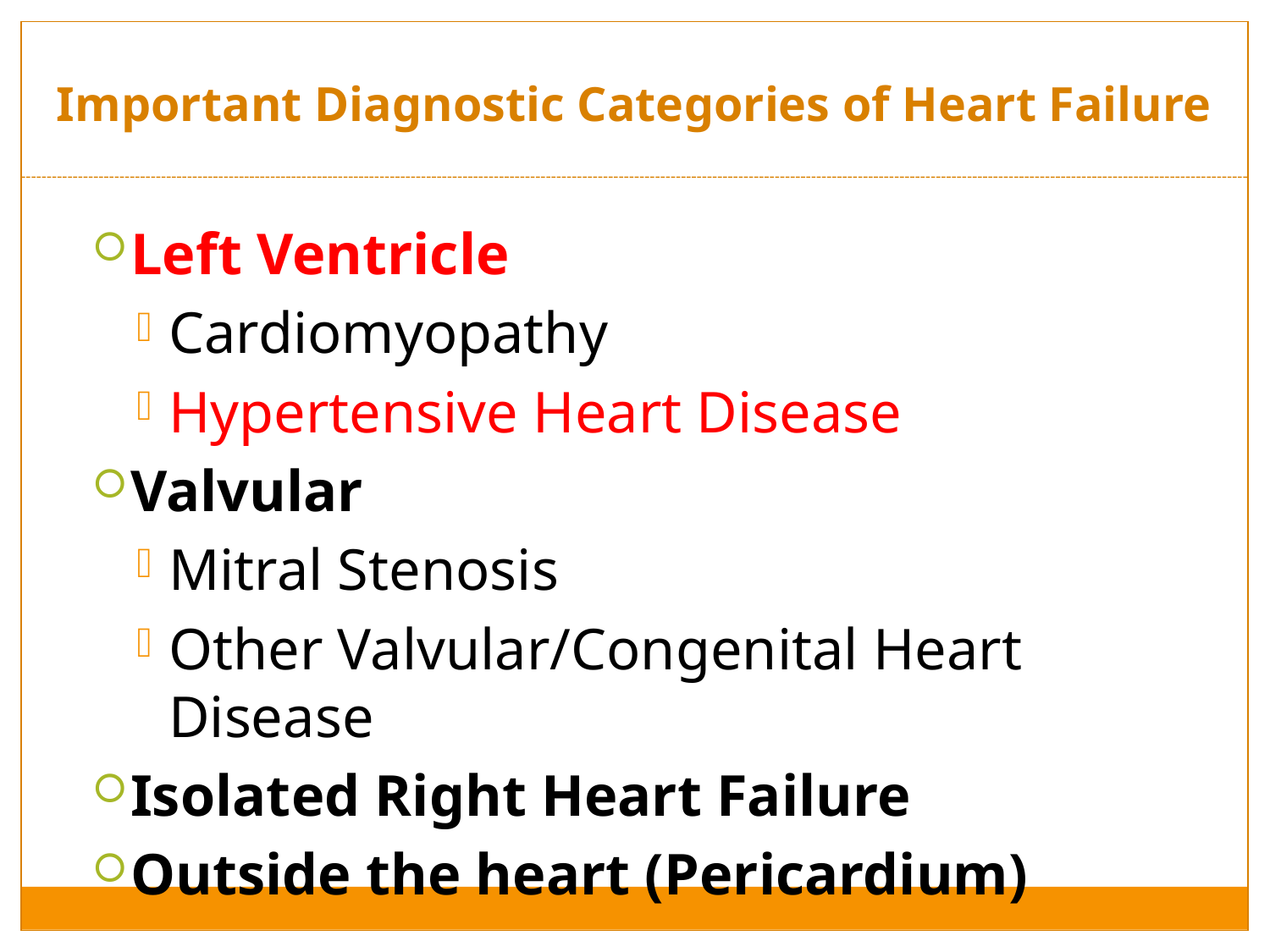

# Important Diagnostic Categories of Heart Failure
Left Ventricle
Cardiomyopathy
Hypertensive Heart Disease
Valvular
Mitral Stenosis
Other Valvular/Congenital Heart Disease
Isolated Right Heart Failure
Outside the heart (Pericardium)

## Slide 36
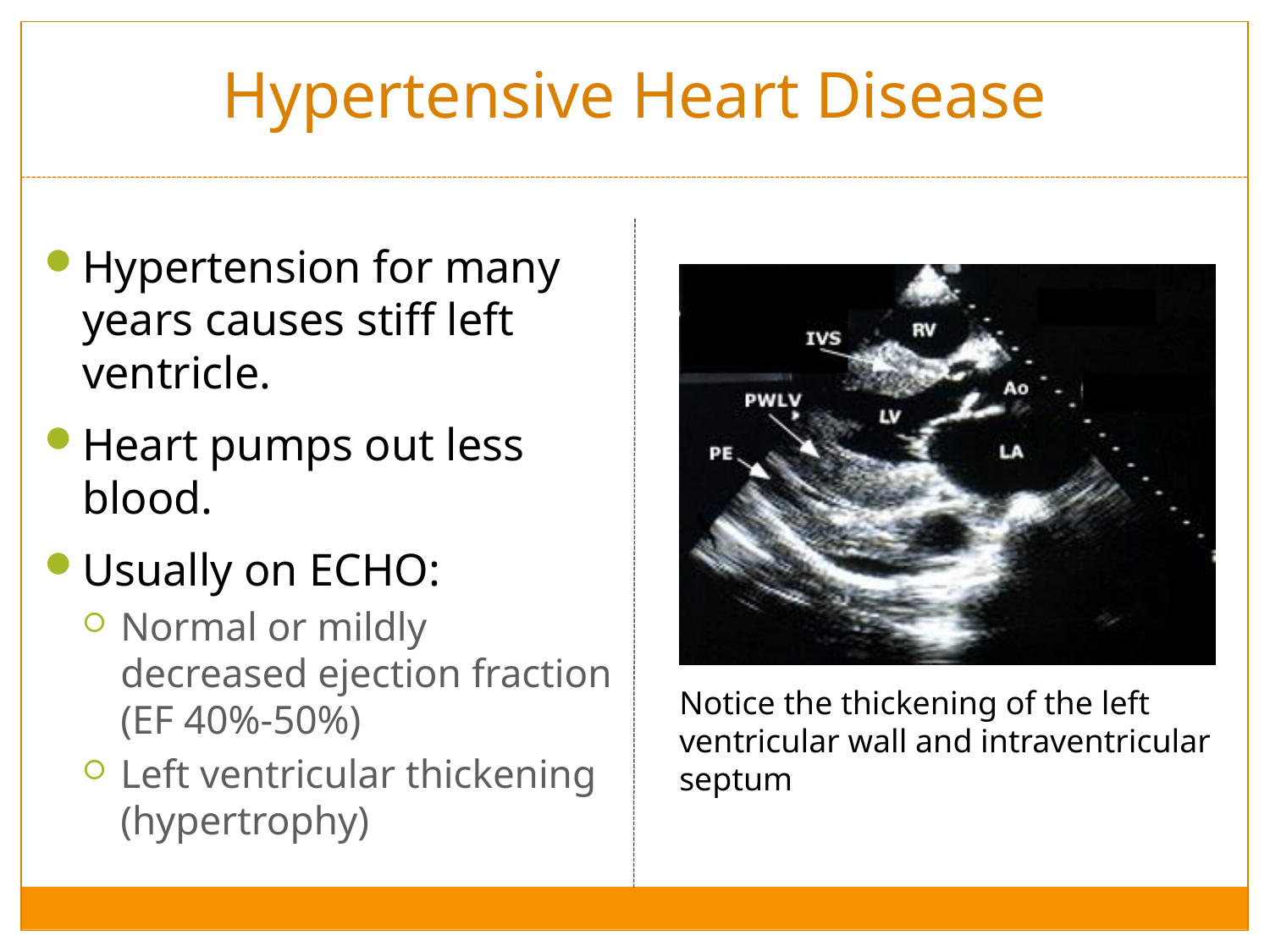

# Hypertensive Heart Disease
Hypertension for many years causes stiff left ventricle.
Heart pumps out less blood.
Usually on ECHO:
Normal or mildly decreased ejection fraction (EF 40%-50%)
Left ventricular thickening (hypertrophy)
Notice the thickening of the left ventricular wall and intraventricular septum

## Slide 37
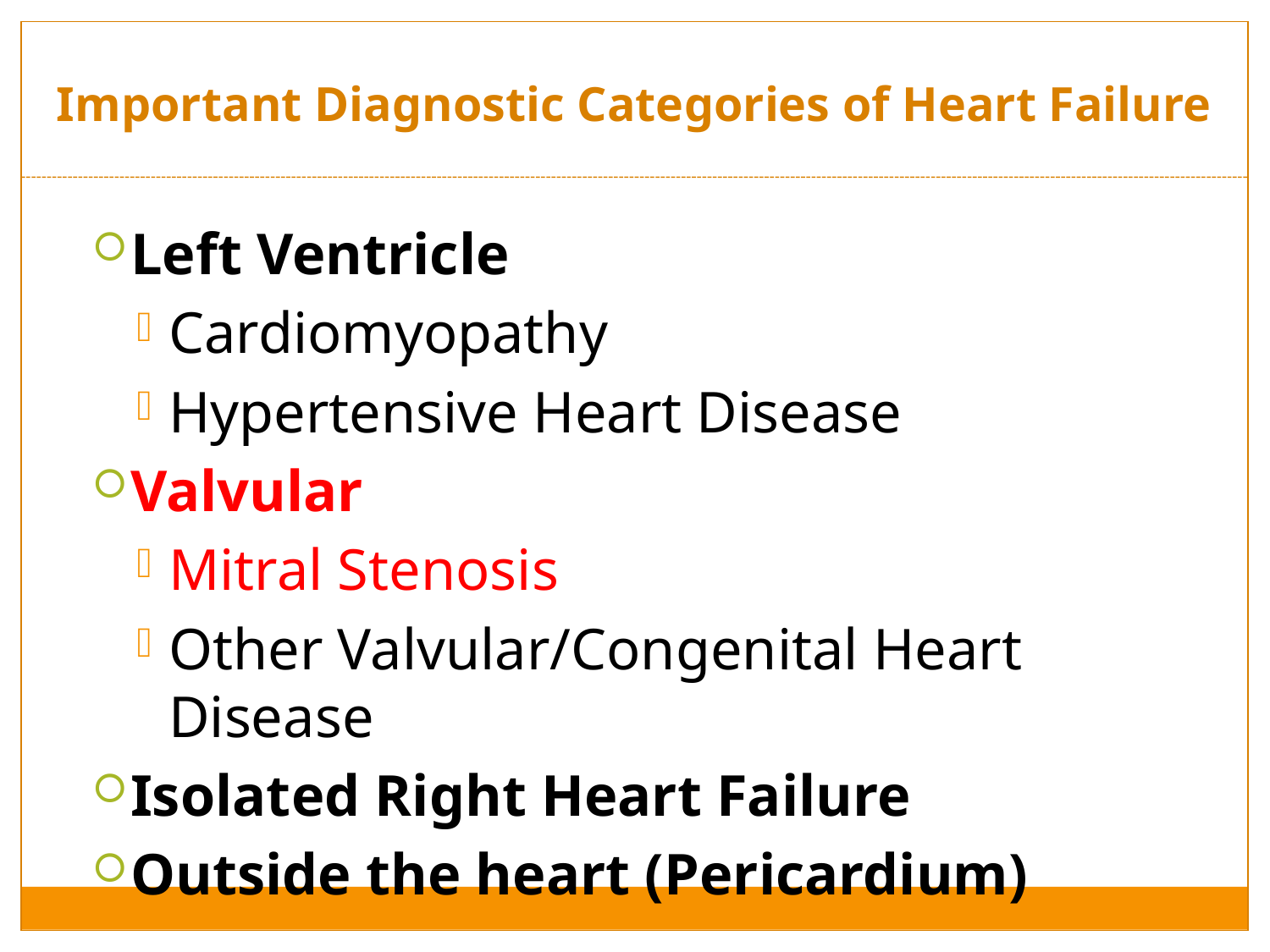

# Important Diagnostic Categories of Heart Failure
Left Ventricle
Cardiomyopathy
Hypertensive Heart Disease
Valvular
Mitral Stenosis
Other Valvular/Congenital Heart Disease
Isolated Right Heart Failure
Outside the heart (Pericardium)

## Slide 38
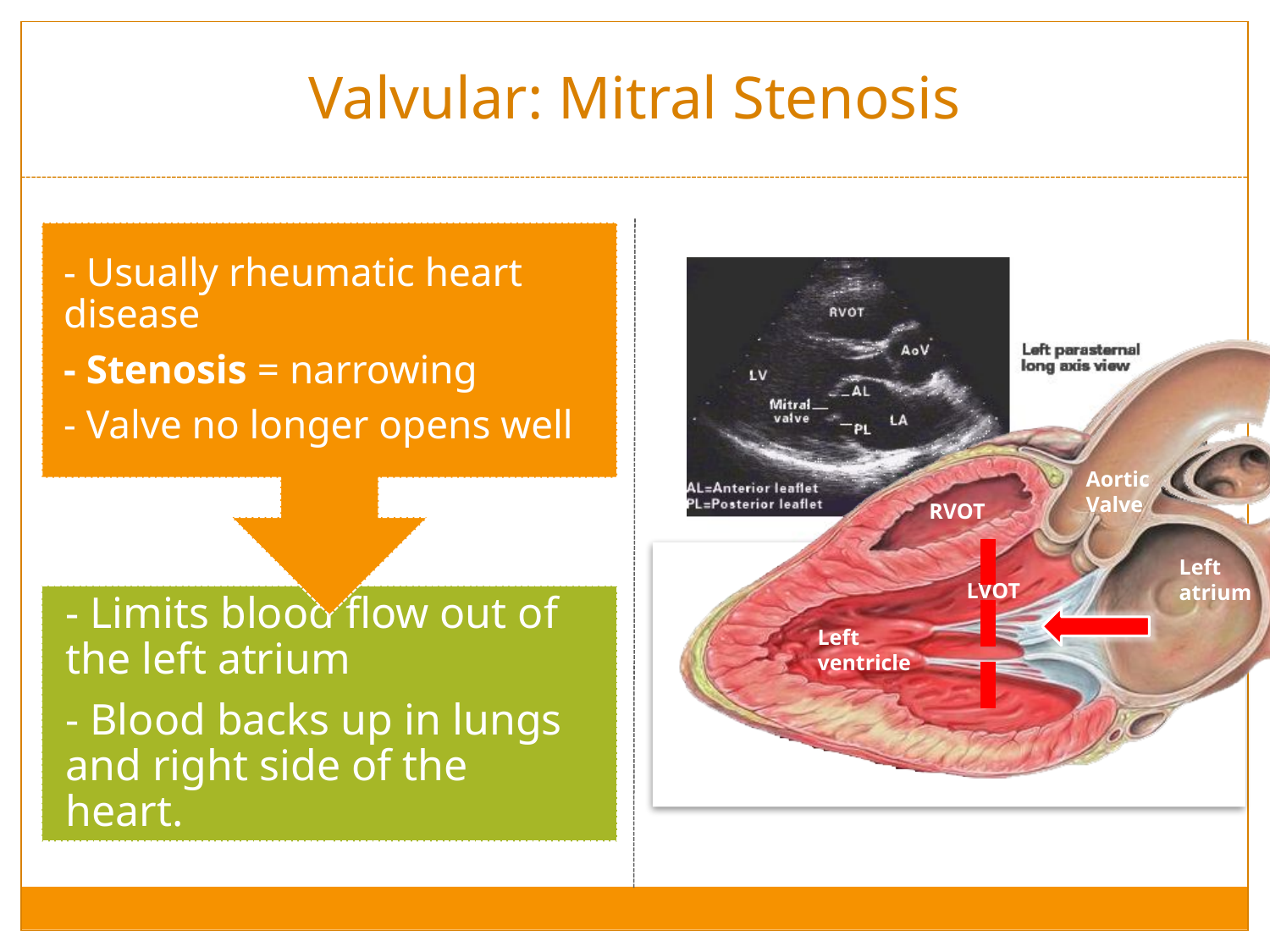

# Valvular: Mitral Stenosis
Aortic Valve
Aortic Valve
RVOT
RVOT
Left atrium
Left atrium
LVOT
LVOT
Left ventricle
Left ventricle

## Slide 39
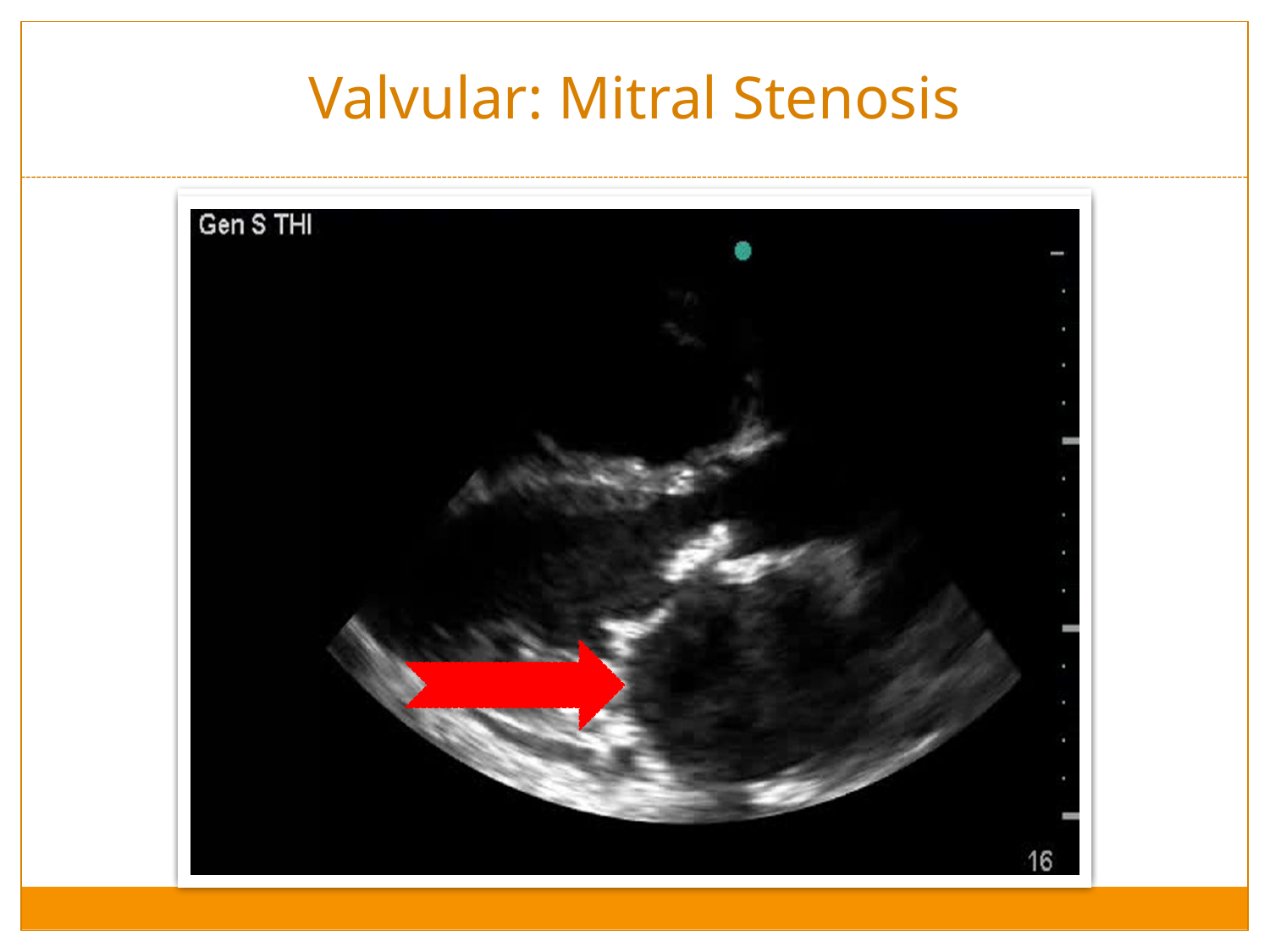

# Valvular: Mitral Stenosis

## Slide 40
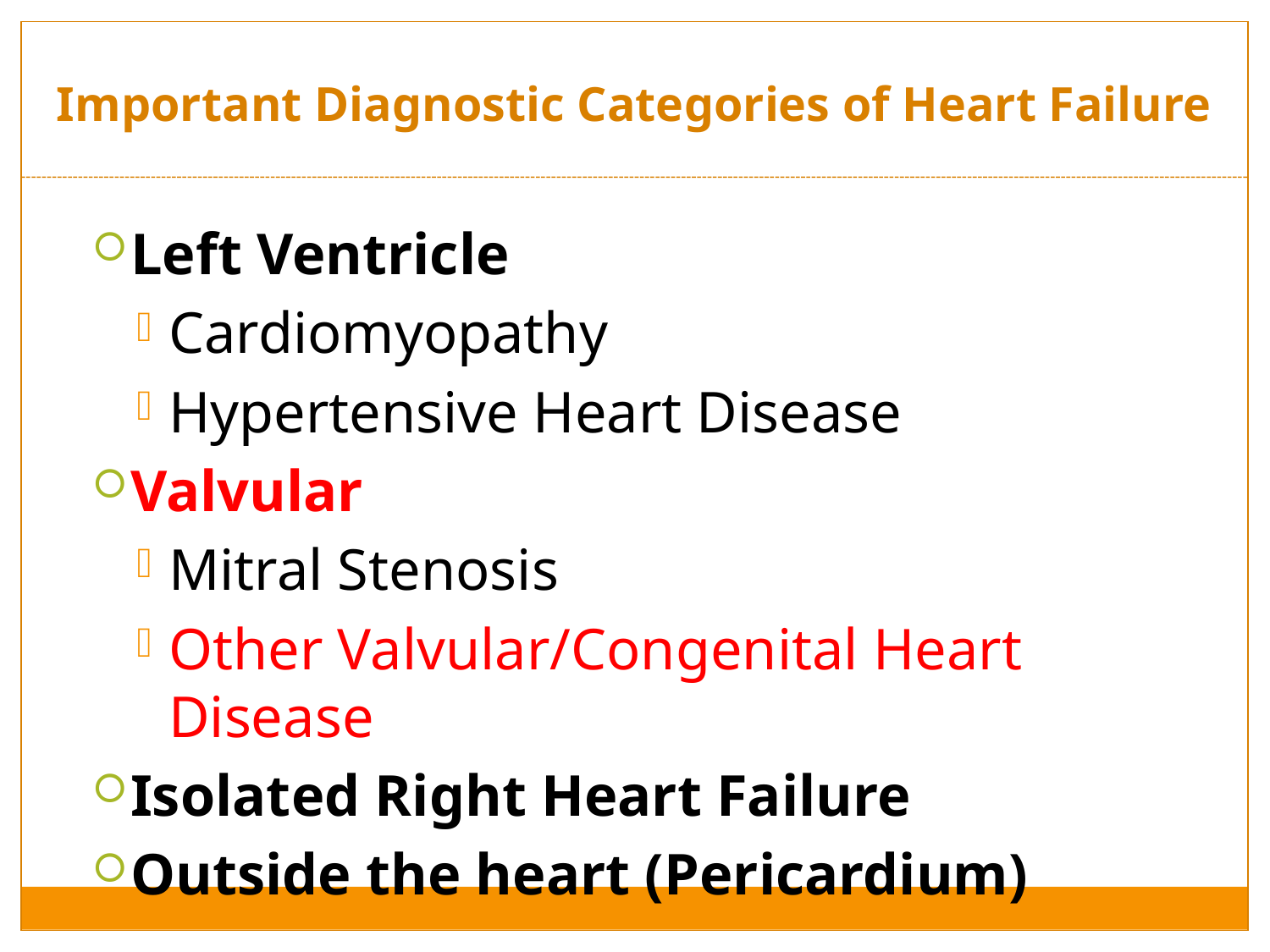

# Important Diagnostic Categories of Heart Failure
Left Ventricle
Cardiomyopathy
Hypertensive Heart Disease
Valvular
Mitral Stenosis
Other Valvular/Congenital Heart Disease
Isolated Right Heart Failure
Outside the heart (Pericardium)

## Slide 41
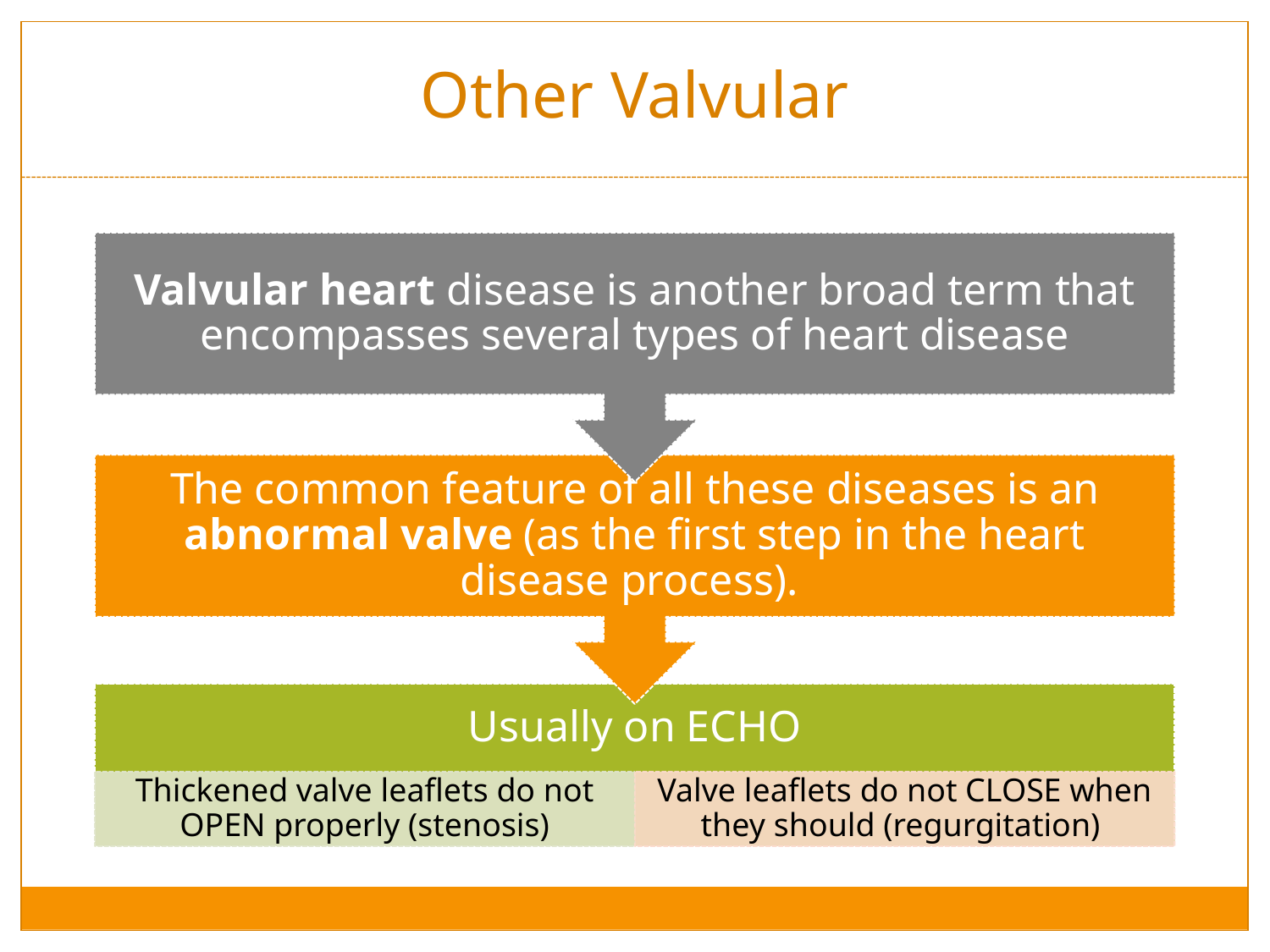

# Other Valvular

## Slide 42
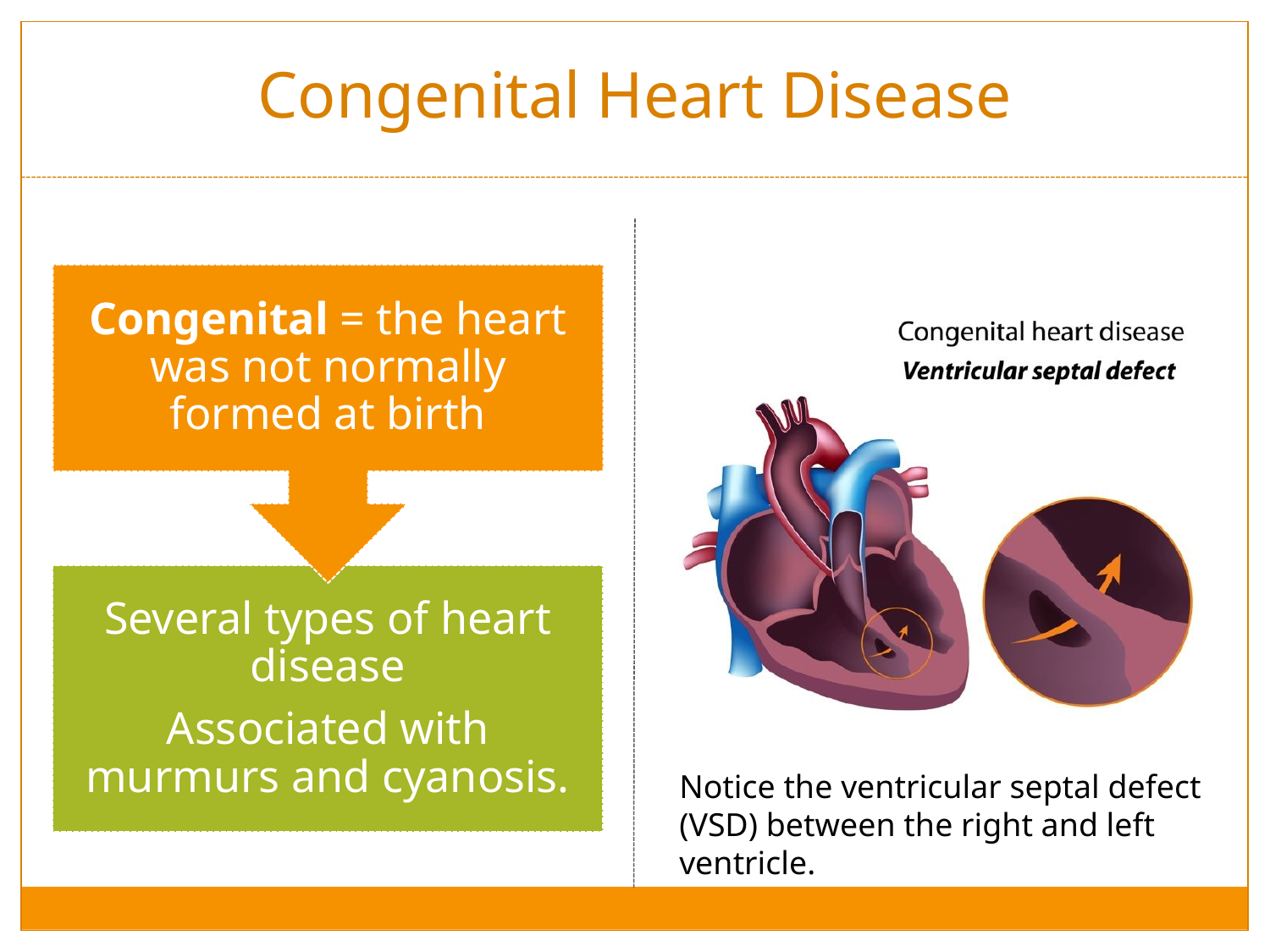

# Congenital Heart Disease
Notice the ventricular septal defect (VSD) between the right and left ventricle.

## Slide 43
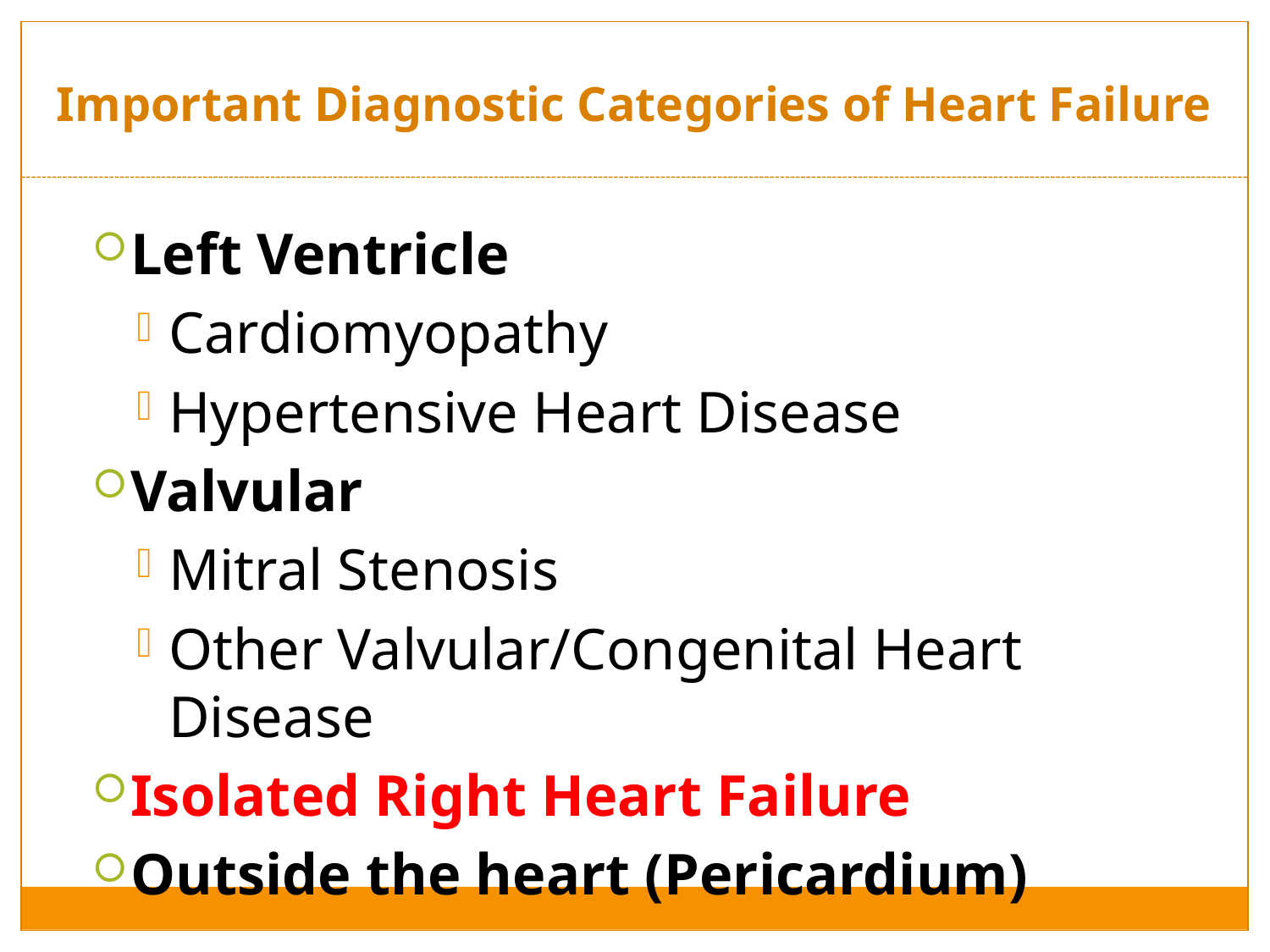

# Important Diagnostic Categories of Heart Failure
Left Ventricle
Cardiomyopathy
Hypertensive Heart Disease
Valvular
Mitral Stenosis
Other Valvular/Congenital Heart Disease
Isolated Right Heart Failure
Outside the heart (Pericardium)

## Slide 44
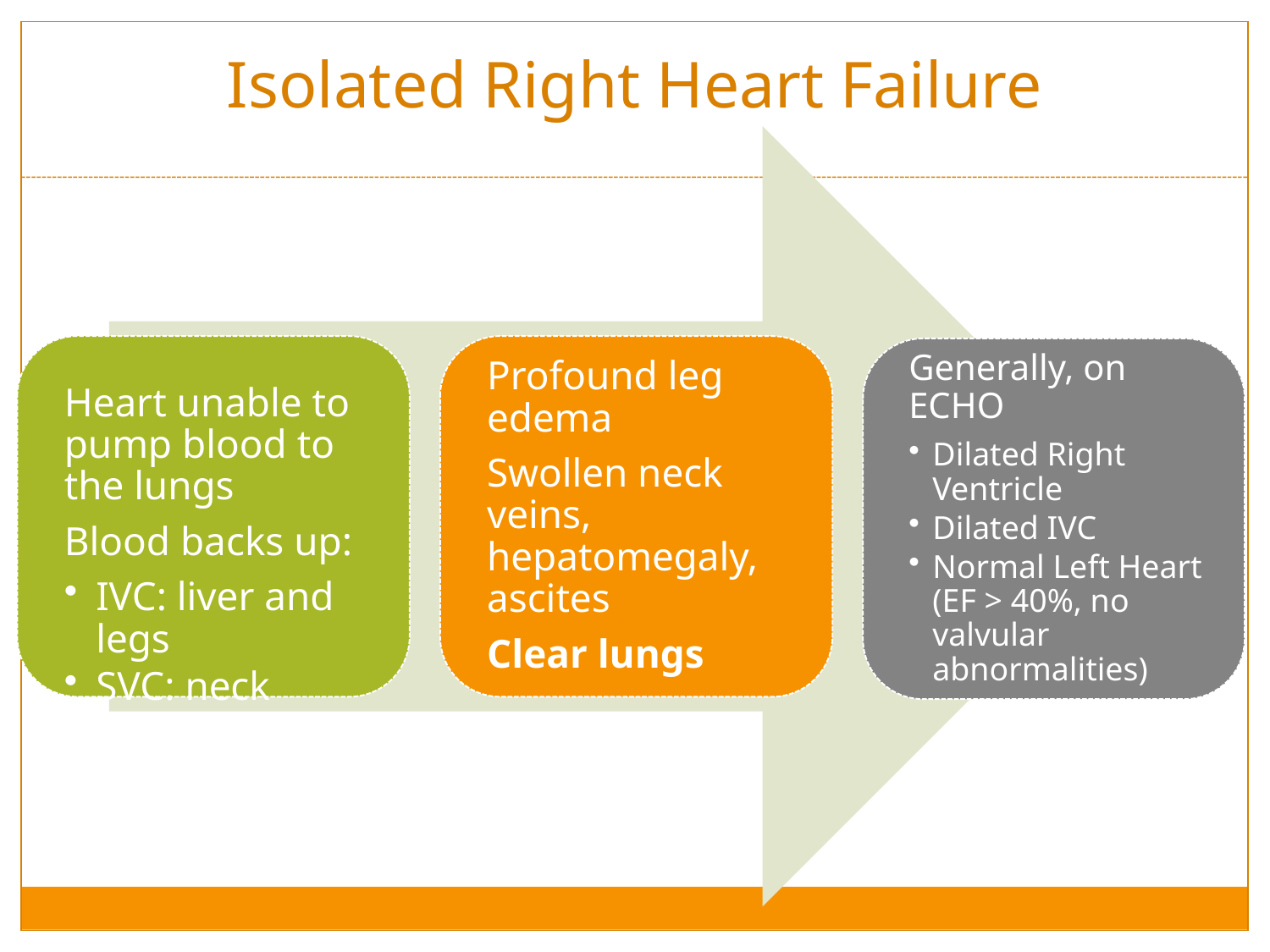

# Isolated Right Heart Failure

## Slide 45
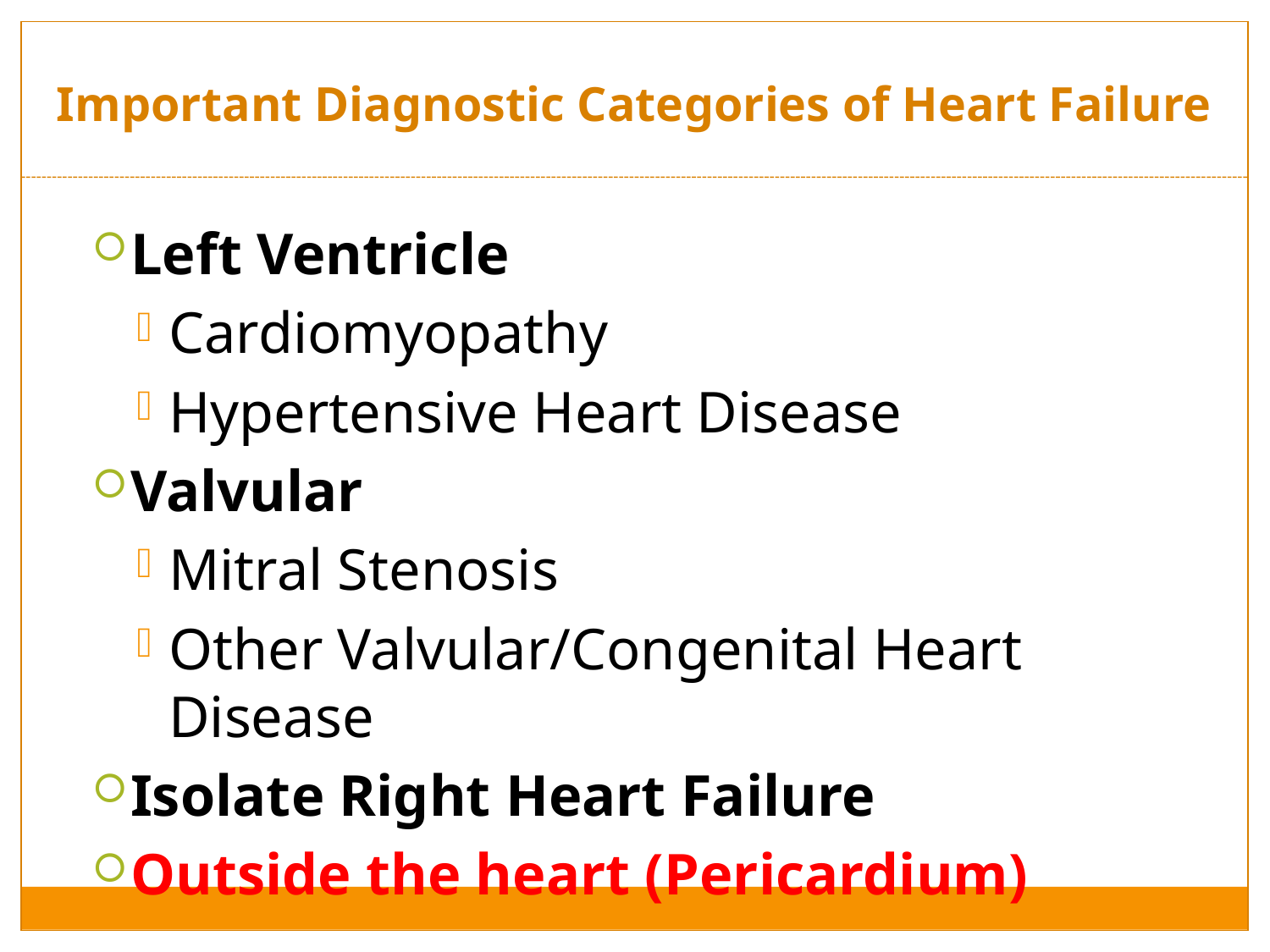

# Important Diagnostic Categories of Heart Failure
Left Ventricle
Cardiomyopathy
Hypertensive Heart Disease
Valvular
Mitral Stenosis
Other Valvular/Congenital Heart Disease
Isolate Right Heart Failure
Outside the heart (Pericardium)

## Slide 46
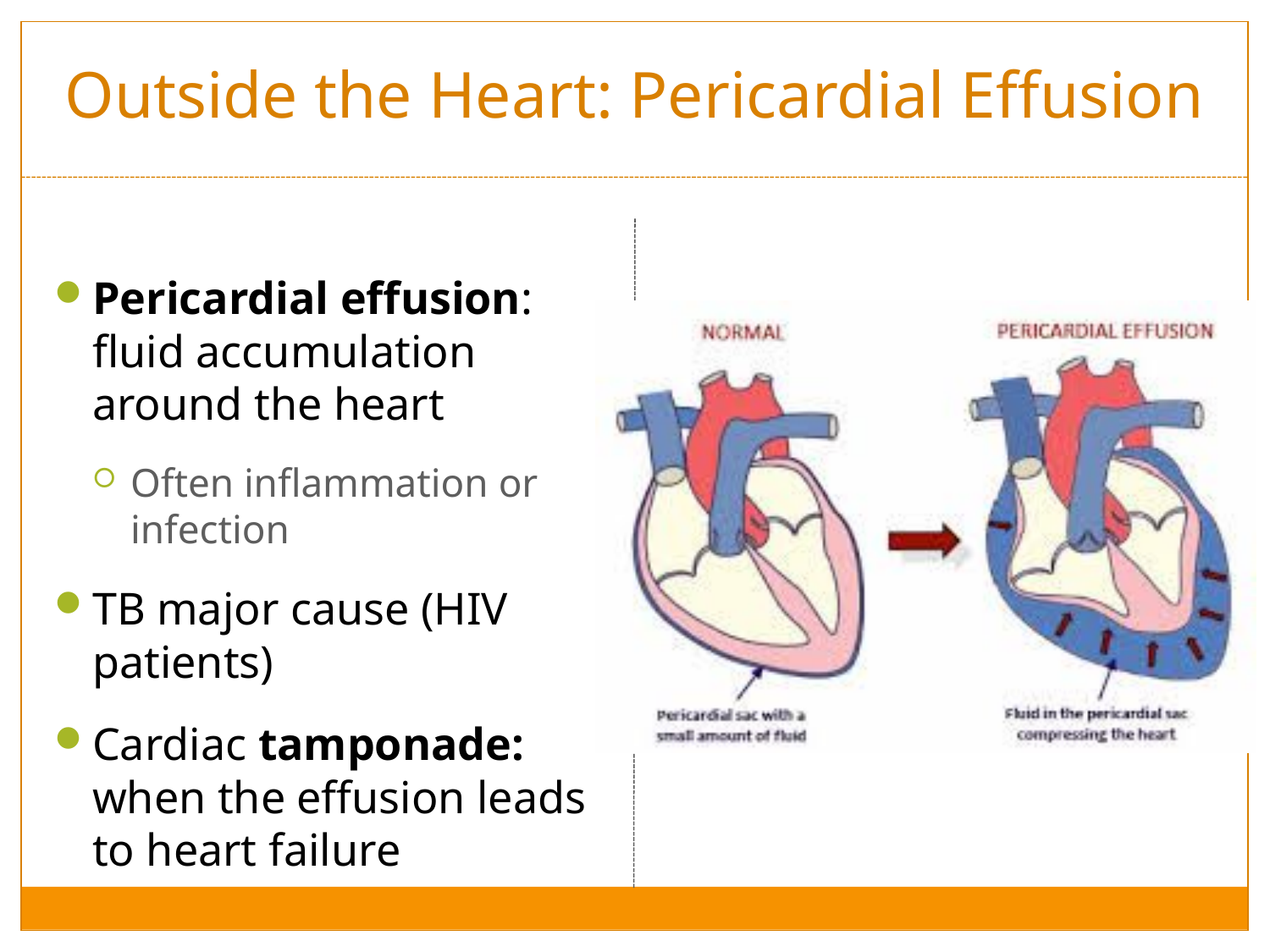

# Outside the Heart: Pericardial Effusion
Pericardial effusion: fluid accumulation around the heart
Often inflammation or infection
TB major cause (HIV patients)
Cardiac tamponade: when the effusion leads to heart failure

## Slide 47
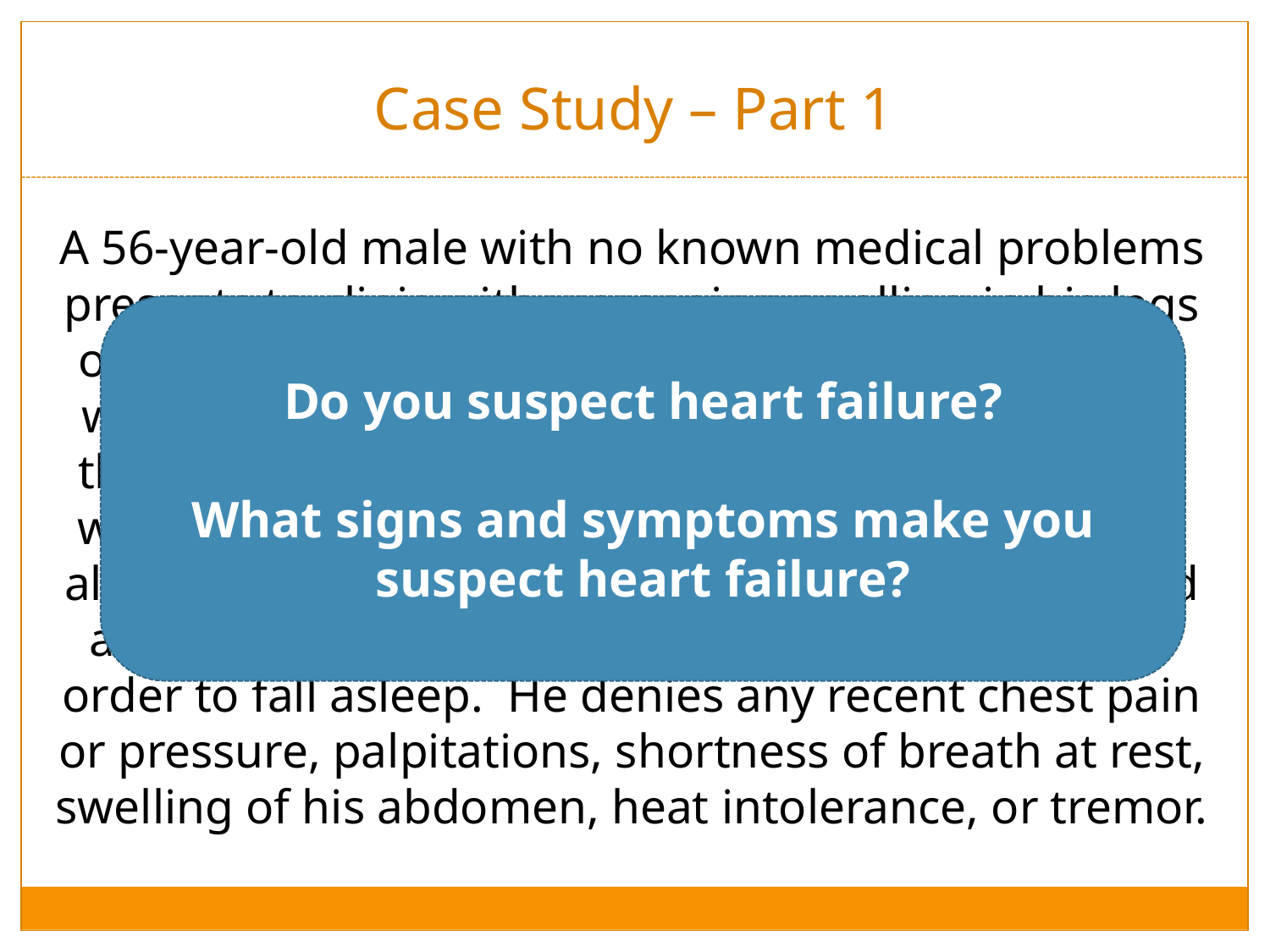

# Case Study – Part 1
A 56-year-old male with no known medical problems presents to clinic with worsening swelling in his legs over the past month.  Until recently, he was able to walk several kilometers without stopping, but over the past three weeks he has become fatigued after walking much shorter distances or up hills.  He has also felt short of breath or coughs while lying in bed at night, and currently needs to use two pillows in order to fall asleep.  He denies any recent chest pain or pressure, palpitations, shortness of breath at rest, swelling of his abdomen, heat intolerance, or tremor.
Do you suspect heart failure?
What signs and symptoms make you suspect heart failure?

## Slide 48
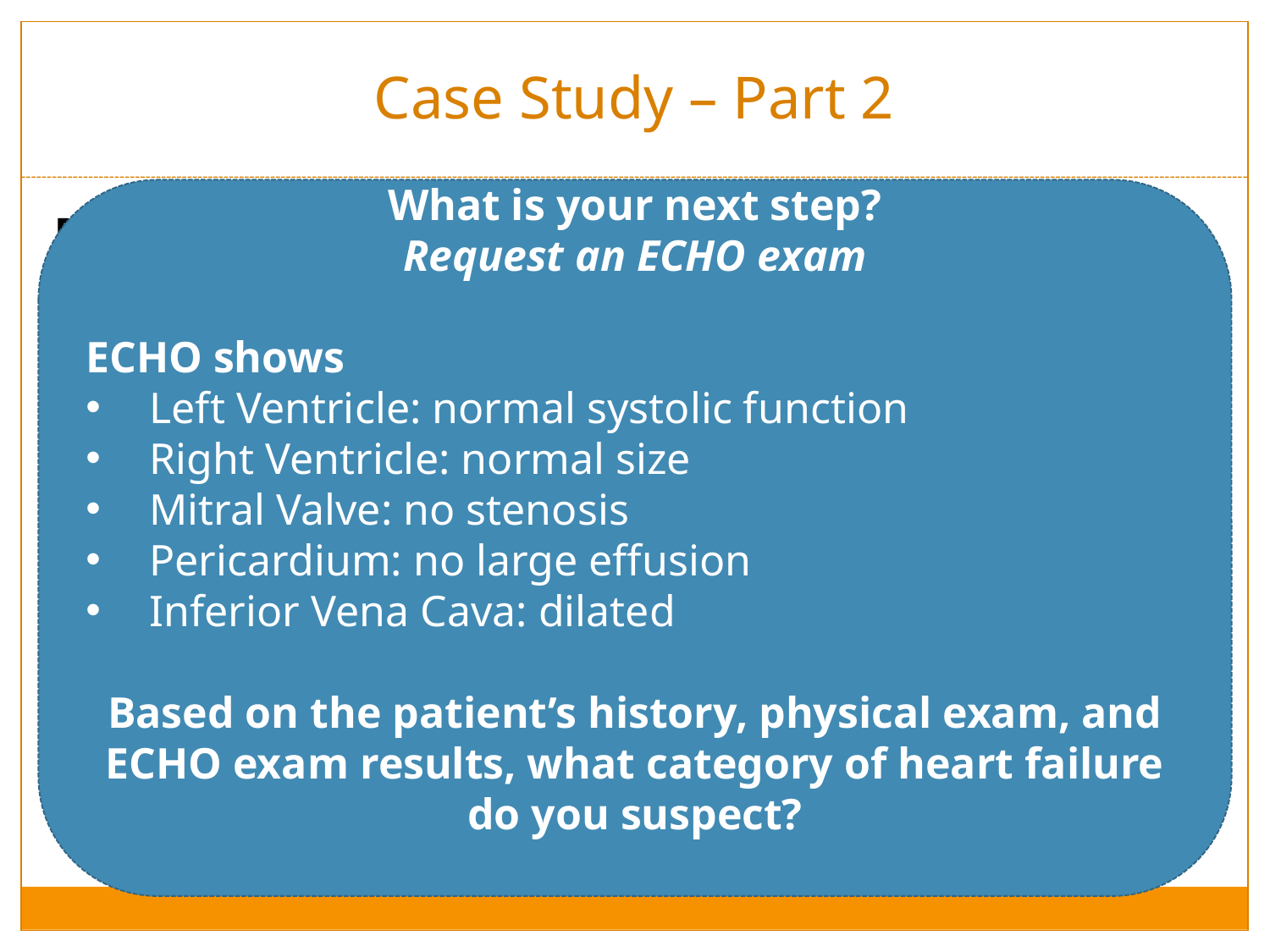

# Case Study – Part 2
What is your next step?
Request an ECHO exam
ECHO shows
Left Ventricle: normal systolic function
Right Ventricle: normal size
Mitral Valve: no stenosis
Pericardium: no large effusion
Inferior Vena Cava: dilated
Based on the patient’s history, physical exam, and ECHO exam results, what category of heart failure do you suspect?
Physical Exam
Temperature 37.1oC, BP 185/110 mmHg, Pulse 80 beats per minute, Respiratory rate 12 breaths per minute , oxygen saturation of 95% on room air, weight 75 kg
Neck veins visibly distended to 3cm above the clavicle in the upright position
Cardiac exam with regular rate and rhythm and no loud murmers
Lung exam with crackles at the bases bilaterally; Good air movement throughout; No wheezes
Extremities are warm and well-perfused; Pitting edema is present to the knees bilaterally

## Slide 49
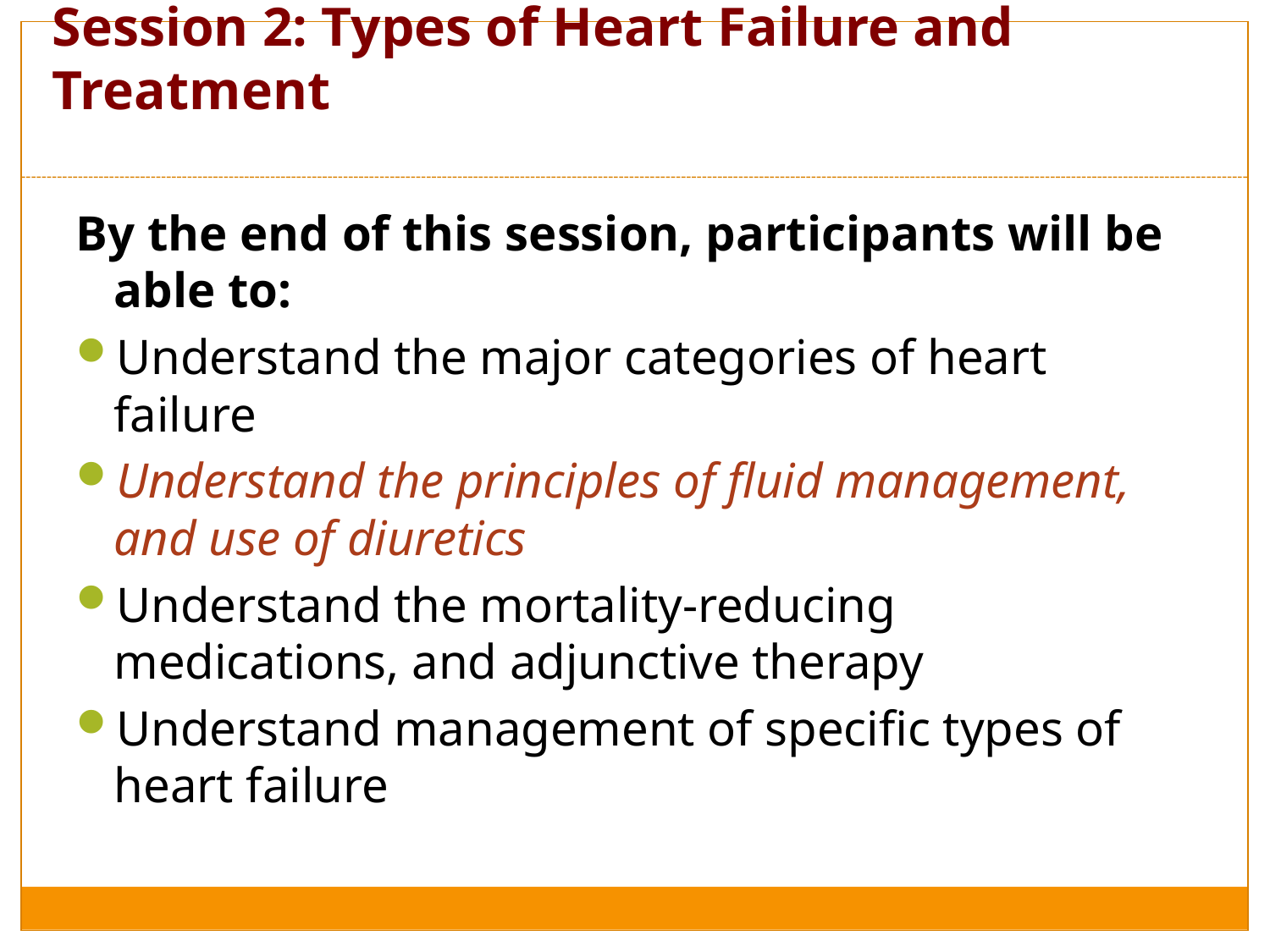

# Session 2: Types of Heart Failure and Treatment
By the end of this session, participants will be able to:
Understand the major categories of heart failure
Understand the principles of fluid management, and use of diuretics
Understand the mortality-reducing medications, and adjunctive therapy
Understand management of specific types of heart failure

## Slide 50
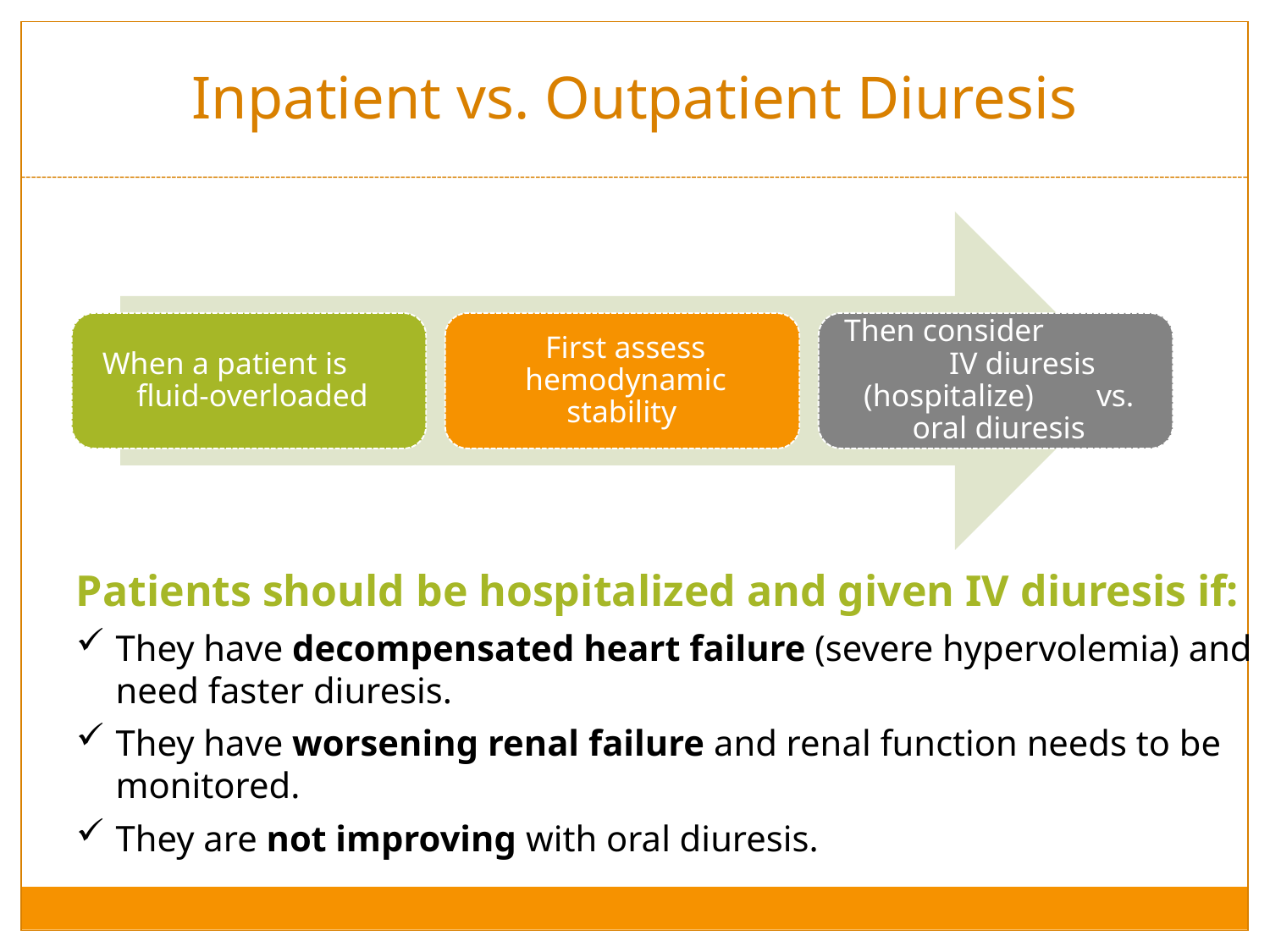

# Inpatient vs. Outpatient Diuresis
Patients should be hospitalized and given IV diuresis if:
They have decompensated heart failure (severe hypervolemia) and need faster diuresis.
They have worsening renal failure and renal function needs to be monitored.
They are not improving with oral diuresis.

## Slide 51
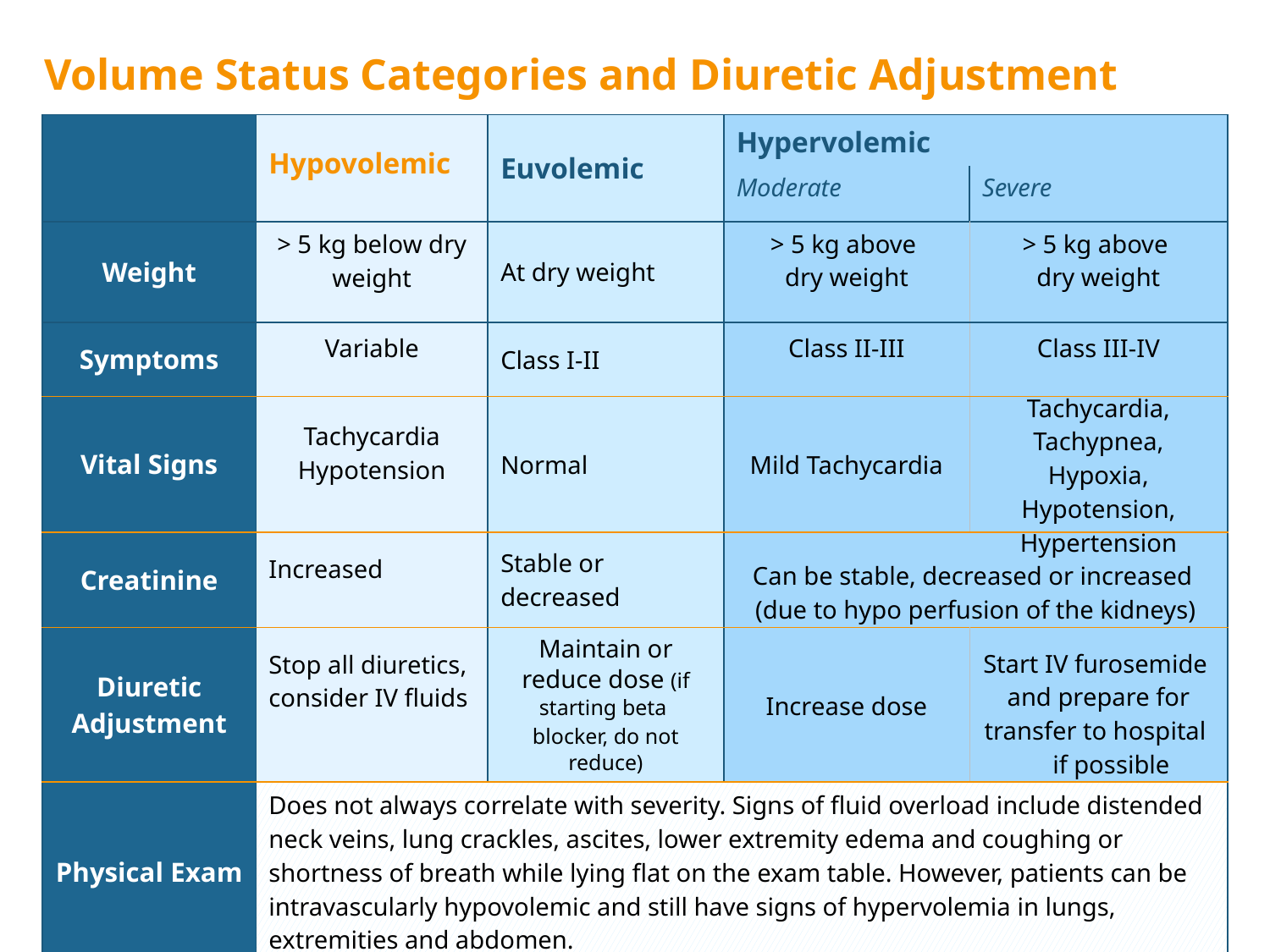

Volume Status Categories and Diuretic Adjustment
| | | Euvolemic | | |
| --- | --- | --- | --- | --- |
| | | | | |
| Weight | | At dry weight | | |
| Symptoms | | Class I-II | | |
| Vital Signs | | Normal | | |
| Creatinine | | Stable or decreased | | |
| Diuretic Adjustment | | Maintain or reduce dose (if starting beta blocker, do not reduce) | | |
| Physical Exam | Does not always correlate with severity. Signs of fluid overload include distended neck veins, lung crackles, ascites, lower extremity edema and coughing or shortness of breath while lying flat on the exam table. However, patients can be intravascularly hypovolemic and still have signs of hypervolemia in lungs, extremities and abdomen. | | | |
| | | | Hypervolemic | |
| --- | --- | --- | --- | --- |
| | | | Moderate | Severe |
| | | | > 5 kg above dry weight | > 5 kg above dry weight |
| | | | Class II-III | Class III-IV |
| | | | Mild Tachycardia | Tachycardia, Tachypnea, Hypoxia, Hypotension, Hypertension |
| | | | Can be stable, decreased or increased (due to hypo perfusion of the kidneys) | |
| | | | Increase dose | Start IV furosemide and prepare for transfer to hospital if possible |
| | | | | |
| | Hypovolemic | | | |
| --- | --- | --- | --- | --- |
| | | | | |
| | > 5 kg below dry weight | | | |
| | Variable | | | |
| | Tachycardia Hypotension | | | |
| | Increased | | | |
| | Stop all diuretics, consider IV fluids | | | |
| | | | | |

## Slide 52
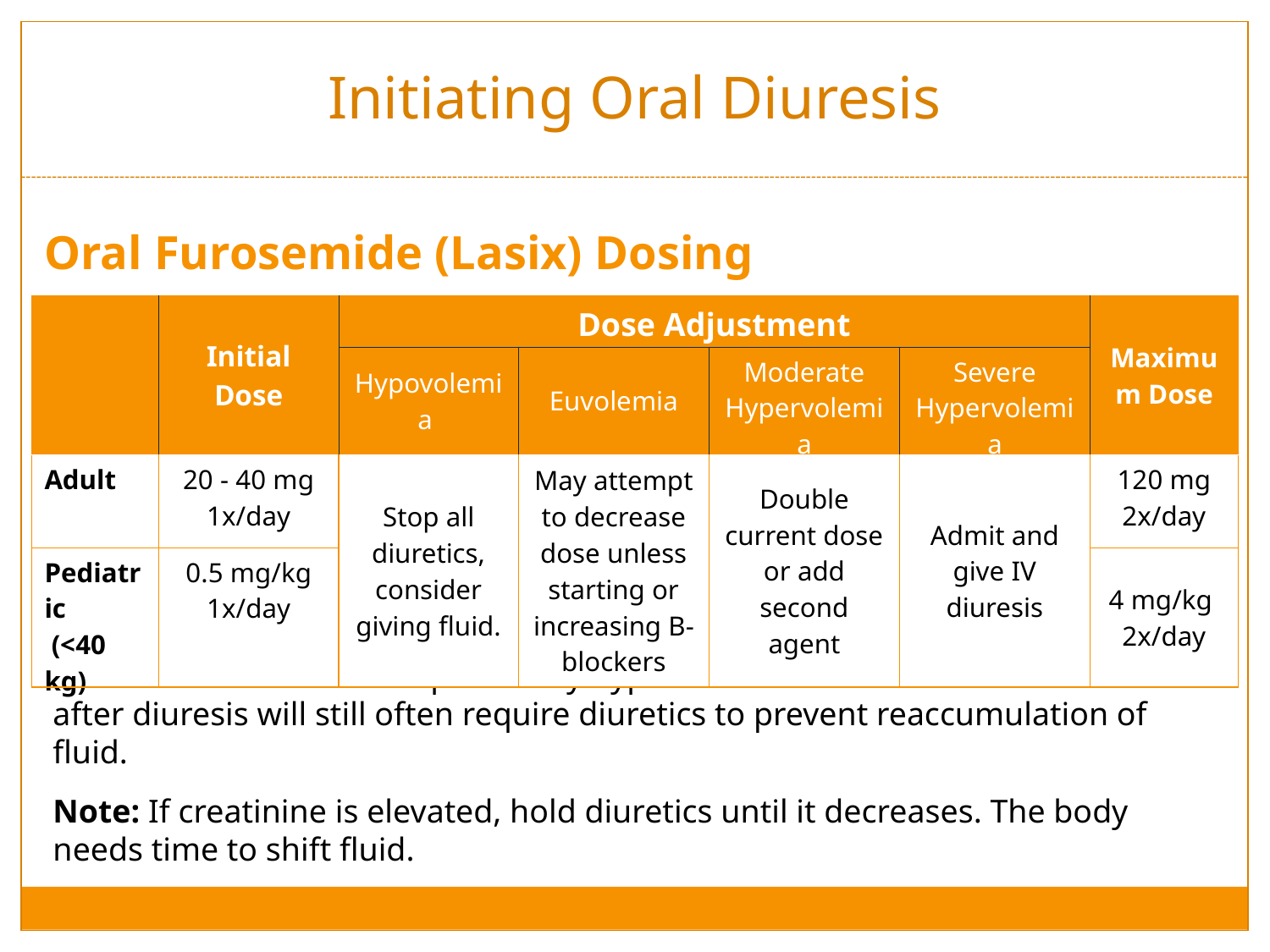

# Initiating Oral Diuresis
Oral Furosemide (Lasix) Dosing
| | Initial Dose | Dose Adjustment | | | | Maximum Dose |
| --- | --- | --- | --- | --- | --- | --- |
| | | Hypovolemia | Euvolemia | Moderate Hypervolemia | Severe Hypervolemia | |
| Adult | 20 - 40 mg 1x/day | Stop all diuretics, consider giving fluid. | May attempt to decrease dose unless starting or increasing B-blockers | Double current dose or add second agent | Admit and give IV diuresis | 120 mg 2x/day |
| Pediatric (<40 kg) | 0.5 mg/kg 1x/day | | | | | 4 mg/kg 2x/day |
Note: Patients who were previously hypervolemic but are now euvolemic after diuresis will still often require diuretics to prevent reaccumulation of fluid.
Note: If creatinine is elevated, hold diuretics until it decreases. The body needs time to shift fluid.

## Slide 53
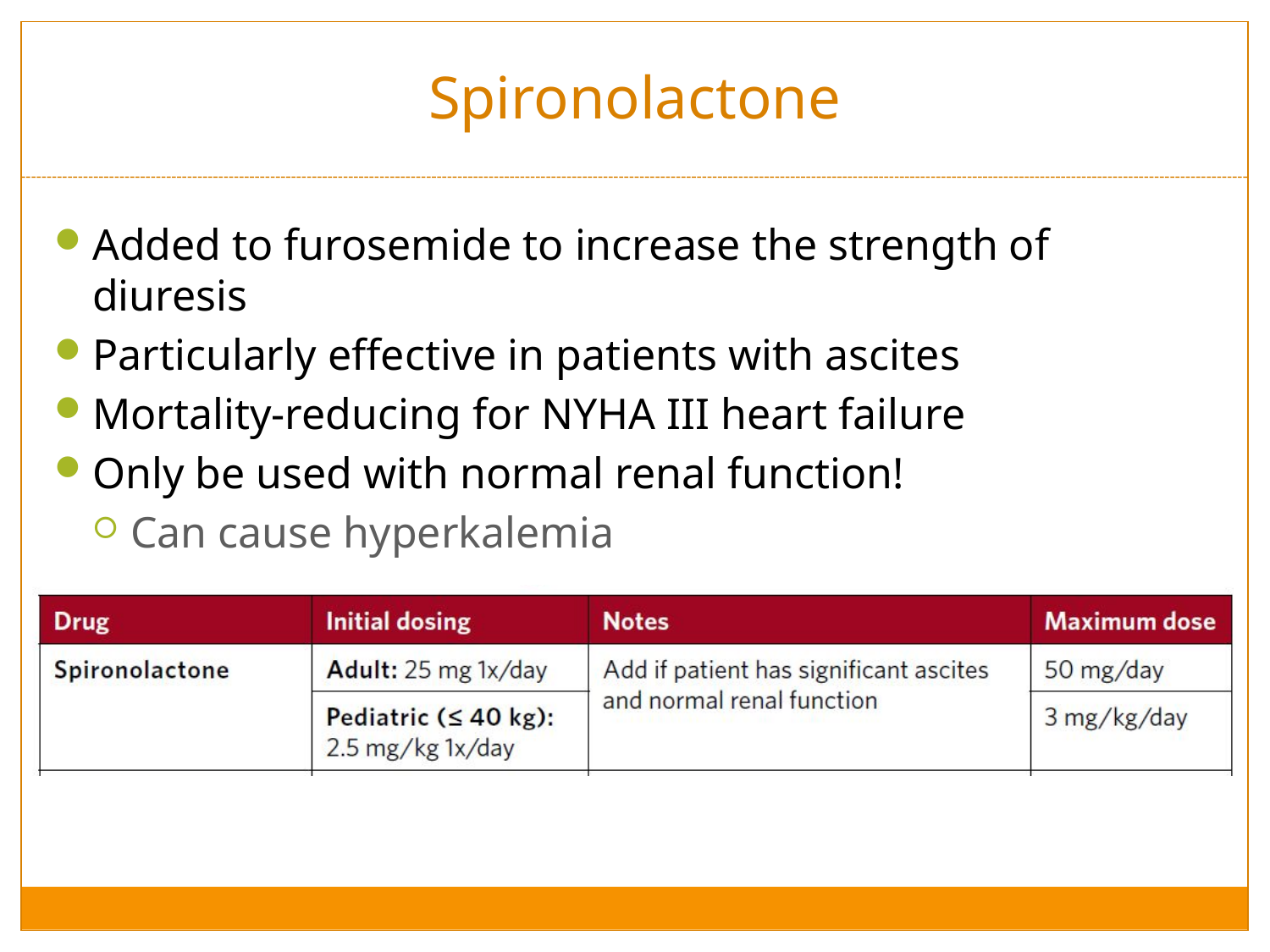

# Spironolactone
Added to furosemide to increase the strength of diuresis
Particularly effective in patients with ascites
Mortality-reducing for NYHA III heart failure
Only be used with normal renal function!
Can cause hyperkalemia

## Slide 54
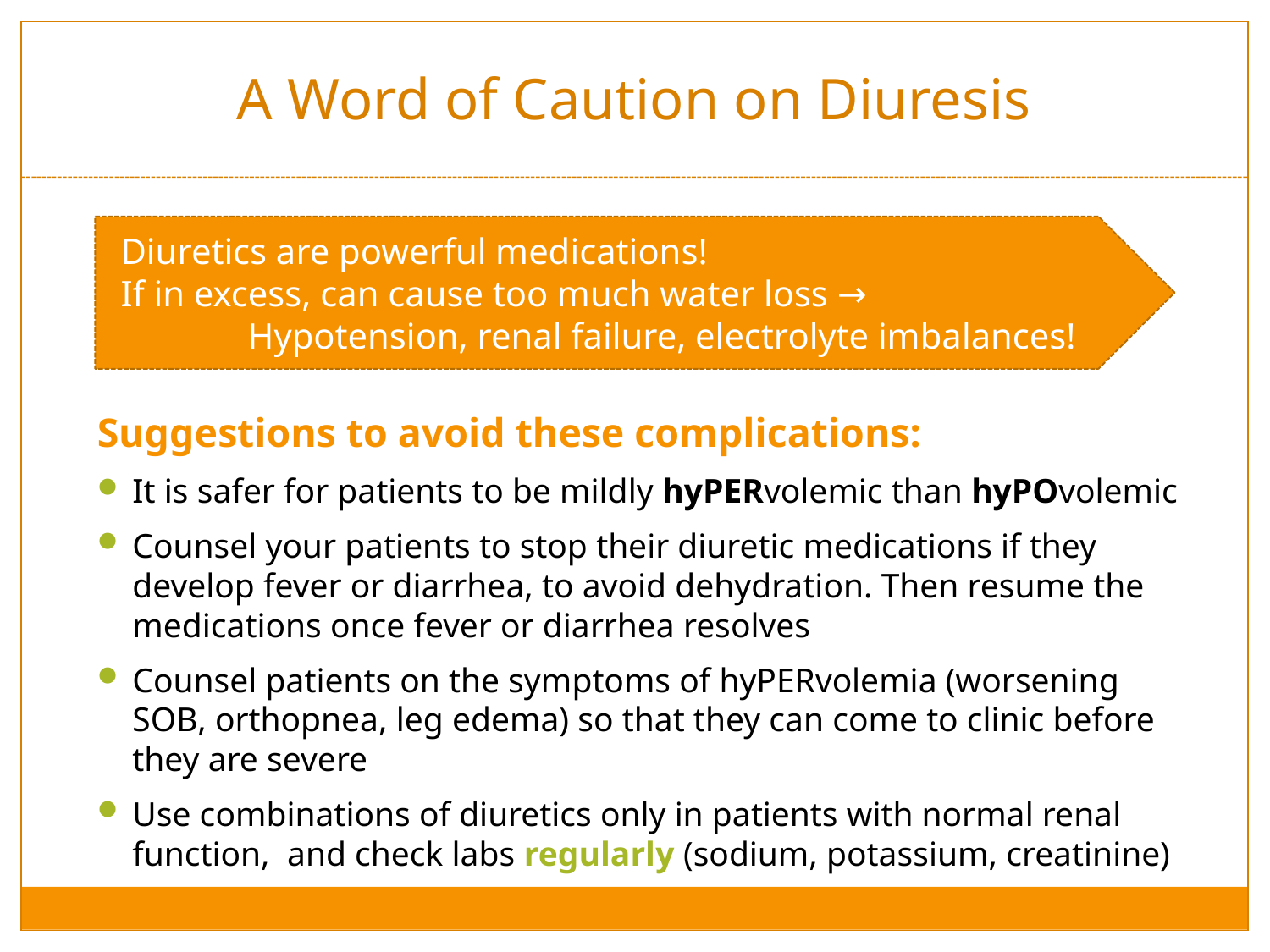

# A Word of Caution on Diuresis
Diuretics are powerful medications!
If in excess, can cause too much water loss →
	Hypotension, renal failure, electrolyte imbalances!
Suggestions to avoid these complications:
It is safer for patients to be mildly hyPERvolemic than hyPOvolemic
Counsel your patients to stop their diuretic medications if they develop fever or diarrhea, to avoid dehydration. Then resume the medications once fever or diarrhea resolves
Counsel patients on the symptoms of hyPERvolemia (worsening SOB, orthopnea, leg edema) so that they can come to clinic before they are severe
Use combinations of diuretics only in patients with normal renal function, and check labs regularly (sodium, potassium, creatinine)

## Slide 55
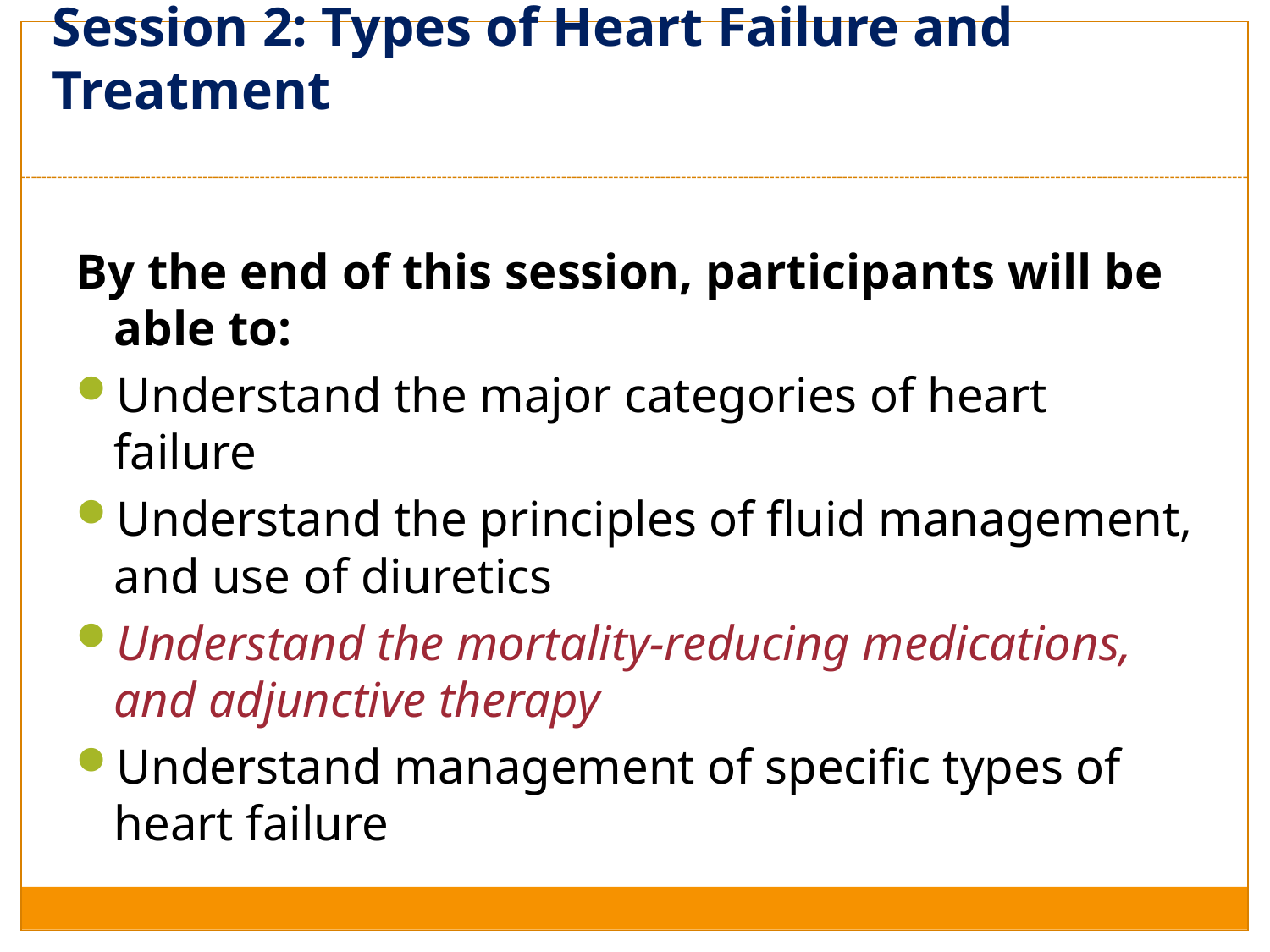

# Session 2: Types of Heart Failure and Treatment
By the end of this session, participants will be able to:
Understand the major categories of heart failure
Understand the principles of fluid management, and use of diuretics
Understand the mortality-reducing medications, and adjunctive therapy
Understand management of specific types of heart failure

## Slide 56
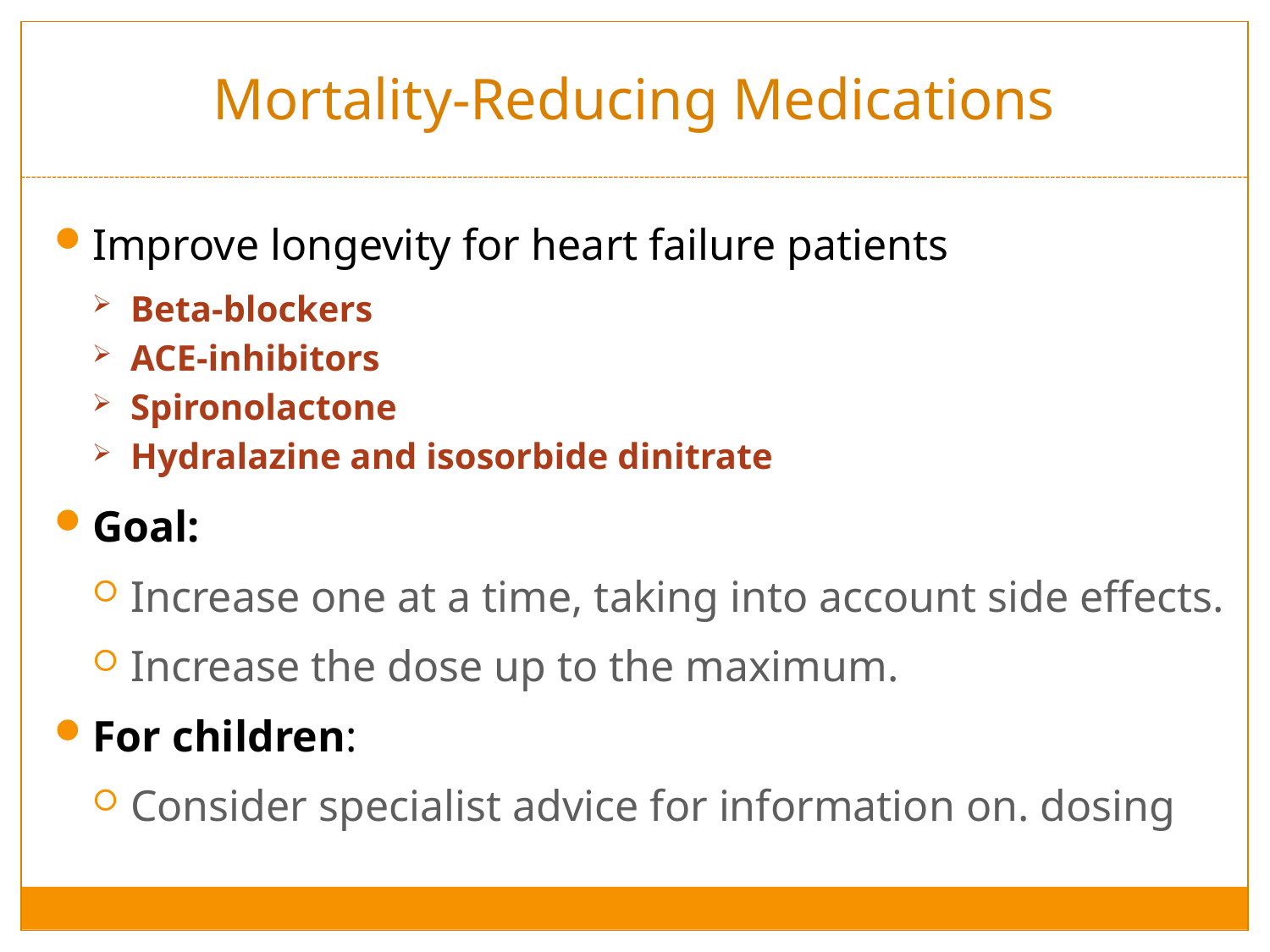

# Mortality-Reducing Medications
Improve longevity for heart failure patients
Beta-blockers
ACE-inhibitors
Spironolactone
Hydralazine and isosorbide dinitrate
Goal:
Increase one at a time, taking into account side effects.
Increase the dose up to the maximum.
For children:
Consider specialist advice for information on. dosing

## Slide 57
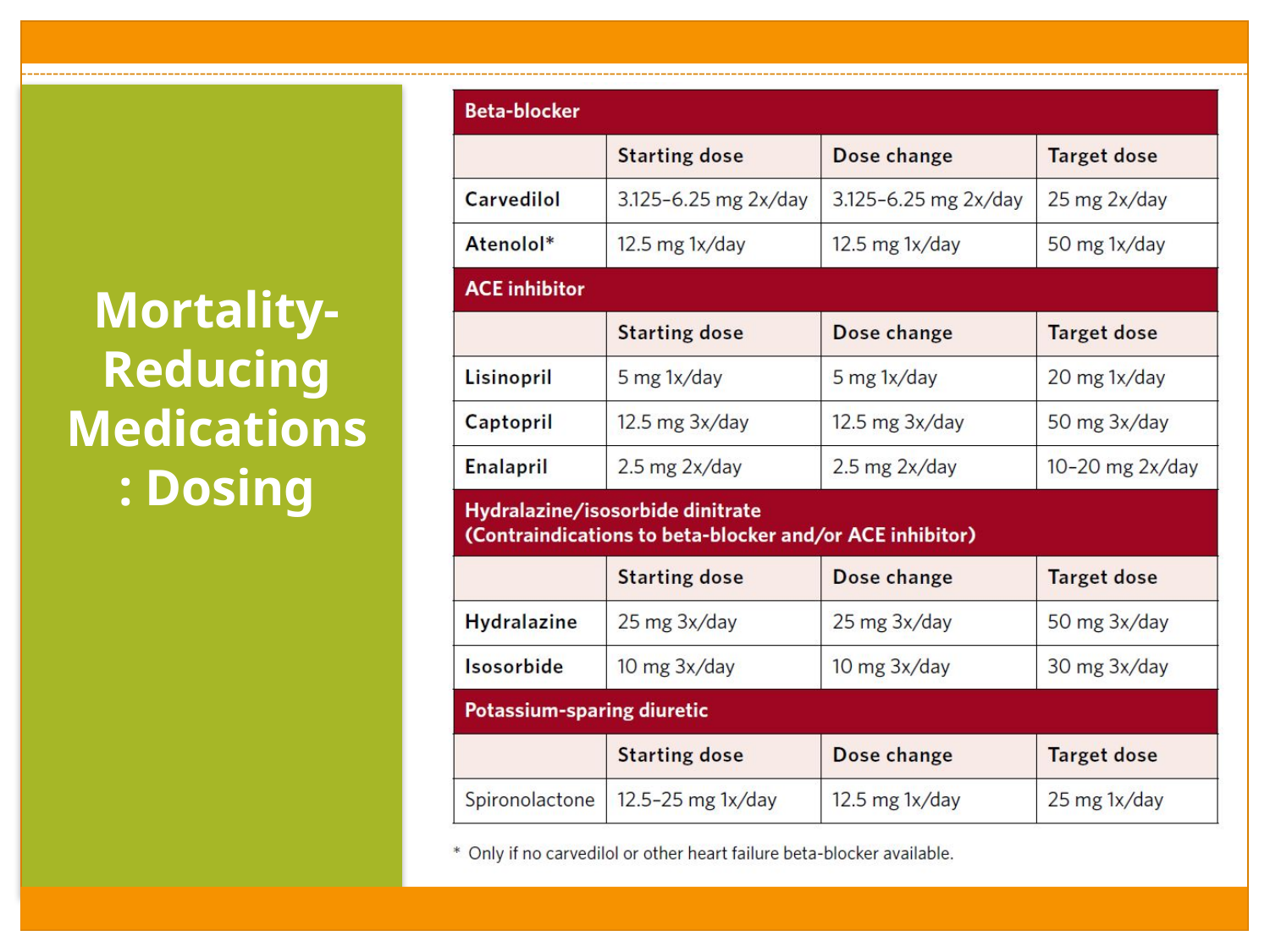

# Mortality-Reducing Medications: Dosing

## Slide 58
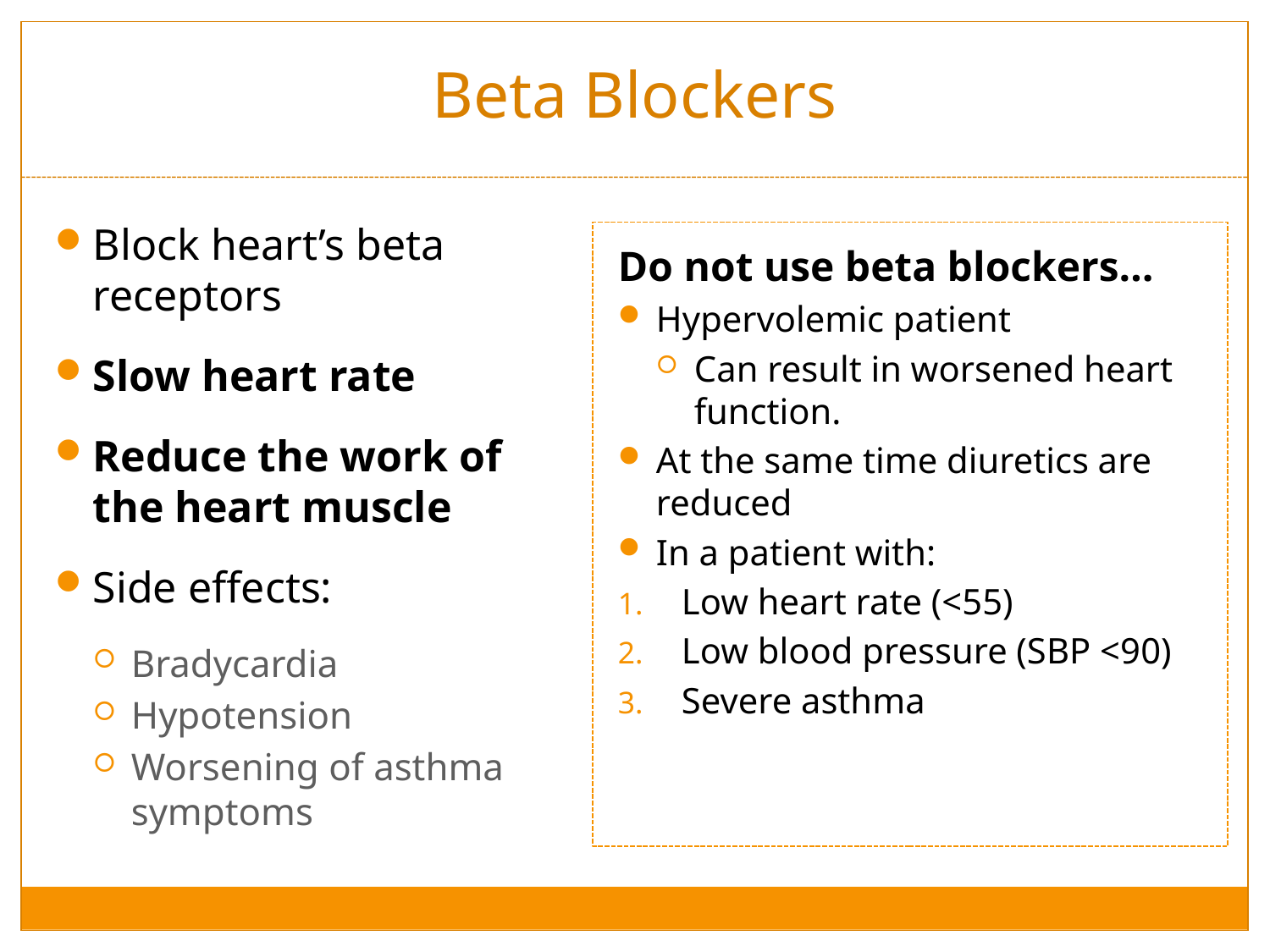

# Beta Blockers
Block heart’s beta receptors
Slow heart rate
Reduce the work of the heart muscle
Side effects:
Bradycardia
Hypotension
Worsening of asthma symptoms
Do not use beta blockers…
Hypervolemic patient
Can result in worsened heart function.
At the same time diuretics are reduced
In a patient with:
Low heart rate (<55)
Low blood pressure (SBP <90)
Severe asthma

## Slide 59
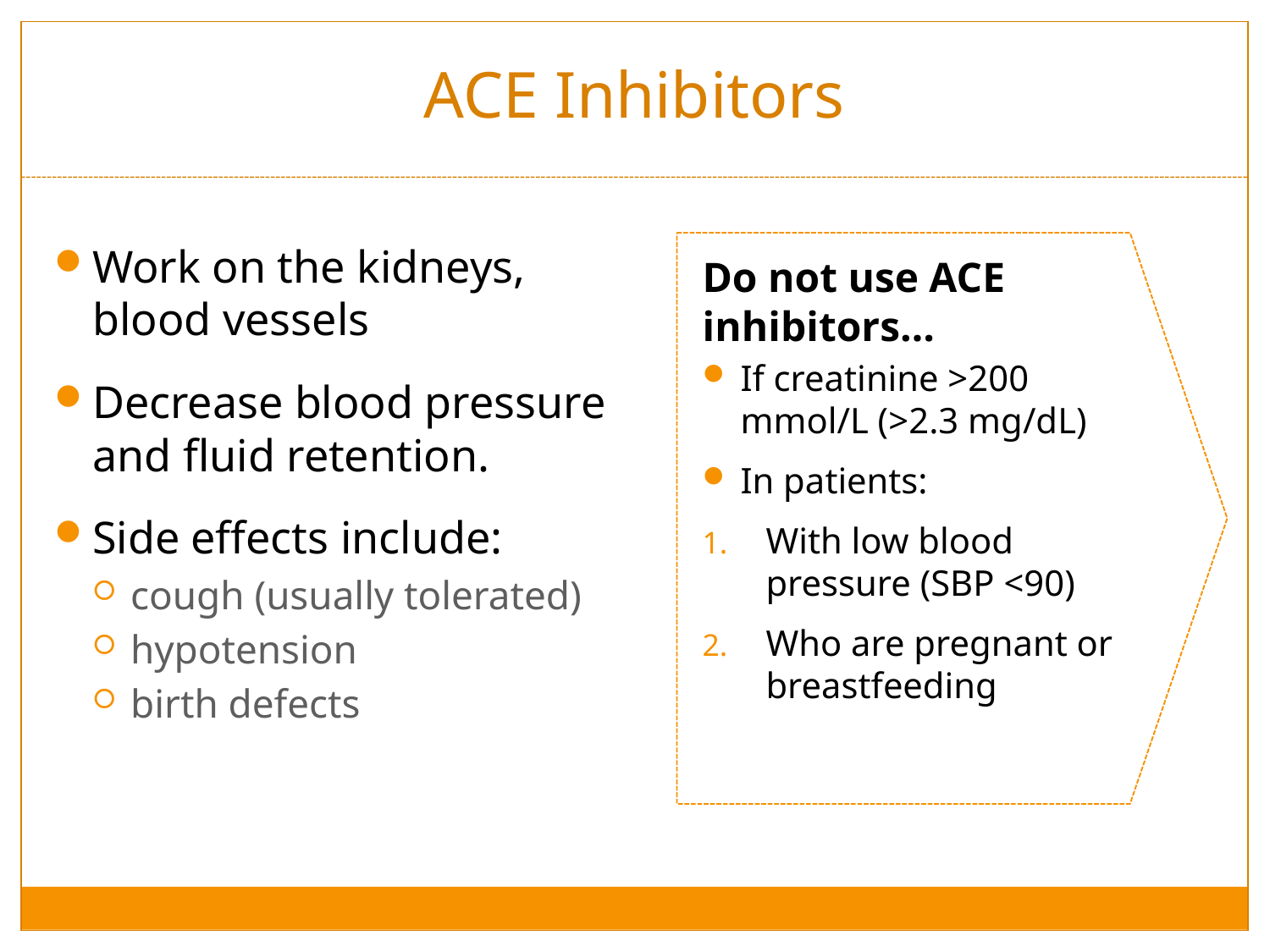

# ACE Inhibitors
Work on the kidneys, blood vessels
Decrease blood pressure and fluid retention.
Side effects include:
cough (usually tolerated)
hypotension
birth defects
Do not use ACE inhibitors…
If creatinine >200 mmol/L (>2.3 mg/dL)
In patients:
With low blood pressure (SBP <90)
Who are pregnant or breastfeeding

## Slide 60
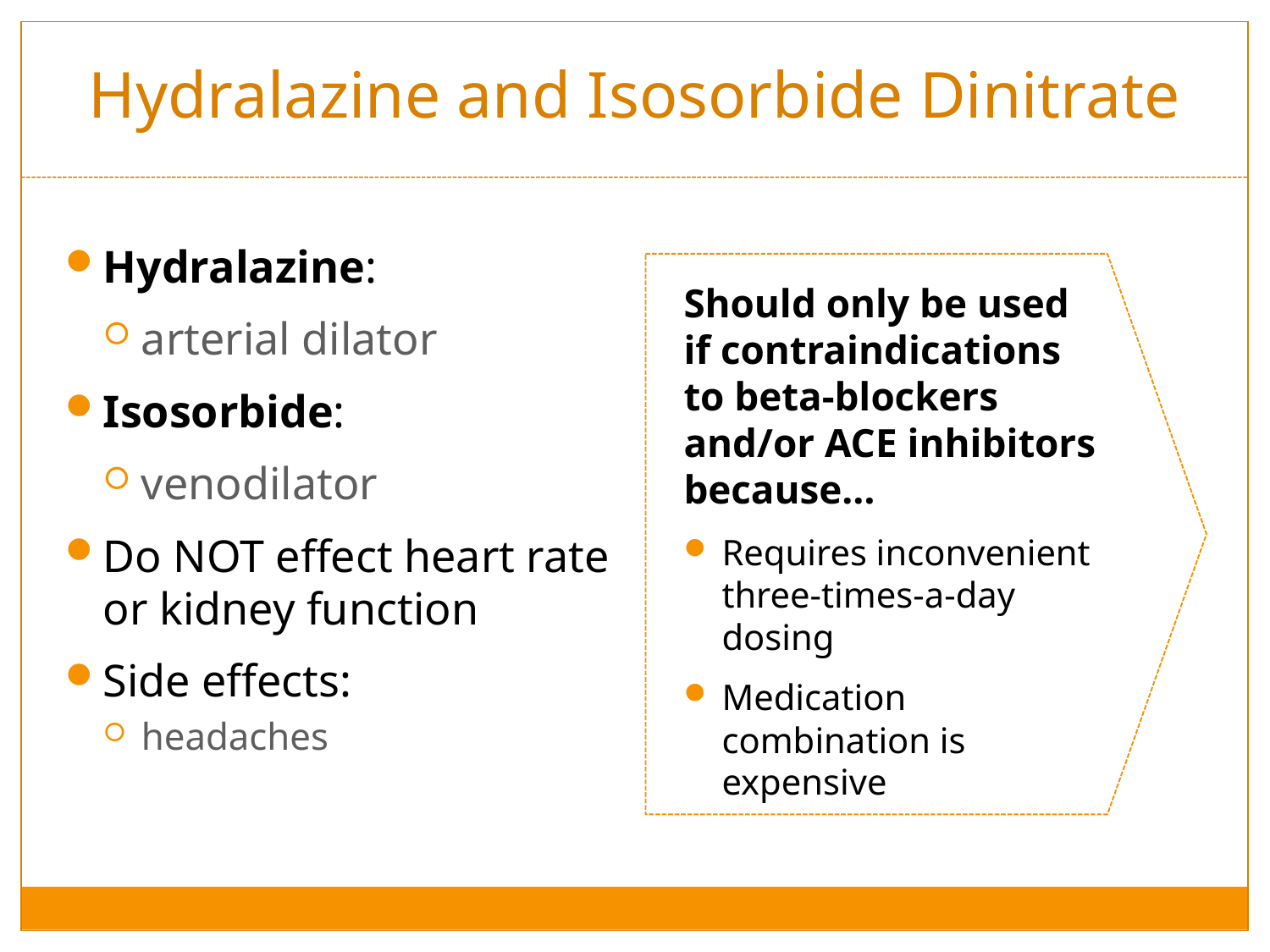

# Hydralazine and Isosorbide Dinitrate
Hydralazine:
arterial dilator
Isosorbide:
venodilator
Do NOT effect heart rate or kidney function
Side effects:
headaches
Should only be used if contraindications to beta-blockers and/or ACE inhibitors because…
Requires inconvenient three-times-a-day dosing
Medication combination is expensive

## Slide 61
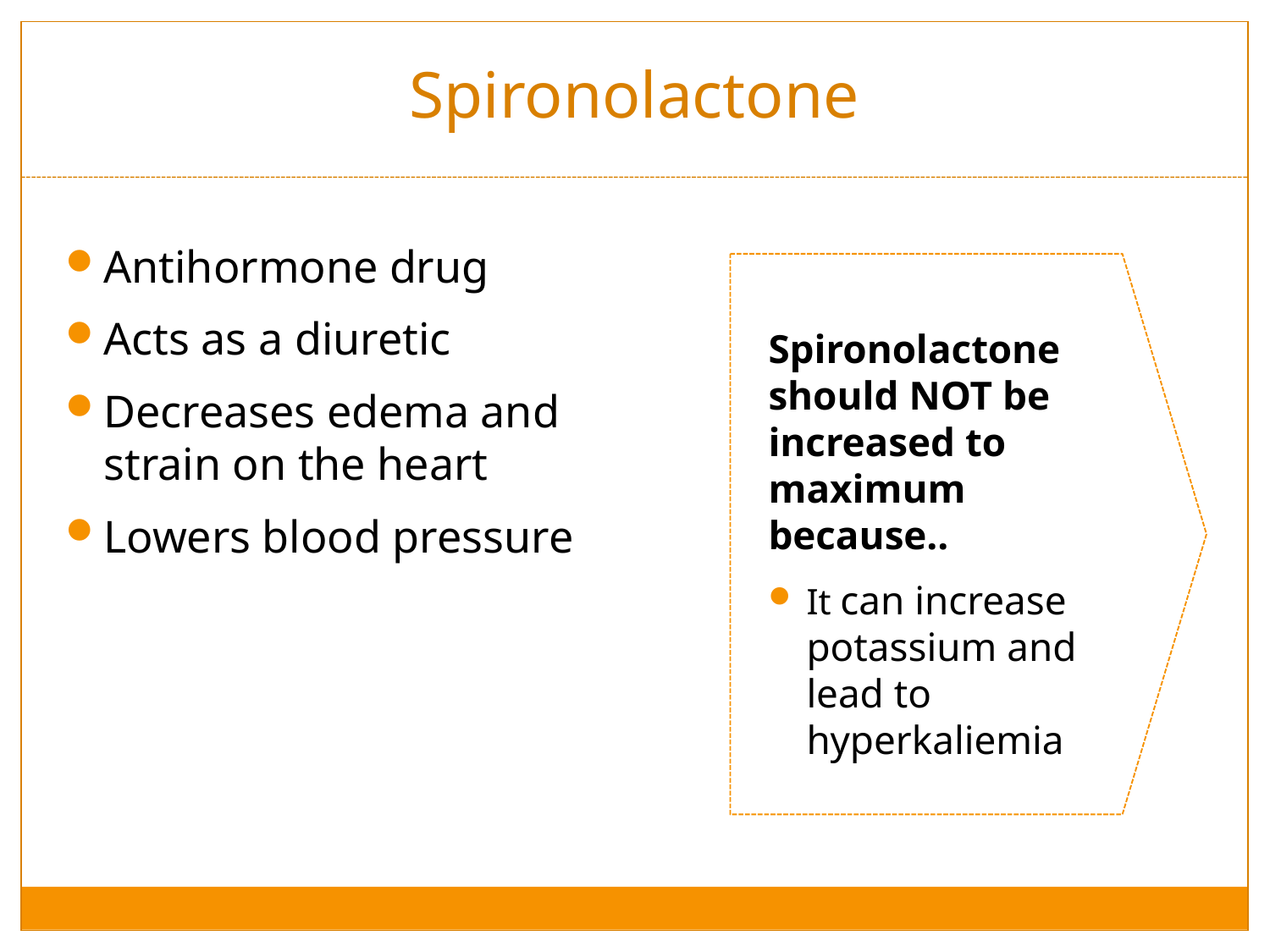

# Spironolactone
Antihormone drug
Acts as a diuretic
Decreases edema and strain on the heart
Lowers blood pressure
Spironolactone should NOT be increased to maximum because..
It can increase potassium and lead to hyperkaliemia

## Slide 62
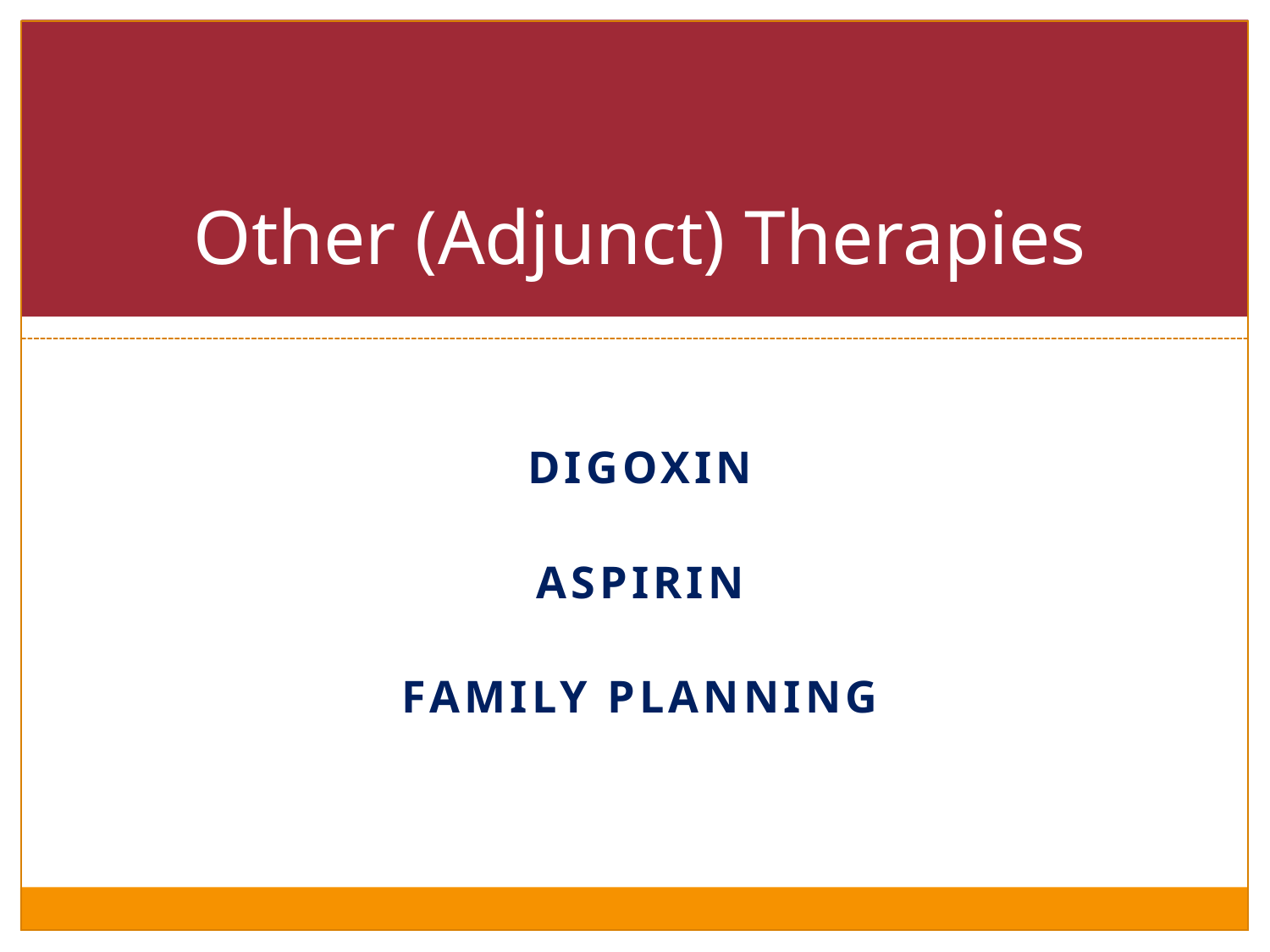

# Other (Adjunct) Therapies
Digoxin
AspIRin
FAMILY PLANNING

## Slide 63
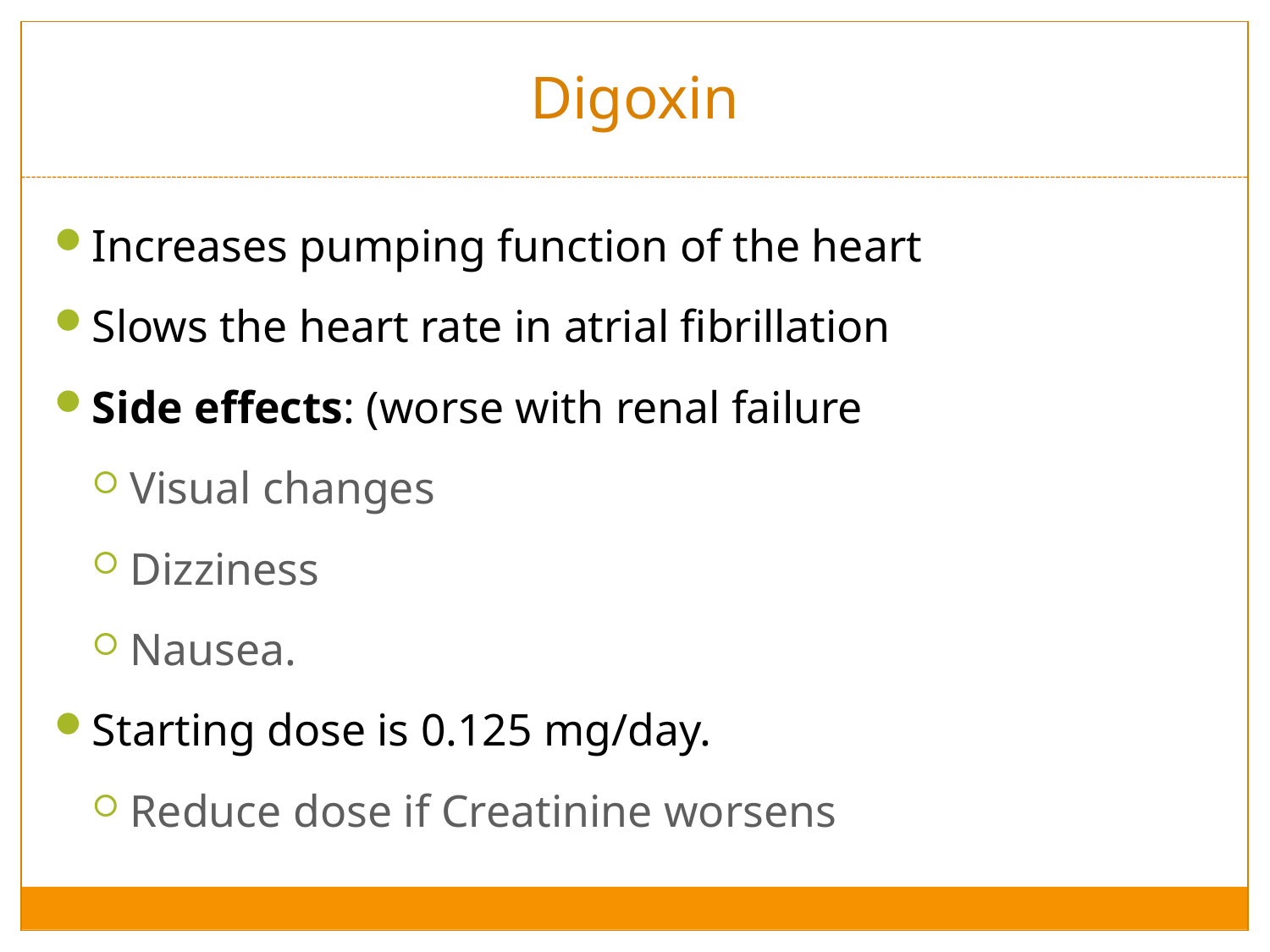

# Digoxin
Increases pumping function of the heart
Slows the heart rate in atrial fibrillation
Side effects: (worse with renal failure
Visual changes
Dizziness
Nausea.
Starting dose is 0.125 mg/day.
Reduce dose if Creatinine worsens

## Slide 64
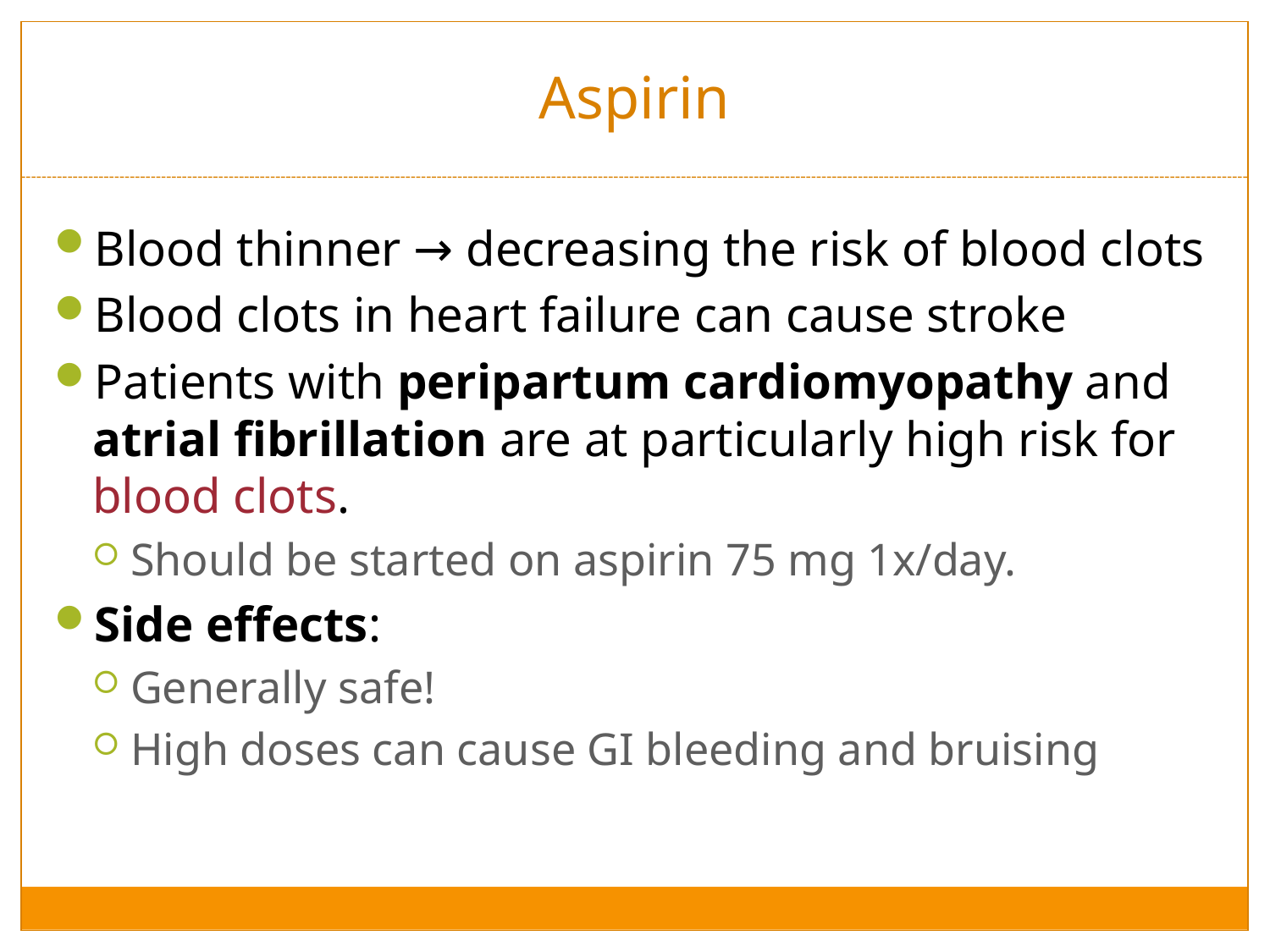

# Aspirin
Blood thinner → decreasing the risk of blood clots
Blood clots in heart failure can cause stroke
Patients with peripartum cardiomyopathy and atrial fibrillation are at particularly high risk for blood clots.
Should be started on aspirin 75 mg 1x/day.
Side effects:
Generally safe!
High doses can cause GI bleeding and bruising

## Slide 65
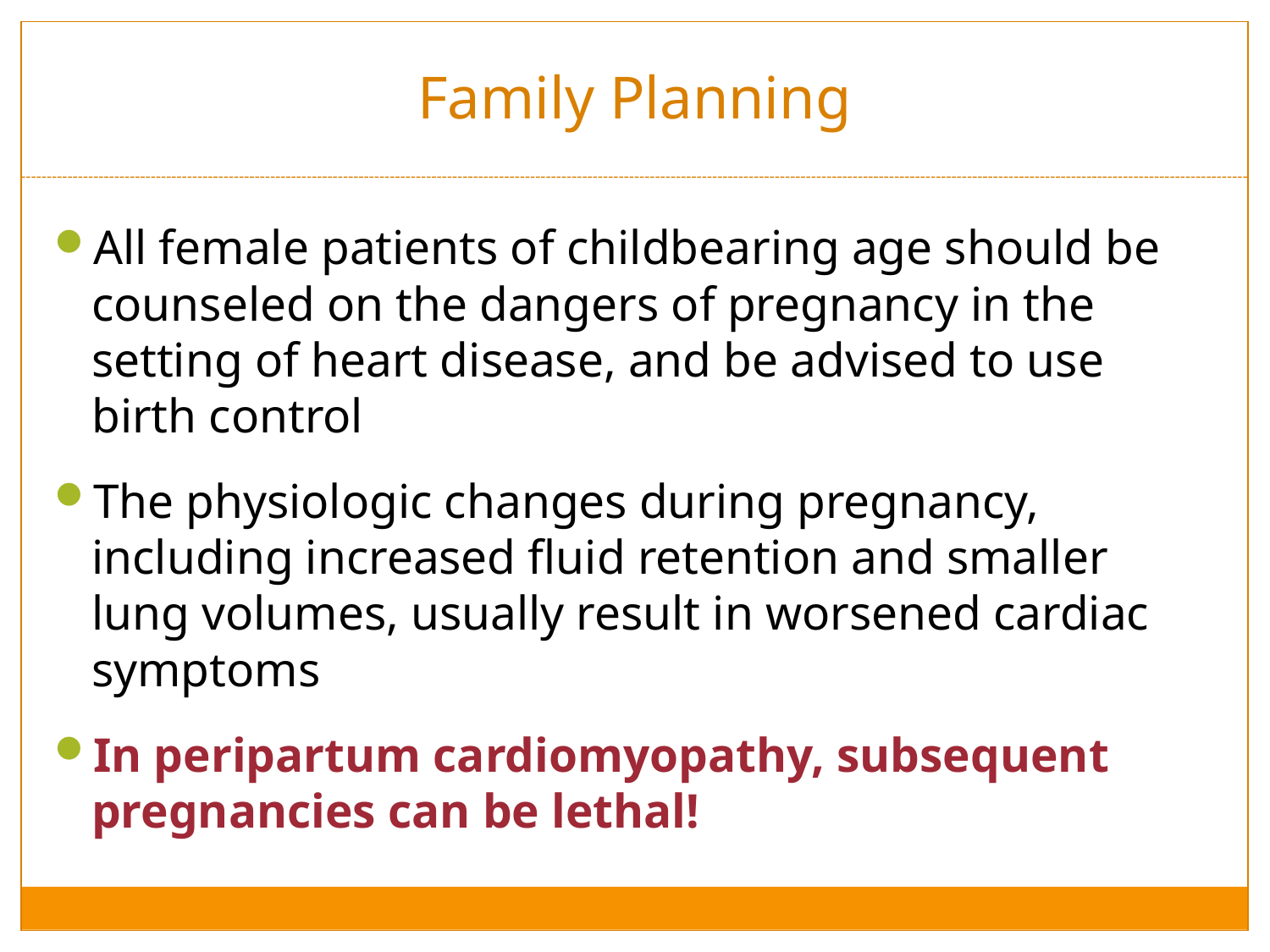

# Family Planning
All female patients of childbearing age should be counseled on the dangers of pregnancy in the setting of heart disease, and be advised to use birth control
The physiologic changes during pregnancy, including increased fluid retention and smaller lung volumes, usually result in worsened cardiac symptoms
In peripartum cardiomyopathy, subsequent pregnancies can be lethal!

## Slide 66
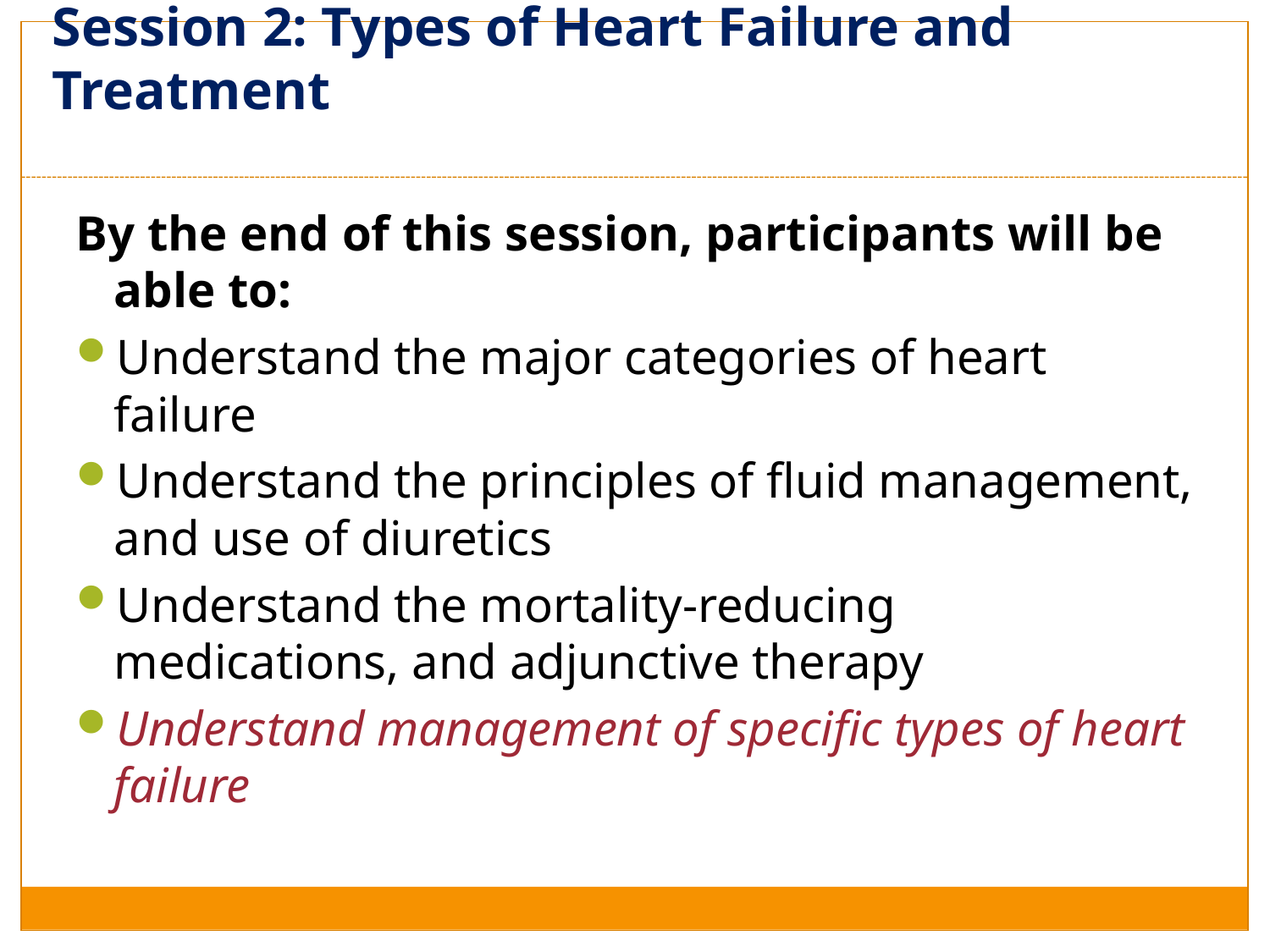

# Session 2: Types of Heart Failure and Treatment
By the end of this session, participants will be able to:
Understand the major categories of heart failure
Understand the principles of fluid management, and use of diuretics
Understand the mortality-reducing medications, and adjunctive therapy
Understand management of specific types of heart failure

## Slide 67
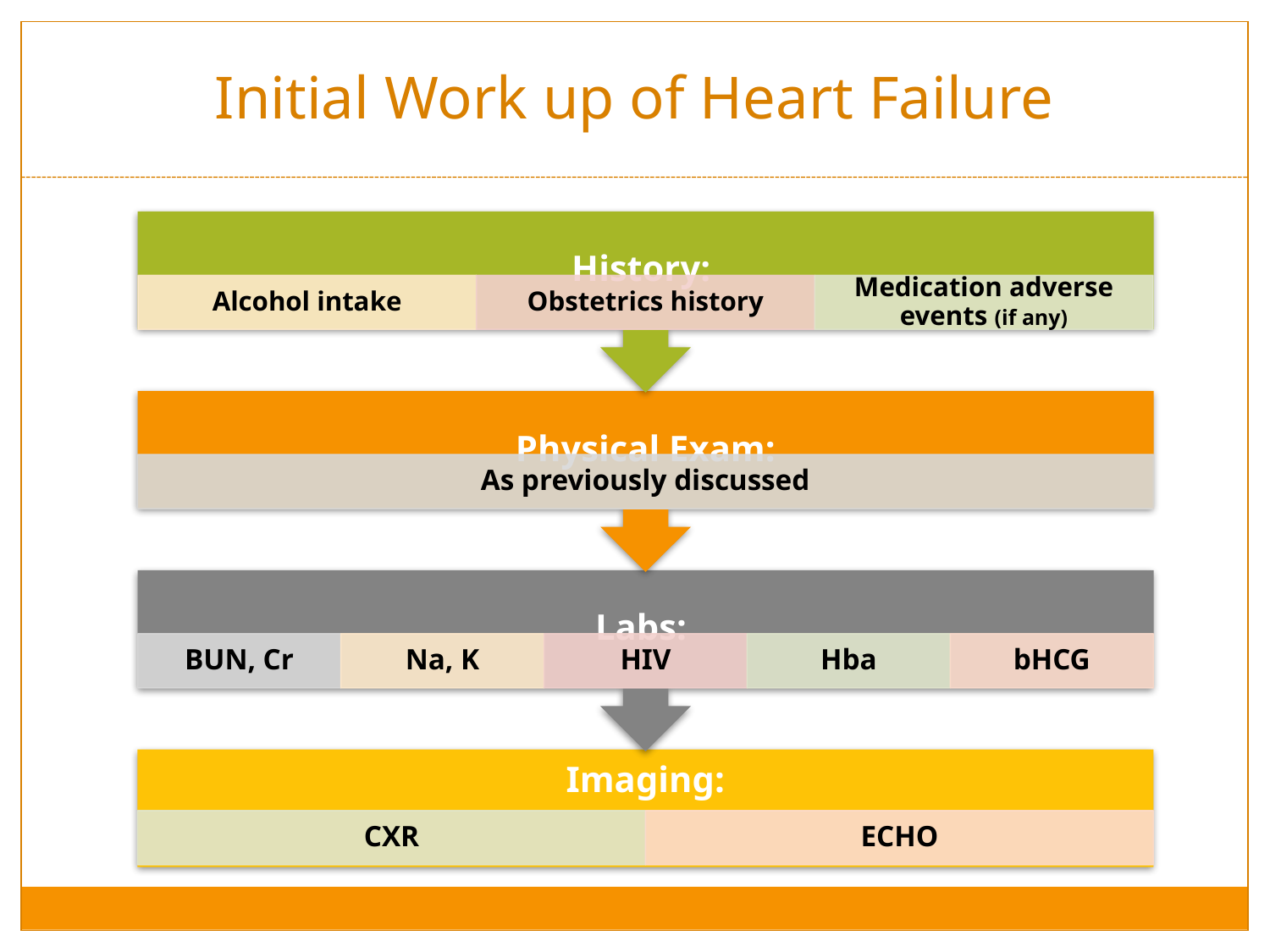

# Initial Work up of Heart Failure

## Slide 68
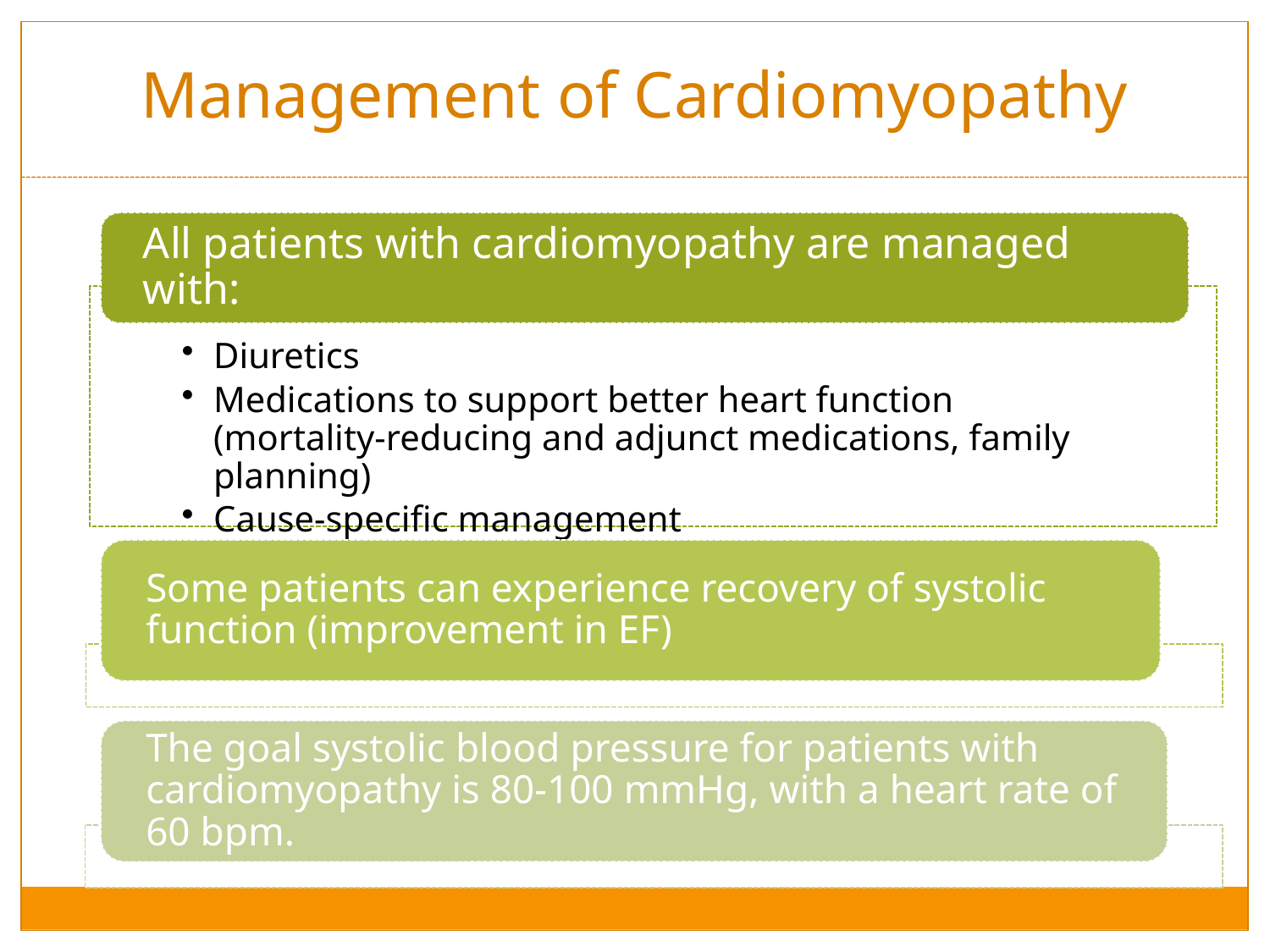

# Management of Cardiomyopathy

## Slide 69
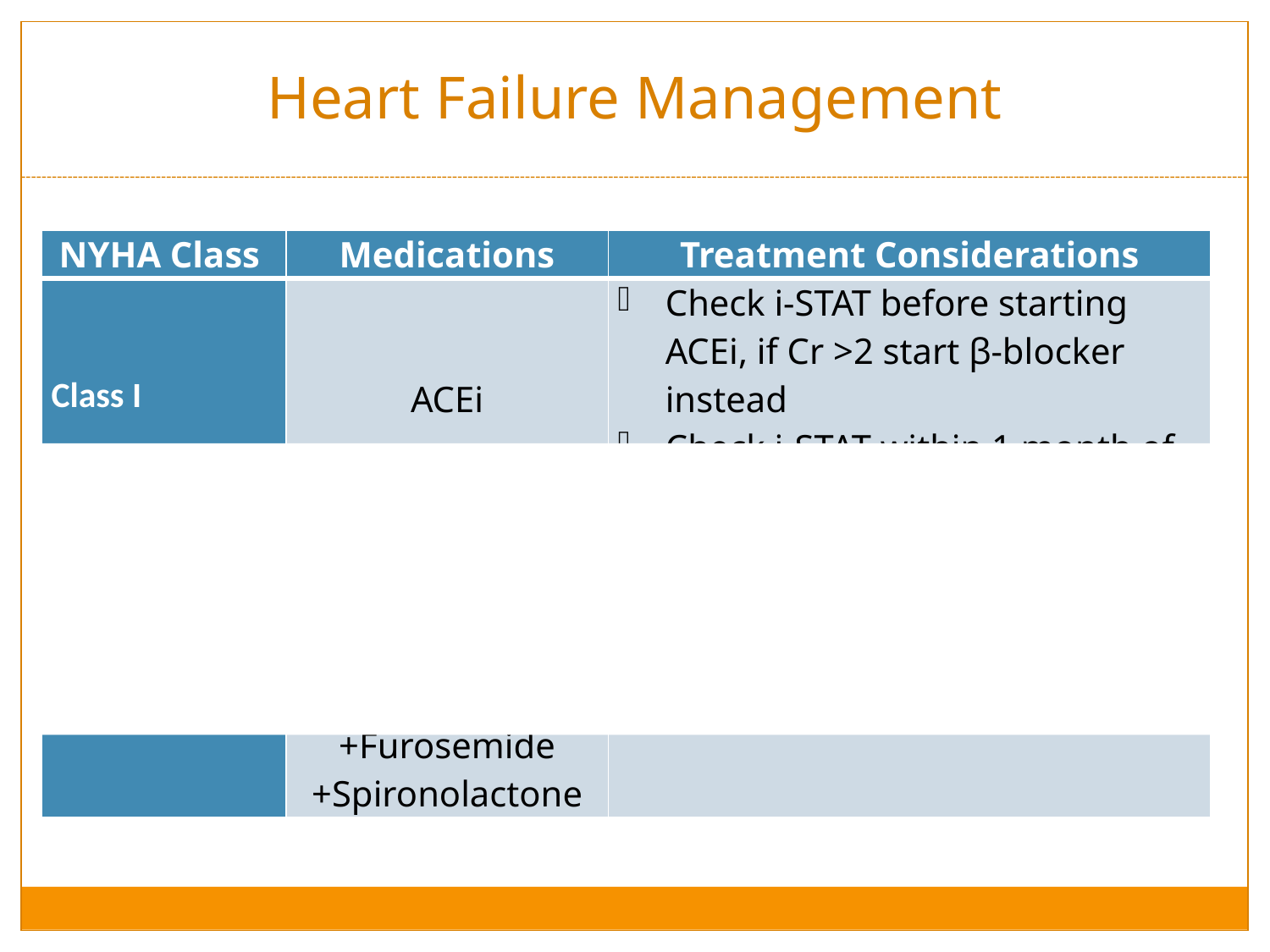

# Heart Failure Management
| NYHA Class | Medications | Treatment Considerations |
| --- | --- | --- |
| Class I | ACEi | Check i-STAT before starting ACEi, if Cr >2 start β-blocker instead Check i-STAT within 1 month of starting ACEi |
| Class II | ACEi +β-blocker | Do NOT start β-blocker in an acute heart failure exacerbation |
| Class III | ACEi +β-blocker +Furosemide +Spironolactone | Check i-STAT within 1 month of starting Spironolactone |

## Slide 70
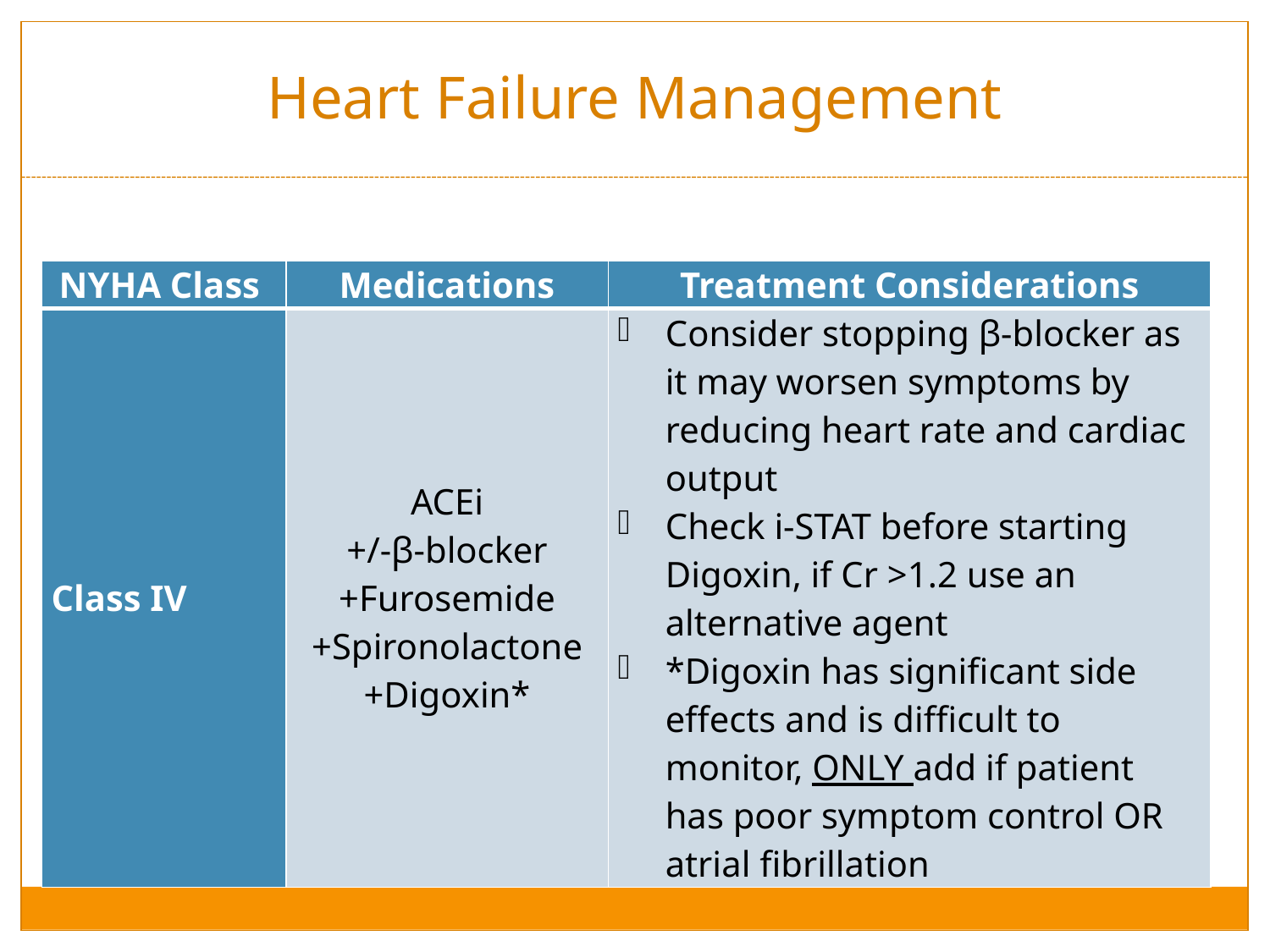

# Heart Failure Management
| NYHA Class | Medications | Treatment Considerations |
| --- | --- | --- |
| Class IV | ACEi +/-β-blocker +Furosemide +Spironolactone +Digoxin\* | Consider stopping β-blocker as it may worsen symptoms by reducing heart rate and cardiac output Check i-STAT before starting Digoxin, if Cr >1.2 use an alternative agent \*Digoxin has significant side effects and is difficult to monitor, ONLY add if patient has poor symptom control OR atrial fibrillation |

## Slide 71
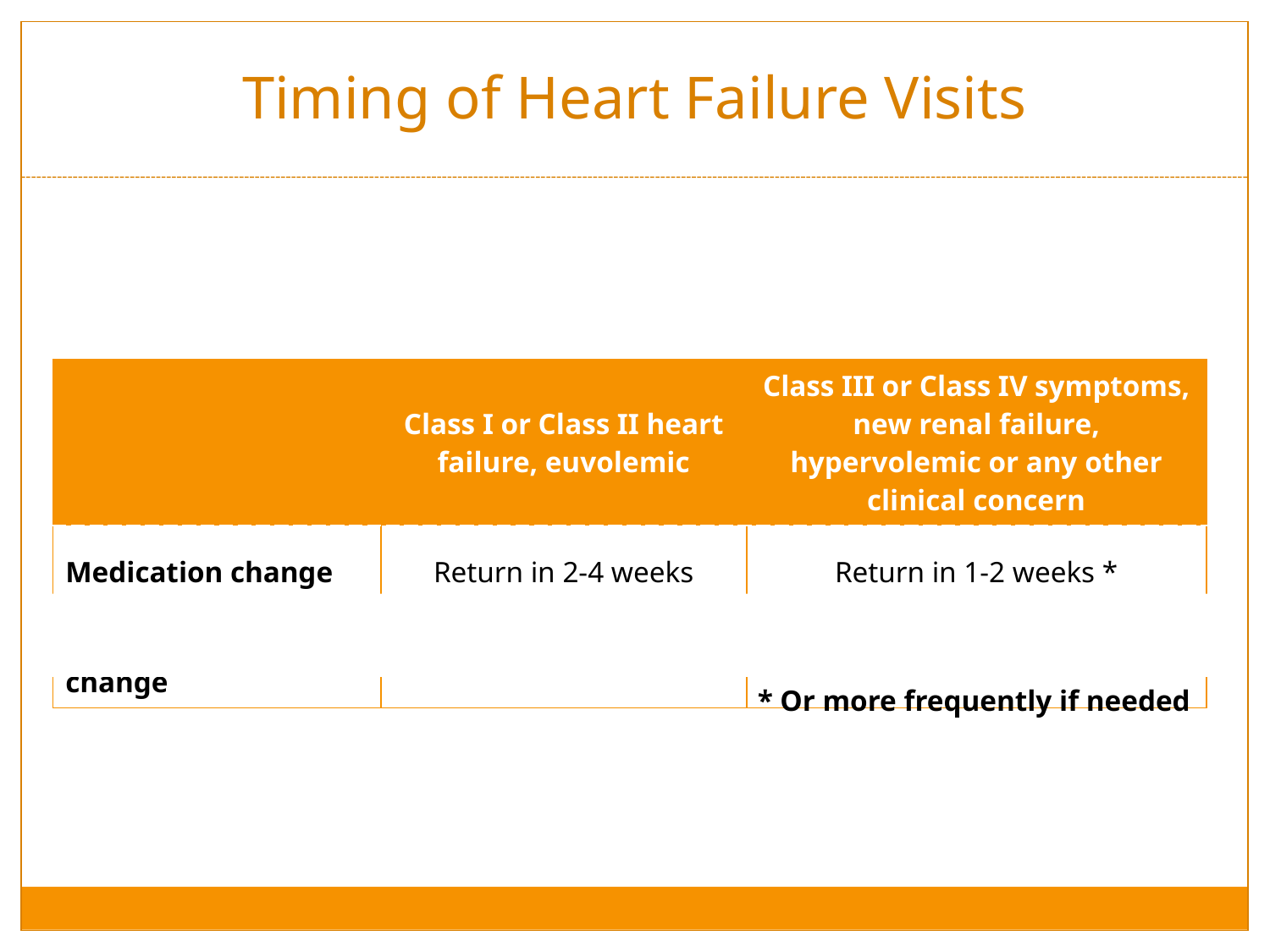

# Timing of Heart Failure Visits
| | Class I or Class II heart failure, euvolemic | Class III or Class IV symptoms, new renal failure, hypervolemic or any other clinical concern |
| --- | --- | --- |
| Medication change | Return in 2-4 weeks | Return in 1-2 weeks \* |
| No medication change | Return in 1-2 months | Return in 2-4 weeks \* |
* Or more frequently if needed

## Slide 72
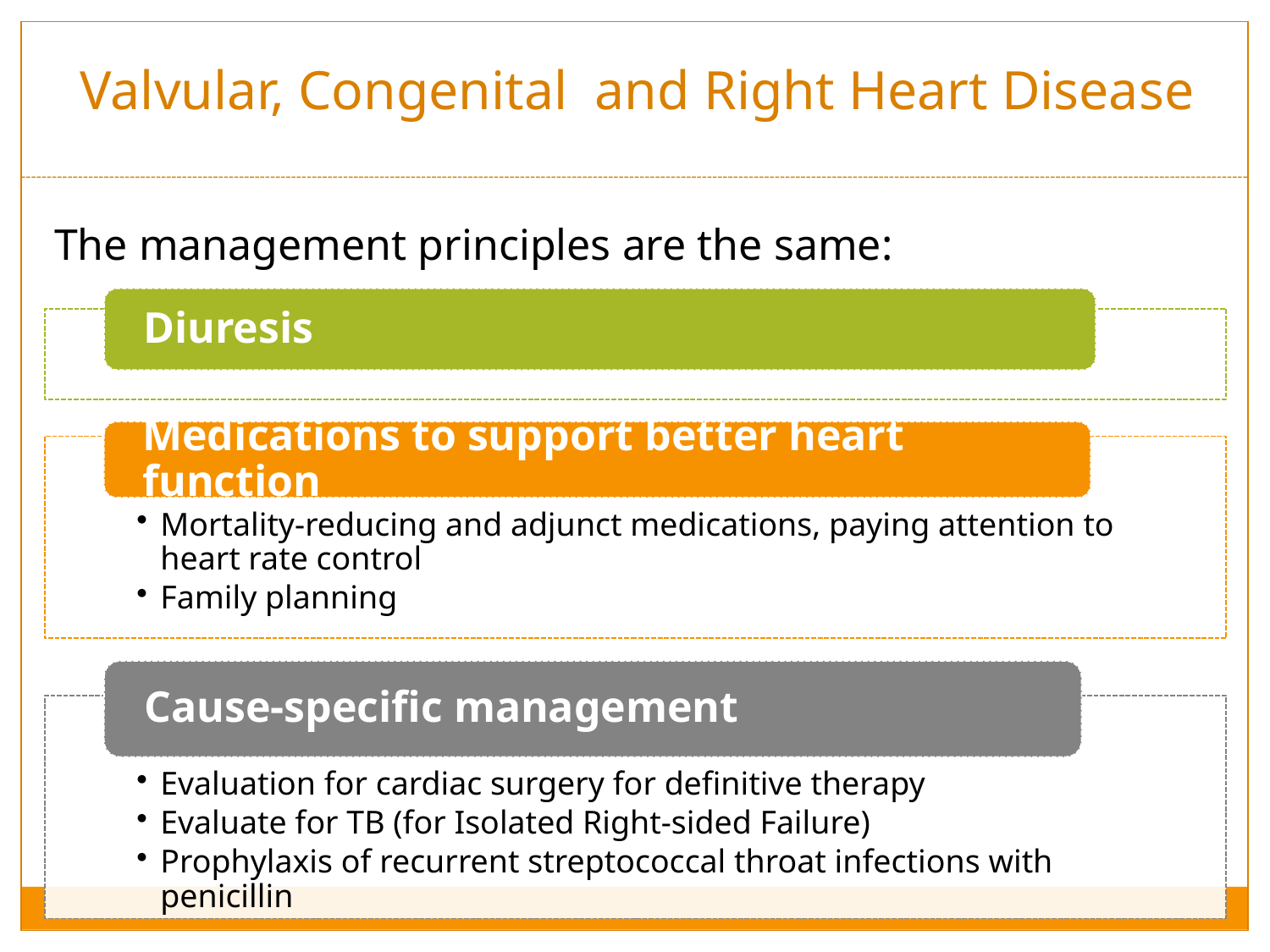

# Valvular, Congenital and Right Heart Disease
The management principles are the same:

## Slide 73
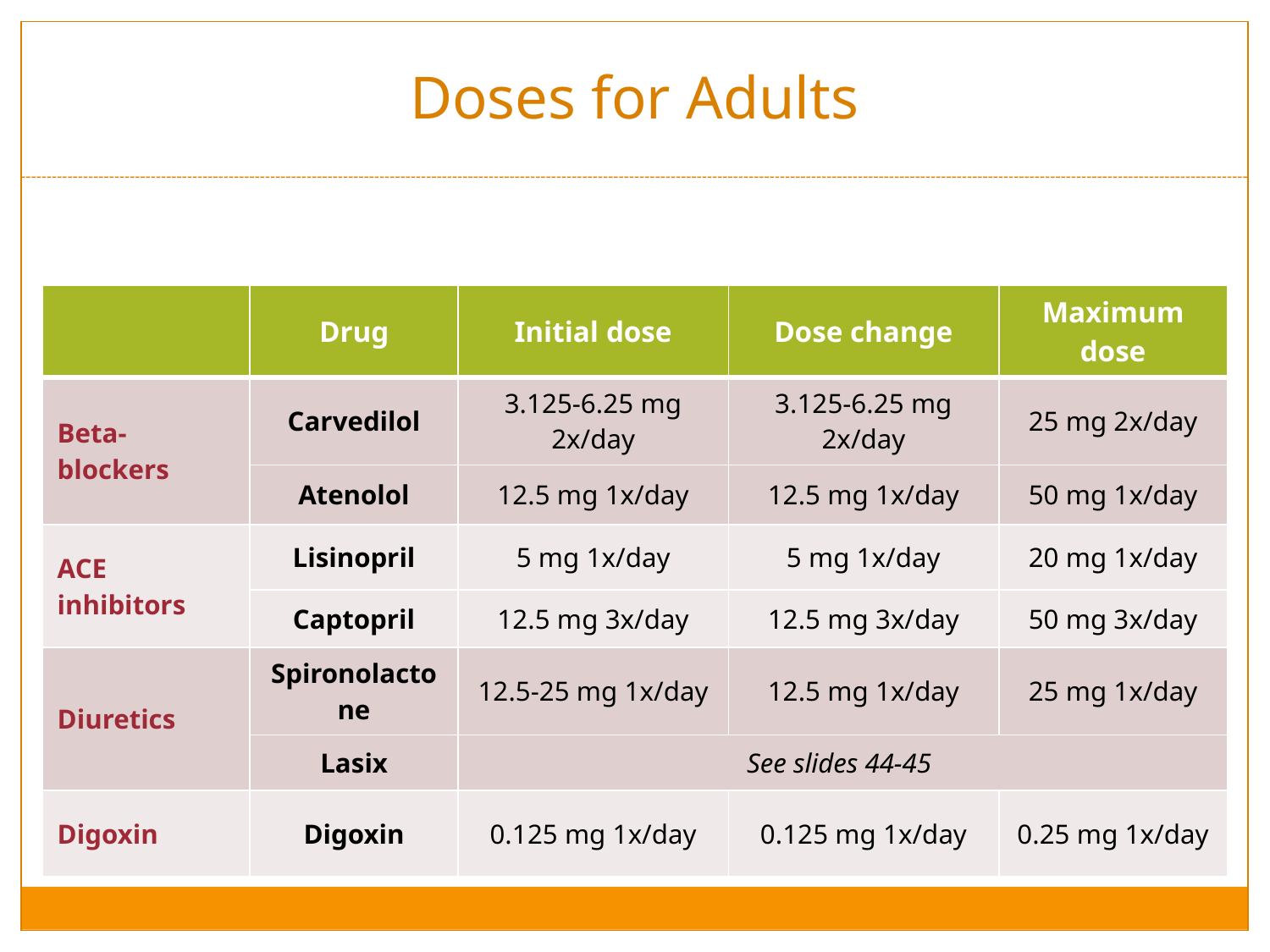

# Doses for Adults
| | Drug | Initial dose | Dose change | Maximum dose |
| --- | --- | --- | --- | --- |
| Beta-blockers | Carvedilol | 3.125-6.25 mg 2x/day | 3.125-6.25 mg 2x/day | 25 mg 2x/day |
| | Atenolol | 12.5 mg 1x/day | 12.5 mg 1x/day | 50 mg 1x/day |
| ACE inhibitors | Lisinopril | 5 mg 1x/day | 5 mg 1x/day | 20 mg 1x/day |
| | Captopril | 12.5 mg 3x/day | 12.5 mg 3x/day | 50 mg 3x/day |
| Diuretics | Spironolactone | 12.5-25 mg 1x/day | 12.5 mg 1x/day | 25 mg 1x/day |
| | Lasix | See slides 44-45 | | |
| Digoxin | Digoxin | 0.125 mg 1x/day | 0.125 mg 1x/day | 0.25 mg 1x/day |

## Slide 74
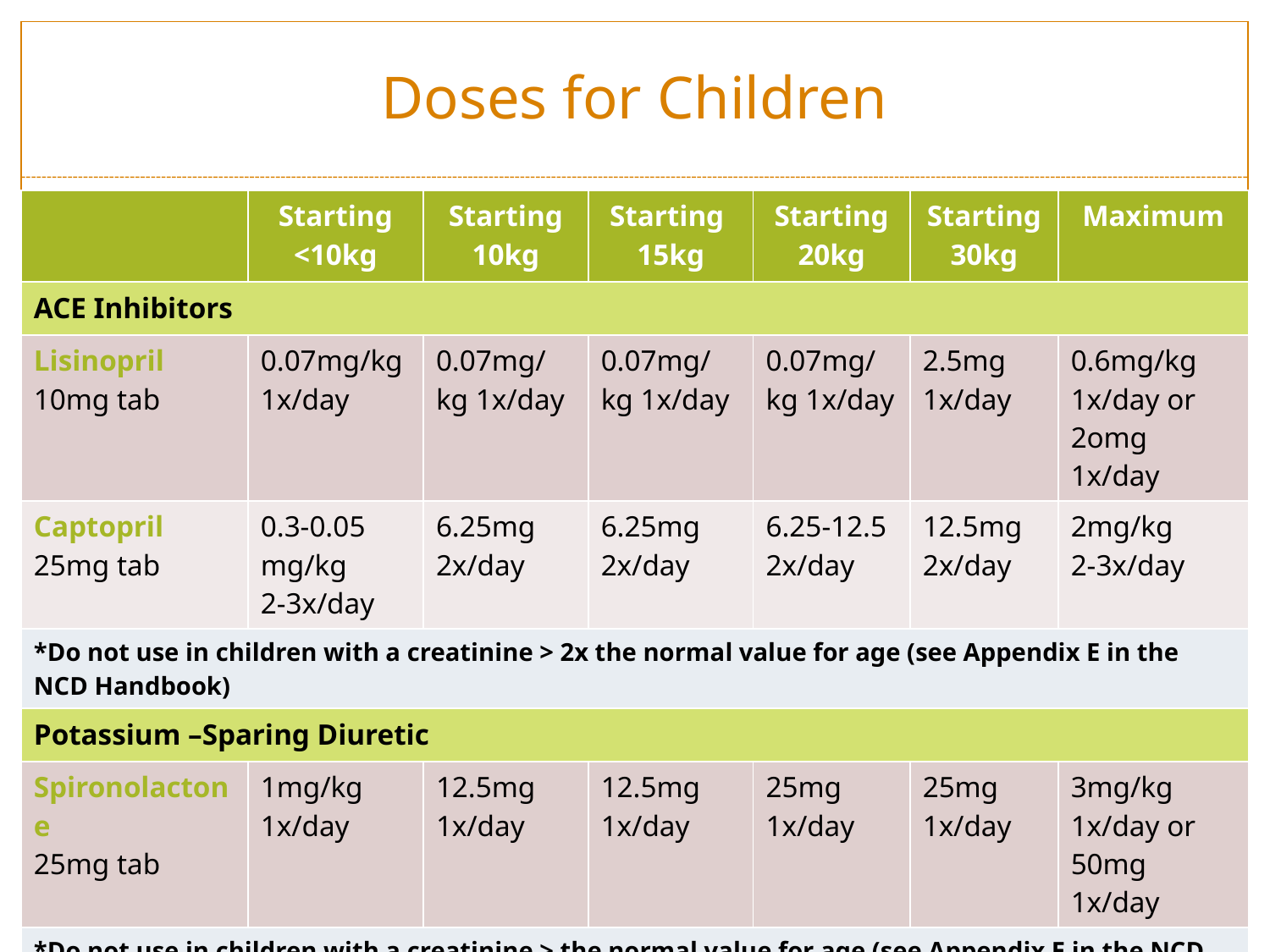

# Doses for Children
| | Starting <10kg | Starting 10kg | Starting 15kg | Starting 20kg | Starting 30kg | Maximum |
| --- | --- | --- | --- | --- | --- | --- |
| ACE Inhibitors | | | | | | |
| Lisinopril 10mg tab | 0.07mg/kg 1x/day | 0.07mg/kg 1x/day | 0.07mg/kg 1x/day | 0.07mg/kg 1x/day | 2.5mg 1x/day | 0.6mg/kg 1x/day or 2omg 1x/day |
| Captopril 25mg tab | 0.3-0.05 mg/kg 2-3x/day | 6.25mg 2x/day | 6.25mg 2x/day | 6.25-12.5 2x/day | 12.5mg 2x/day | 2mg/kg 2-3x/day |
| \*Do not use in children with a creatinine > 2x the normal value for age (see Appendix E in the NCD Handbook) | | | | | | |
| Potassium –Sparing Diuretic | | | | | | |
| Spironolactone 25mg tab | 1mg/kg 1x/day | 12.5mg 1x/day | 12.5mg 1x/day | 25mg 1x/day | 25mg 1x/day | 3mg/kg 1x/day or 50mg 1x/day |
| \*Do not use in children with a creatinine > the normal value for age (see Appendix E in the NCD Handbook) | | | | | | |

## Slide 75
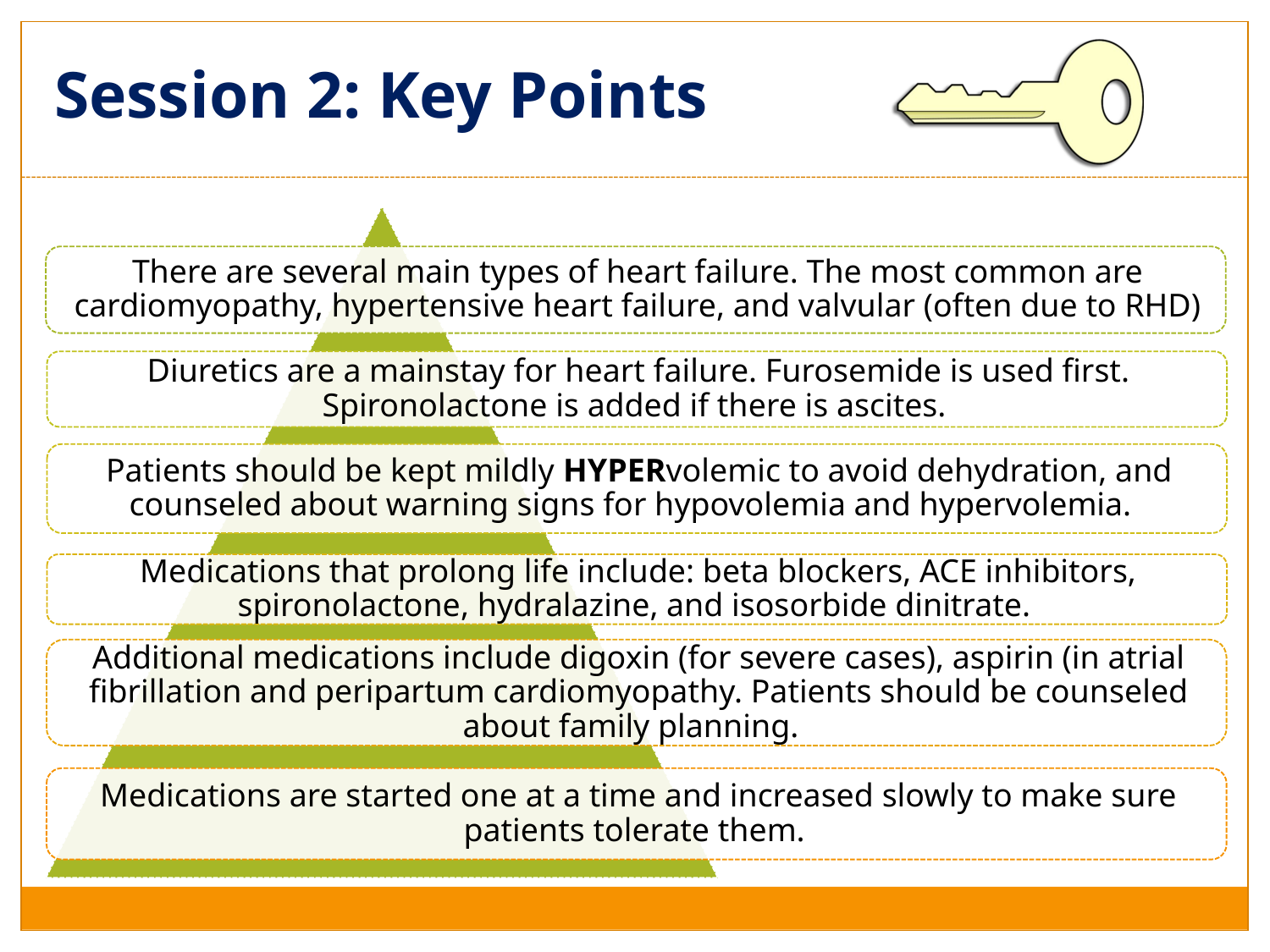

# Session 2: Key Points

## Slide 76
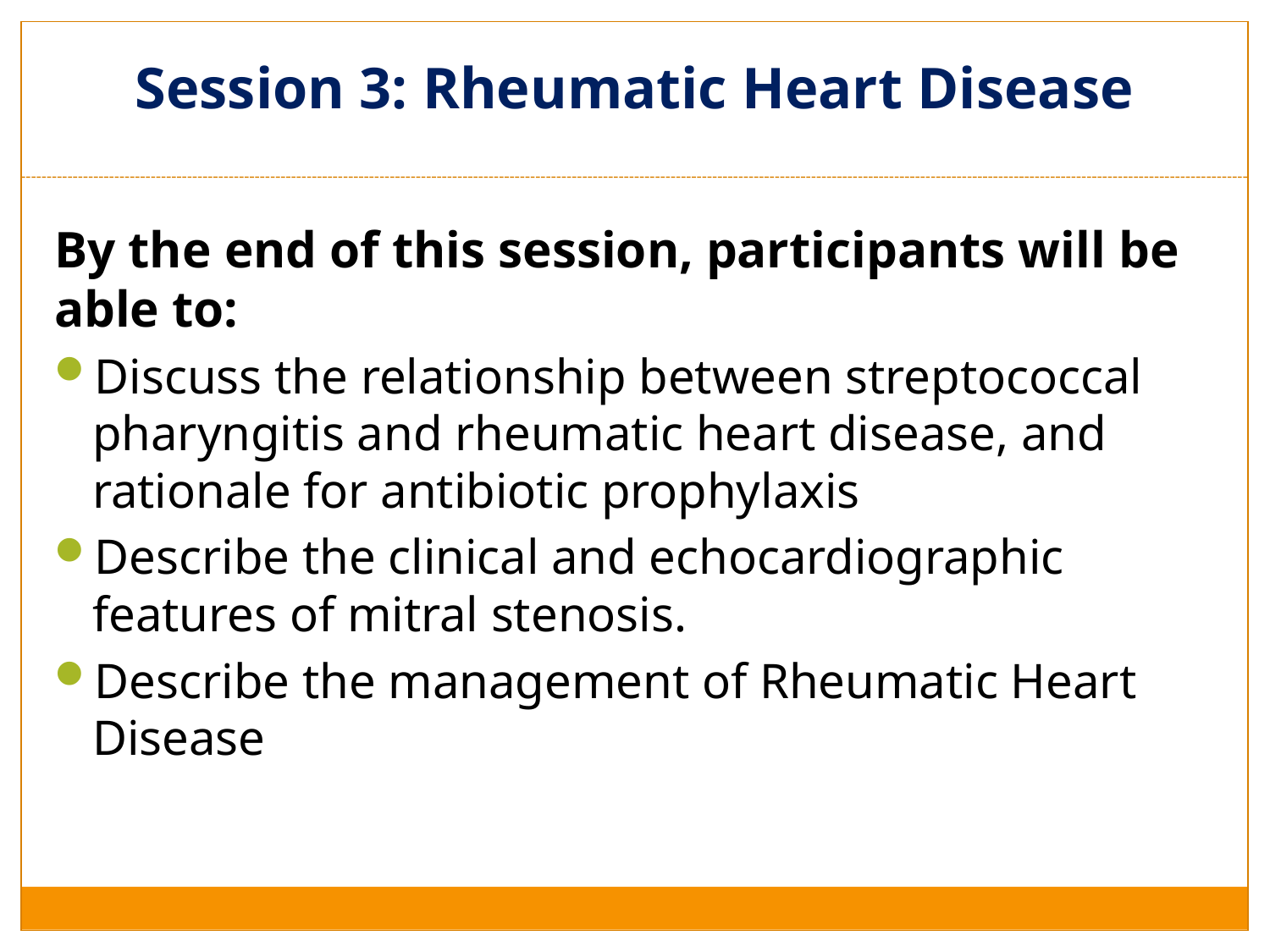

# Session 3: Rheumatic Heart Disease
By the end of this session, participants will be able to:
Discuss the relationship between streptococcal pharyngitis and rheumatic heart disease, and rationale for antibiotic prophylaxis
Describe the clinical and echocardiographic features of mitral stenosis.
Describe the management of Rheumatic Heart Disease

## Slide 77
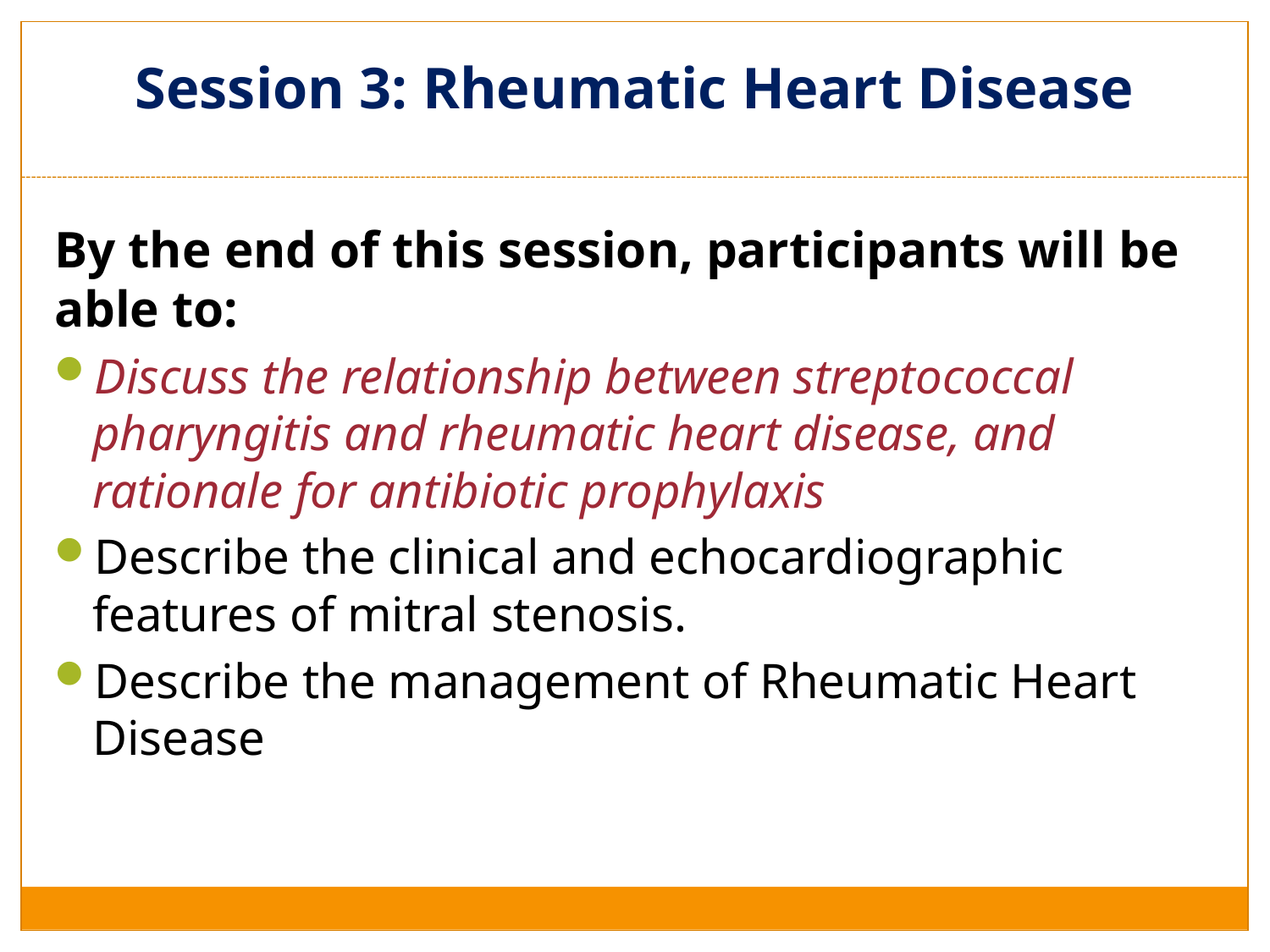

# Session 3: Rheumatic Heart Disease
By the end of this session, participants will be able to:
Discuss the relationship between streptococcal pharyngitis and rheumatic heart disease, and rationale for antibiotic prophylaxis
Describe the clinical and echocardiographic features of mitral stenosis.
Describe the management of Rheumatic Heart Disease

## Slide 78
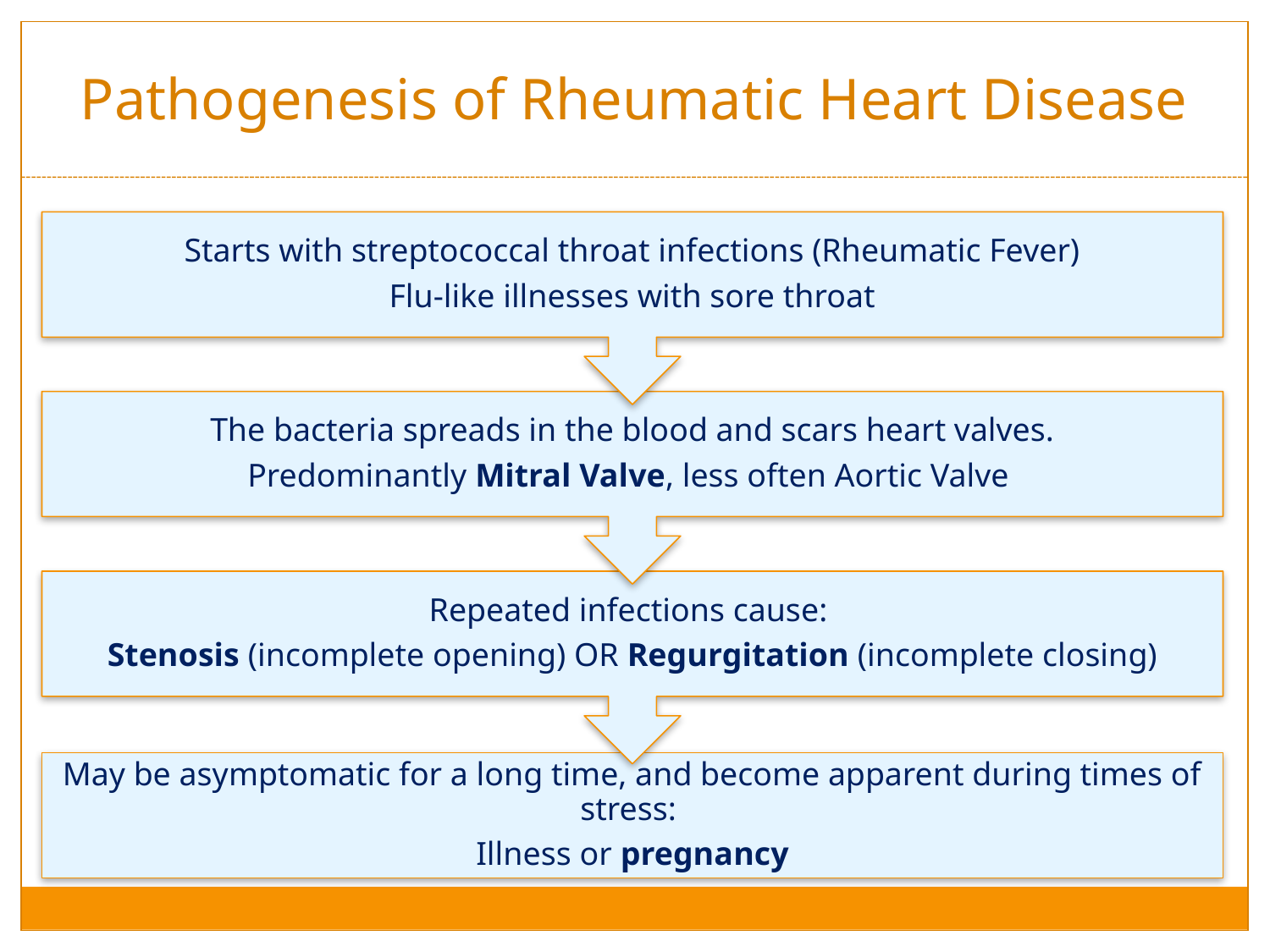

# Pathogenesis of Rheumatic Heart Disease

## Slide 79
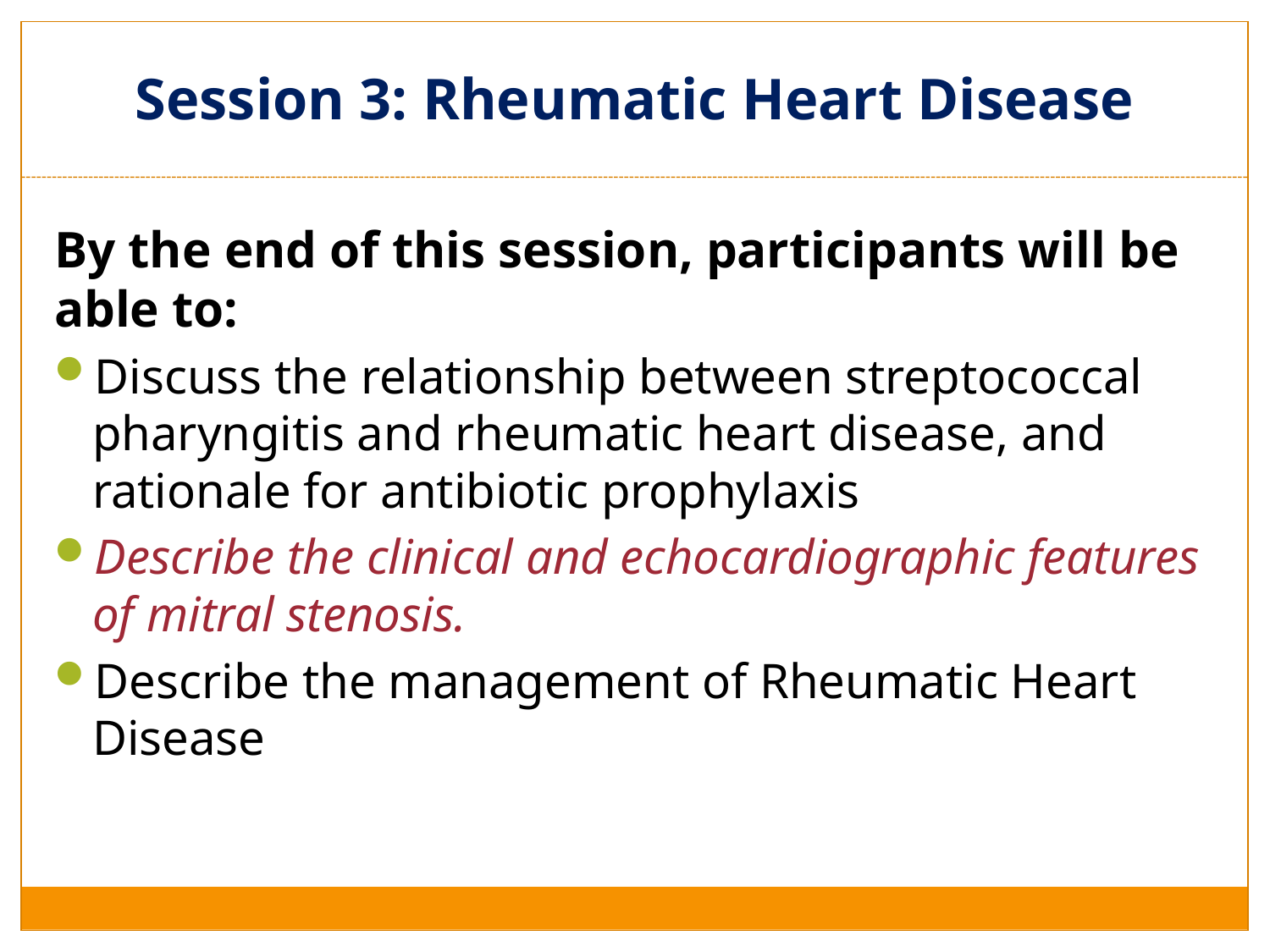

# Session 3: Rheumatic Heart Disease
By the end of this session, participants will be able to:
Discuss the relationship between streptococcal pharyngitis and rheumatic heart disease, and rationale for antibiotic prophylaxis
Describe the clinical and echocardiographic features of mitral stenosis.
Describe the management of Rheumatic Heart Disease

## Slide 80
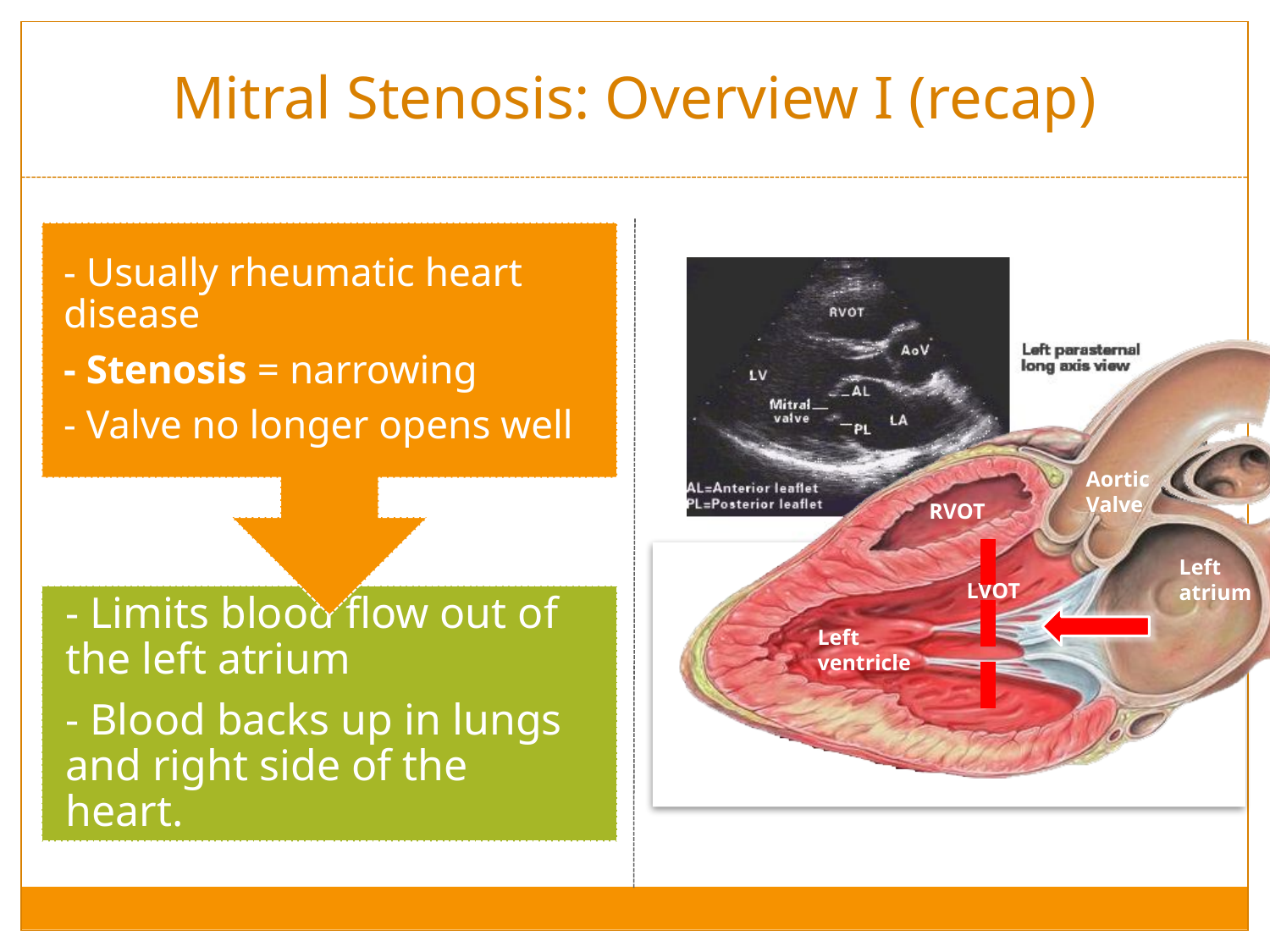

# Mitral Stenosis: Overview I (recap)
Aortic Valve
Aortic Valve
RVOT
RVOT
Left atrium
Left atrium
LVOT
LVOT
Left ventricle
Left ventricle

## Slide 81
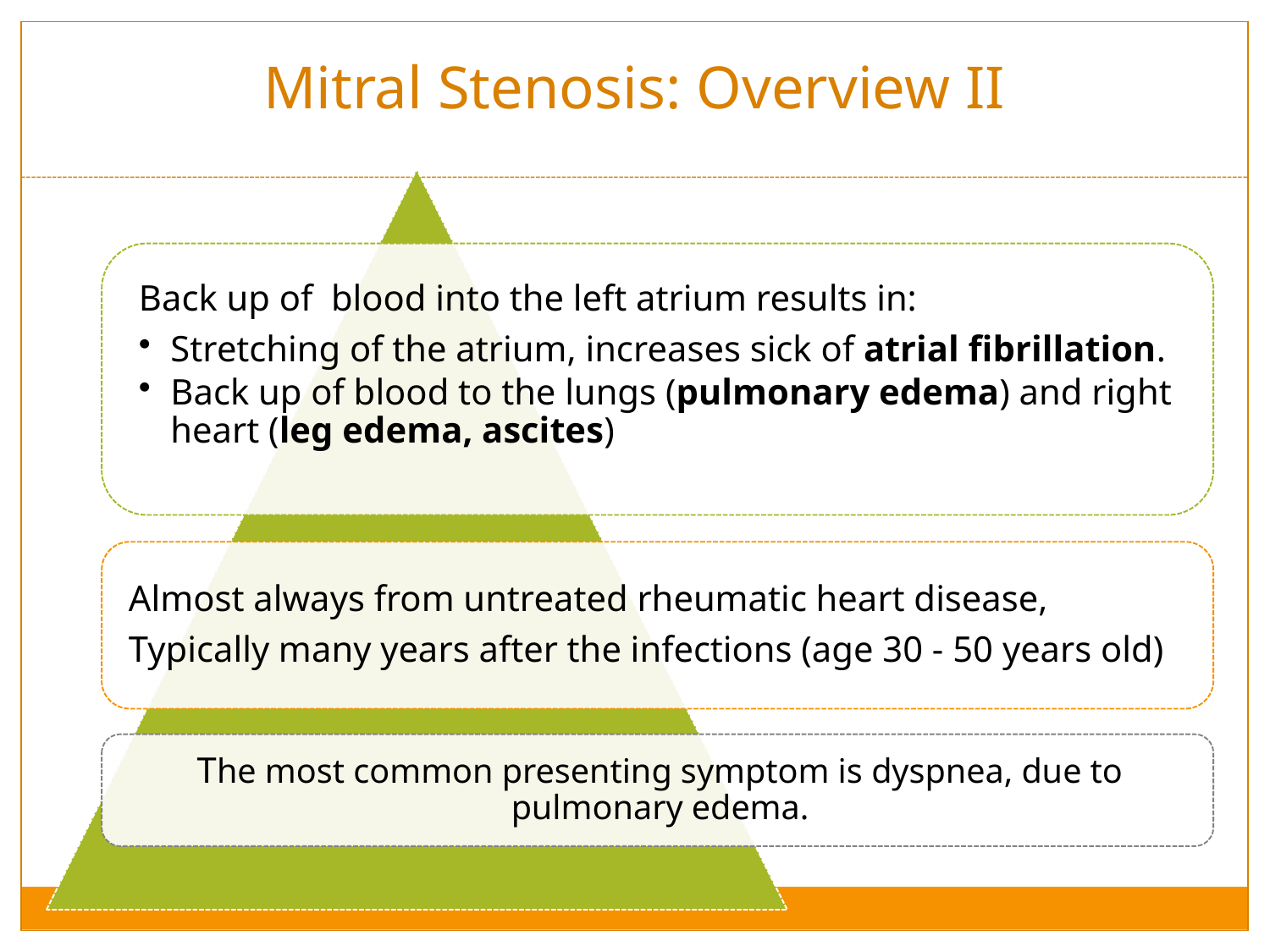

# Mitral Stenosis: Overview II

## Slide 82
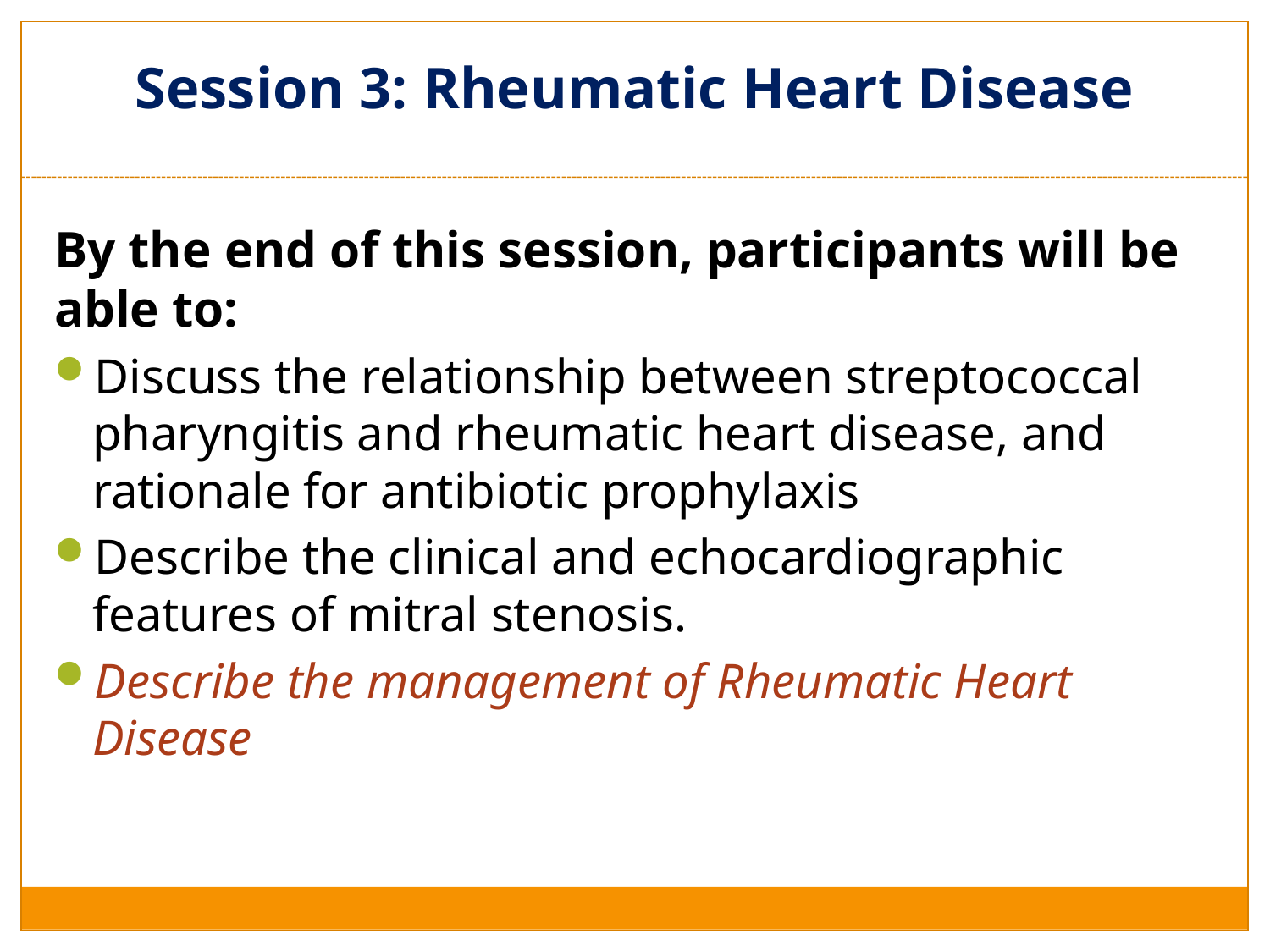

# Session 3: Rheumatic Heart Disease
By the end of this session, participants will be able to:
Discuss the relationship between streptococcal pharyngitis and rheumatic heart disease, and rationale for antibiotic prophylaxis
Describe the clinical and echocardiographic features of mitral stenosis.
Describe the management of Rheumatic Heart Disease

## Slide 83
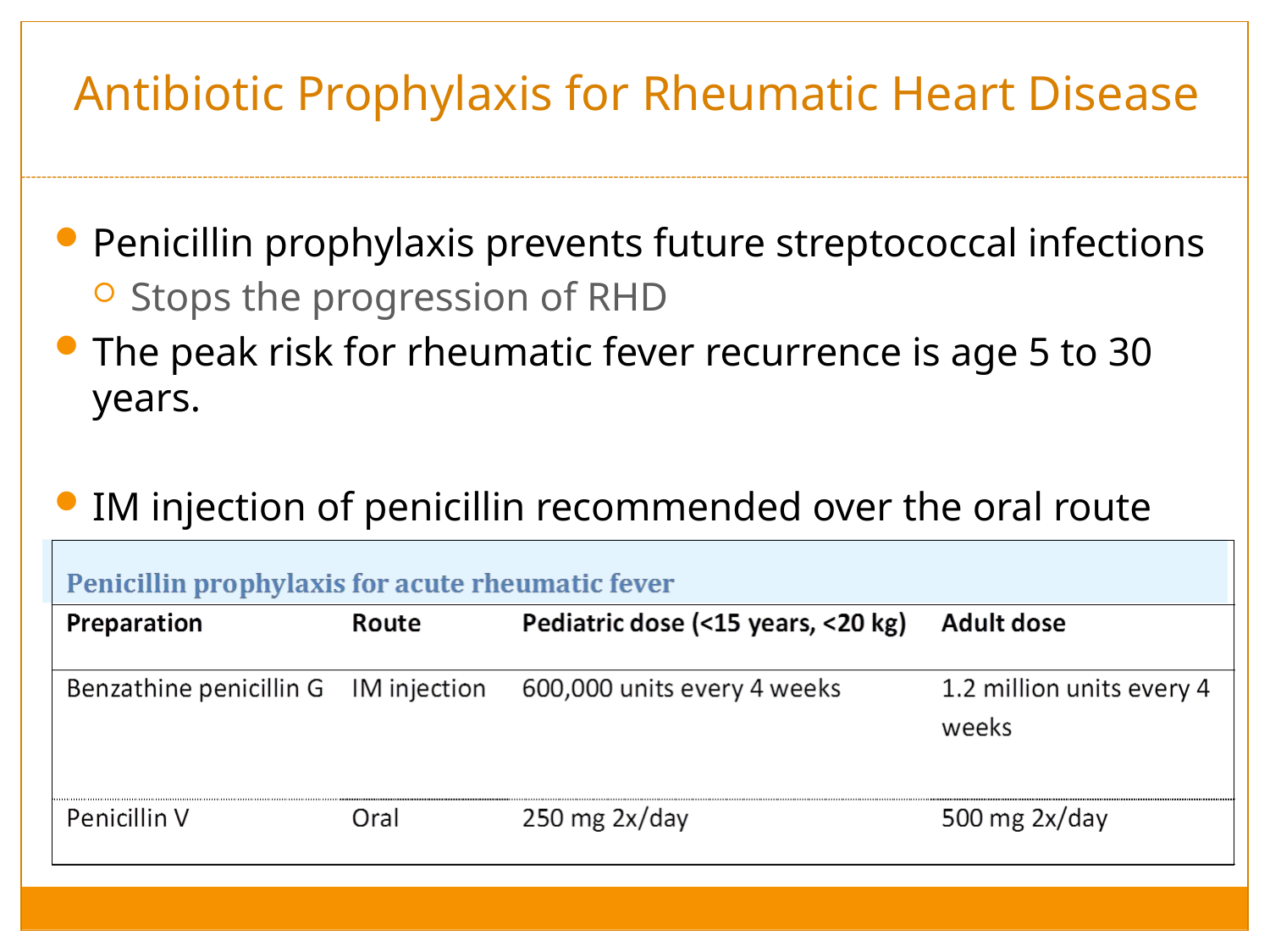

# Antibiotic Prophylaxis for Rheumatic Heart Disease
Penicillin prophylaxis prevents future streptococcal infections
Stops the progression of RHD
The peak risk for rheumatic fever recurrence is age 5 to 30 years.
IM injection of penicillin recommended over the oral route

## Slide 84
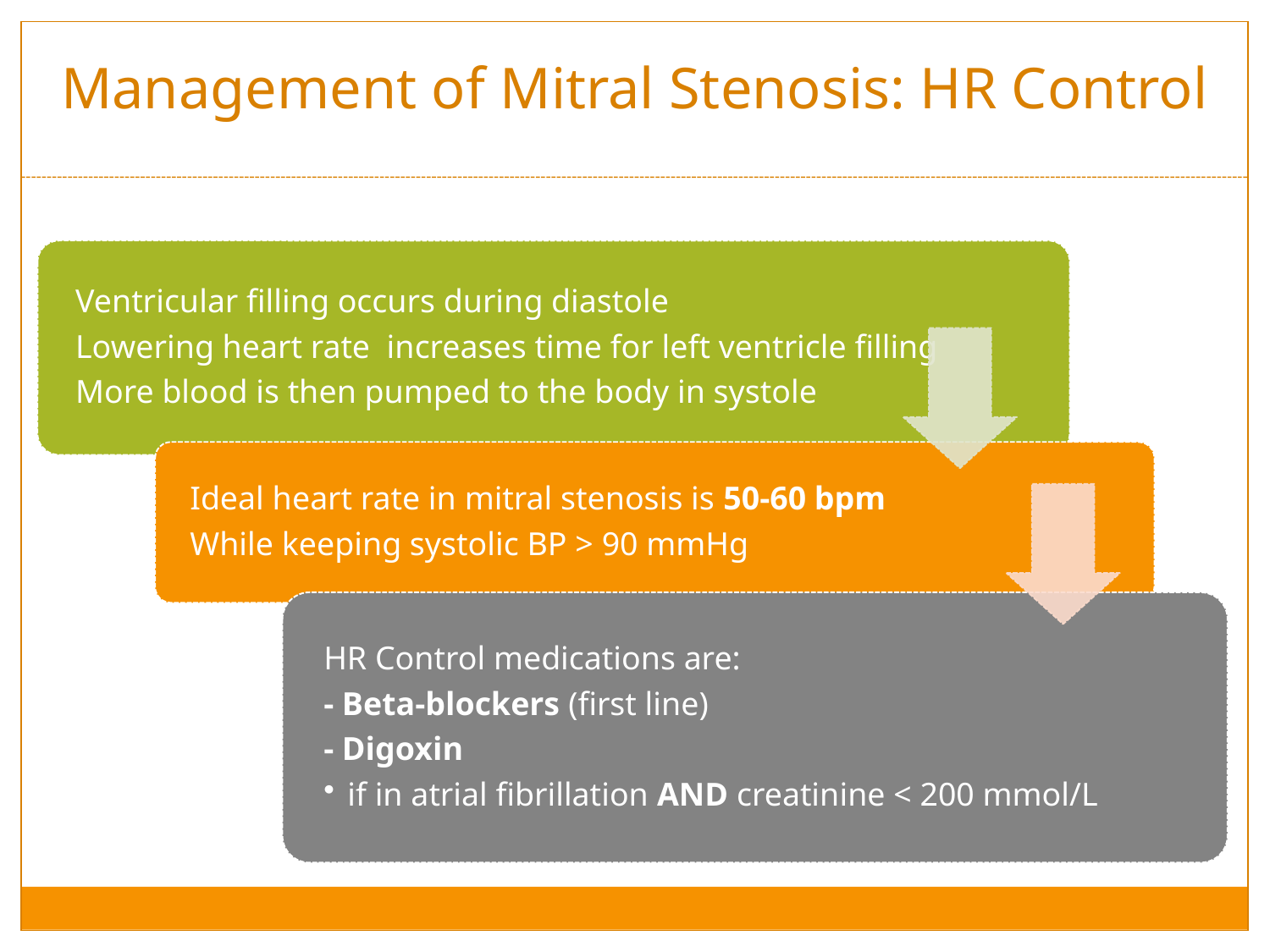

# Management of Mitral Stenosis: HR Control

## Slide 85
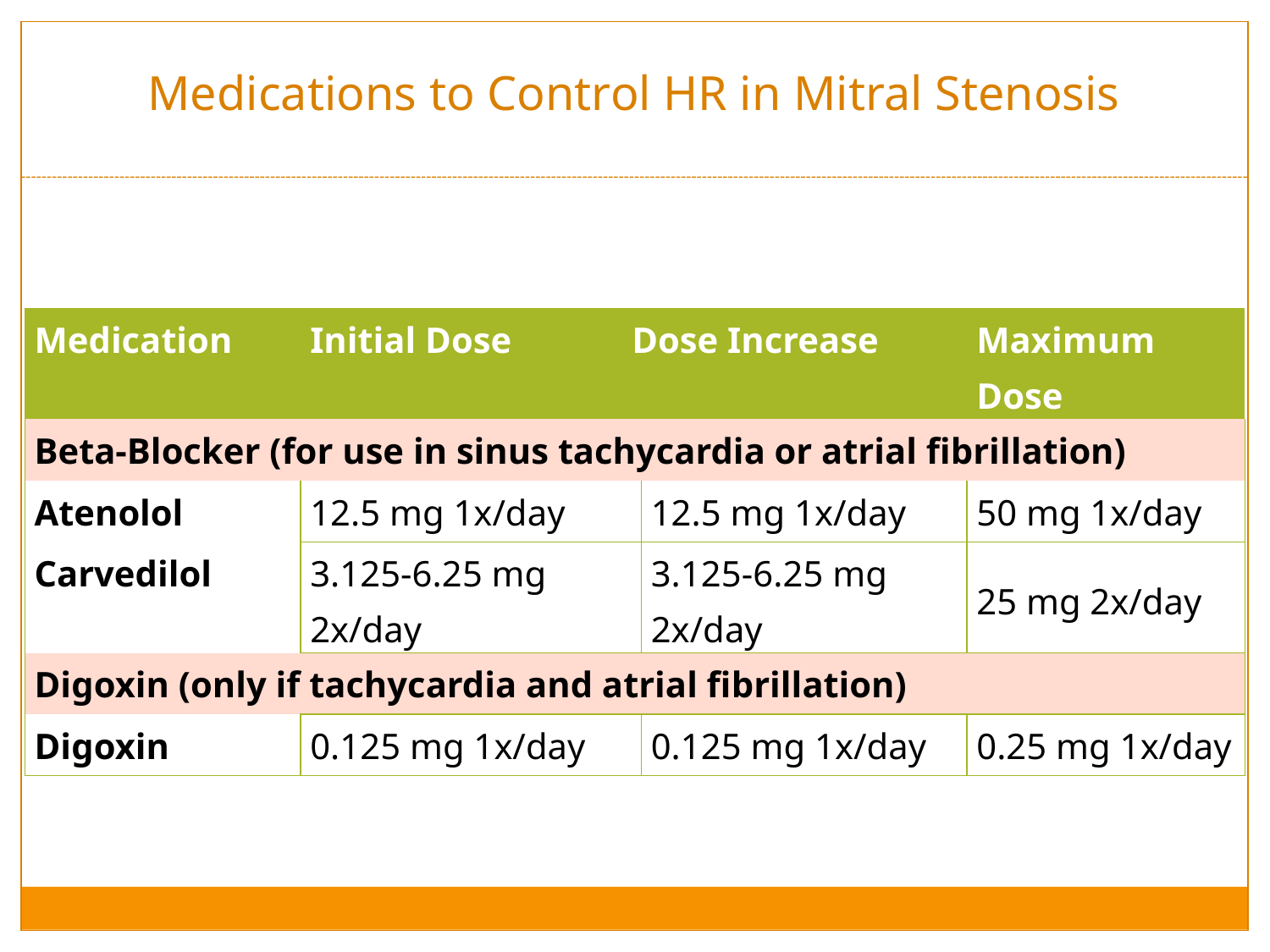

# Medications to Control HR in Mitral Stenosis
| Medication | Initial Dose | Dose Increase | | Maximum Dose |
| --- | --- | --- | --- | --- |
| Beta-Blocker (for use in sinus tachycardia or atrial fibrillation) | | | | |
| Atenolol | 12.5 mg 1x/day | | 12.5 mg 1x/day | 50 mg 1x/day |
| Carvedilol | 3.125-6.25 mg 2x/day | | 3.125-6.25 mg 2x/day | 25 mg 2x/day |
| Digoxin (only if tachycardia and atrial fibrillation) | | | | |
| Digoxin | 0.125 mg 1x/day | | 0.125 mg 1x/day | 0.25 mg 1x/day |

## Slide 86
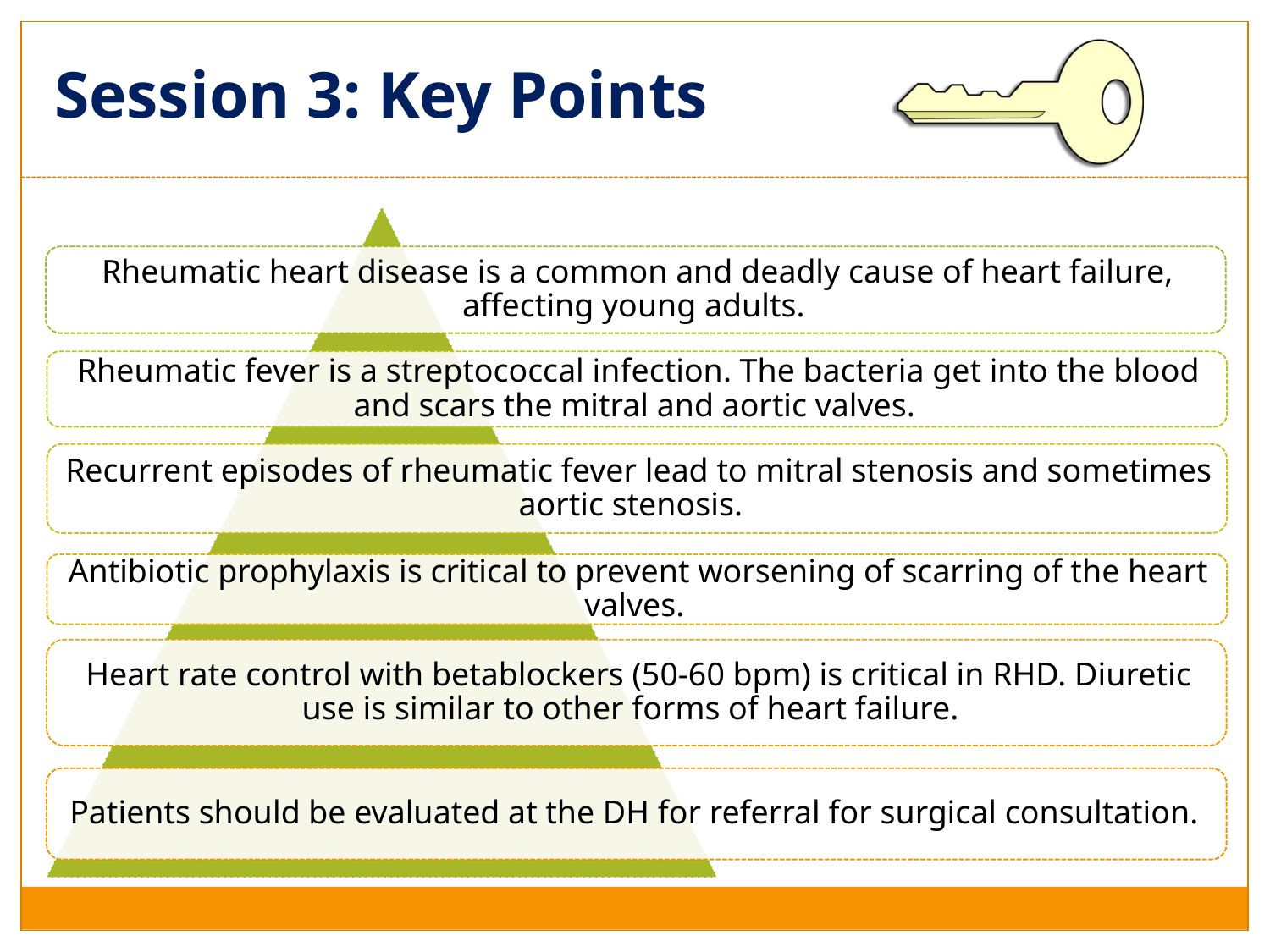

# Session 3: Key Points
