# Supplementary material for: Training Mid-Level Providers to Treat Severe Non-Communicable Diseases in Neno, Malawi through PEN-Plus Strategies
Source: Ann Glob Health. 2022 Aug 11;88(1):69. doi: 10.5334/aogh.3750 (PMC9389951; doi:10.5334/aogh.3750)
Supplement: Didactic Materials. — The supplementary materials contain a suggested didactic training schedule and the PowerPoint presentations used for PEN-Plus training in Neno, Malawi. These materials have been reviewed and accepted by the Malawi Ministry of Health for future PEN-Plus trainings in Malawi. [file agh-88-1-3750-s2.zip › Didactic_Materials/CV_Counseling.pptx]

## Slide 1
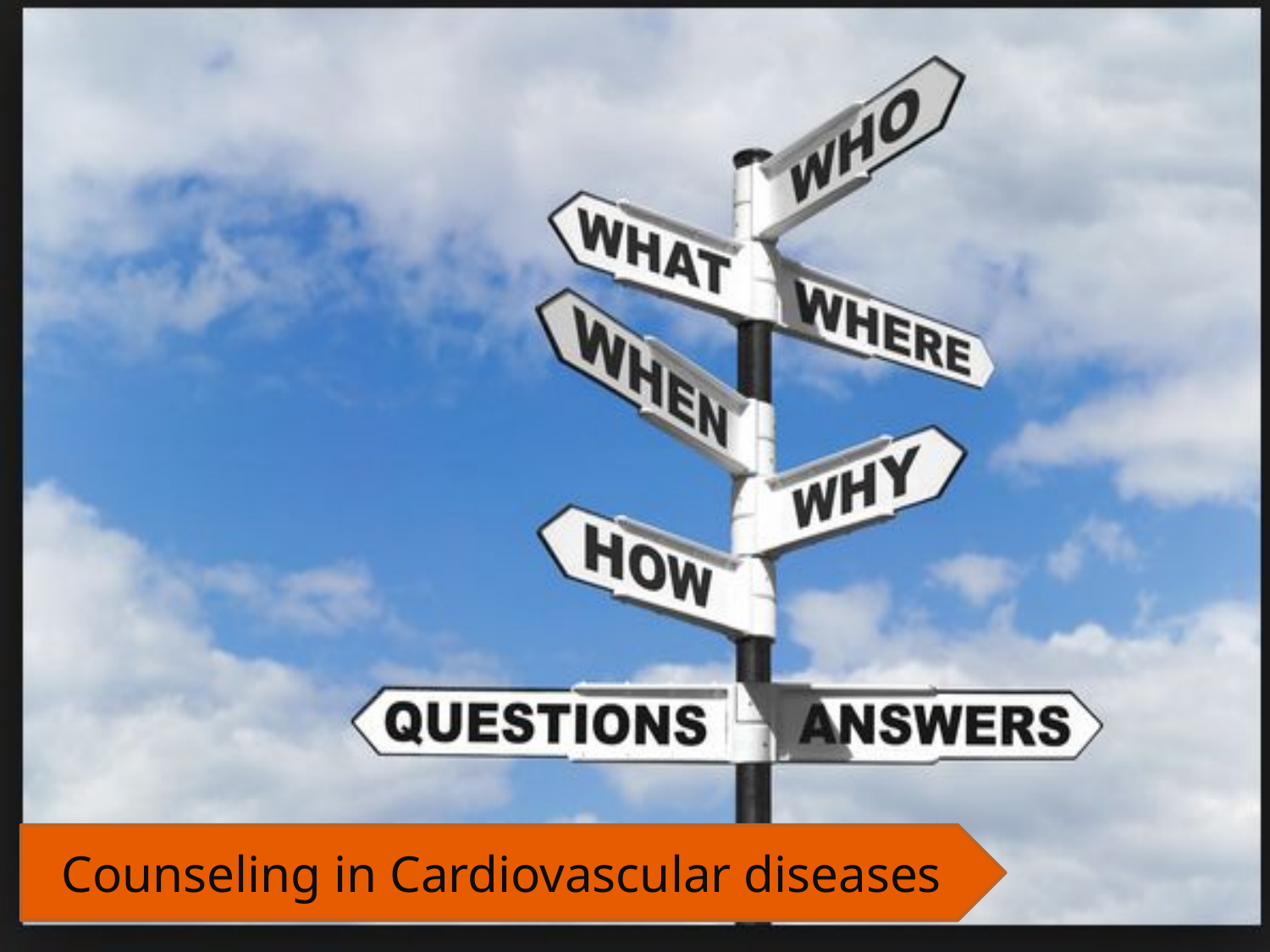

Counseling in Cardiovascular diseases

## Slide 2
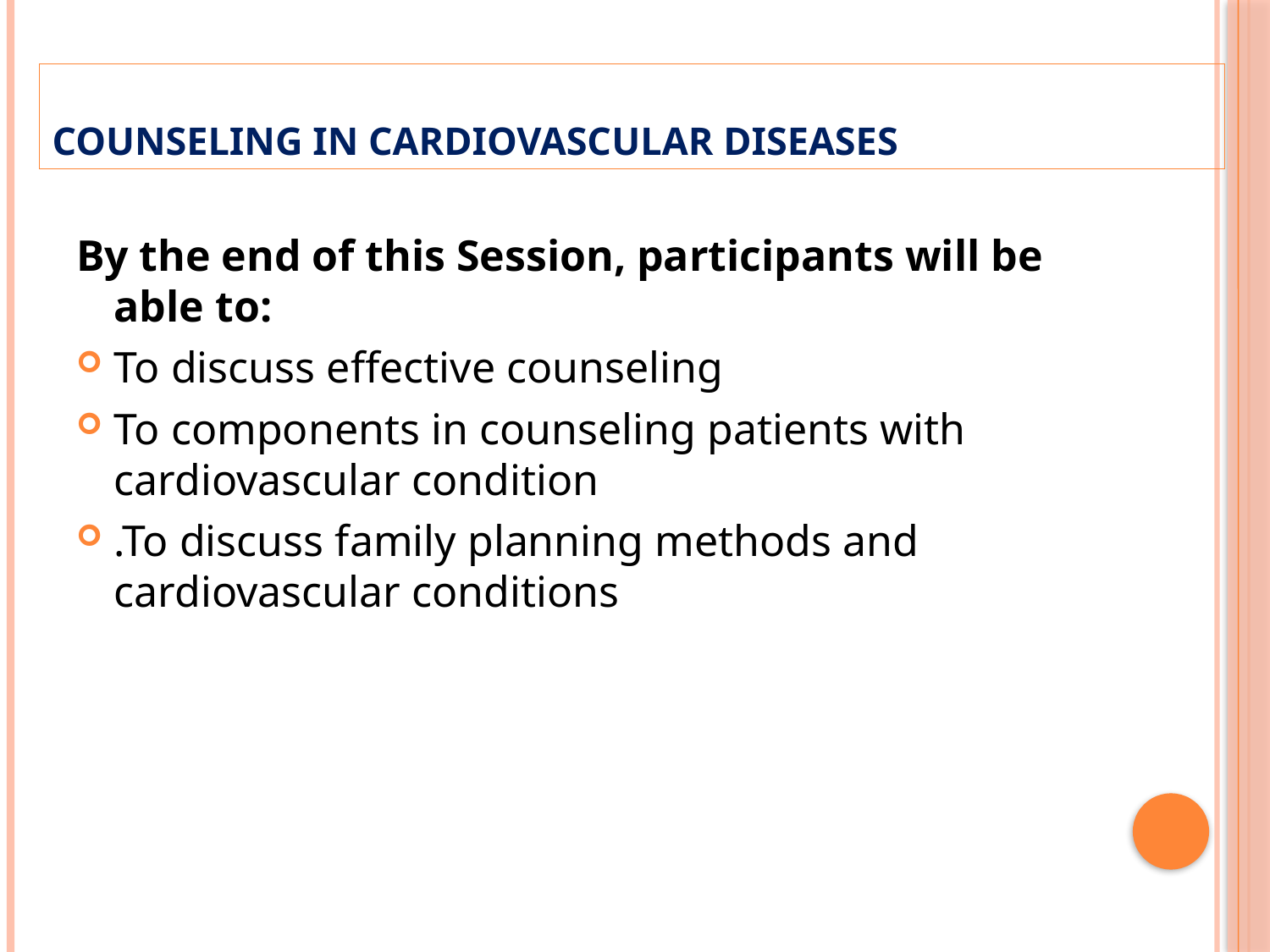

# Counseling in cardiovascular diseases
By the end of this Session, participants will be able to:
To discuss effective counseling
To components in counseling patients with cardiovascular condition
.To discuss family planning methods and cardiovascular conditions

## Slide 3
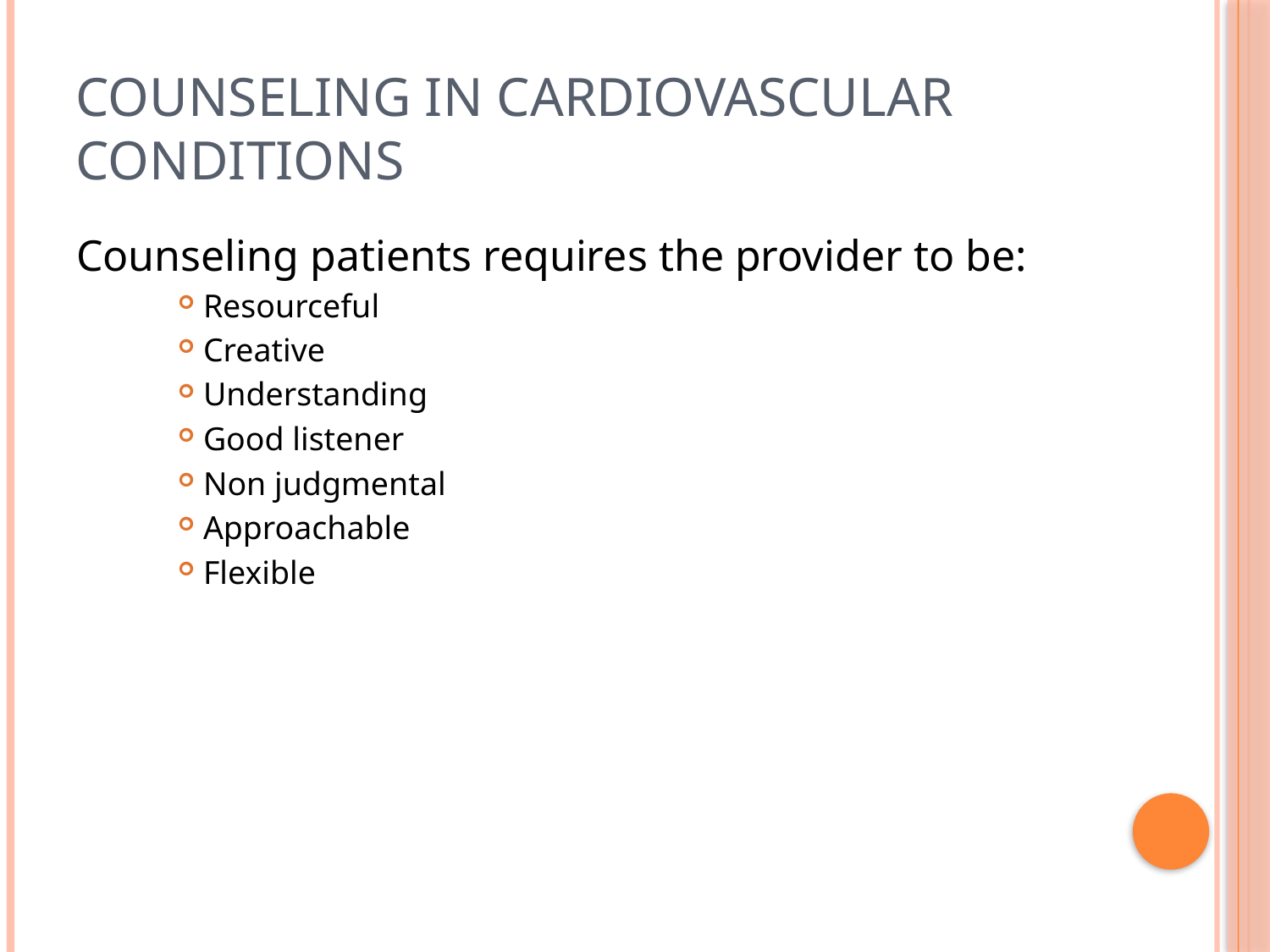

# Counseling in cardiovascular conditions
Counseling patients requires the provider to be:
Resourceful
Creative
Understanding
Good listener
Non judgmental
Approachable
Flexible

## Slide 4
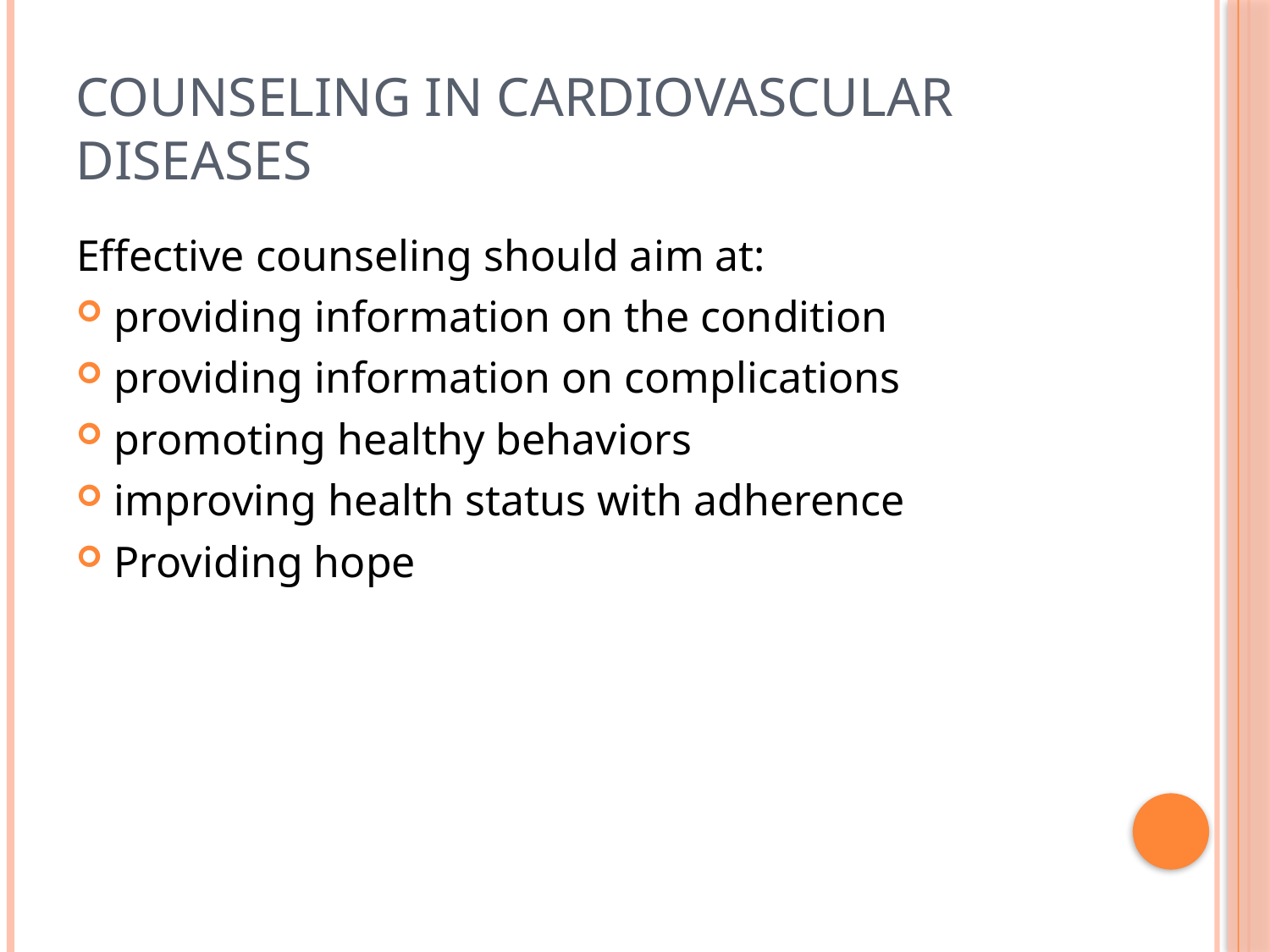

# counseling in cardiovascular diseases
Effective counseling should aim at:
providing information on the condition
providing information on complications
promoting healthy behaviors
improving health status with adherence
Providing hope

## Slide 5
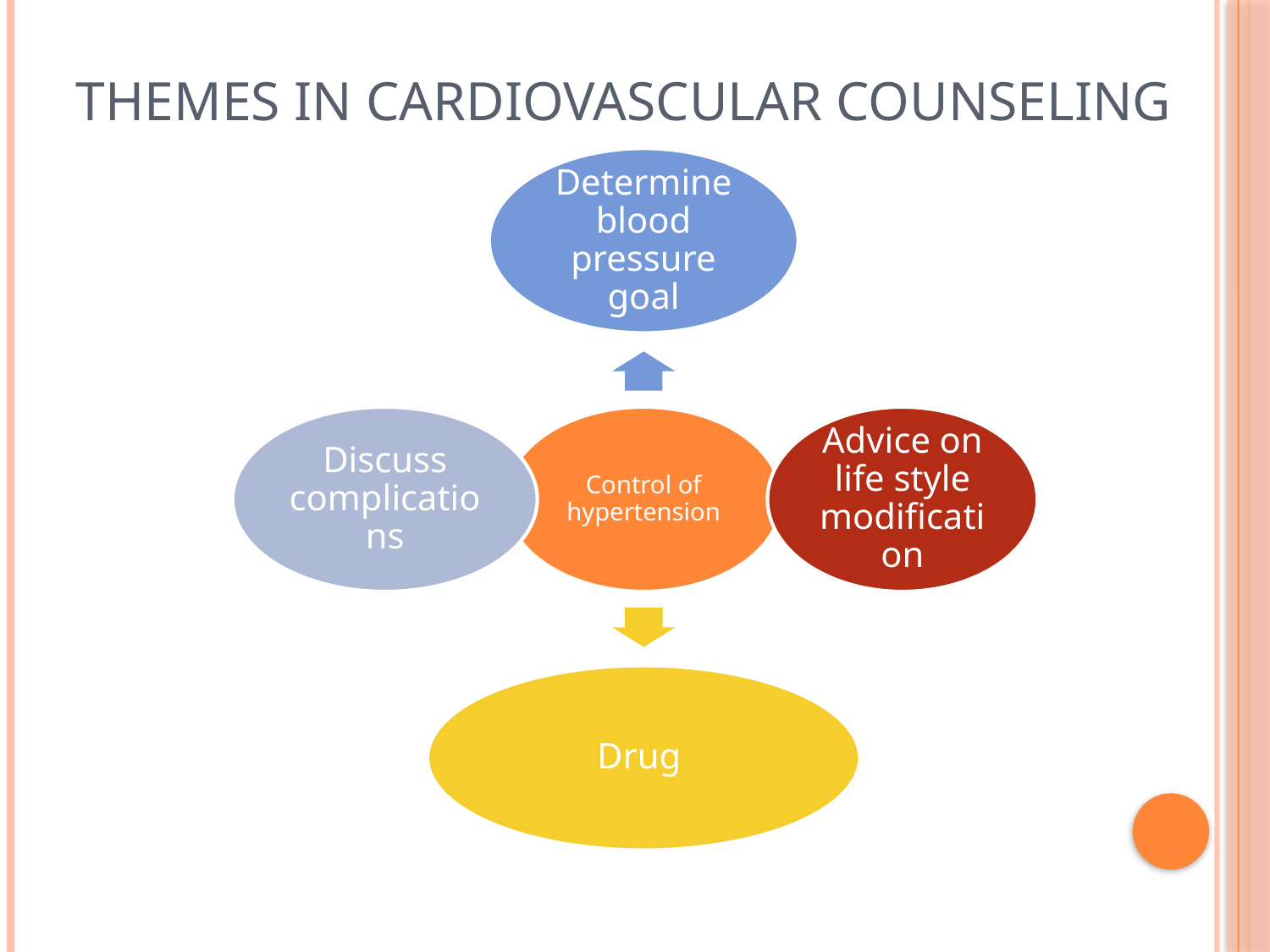

# Themes in cardiovascular counseling

## Slide 6
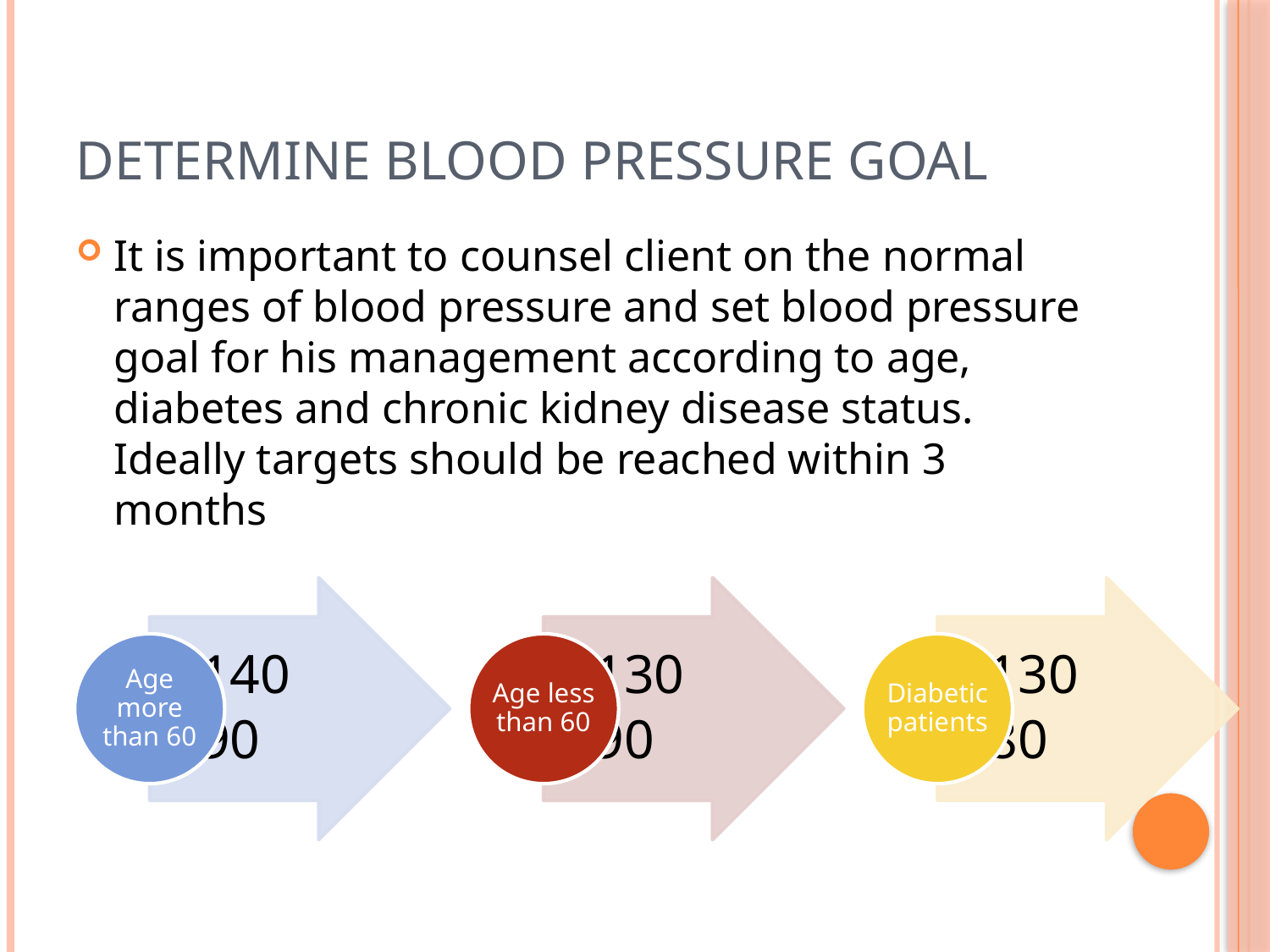

# Determine blood pressure goal
It is important to counsel client on the normal ranges of blood pressure and set blood pressure goal for his management according to age, diabetes and chronic kidney disease status. Ideally targets should be reached within 3 months

## Slide 7
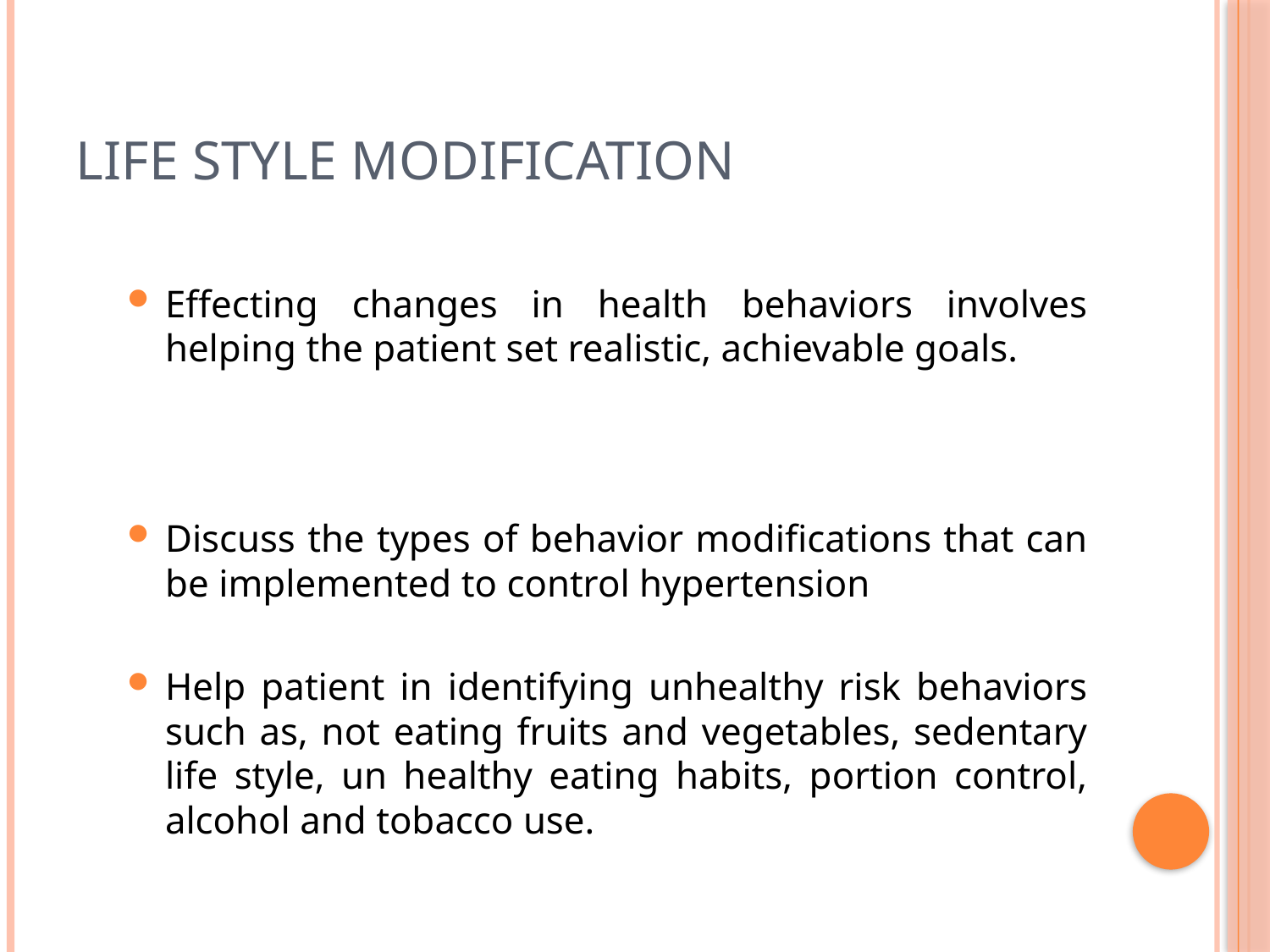

# Life style modification
Effecting changes in health behaviors involves helping the patient set realistic, achievable goals.
Discuss the types of behavior modifications that can be implemented to control hypertension
Help patient in identifying unhealthy risk behaviors such as, not eating fruits and vegetables, sedentary life style, un healthy eating habits, portion control, alcohol and tobacco use.

## Slide 8
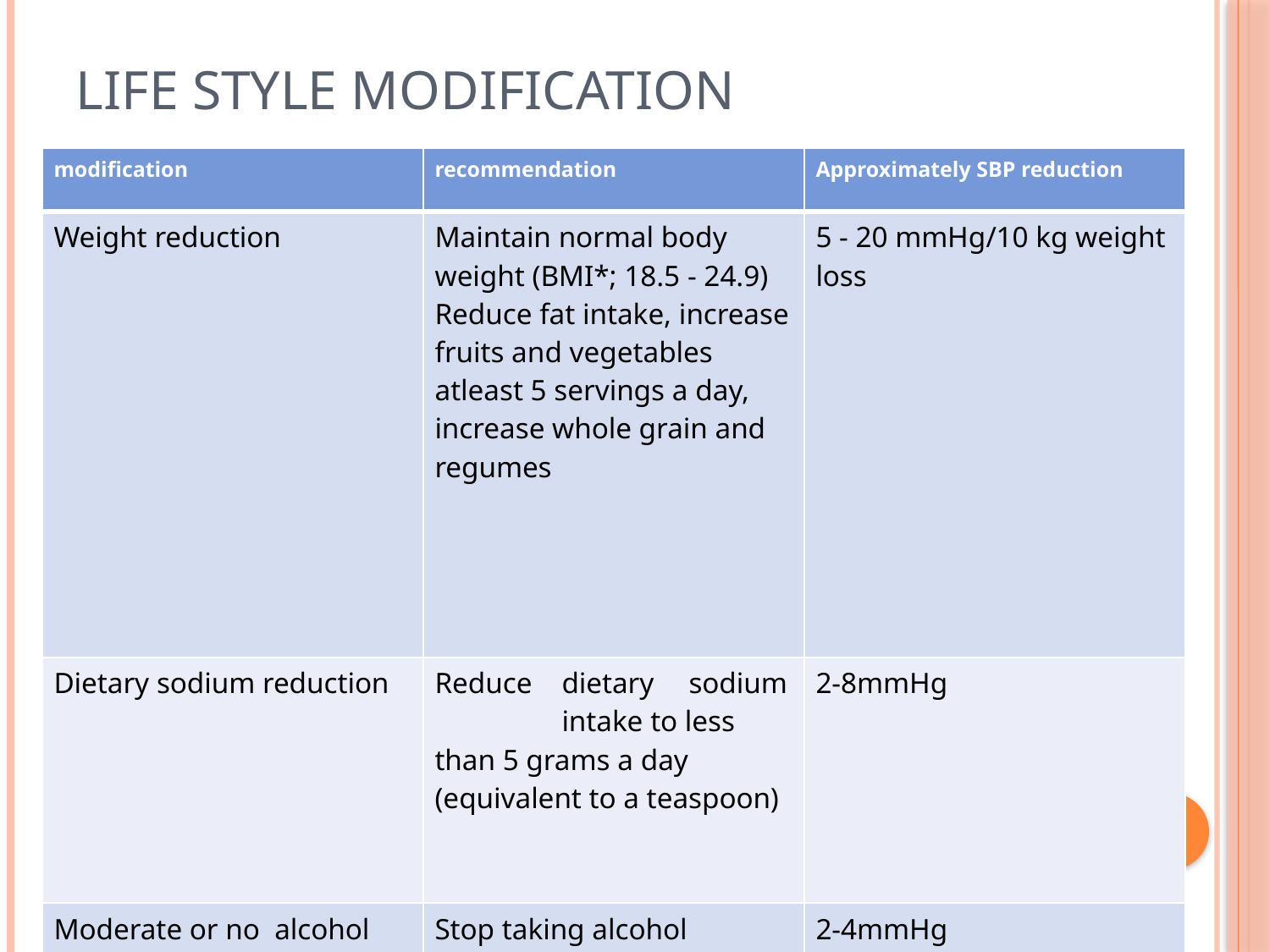

# Life style modification
| modification | recommendation | Approximately SBP reduction |
| --- | --- | --- |
| Weight reduction | Maintain normal body weight (BMI\*; 18.5 - 24.9) Reduce fat intake, increase fruits and vegetables atleast 5 servings a day, increase whole grain and regumes | 5 - 20 mmHg/10 kg weight loss |
| Dietary sodium reduction | Reduce dietary sodium intake to less than 5 grams a day (equivalent to a teaspoon) | 2-8mmHg |
| Moderate or no alcohol consumption | Stop taking alcohol | 2-4mmHg |
| Physical activity | walking at least 30 min/day, most days of the week, minimum of 150 min/week | 4 - 9 mmHg |

## Slide 9
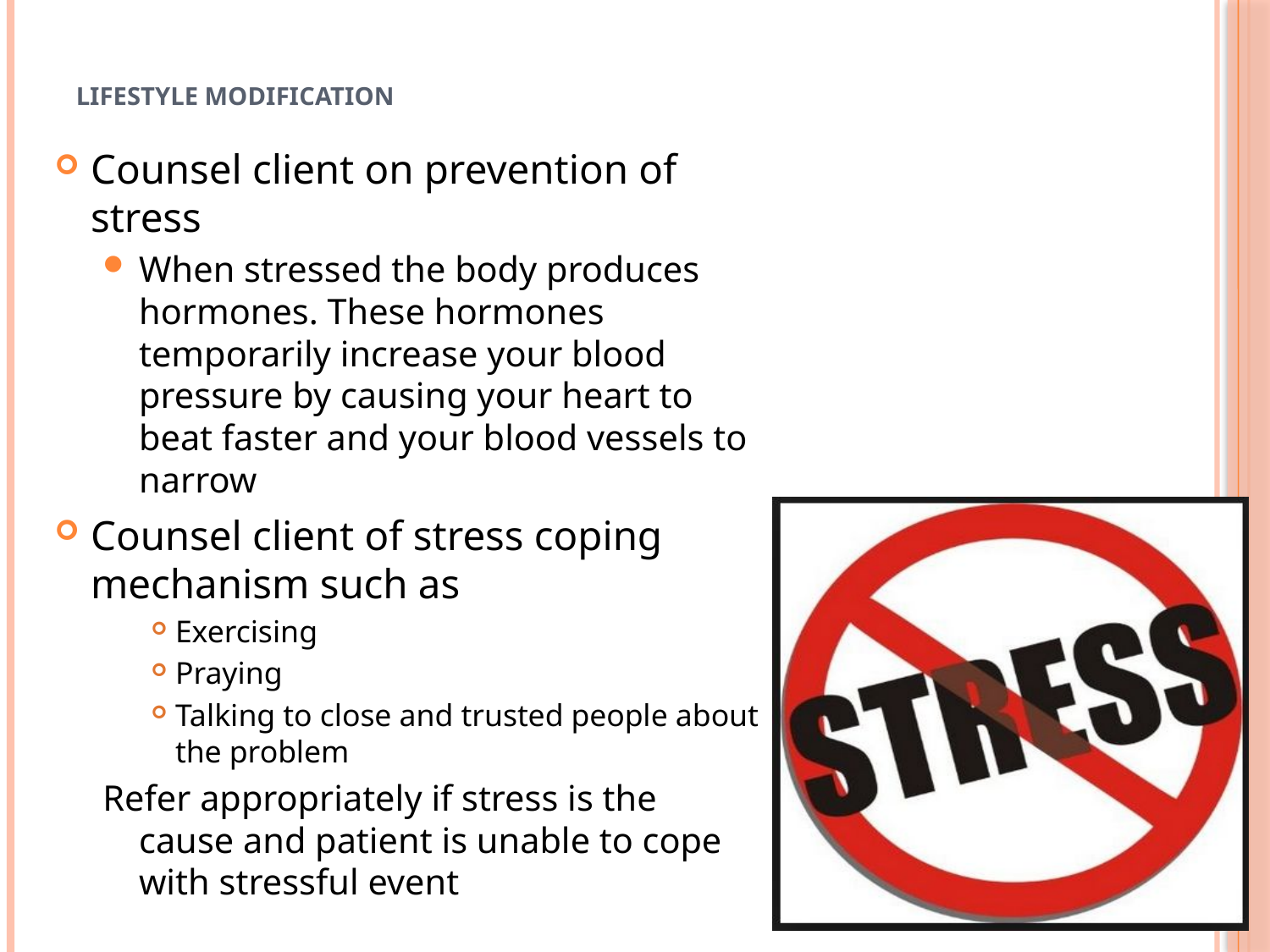

# Lifestyle modification
Counsel client on prevention of stress
When stressed the body produces hormones. These hormones temporarily increase your blood pressure by causing your heart to beat faster and your blood vessels to narrow
Counsel client of stress coping mechanism such as
Exercising
Praying
Talking to close and trusted people about the problem
Refer appropriately if stress is the cause and patient is unable to cope with stressful event

## Slide 10
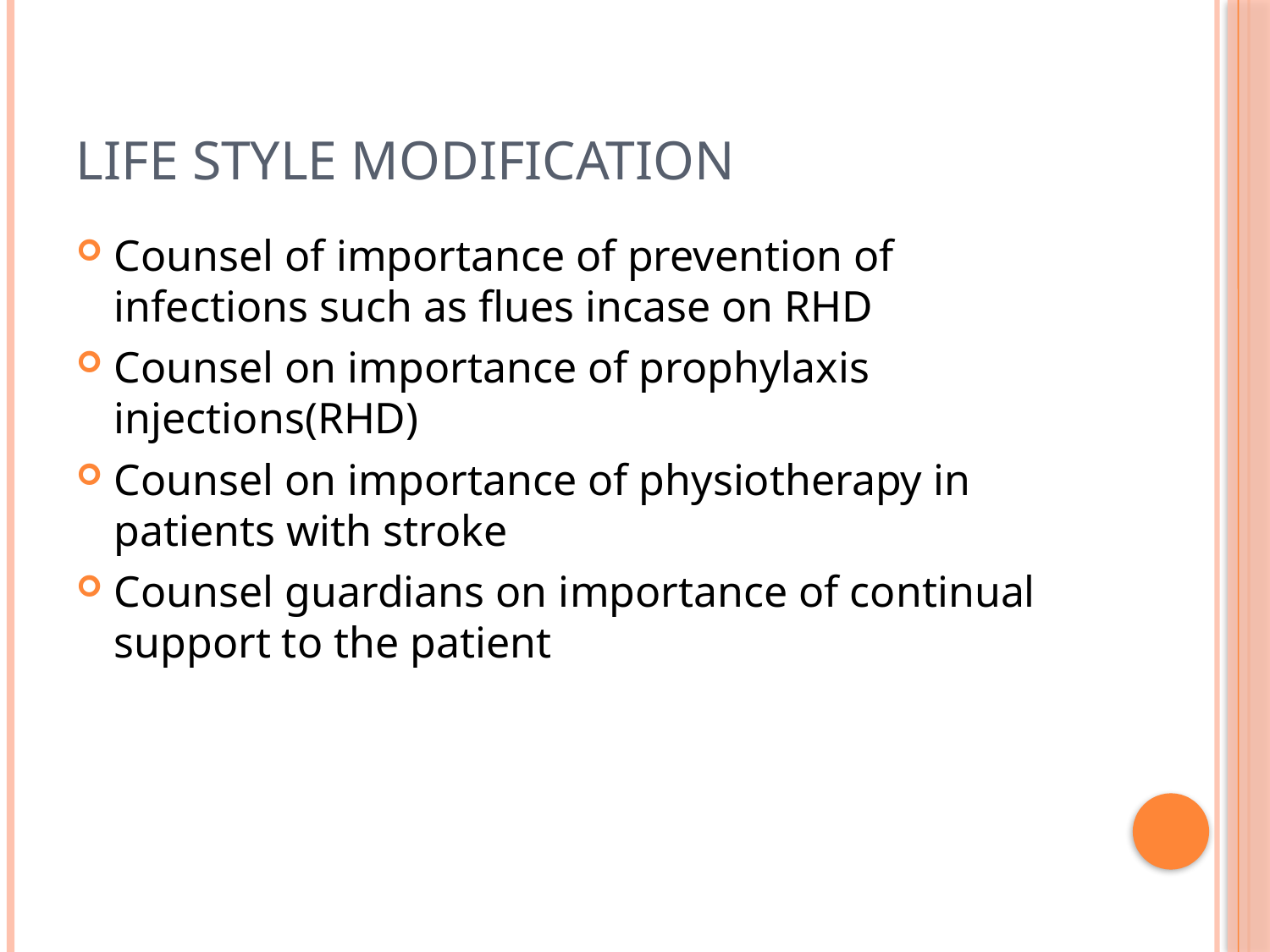

# Life style modification
Counsel of importance of prevention of infections such as flues incase on RHD
Counsel on importance of prophylaxis injections(RHD)
Counsel on importance of physiotherapy in patients with stroke
Counsel guardians on importance of continual support to the patient

## Slide 11
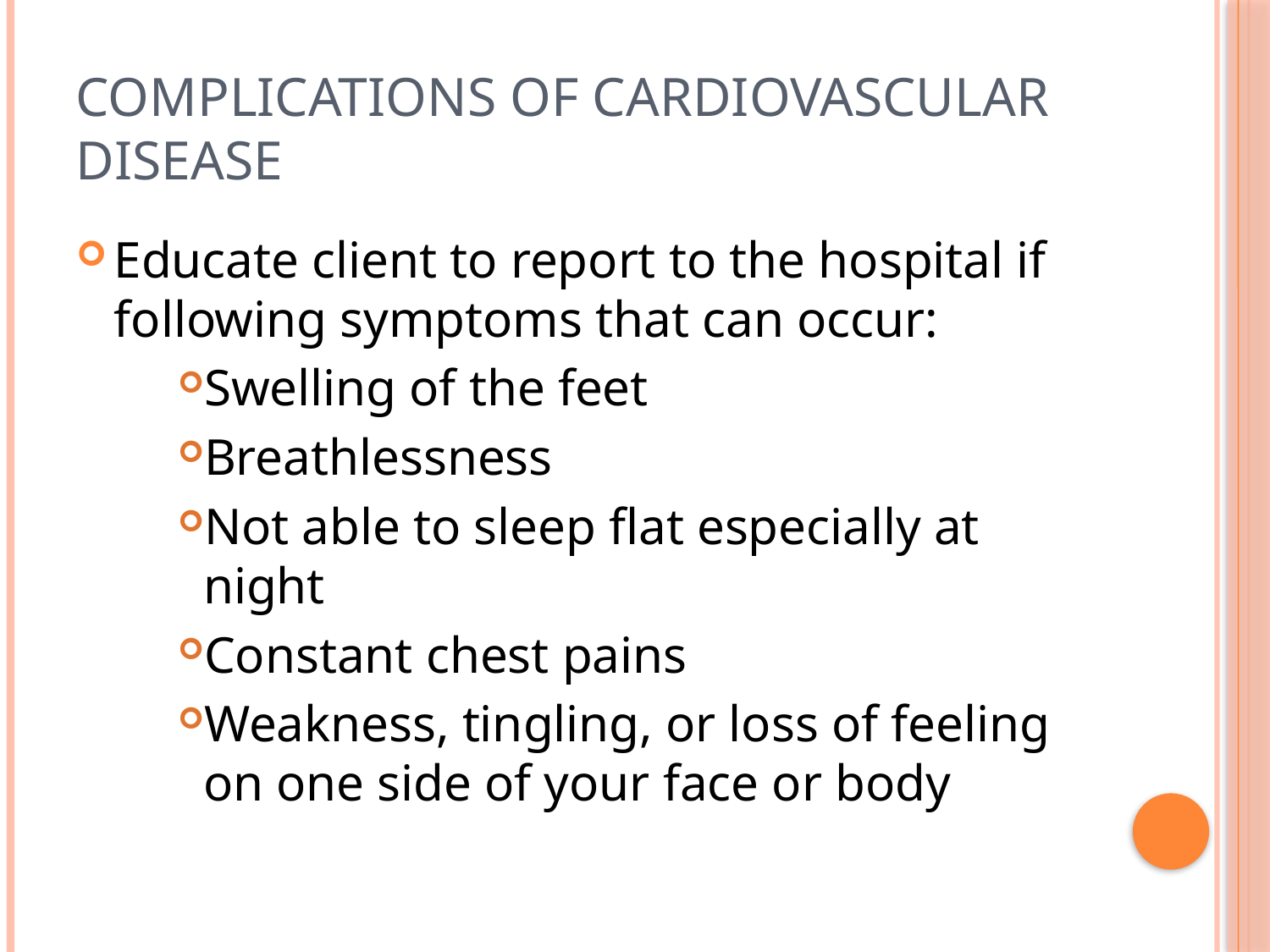

# Complications of cardiovascular disease
Educate client to report to the hospital if following symptoms that can occur:
Swelling of the feet
Breathlessness
Not able to sleep flat especially at night
Constant chest pains
Weakness, tingling, or loss of feeling on one side of your face or body

## Slide 12
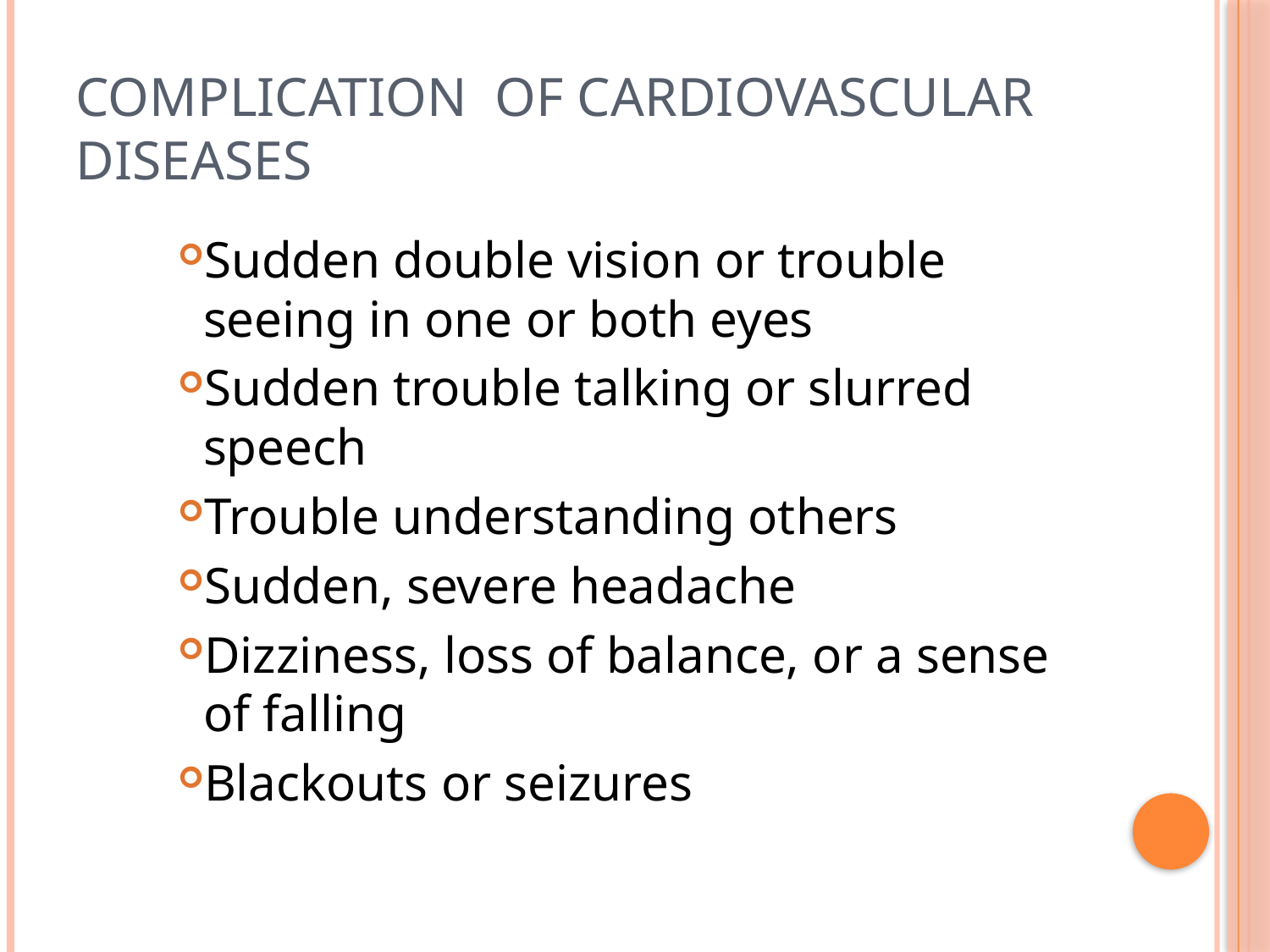

# Complication of cardiovascular diseases
Sudden double vision or trouble seeing in one or both eyes
Sudden trouble talking or slurred speech
Trouble understanding others
Sudden, severe headache
Dizziness, loss of balance, or a sense of falling
Blackouts or seizures

## Slide 13
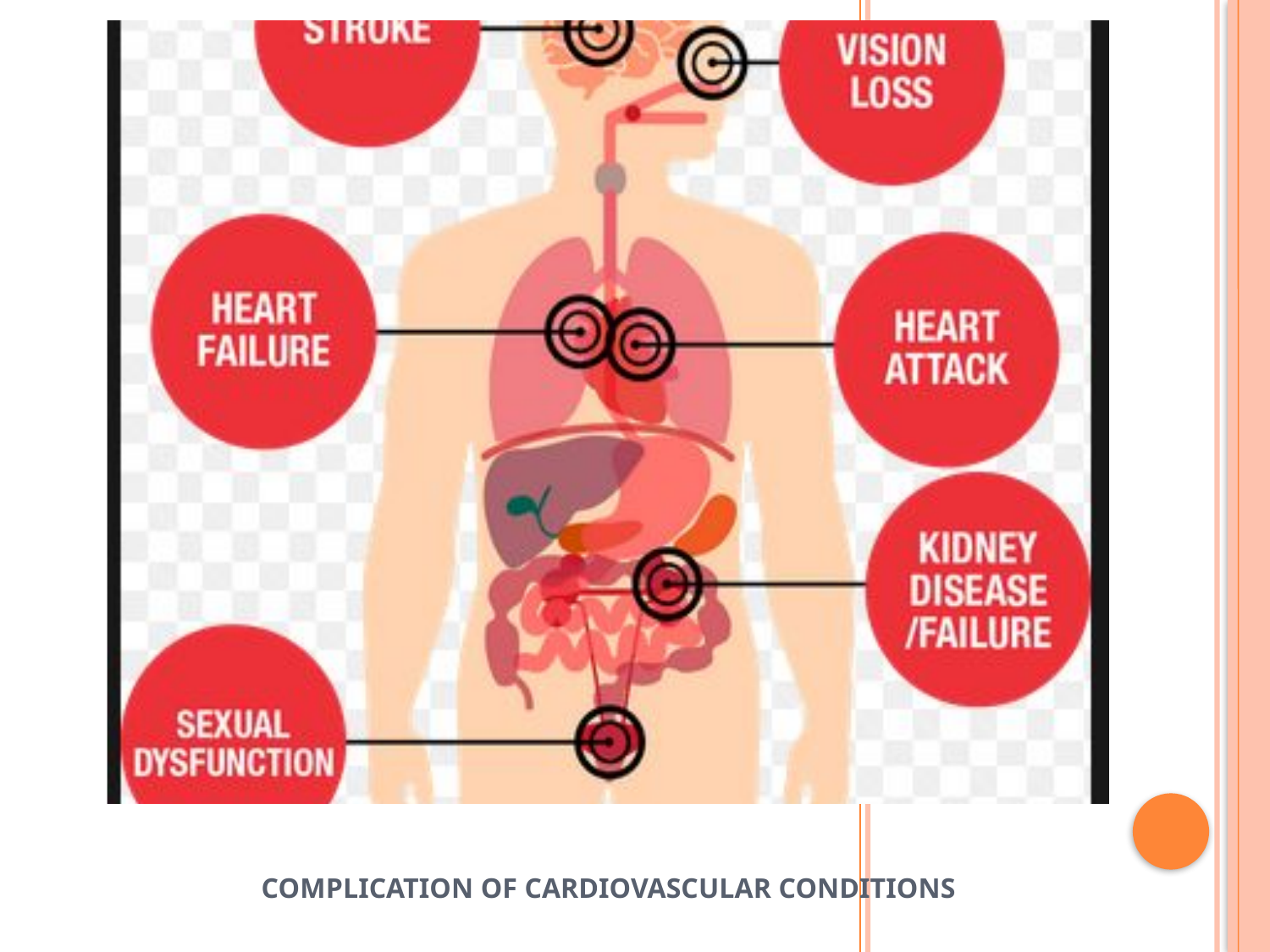

# Complication of cardiovascular conditions

## Slide 14
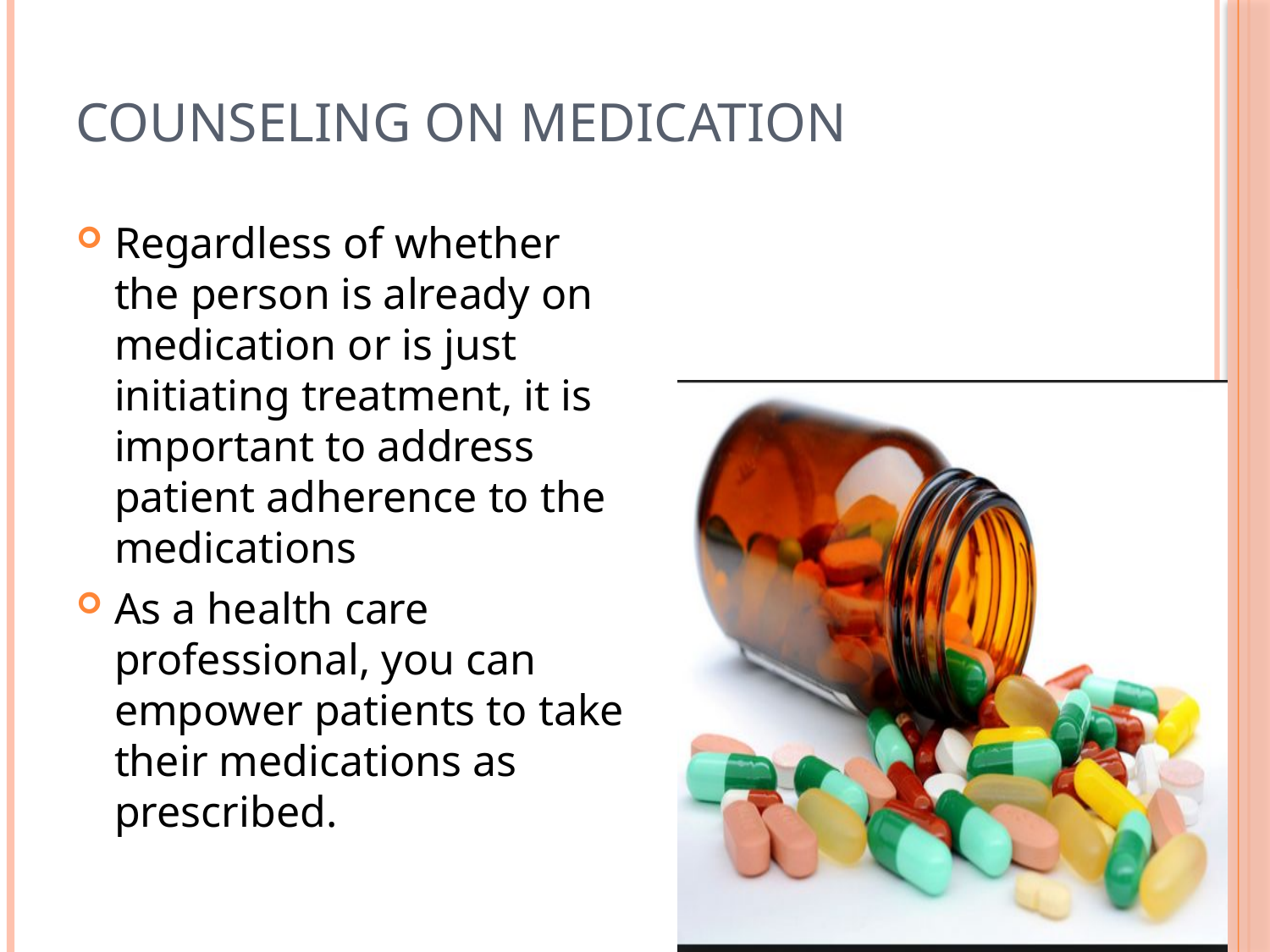

# Counseling on medication
Regardless of whether the person is already on medication or is just initiating treatment, it is important to address patient adherence to the medications
As a health care professional, you can empower patients to take their medications as prescribed.

## Slide 15
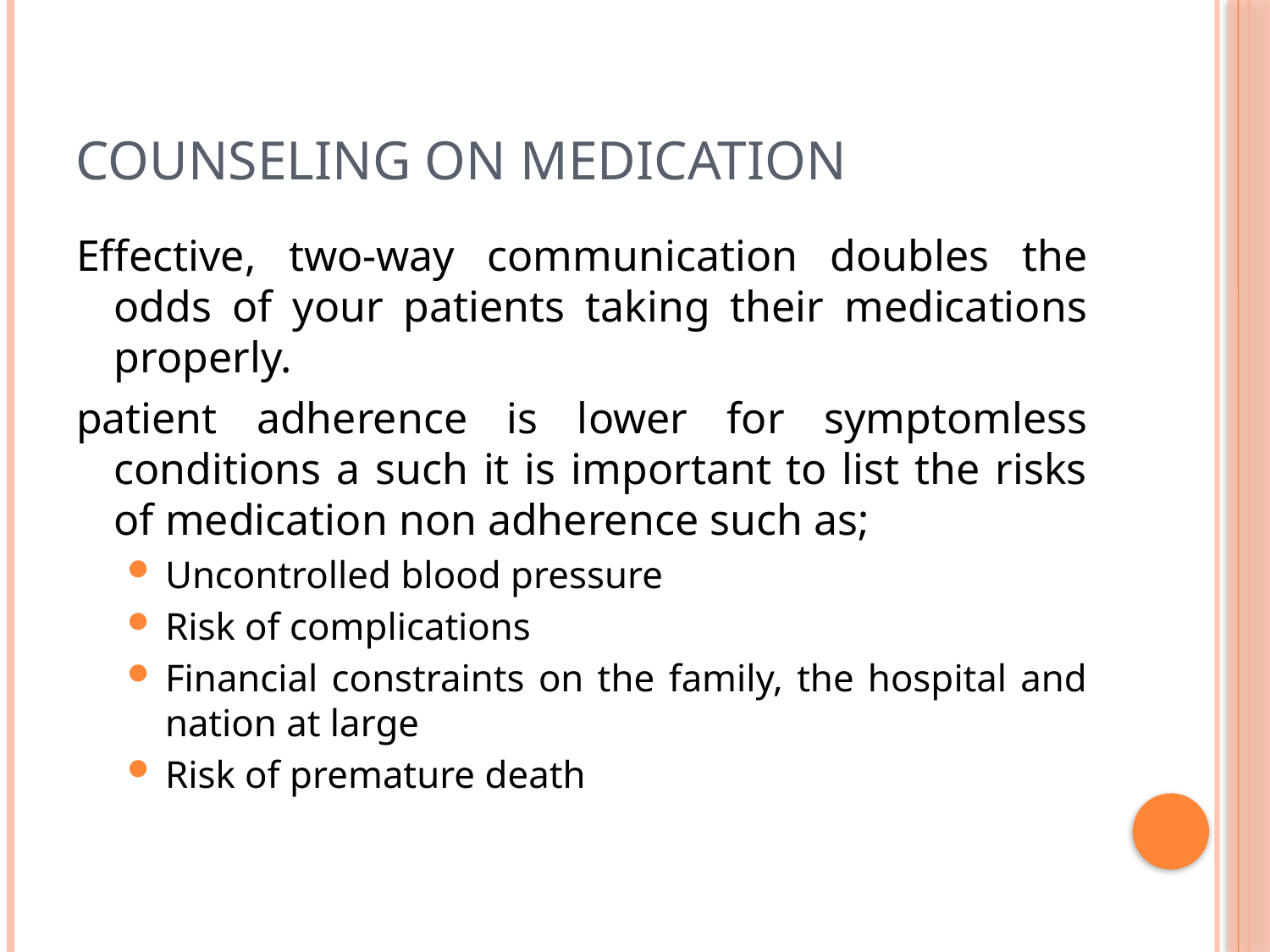

# Counseling on medication
Effective, two-way communication doubles the odds of your patients taking their medications properly.
patient adherence is lower for symptomless conditions a such it is important to list the risks of medication non adherence such as;
Uncontrolled blood pressure
Risk of complications
Financial constraints on the family, the hospital and nation at large
Risk of premature death

## Slide 16
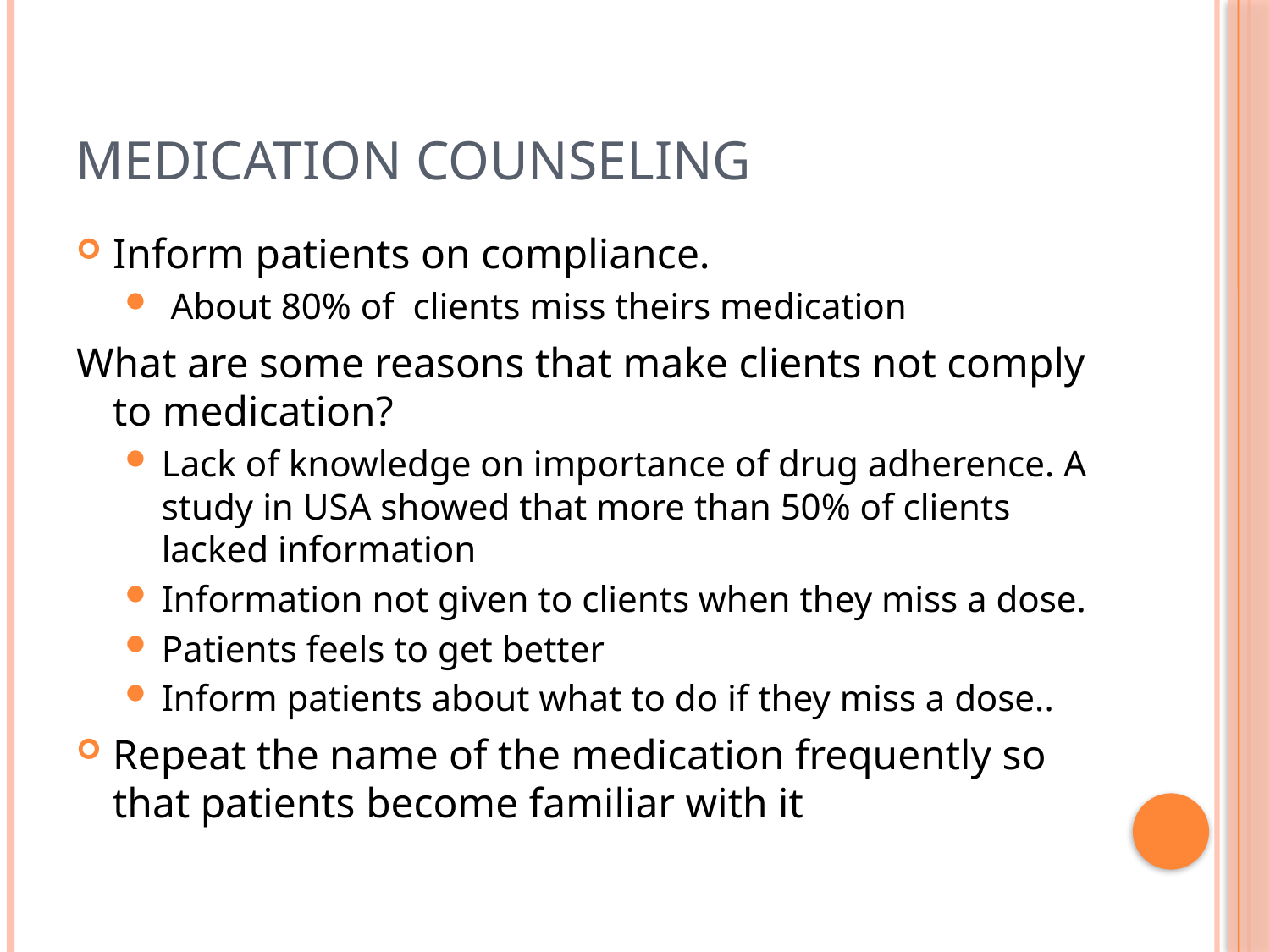

# Medication counseling
Inform patients on compliance.
 About 80% of clients miss theirs medication
What are some reasons that make clients not comply to medication?
Lack of knowledge on importance of drug adherence. A study in USA showed that more than 50% of clients lacked information
Information not given to clients when they miss a dose.
Patients feels to get better
Inform patients about what to do if they miss a dose..
Repeat the name of the medication frequently so that patients become familiar with it

## Slide 17
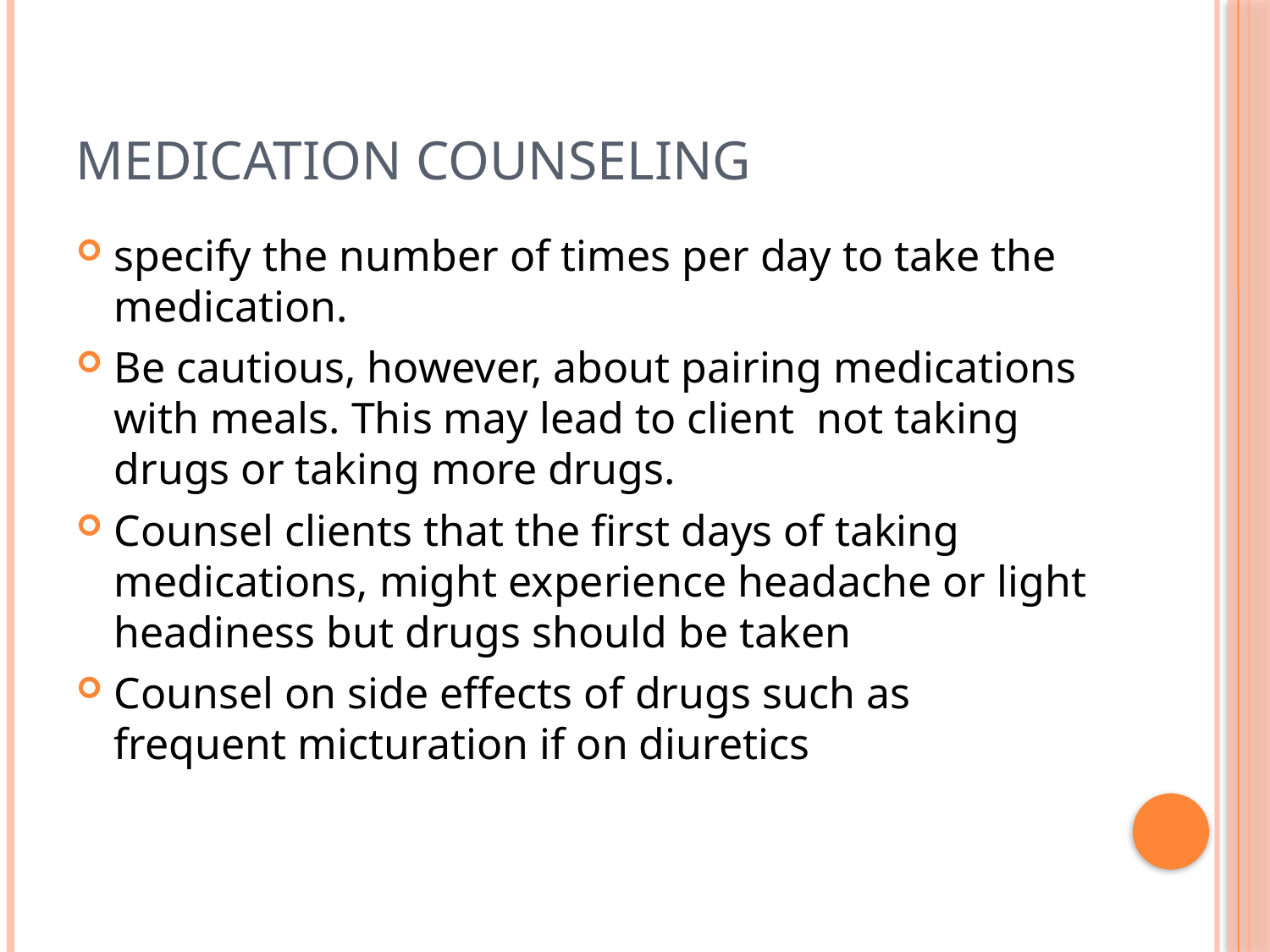

# Medication counseling
specify the number of times per day to take the medication.
Be cautious, however, about pairing medications with meals. This may lead to client not taking drugs or taking more drugs.
Counsel clients that the first days of taking medications, might experience headache or light headiness but drugs should be taken
Counsel on side effects of drugs such as frequent micturation if on diuretics

## Slide 18
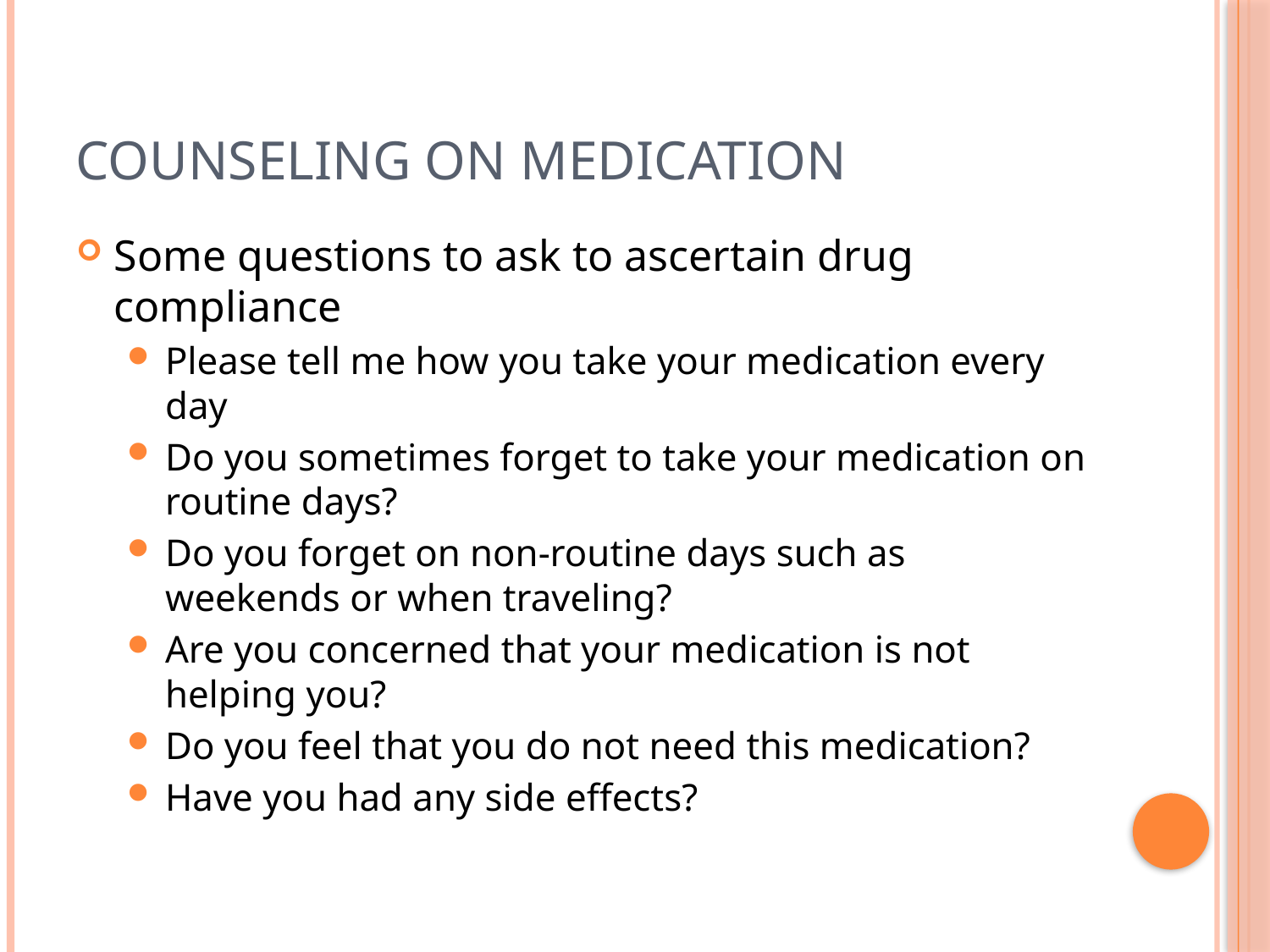

# Counseling on medication
Some questions to ask to ascertain drug compliance
Please tell me how you take your medication every day
Do you sometimes forget to take your medication on routine days?
Do you forget on non-routine days such as weekends or when traveling?
Are you concerned that your medication is not helping you?
Do you feel that you do not need this medication?
Have you had any side effects?

## Slide 19
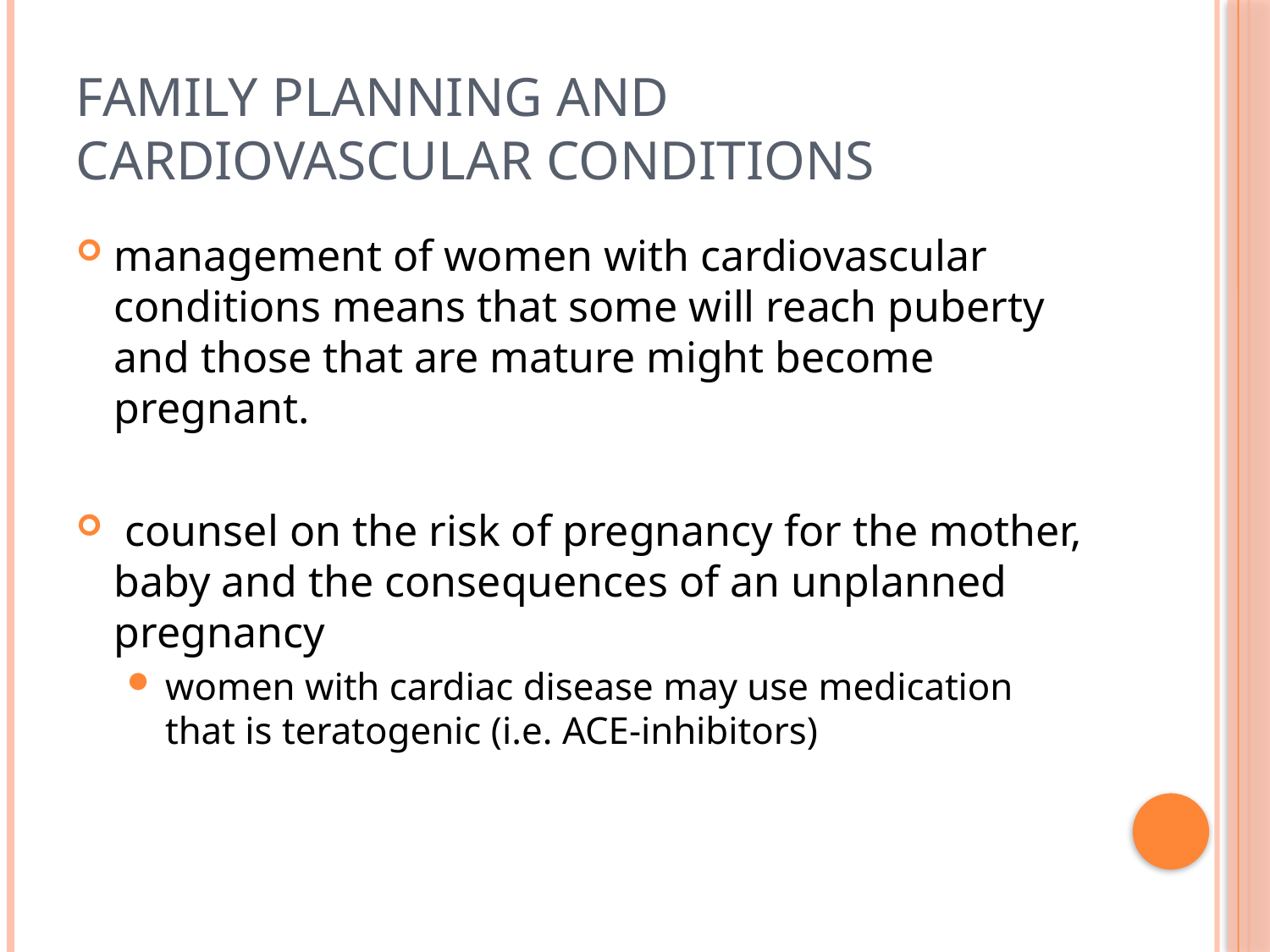

# Family planning and cardiovascular conditions
management of women with cardiovascular conditions means that some will reach puberty and those that are mature might become pregnant.
 counsel on the risk of pregnancy for the mother, baby and the consequences of an unplanned pregnancy
women with cardiac disease may use medication that is teratogenic (i.e. ACE-inhibitors)

## Slide 20
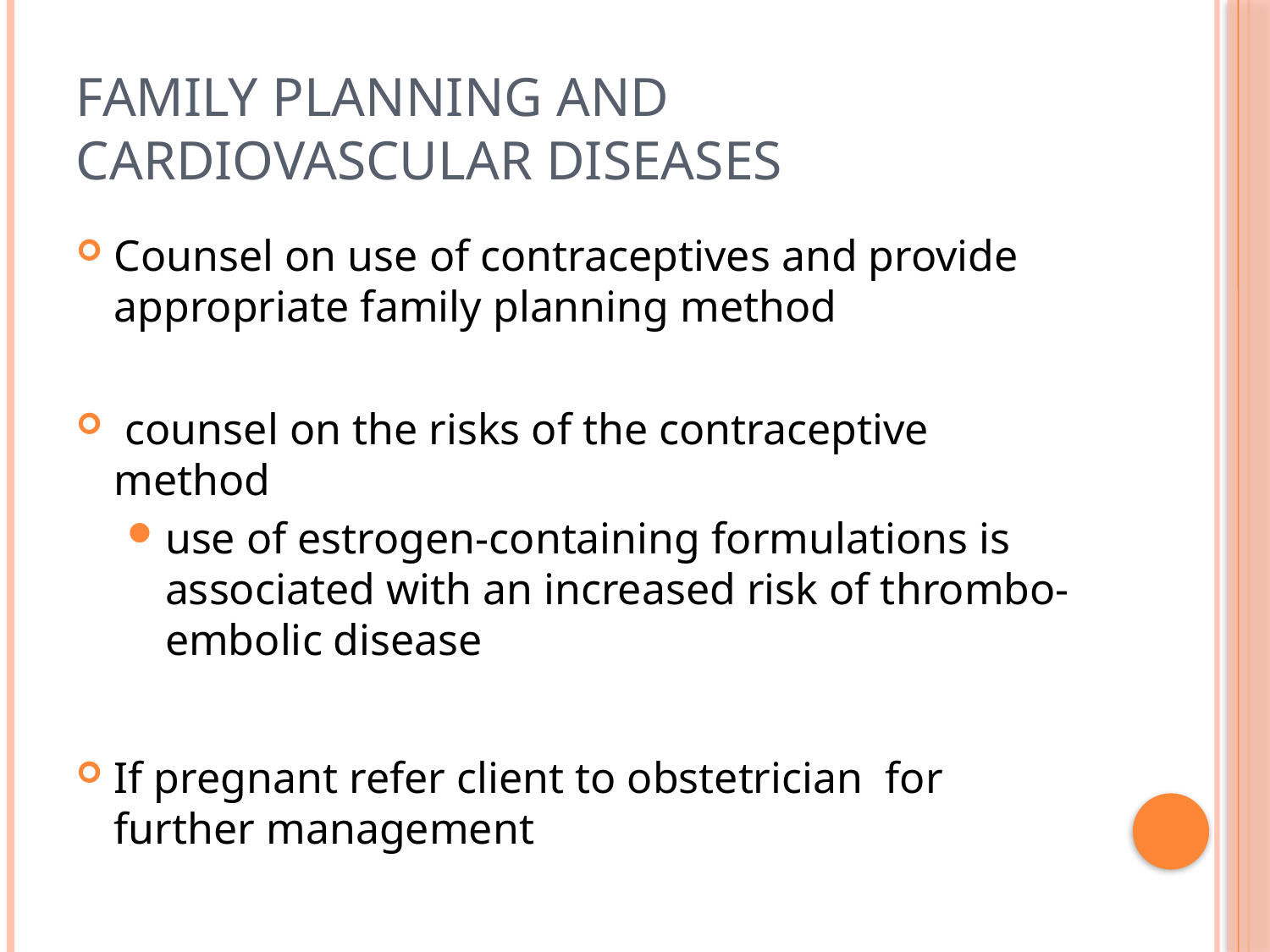

# Family planning and cardiovascular diseases
Counsel on use of contraceptives and provide appropriate family planning method
 counsel on the risks of the contraceptive method
use of estrogen-containing formulations is associated with an increased risk of thrombo-embolic disease
If pregnant refer client to obstetrician for further management

## Slide 21
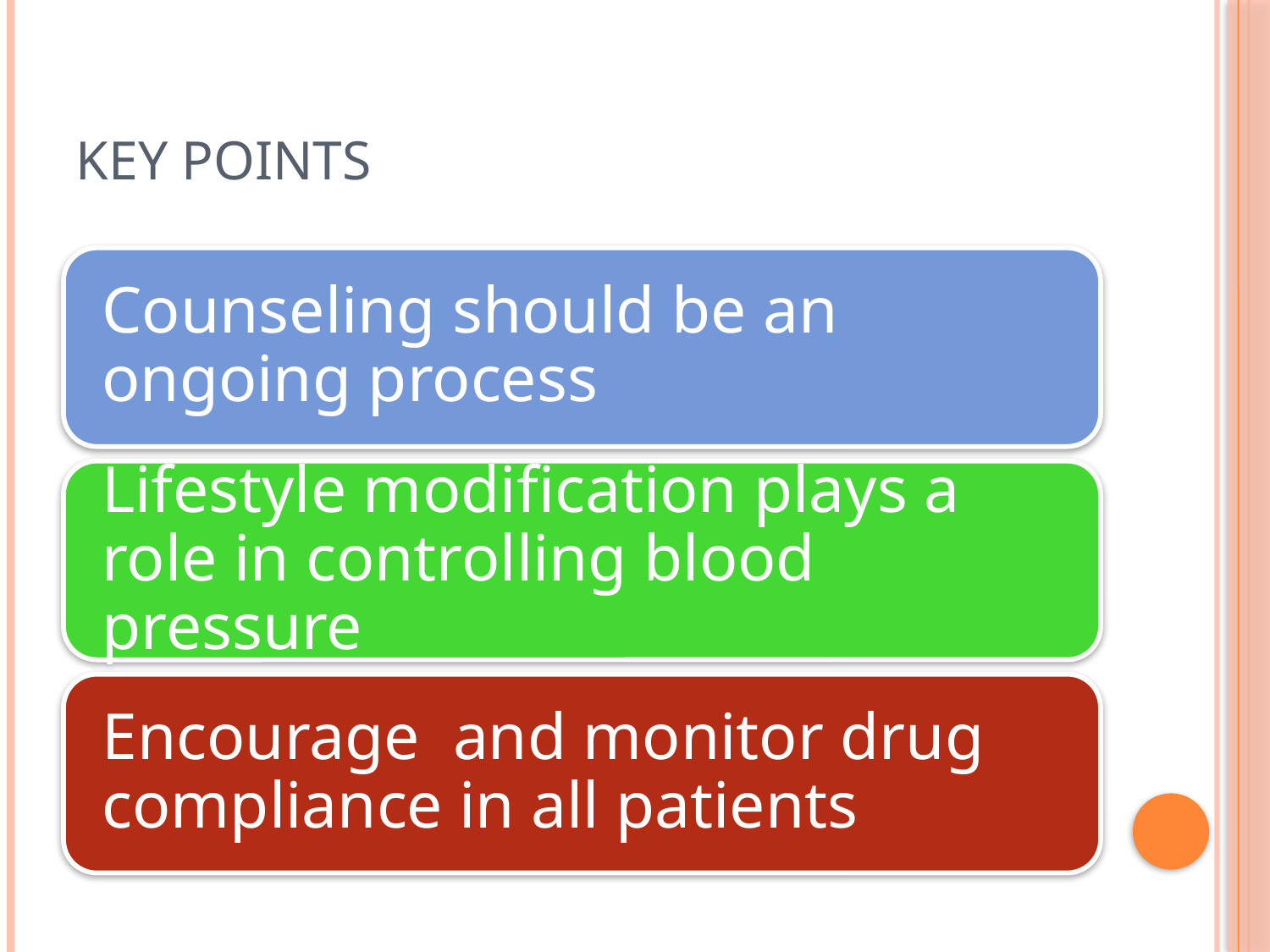

# Key points
