# Supplementary material for: Training Mid-Level Providers to Treat Severe Non-Communicable Diseases in Neno, Malawi through PEN-Plus Strategies
Source: Ann Glob Health. 2022 Aug 11;88(1):69. doi: 10.5334/aogh.3750 (PMC9389951; doi:10.5334/aogh.3750)
Supplement: Didactic Materials. — The supplementary materials contain a suggested didactic training schedule and the PowerPoint presentations used for PEN-Plus training in Neno, Malawi. These materials have been reviewed and accepted by the Malawi Ministry of Health for future PEN-Plus trainings in Malawi. [file agh-88-1-3750-s2.zip › Didactic_Materials/CV_DVT, PE and Anticoagulation.pptx]

## Slide 1
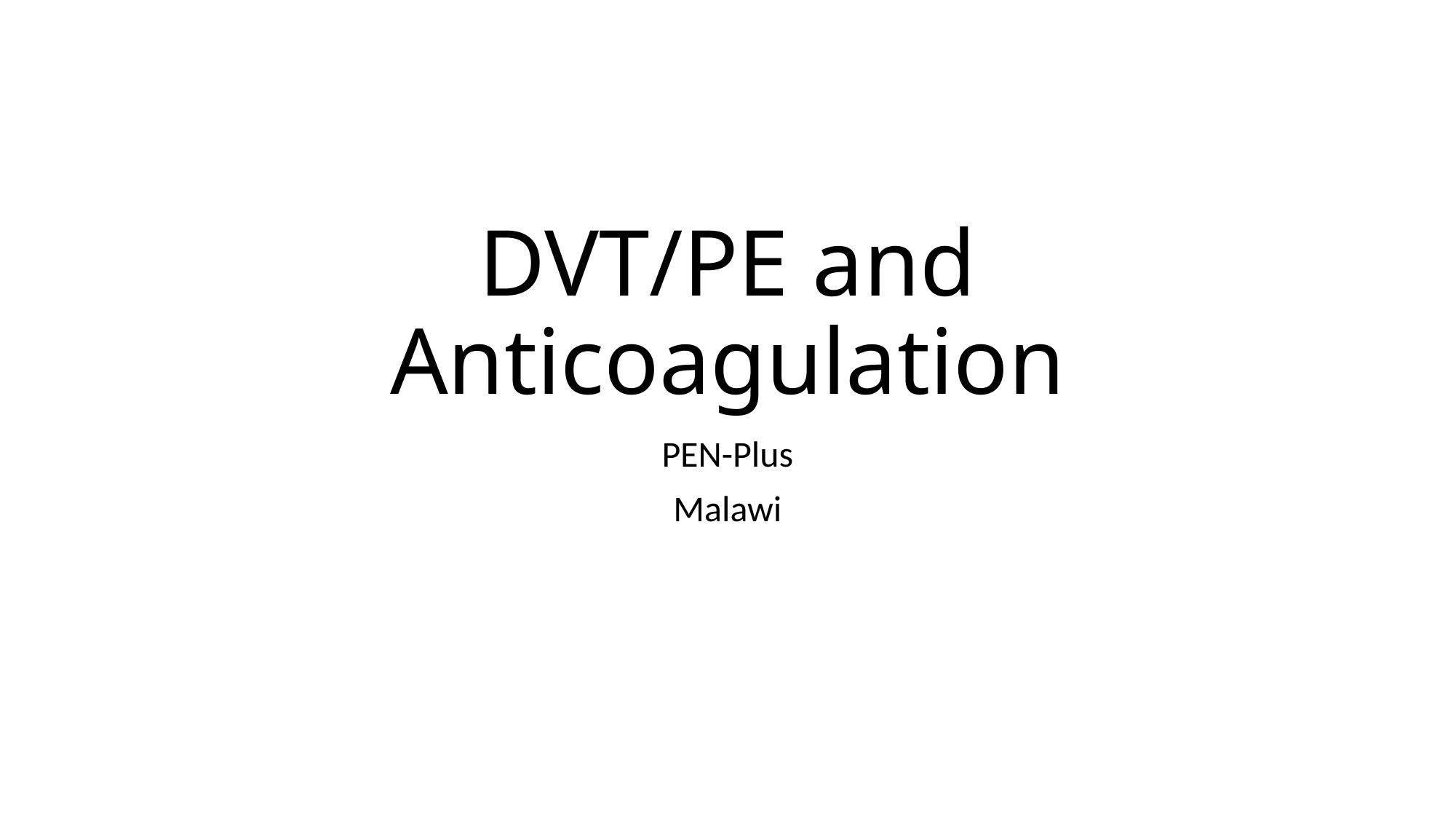

# DVT/PE and Anticoagulation
PEN-Plus
Malawi

## Slide 2
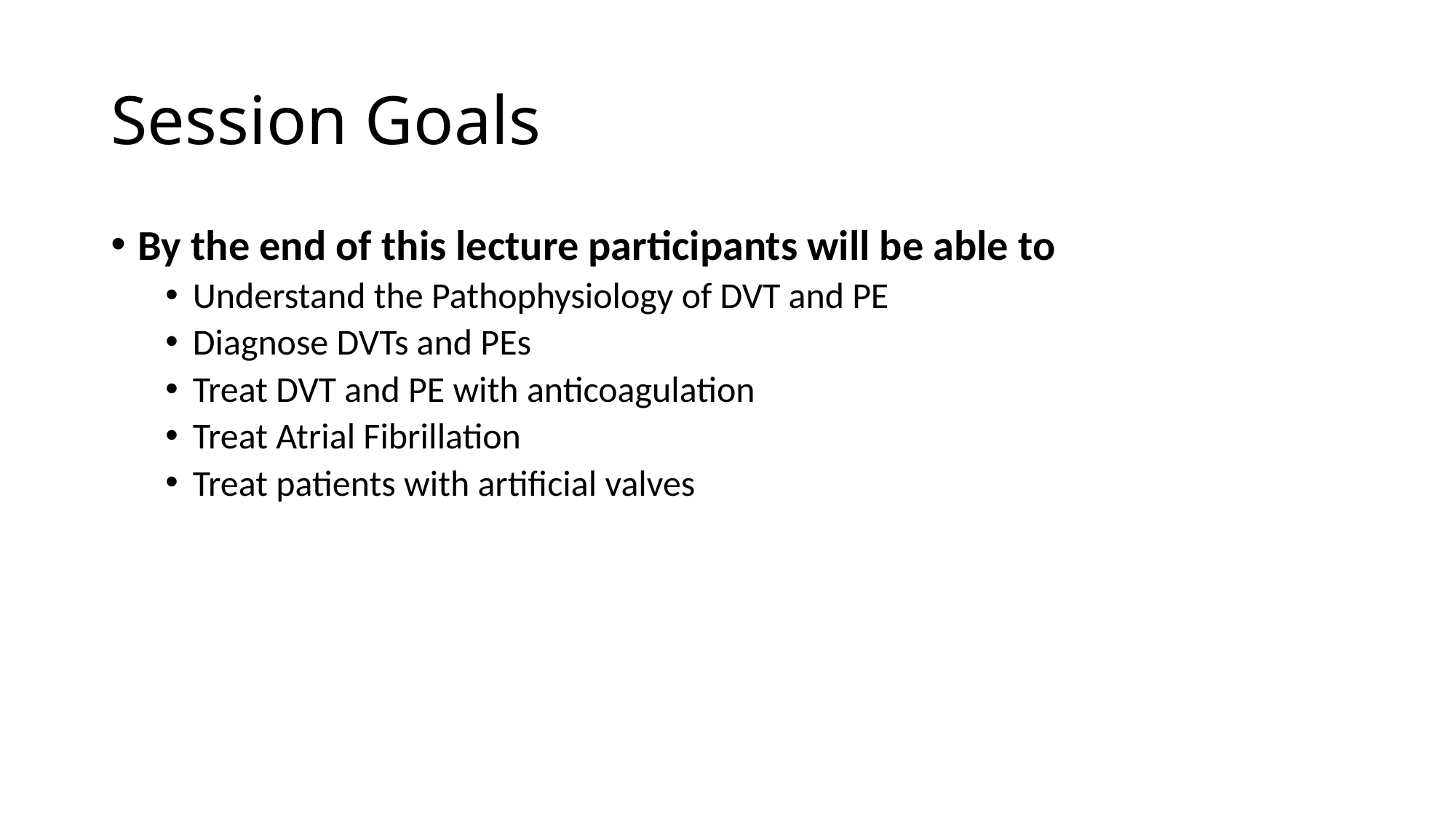

# Session Goals
By the end of this lecture participants will be able to
Understand the Pathophysiology of DVT and PE
Diagnose DVTs and PEs
Treat DVT and PE with anticoagulation
Treat Atrial Fibrillation
Treat patients with artificial valves

## Slide 3
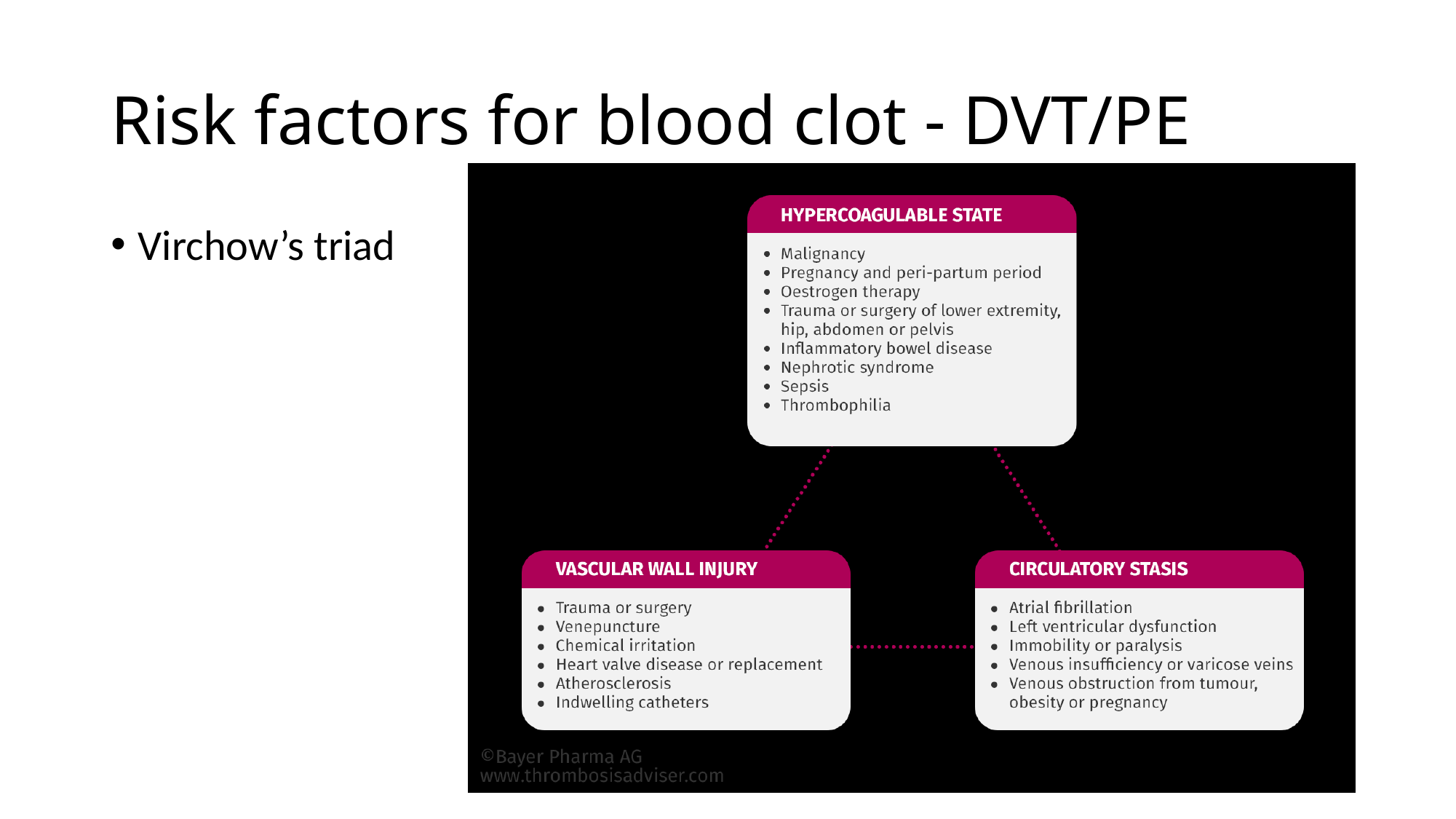

# Risk factors for blood clot - DVT/PE
Virchow’s triad

## Slide 4
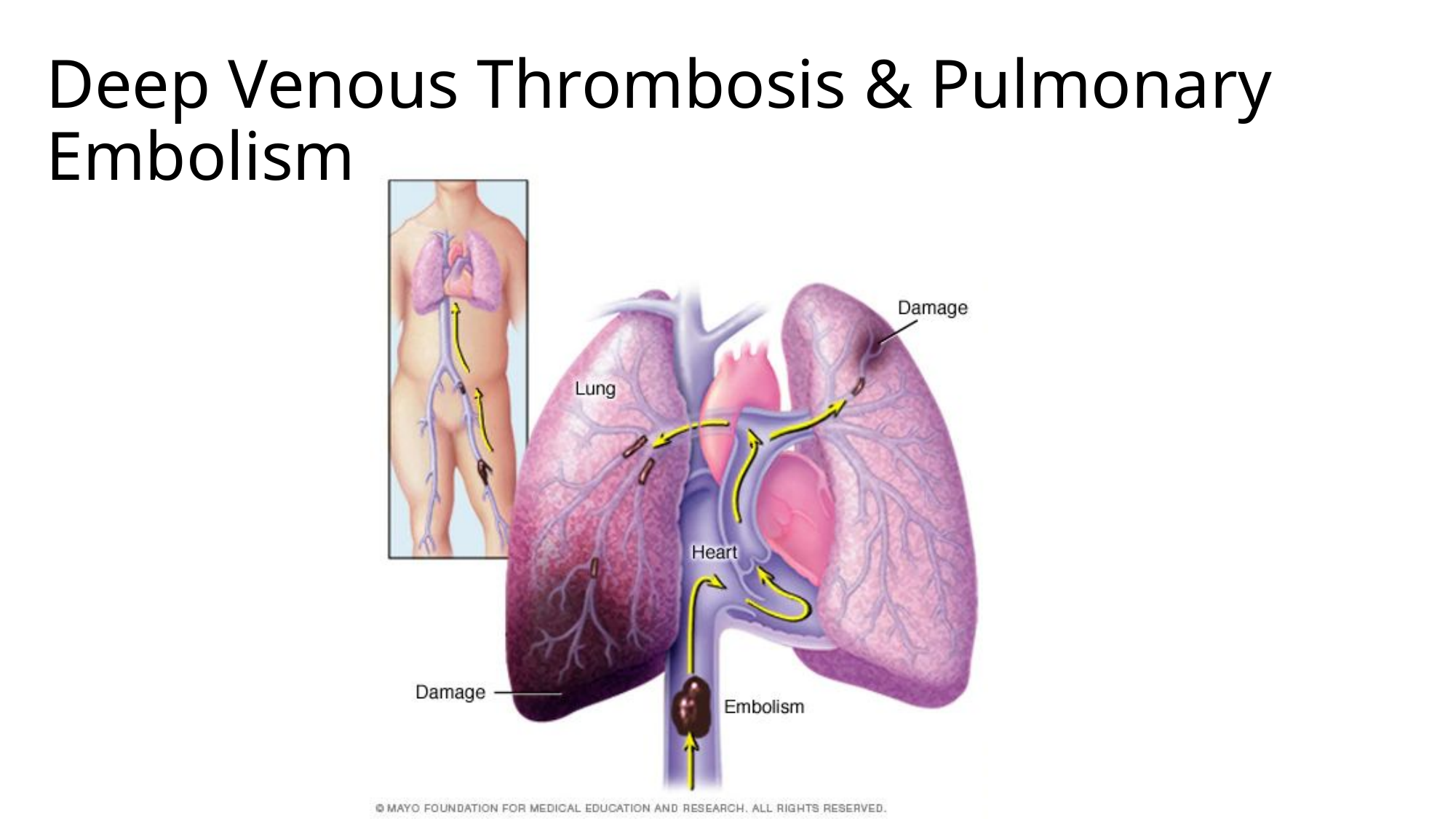

# Deep Venous Thrombosis & Pulmonary Embolism

## Slide 5
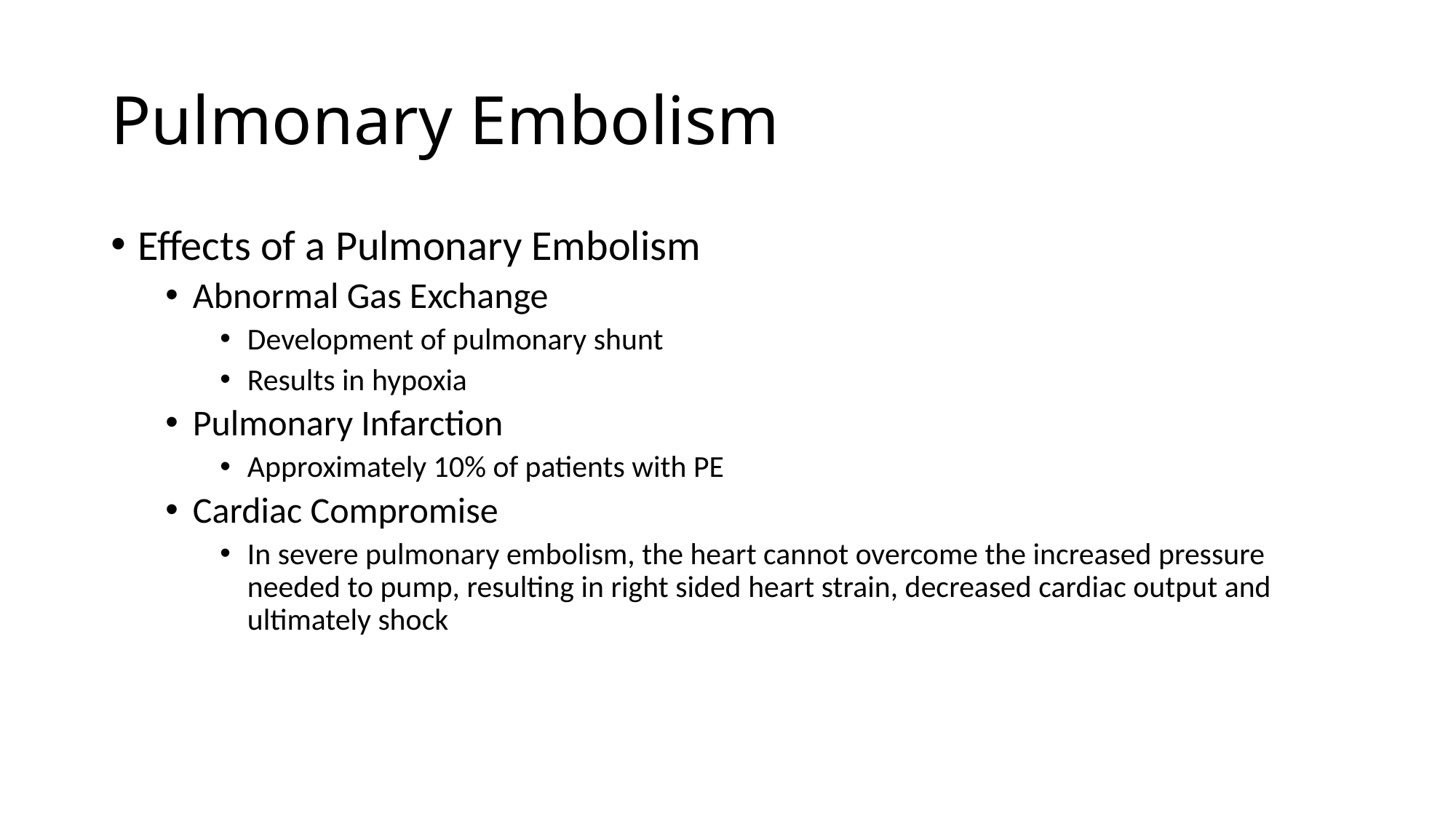

# Pulmonary Embolism
Effects of a Pulmonary Embolism
Abnormal Gas Exchange
Development of pulmonary shunt
Results in hypoxia
Pulmonary Infarction
Approximately 10% of patients with PE
Cardiac Compromise
In severe pulmonary embolism, the heart cannot overcome the increased pressure needed to pump, resulting in right sided heart strain, decreased cardiac output and ultimately shock

## Slide 6
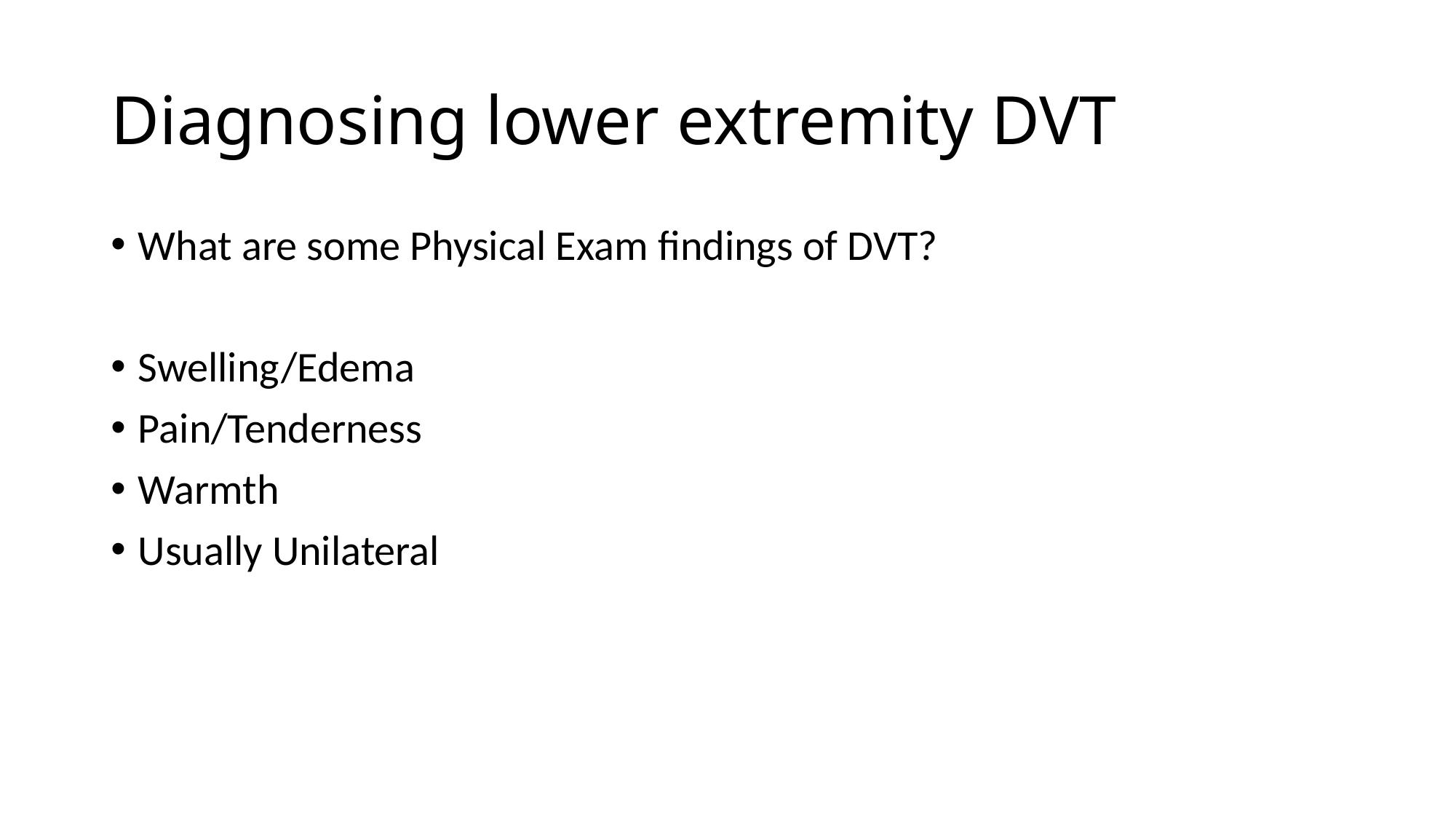

# Diagnosing lower extremity DVT
What are some Physical Exam findings of DVT?
Swelling/Edema
Pain/Tenderness
Warmth
Usually Unilateral

## Slide 7
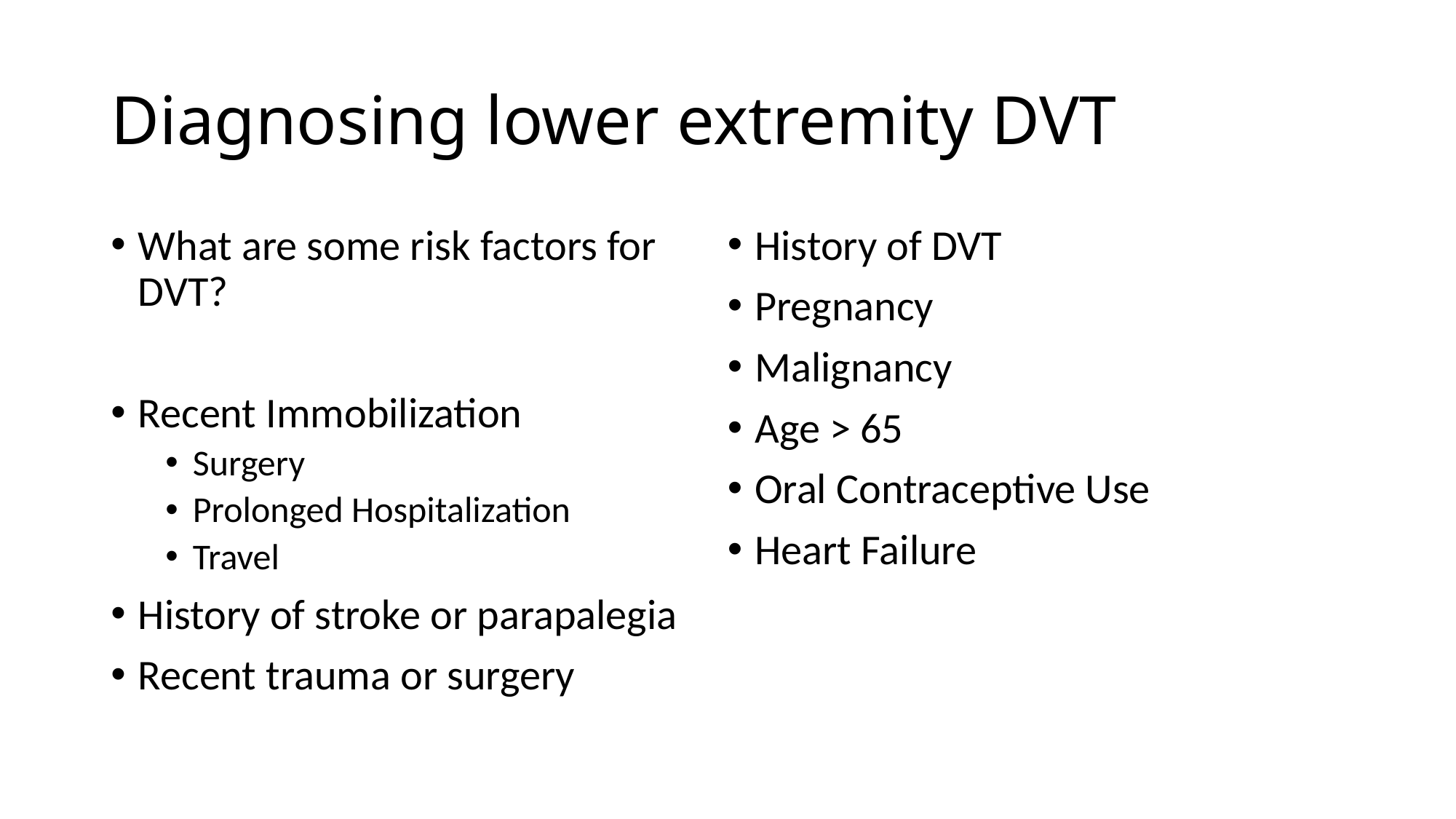

# Diagnosing lower extremity DVT
What are some risk factors for DVT?
Recent Immobilization
Surgery
Prolonged Hospitalization
Travel
History of stroke or parapalegia
Recent trauma or surgery
History of DVT
Pregnancy
Malignancy
Age > 65
Oral Contraceptive Use
Heart Failure

## Slide 8
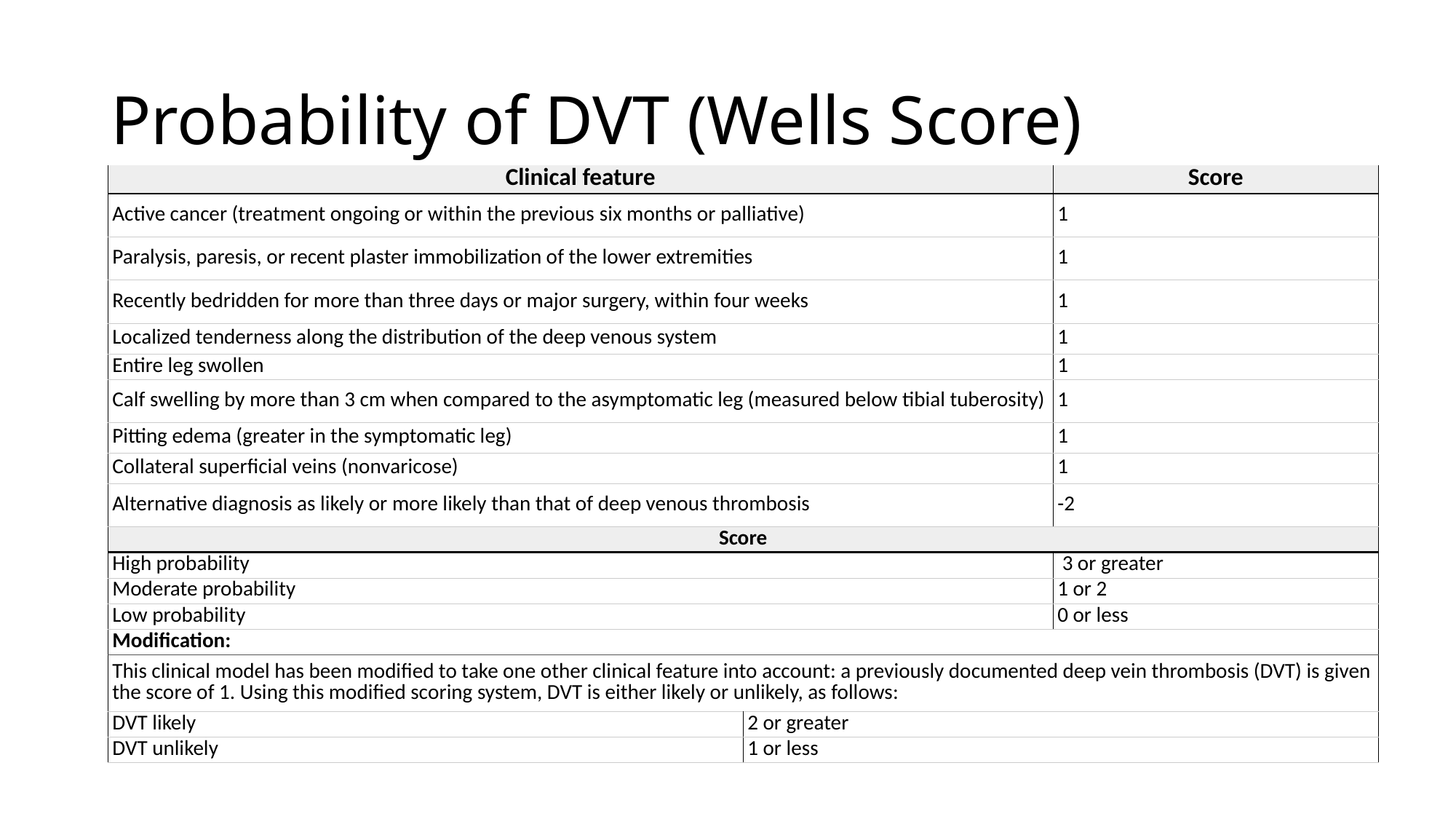

Pretest probability of deep vein thrombosis (Wells score)
# Probability of DVT (Wells Score)
| Clinical feature | Score | Score |
| --- | --- | --- |
| Active cancer (treatment ongoing or within the previous six months or palliative) | 1 | 1 |
| Paralysis, paresis, or recent plaster immobilization of the lower extremities | 1 | 1 |
| Recently bedridden for more than three days or major surgery, within four weeks | 1 | 1 |
| Localized tenderness along the distribution of the deep venous system | 1 | 1 |
| Entire leg swollen | 1 | 1 |
| Calf swelling by more than 3 cm when compared to the asymptomatic leg (measured below tibial tuberosity) | 1 | 1 |
| Pitting edema (greater in the symptomatic leg) | 1 | 1 |
| Collateral superficial veins (nonvaricose) | 1 | 1 |
| Alternative diagnosis as likely or more likely than that of deep venous thrombosis | -2 | -2 |
| Score | | |
| High probability | 3 or greater | 3 or greater |
| Moderate probability | 1 or 2 | 1 or 2 |
| Low probability | 0 or less | 0 or less |
| Modification: | | |
| This clinical model has been modified to take one other clinical feature into account: a previously documented deep vein thrombosis (DVT) is given the score of 1. Using this modified scoring system, DVT is either likely or unlikely, as follows: | | |
| DVT likely | 2 or greater | |
| DVT unlikely | 1 or less | |

## Slide 9
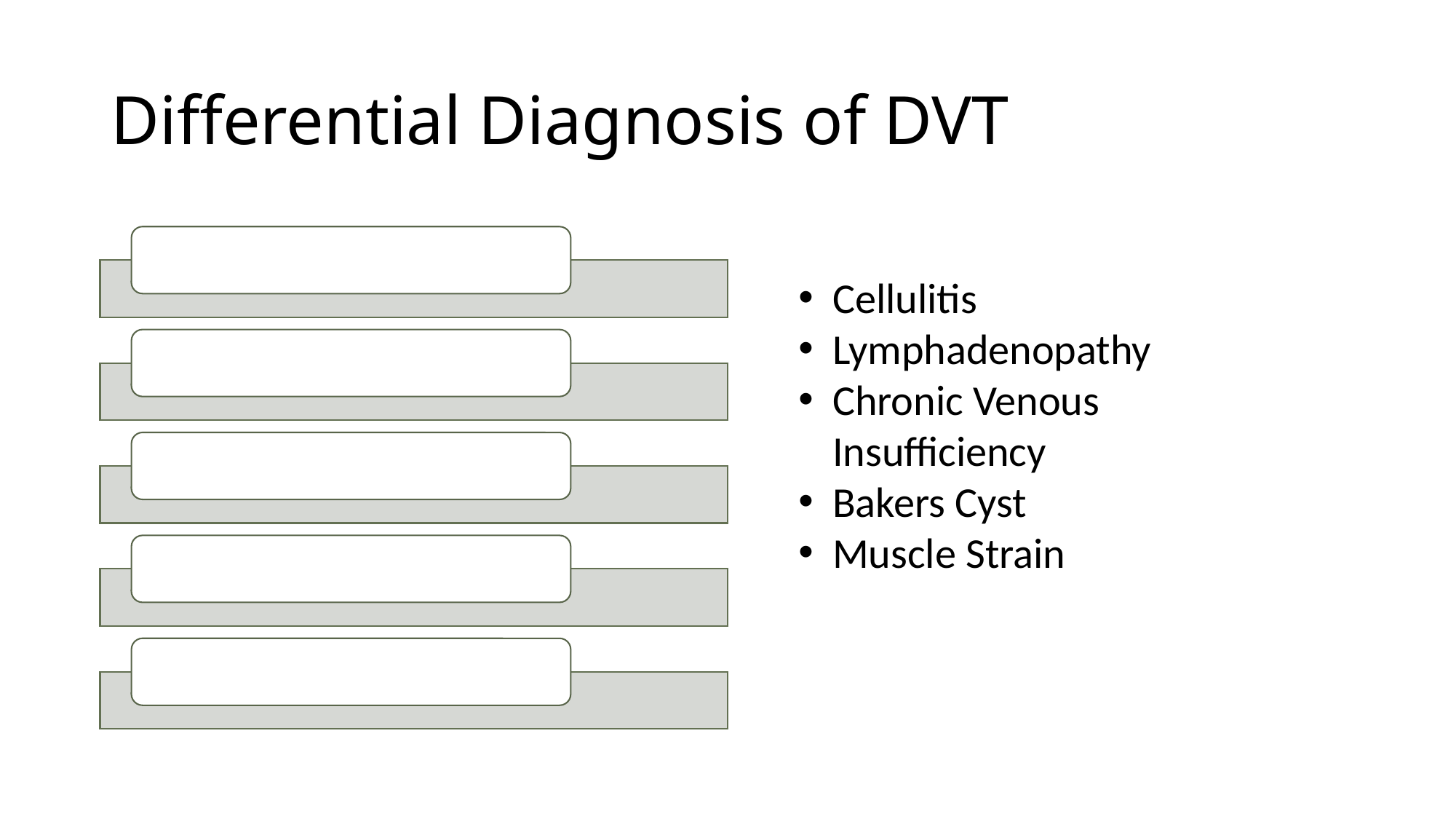

# Differential Diagnosis of DVT
Cellulitis
Lymphadenopathy
Chronic Venous Insufficiency
Bakers Cyst
Muscle Strain

## Slide 10
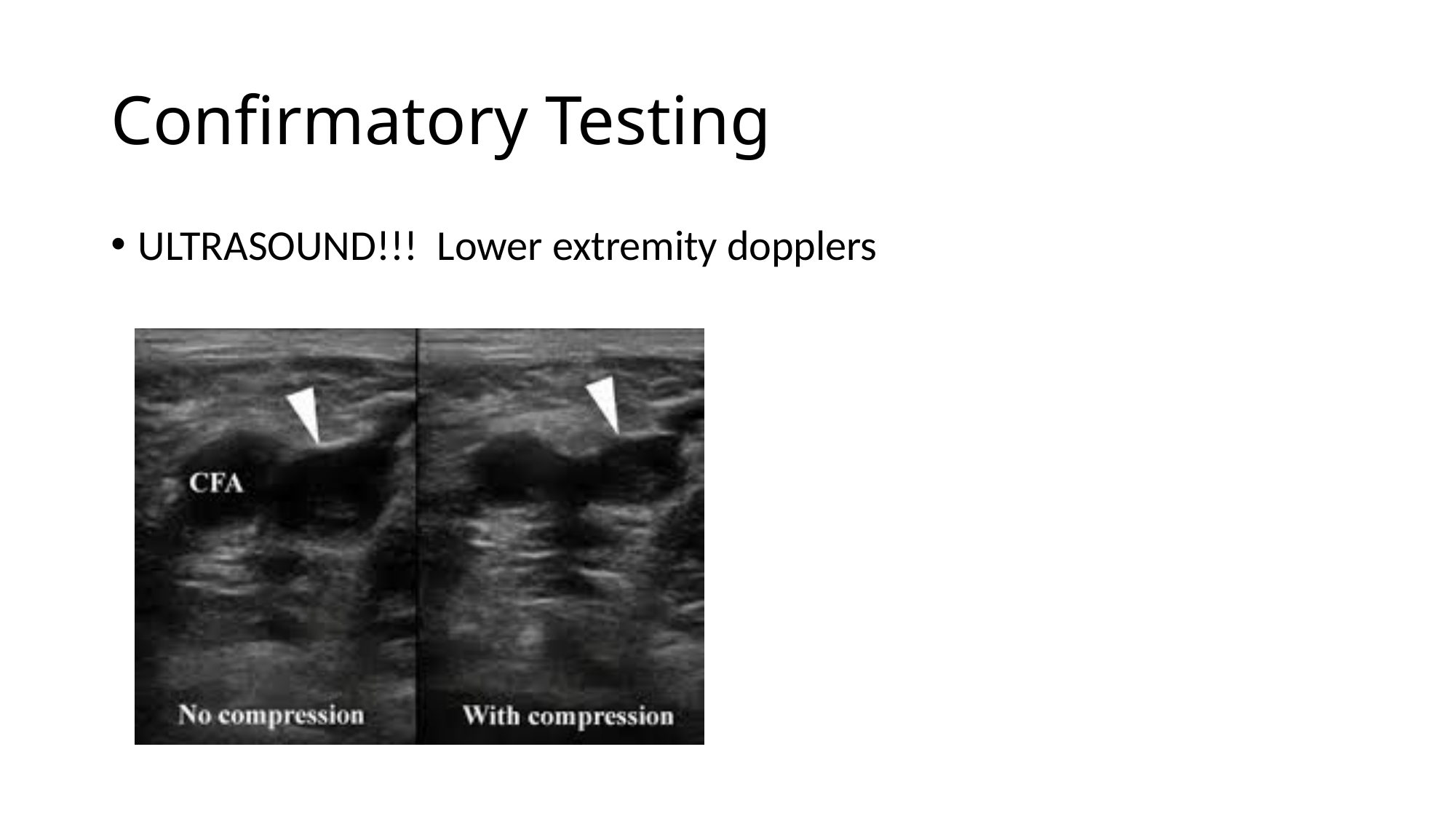

# Confirmatory Testing
ULTRASOUND!!! Lower extremity dopplers

## Slide 11
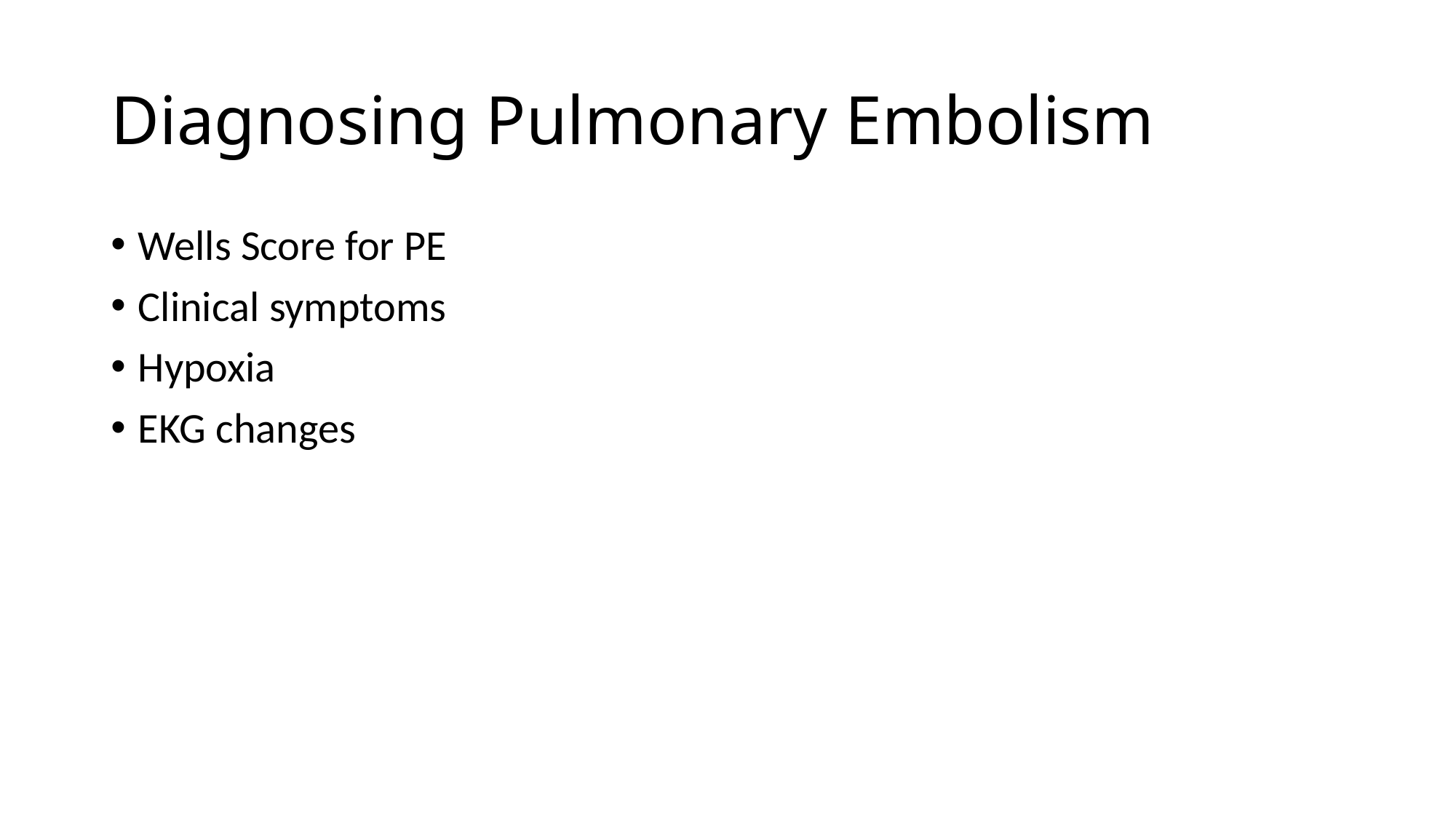

# Diagnosing Pulmonary Embolism
Wells Score for PE
Clinical symptoms
Hypoxia
EKG changes

## Slide 12
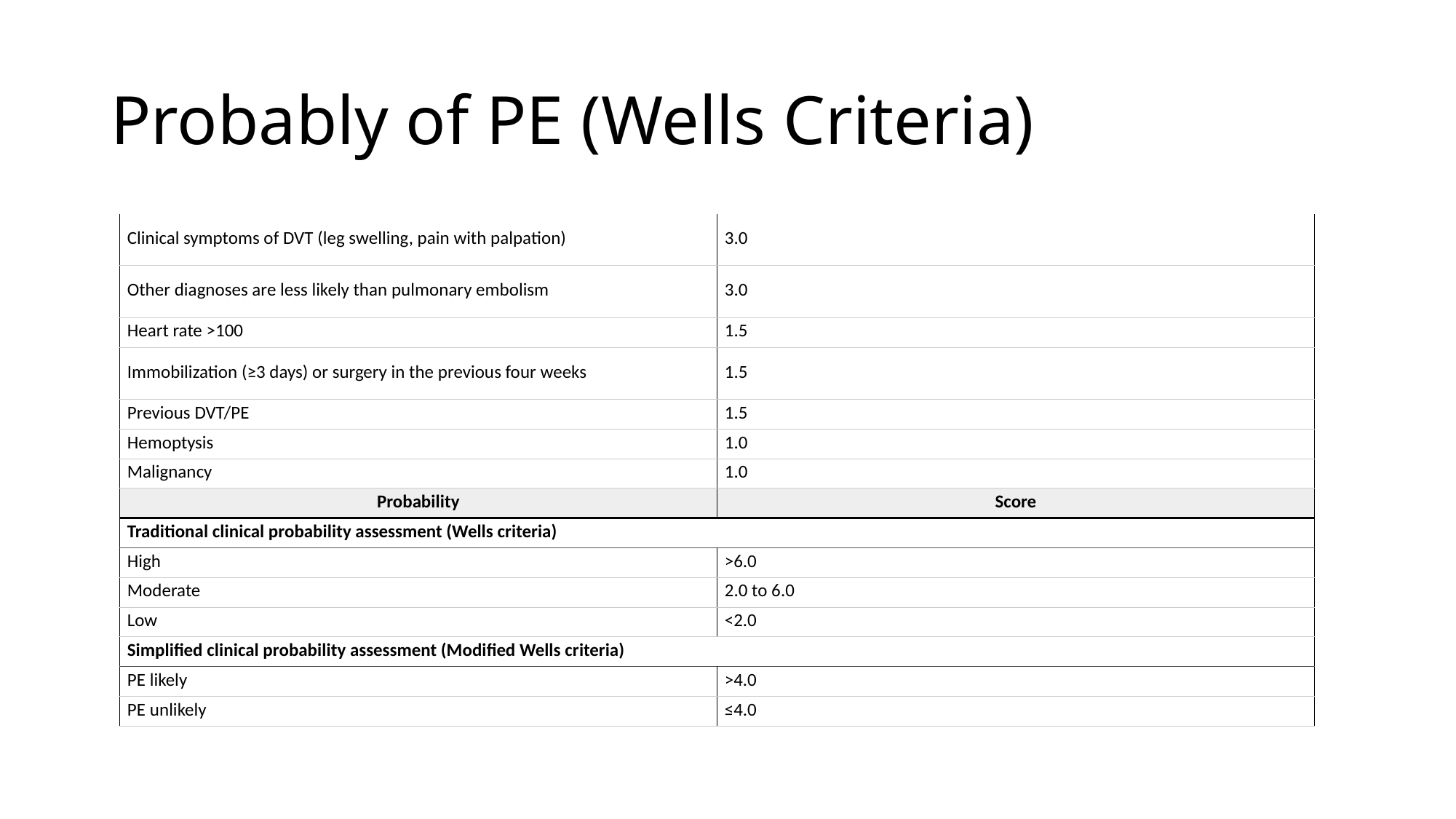

# Probably of PE (Wells Criteria)
| Clinical symptoms of DVT (leg swelling, pain with palpation) | 3.0 |
| --- | --- |
| Other diagnoses are less likely than pulmonary embolism | 3.0 |
| Heart rate >100 | 1.5 |
| Immobilization (≥3 days) or surgery in the previous four weeks | 1.5 |
| Previous DVT/PE | 1.5 |
| Hemoptysis | 1.0 |
| Malignancy | 1.0 |
| Probability | Score |
| Traditional clinical probability assessment (Wells criteria) | |
| High | >6.0 |
| Moderate | 2.0 to 6.0 |
| Low | <2.0 |
| Simplified clinical probability assessment (Modified Wells criteria) | |
| PE likely | >4.0 |
| PE unlikely | ≤4.0 |

## Slide 13
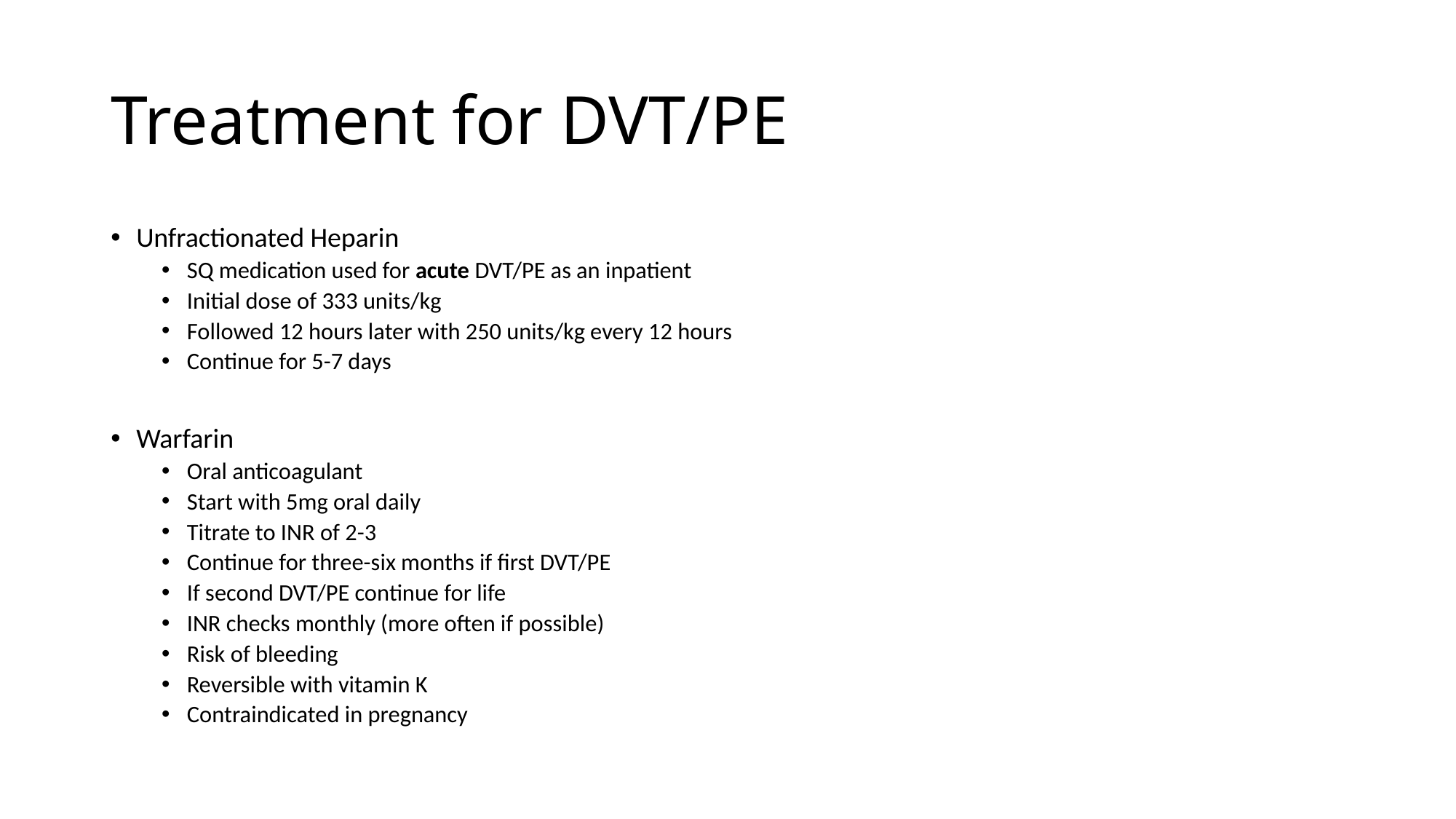

# Treatment for DVT/PE
Unfractionated Heparin
SQ medication used for acute DVT/PE as an inpatient
Initial dose of 333 units/kg
Followed 12 hours later with 250 units/kg every 12 hours
Continue for 5-7 days
Warfarin
Oral anticoagulant
Start with 5mg oral daily
Titrate to INR of 2-3
Continue for three-six months if first DVT/PE
If second DVT/PE continue for life
INR checks monthly (more often if possible)
Risk of bleeding
Reversible with vitamin K
Contraindicated in pregnancy

## Slide 14
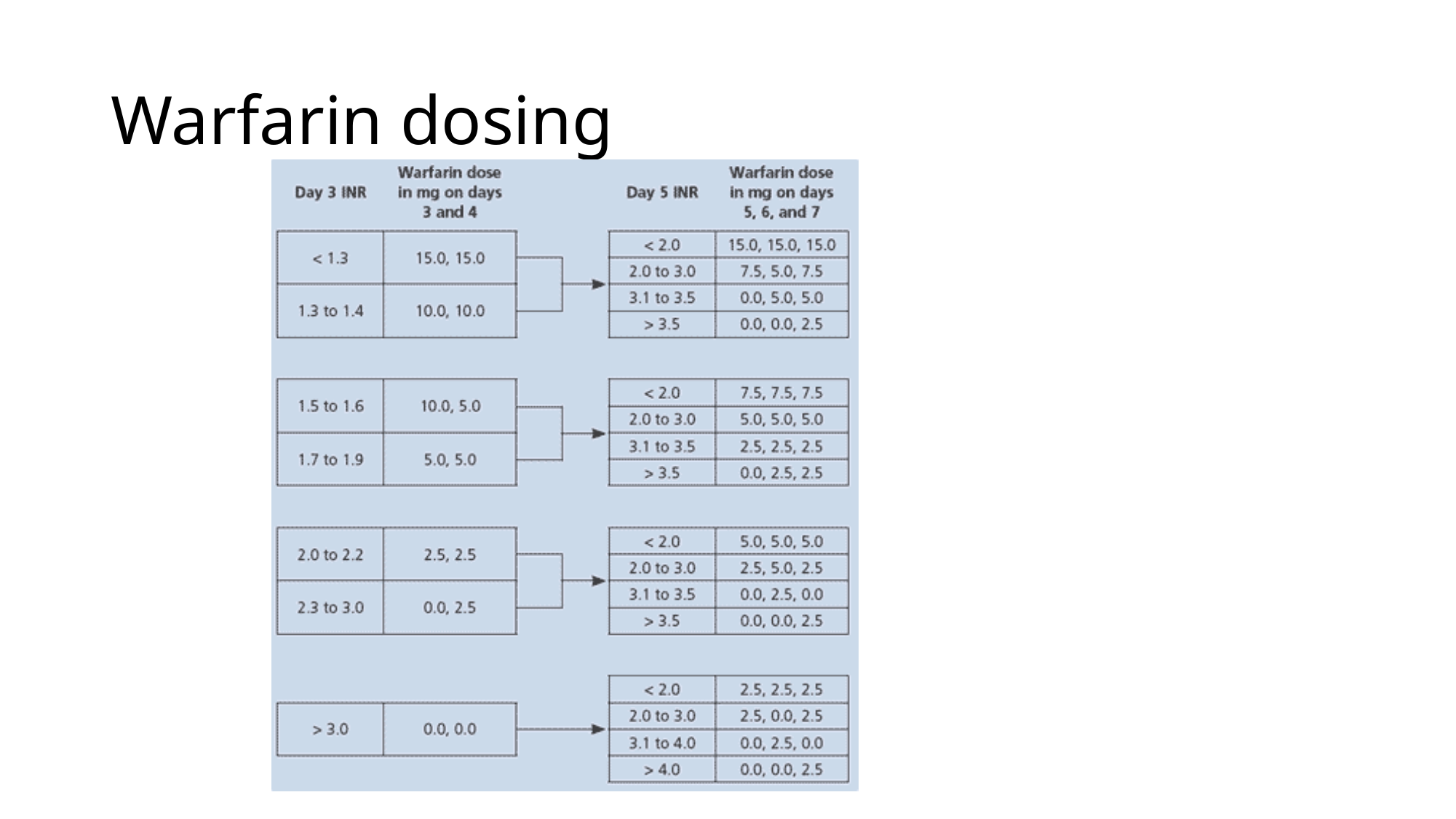

# Warfarin dosing

## Slide 15
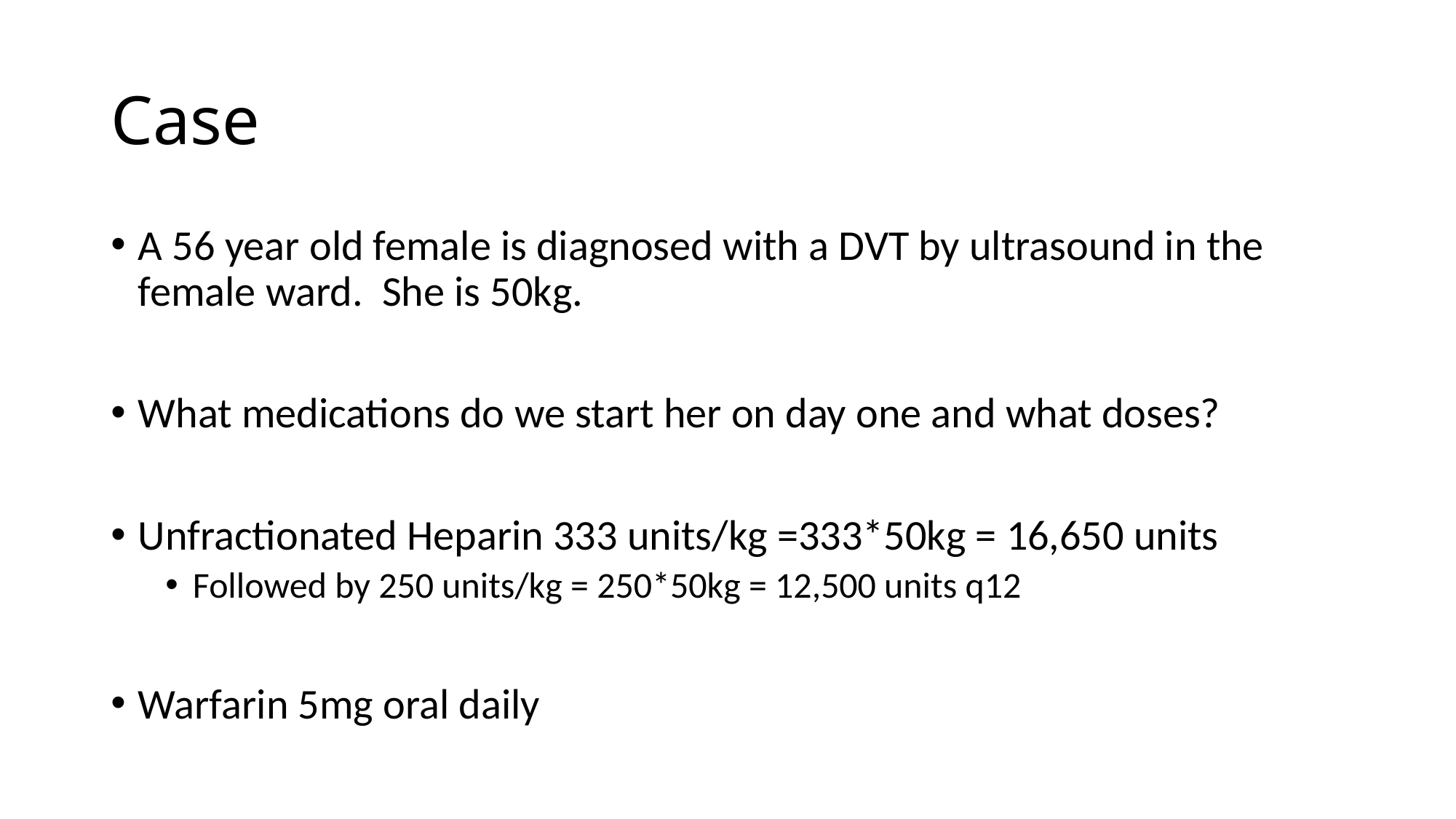

# Case
A 56 year old female is diagnosed with a DVT by ultrasound in the female ward. She is 50kg.
What medications do we start her on day one and what doses?
Unfractionated Heparin 333 units/kg =333*50kg = 16,650 units
Followed by 250 units/kg = 250*50kg = 12,500 units q12
Warfarin 5mg oral daily

## Slide 16
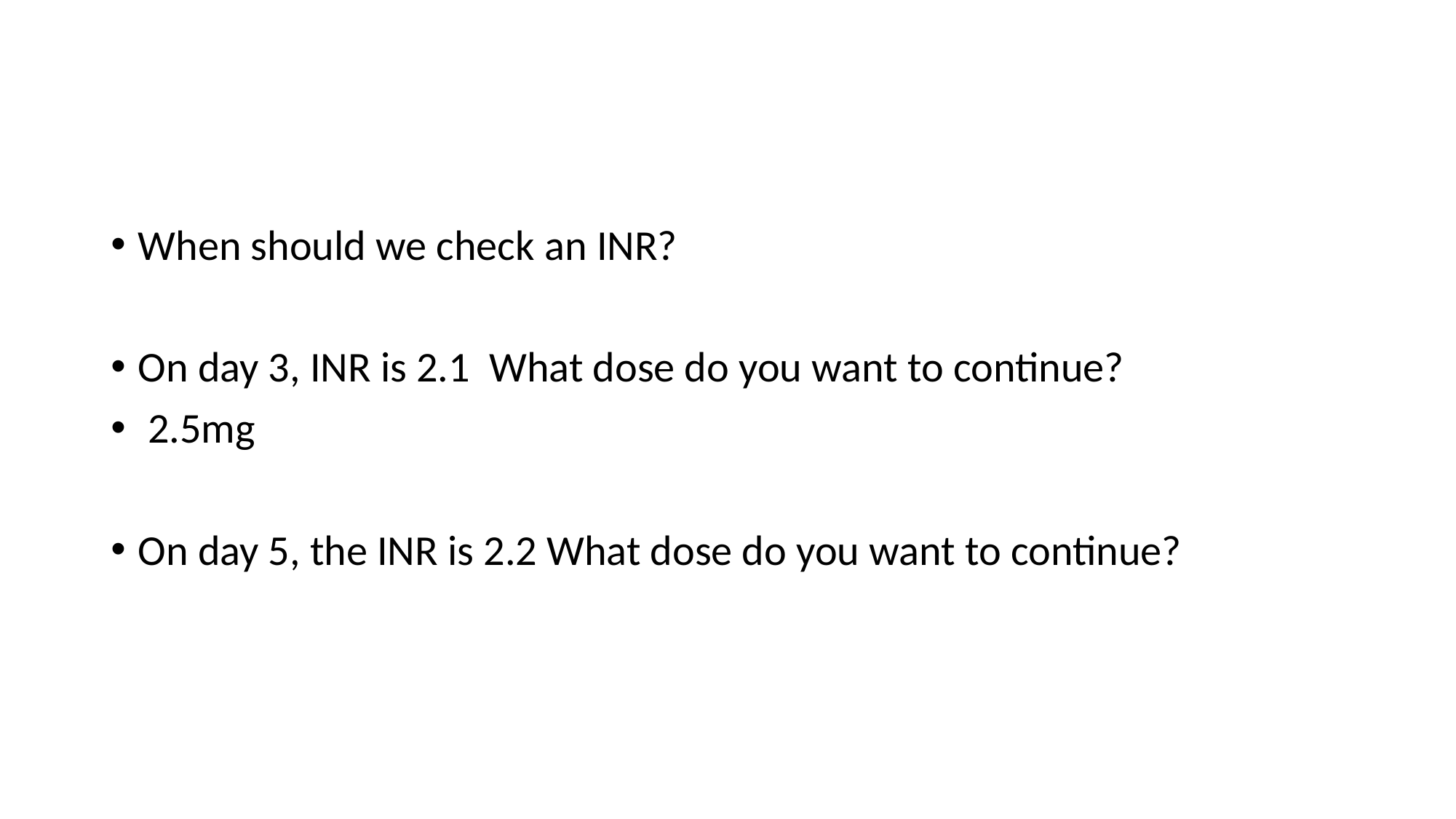

#
When should we check an INR?
On day 3, INR is 2.1 What dose do you want to continue?
 2.5mg
On day 5, the INR is 2.2 What dose do you want to continue?

## Slide 17
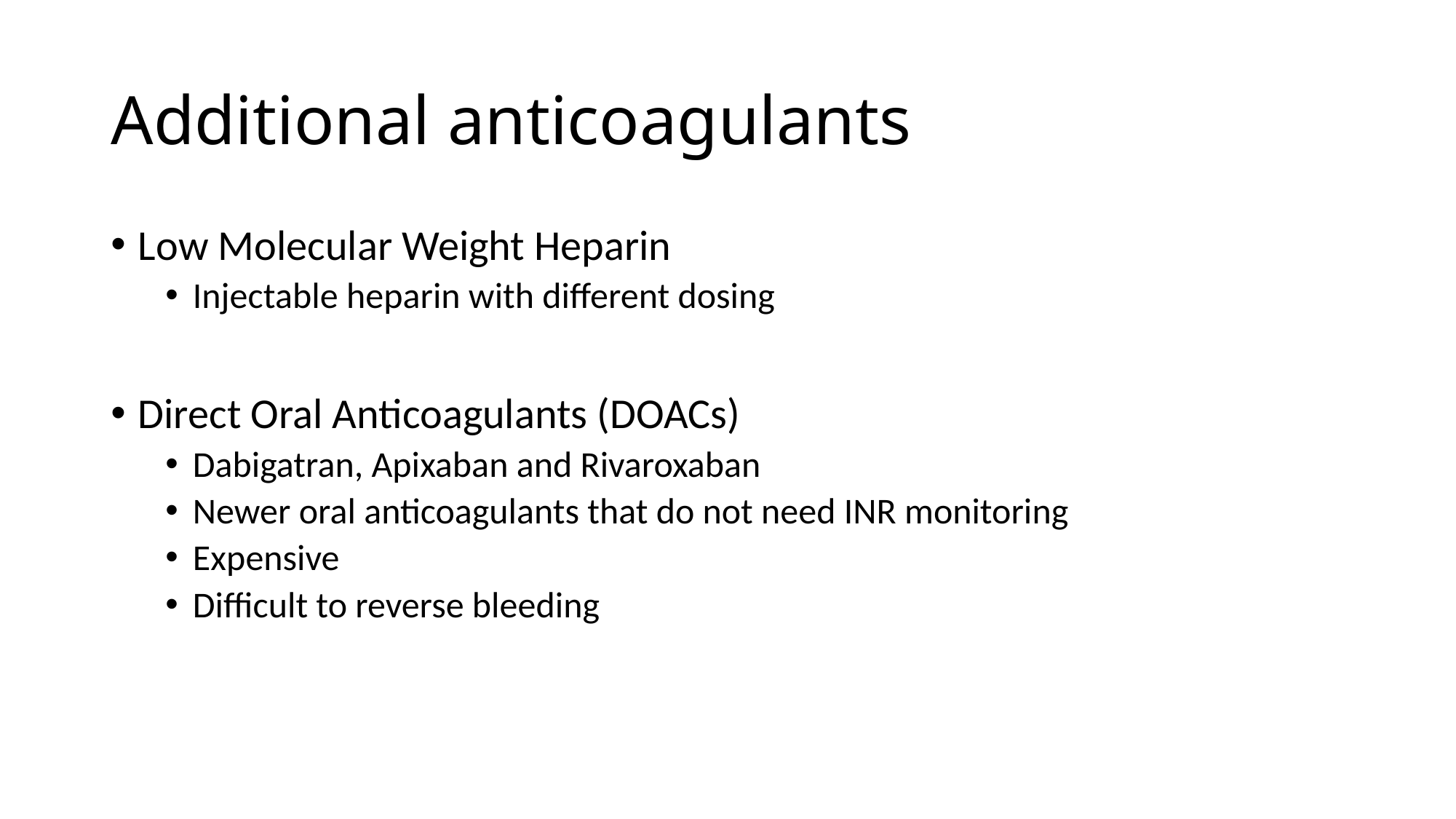

# Additional anticoagulants
Low Molecular Weight Heparin
Injectable heparin with different dosing
Direct Oral Anticoagulants (DOACs)
Dabigatran, Apixaban and Rivaroxaban
Newer oral anticoagulants that do not need INR monitoring
Expensive
Difficult to reverse bleeding

## Slide 18
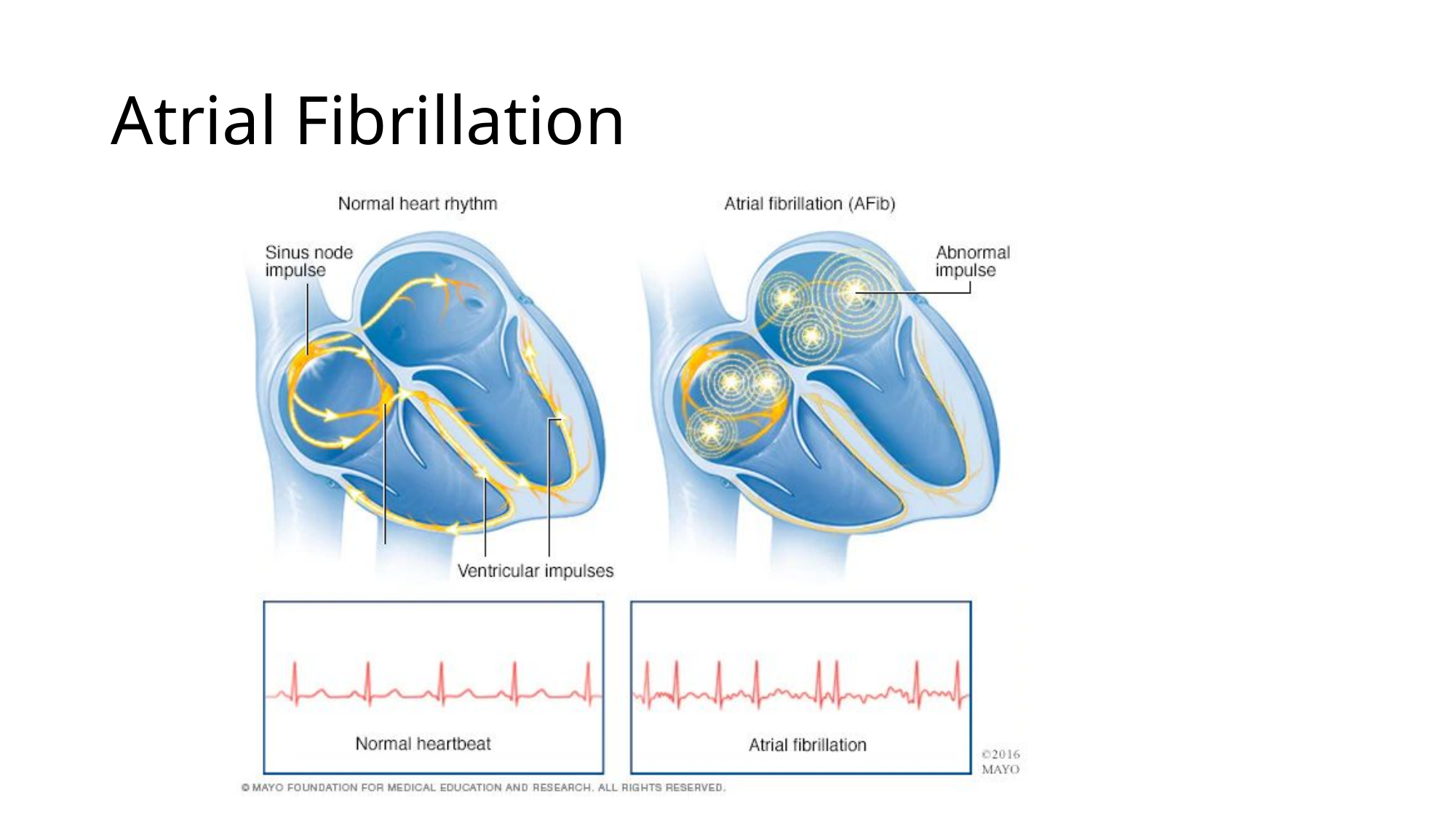

# Atrial Fibrillation

## Slide 19
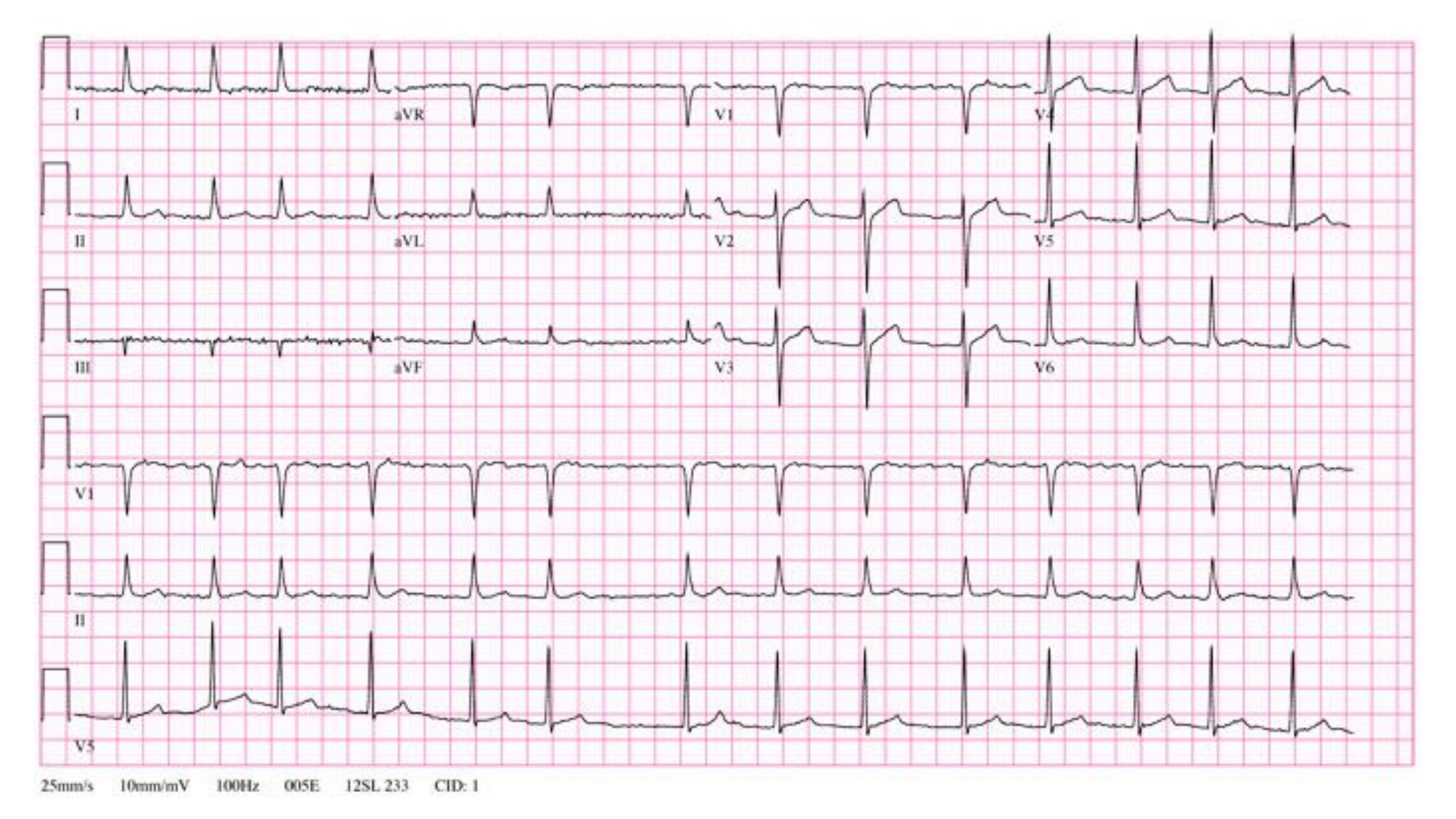

#

## Slide 20
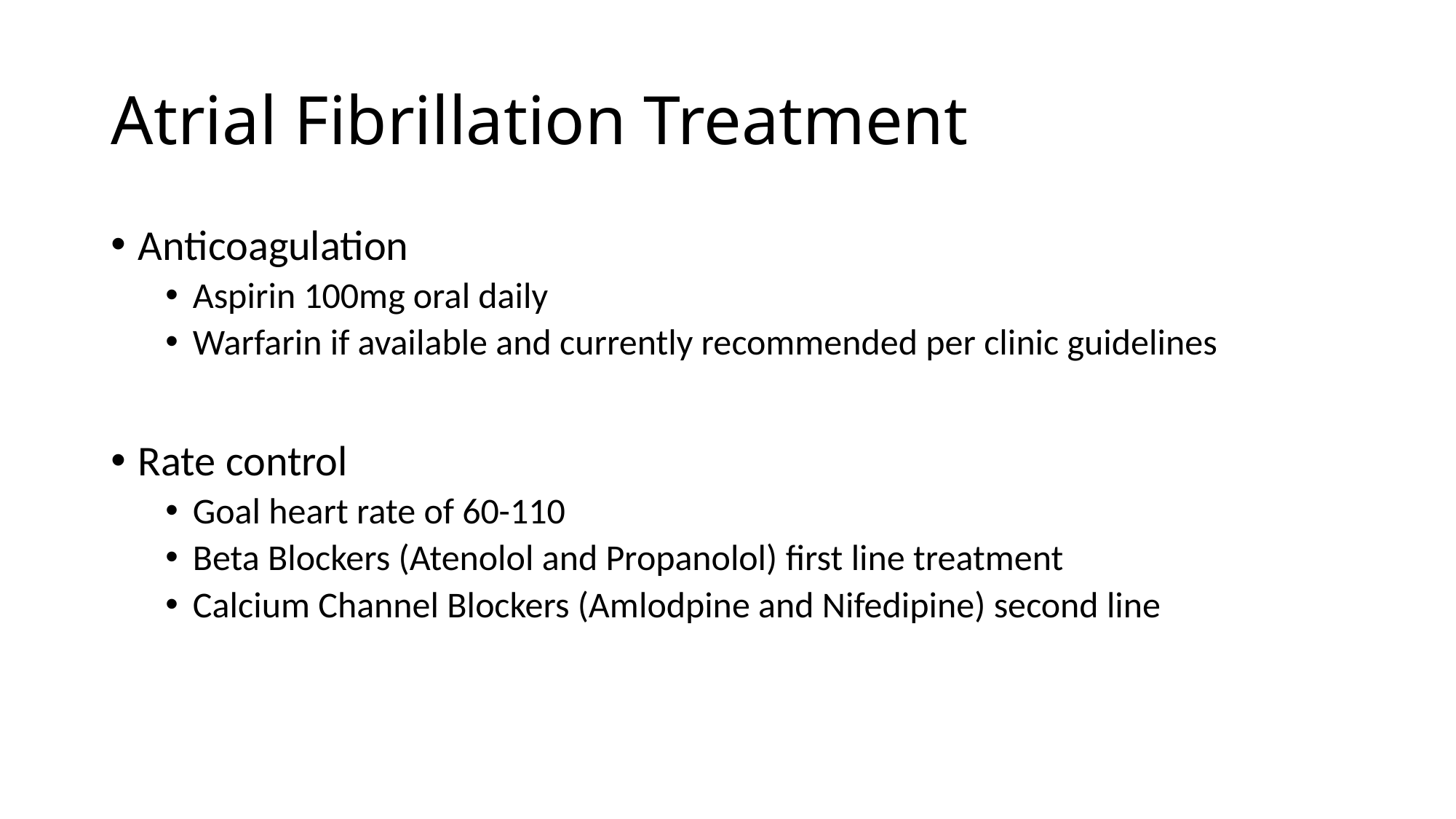

# Atrial Fibrillation Treatment
Anticoagulation
Aspirin 100mg oral daily
Warfarin if available and currently recommended per clinic guidelines
Rate control
Goal heart rate of 60-110
Beta Blockers (Atenolol and Propanolol) first line treatment
Calcium Channel Blockers (Amlodpine and Nifedipine) second line

## Slide 21
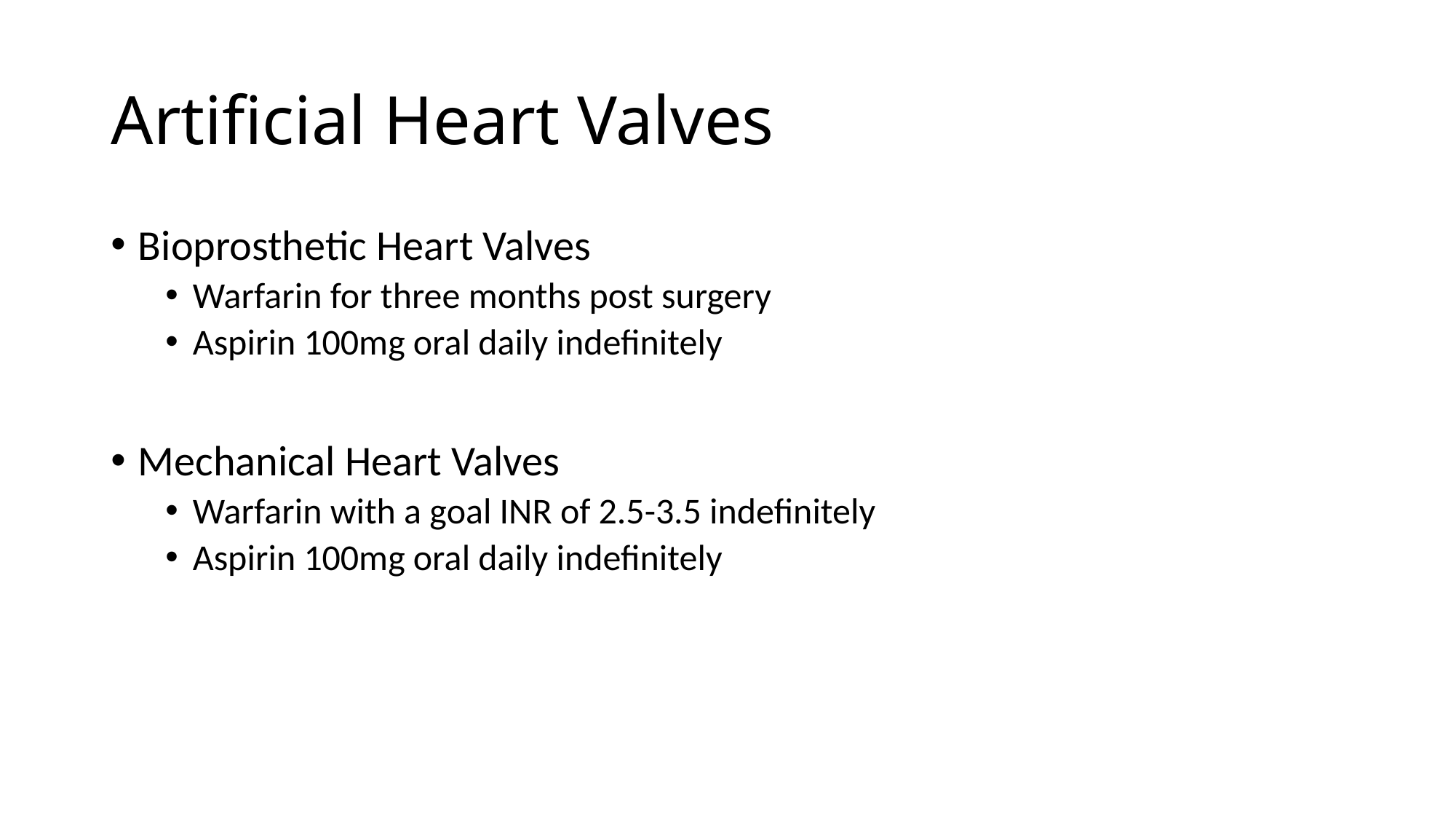

# Artificial Heart Valves
Bioprosthetic Heart Valves
Warfarin for three months post surgery
Aspirin 100mg oral daily indefinitely
Mechanical Heart Valves
Warfarin with a goal INR of 2.5-3.5 indefinitely
Aspirin 100mg oral daily indefinitely

## Slide 22
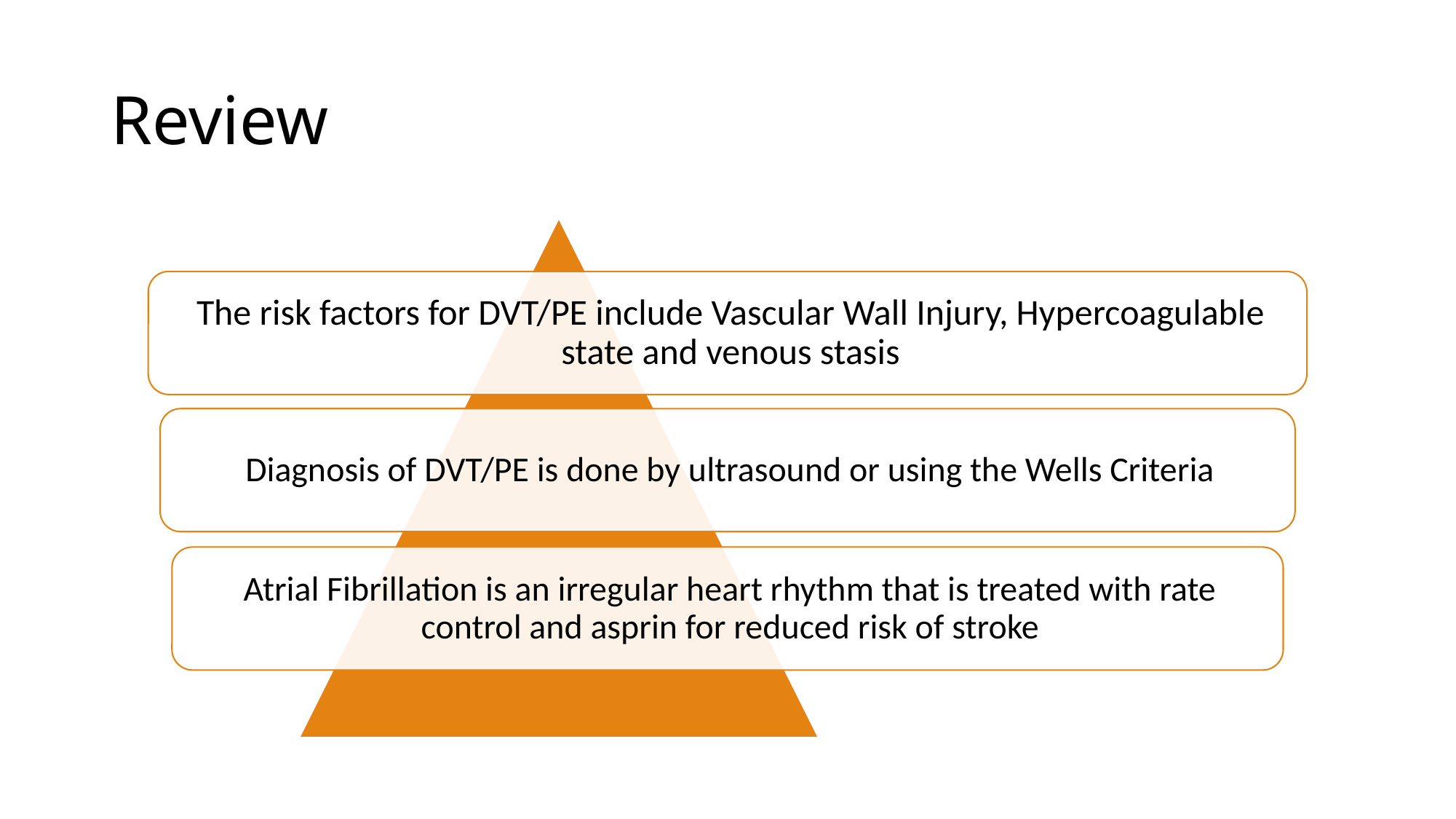

# Review
