# Supplementary material for: Training Mid-Level Providers to Treat Severe Non-Communicable Diseases in Neno, Malawi through PEN-Plus Strategies
Source: Ann Glob Health. 2022 Aug 11;88(1):69. doi: 10.5334/aogh.3750 (PMC9389951; doi:10.5334/aogh.3750)
Supplement: Didactic Materials. — The supplementary materials contain a suggested didactic training schedule and the PowerPoint presentations used for PEN-Plus training in Neno, Malawi. These materials have been reviewed and accepted by the Malawi Ministry of Health for future PEN-Plus trainings in Malawi. [file agh-88-1-3750-s2.zip › Didactic_Materials/CV_Epi and Path.pptx]

## Slide 1
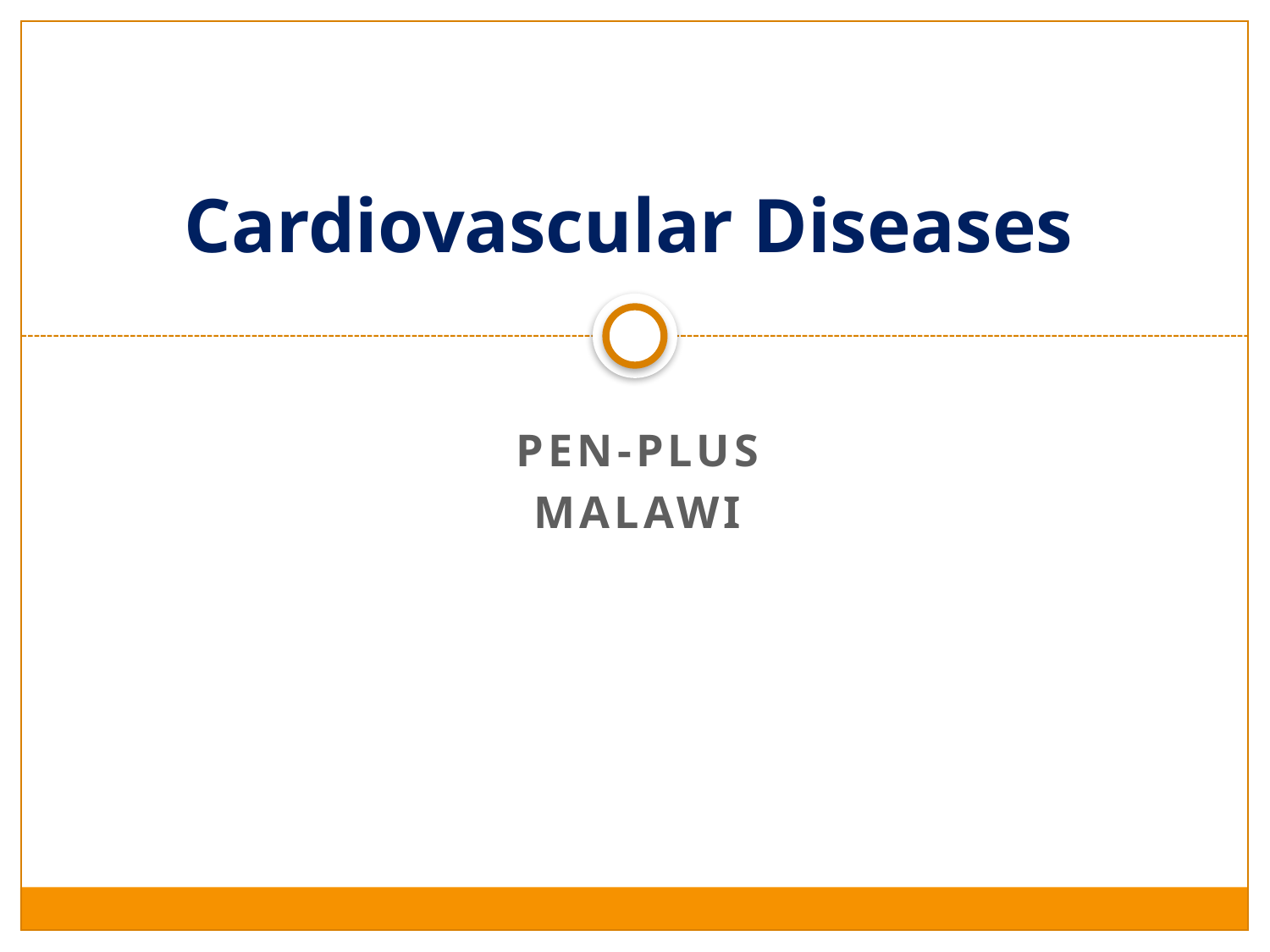

# Cardiovascular Diseases
PeN-Plus
Malawi

## Slide 2
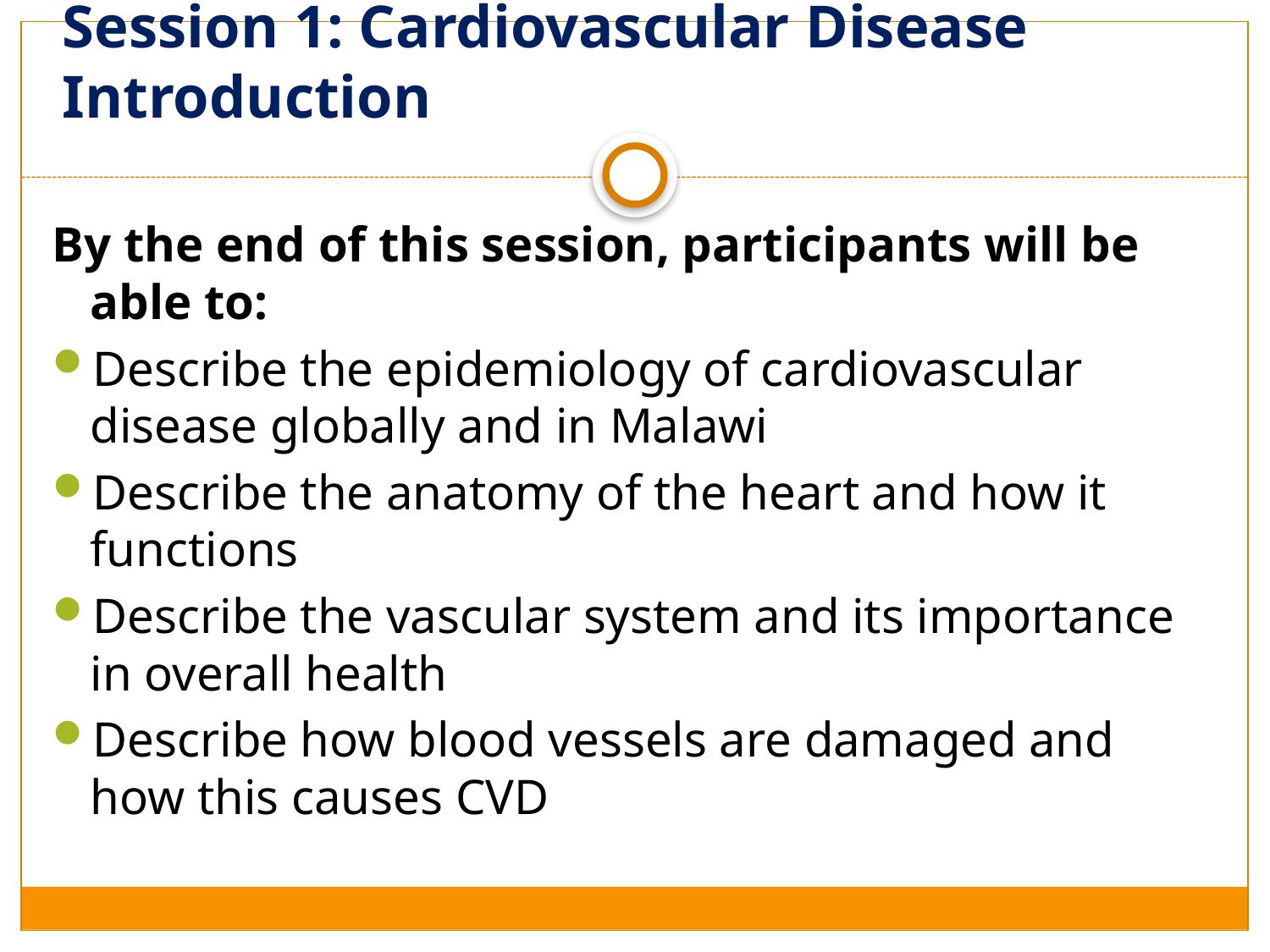

# Session 1: Cardiovascular Disease Introduction
By the end of this session, participants will be able to:
Describe the epidemiology of cardiovascular disease globally and in Malawi
Describe the anatomy of the heart and how it functions
Describe the vascular system and its importance in overall health
Describe how blood vessels are damaged and how this causes CVD

## Slide 3
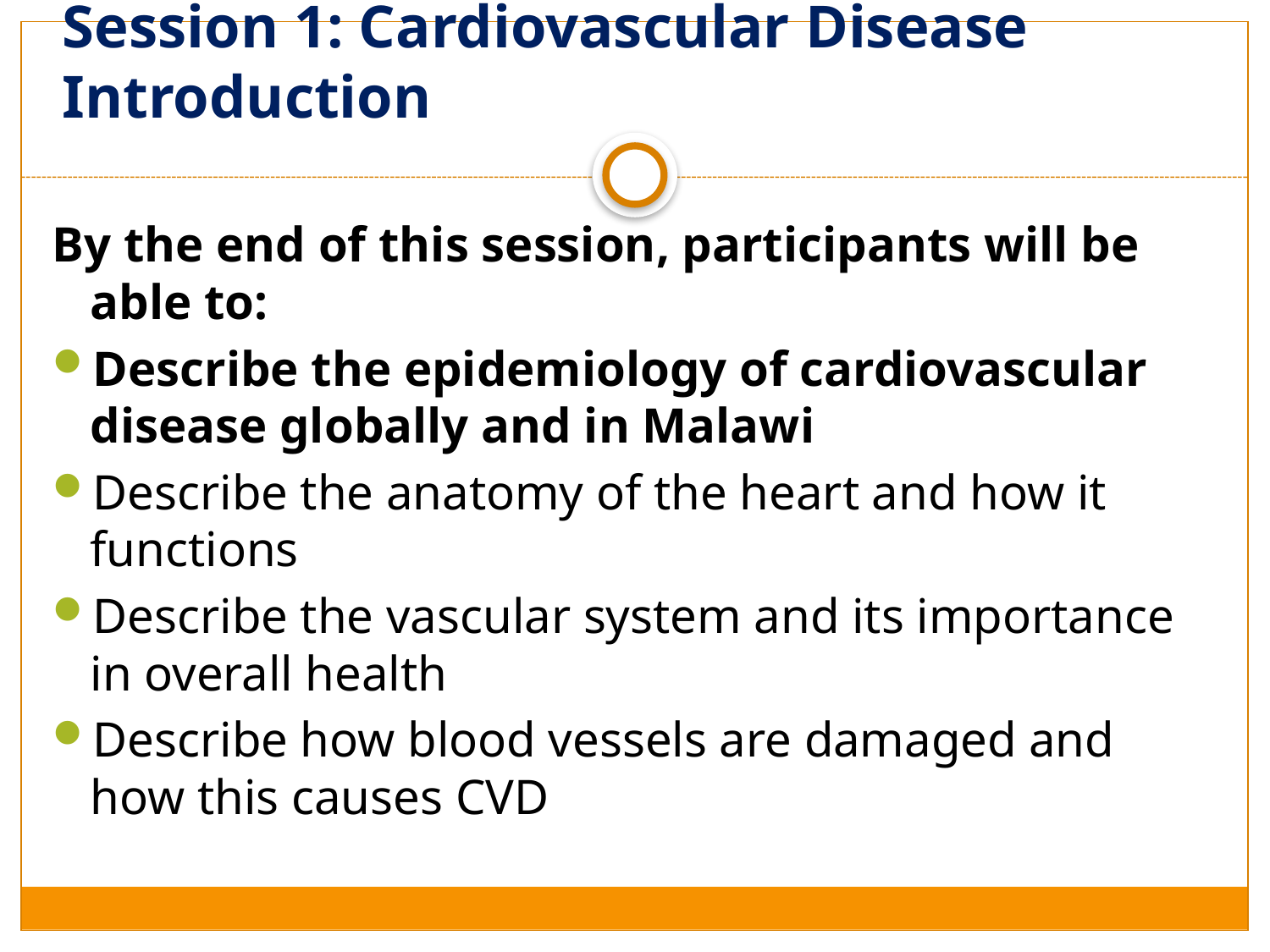

# Session 1: Cardiovascular Disease Introduction
By the end of this session, participants will be able to:
Describe the epidemiology of cardiovascular disease globally and in Malawi
Describe the anatomy of the heart and how it functions
Describe the vascular system and its importance in overall health
Describe how blood vessels are damaged and how this causes CVD

## Slide 4
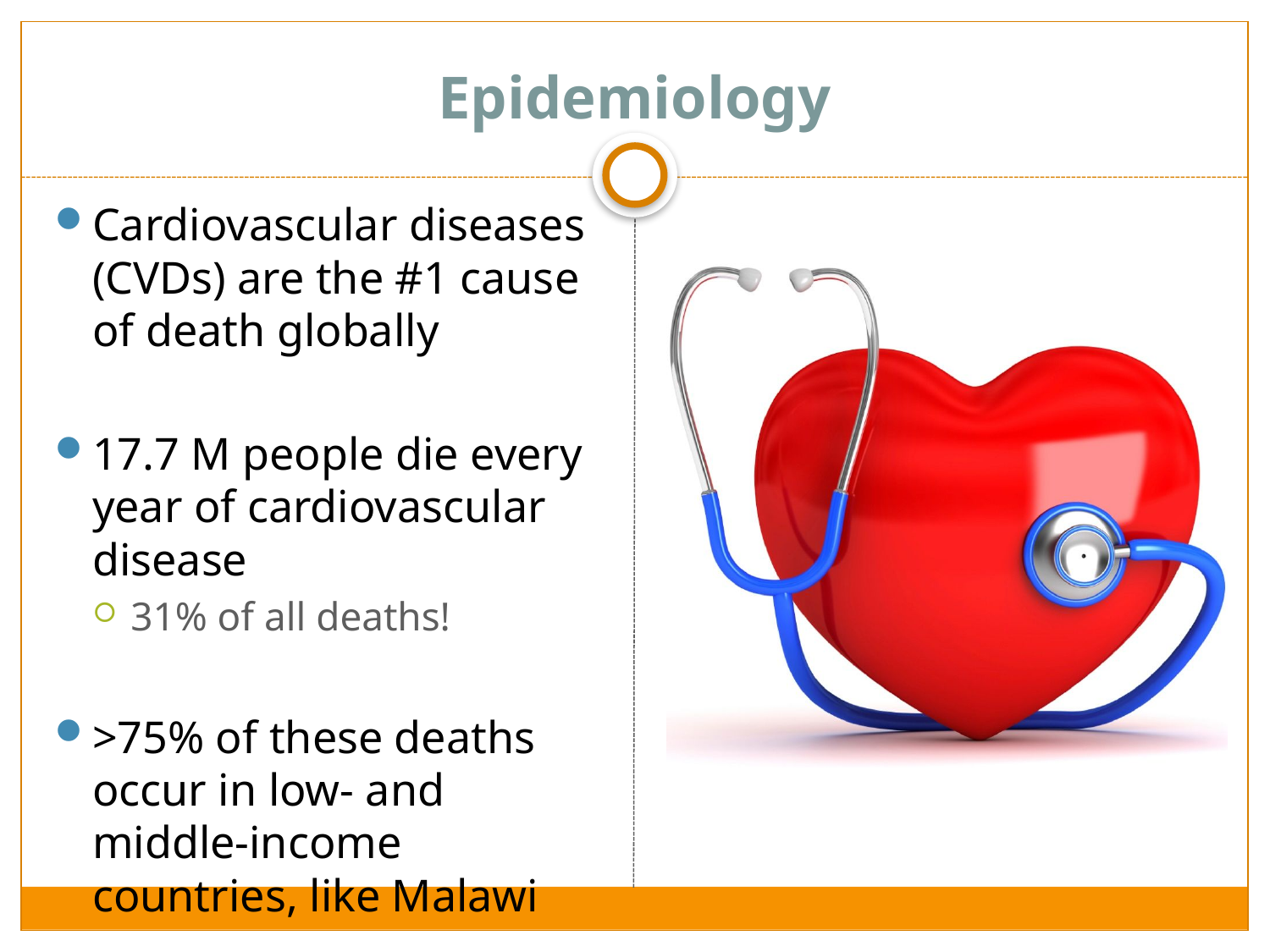

# Epidemiology
Cardiovascular diseases (CVDs) are the #1 cause of death globally
17.7 M people die every year of cardiovascular disease
31% of all deaths!
>75% of these deaths occur in low- and middle-income countries, like Malawi

## Slide 5
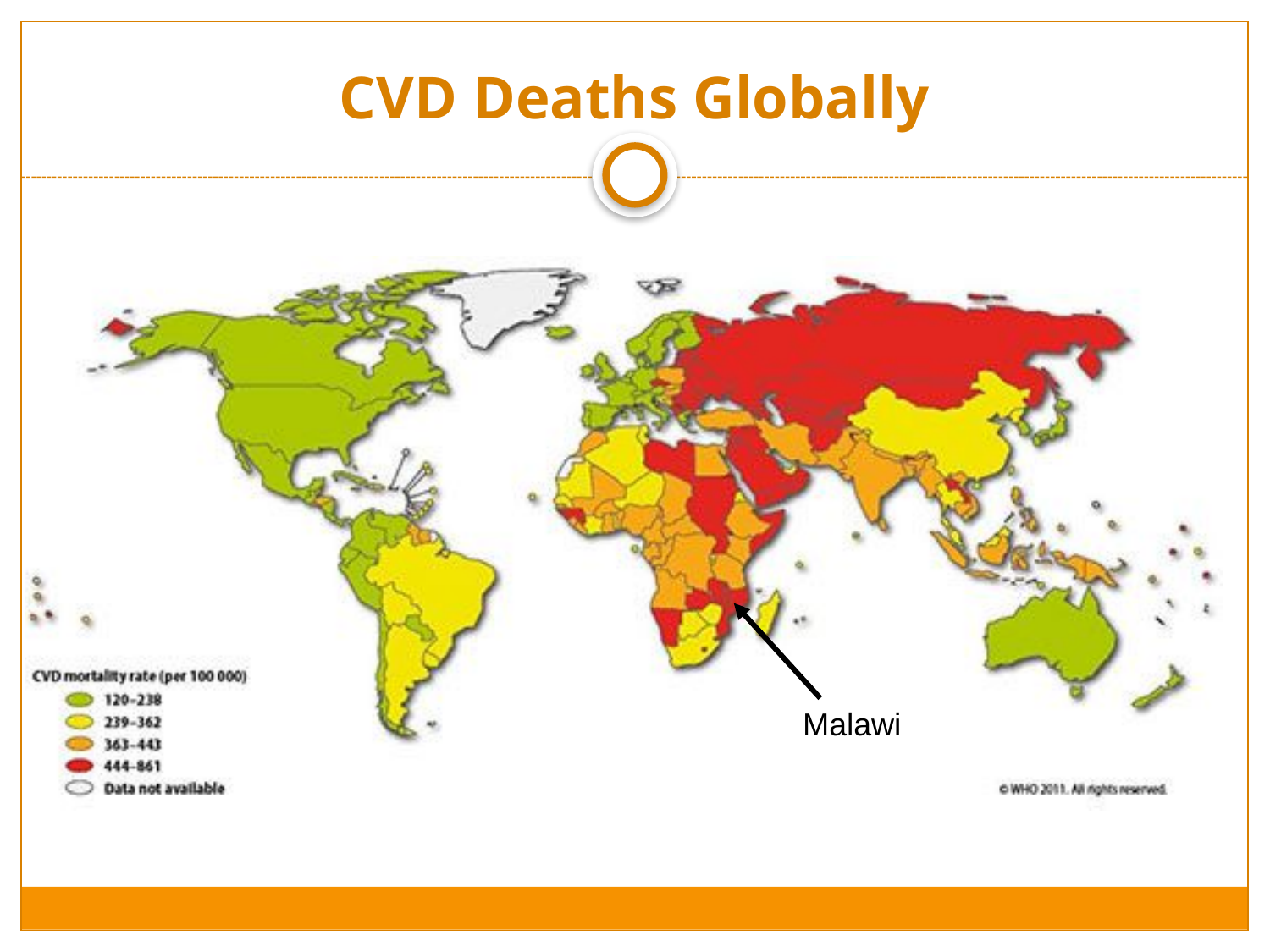

# CVD Deaths Globally
Malawi

## Slide 6
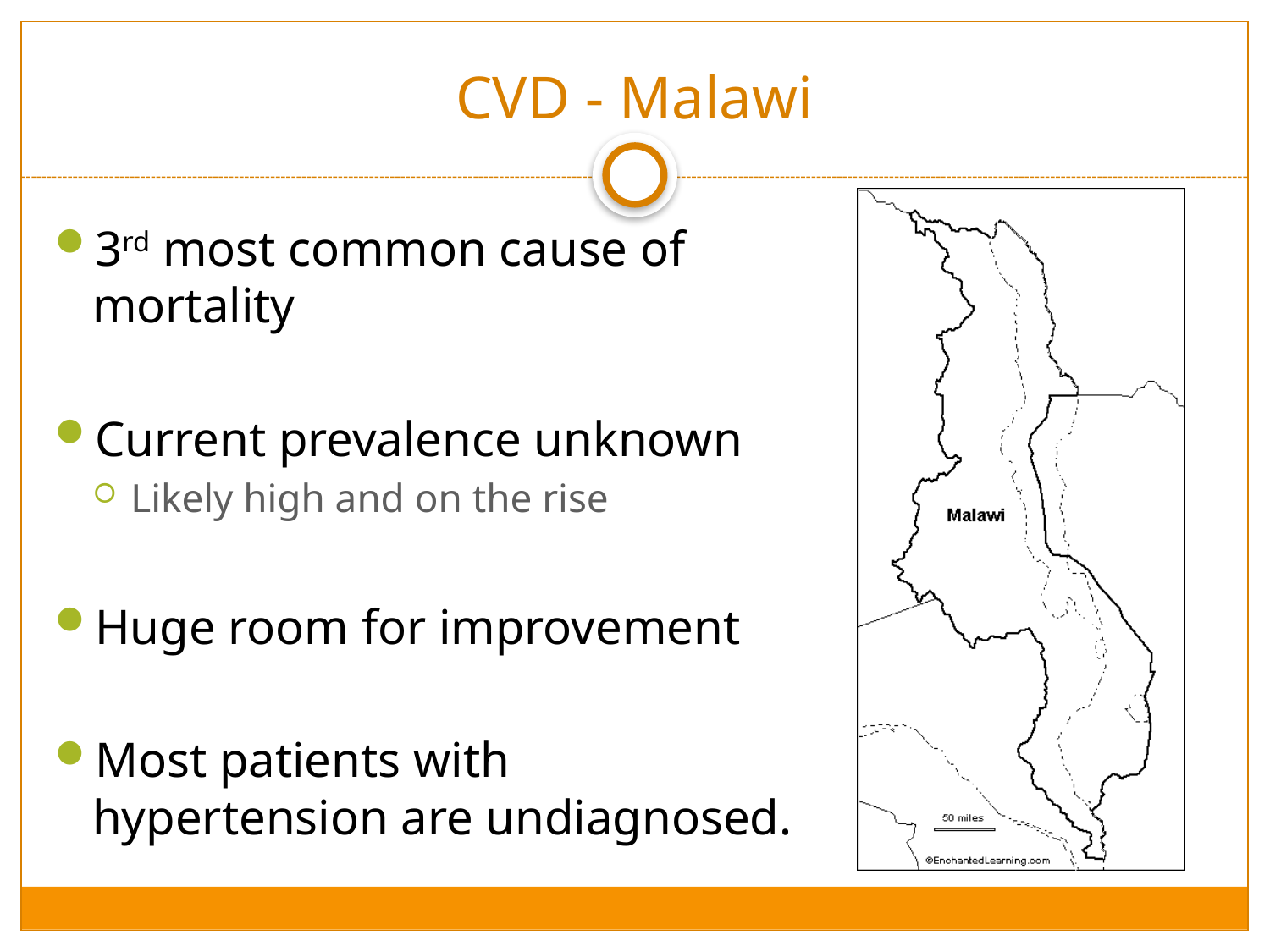

# CVD - Malawi
3rd most common cause of mortality
Current prevalence unknown
Likely high and on the rise
Huge room for improvement
Most patients with hypertension are undiagnosed.

## Slide 7
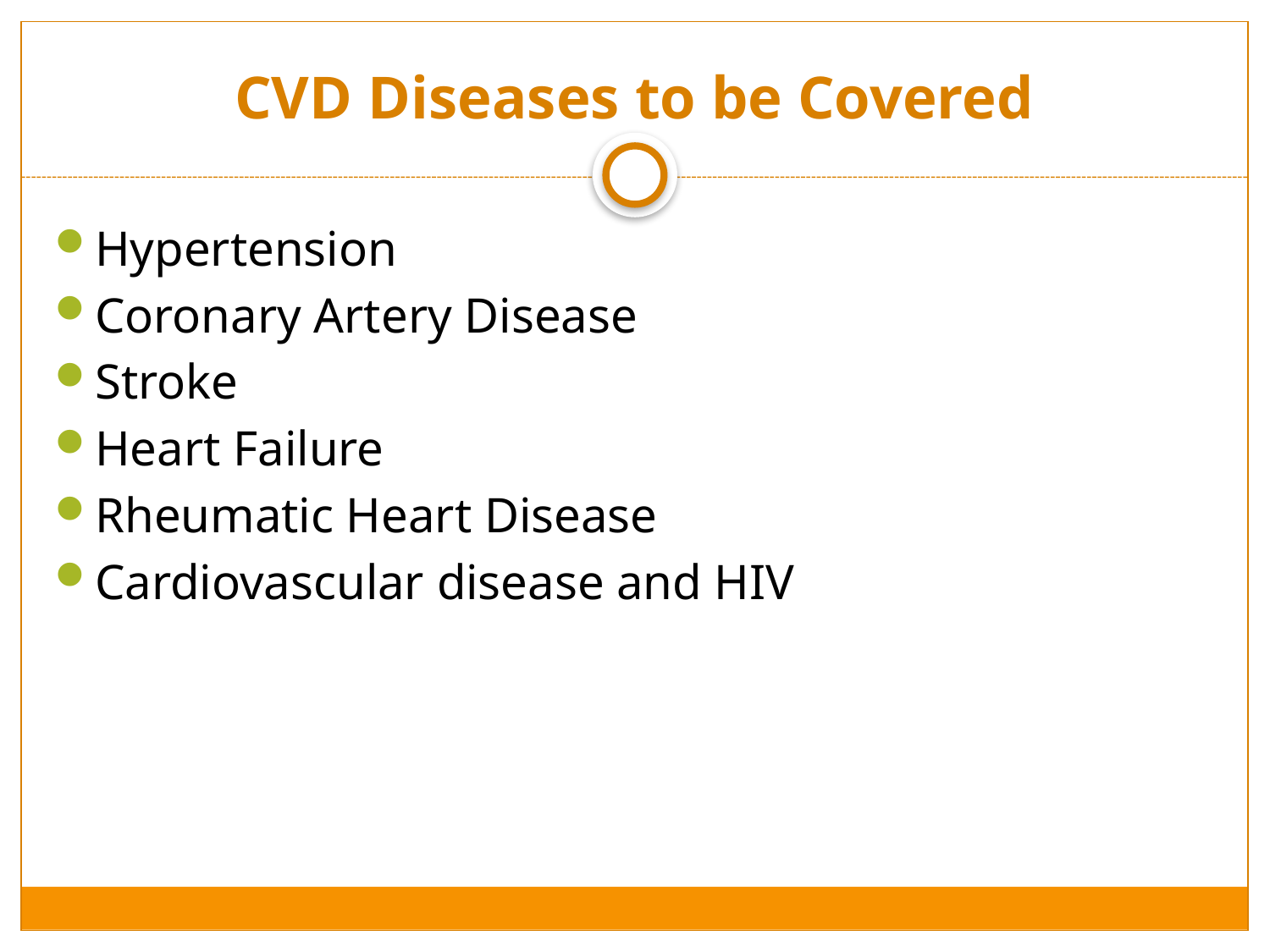

# CVD Diseases to be Covered
Hypertension
Coronary Artery Disease
Stroke
Heart Failure
Rheumatic Heart Disease
Cardiovascular disease and HIV

## Slide 8
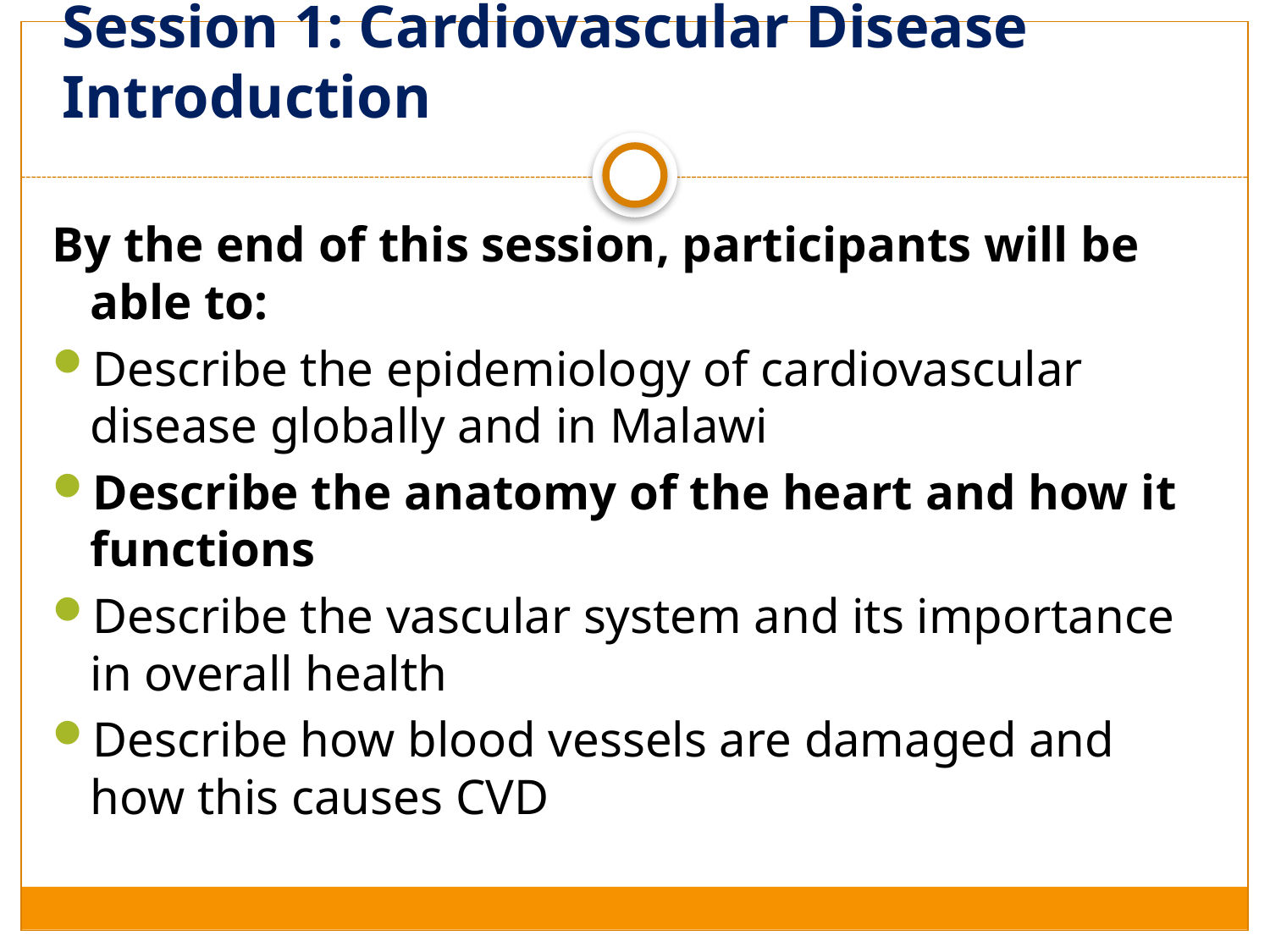

# Session 1: Cardiovascular Disease Introduction
By the end of this session, participants will be able to:
Describe the epidemiology of cardiovascular disease globally and in Malawi
Describe the anatomy of the heart and how it functions
Describe the vascular system and its importance in overall health
Describe how blood vessels are damaged and how this causes CVD

## Slide 9
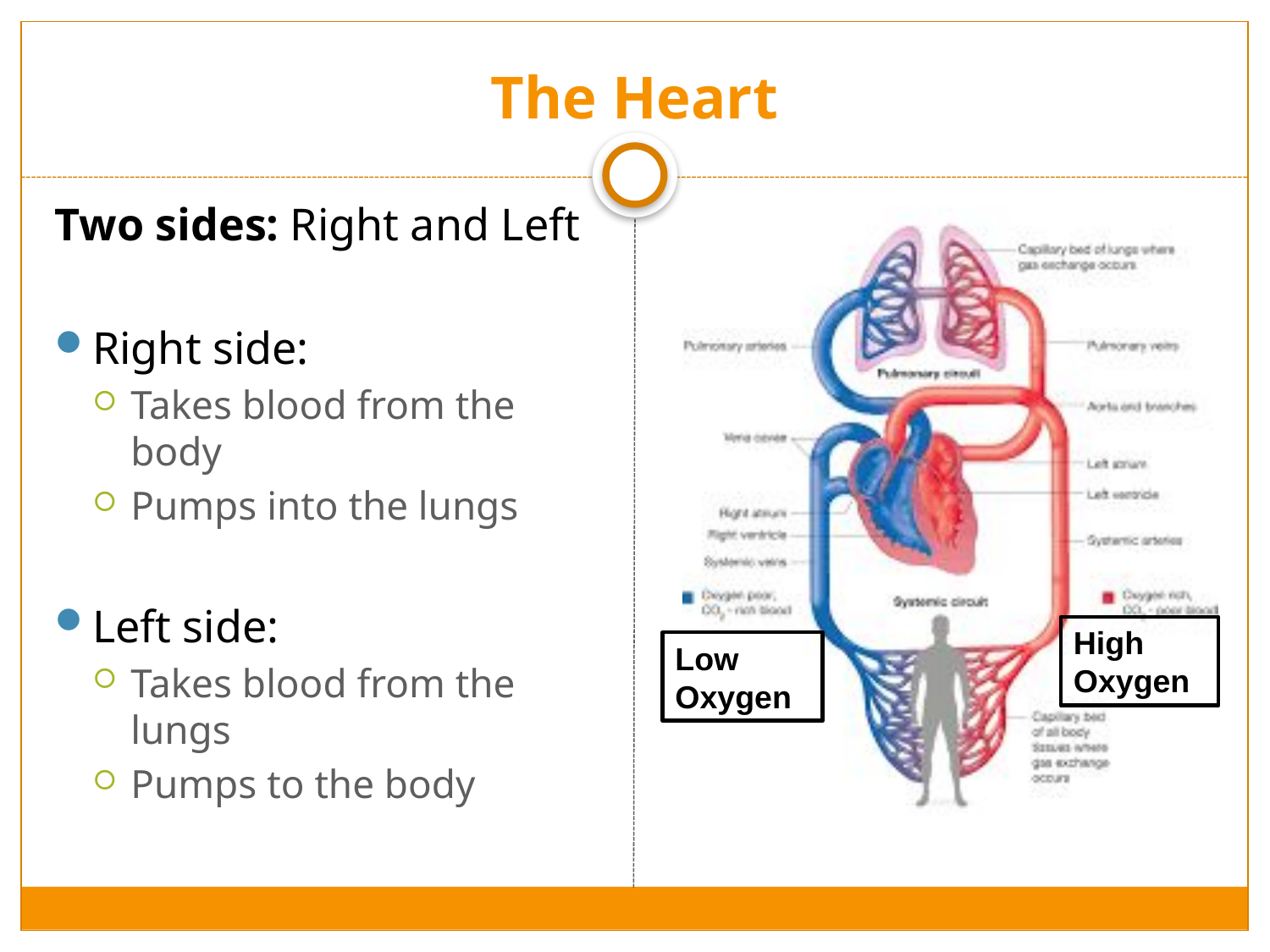

# The Heart
Two sides: Right and Left
Right side:
Takes blood from the body
Pumps into the lungs
Left side:
Takes blood from the lungs
Pumps to the body
High Oxygen
Low Oxygen

## Slide 10
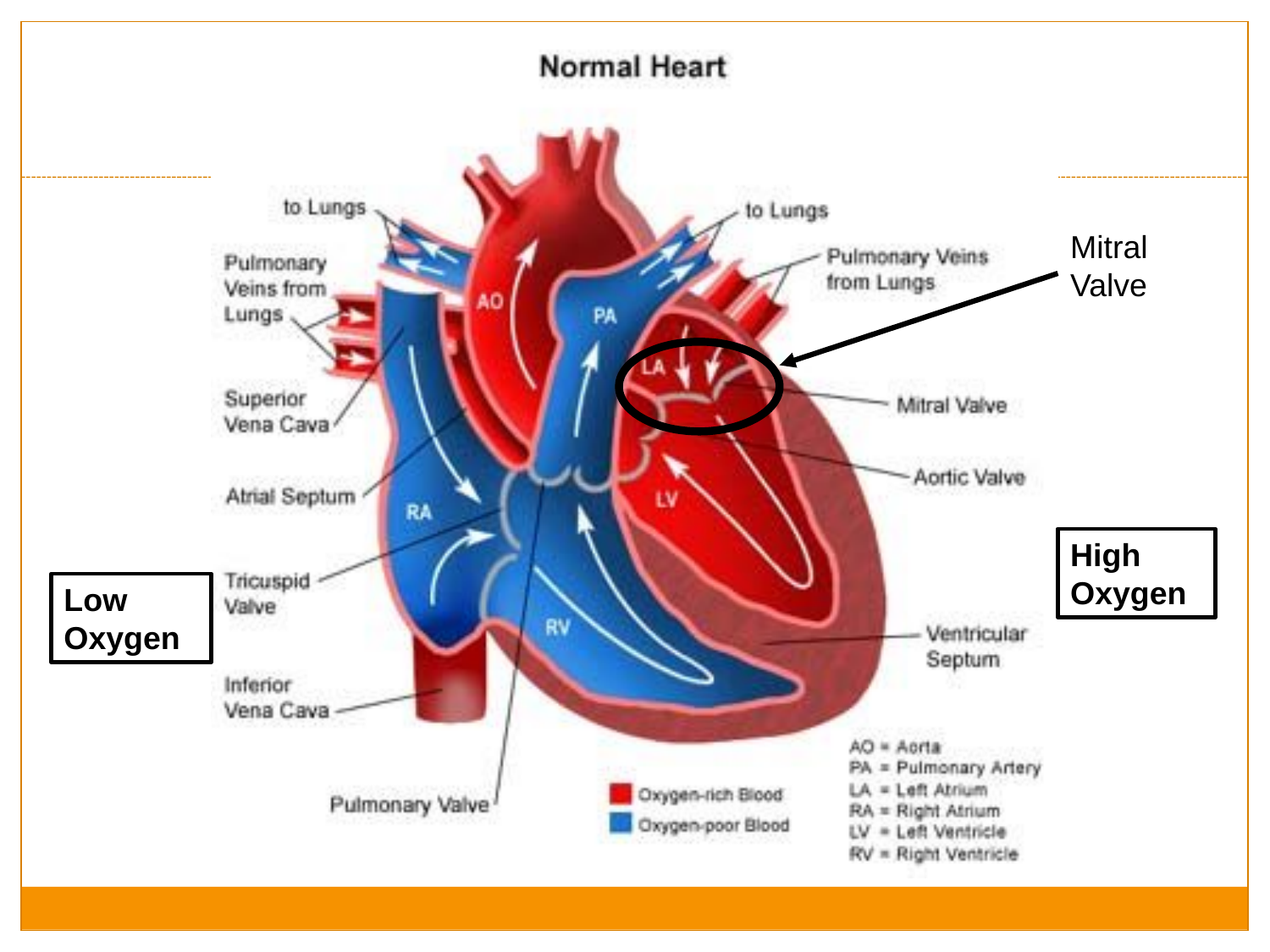

# The Heart
Mitral Valve
High Oxygen
Low Oxygen

## Slide 11
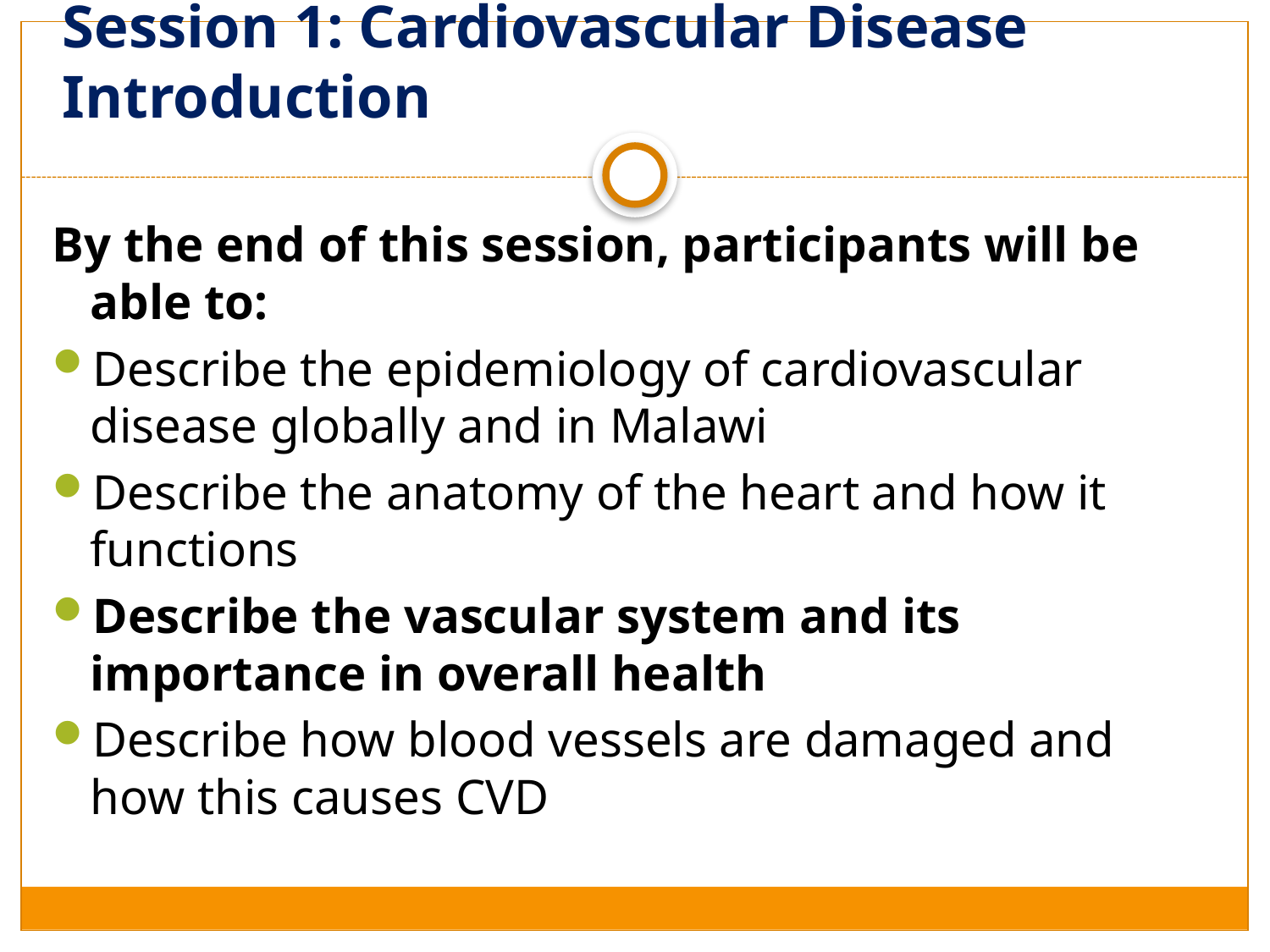

# Session 1: Cardiovascular Disease Introduction
By the end of this session, participants will be able to:
Describe the epidemiology of cardiovascular disease globally and in Malawi
Describe the anatomy of the heart and how it functions
Describe the vascular system and its importance in overall health
Describe how blood vessels are damaged and how this causes CVD

## Slide 12
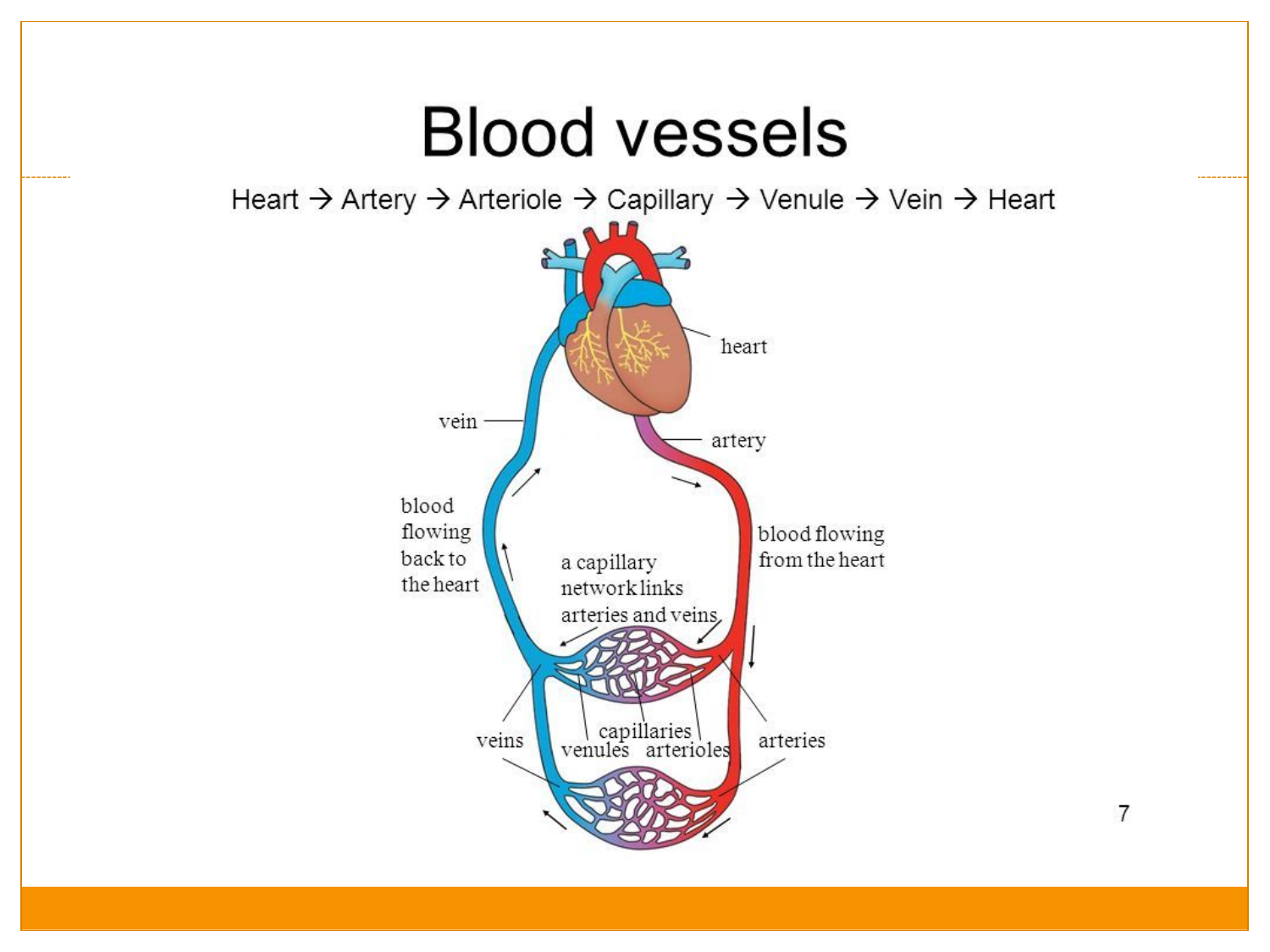

## Slide 13
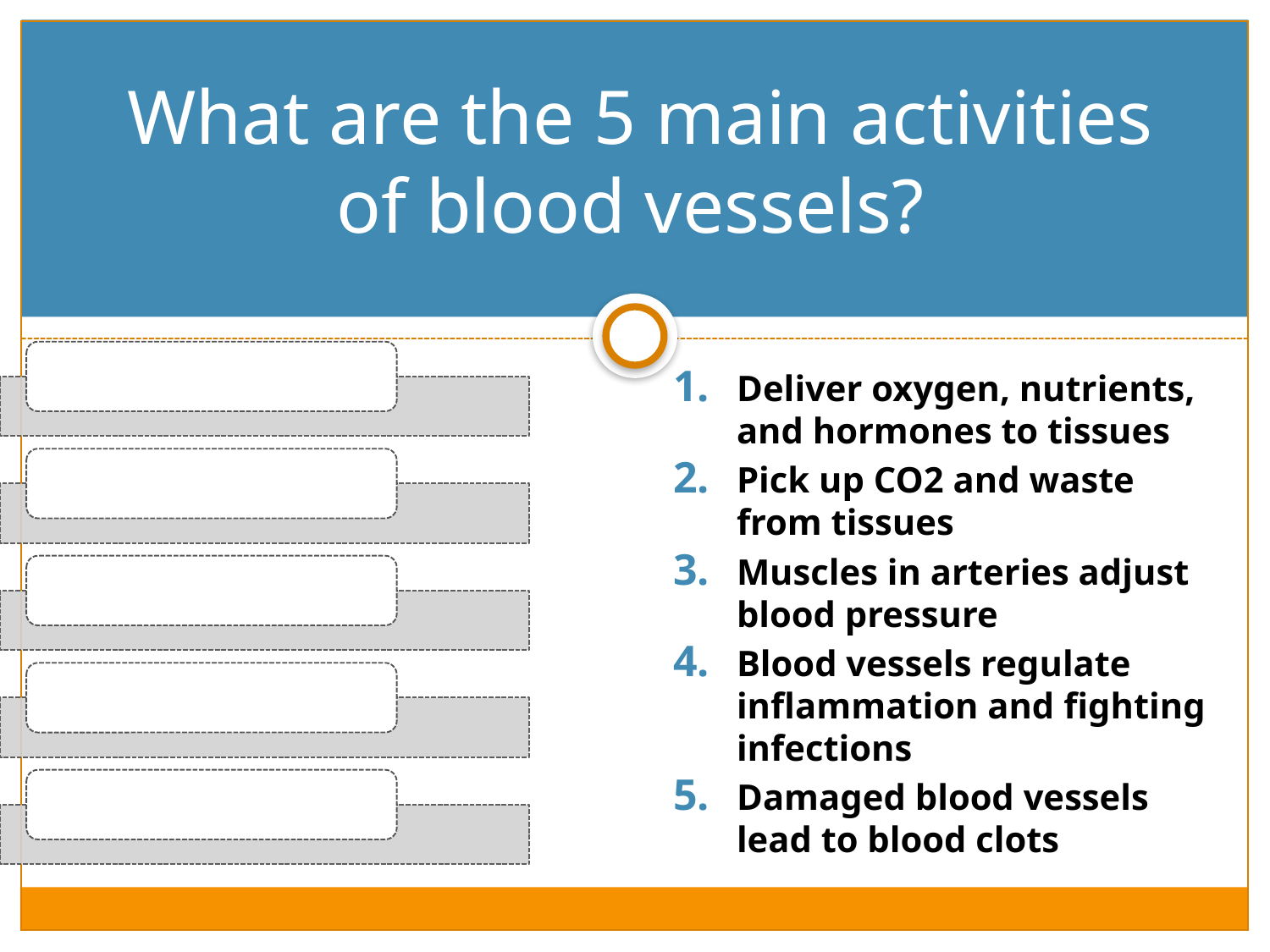

# What are the 5 main activities of blood vessels?
Deliver oxygen, nutrients, and hormones to tissues
Pick up CO2 and waste from tissues
Muscles in arteries adjust blood pressure
Blood vessels regulate inflammation and fighting infections
Damaged blood vessels lead to blood clots

## Slide 14
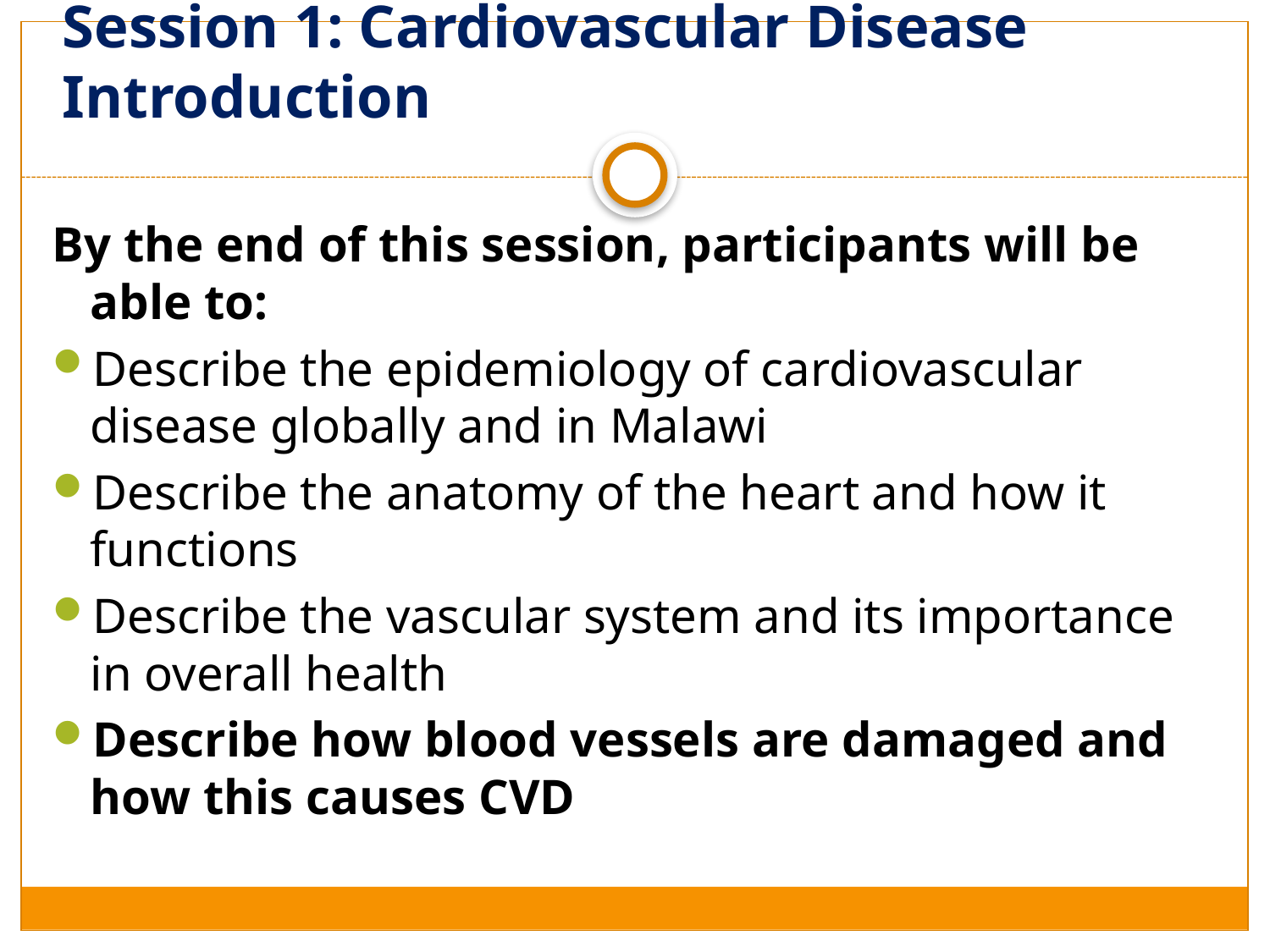

# Session 1: Cardiovascular Disease Introduction
By the end of this session, participants will be able to:
Describe the epidemiology of cardiovascular disease globally and in Malawi
Describe the anatomy of the heart and how it functions
Describe the vascular system and its importance in overall health
Describe how blood vessels are damaged and how this causes CVD

## Slide 15
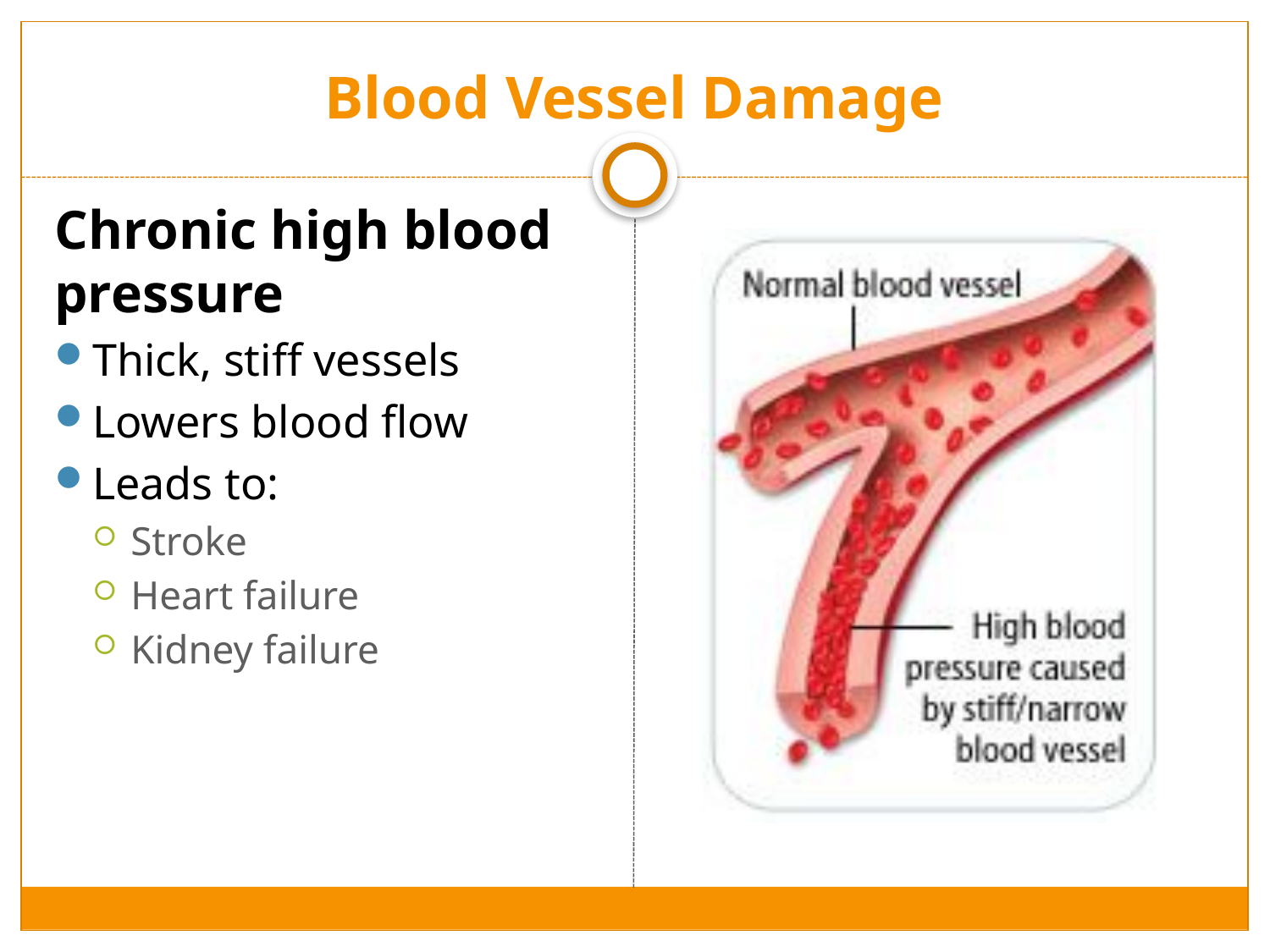

# Blood Vessel Damage
Chronic high blood pressure
Thick, stiff vessels
Lowers blood flow
Leads to:
Stroke
Heart failure
Kidney failure

## Slide 16
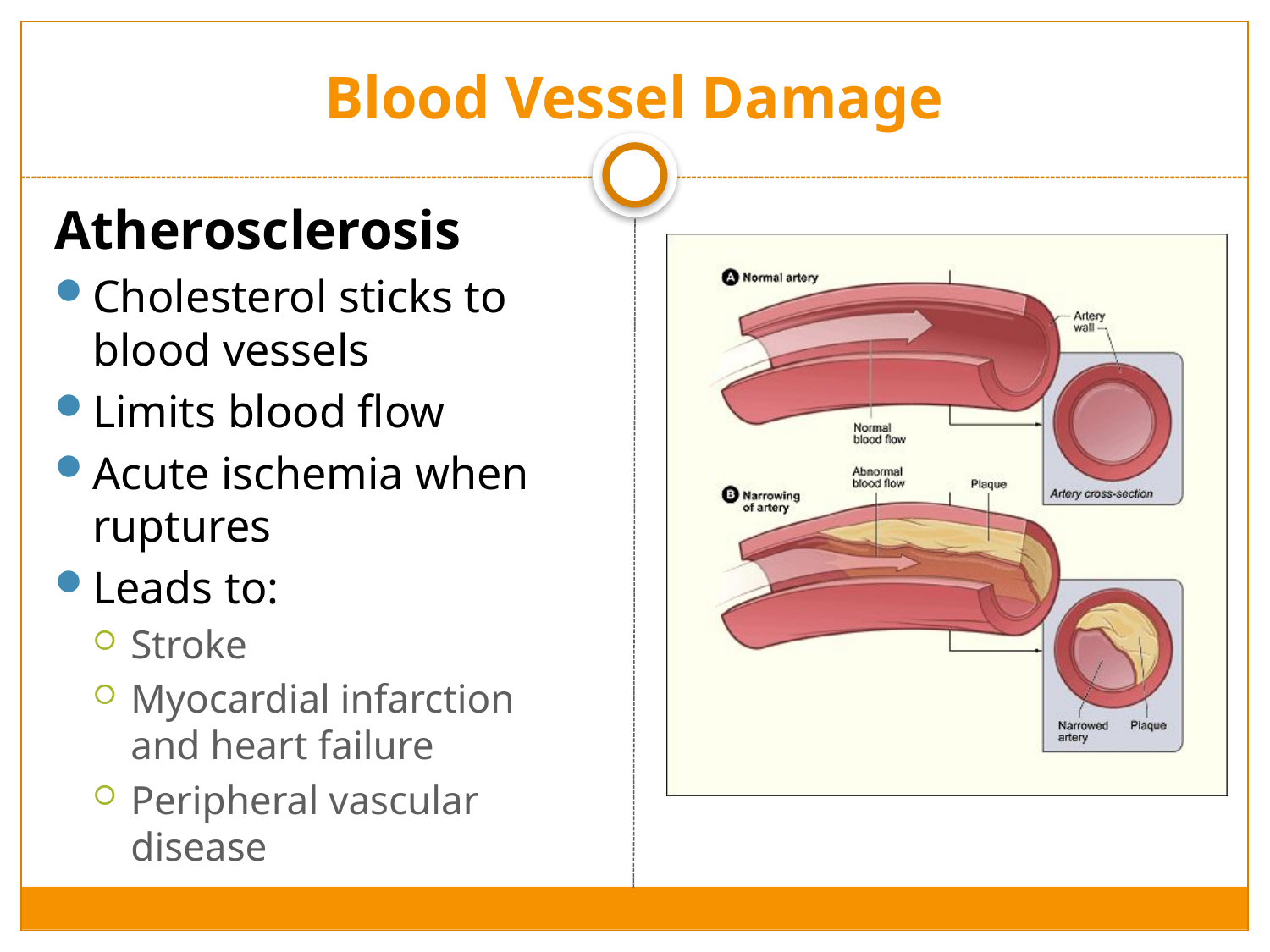

# Blood Vessel Damage
Atherosclerosis
Cholesterol sticks to blood vessels
Limits blood flow
Acute ischemia when ruptures
Leads to:
Stroke
Myocardial infarction and heart failure
Peripheral vascular disease

## Slide 17
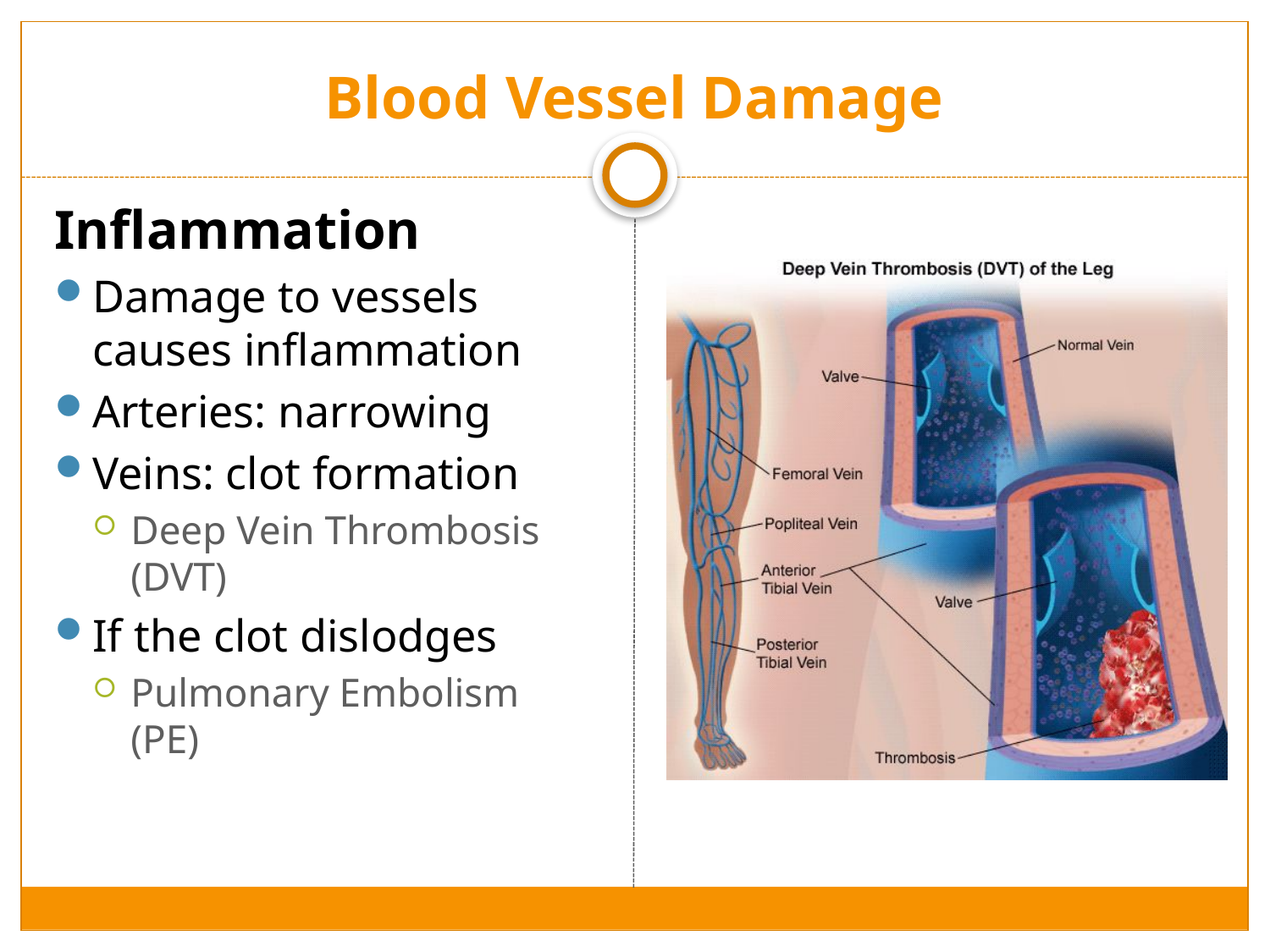

# Blood Vessel Damage
Inflammation
Damage to vessels causes inflammation
Arteries: narrowing
Veins: clot formation
Deep Vein Thrombosis (DVT)
If the clot dislodges
Pulmonary Embolism (PE)

## Slide 18
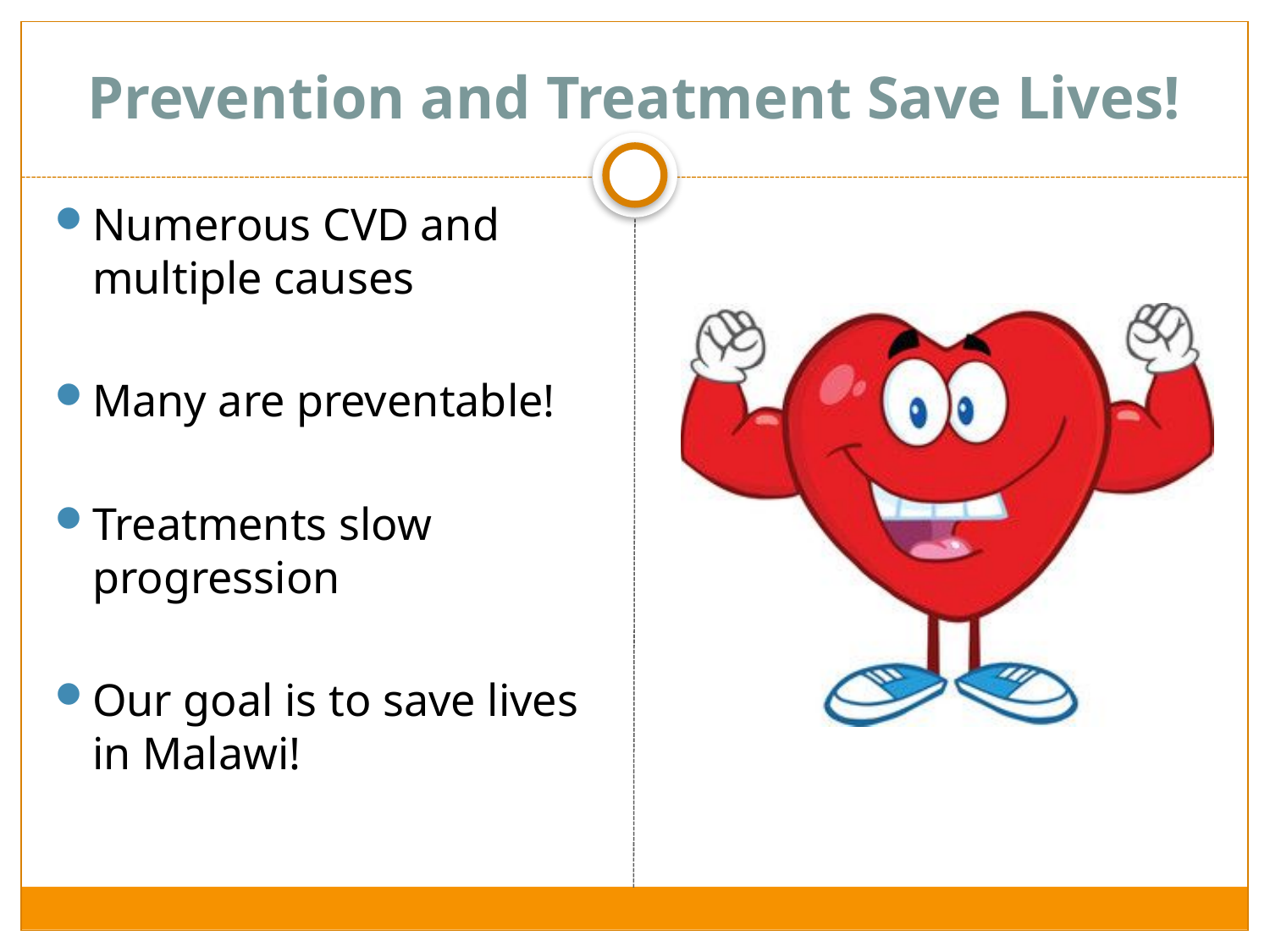

# Prevention and Treatment Save Lives!
Numerous CVD and multiple causes
Many are preventable!
Treatments slow progression
Our goal is to save lives in Malawi!

## Slide 19
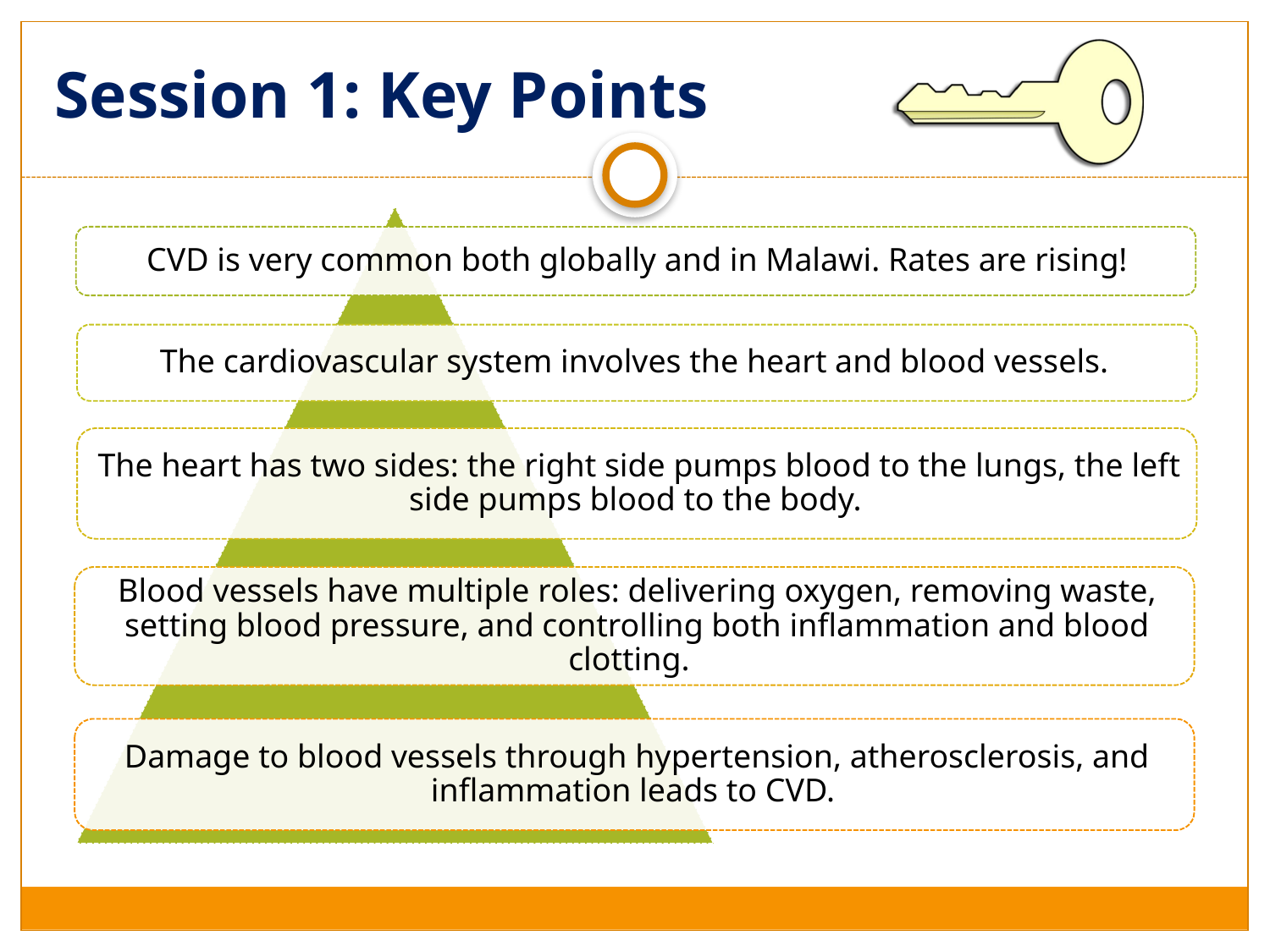

# Session 1: Key Points
