# Supplementary material for: Training Mid-Level Providers to Treat Severe Non-Communicable Diseases in Neno, Malawi through PEN-Plus Strategies
Source: Ann Glob Health. 2022 Aug 11;88(1):69. doi: 10.5334/aogh.3750 (PMC9389951; doi:10.5334/aogh.3750)
Supplement: Didactic Materials. — The supplementary materials contain a suggested didactic training schedule and the PowerPoint presentations used for PEN-Plus training in Neno, Malawi. These materials have been reviewed and accepted by the Malawi Ministry of Health for future PEN-Plus trainings in Malawi. [file agh-88-1-3750-s2.zip › Didactic_Materials/CV_HTN and Preeclampsia.pptx]

## Slide 1
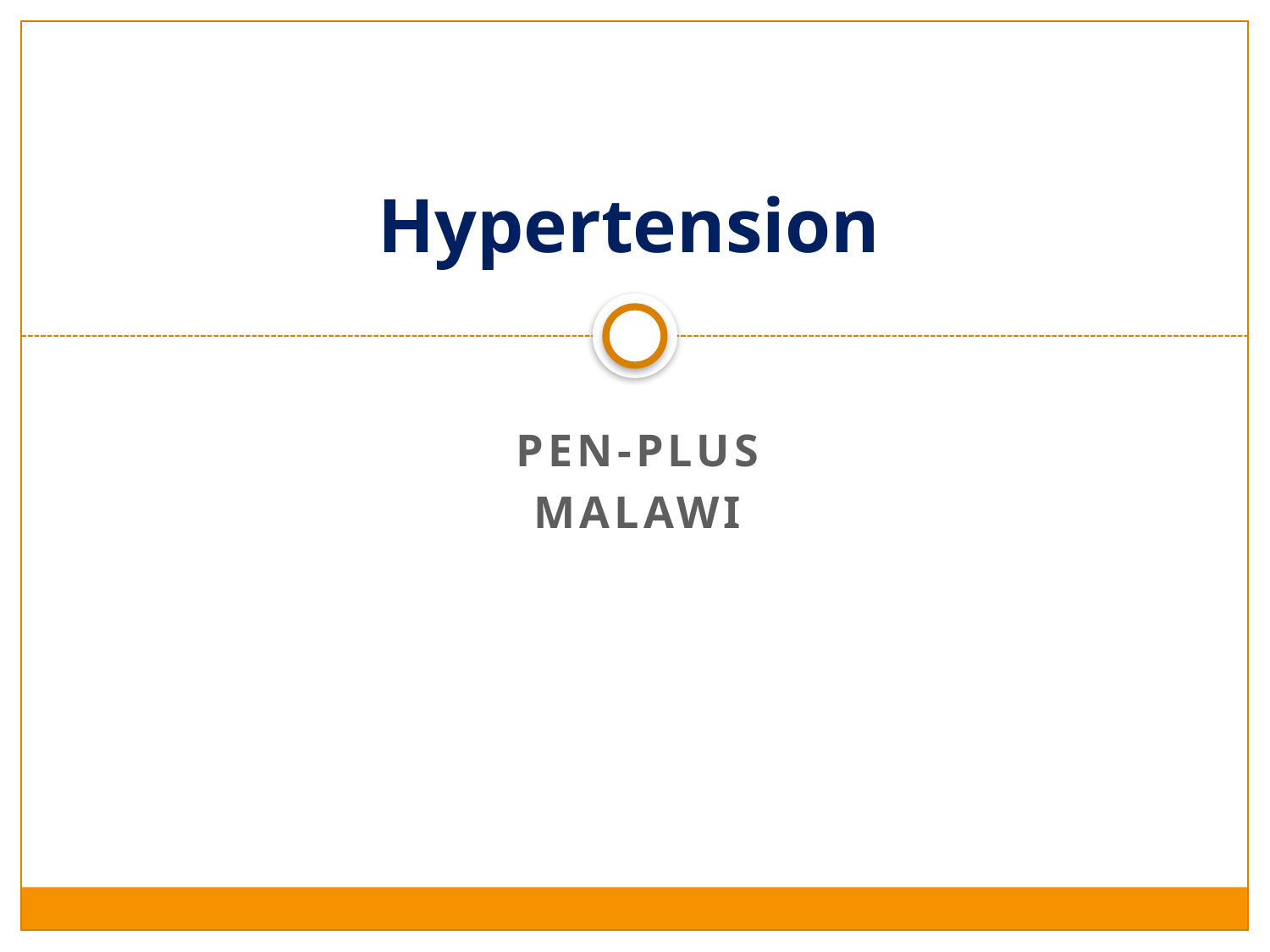

# Hypertension
PEN-Plus
Malawi

## Slide 2
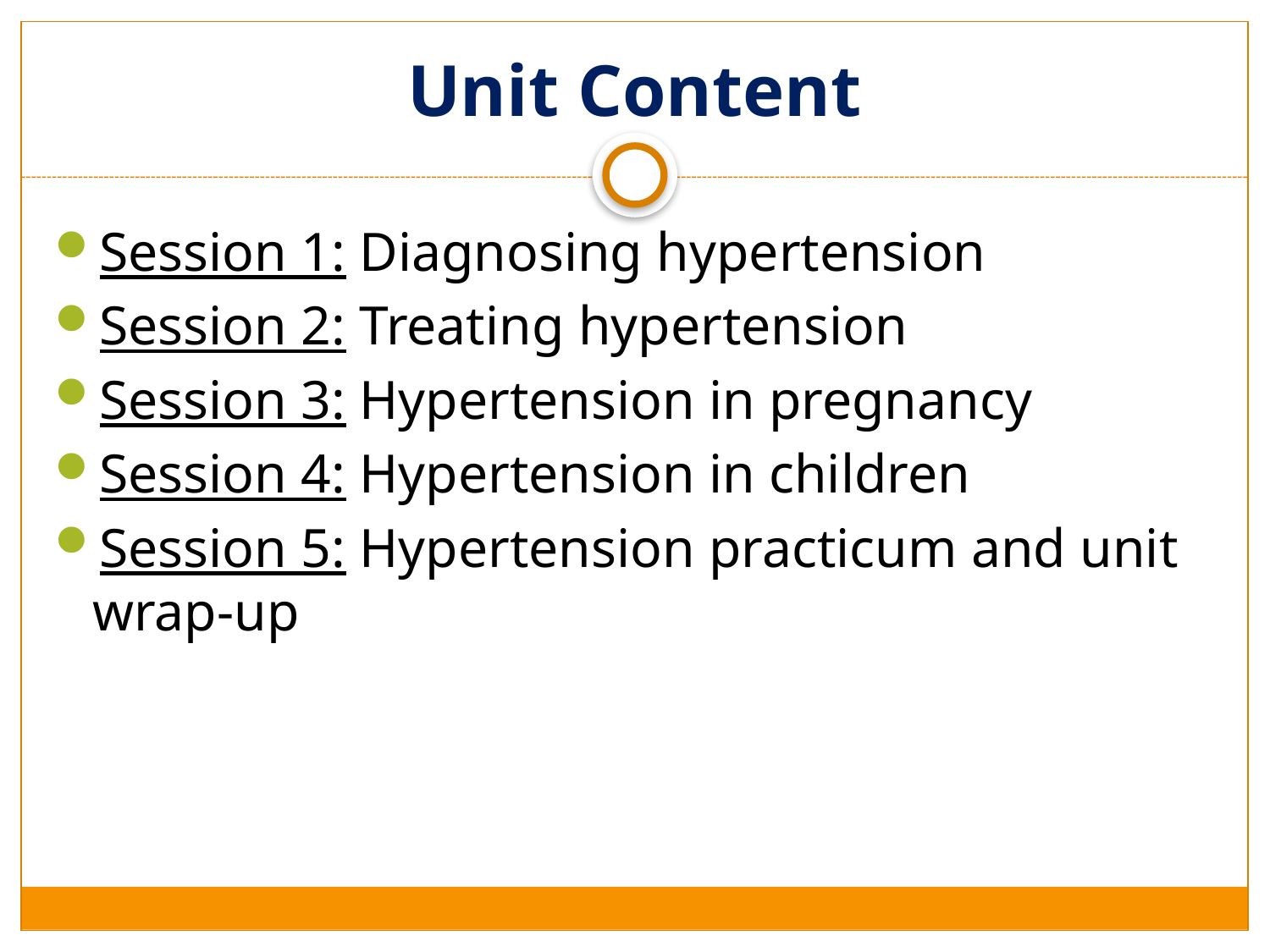

# Unit Content
Session 1: Diagnosing hypertension
Session 2: Treating hypertension
Session 3: Hypertension in pregnancy
Session 4: Hypertension in children
Session 5: Hypertension practicum and unit wrap-up

## Slide 3
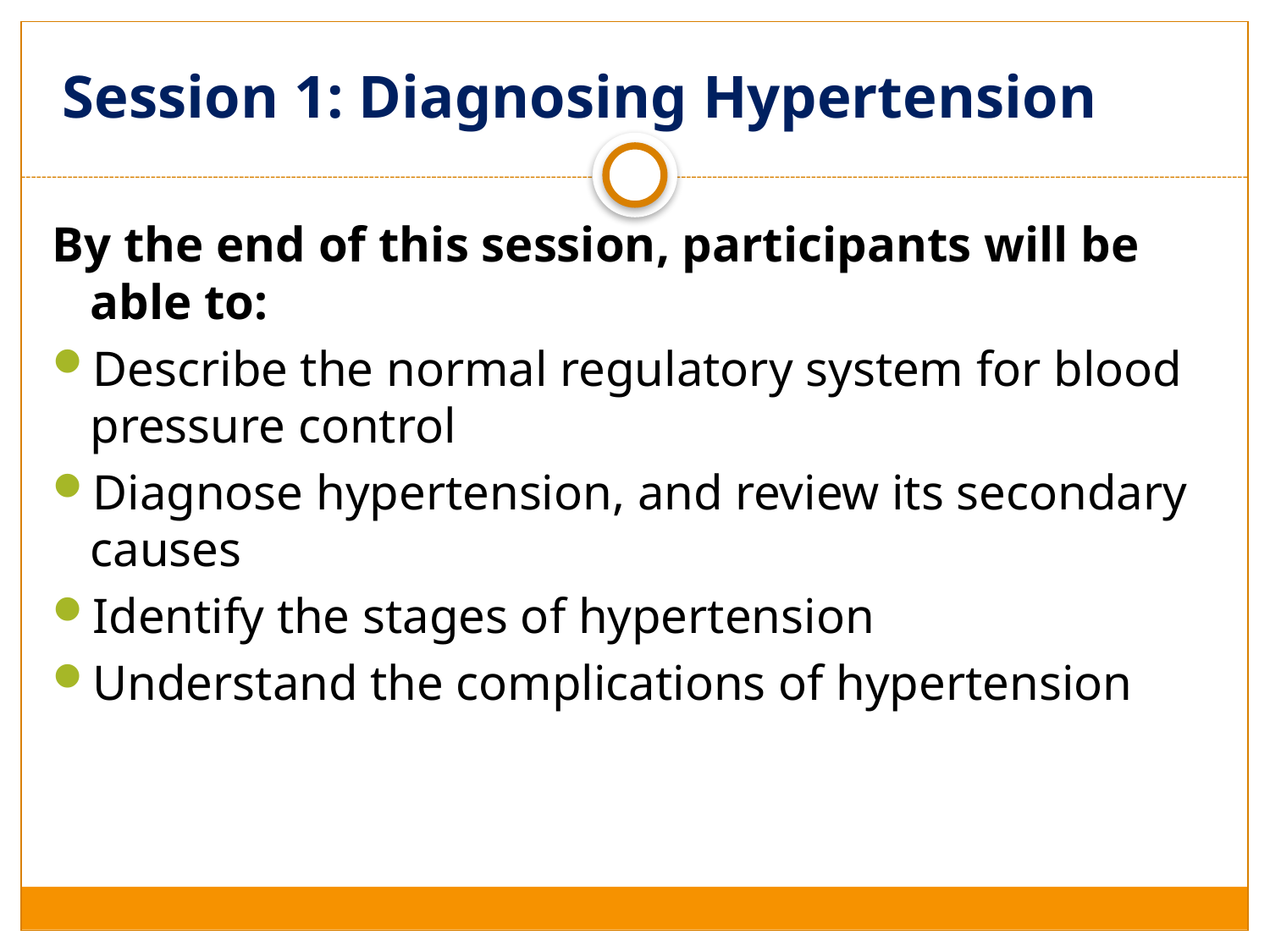

# Session 1: Diagnosing Hypertension
By the end of this session, participants will be able to:
Describe the normal regulatory system for blood pressure control
Diagnose hypertension, and review its secondary causes
Identify the stages of hypertension
Understand the complications of hypertension

## Slide 4
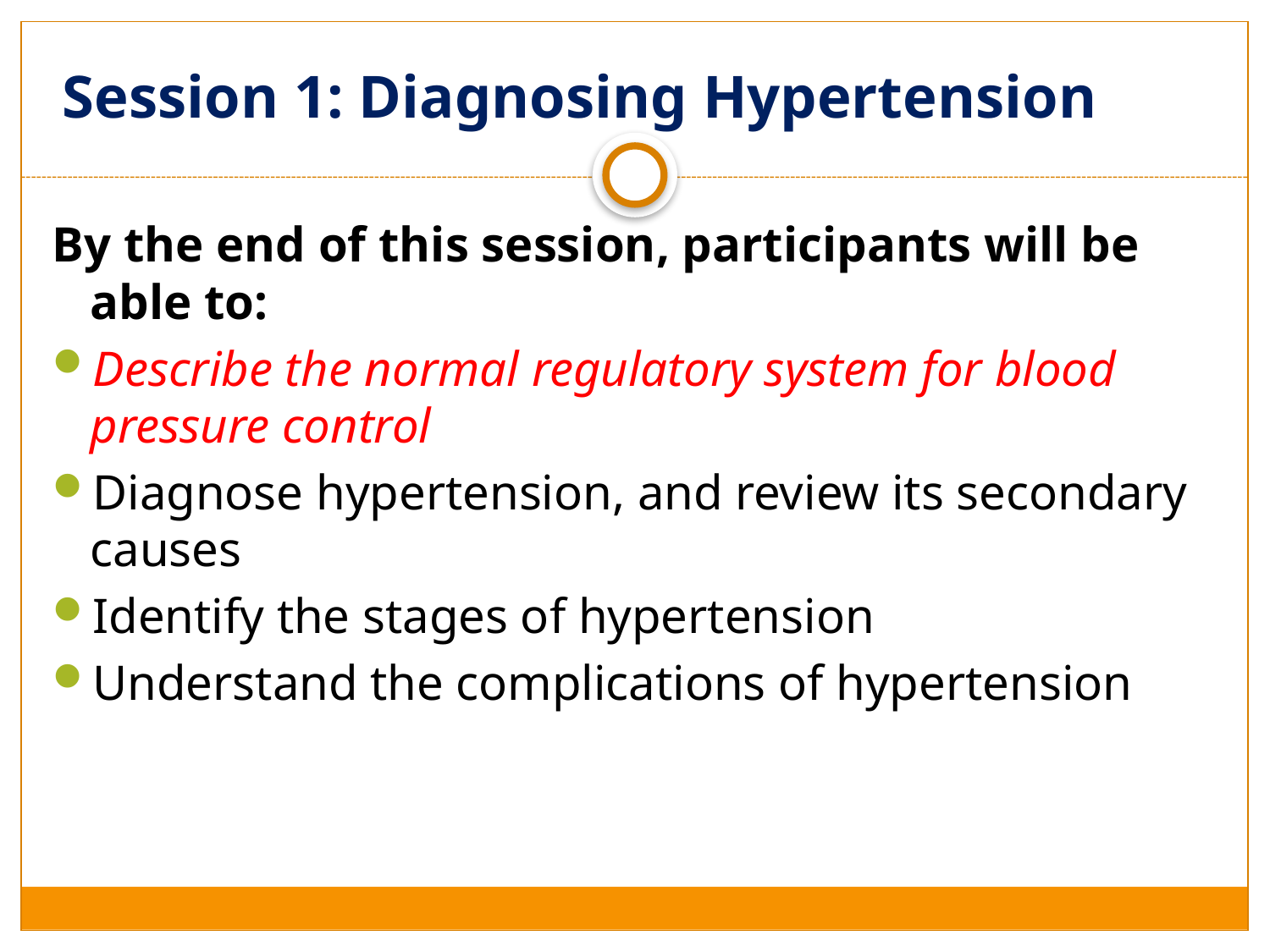

# Session 1: Diagnosing Hypertension
By the end of this session, participants will be able to:
Describe the normal regulatory system for blood pressure control
Diagnose hypertension, and review its secondary causes
Identify the stages of hypertension
Understand the complications of hypertension

## Slide 5
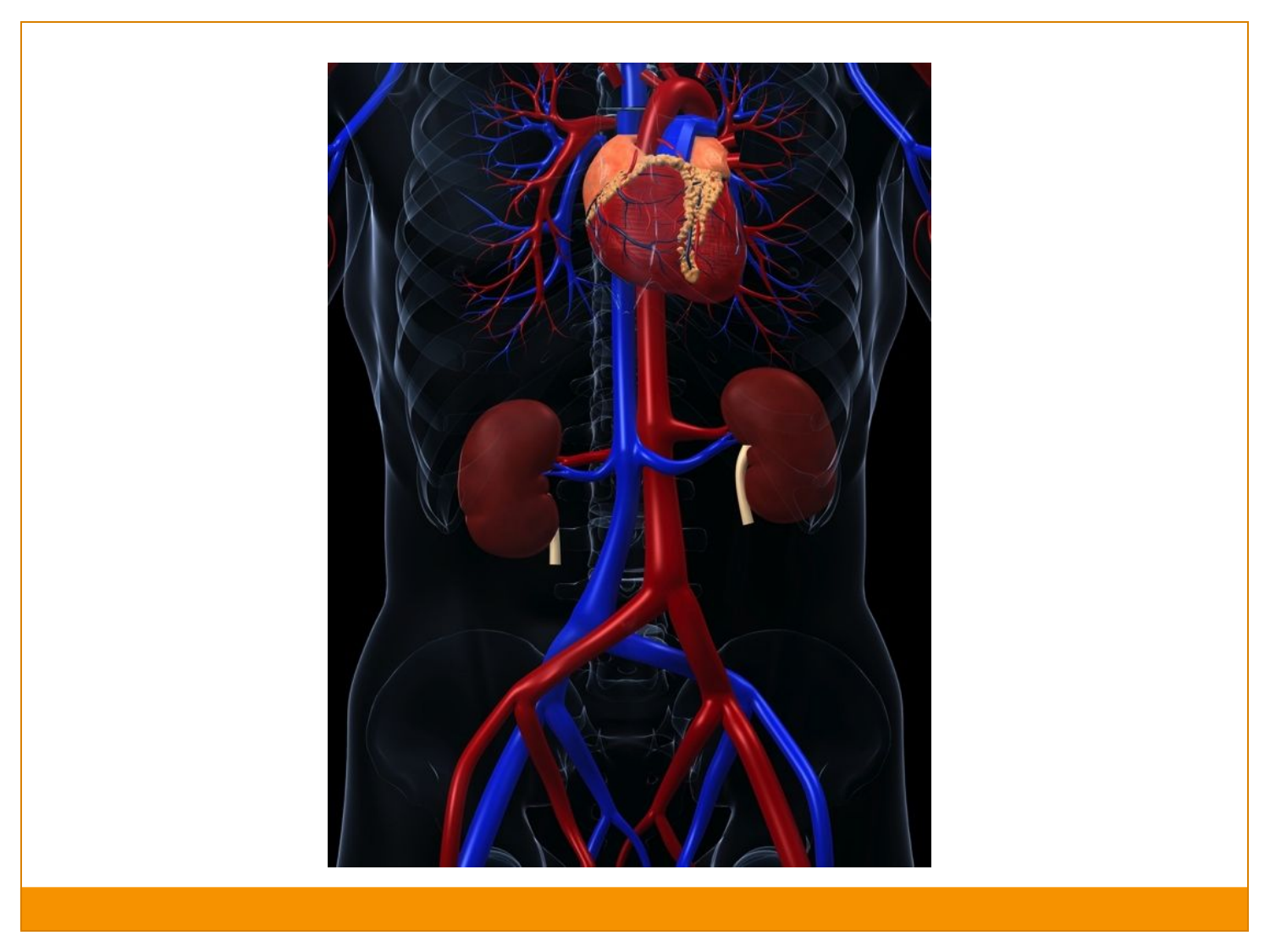

## Slide 6
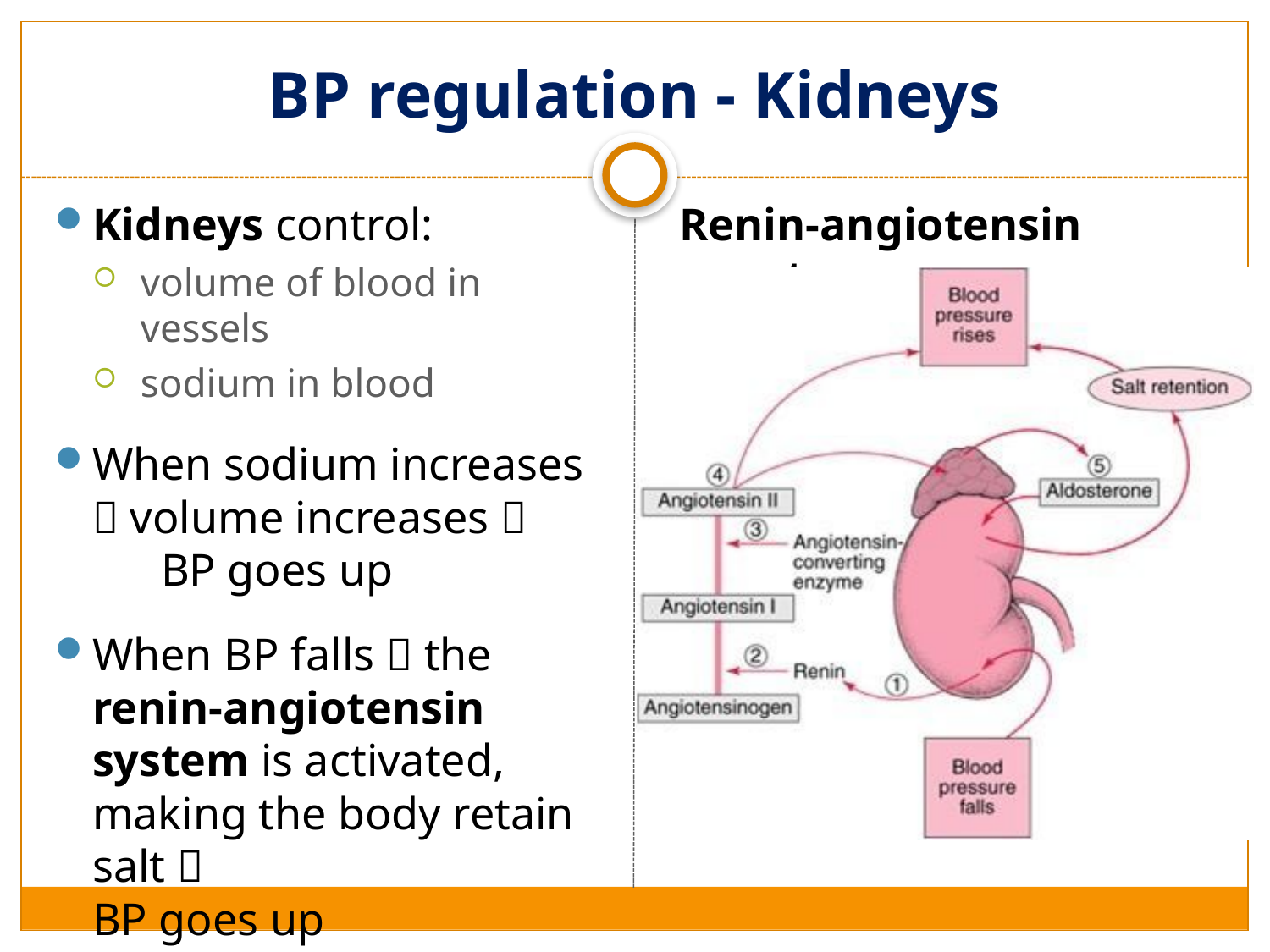

# BP regulation - Kidneys
Kidneys control:
volume of blood in vessels
sodium in blood
When sodium increases  volume increases  BP goes up
When BP falls  the
renin-angiotensin system is activated, making the body retain salt 
BP goes up
Renin-angiotensin system

## Slide 7
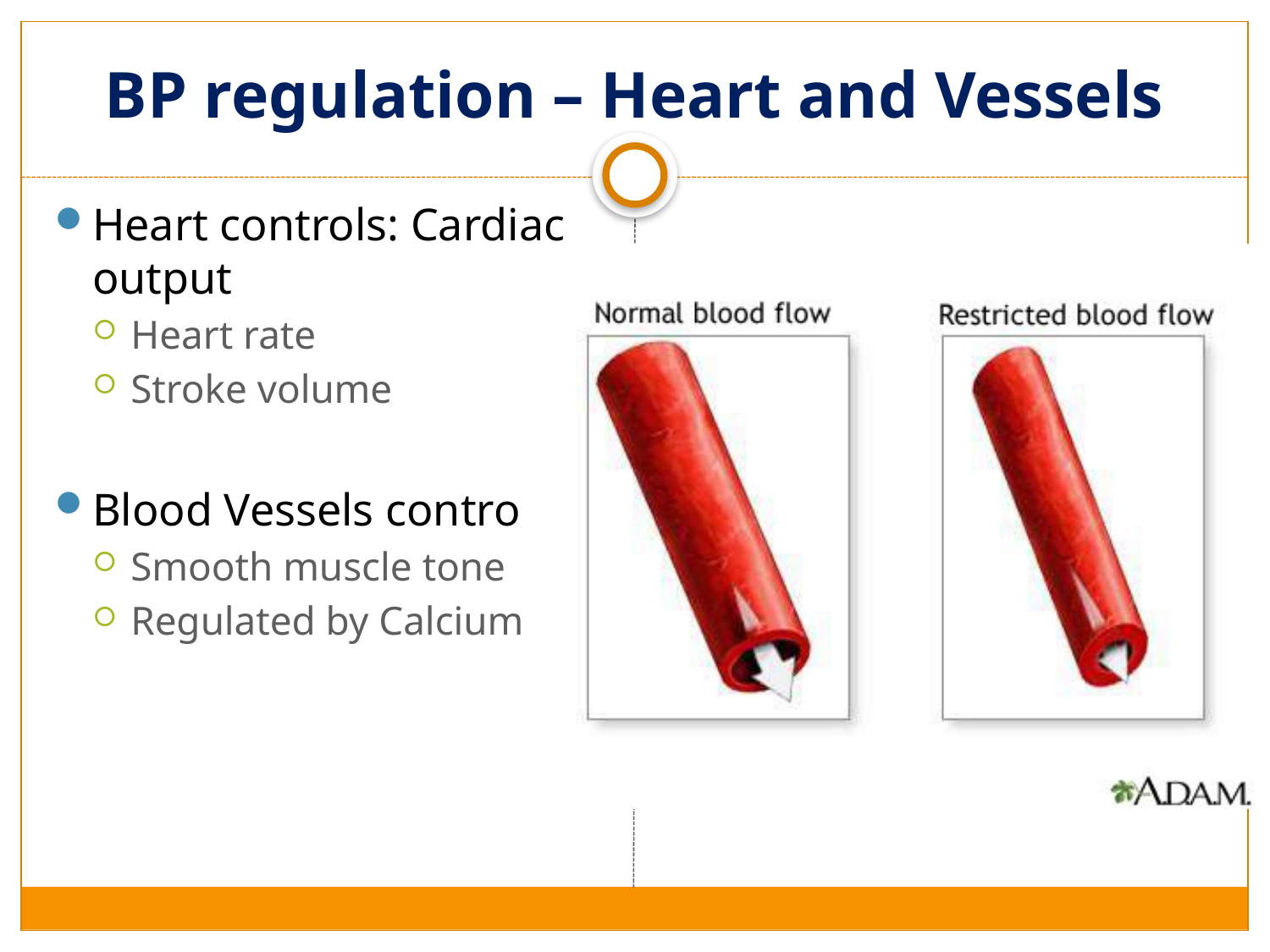

# BP regulation – Heart and Vessels
Heart controls: Cardiac output
Heart rate
Stroke volume
Blood Vessels control:
Smooth muscle tone
Regulated by Calcium

## Slide 8
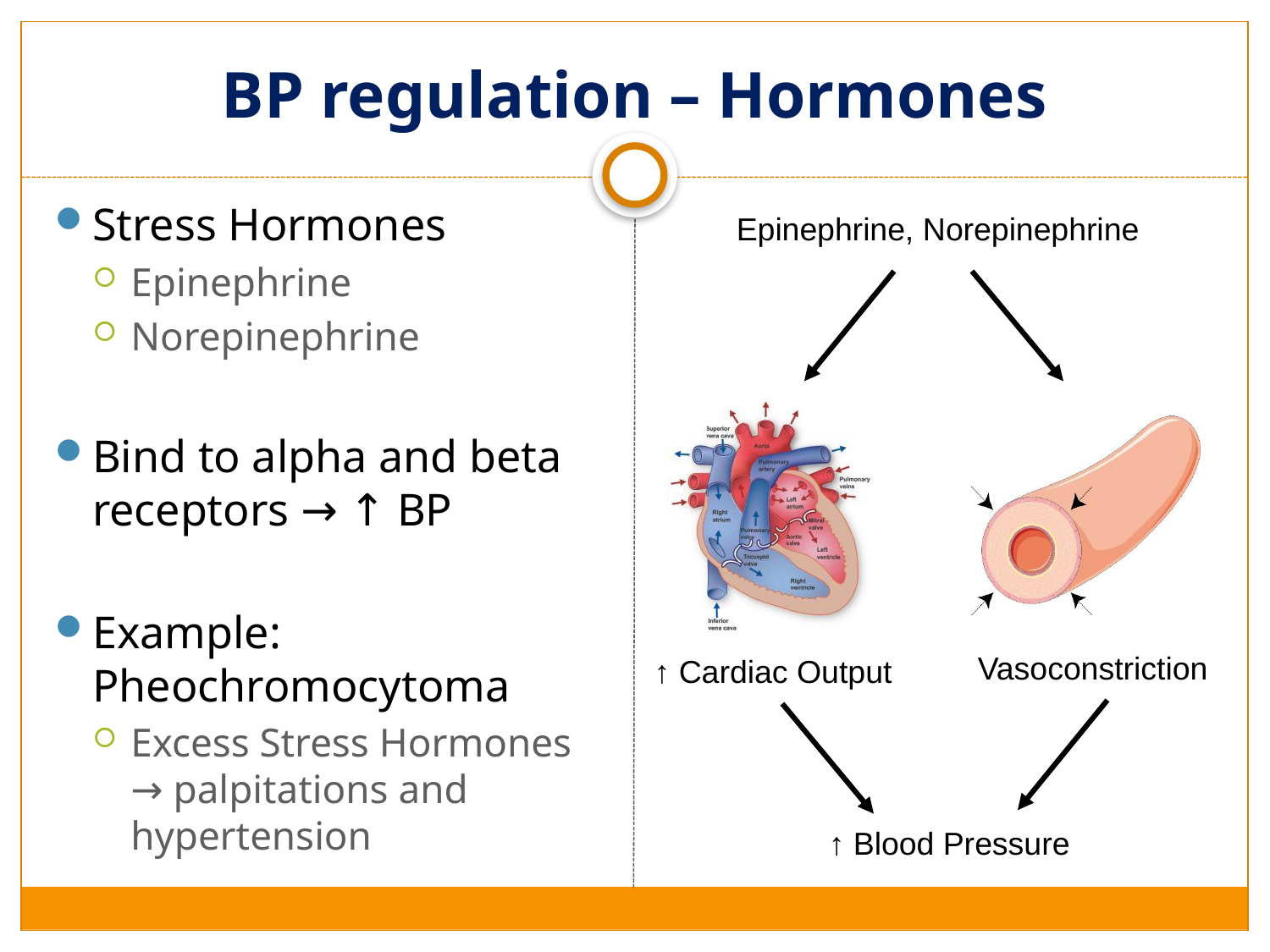

# BP regulation – Hormones
Stress Hormones
Epinephrine
Norepinephrine
Bind to alpha and beta receptors → ↑ BP
Example: Pheochromocytoma
Excess Stress Hormones → palpitations and hypertension
Epinephrine, Norepinephrine
Vasoconstriction
↑ Cardiac Output
↑ Blood Pressure

## Slide 9
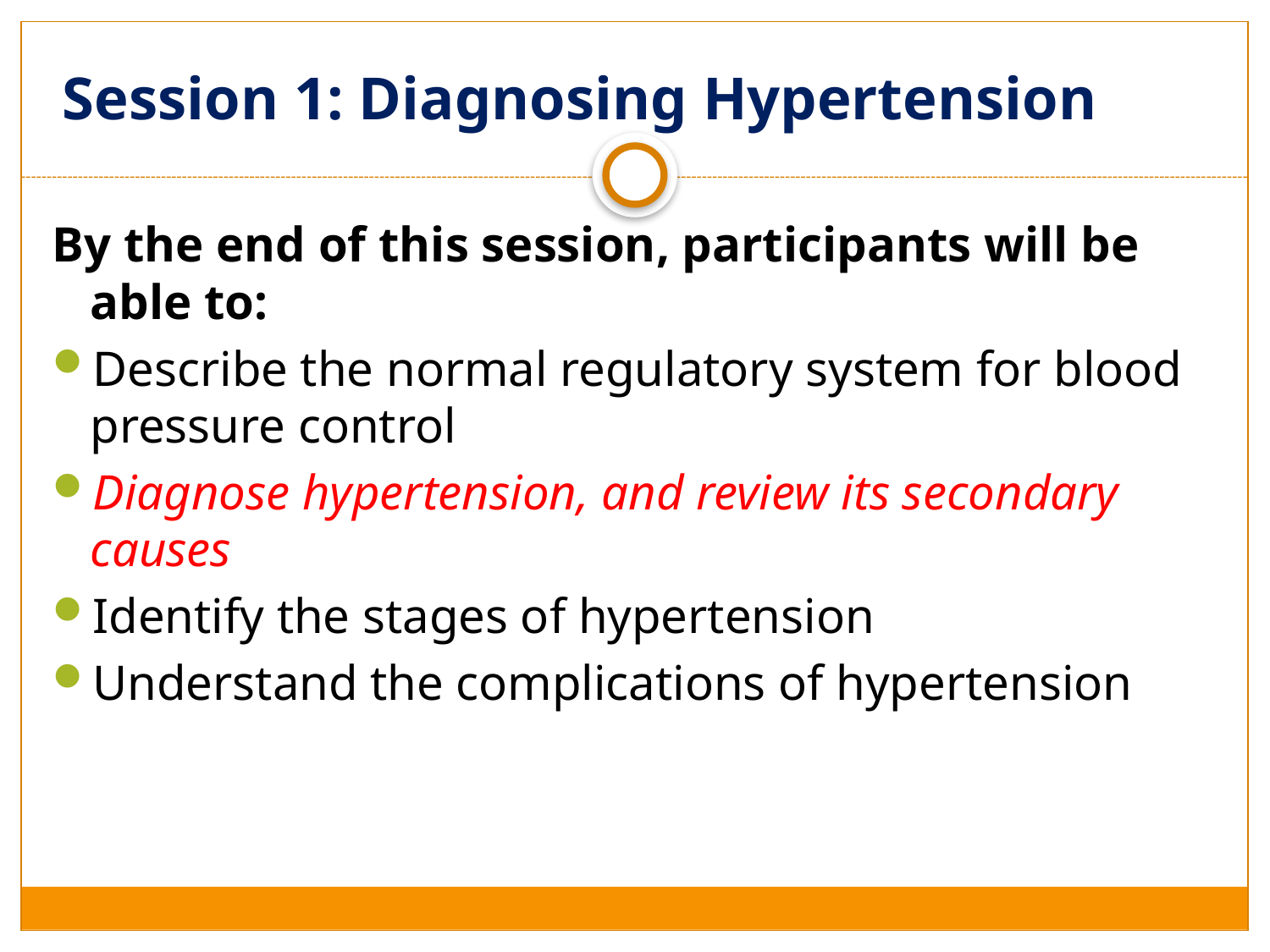

# Session 1: Diagnosing Hypertension
By the end of this session, participants will be able to:
Describe the normal regulatory system for blood pressure control
Diagnose hypertension, and review its secondary causes
Identify the stages of hypertension
Understand the complications of hypertension

## Slide 10
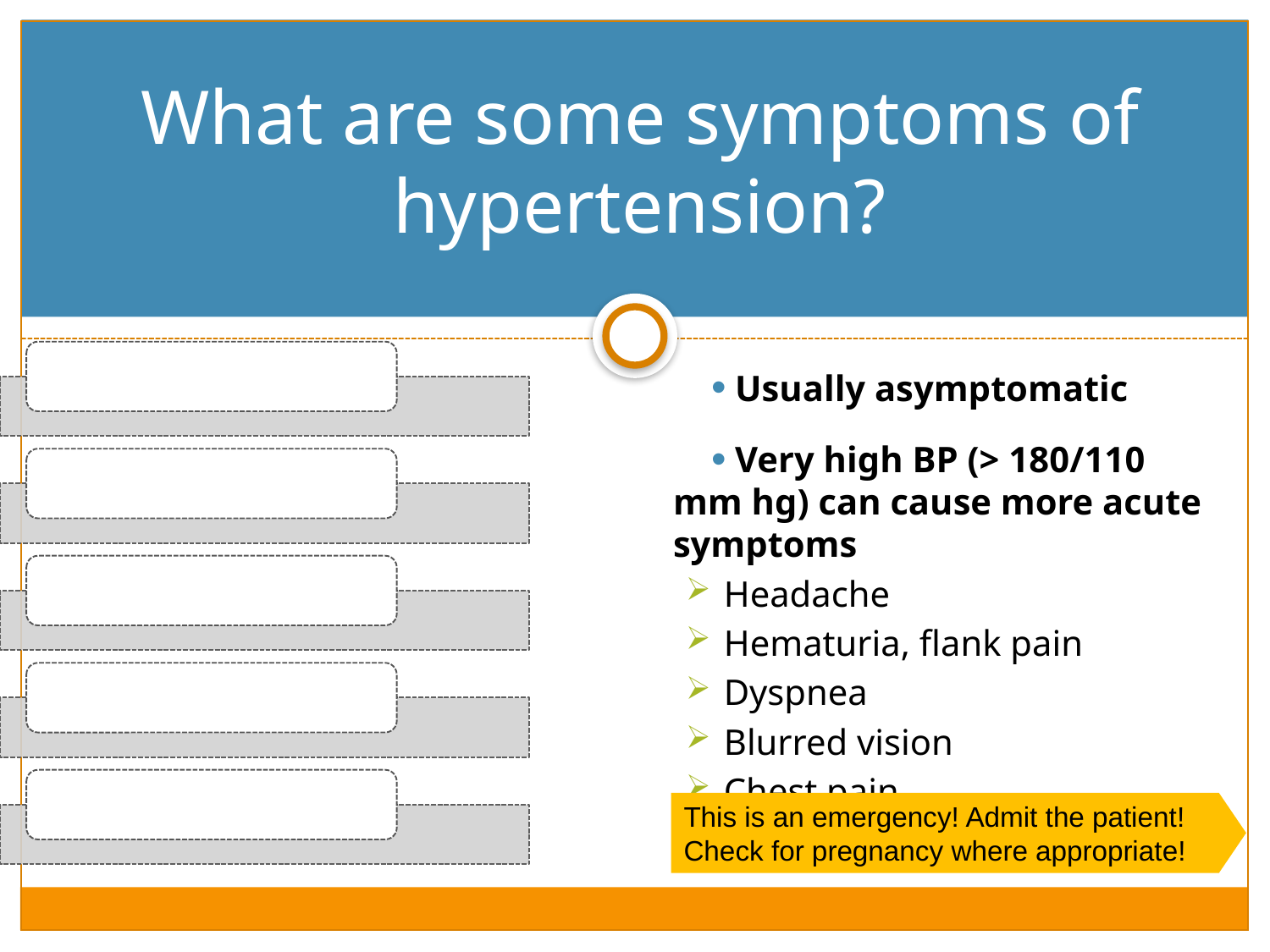

# What are some symptoms of hypertension?
 Usually asymptomatic
 Very high BP (> 180/110 mm hg) can cause more acute symptoms
Headache
Hematuria, flank pain
Dyspnea
Blurred vision
Chest pain
This is an emergency! Admit the patient!
Check for pregnancy where appropriate!

## Slide 11
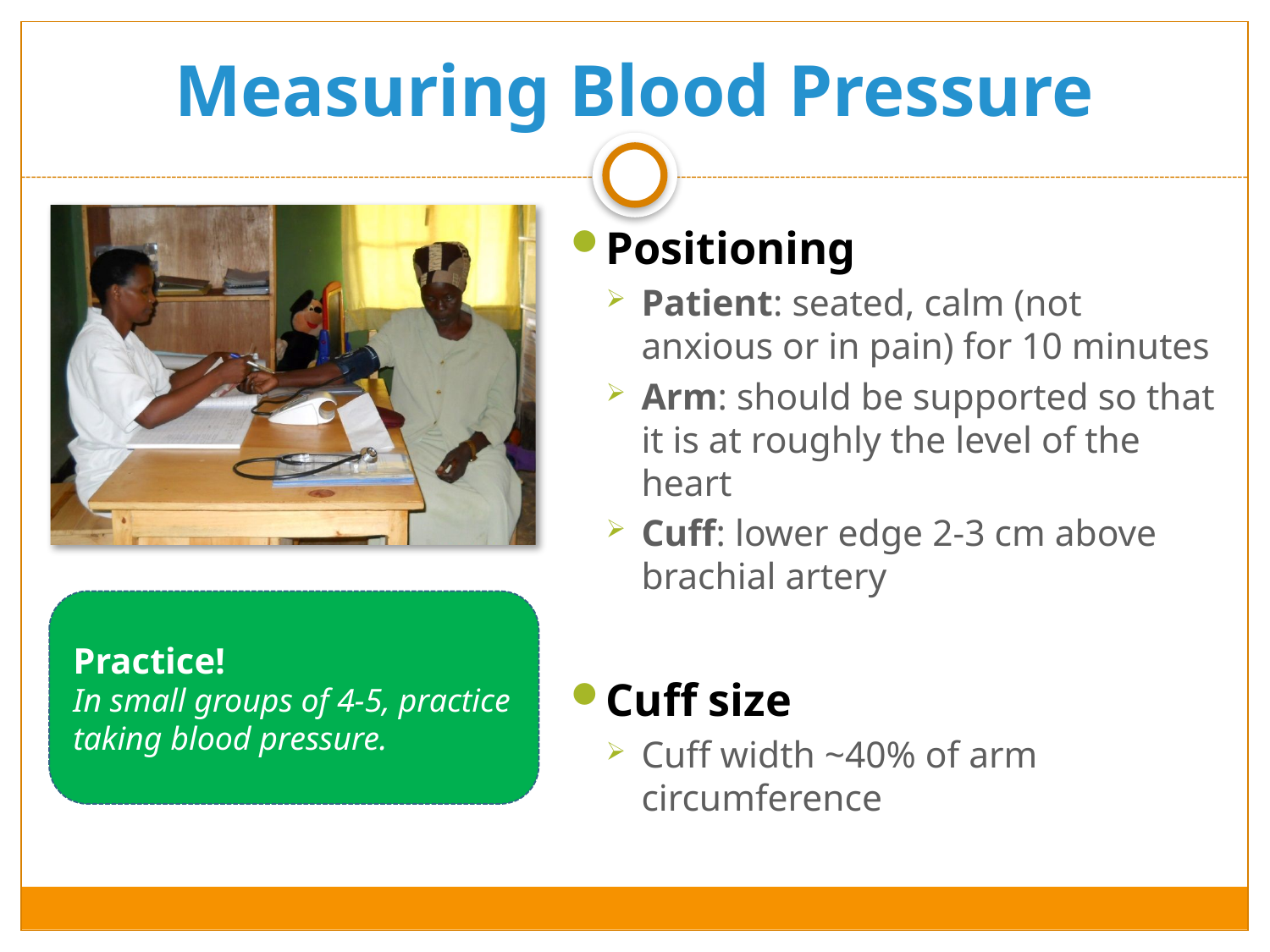

# Measuring Blood Pressure
Positioning
Patient: seated, calm (not anxious or in pain) for 10 minutes
Arm: should be supported so that it is at roughly the level of the heart
Cuff: lower edge 2-3 cm above brachial artery
Cuff size
Cuff width ~40% of arm circumference
Practice!
In small groups of 4-5, practice taking blood pressure.

## Slide 12
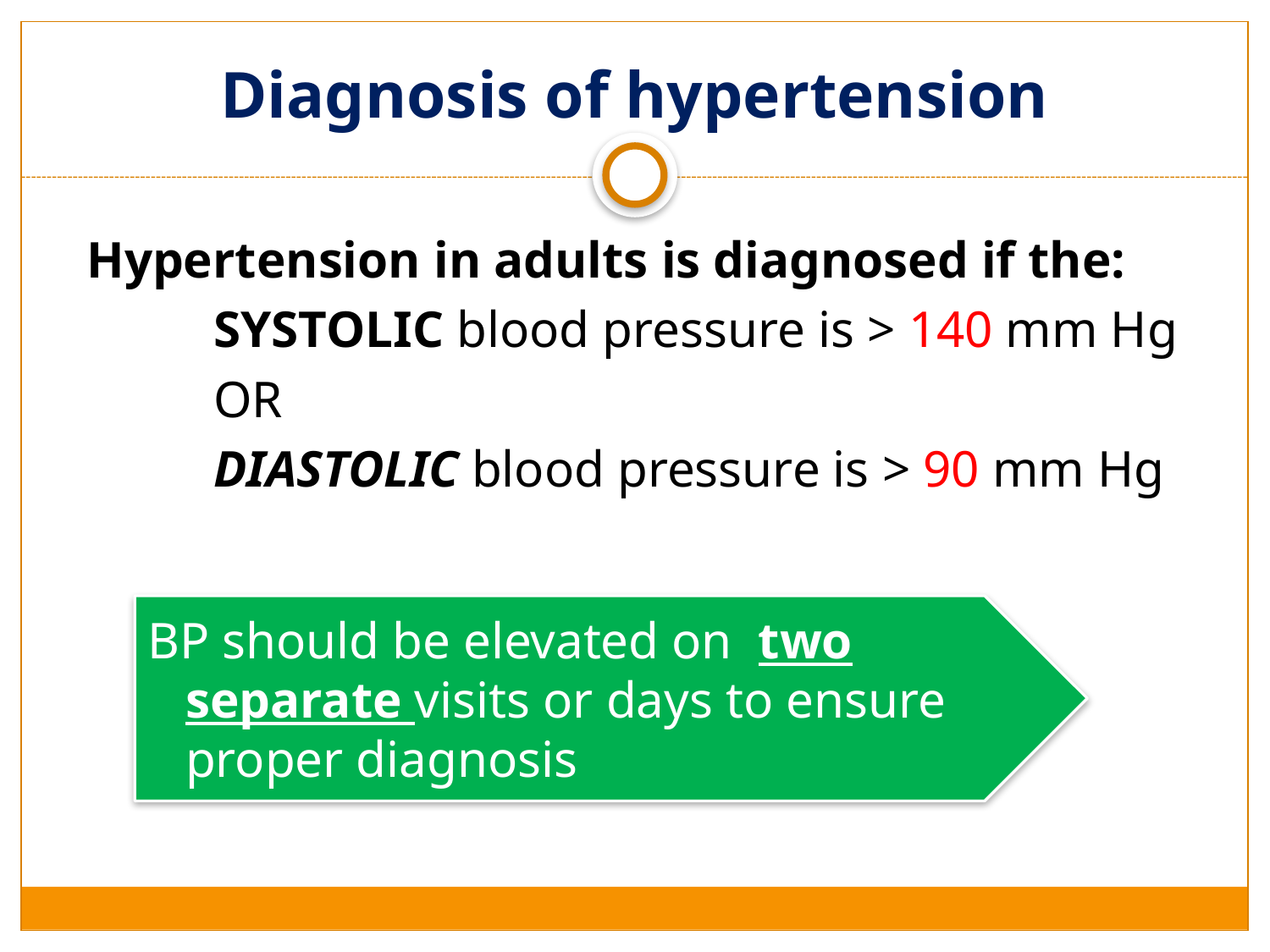

# Diagnosis of hypertension
Hypertension in adults is diagnosed if the:
	SYSTOLIC blood pressure is > 140 mm Hg
	OR
	DIASTOLIC blood pressure is > 90 mm Hg
BP should be elevated on two separate visits or days to ensure proper diagnosis

## Slide 13
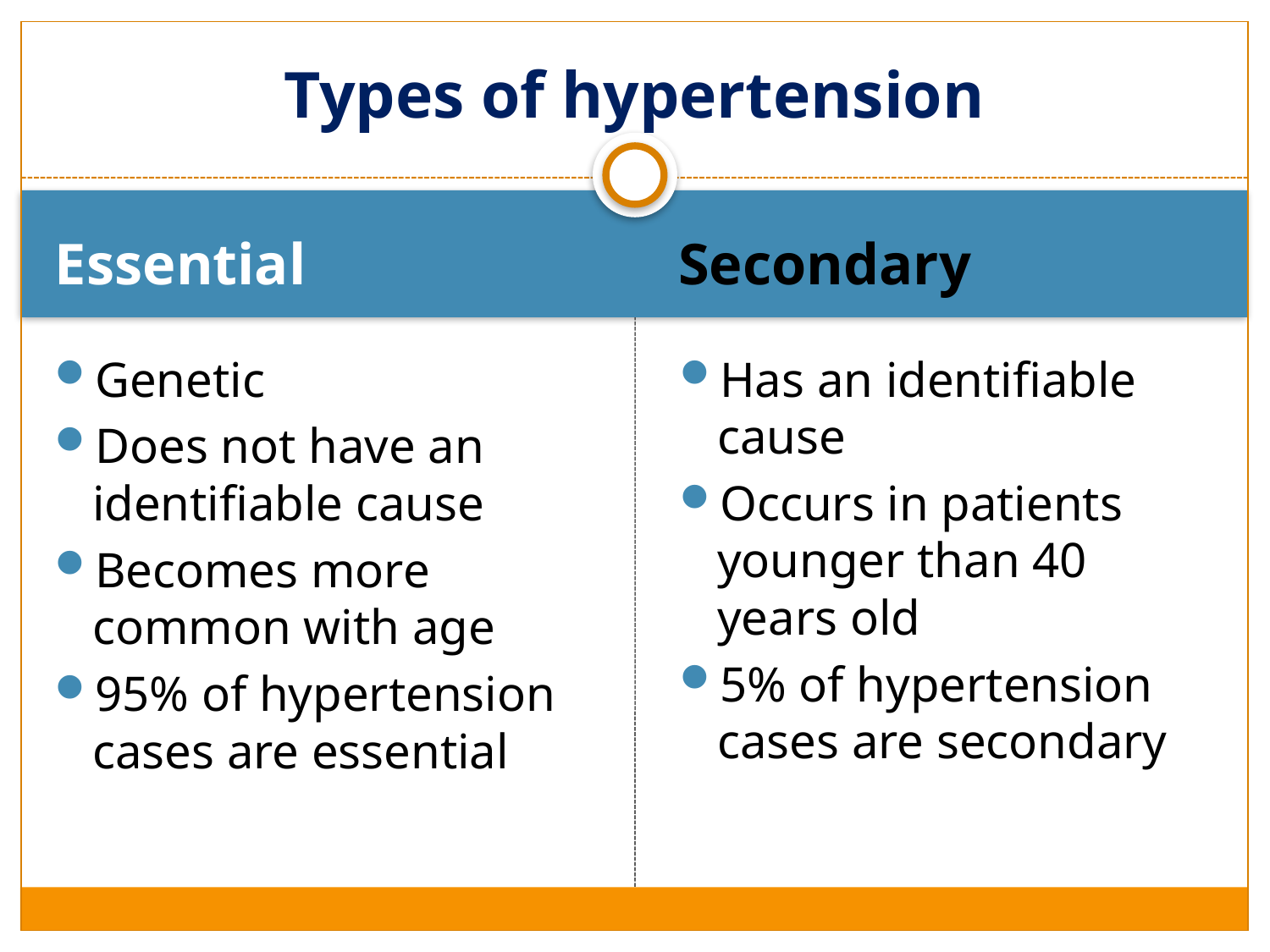

# Types of hypertension
Essential
Secondary
Genetic
Does not have an identifiable cause
Becomes more common with age
95% of hypertension cases are essential
Has an identifiable cause
Occurs in patients younger than 40 years old
5% of hypertension cases are secondary

## Slide 14
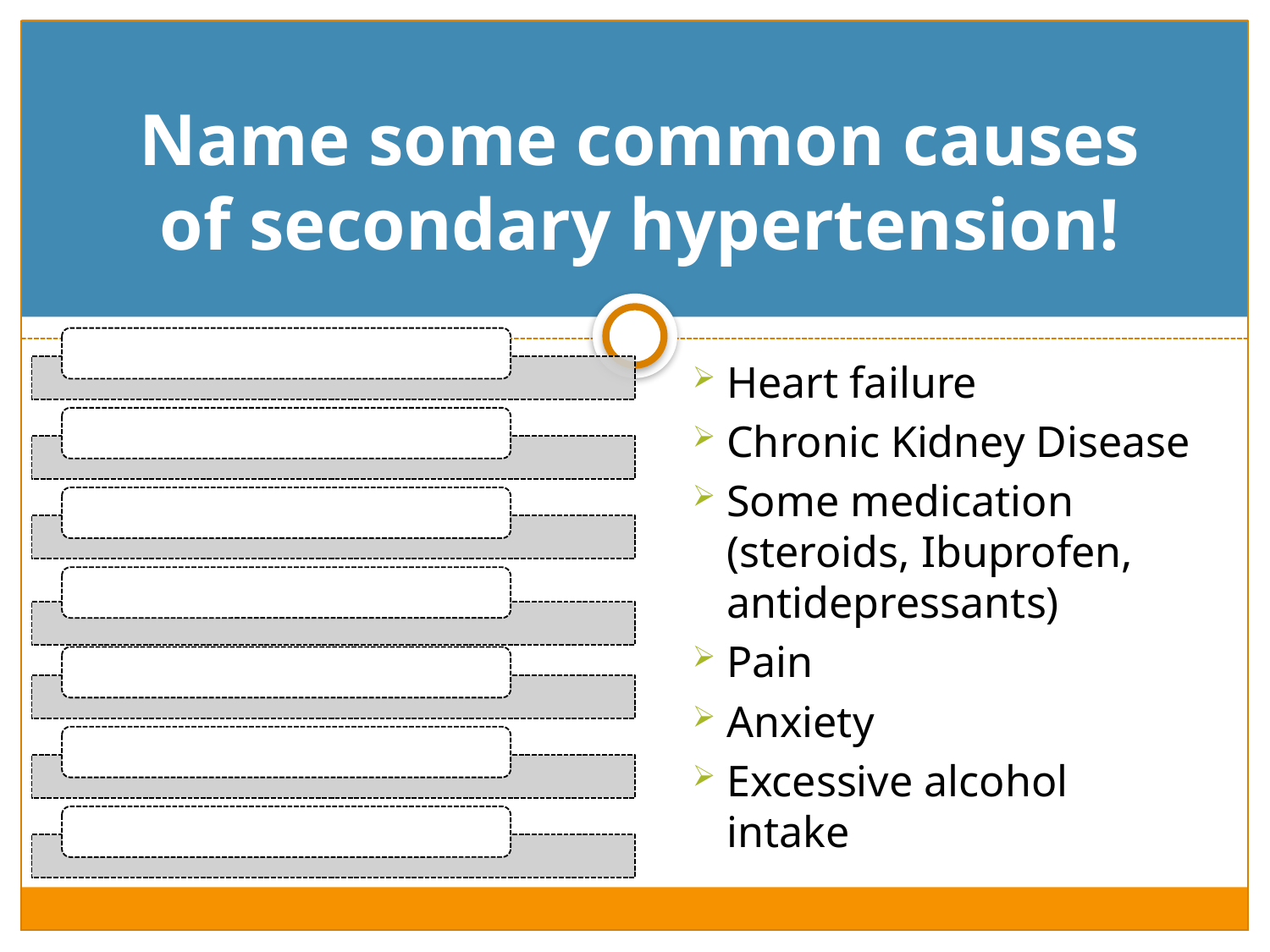

# Name some common causes of secondary hypertension!
Heart failure
Chronic Kidney Disease
Some medication (steroids, Ibuprofen, antidepressants)
Pain
Anxiety
Excessive alcohol intake

## Slide 15
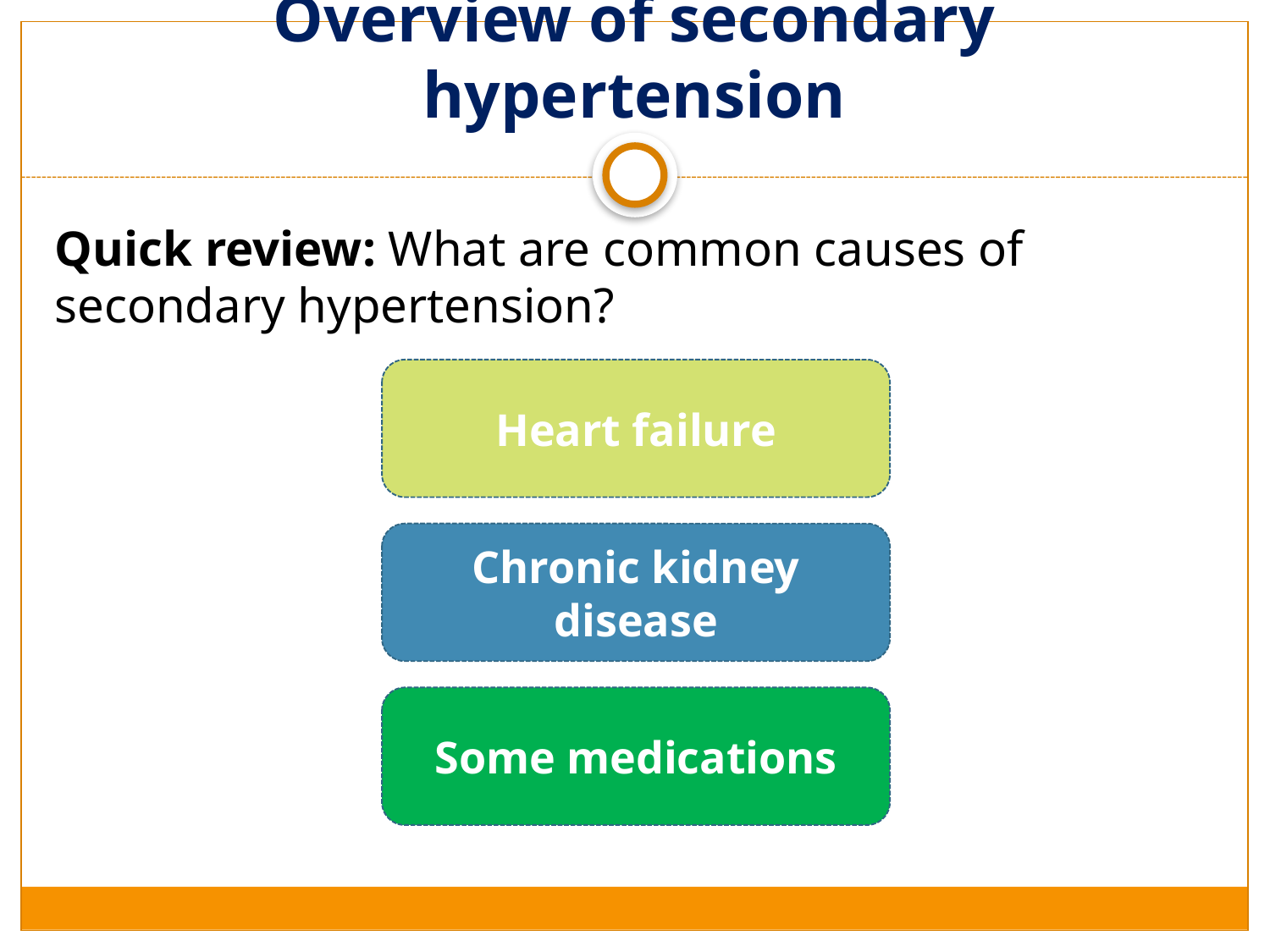

# Overview of secondary hypertension
Quick review: What are common causes of secondary hypertension?
Heart failure
Chronic kidney disease
Some medications

## Slide 16
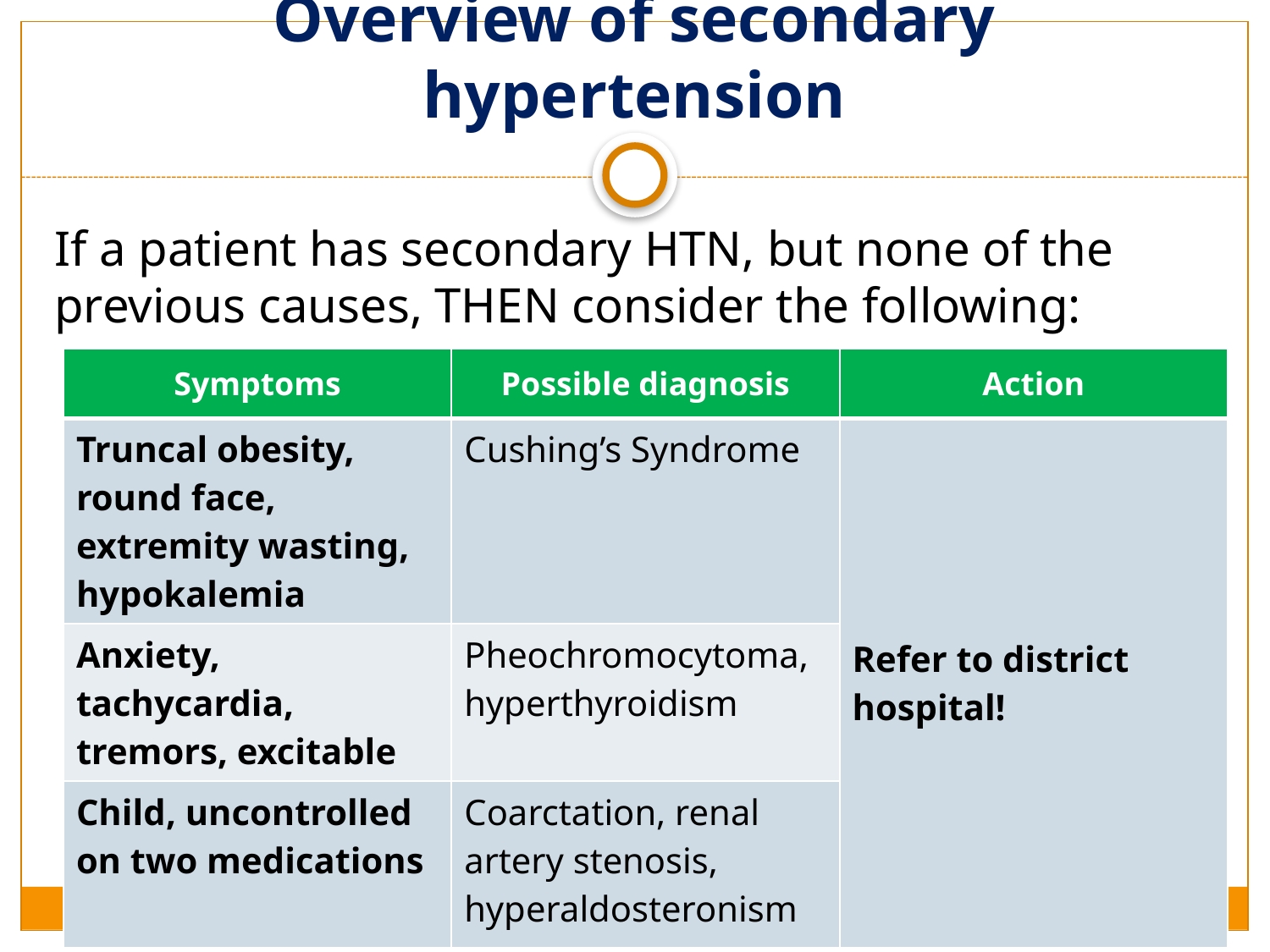

# Overview of secondary hypertension
If a patient has secondary HTN, but none of the previous causes, THEN consider the following:
| Symptoms | Possible diagnosis | Action |
| --- | --- | --- |
| Truncal obesity, round face, extremity wasting, hypokalemia | Cushing’s Syndrome | Refer to district hospital! |
| Anxiety, tachycardia, tremors, excitable | Pheochromocytoma, hyperthyroidism | |
| Child, uncontrolled on two medications | Coarctation, renal artery stenosis, hyperaldosteronism | |

## Slide 17
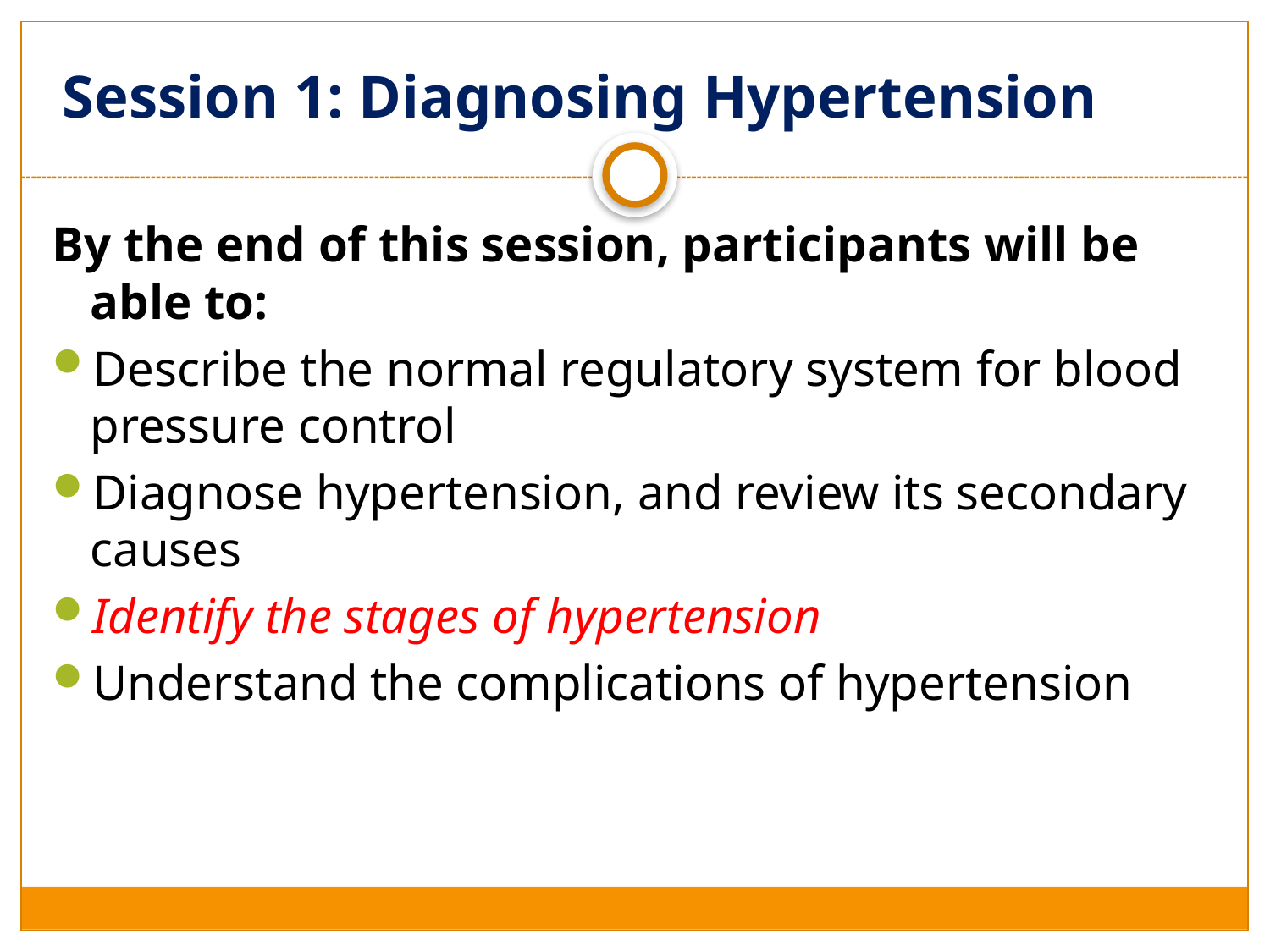

# Session 1: Diagnosing Hypertension
By the end of this session, participants will be able to:
Describe the normal regulatory system for blood pressure control
Diagnose hypertension, and review its secondary causes
Identify the stages of hypertension
Understand the complications of hypertension

## Slide 18
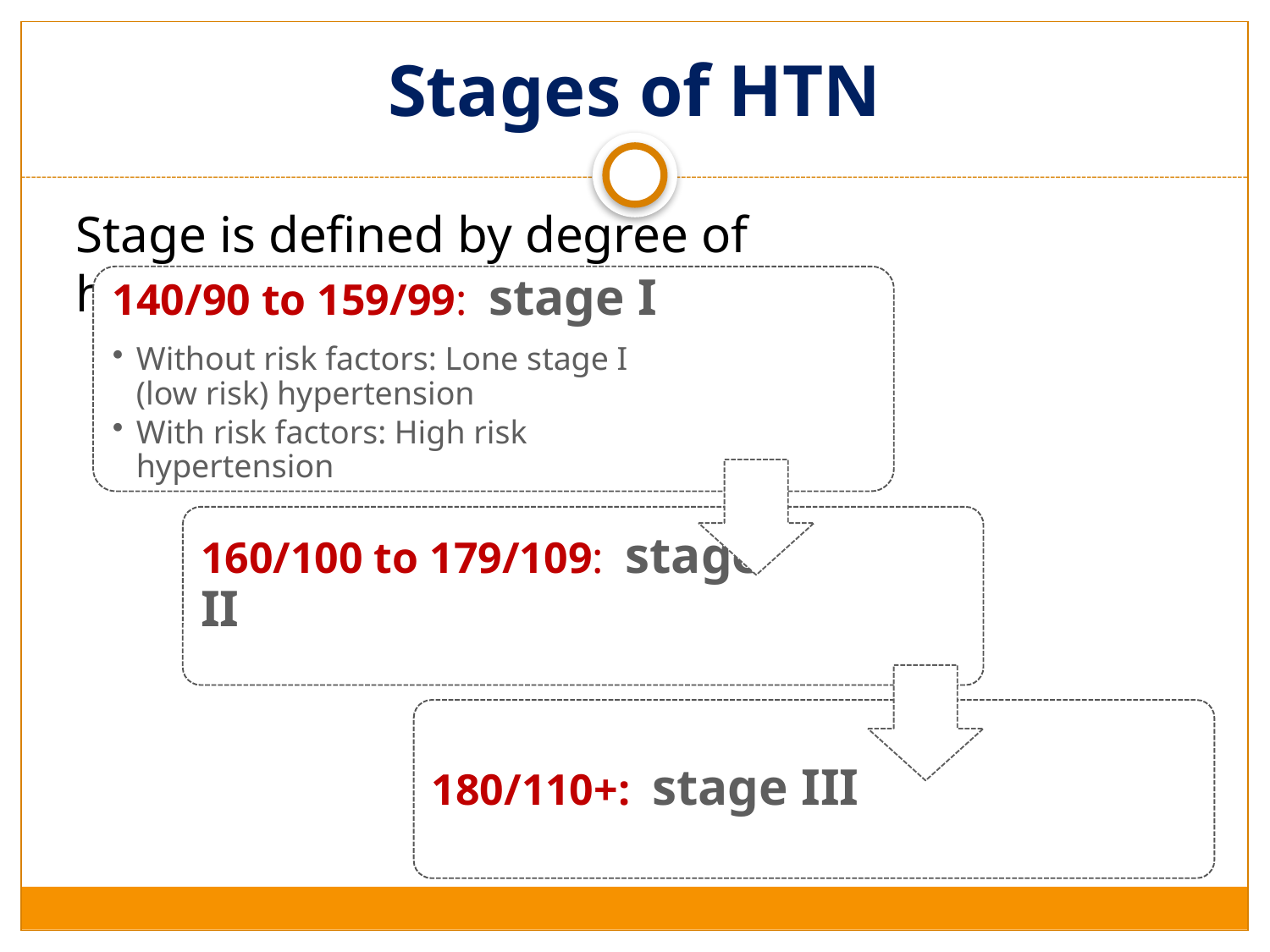

# Stages of HTN
Stage is defined by degree of hypertension
140/90 to 159/99: stage I
Without risk factors: Lone stage I (low risk) hypertension
With risk factors: High risk hypertension
160/100 to 179/109: stage II
180/110+: stage III

## Slide 19
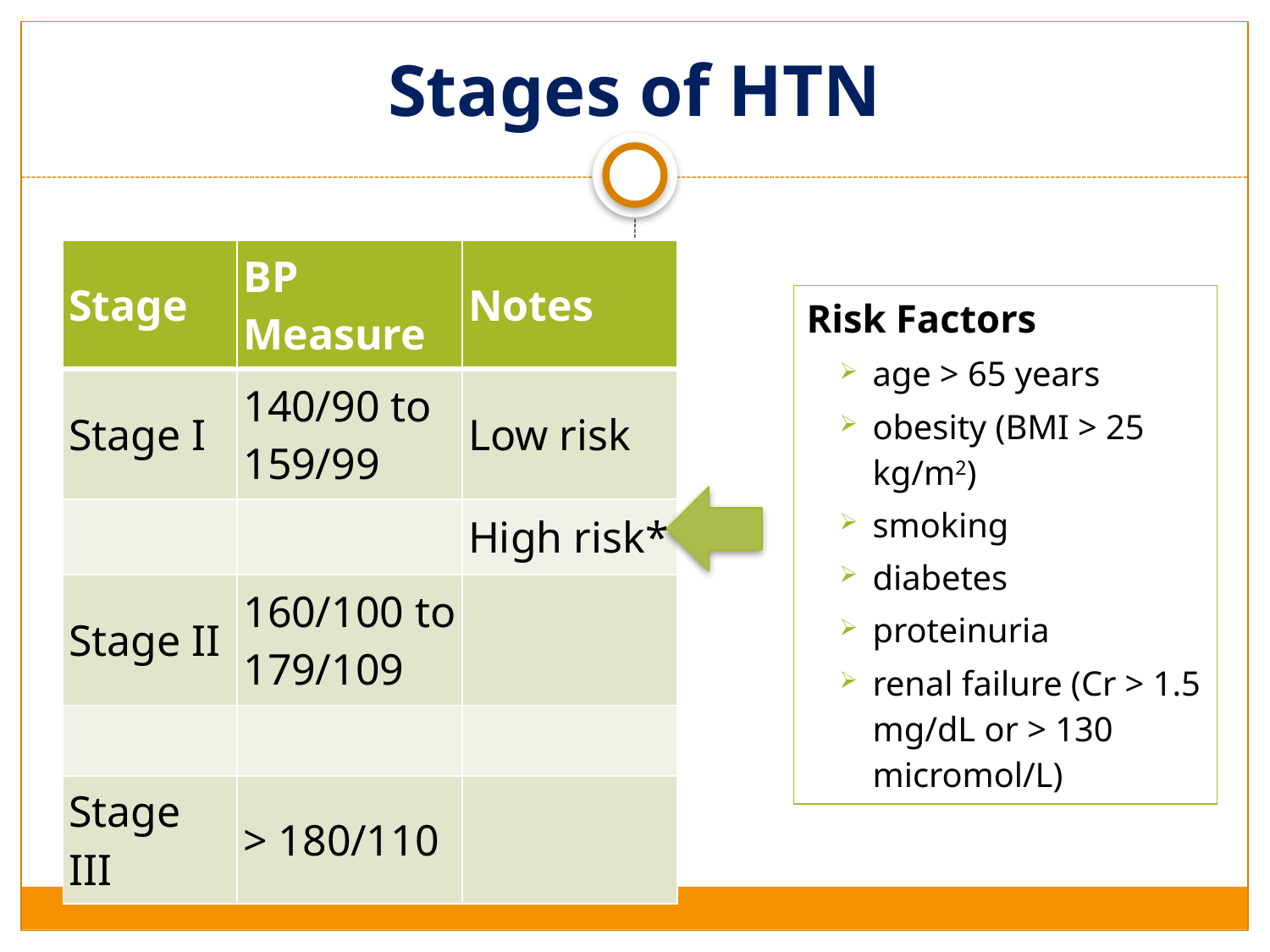

# Stages of HTN
| Stage | BP Measure | Notes |
| --- | --- | --- |
| Stage I | 140/90 to 159/99 | Low risk |
| | | High risk\* |
| Stage II | 160/100 to 179/109 | |
| | | |
| Stage III | > 180/110 | |
Risk Factors
age > 65 years
obesity (BMI > 25 kg/m2)
smoking
diabetes
proteinuria
renal failure (Cr > 1.5 mg/dL or > 130 micromol/L)

## Slide 20
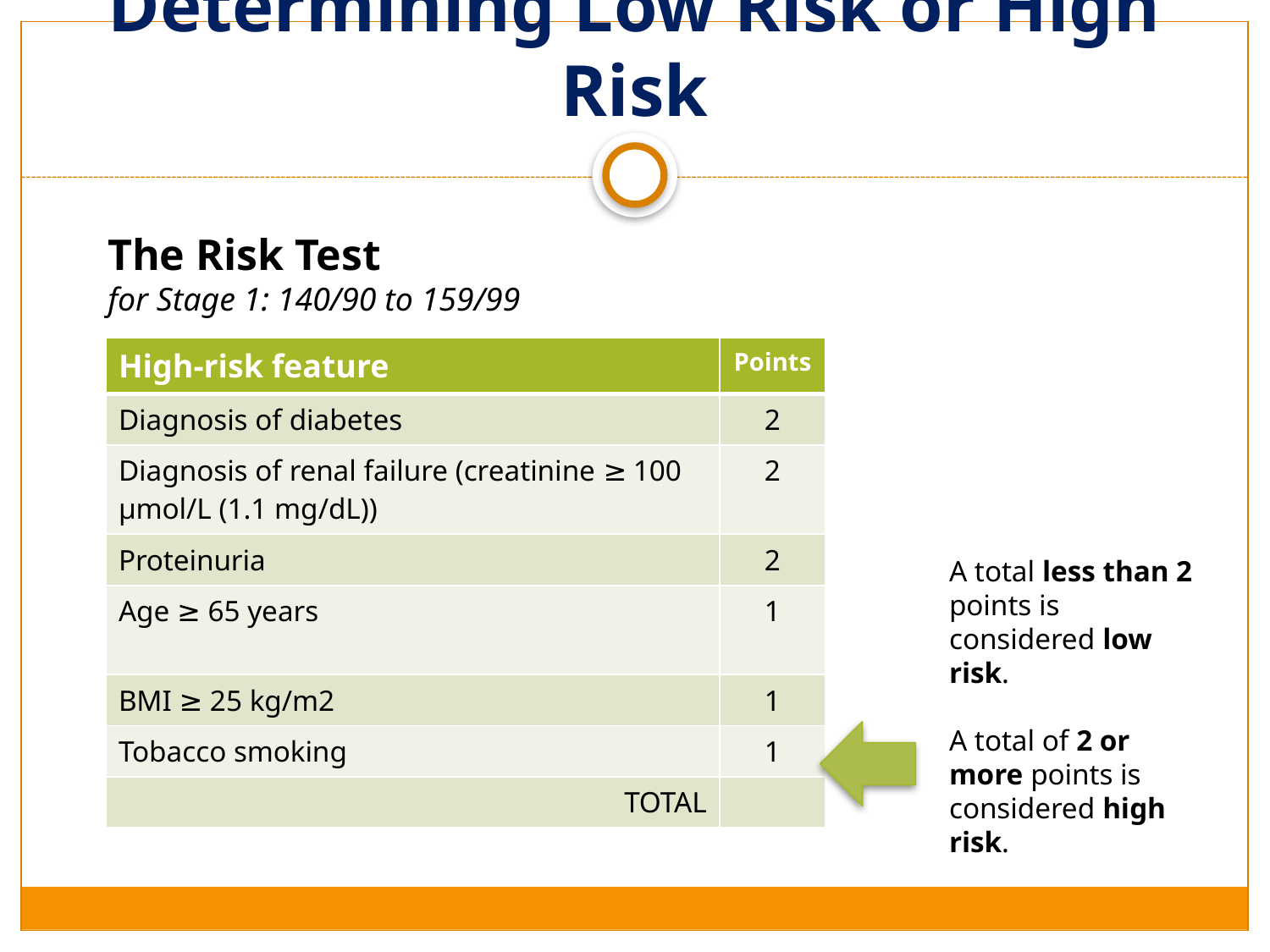

# Determining Low Risk or High Risk
The Risk Test
for Stage 1: 140/90 to 159/99
| High-risk feature | Points |
| --- | --- |
| Diagnosis of diabetes | 2 |
| Diagnosis of renal failure (creatinine ≥ 100 μmol/L (1.1 mg/dL)) | 2 |
| Proteinuria | 2 |
| Age ≥ 65 years | 1 |
| BMI ≥ 25 kg/m2 | 1 |
| Tobacco smoking | 1 |
| TOTAL | |
A total less than 2 points is considered low risk.
A total of 2 or more points is considered high risk.

## Slide 21
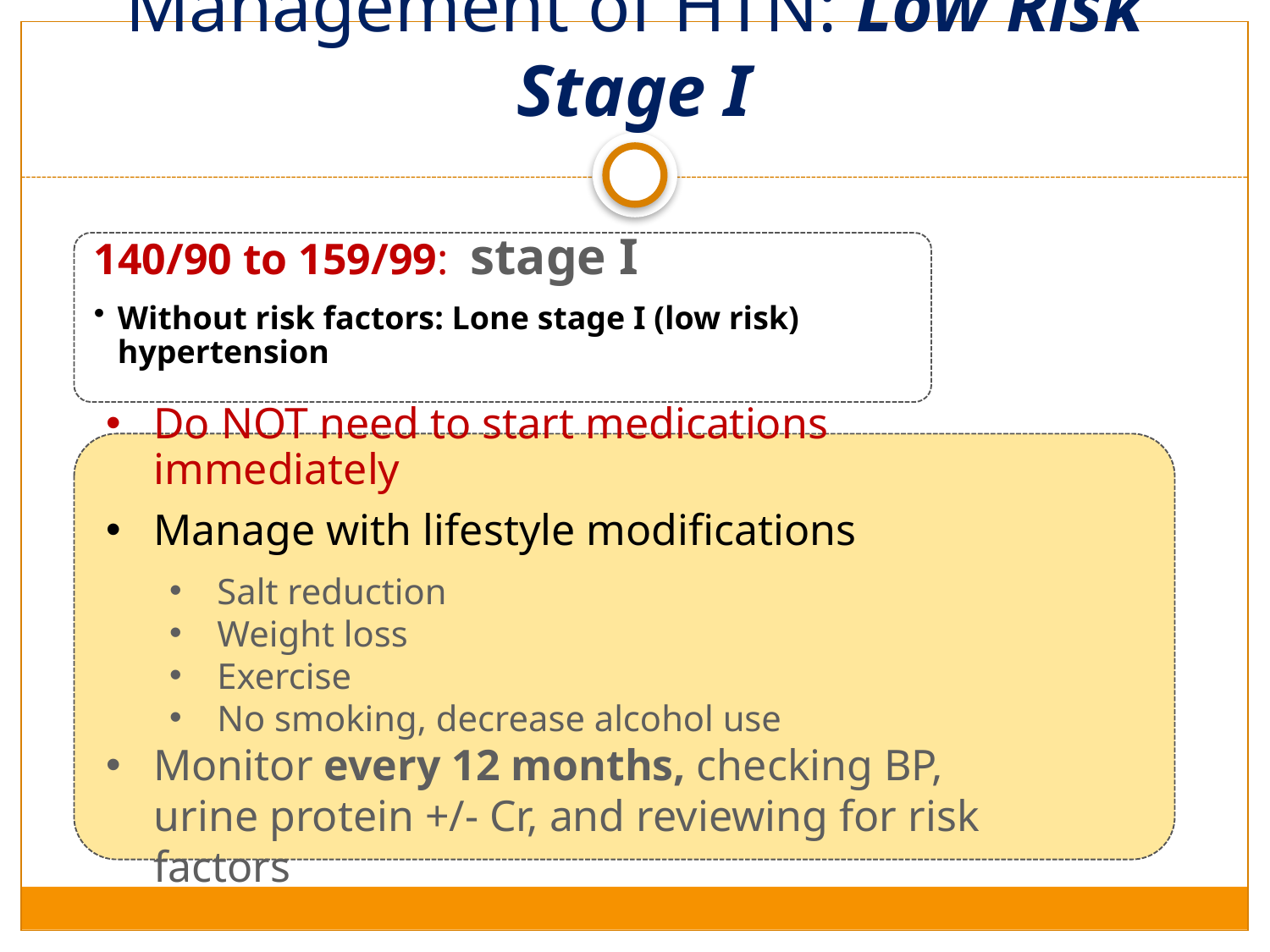

# Management of HTN: Low Risk Stage I
140/90 to 159/99: stage I
Without risk factors: Lone stage I (low risk) hypertension
Do NOT need to start medications immediately
Manage with lifestyle modifications
Salt reduction
Weight loss
Exercise
No smoking, decrease alcohol use
Monitor every 12 months, checking BP, urine protein +/- Cr, and reviewing for risk factors

## Slide 22
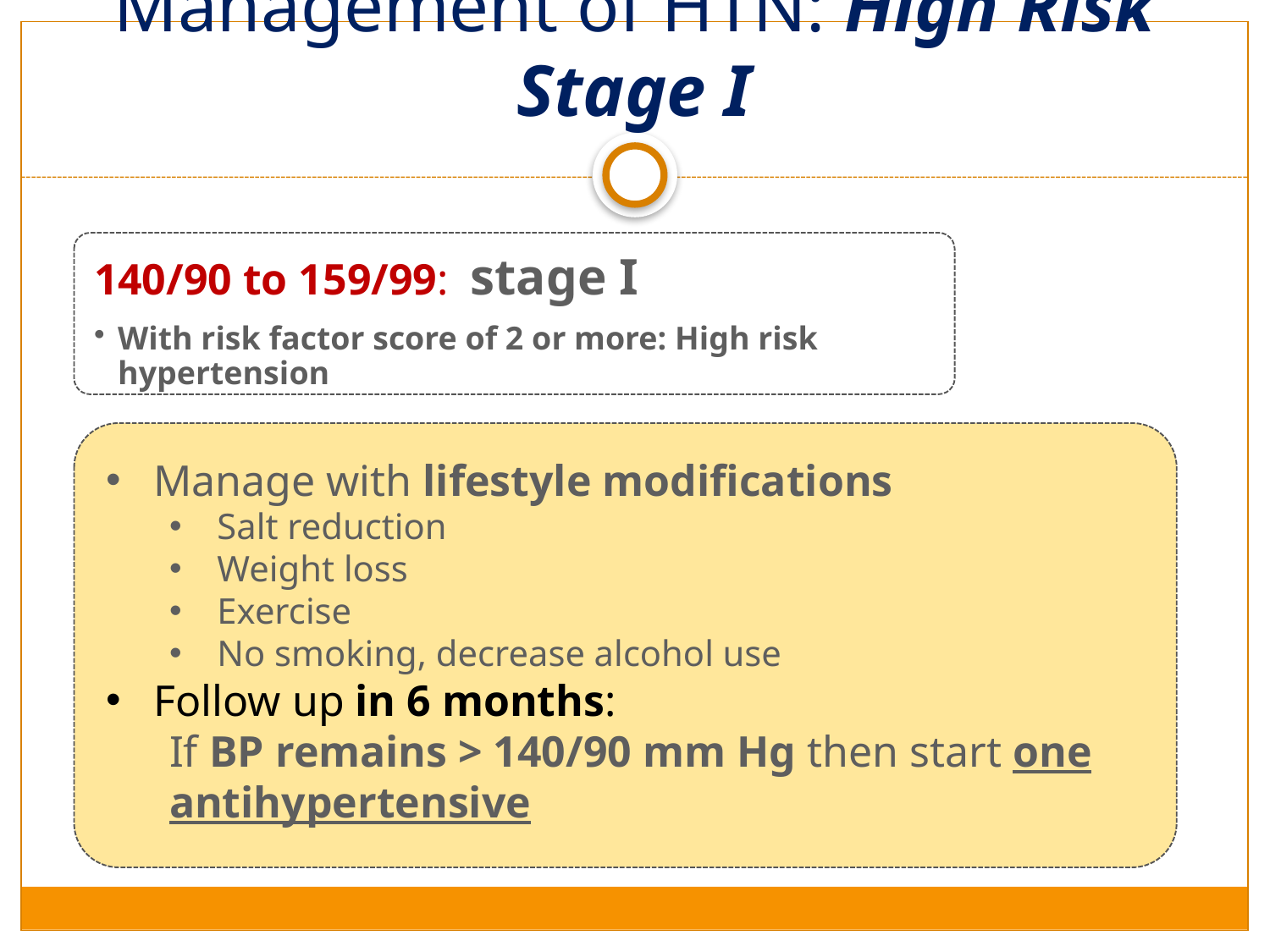

# Management of HTN: High Risk Stage I
140/90 to 159/99: stage I
With risk factor score of 2 or more: High risk hypertension
Manage with lifestyle modifications
Salt reduction
Weight loss
Exercise
No smoking, decrease alcohol use
Follow up in 6 months:
If BP remains > 140/90 mm Hg then start one antihypertensive

## Slide 23
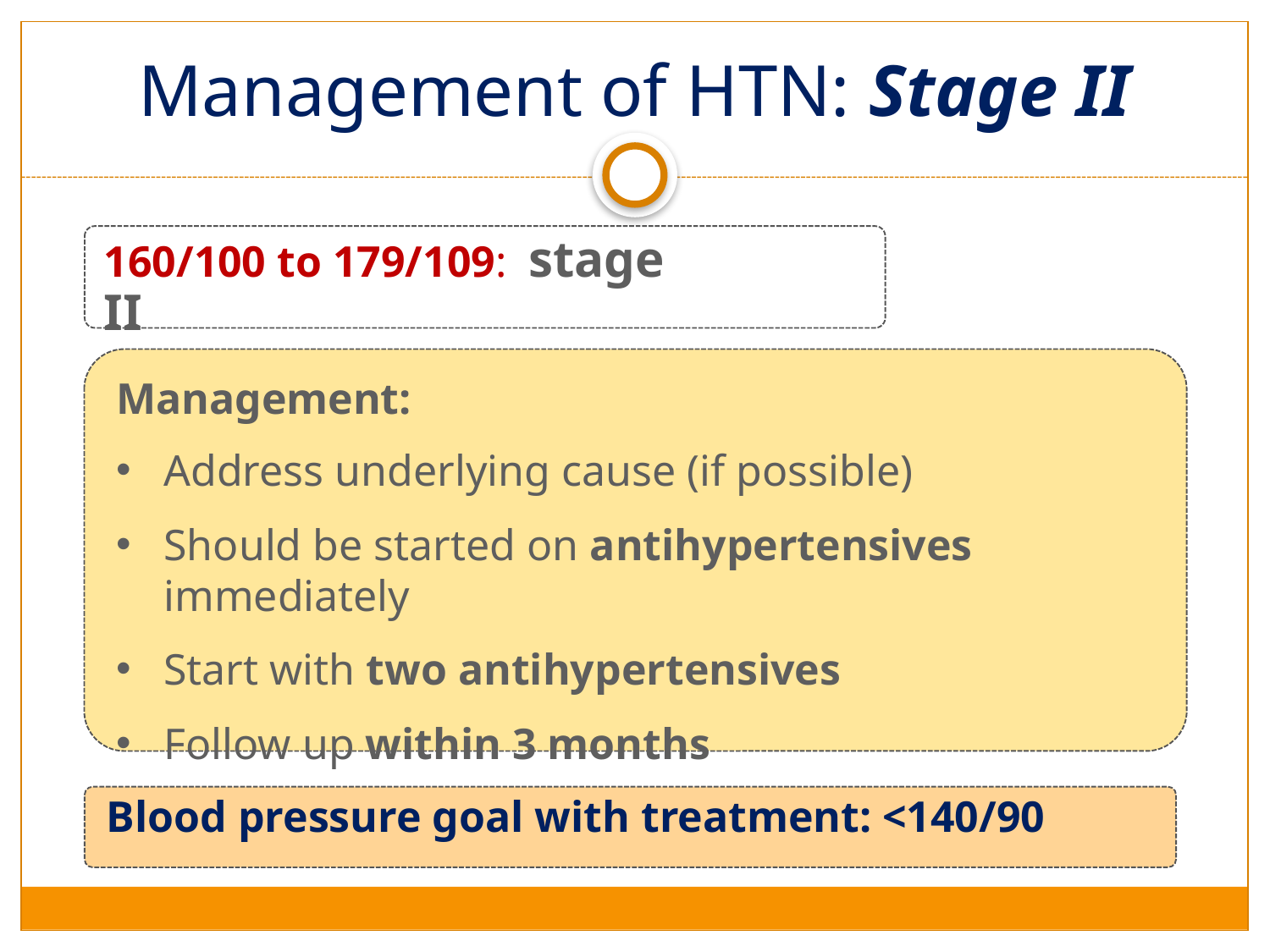

# Management of HTN: Stage II
160/100 to 179/109: stage II
Management:
Address underlying cause (if possible)
Should be started on antihypertensives immediately
Start with two antihypertensives
Follow up within 3 months
Blood pressure goal with treatment: <140/90

## Slide 24
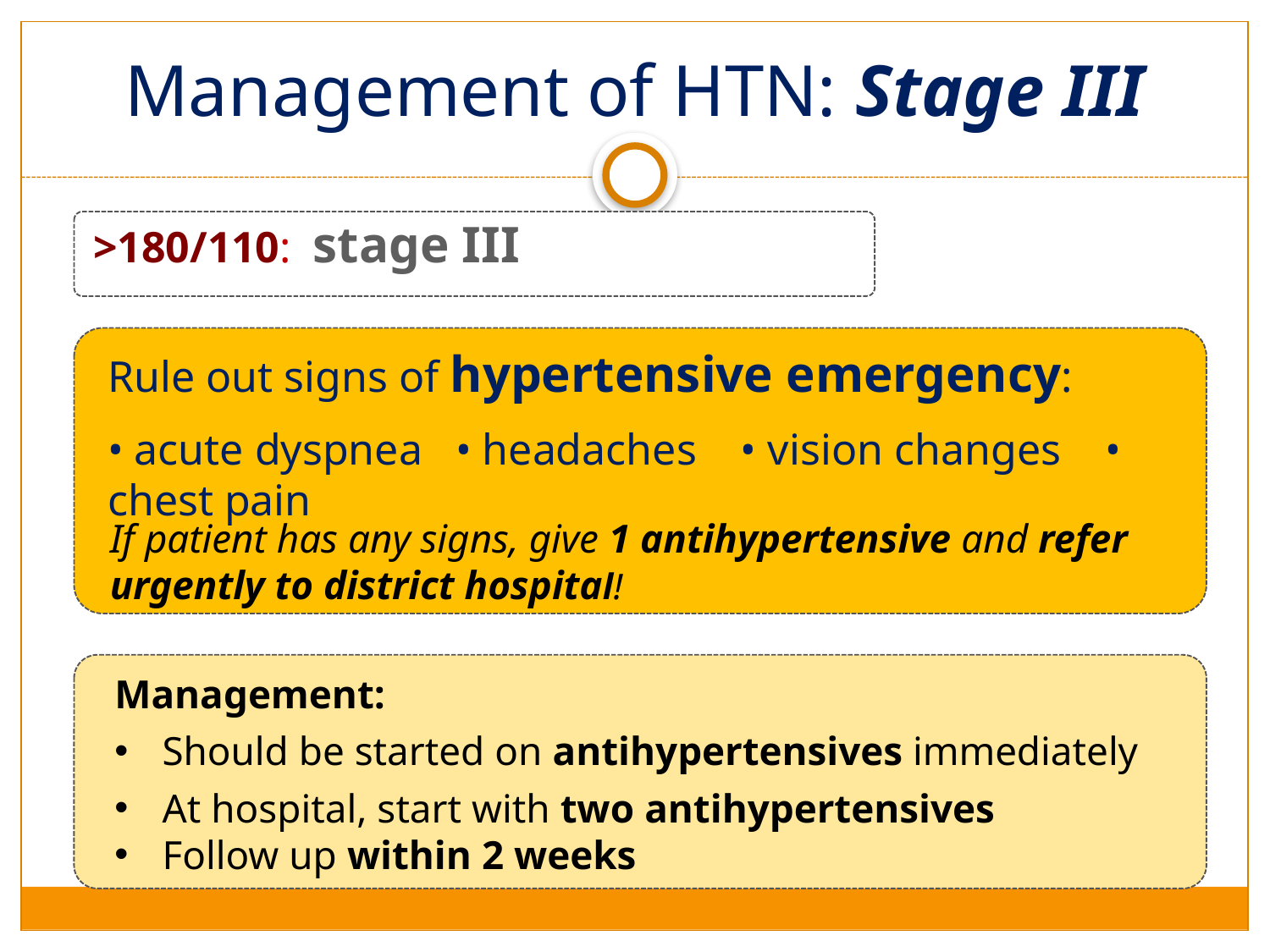

# Management of HTN: Stage III
>180/110: stage III
Rule out signs of hypertensive emergency:
• acute dyspnea • headaches • vision changes • chest pain
If patient has any signs, give 1 antihypertensive and refer urgently to district hospital!
Management:
Should be started on antihypertensives immediately
At hospital, start with two antihypertensives
Follow up within 2 weeks

## Slide 25
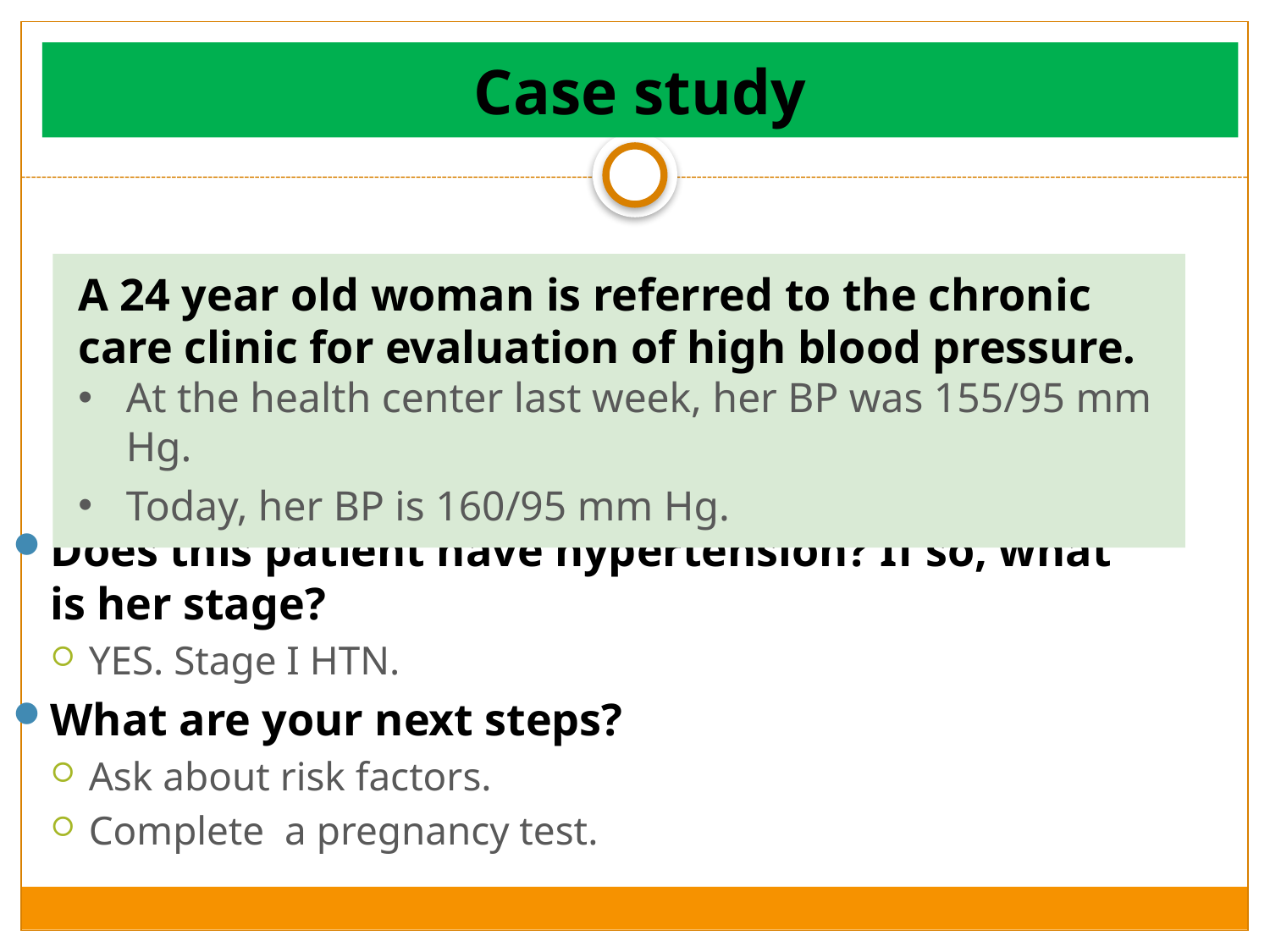

# CASE
Case study
A 24 year old woman is referred to the chronic care clinic for evaluation of high blood pressure.
At the health center last week, her BP was 155/95 mm Hg.
Today, her BP is 160/95 mm Hg.
Does this patient have hypertension? If so, what is her stage?
YES. Stage I HTN.
What are your next steps?
Ask about risk factors.
Complete a pregnancy test.

## Slide 26
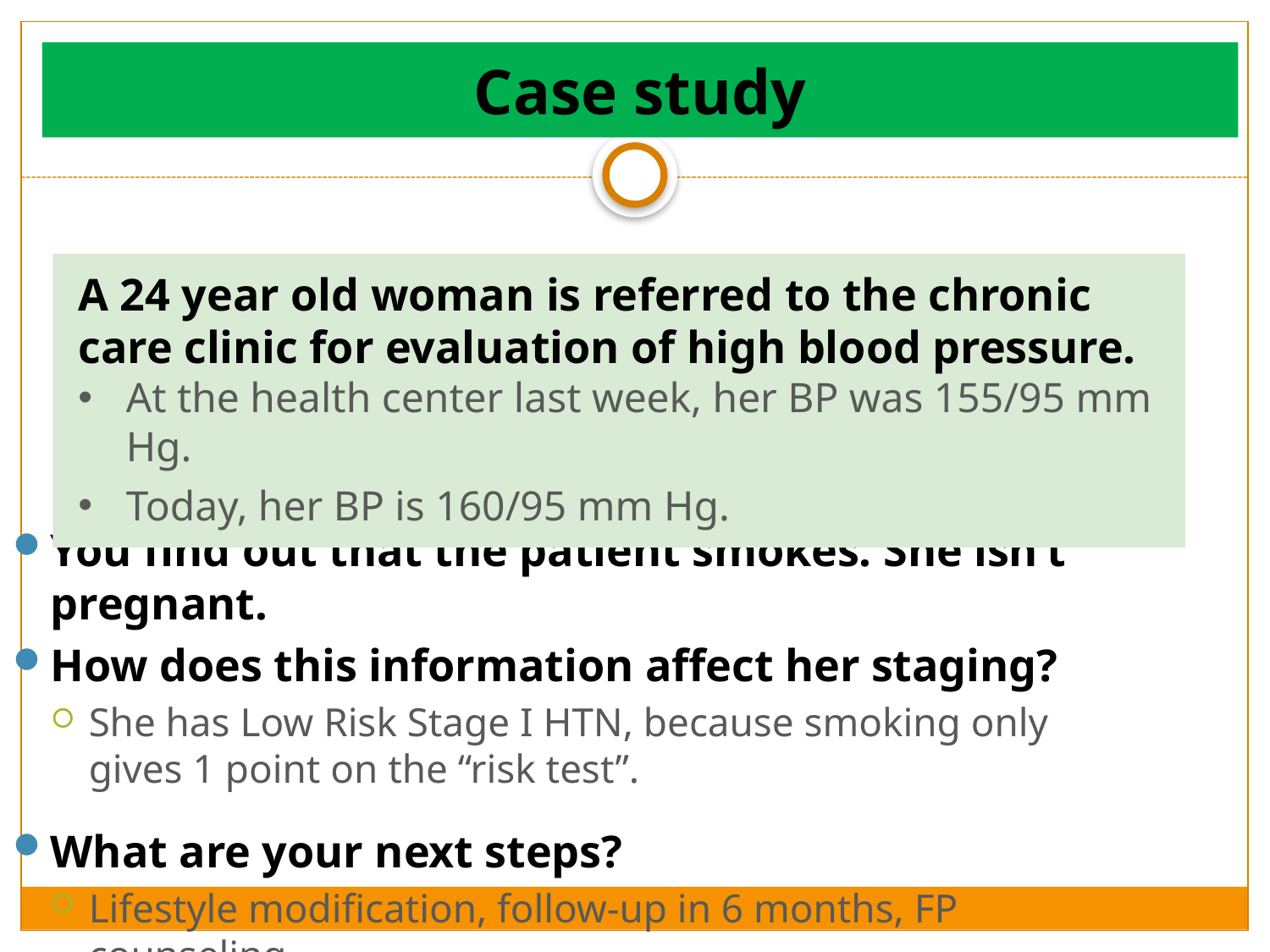

# CASE
Case study
A 24 year old woman is referred to the chronic care clinic for evaluation of high blood pressure.
At the health center last week, her BP was 155/95 mm Hg.
Today, her BP is 160/95 mm Hg.
You find out that the patient smokes. She isn’t pregnant.
How does this information affect her staging?
She has Low Risk Stage I HTN, because smoking only gives 1 point on the “risk test”.
What are your next steps?
Lifestyle modification, follow-up in 6 months, FP counseling.

## Slide 27
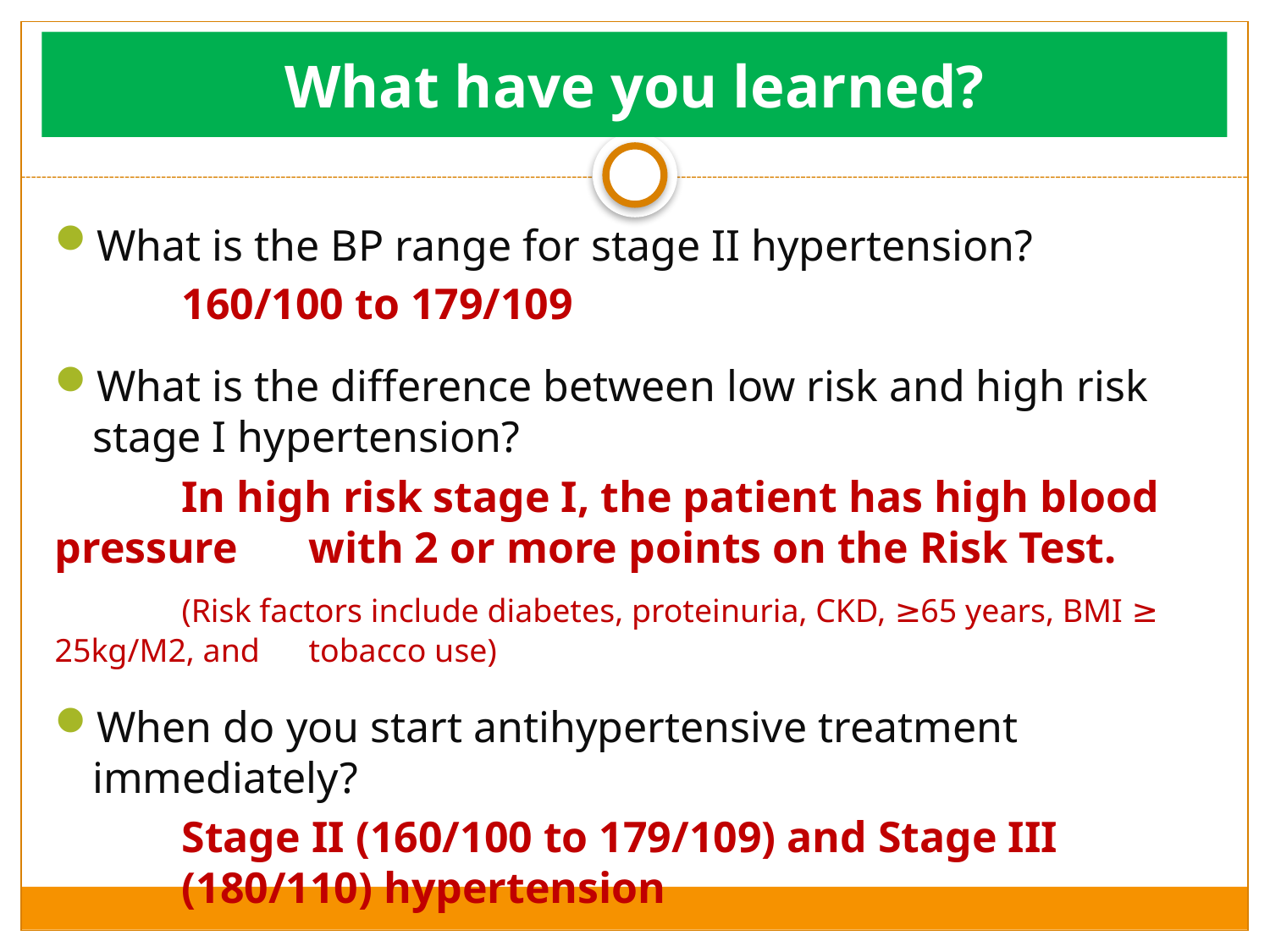

# What have you learned?
What is the BP range for stage II hypertension?
	160/100 to 179/109
What is the difference between low risk and high risk stage I hypertension?
	In high risk stage I, the patient has high blood pressure 	with 2 or more points on the Risk Test.
	(Risk factors include diabetes, proteinuria, CKD, ≥65 years, BMI ≥ 25kg/M2, and 	tobacco use)
When do you start antihypertensive treatment immediately?
	Stage II (160/100 to 179/109) and Stage III 			(180/110) hypertension

## Slide 28
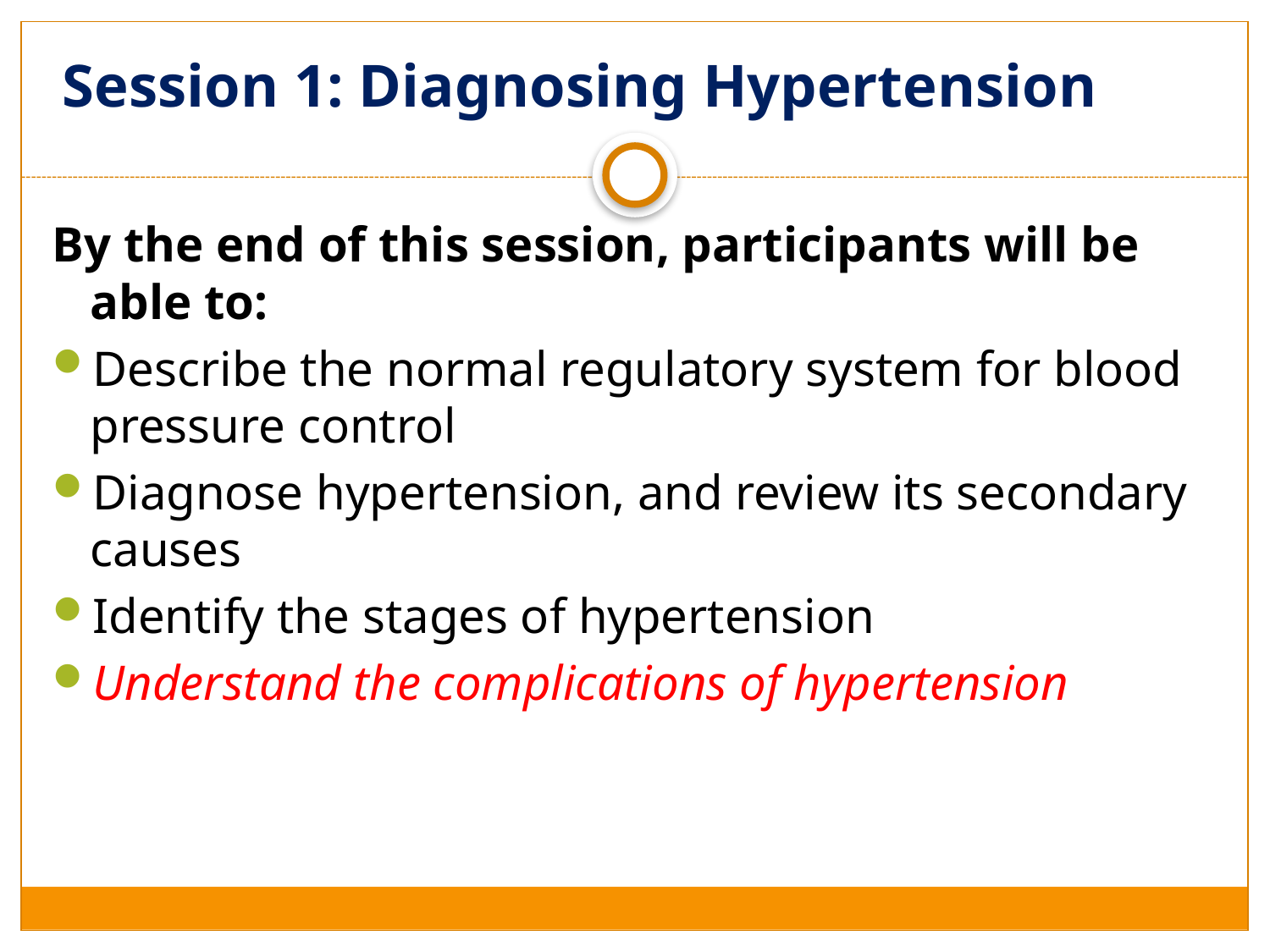

# Session 1: Diagnosing Hypertension
By the end of this session, participants will be able to:
Describe the normal regulatory system for blood pressure control
Diagnose hypertension, and review its secondary causes
Identify the stages of hypertension
Understand the complications of hypertension

## Slide 29
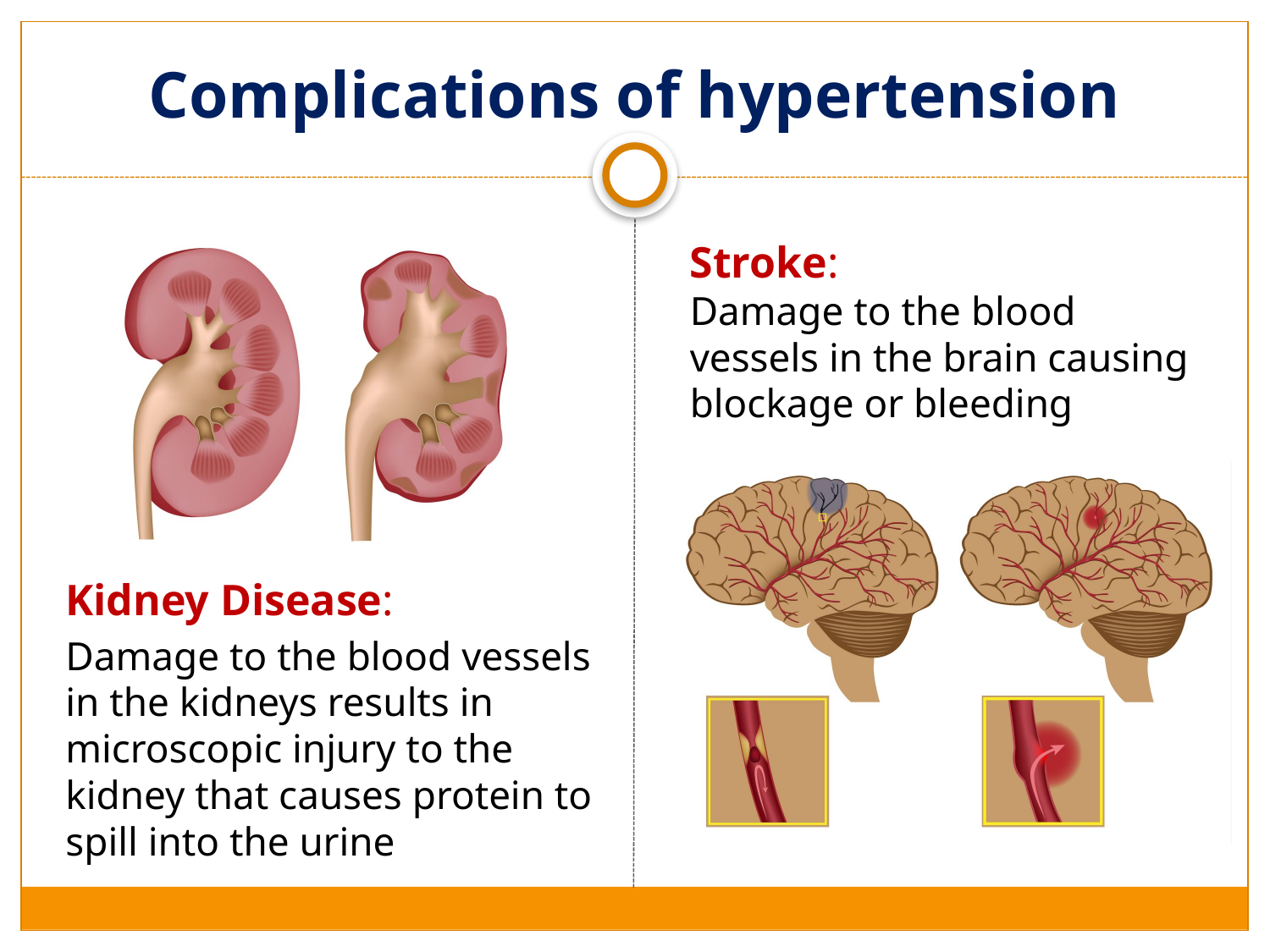

# Complications of hypertension
Stroke:
Damage to the blood vessels in the brain causing blockage or bleeding
Kidney Disease:
Damage to the blood vessels in the kidneys results in microscopic injury to the kidney that causes protein to spill into the urine

## Slide 30
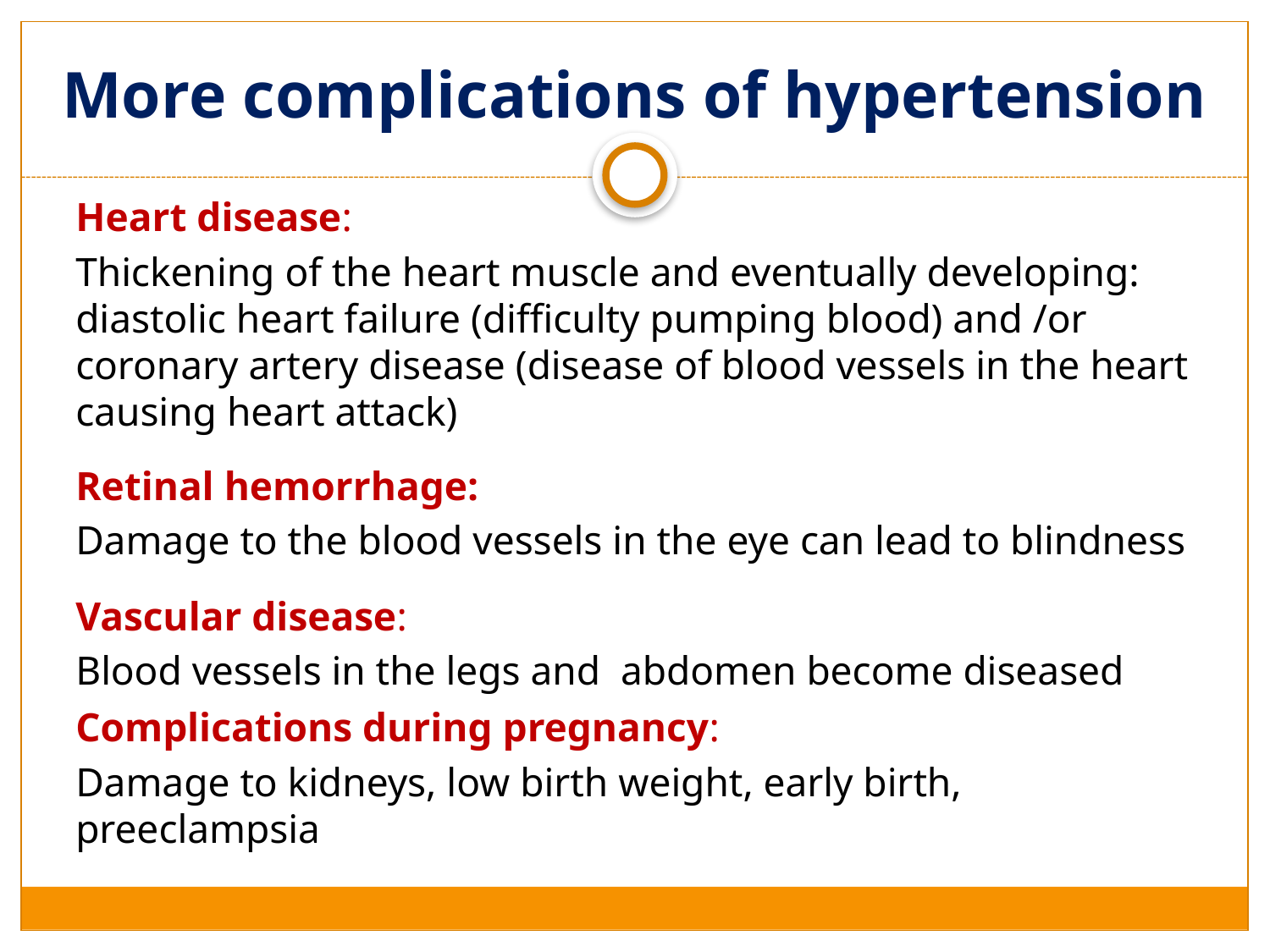

# More complications of hypertension
Heart disease:
Thickening of the heart muscle and eventually developing: diastolic heart failure (difficulty pumping blood) and /or coronary artery disease (disease of blood vessels in the heart causing heart attack)
Retinal hemorrhage:
Damage to the blood vessels in the eye can lead to blindness
Vascular disease:
Blood vessels in the legs and abdomen become diseased
Complications during pregnancy:
Damage to kidneys, low birth weight, early birth, preeclampsia

## Slide 31
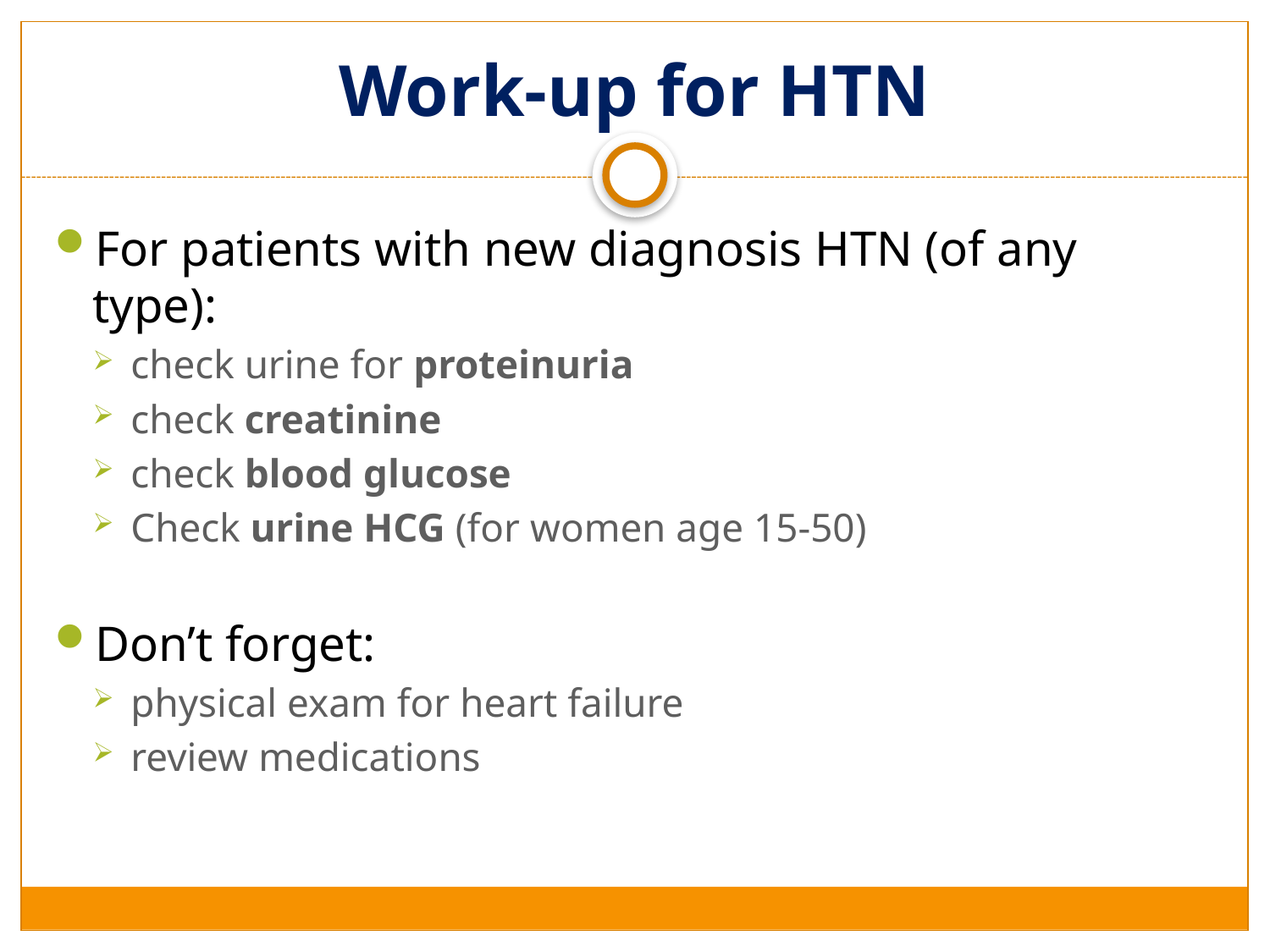

# Work-up for HTN
For patients with new diagnosis HTN (of any type):
check urine for proteinuria
check creatinine
check blood glucose
Check urine HCG (for women age 15-50)
Don’t forget:
physical exam for heart failure
review medications

## Slide 32
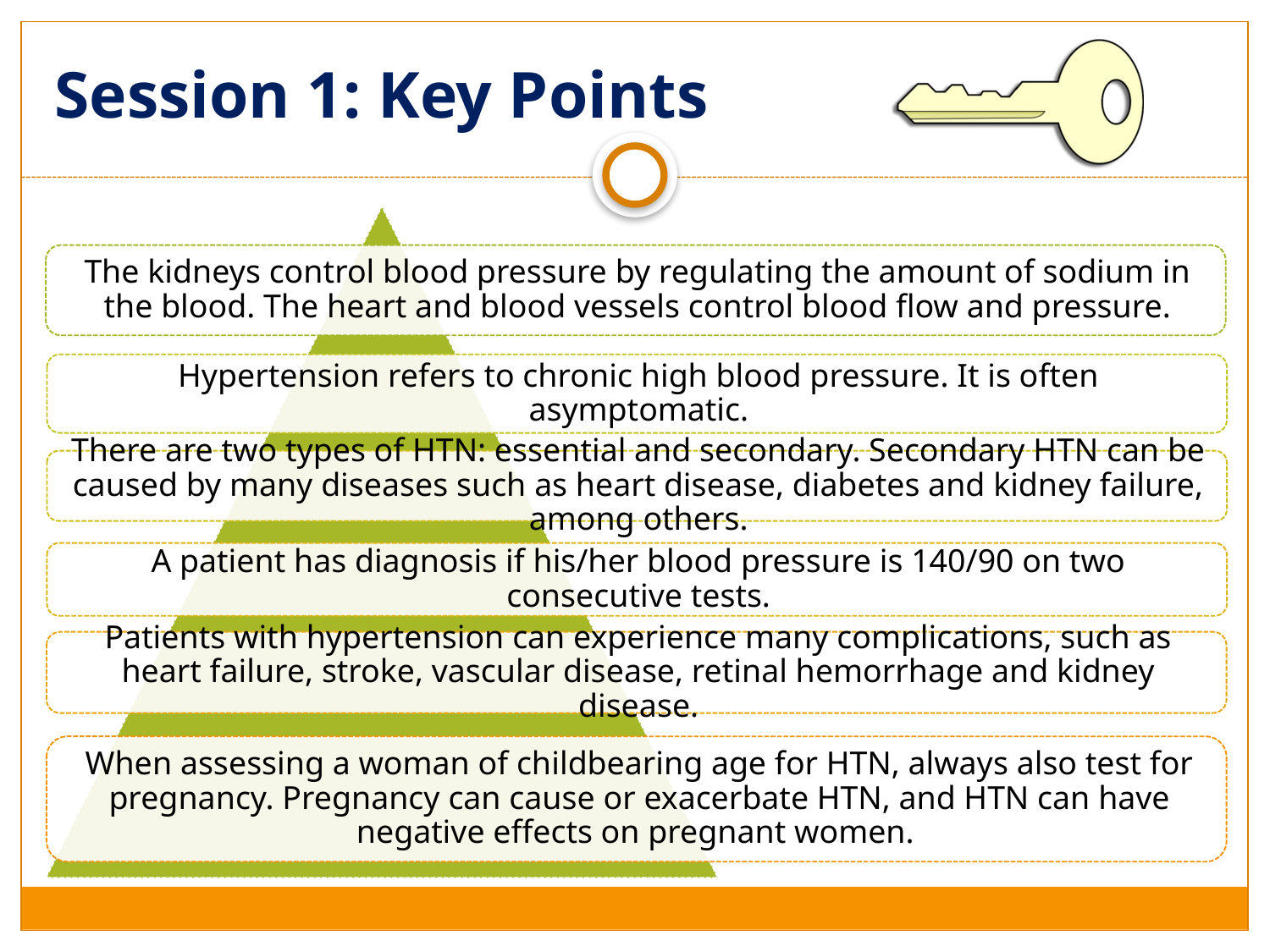

# Session 1: Key Points

## Slide 33
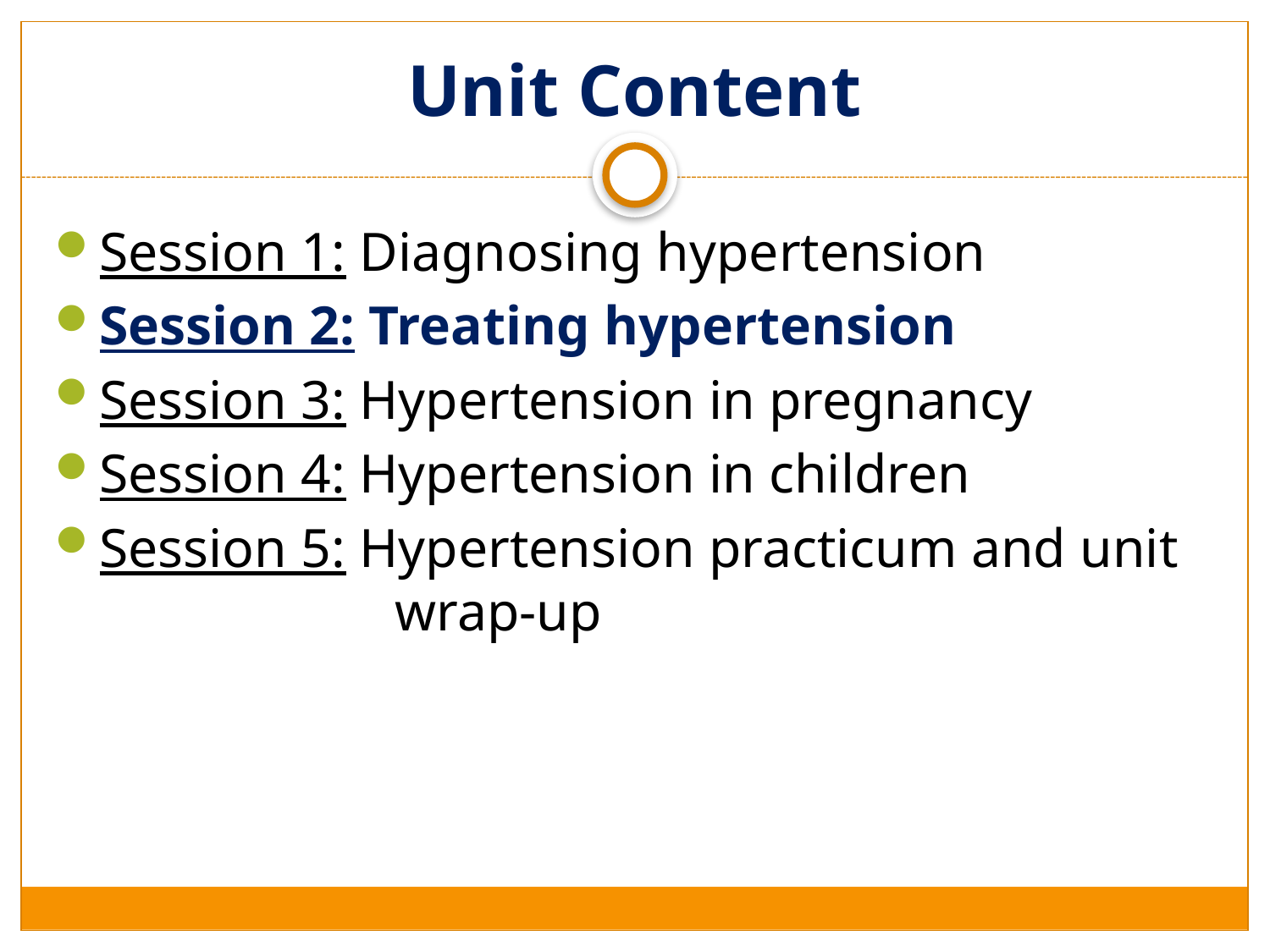

# Unit Content
Session 1: Diagnosing hypertension
Session 2: Treating hypertension
Session 3: Hypertension in pregnancy
Session 4: Hypertension in children
Session 5: Hypertension practicum and unit
 wrap-up

## Slide 34
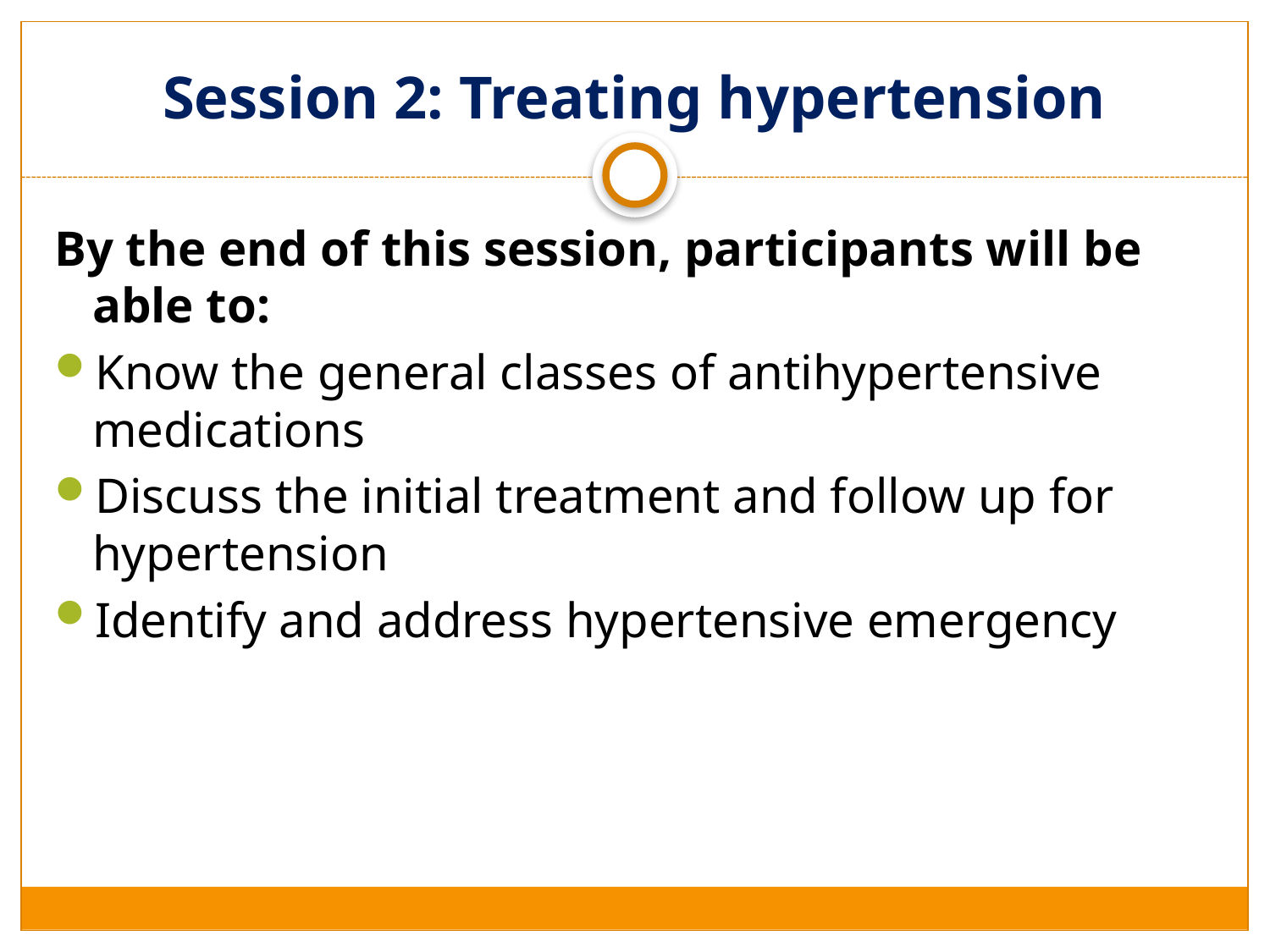

# Session 2: Treating hypertension
By the end of this session, participants will be able to:
Know the general classes of antihypertensive medications
Discuss the initial treatment and follow up for hypertension
Identify and address hypertensive emergency

## Slide 35
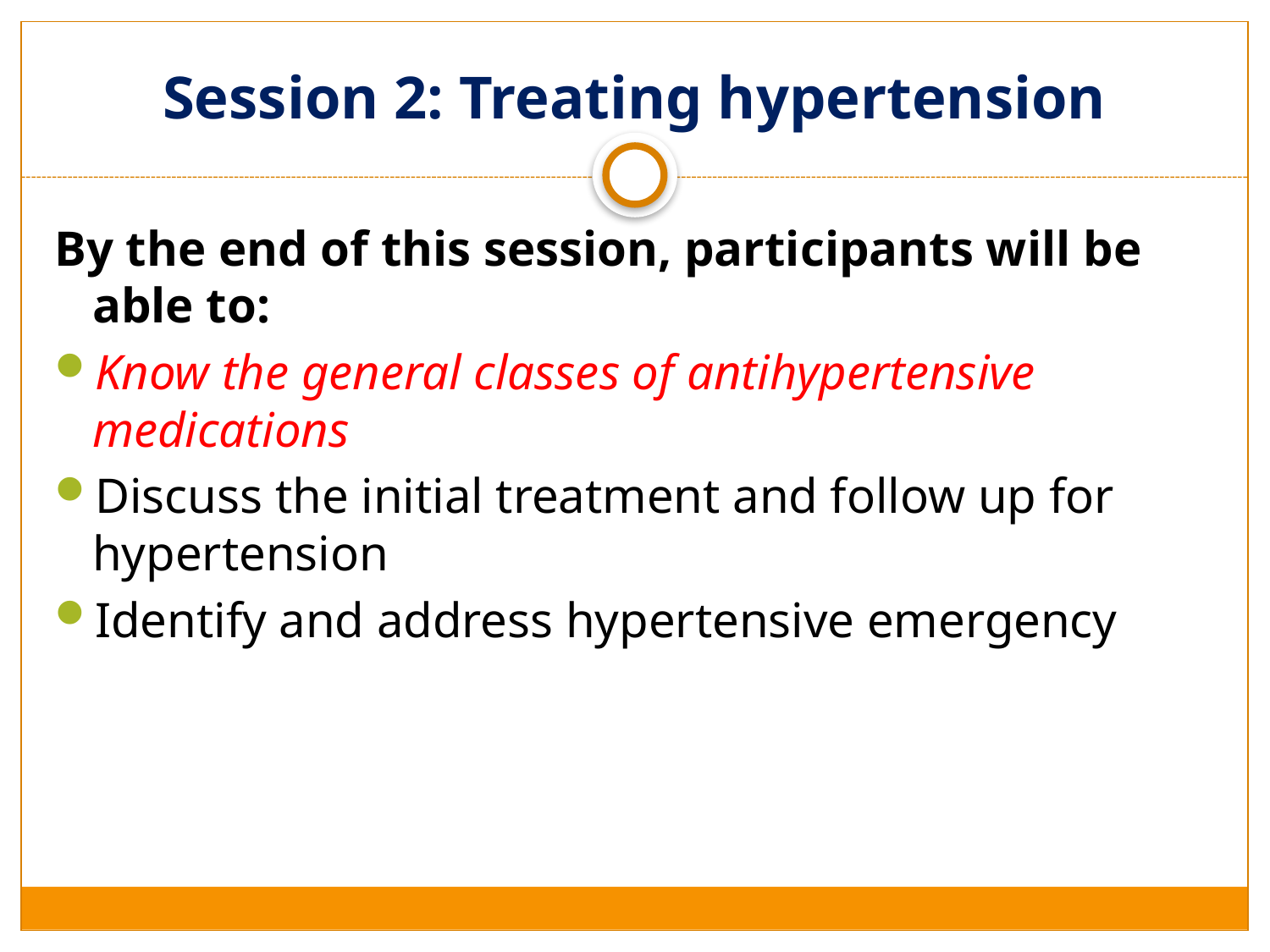

# Session 2: Treating hypertension
By the end of this session, participants will be able to:
Know the general classes of antihypertensive medications
Discuss the initial treatment and follow up for hypertension
Identify and address hypertensive emergency

## Slide 36
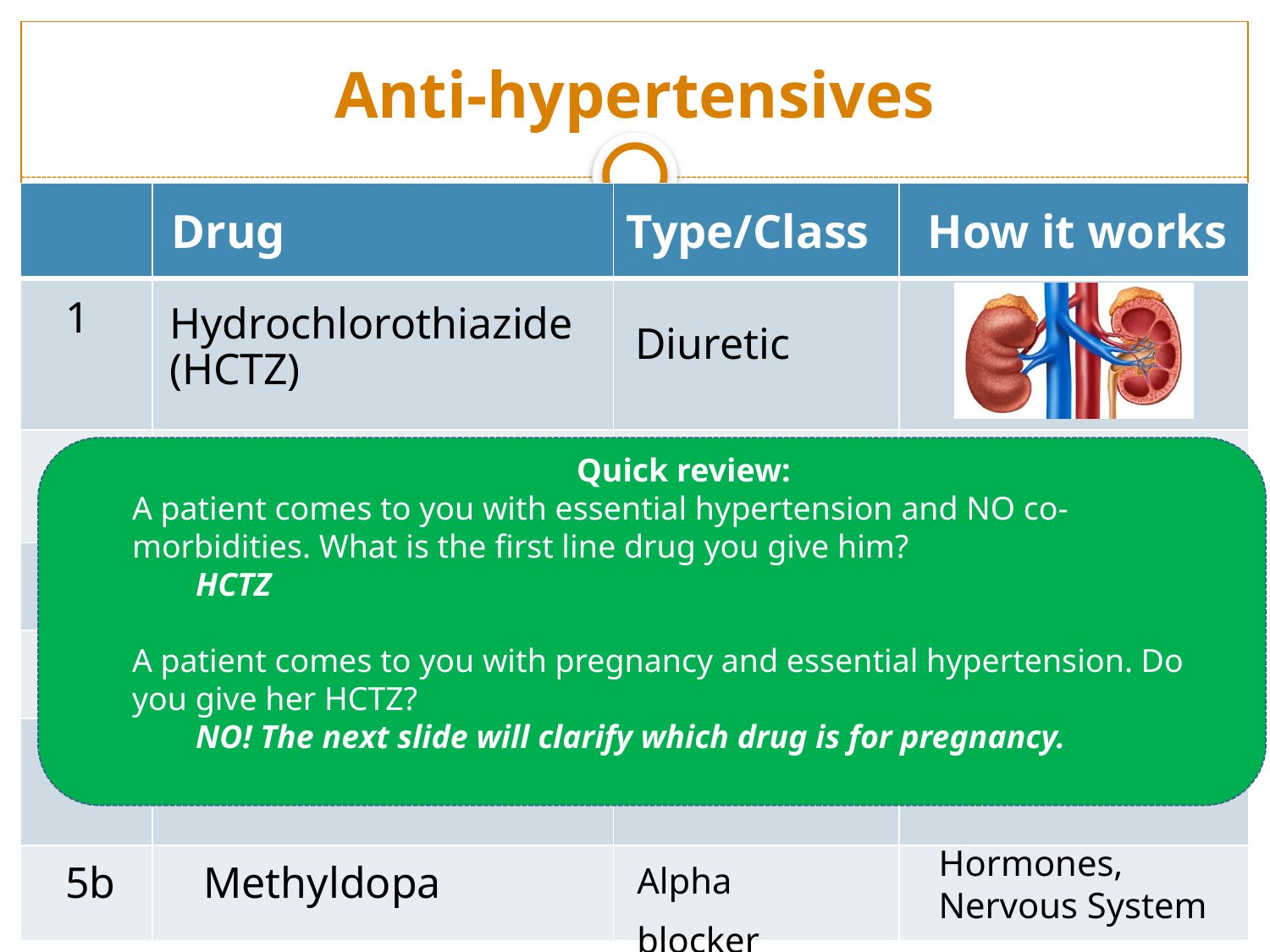

# Anti-hypertensives
Type/Class	 How it works
	Drug
| | | | |
| --- | --- | --- | --- |
| | | | |
| | | | |
| | | | |
| | | | |
| | | | |
| | | | |
1
Diuretic
Hydrochlorothiazide (HCTZ)
2 Amlodipine, Nifedipine
Calcium channel blocker
Quick review:
A patient comes to you with essential hypertension and NO co-morbidities. What is the first line drug you give him?
HCTZ
A patient comes to you with pregnancy and essential hypertension. Do you give her HCTZ?
NO! The next slide will clarify which drug is for pregnancy.
Blood Vessels
Renin-Angiotensin System
3 Lisinopril, captopril, enalapril
ACE inhibitor
4 Atenolol, Propranolol
Beta blocker
Heart, Blood Vessels
5a Hydralazine
Vasodilator
Blood Vessels
5b Methyldopa
Hormones,
Nervous System
Alpha blocker

## Slide 37
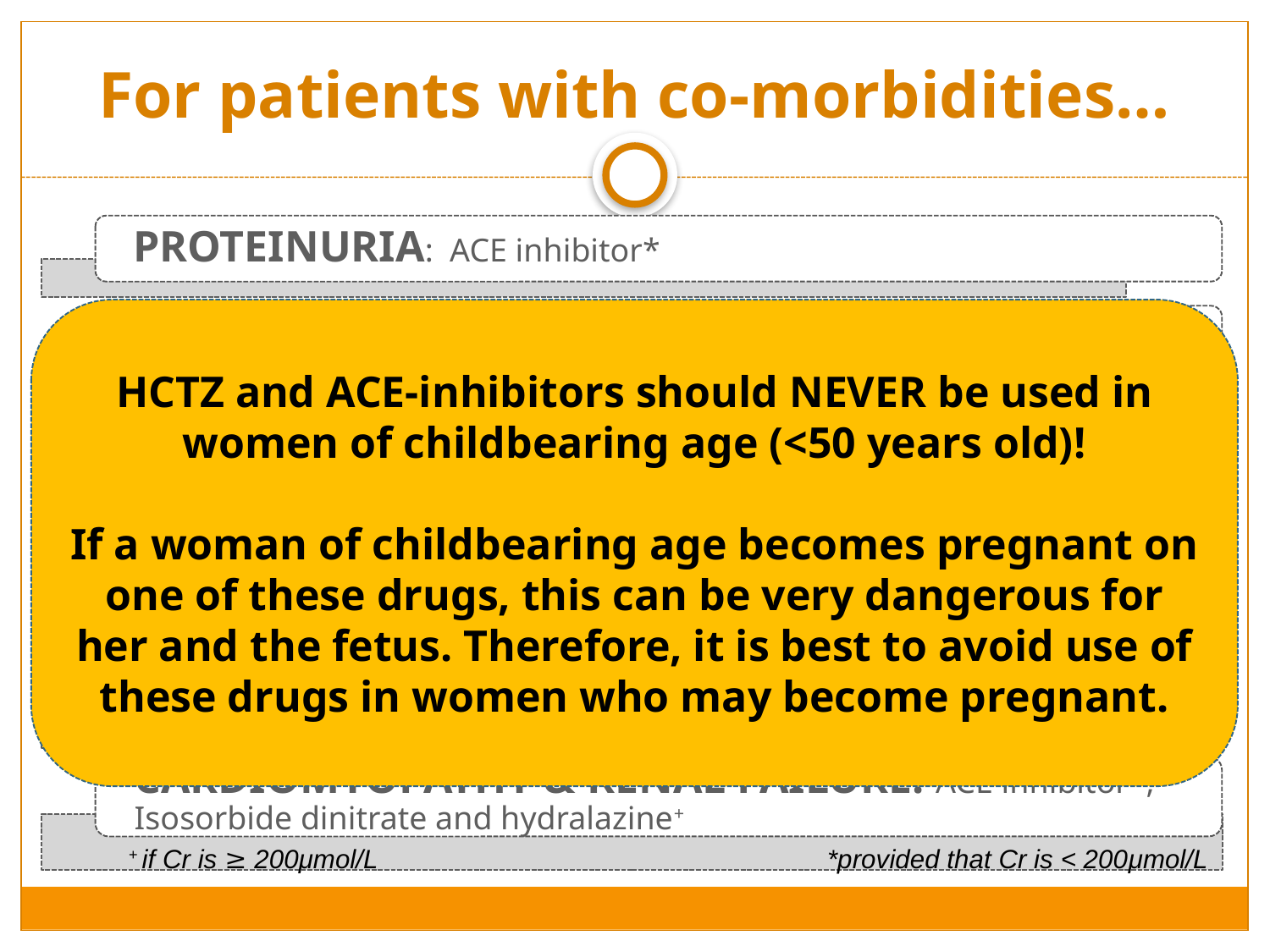

# For patients with co-morbidities…
HCTZ and ACE-inhibitors should NEVER be used in women of childbearing age (<50 years old)!
If a woman of childbearing age becomes pregnant on one of these drugs, this can be very dangerous for her and the fetus. Therefore, it is best to avoid use of these drugs in women who may become pregnant.
+ if Cr is ≥ 200μmol/L
*provided that Cr is < 200μmol/L

## Slide 38
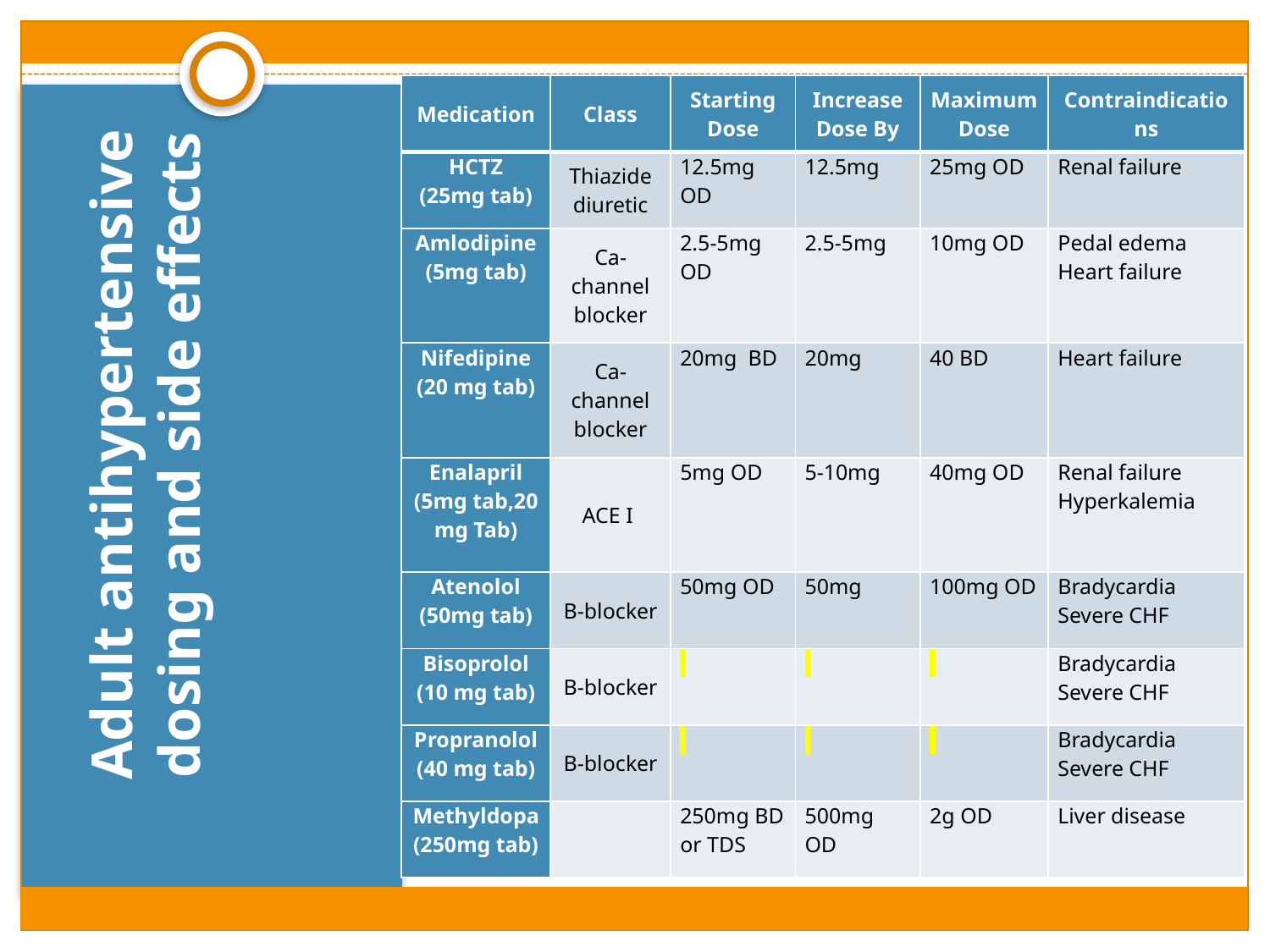

| Medication | Class | Starting Dose | Increase Dose By | Maximum Dose | Contraindications |
| --- | --- | --- | --- | --- | --- |
| HCTZ (25mg tab) | Thiazide diuretic | 12.5mg OD | 12.5mg | 25mg OD | Renal failure |
| Amlodipine (5mg tab) | Ca-channel blocker | 2.5-5mg OD | 2.5-5mg | 10mg OD | Pedal edema Heart failure |
| Nifedipine (20 mg tab) | Ca-channel blocker | 20mg BD | 20mg | 40 BD | Heart failure |
| Enalapril (5mg tab,20 mg Tab) | ACE I | 5mg OD | 5-10mg | 40mg OD | Renal failure Hyperkalemia |
| Atenolol (50mg tab) | Β-blocker | 50mg OD | 50mg | 100mg OD | Bradycardia Severe CHF |
| Bisoprolol (10 mg tab) | Β-blocker | | | | Bradycardia Severe CHF |
| Propranolol (40 mg tab) | Β-blocker | | | | Bradycardia Severe CHF |
| Methyldopa (250mg tab) | | 250mg BD or TDS | 500mg OD | 2g OD | Liver disease |
Adult antihypertensive dosing and side effects

## Slide 39
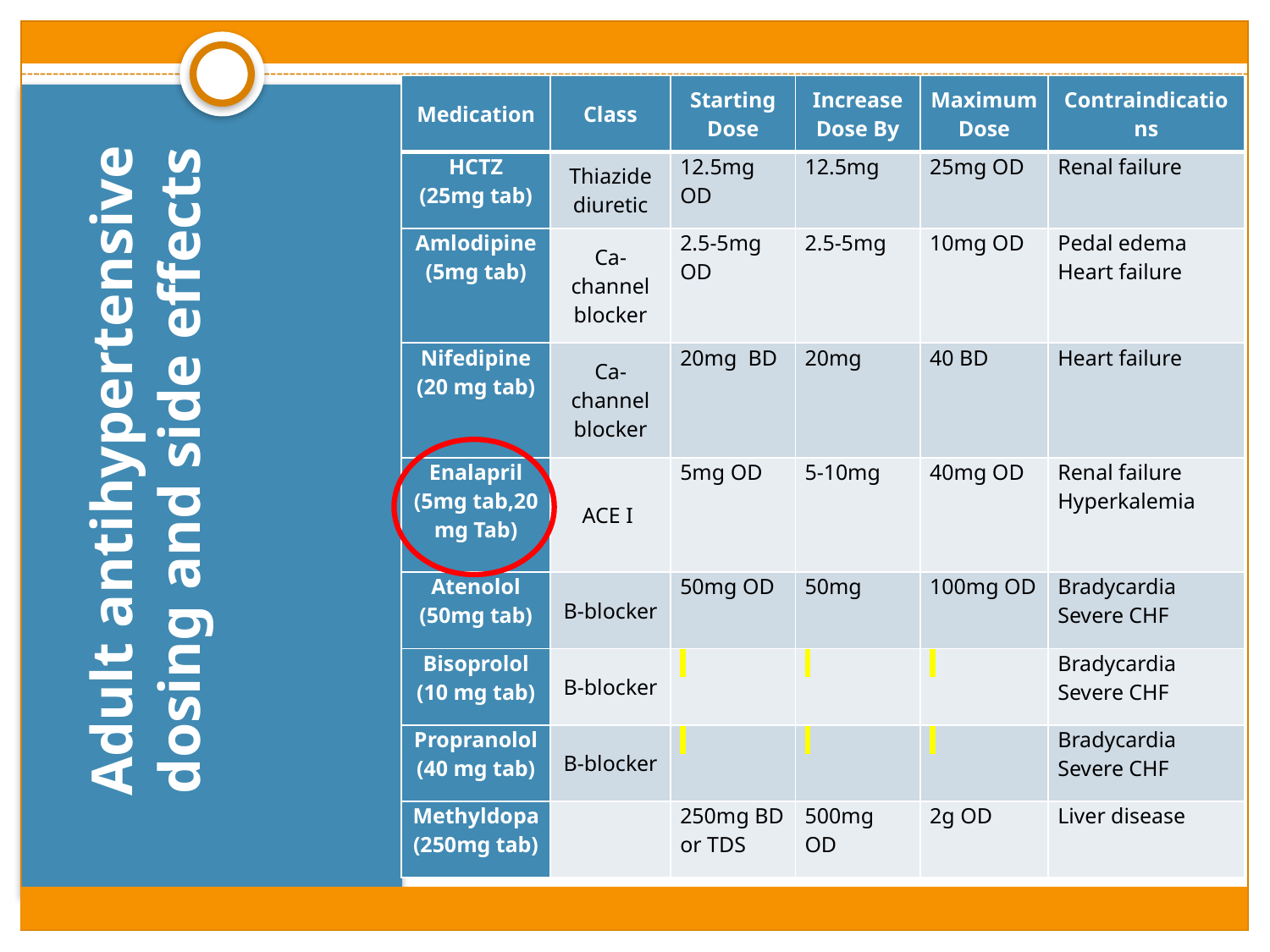

| Medication | Class | Starting Dose | Increase Dose By | Maximum Dose | Contraindications |
| --- | --- | --- | --- | --- | --- |
| HCTZ (25mg tab) | Thiazide diuretic | 12.5mg OD | 12.5mg | 25mg OD | Renal failure |
| Amlodipine (5mg tab) | Ca-channel blocker | 2.5-5mg OD | 2.5-5mg | 10mg OD | Pedal edema Heart failure |
| Nifedipine (20 mg tab) | Ca-channel blocker | 20mg BD | 20mg | 40 BD | Heart failure |
| Enalapril (5mg tab,20 mg Tab) | ACE I | 5mg OD | 5-10mg | 40mg OD | Renal failure Hyperkalemia |
| Atenolol (50mg tab) | Β-blocker | 50mg OD | 50mg | 100mg OD | Bradycardia Severe CHF |
| Bisoprolol (10 mg tab) | Β-blocker | | | | Bradycardia Severe CHF |
| Propranolol (40 mg tab) | Β-blocker | | | | Bradycardia Severe CHF |
| Methyldopa (250mg tab) | | 250mg BD or TDS | 500mg OD | 2g OD | Liver disease |
Adult antihypertensive dosing and side effects

## Slide 40
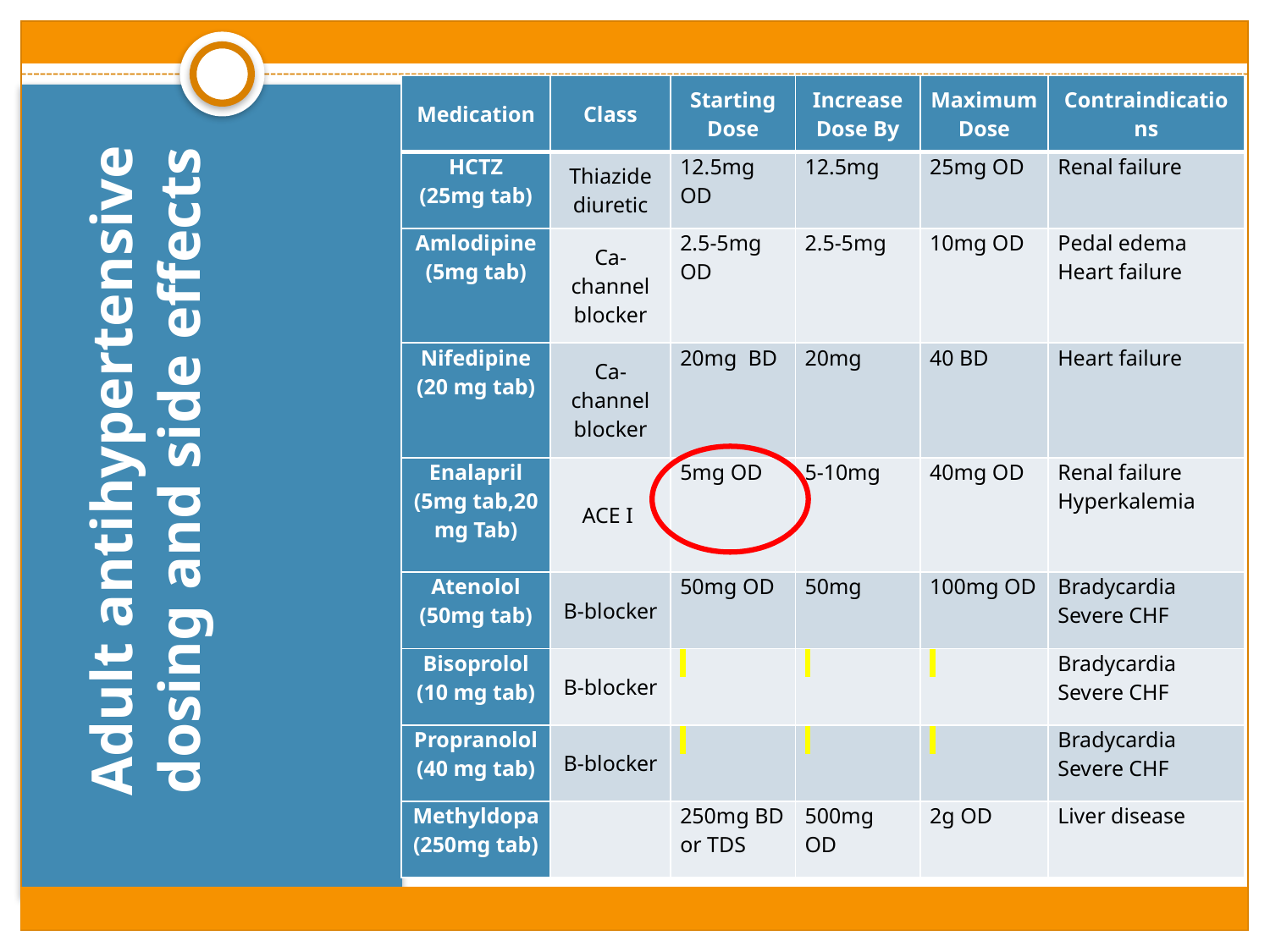

| Medication | Class | Starting Dose | Increase Dose By | Maximum Dose | Contraindications |
| --- | --- | --- | --- | --- | --- |
| HCTZ (25mg tab) | Thiazide diuretic | 12.5mg OD | 12.5mg | 25mg OD | Renal failure |
| Amlodipine (5mg tab) | Ca-channel blocker | 2.5-5mg OD | 2.5-5mg | 10mg OD | Pedal edema Heart failure |
| Nifedipine (20 mg tab) | Ca-channel blocker | 20mg BD | 20mg | 40 BD | Heart failure |
| Enalapril (5mg tab,20 mg Tab) | ACE I | 5mg OD | 5-10mg | 40mg OD | Renal failure Hyperkalemia |
| Atenolol (50mg tab) | Β-blocker | 50mg OD | 50mg | 100mg OD | Bradycardia Severe CHF |
| Bisoprolol (10 mg tab) | Β-blocker | | | | Bradycardia Severe CHF |
| Propranolol (40 mg tab) | Β-blocker | | | | Bradycardia Severe CHF |
| Methyldopa (250mg tab) | | 250mg BD or TDS | 500mg OD | 2g OD | Liver disease |
Adult antihypertensive dosing and side effects

## Slide 41
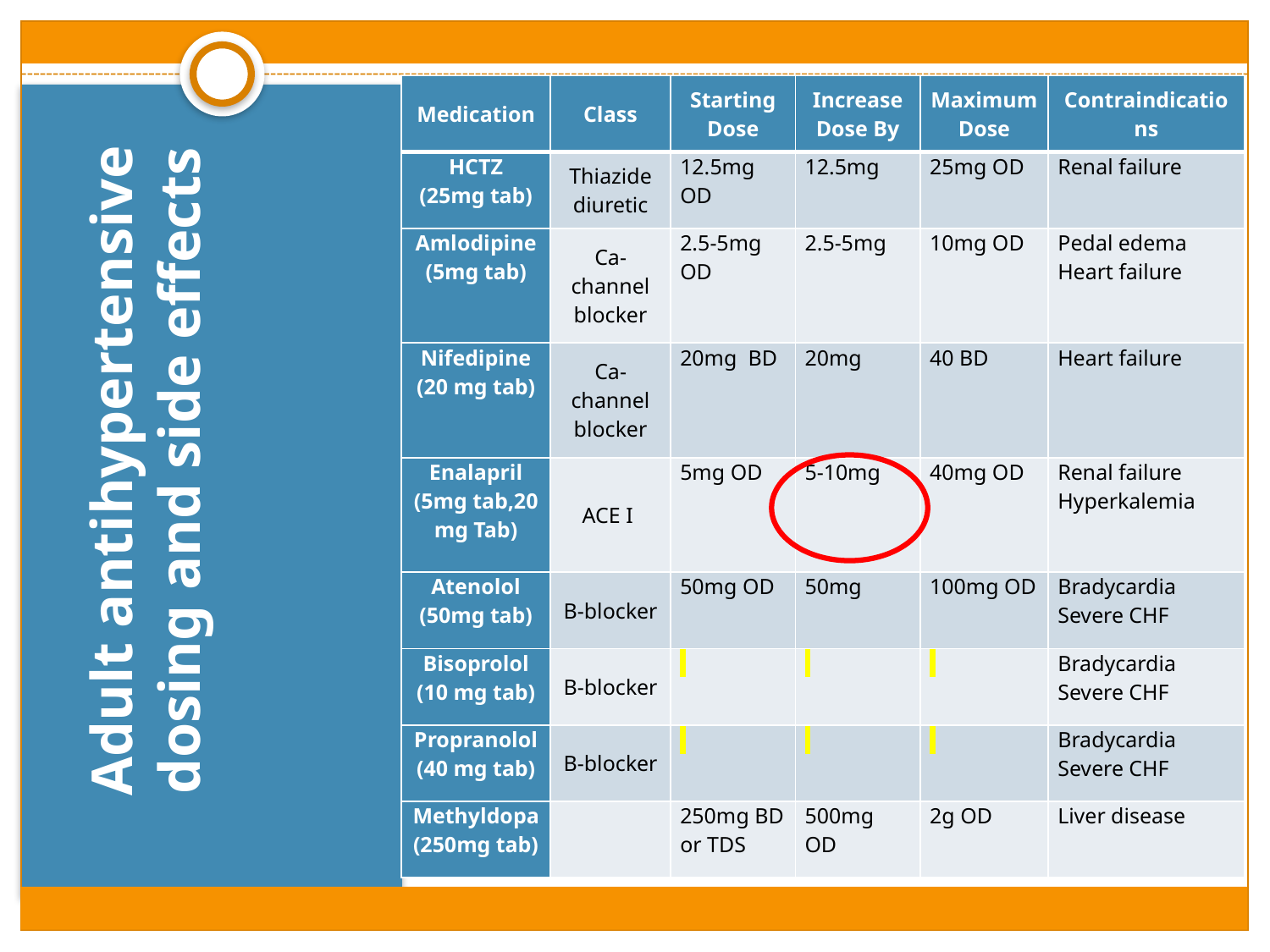

| Medication | Class | Starting Dose | Increase Dose By | Maximum Dose | Contraindications |
| --- | --- | --- | --- | --- | --- |
| HCTZ (25mg tab) | Thiazide diuretic | 12.5mg OD | 12.5mg | 25mg OD | Renal failure |
| Amlodipine (5mg tab) | Ca-channel blocker | 2.5-5mg OD | 2.5-5mg | 10mg OD | Pedal edema Heart failure |
| Nifedipine (20 mg tab) | Ca-channel blocker | 20mg BD | 20mg | 40 BD | Heart failure |
| Enalapril (5mg tab,20 mg Tab) | ACE I | 5mg OD | 5-10mg | 40mg OD | Renal failure Hyperkalemia |
| Atenolol (50mg tab) | Β-blocker | 50mg OD | 50mg | 100mg OD | Bradycardia Severe CHF |
| Bisoprolol (10 mg tab) | Β-blocker | | | | Bradycardia Severe CHF |
| Propranolol (40 mg tab) | Β-blocker | | | | Bradycardia Severe CHF |
| Methyldopa (250mg tab) | | 250mg BD or TDS | 500mg OD | 2g OD | Liver disease |
Adult antihypertensive dosing and side effects

## Slide 42
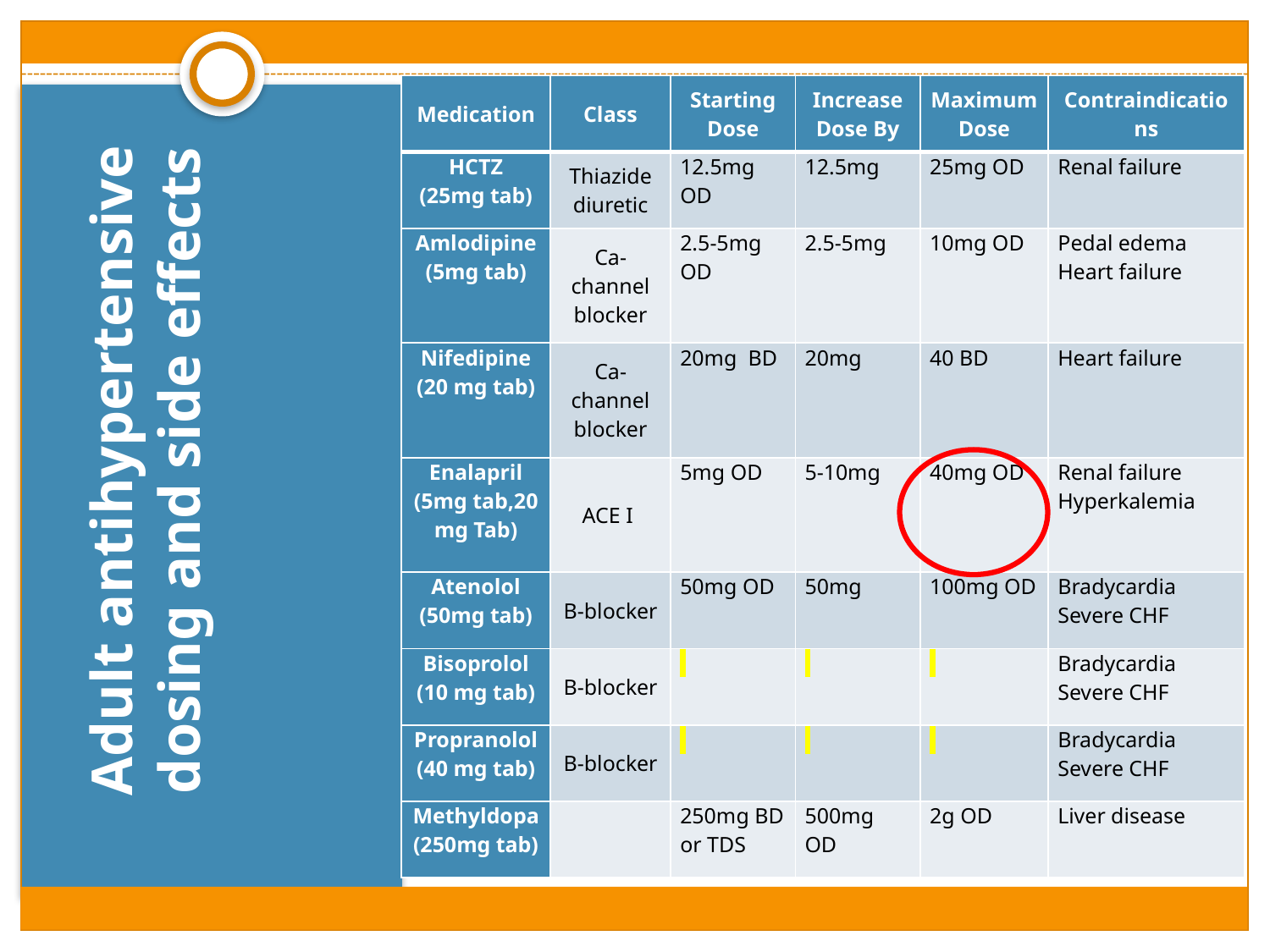

| Medication | Class | Starting Dose | Increase Dose By | Maximum Dose | Contraindications |
| --- | --- | --- | --- | --- | --- |
| HCTZ (25mg tab) | Thiazide diuretic | 12.5mg OD | 12.5mg | 25mg OD | Renal failure |
| Amlodipine (5mg tab) | Ca-channel blocker | 2.5-5mg OD | 2.5-5mg | 10mg OD | Pedal edema Heart failure |
| Nifedipine (20 mg tab) | Ca-channel blocker | 20mg BD | 20mg | 40 BD | Heart failure |
| Enalapril (5mg tab,20 mg Tab) | ACE I | 5mg OD | 5-10mg | 40mg OD | Renal failure Hyperkalemia |
| Atenolol (50mg tab) | Β-blocker | 50mg OD | 50mg | 100mg OD | Bradycardia Severe CHF |
| Bisoprolol (10 mg tab) | Β-blocker | | | | Bradycardia Severe CHF |
| Propranolol (40 mg tab) | Β-blocker | | | | Bradycardia Severe CHF |
| Methyldopa (250mg tab) | | 250mg BD or TDS | 500mg OD | 2g OD | Liver disease |
Adult antihypertensive dosing and side effects

## Slide 43
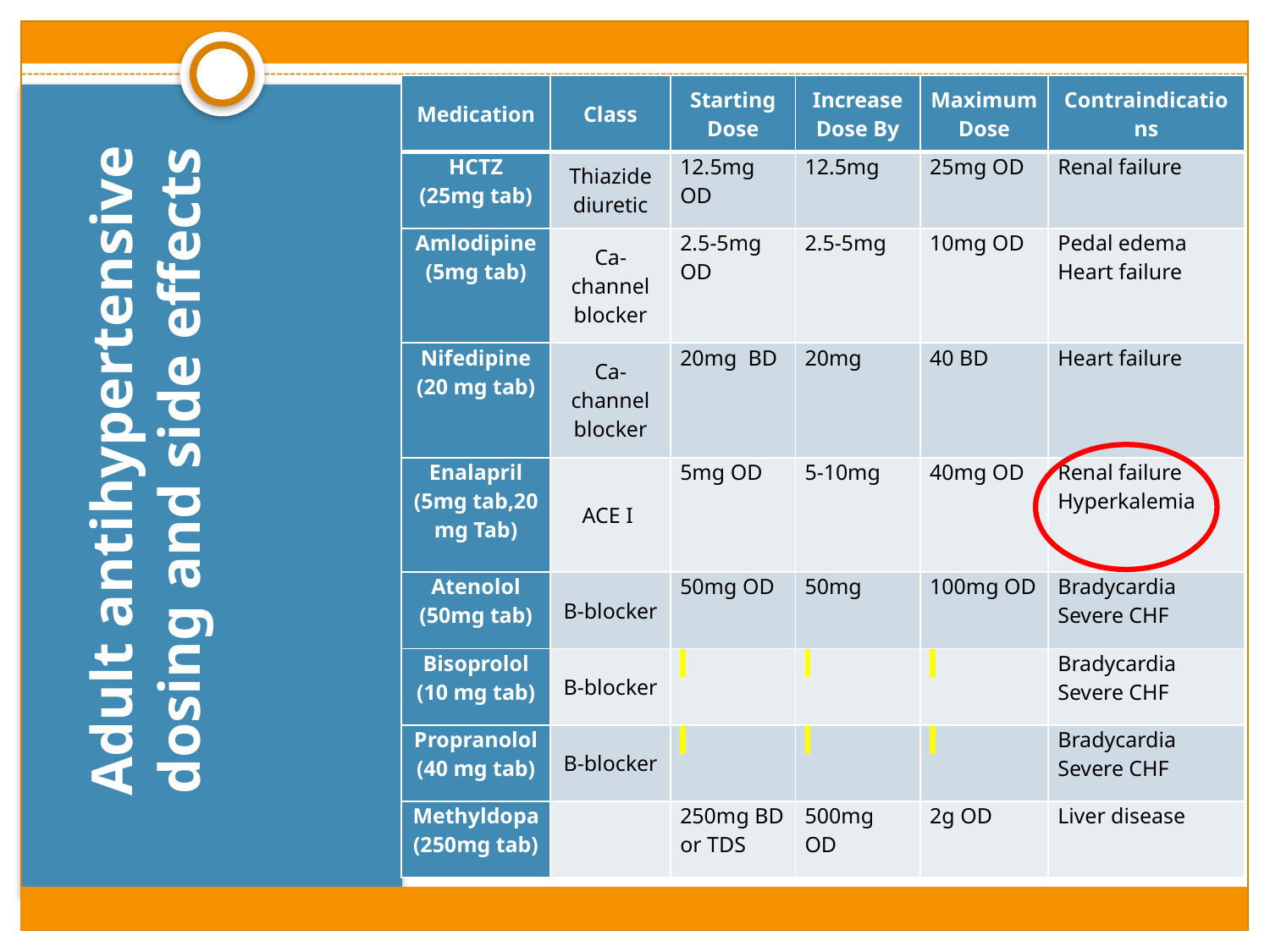

| Medication | Class | Starting Dose | Increase Dose By | Maximum Dose | Contraindications |
| --- | --- | --- | --- | --- | --- |
| HCTZ (25mg tab) | Thiazide diuretic | 12.5mg OD | 12.5mg | 25mg OD | Renal failure |
| Amlodipine (5mg tab) | Ca-channel blocker | 2.5-5mg OD | 2.5-5mg | 10mg OD | Pedal edema Heart failure |
| Nifedipine (20 mg tab) | Ca-channel blocker | 20mg BD | 20mg | 40 BD | Heart failure |
| Enalapril (5mg tab,20 mg Tab) | ACE I | 5mg OD | 5-10mg | 40mg OD | Renal failure Hyperkalemia |
| Atenolol (50mg tab) | Β-blocker | 50mg OD | 50mg | 100mg OD | Bradycardia Severe CHF |
| Bisoprolol (10 mg tab) | Β-blocker | | | | Bradycardia Severe CHF |
| Propranolol (40 mg tab) | Β-blocker | | | | Bradycardia Severe CHF |
| Methyldopa (250mg tab) | | 250mg BD or TDS | 500mg OD | 2g OD | Liver disease |
Adult antihypertensive dosing and side effects

## Slide 44
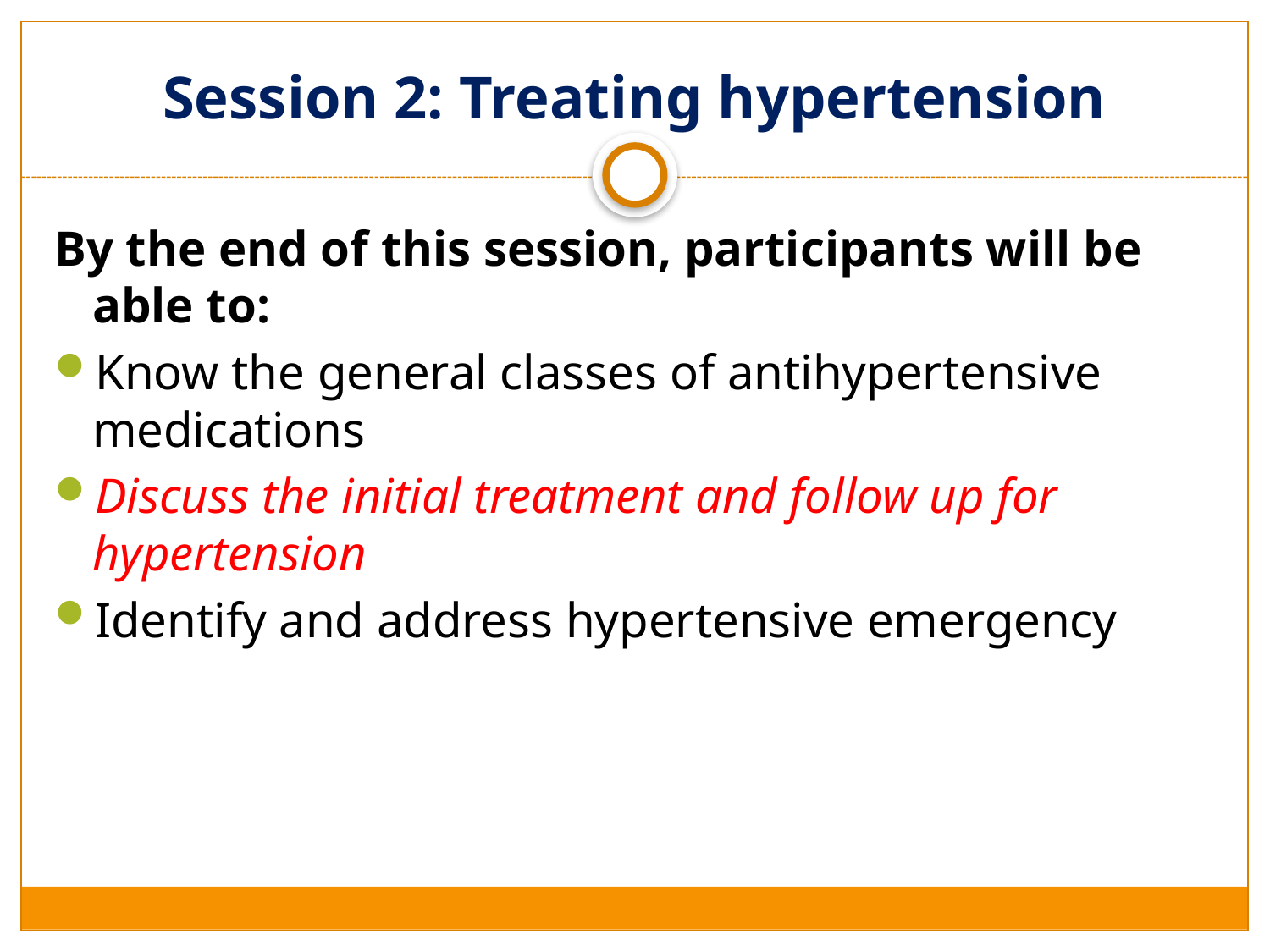

# Session 2: Treating hypertension
By the end of this session, participants will be able to:
Know the general classes of antihypertensive medications
Discuss the initial treatment and follow up for hypertension
Identify and address hypertensive emergency

## Slide 45
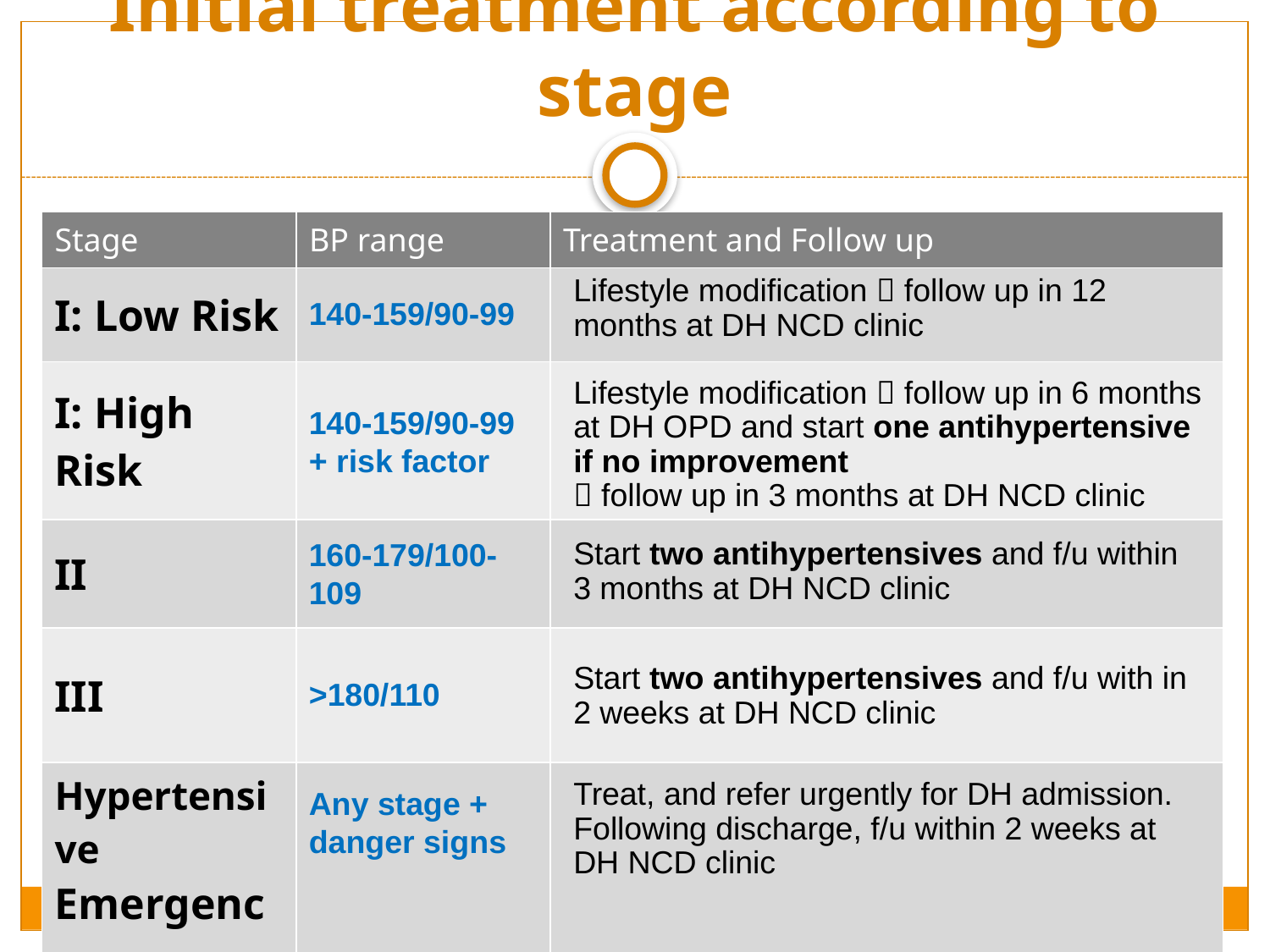

# Initial treatment according to stage
| Stage | BP range | Treatment and Follow up |
| --- | --- | --- |
| I: Low Risk | | |
| I: High Risk | | |
| II | | |
| III | | |
| Hypertensive Emergency | | |
Lifestyle modification  follow up in 12 months at DH NCD clinic
140-159/90-99
Lifestyle modification  follow up in 6 months at DH OPD and start one antihypertensive if no improvement
 follow up in 3 months at DH NCD clinic
140-159/90-99 + risk factor
160-179/100-109
Start two antihypertensives and f/u within 3 months at DH NCD clinic
Start two antihypertensives and f/u with in 2 weeks at DH NCD clinic
>180/110
Treat, and refer urgently for DH admission. Following discharge, f/u within 2 weeks at DH NCD clinic
Any stage + danger signs

## Slide 46
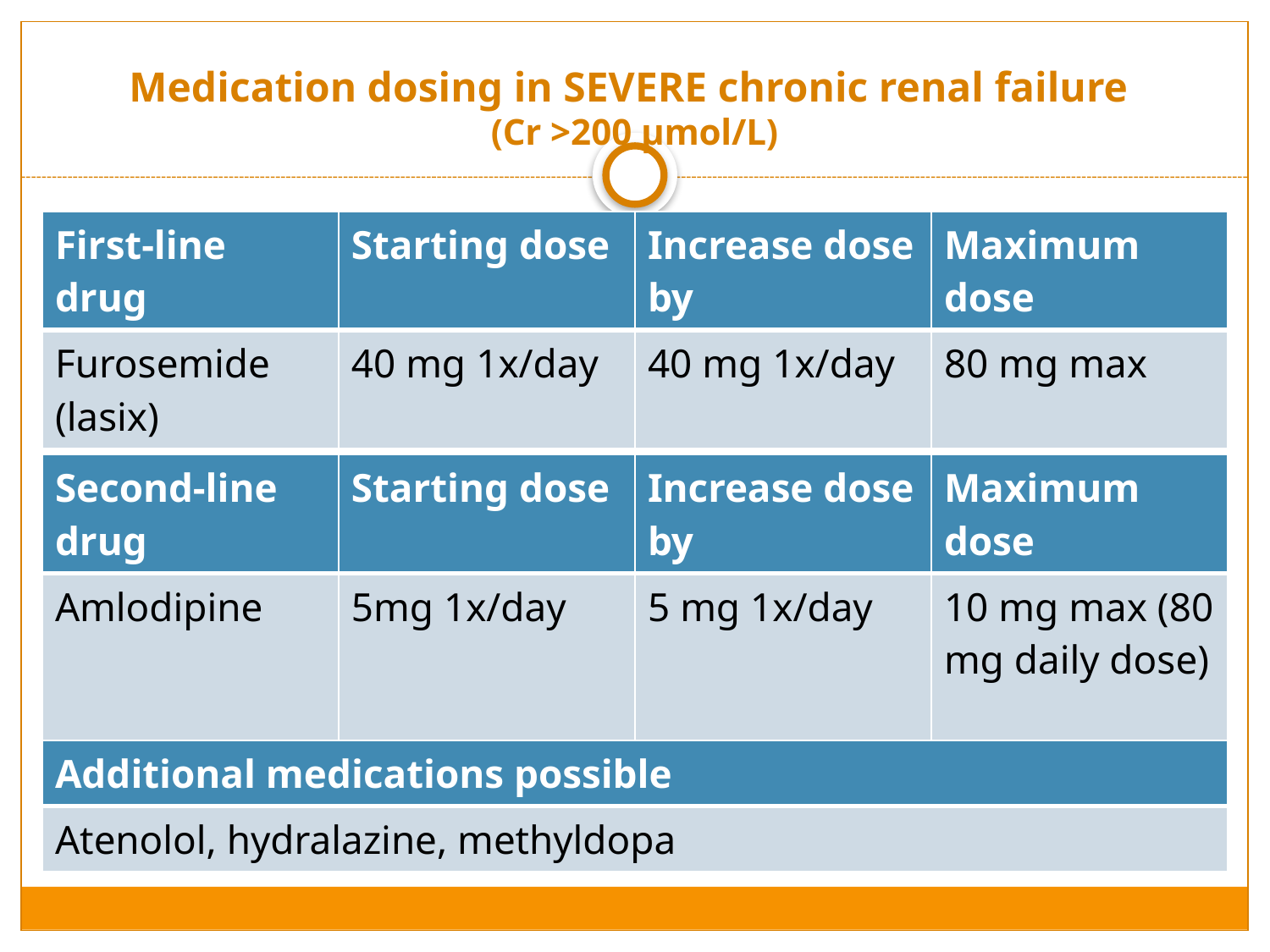

# Medication dosing in SEVERE chronic renal failure (Cr >200 μmol/L)
| First-line drug | Starting dose | Increase dose by | Maximum dose |
| --- | --- | --- | --- |
| Furosemide (lasix) | 40 mg 1x/day | 40 mg 1x/day | 80 mg max |
| Second-line drug | Starting dose | Increase dose by | Maximum dose |
| --- | --- | --- | --- |
| Amlodipine | 5mg 1x/day | 5 mg 1x/day | 10 mg max (80 mg daily dose) |
| Additional medications possible |
| --- |
| Atenolol, hydralazine, methyldopa |

## Slide 47
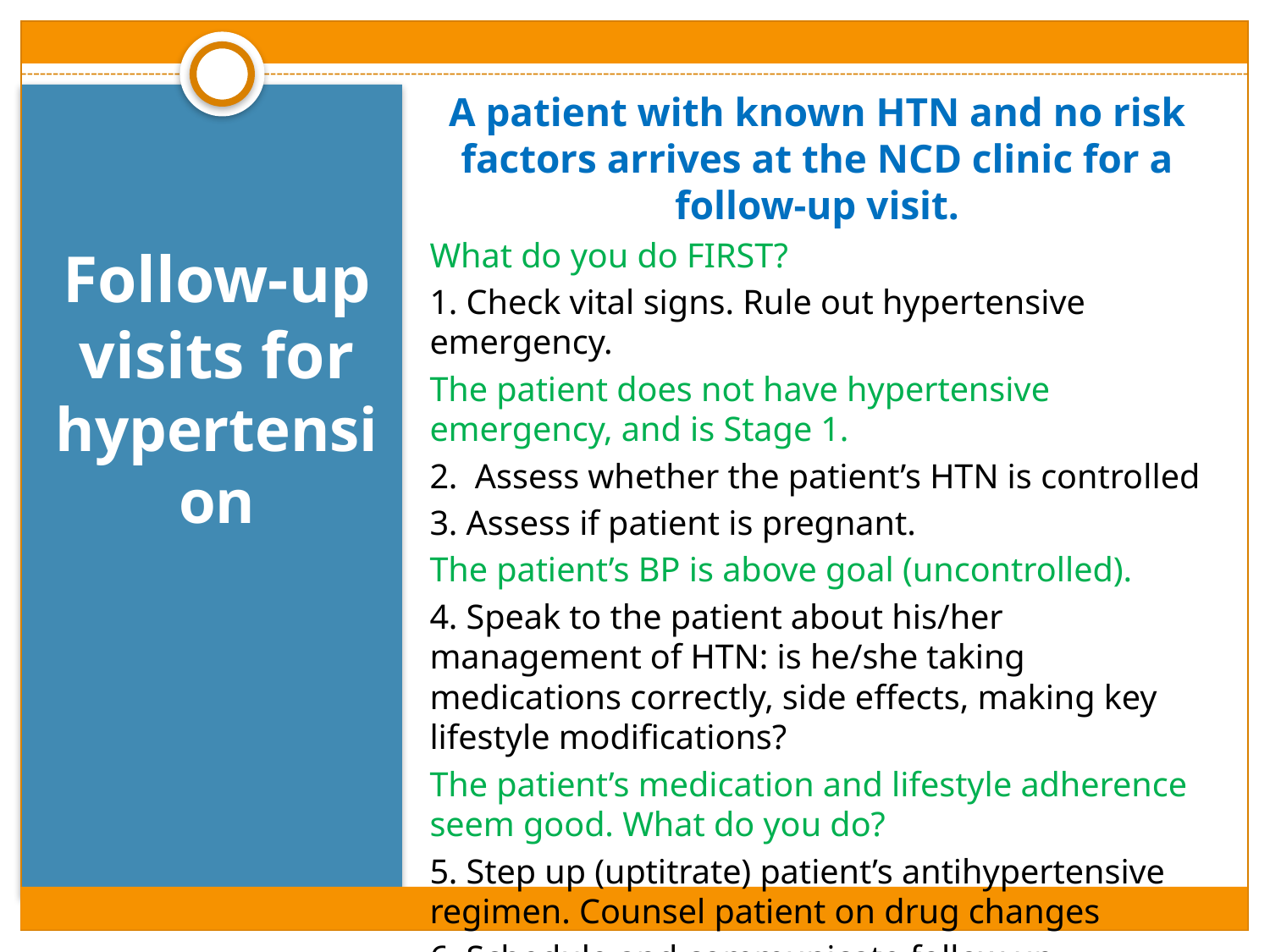

A patient with known HTN and no risk factors arrives at the NCD clinic for a follow-up visit.
What do you do FIRST?
1. Check vital signs. Rule out hypertensive emergency.
The patient does not have hypertensive emergency, and is Stage 1.
2. Assess whether the patient’s HTN is controlled
3. Assess if patient is pregnant.
The patient’s BP is above goal (uncontrolled).
4. Speak to the patient about his/her management of HTN: is he/she taking medications correctly, side effects, making key lifestyle modifications?
The patient’s medication and lifestyle adherence seem good. What do you do?
5. Step up (uptitrate) patient’s antihypertensive regimen. Counsel patient on drug changes
6. Schedule and communicate follow up appointment.
Follow-up visits for hypertension

## Slide 48
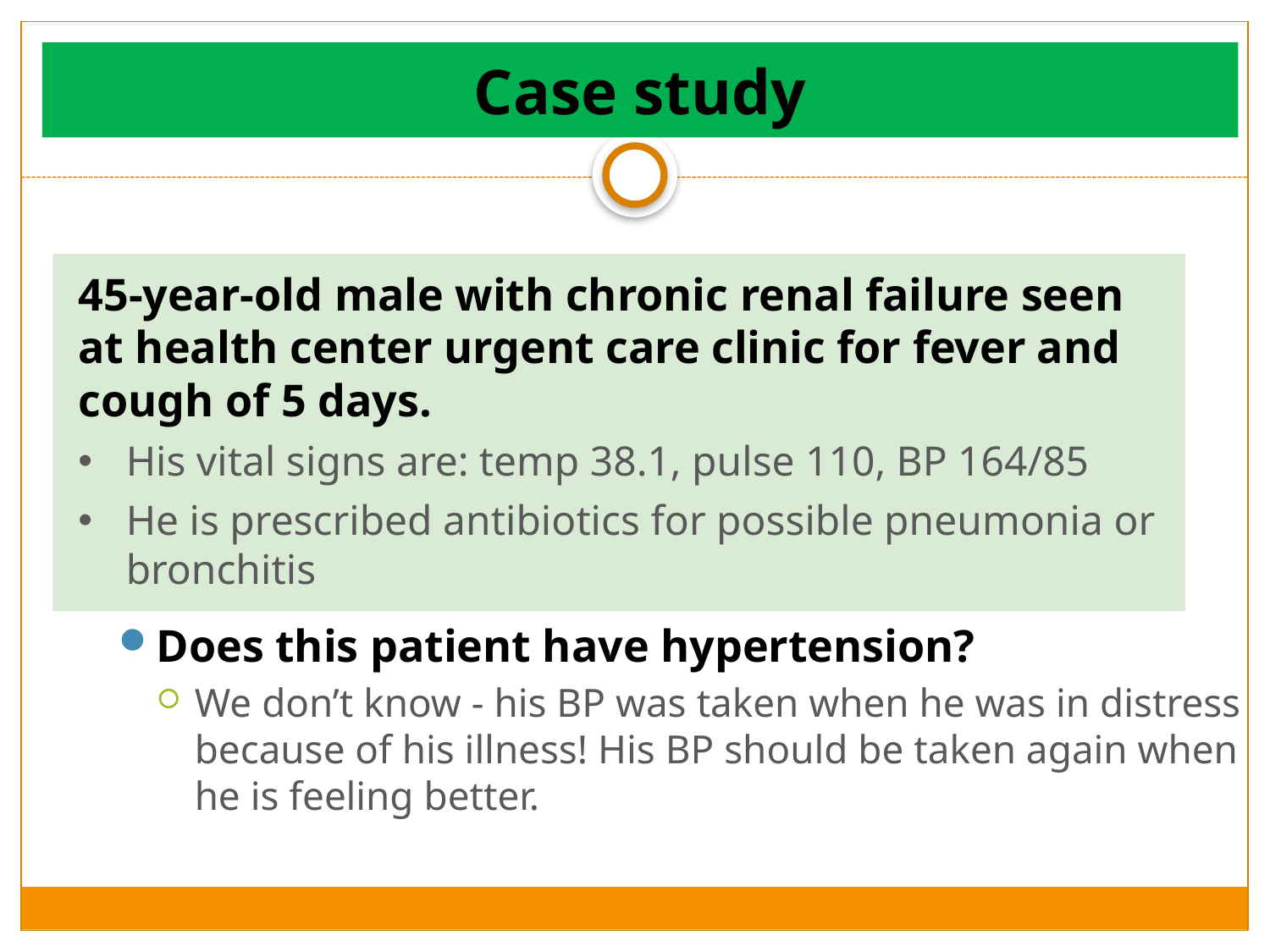

# CASE
Case study
45-year-old male with chronic renal failure seen at health center urgent care clinic for fever and cough of 5 days.
His vital signs are: temp 38.1, pulse 110, BP 164/85
He is prescribed antibiotics for possible pneumonia or bronchitis
Does this patient have hypertension?
We don’t know - his BP was taken when he was in distress because of his illness! His BP should be taken again when he is feeling better.

## Slide 49
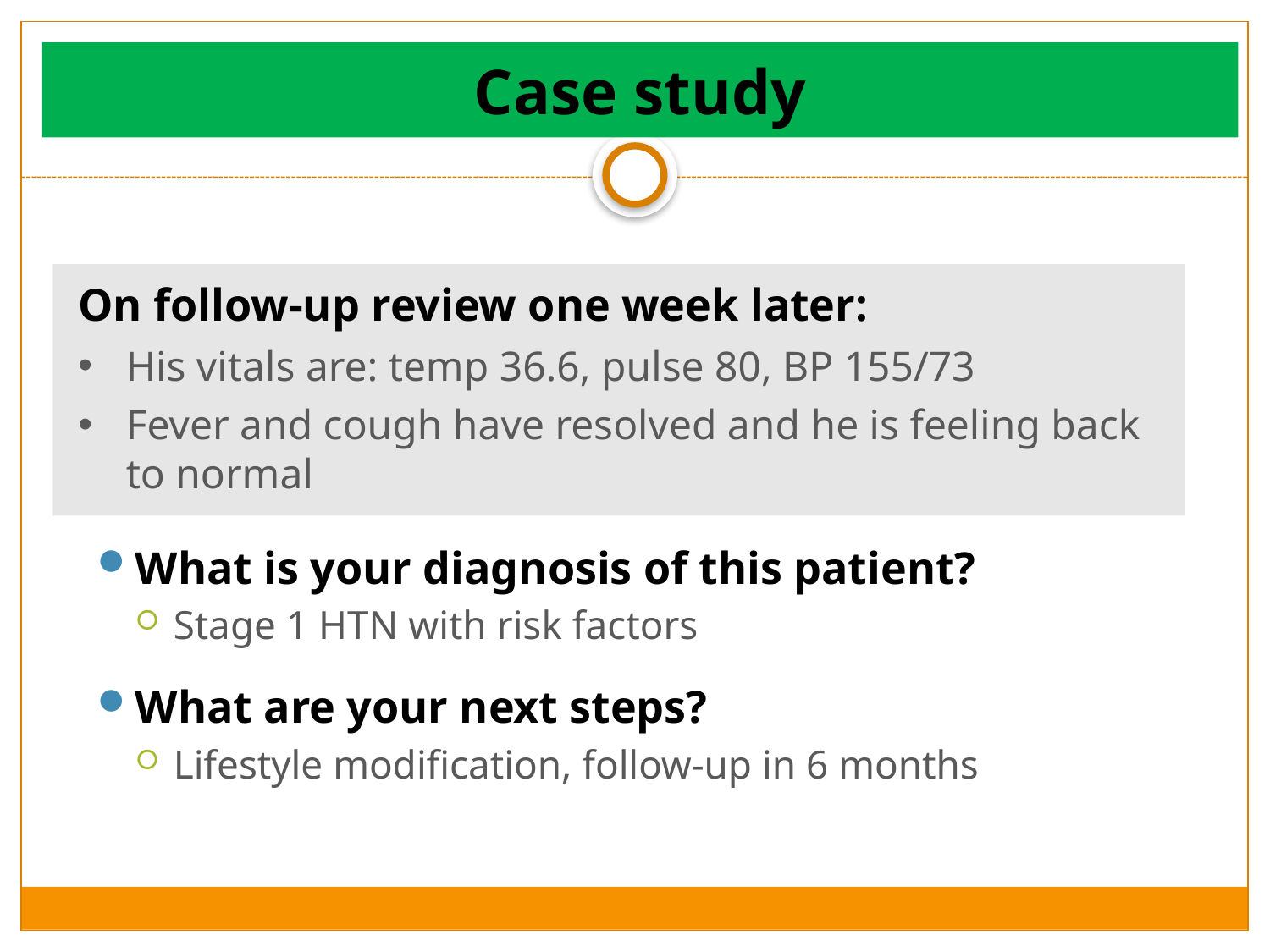

# CASE
Case study
On follow-up review one week later:
His vitals are: temp 36.6, pulse 80, BP 155/73
Fever and cough have resolved and he is feeling back to normal
What is your diagnosis of this patient?
Stage 1 HTN with risk factors
What are your next steps?
Lifestyle modification, follow-up in 6 months

## Slide 50
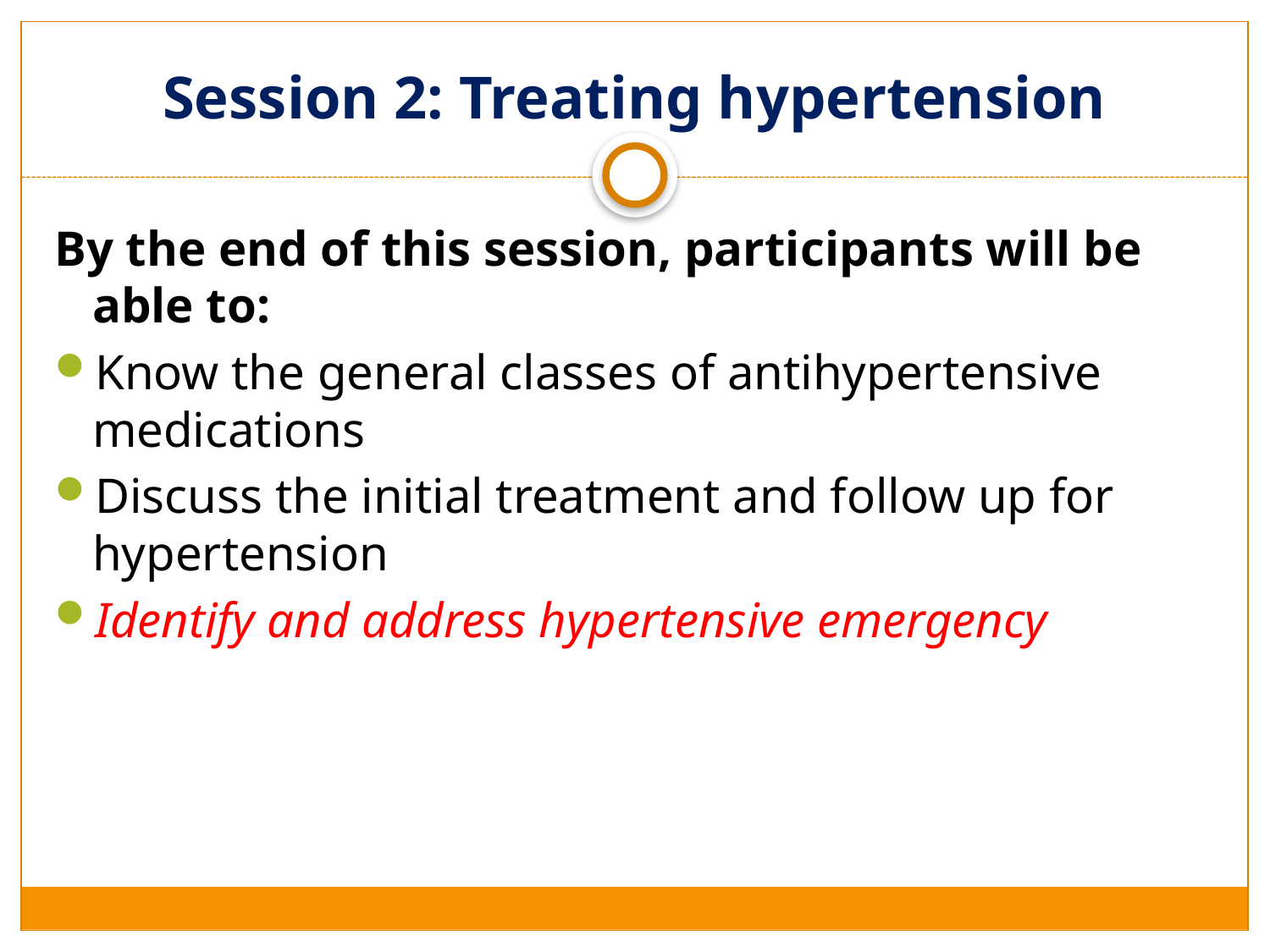

# Session 2: Treating hypertension
By the end of this session, participants will be able to:
Know the general classes of antihypertensive medications
Discuss the initial treatment and follow up for hypertension
Identify and address hypertensive emergency

## Slide 51
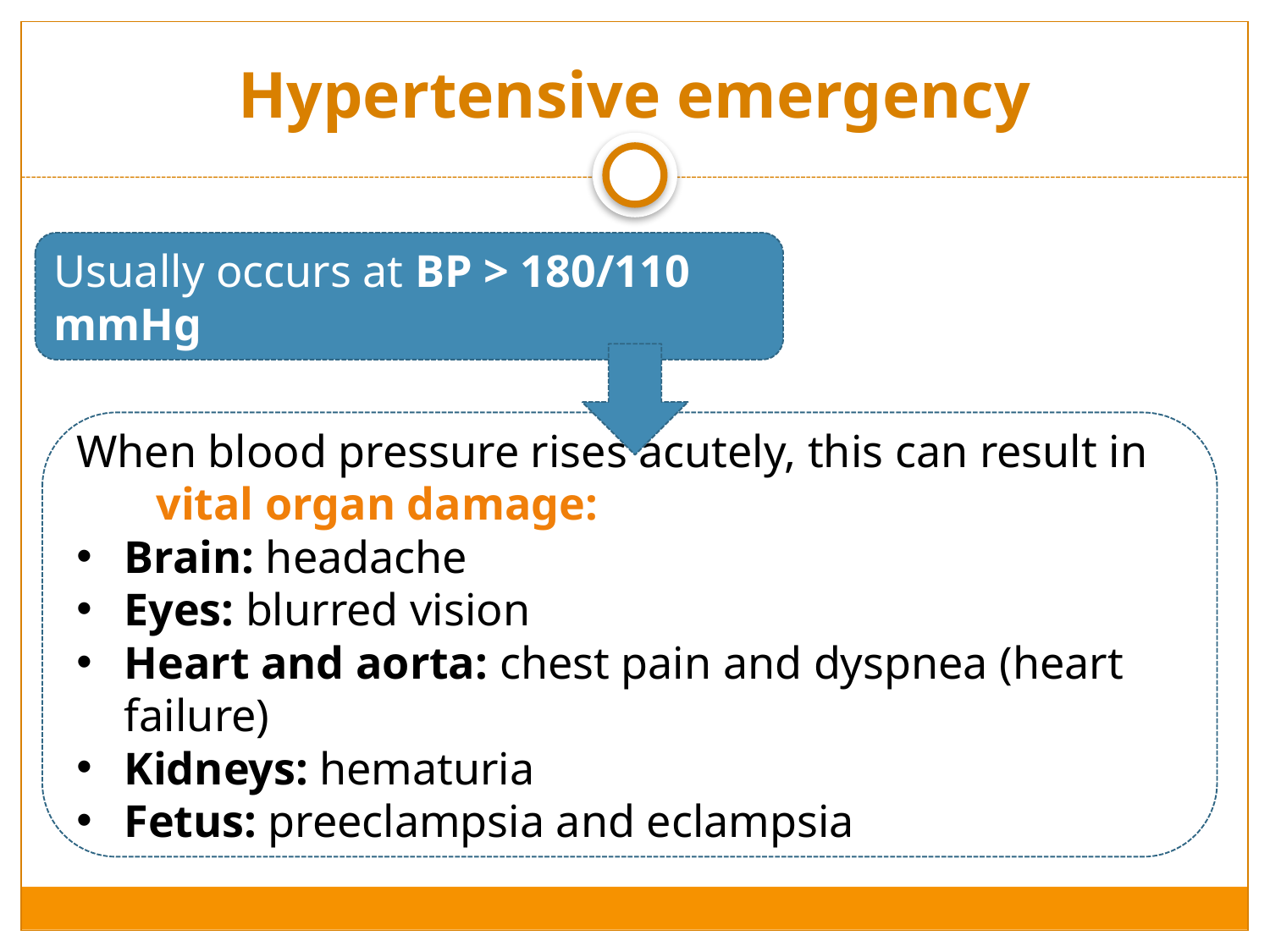

# Hypertensive emergency
Usually occurs at BP > 180/110 mmHg
When blood pressure rises acutely, this can result in vital organ damage:
Brain: headache
Eyes: blurred vision
Heart and aorta: chest pain and dyspnea (heart failure)
Kidneys: hematuria
Fetus: preeclampsia and eclampsia

## Slide 52
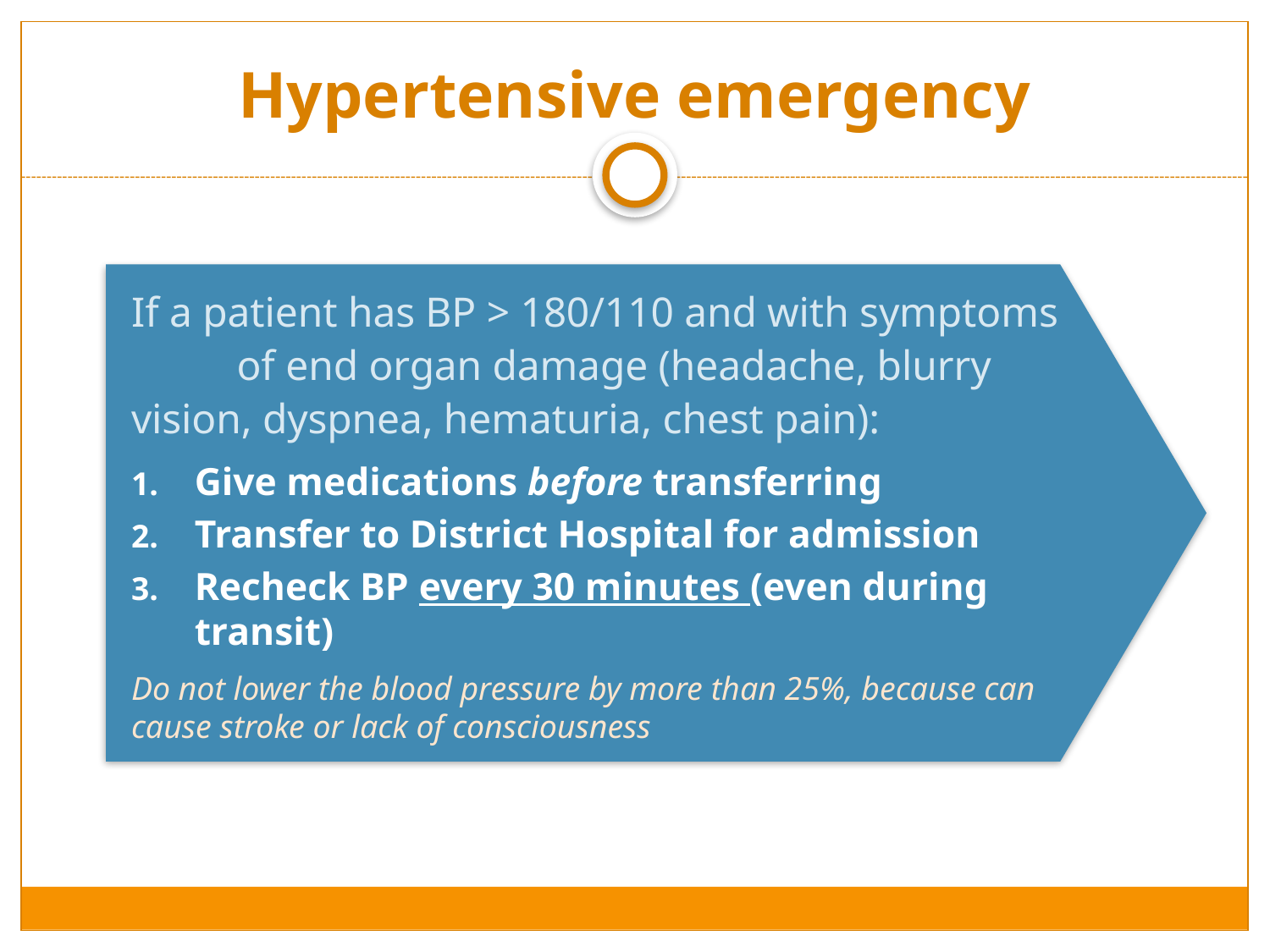

# Hypertensive emergency
If a patient has BP > 180/110 and with symptoms of end organ damage (headache, blurry vision, dyspnea, hematuria, chest pain):
Give medications before transferring
Transfer to District Hospital for admission
Recheck BP every 30 minutes (even during transit)
Do not lower the blood pressure by more than 25%, because can cause stroke or lack of consciousness

## Slide 53
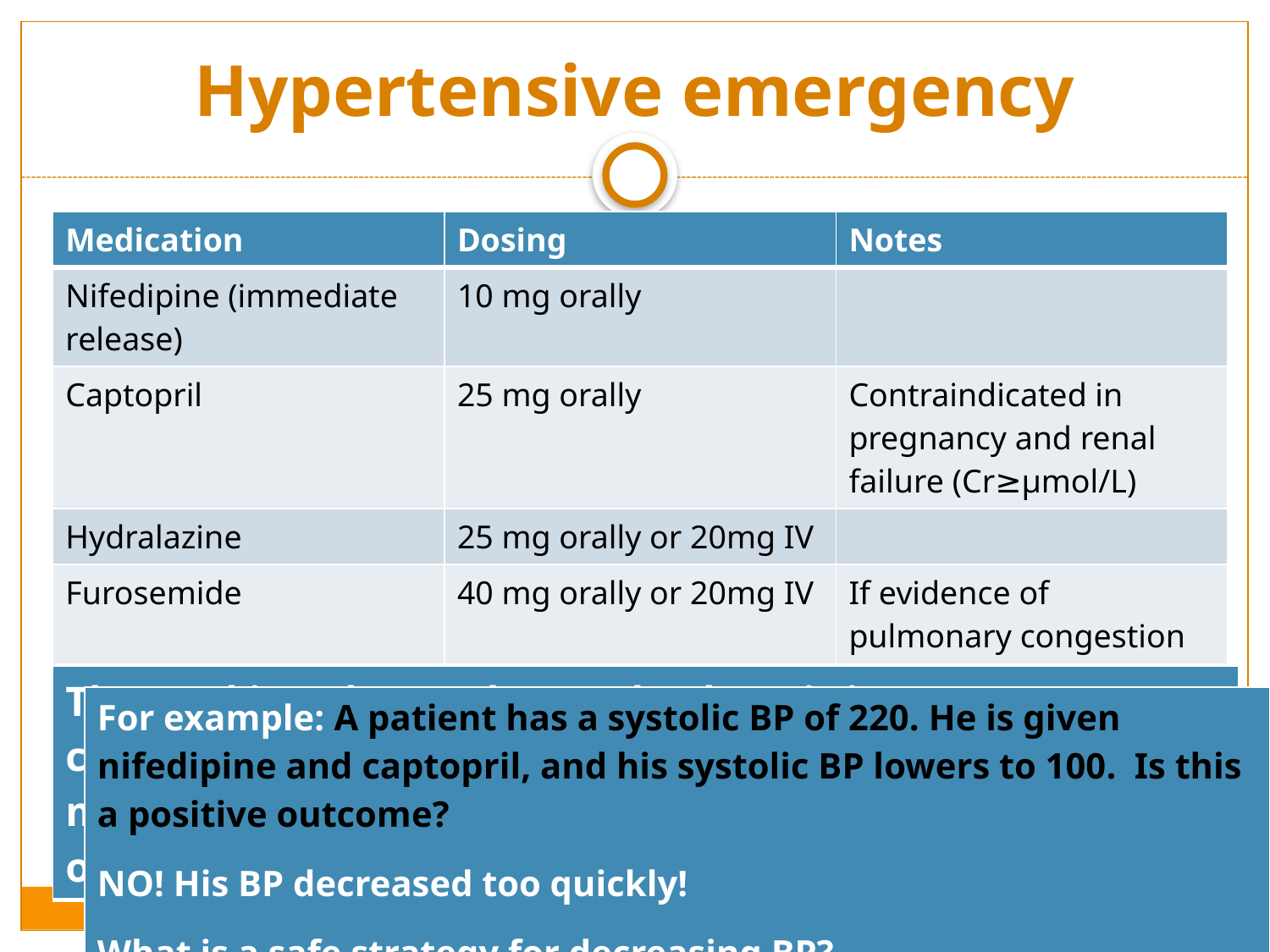

# Hypertensive emergency
| Medication | Dosing | Notes |
| --- | --- | --- |
| Nifedipine (immediate release) | 10 mg orally | |
| Captopril | 25 mg orally | Contraindicated in pregnancy and renal failure (Cr≥µmol/L) |
| Hydralazine | 25 mg orally or 20mg IV | |
| Furosemide | 40 mg orally or 20mg IV | If evidence of pulmonary congestion |
| The goal is to lower the BP slowly. It is important to check the patient’s BP every 30 minutes because these medications take effect in 30 min – 1 hour (when given orally). |
| --- |
| For example: A patient has a systolic BP of 220. He is given nifedipine and captopril, and his systolic BP lowers to 100. Is this a positive outcome? NO! His BP decreased too quickly! What is a safe strategy for decreasing BP? 30 minutes after receiving medication, BP should be ~180. It should remain at this BP for 4 hours, and then can be lowered further. Within 24-36 hours, can reach normal blood pressure (go slowly!). |
| --- |

## Slide 54
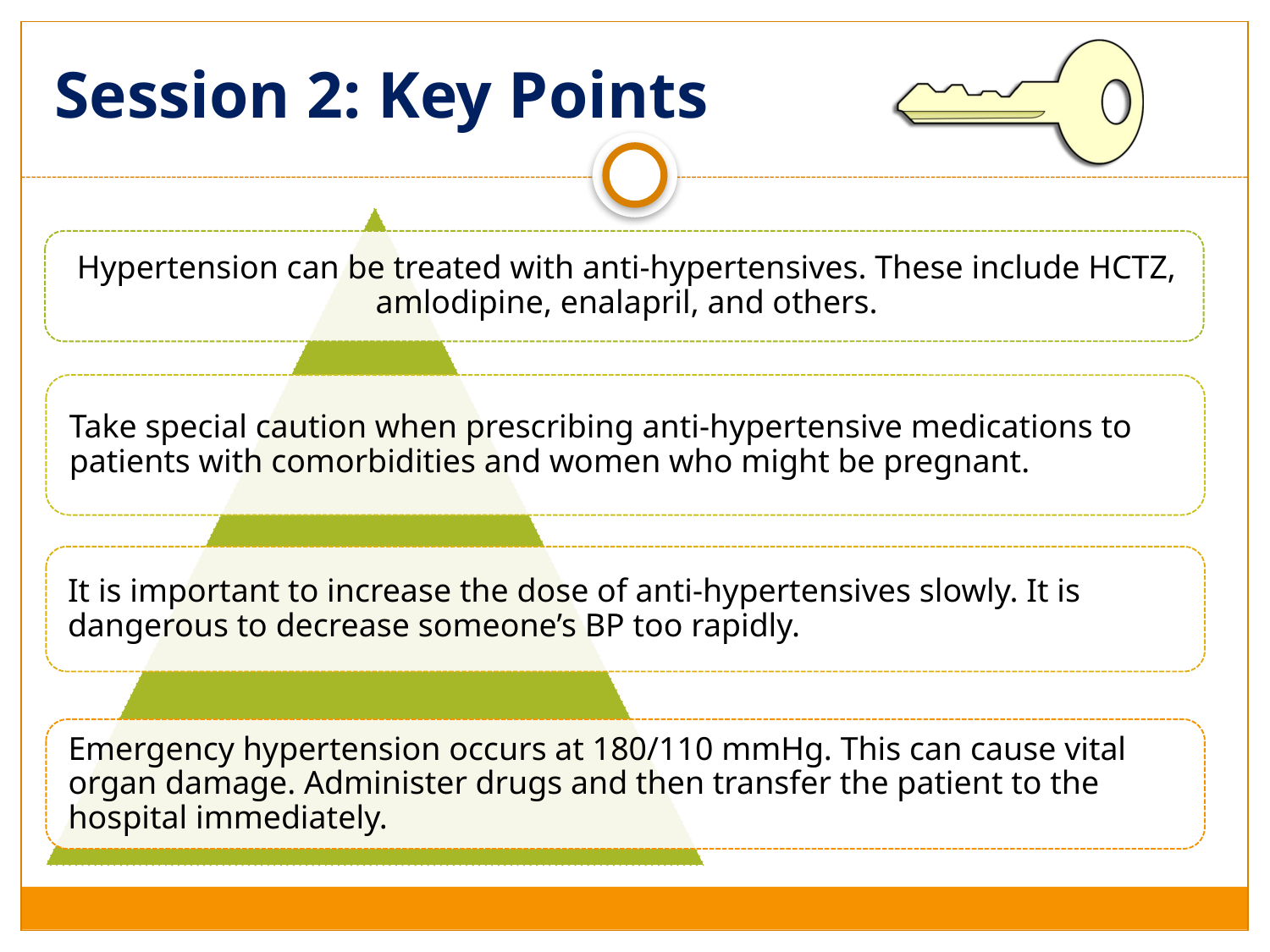

# Session 2: Key Points

## Slide 55
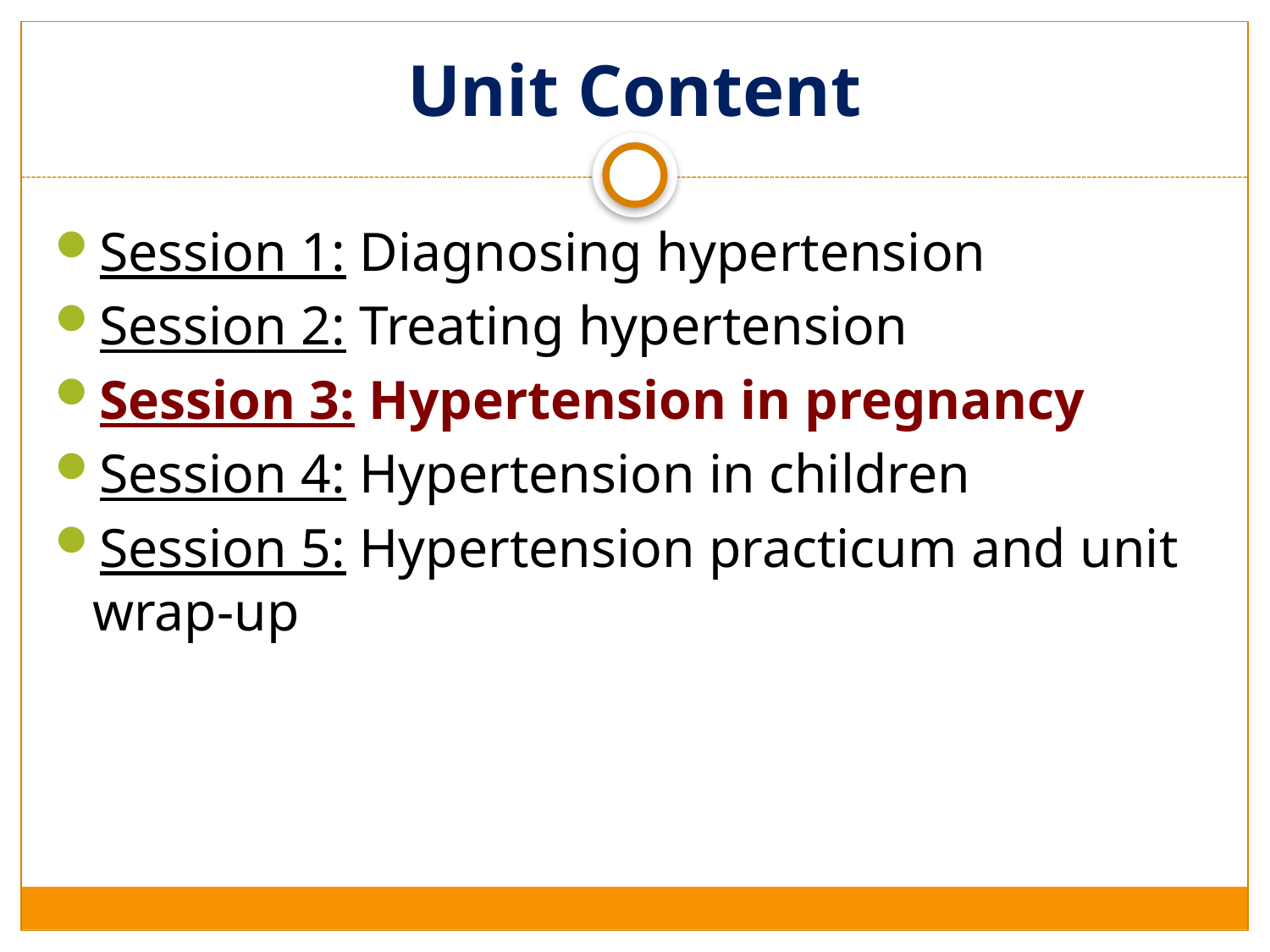

# Unit Content
Session 1: Diagnosing hypertension
Session 2: Treating hypertension
Session 3: Hypertension in pregnancy
Session 4: Hypertension in children
Session 5: Hypertension practicum and unit wrap-up

## Slide 56
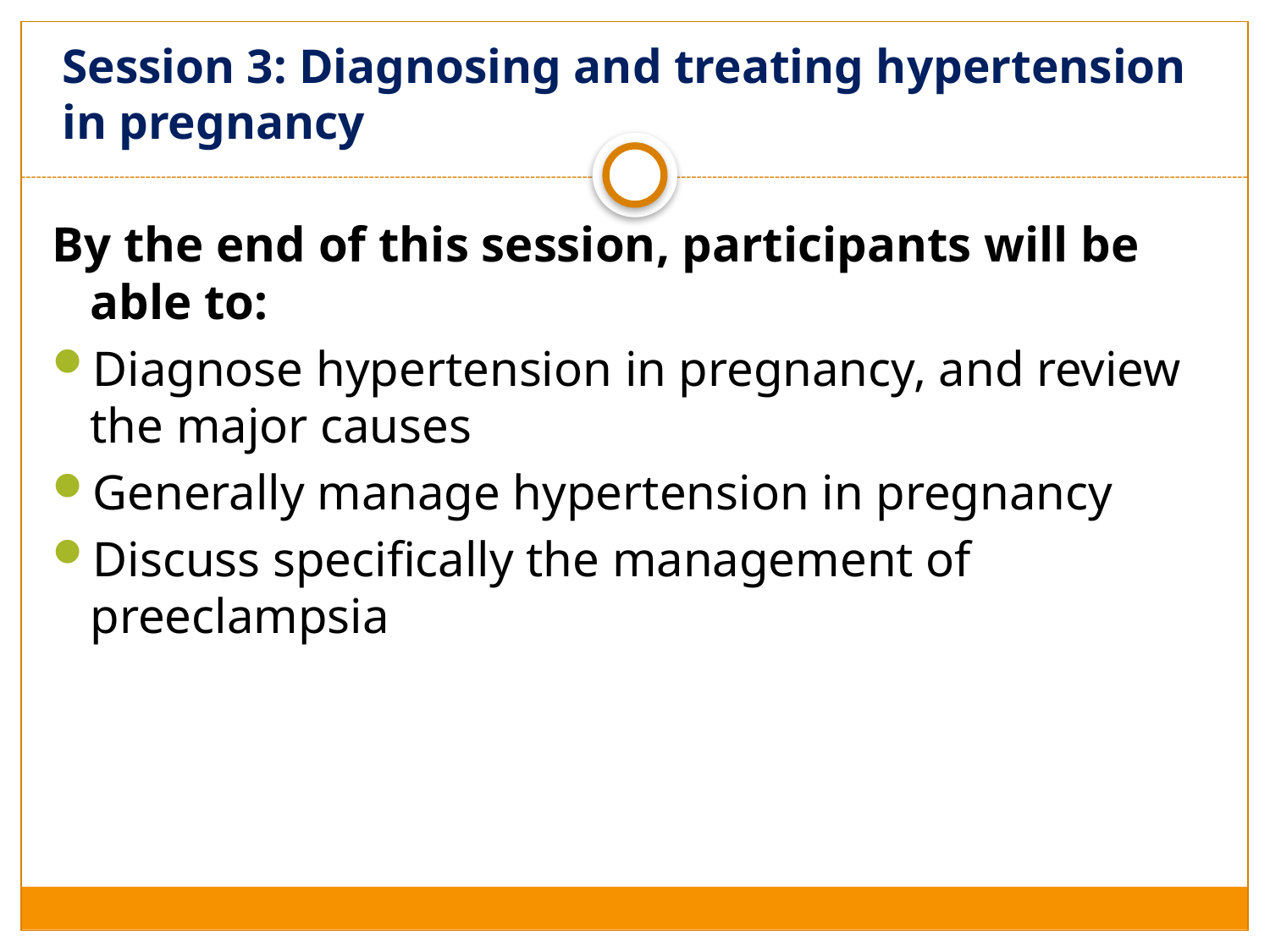

# Session 3: Diagnosing and treating hypertension in pregnancy
By the end of this session, participants will be able to:
Diagnose hypertension in pregnancy, and review the major causes
Generally manage hypertension in pregnancy
Discuss specifically the management of preeclampsia

## Slide 57
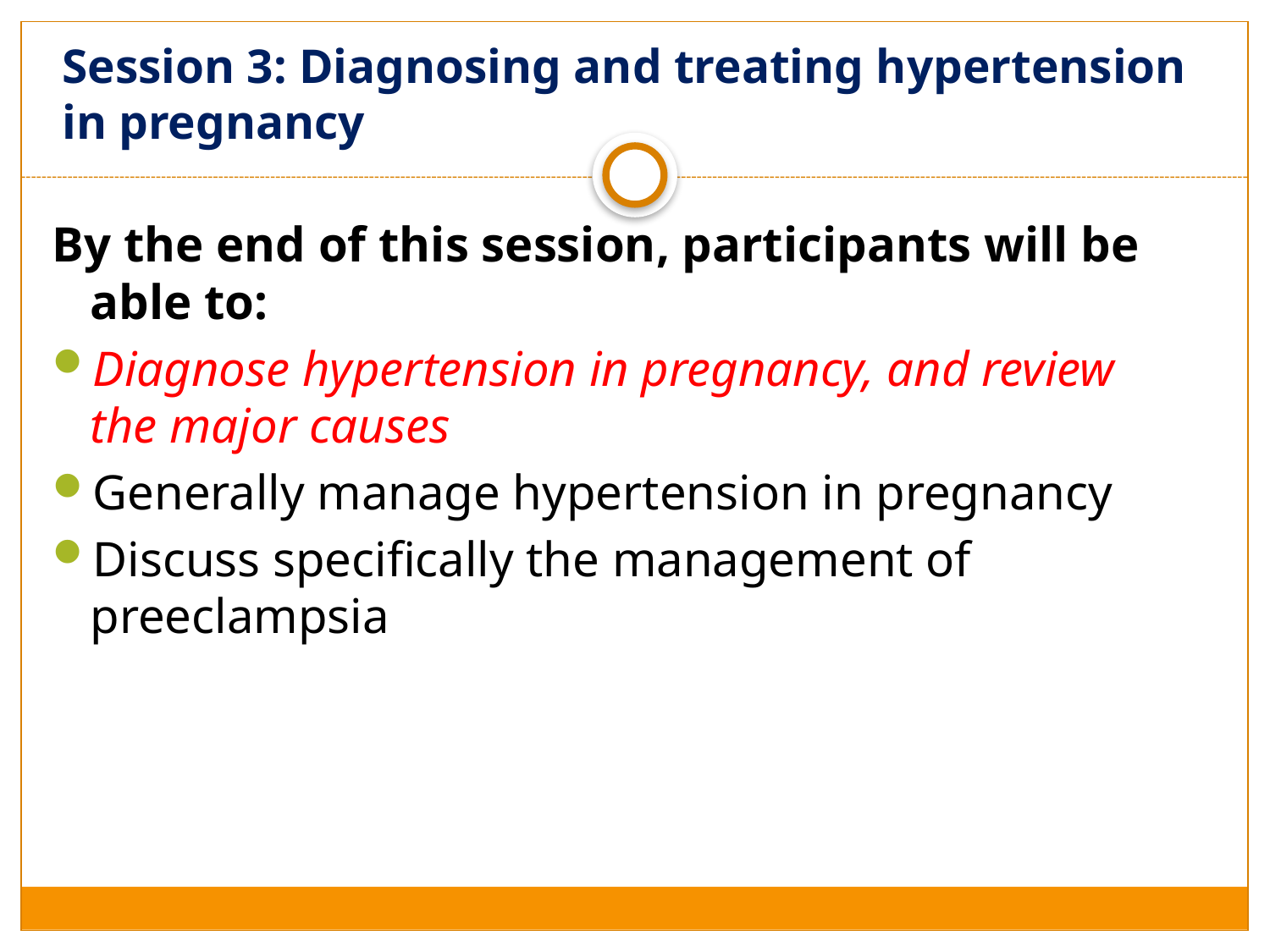

# Session 3: Diagnosing and treating hypertension in pregnancy
By the end of this session, participants will be able to:
Diagnose hypertension in pregnancy, and review the major causes
Generally manage hypertension in pregnancy
Discuss specifically the management of preeclampsia

## Slide 58
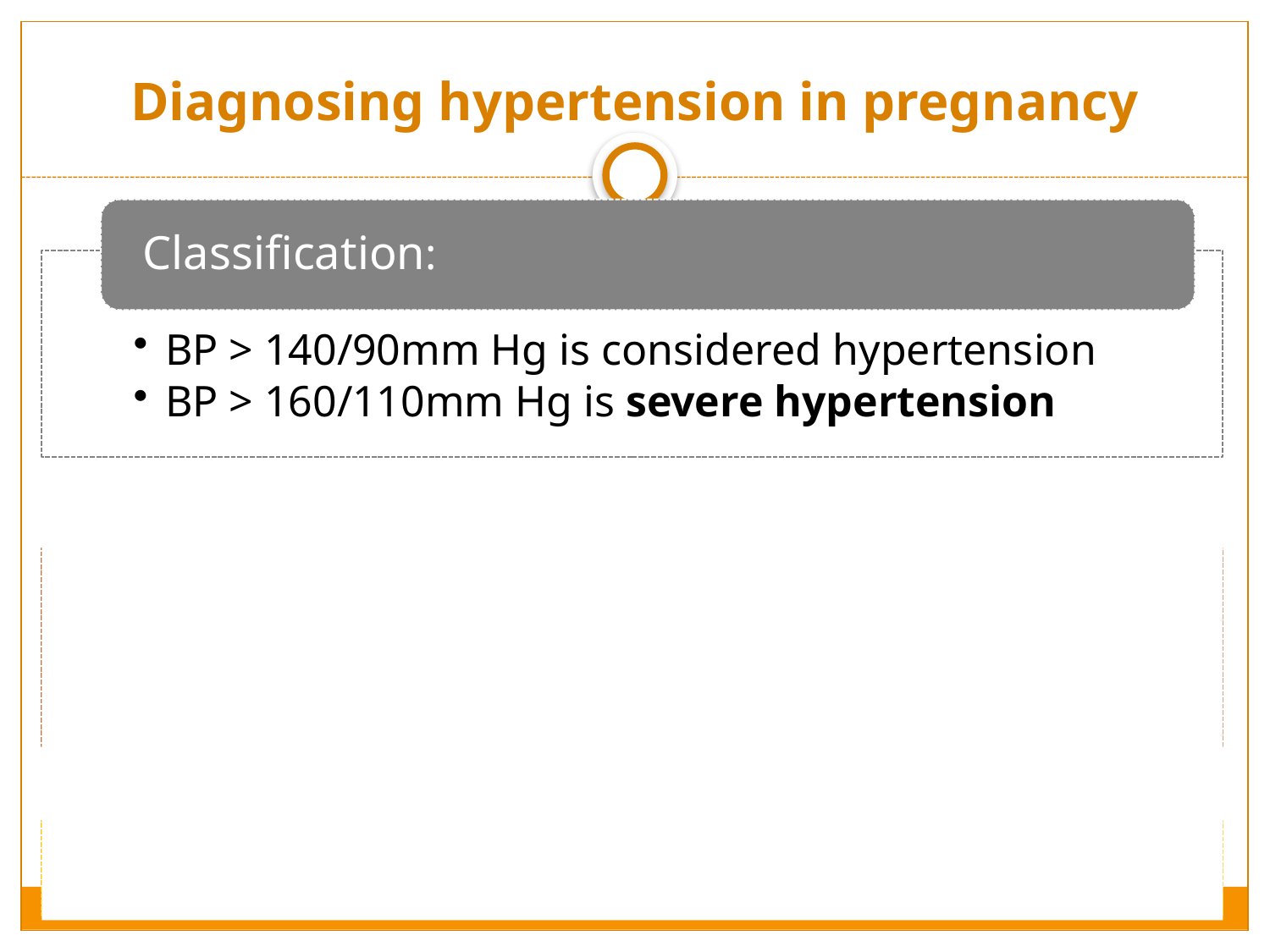

# Diagnosing hypertension in pregnancy

## Slide 59
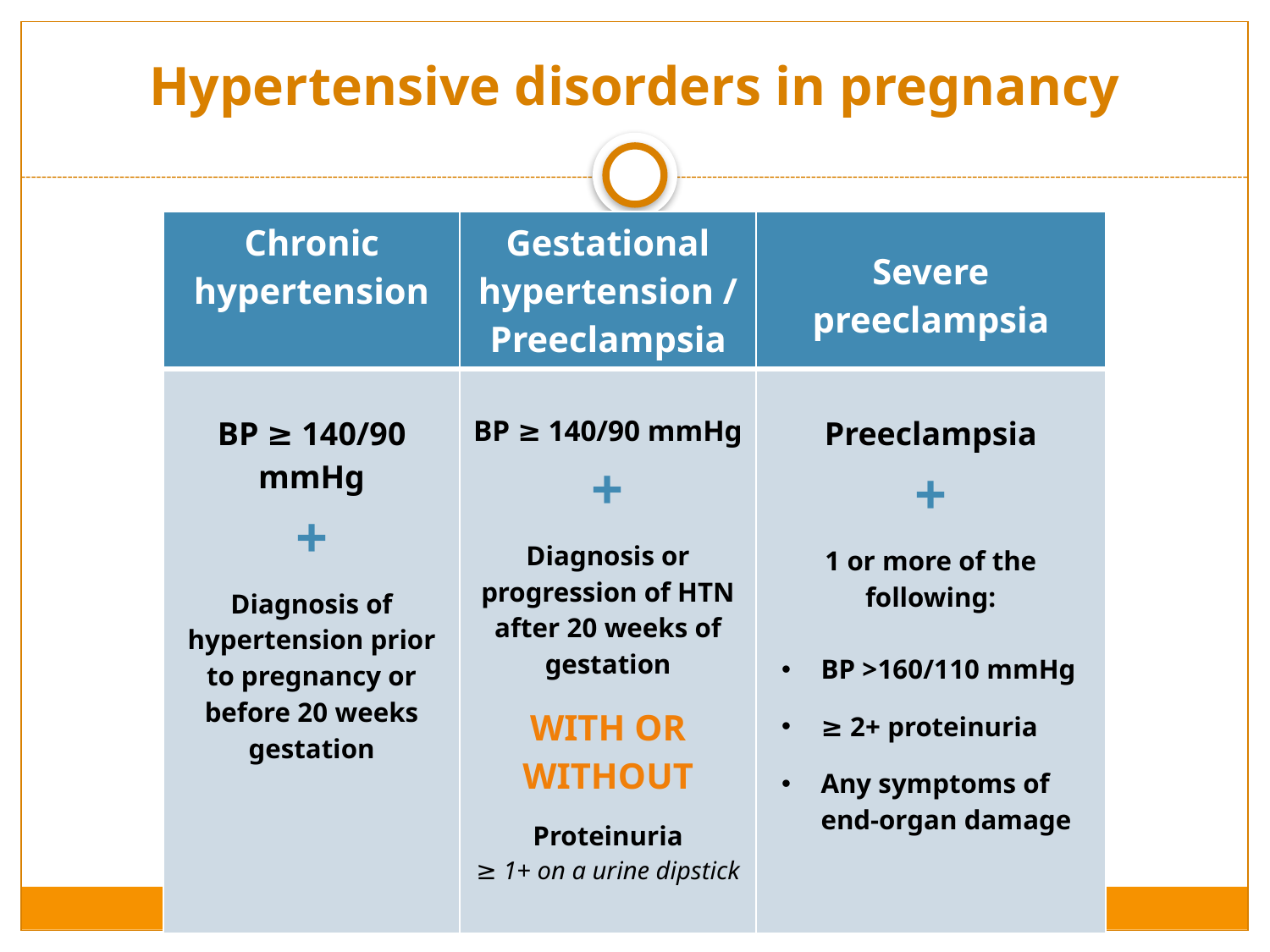

# Hypertensive disorders in pregnancy
| Chronic hypertension | Gestational hypertension / Preeclampsia | Severe preeclampsia |
| --- | --- | --- |
| BP ≥ 140/90 mmHg + Diagnosis of hypertension prior to pregnancy or before 20 weeks gestation | BP ≥ 140/90 mmHg + Diagnosis or progression of HTN after 20 weeks of gestation WITH OR WITHOUT Proteinuria ≥ 1+ on a urine dipstick | Preeclampsia + 1 or more of the following: BP >160/110 mmHg ≥ 2+ proteinuria Any symptoms of end-organ damage |

## Slide 60
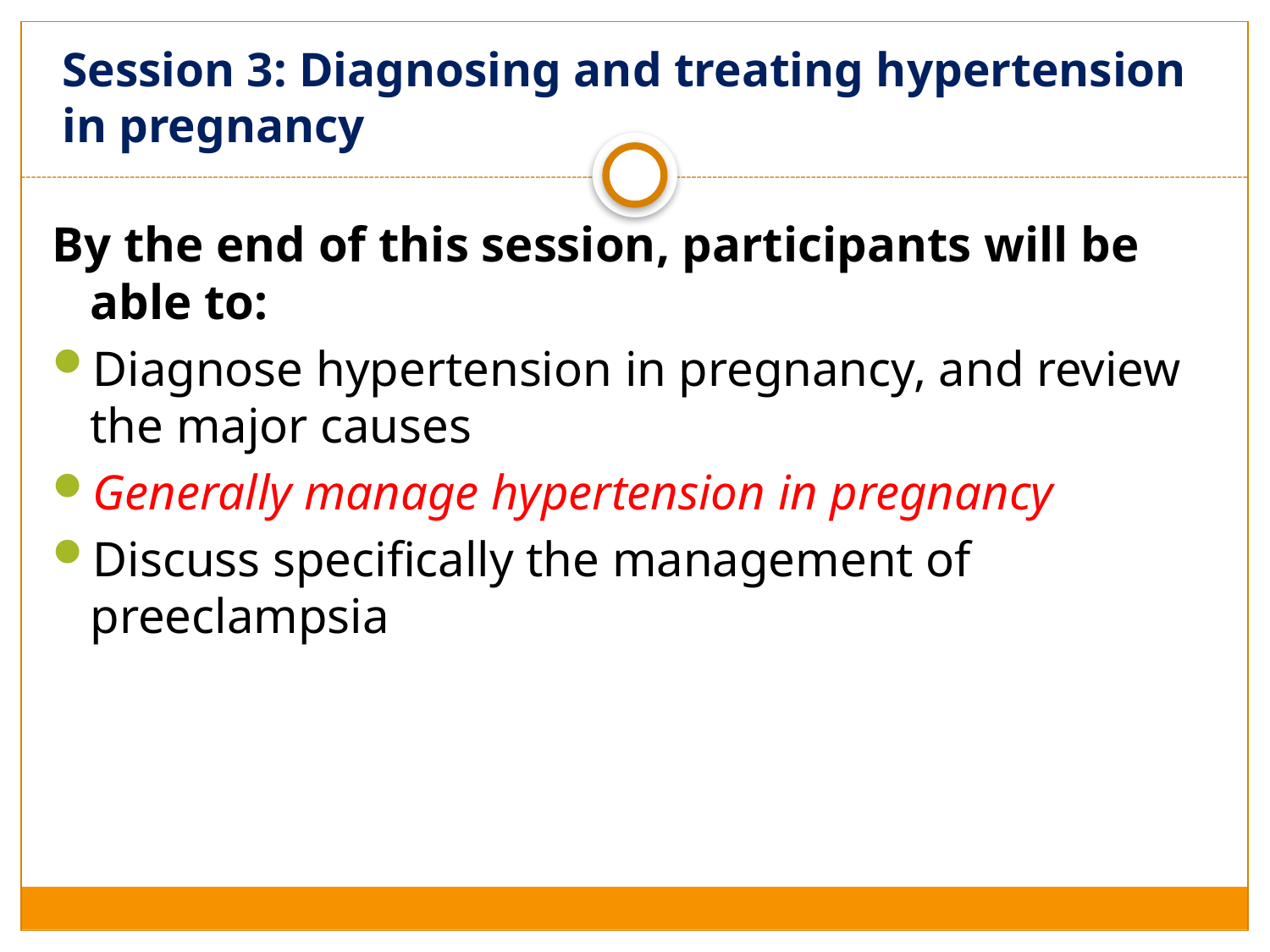

# Session 3: Diagnosing and treating hypertension in pregnancy
By the end of this session, participants will be able to:
Diagnose hypertension in pregnancy, and review the major causes
Generally manage hypertension in pregnancy
Discuss specifically the management of preeclampsia

## Slide 61
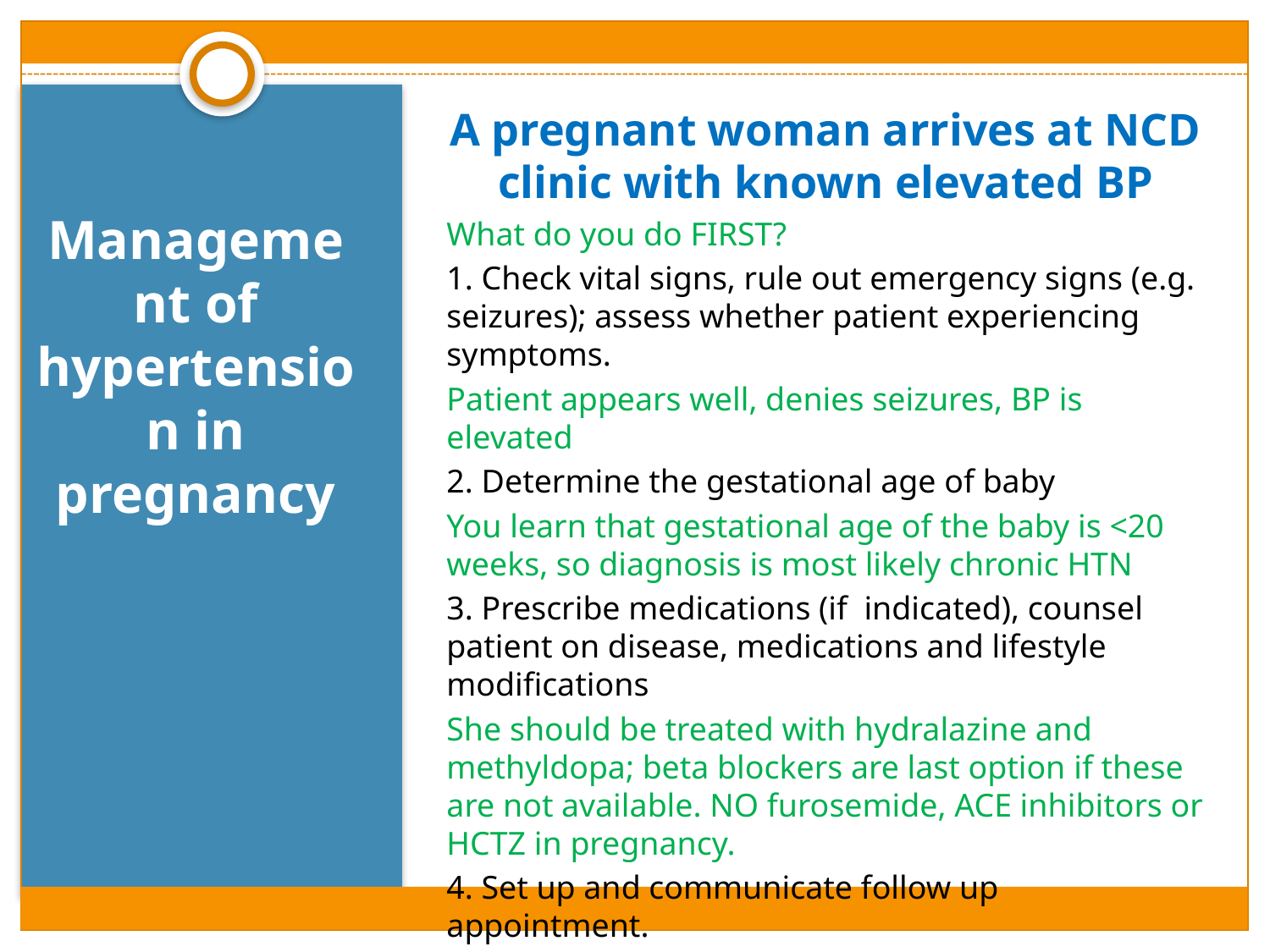

A pregnant woman arrives at NCD clinic with known elevated BP
What do you do FIRST?
1. Check vital signs, rule out emergency signs (e.g. seizures); assess whether patient experiencing symptoms.
Patient appears well, denies seizures, BP is elevated
2. Determine the gestational age of baby
You learn that gestational age of the baby is <20 weeks, so diagnosis is most likely chronic HTN
3. Prescribe medications (if indicated), counsel patient on disease, medications and lifestyle modifications
She should be treated with hydralazine and methyldopa; beta blockers are last option if these are not available. NO furosemide, ACE inhibitors or HCTZ in pregnancy.
4. Set up and communicate follow up appointment.
She should return at 20 weeks gestation. At next visit, if she still has elevated BP, test urine protein (dipstick)
# Management of hypertension in pregnancy

## Slide 62
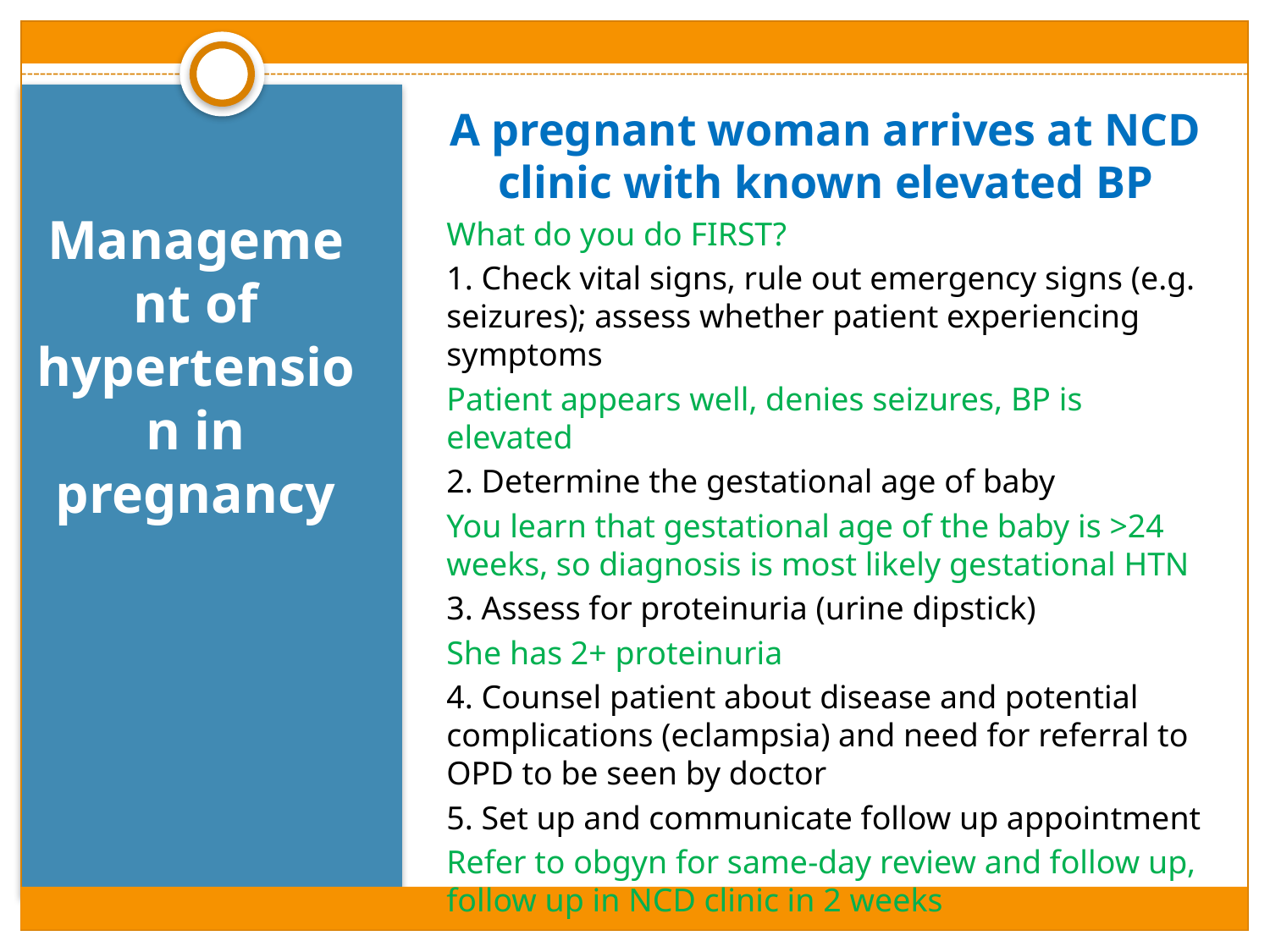

A pregnant woman arrives at NCD clinic with known elevated BP
What do you do FIRST?
1. Check vital signs, rule out emergency signs (e.g. seizures); assess whether patient experiencing symptoms
Patient appears well, denies seizures, BP is elevated
2. Determine the gestational age of baby
You learn that gestational age of the baby is >24 weeks, so diagnosis is most likely gestational HTN
3. Assess for proteinuria (urine dipstick)
She has 2+ proteinuria
4. Counsel patient about disease and potential complications (eclampsia) and need for referral to OPD to be seen by doctor
5. Set up and communicate follow up appointment
Refer to obgyn for same-day review and follow up, follow up in NCD clinic in 2 weeks
# Management of hypertension in pregnancy

## Slide 63
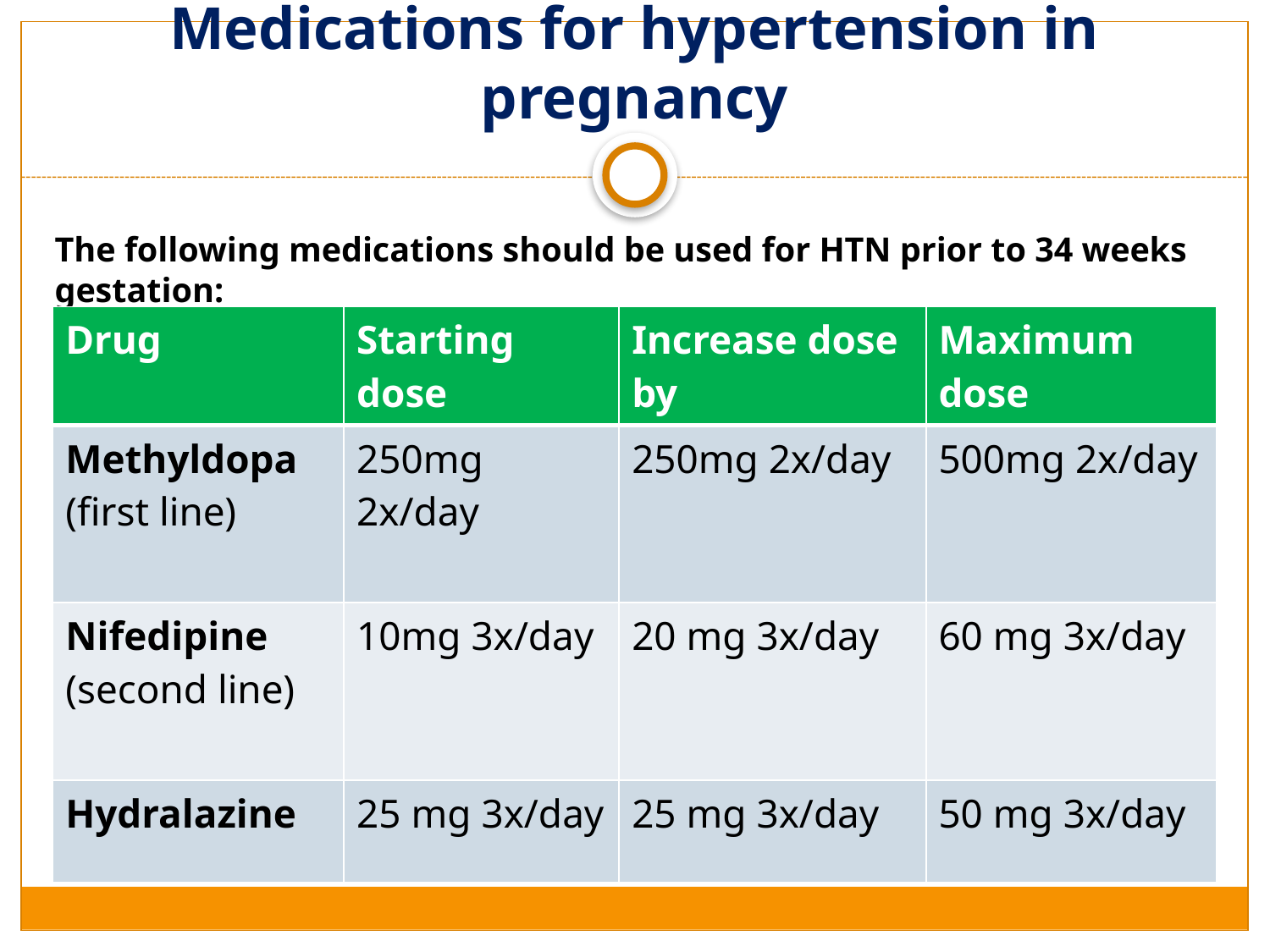

# Medications for hypertension in pregnancy
The following medications should be used for HTN prior to 34 weeks gestation:
| Drug | Starting dose | Increase dose by | Maximum dose |
| --- | --- | --- | --- |
| Methyldopa (first line) | 250mg 2x/day | 250mg 2x/day | 500mg 2x/day |
| Nifedipine (second line) | 10mg 3x/day | 20 mg 3x/day | 60 mg 3x/day |
| Hydralazine | 25 mg 3x/day | 25 mg 3x/day | 50 mg 3x/day |

## Slide 64
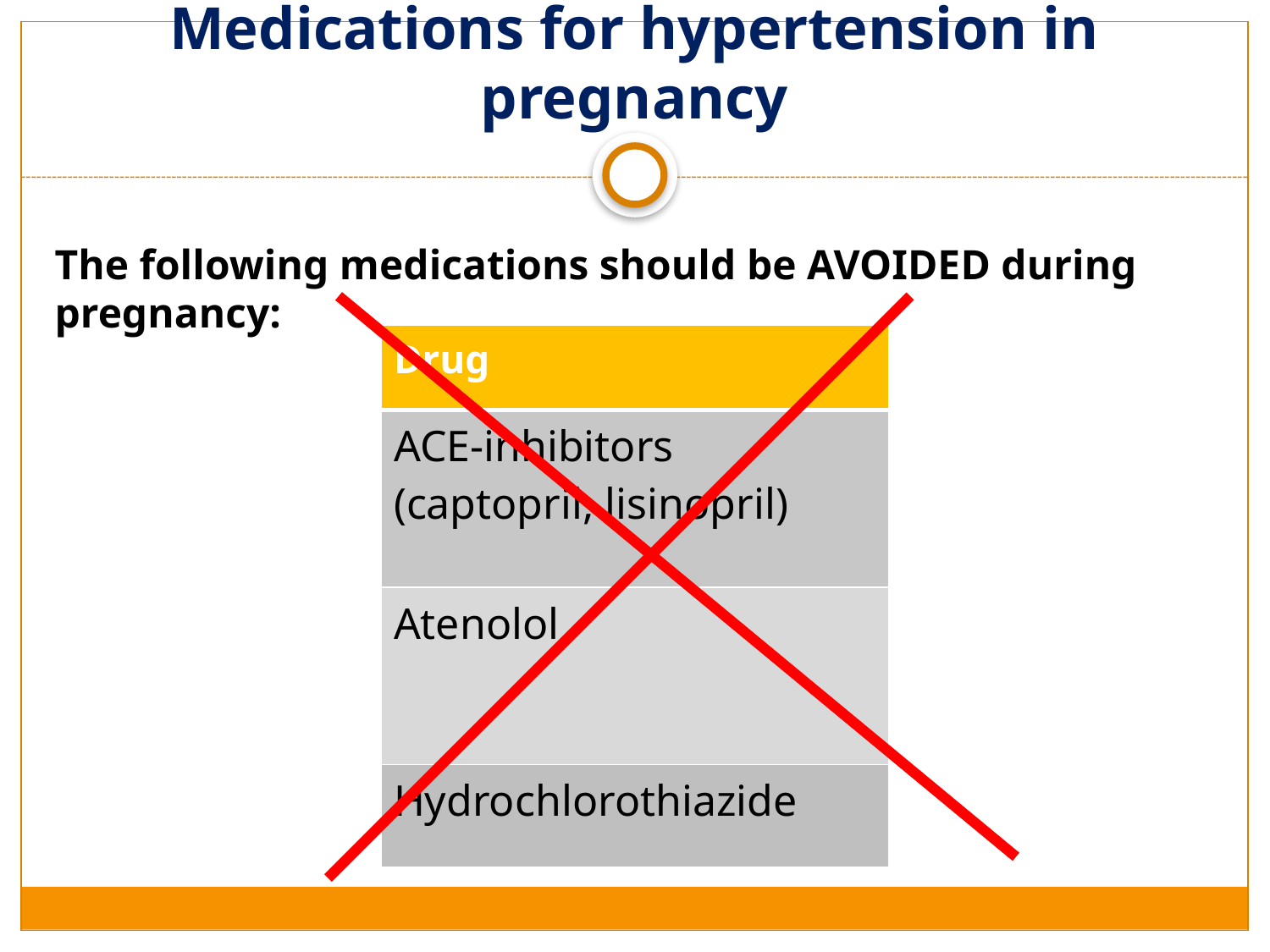

# Medications for hypertension in pregnancy
The following medications should be AVOIDED during pregnancy:
| Drug |
| --- |
| ACE-inhibitors (captopril, lisinopril) |
| Atenolol |
| Hydrochlorothiazide |

## Slide 65
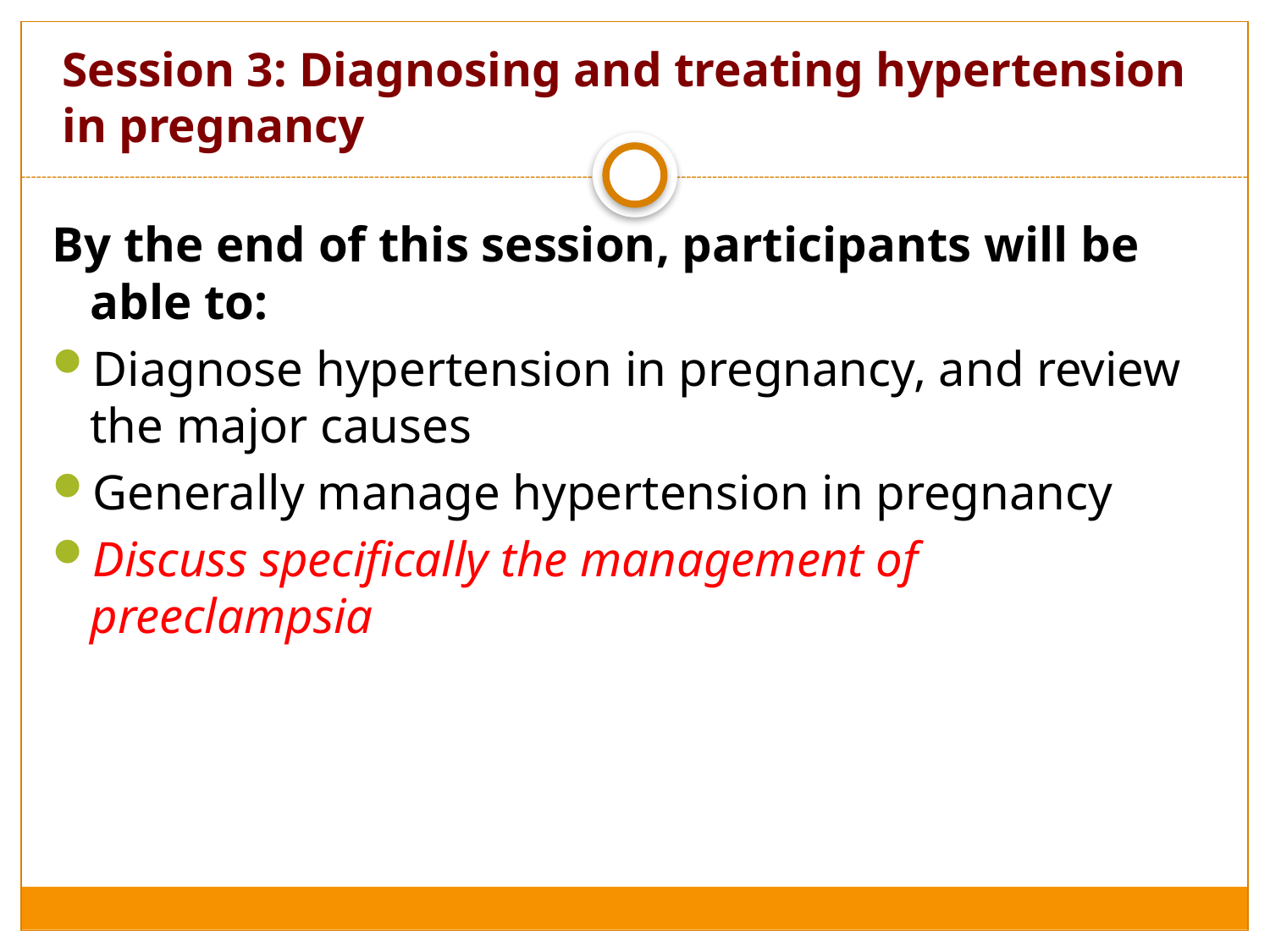

# Session 3: Diagnosing and treating hypertension in pregnancy
By the end of this session, participants will be able to:
Diagnose hypertension in pregnancy, and review the major causes
Generally manage hypertension in pregnancy
Discuss specifically the management of preeclampsia

## Slide 66
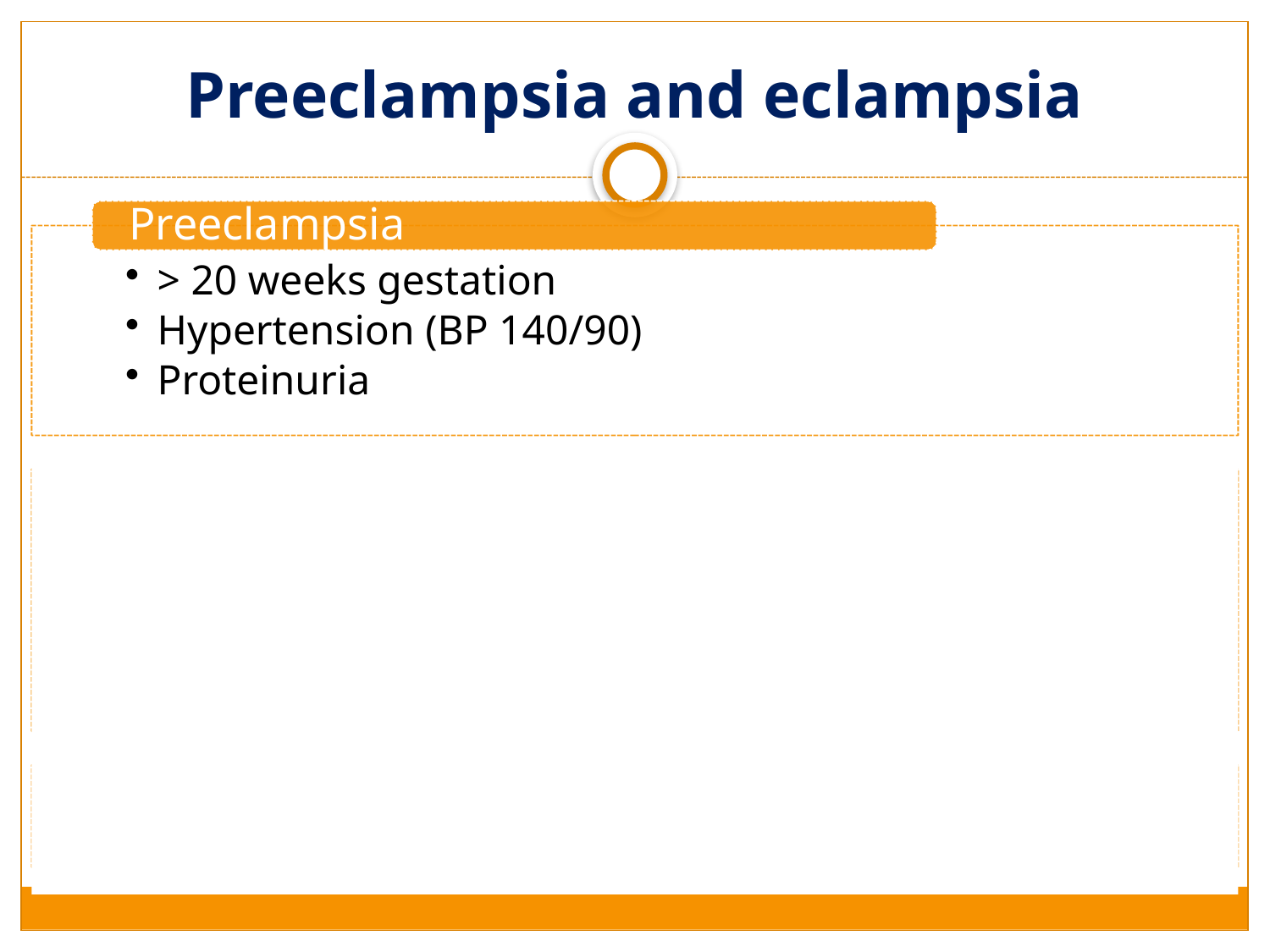

# Preeclampsia and eclampsia

## Slide 67
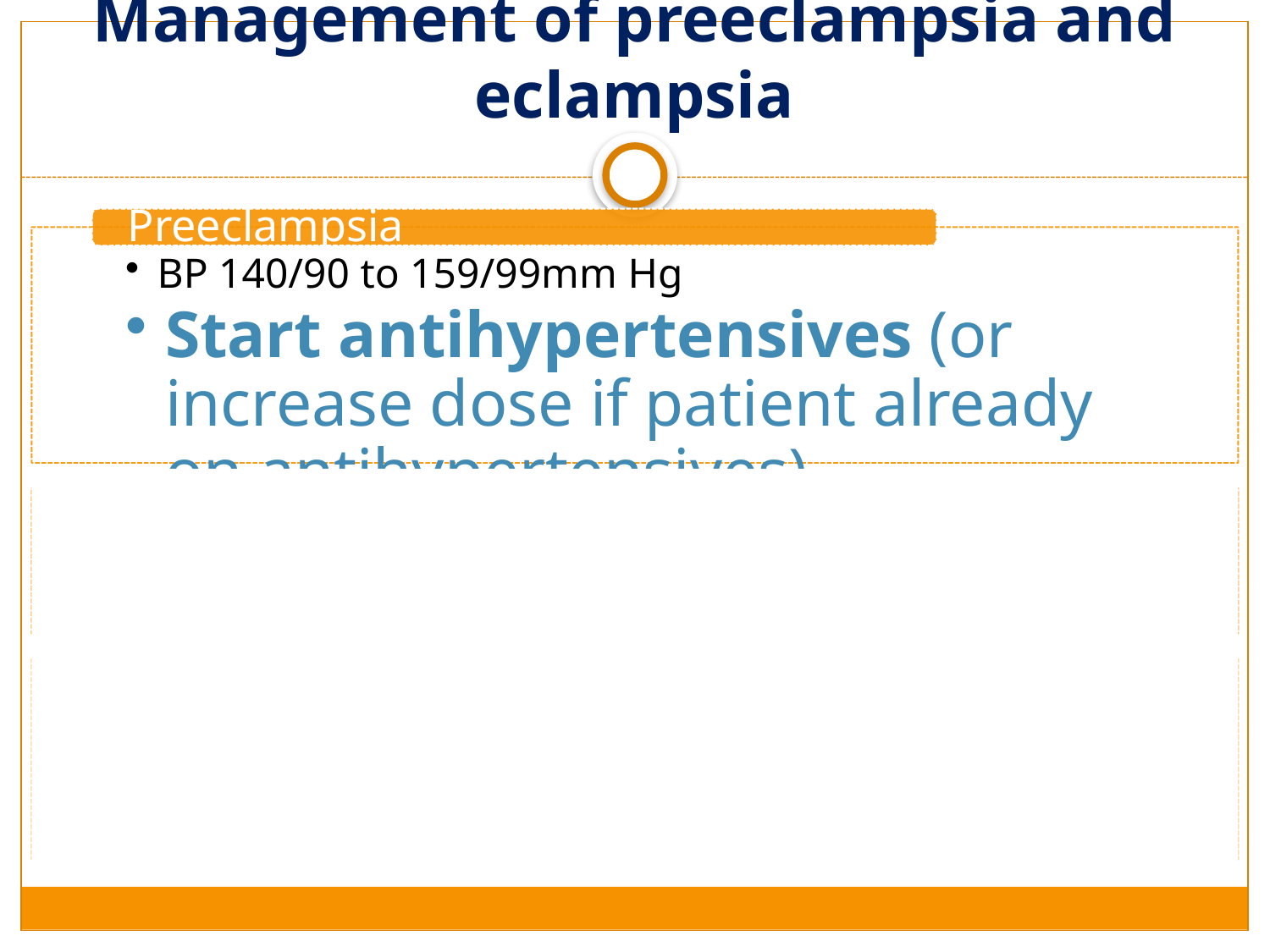

# Management of preeclampsia and eclampsia

## Slide 68
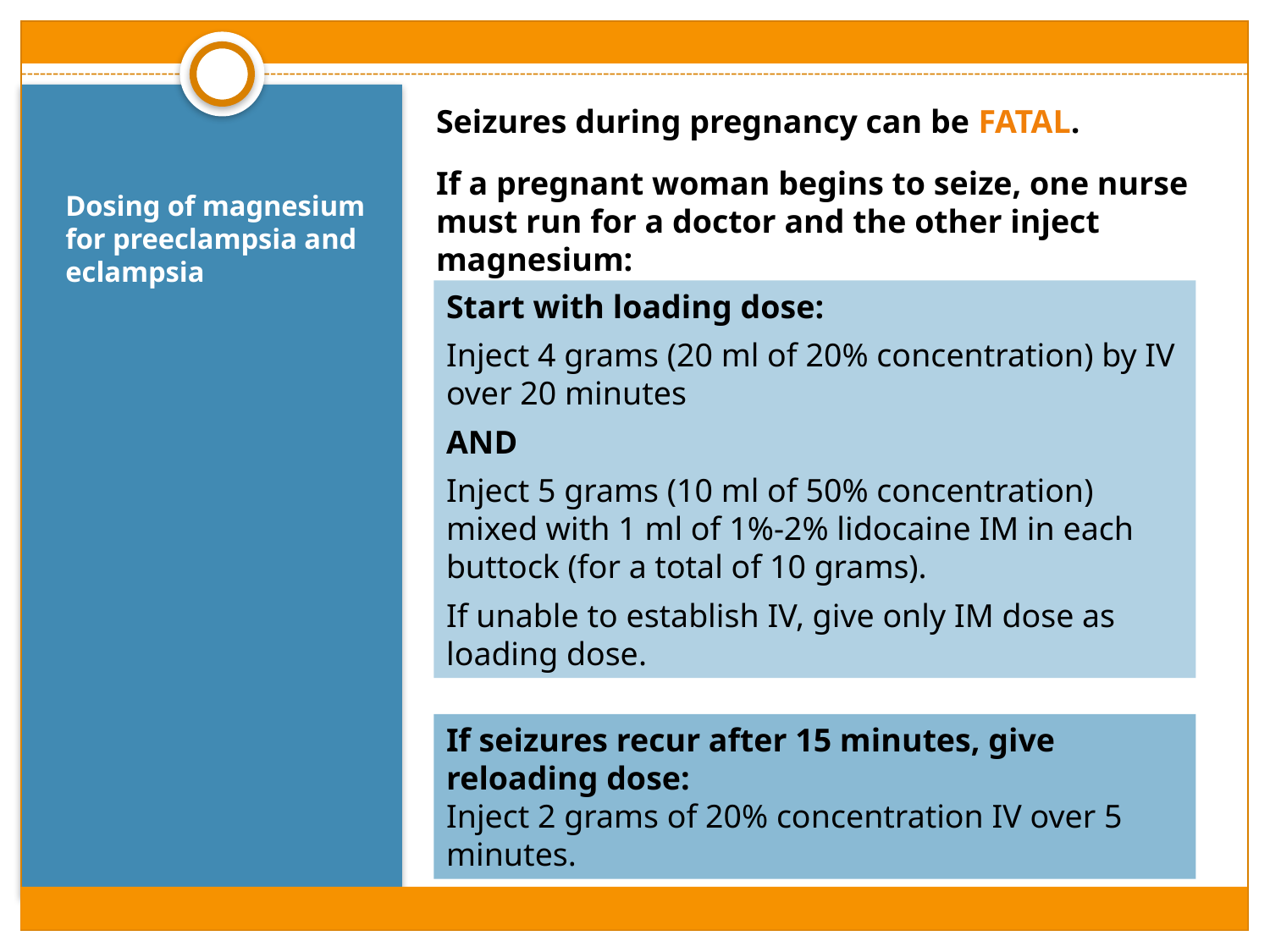

Seizures during pregnancy can be FATAL.
If a pregnant woman begins to seize, one nurse must run for a doctor and the other inject magnesium:
# Dosing of magnesium for preeclampsia and eclampsia
Start with loading dose:
Inject 4 grams (20 ml of 20% concentration) by IV over 20 minutes
AND
Inject 5 grams (10 ml of 50% concentration) mixed with 1 ml of 1%-2% lidocaine IM in each buttock (for a total of 10 grams).
If unable to establish IV, give only IM dose as loading dose.
If seizures recur after 15 minutes, give reloading dose:
Inject 2 grams of 20% concentration IV over 5 minutes.

## Slide 69
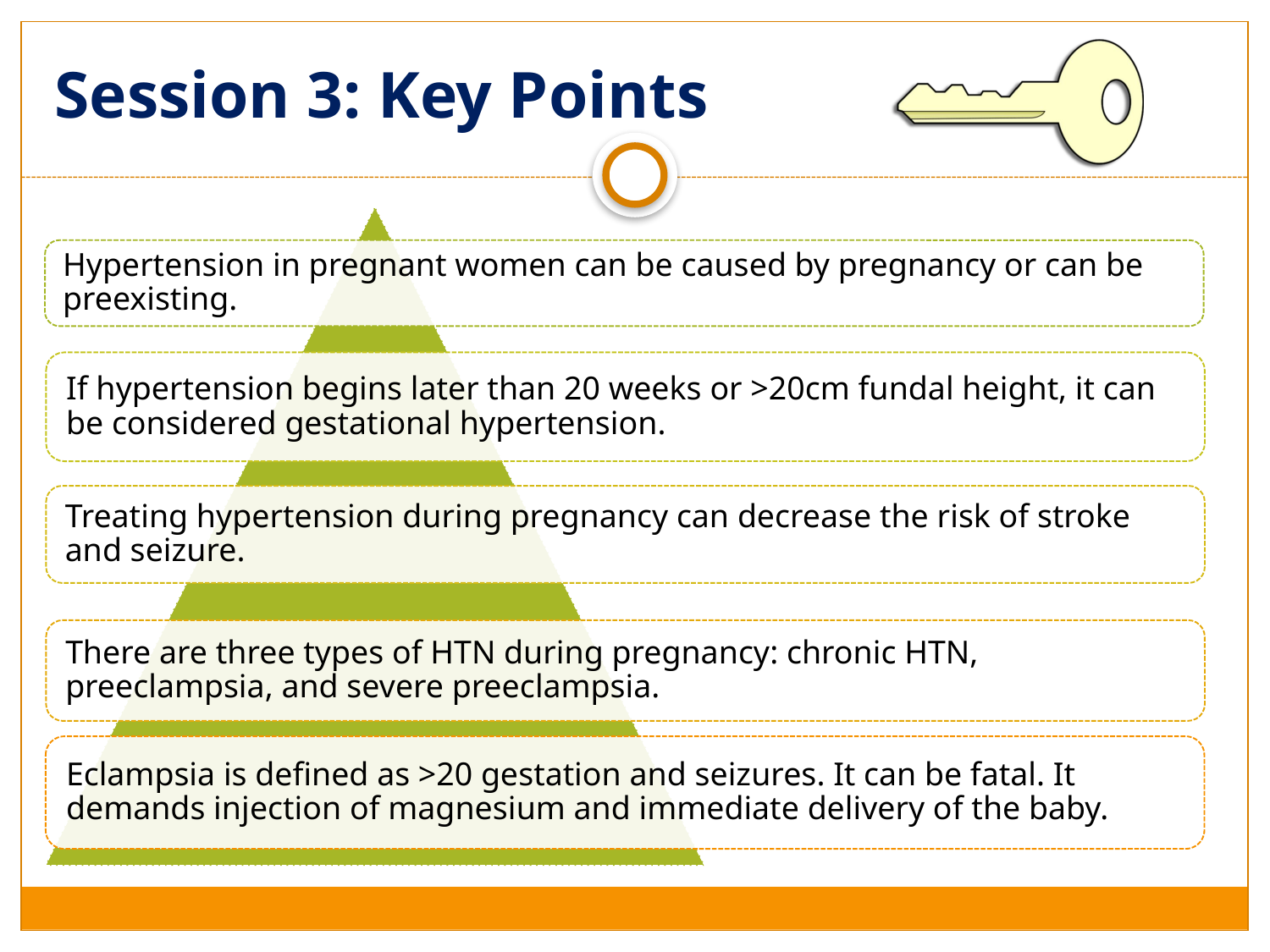

# Session 3: Key Points

## Slide 70
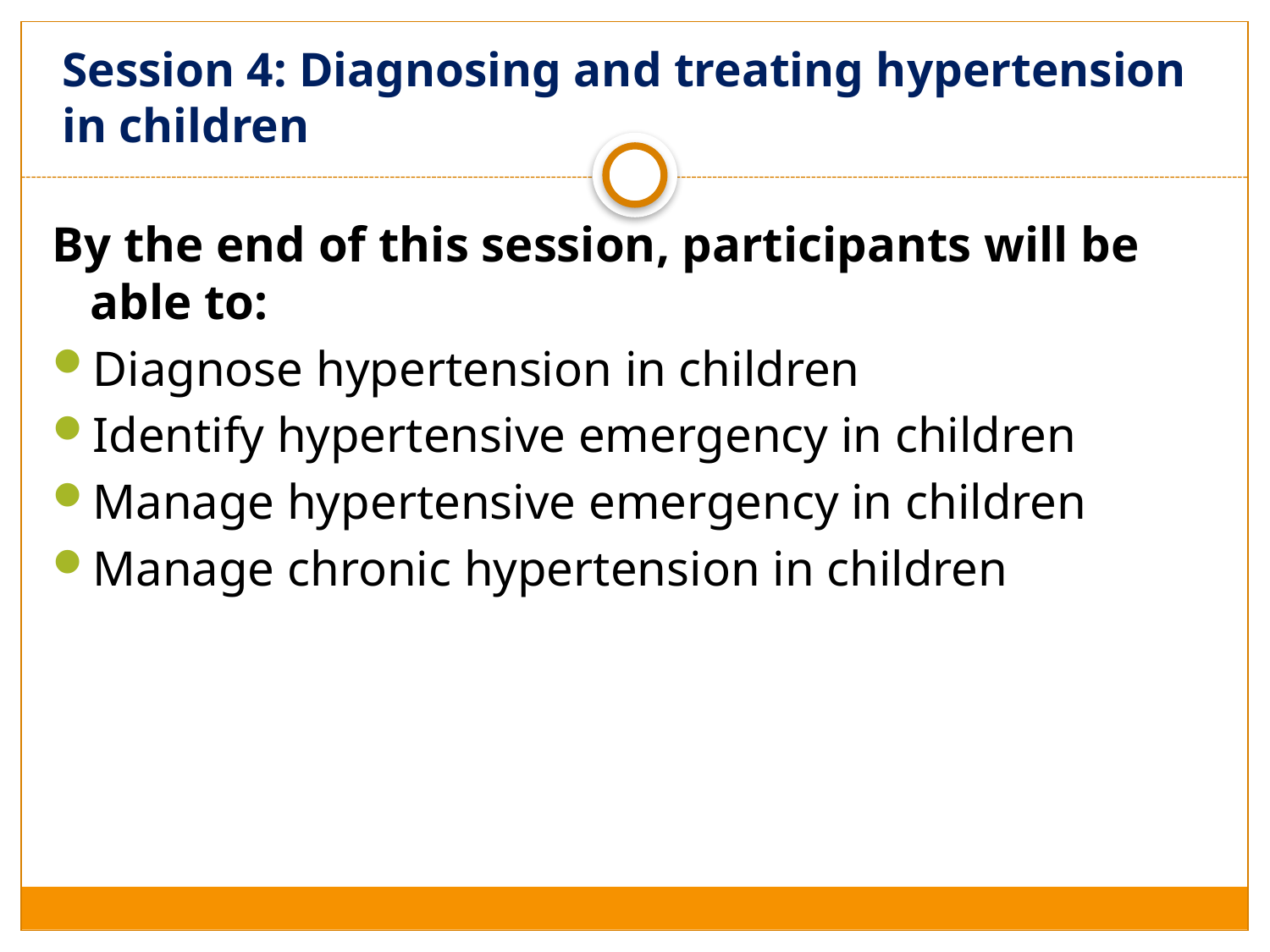

# Session 4: Diagnosing and treating hypertension in children
By the end of this session, participants will be able to:
Diagnose hypertension in children
Identify hypertensive emergency in children
Manage hypertensive emergency in children
Manage chronic hypertension in children

## Slide 71
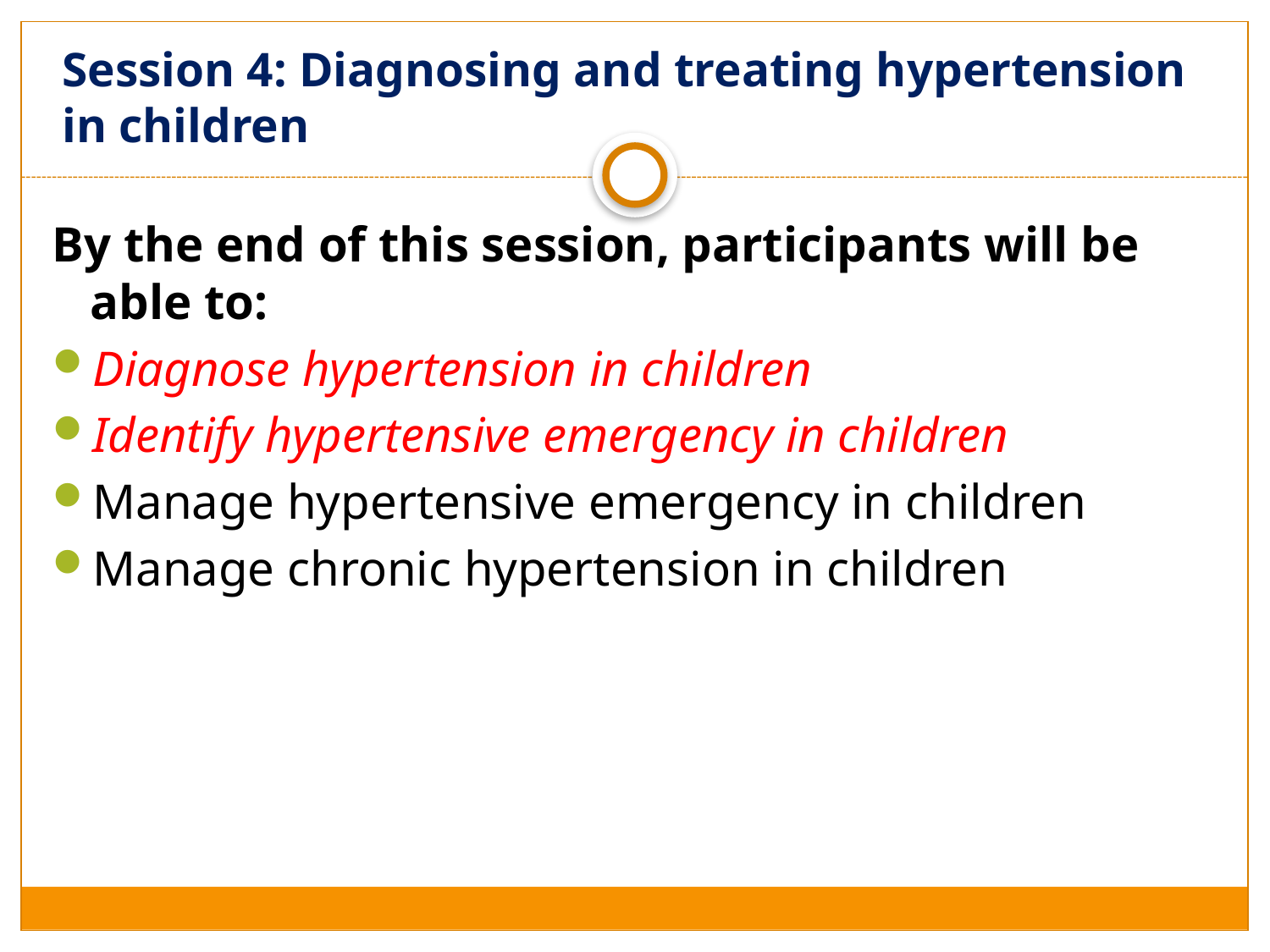

# Session 4: Diagnosing and treating hypertension in children
By the end of this session, participants will be able to:
Diagnose hypertension in children
Identify hypertensive emergency in children
Manage hypertensive emergency in children
Manage chronic hypertension in children

## Slide 72
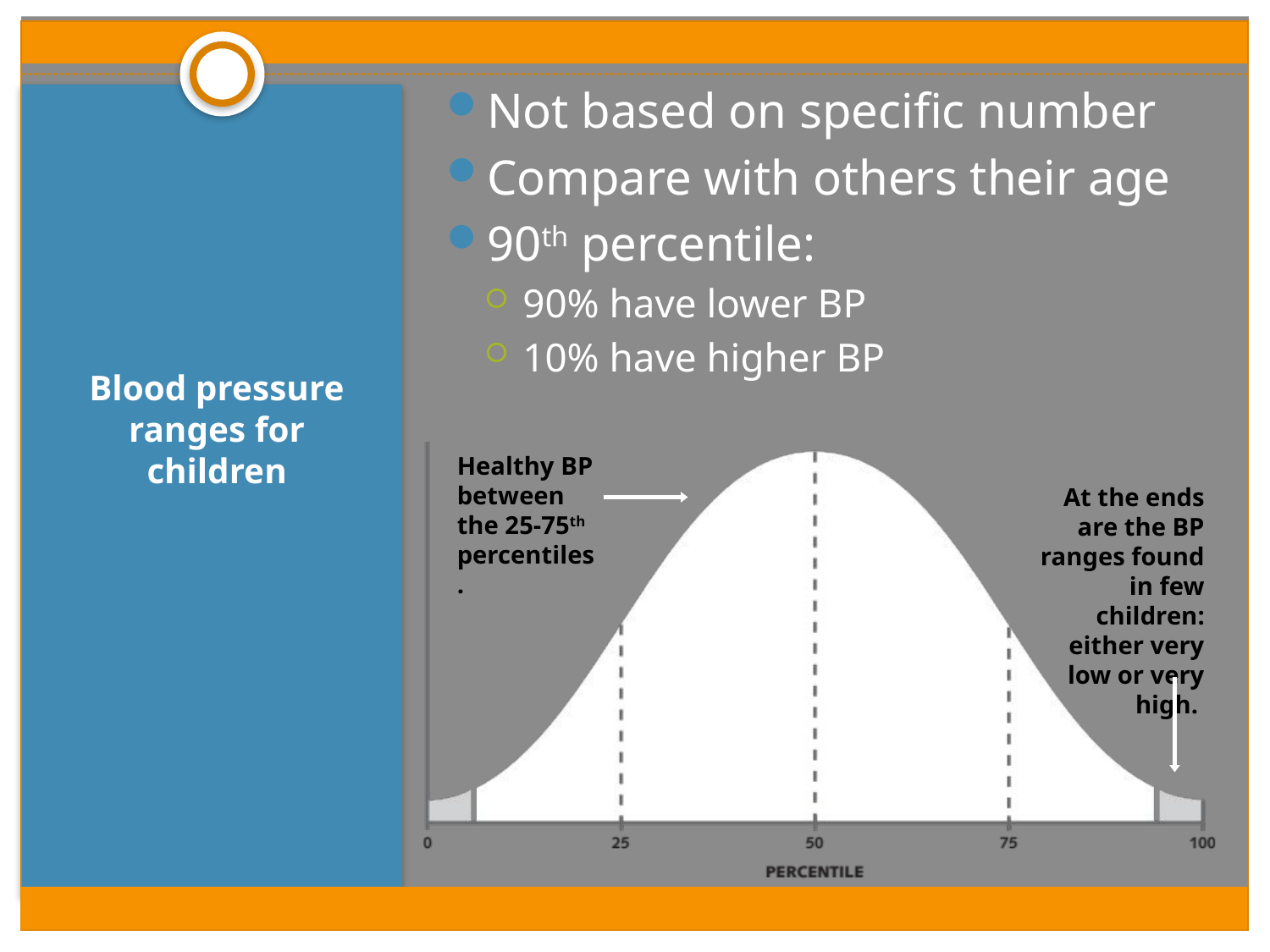

Not based on specific number
Compare with others their age
90th percentile:
90% have lower BP
10% have higher BP
# Blood pressure ranges for children
Healthy BP between the 25-75th percentiles.
At the ends are the BP ranges found in few children: either very low or very high.

## Slide 73
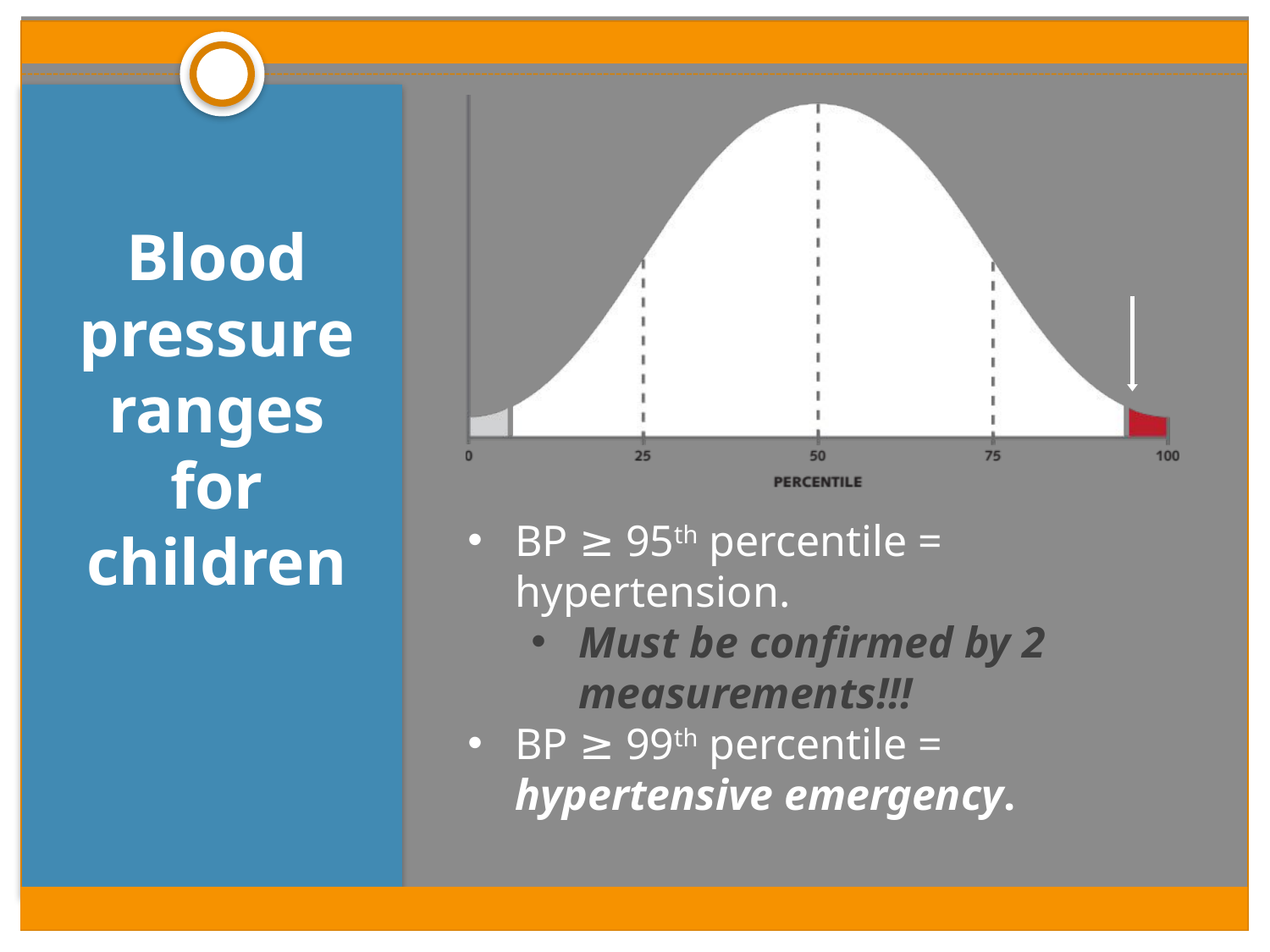

# Blood pressure ranges for children
BP ≥ 95th percentile = hypertension.
Must be confirmed by 2 measurements!!!
BP ≥ 99th percentile = hypertensive emergency.

## Slide 74
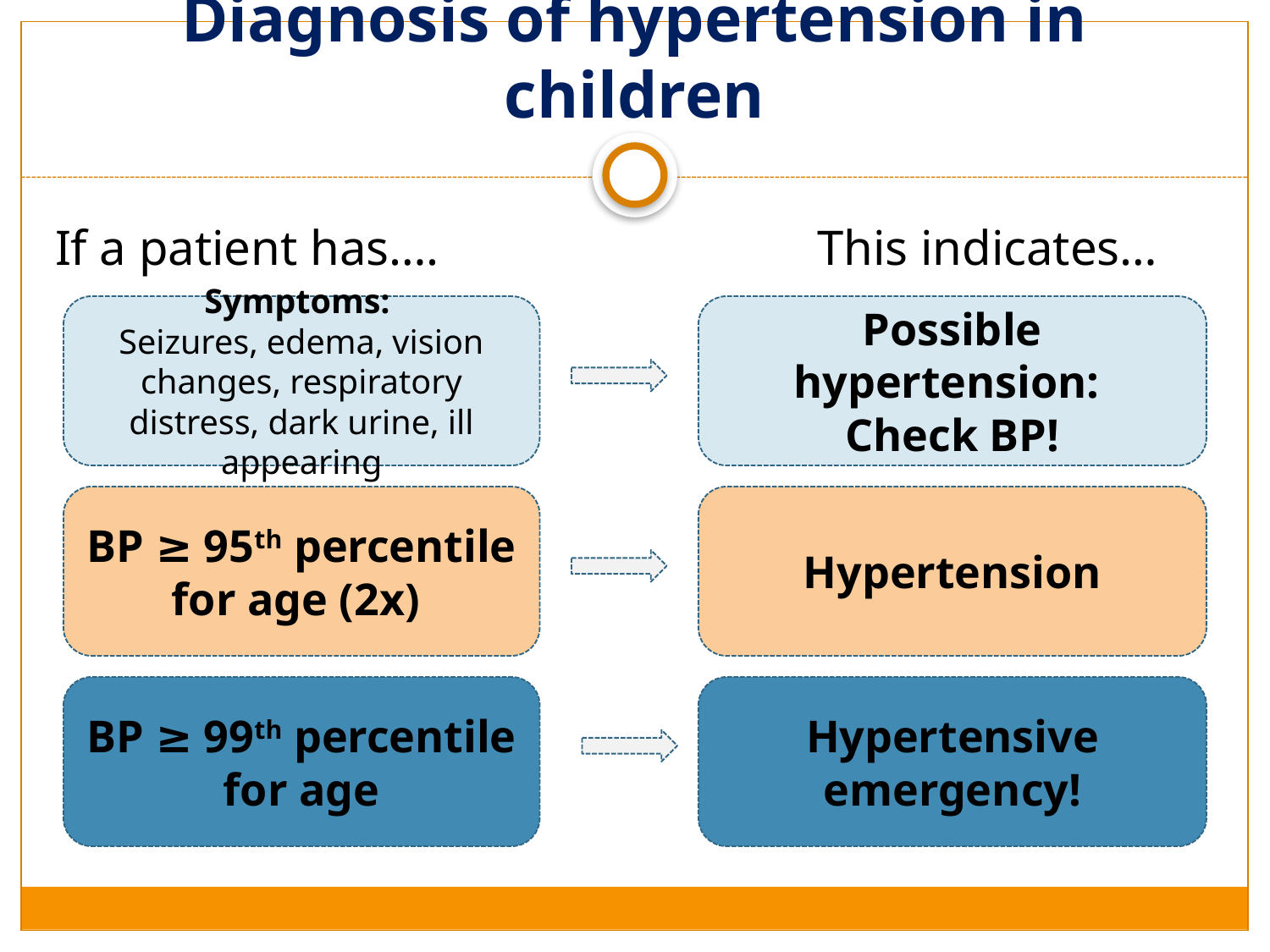

# Diagnosis of hypertension in children
If a patient has….			This indicates…
Symptoms:
Seizures, edema, vision changes, respiratory distress, dark urine, ill appearing
Possible hypertension:
Check BP!
BP ≥ 95th percentile
for age (2x)
Hypertension
BP ≥ 99th percentile
for age
Hypertensive emergency!

## Slide 75
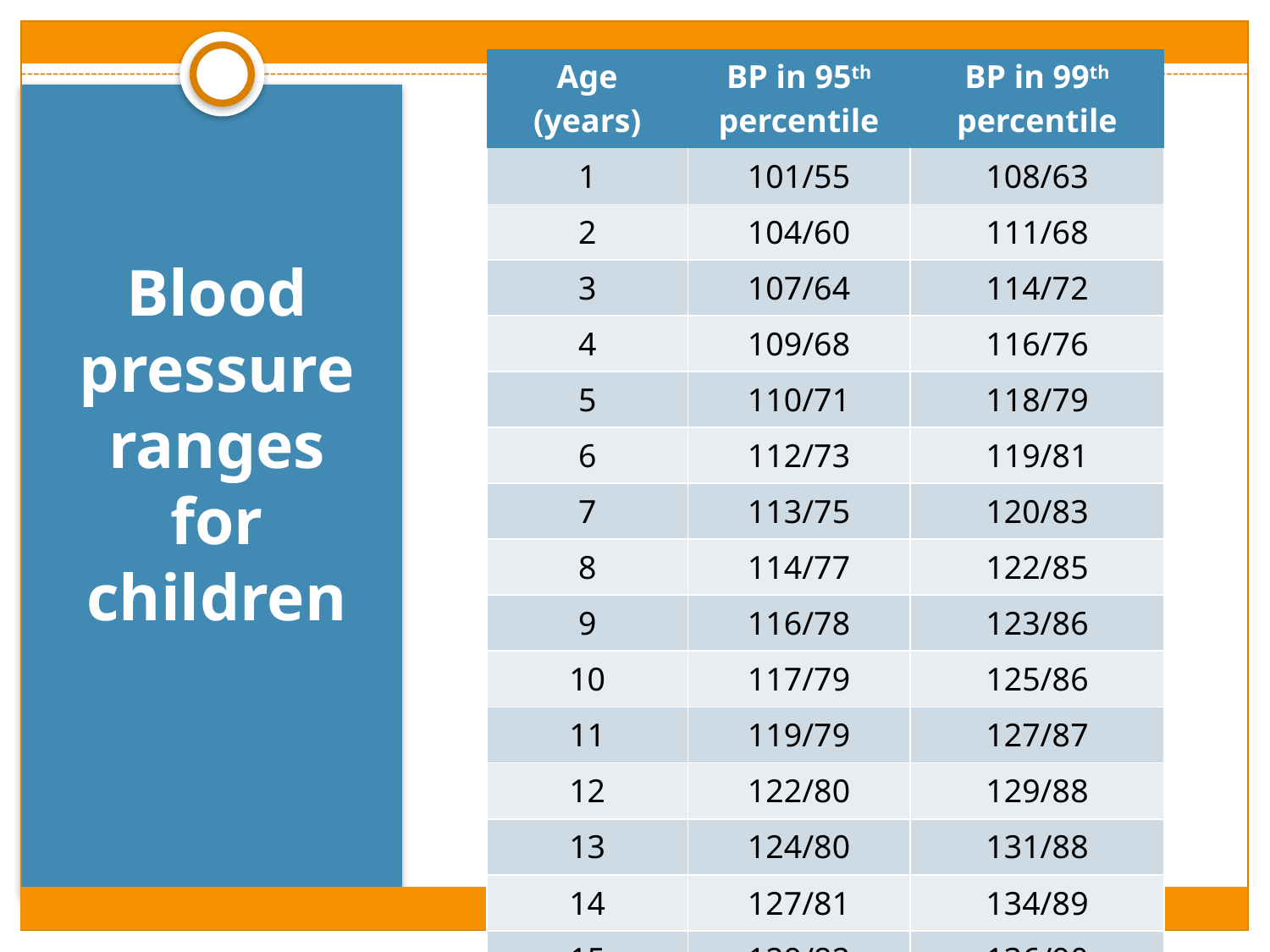

| Age (years) | BP in 95th percentile | BP in 99th percentile |
| --- | --- | --- |
| 1 | 101/55 | 108/63 |
| 2 | 104/60 | 111/68 |
| 3 | 107/64 | 114/72 |
| 4 | 109/68 | 116/76 |
| 5 | 110/71 | 118/79 |
| 6 | 112/73 | 119/81 |
| 7 | 113/75 | 120/83 |
| 8 | 114/77 | 122/85 |
| 9 | 116/78 | 123/86 |
| 10 | 117/79 | 125/86 |
| 11 | 119/79 | 127/87 |
| 12 | 122/80 | 129/88 |
| 13 | 124/80 | 131/88 |
| 14 | 127/81 | 134/89 |
| 15 | 129/82 | 136/90 |
# Blood pressure ranges for children

## Slide 76
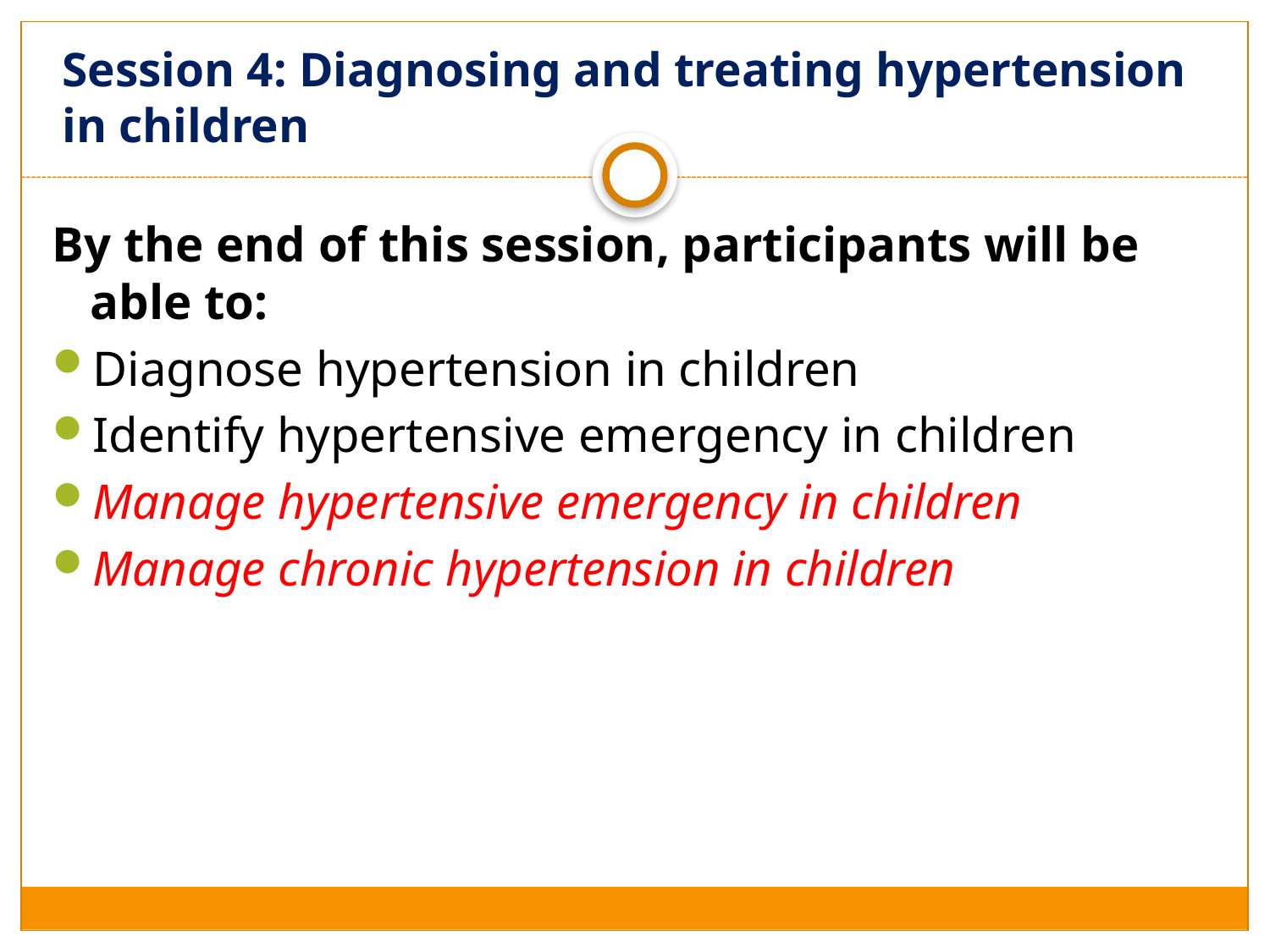

# Session 4: Diagnosing and treating hypertension in children
By the end of this session, participants will be able to:
Diagnose hypertension in children
Identify hypertensive emergency in children
Manage hypertensive emergency in children
Manage chronic hypertension in children

## Slide 77
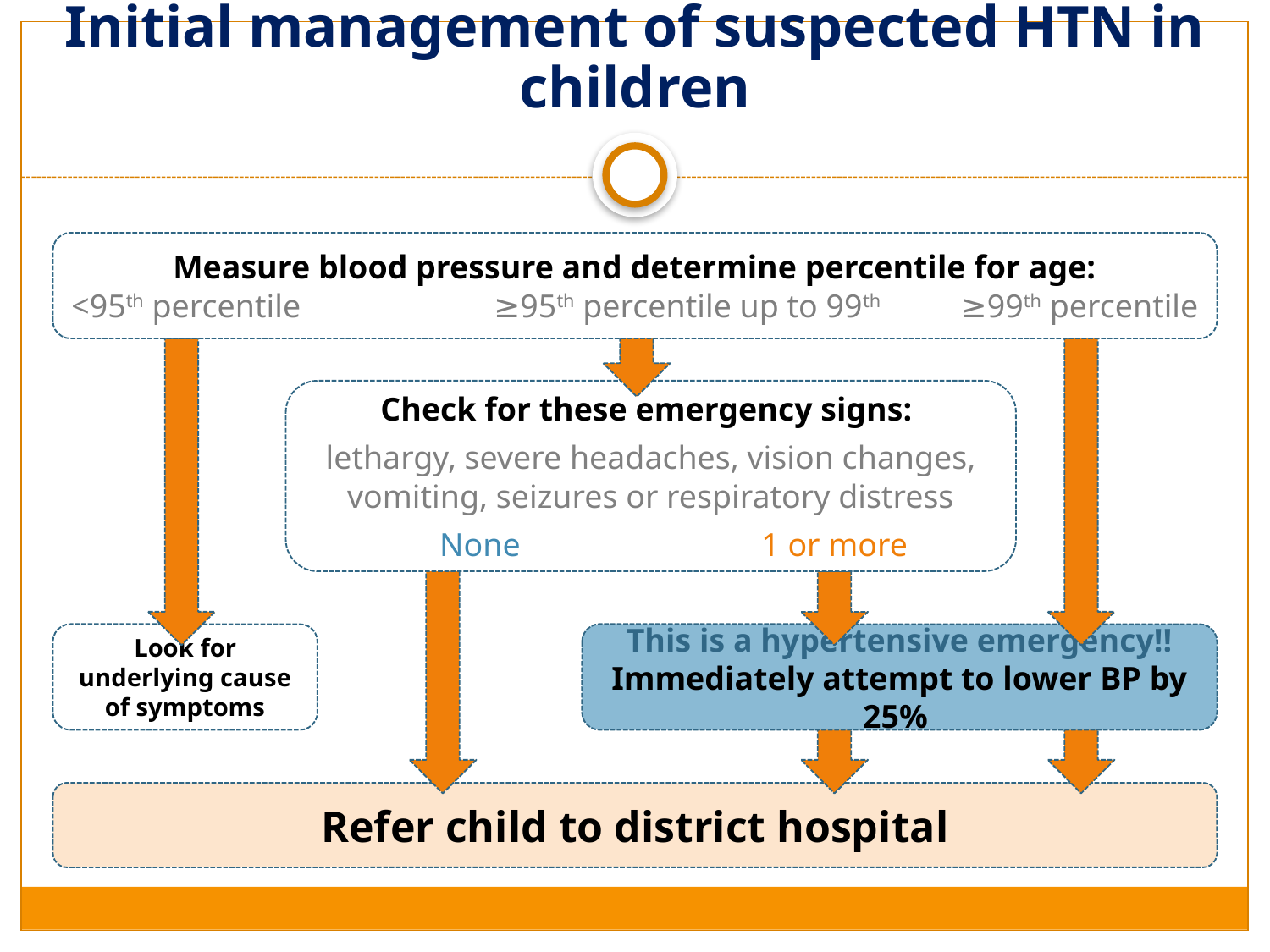

# Initial management of suspected HTN in children
Measure blood pressure and determine percentile for age:
<95th percentile 		 ≥95th percentile up to 99th 	≥99th percentile
Check for these emergency signs:
lethargy, severe headaches, vision changes, vomiting, seizures or respiratory distress
 None
1 or more
Look for underlying cause of symptoms
This is a hypertensive emergency!!
Immediately attempt to lower BP by 25%
Refer child to district hospital

## Slide 78
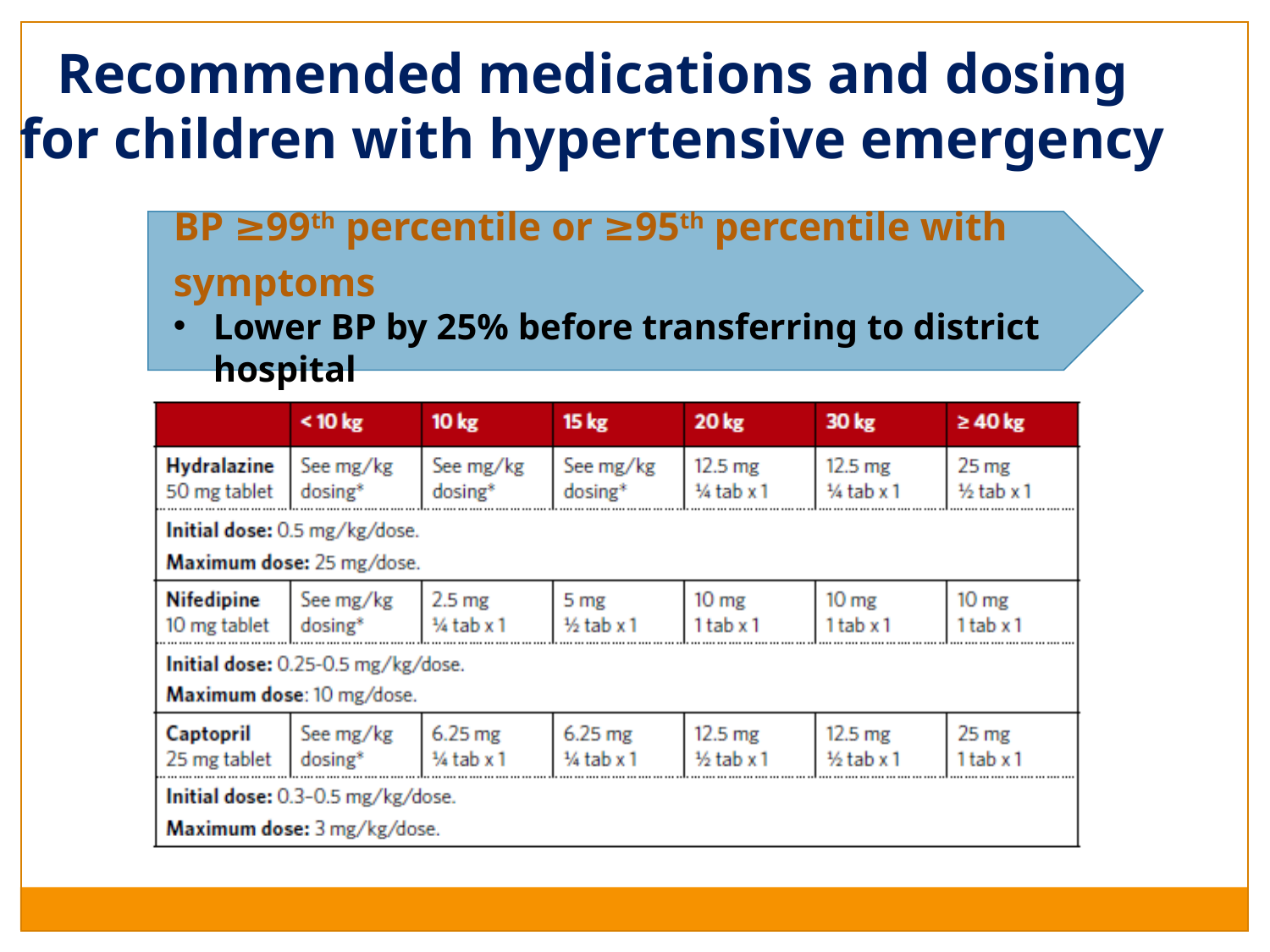

Recommended medications and dosing for children with hypertensive emergency
BP ≥99th percentile or ≥95th percentile with symptoms
Lower BP by 25% before transferring to district hospital

## Slide 79
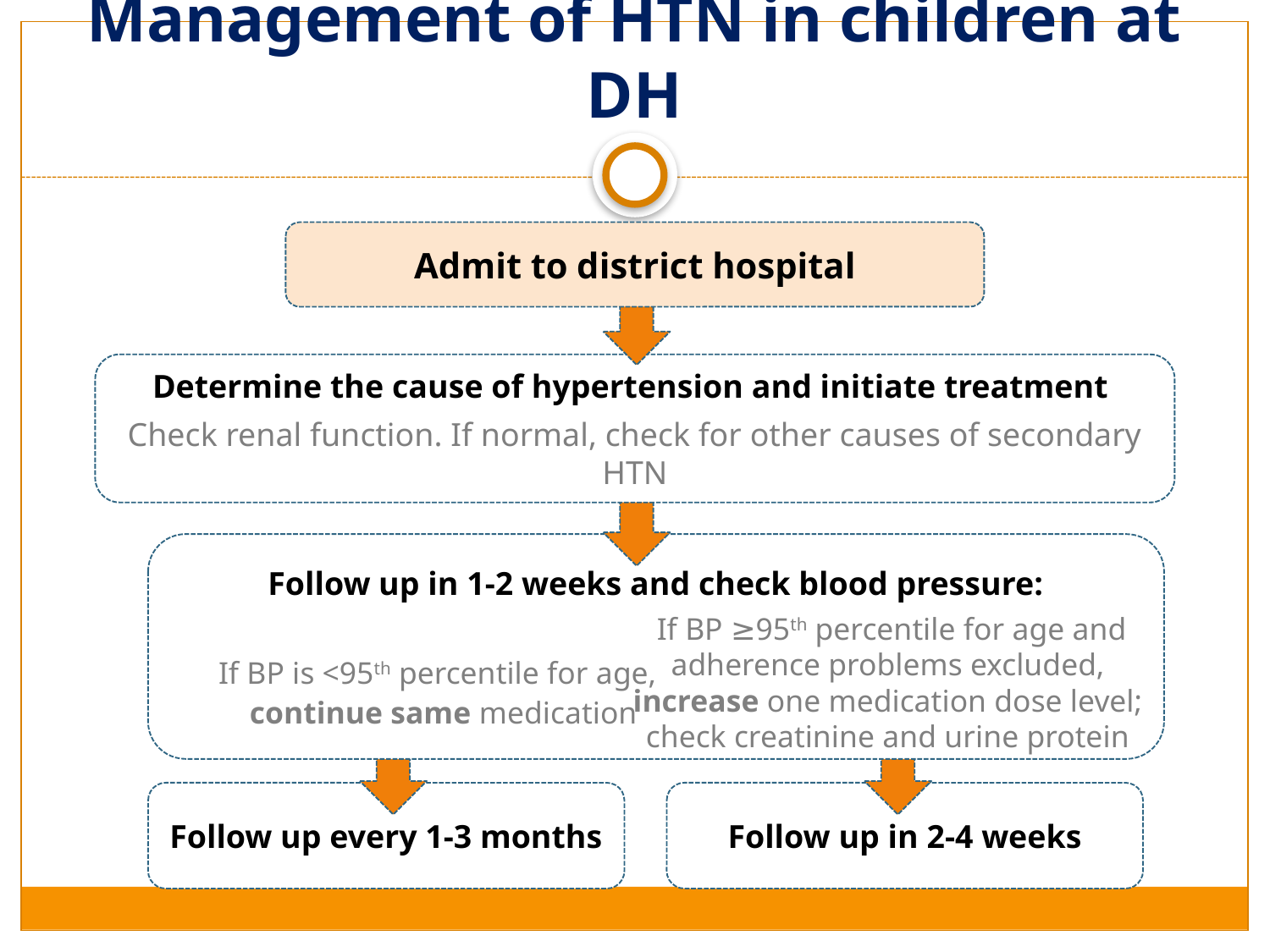

# Management of HTN in children at DH
Admit to district hospital
Determine the cause of hypertension and initiate treatment
Check renal function. If normal, check for other causes of secondary HTN
Follow up in 1-2 weeks and check blood pressure:
 If BP is <95th percentile for age,
 continue same medication 	 If BP ≥95th percentile for age
 If BP ≥95th percentile for age and adherence problems excluded,
increase one medication dose level; check creatinine and urine protein
Follow up every 1-3 months
Follow up in 2-4 weeks

## Slide 80
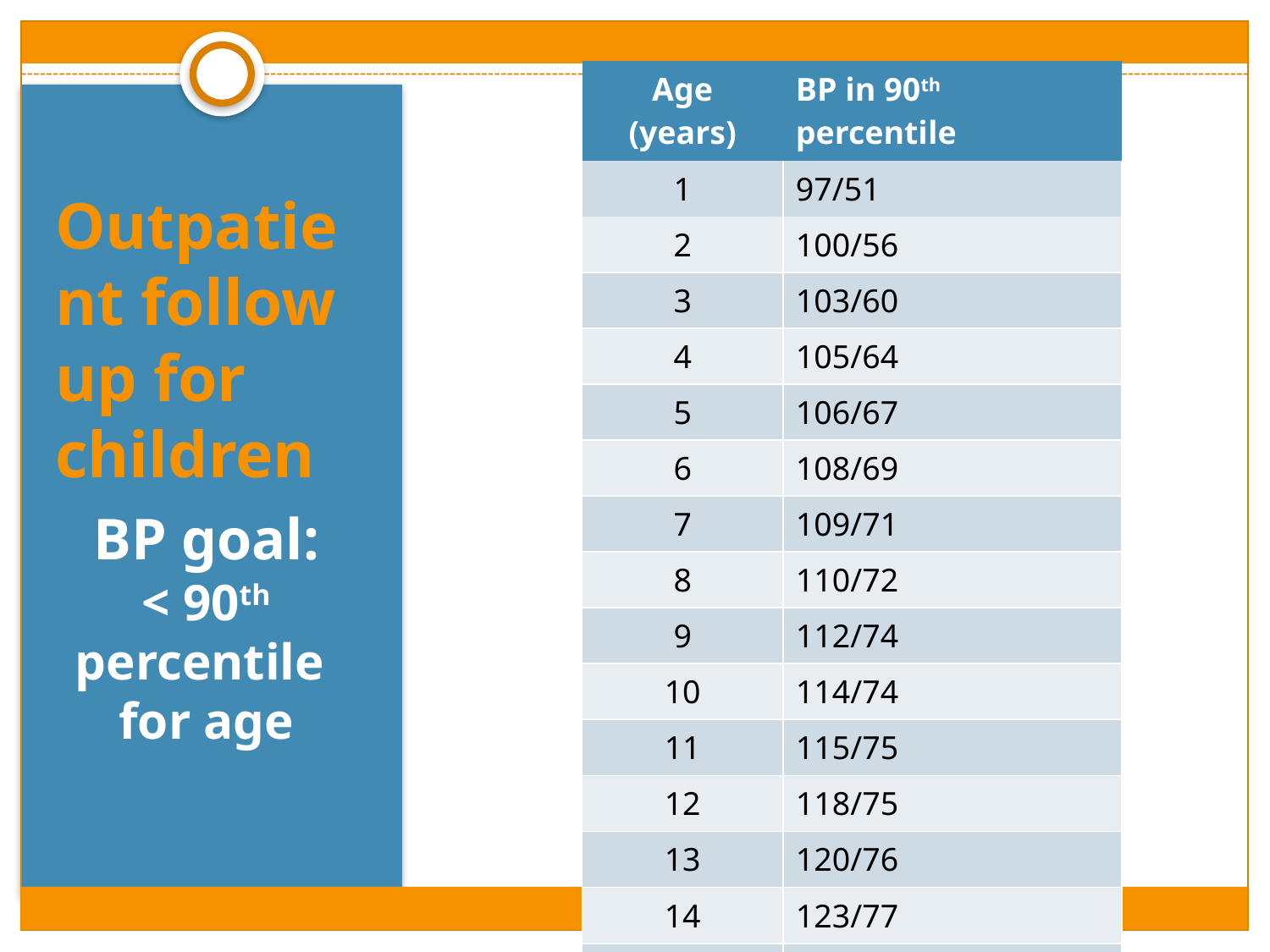

| Age (years) | BP in 90th percentile |
| --- | --- |
| 1 | 97/51 |
| 2 | 100/56 |
| 3 | 103/60 |
| 4 | 105/64 |
| 5 | 106/67 |
| 6 | 108/69 |
| 7 | 109/71 |
| 8 | 110/72 |
| 9 | 112/74 |
| 10 | 114/74 |
| 11 | 115/75 |
| 12 | 118/75 |
| 13 | 120/76 |
| 14 | 123/77 |
| 15 | 125/78 |
Outpatient follow up for children
BP goal:
< 90th percentile
for age

## Slide 81
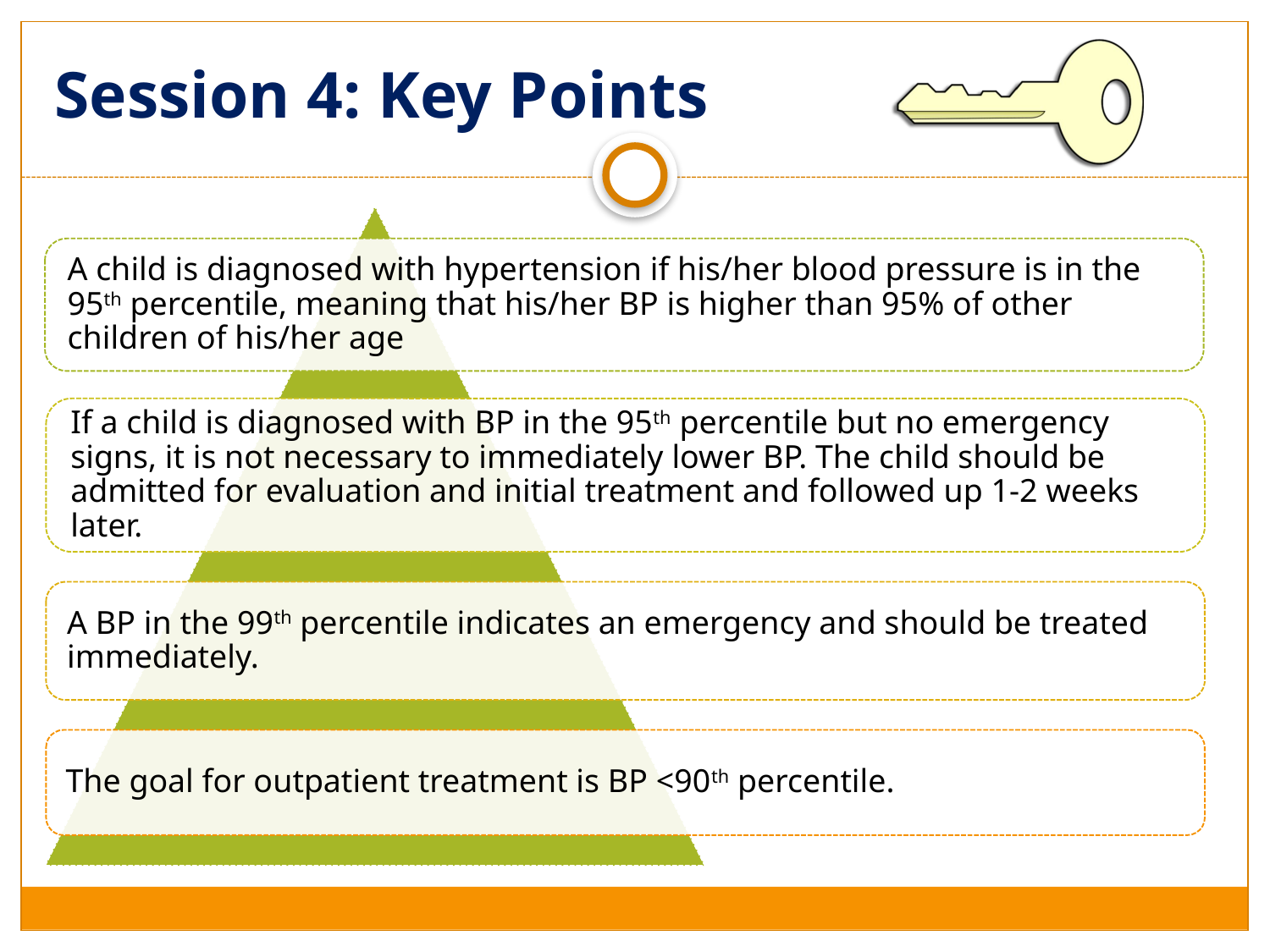

# Session 4: Key Points
