# Supplementary material for: Training Mid-Level Providers to Treat Severe Non-Communicable Diseases in Neno, Malawi through PEN-Plus Strategies
Source: Ann Glob Health. 2022 Aug 11;88(1):69. doi: 10.5334/aogh.3750 (PMC9389951; doi:10.5334/aogh.3750)
Supplement: Didactic Materials. — The supplementary materials contain a suggested didactic training schedule and the PowerPoint presentations used for PEN-Plus training in Neno, Malawi. These materials have been reviewed and accepted by the Malawi Ministry of Health for future PEN-Plus trainings in Malawi. [file agh-88-1-3750-s2.zip › Didactic_Materials/DM_Complications.pptx]

## Slide 1
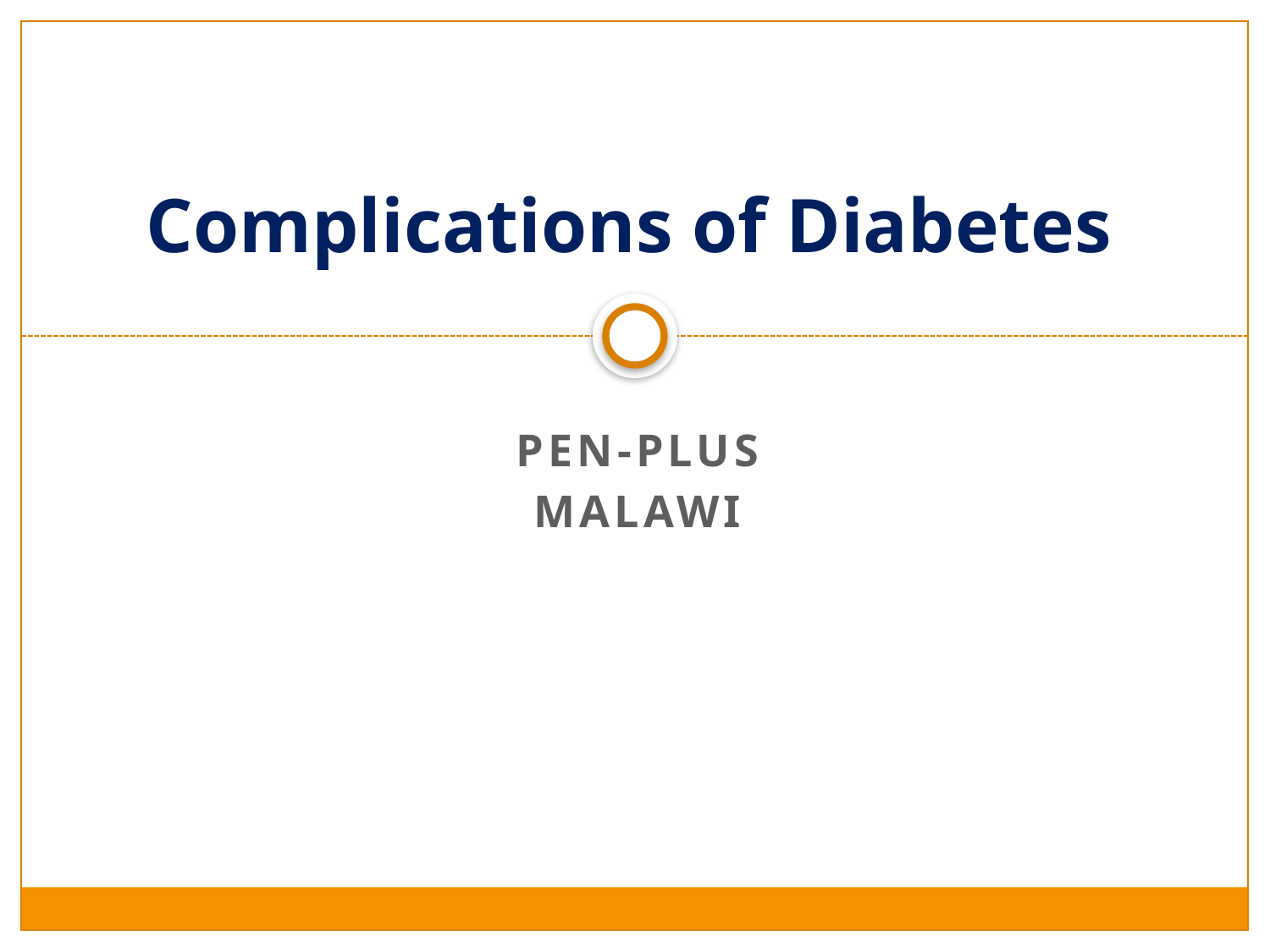

# Complications of Diabetes
PEN-Plus
Malawi

## Slide 2
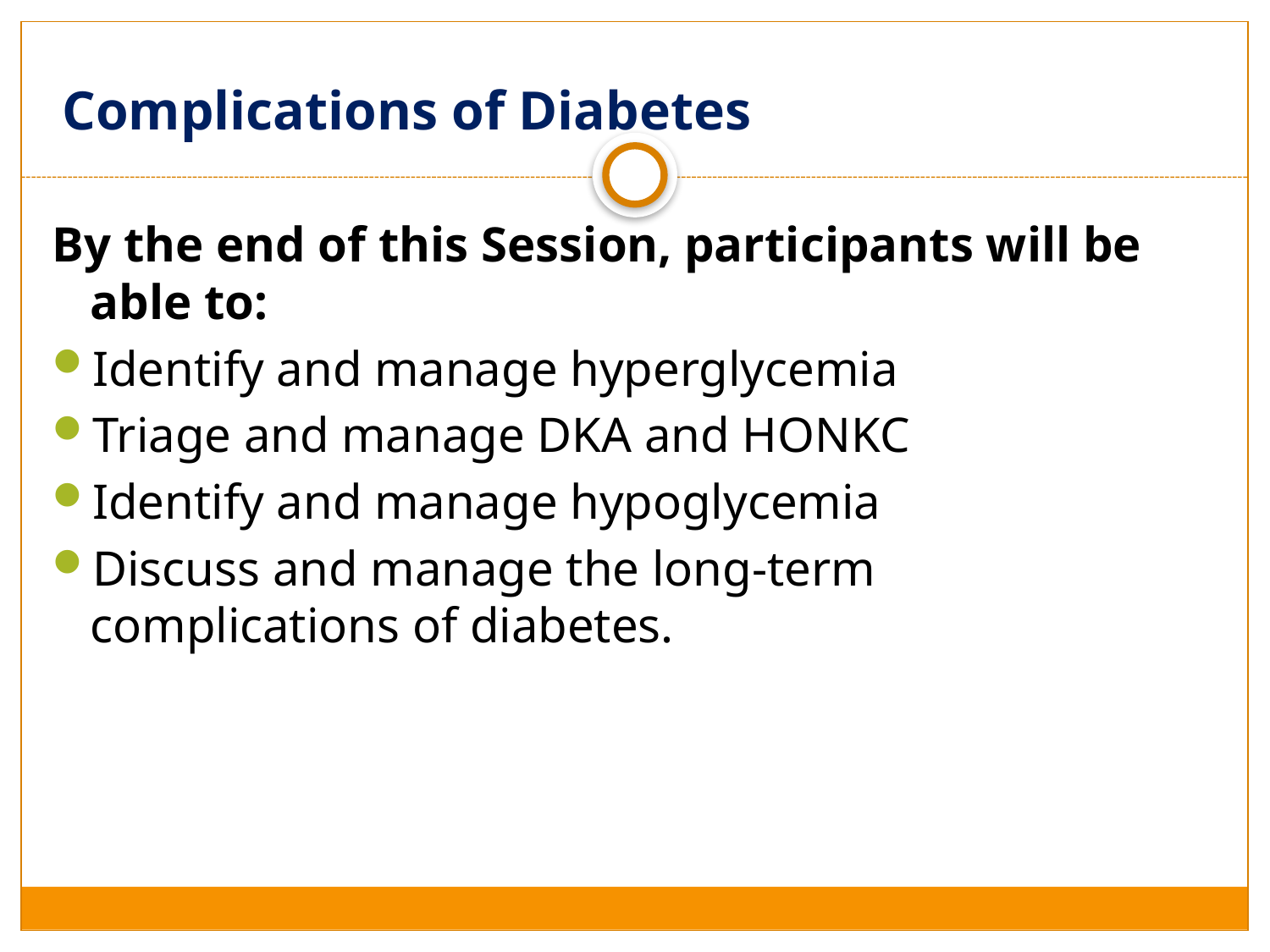

# Complications of Diabetes
By the end of this Session, participants will be able to:
Identify and manage hyperglycemia
Triage and manage DKA and HONKC
Identify and manage hypoglycemia
Discuss and manage the long-term complications of diabetes.

## Slide 3
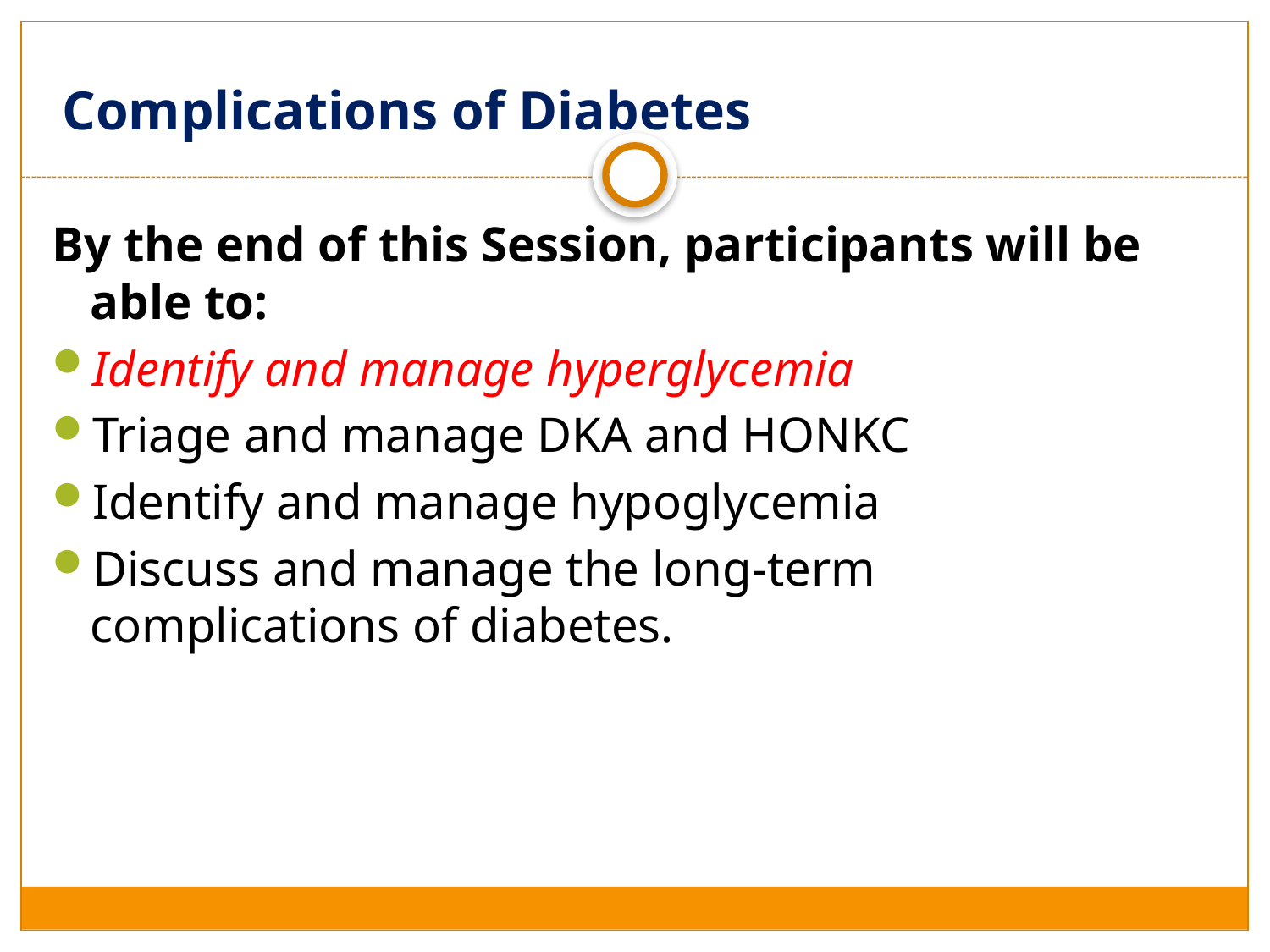

# Complications of Diabetes
By the end of this Session, participants will be able to:
Identify and manage hyperglycemia
Triage and manage DKA and HONKC
Identify and manage hypoglycemia
Discuss and manage the long-term complications of diabetes.

## Slide 4
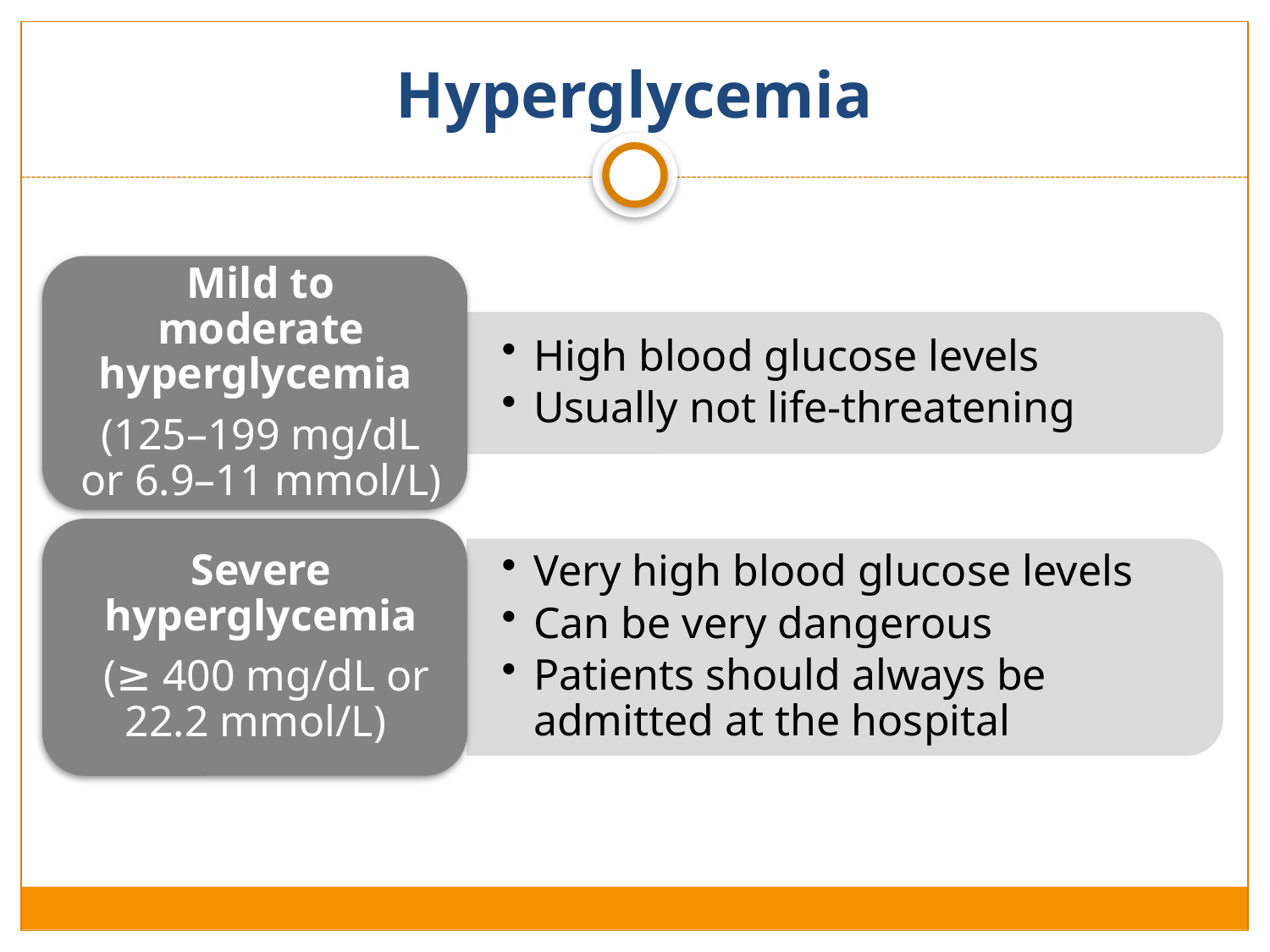

# Hyperglycemia

## Slide 5
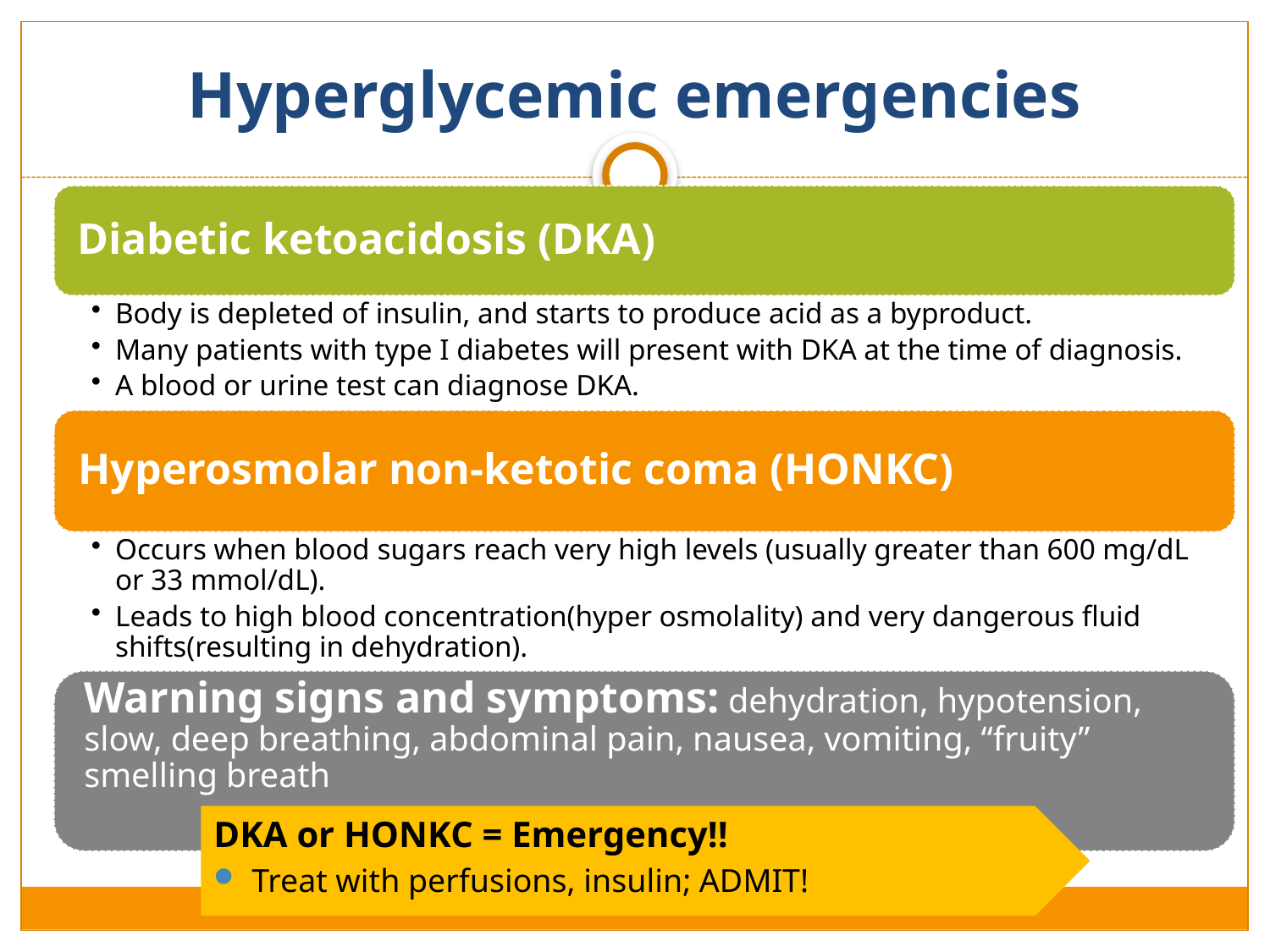

# Hyperglycemic emergencies
DKA or HONKC = Emergency!!
Treat with perfusions, insulin; ADMIT!

## Slide 6
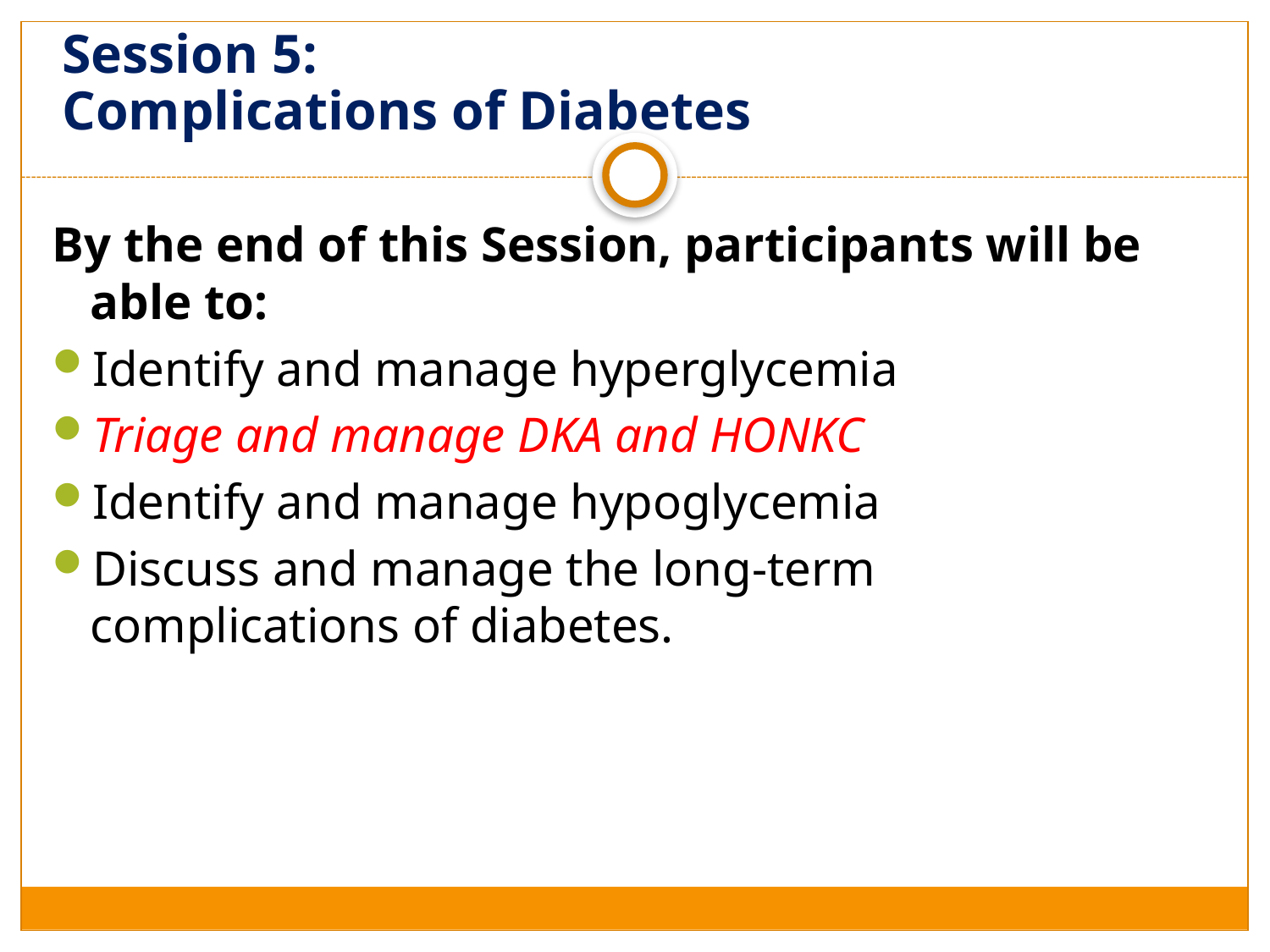

# Session 5: Complications of Diabetes
By the end of this Session, participants will be able to:
Identify and manage hyperglycemia
Triage and manage DKA and HONKC
Identify and manage hypoglycemia
Discuss and manage the long-term complications of diabetes.

## Slide 7
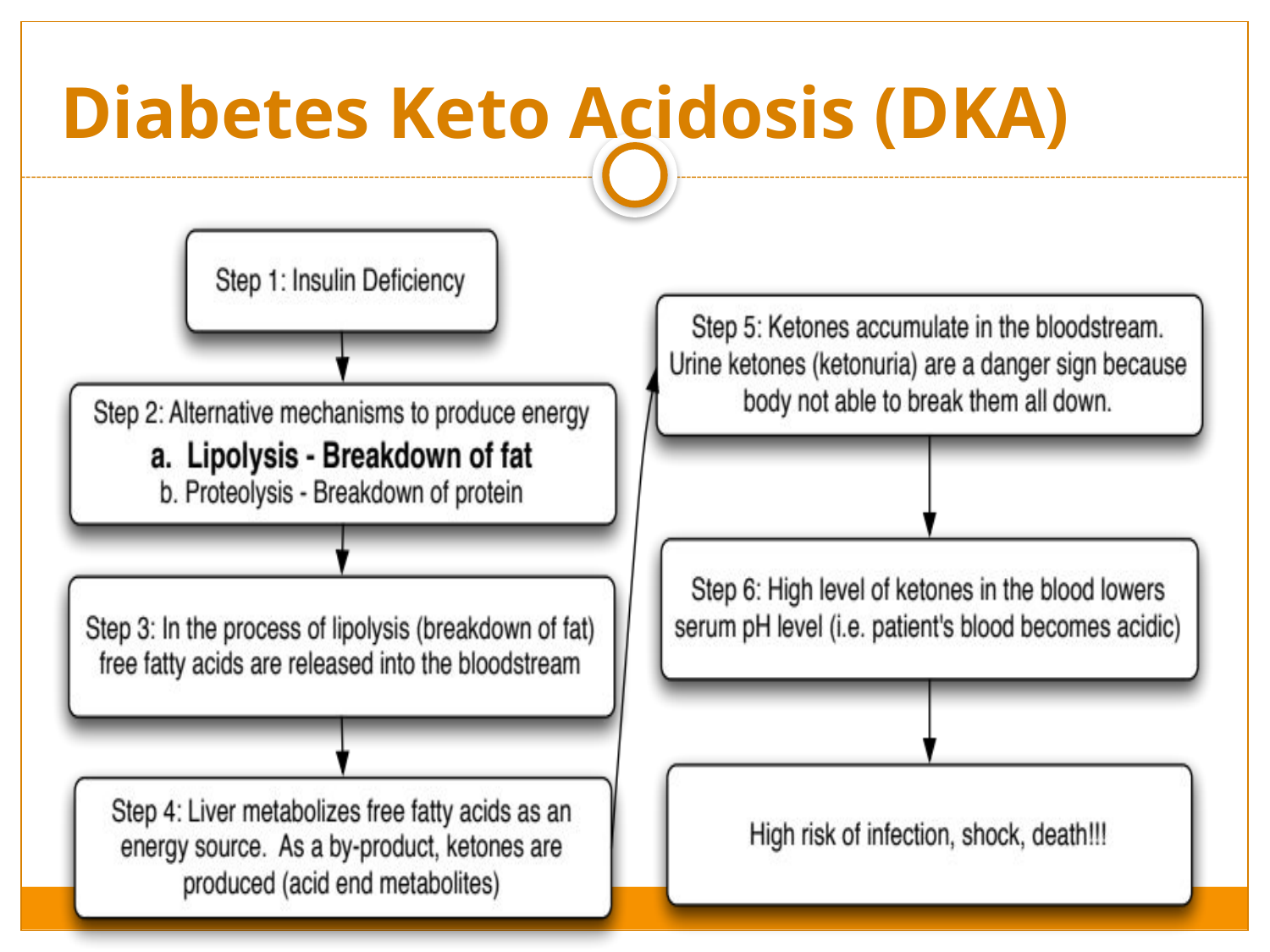

# Diabetes Keto Acidosis (DKA)

## Slide 8
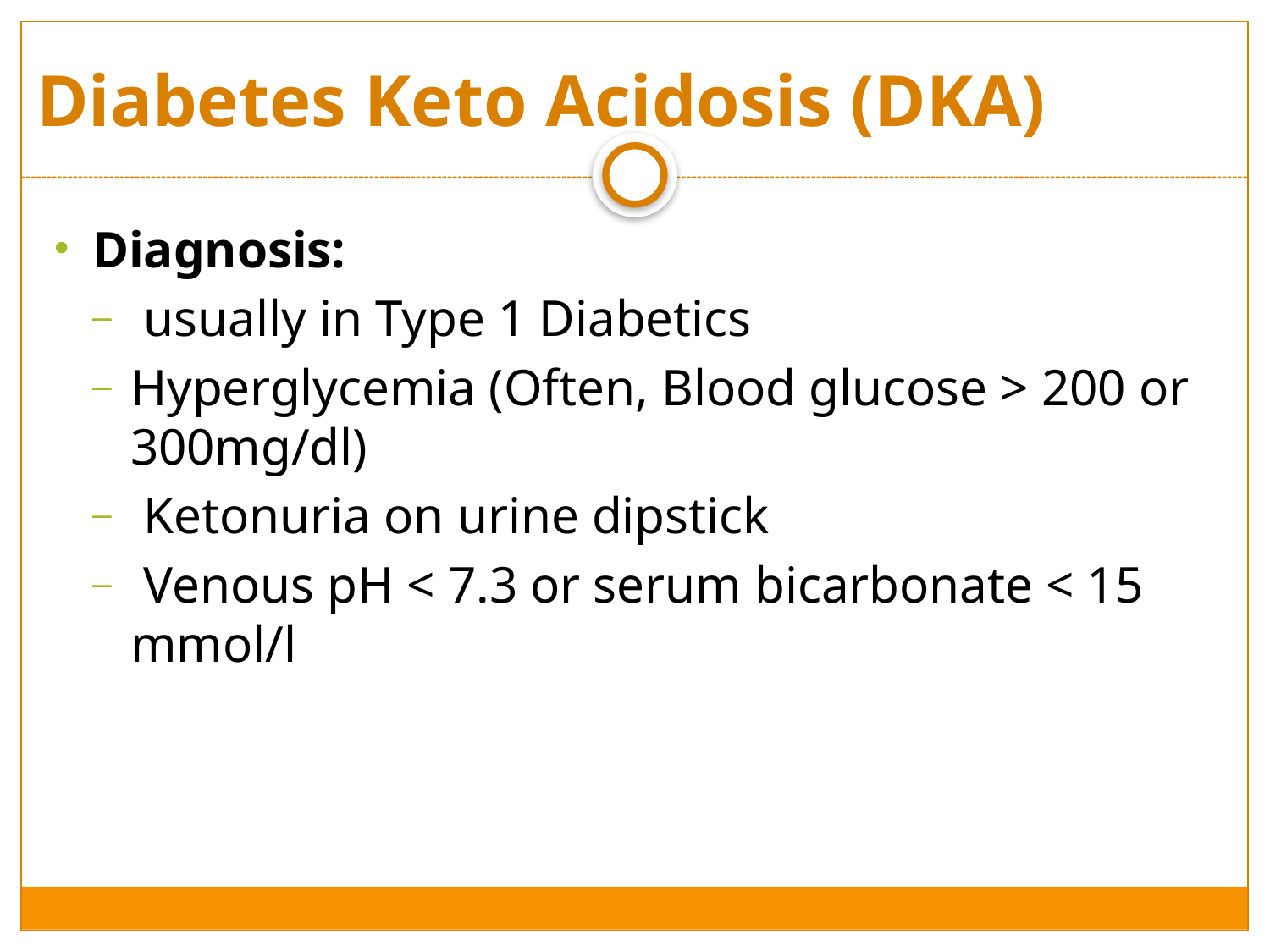

# Diabetes Keto Acidosis (DKA)
Diagnosis:
 usually in Type 1 Diabetics
Hyperglycemia (Often, Blood glucose > 200 or 300mg/dl)
 Ketonuria on urine dipstick
 Venous pH < 7.3 or serum bicarbonate < 15 mmol/l

## Slide 9
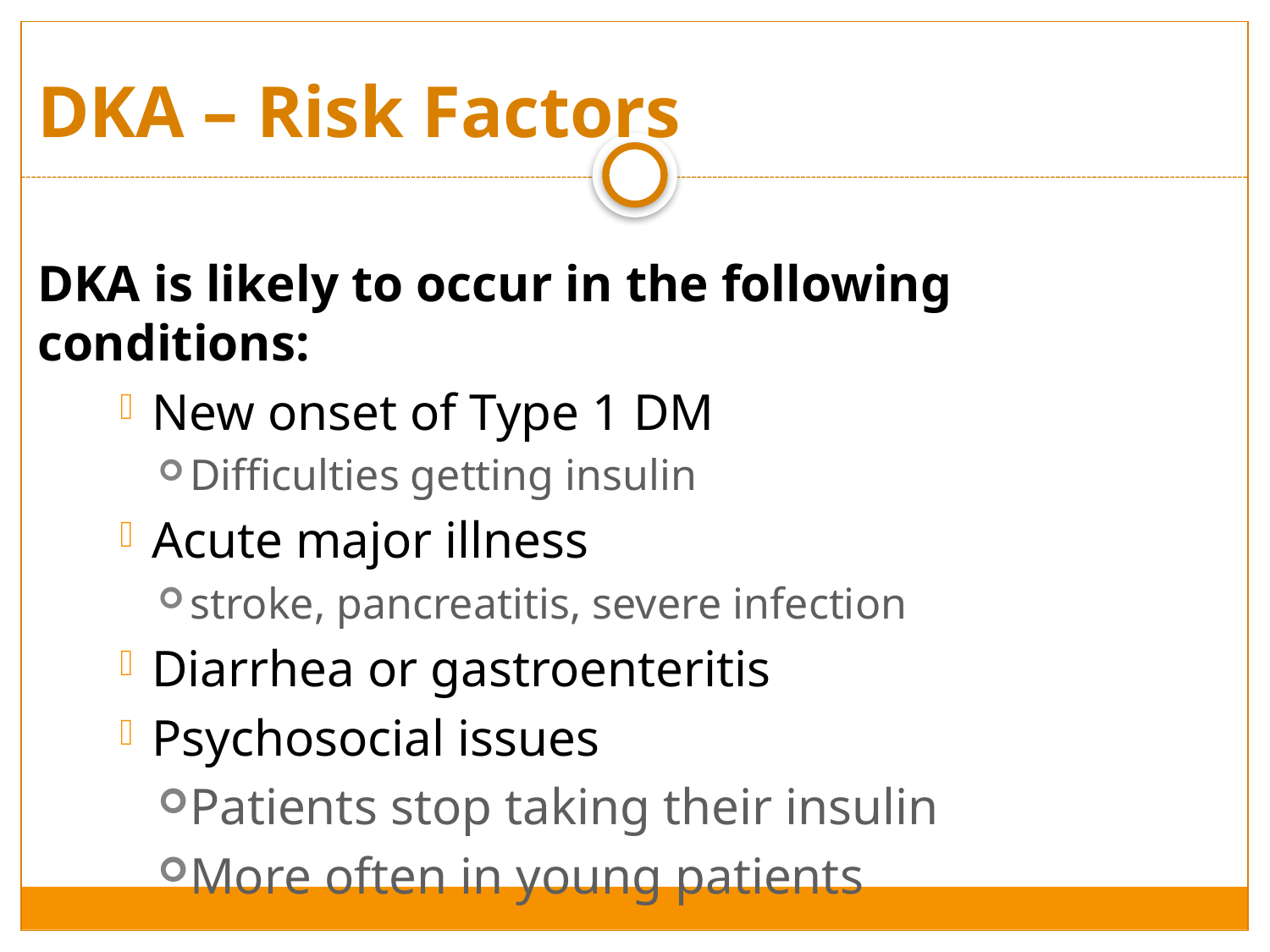

# DKA – Risk Factors
DKA is likely to occur in the following conditions:
New onset of Type 1 DM
Difficulties getting insulin
Acute major illness
stroke, pancreatitis, severe infection
Diarrhea or gastroenteritis
Psychosocial issues
Patients stop taking their insulin
More often in young patients

## Slide 10
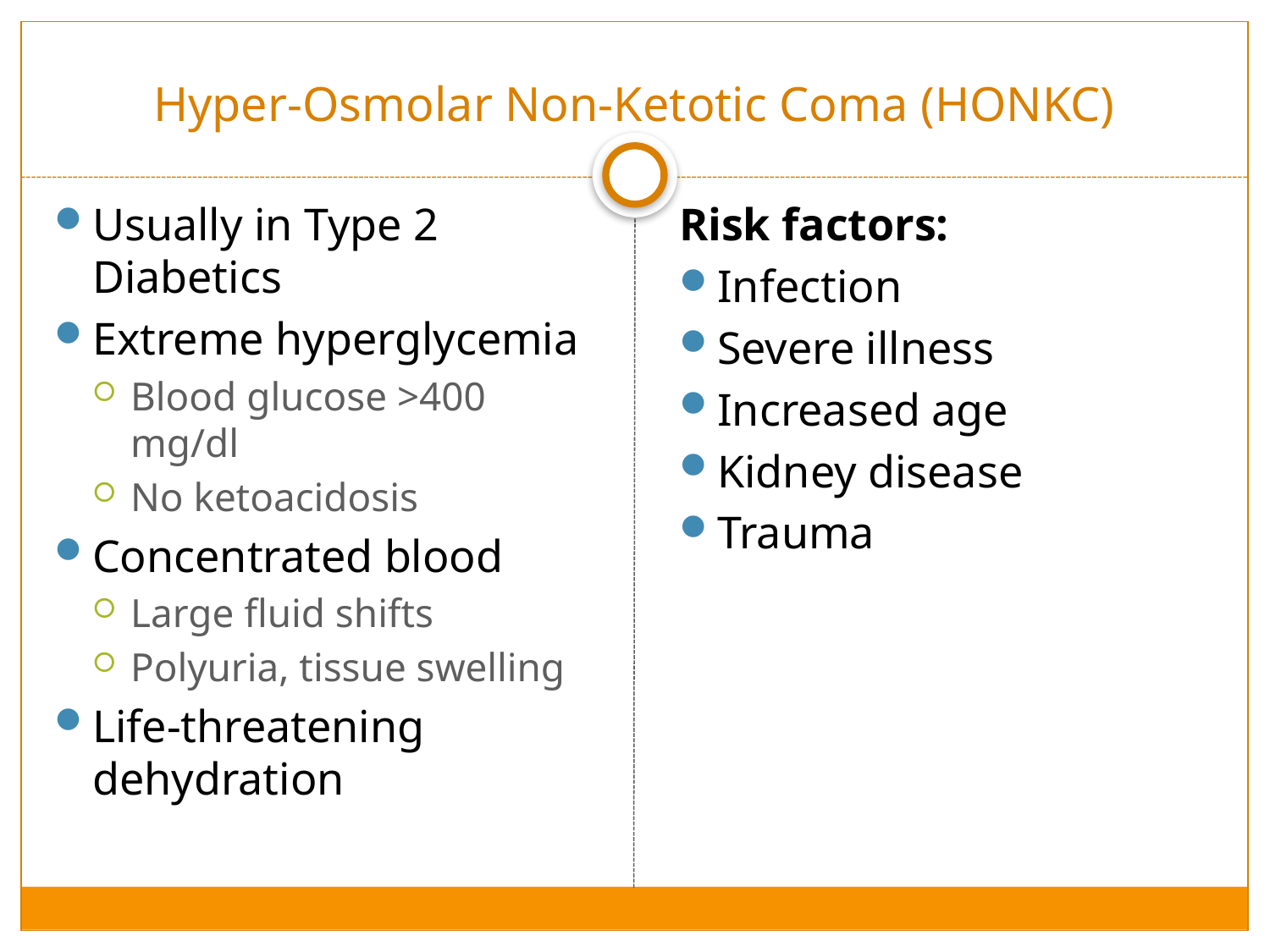

# Hyper-Osmolar Non-Ketotic Coma (HONKC)
Usually in Type 2 Diabetics
Extreme hyperglycemia
Blood glucose >400 mg/dl
No ketoacidosis
Concentrated blood
Large fluid shifts
Polyuria, tissue swelling
Life-threatening dehydration
Risk factors:
Infection
Severe illness
Increased age
Kidney disease
Trauma

## Slide 11
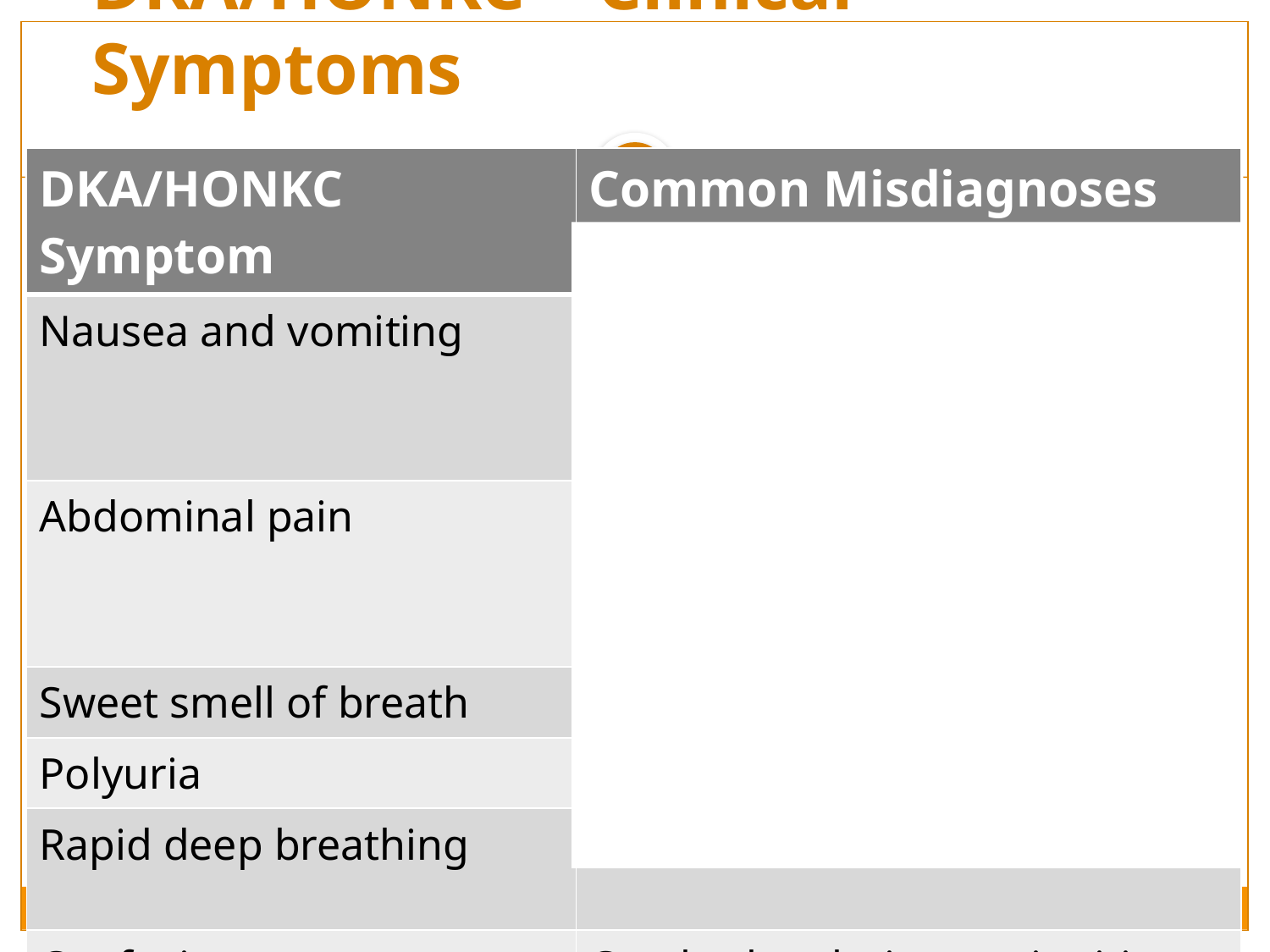

# DKA/HONKC – Clinical Symptoms
| DKA/HONKC Symptom | Common Misdiagnoses |
| --- | --- |
| Nausea and vomiting | Malaria, typhoid, gastroenteritis, pancreatitis, appendicitis |
| Abdominal pain | Malaria, typhoid, gastroenteritis, pancreatitis, appendicitis |
| Sweet smell of breath | None |
| Polyuria | Urinary tract infection |
| Rapid deep breathing | Pneumonia or Asthma |
| Confusion or even coma | Cerebral malaria, meningitis, HIV/AIDs, Tuberculosis meningitis |

## Slide 12
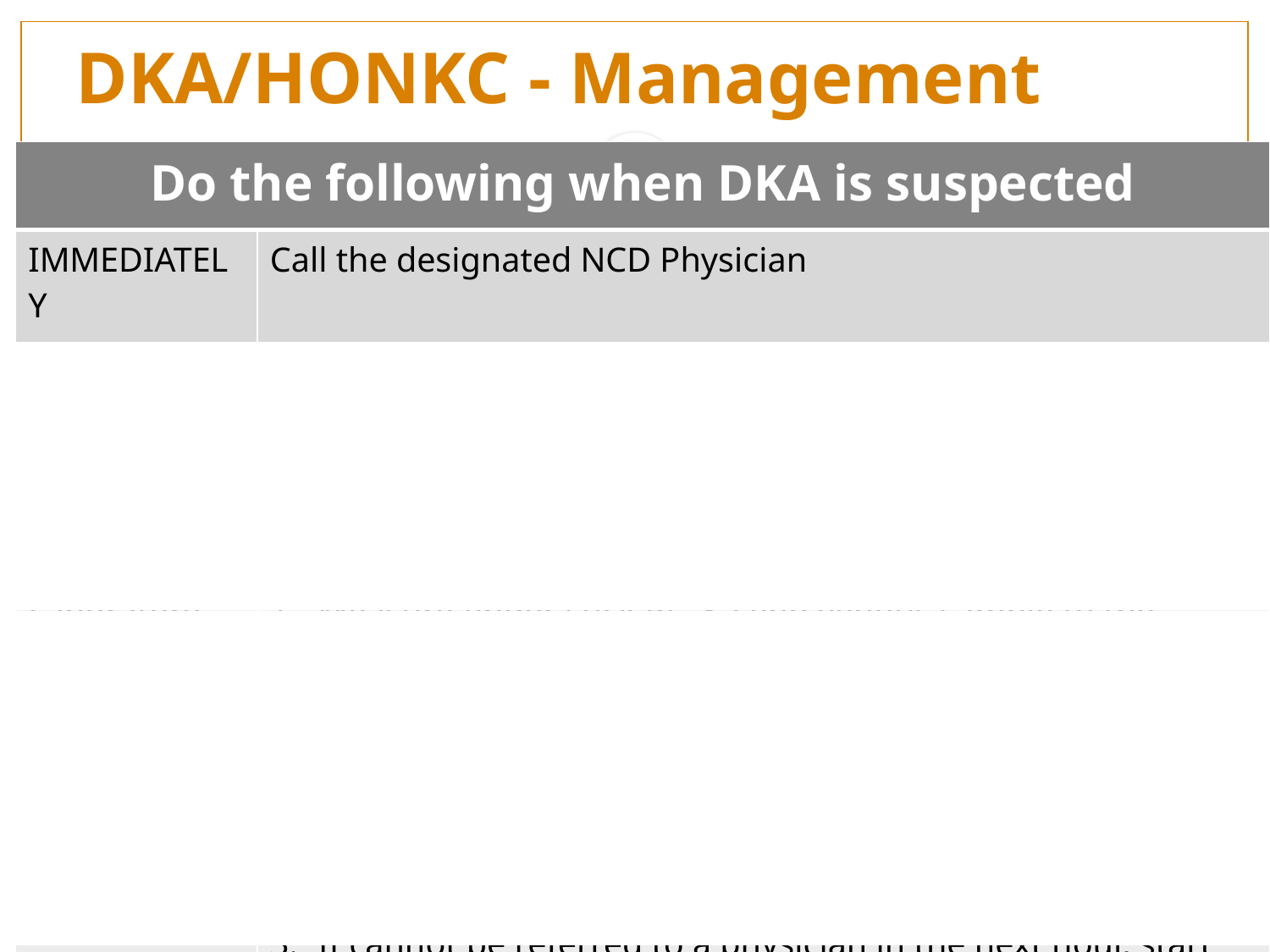

# DKA/HONKC - Management
| Do the following when DKA is suspected | |
| --- | --- |
| IMMEDIATELY | Call the designated NCD Physician |
| Physical Exam | Carefully record vital signs Assess for: Level of Consciousness, severity of dehydration, infection Weigh the patient |
| Laboratory | Measure finger stick BG & send venous sample to the laboratory Urine dipstick (look for ketones) |
| Treatment | Place IVF, correct shock with RL or NS 20ml/kg/h, if persists continue with 10mls/kg/h cautiously. If not in shock, start 0.9% NS or RL at 10cc/kg/h, to be repeated until reaches next referral level If cannot be referred to a physician in the next hour, start 0.1u/kg/h s/c short acting insulin (rapid), check BG hourly and repeat accordingly until he reaches next referral level. K+ will be started at the referral level. |

## Slide 13
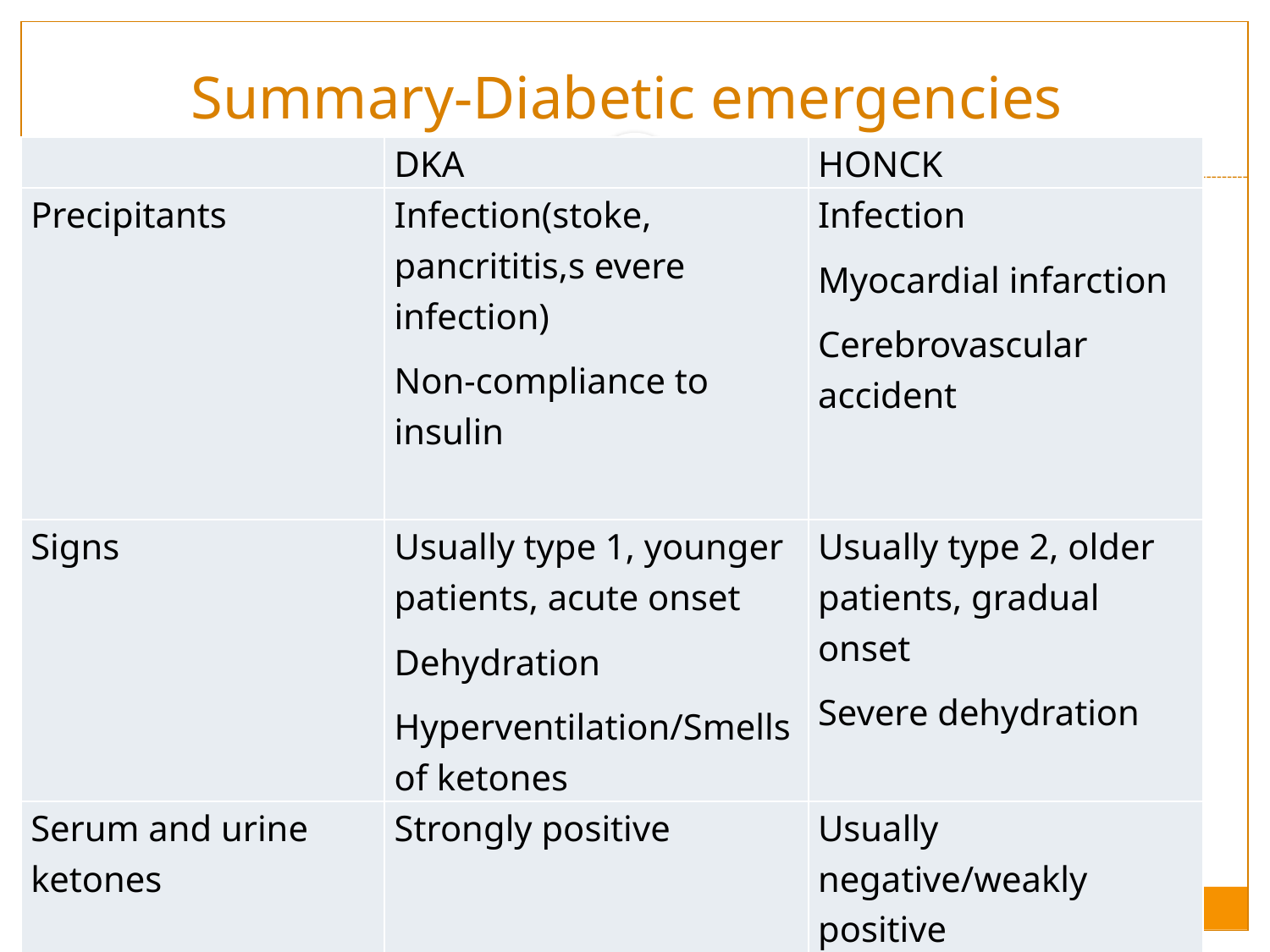

# Summary-Diabetic emergencies
| | DKA | HONCK |
| --- | --- | --- |
| Precipitants | Infection(stoke, pancrititis,s evere infection) Non-compliance to insulin | Infection Myocardial infarction Cerebrovascular accident |
| Signs | Usually type 1, younger patients, acute onset Dehydration Hyperventilation/Smells of ketones | Usually type 2, older patients, gradual onset Severe dehydration |
| Serum and urine ketones | Strongly positive | Usually negative/weakly positive |
| Blood glucose | Raised(over 200-300md/dl) | Markedly raised(over 400 mg/dl) |
| Serum pH | Decreased | Usually normal |
| Serum bicarbonate | Low | Usually normal |

## Slide 14
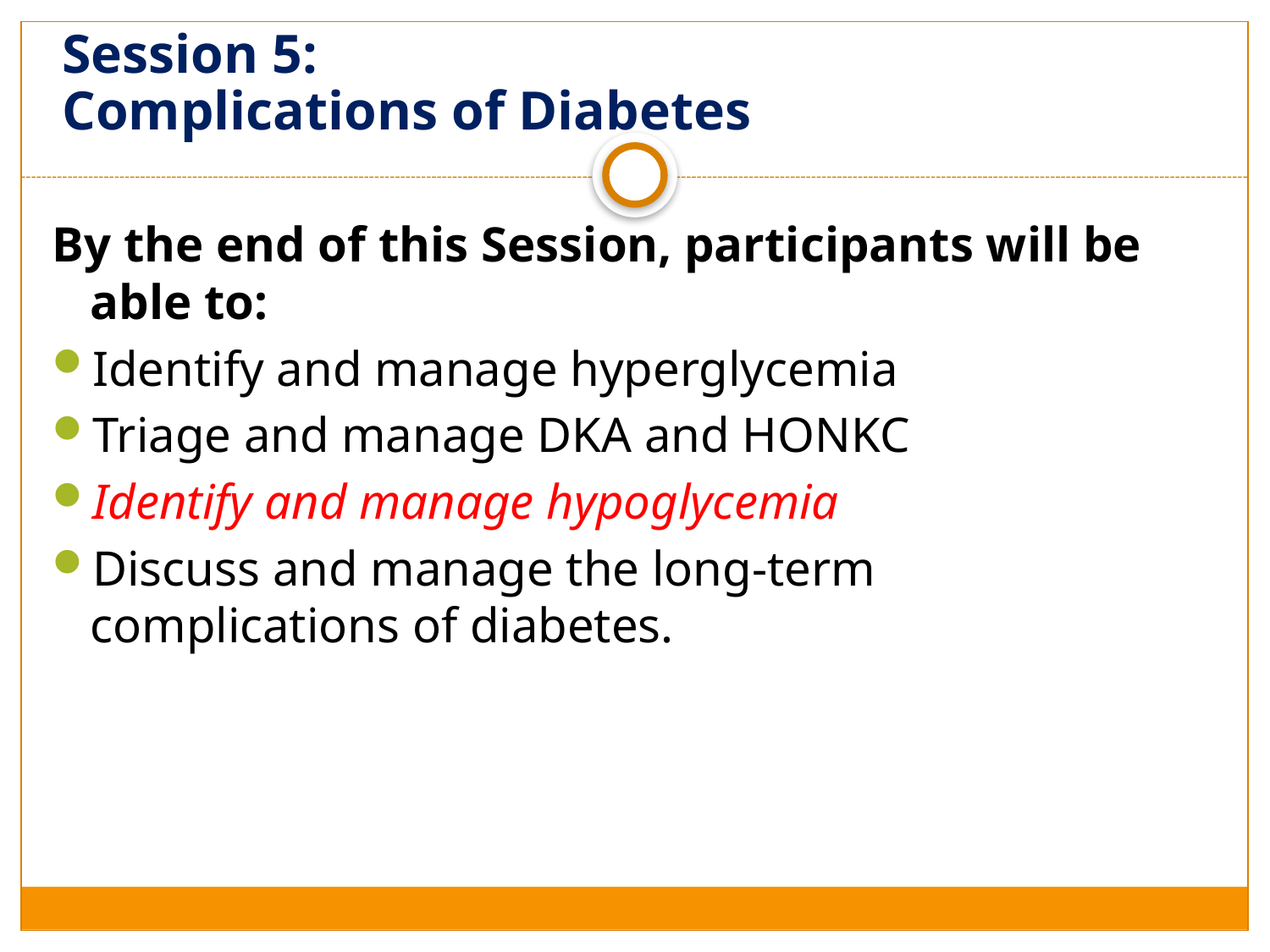

# Session 5: Complications of Diabetes
By the end of this Session, participants will be able to:
Identify and manage hyperglycemia
Triage and manage DKA and HONKC
Identify and manage hypoglycemia
Discuss and manage the long-term complications of diabetes.

## Slide 15
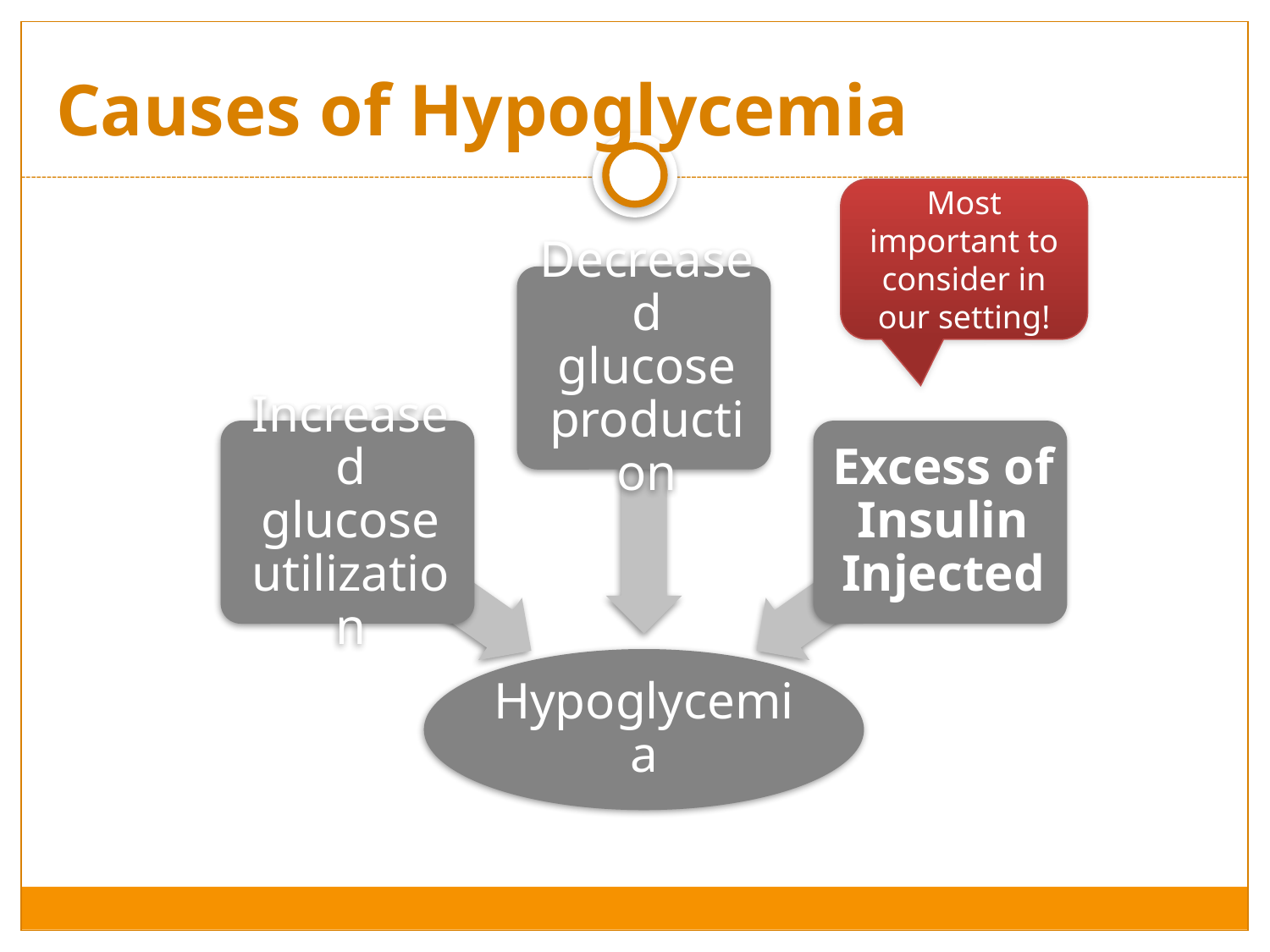

# Causes of Hypoglycemia
Most important to consider in our setting!

## Slide 16
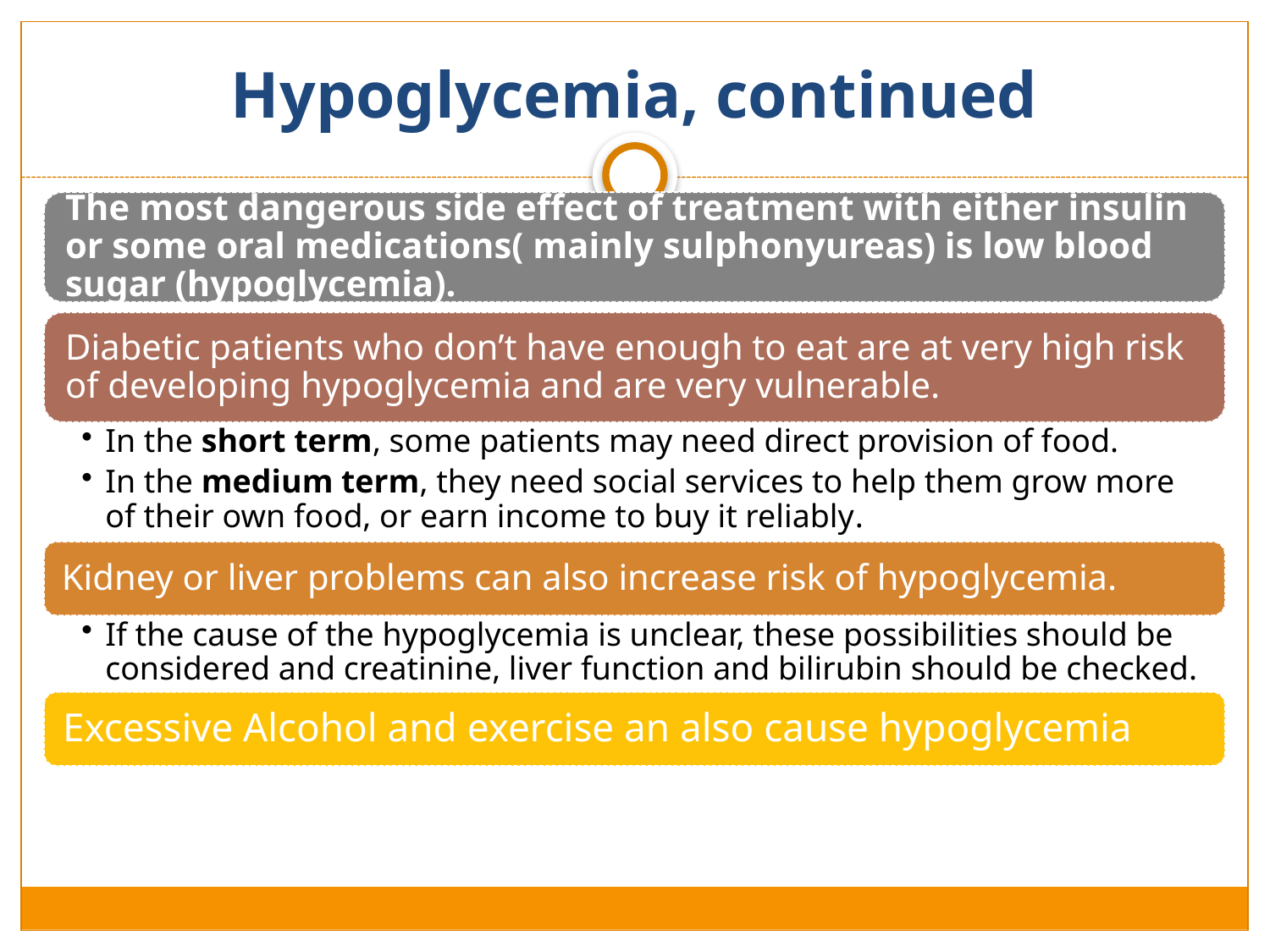

# Hypoglycemia, continued

## Slide 17
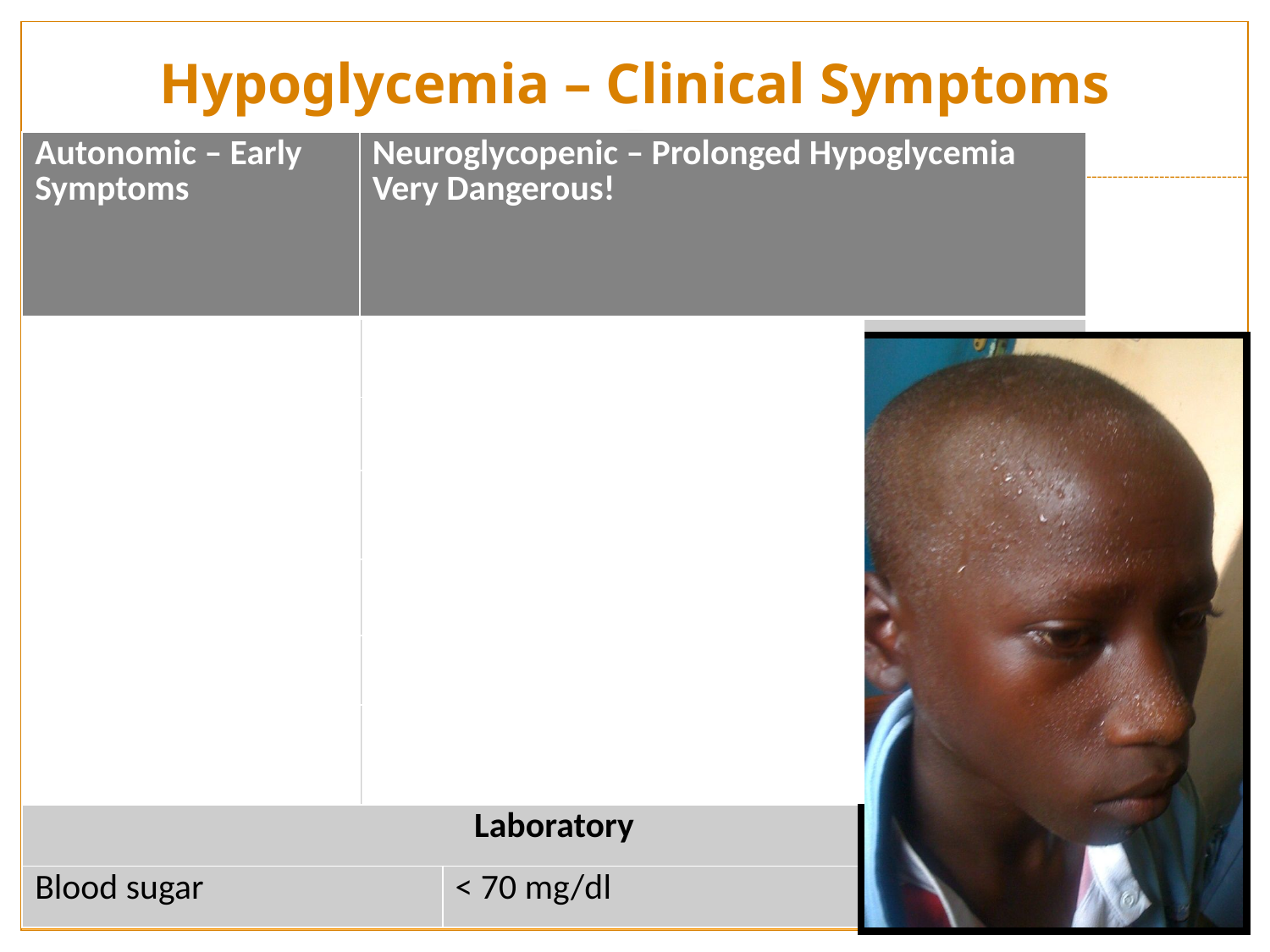

# Hypoglycemia – Clinical Symptoms
| Autonomic – Early Symptoms | Neuroglycopenic – Prolonged Hypoglycemia Very Dangerous! | |
| --- | --- | --- |
| Sweating (cold) | Lethargy | |
| Weakness | Irritability | |
| Tachycardia | Confusion | |
| Tremor | Sudden personality change | |
| Nervousness | Hypothermia | |
| Sudden and extreme hunger | Seizure and coma | |
| Laboratory | | |
| Blood sugar | | < 70 mg/dl |

## Slide 18
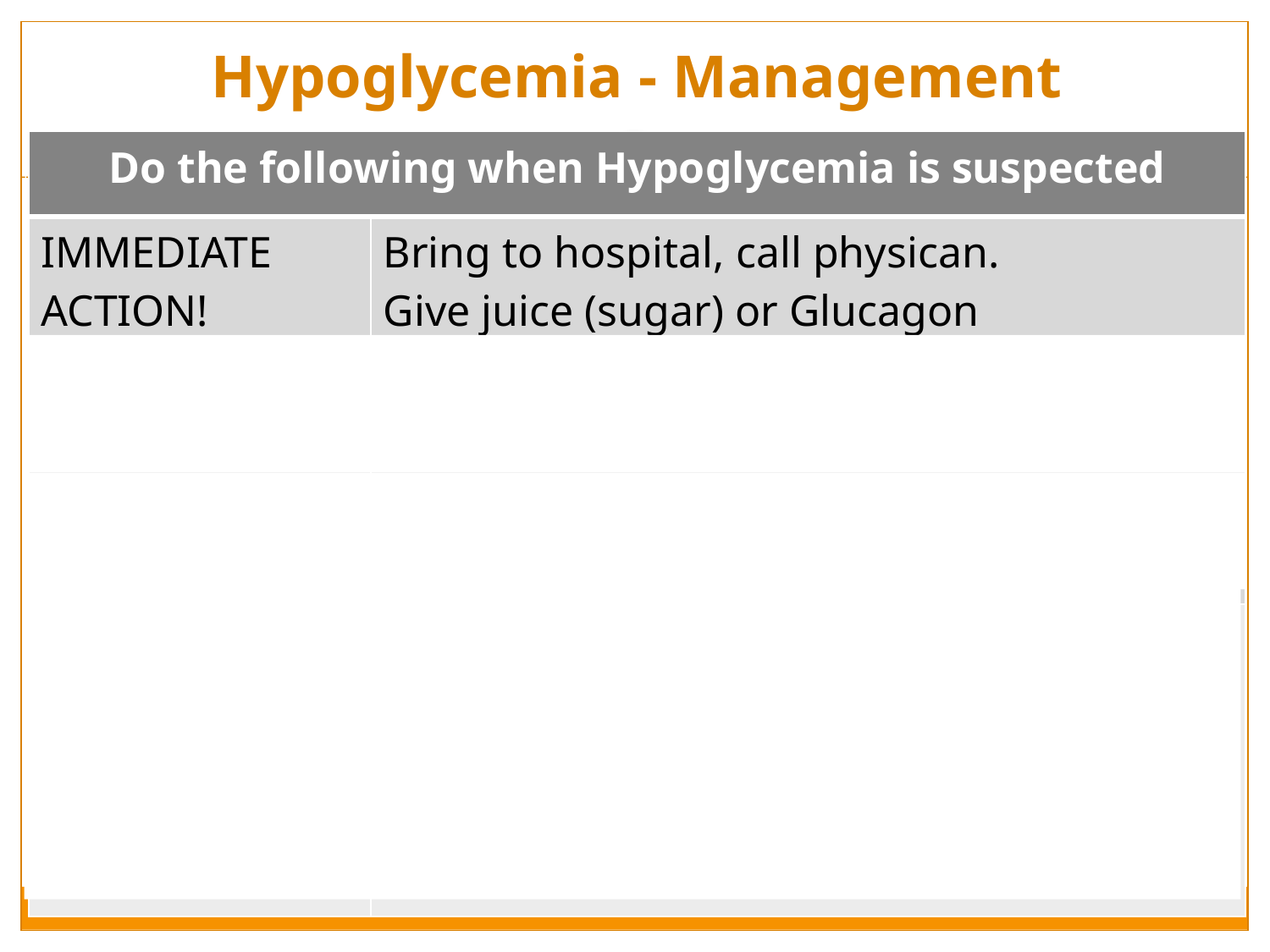

# Hypoglycemia - Management
| Do the following when Hypoglycemia is suspected | |
| --- | --- |
| IMMEDIATE ACTION! | Bring to hospital, call physican. Give juice (sugar) or Glucagon |
| Physical Exam | Carefully record vital signs Assess for Level of Consciousness (LOC) |
| Laboratory | Finger stick blood glucose, I-STAT, full blood count (FBC), liver function tests |
| Treatment | Normal LOC: rapidly absorbable glucose (sugar or fruit juice) Impaired LOC: 2.5mL/kg of 10% dextrose injected over 5 minutes |

## Slide 19
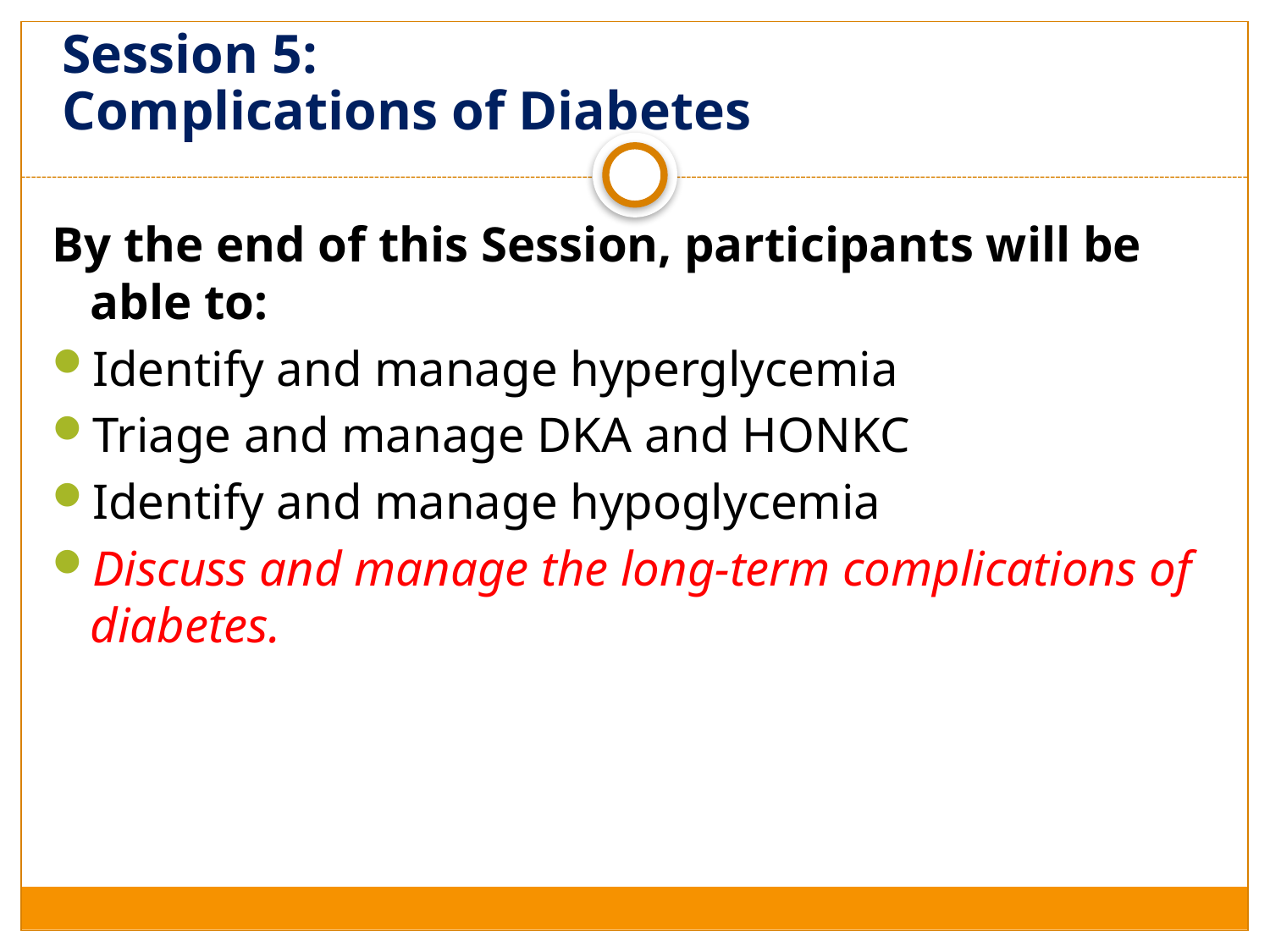

# Session 5: Complications of Diabetes
By the end of this Session, participants will be able to:
Identify and manage hyperglycemia
Triage and manage DKA and HONKC
Identify and manage hypoglycemia
Discuss and manage the long-term complications of diabetes.

## Slide 20
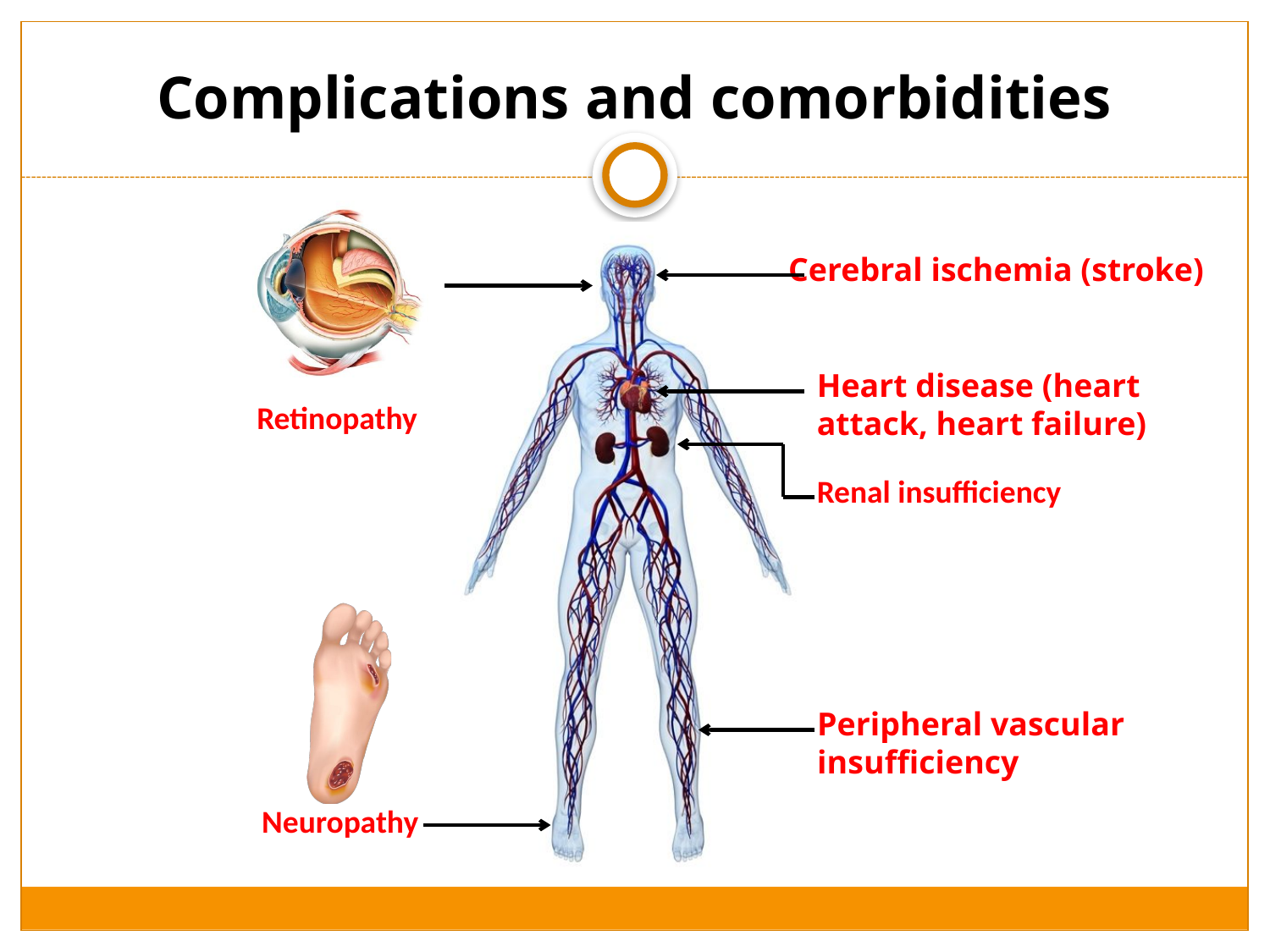

# Complications and comorbidities
Cerebral ischemia (stroke)
Heart disease (heart attack, heart failure)
Retinopathy
Renal insufficiency
Peripheral vascular insufficiency
Neuropathy

## Slide 21
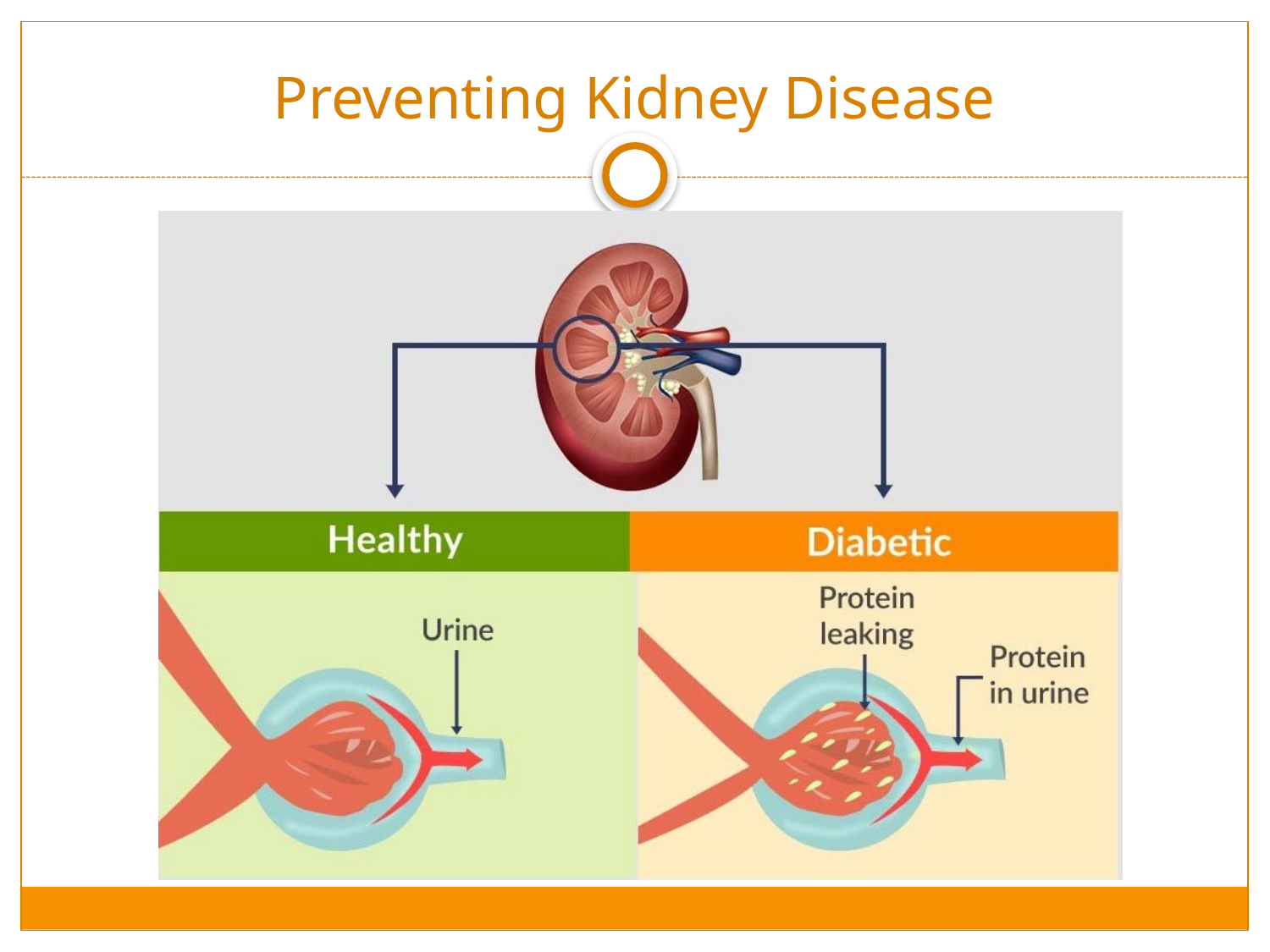

# Preventing Kidney Disease

## Slide 22
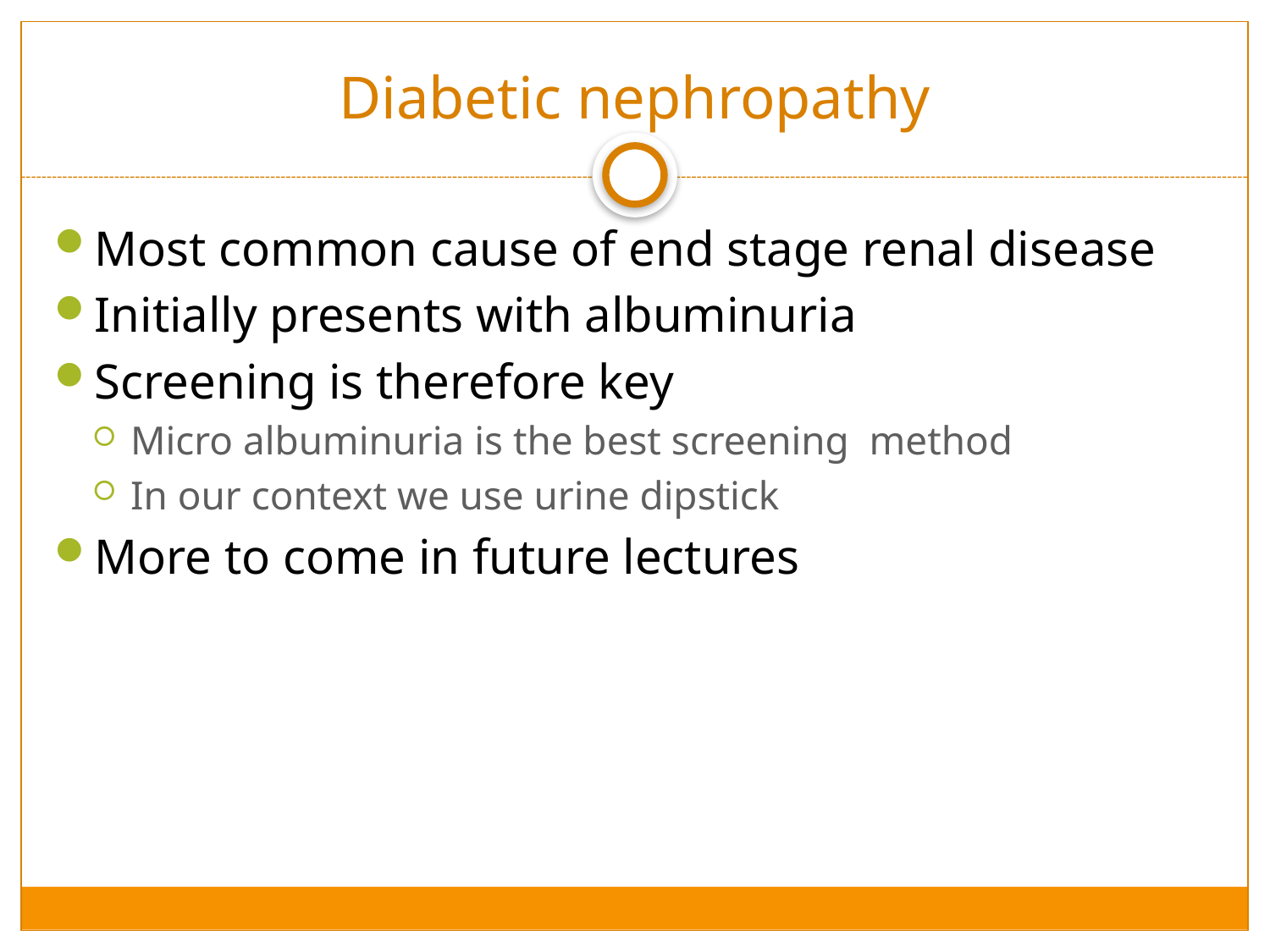

# Diabetic nephropathy
Most common cause of end stage renal disease
Initially presents with albuminuria
Screening is therefore key
Micro albuminuria is the best screening method
In our context we use urine dipstick
More to come in future lectures

## Slide 23
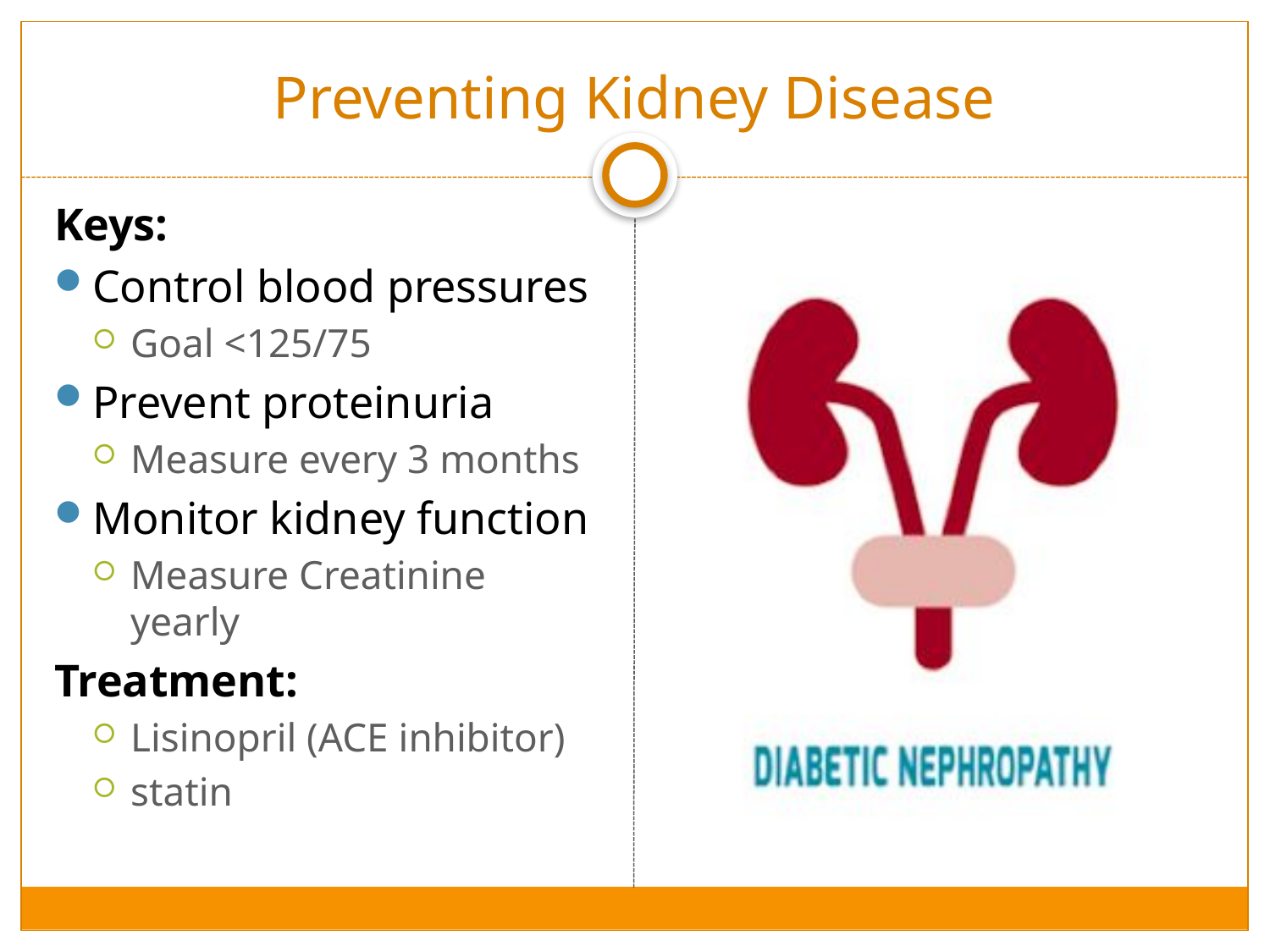

# Preventing Kidney Disease
Keys:
Control blood pressures
Goal <125/75
Prevent proteinuria
Measure every 3 months
Monitor kidney function
Measure Creatinine yearly
Treatment:
Lisinopril (ACE inhibitor)
statin

## Slide 24
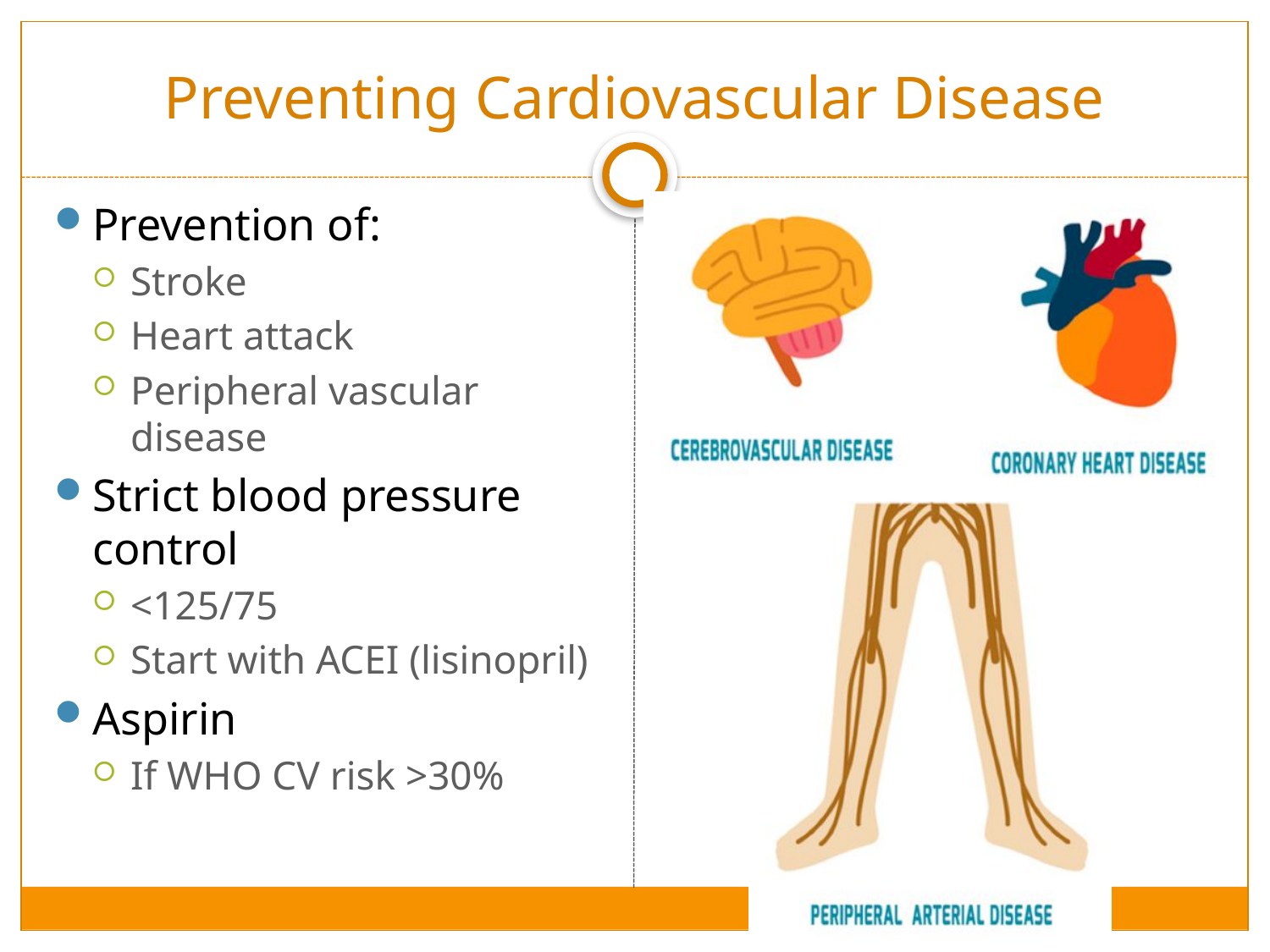

# Preventing Cardiovascular Disease
Prevention of:
Stroke
Heart attack
Peripheral vascular disease
Strict blood pressure control
<125/75
Start with ACEI (lisinopril)
Aspirin
If WHO CV risk >30%

## Slide 25
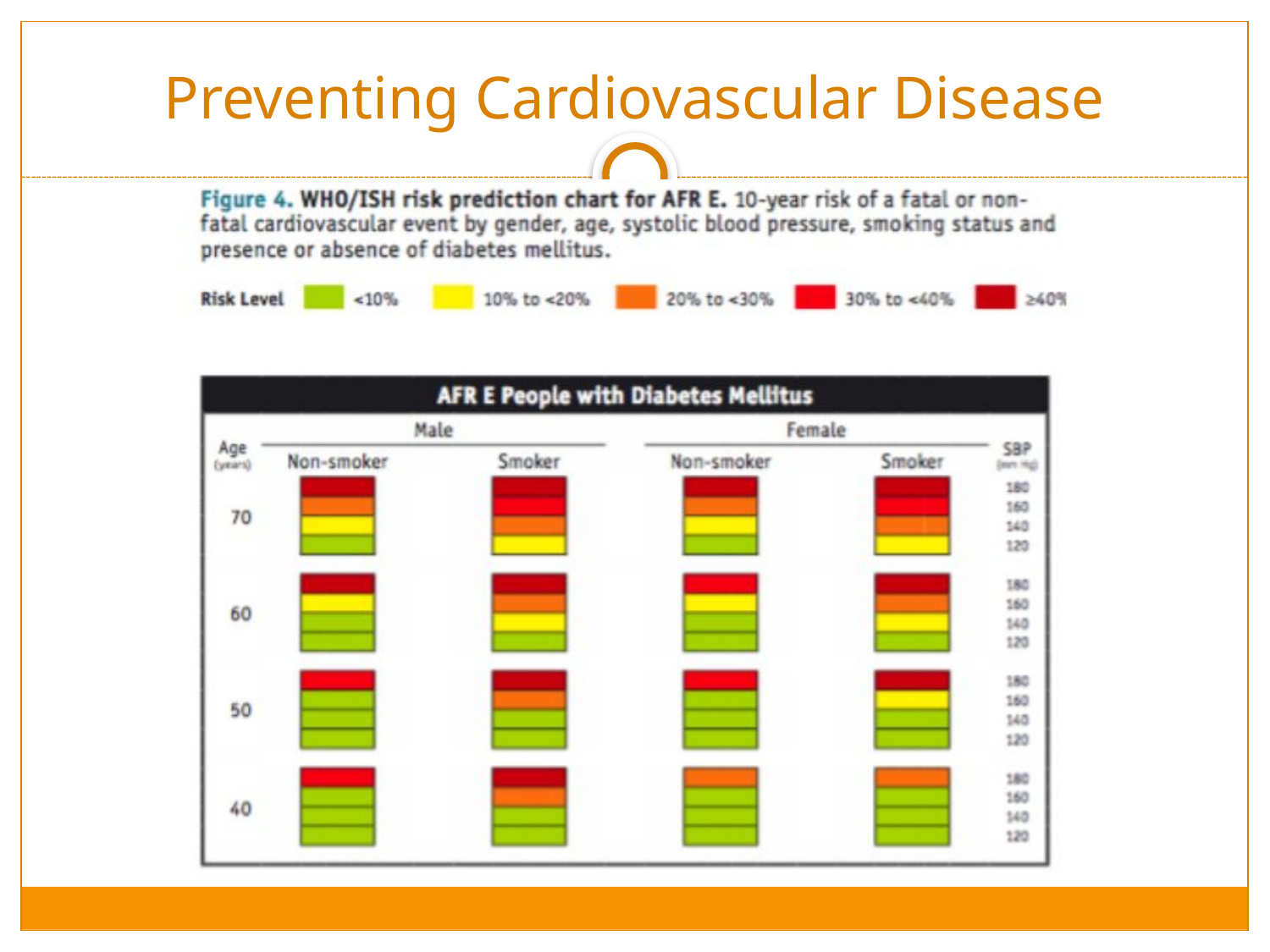

# Preventing Cardiovascular Disease

## Slide 26
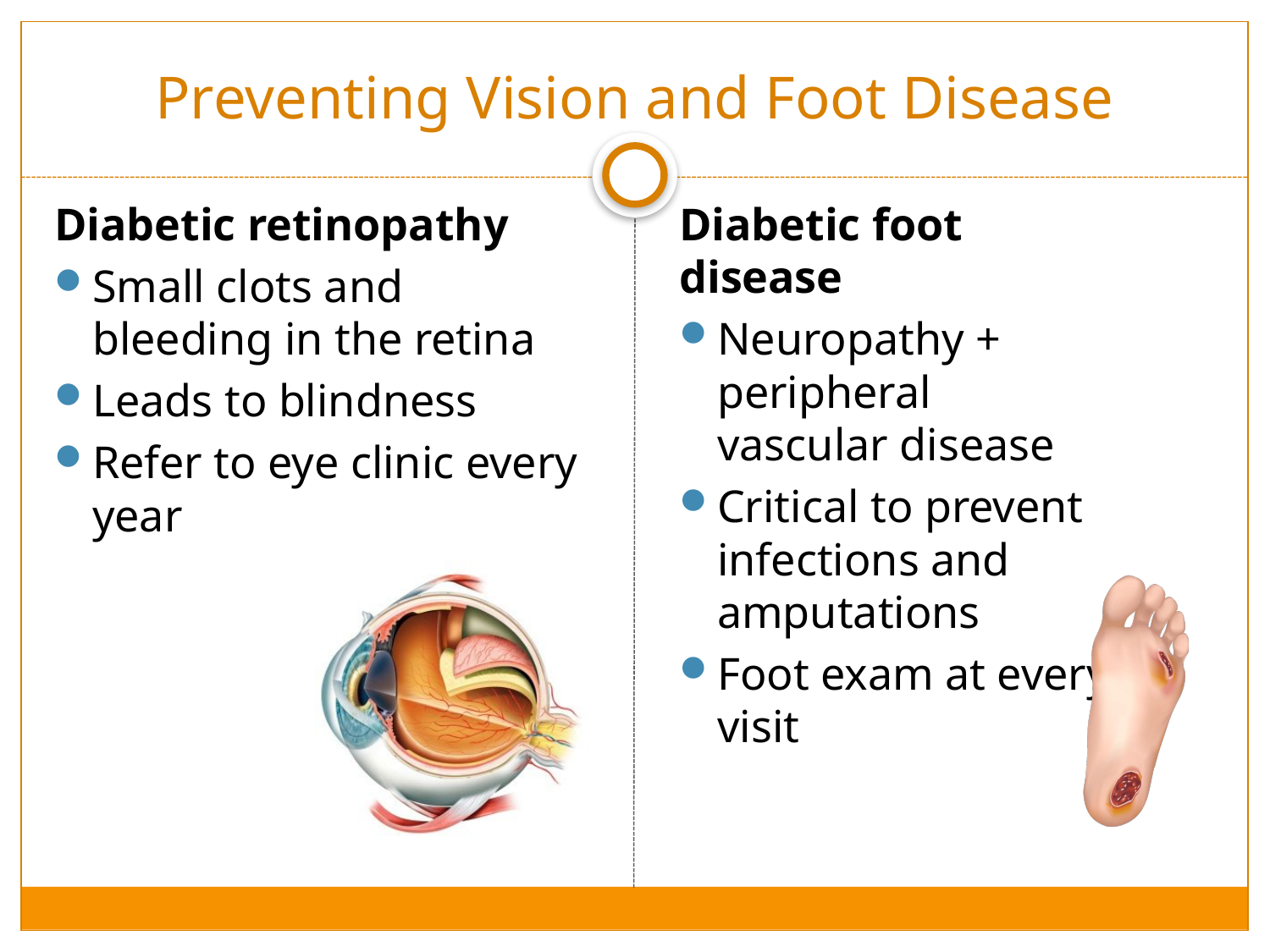

# Preventing Vision and Foot Disease
Diabetic retinopathy
Small clots and bleeding in the retina
Leads to blindness
Refer to eye clinic every year
Diabetic foot disease
Neuropathy + peripheral vascular disease
Critical to prevent infections and amputations
Foot exam at every visit

## Slide 27
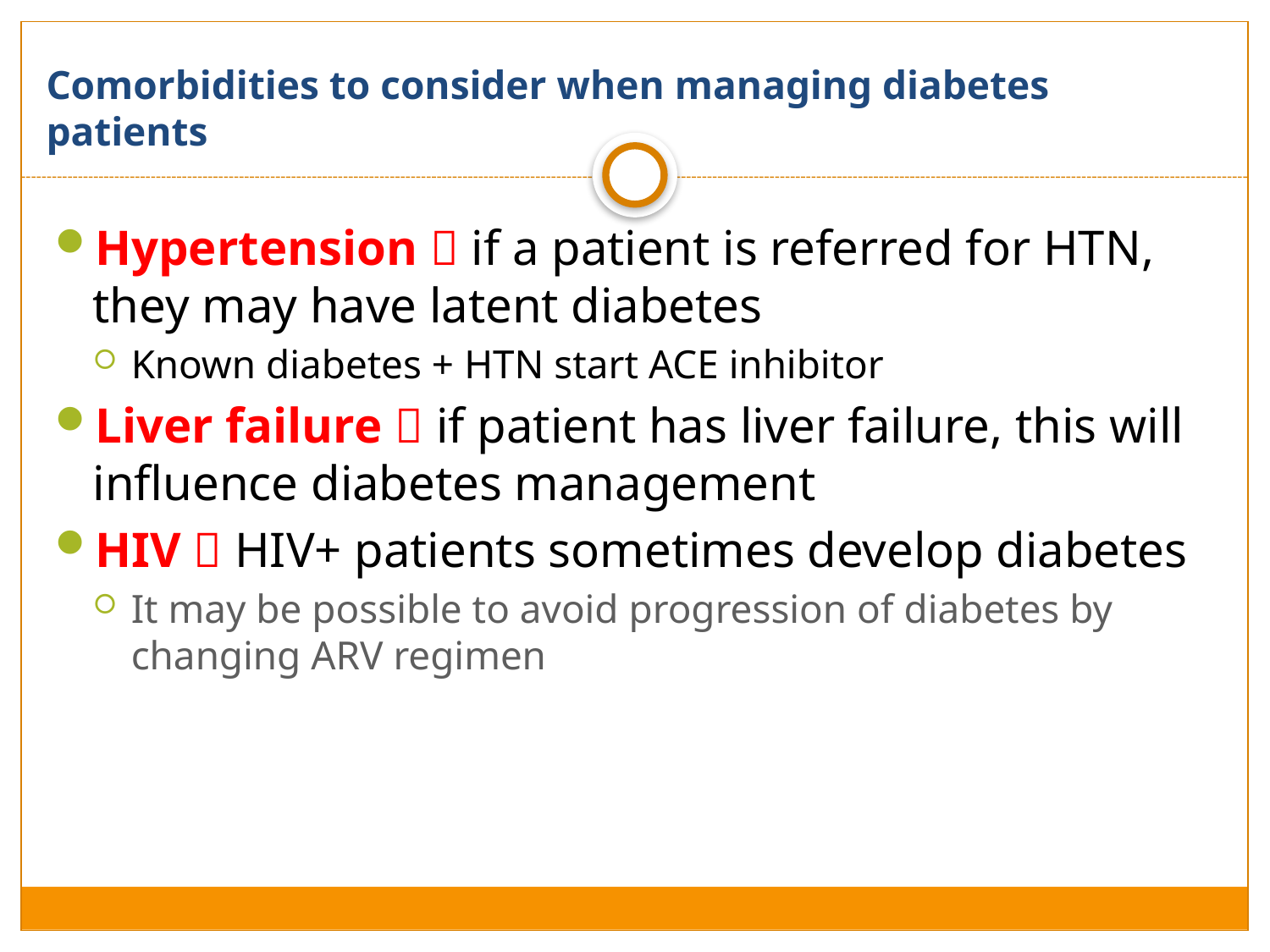

# Comorbidities to consider when managing diabetes patients
Hypertension  if a patient is referred for HTN, they may have latent diabetes
Known diabetes + HTN start ACE inhibitor
Liver failure  if patient has liver failure, this will influence diabetes management
HIV  HIV+ patients sometimes develop diabetes
It may be possible to avoid progression of diabetes by changing ARV regimen

## Slide 28
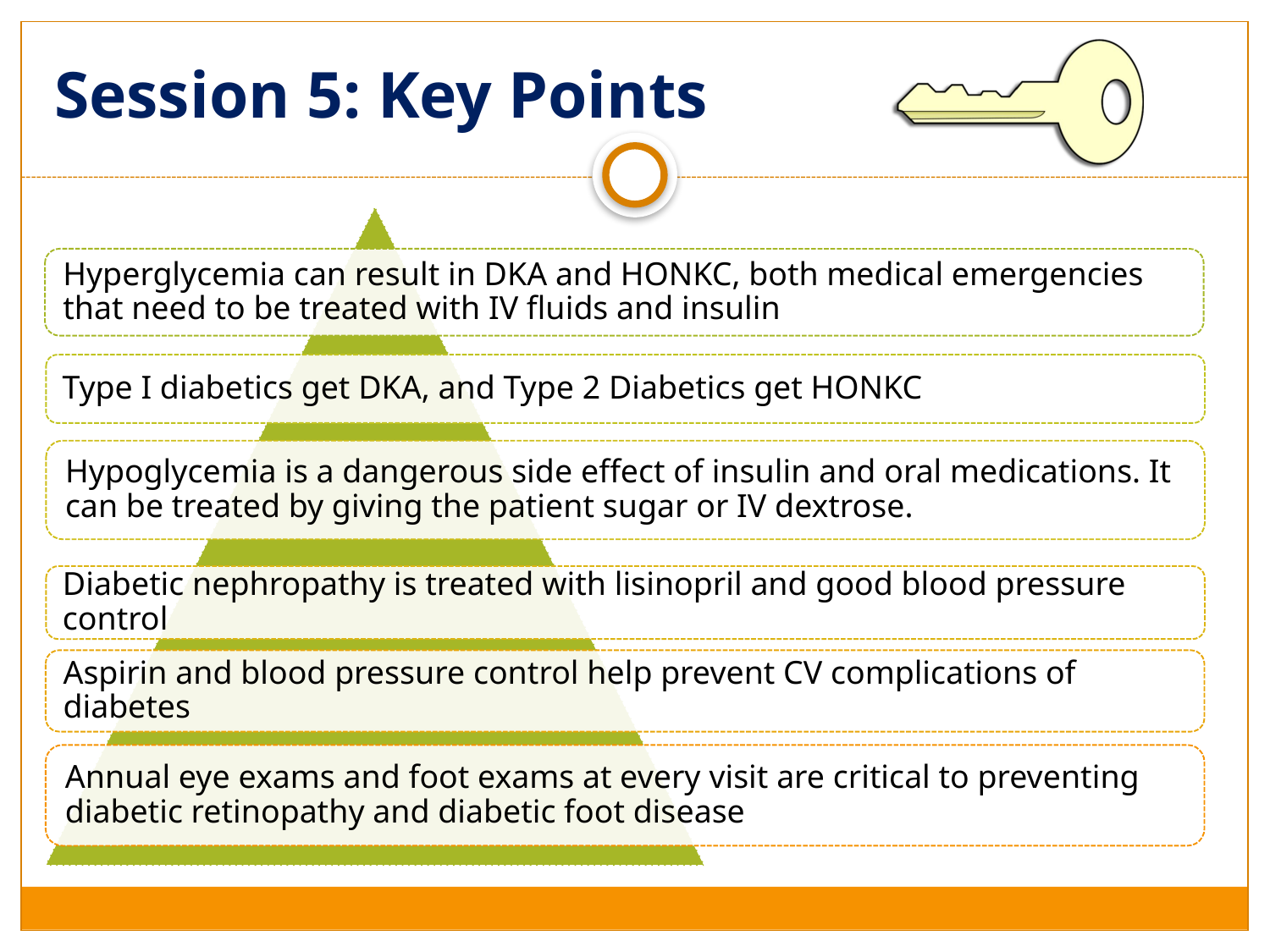

# Session 5: Key Points
