# Supplementary material for: Training Mid-Level Providers to Treat Severe Non-Communicable Diseases in Neno, Malawi through PEN-Plus Strategies
Source: Ann Glob Health. 2022 Aug 11;88(1):69. doi: 10.5334/aogh.3750 (PMC9389951; doi:10.5334/aogh.3750)
Supplement: Didactic Materials. — The supplementary materials contain a suggested didactic training schedule and the PowerPoint presentations used for PEN-Plus training in Neno, Malawi. These materials have been reviewed and accepted by the Malawi Ministry of Health for future PEN-Plus trainings in Malawi. [file agh-88-1-3750-s2.zip › Didactic_Materials/DM_Counseling.pptx]

## Slide 1
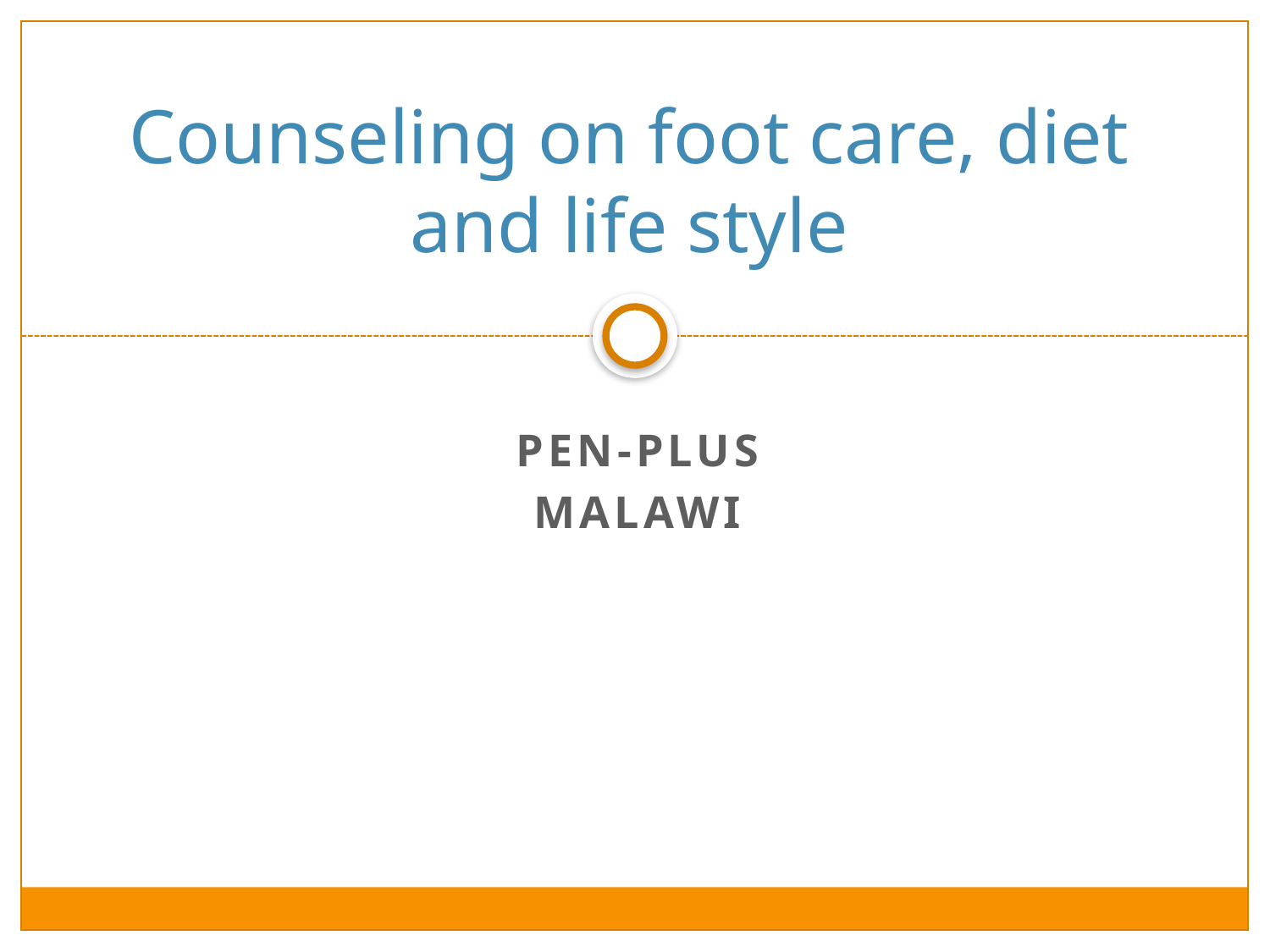

# Counseling on foot care, diet and life style
PEN-Plus
Malawi

## Slide 2
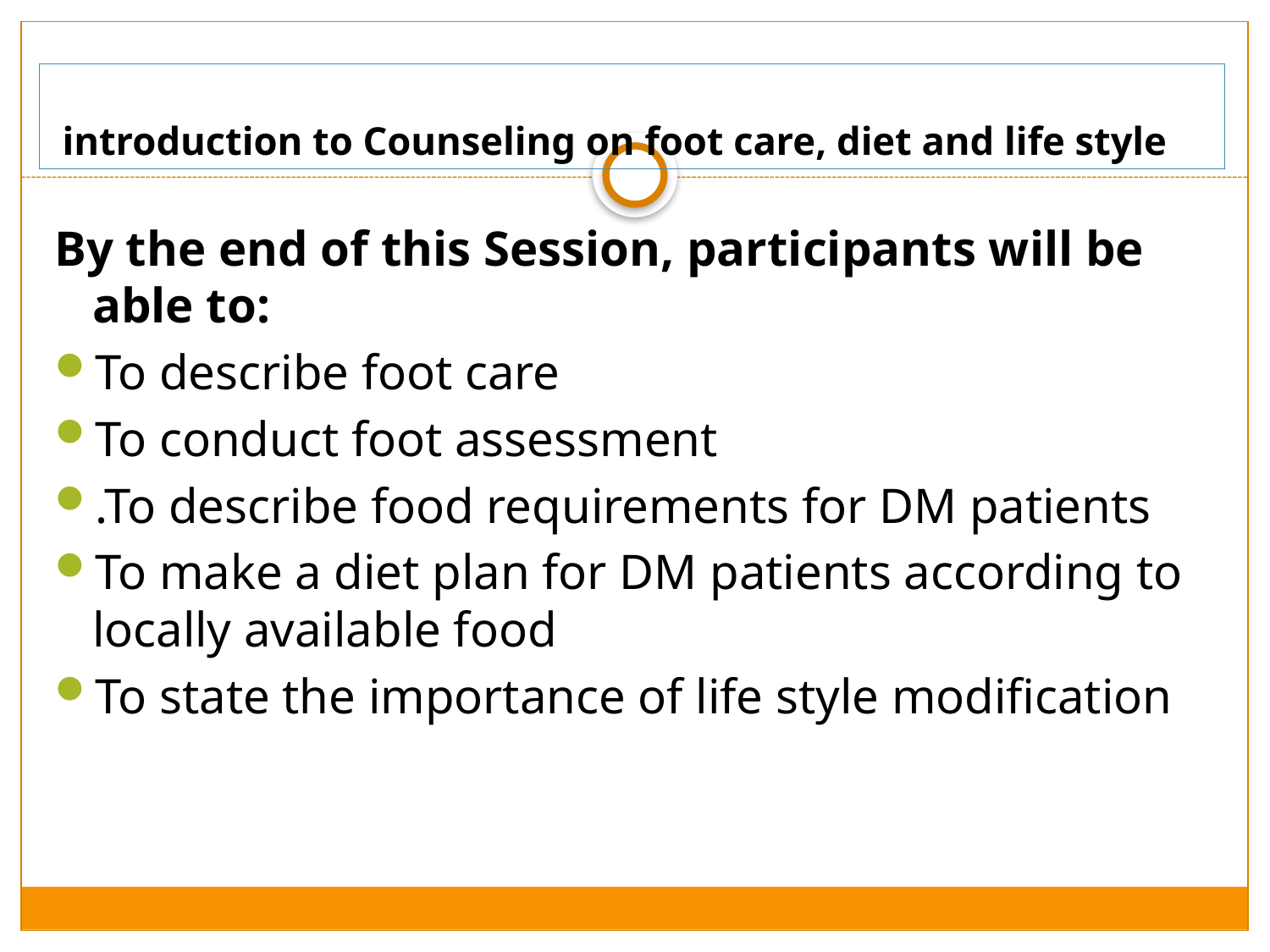

# introduction to Counseling on foot care, diet and life style
By the end of this Session, participants will be able to:
To describe foot care
To conduct foot assessment
.To describe food requirements for DM patients
To make a diet plan for DM patients according to locally available food
To state the importance of life style modification

## Slide 3
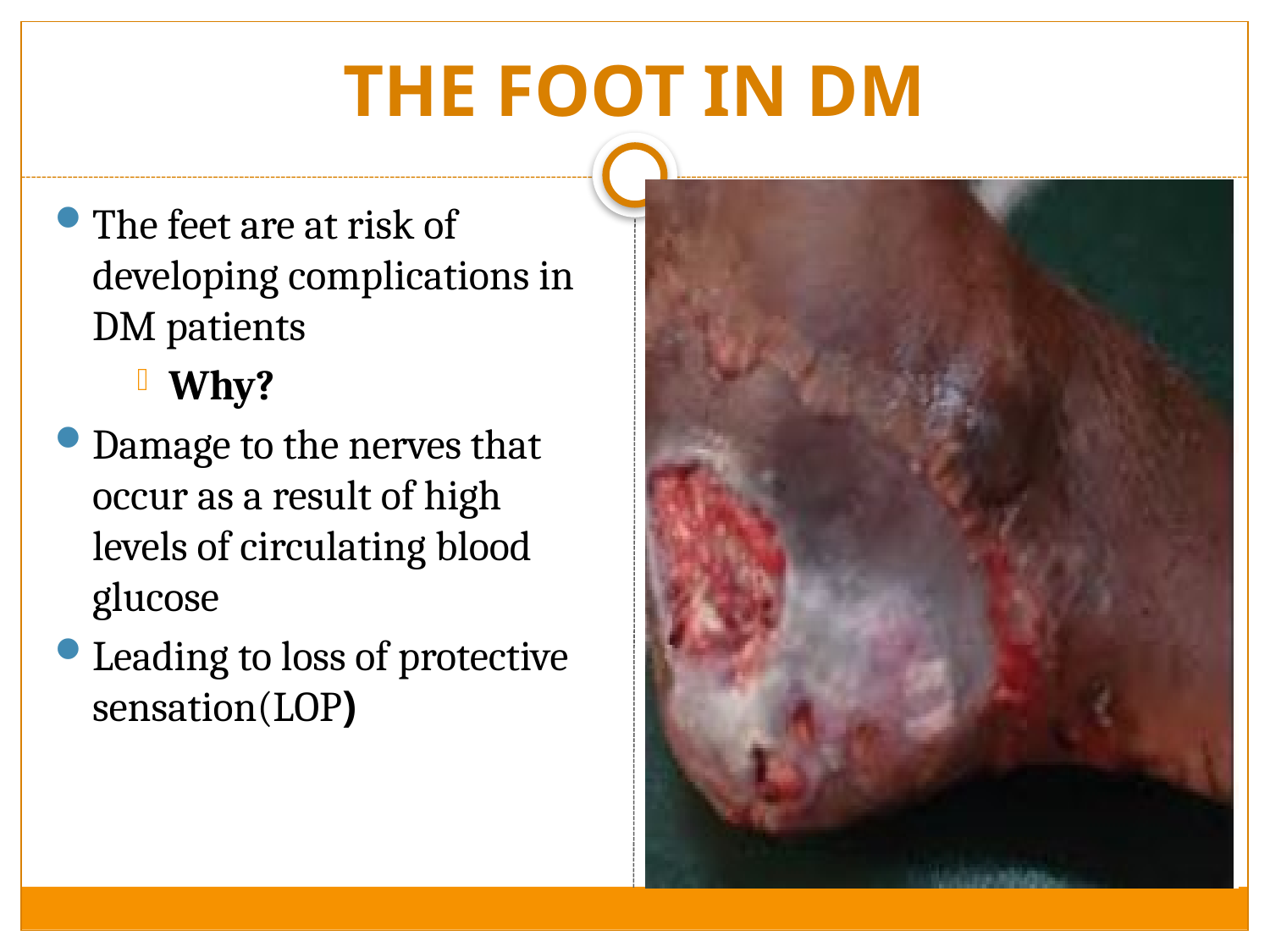

# THE FOOT IN DM
The feet are at risk of developing complications in DM patients
Why?
Damage to the nerves that occur as a result of high levels of circulating blood glucose
Leading to loss of protective sensation(LOP)

## Slide 4
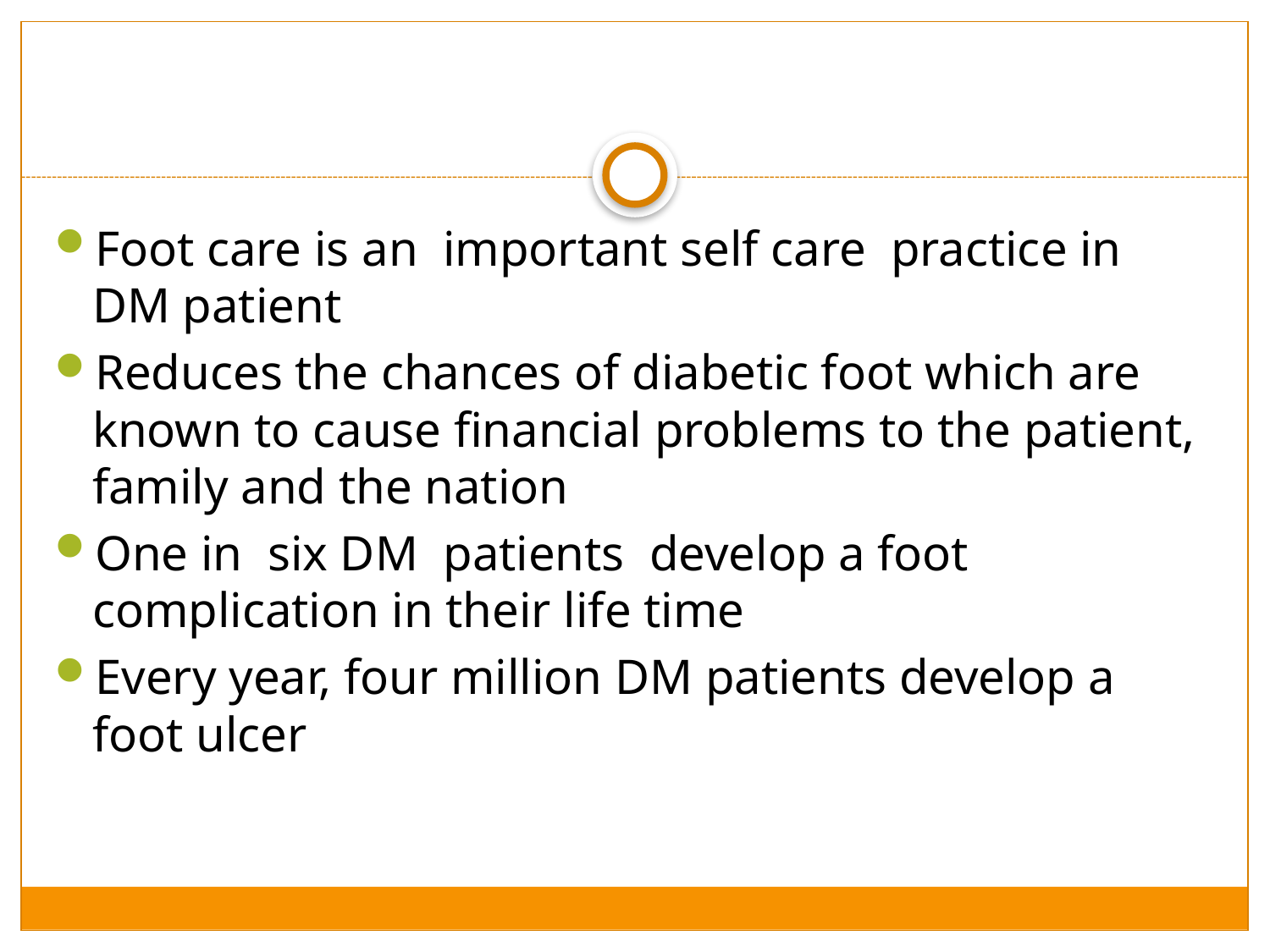

Foot care is an important self care practice in DM patient
Reduces the chances of diabetic foot which are known to cause financial problems to the patient, family and the nation
One in six DM patients develop a foot complication in their life time
Every year, four million DM patients develop a foot ulcer

## Slide 5
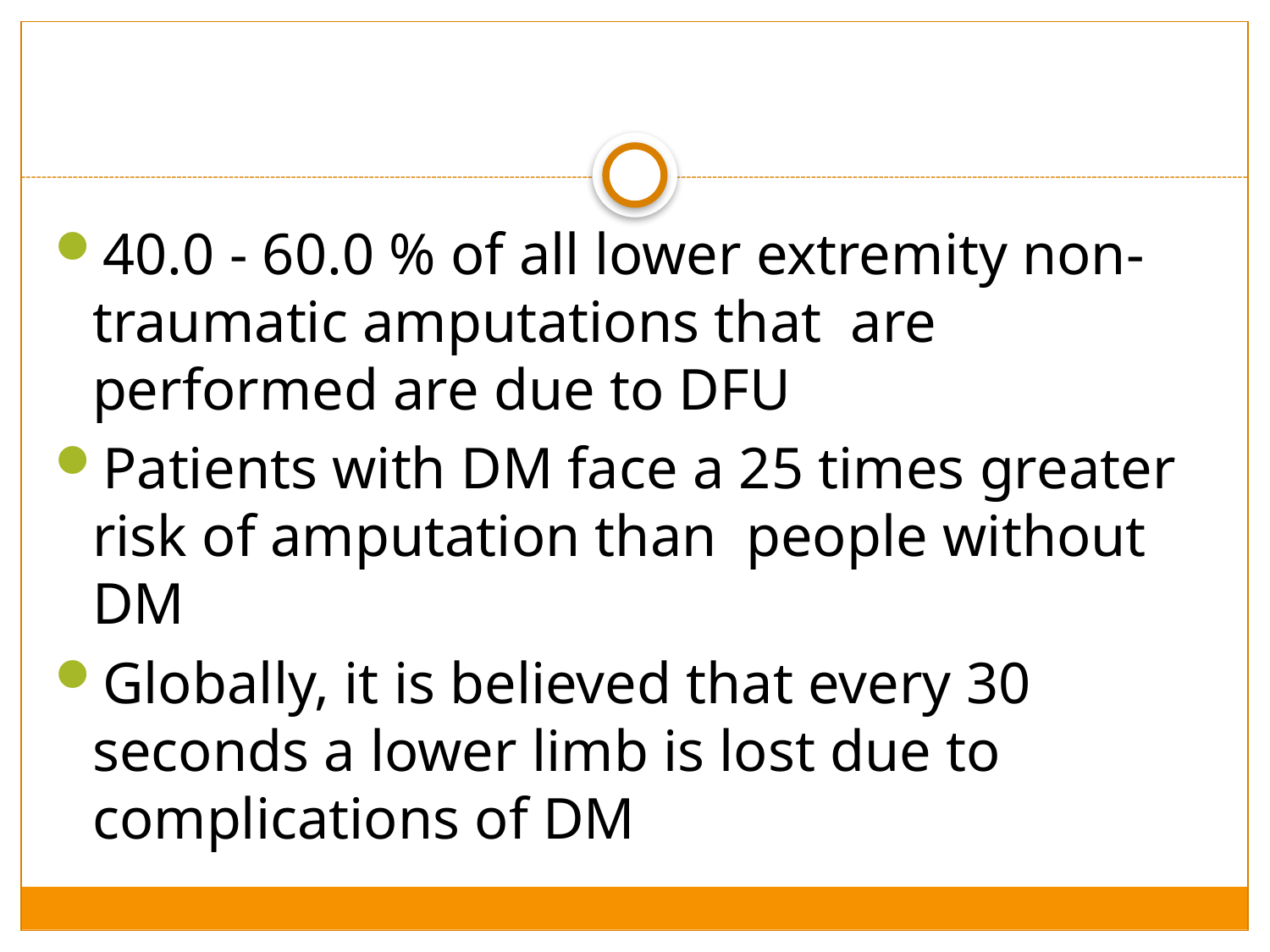

40.0 - 60.0 % of all lower extremity non-traumatic amputations that are performed are due to DFU
Patients with DM face a 25 times greater risk of amputation than people without DM
Globally, it is believed that every 30 seconds a lower limb is lost due to complications of DM

## Slide 6
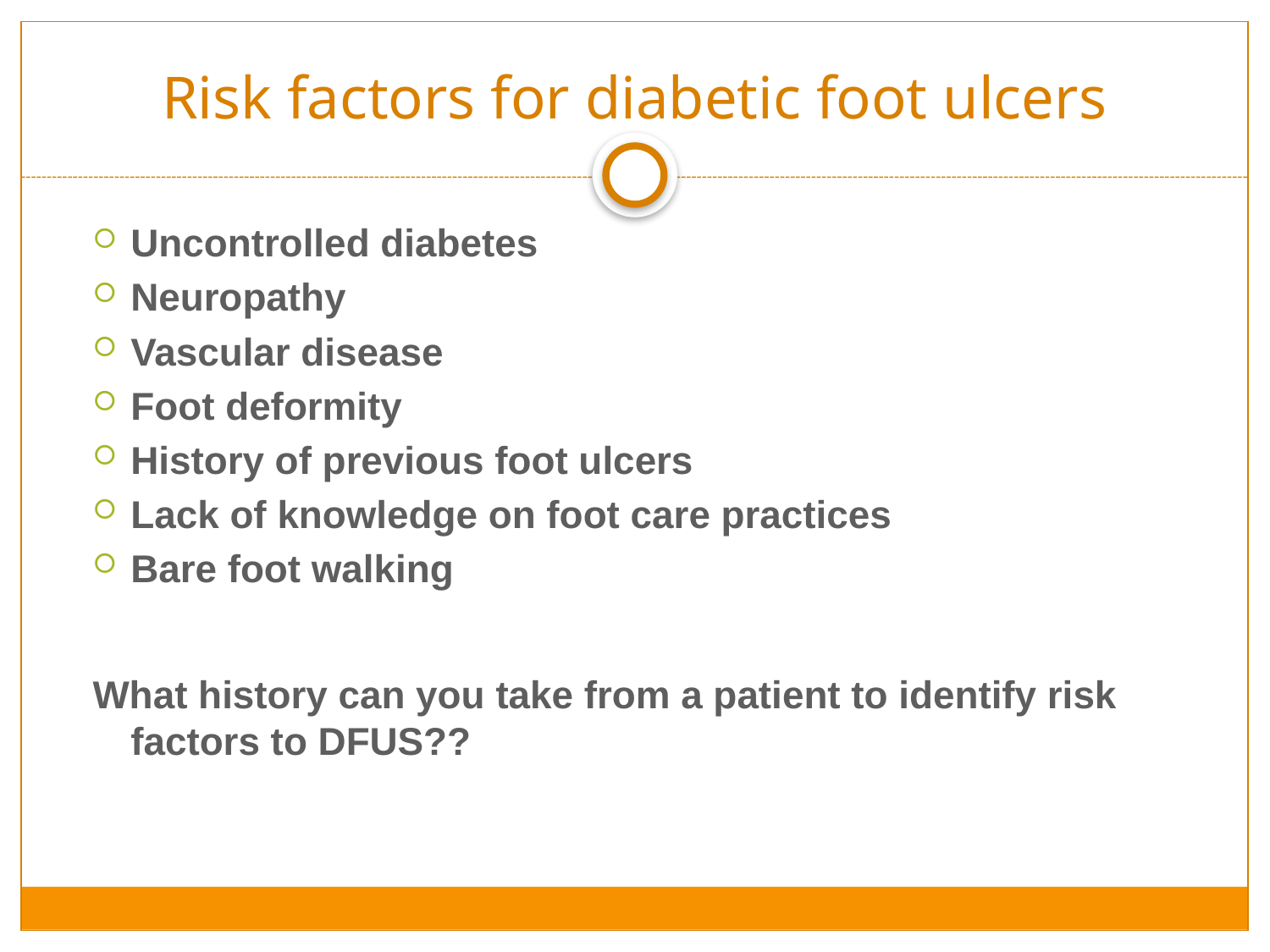

# Risk factors for diabetic foot ulcers
Uncontrolled diabetes
Neuropathy
Vascular disease
Foot deformity
History of previous foot ulcers
Lack of knowledge on foot care practices
Bare foot walking
What history can you take from a patient to identify risk factors to DFUS??

## Slide 7
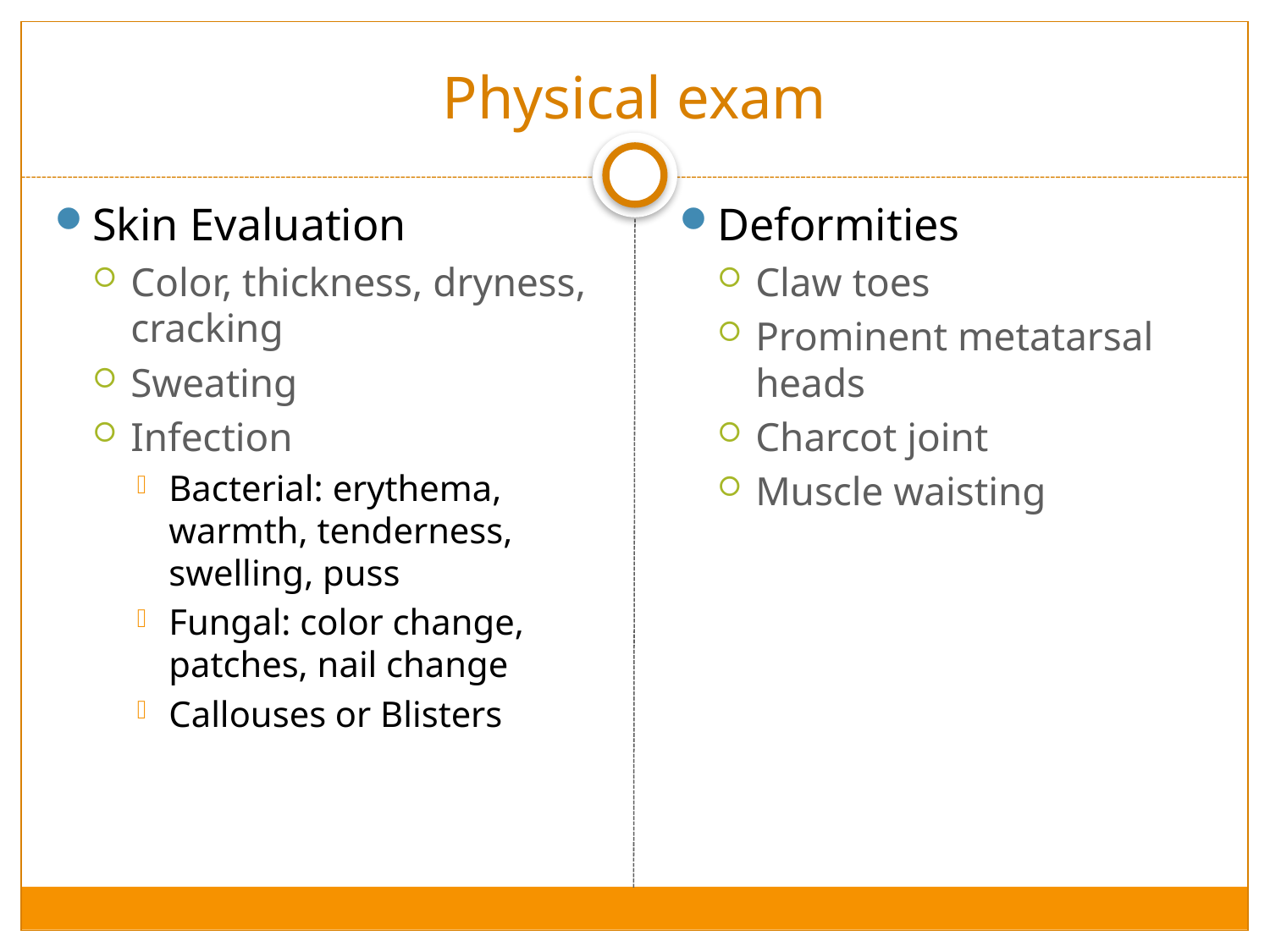

# Physical exam
Skin Evaluation
Color, thickness, dryness, cracking
Sweating
Infection
Bacterial: erythema, warmth, tenderness, swelling, puss
Fungal: color change, patches, nail change
Callouses or Blisters
Deformities
Claw toes
Prominent metatarsal heads
Charcot joint
Muscle waisting

## Slide 8
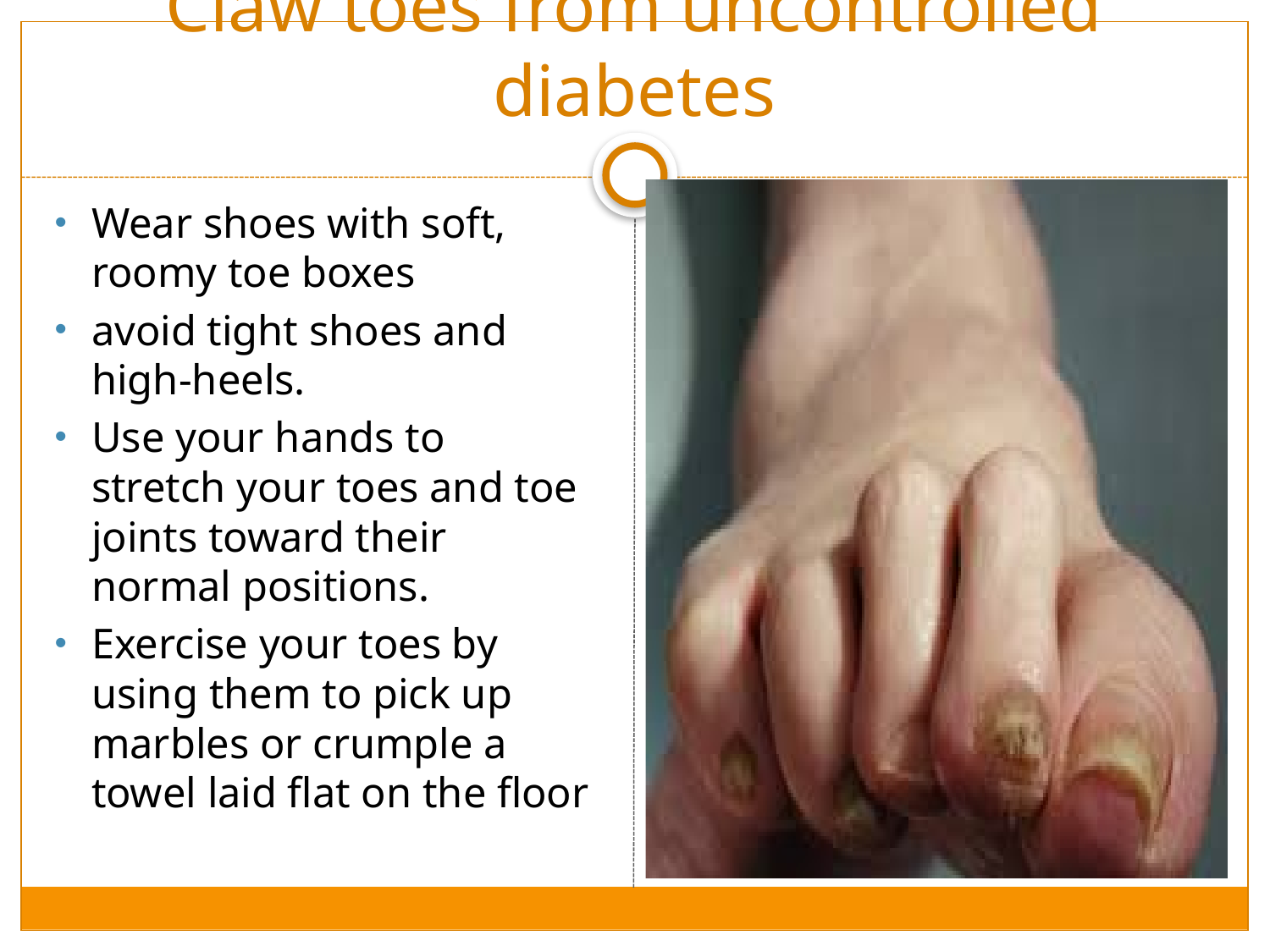

# Claw toes from uncontrolled diabetes
Wear shoes with soft, roomy toe boxes
avoid tight shoes and high-heels.
Use your hands to stretch your toes and toe joints toward their normal positions.
Exercise your toes by using them to pick up marbles or crumple a towel laid flat on the floor

## Slide 9
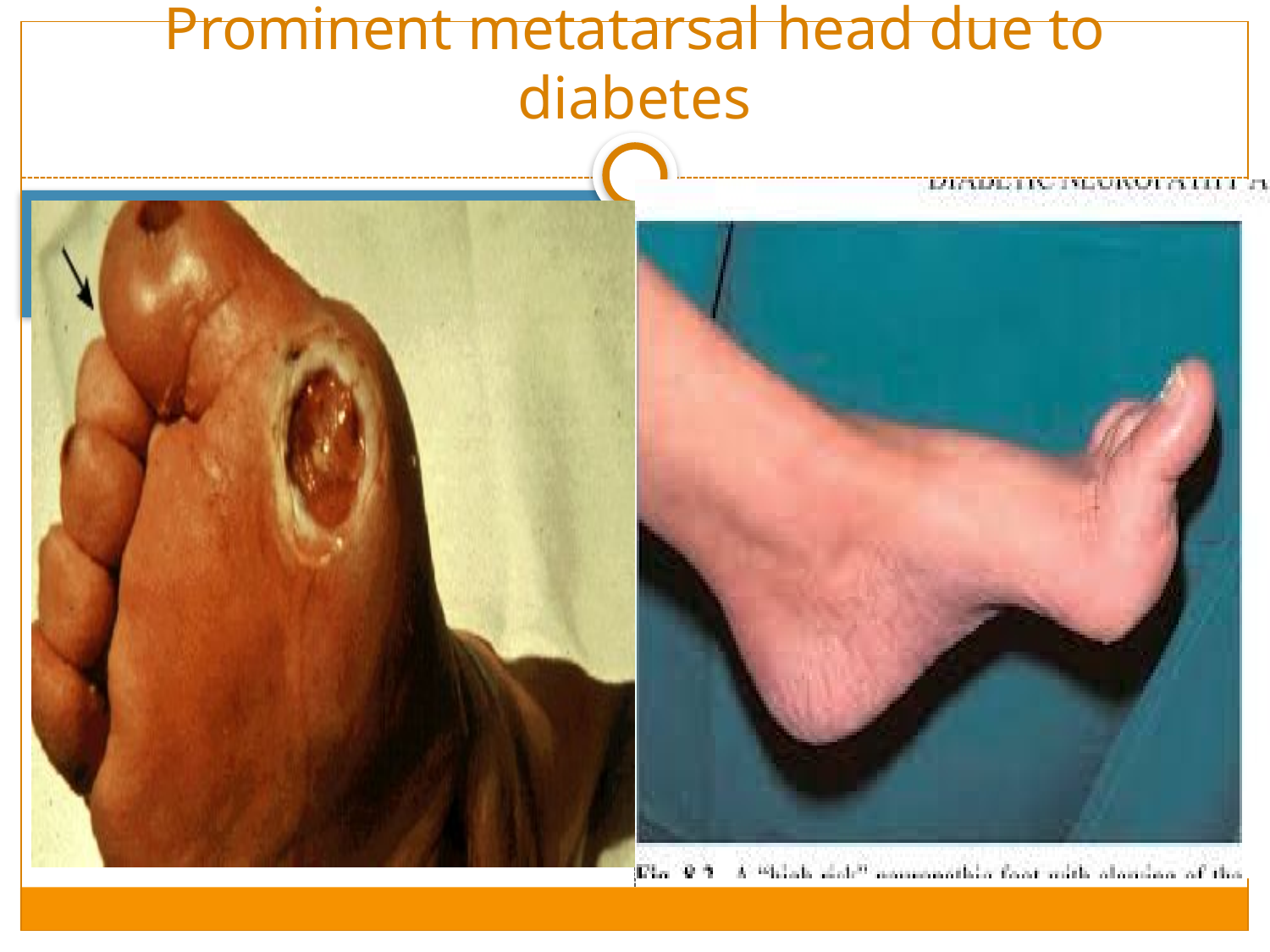

# Prominent metatarsal head due to diabetes

## Slide 10
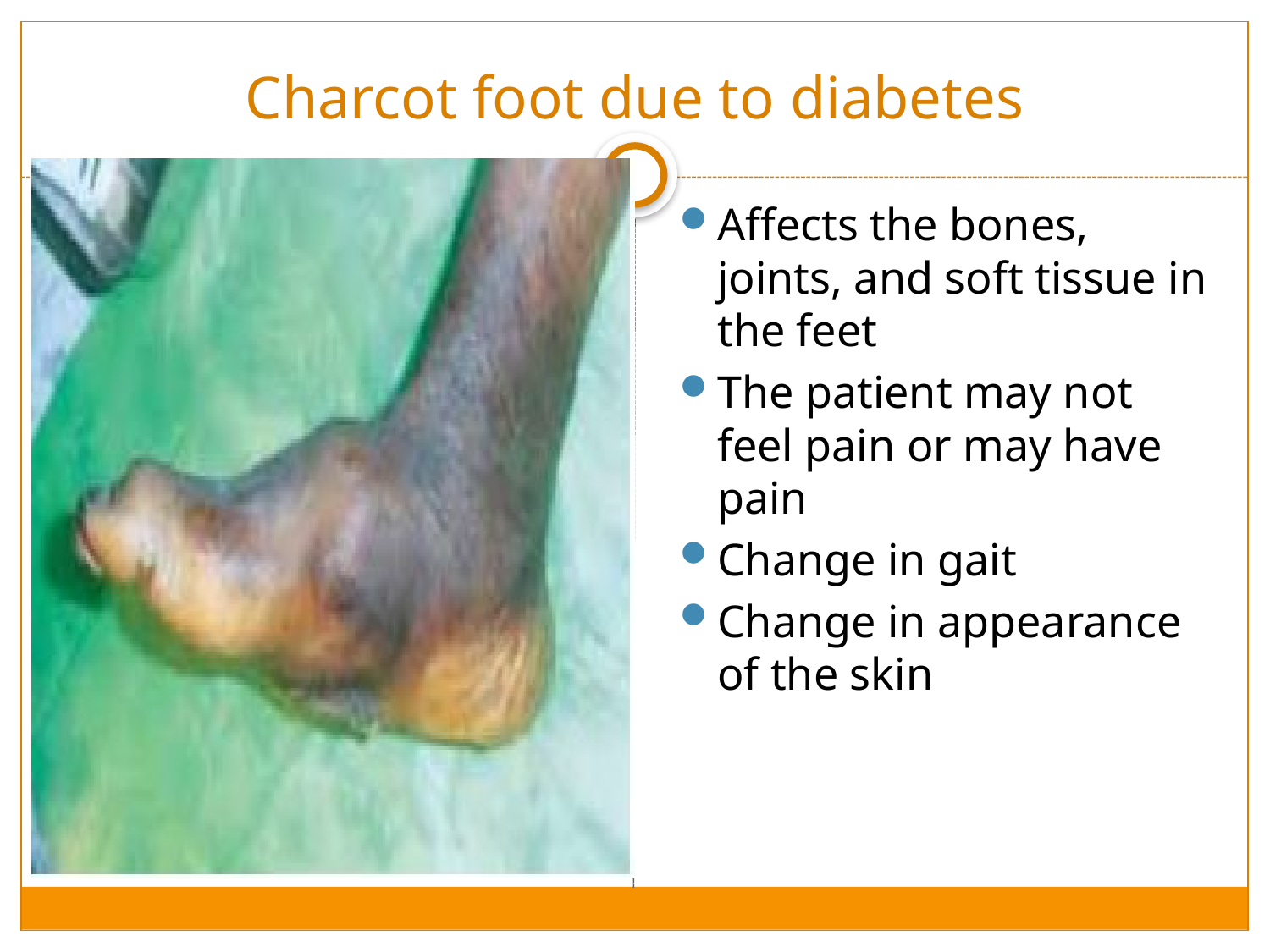

# Charcot foot due to diabetes
Affects the bones, joints, and soft tissue in the feet
The patient may not feel pain or may have pain
Change in gait
Change in appearance of the skin

## Slide 11
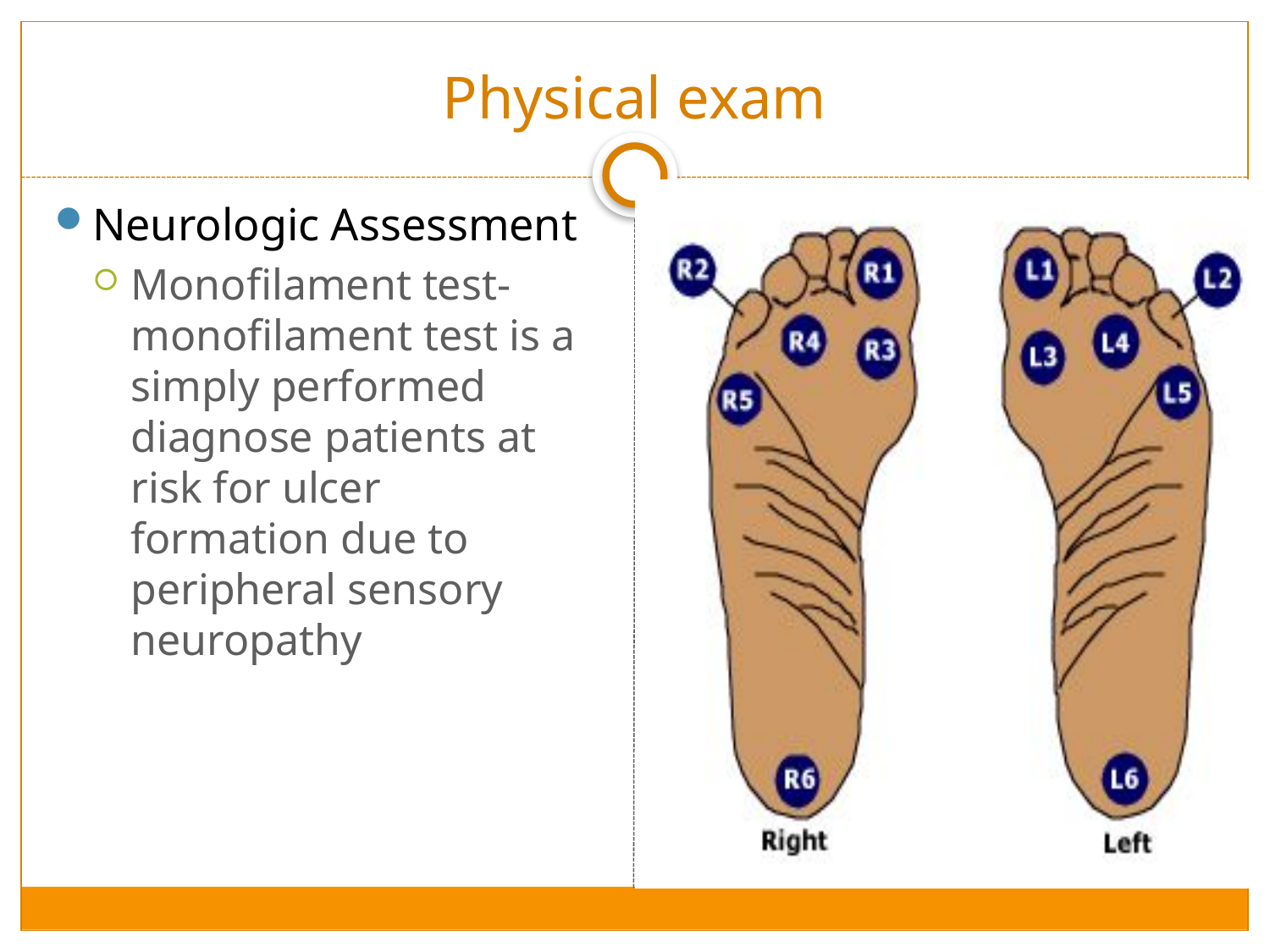

# Physical exam
Neurologic Assessment
Monofilament test-	monofilament test is a simply performed diagnose patients at risk for ulcer formation due to peripheral sensory neuropathy

## Slide 12
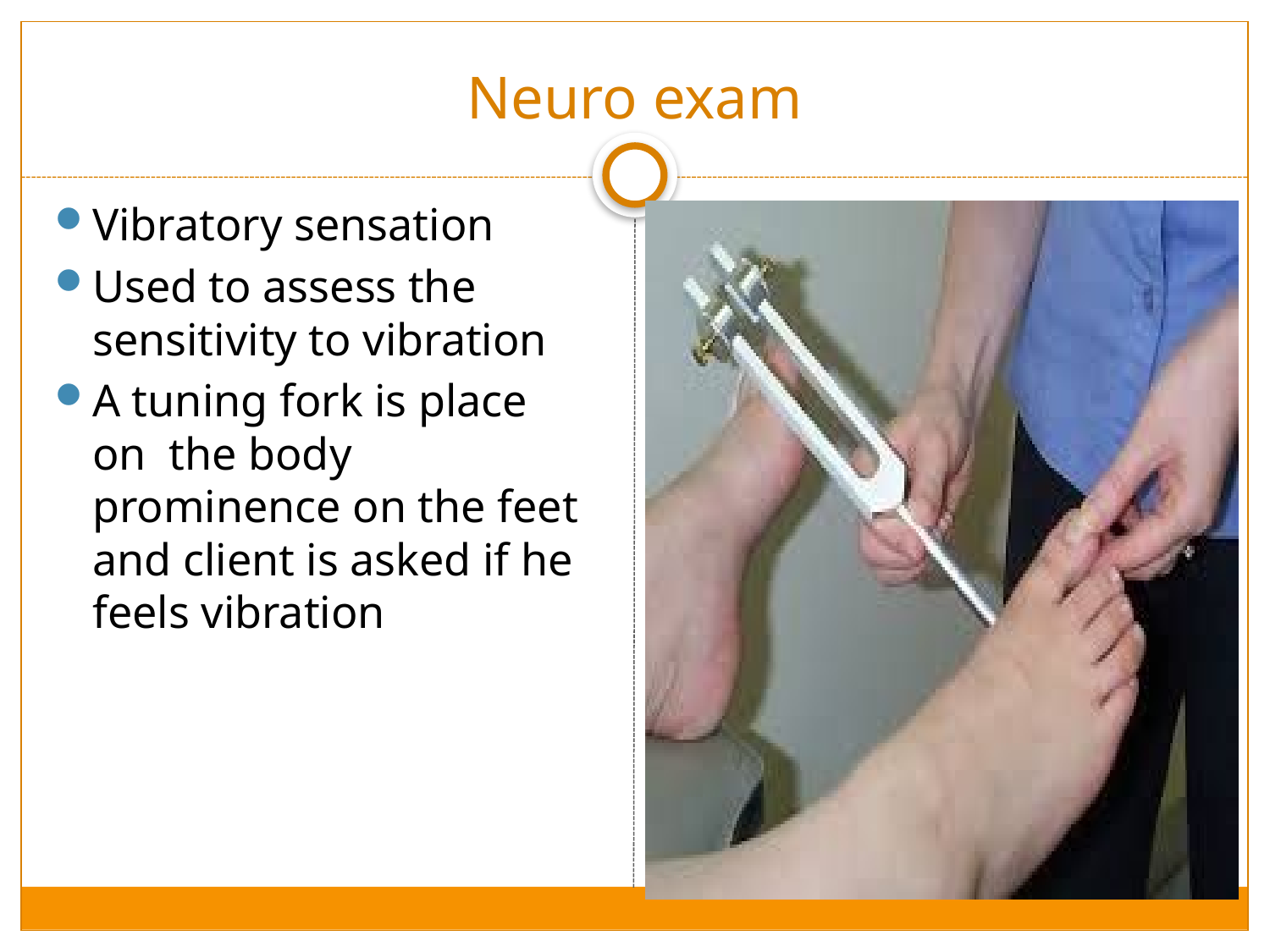

# Neuro exam
Vibratory sensation
Used to assess the sensitivity to vibration
A tuning fork is place on the body prominence on the feet and client is asked if he feels vibration

## Slide 13
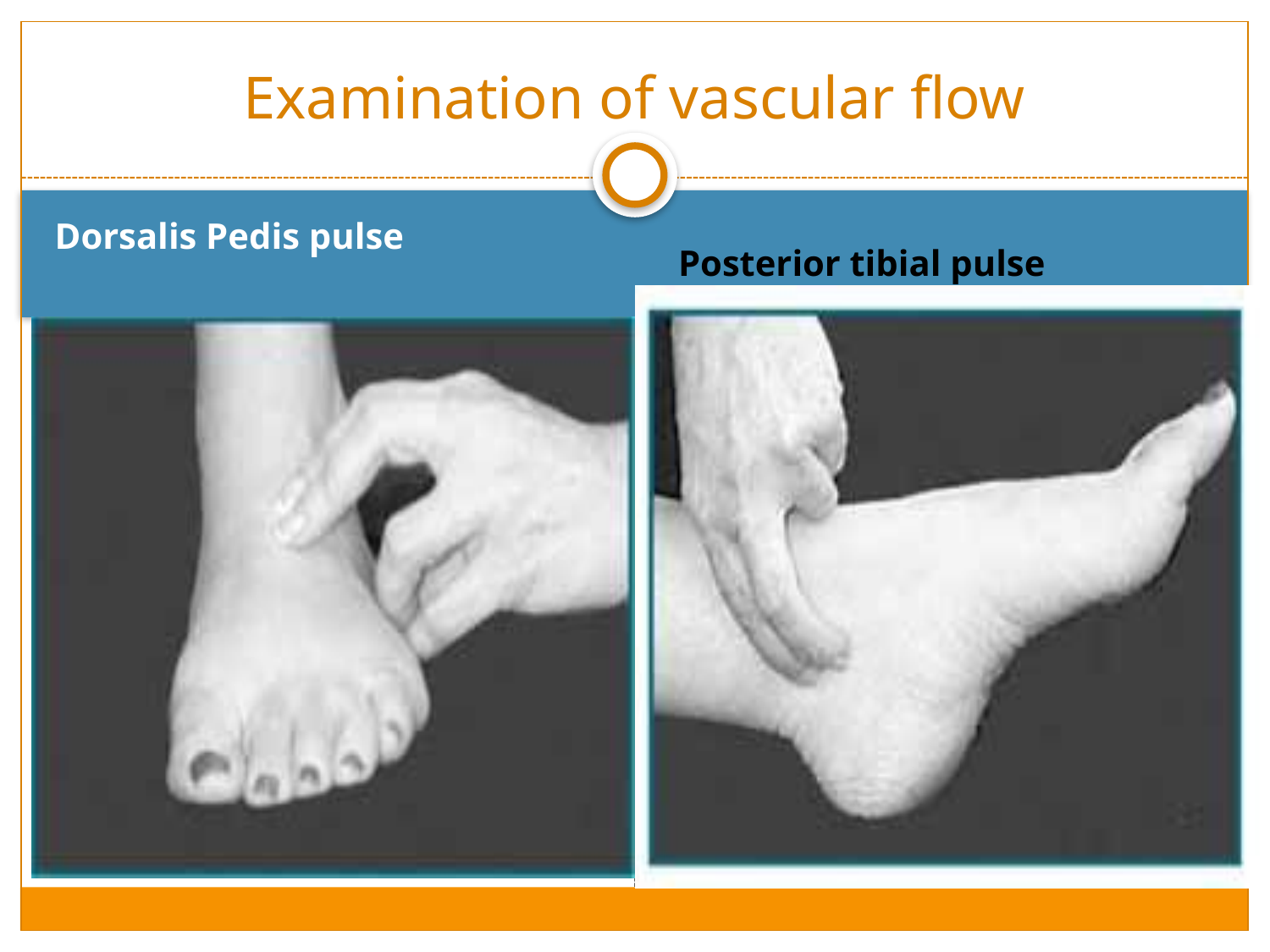

# Examination of vascular flow
Dorsalis Pedis pulse
Posterior tibial pulse

## Slide 14
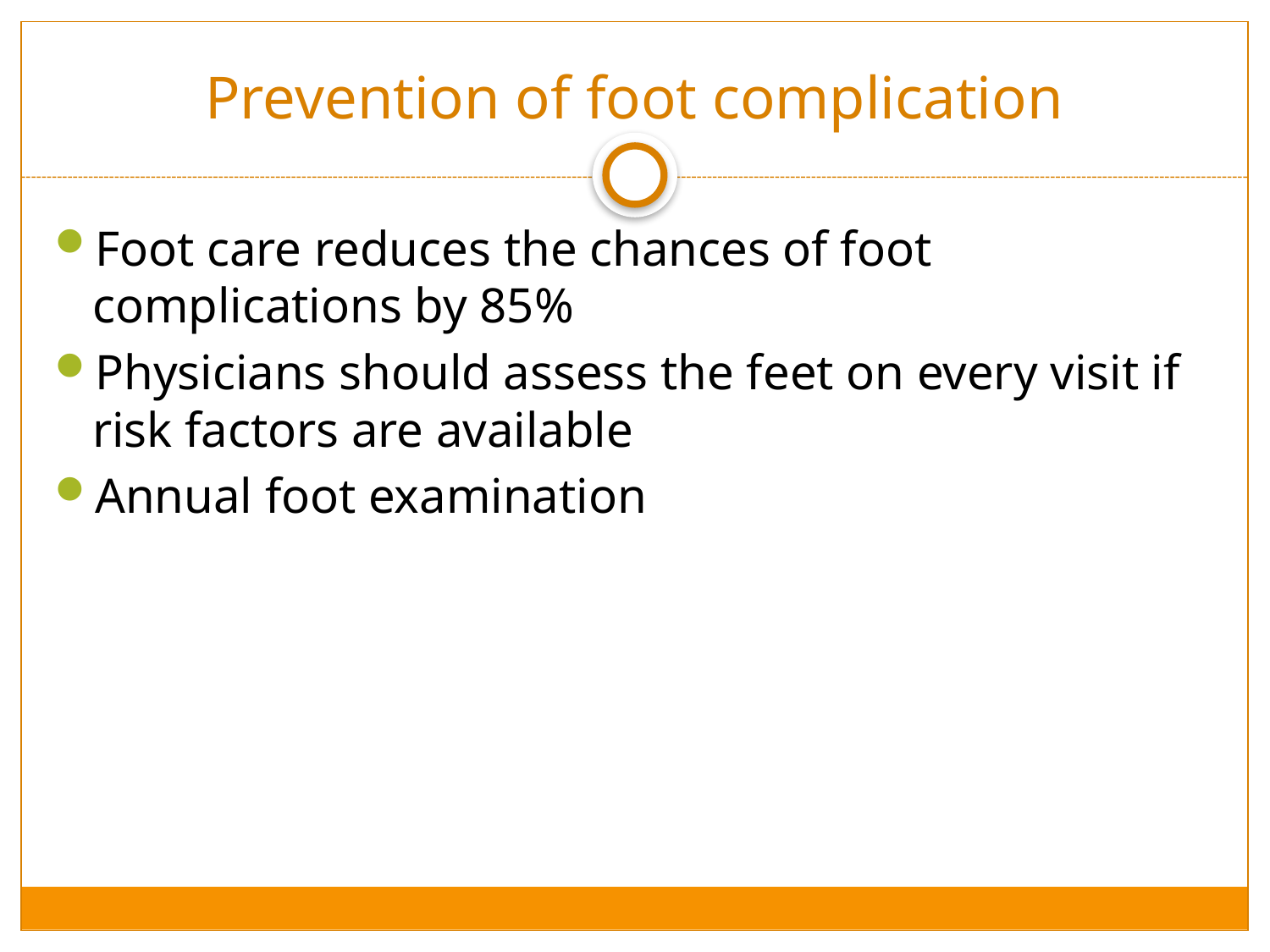

# Prevention of foot complication
Foot care reduces the chances of foot complications by 85%
Physicians should assess the feet on every visit if risk factors are available
Annual foot examination

## Slide 15
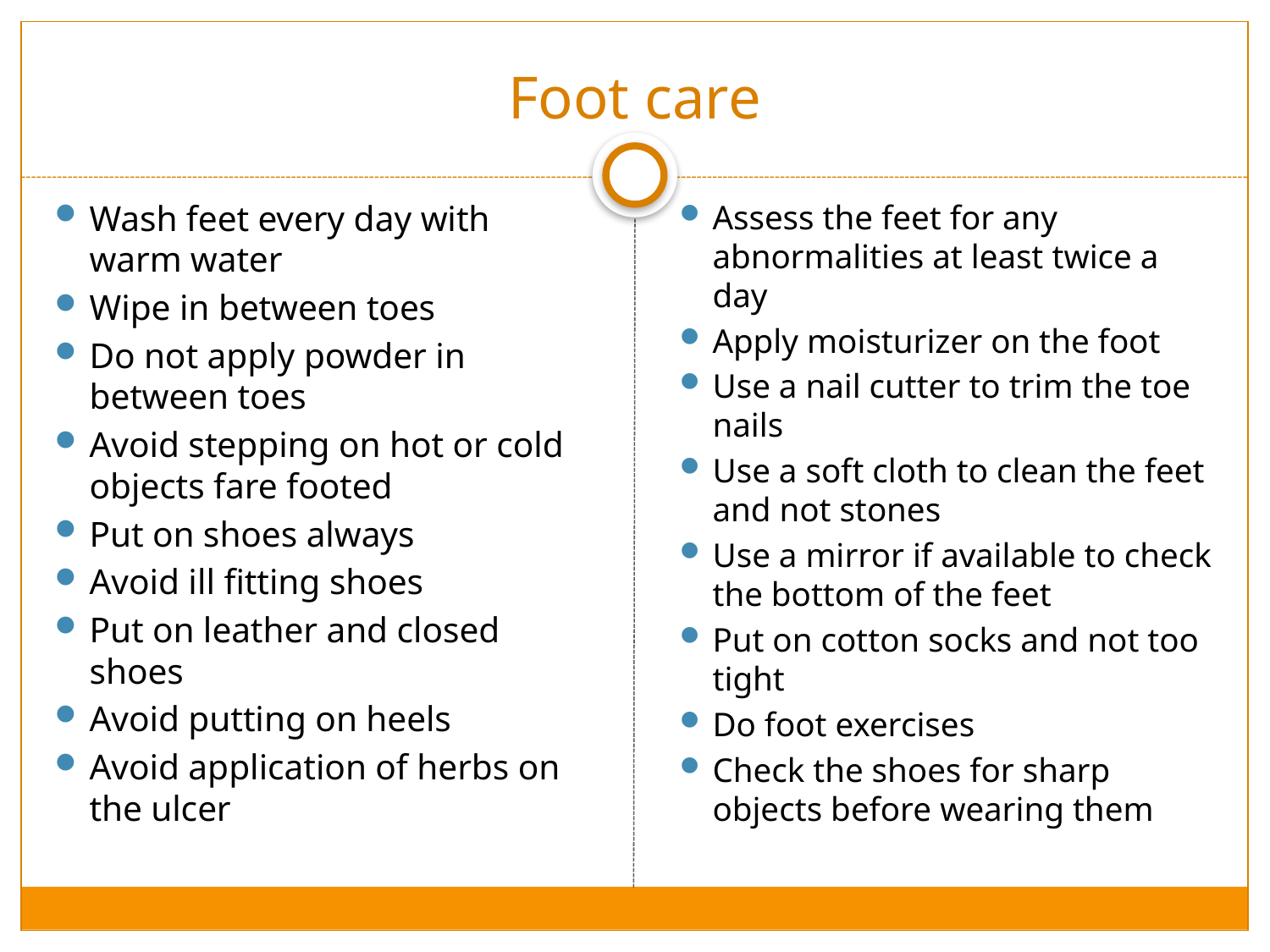

# Foot care
Wash feet every day with warm water
Wipe in between toes
Do not apply powder in between toes
Avoid stepping on hot or cold objects fare footed
Put on shoes always
Avoid ill fitting shoes
Put on leather and closed shoes
Avoid putting on heels
Avoid application of herbs on the ulcer
Assess the feet for any abnormalities at least twice a day
Apply moisturizer on the foot
Use a nail cutter to trim the toe nails
Use a soft cloth to clean the feet and not stones
Use a mirror if available to check the bottom of the feet
Put on cotton socks and not too tight
Do foot exercises
Check the shoes for sharp objects before wearing them

## Slide 16
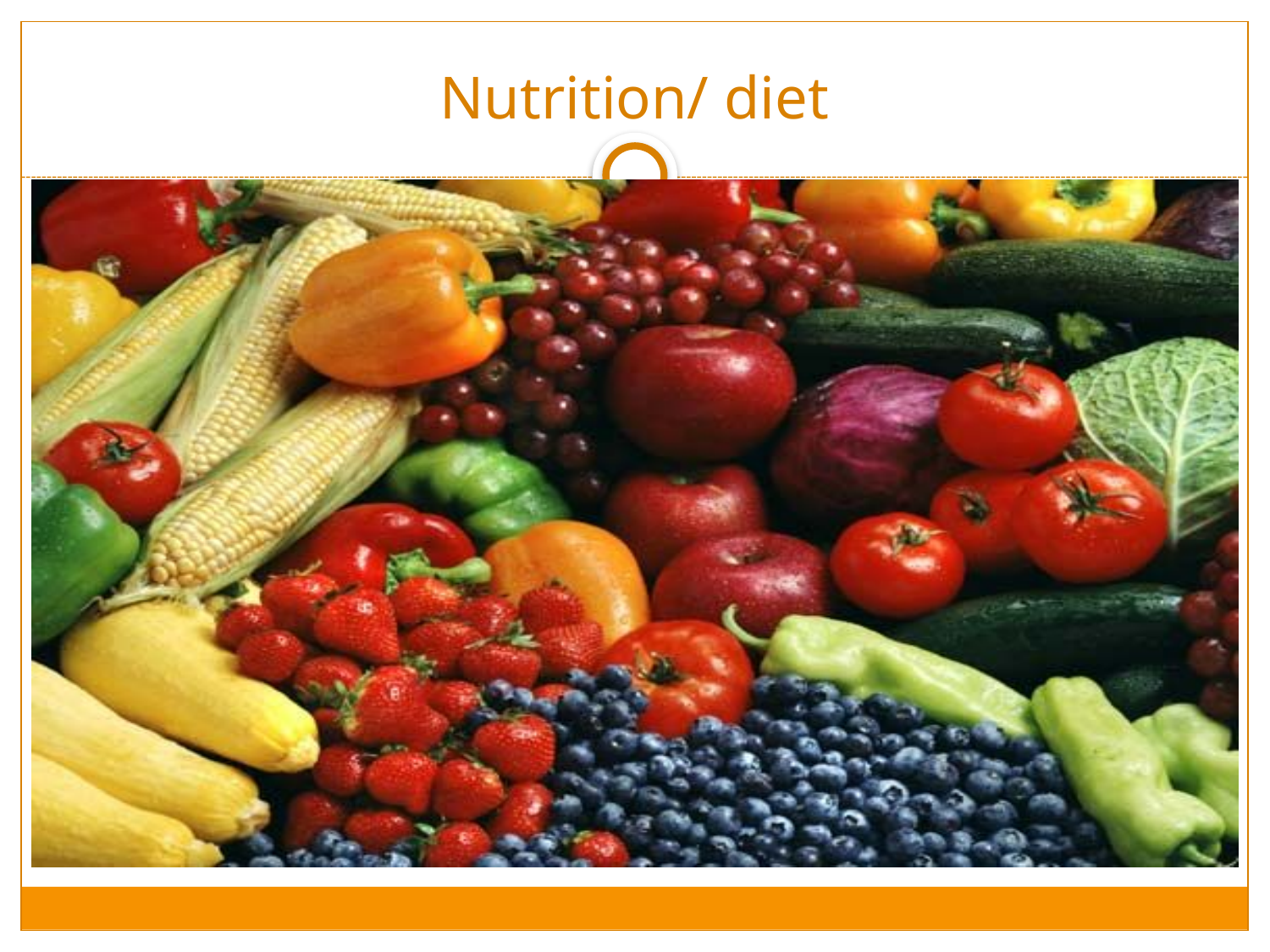

# Nutrition/ diet

## Slide 17
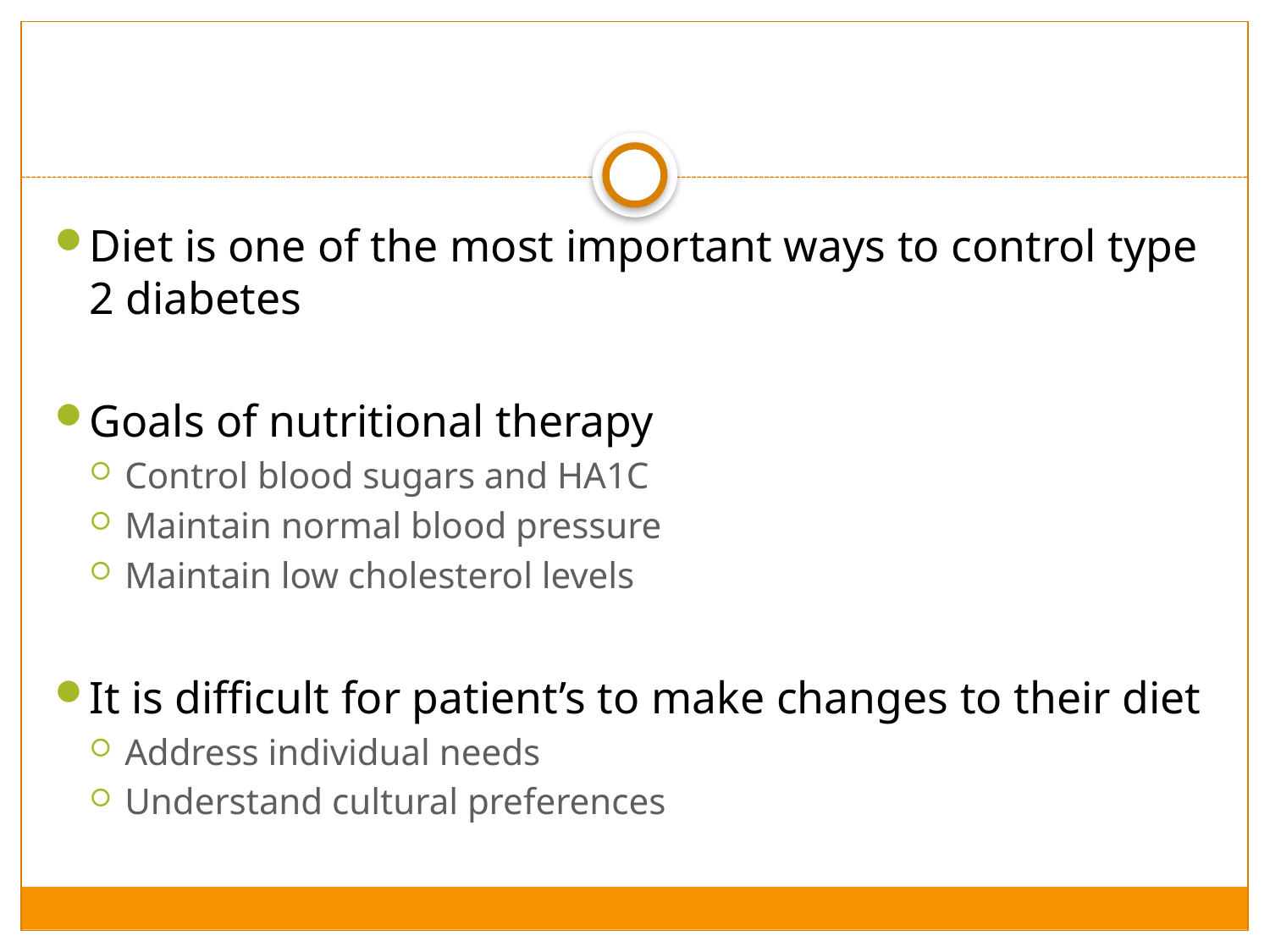

Diet is one of the most important ways to control type 2 diabetes
Goals of nutritional therapy
Control blood sugars and HA1C
Maintain normal blood pressure
Maintain low cholesterol levels
It is difficult for patient’s to make changes to their diet
Address individual needs
Understand cultural preferences

## Slide 18
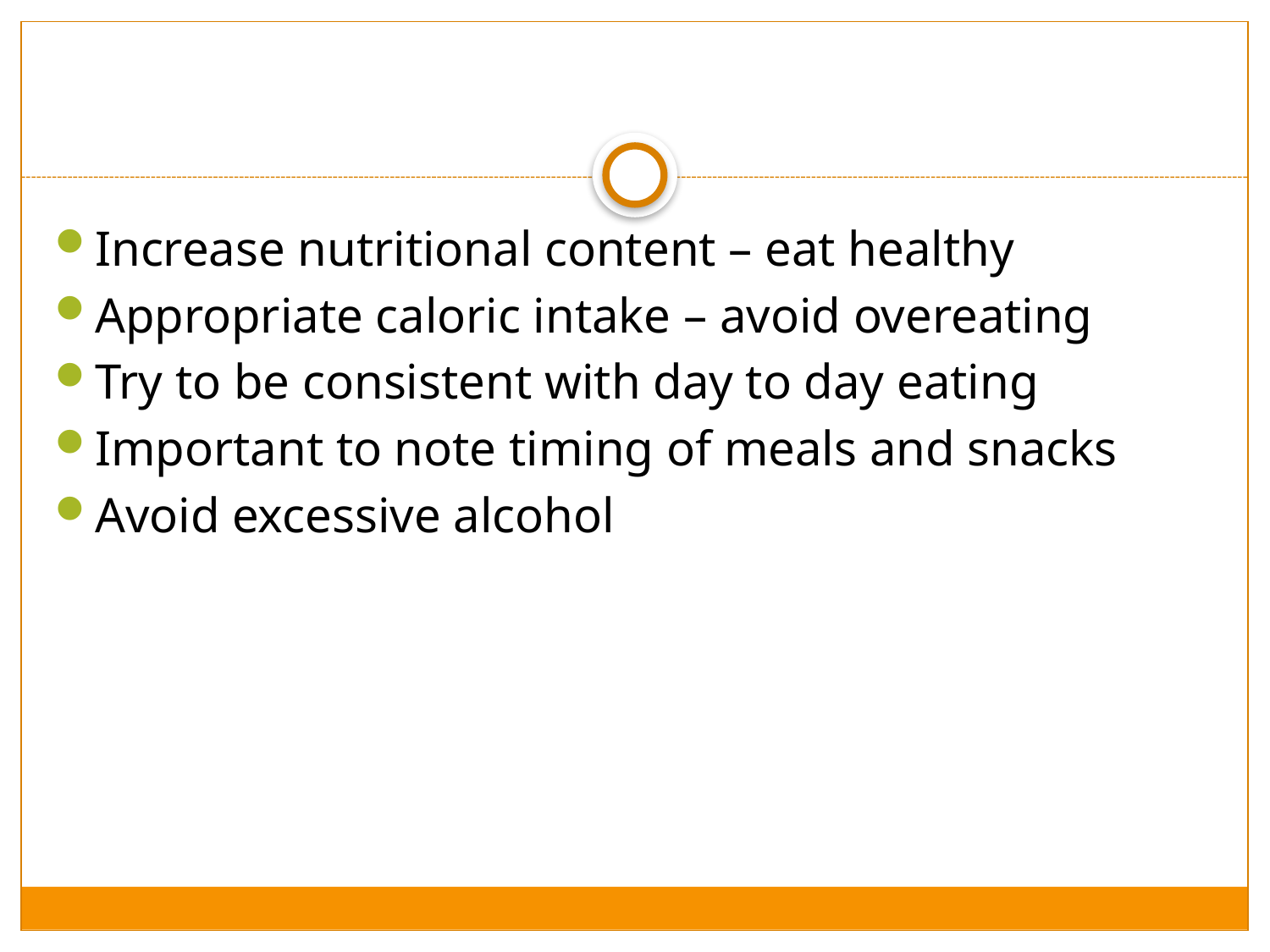

Increase nutritional content – eat healthy
Appropriate caloric intake – avoid overeating
Try to be consistent with day to day eating
Important to note timing of meals and snacks
Avoid excessive alcohol

## Slide 19
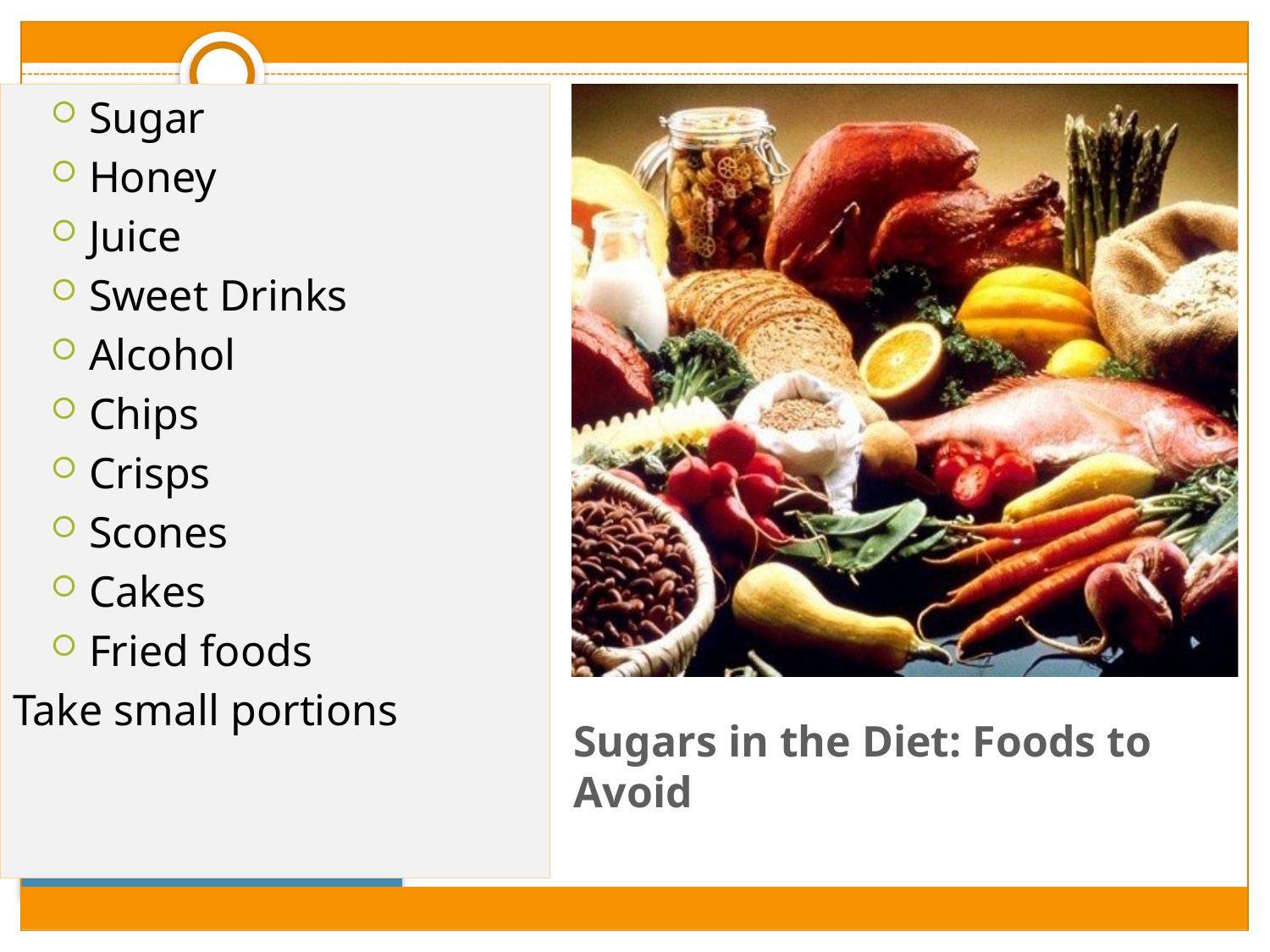

Sugar
Honey
Juice
Sweet Drinks
Alcohol
Chips
Crisps
Scones
Cakes
Fried foods
Take small portions
# Sugars in the Diet: Foods to Avoid

## Slide 20
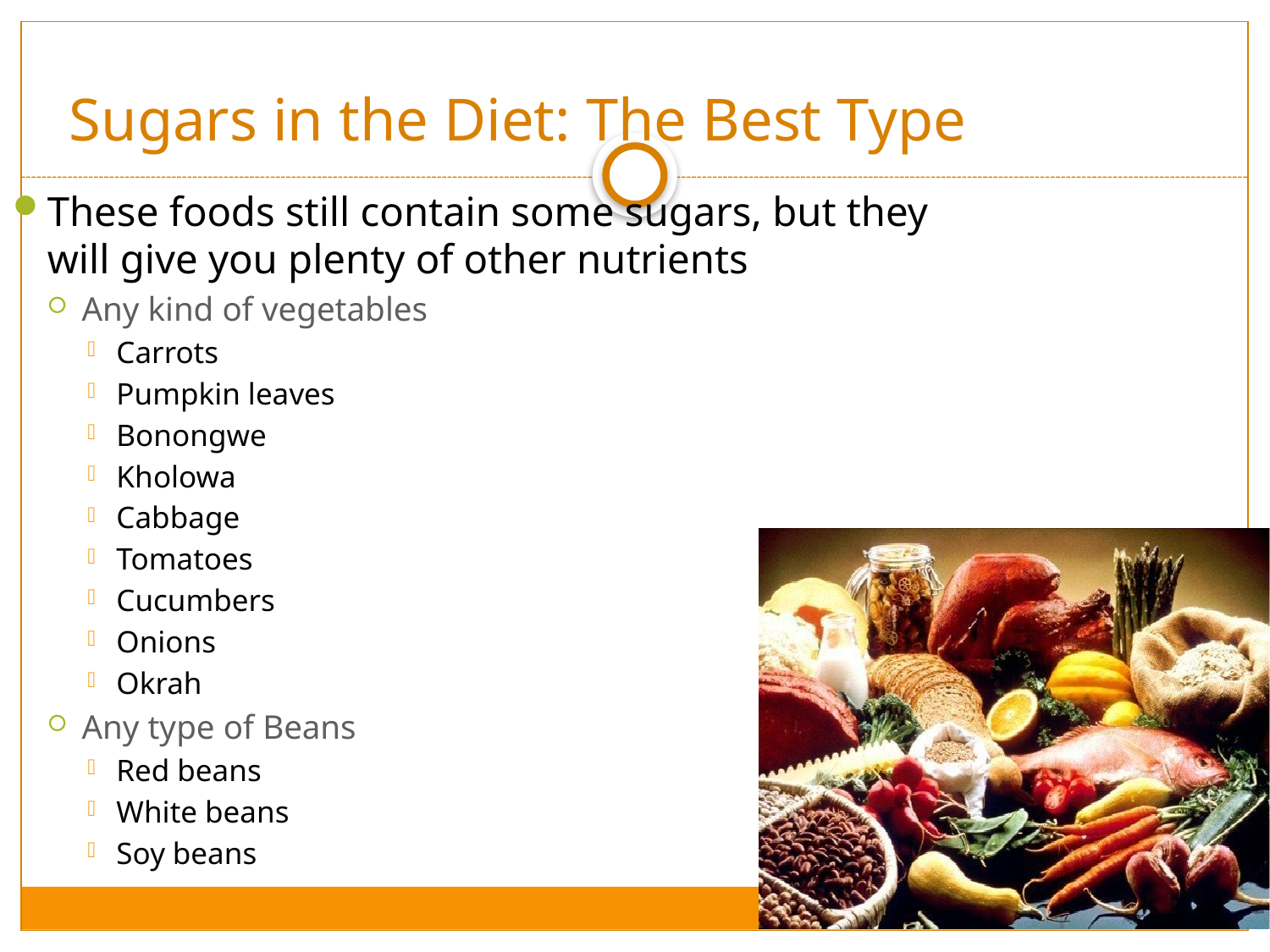

# Sugars in the Diet: The Best Type
These foods still contain some sugars, but they will give you plenty of other nutrients
Any kind of vegetables
Carrots
Pumpkin leaves
Bonongwe
Kholowa
Cabbage
Tomatoes
Cucumbers
Onions
Okrah
Any type of Beans
Red beans
White beans
Soy beans

## Slide 21
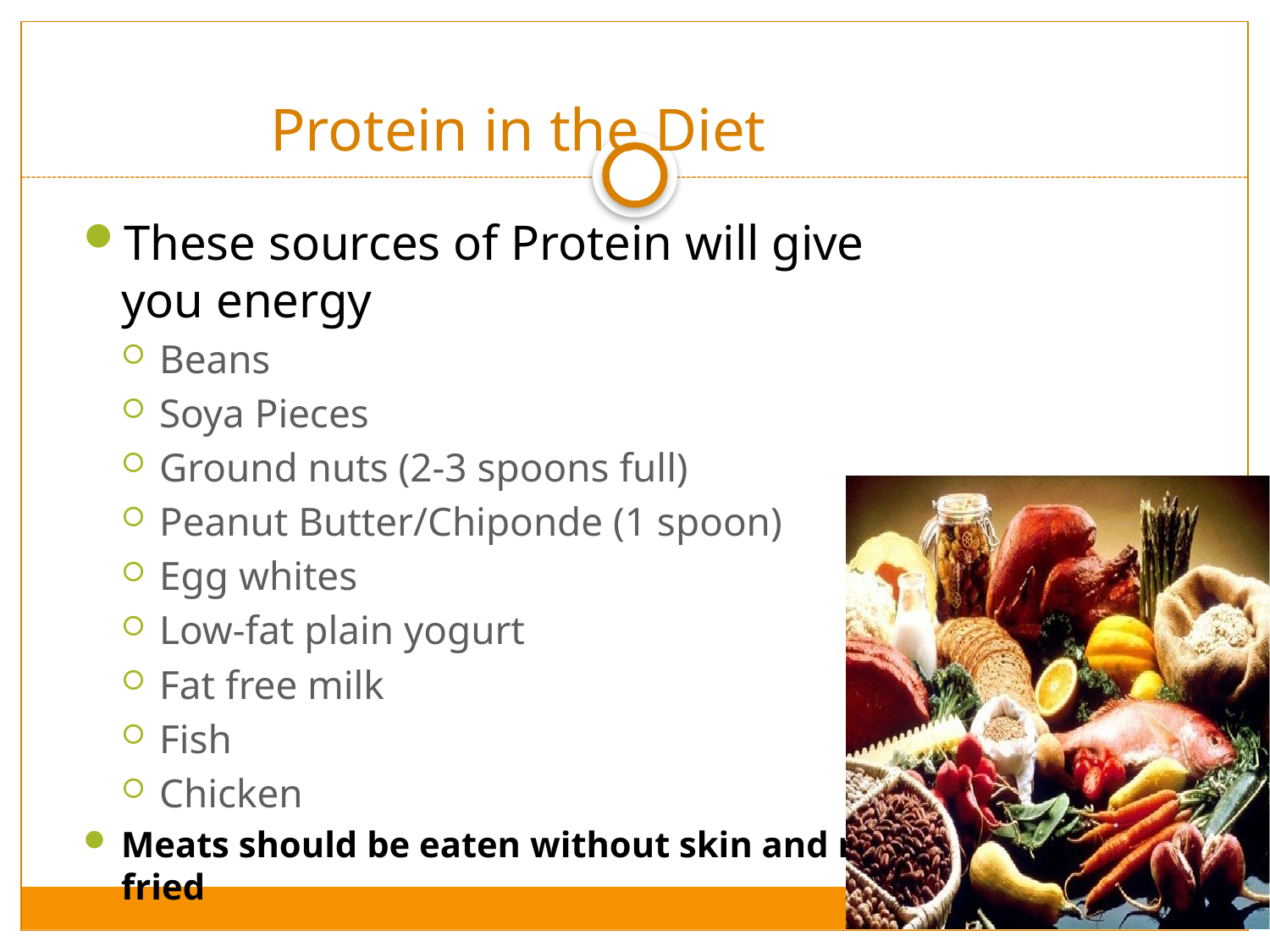

# Protein in the Diet
These sources of Protein will give you energy
Beans
Soya Pieces
Ground nuts (2-3 spoons full)
Peanut Butter/Chiponde (1 spoon)
Egg whites
Low-fat plain yogurt
Fat free milk
Fish
Chicken
Meats should be eaten without skin and not fried

## Slide 22
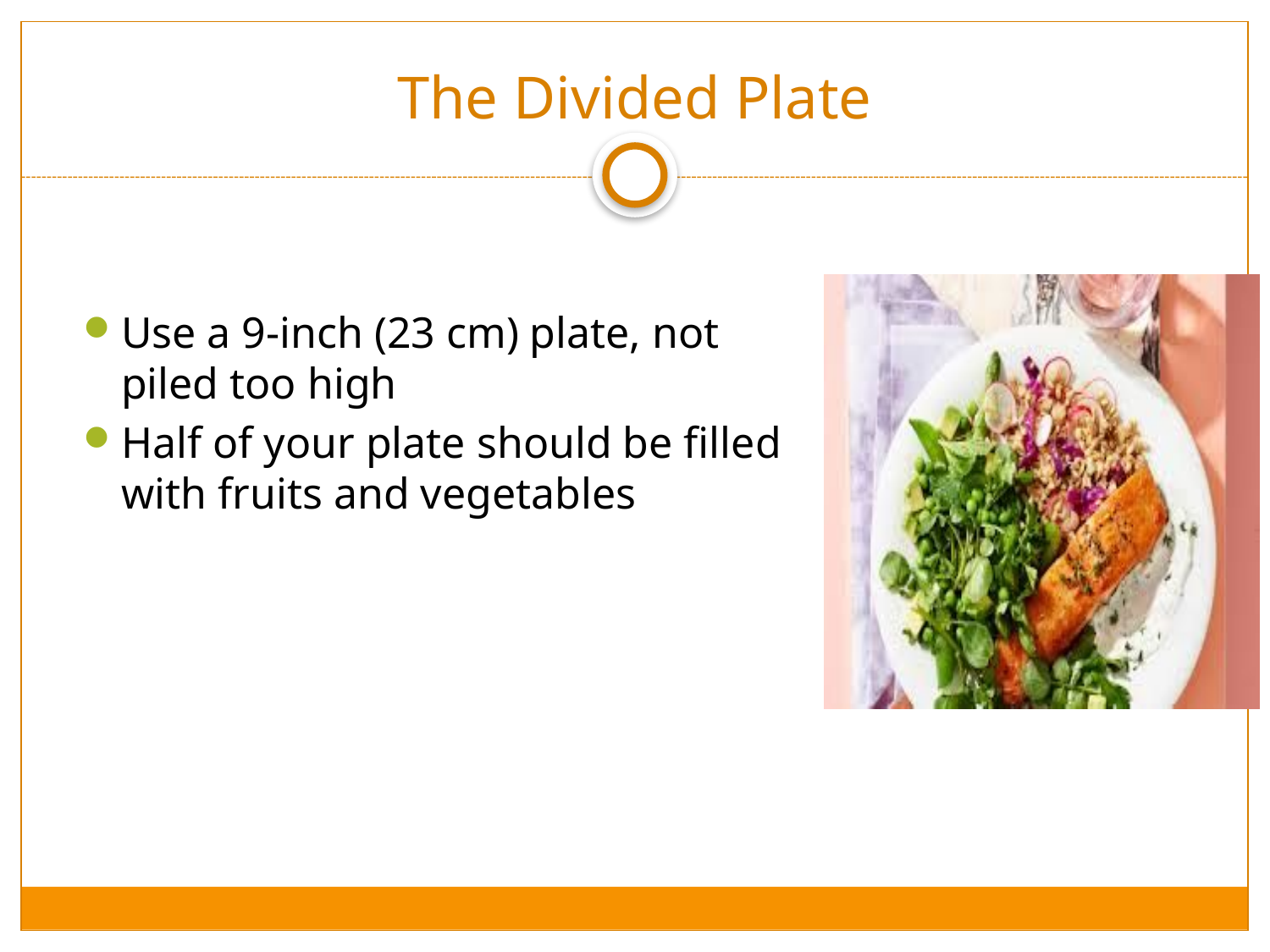

# The Divided Plate
Use a 9-inch (23 cm) plate, not piled too high
Half of your plate should be filled with fruits and vegetables

## Slide 23
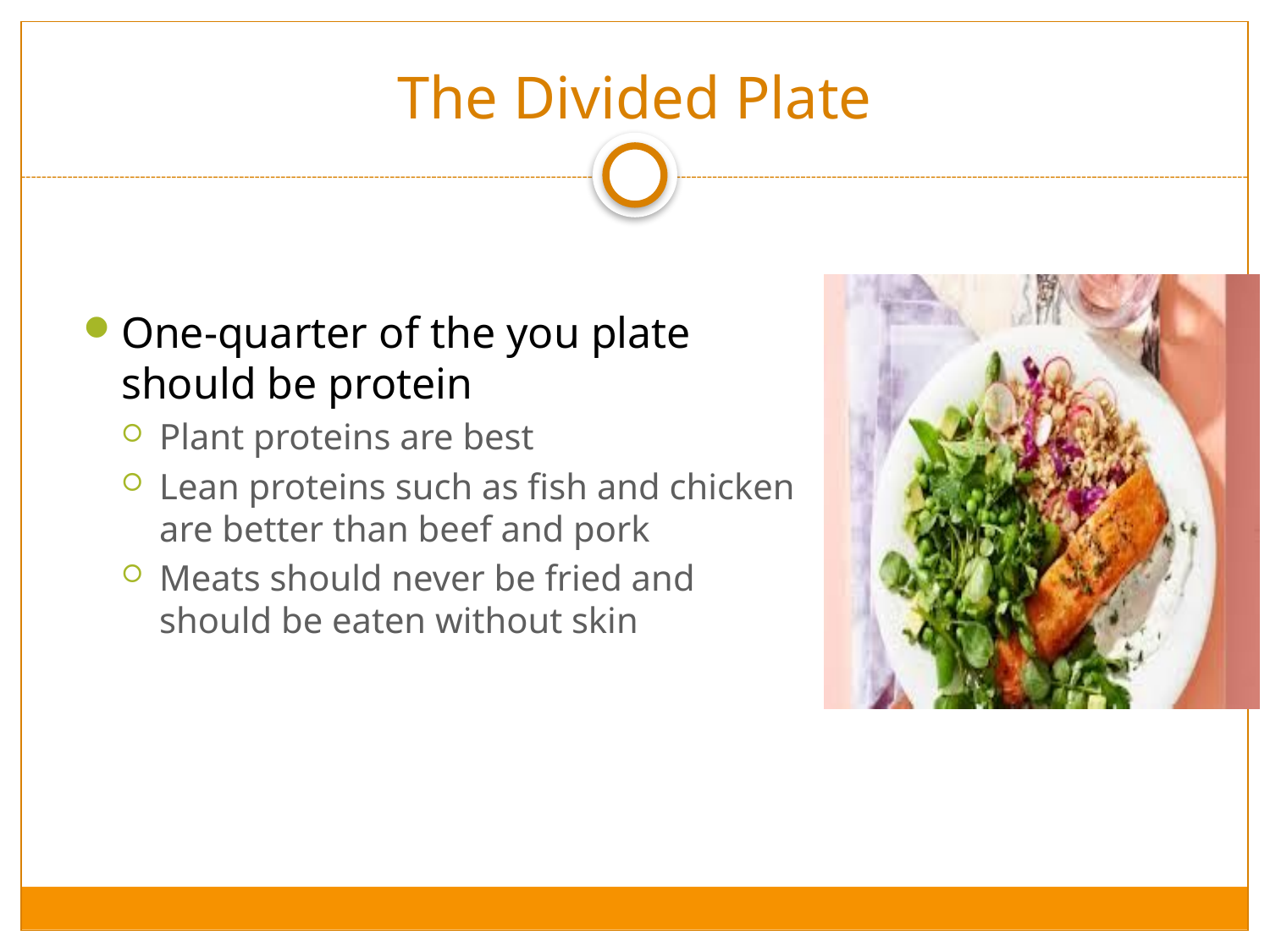

# The Divided Plate
One-quarter of the you plate should be protein
Plant proteins are best
Lean proteins such as fish and chicken are better than beef and pork
Meats should never be fried and should be eaten without skin

## Slide 24
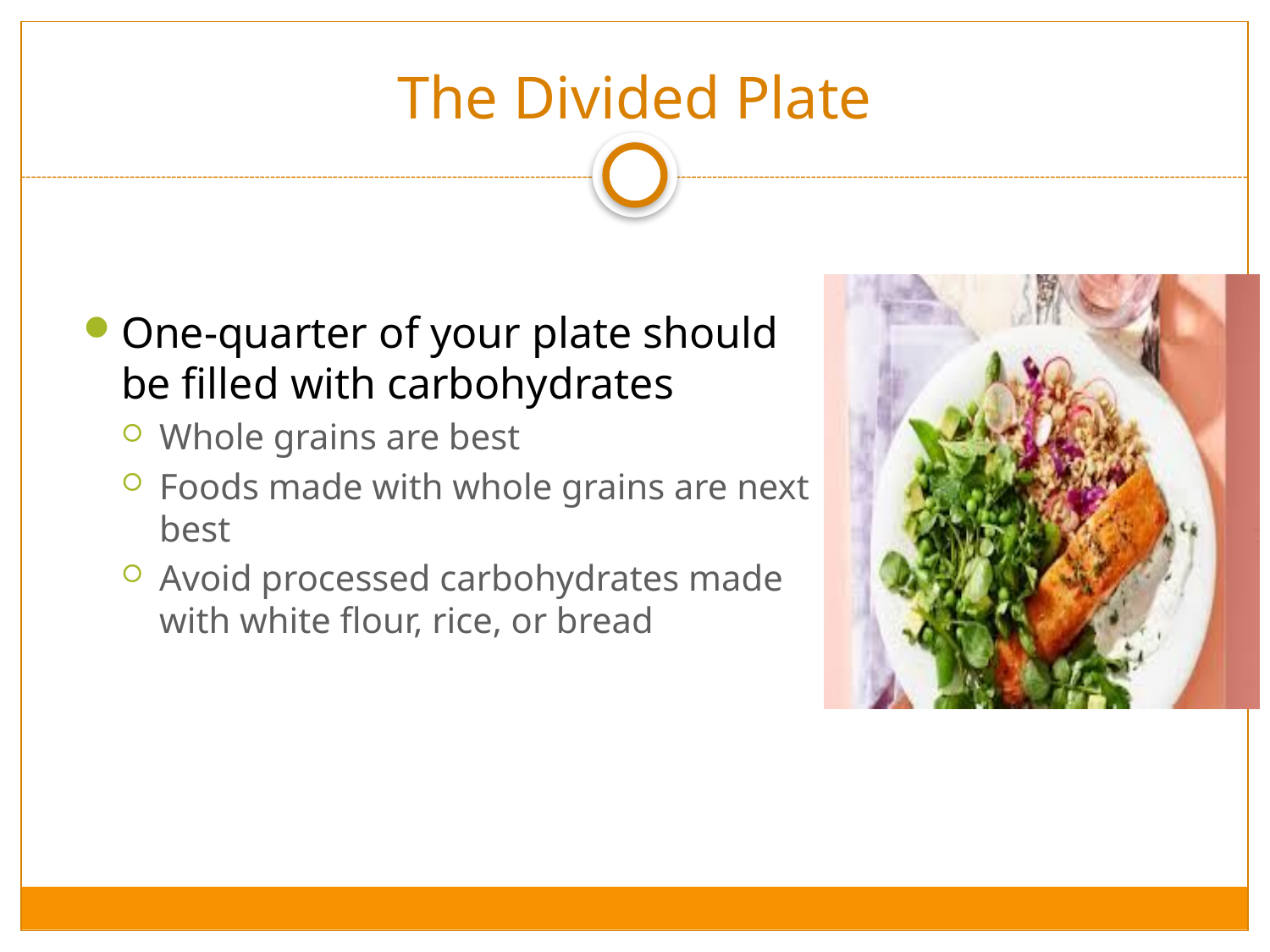

# The Divided Plate
One-quarter of your plate should be filled with carbohydrates
Whole grains are best
Foods made with whole grains are next best
Avoid processed carbohydrates made with white flour, rice, or bread

## Slide 25
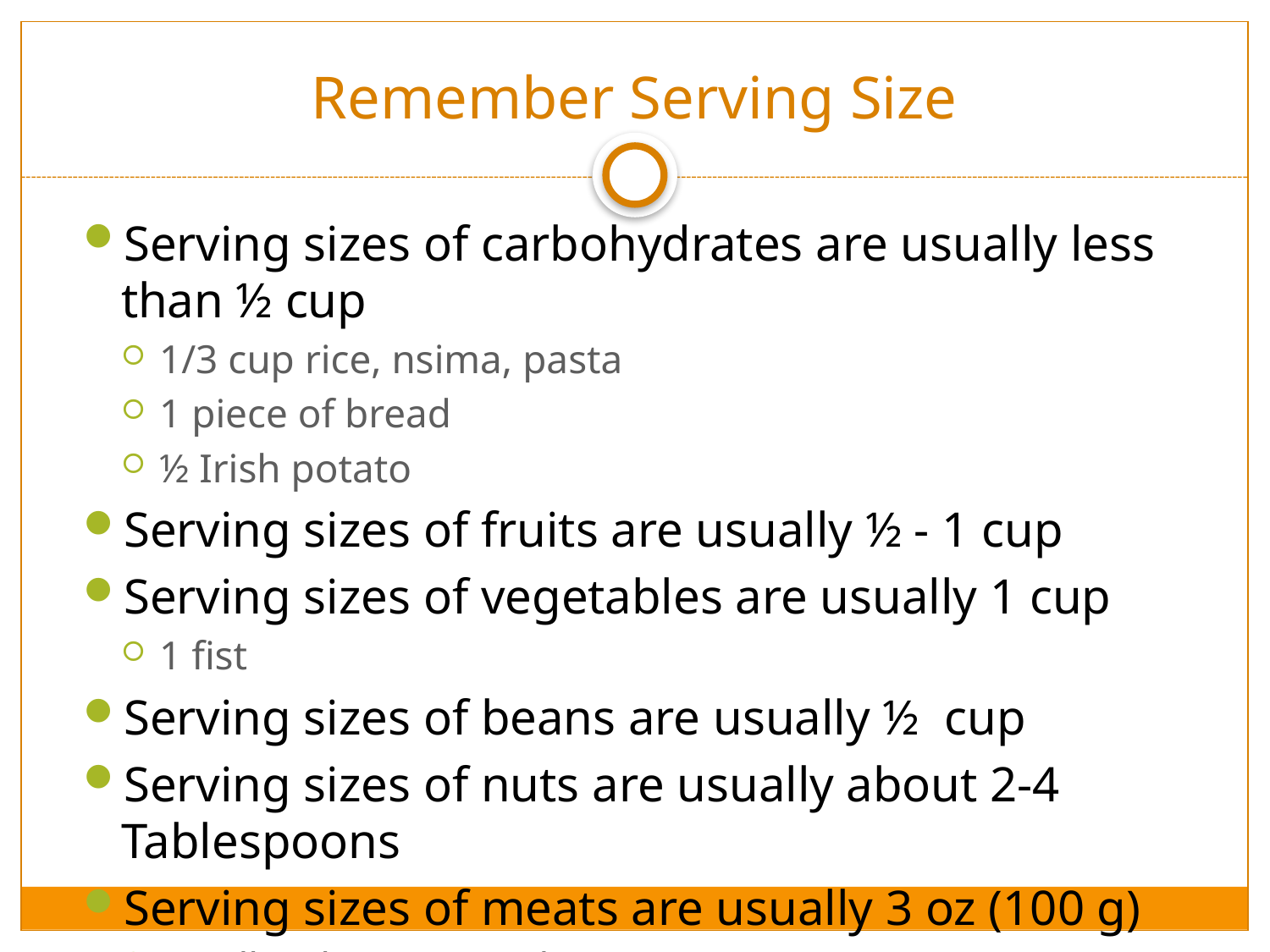

# Remember Serving Size
Serving sizes of carbohydrates are usually less than ½ cup
1/3 cup rice, nsima, pasta
1 piece of bread
½ Irish potato
Serving sizes of fruits are usually ½ - 1 cup
Serving sizes of vegetables are usually 1 cup
1 fist
Serving sizes of beans are usually ½ cup
Serving sizes of nuts are usually about 2-4 Tablespoons
Serving sizes of meats are usually 3 oz (100 g)
Smaller than your palm

## Slide 26
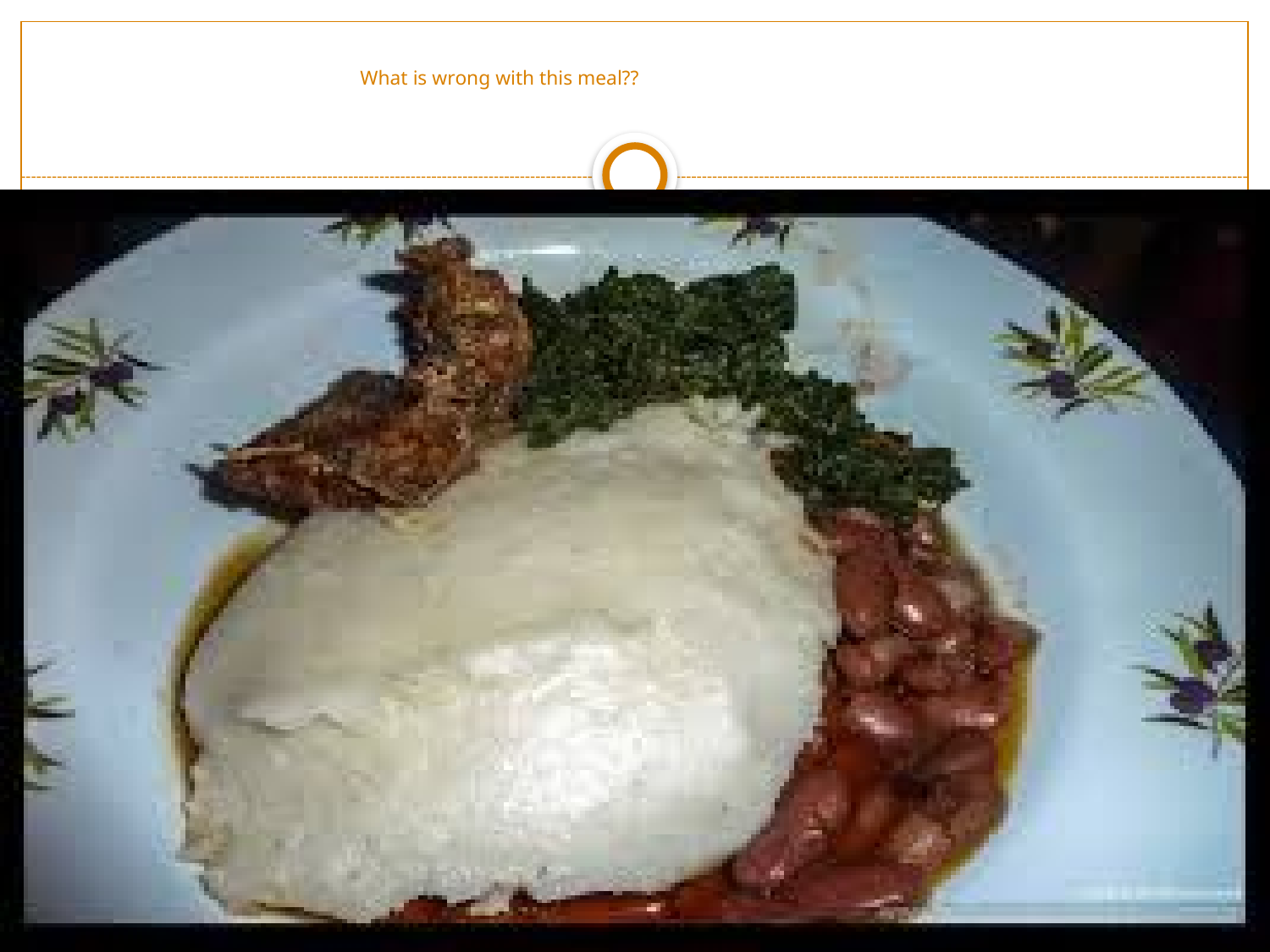

# What is wrong with this meal??

## Slide 27
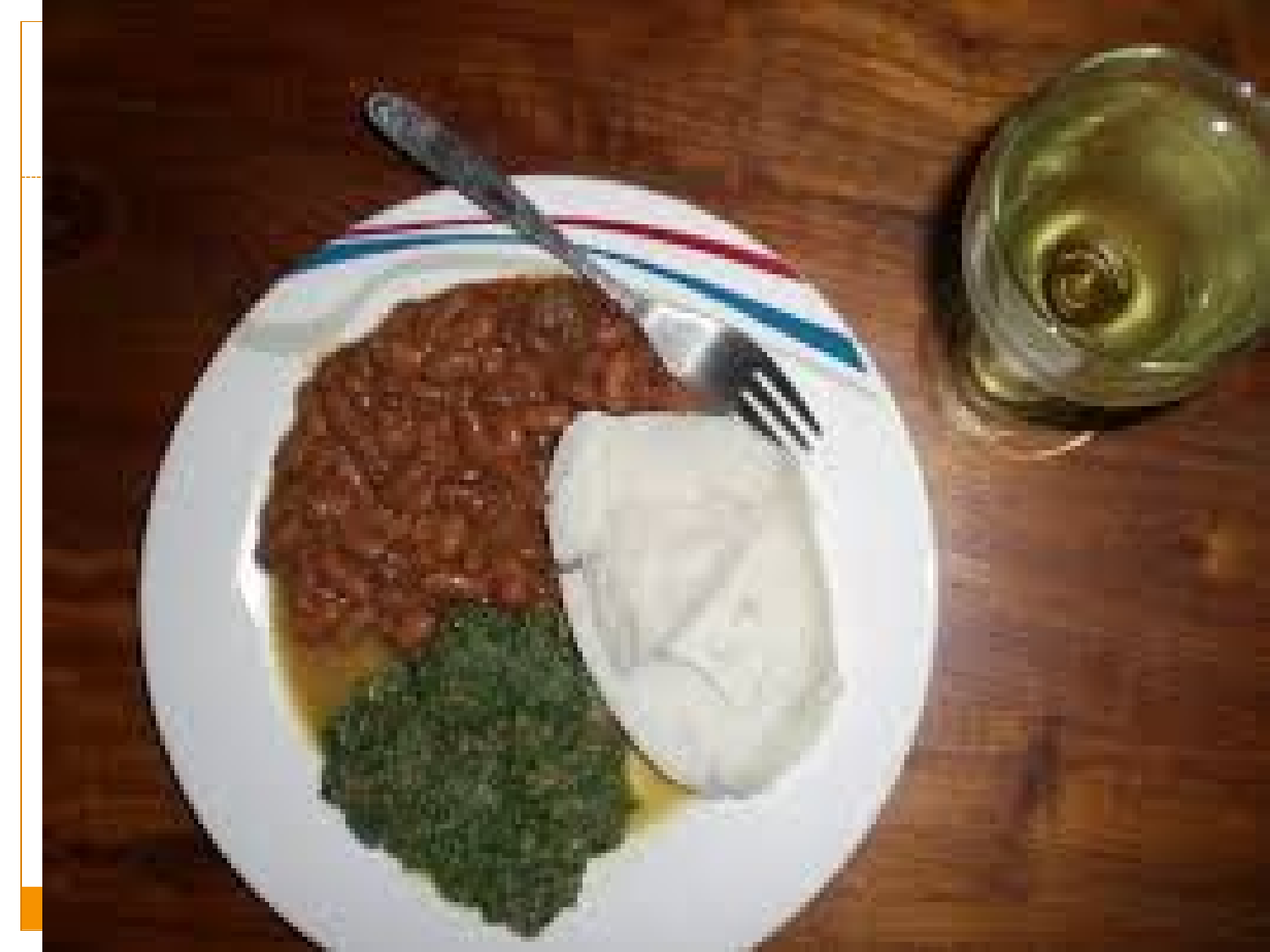

# Pictures

## Slide 28
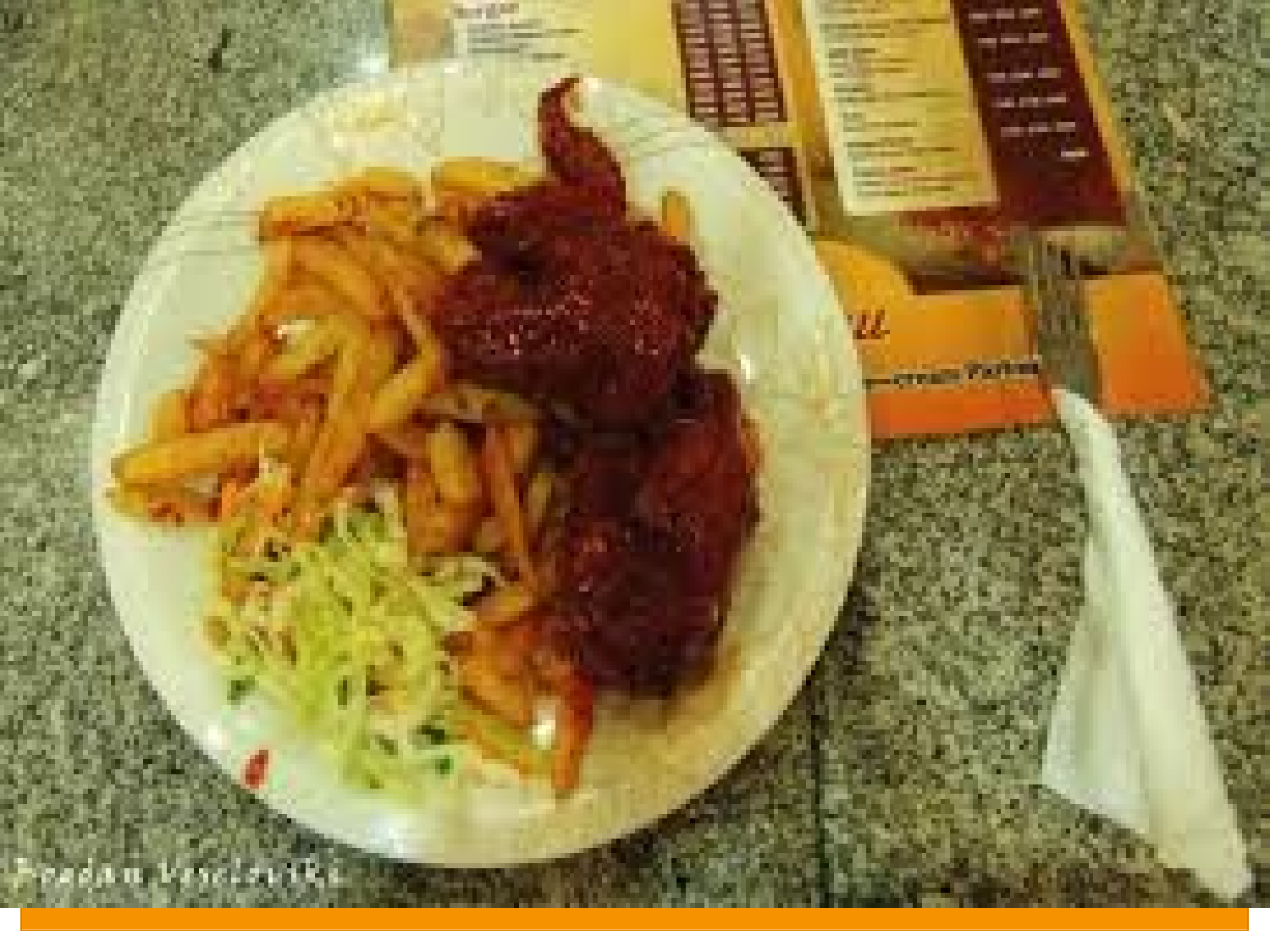

# Pictures

## Slide 29
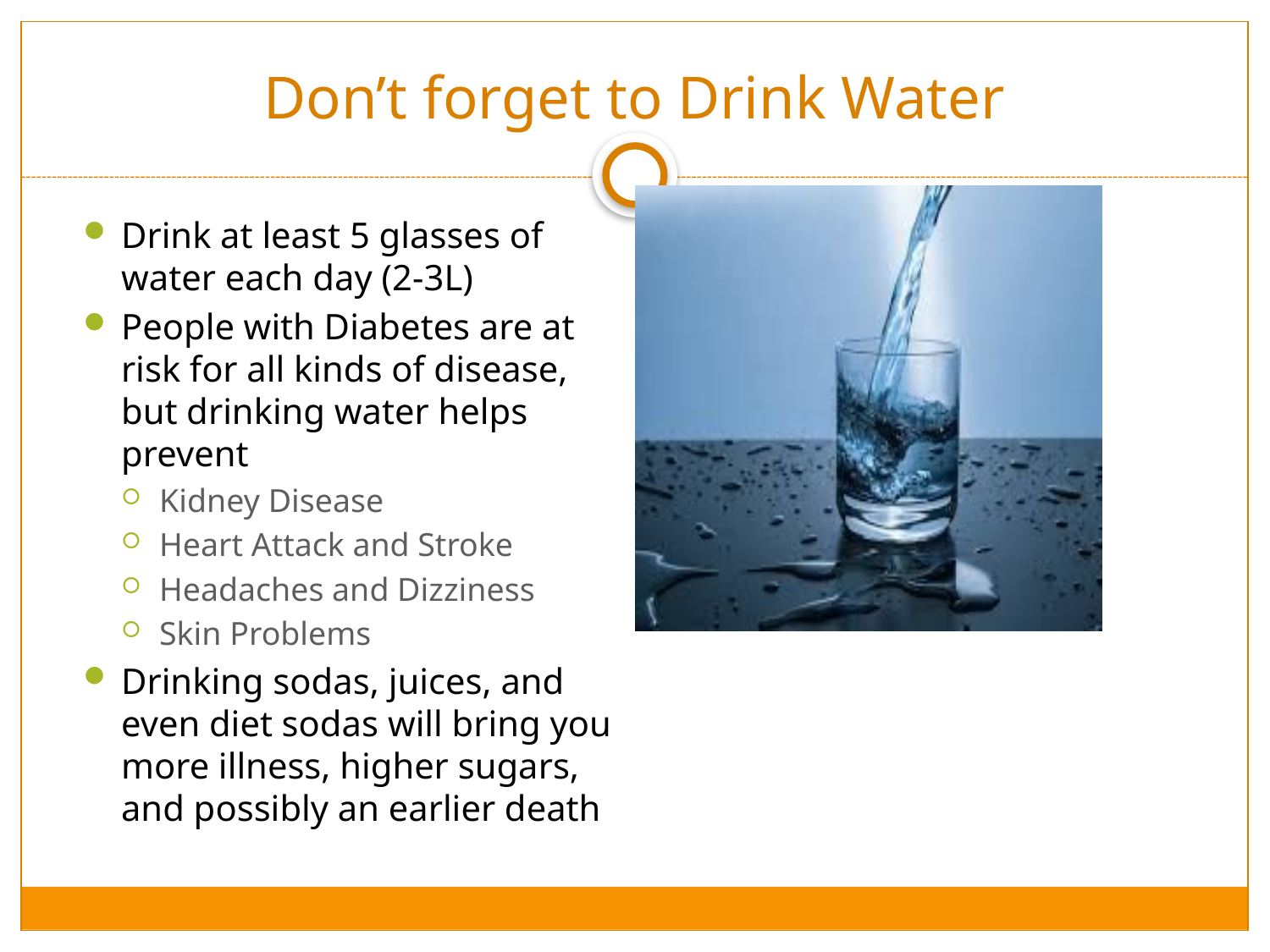

# Don’t forget to Drink Water
Drink at least 5 glasses of water each day (2-3L)
People with Diabetes are at risk for all kinds of disease, but drinking water helps prevent
Kidney Disease
Heart Attack and Stroke
Headaches and Dizziness
Skin Problems
Drinking sodas, juices, and even diet sodas will bring you more illness, higher sugars, and possibly an earlier death

## Slide 30
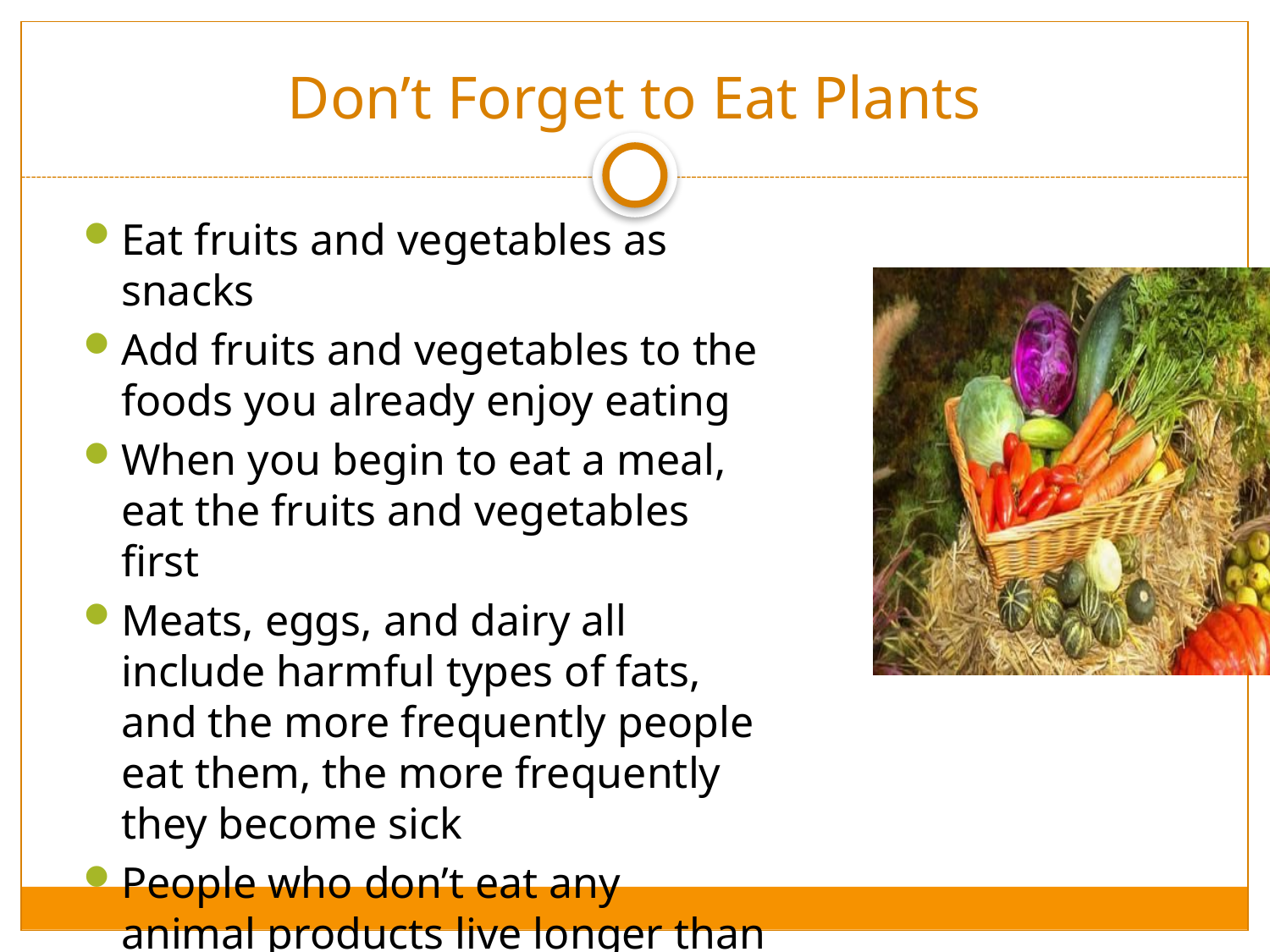

# Don’t Forget to Eat Plants
Eat fruits and vegetables as snacks
Add fruits and vegetables to the foods you already enjoy eating
When you begin to eat a meal, eat the fruits and vegetables first
Meats, eggs, and dairy all include harmful types of fats, and the more frequently people eat them, the more frequently they become sick
People who don’t eat any animal products live longer than those who do

## Slide 31
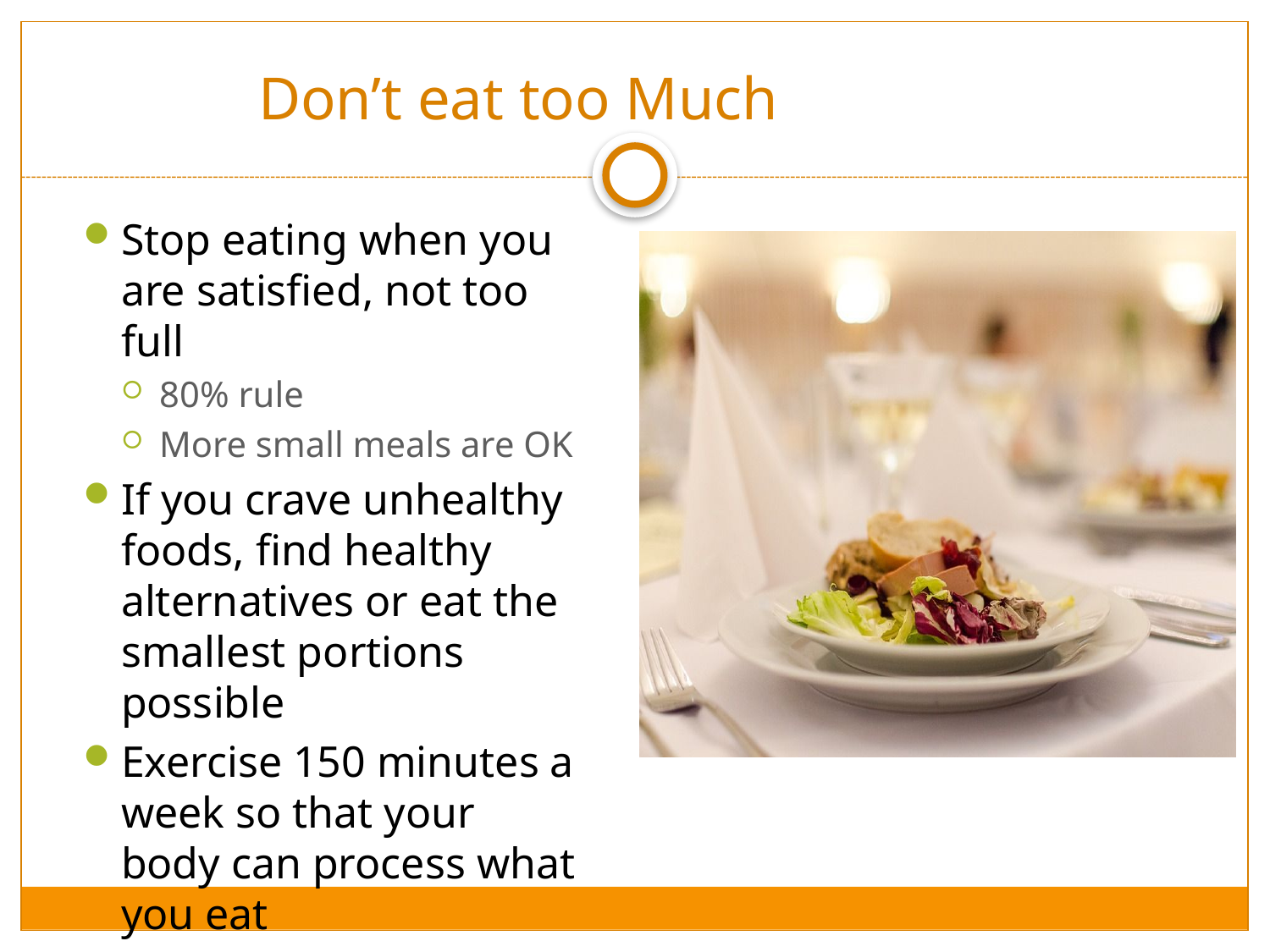

# Don’t eat too Much
Stop eating when you are satisfied, not too full
80% rule
More small meals are OK
If you crave unhealthy foods, find healthy alternatives or eat the smallest portions possible
Exercise 150 minutes a week so that your body can process what you eat

## Slide 32
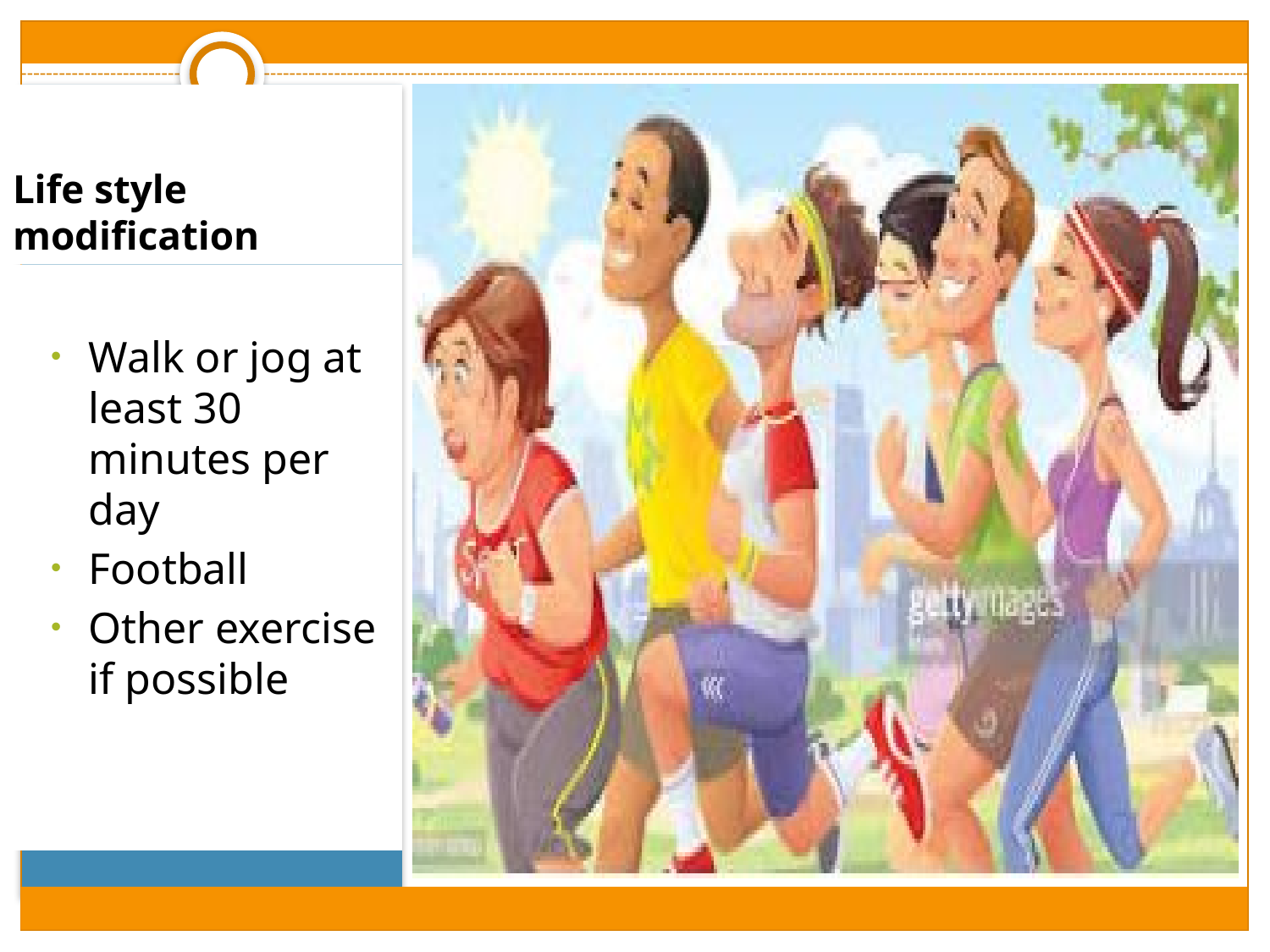

# Life style modification
Exercise
Walk or jog at least 30 minutes per day
Football
Other exercise if possible

## Slide 33
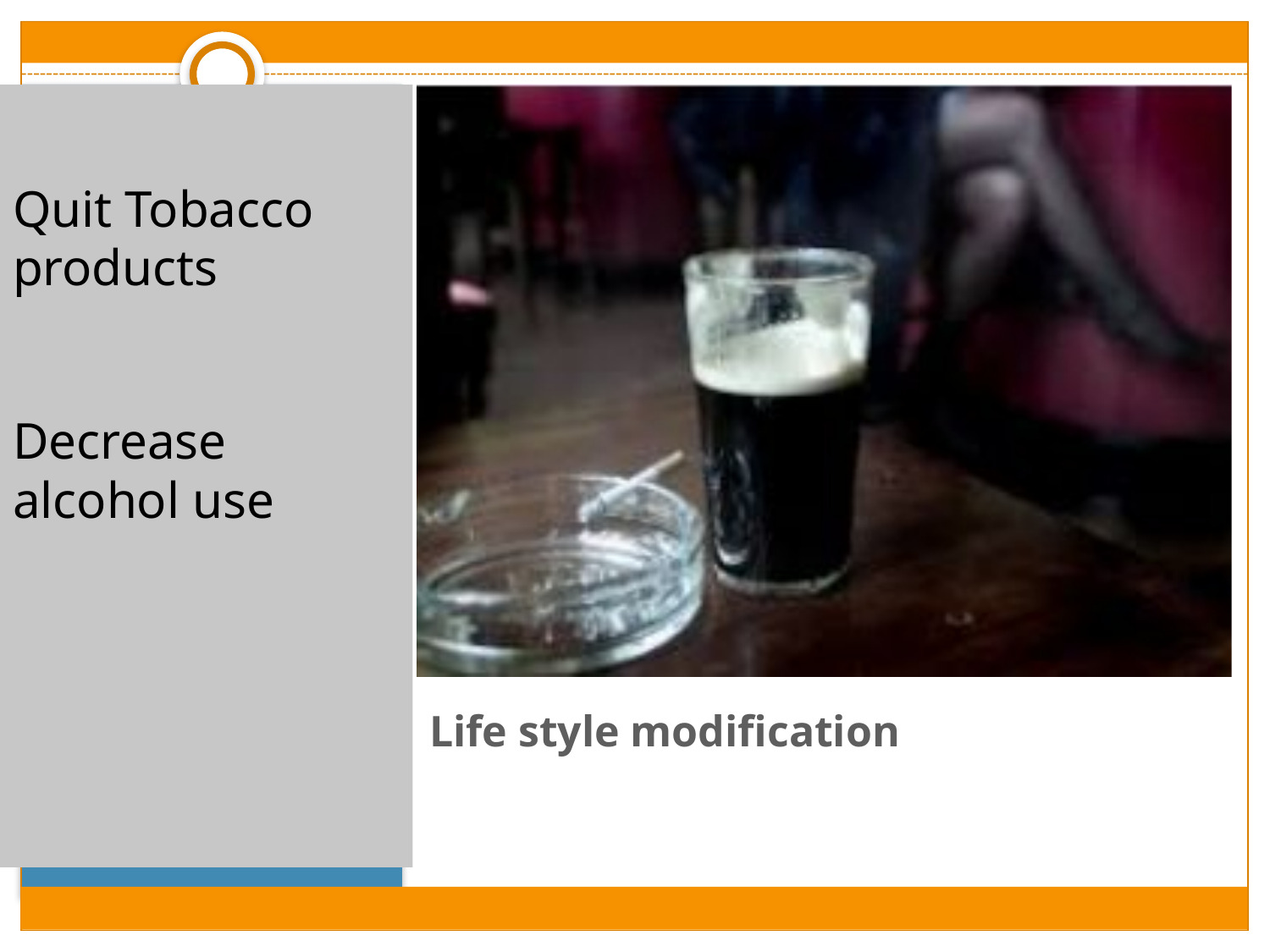

Quit Tobacco products
Decrease alcohol use
# Life style modification

## Slide 34
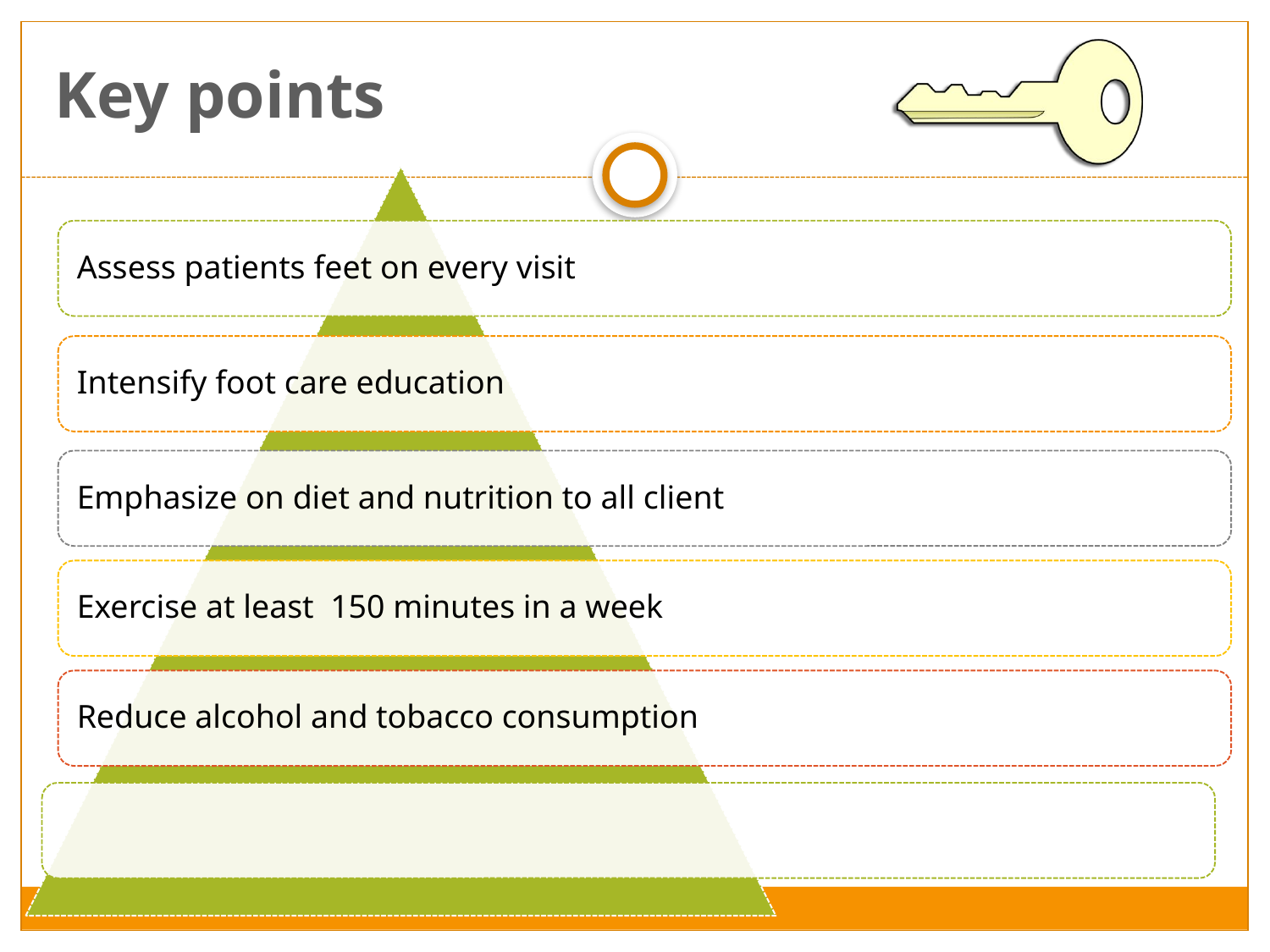

# Key points
