# Supplementary material for: Training Mid-Level Providers to Treat Severe Non-Communicable Diseases in Neno, Malawi through PEN-Plus Strategies
Source: Ann Glob Health. 2022 Aug 11;88(1):69. doi: 10.5334/aogh.3750 (PMC9389951; doi:10.5334/aogh.3750)
Supplement: Didactic Materials. — The supplementary materials contain a suggested didactic training schedule and the PowerPoint presentations used for PEN-Plus training in Neno, Malawi. These materials have been reviewed and accepted by the Malawi Ministry of Health for future PEN-Plus trainings in Malawi. [file agh-88-1-3750-s2.zip › Didactic_Materials/DM_Diagnosis.pptx]

## Slide 1
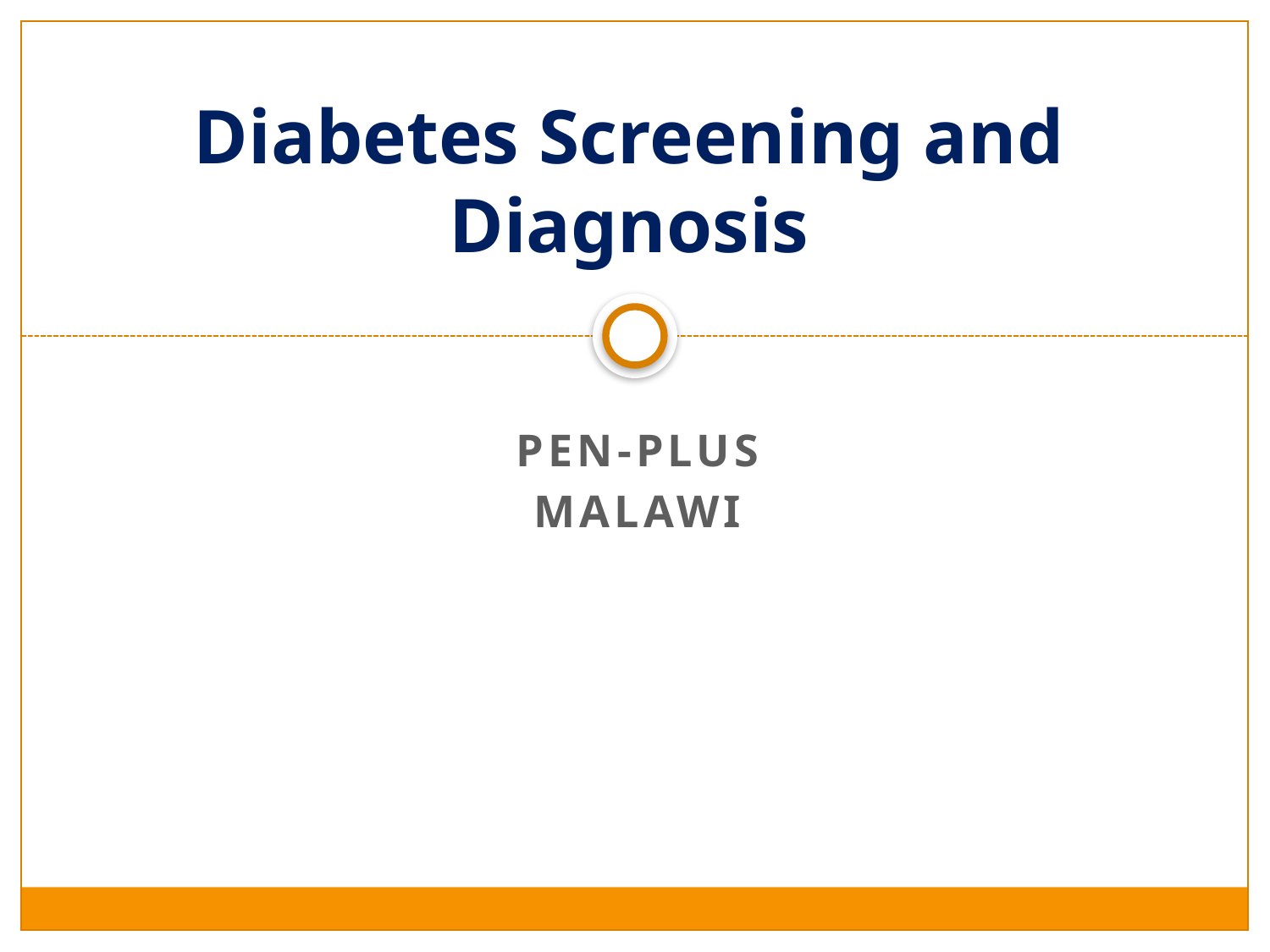

# Diabetes Screening and Diagnosis
Pen-Plus
Malawi

## Slide 2
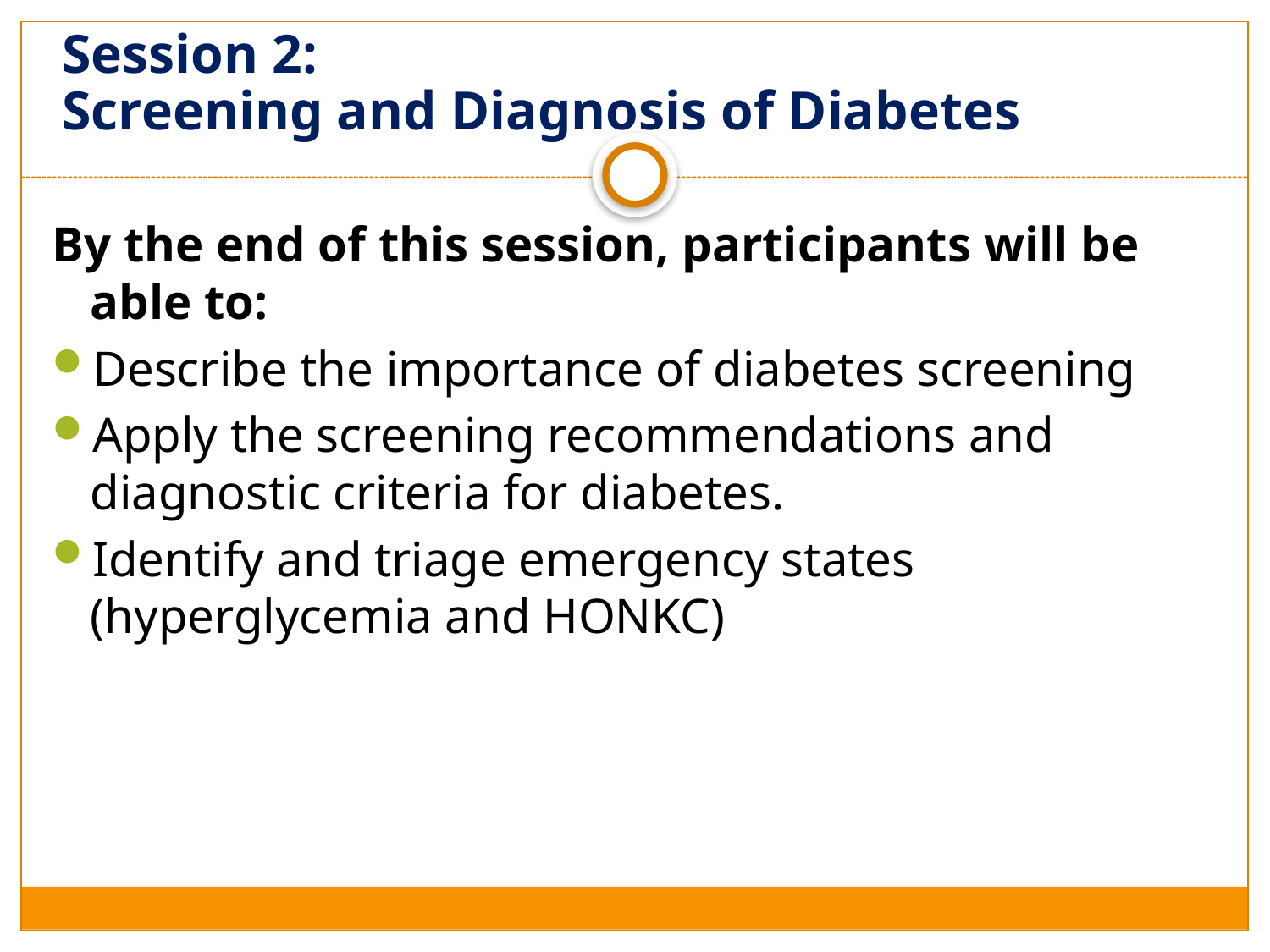

# Session 2: Screening and Diagnosis of Diabetes
By the end of this session, participants will be able to:
Describe the importance of diabetes screening
Apply the screening recommendations and diagnostic criteria for diabetes.
Identify and triage emergency states (hyperglycemia and HONKC)

## Slide 3
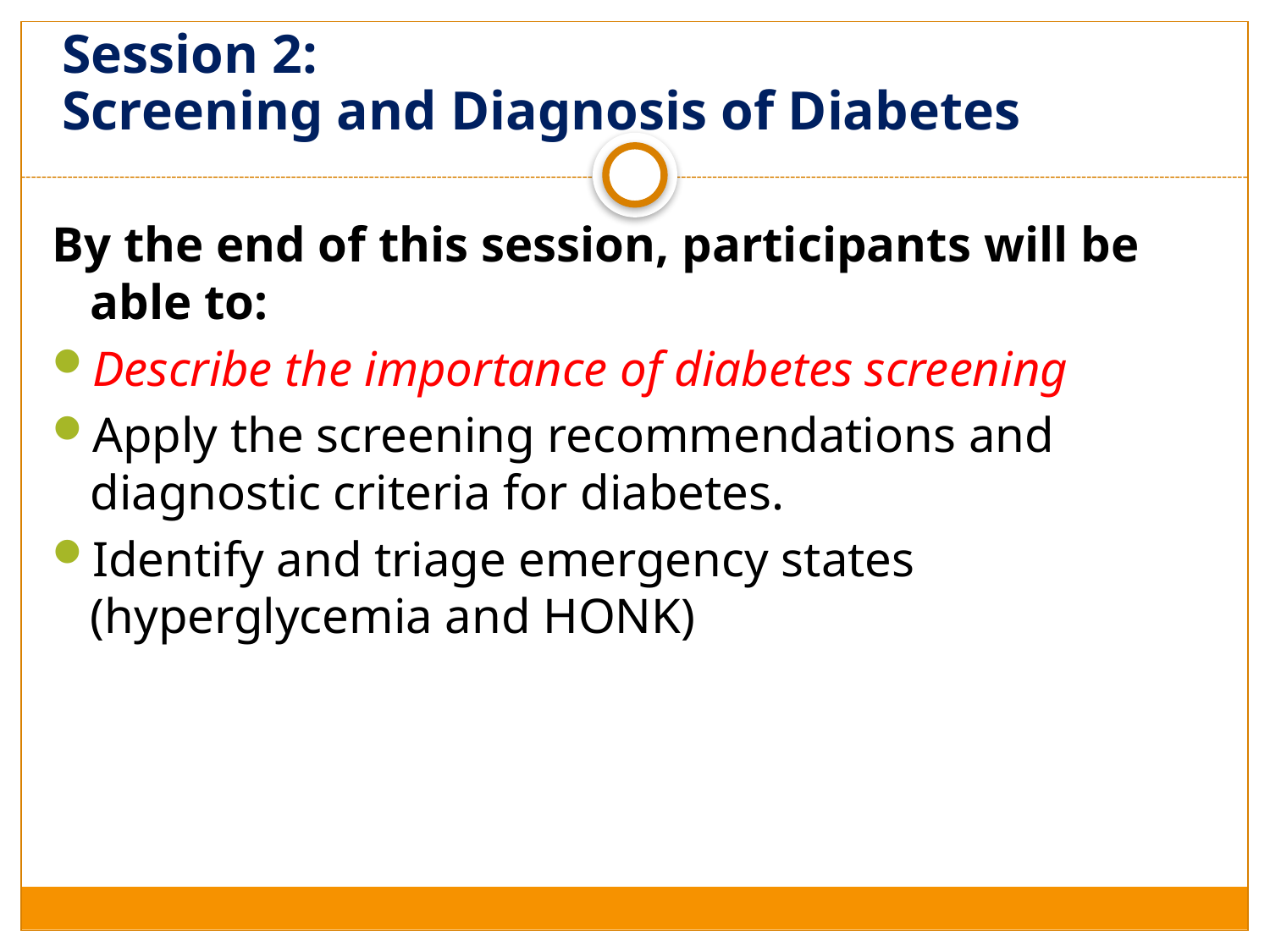

# Session 2: Screening and Diagnosis of Diabetes
By the end of this session, participants will be able to:
Describe the importance of diabetes screening
Apply the screening recommendations and diagnostic criteria for diabetes.
Identify and triage emergency states (hyperglycemia and HONK)

## Slide 4
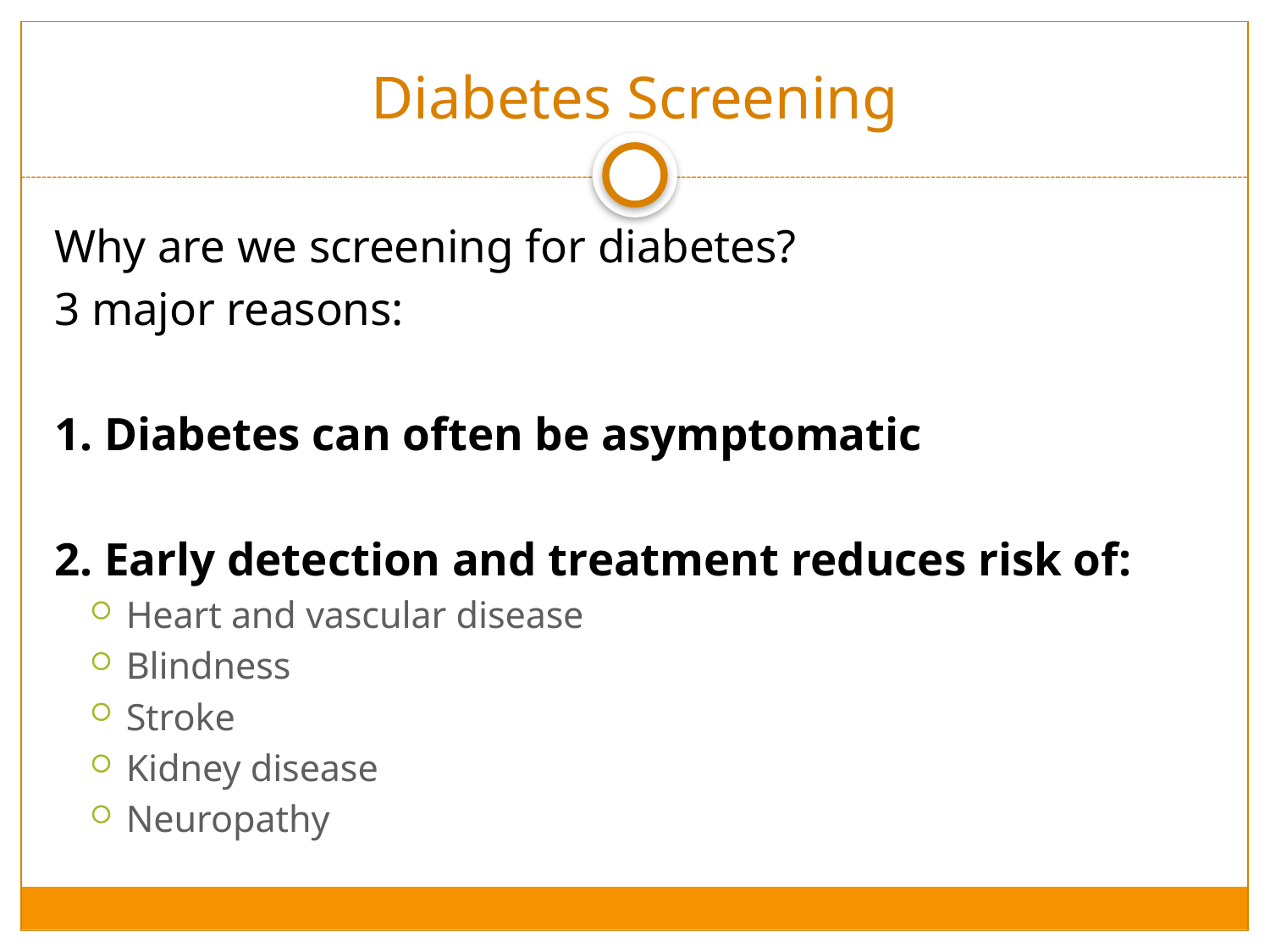

# Diabetes Screening
Why are we screening for diabetes?
3 major reasons:
1. Diabetes can often be asymptomatic
2. Early detection and treatment reduces risk of:
Heart and vascular disease
Blindness
Stroke
Kidney disease
Neuropathy

## Slide 5
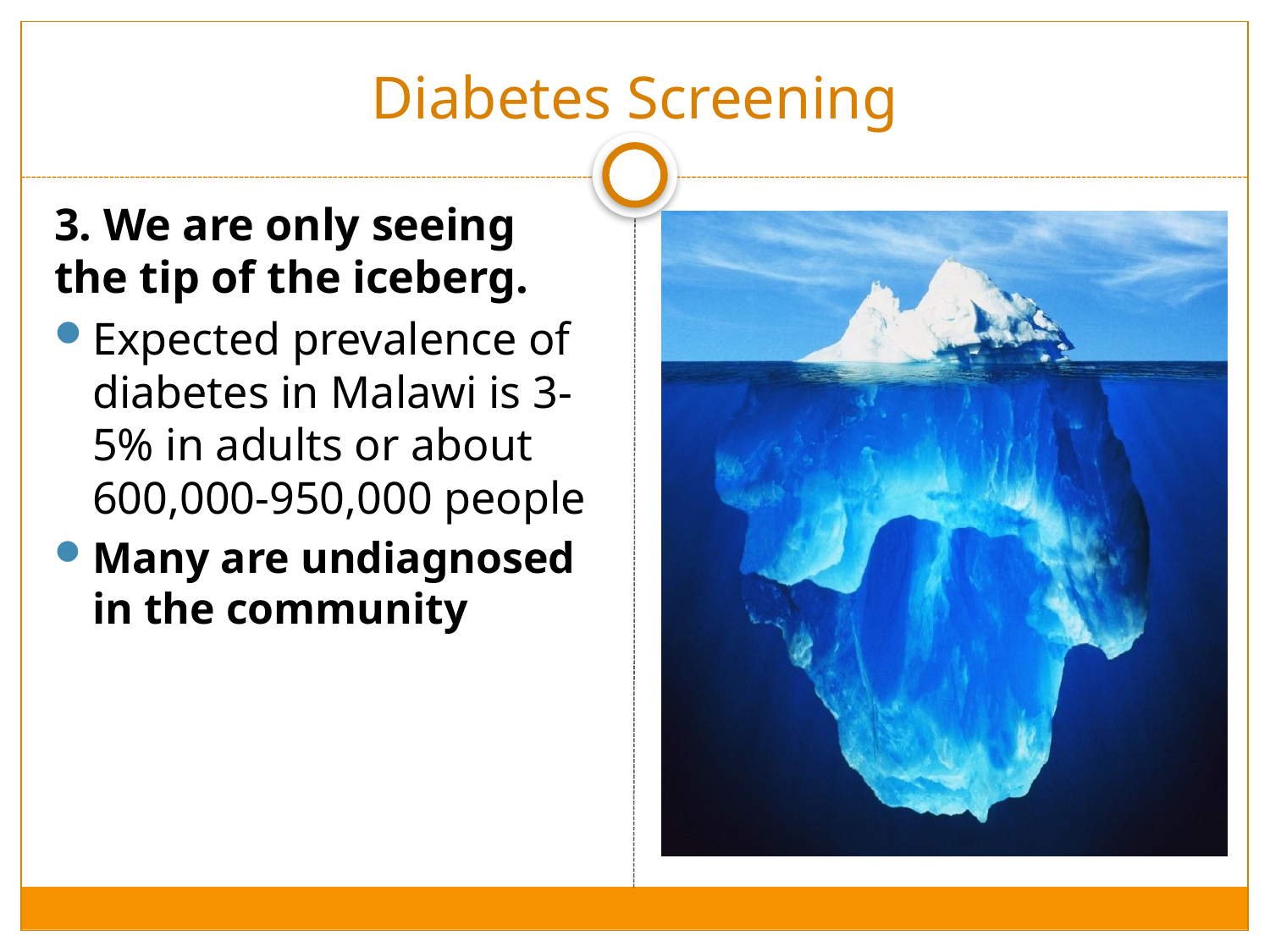

# Diabetes Screening
3. We are only seeing the tip of the iceberg.
Expected prevalence of diabetes in Malawi is 3-5% in adults or about 600,000-950,000 people
Many are undiagnosed in the community

## Slide 6
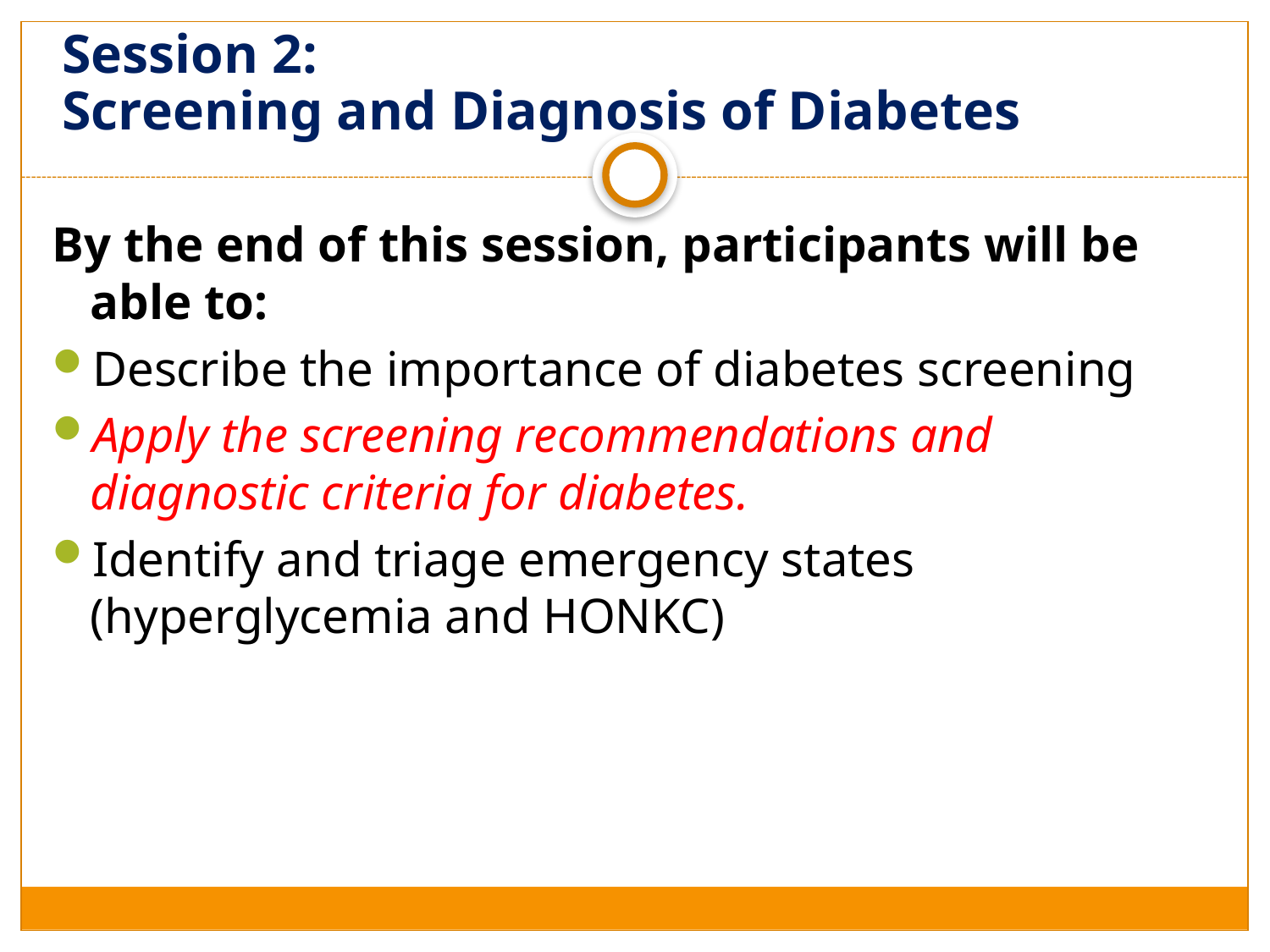

# Session 2: Screening and Diagnosis of Diabetes
By the end of this session, participants will be able to:
Describe the importance of diabetes screening
Apply the screening recommendations and diagnostic criteria for diabetes.
Identify and triage emergency states (hyperglycemia and HONKC)

## Slide 7
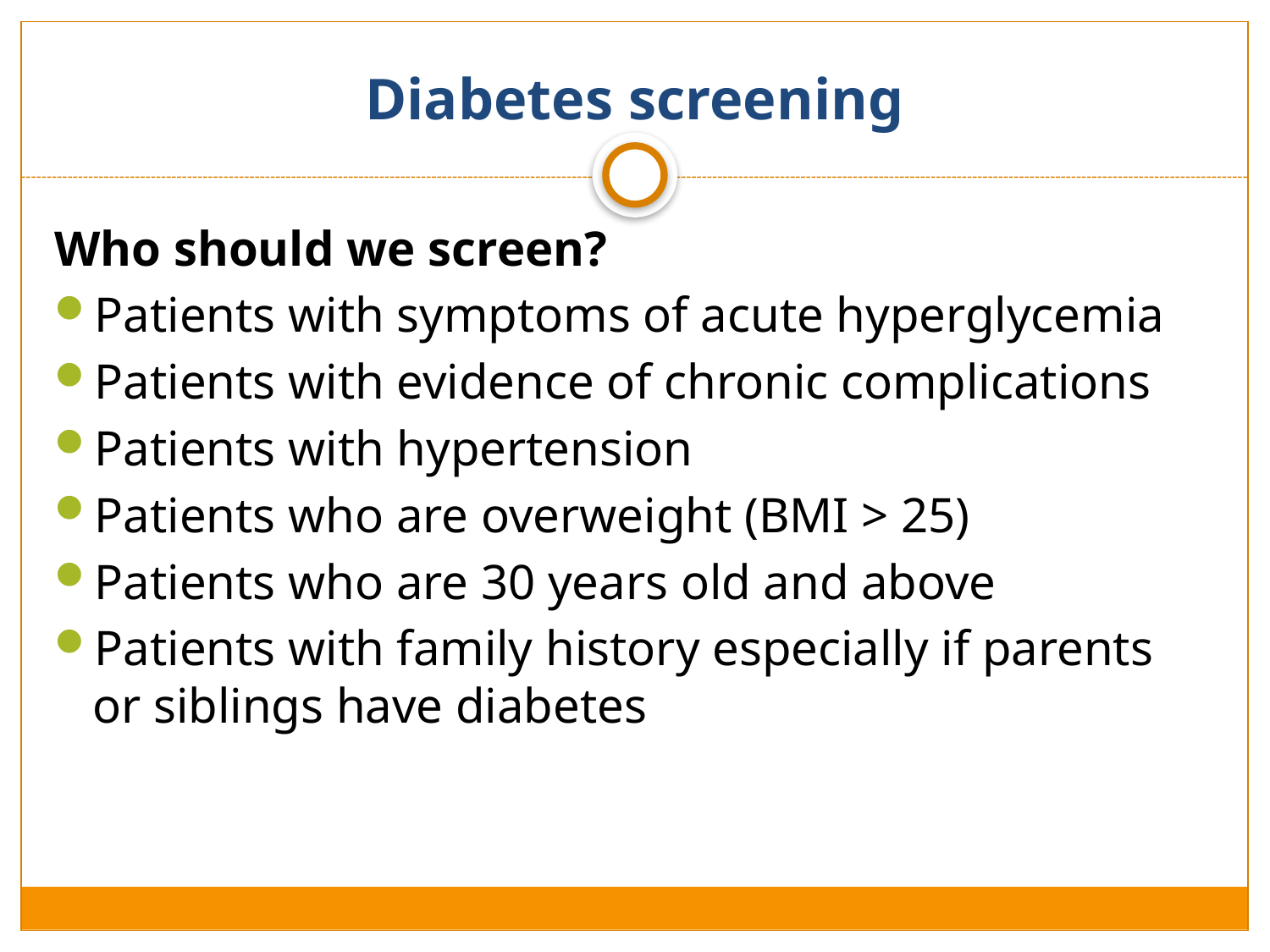

# Diabetes screening
Who should we screen?
Patients with symptoms of acute hyperglycemia
Patients with evidence of chronic complications
Patients with hypertension
Patients who are overweight (BMI > 25)
Patients who are 30 years old and above
Patients with family history especially if parents or siblings have diabetes

## Slide 8
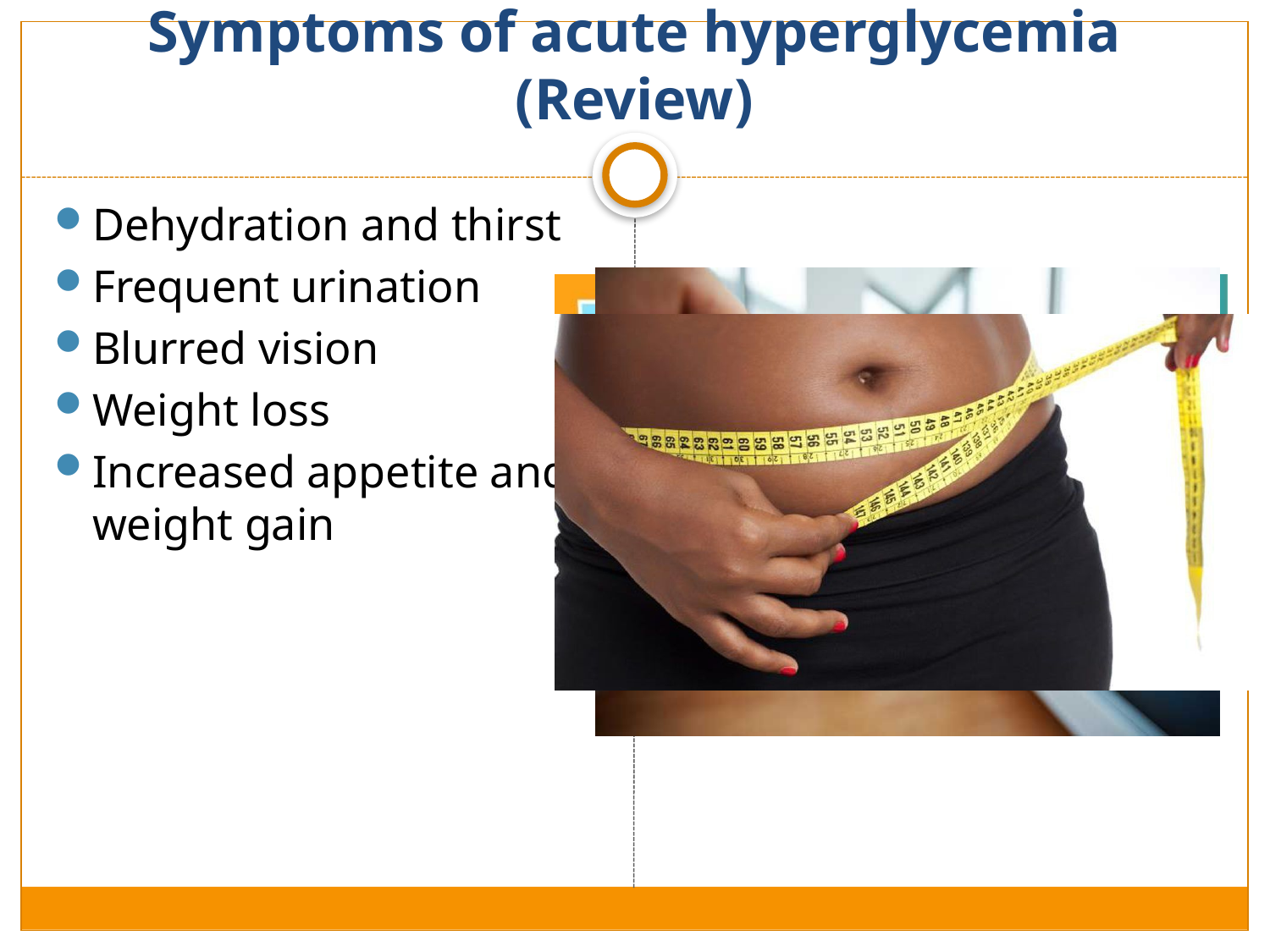

# Symptoms of acute hyperglycemia (Review)
Dehydration and thirst
Frequent urination
Blurred vision
Weight loss
Increased appetite and weight gain

## Slide 9
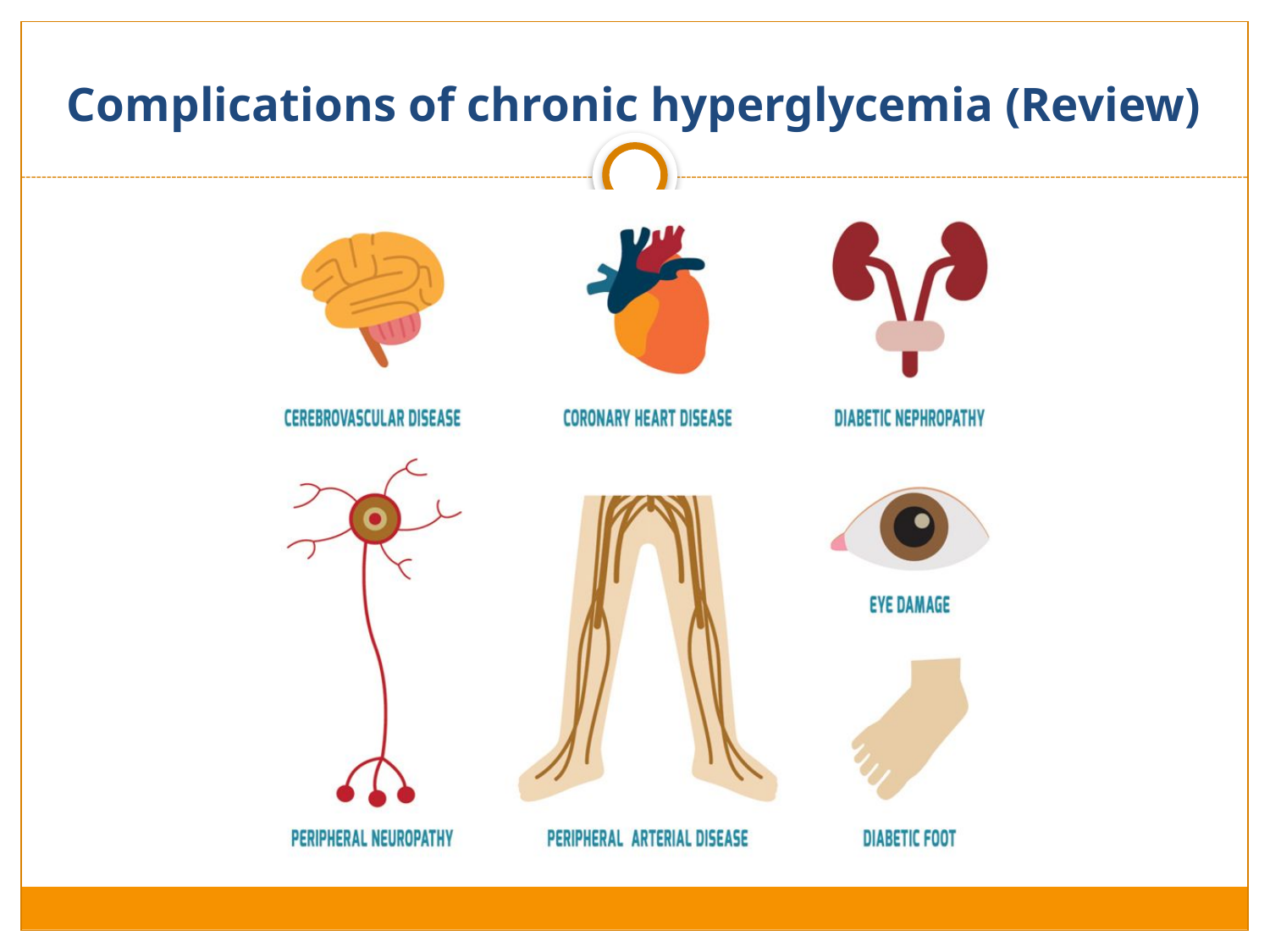

# Complications of chronic hyperglycemia (Review)

## Slide 10
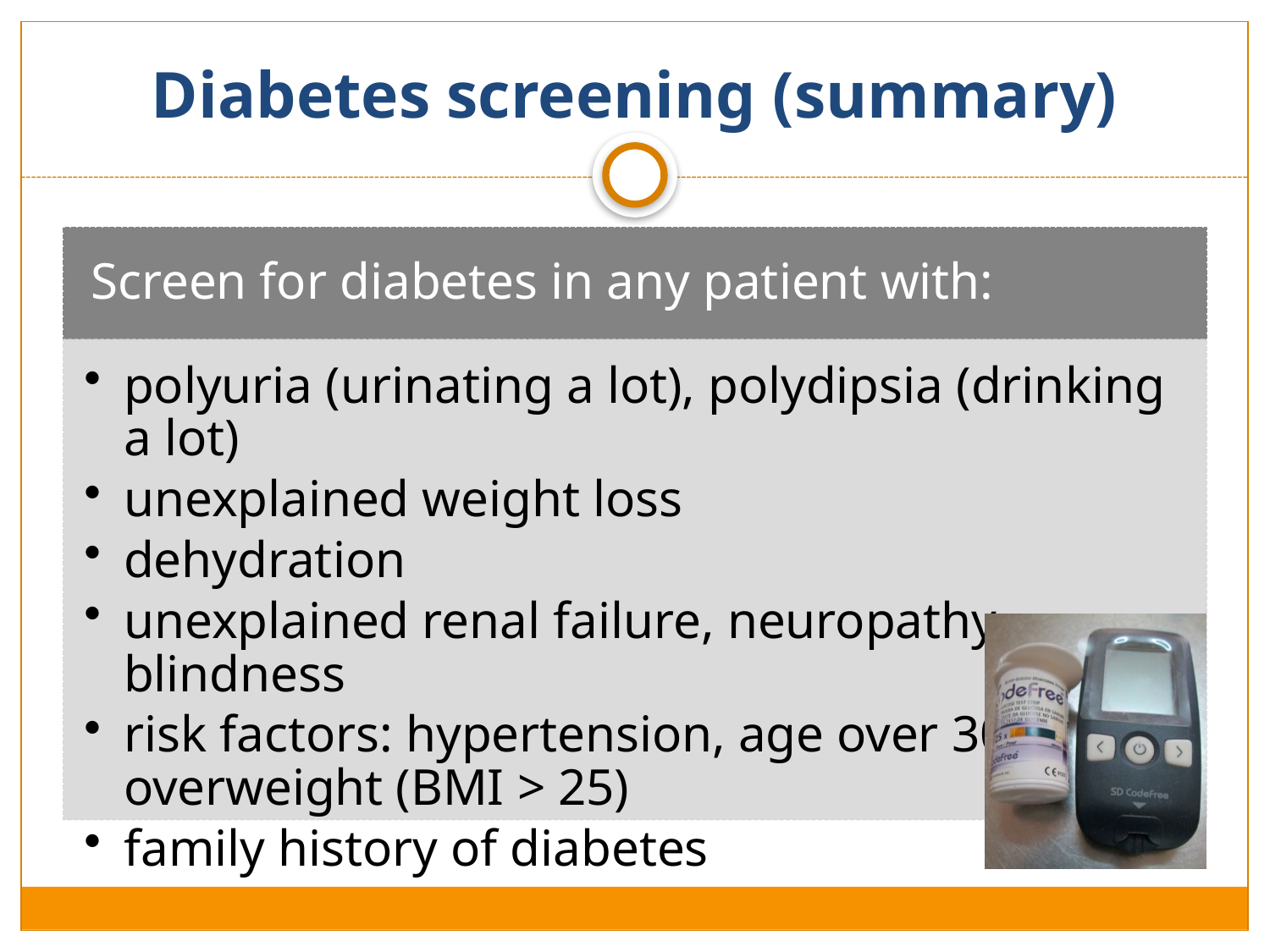

# Diabetes screening (summary)

## Slide 11
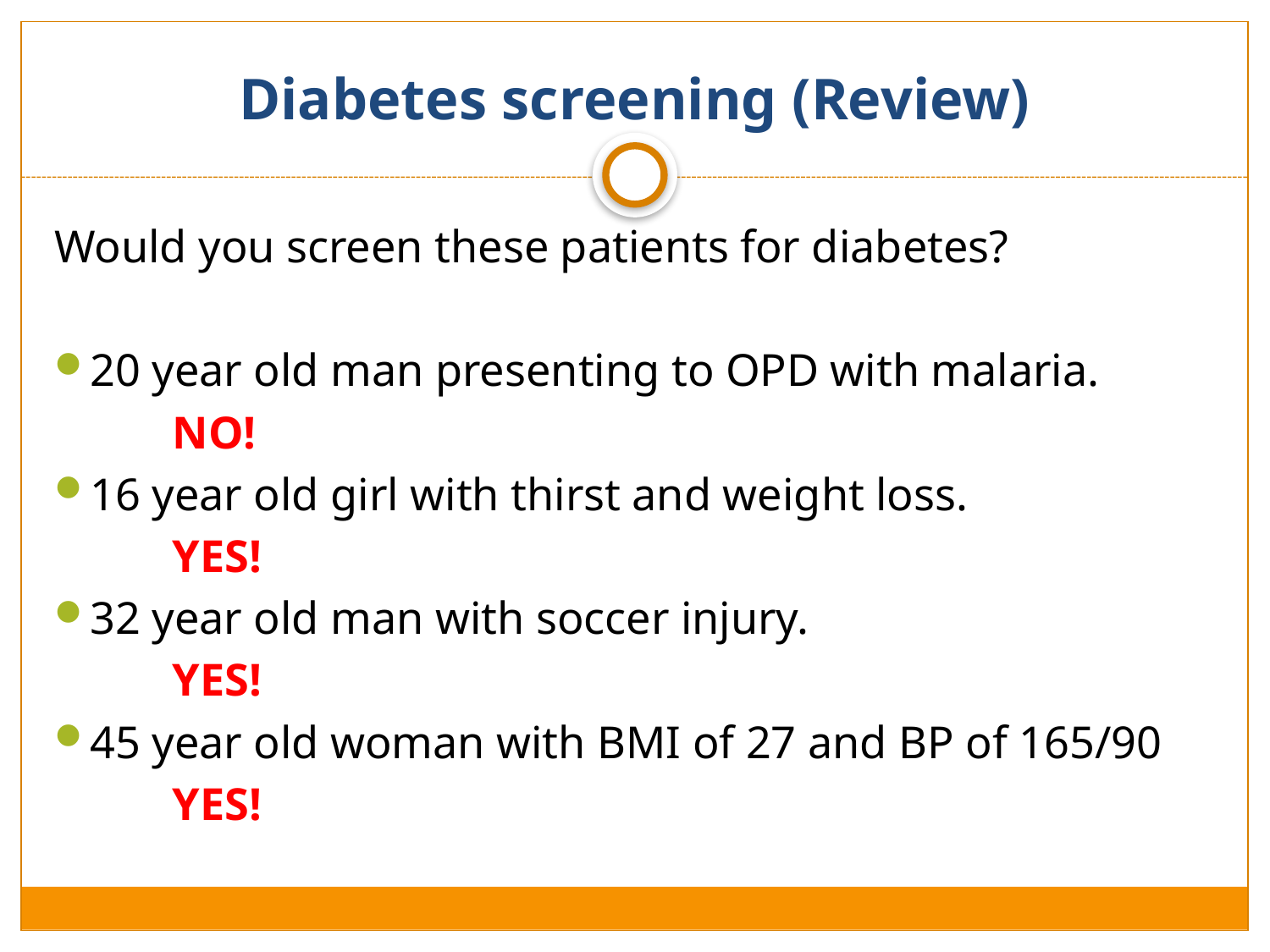

# Diabetes screening (Review)
Would you screen these patients for diabetes?
20 year old man presenting to OPD with malaria.
	NO!
16 year old girl with thirst and weight loss.
	YES!
32 year old man with soccer injury.
	YES!
45 year old woman with BMI of 27 and BP of 165/90
	YES!

## Slide 12
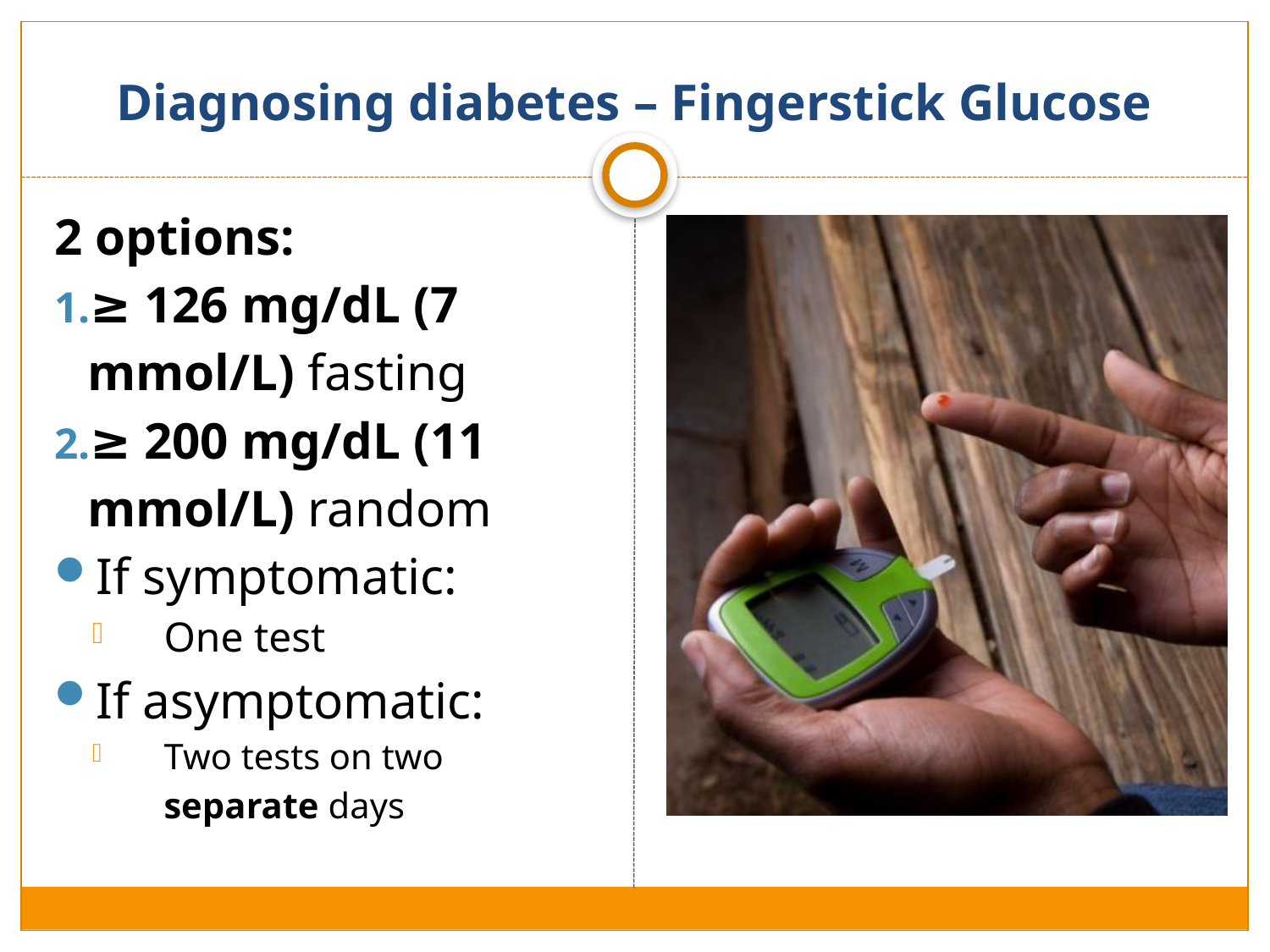

# Diagnosing diabetes – Fingerstick Glucose
2 options:
≥ 126 mg/dL (7 mmol/L) fasting
≥ 200 mg/dL (11 mmol/L) random
If symptomatic:
One test
If asymptomatic:
Two tests on two separate days

## Slide 13
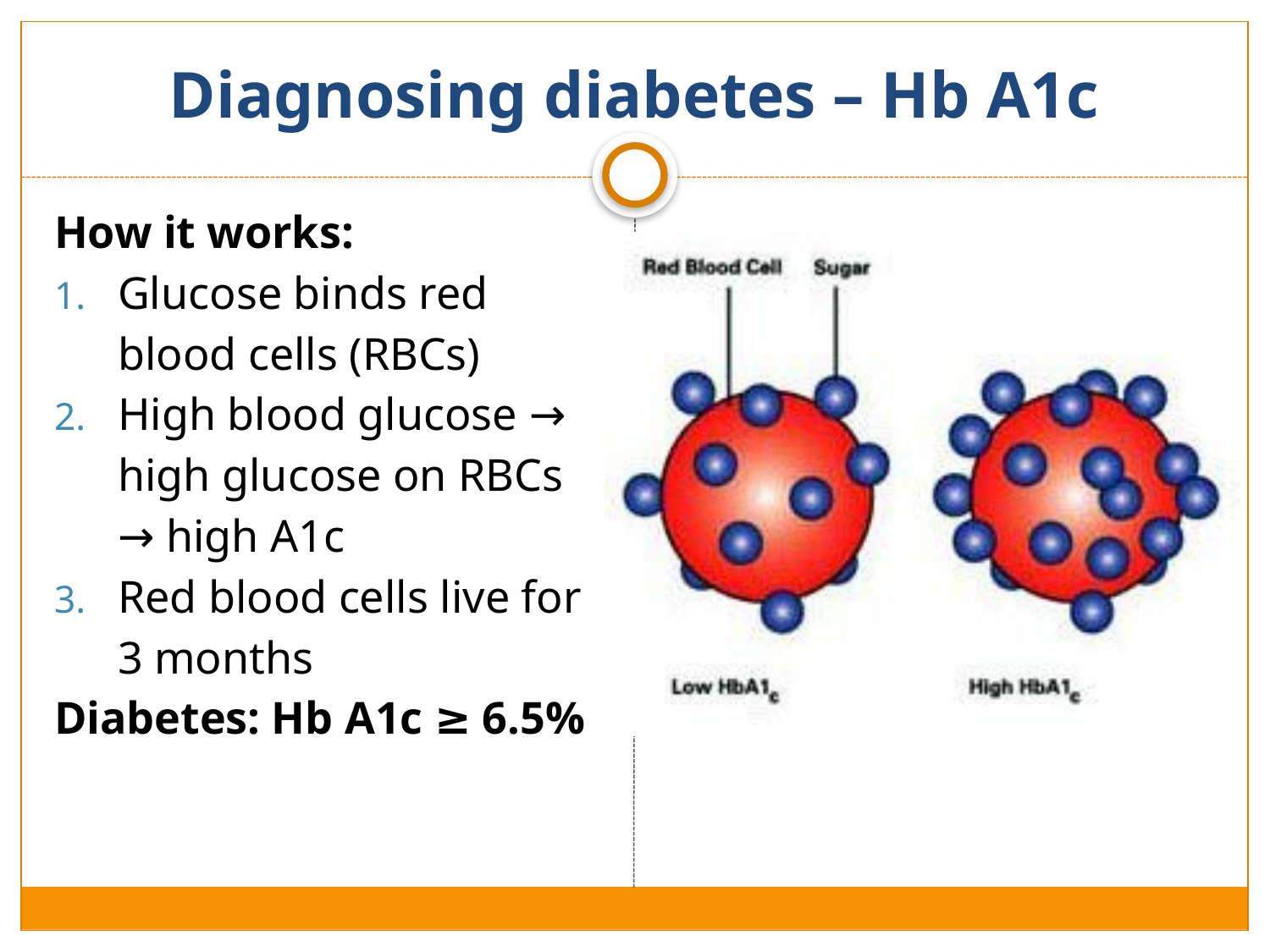

# Diagnosing diabetes – Hb A1c
How it works:
Glucose binds red blood cells (RBCs)
High blood glucose → high glucose on RBCs → high A1c
Red blood cells live for 3 months
Diabetes: Hb A1c ≥ 6.5%

## Slide 14
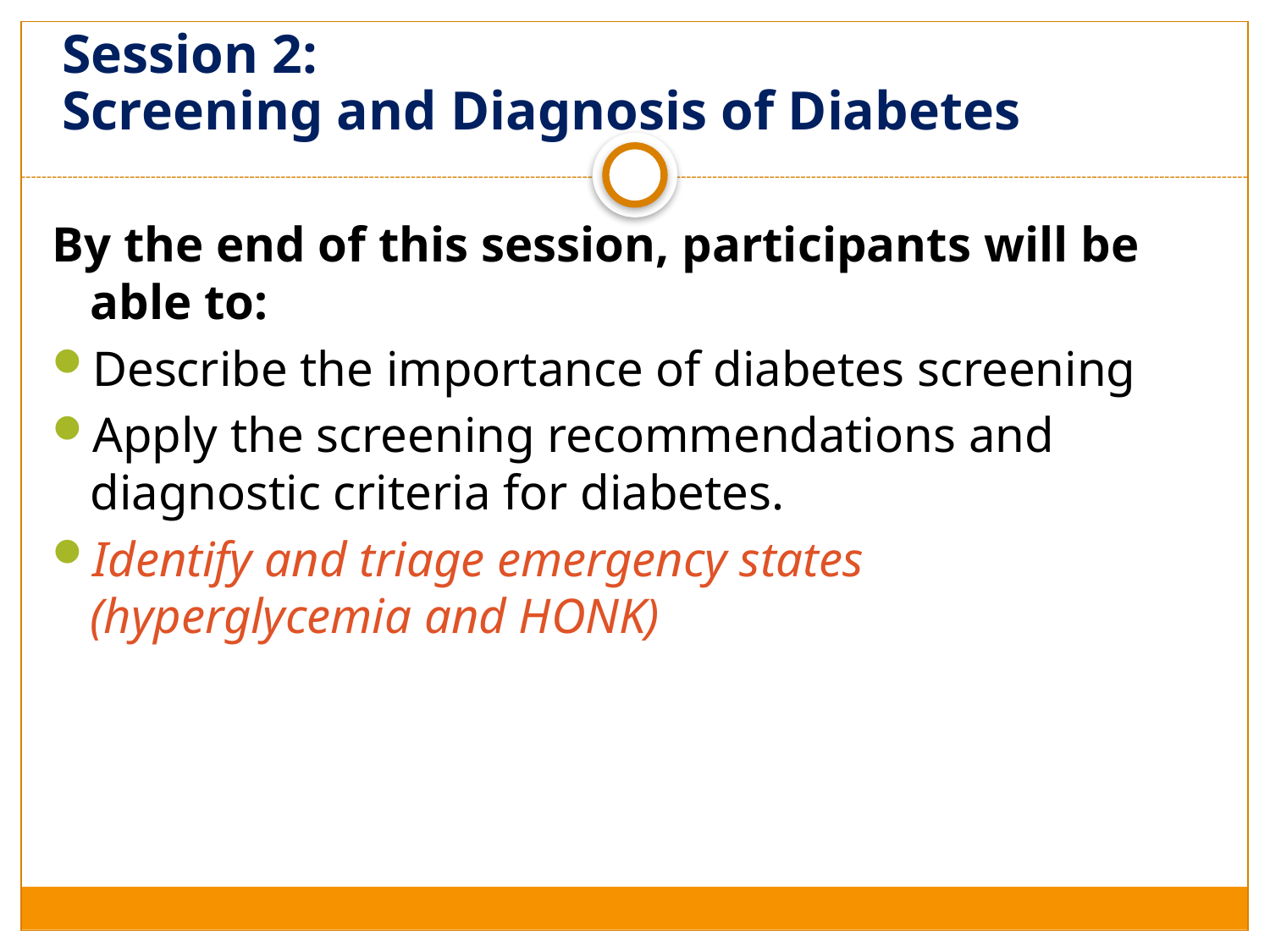

# Session 2: Screening and Diagnosis of Diabetes
By the end of this session, participants will be able to:
Describe the importance of diabetes screening
Apply the screening recommendations and diagnostic criteria for diabetes.
Identify and triage emergency states (hyperglycemia and HONK)

## Slide 15
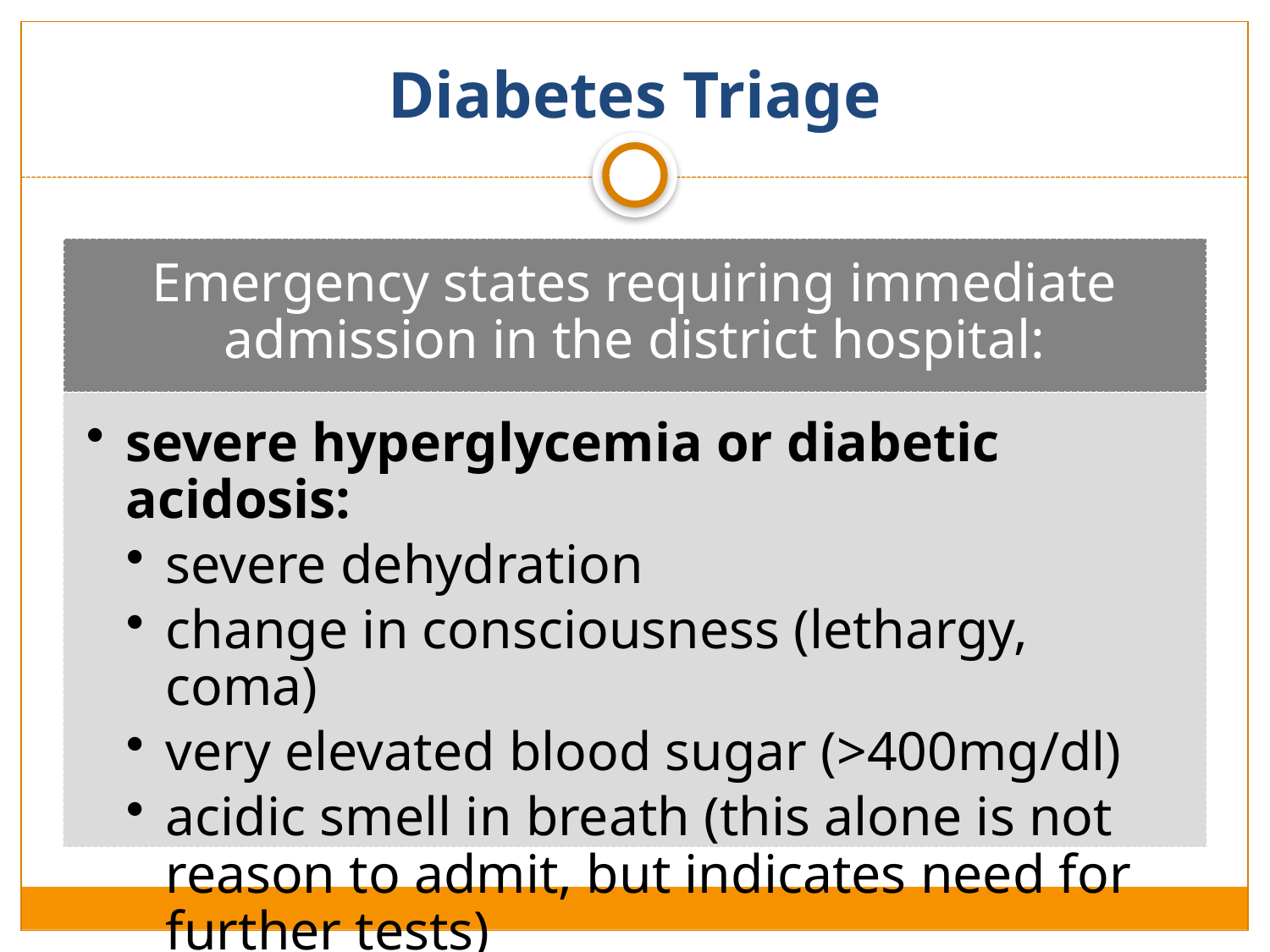

# Diabetes Triage

## Slide 16
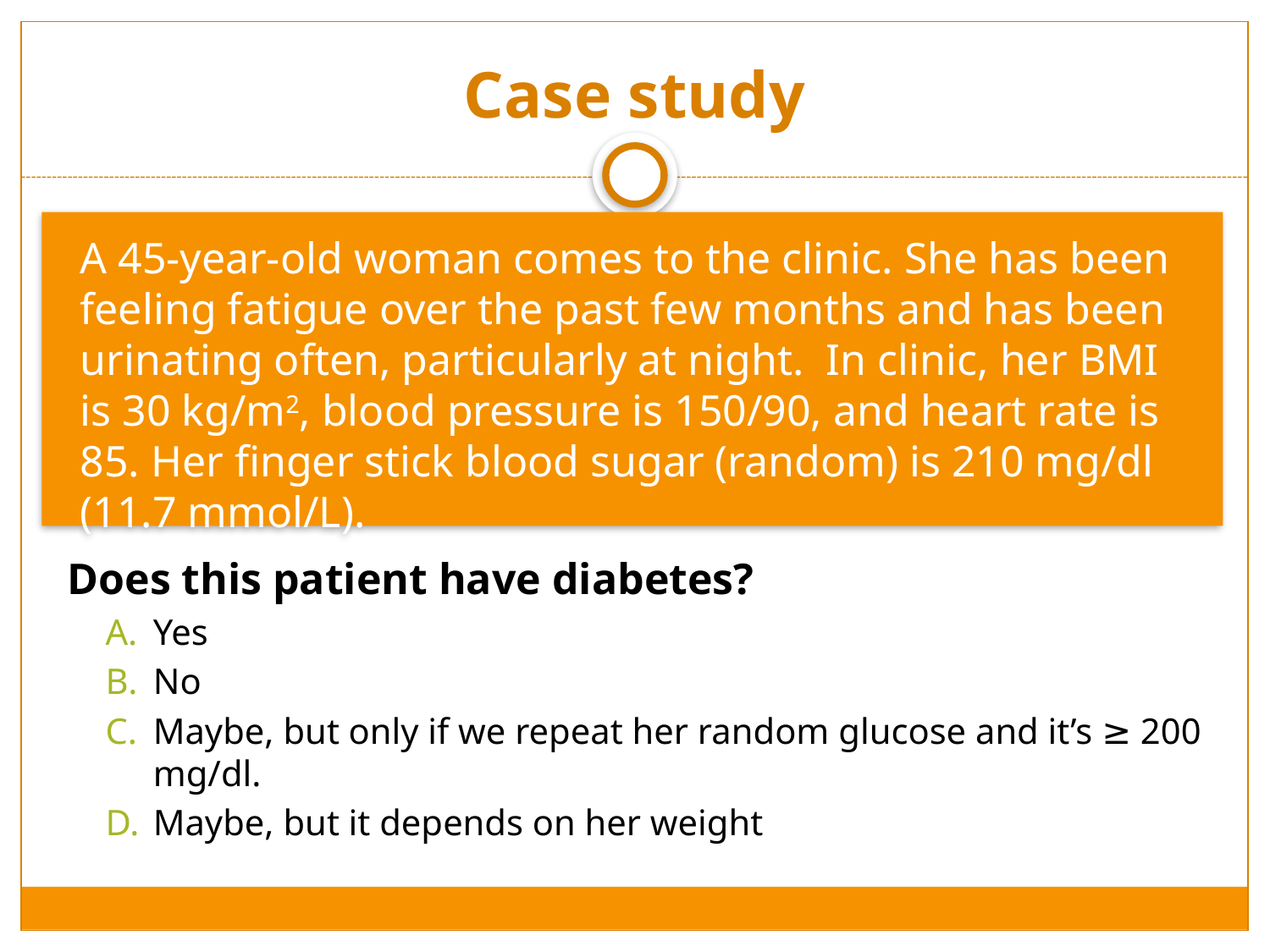

# Case study
A 45-year-old woman comes to the clinic. She has been feeling fatigue over the past few months and has been urinating often, particularly at night. In clinic, her BMI is 30 kg/m2, blood pressure is 150/90, and heart rate is 85. Her finger stick blood sugar (random) is 210 mg/dl (11.7 mmol/L).
Does this patient have diabetes?
Yes
No
Maybe, but only if we repeat her random glucose and it’s ≥ 200 mg/dl.
Maybe, but it depends on her weight

## Slide 17
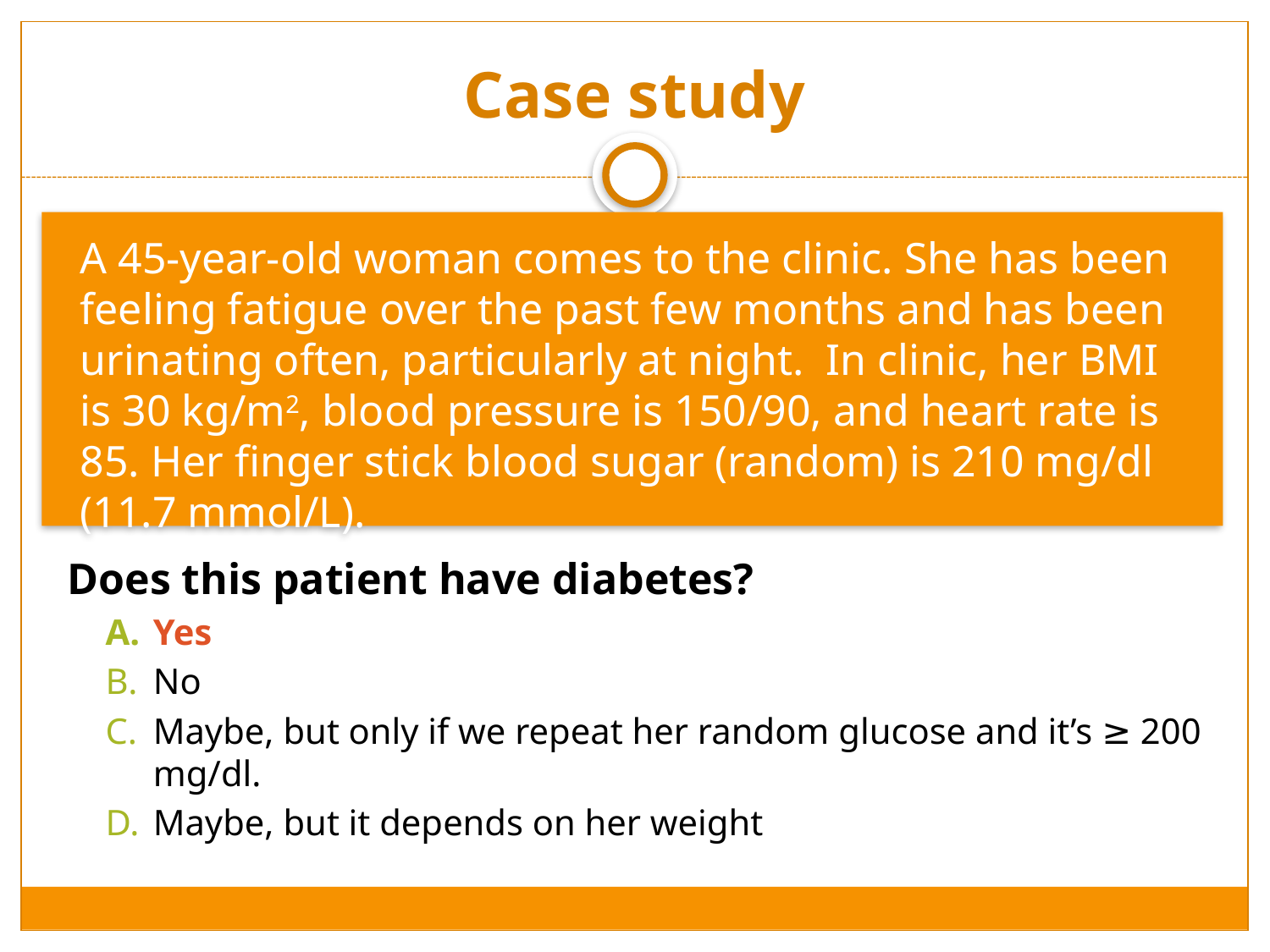

# Case study
A 45-year-old woman comes to the clinic. She has been feeling fatigue over the past few months and has been urinating often, particularly at night. In clinic, her BMI is 30 kg/m2, blood pressure is 150/90, and heart rate is 85. Her finger stick blood sugar (random) is 210 mg/dl (11.7 mmol/L).
Does this patient have diabetes?
Yes
No
Maybe, but only if we repeat her random glucose and it’s ≥ 200 mg/dl.
Maybe, but it depends on her weight

## Slide 18
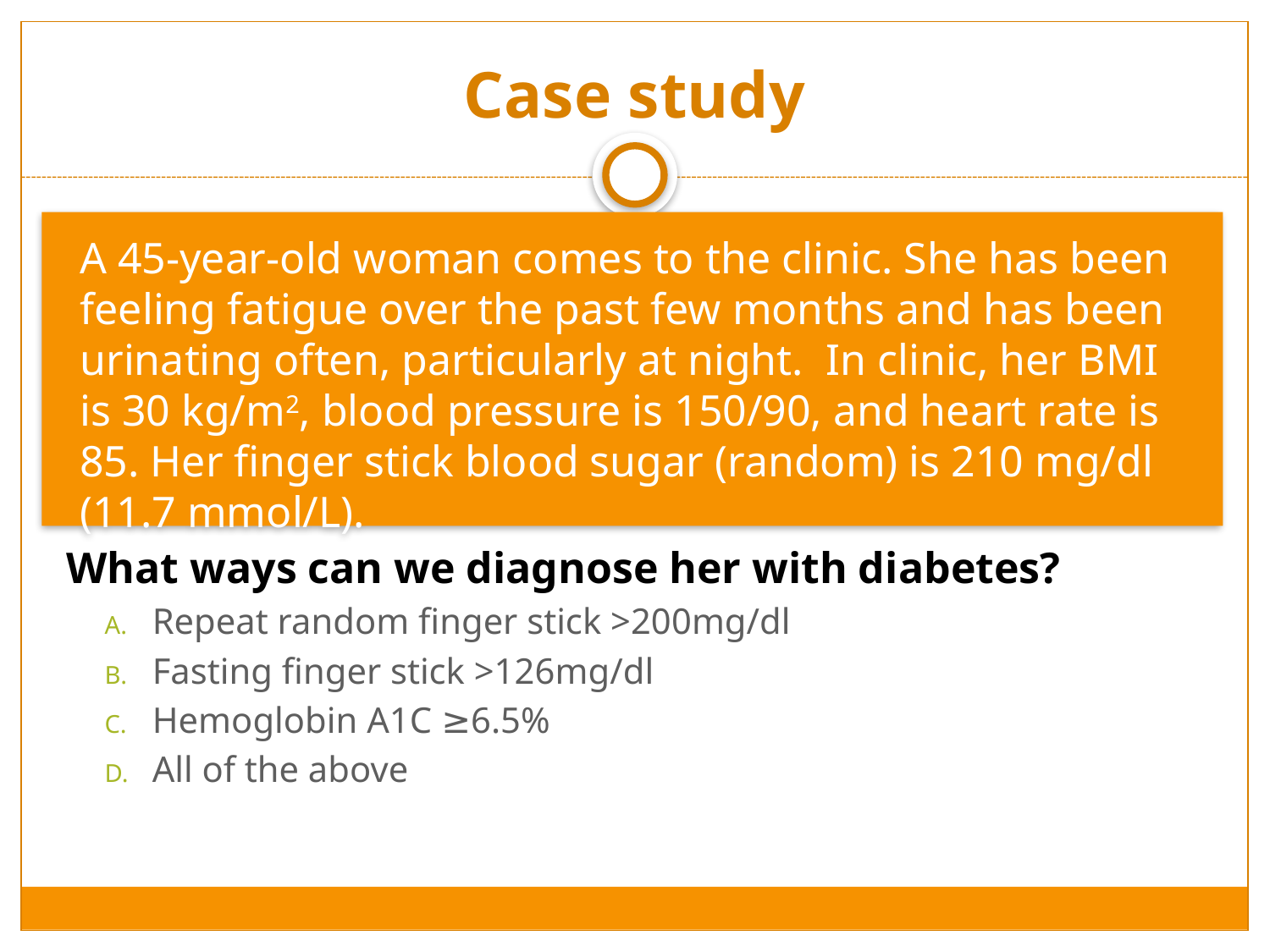

# Case study
A 45-year-old woman comes to the clinic. She has been feeling fatigue over the past few months and has been urinating often, particularly at night. In clinic, her BMI is 30 kg/m2, blood pressure is 150/90, and heart rate is 85. Her finger stick blood sugar (random) is 210 mg/dl (11.7 mmol/L).
What ways can we diagnose her with diabetes?
Repeat random finger stick >200mg/dl
Fasting finger stick >126mg/dl
Hemoglobin A1C ≥6.5%
All of the above

## Slide 19
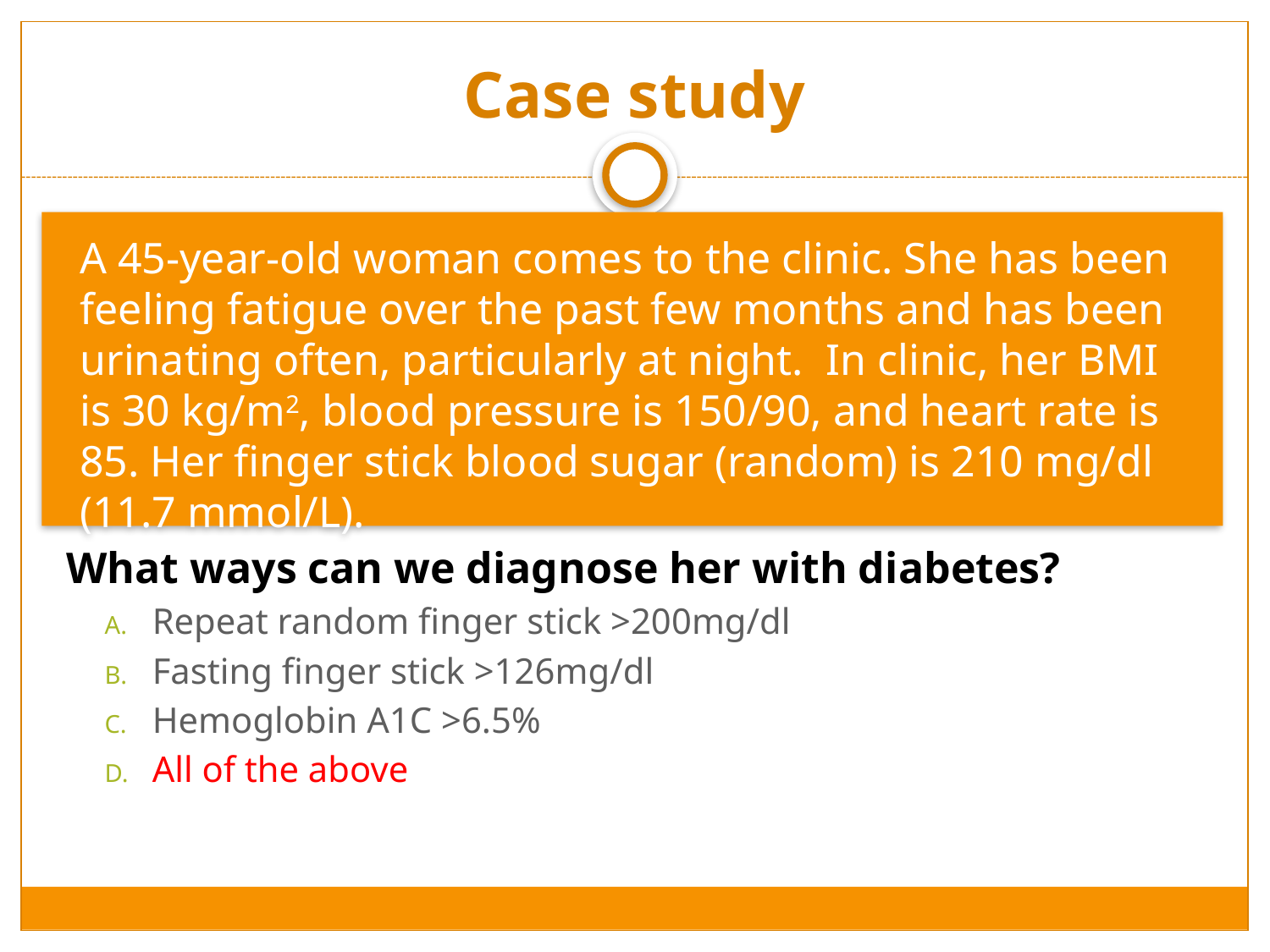

# Case study
A 45-year-old woman comes to the clinic. She has been feeling fatigue over the past few months and has been urinating often, particularly at night. In clinic, her BMI is 30 kg/m2, blood pressure is 150/90, and heart rate is 85. Her finger stick blood sugar (random) is 210 mg/dl (11.7 mmol/L).
What ways can we diagnose her with diabetes?
Repeat random finger stick >200mg/dl
Fasting finger stick >126mg/dl
Hemoglobin A1C >6.5%
All of the above

## Slide 20
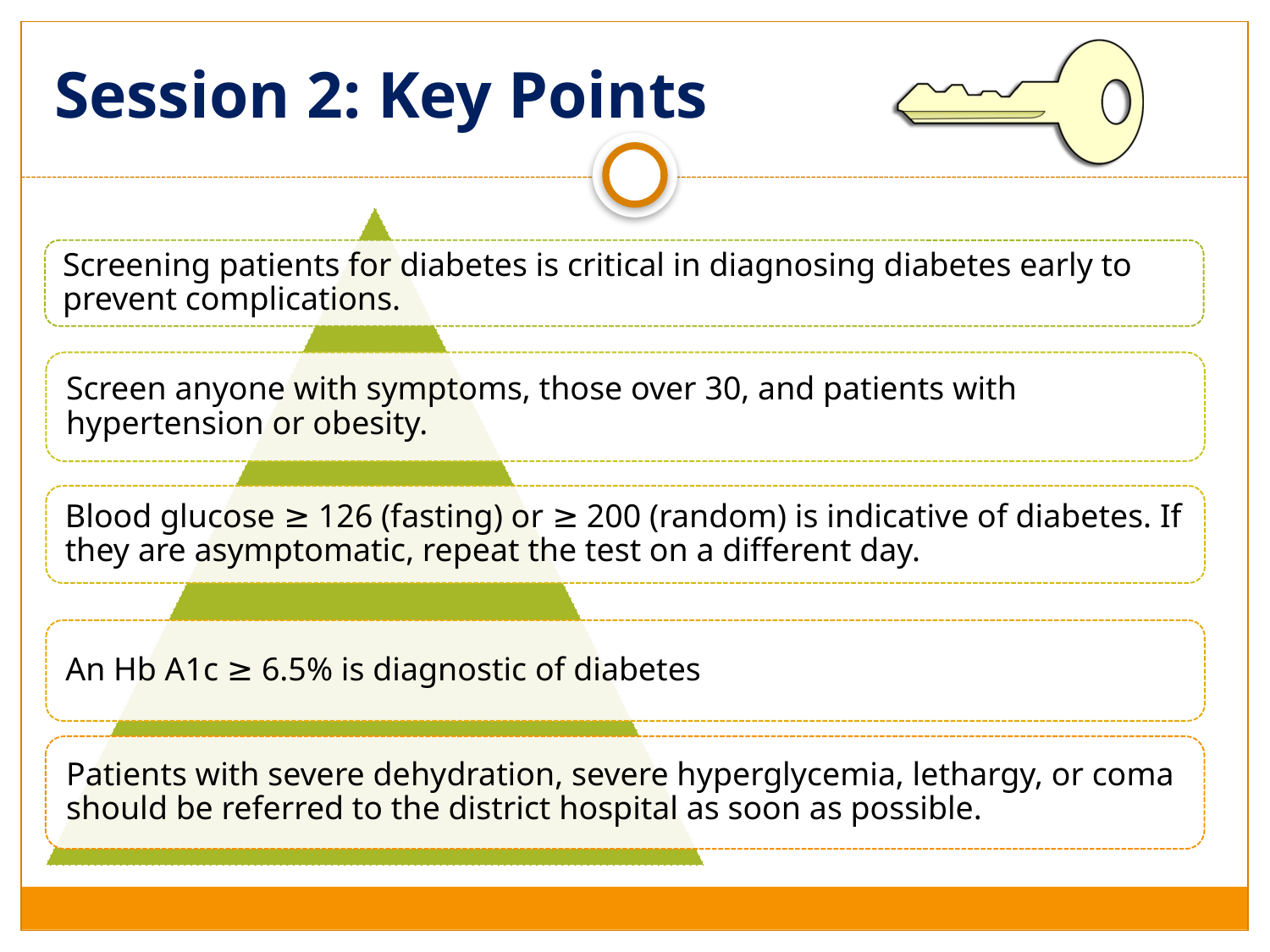

# Session 2: Key Points
