# Supplementary material for: Training Mid-Level Providers to Treat Severe Non-Communicable Diseases in Neno, Malawi through PEN-Plus Strategies
Source: Ann Glob Health. 2022 Aug 11;88(1):69. doi: 10.5334/aogh.3750 (PMC9389951; doi:10.5334/aogh.3750)
Supplement: Didactic Materials. — The supplementary materials contain a suggested didactic training schedule and the PowerPoint presentations used for PEN-Plus training in Neno, Malawi. These materials have been reviewed and accepted by the Malawi Ministry of Health for future PEN-Plus trainings in Malawi. [file agh-88-1-3750-s2.zip › Didactic_Materials/DM_Epi and Path.pptx]

## Slide 1
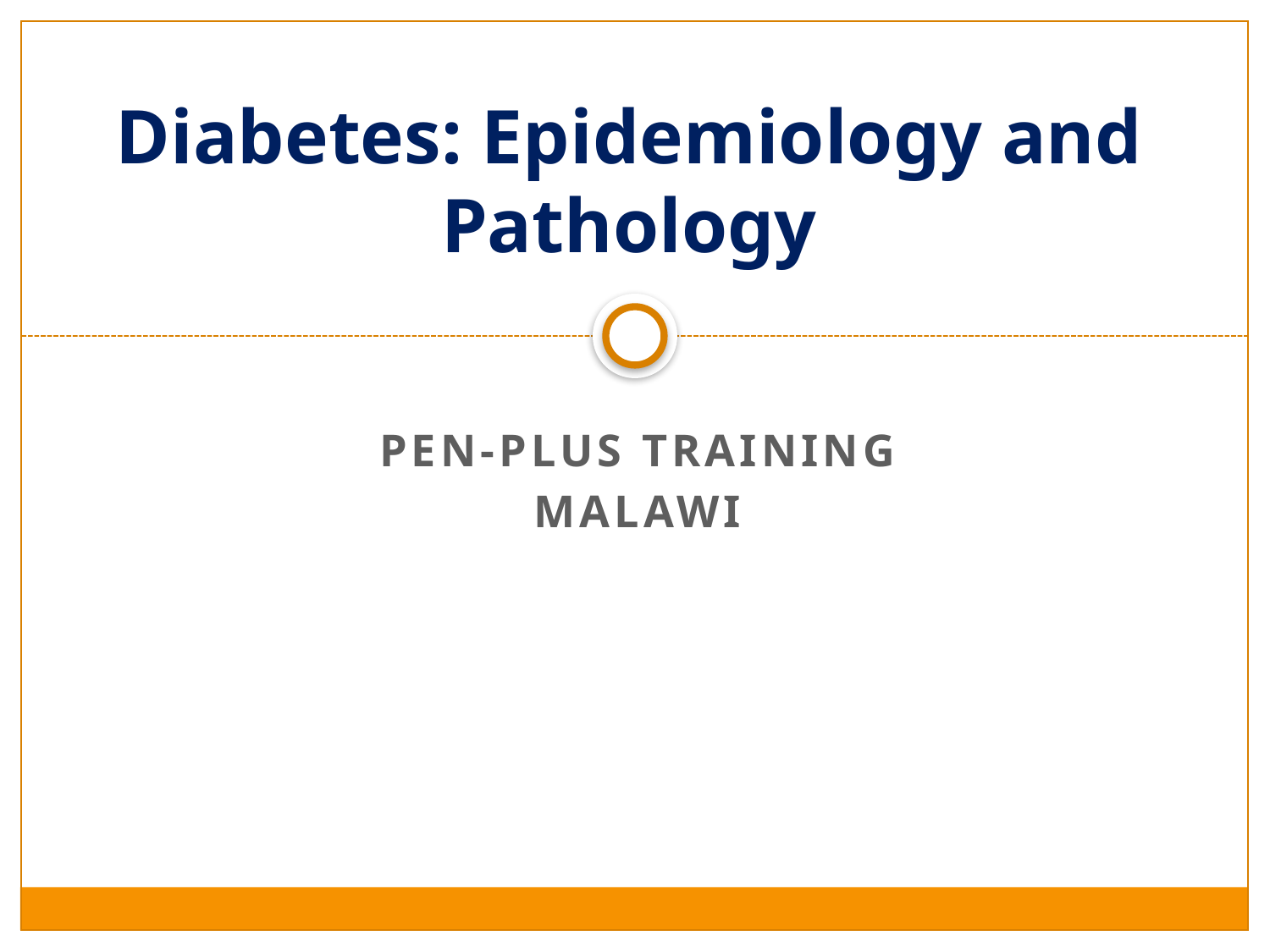

# Diabetes: Epidemiology and Pathology
PEN-Plus training
Malawi

## Slide 2
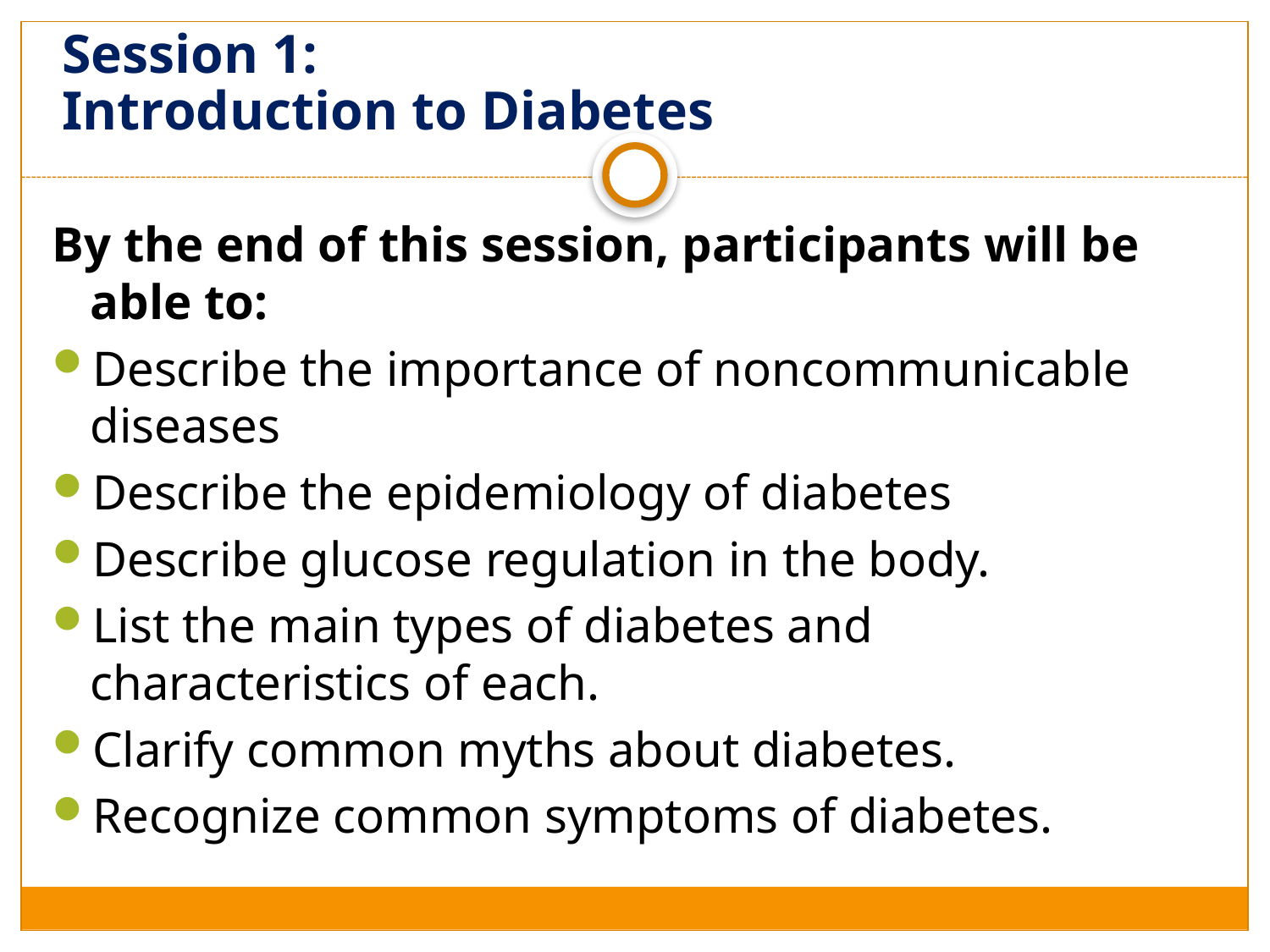

# Session 1: Introduction to Diabetes
By the end of this session, participants will be able to:
Describe the importance of noncommunicable diseases
Describe the epidemiology of diabetes
Describe glucose regulation in the body.
List the main types of diabetes and characteristics of each.
Clarify common myths about diabetes.
Recognize common symptoms of diabetes.

## Slide 3
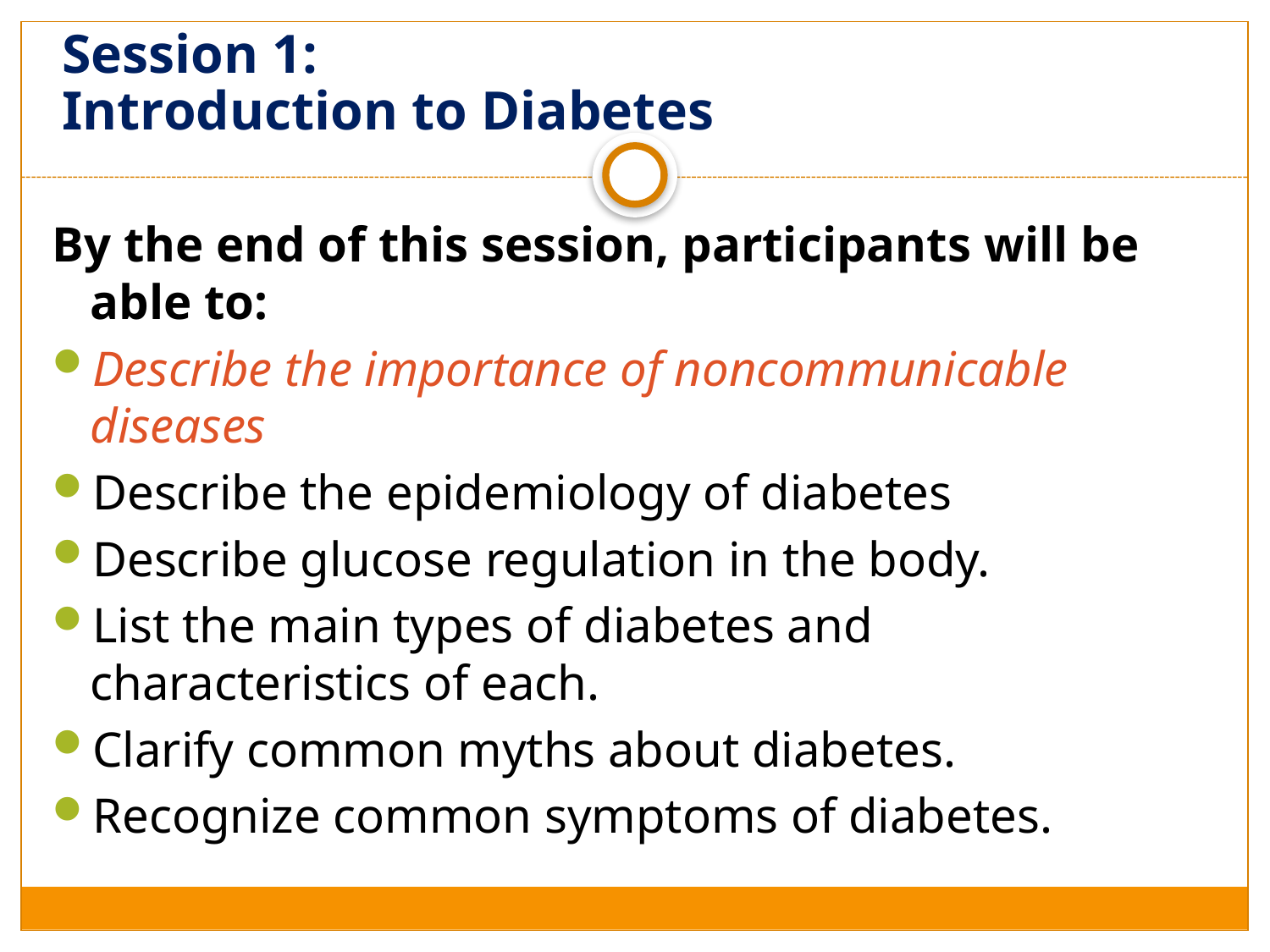

# Session 1: Introduction to Diabetes
By the end of this session, participants will be able to:
Describe the importance of noncommunicable diseases
Describe the epidemiology of diabetes
Describe glucose regulation in the body.
List the main types of diabetes and characteristics of each.
Clarify common myths about diabetes.
Recognize common symptoms of diabetes.

## Slide 4
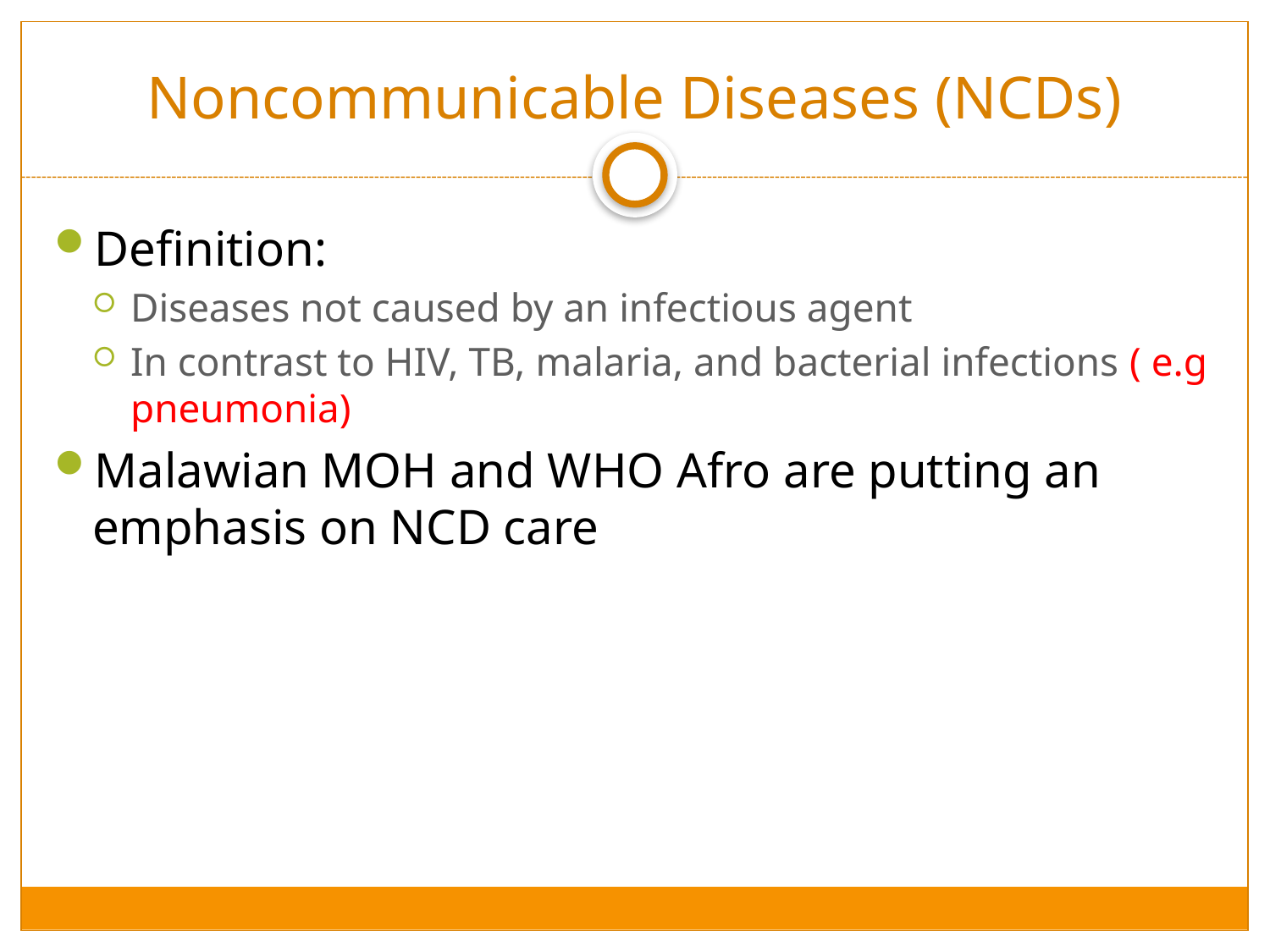

# Noncommunicable Diseases (NCDs)
Definition:
Diseases not caused by an infectious agent
In contrast to HIV, TB, malaria, and bacterial infections ( e.g pneumonia)
Malawian MOH and WHO Afro are putting an emphasis on NCD care

## Slide 5
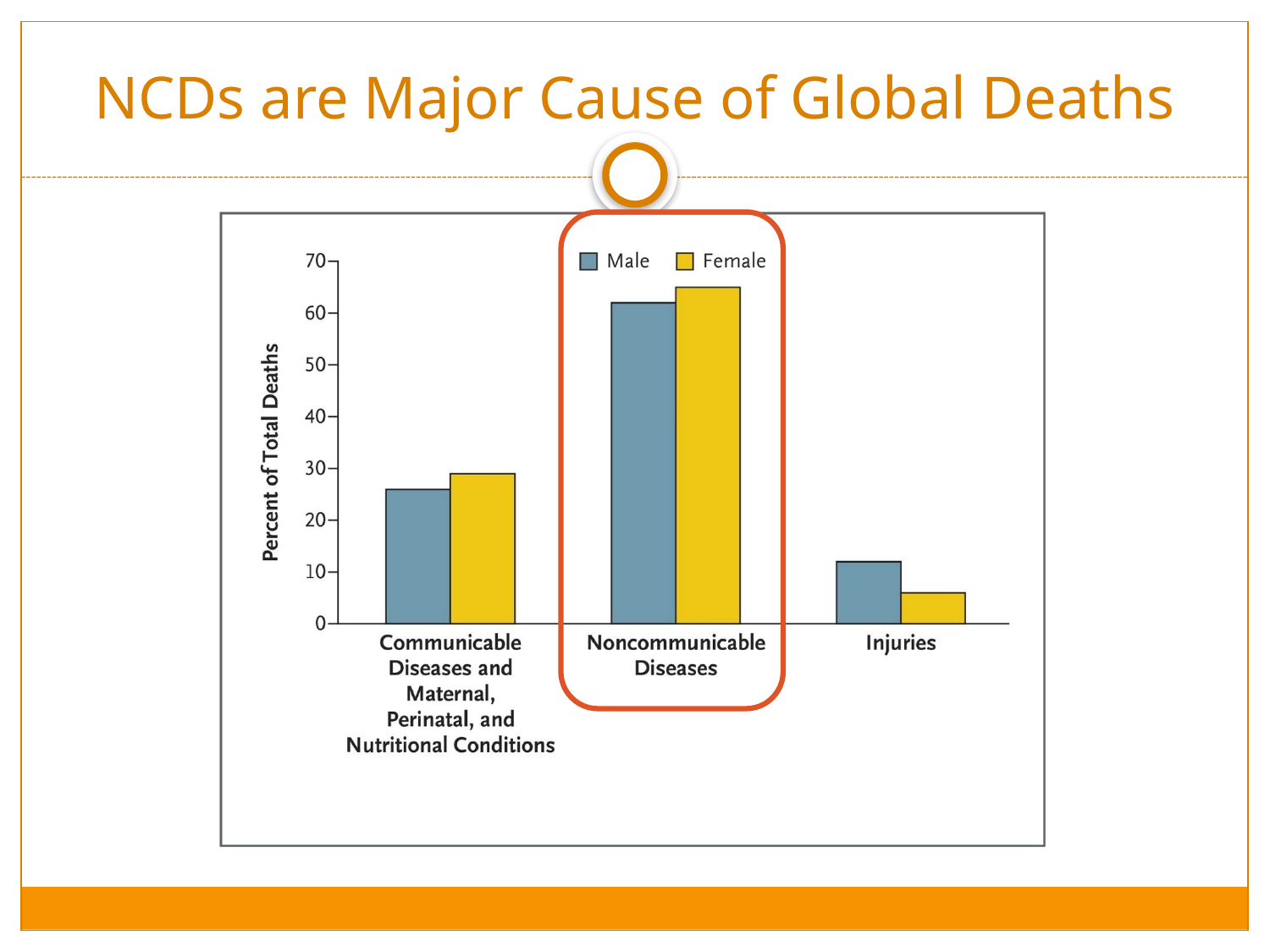

# NCDs are Major Cause of Global Deaths

## Slide 6
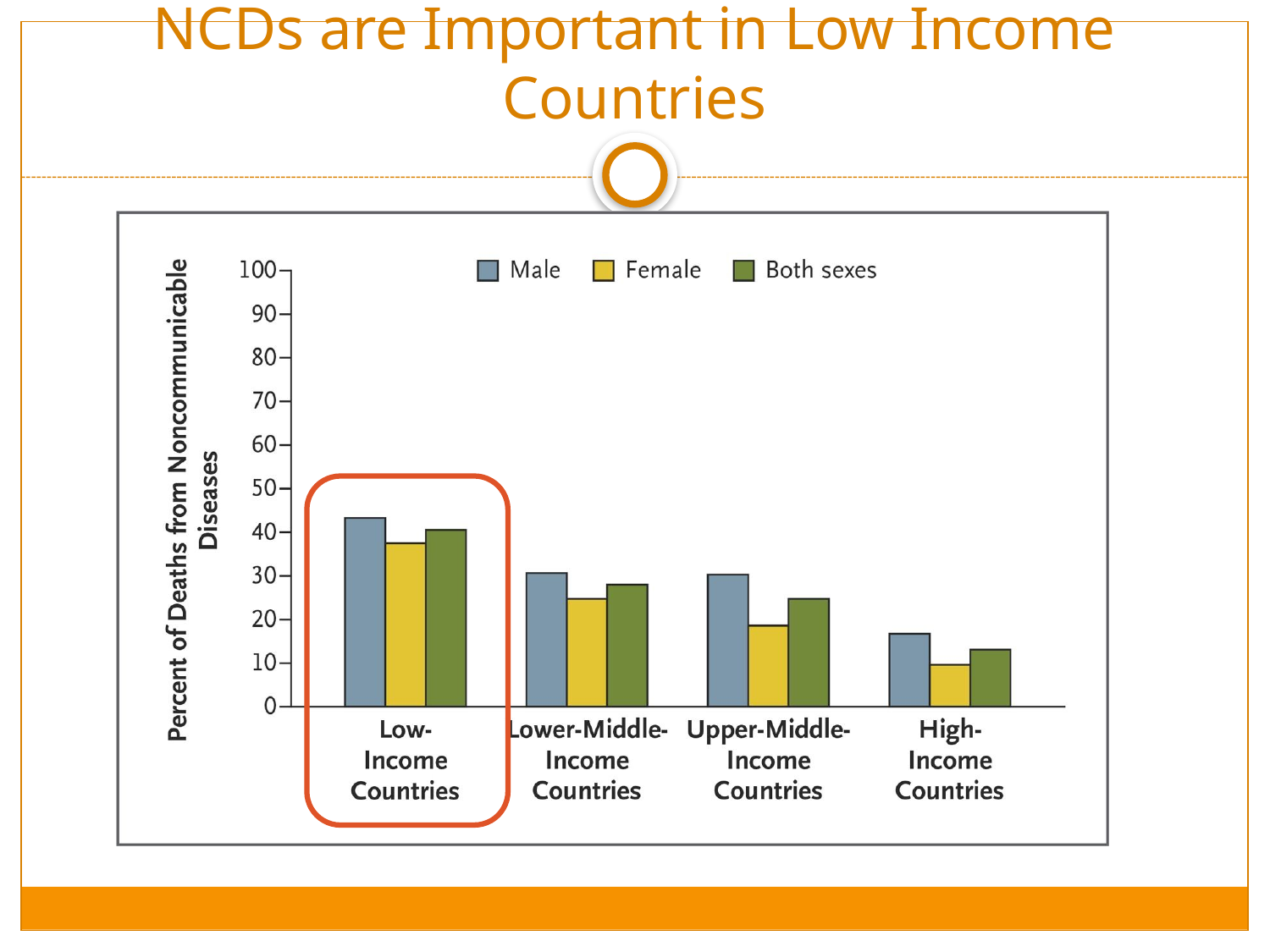

# NCDs are Important in Low Income Countries

## Slide 7
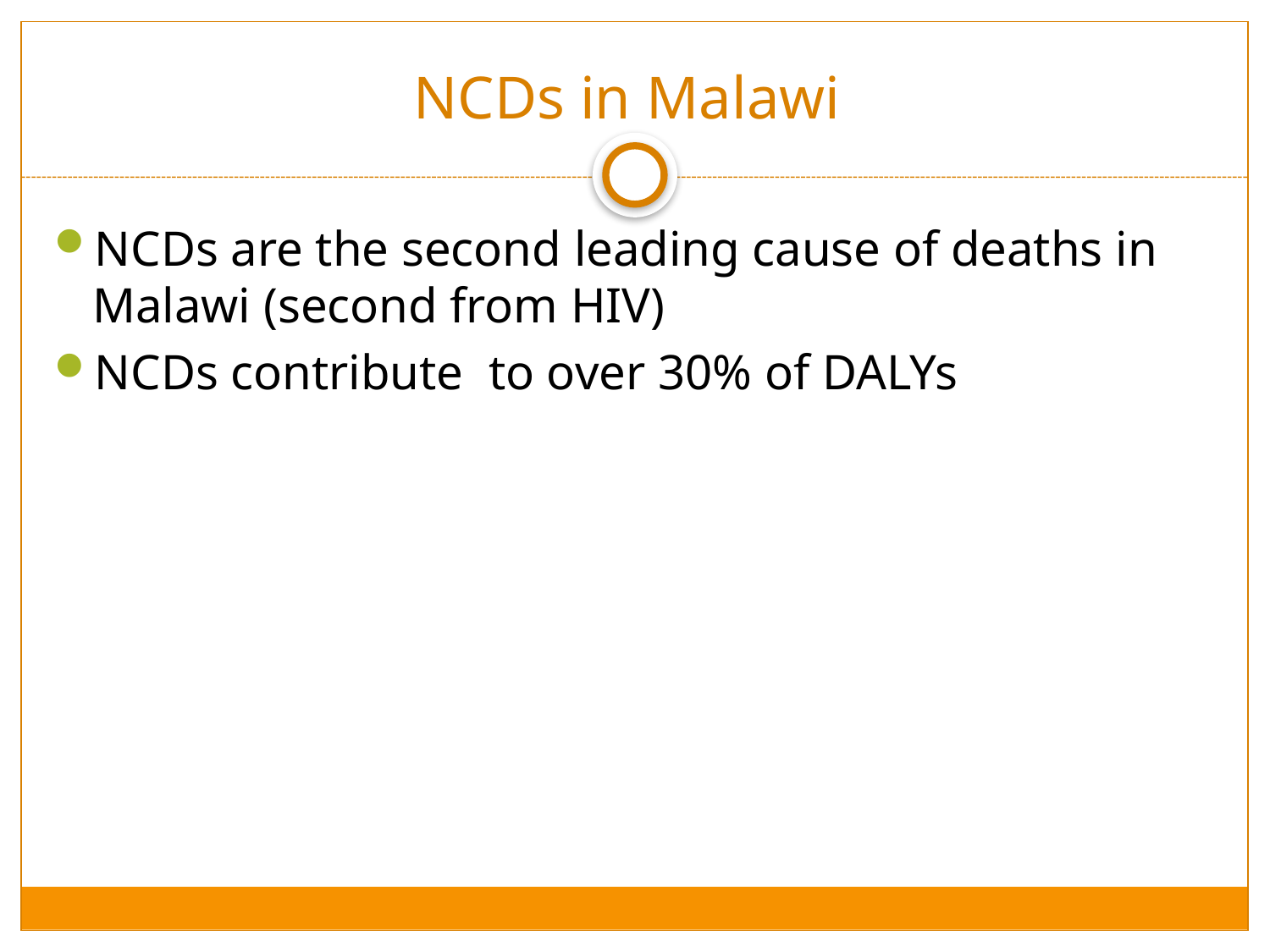

# NCDs in Malawi
NCDs are the second leading cause of deaths in Malawi (second from HIV)
NCDs contribute to over 30% of DALYs

## Slide 8
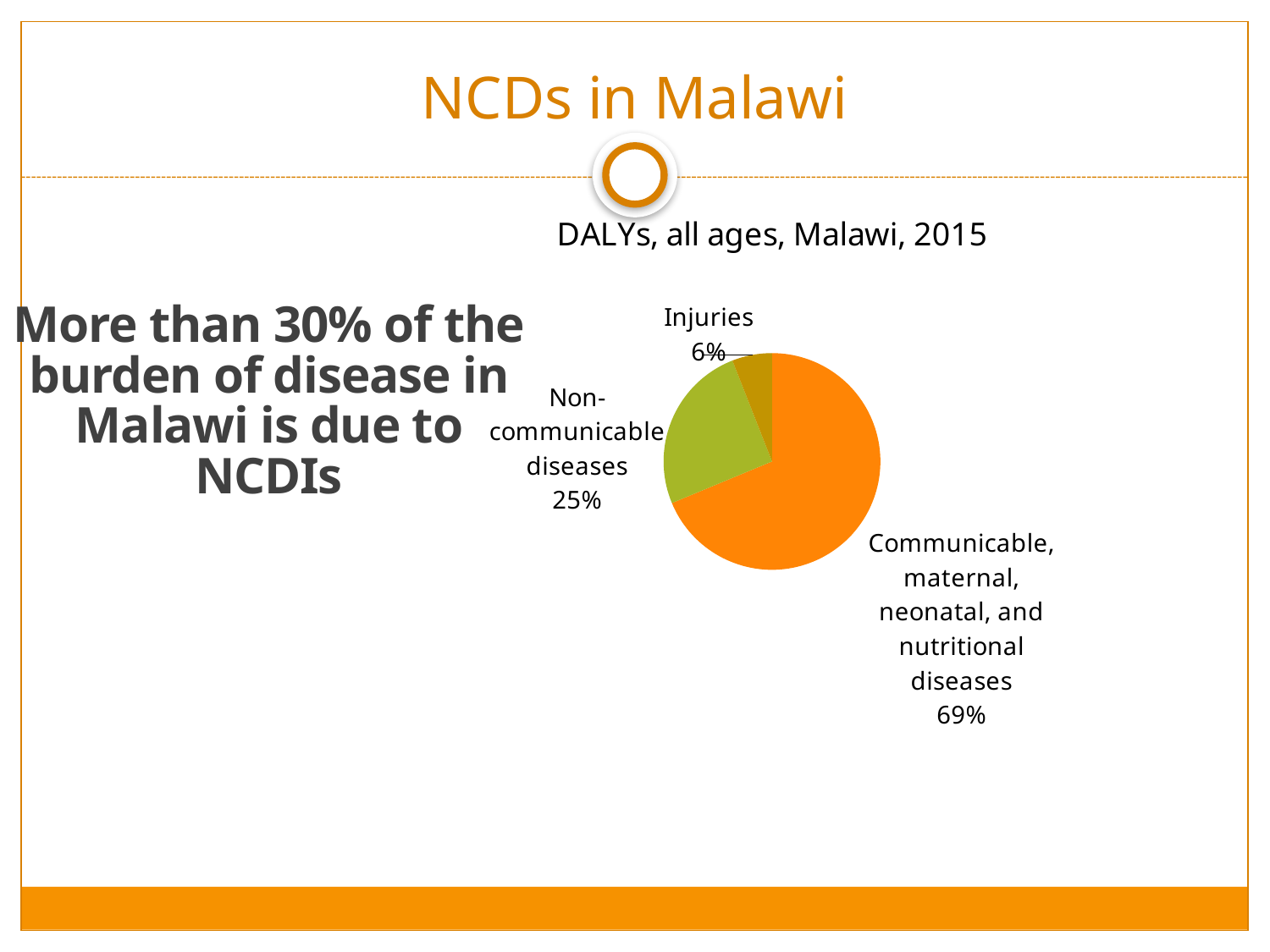

# NCDs in Malawi
### Chart: DALYs, all ages, Malawi, 2015
| Category | |
|---|---|
| Communicable, maternal, neonatal, and nutritional diseases | 0.68723782518302 |
| Non-communicable diseases | 0.25326703805596 |
| Injuries | 0.05949513676102 |More than 30% of the burden of disease in Malawi is due to NCDIs

## Slide 9
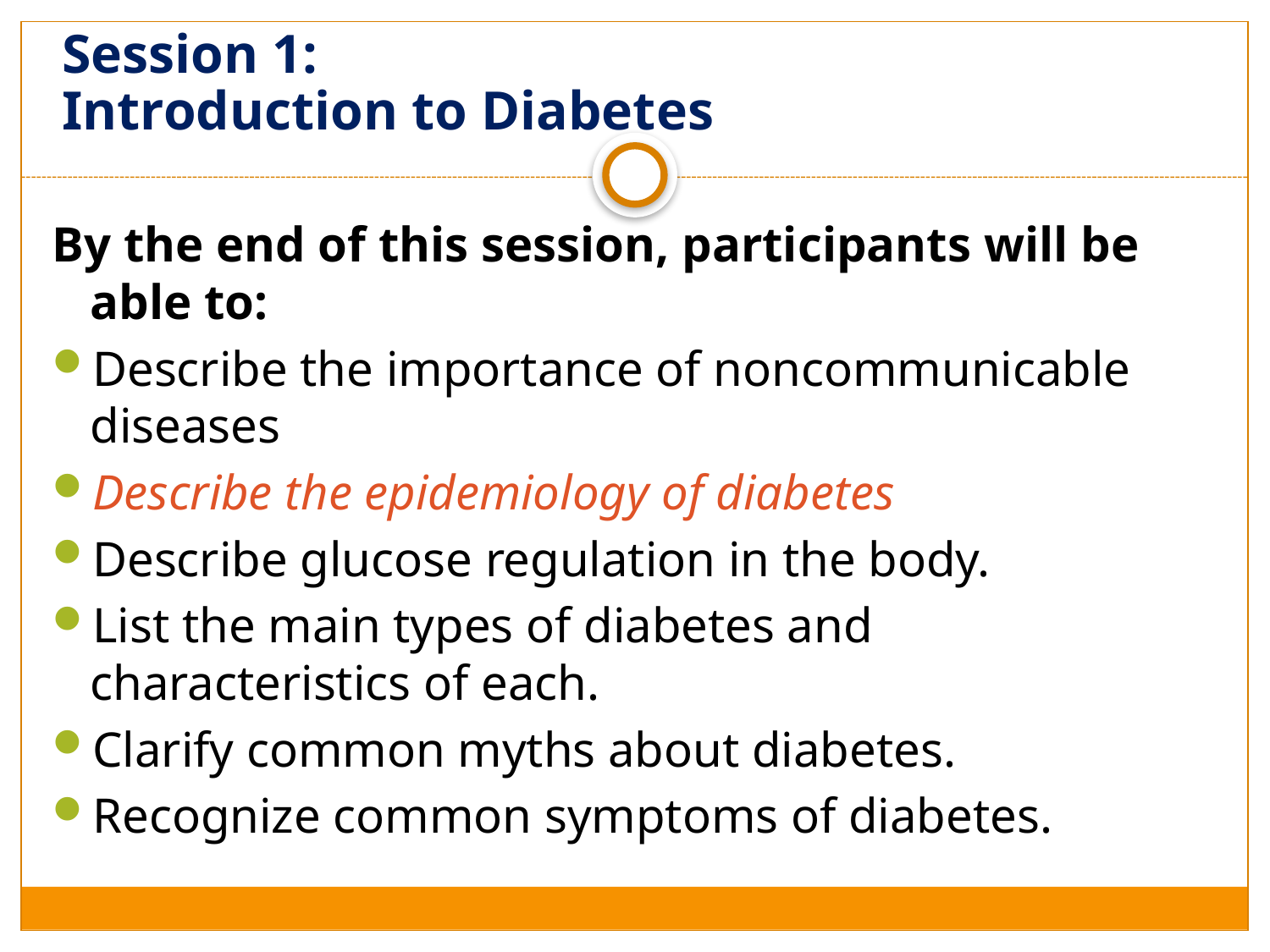

# Session 1: Introduction to Diabetes
By the end of this session, participants will be able to:
Describe the importance of noncommunicable diseases
Describe the epidemiology of diabetes
Describe glucose regulation in the body.
List the main types of diabetes and characteristics of each.
Clarify common myths about diabetes.
Recognize common symptoms of diabetes.

## Slide 10
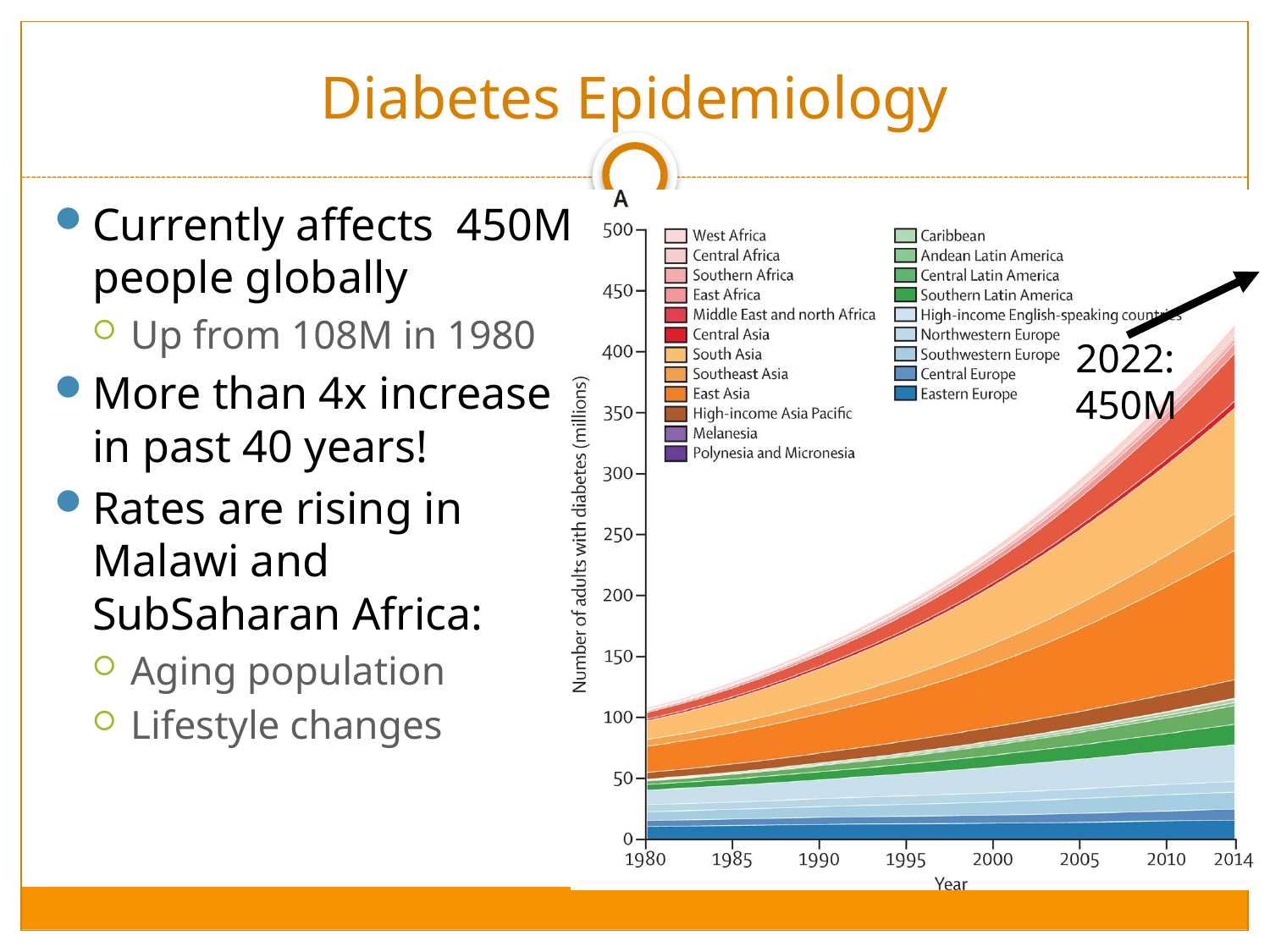

# Diabetes Epidemiology
Currently affects 450M people globally
Up from 108M in 1980
More than 4x increase in past 40 years!
Rates are rising in Malawi and SubSaharan Africa:
Aging population
Lifestyle changes
2022: 450M

## Slide 11
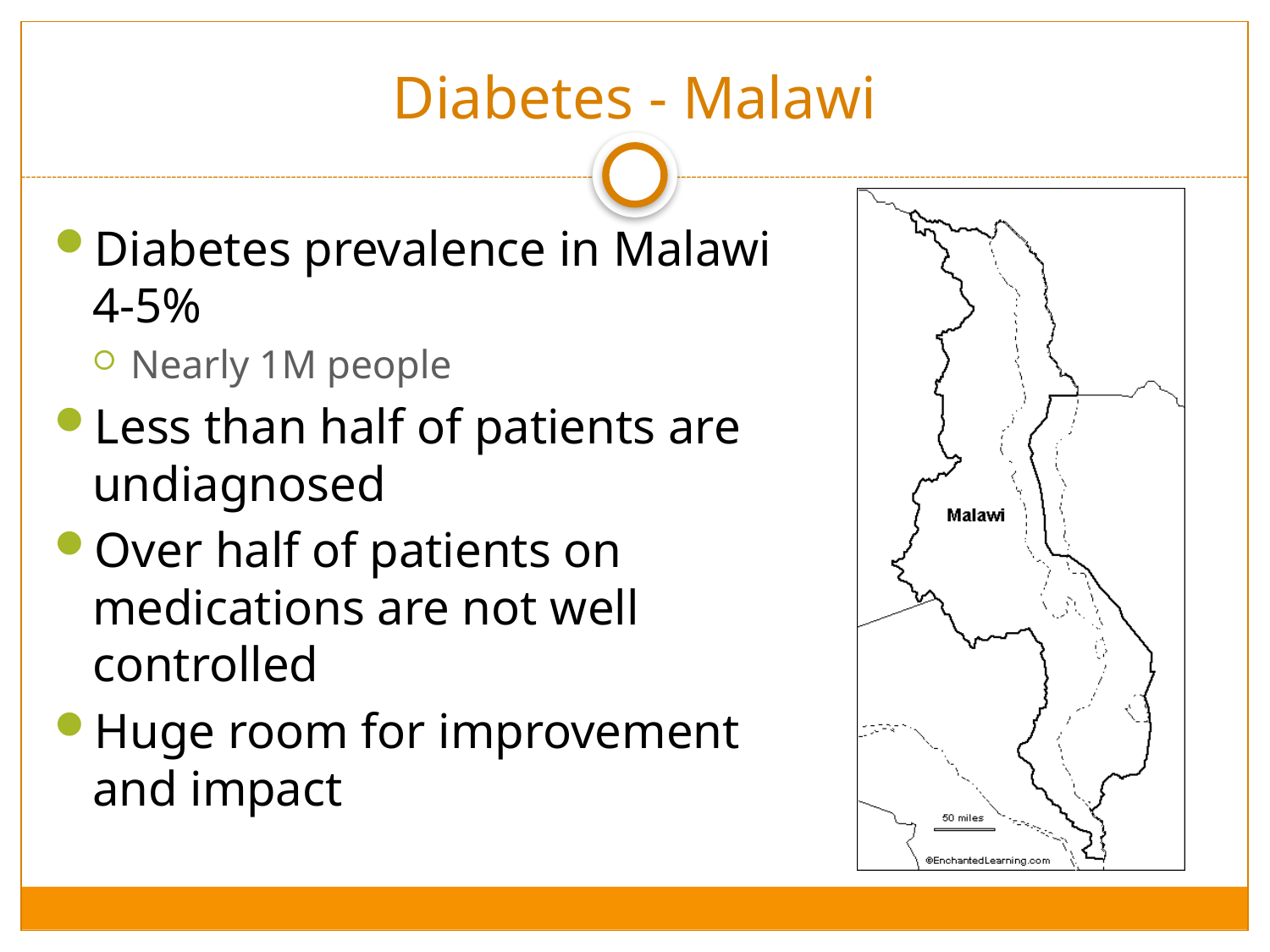

# Diabetes - Malawi
Diabetes prevalence in Malawi 4-5%
Nearly 1M people
Less than half of patients are undiagnosed
Over half of patients on medications are not well controlled
Huge room for improvement and impact

## Slide 12
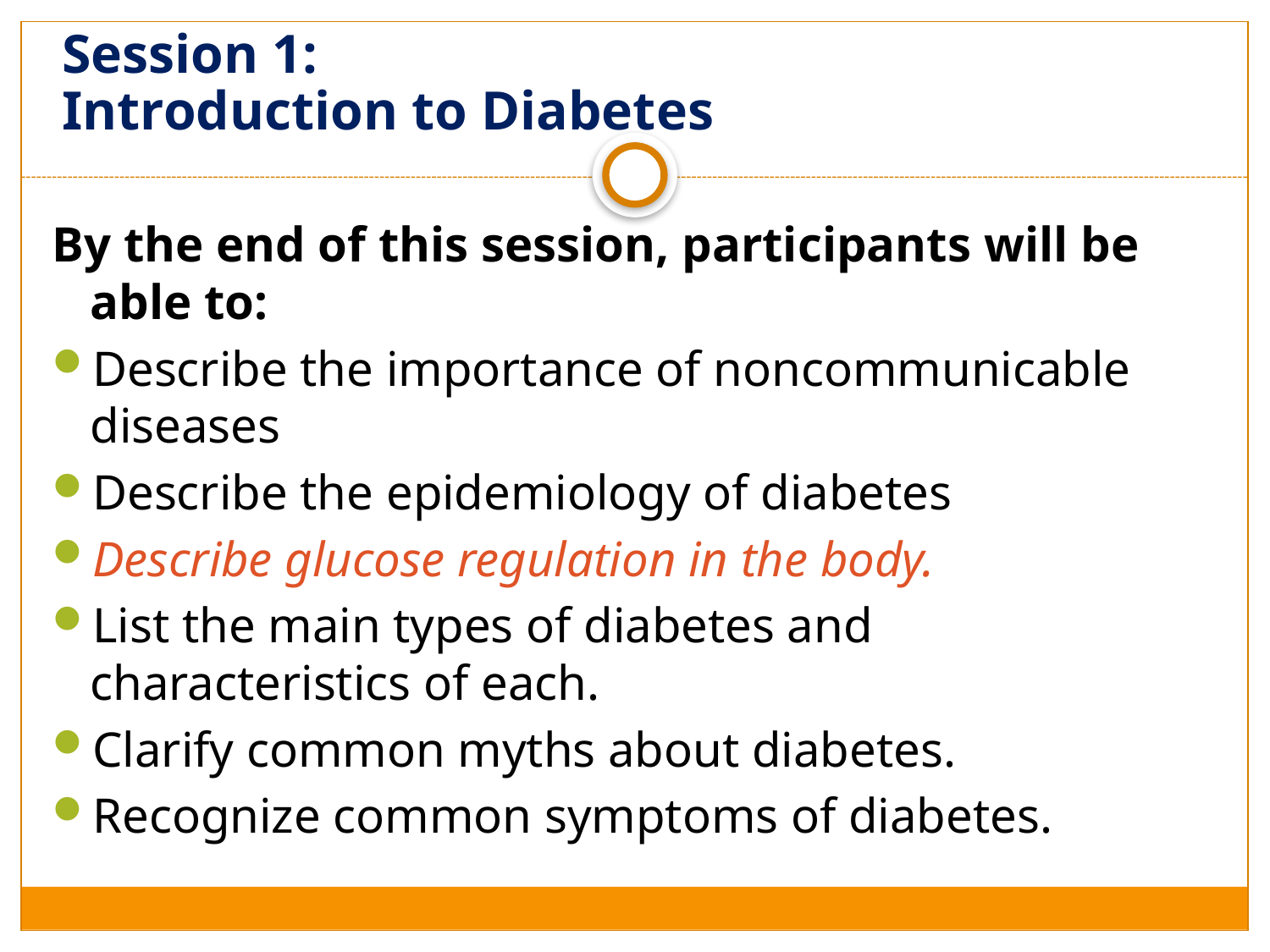

# Session 1: Introduction to Diabetes
By the end of this session, participants will be able to:
Describe the importance of noncommunicable diseases
Describe the epidemiology of diabetes
Describe glucose regulation in the body.
List the main types of diabetes and characteristics of each.
Clarify common myths about diabetes.
Recognize common symptoms of diabetes.

## Slide 13
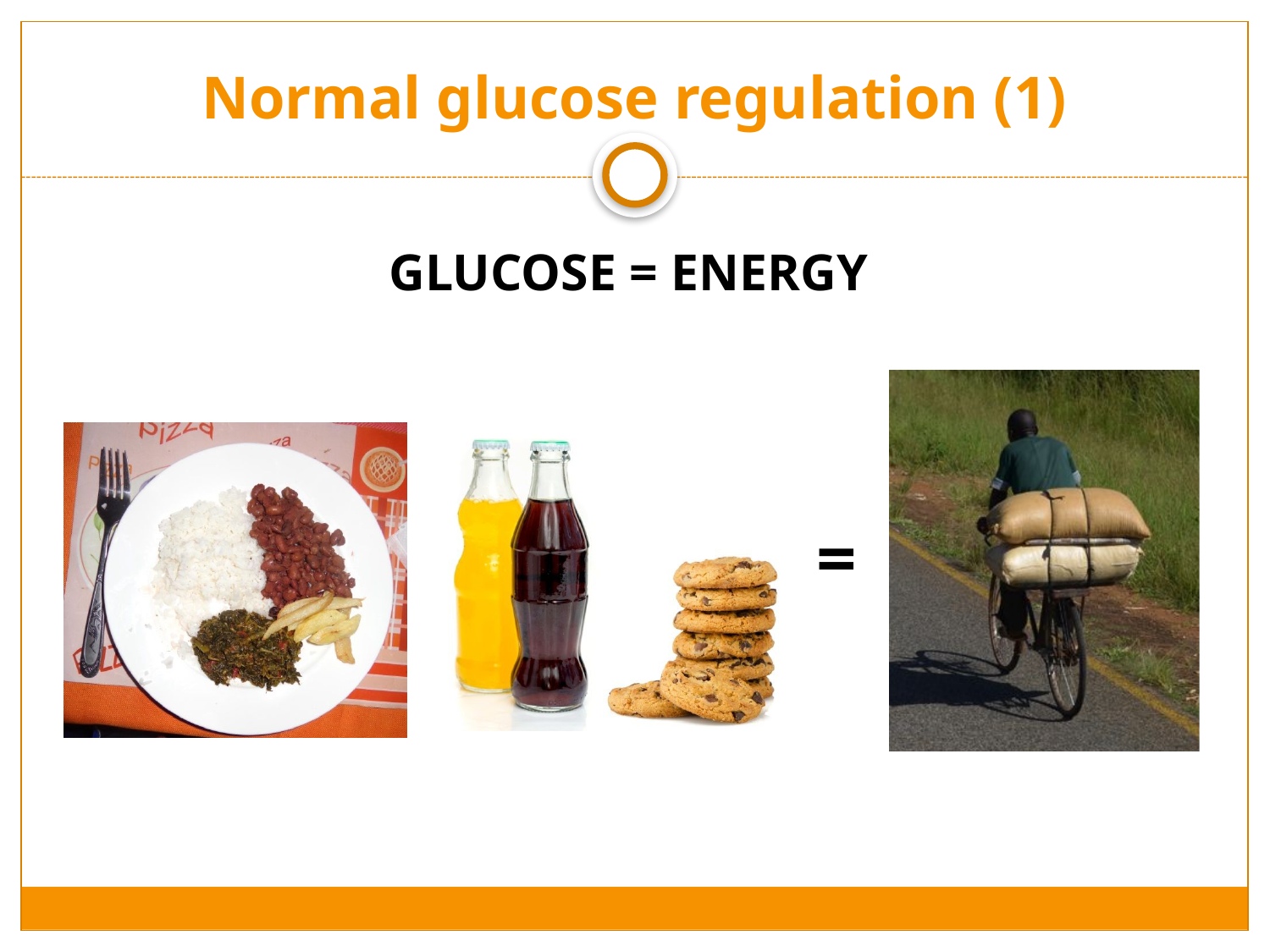

# Normal glucose regulation (1)
GLUCOSE = ENERGY
=

## Slide 14
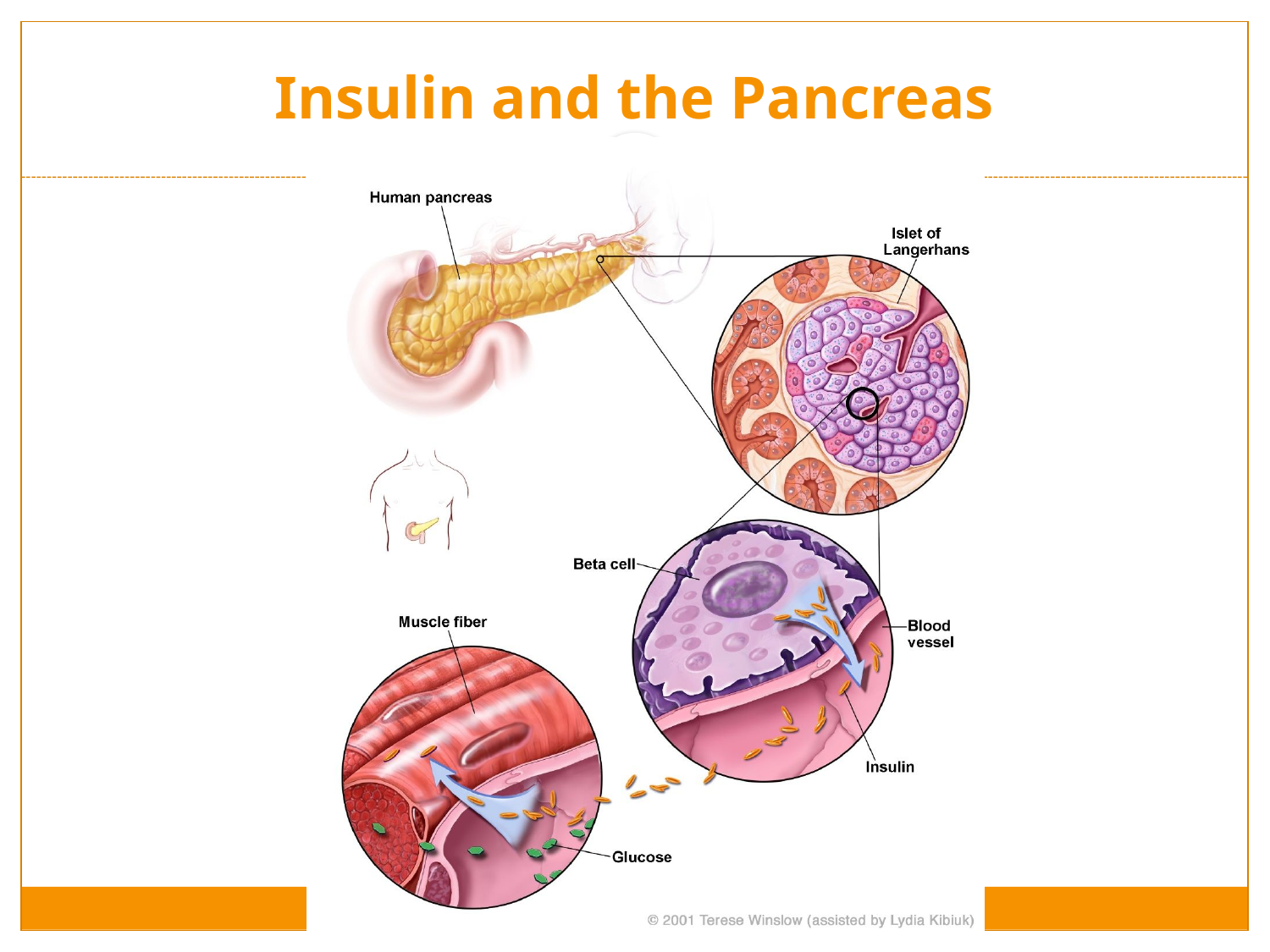

# Insulin and the Pancreas

## Slide 15
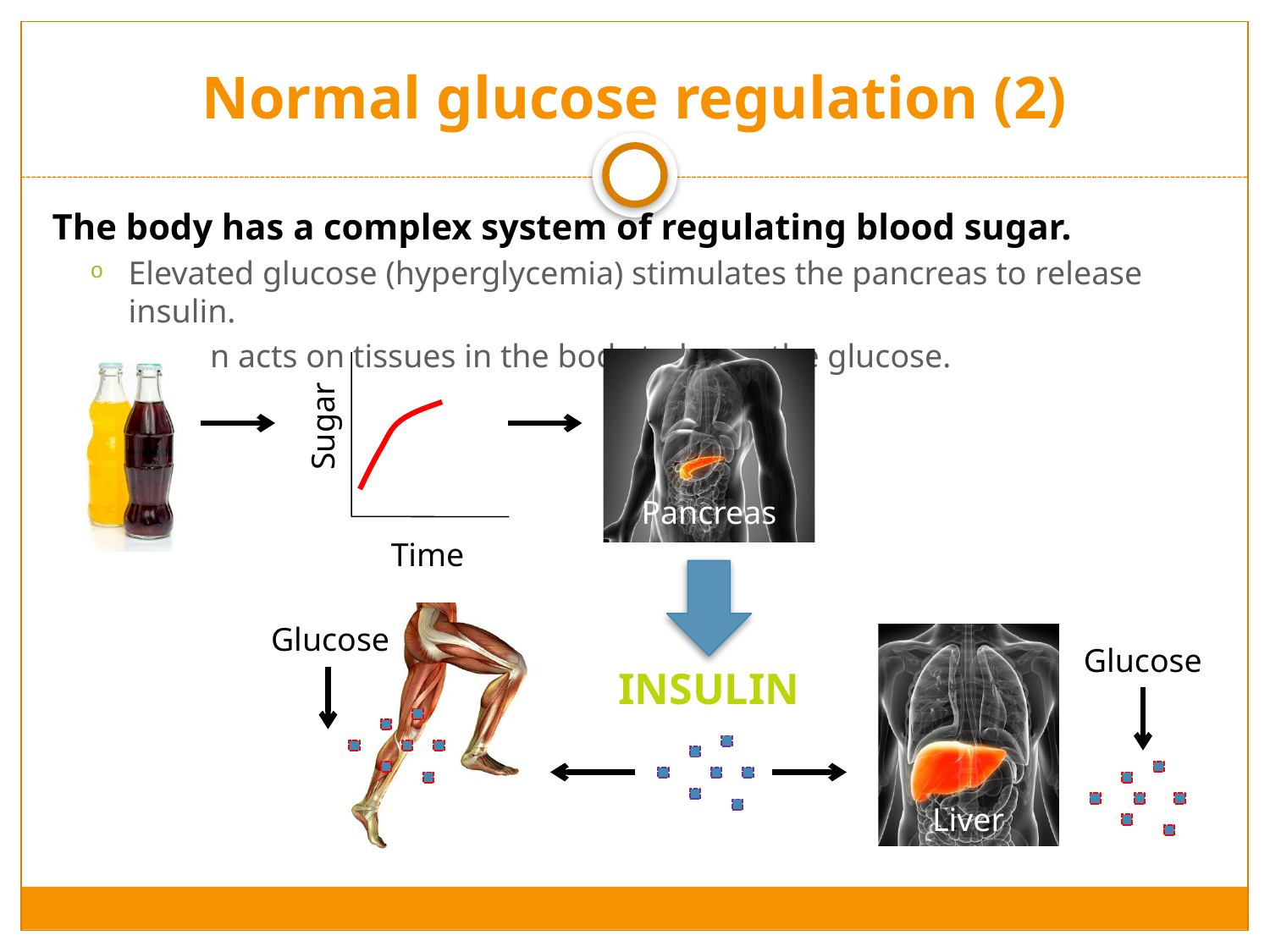

# Normal glucose regulation (2)
The body has a complex system of regulating blood sugar.
Elevated glucose (hyperglycemia) stimulates the pancreas to release insulin.
Insulin acts on tissues in the body to lower the glucose.
Pancreas
Sugar
Time
Glucose
Glucose
INSULIN
Liver

## Slide 16
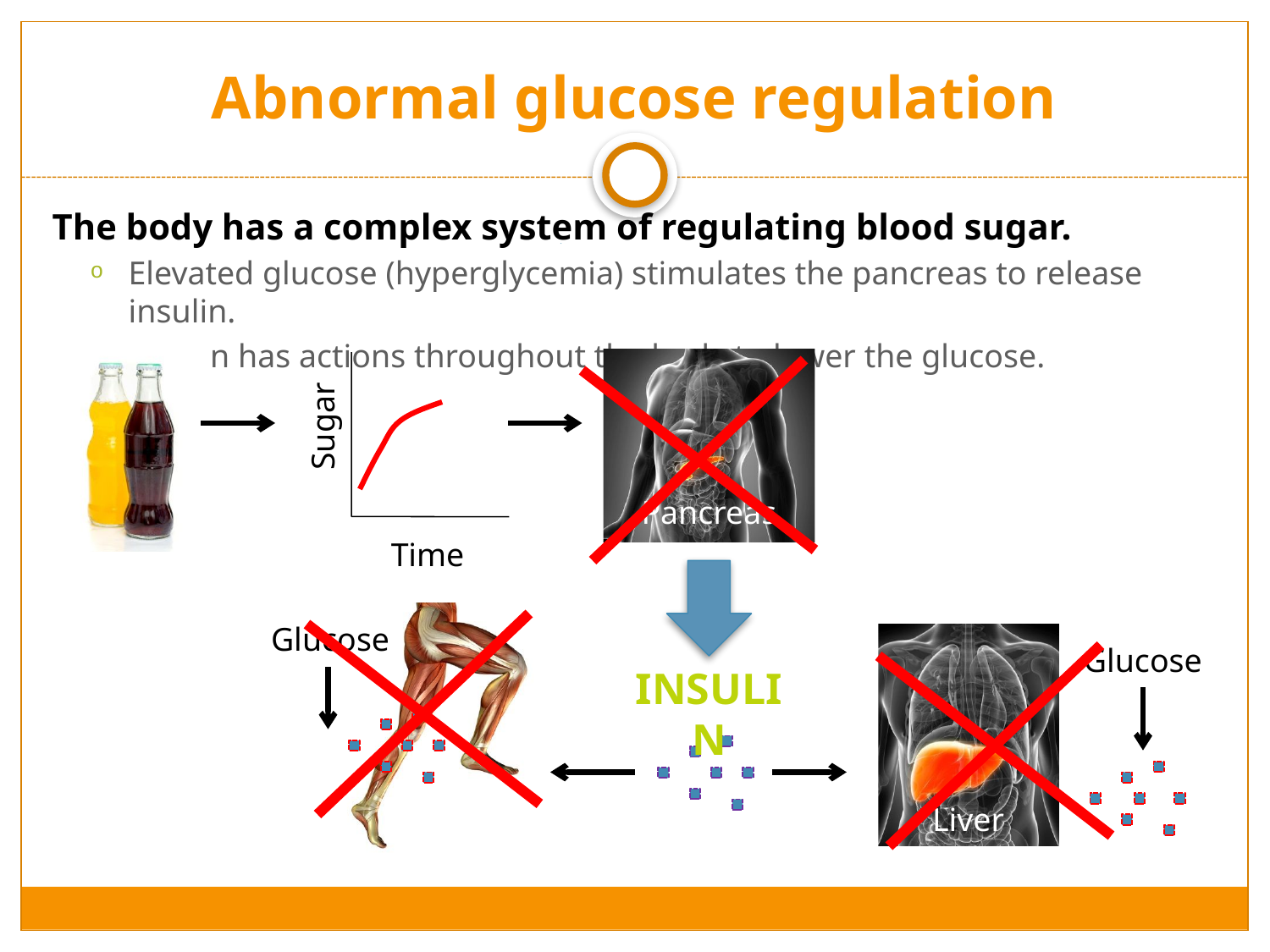

# Abnormal glucose regulation
The body has a complex system of regulating blood sugar.
Elevated glucose (hyperglycemia) stimulates the pancreas to release insulin.
Insulin has actions throughout the body to lower the glucose.
Pancreas
Sugar
Time
Glucose
Glucose
INSULIN
Liver

## Slide 17
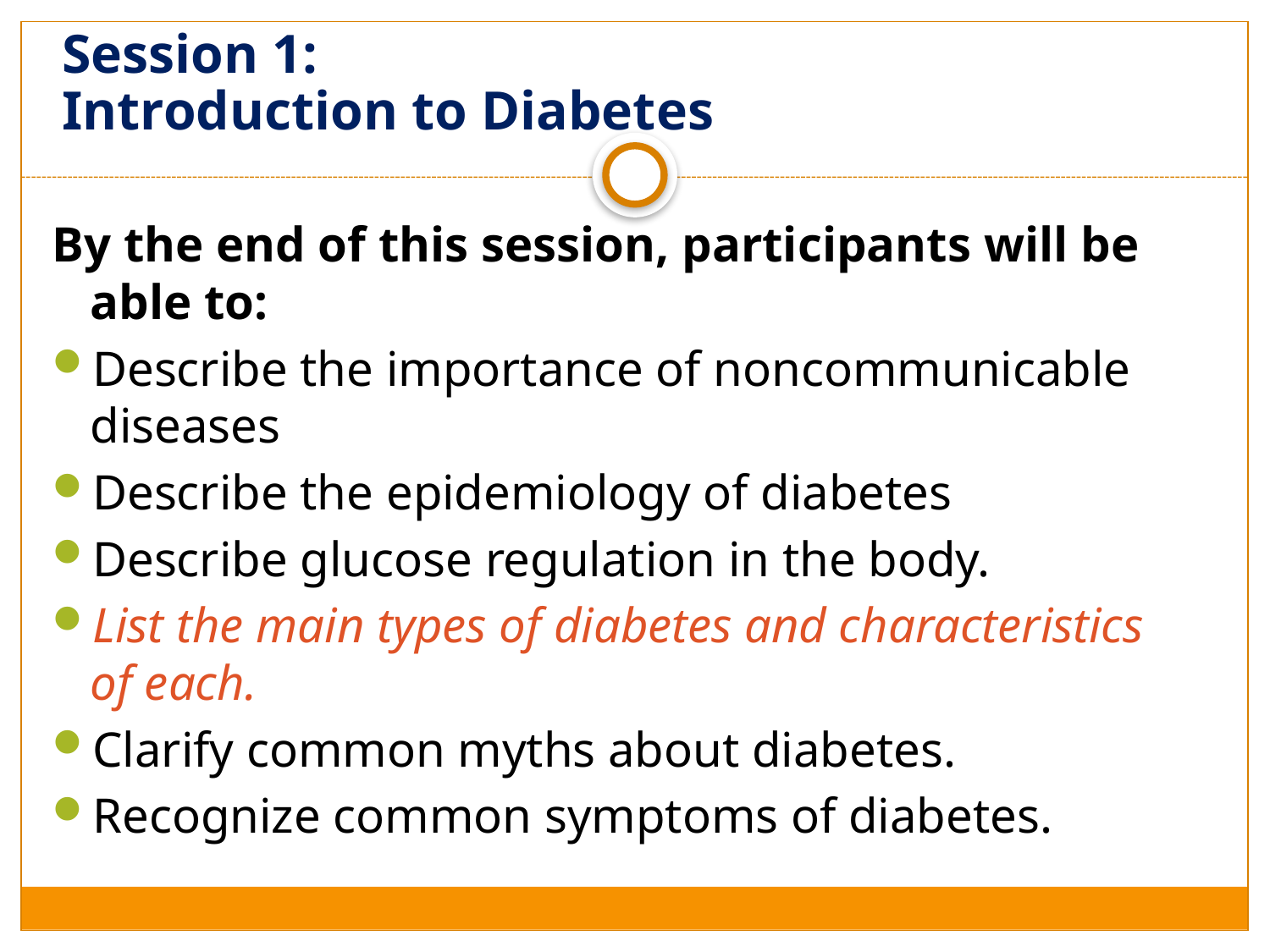

# Session 1: Introduction to Diabetes
By the end of this session, participants will be able to:
Describe the importance of noncommunicable diseases
Describe the epidemiology of diabetes
Describe glucose regulation in the body.
List the main types of diabetes and characteristics of each.
Clarify common myths about diabetes.
Recognize common symptoms of diabetes.

## Slide 18
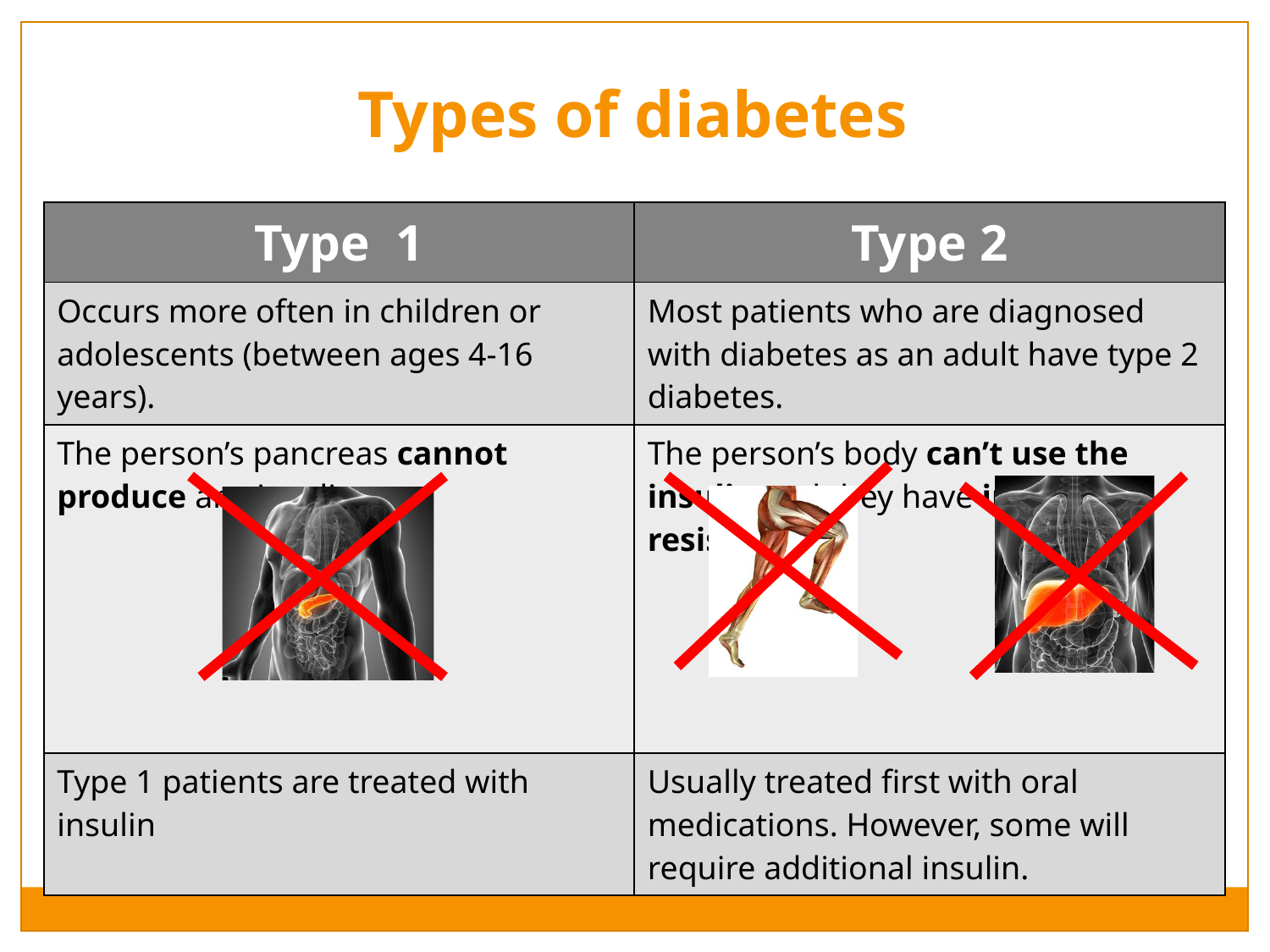

Types of diabetes
| Type 1 | Type 2 |
| --- | --- |
| Occurs more often in children or adolescents (between ages 4-16 years). | Most patients who are diagnosed with diabetes as an adult have type 2 diabetes. |
| The person’s pancreas cannot produce any insulin | The person’s body can’t use the insulin and they have insulin resistance. |
| Type 1 patients are treated with insulin | Usually treated first with oral medications. However, some will require additional insulin. |

## Slide 19
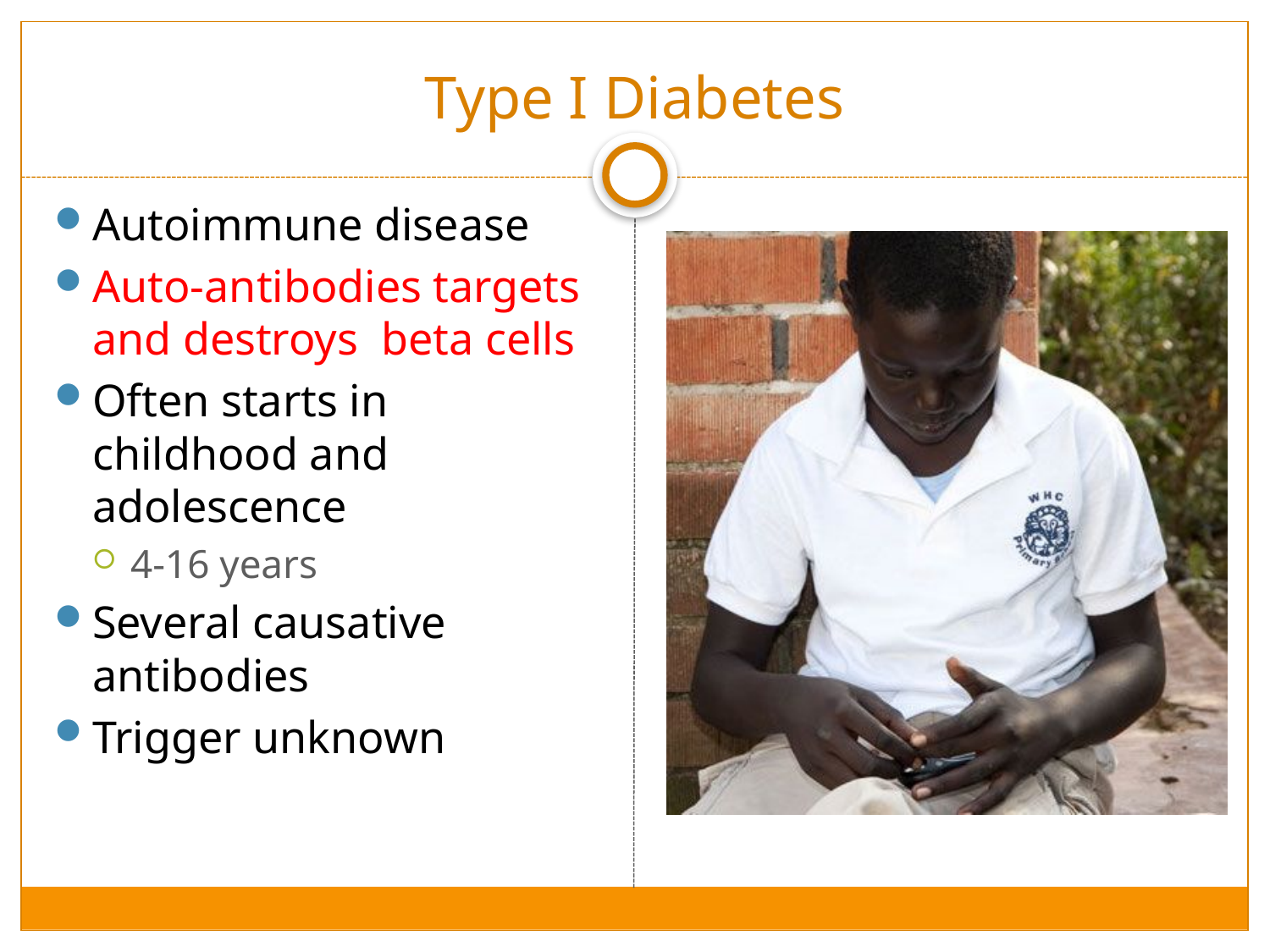

# Type I Diabetes
Autoimmune disease
Auto-antibodies targets and destroys beta cells
Often starts in childhood and adolescence
4-16 years
Several causative antibodies
Trigger unknown

## Slide 20
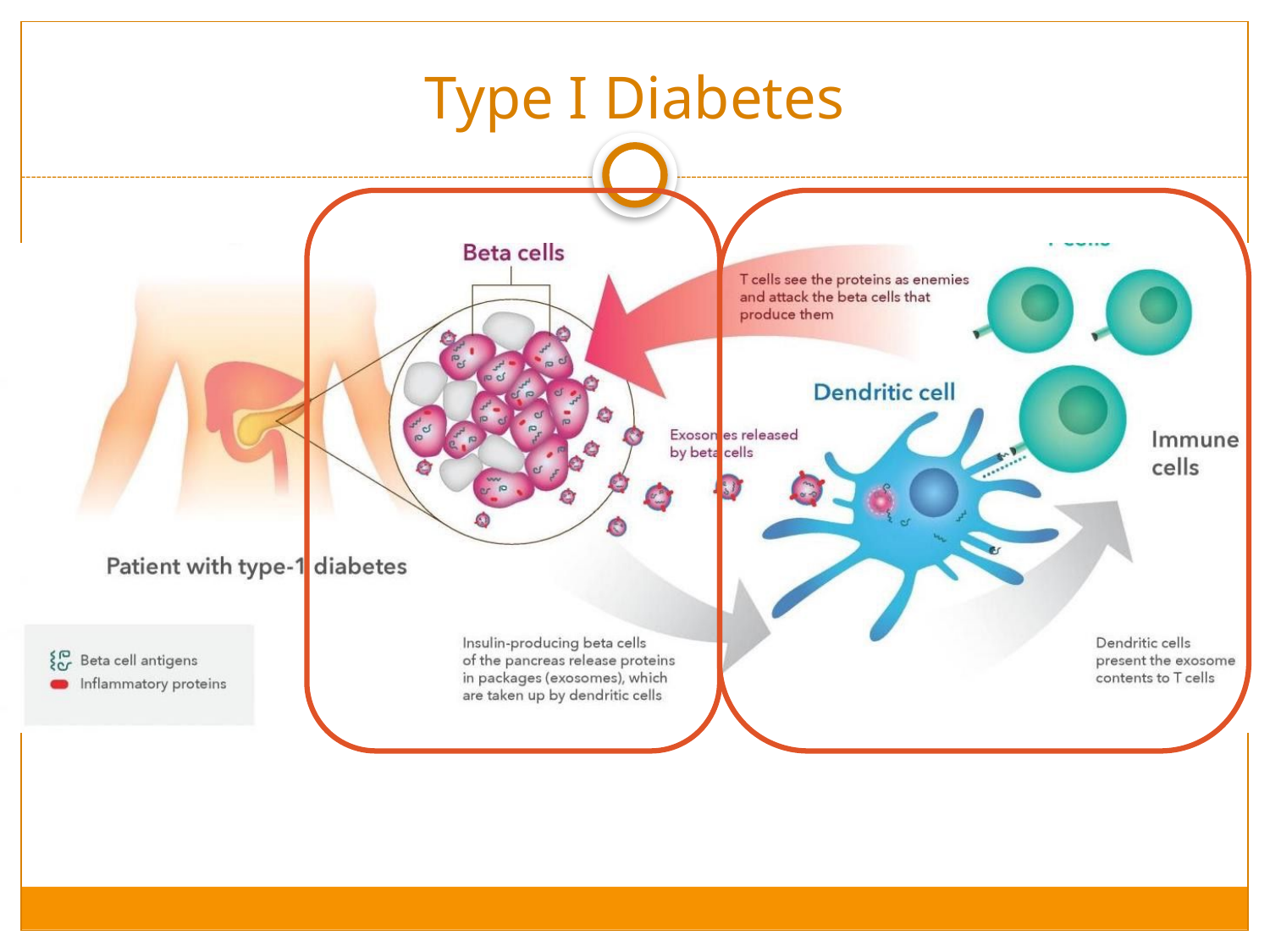

# Type I Diabetes

## Slide 21
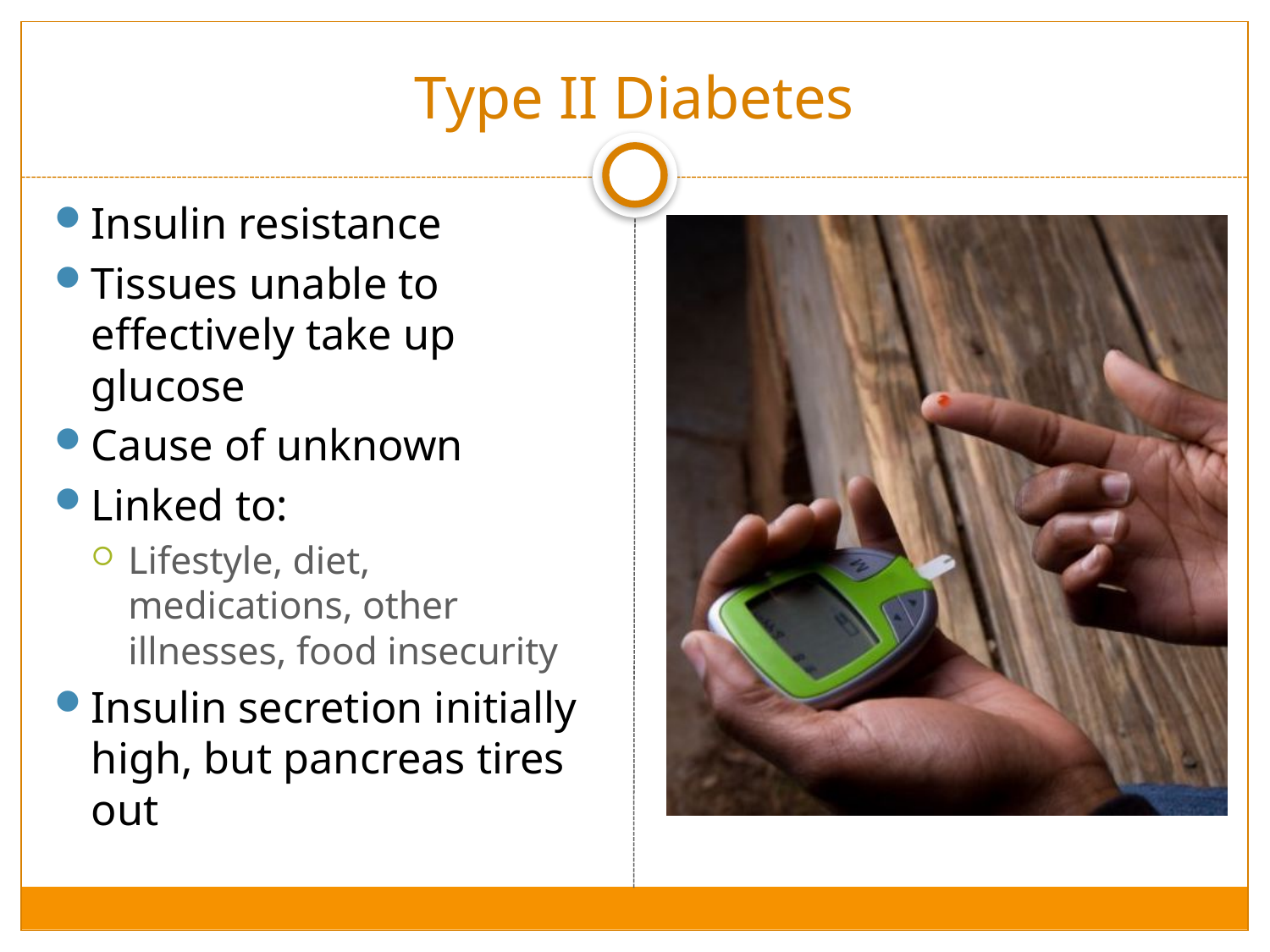

# Type II Diabetes
Insulin resistance
Tissues unable to effectively take up glucose
Cause of unknown
Linked to:
Lifestyle, diet, medications, other illnesses, food insecurity
Insulin secretion initially high, but pancreas tires out

## Slide 22
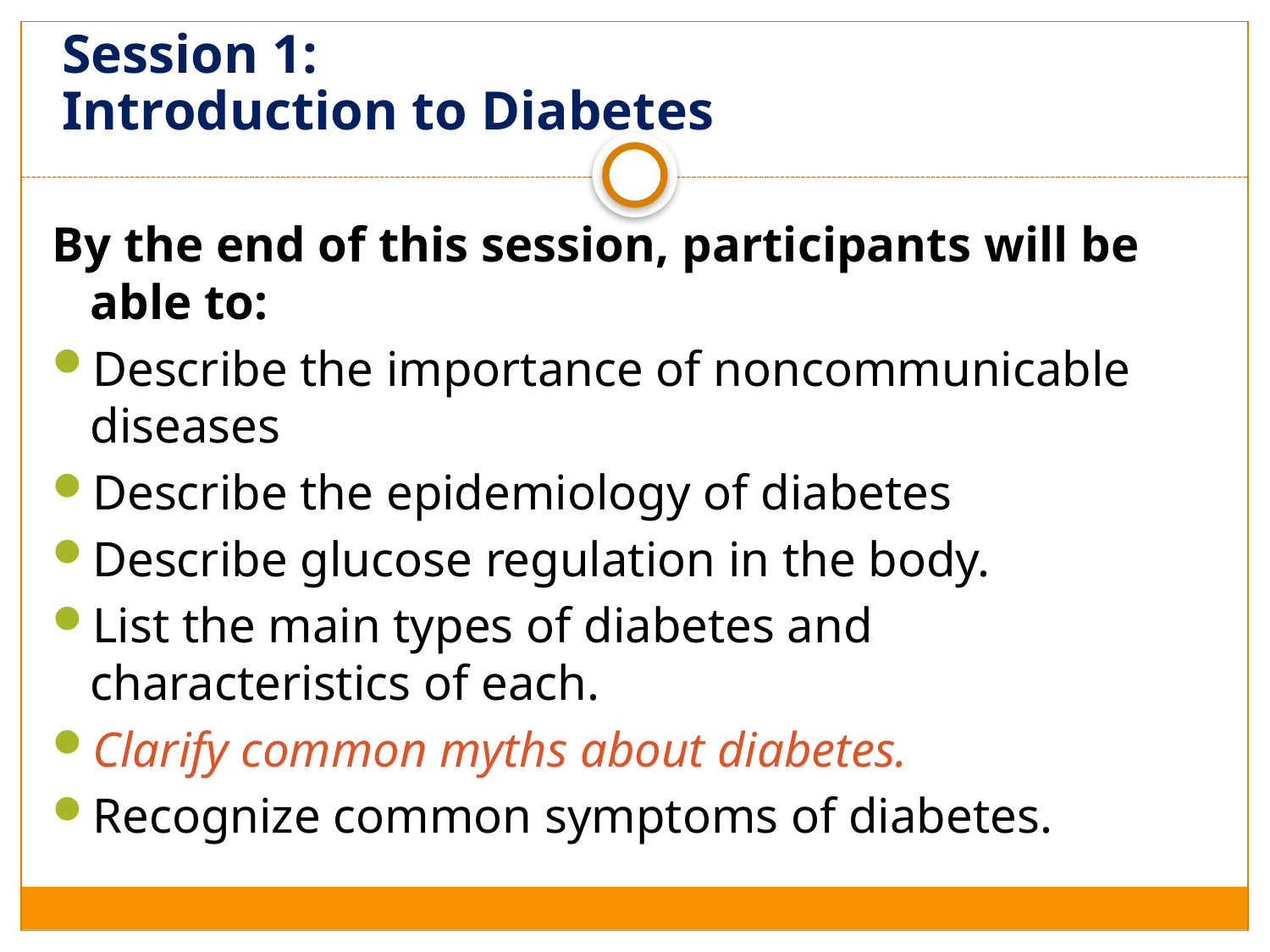

# Session 1: Introduction to Diabetes
By the end of this session, participants will be able to:
Describe the importance of noncommunicable diseases
Describe the epidemiology of diabetes
Describe glucose regulation in the body.
List the main types of diabetes and characteristics of each.
Clarify common myths about diabetes.
Recognize common symptoms of diabetes.

## Slide 23
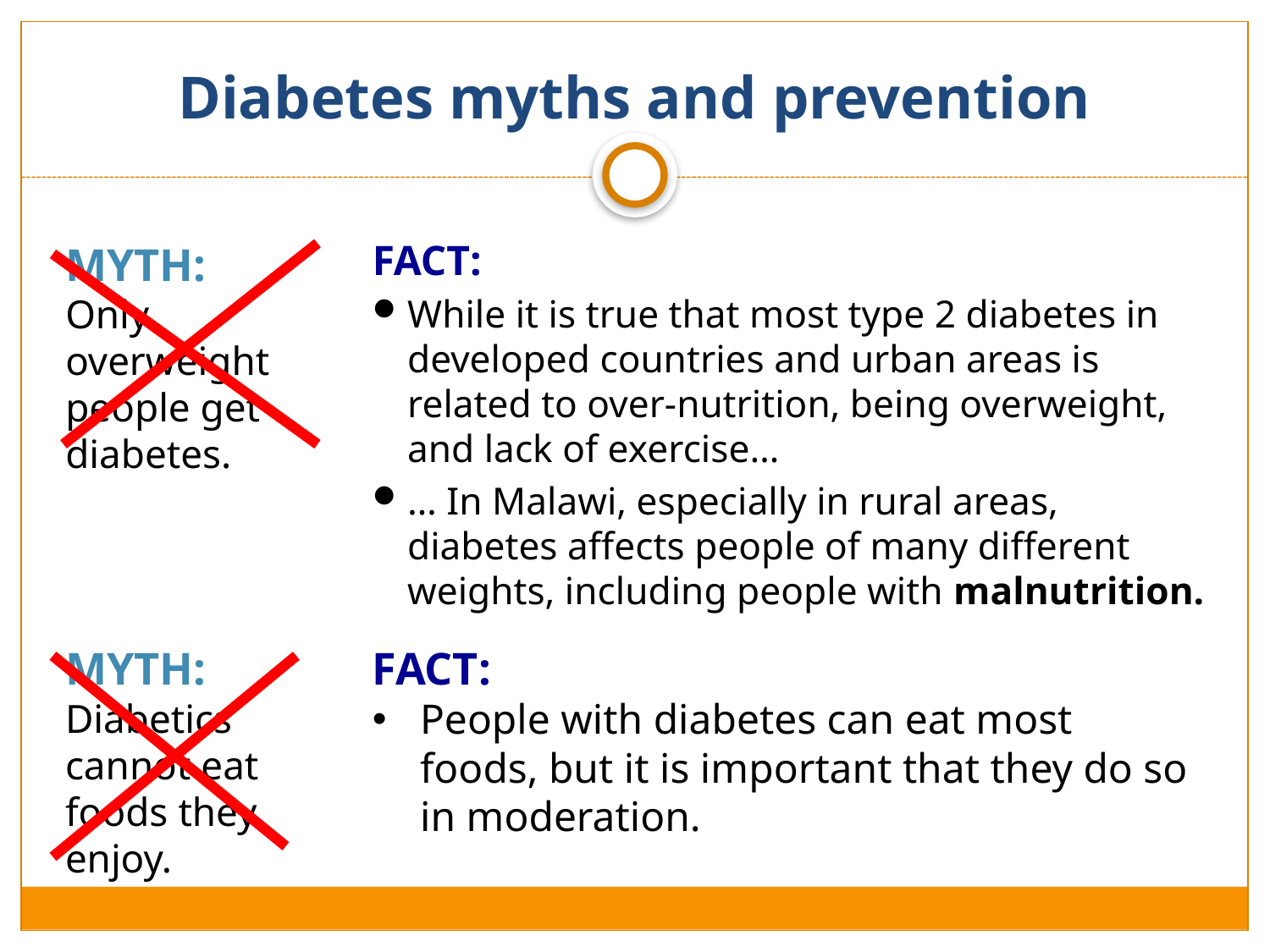

# Diabetes myths and prevention
FACT:
While it is true that most type 2 diabetes in developed countries and urban areas is related to over-nutrition, being overweight, and lack of exercise…
… In Malawi, especially in rural areas, diabetes affects people of many different weights, including people with malnutrition.
MYTH:
Only overweight people get diabetes.
FACT:
People with diabetes can eat most foods, but it is important that they do so in moderation.
MYTH:
Diabetics cannot eat foods they enjoy.

## Slide 24
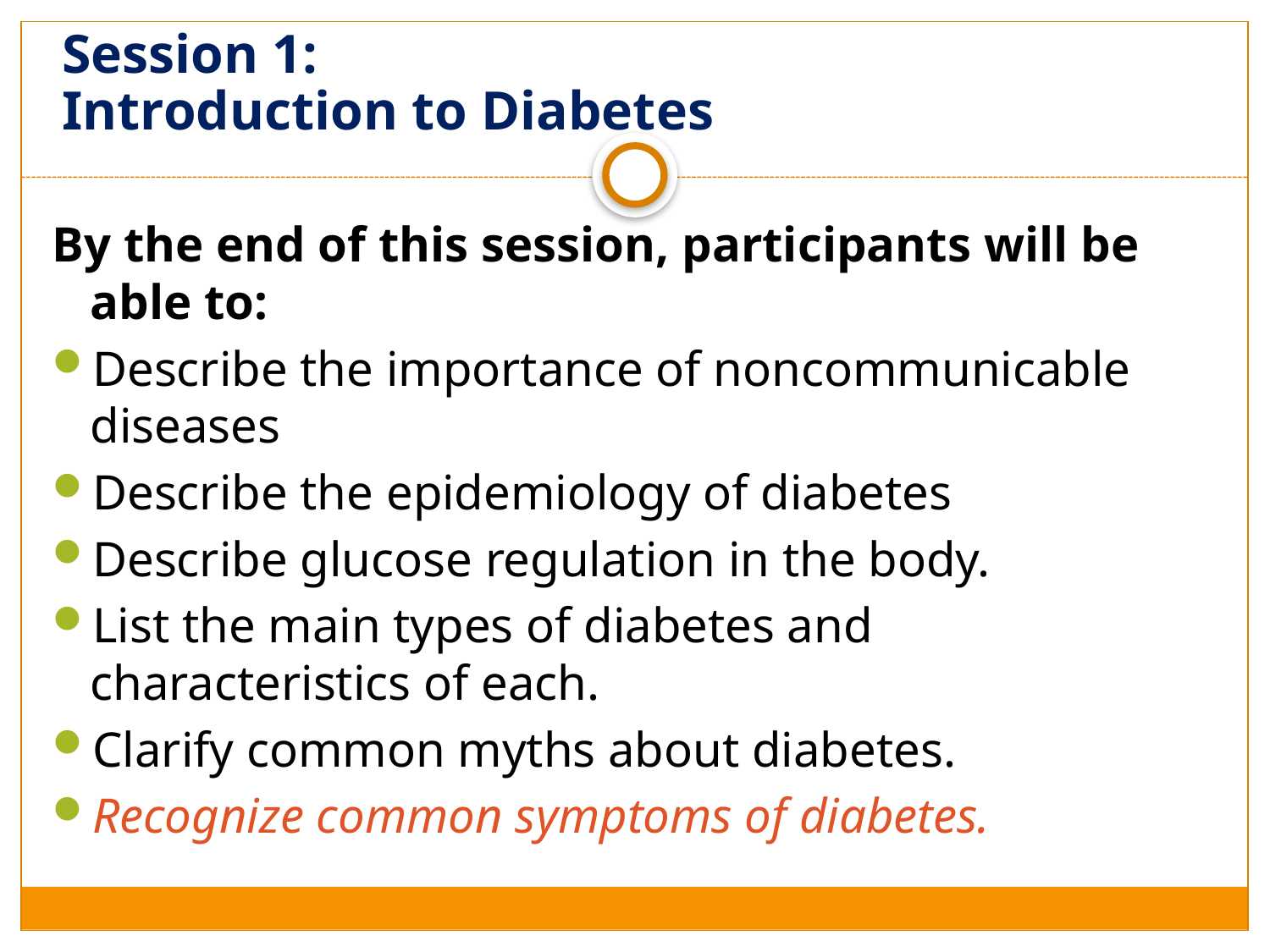

# Session 1: Introduction to Diabetes
By the end of this session, participants will be able to:
Describe the importance of noncommunicable diseases
Describe the epidemiology of diabetes
Describe glucose regulation in the body.
List the main types of diabetes and characteristics of each.
Clarify common myths about diabetes.
Recognize common symptoms of diabetes.

## Slide 25
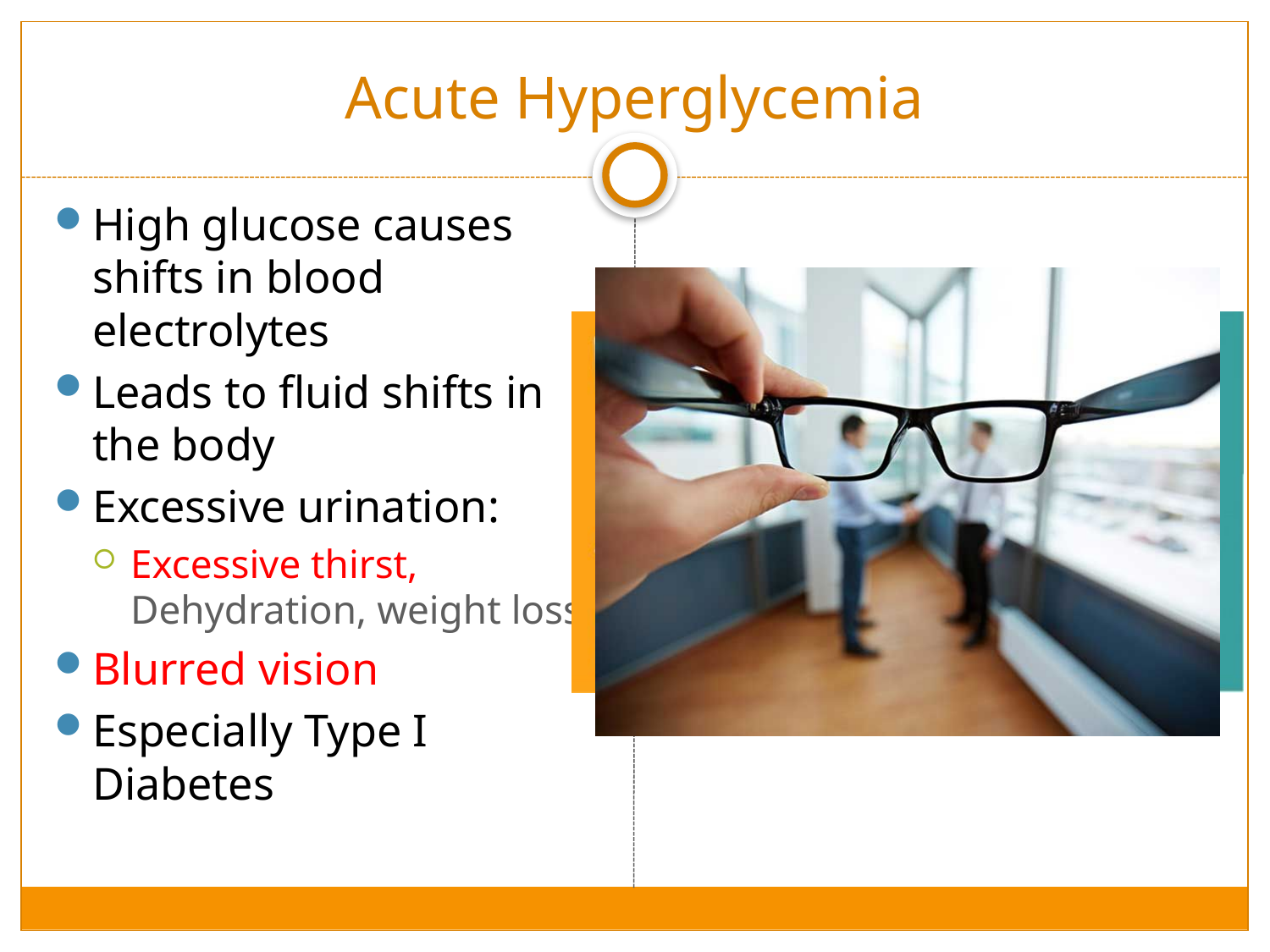

# Acute Hyperglycemia
High glucose causes shifts in blood electrolytes
Leads to fluid shifts in the body
Excessive urination:
Excessive thirst, Dehydration, weight loss
Blurred vision
Especially Type I Diabetes

## Slide 26
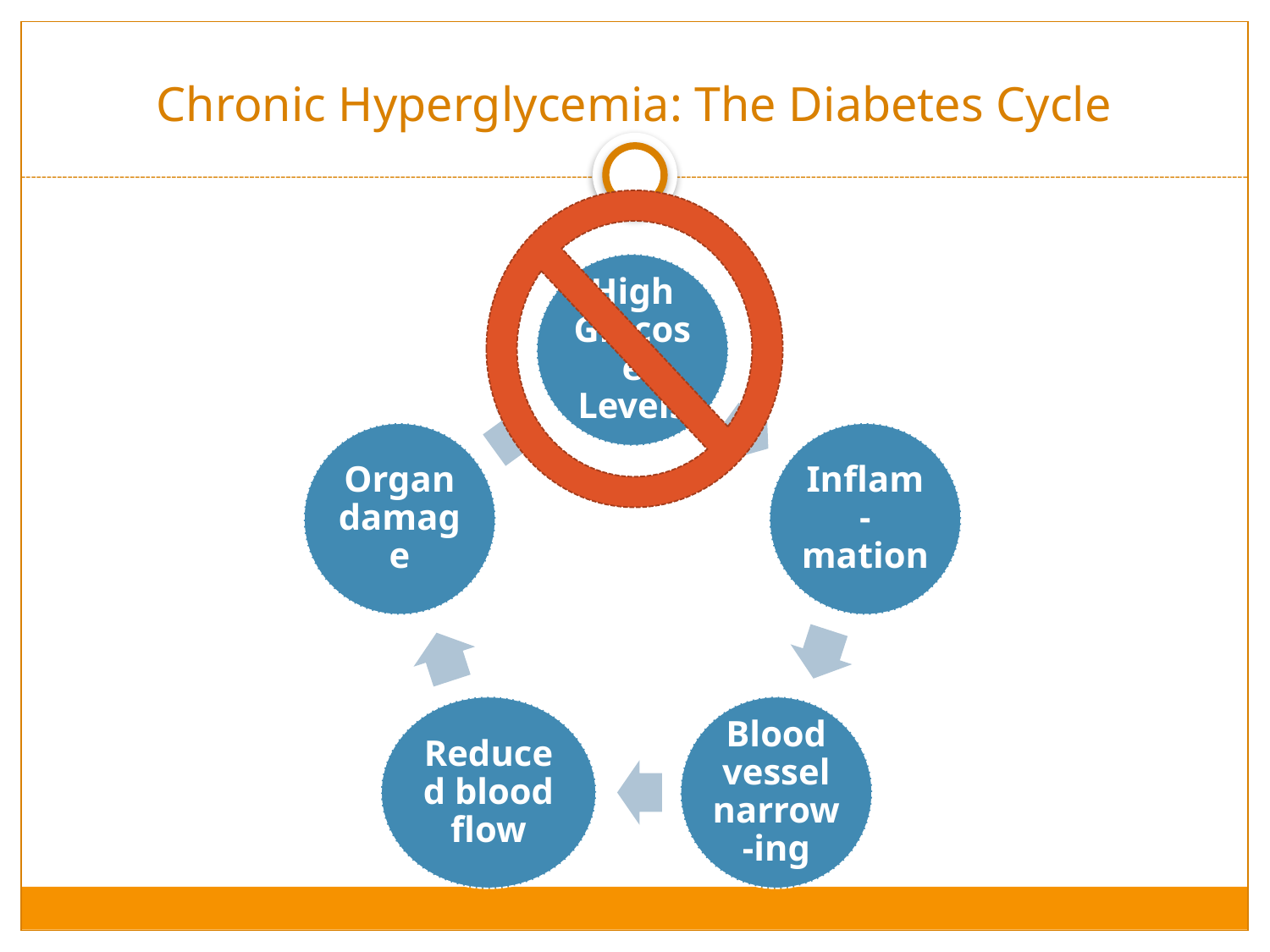

# Chronic Hyperglycemia: The Diabetes Cycle

## Slide 27
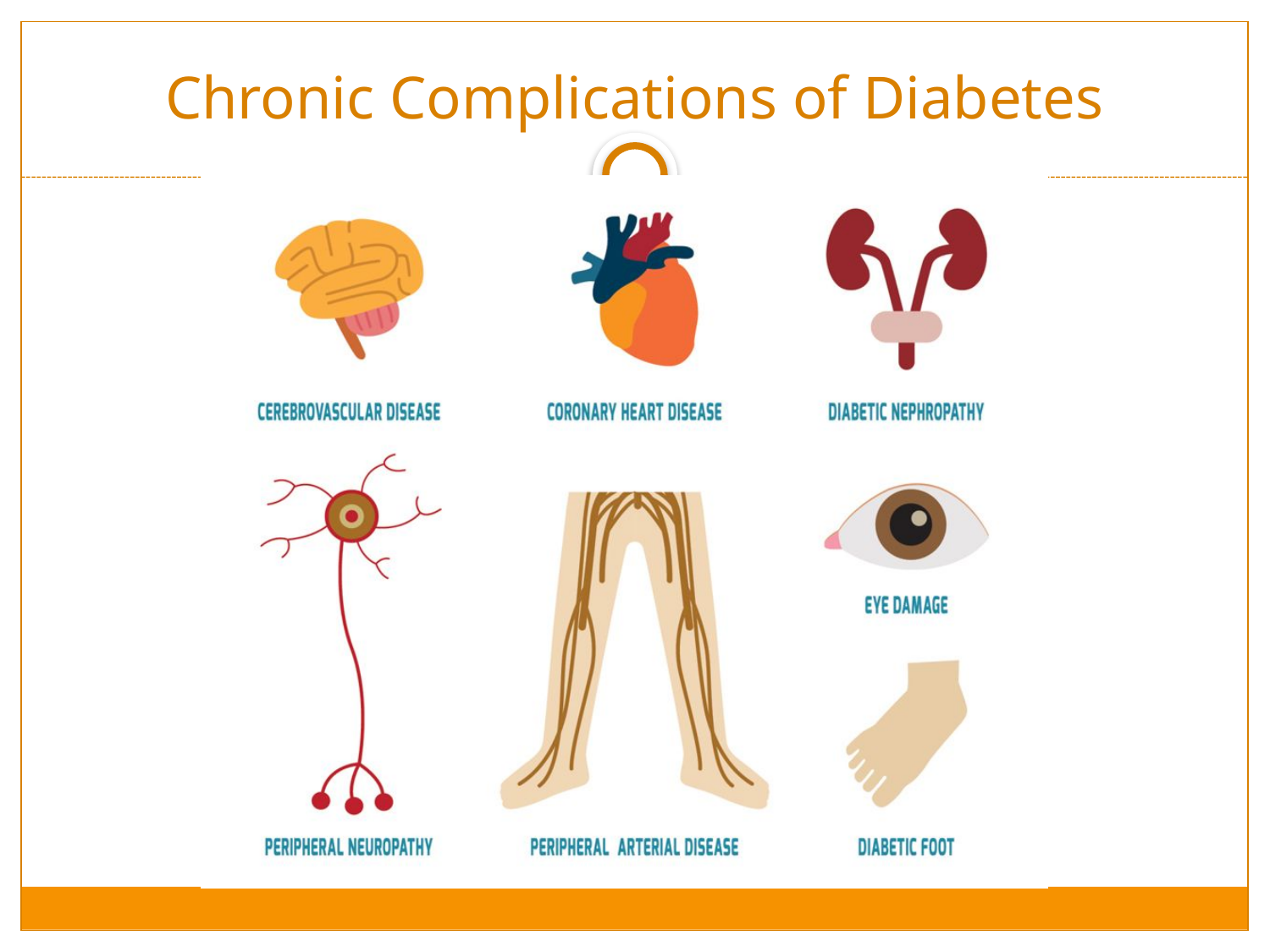

# Chronic Complications of Diabetes

## Slide 28
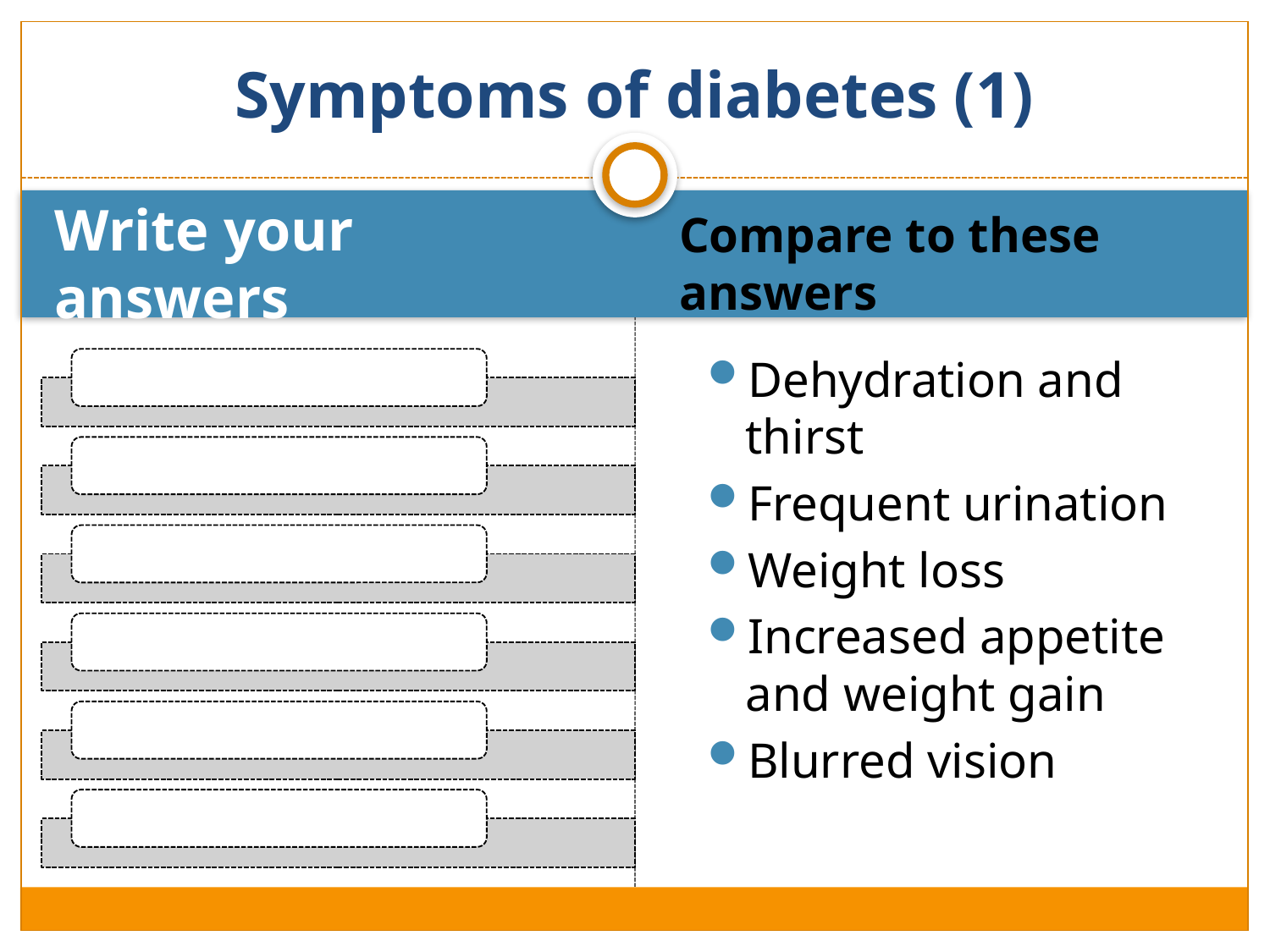

# Symptoms of diabetes (1)
Write your answers
Compare to these answers
Dehydration and thirst
Frequent urination
Weight loss
Increased appetite and weight gain
Blurred vision

## Slide 29
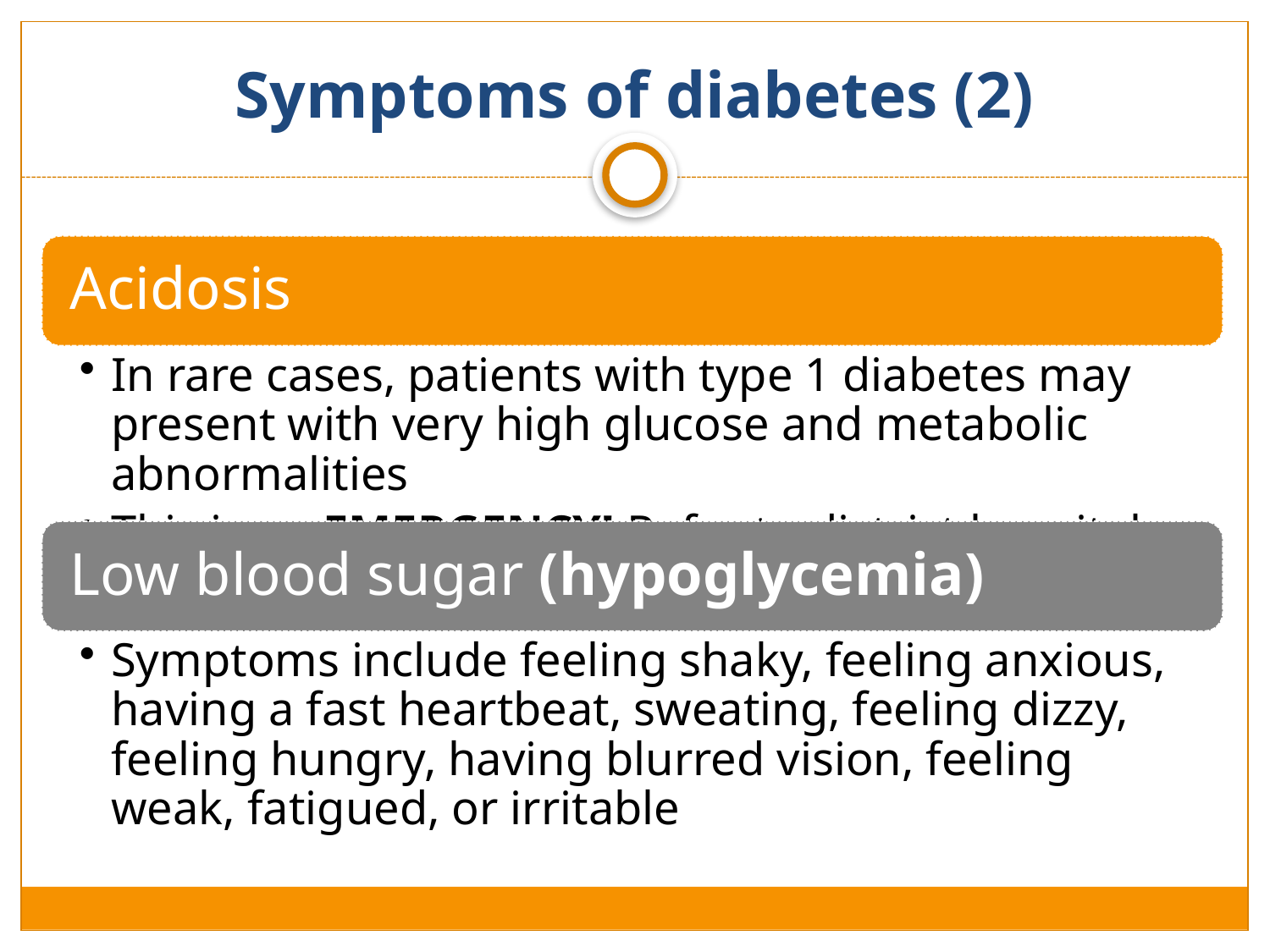

# Symptoms of diabetes (2)

## Slide 30
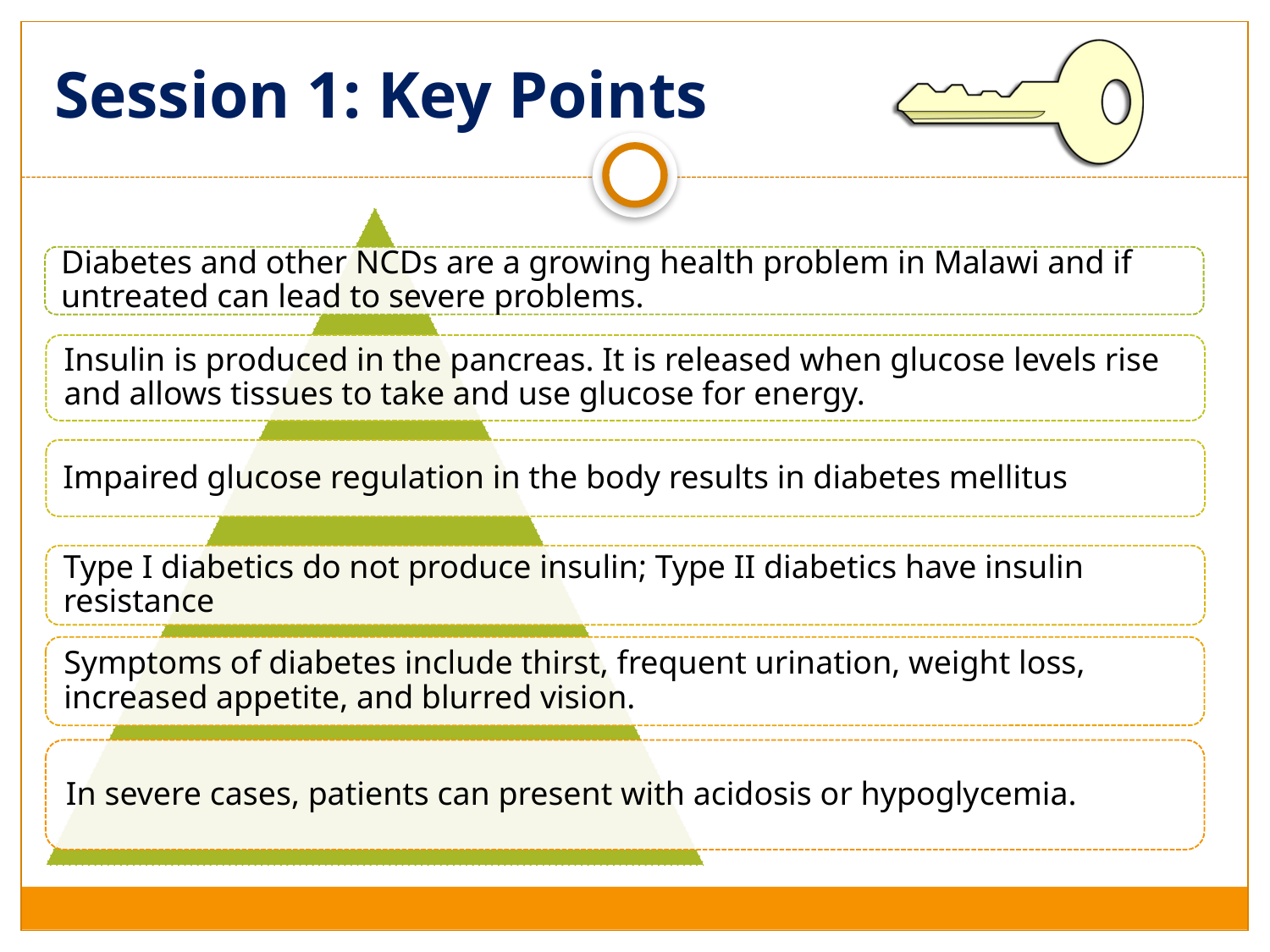

# Session 1: Key Points
