# Supplementary material for: Training Mid-Level Providers to Treat Severe Non-Communicable Diseases in Neno, Malawi through PEN-Plus Strategies
Source: Ann Glob Health. 2022 Aug 11;88(1):69. doi: 10.5334/aogh.3750 (PMC9389951; doi:10.5334/aogh.3750)
Supplement: Didactic Materials. — The supplementary materials contain a suggested didactic training schedule and the PowerPoint presentations used for PEN-Plus training in Neno, Malawi. These materials have been reviewed and accepted by the Malawi Ministry of Health for future PEN-Plus trainings in Malawi. [file agh-88-1-3750-s2.zip › Didactic_Materials/DM_Insulin.pptx]

## Slide 1
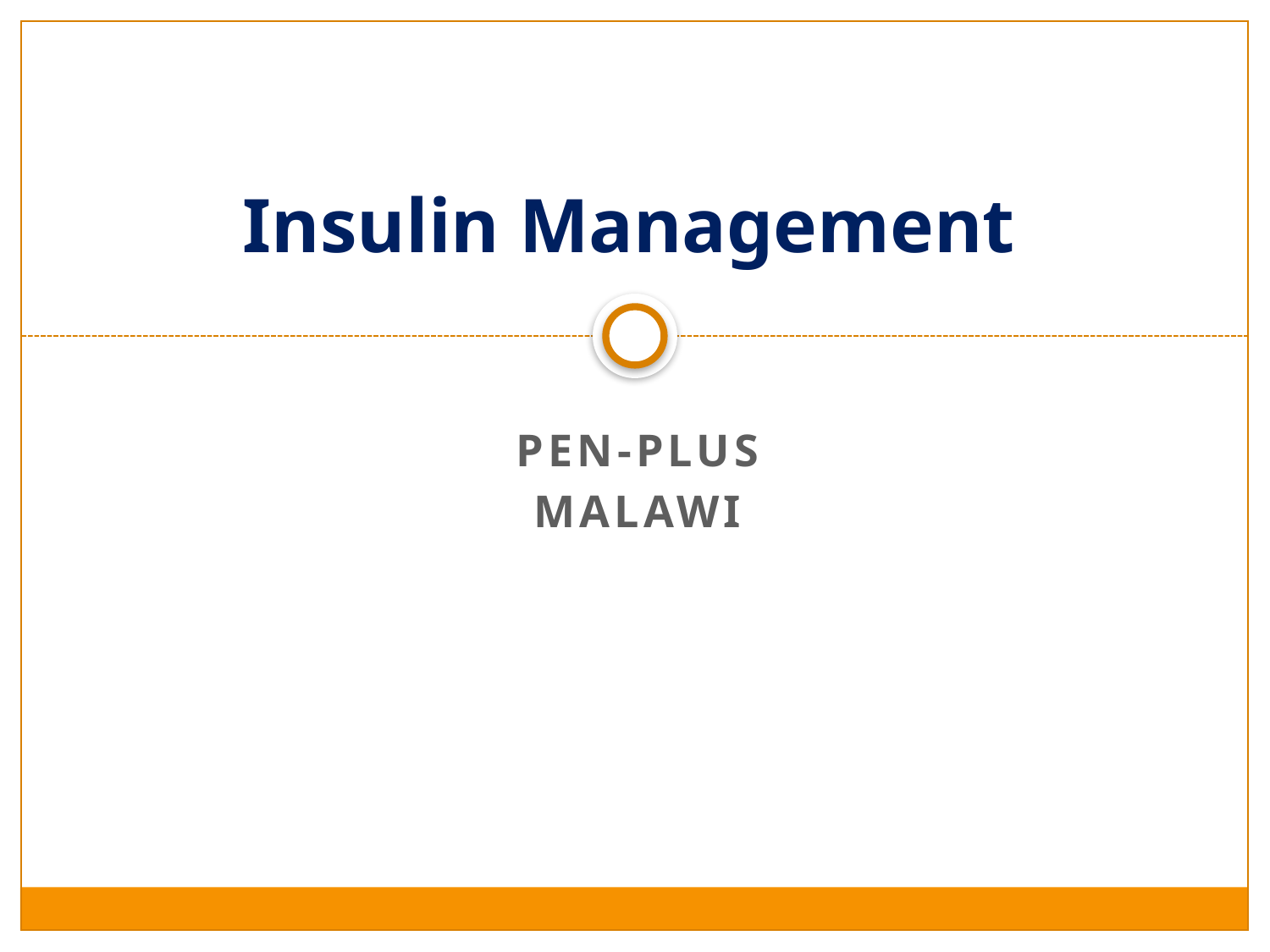

# Insulin Management
PEN-Plus
Malawi

## Slide 2
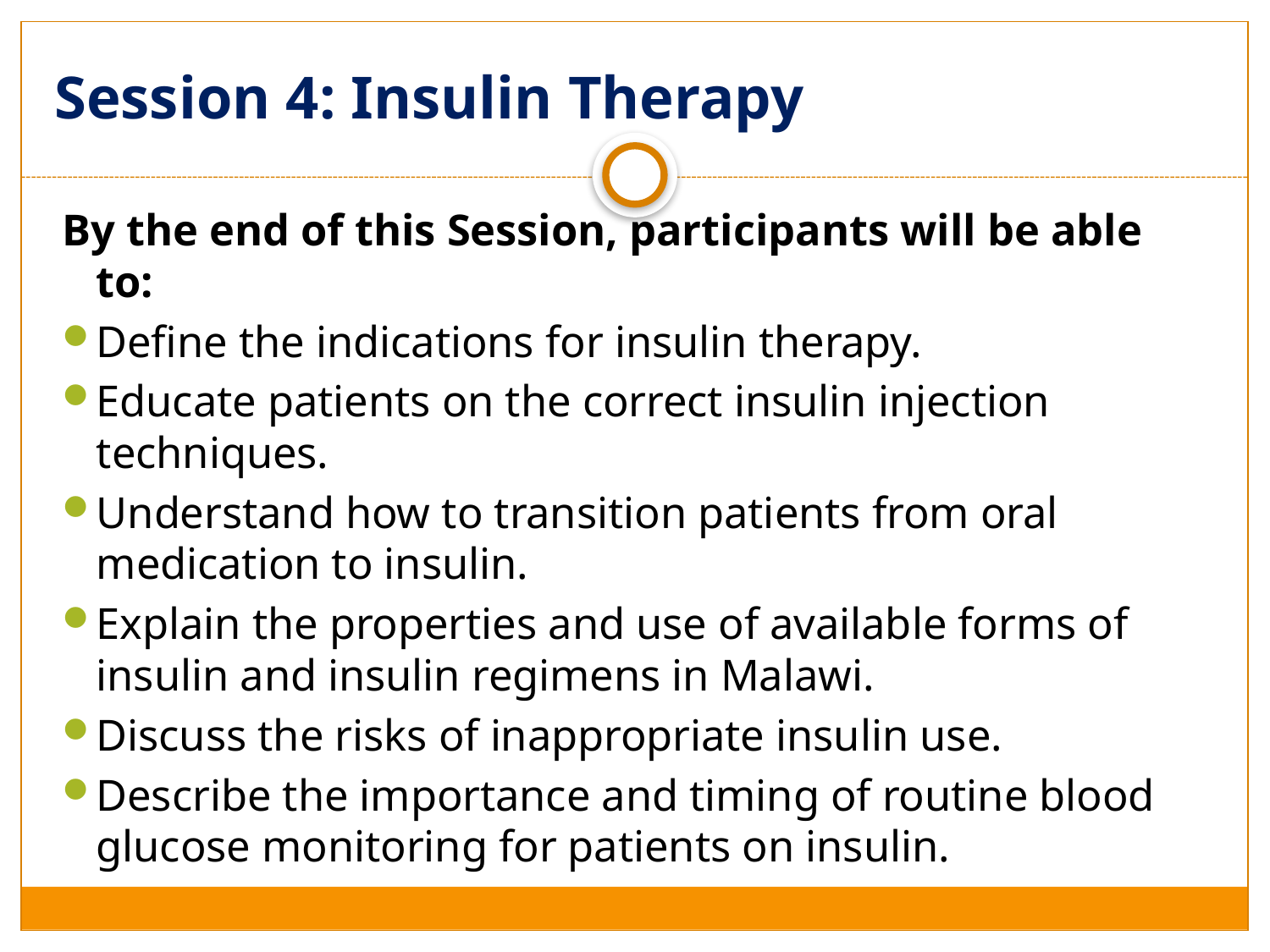

# Session 4: Insulin Therapy
By the end of this Session, participants will be able to:
Define the indications for insulin therapy.
Educate patients on the correct insulin injection techniques.
Understand how to transition patients from oral medication to insulin.
Explain the properties and use of available forms of insulin and insulin regimens in Malawi.
Discuss the risks of inappropriate insulin use.
Describe the importance and timing of routine blood glucose monitoring for patients on insulin.

## Slide 3
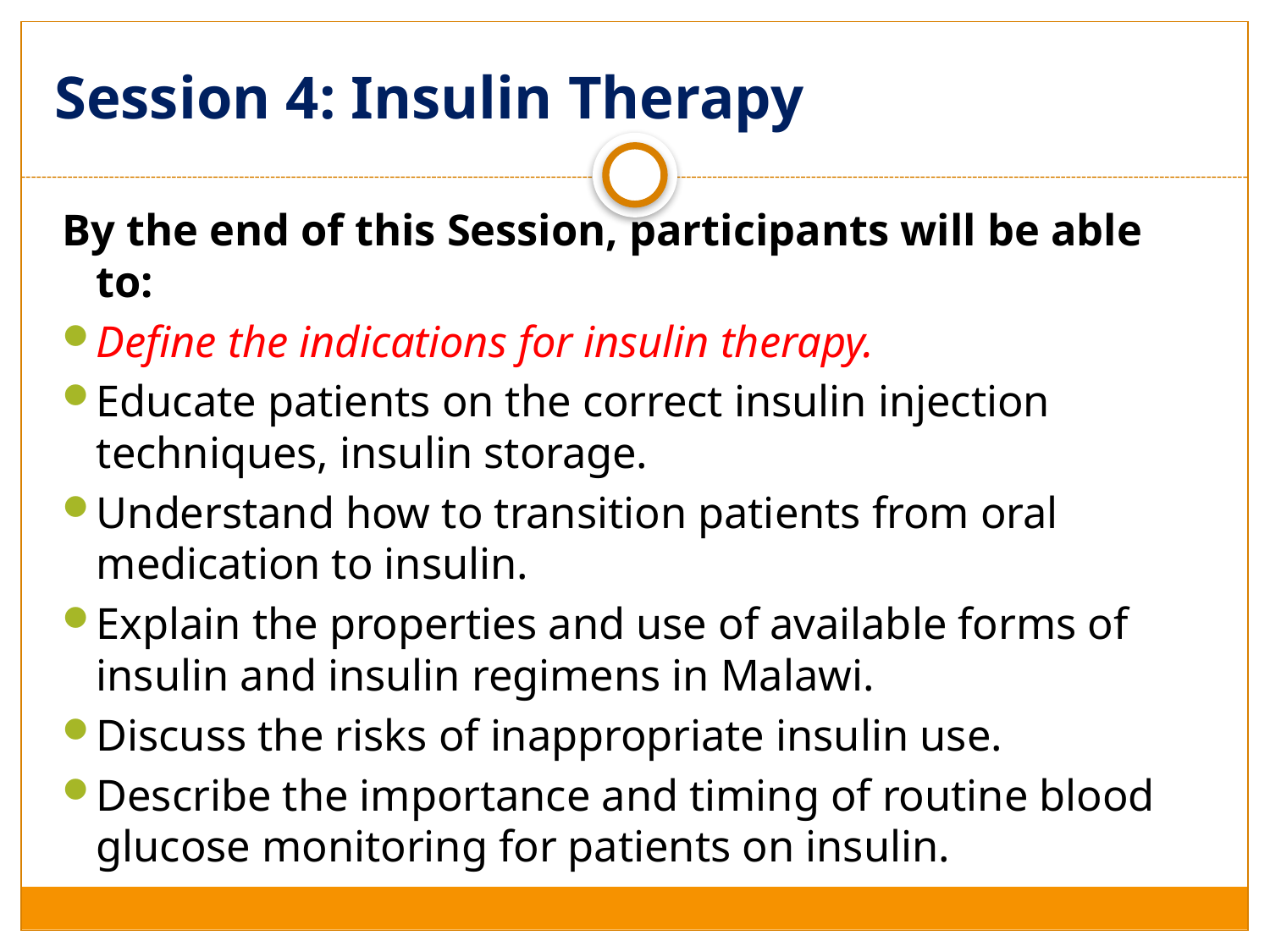

# Session 4: Insulin Therapy
By the end of this Session, participants will be able to:
Define the indications for insulin therapy.
Educate patients on the correct insulin injection techniques, insulin storage.
Understand how to transition patients from oral medication to insulin.
Explain the properties and use of available forms of insulin and insulin regimens in Malawi.
Discuss the risks of inappropriate insulin use.
Describe the importance and timing of routine blood glucose monitoring for patients on insulin.

## Slide 4
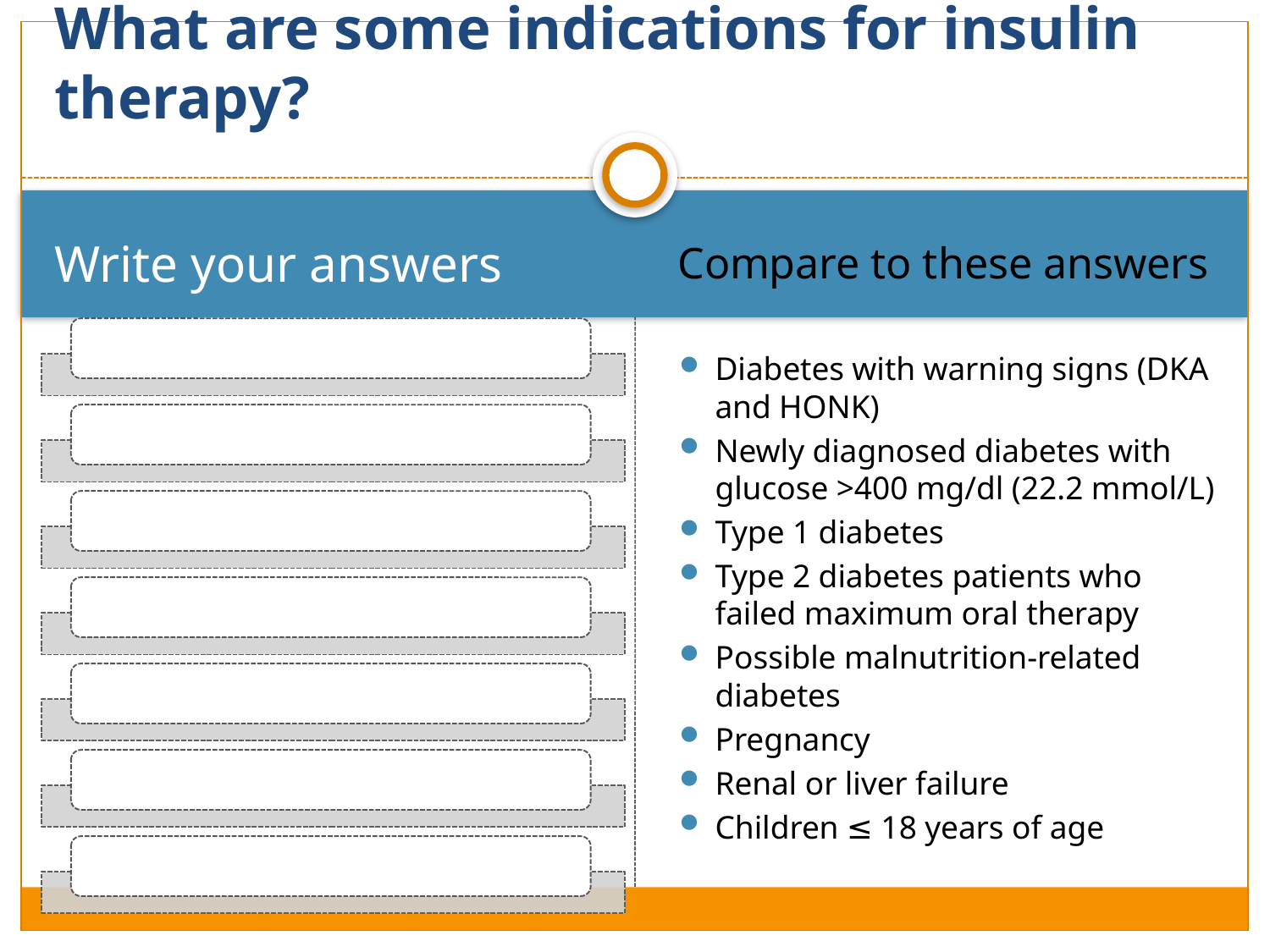

# What are some indications for insulin therapy?
Write your answers
Compare to these answers
Diabetes with warning signs (DKA and HONK)
Newly diagnosed diabetes with glucose >400 mg/dl (22.2 mmol/L)
Type 1 diabetes
Type 2 diabetes patients who failed maximum oral therapy
Possible malnutrition-related diabetes
Pregnancy
Renal or liver failure
Children ≤ 18 years of age

## Slide 5
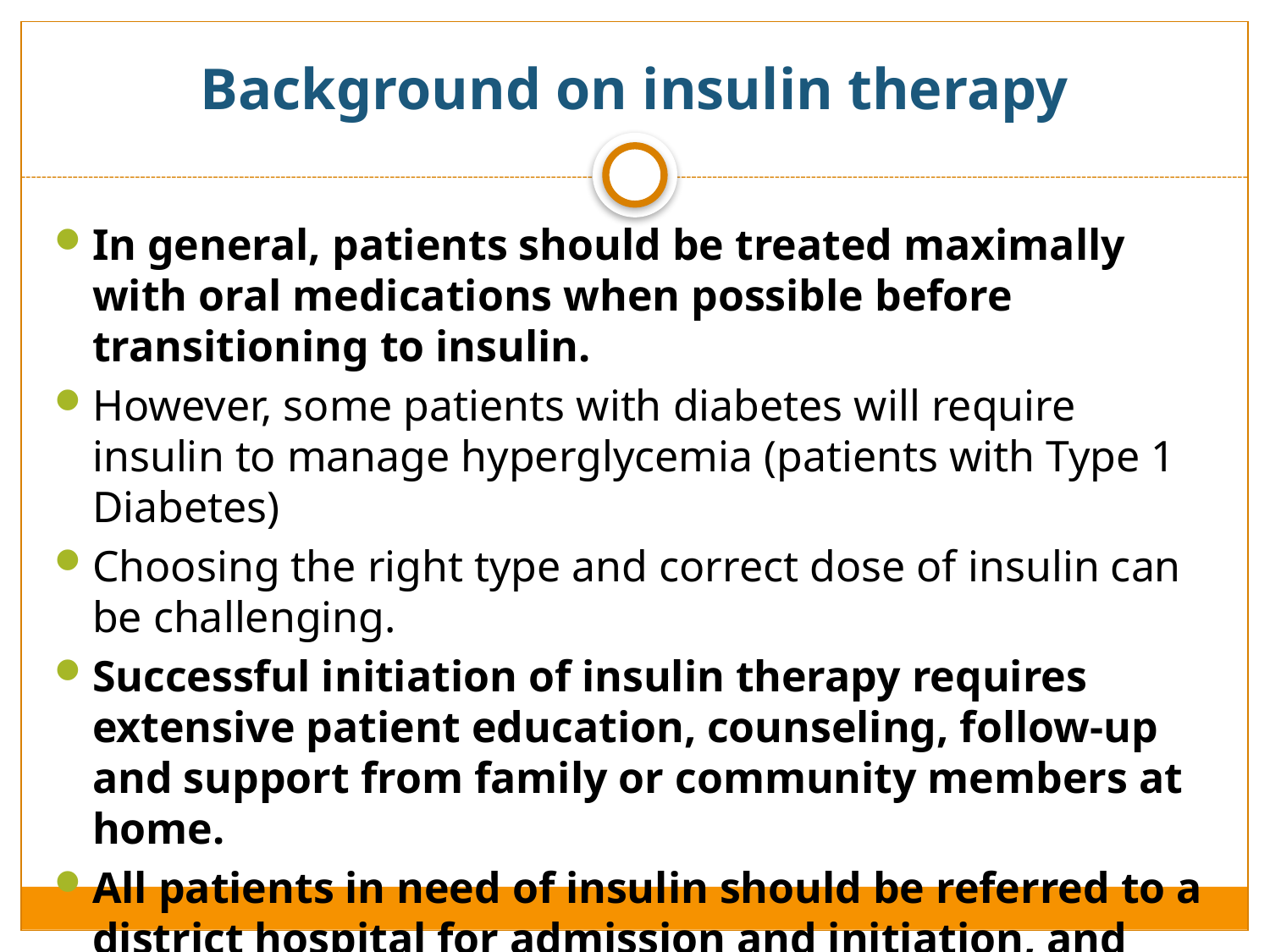

# Background on insulin therapy
In general, patients should be treated maximally with oral medications when possible before transitioning to insulin.
However, some patients with diabetes will require insulin to manage hyperglycemia (patients with Type 1 Diabetes)
Choosing the right type and correct dose of insulin can be challenging.
Successful initiation of insulin therapy requires extensive patient education, counseling, follow-up and support from family or community members at home.
All patients in need of insulin should be referred to a district hospital for admission and initiation, and patient education and will continue being followed in advanced NCD clinic .

## Slide 6
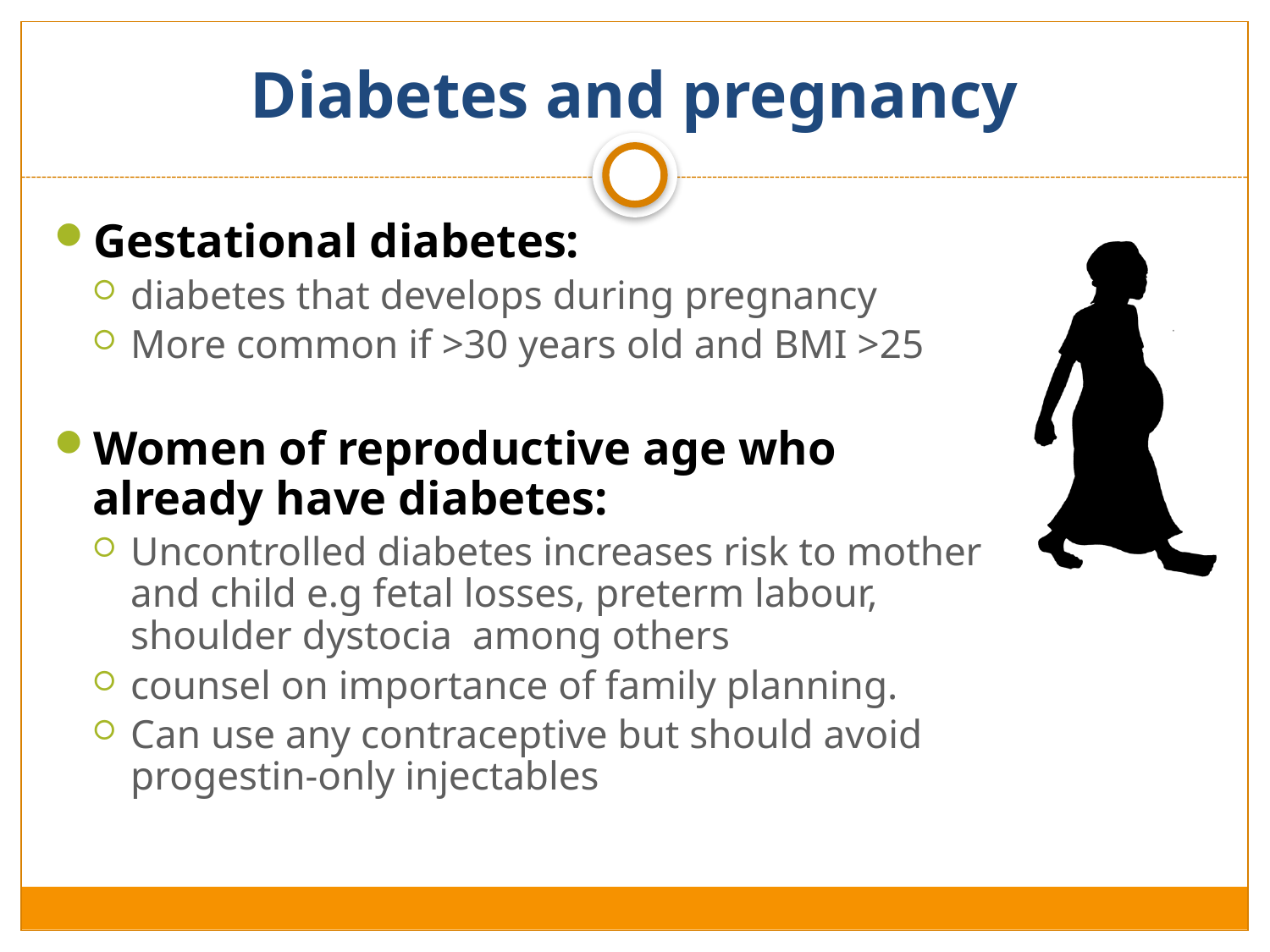

# Diabetes and pregnancy
Gestational diabetes:
diabetes that develops during pregnancy
More common if >30 years old and BMI >25
Women of reproductive age who already have diabetes:
Uncontrolled diabetes increases risk to mother and child e.g fetal losses, preterm labour, shoulder dystocia among others
counsel on importance of family planning.
Can use any contraceptive but should avoid progestin-only injectables

## Slide 7
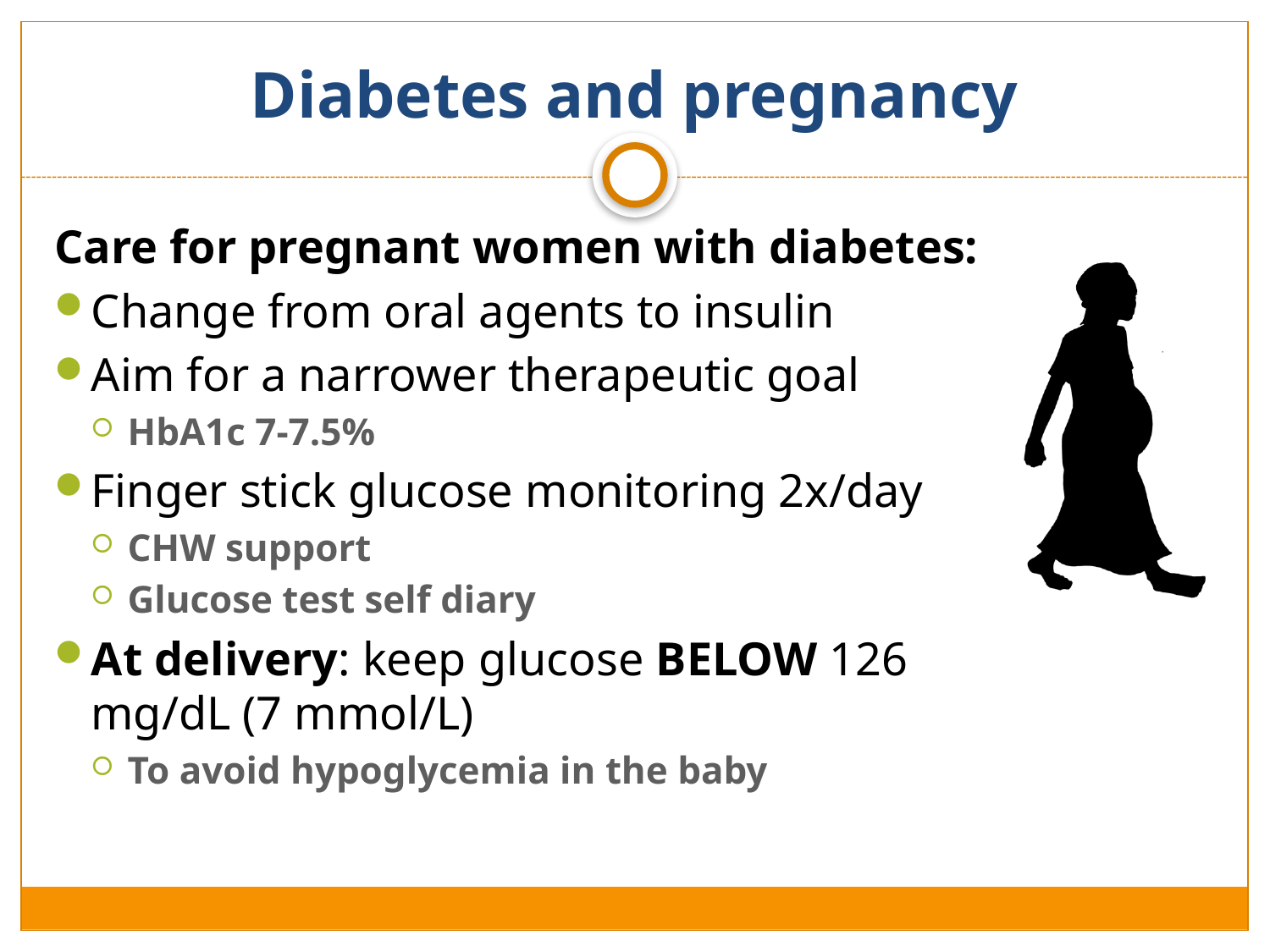

# Diabetes and pregnancy
Care for pregnant women with diabetes:
Change from oral agents to insulin
Aim for a narrower therapeutic goal
HbA1c 7-7.5%
Finger stick glucose monitoring 2x/day
CHW support
Glucose test self diary
At delivery: keep glucose BELOW 126 mg/dL (7 mmol/L)
To avoid hypoglycemia in the baby

## Slide 8
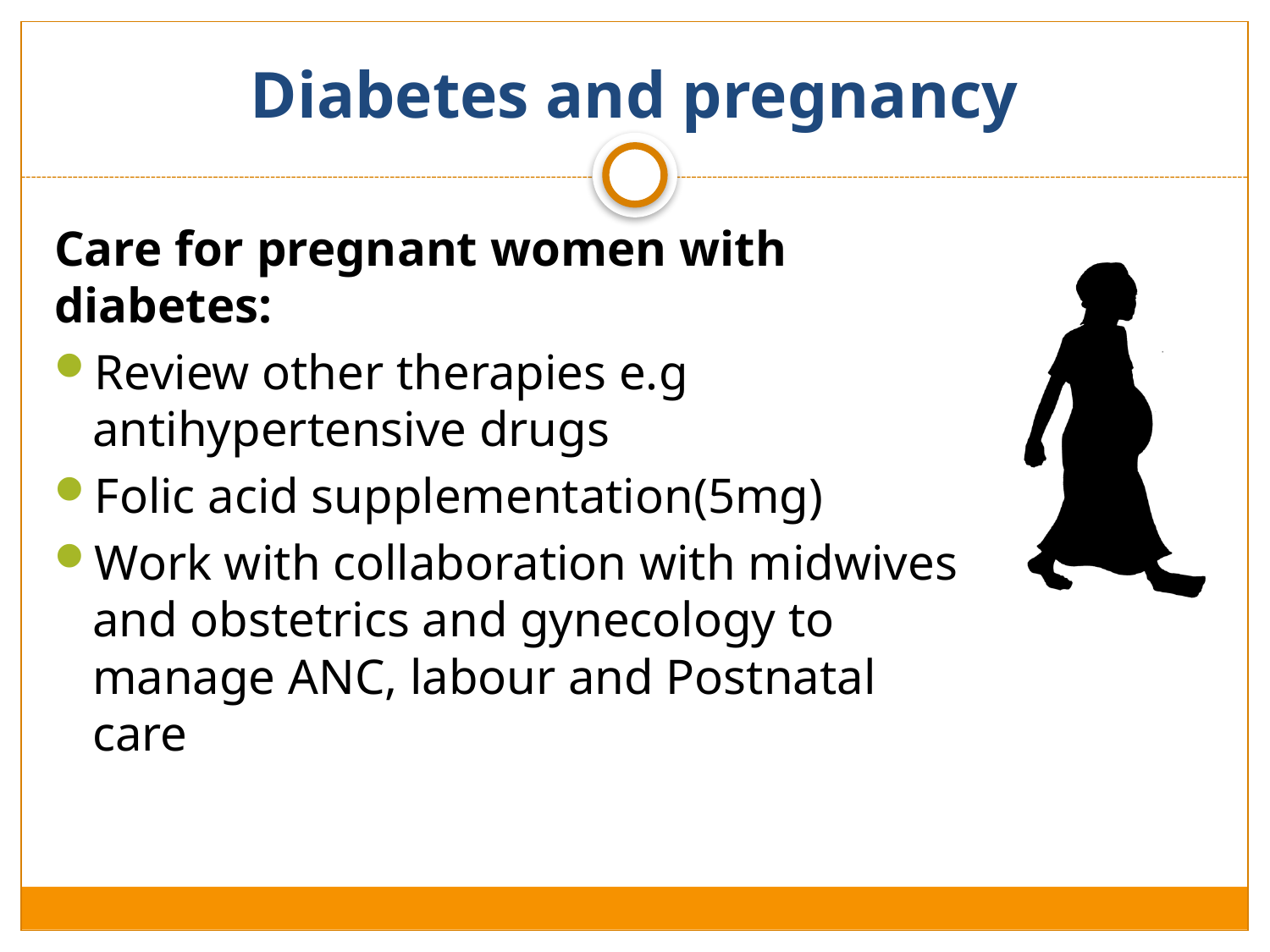

# Diabetes and pregnancy
Care for pregnant women with diabetes:
Review other therapies e.g antihypertensive drugs
Folic acid supplementation(5mg)
Work with collaboration with midwives and obstetrics and gynecology to manage ANC, labour and Postnatal care

## Slide 9
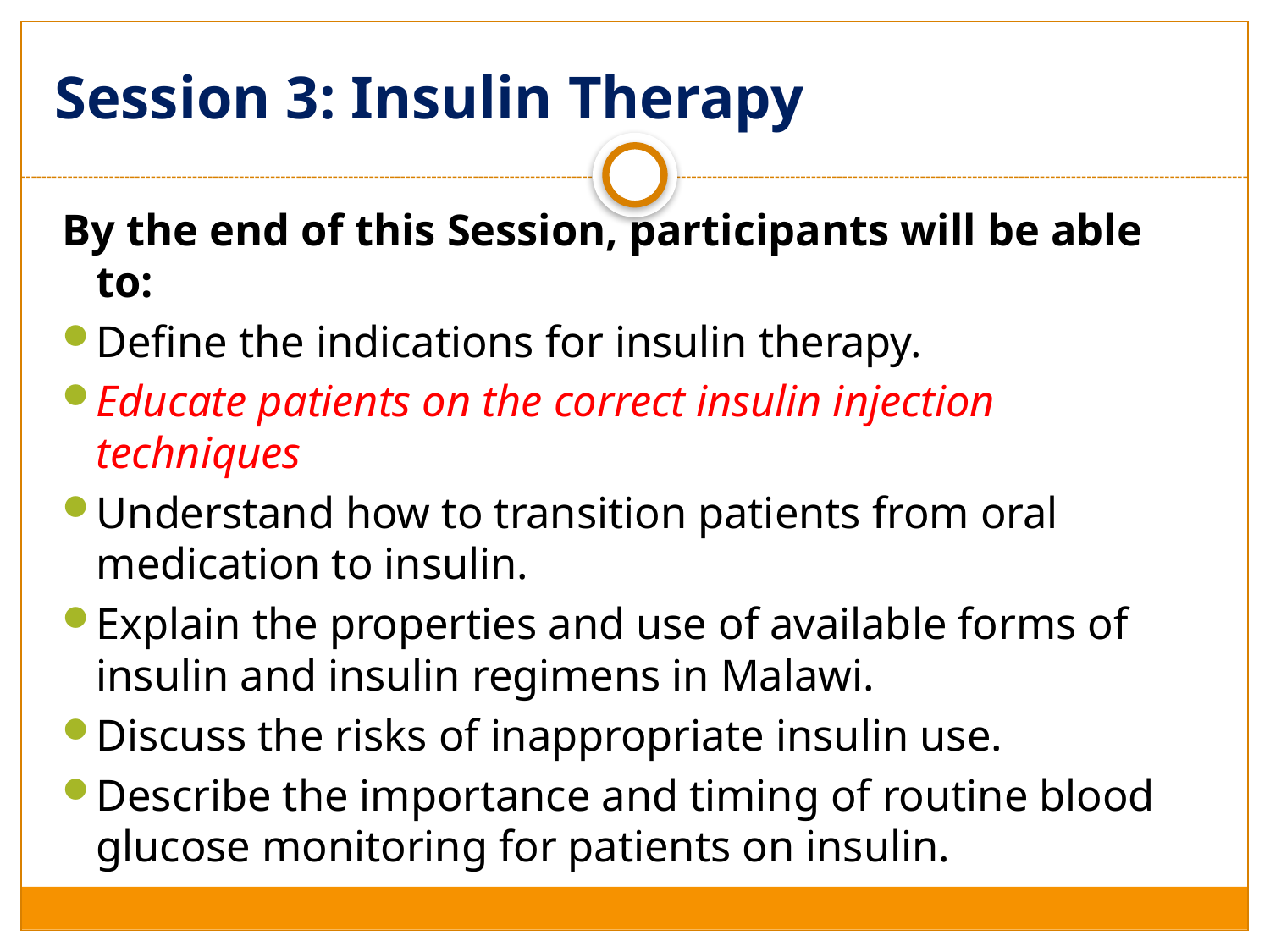

# Session 3: Insulin Therapy
By the end of this Session, participants will be able to:
Define the indications for insulin therapy.
Educate patients on the correct insulin injection techniques
Understand how to transition patients from oral medication to insulin.
Explain the properties and use of available forms of insulin and insulin regimens in Malawi.
Discuss the risks of inappropriate insulin use.
Describe the importance and timing of routine blood glucose monitoring for patients on insulin.

## Slide 10
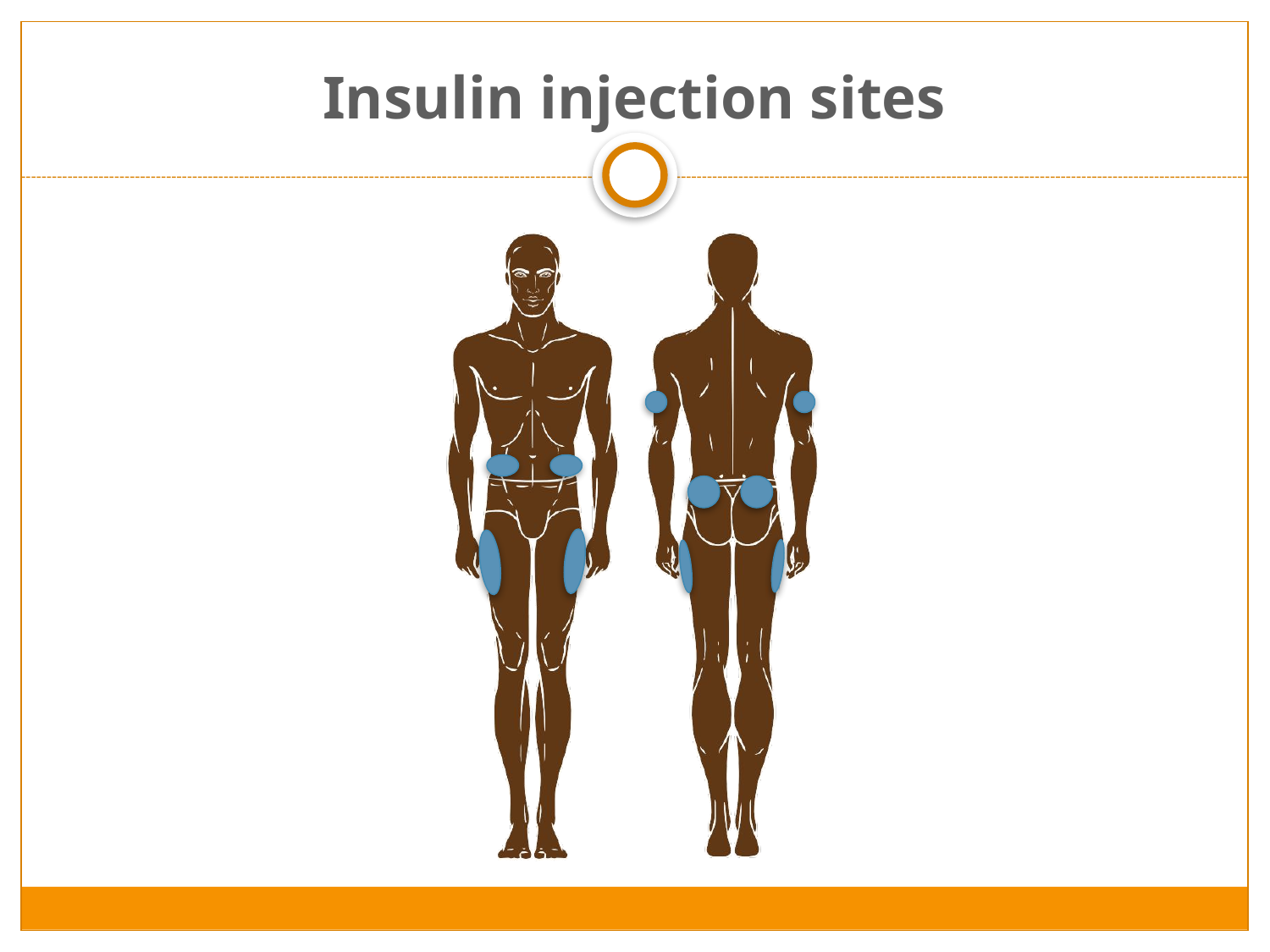

# Insulin injection sites

## Slide 11
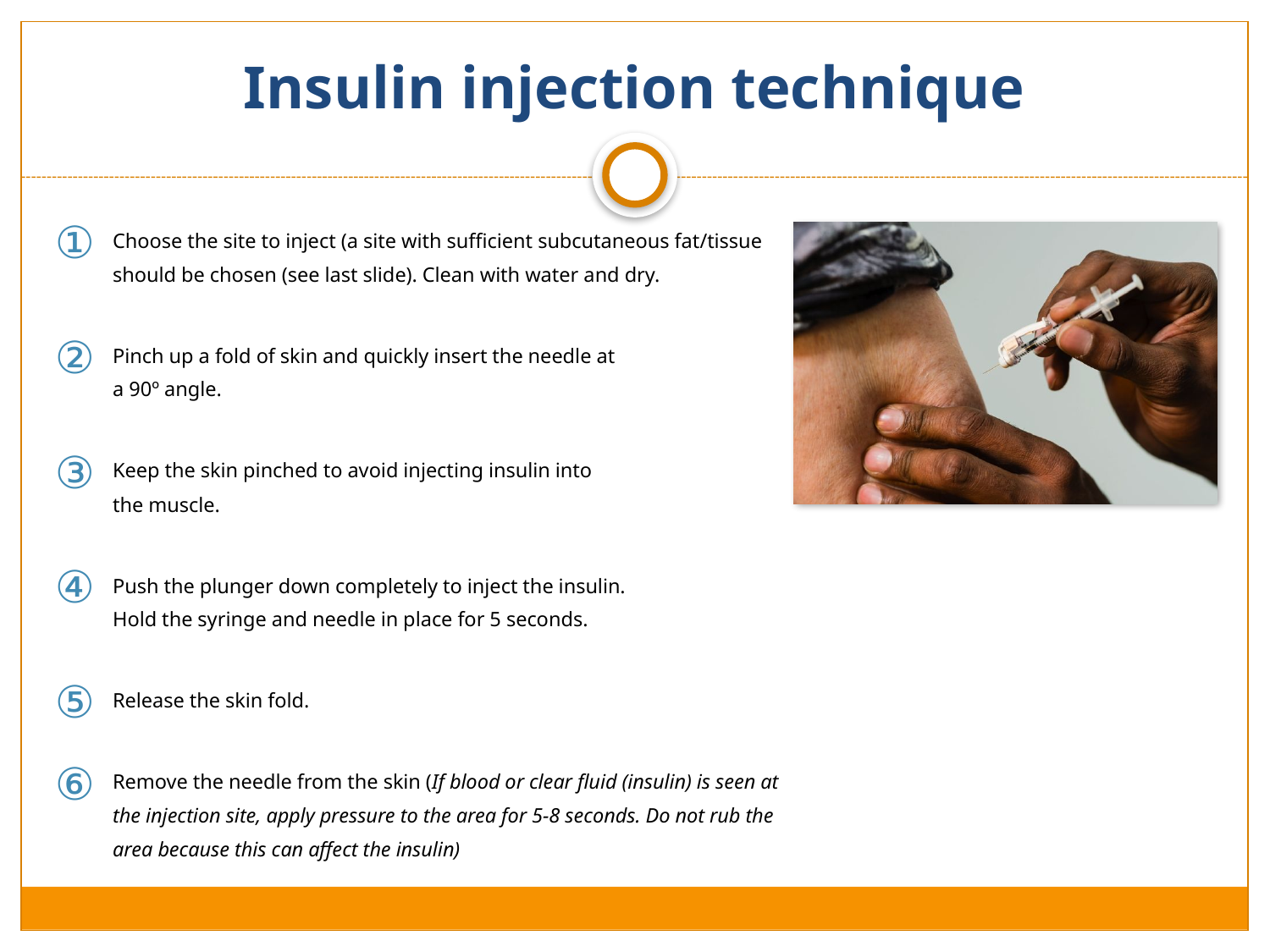

# Insulin injection technique
Choose the site to inject (a site with sufficient subcutaneous fat/tissue should be chosen (see last slide). Clean with water and dry.
Pinch up a fold of skin and quickly insert the needle at
a 90º angle.
Keep the skin pinched to avoid injecting insulin into
the muscle.
Push the plunger down completely to inject the insulin.
Hold the syringe and needle in place for 5 seconds.
Release the skin fold.
Remove the needle from the skin (If blood or clear fluid (insulin) is seen at the injection site, apply pressure to the area for 5-8 seconds. Do not rub the area because this can affect the insulin)

## Slide 12
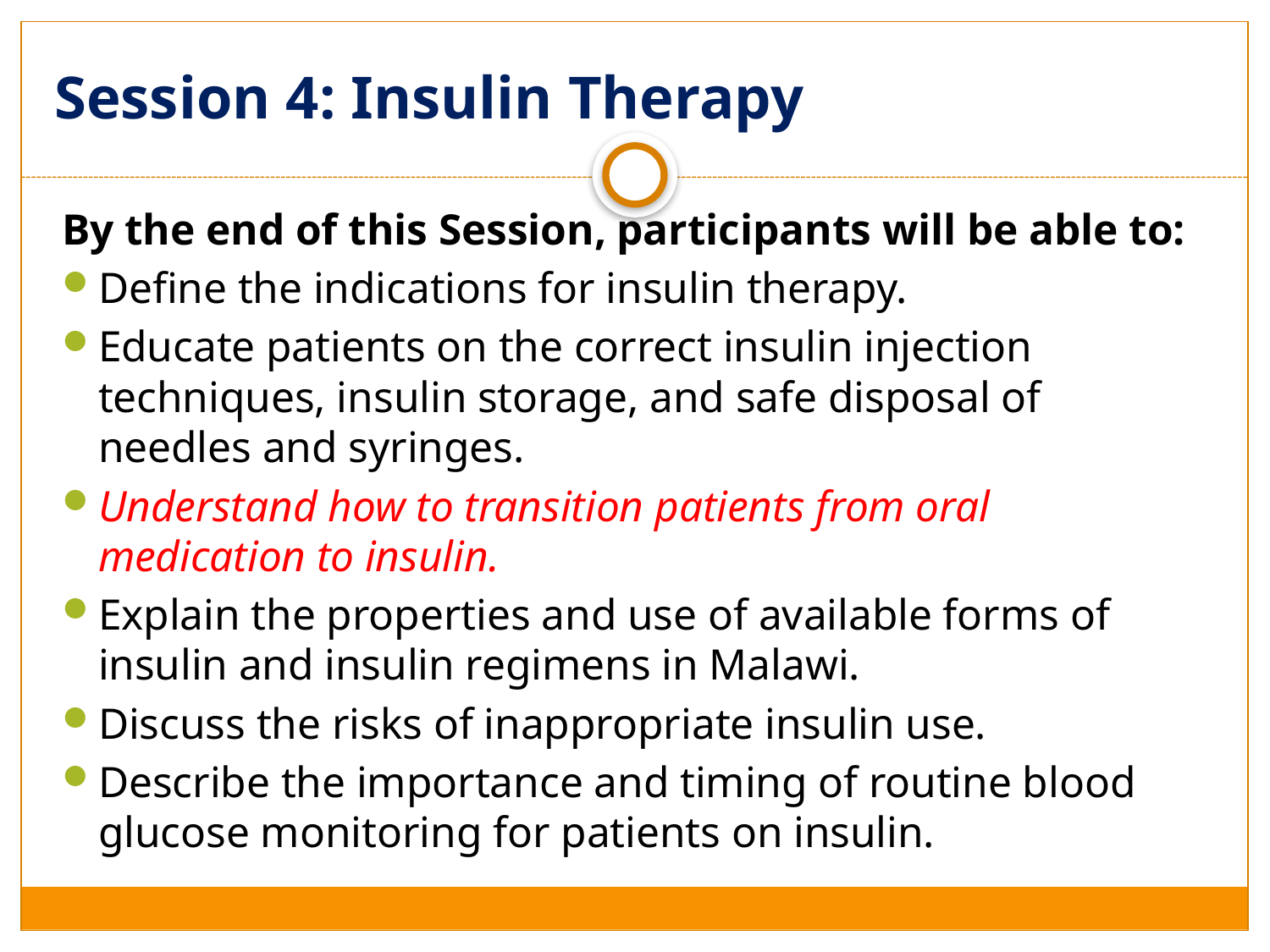

# Session 4: Insulin Therapy
By the end of this Session, participants will be able to:
Define the indications for insulin therapy.
Educate patients on the correct insulin injection techniques, insulin storage, and safe disposal of needles and syringes.
Understand how to transition patients from oral medication to insulin.
Explain the properties and use of available forms of insulin and insulin regimens in Malawi.
Discuss the risks of inappropriate insulin use.
Describe the importance and timing of routine blood glucose monitoring for patients on insulin.

## Slide 13
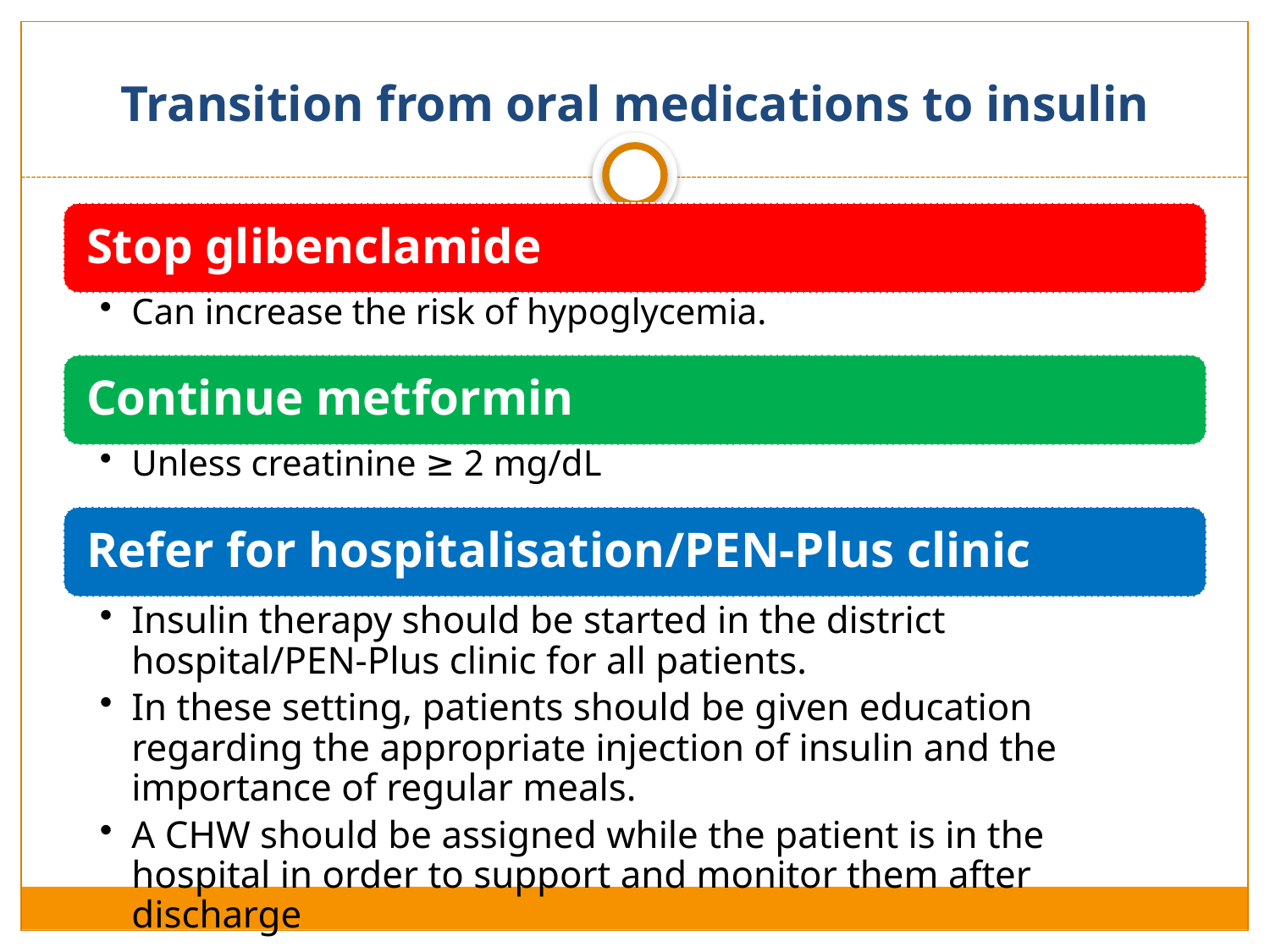

# Transition from oral medications to insulin

## Slide 14
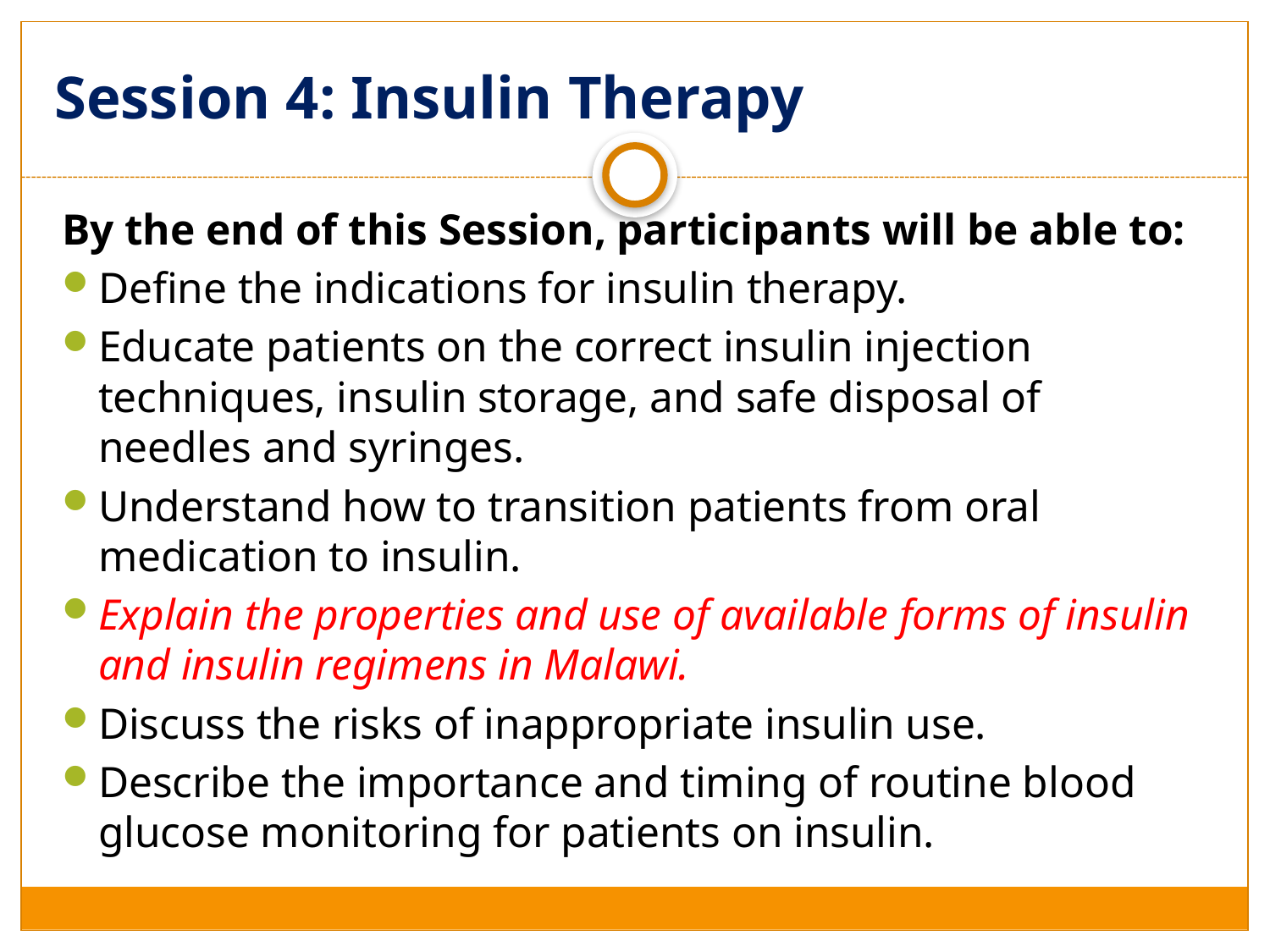

# Session 4: Insulin Therapy
By the end of this Session, participants will be able to:
Define the indications for insulin therapy.
Educate patients on the correct insulin injection techniques, insulin storage, and safe disposal of needles and syringes.
Understand how to transition patients from oral medication to insulin.
Explain the properties and use of available forms of insulin and insulin regimens in Malawi.
Discuss the risks of inappropriate insulin use.
Describe the importance and timing of routine blood glucose monitoring for patients on insulin.

## Slide 15
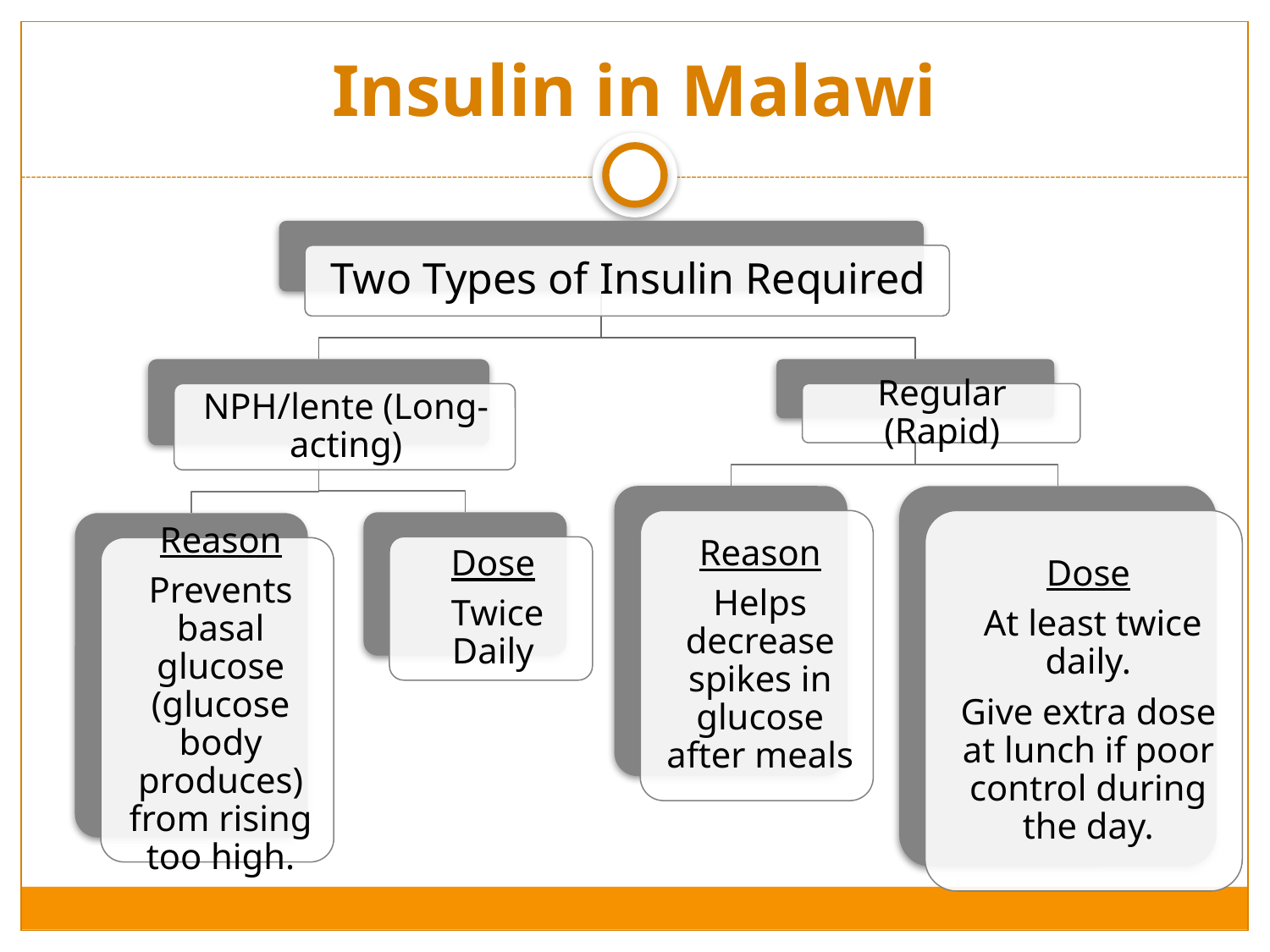

# Insulin in Malawi

## Slide 16
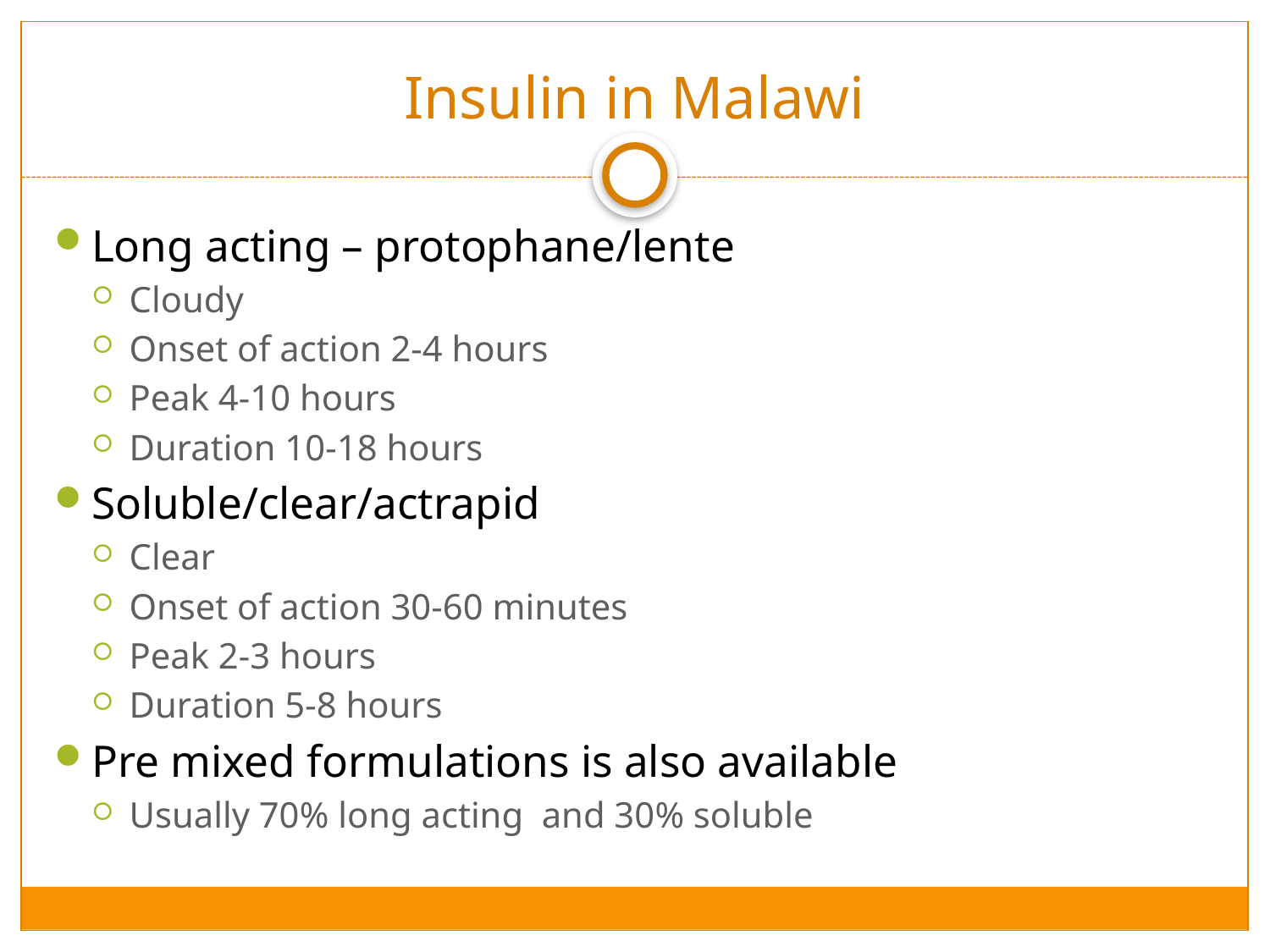

# Insulin in Malawi
Long acting – protophane/lente
Cloudy
Onset of action 2-4 hours
Peak 4-10 hours
Duration 10-18 hours
Soluble/clear/actrapid
Clear
Onset of action 30-60 minutes
Peak 2-3 hours
Duration 5-8 hours
Pre mixed formulations is also available
Usually 70% long acting and 30% soluble

## Slide 17
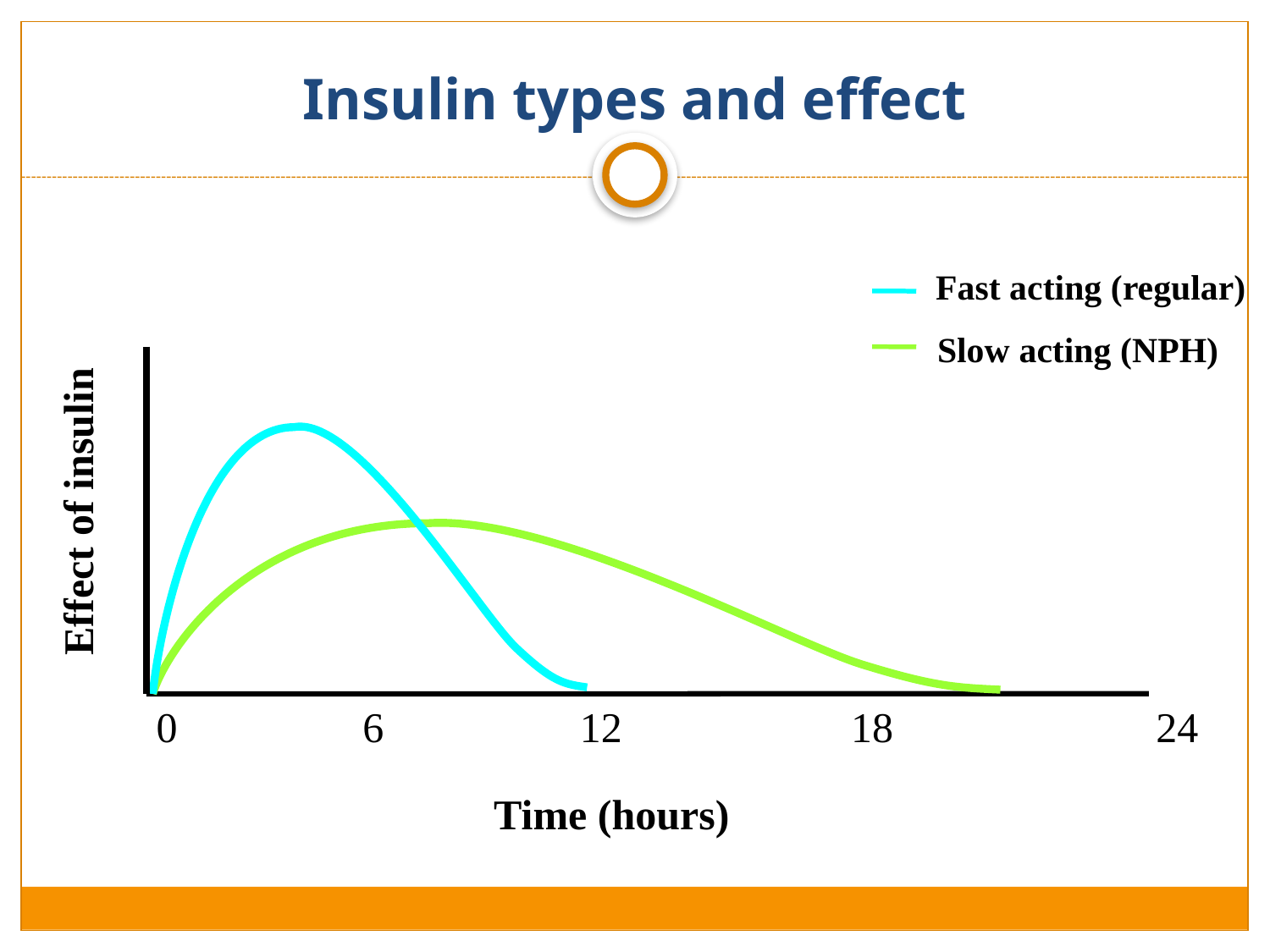

# Insulin types and effect
Fast acting (regular)
Slow acting (NPH)
Effect of insulin
0
6
12
18
24
Time (hours)

## Slide 18
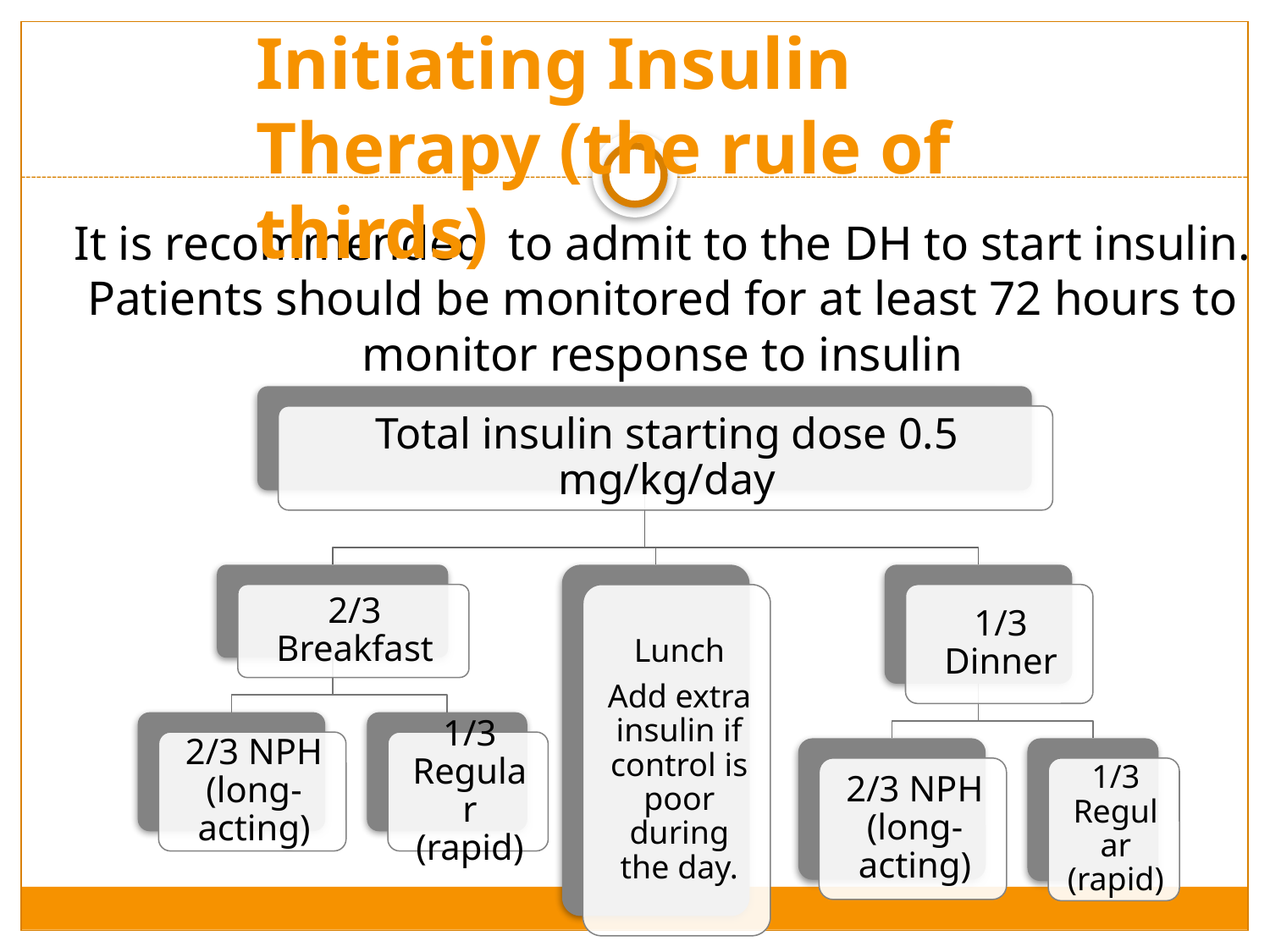

Initiating Insulin Therapy (the rule of thirds)
# It is recommended to admit to the DH to start insulin. Patients should be monitored for at least 72 hours to monitor response to insulin

## Slide 19
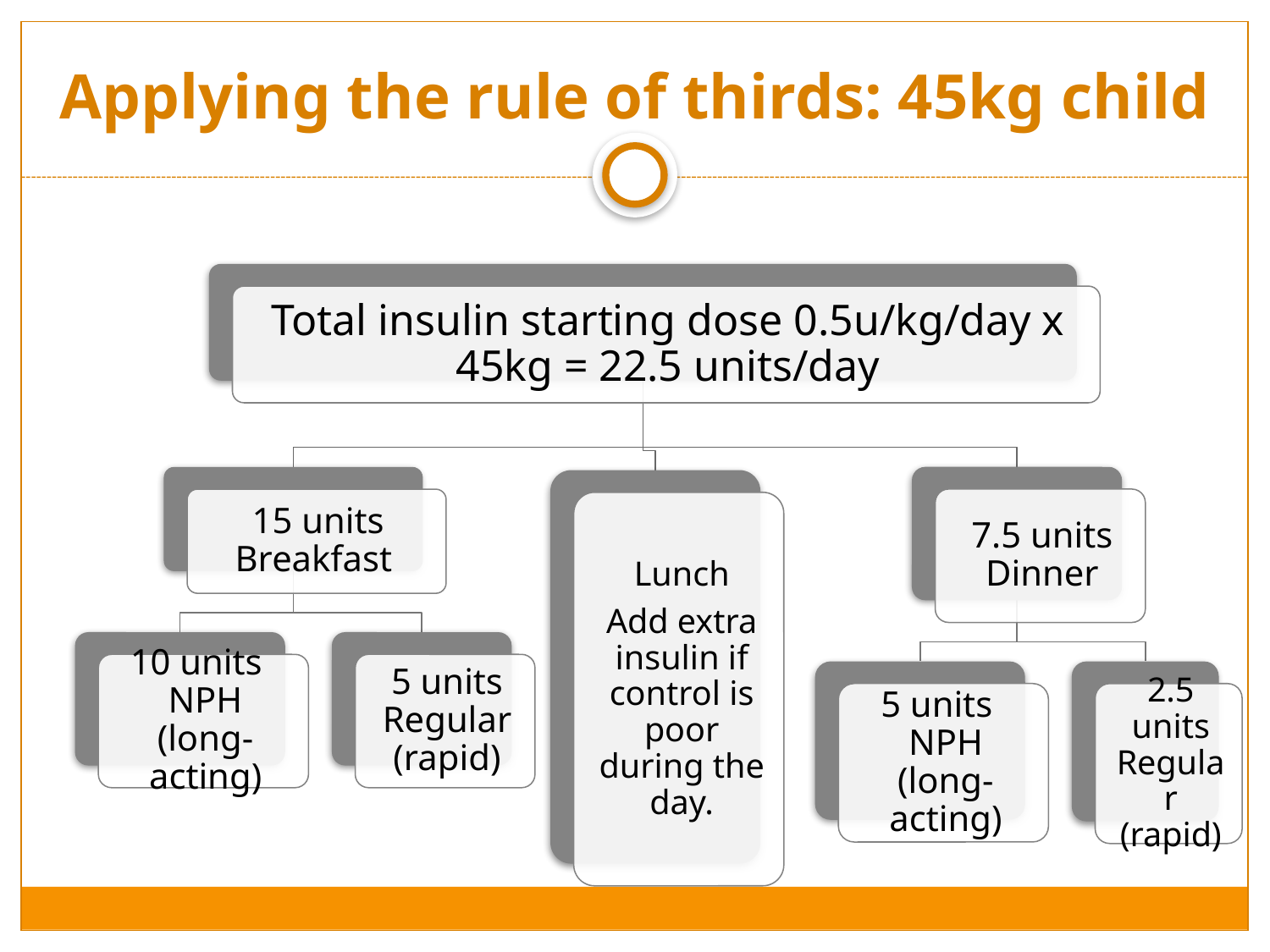

# Applying the rule of thirds: 45kg child

## Slide 20
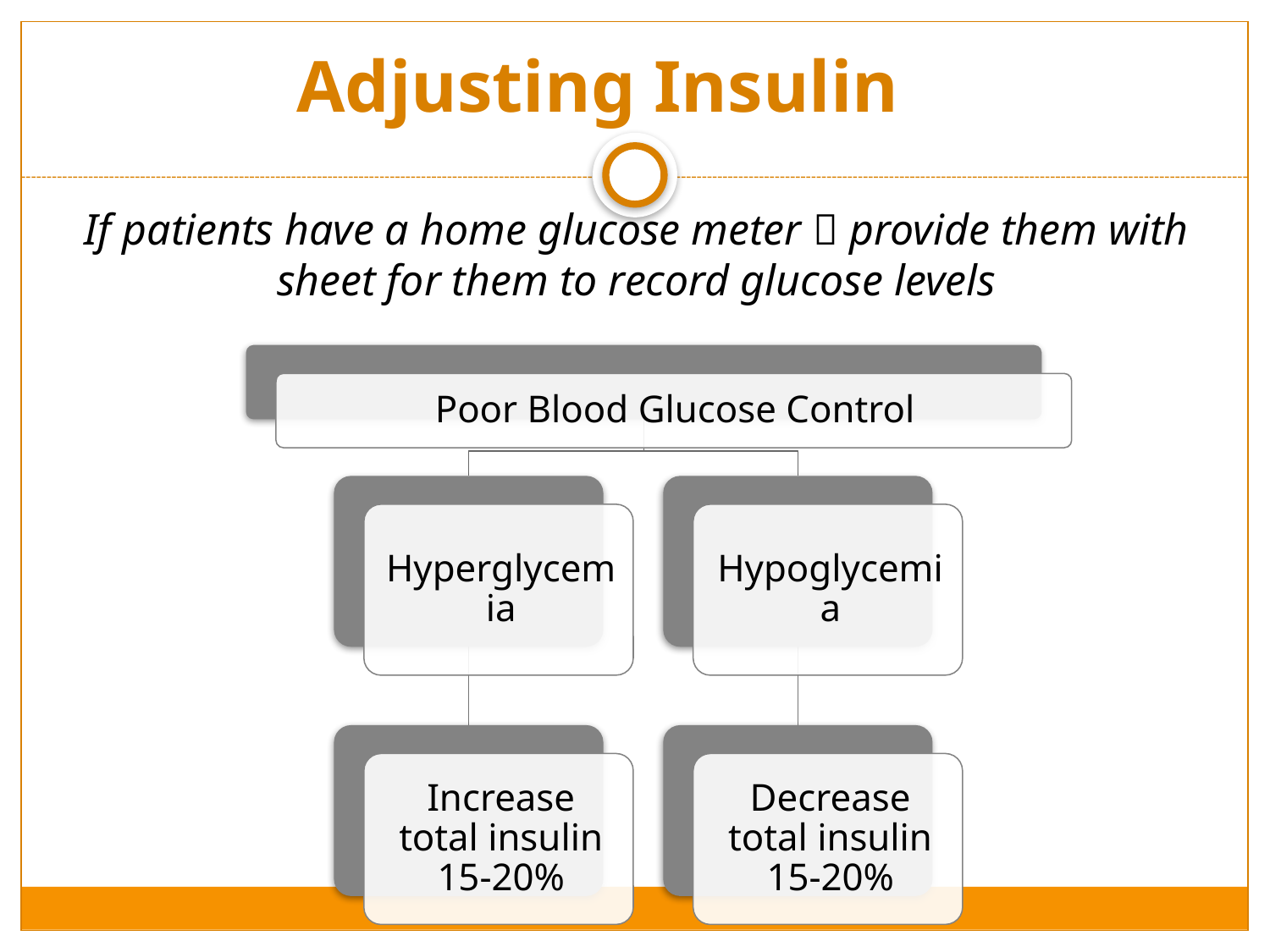

# Adjusting Insulin
If patients have a home glucose meter  provide them with sheet for them to record glucose levels

## Slide 21
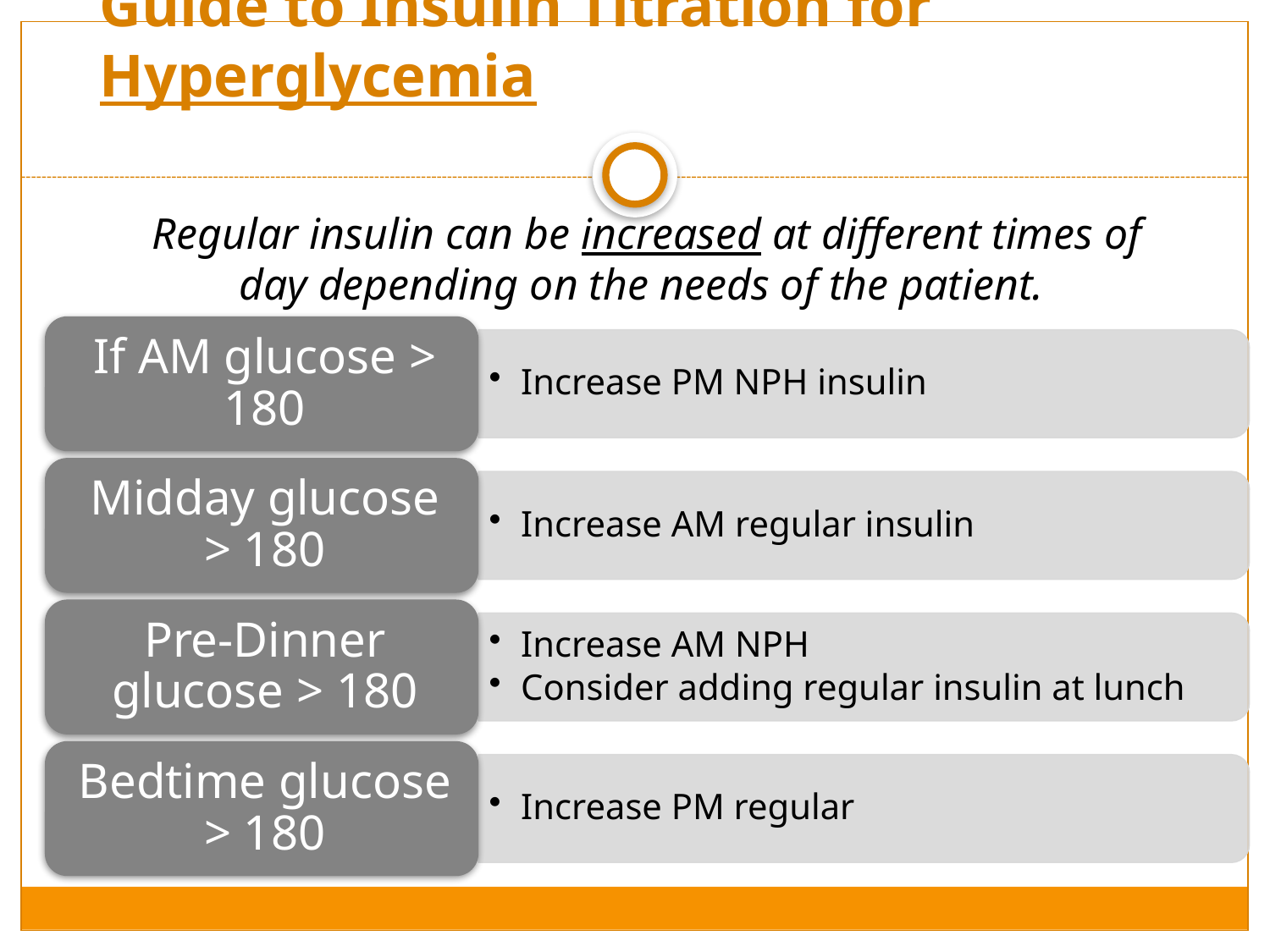

# Guide to Insulin Titration for Hyperglycemia
Regular insulin can be increased at different times of day depending on the needs of the patient.

## Slide 22
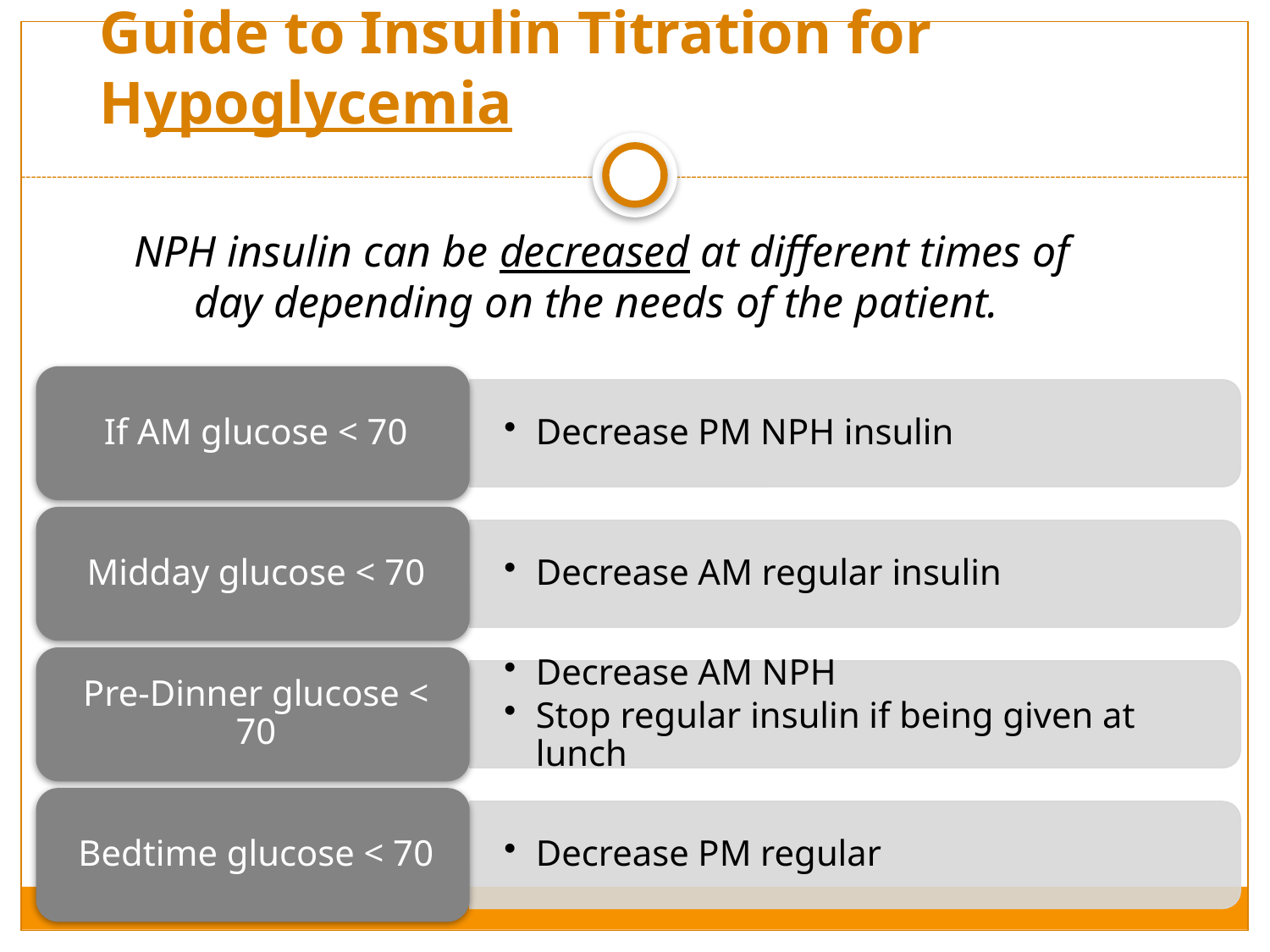

# Guide to Insulin Titration for Hypoglycemia
NPH insulin can be decreased at different times of day depending on the needs of the patient.

## Slide 23
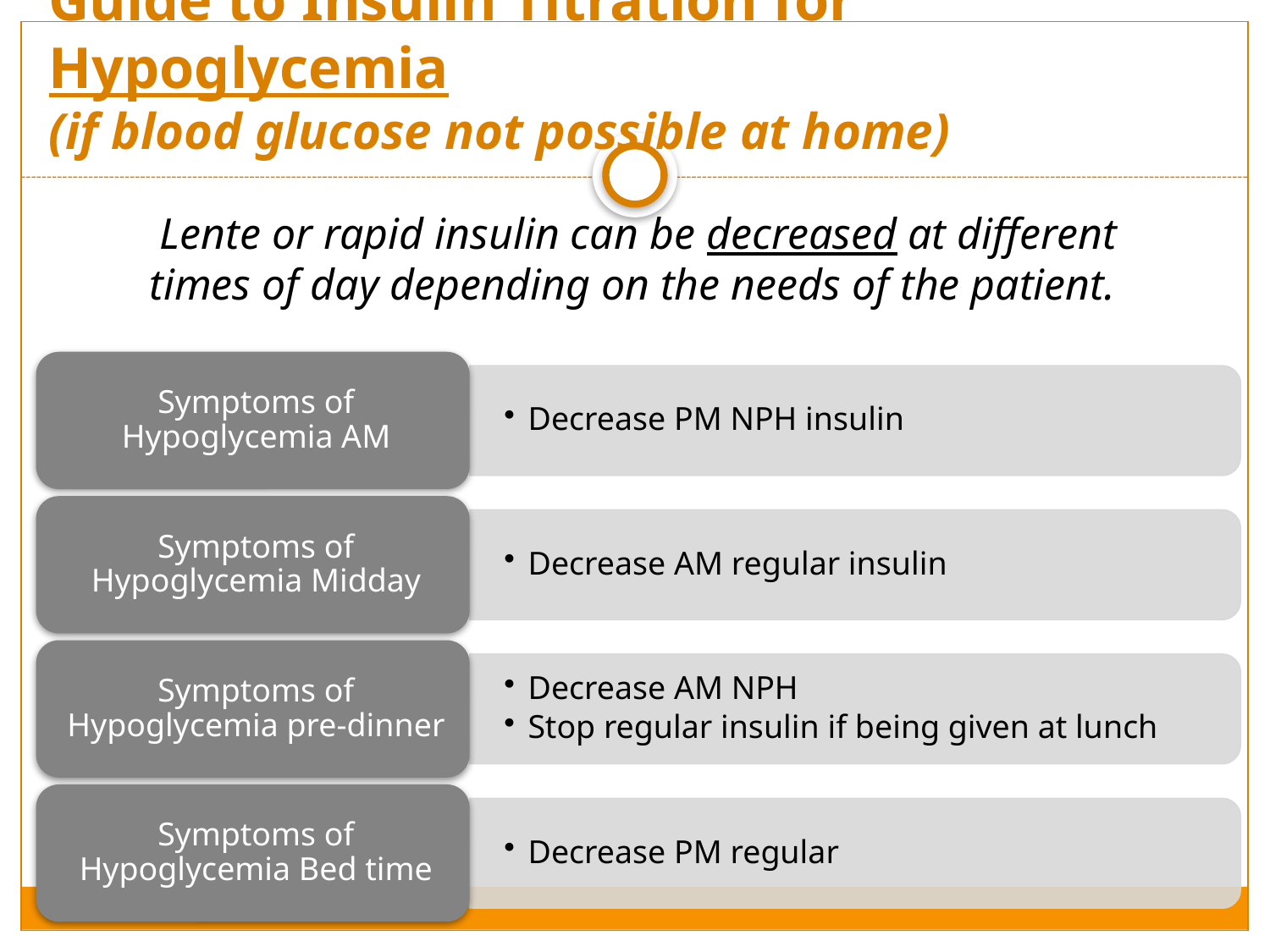

# Guide to Insulin Titration for Hypoglycemia (if blood glucose not possible at home)
Lente or rapid insulin can be decreased at different times of day depending on the needs of the patient.

## Slide 24
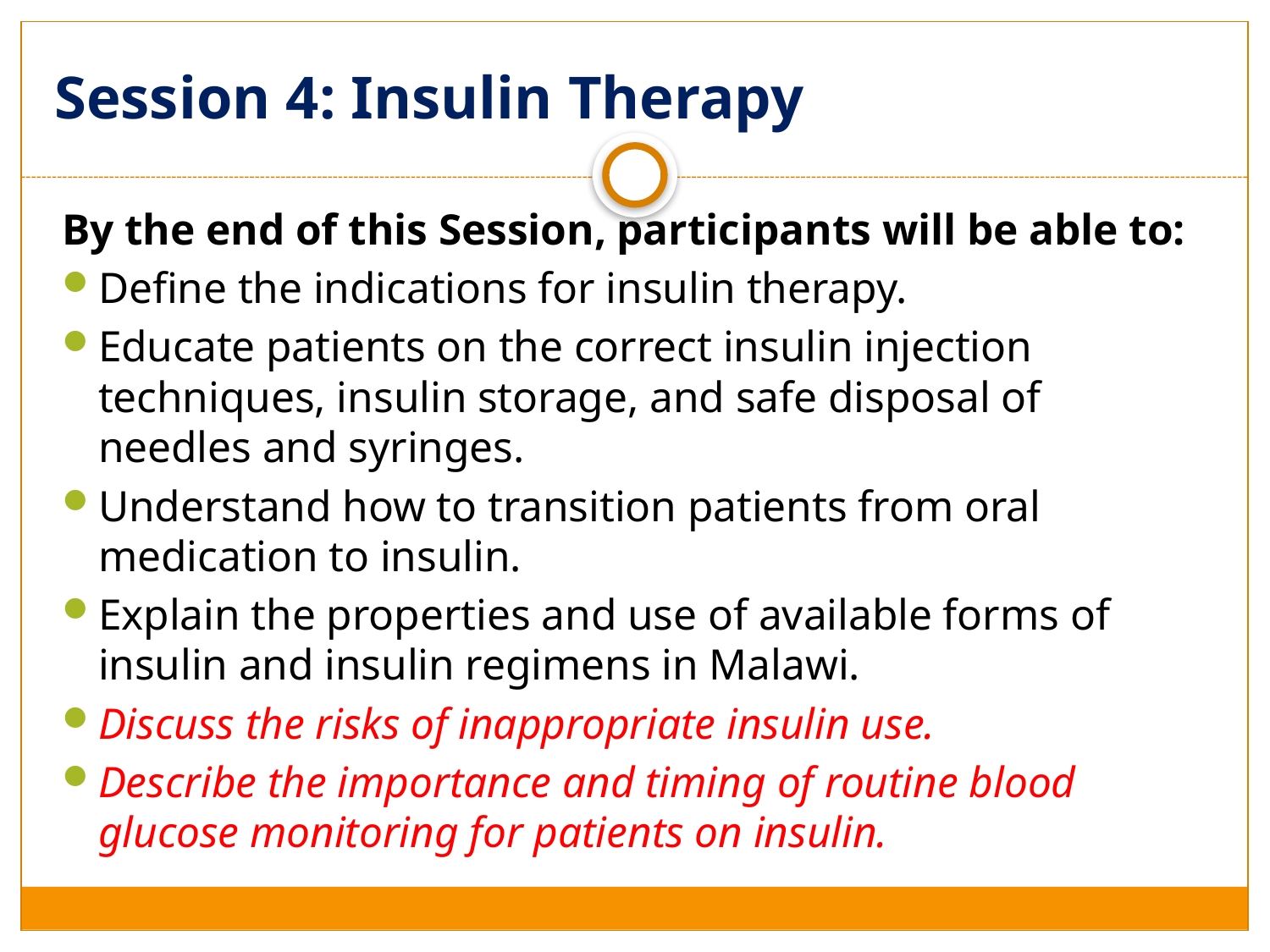

# Session 4: Insulin Therapy
By the end of this Session, participants will be able to:
Define the indications for insulin therapy.
Educate patients on the correct insulin injection techniques, insulin storage, and safe disposal of needles and syringes.
Understand how to transition patients from oral medication to insulin.
Explain the properties and use of available forms of insulin and insulin regimens in Malawi.
Discuss the risks of inappropriate insulin use.
Describe the importance and timing of routine blood glucose monitoring for patients on insulin.

## Slide 25
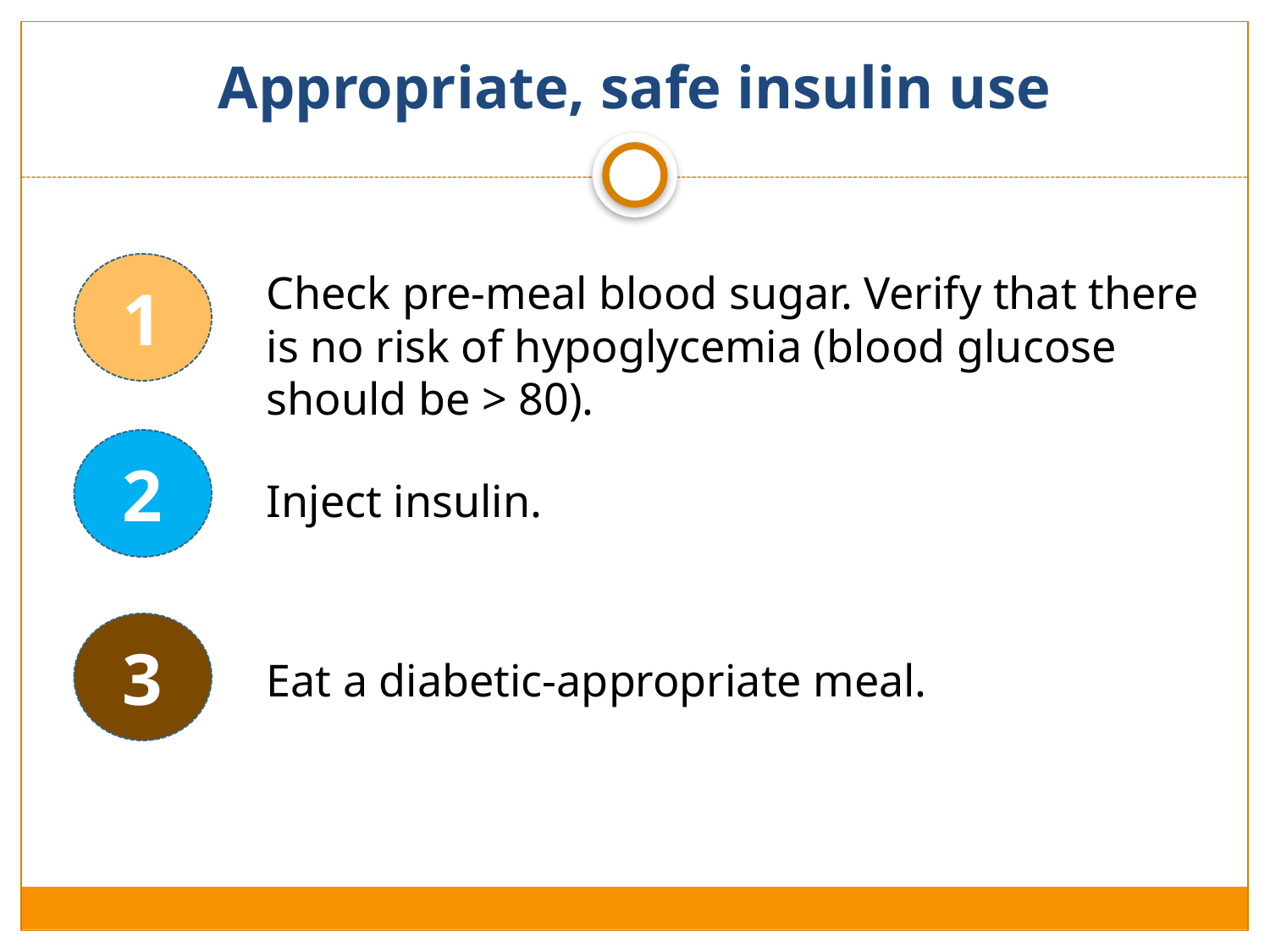

# Appropriate, safe insulin use
1
Check pre-meal blood sugar. Verify that there is no risk of hypoglycemia (blood glucose should be > 80).
2
Inject insulin.
3
Eat a diabetic-appropriate meal.

## Slide 26
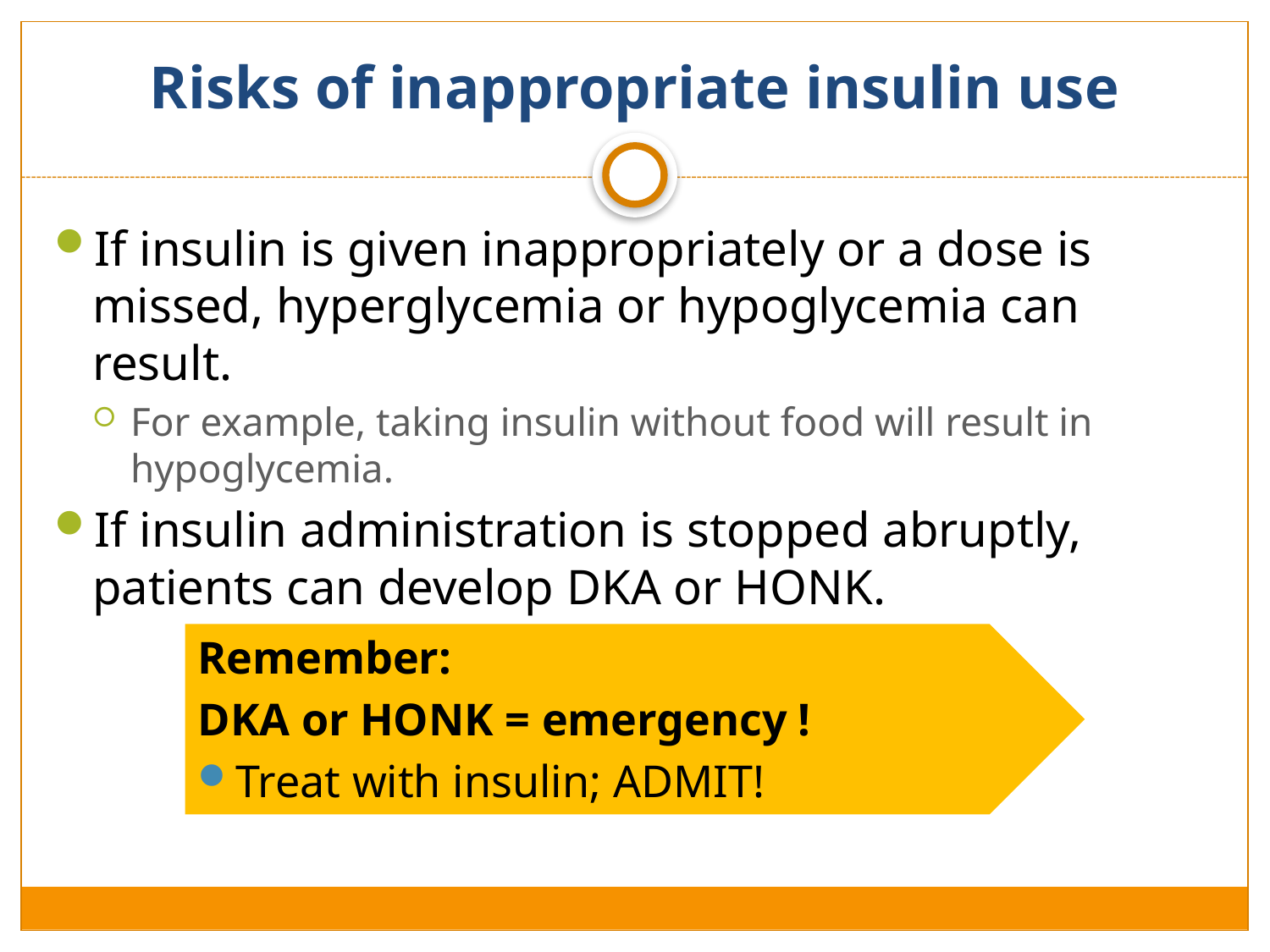

# Risks of inappropriate insulin use
If insulin is given inappropriately or a dose is missed, hyperglycemia or hypoglycemia can result.
For example, taking insulin without food will result in hypoglycemia.
If insulin administration is stopped abruptly, patients can develop DKA or HONK.
Remember:
DKA or HONK = emergency !
Treat with insulin; ADMIT!

## Slide 27
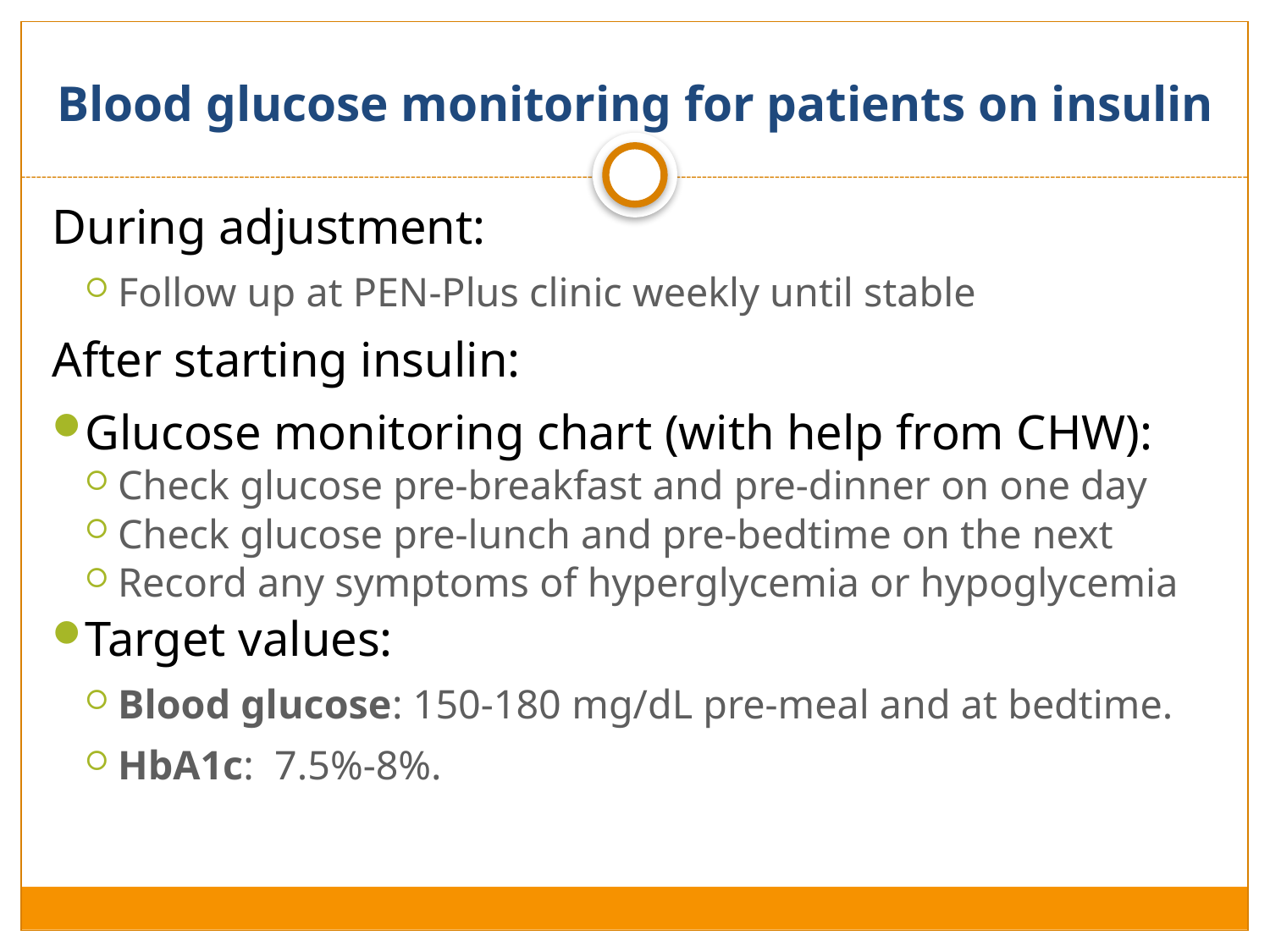

# Blood glucose monitoring for patients on insulin
During adjustment:
Follow up at PEN-Plus clinic weekly until stable
After starting insulin:
Glucose monitoring chart (with help from CHW):
Check glucose pre-breakfast and pre-dinner on one day
Check glucose pre-lunch and pre-bedtime on the next
Record any symptoms of hyperglycemia or hypoglycemia
Target values:
Blood glucose: 150-180 mg/dL pre-meal and at bedtime.
HbA1c: 7.5%-8%.

## Slide 28
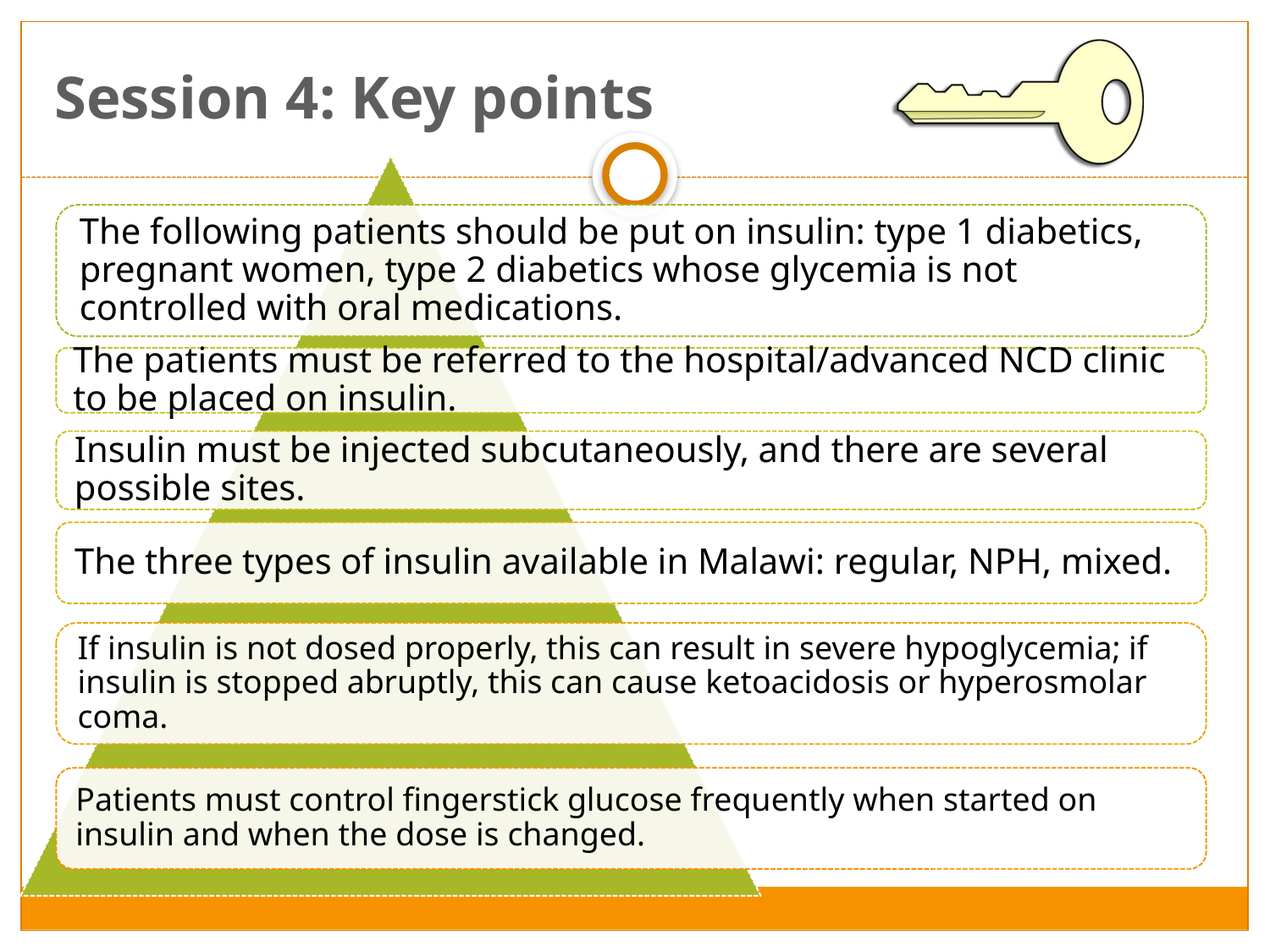

# Session 4: Key points
