# Supplementary material for: Training Mid-Level Providers to Treat Severe Non-Communicable Diseases in Neno, Malawi through PEN-Plus Strategies
Source: Ann Glob Health. 2022 Aug 11;88(1):69. doi: 10.5334/aogh.3750 (PMC9389951; doi:10.5334/aogh.3750)
Supplement: Didactic Materials. — The supplementary materials contain a suggested didactic training schedule and the PowerPoint presentations used for PEN-Plus training in Neno, Malawi. These materials have been reviewed and accepted by the Malawi Ministry of Health for future PEN-Plus trainings in Malawi. [file agh-88-1-3750-s2.zip › Didactic_Materials/DM_Medications.pptx]

## Slide 1
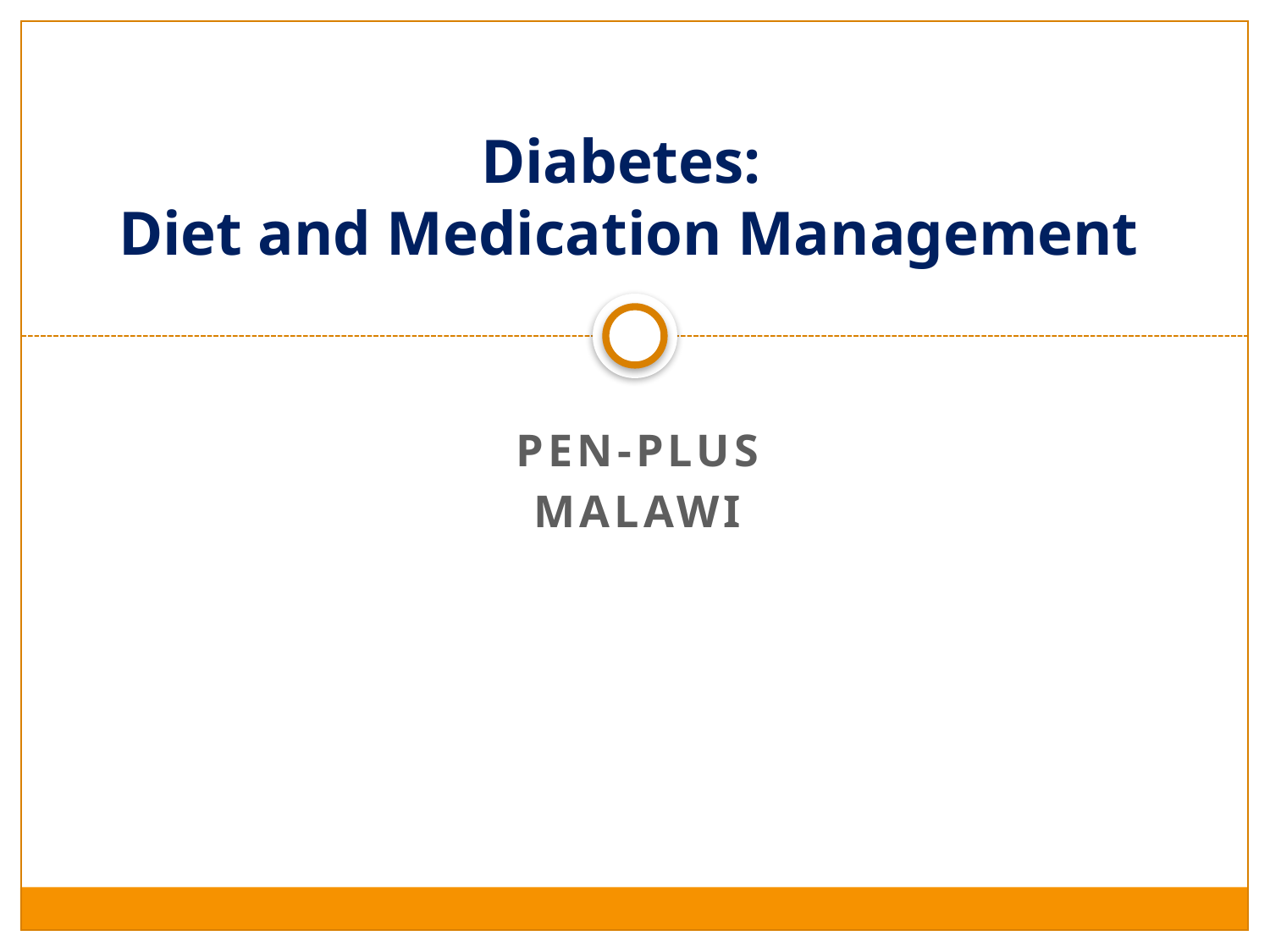

# Diabetes: Diet and Medication Management
PEN-Plus
Malawi

## Slide 2
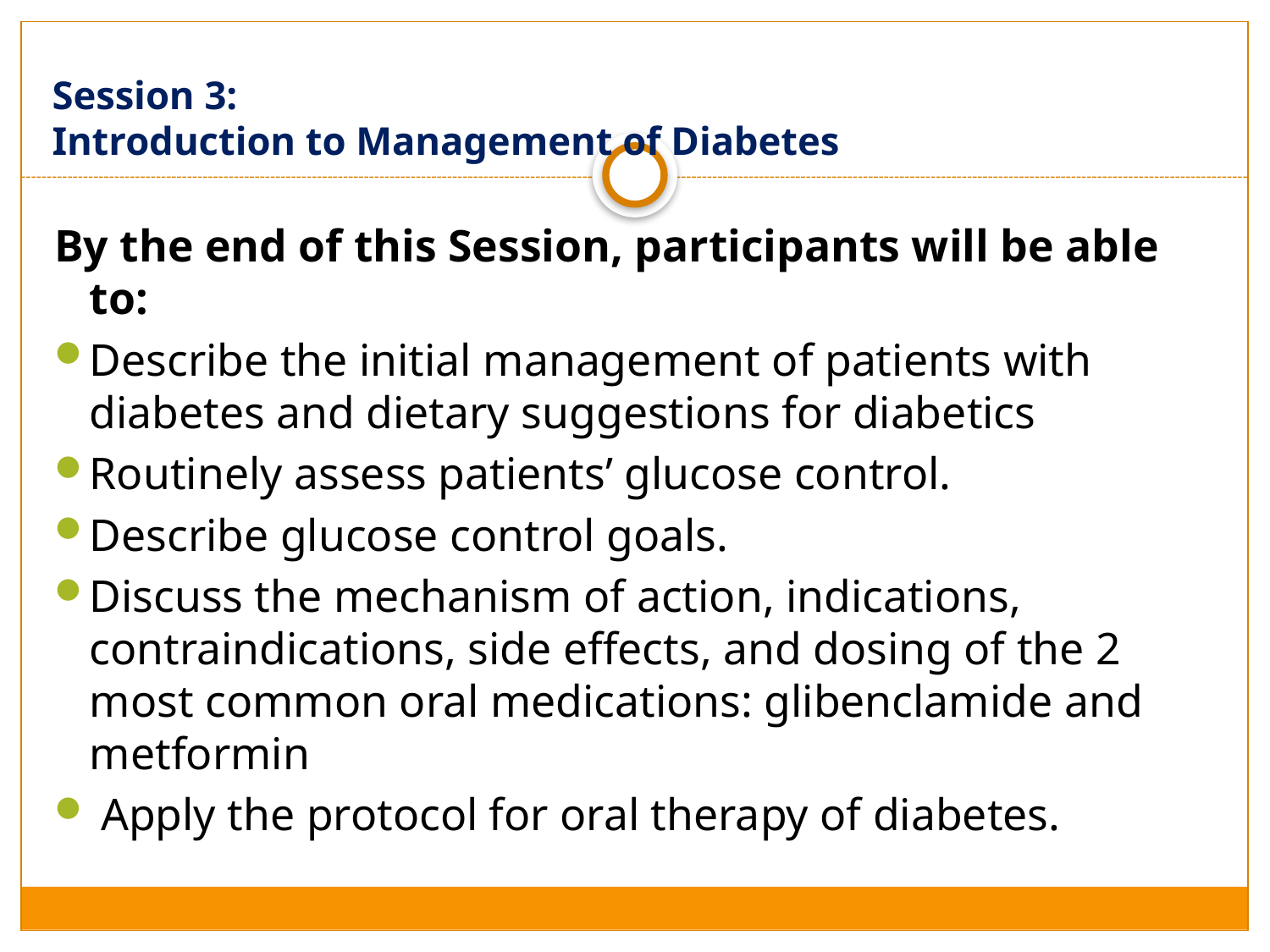

# Session 3: Introduction to Management of Diabetes
By the end of this Session, participants will be able to:
Describe the initial management of patients with diabetes and dietary suggestions for diabetics
Routinely assess patients’ glucose control.
Describe glucose control goals.
Discuss the mechanism of action, indications, contraindications, side effects, and dosing of the 2 most common oral medications: glibenclamide and metformin
 Apply the protocol for oral therapy of diabetes.

## Slide 3
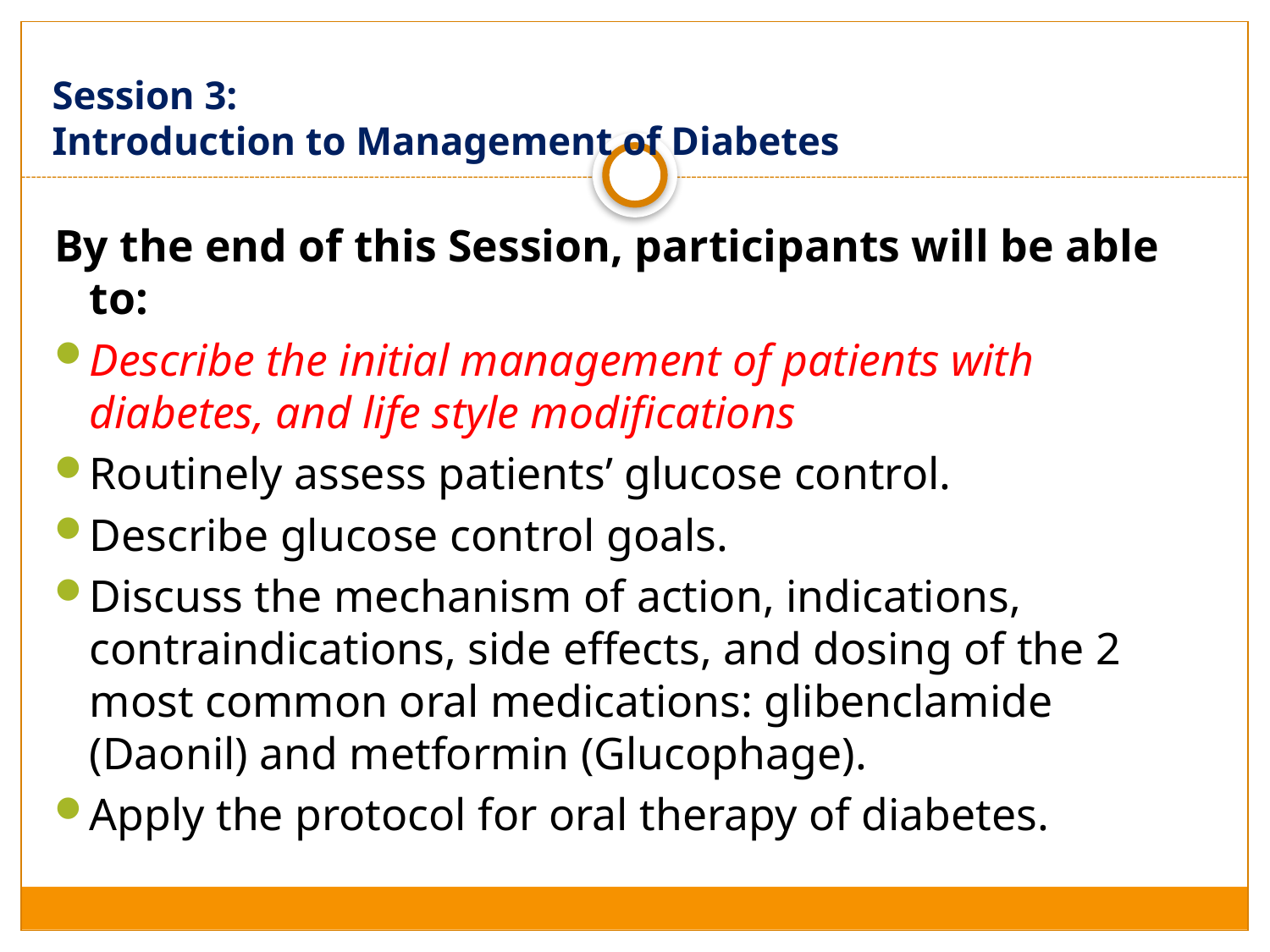

# Session 3: Introduction to Management of Diabetes
By the end of this Session, participants will be able to:
Describe the initial management of patients with diabetes, and life style modifications
Routinely assess patients’ glucose control.
Describe glucose control goals.
Discuss the mechanism of action, indications, contraindications, side effects, and dosing of the 2 most common oral medications: glibenclamide (Daonil) and metformin (Glucophage).
Apply the protocol for oral therapy of diabetes.

## Slide 4
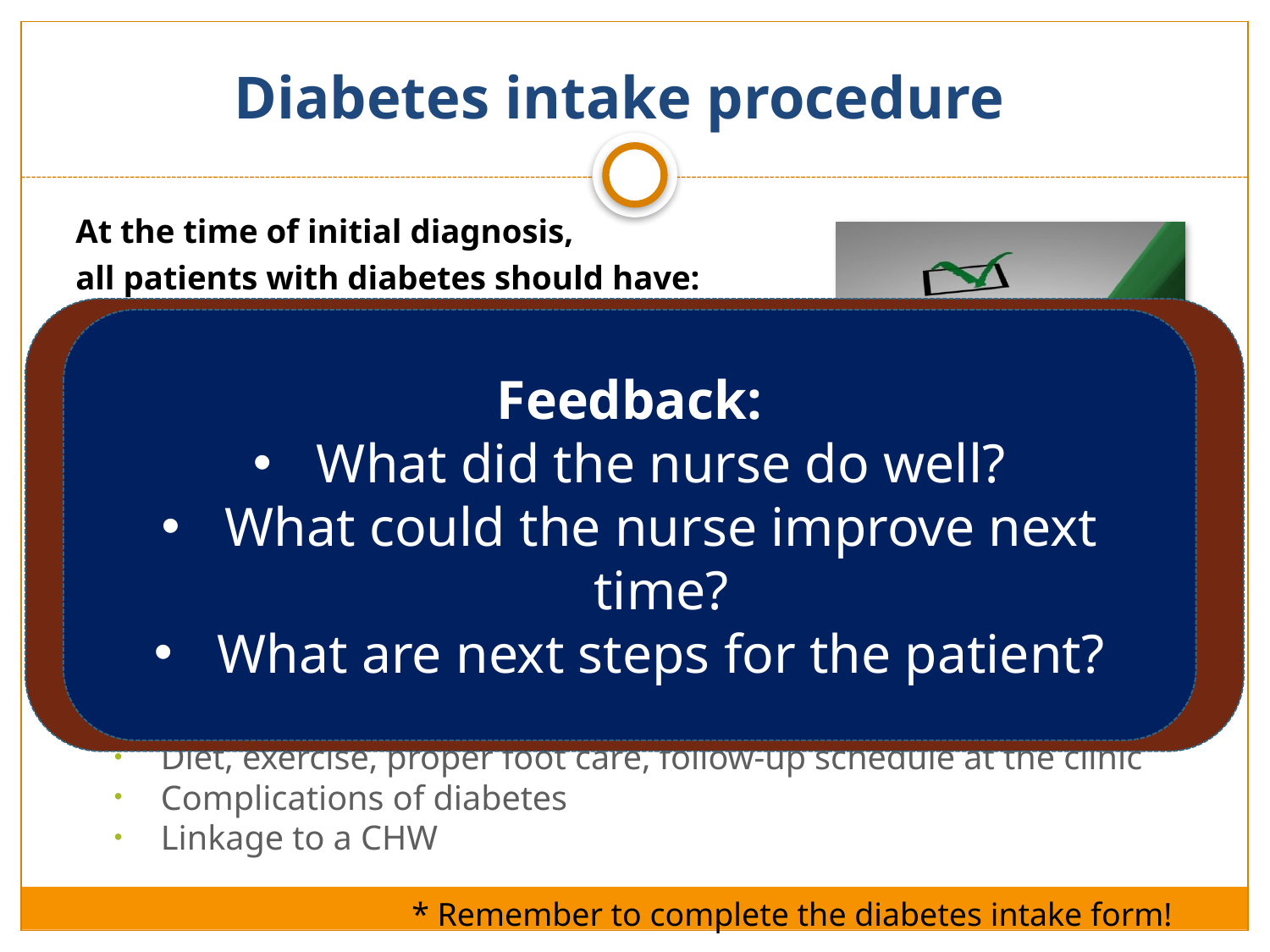

# Diabetes intake procedure
At the time of initial diagnosis,
all patients with diabetes should have:
Blood pressure measurement
Foot examination with a monofilament
Fundoscopic examination
Creatinine test and Urine dipstick
Nutrition assessment
HbA1c (hemoglobin A1c)
Risk assessment and CV risk %
HIV test
Role Play:
Participant #1 is a 35 year old person just diagnosed with Type II diabetes.
Participant #2 is a nurse who is completing his intake and teaching the patient about his condition.
Feedback:
What did the nurse do well?
What could the nurse improve next time?
What are next steps for the patient?
Patient education and linkages to community support:
 Diet, exercise, proper foot care, follow-up schedule at the clinic
 Complications of diabetes
 Linkage to a CHW
* Remember to complete the diabetes intake form!

## Slide 5
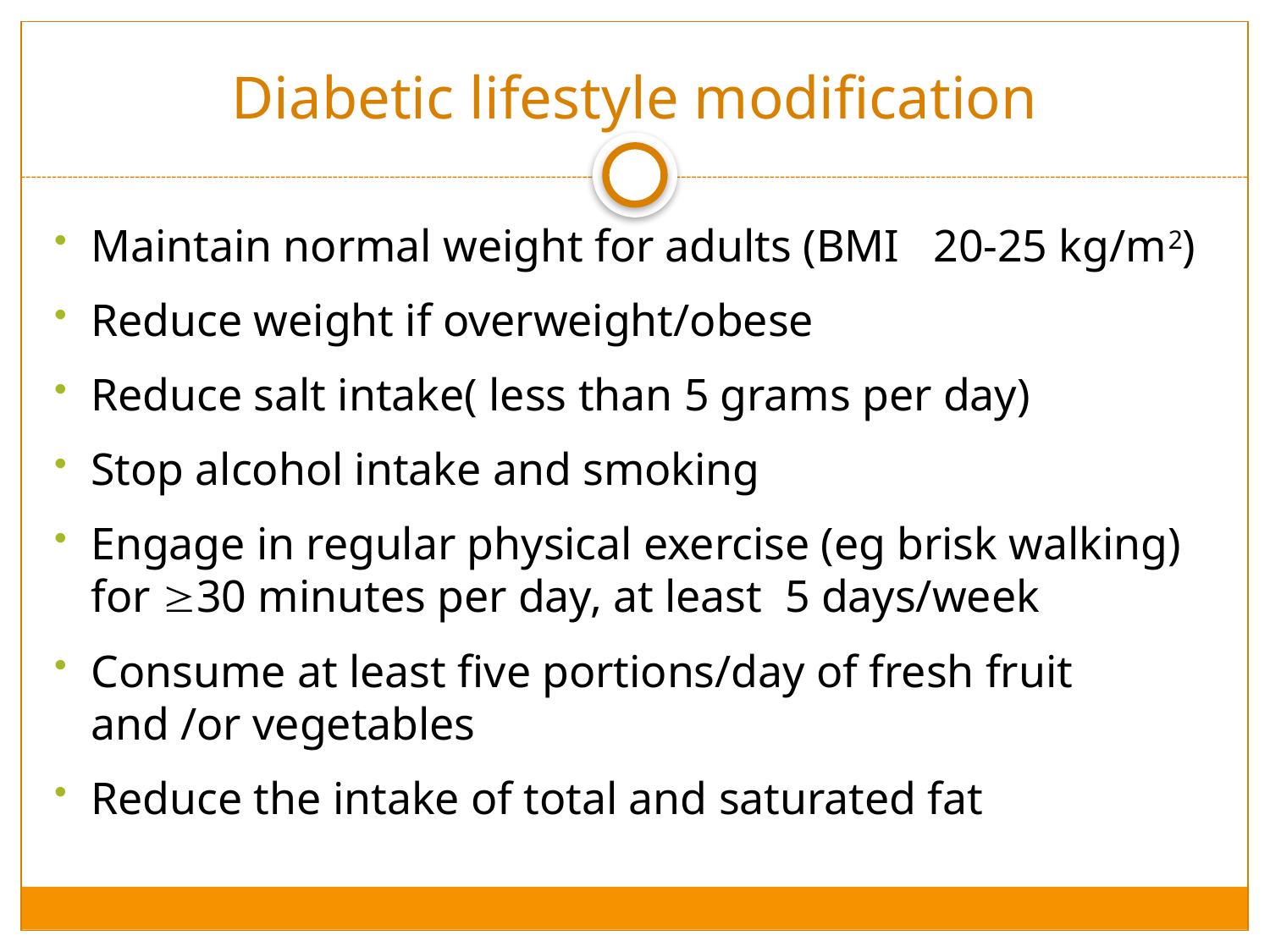

# Diabetic lifestyle modification
Maintain normal weight for adults (BMI 20-25 kg/m2)
Reduce weight if overweight/obese
Reduce salt intake( less than 5 grams per day)
Stop alcohol intake and smoking
Engage in regular physical exercise (eg brisk walking) for ≥30 minutes per day, at least 5 days/week
Consume at least five portions/day of fresh fruit and /or vegetables
Reduce the intake of total and saturated fat

## Slide 6
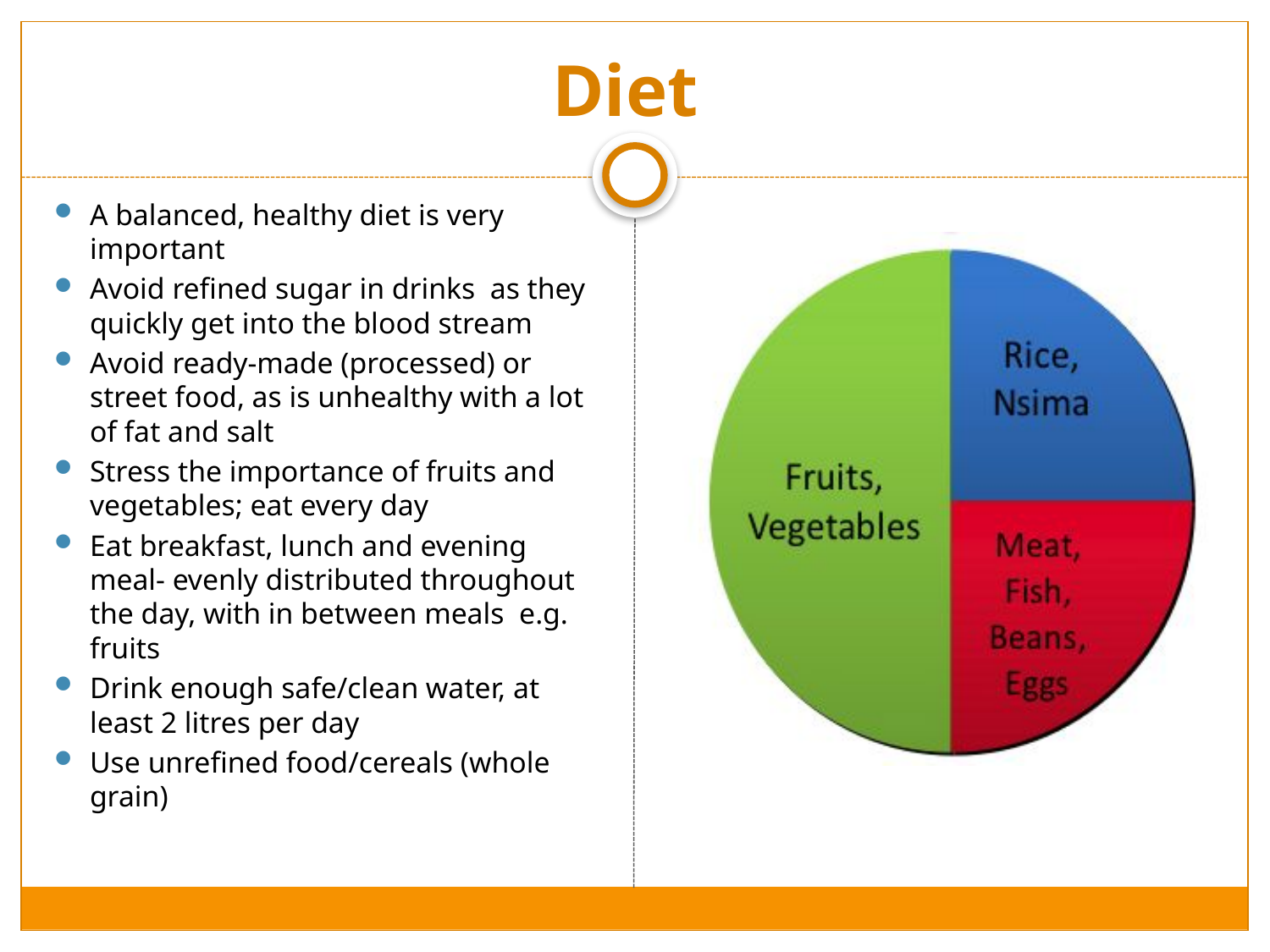

# Diet
A balanced, healthy diet is very important
Avoid refined sugar in drinks as they quickly get into the blood stream
Avoid ready-made (processed) or street food, as is unhealthy with a lot of fat and salt
Stress the importance of fruits and vegetables; eat every day
Eat breakfast, lunch and evening meal- evenly distributed throughout the day, with in between meals e.g. fruits
Drink enough safe/clean water, at least 2 litres per day
Use unrefined food/cereals (whole grain)

## Slide 7
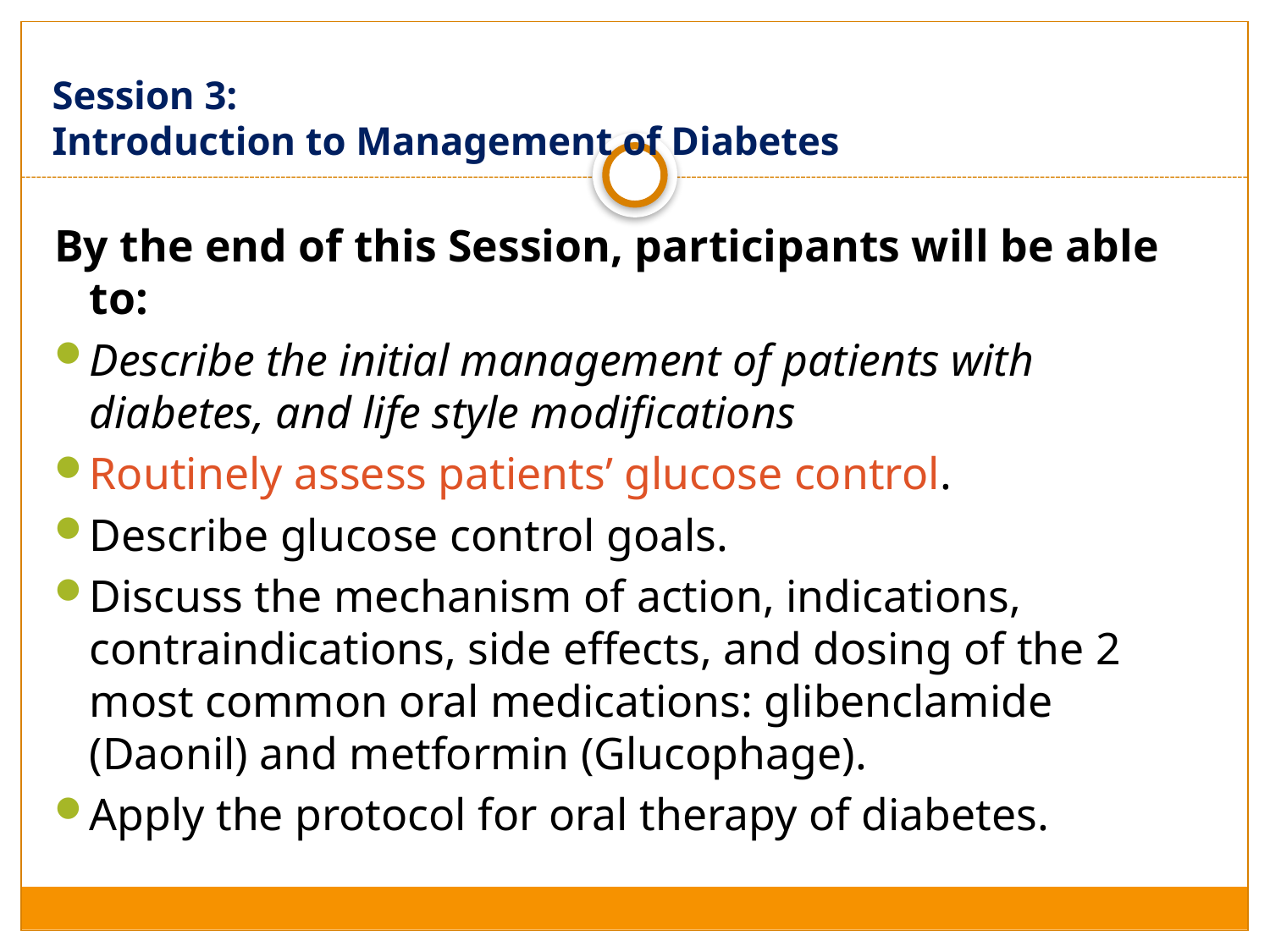

# Session 3: Introduction to Management of Diabetes
By the end of this Session, participants will be able to:
Describe the initial management of patients with diabetes, and life style modifications
Routinely assess patients’ glucose control.
Describe glucose control goals.
Discuss the mechanism of action, indications, contraindications, side effects, and dosing of the 2 most common oral medications: glibenclamide (Daonil) and metformin (Glucophage).
Apply the protocol for oral therapy of diabetes.

## Slide 8
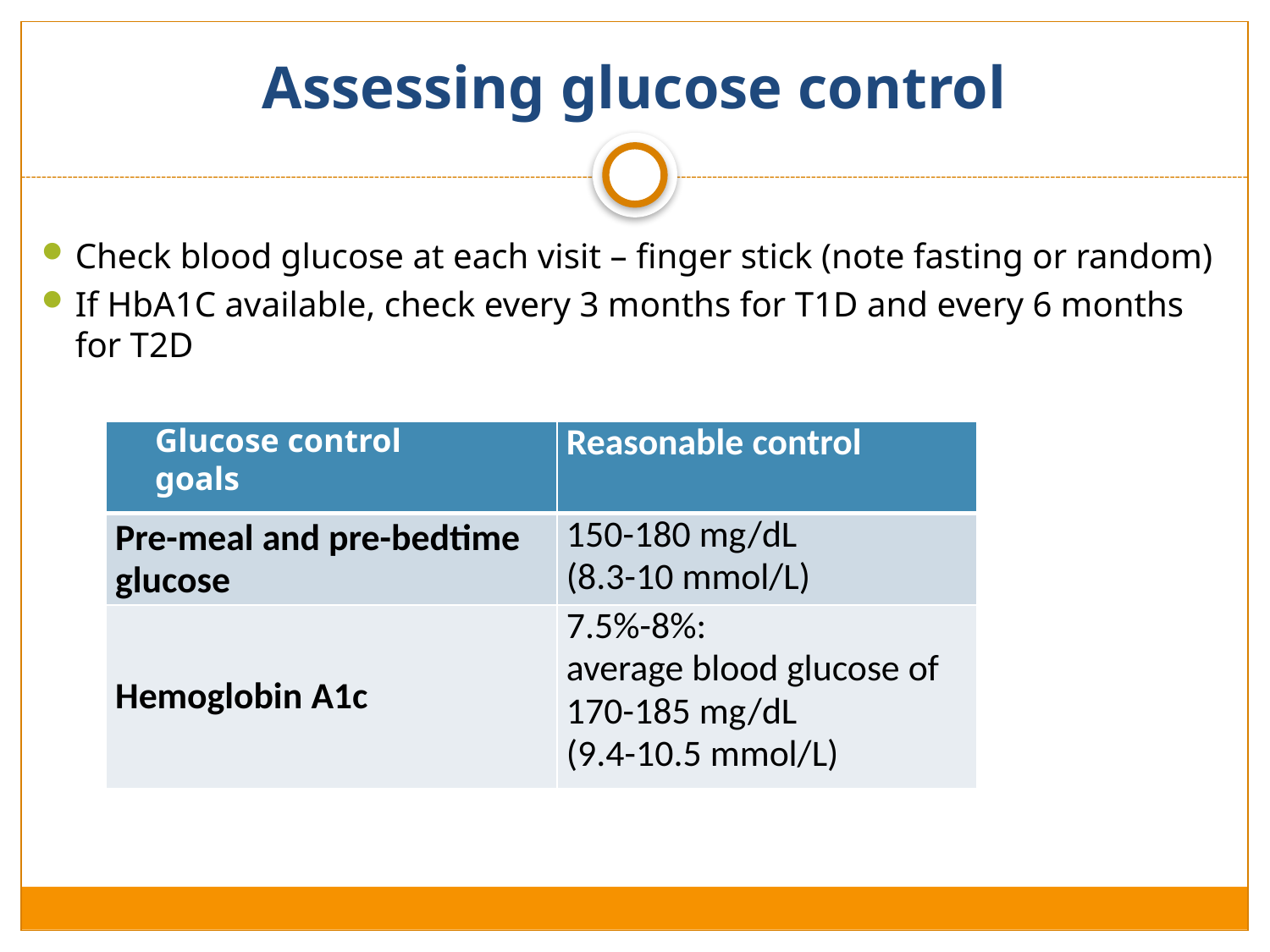

# Assessing glucose control
Check blood glucose at each visit – finger stick (note fasting or random)
If HbA1C available, check every 3 months for T1D and every 6 months for T2D
Table 8.4: Glucose control goals
| | Reasonable control |
| --- | --- |
| Pre-meal and pre-bedtime glucose | 150-180 mg/dL (8.3-10 mmol/L) |
| Hemoglobin A1c | 7.5%-8%: average blood glucose of 170-185 mg/dL (9.4-10.5 mmol/L) |

## Slide 9
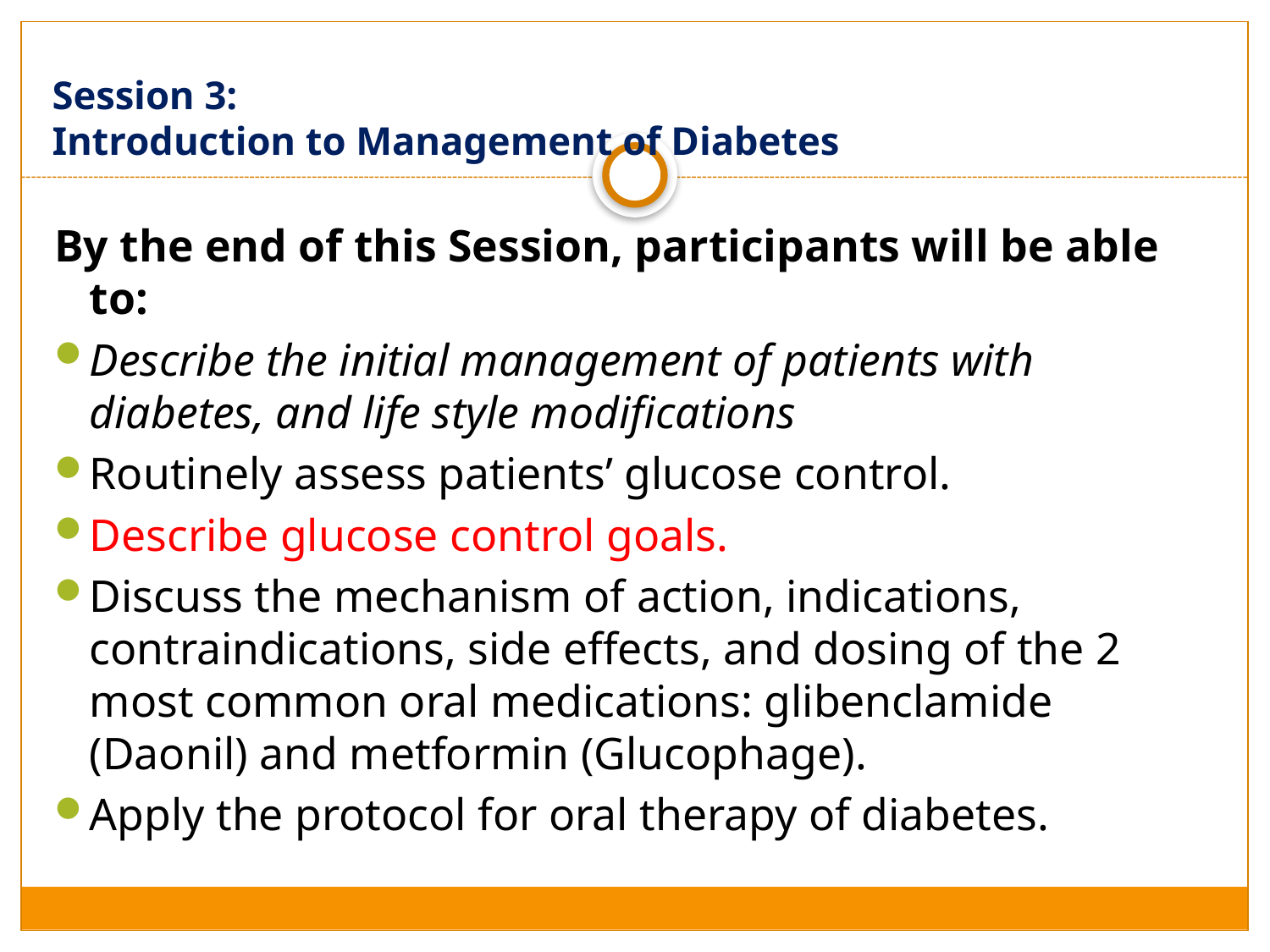

# Session 3: Introduction to Management of Diabetes
By the end of this Session, participants will be able to:
Describe the initial management of patients with diabetes, and life style modifications
Routinely assess patients’ glucose control.
Describe glucose control goals.
Discuss the mechanism of action, indications, contraindications, side effects, and dosing of the 2 most common oral medications: glibenclamide (Daonil) and metformin (Glucophage).
Apply the protocol for oral therapy of diabetes.

## Slide 10
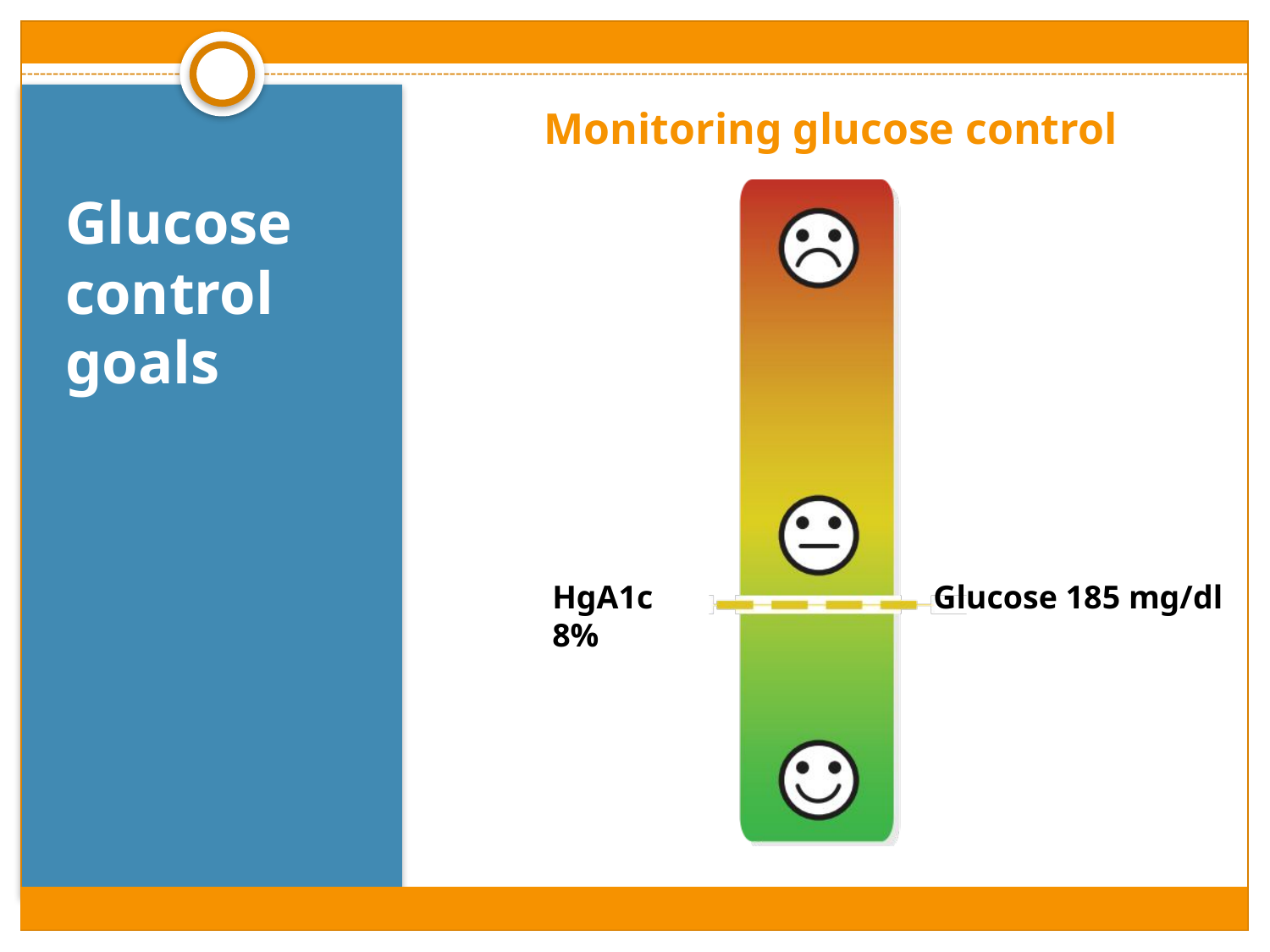

Monitoring glucose control
# Glucose control goals
HgA1c 8%
Glucose 185 mg/dl

## Slide 11
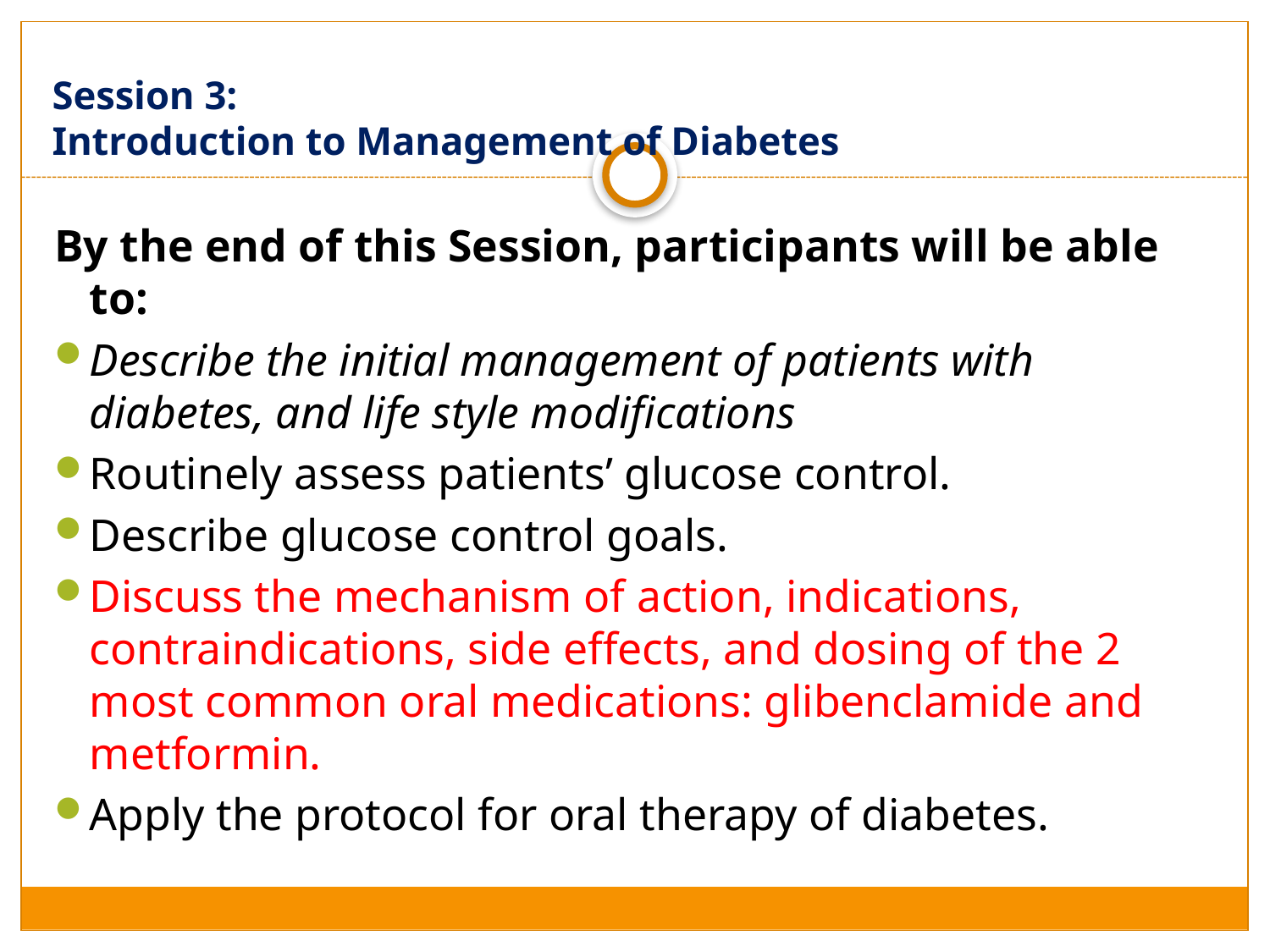

# Session 3: Introduction to Management of Diabetes
By the end of this Session, participants will be able to:
Describe the initial management of patients with diabetes, and life style modifications
Routinely assess patients’ glucose control.
Describe glucose control goals.
Discuss the mechanism of action, indications, contraindications, side effects, and dosing of the 2 most common oral medications: glibenclamide and metformin.
Apply the protocol for oral therapy of diabetes.

## Slide 12
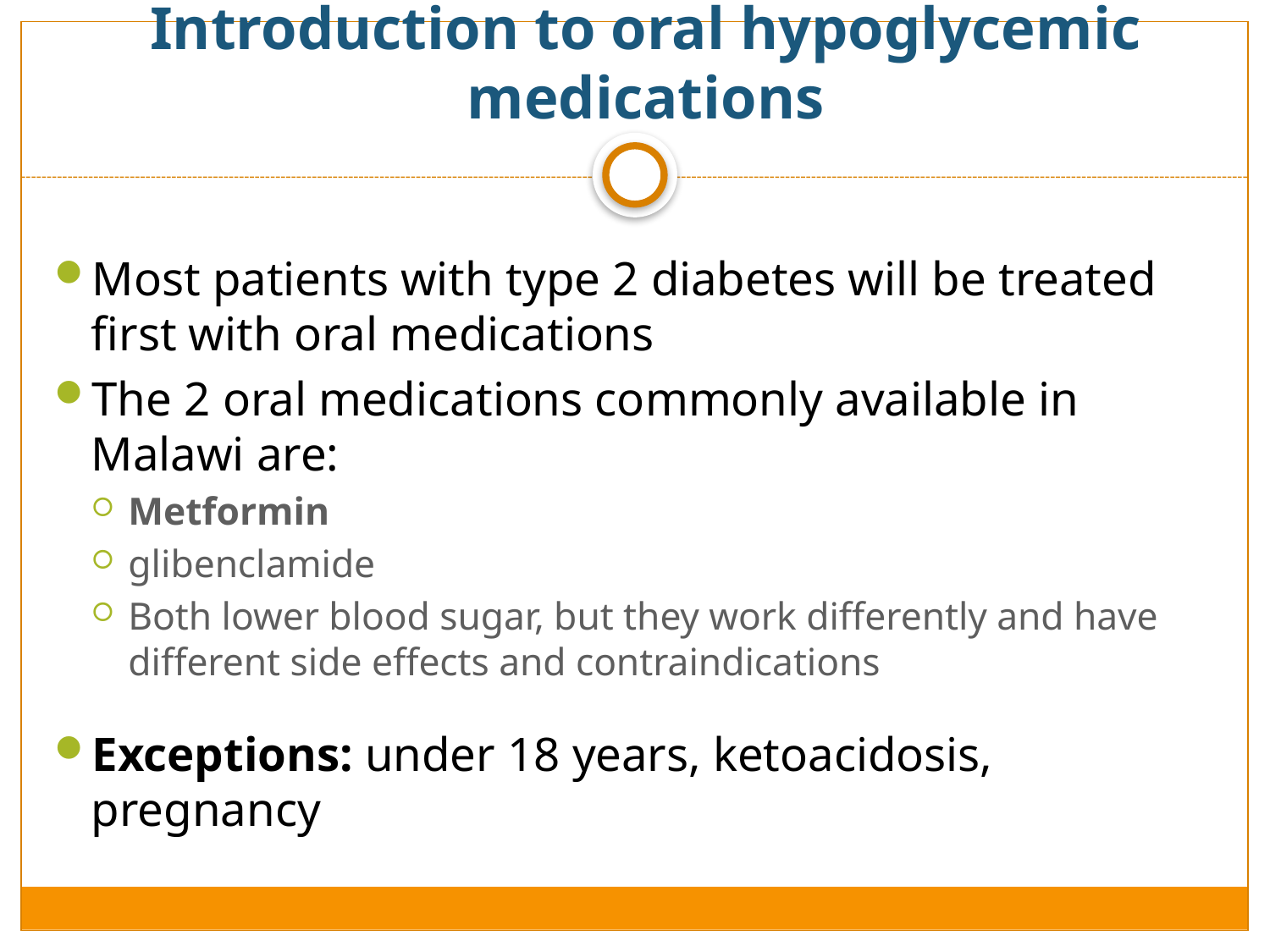

# Introduction to oral hypoglycemic medications
Most patients with type 2 diabetes will be treated first with oral medications
The 2 oral medications commonly available in Malawi are:
Metformin
glibenclamide
Both lower blood sugar, but they work differently and have different side effects and contraindications
Exceptions: under 18 years, ketoacidosis, pregnancy

## Slide 13
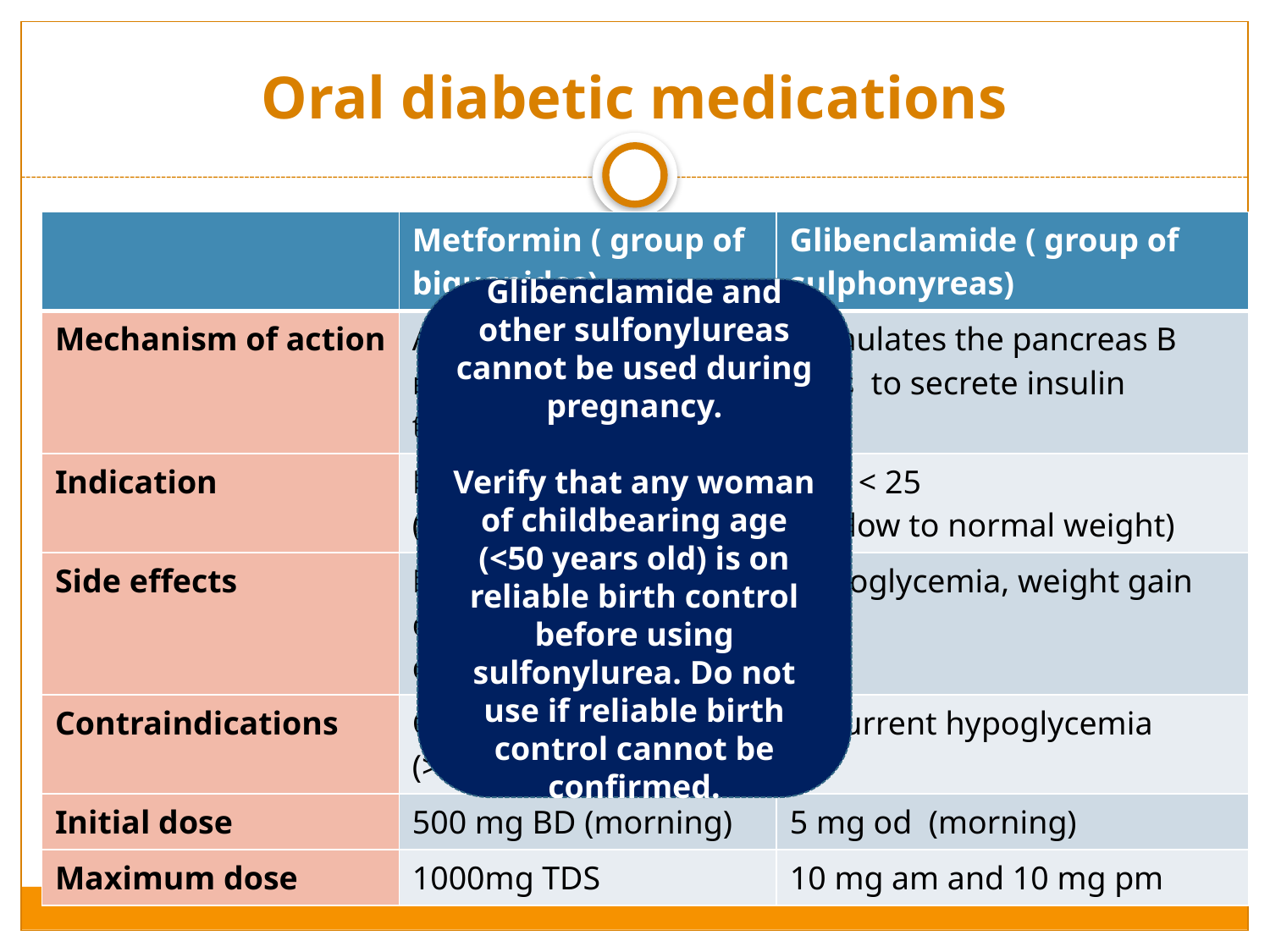

# Oral diabetic medications
| | Metformin ( group of biguanides) | Glibenclamide ( group of sulphonyreas) |
| --- | --- | --- |
| Mechanism of action | Acts on the body (liver, muscles), to modulate the action of insulin | Stimulates the pancreas B cells to secrete insulin |
| Indication | BMI > 25 (overweight) | BMI < 25 (low to normal weight) |
| Side effects | Nausea, abdominal discomfort (gas, diarrhea) | Hypoglycemia, weight gain |
| Contraindications | Cr >130 μmol/L (>2.0 mg/dL) | Recurrent hypoglycemia |
| Initial dose | 500 mg BD (morning) | 5 mg od (morning) |
| Maximum dose | 1000mg TDS | 10 mg am and 10 mg pm |
Glibenclamide and other sulfonylureas cannot be used during pregnancy.
Verify that any woman of childbearing age (<50 years old) is on reliable birth control before using sulfonylurea. Do not use if reliable birth control cannot be confirmed.

## Slide 14
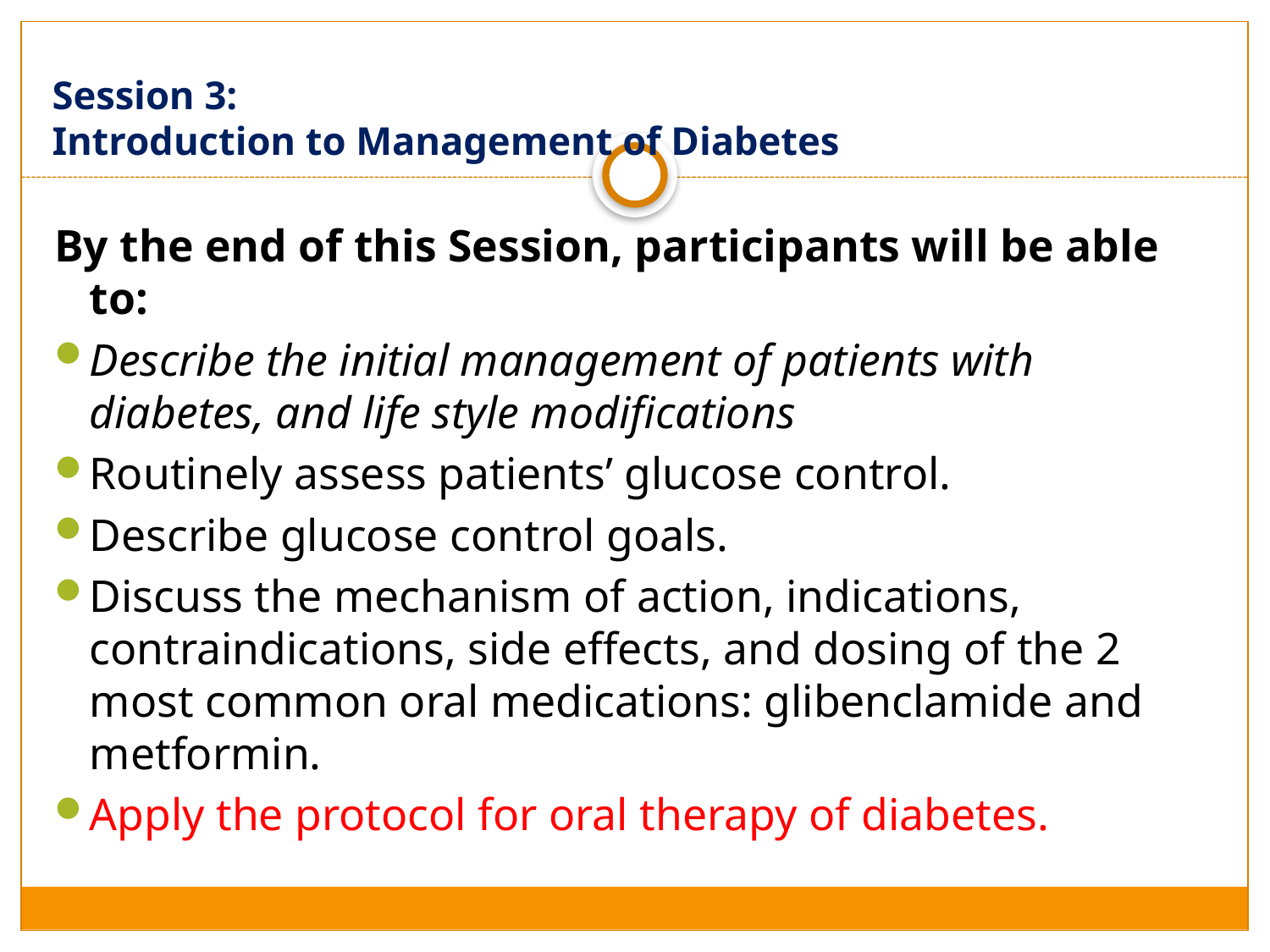

# Session 3: Introduction to Management of Diabetes
By the end of this Session, participants will be able to:
Describe the initial management of patients with diabetes, and life style modifications
Routinely assess patients’ glucose control.
Describe glucose control goals.
Discuss the mechanism of action, indications, contraindications, side effects, and dosing of the 2 most common oral medications: glibenclamide and metformin.
Apply the protocol for oral therapy of diabetes.

## Slide 15
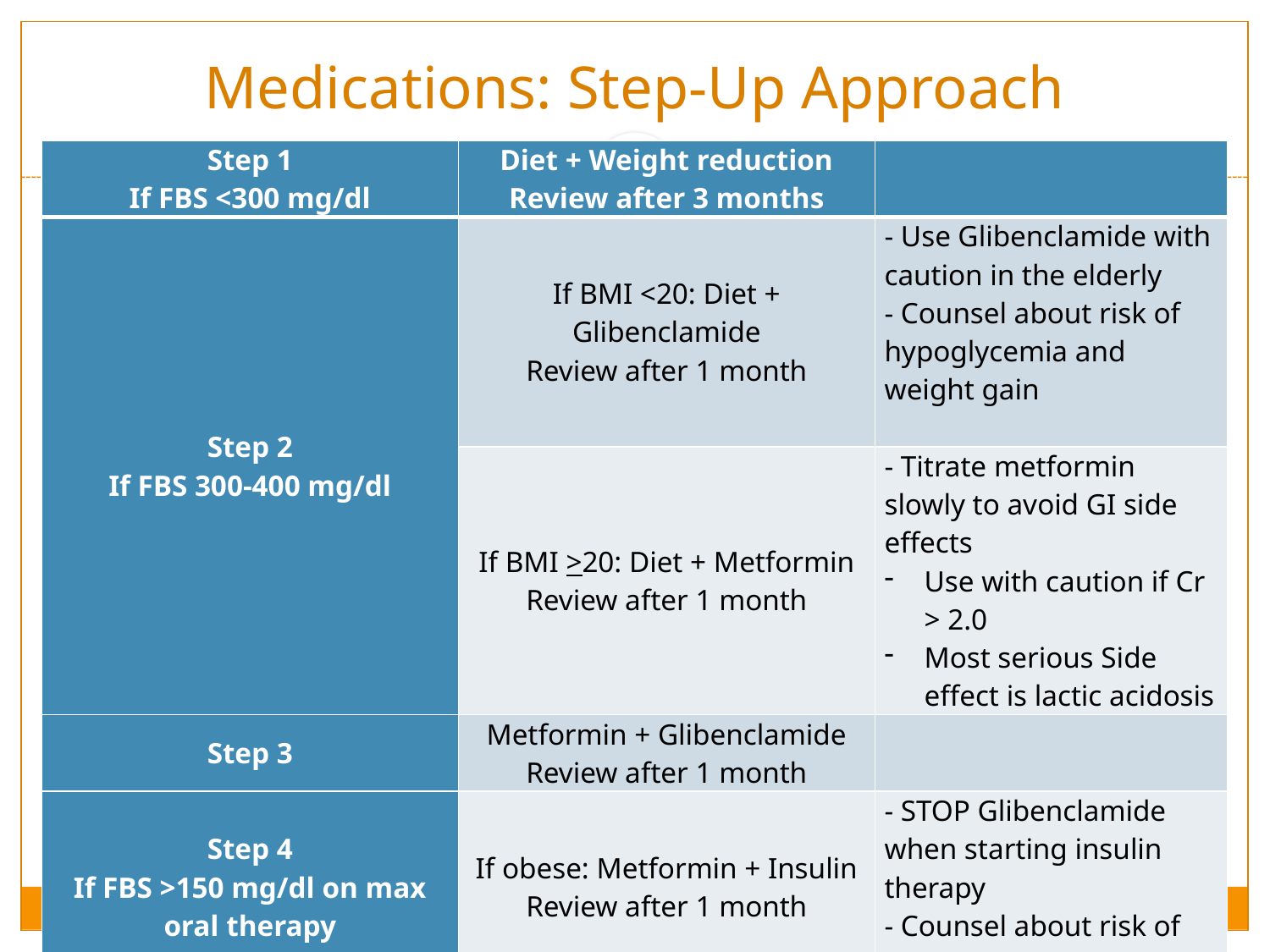

# Medications: Step-Up Approach
| Step 1 If FBS <300 mg/dl | Diet + Weight reduction Review after 3 months | |
| --- | --- | --- |
| Step 2 If FBS 300-400 mg/dl | If BMI <20: Diet + Glibenclamide Review after 1 month | - Use Glibenclamide with caution in the elderly - Counsel about risk of hypoglycemia and weight gain |
| | If BMI >20: Diet + Metformin Review after 1 month | - Titrate metformin slowly to avoid GI side effects Use with caution if Cr > 2.0 Most serious Side effect is lactic acidosis |
| Step 3 | Metformin + Glibenclamide Review after 1 month | |
| Step 4 If FBS >150 mg/dl on max oral therapy | If obese: Metformin + Insulin Review after 1 month | - STOP Glibenclamide when starting insulin therapy - Counsel about risk of hypoglycemia |
| Step 5 | Insulin Only 0.5 U/kg/day, BD according to the rules of ‘thirds’ Review after 1 month | - Insulin is 1st line therapy for Type I Diabetes - Counsel about risk of hypoglycemia |

## Slide 16
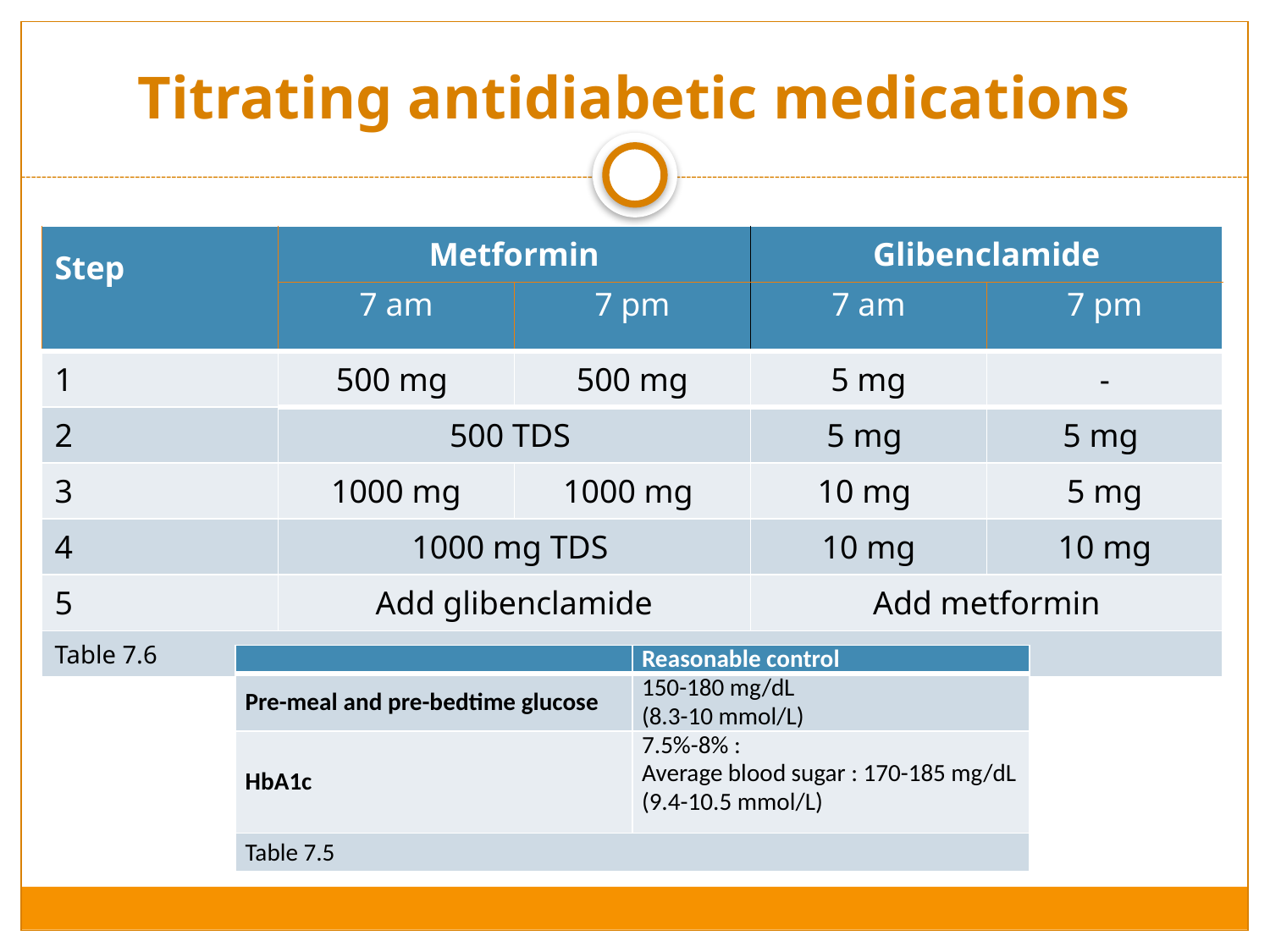

# Titrating antidiabetic medications
| Step | Metformin | | Glibenclamide | |
| --- | --- | --- | --- | --- |
| | 7 am | 7 pm | 7 am | 7 pm |
| 1 | 500 mg | 500 mg | 5 mg | - |
| 2 | 500 TDS | | 5 mg | 5 mg |
| 3 | 1000 mg | 1000 mg | 10 mg | 5 mg |
| 4 | 1000 mg TDS | | 10 mg | 10 mg |
| 5 | Add glibenclamide | | Add metformin | |
| Table 7.6 | | | | |
| | Reasonable control |
| --- | --- |
| Pre-meal and pre-bedtime glucose | 150-180 mg/dL (8.3-10 mmol/L) |
| HbA1c | 7.5%-8% : Average blood sugar : 170-185 mg/dL (9.4-10.5 mmol/L) |
| Table 7.5 | |

## Slide 17
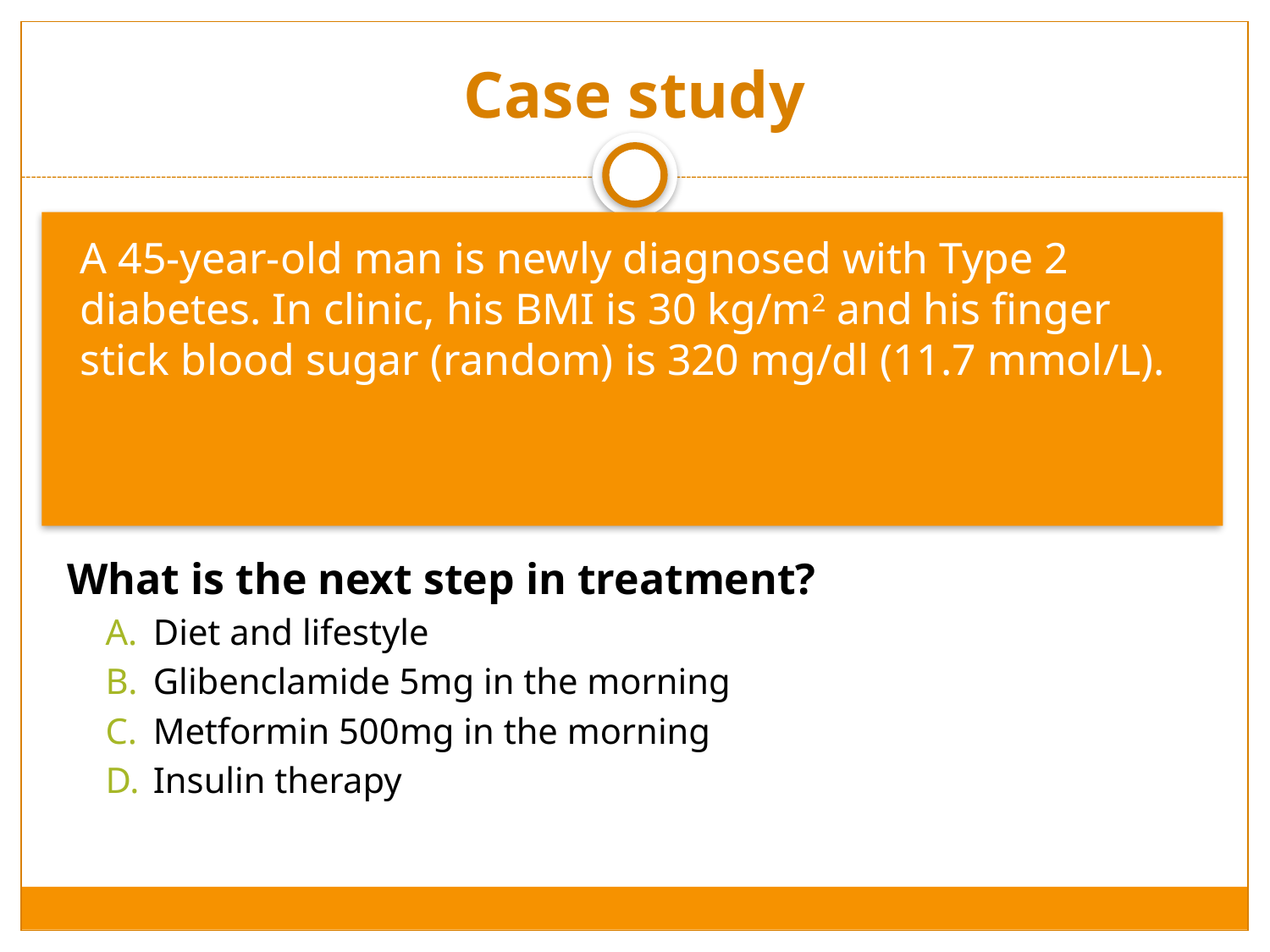

# Case study
A 45-year-old man is newly diagnosed with Type 2 diabetes. In clinic, his BMI is 30 kg/m2 and his finger stick blood sugar (random) is 320 mg/dl (11.7 mmol/L).
What is the next step in treatment?
Diet and lifestyle
Glibenclamide 5mg in the morning
Metformin 500mg in the morning
Insulin therapy

## Slide 18
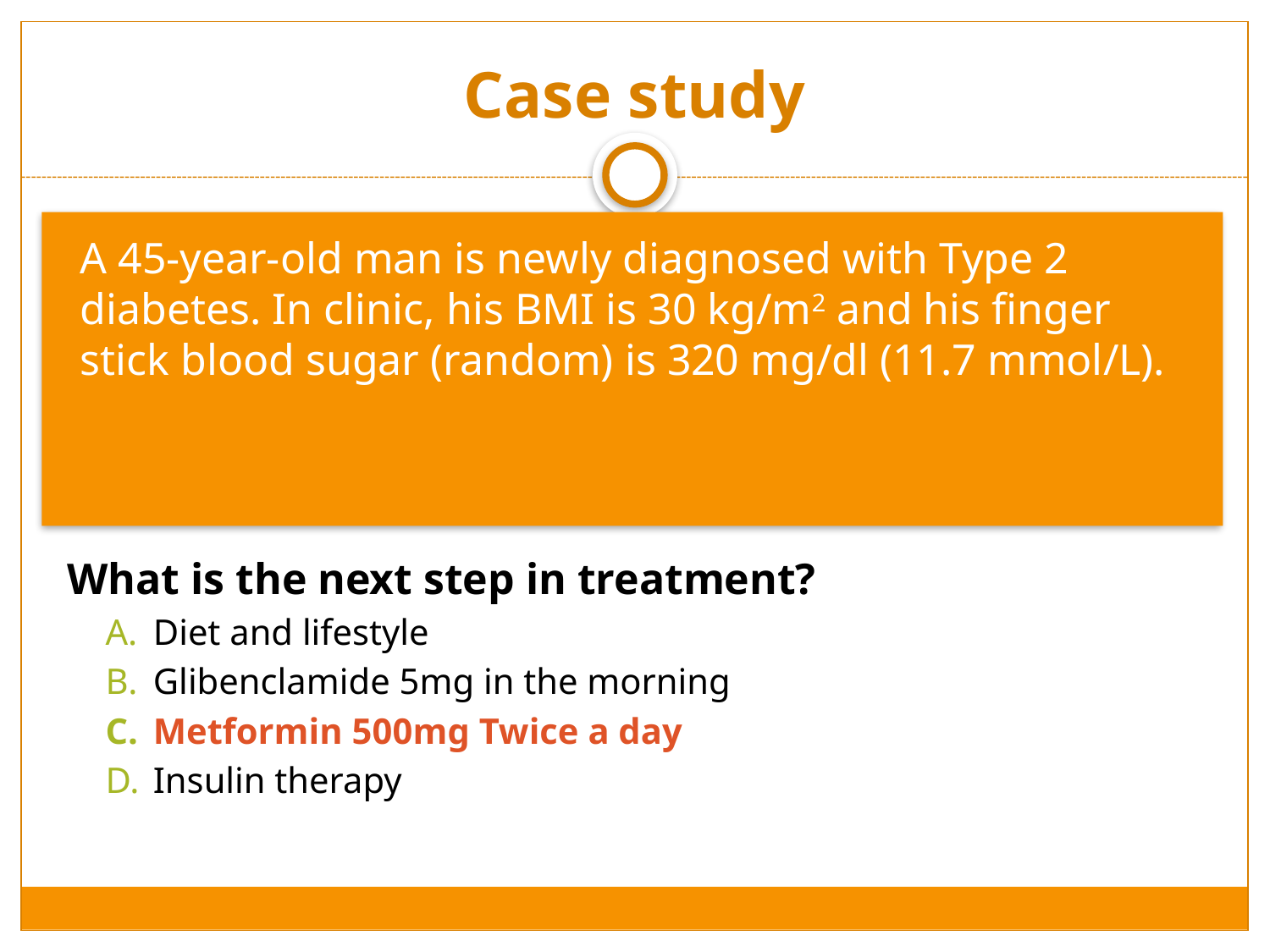

# Case study
A 45-year-old man is newly diagnosed with Type 2 diabetes. In clinic, his BMI is 30 kg/m2 and his finger stick blood sugar (random) is 320 mg/dl (11.7 mmol/L).
What is the next step in treatment?
Diet and lifestyle
Glibenclamide 5mg in the morning
Metformin 500mg Twice a day
Insulin therapy

## Slide 19
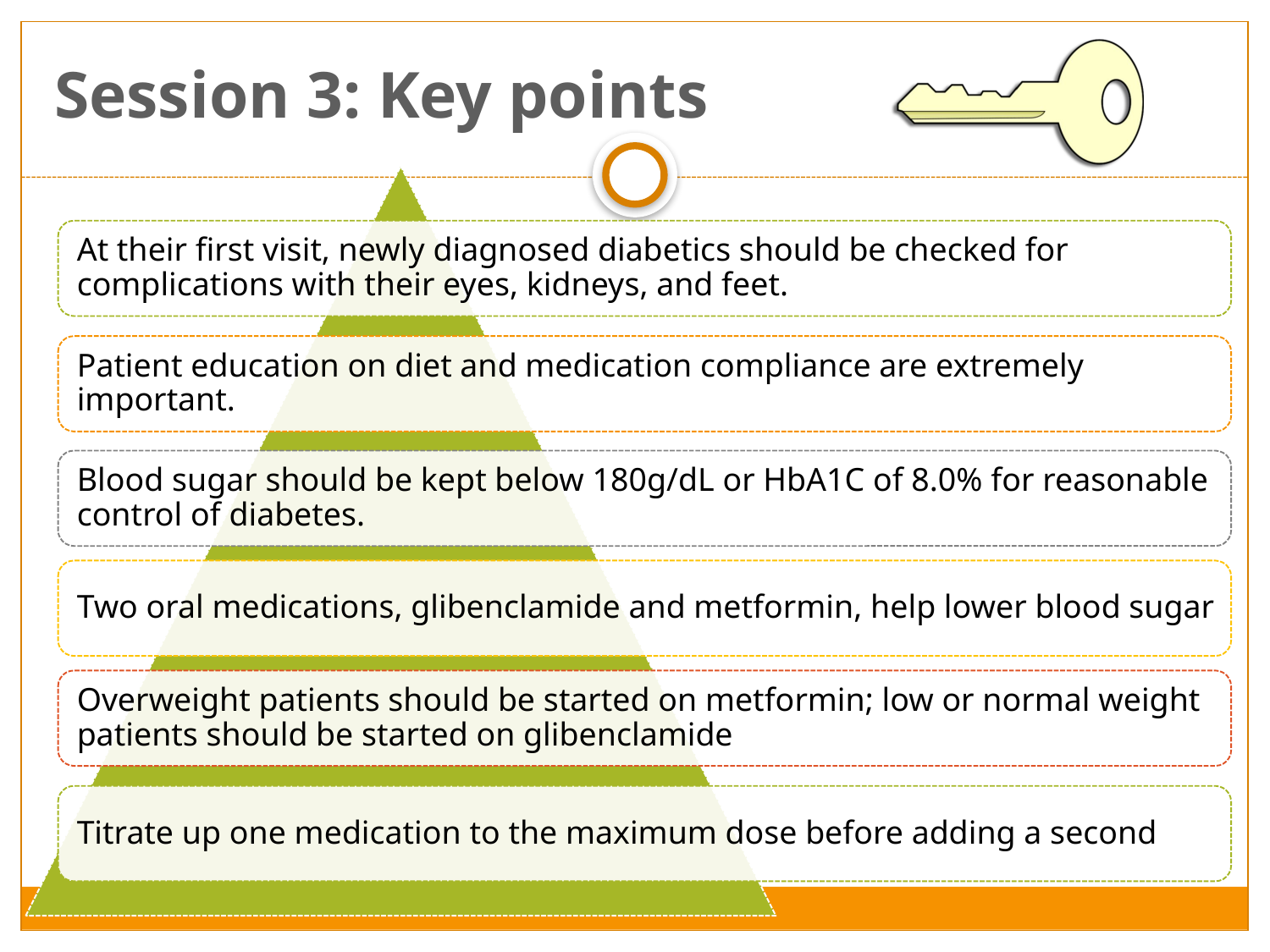

# Session 3: Key points
