# Supplementary material for: Training Mid-Level Providers to Treat Severe Non-Communicable Diseases in Neno, Malawi through PEN-Plus Strategies
Source: Ann Glob Health. 2022 Aug 11;88(1):69. doi: 10.5334/aogh.3750 (PMC9389951; doi:10.5334/aogh.3750)
Supplement: Didactic Materials. — The supplementary materials contain a suggested didactic training schedule and the PowerPoint presentations used for PEN-Plus training in Neno, Malawi. These materials have been reviewed and accepted by the Malawi Ministry of Health for future PEN-Plus trainings in Malawi. [file agh-88-1-3750-s2.zip › Didactic_Materials/GI_Hepatitis & Cirrhosis.pptx]

## Slide 1
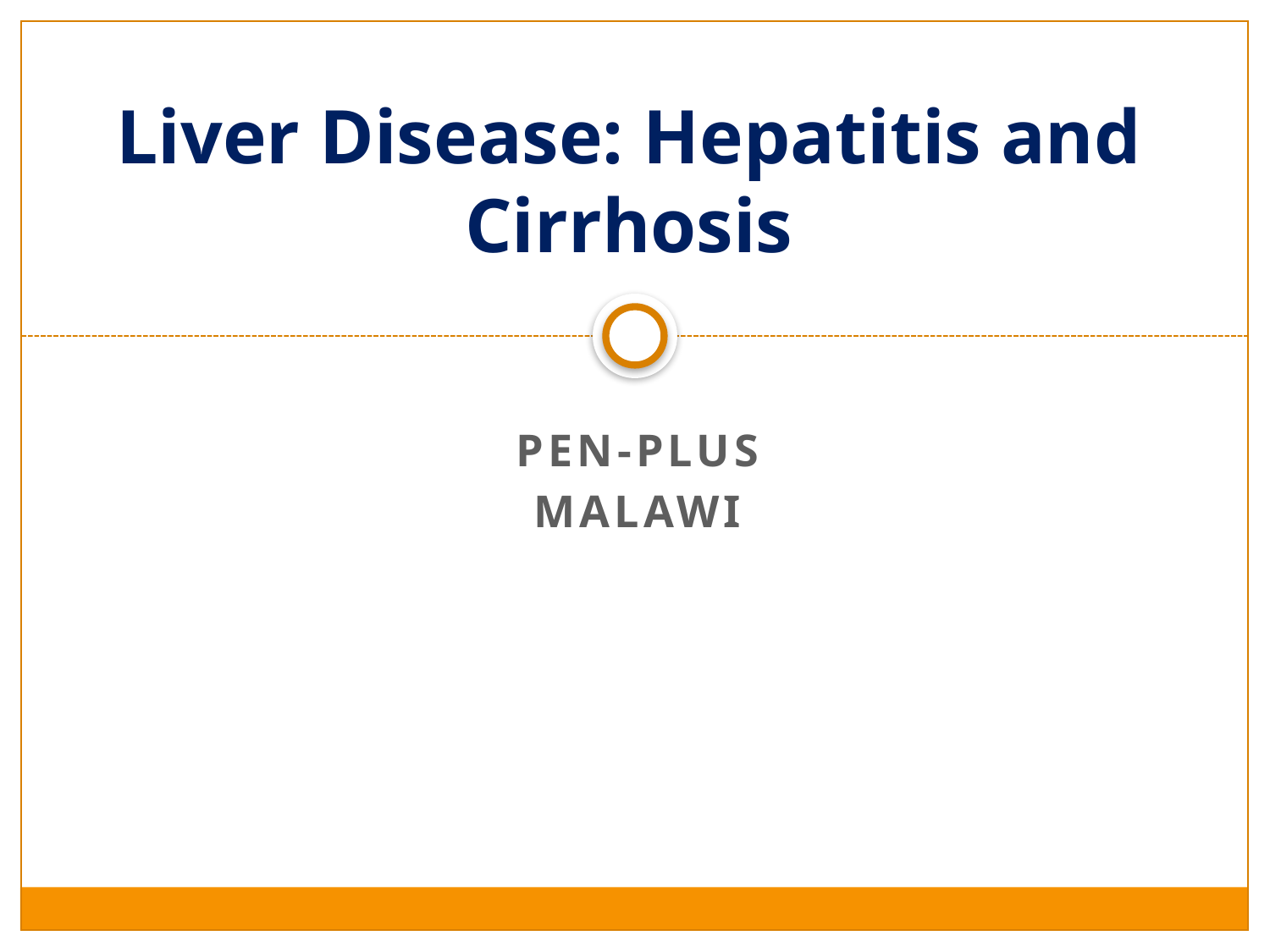

# Liver Disease: Hepatitis and Cirrhosis
PEN-Plus
Malawi

## Slide 2
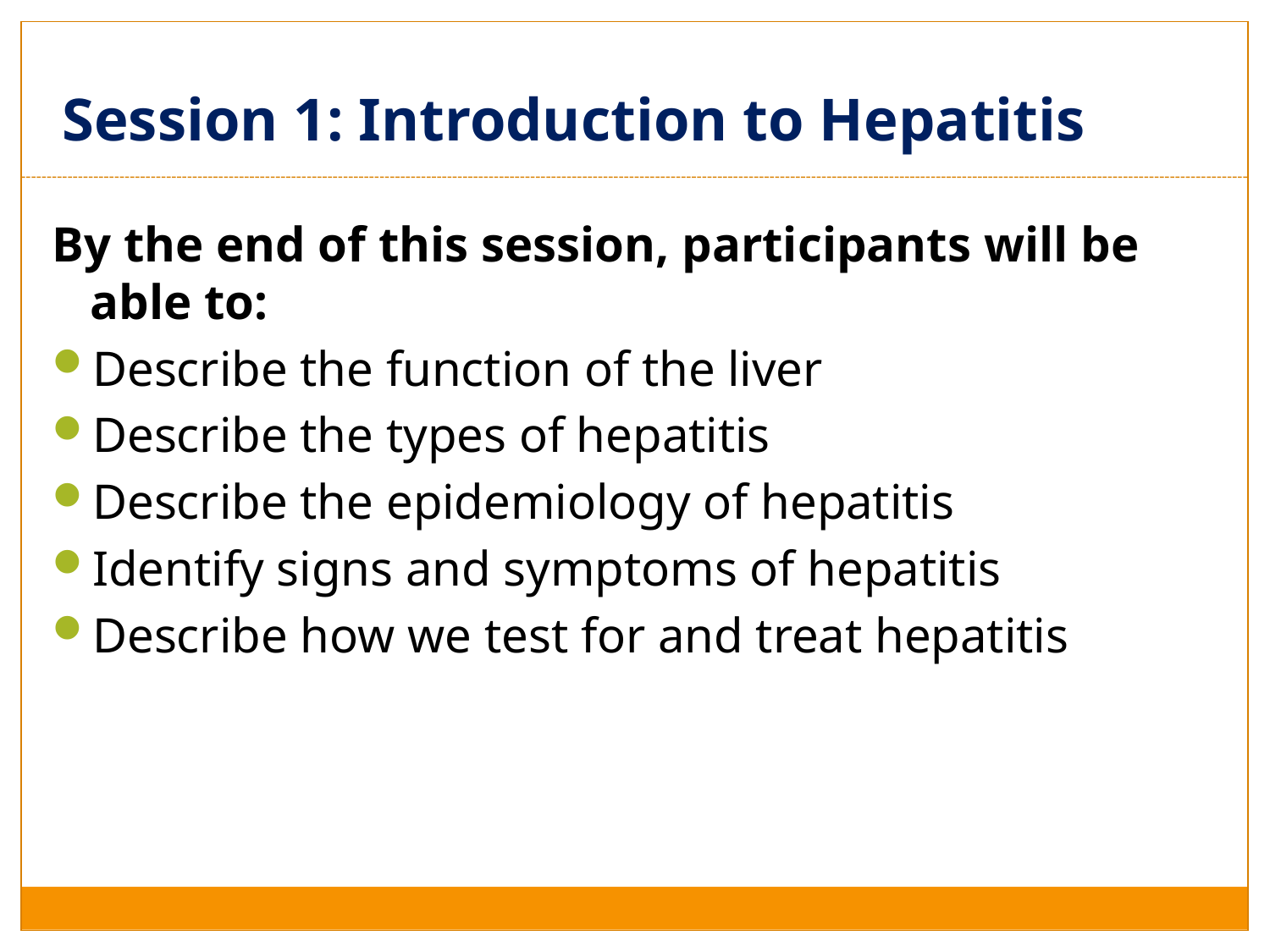

# Session 1: Introduction to Hepatitis
By the end of this session, participants will be able to:
Describe the function of the liver
Describe the types of hepatitis
Describe the epidemiology of hepatitis
Identify signs and symptoms of hepatitis
Describe how we test for and treat hepatitis

## Slide 3
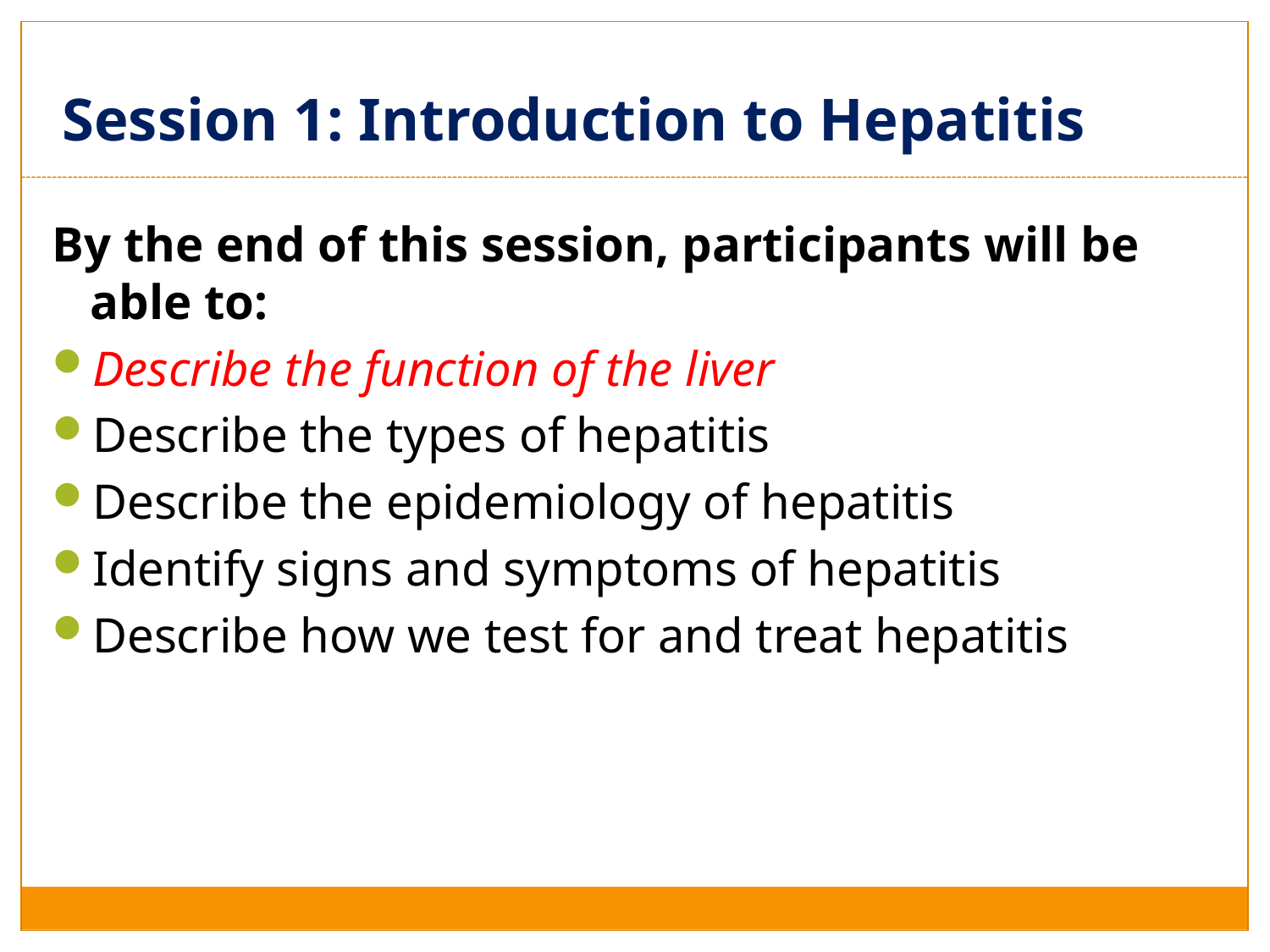

# Session 1: Introduction to Hepatitis
By the end of this session, participants will be able to:
Describe the function of the liver
Describe the types of hepatitis
Describe the epidemiology of hepatitis
Identify signs and symptoms of hepatitis
Describe how we test for and treat hepatitis

## Slide 4
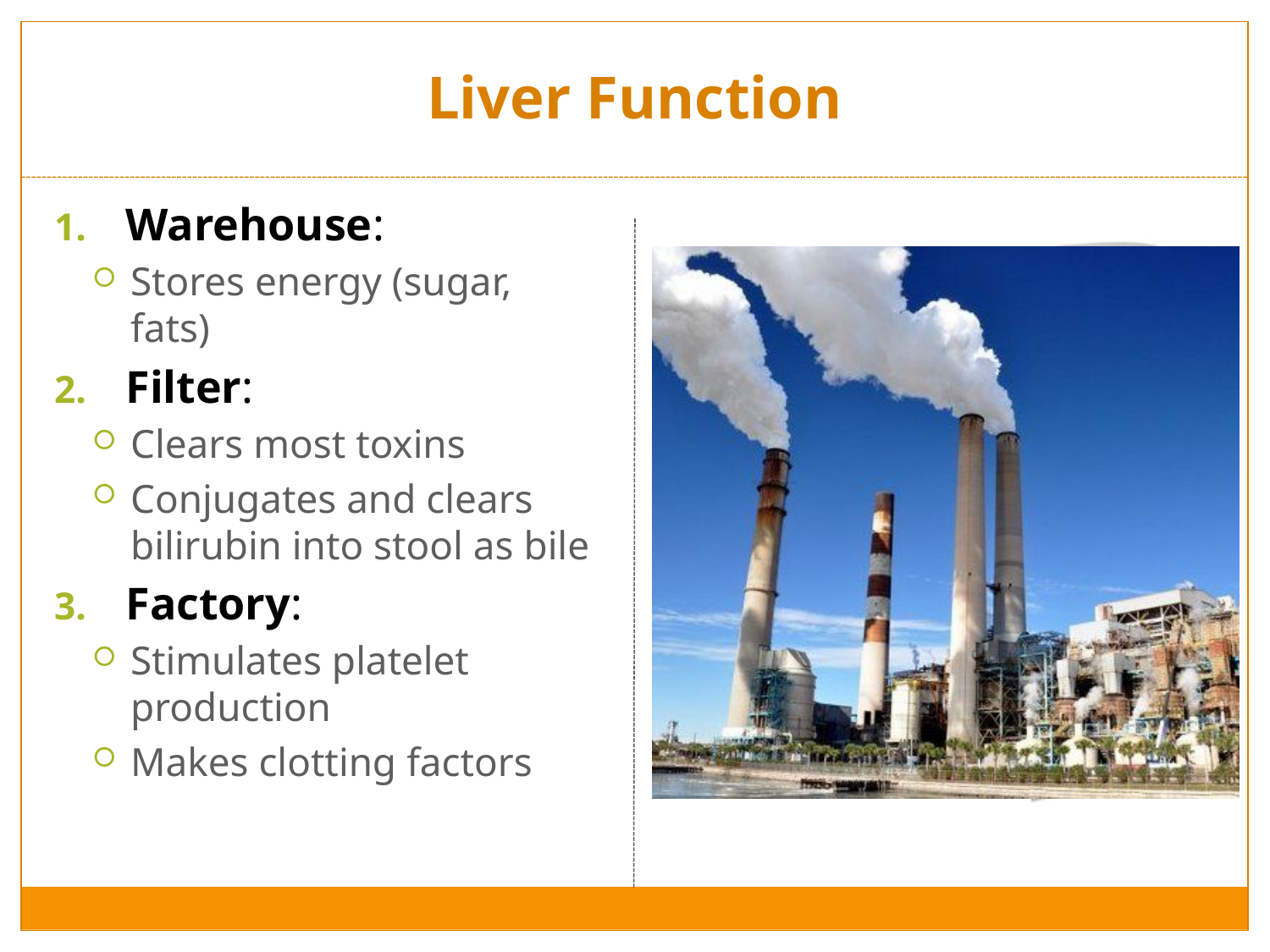

# Liver Function
Warehouse:
Stores energy (sugar, fats)
Filter:
Clears most toxins
Conjugates and clears bilirubin into stool as bile
Factory:
Stimulates platelet production
Makes clotting factors

## Slide 5
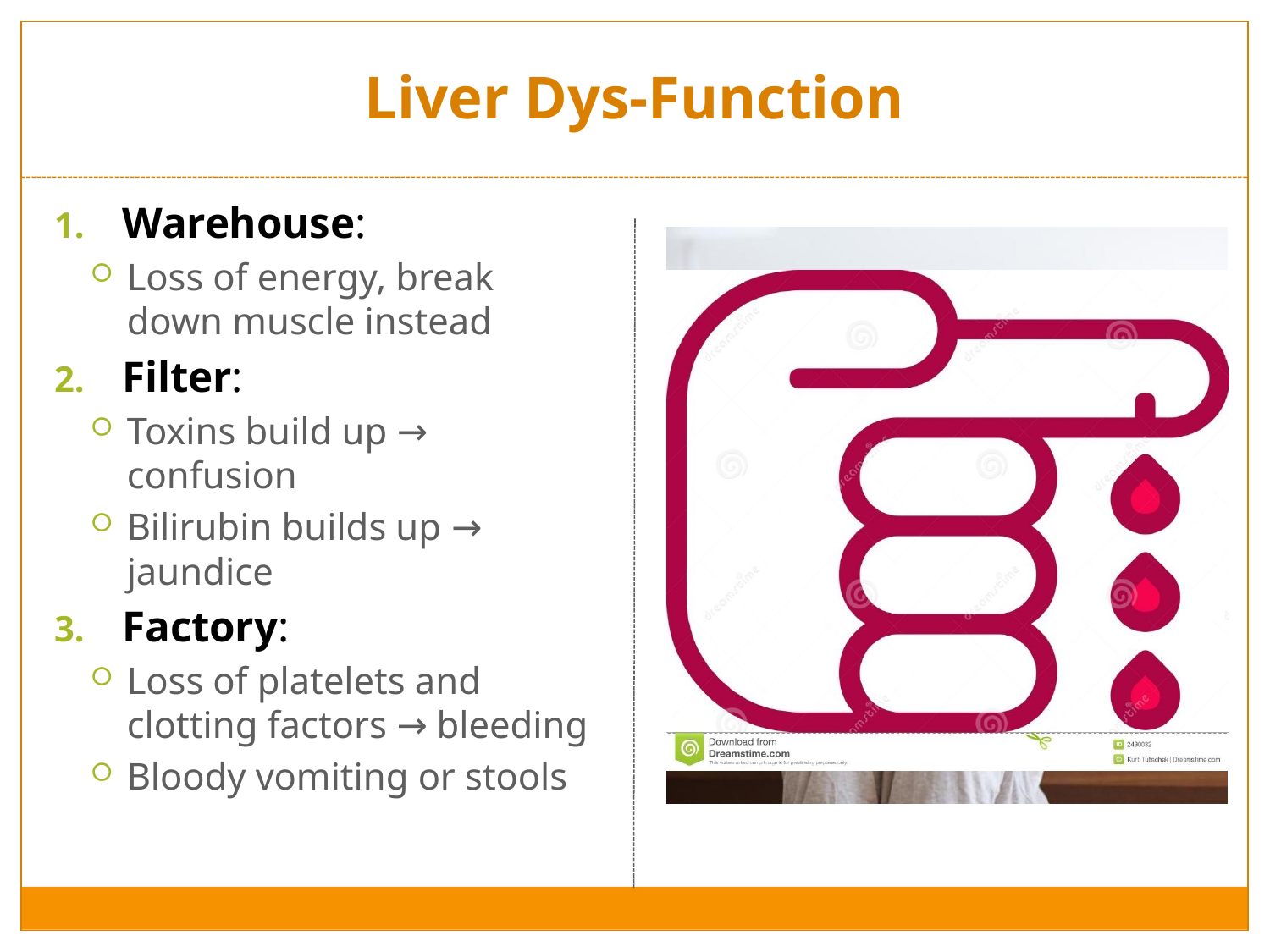

# Liver Dys-Function
Warehouse:
Loss of energy, break down muscle instead
Filter:
Toxins build up → confusion
Bilirubin builds up → jaundice
Factory:
Loss of platelets and clotting factors → bleeding
Bloody vomiting or stools

## Slide 6
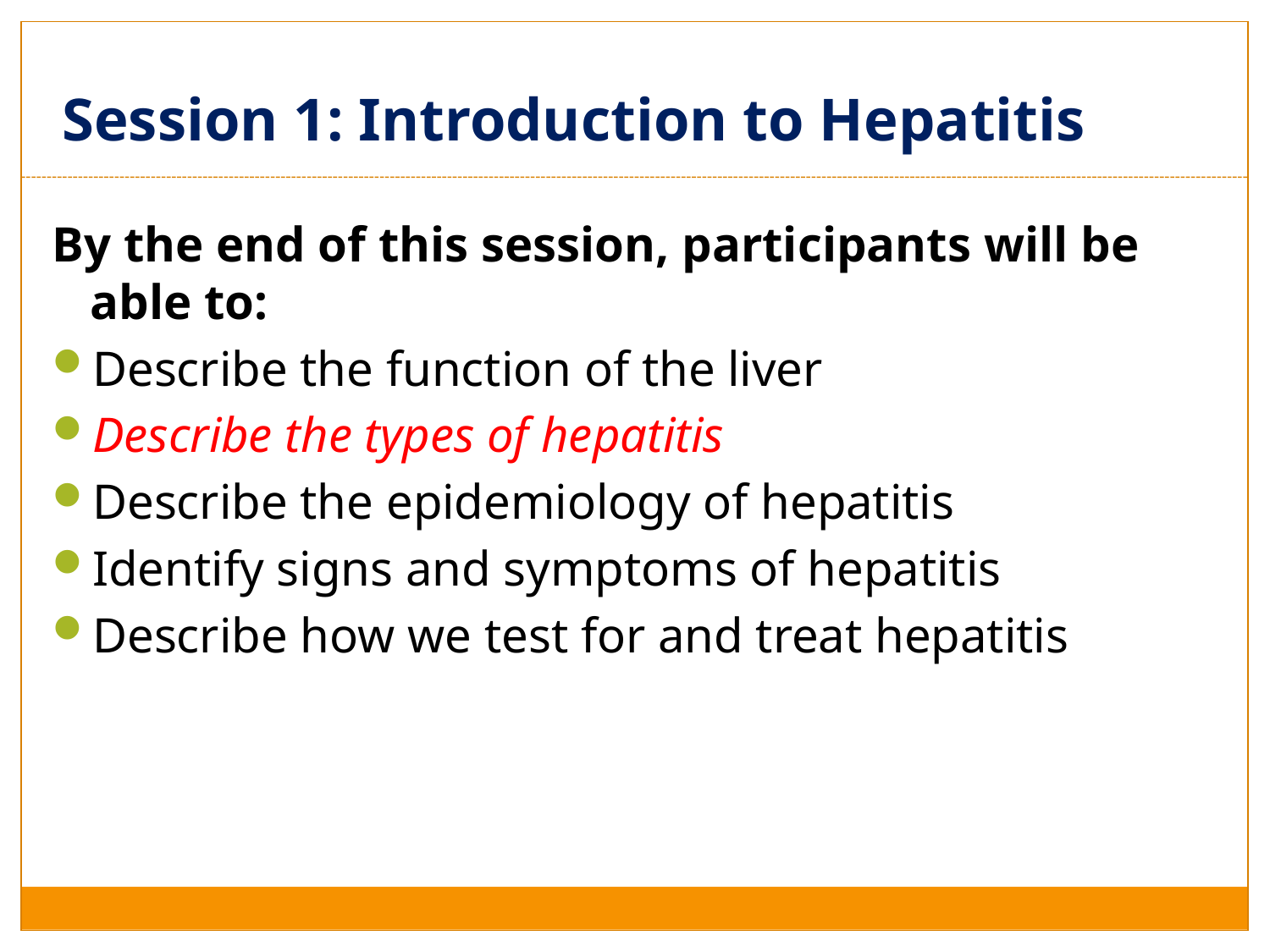

# Session 1: Introduction to Hepatitis
By the end of this session, participants will be able to:
Describe the function of the liver
Describe the types of hepatitis
Describe the epidemiology of hepatitis
Identify signs and symptoms of hepatitis
Describe how we test for and treat hepatitis

## Slide 7
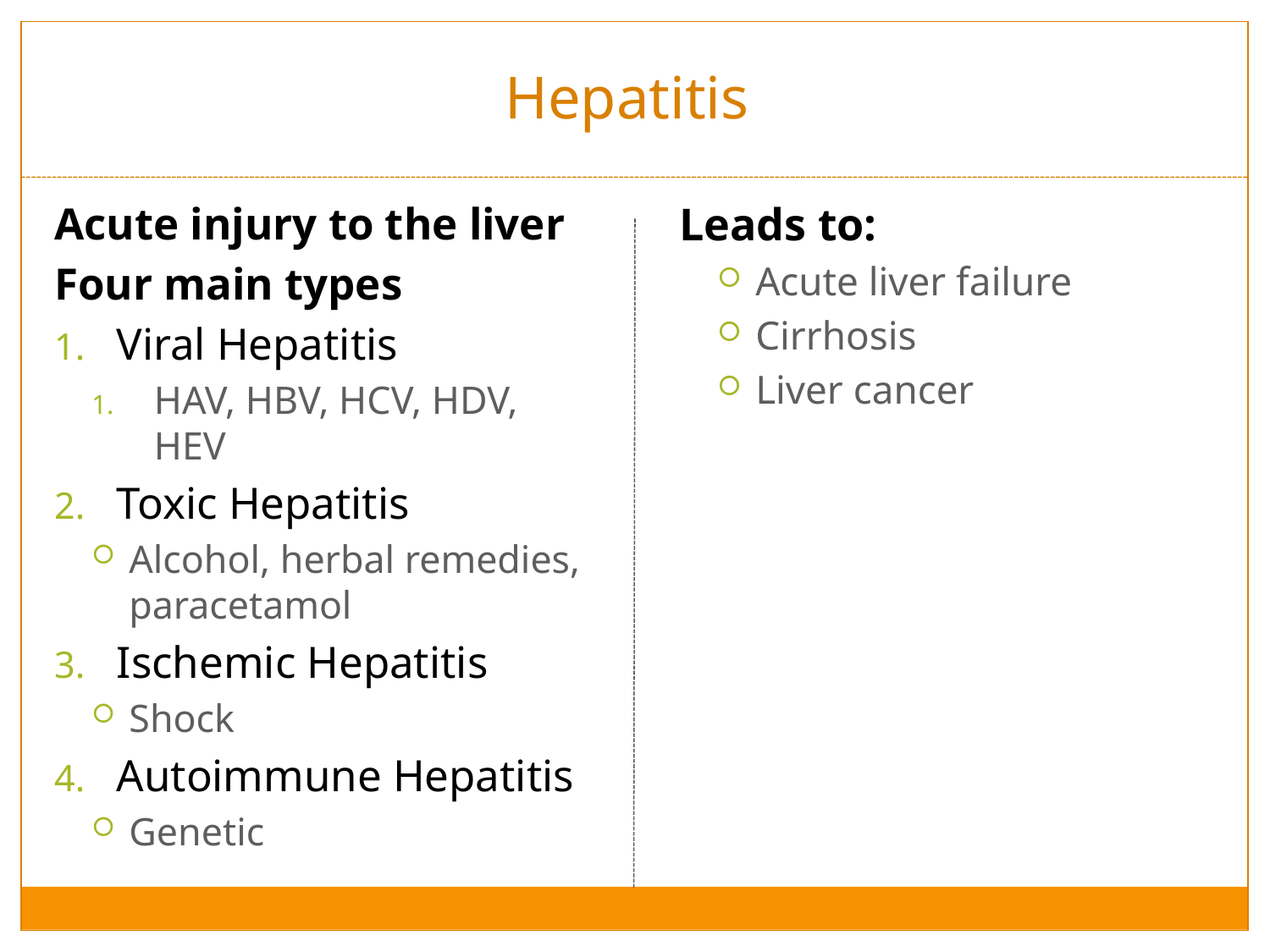

# Hepatitis
Acute injury to the liver
Four main types
Viral Hepatitis
HAV, HBV, HCV, HDV, HEV
Toxic Hepatitis
Alcohol, herbal remedies, paracetamol
Ischemic Hepatitis
Shock
Autoimmune Hepatitis
Genetic
Leads to:
Acute liver failure
Cirrhosis
Liver cancer

## Slide 8
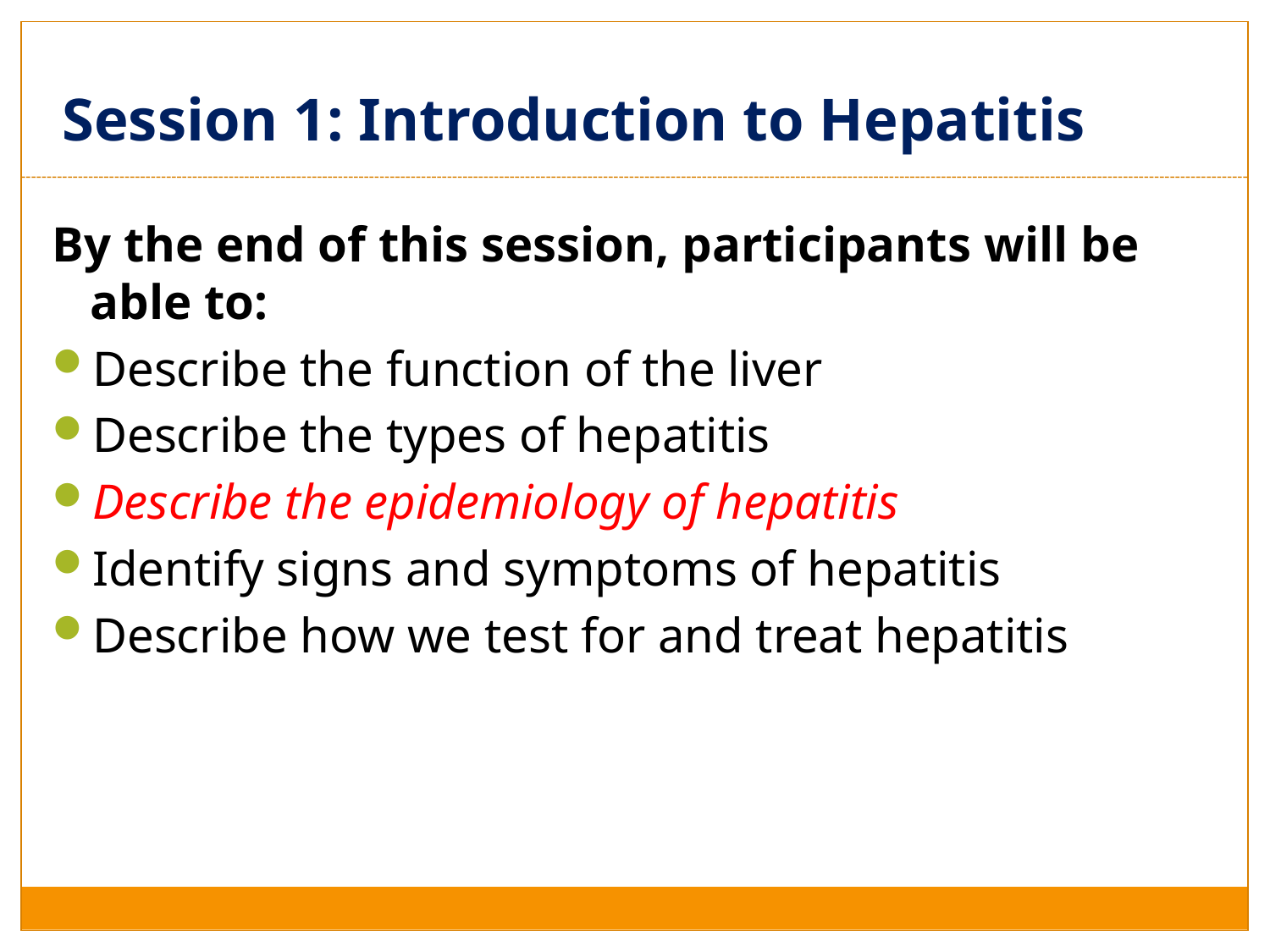

# Session 1: Introduction to Hepatitis
By the end of this session, participants will be able to:
Describe the function of the liver
Describe the types of hepatitis
Describe the epidemiology of hepatitis
Identify signs and symptoms of hepatitis
Describe how we test for and treat hepatitis

## Slide 9
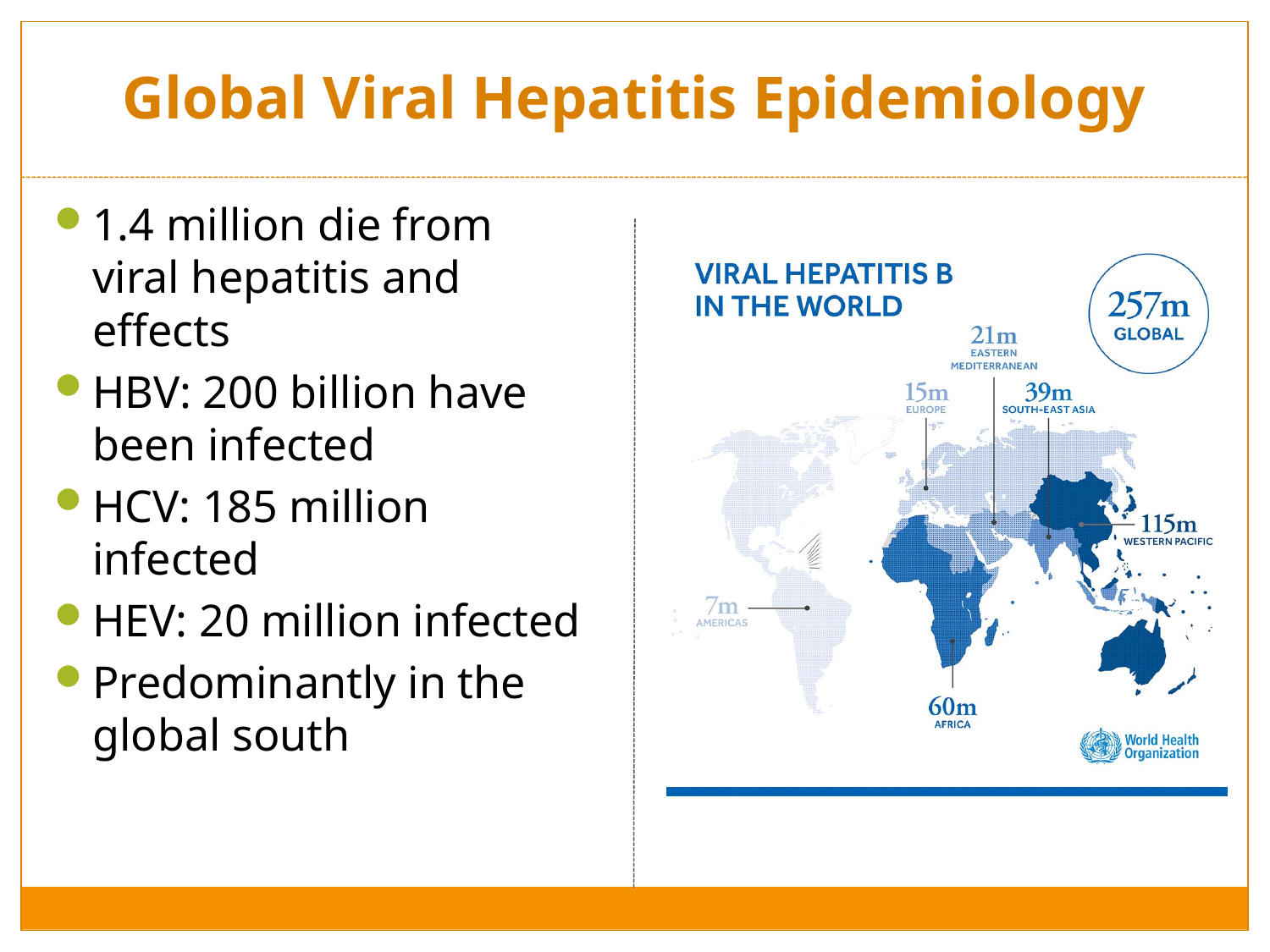

# Global Viral Hepatitis Epidemiology
1.4 million die from viral hepatitis and effects
HBV: 200 billion have been infected
HCV: 185 million infected
HEV: 20 million infected
Predominantly in the global south

## Slide 10
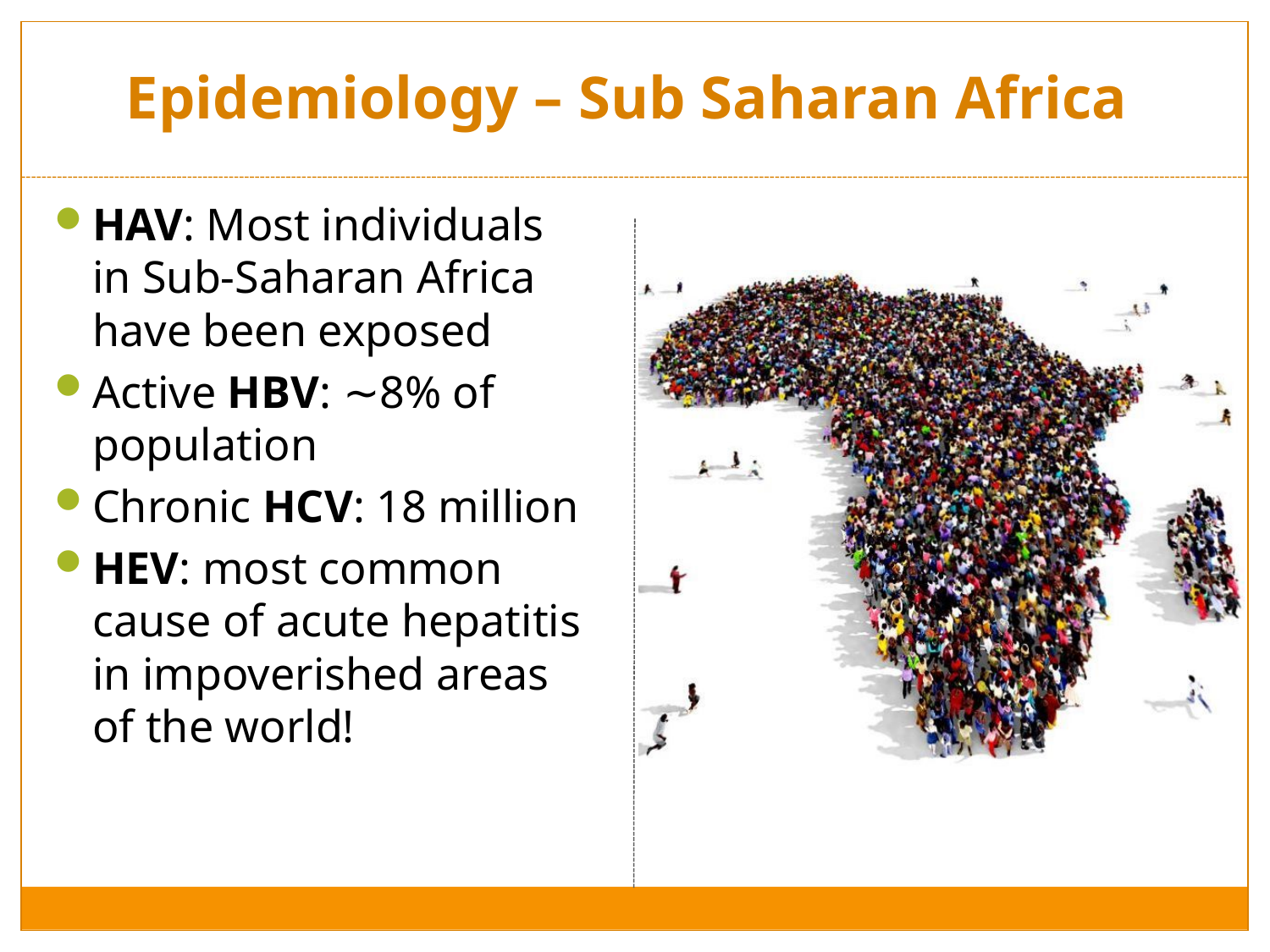

# Epidemiology – Sub Saharan Africa
HAV: Most individuals in Sub-Saharan Africa have been exposed
Active HBV: ∼8% of population
Chronic HCV: 18 million
HEV: most common cause of acute hepatitis in impoverished areas of the world!

## Slide 11
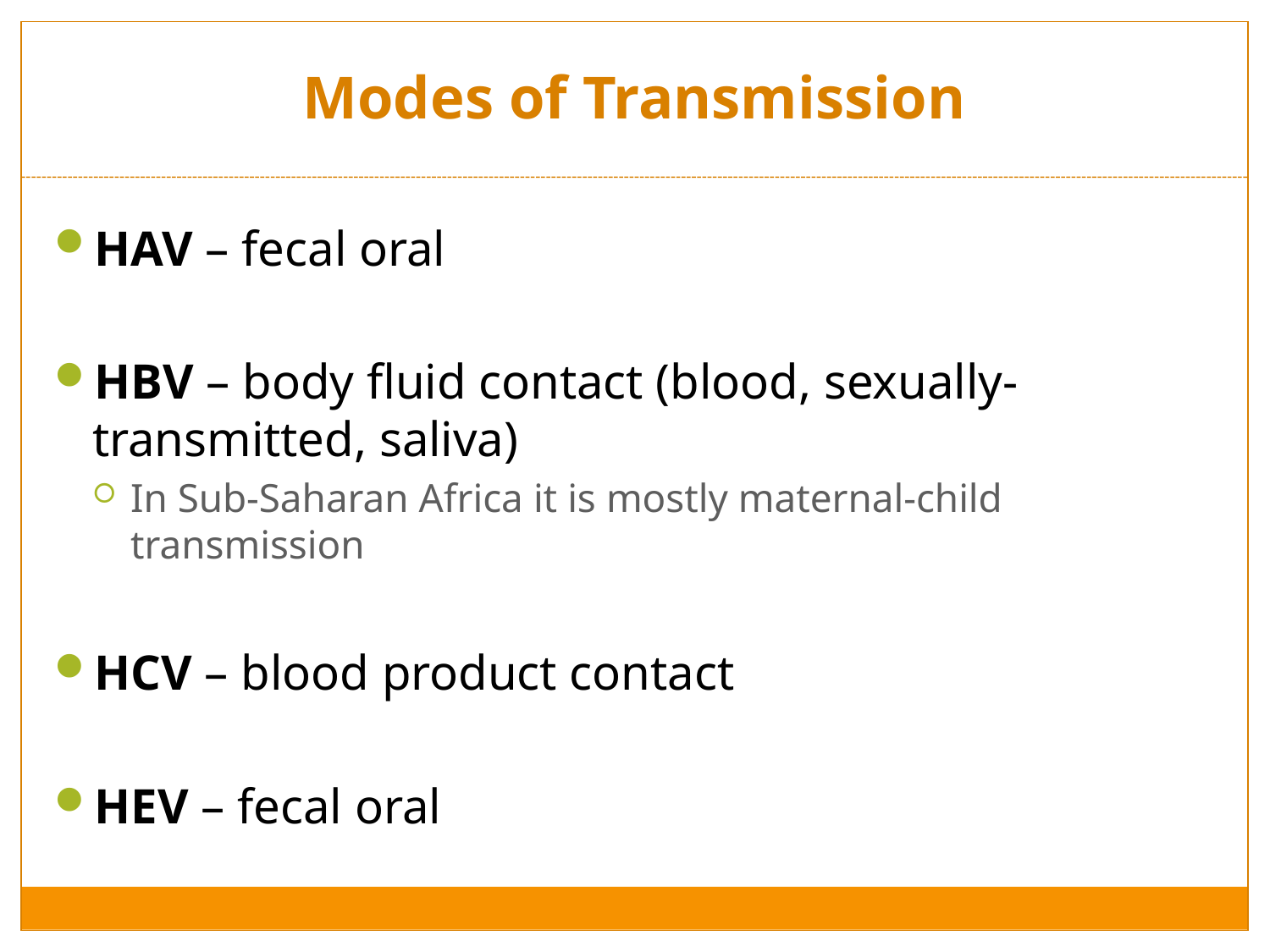

# Modes of Transmission
HAV – fecal oral
HBV – body fluid contact (blood, sexually-transmitted, saliva)
In Sub-Saharan Africa it is mostly maternal-child transmission
HCV – blood product contact
HEV – fecal oral

## Slide 12
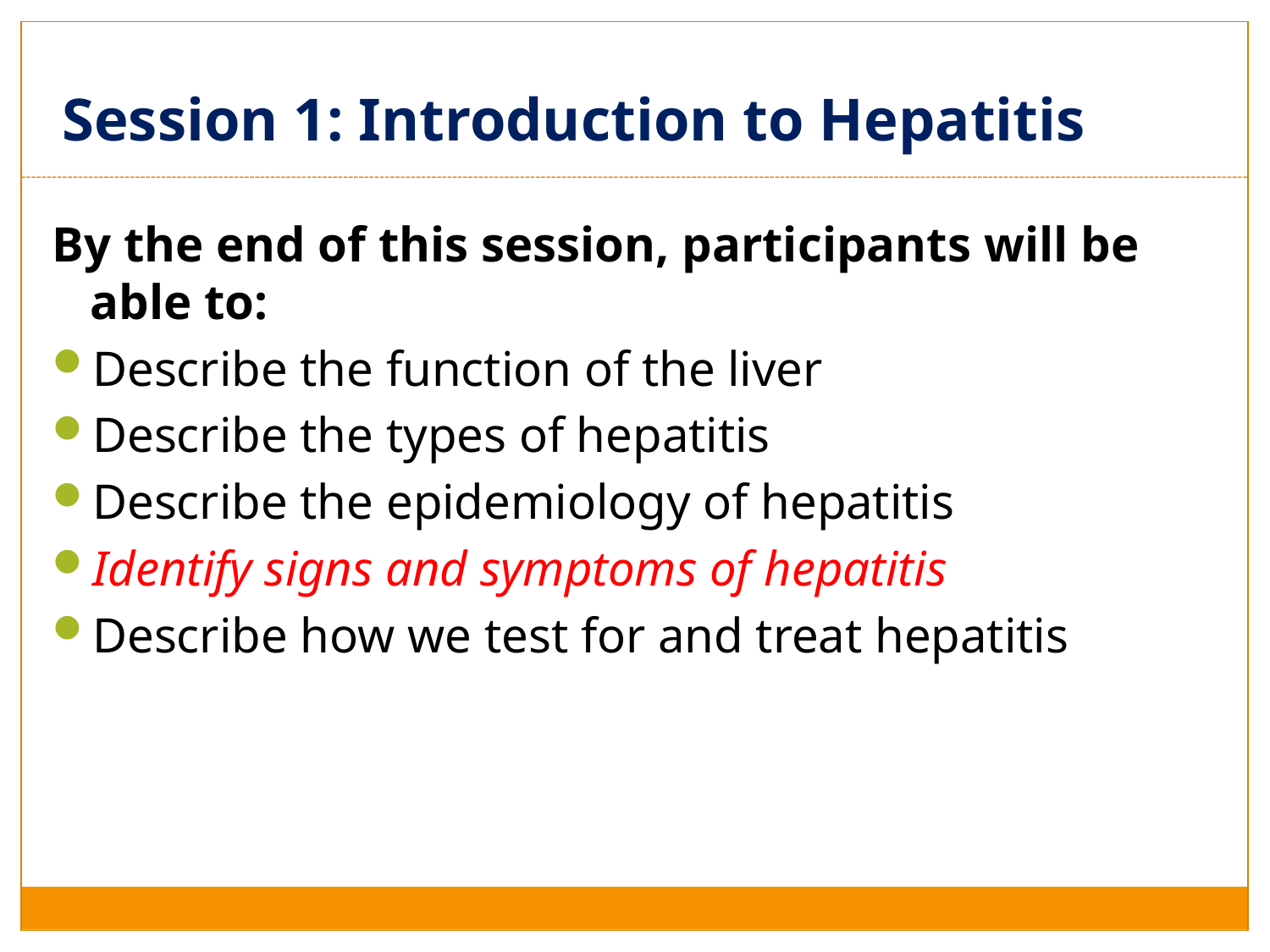

# Session 1: Introduction to Hepatitis
By the end of this session, participants will be able to:
Describe the function of the liver
Describe the types of hepatitis
Describe the epidemiology of hepatitis
Identify signs and symptoms of hepatitis
Describe how we test for and treat hepatitis

## Slide 13
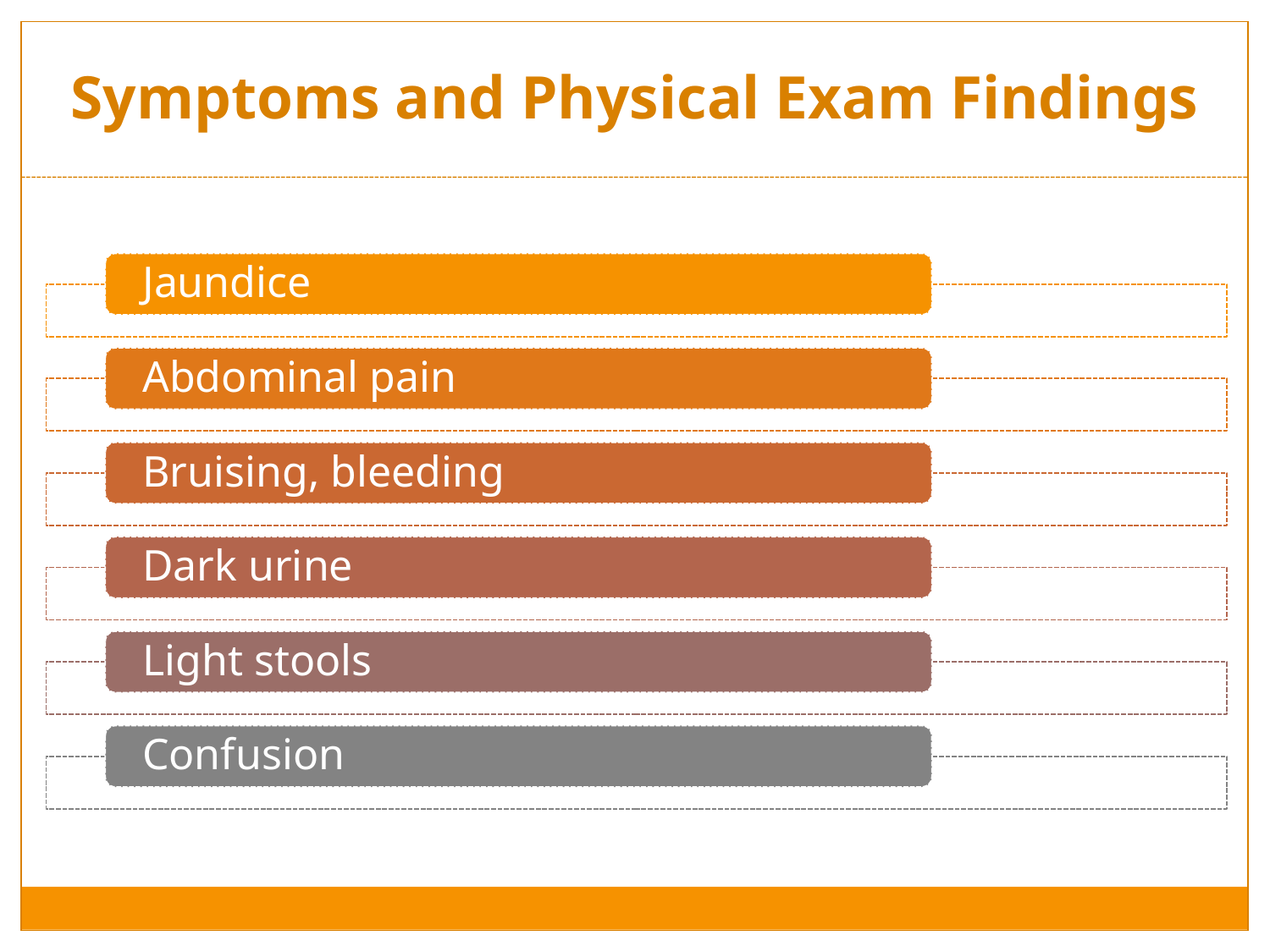

# Symptoms and Physical Exam Findings

## Slide 14
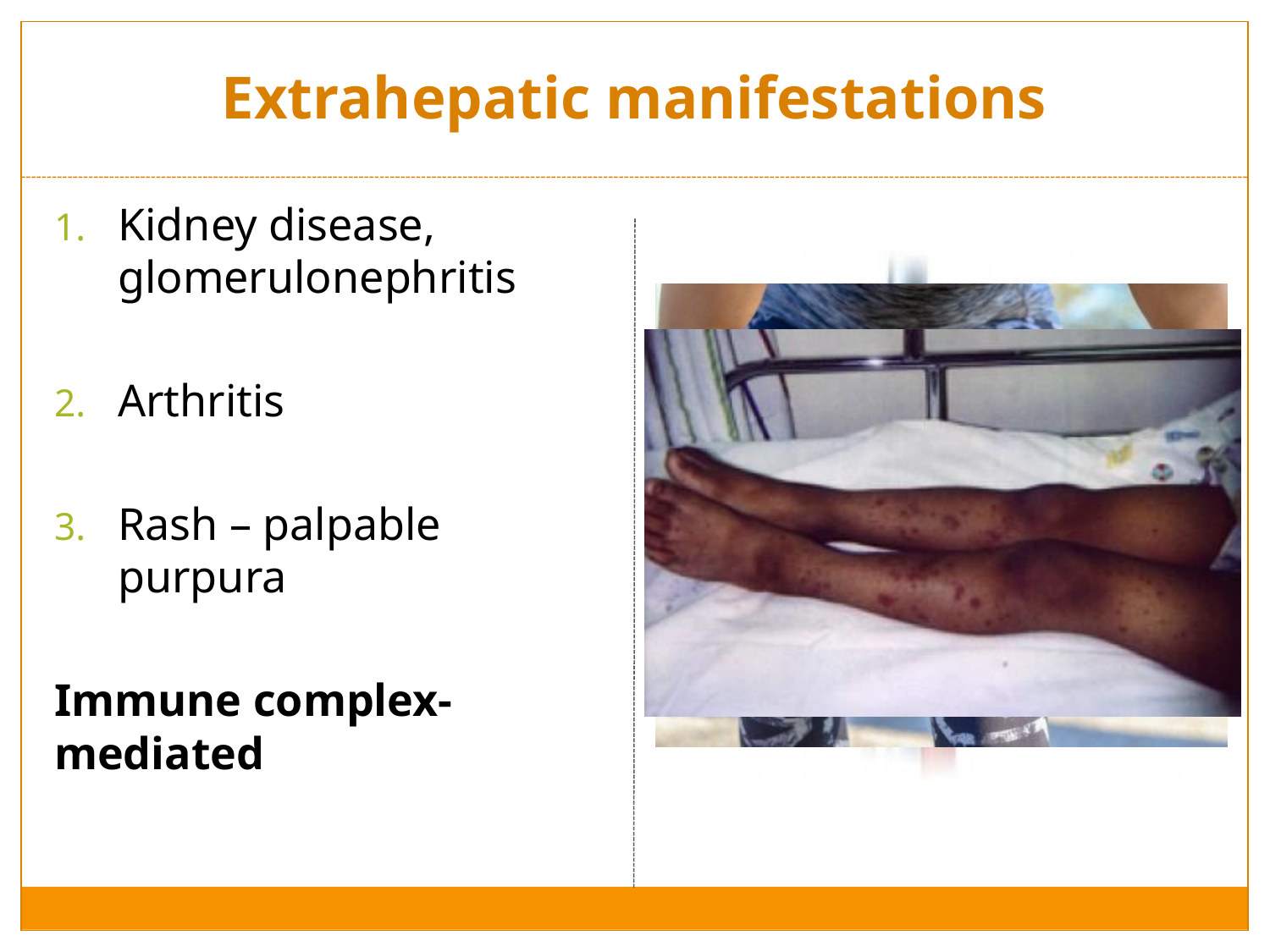

# Extrahepatic manifestations
Kidney disease, glomerulonephritis
Arthritis
Rash – palpable purpura
Immune complex-mediated

## Slide 15
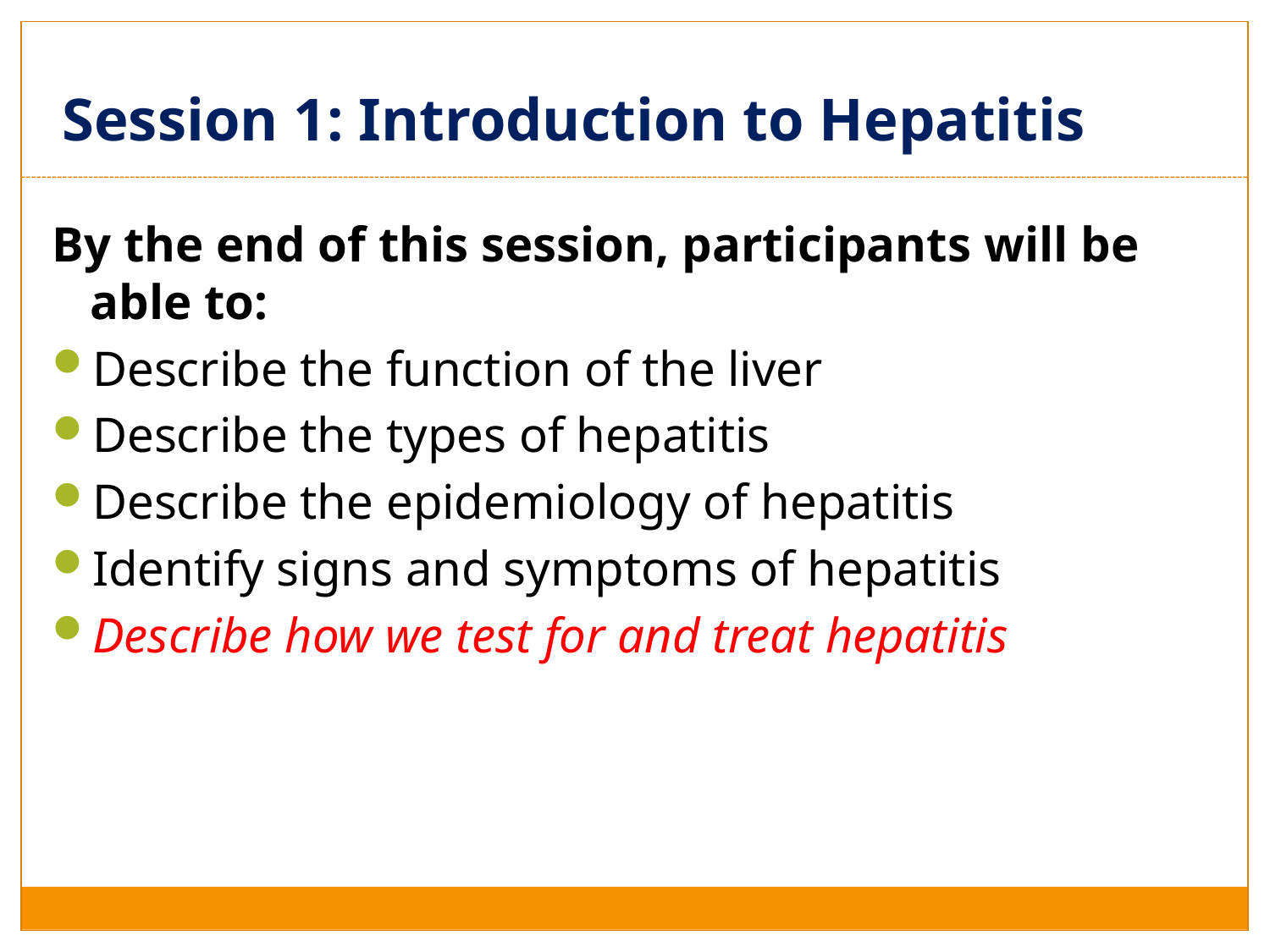

# Session 1: Introduction to Hepatitis
By the end of this session, participants will be able to:
Describe the function of the liver
Describe the types of hepatitis
Describe the epidemiology of hepatitis
Identify signs and symptoms of hepatitis
Describe how we test for and treat hepatitis

## Slide 16
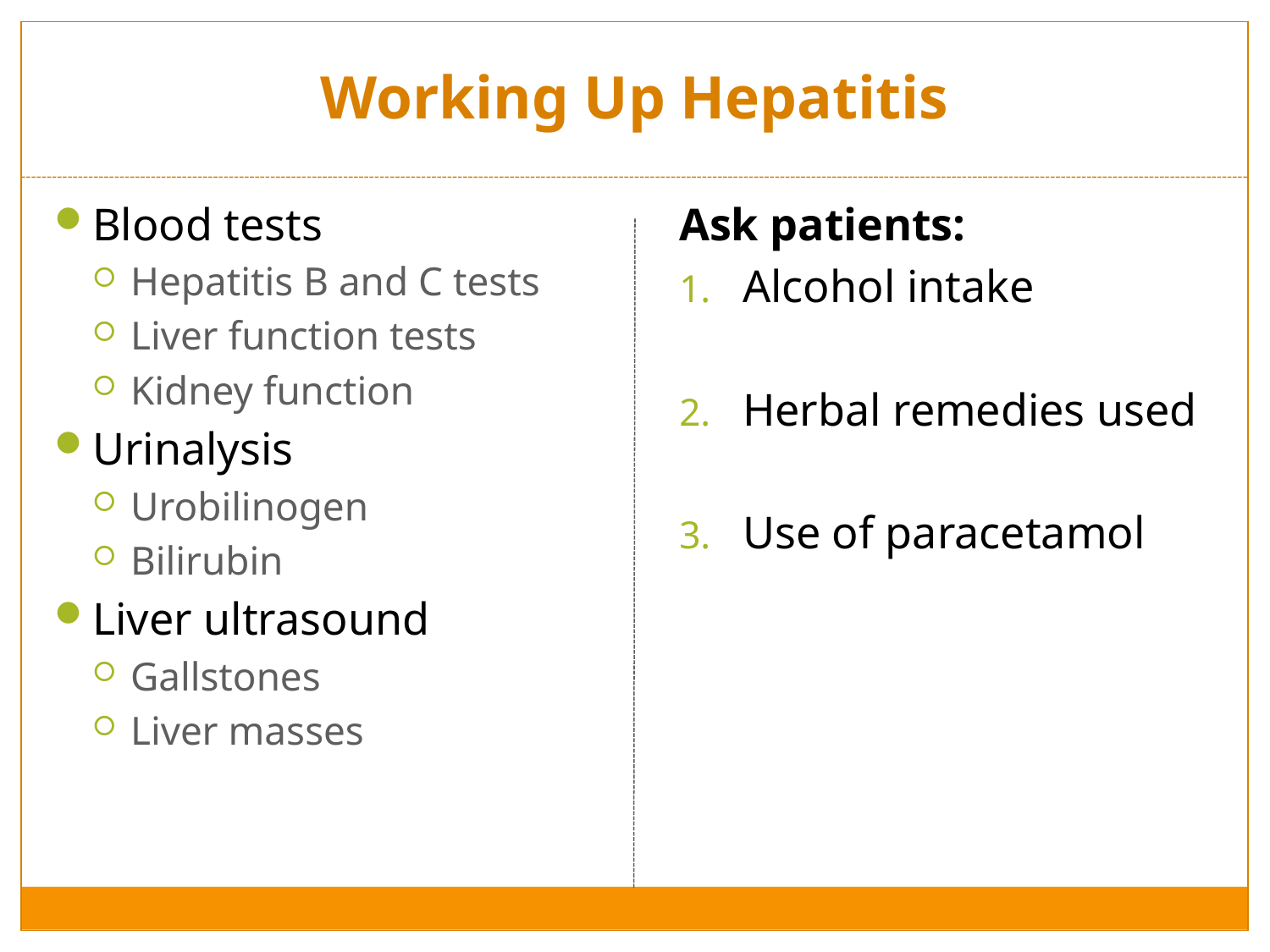

# Working Up Hepatitis
Blood tests
Hepatitis B and C tests
Liver function tests
Kidney function
Urinalysis
Urobilinogen
Bilirubin
Liver ultrasound
Gallstones
Liver masses
Ask patients:
Alcohol intake
Herbal remedies used
Use of paracetamol

## Slide 17
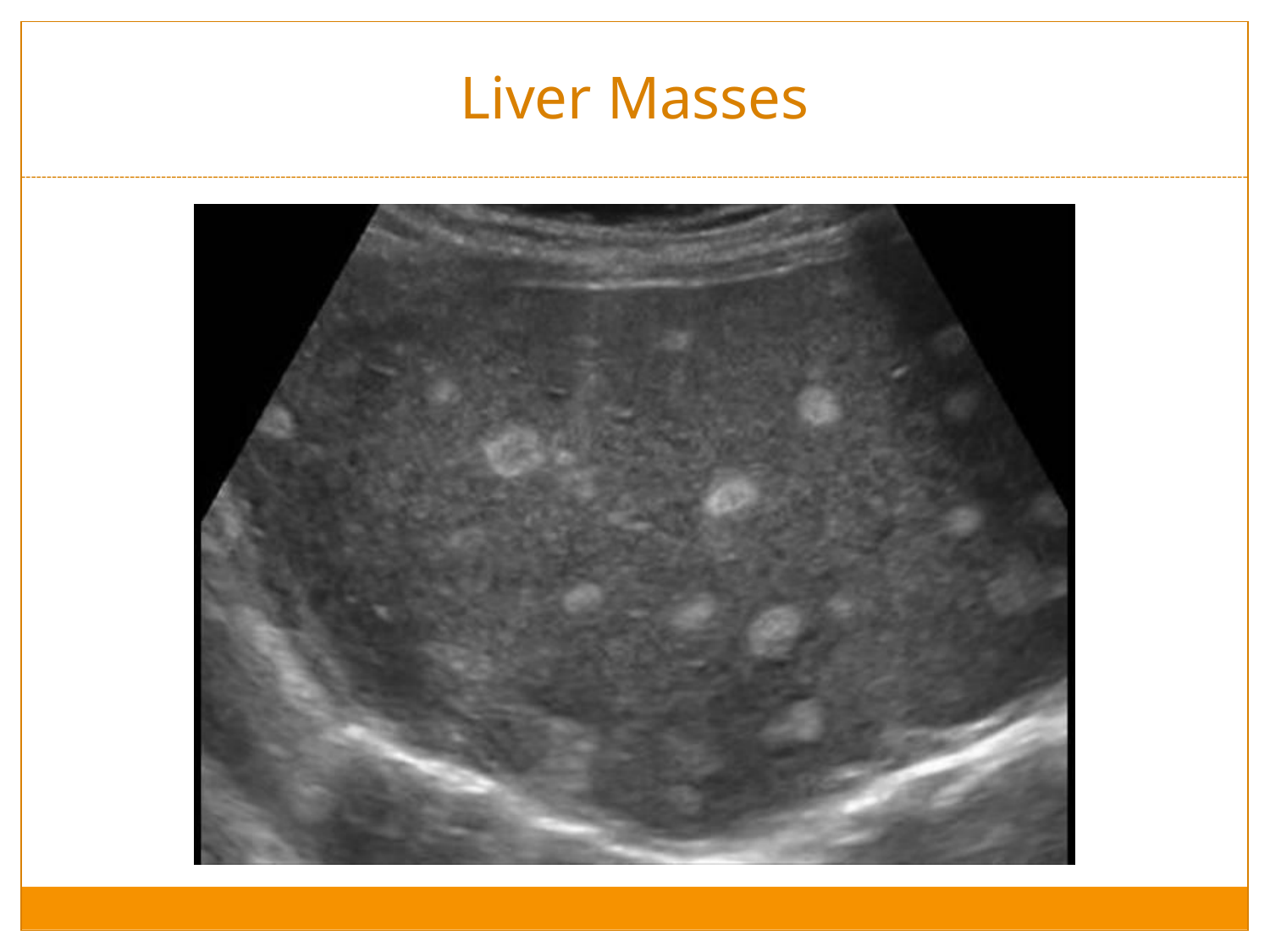

# Liver Masses

## Slide 18
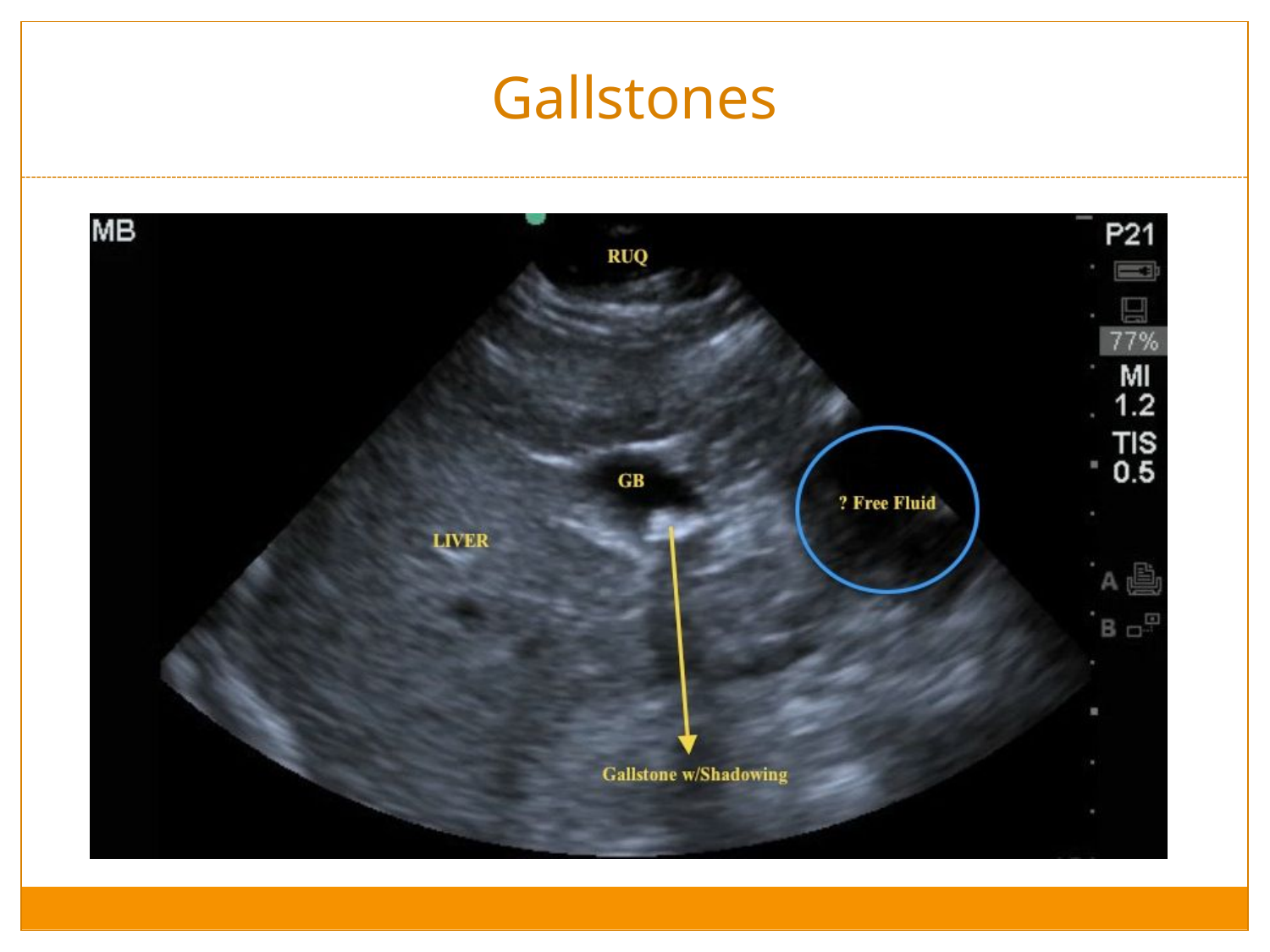

# Gallstones

## Slide 19
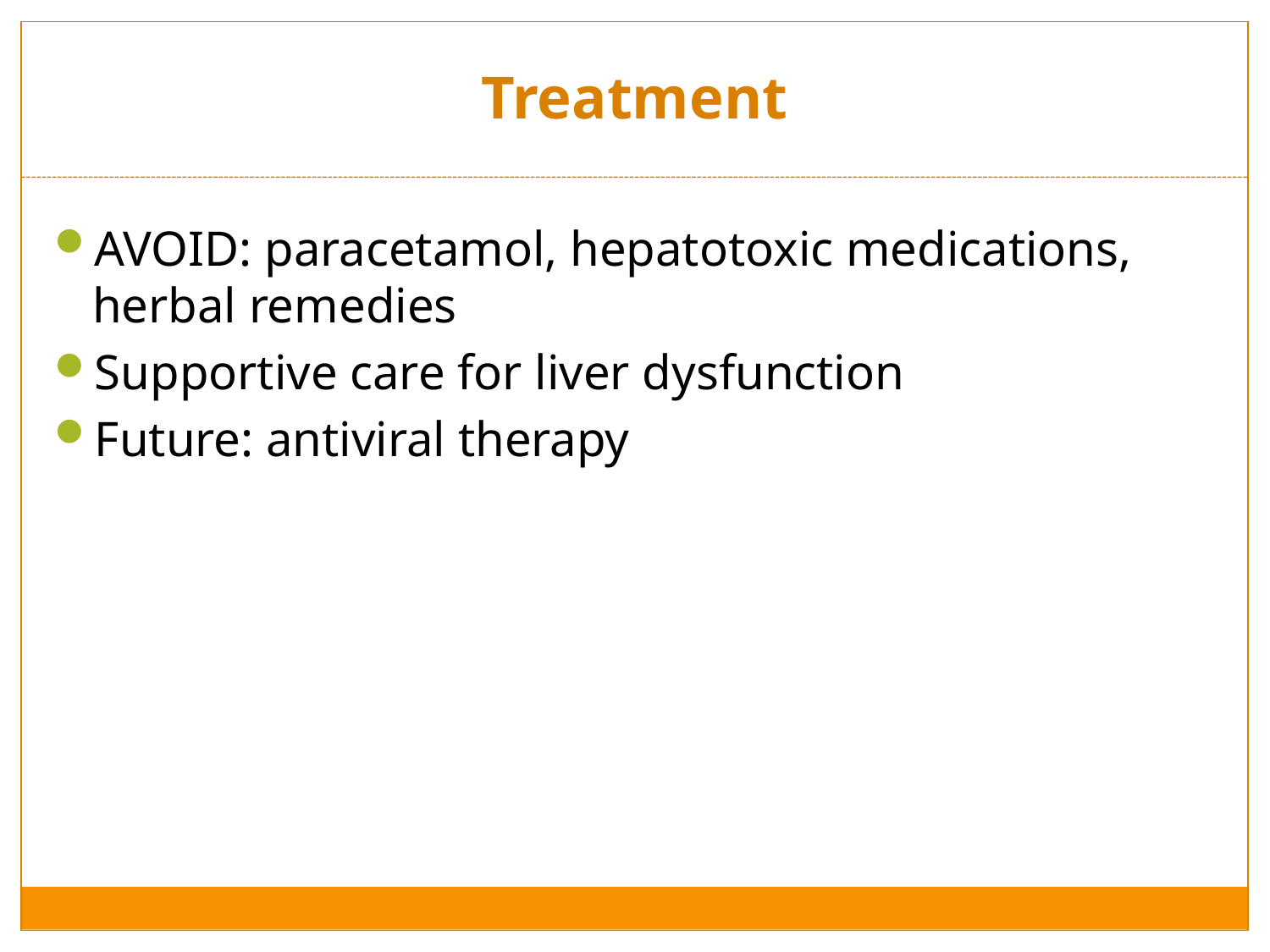

# Treatment
AVOID: paracetamol, hepatotoxic medications, herbal remedies
Supportive care for liver dysfunction
Future: antiviral therapy

## Slide 20
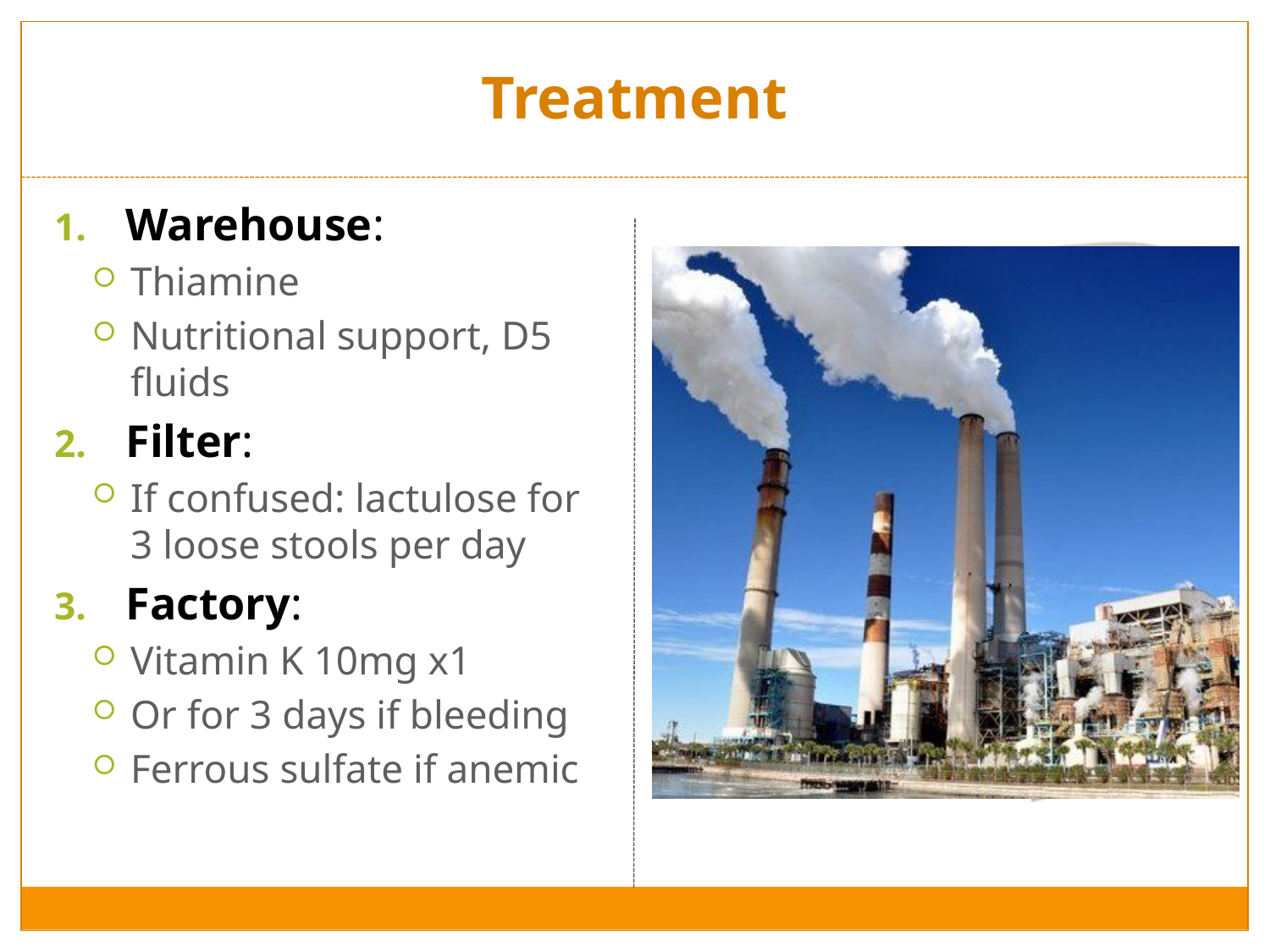

# Treatment
Warehouse:
Thiamine
Nutritional support, D5 fluids
Filter:
If confused: lactulose for 3 loose stools per day
Factory:
Vitamin K 10mg x1
Or for 3 days if bleeding
Ferrous sulfate if anemic

## Slide 21
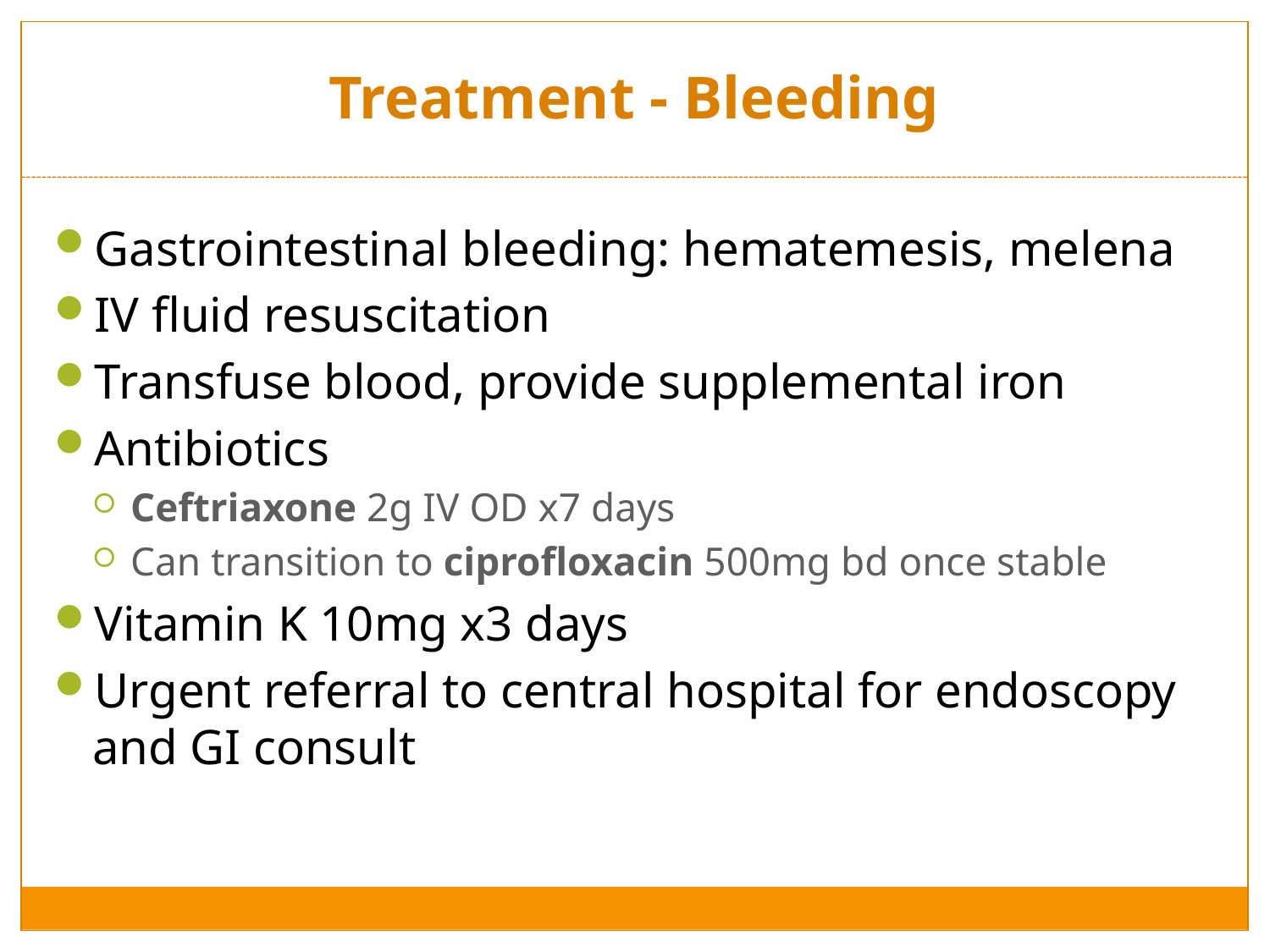

# Treatment - Bleeding
Gastrointestinal bleeding: hematemesis, melena
IV fluid resuscitation
Transfuse blood, provide supplemental iron
Antibiotics
Ceftriaxone 2g IV OD x7 days
Can transition to ciprofloxacin 500mg bd once stable
Vitamin K 10mg x3 days
Urgent referral to central hospital for endoscopy and GI consult

## Slide 22
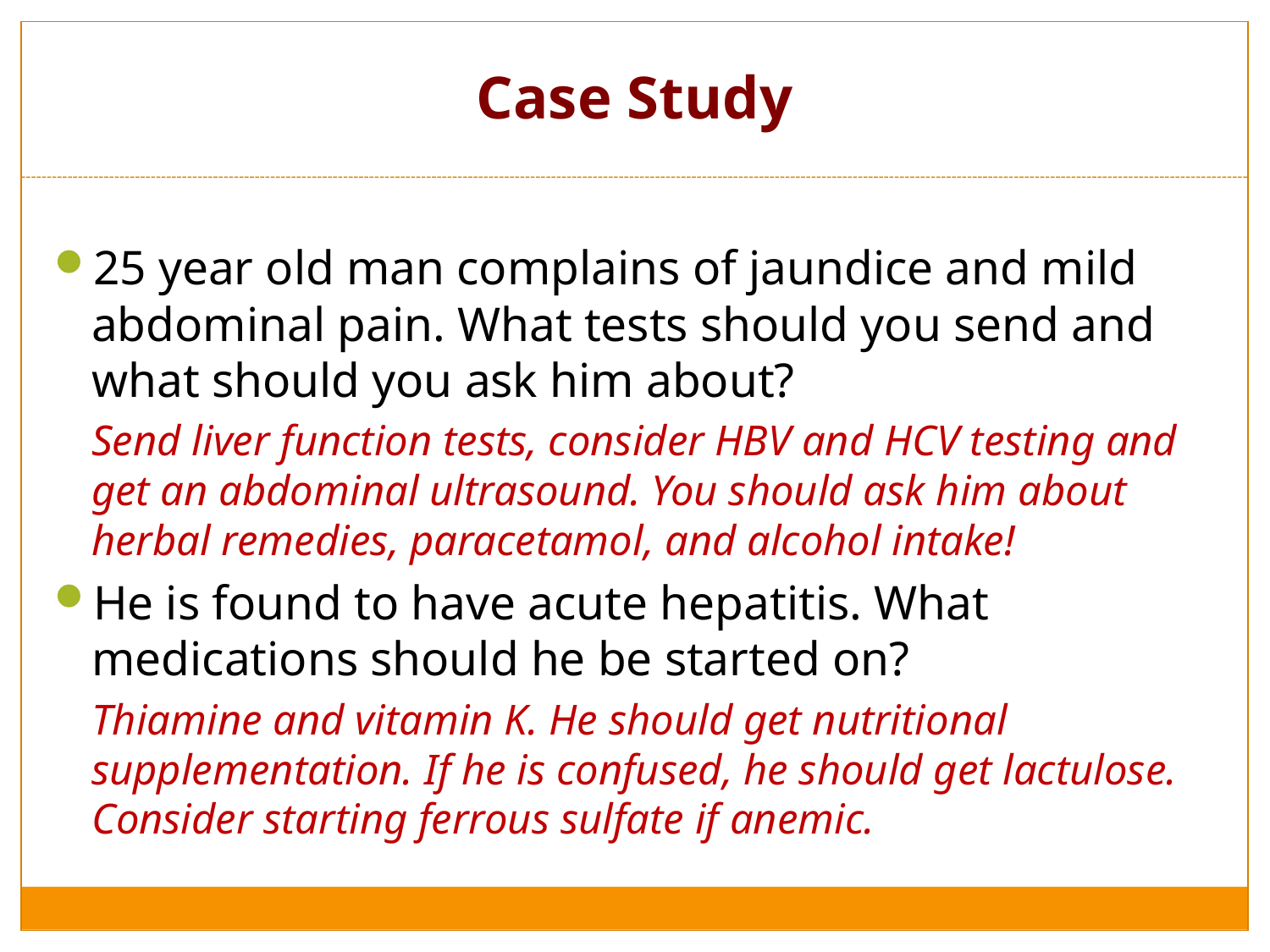

# Case Study
25 year old man complains of jaundice and mild abdominal pain. What tests should you send and what should you ask him about?
Send liver function tests, consider HBV and HCV testing and get an abdominal ultrasound. You should ask him about herbal remedies, paracetamol, and alcohol intake!
He is found to have acute hepatitis. What medications should he be started on?
Thiamine and vitamin K. He should get nutritional supplementation. If he is confused, he should get lactulose. Consider starting ferrous sulfate if anemic.

## Slide 23
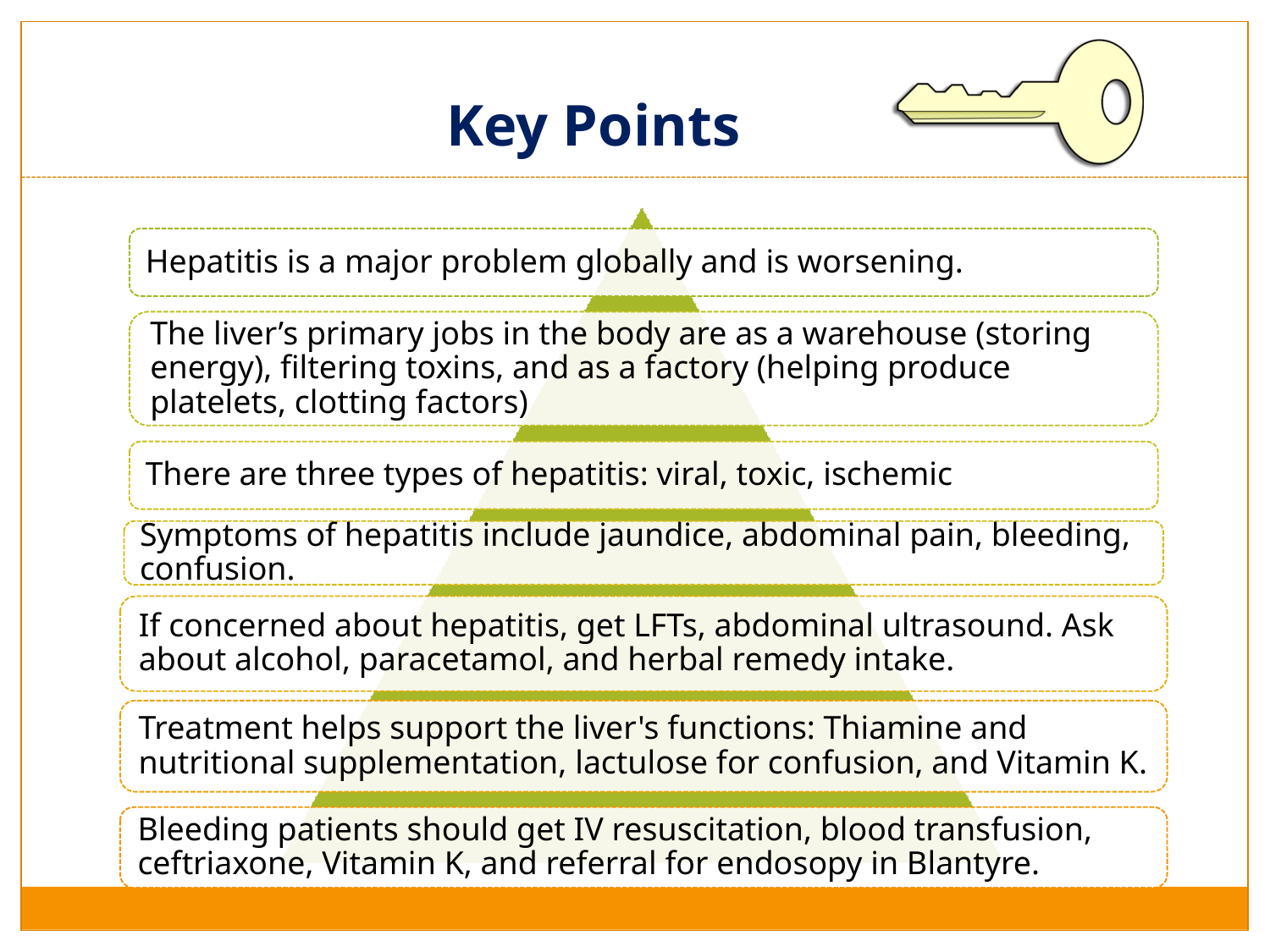

# Key Points

## Slide 24
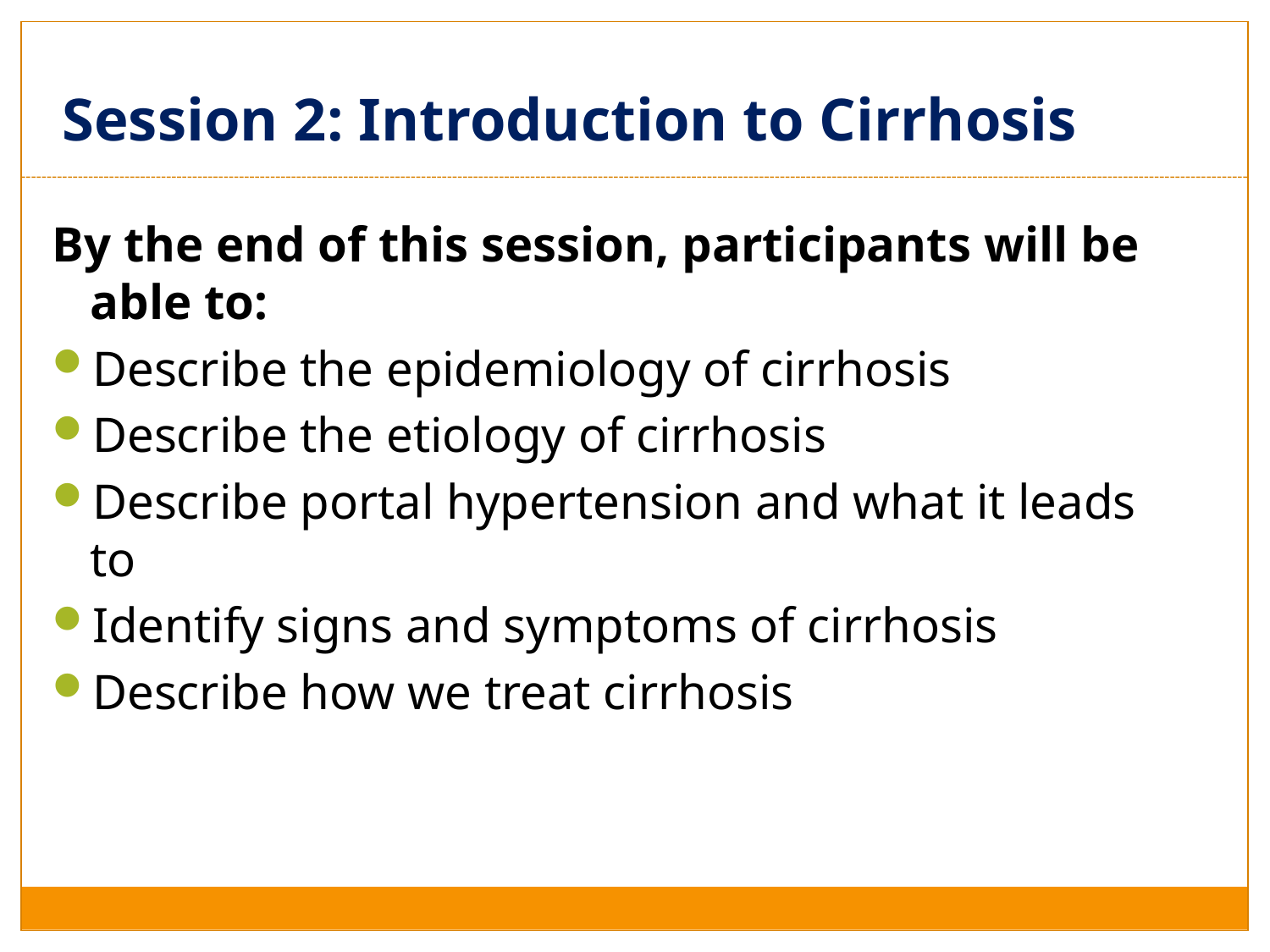

# Session 2: Introduction to Cirrhosis
By the end of this session, participants will be able to:
Describe the epidemiology of cirrhosis
Describe the etiology of cirrhosis
Describe portal hypertension and what it leads to
Identify signs and symptoms of cirrhosis
Describe how we treat cirrhosis

## Slide 25
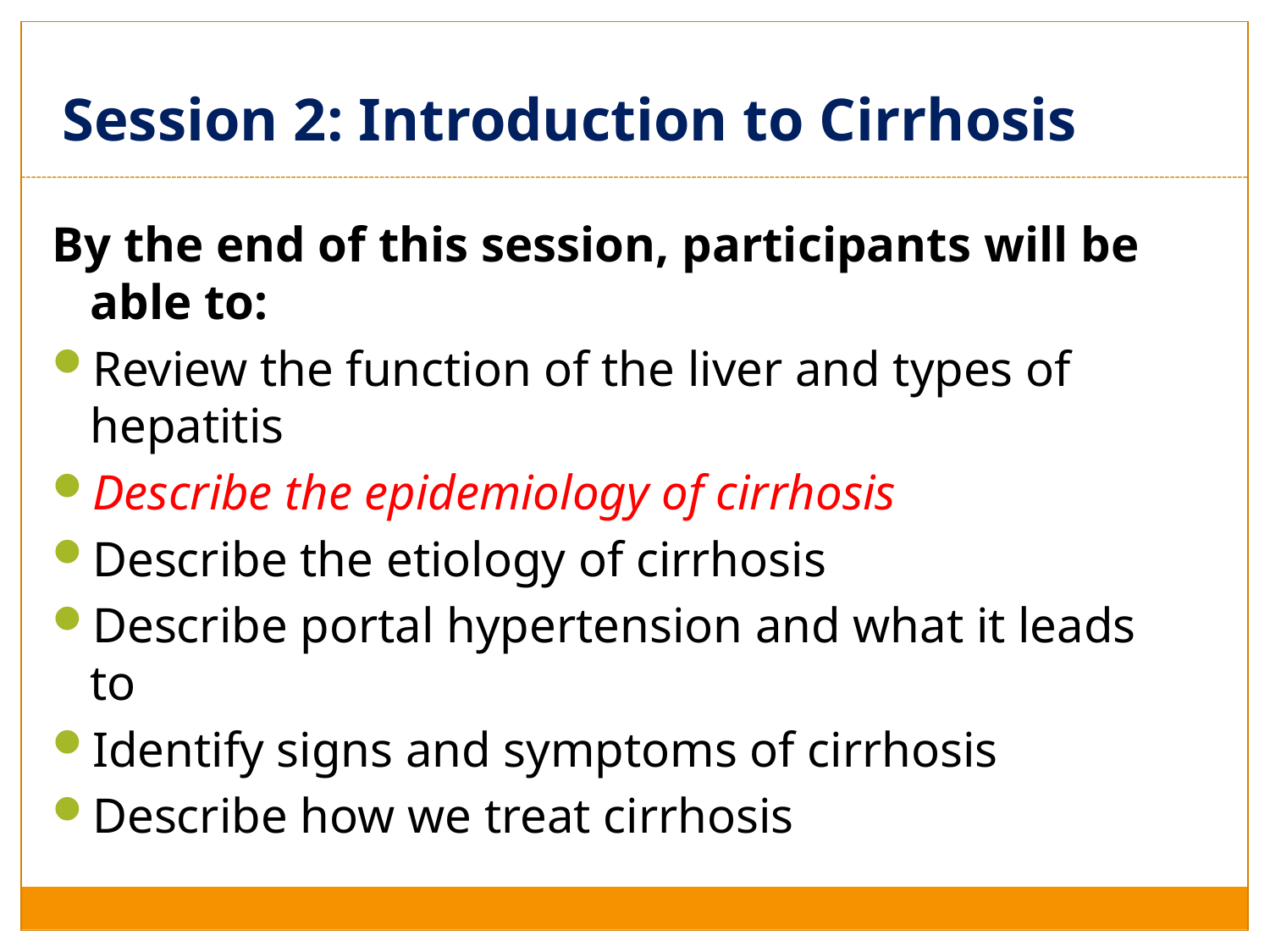

# Session 2: Introduction to Cirrhosis
By the end of this session, participants will be able to:
Review the function of the liver and types of hepatitis
Describe the epidemiology of cirrhosis
Describe the etiology of cirrhosis
Describe portal hypertension and what it leads to
Identify signs and symptoms of cirrhosis
Describe how we treat cirrhosis

## Slide 26
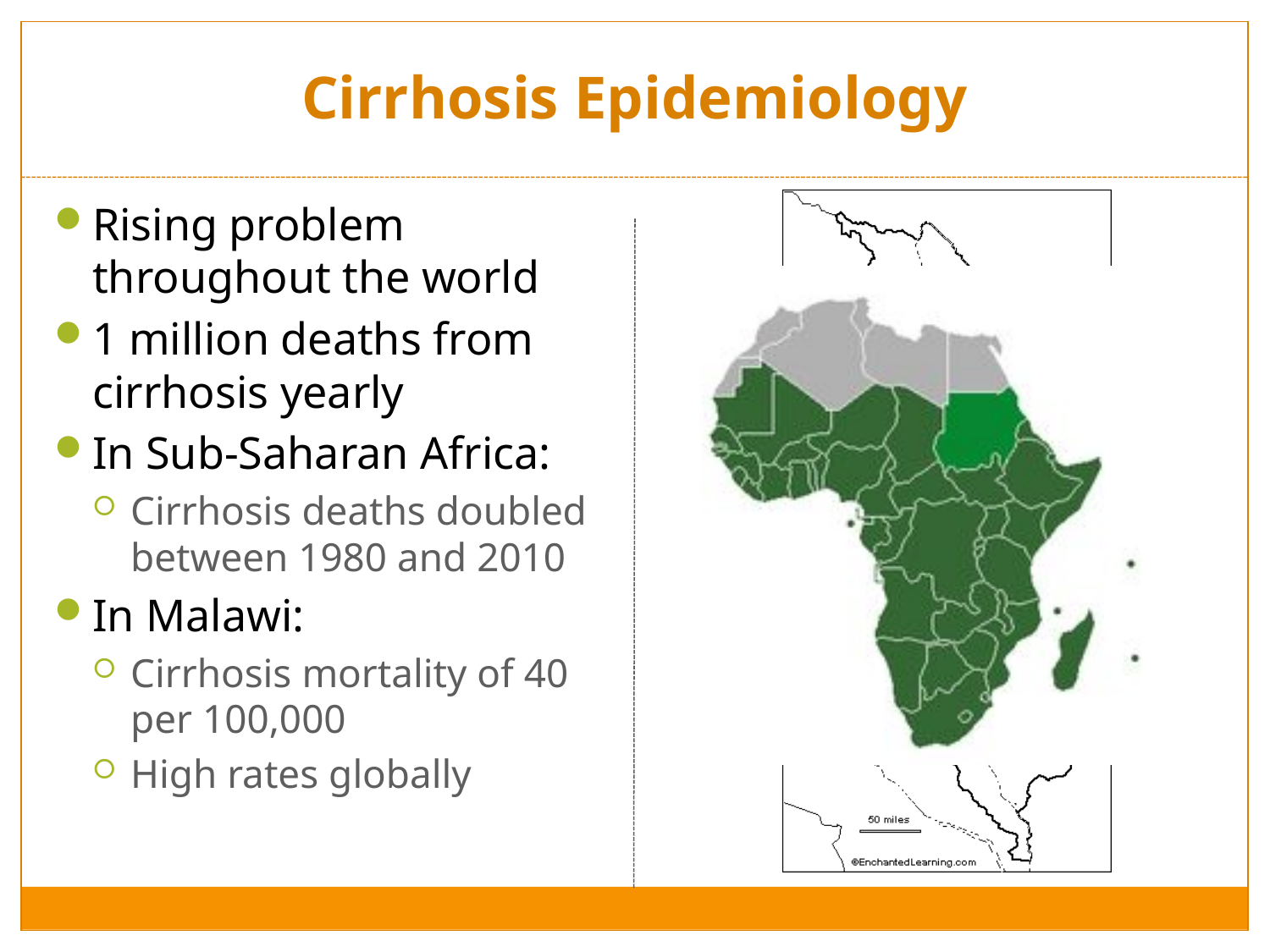

# Cirrhosis Epidemiology
Rising problem throughout the world
1 million deaths from cirrhosis yearly
In Sub-Saharan Africa:
Cirrhosis deaths doubled between 1980 and 2010
In Malawi:
Cirrhosis mortality of 40 per 100,000
High rates globally

## Slide 27
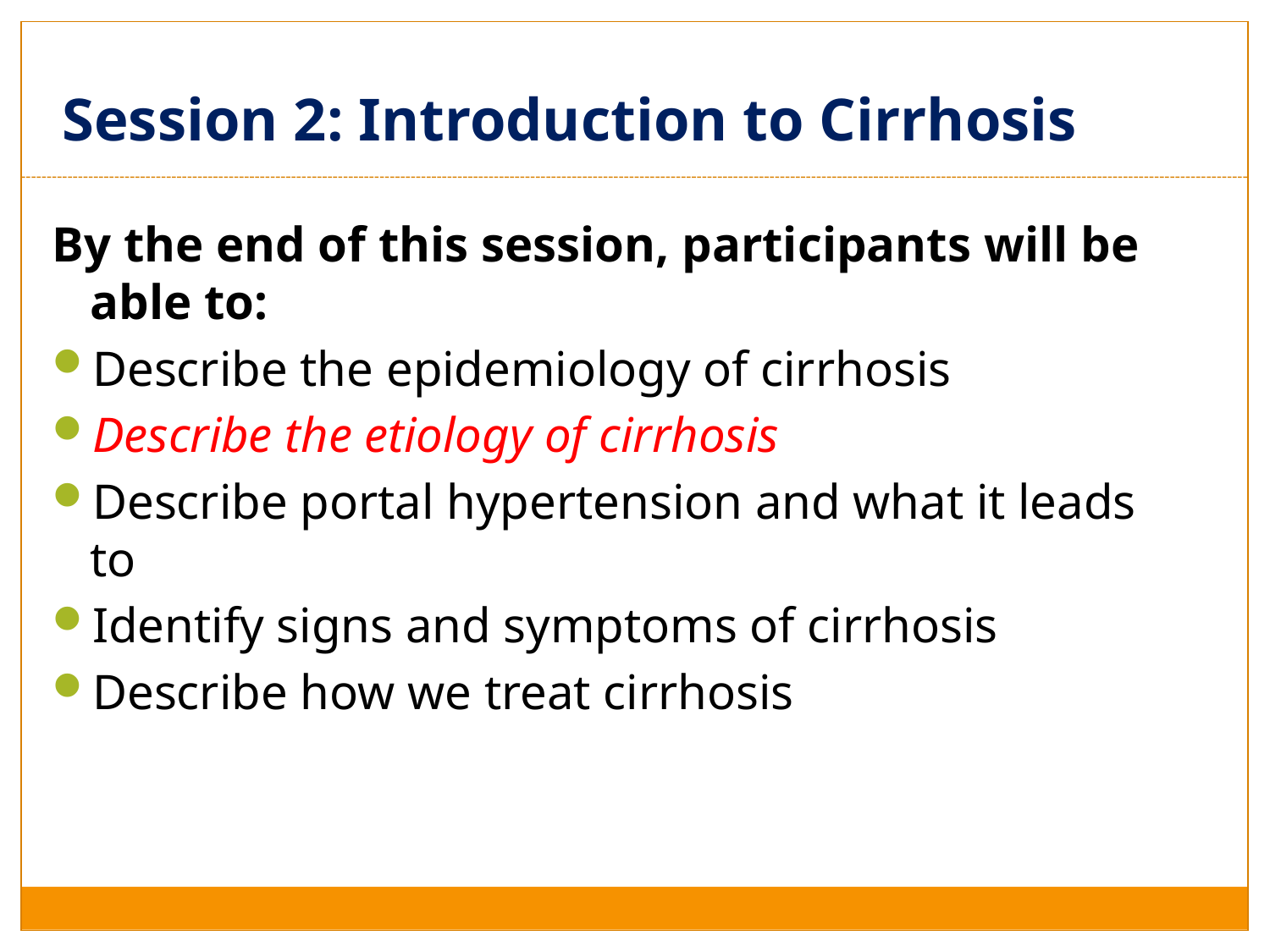

# Session 2: Introduction to Cirrhosis
By the end of this session, participants will be able to:
Describe the epidemiology of cirrhosis
Describe the etiology of cirrhosis
Describe portal hypertension and what it leads to
Identify signs and symptoms of cirrhosis
Describe how we treat cirrhosis

## Slide 28
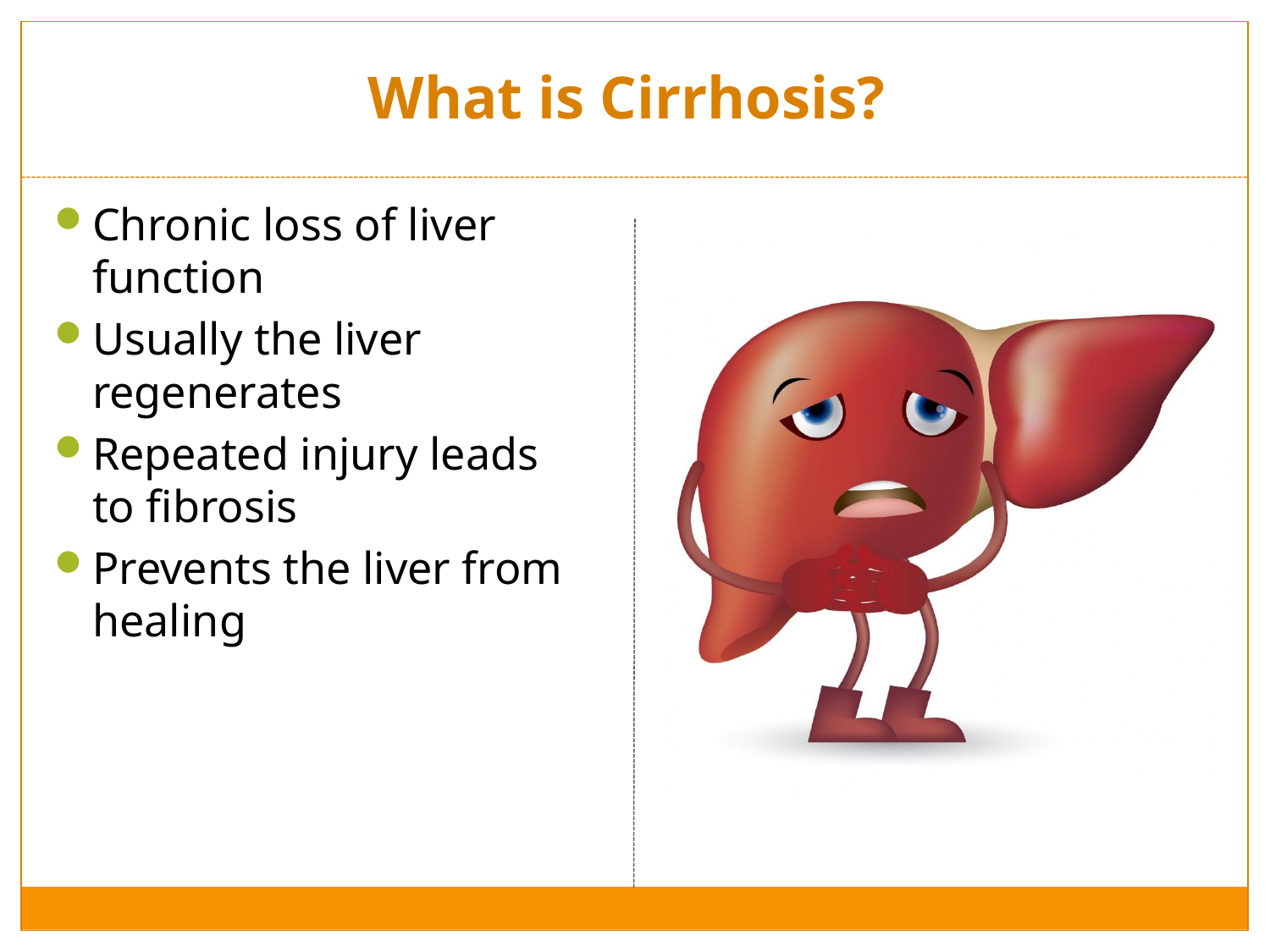

# What is Cirrhosis?
Chronic loss of liver function
Usually the liver regenerates
Repeated injury leads to fibrosis
Prevents the liver from healing

## Slide 29
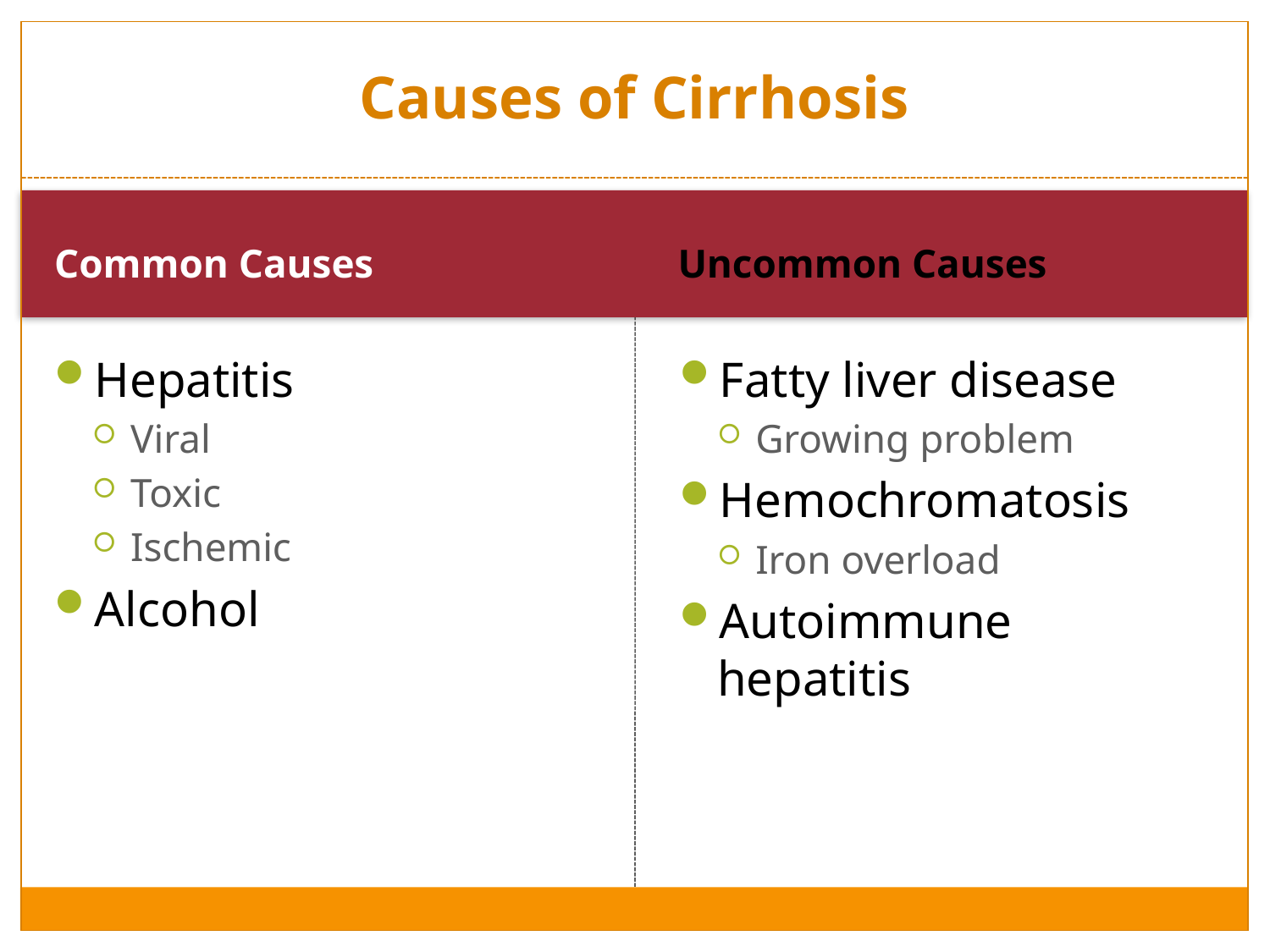

# Causes of Cirrhosis
Common Causes
Uncommon Causes
Hepatitis
Viral
Toxic
Ischemic
Alcohol
Fatty liver disease
Growing problem
Hemochromatosis
Iron overload
Autoimmune hepatitis

## Slide 30
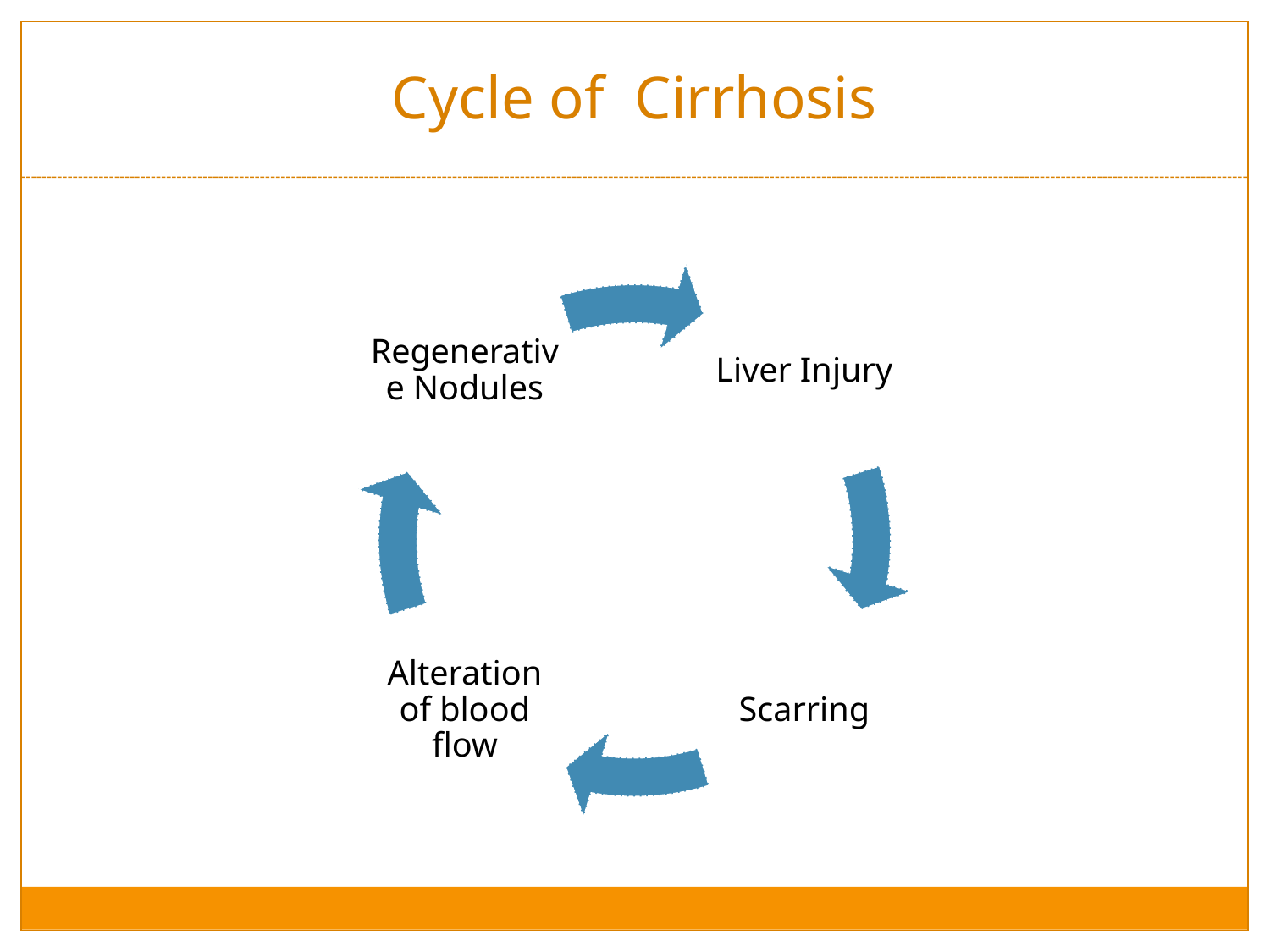

# Cycle of Cirrhosis

## Slide 31
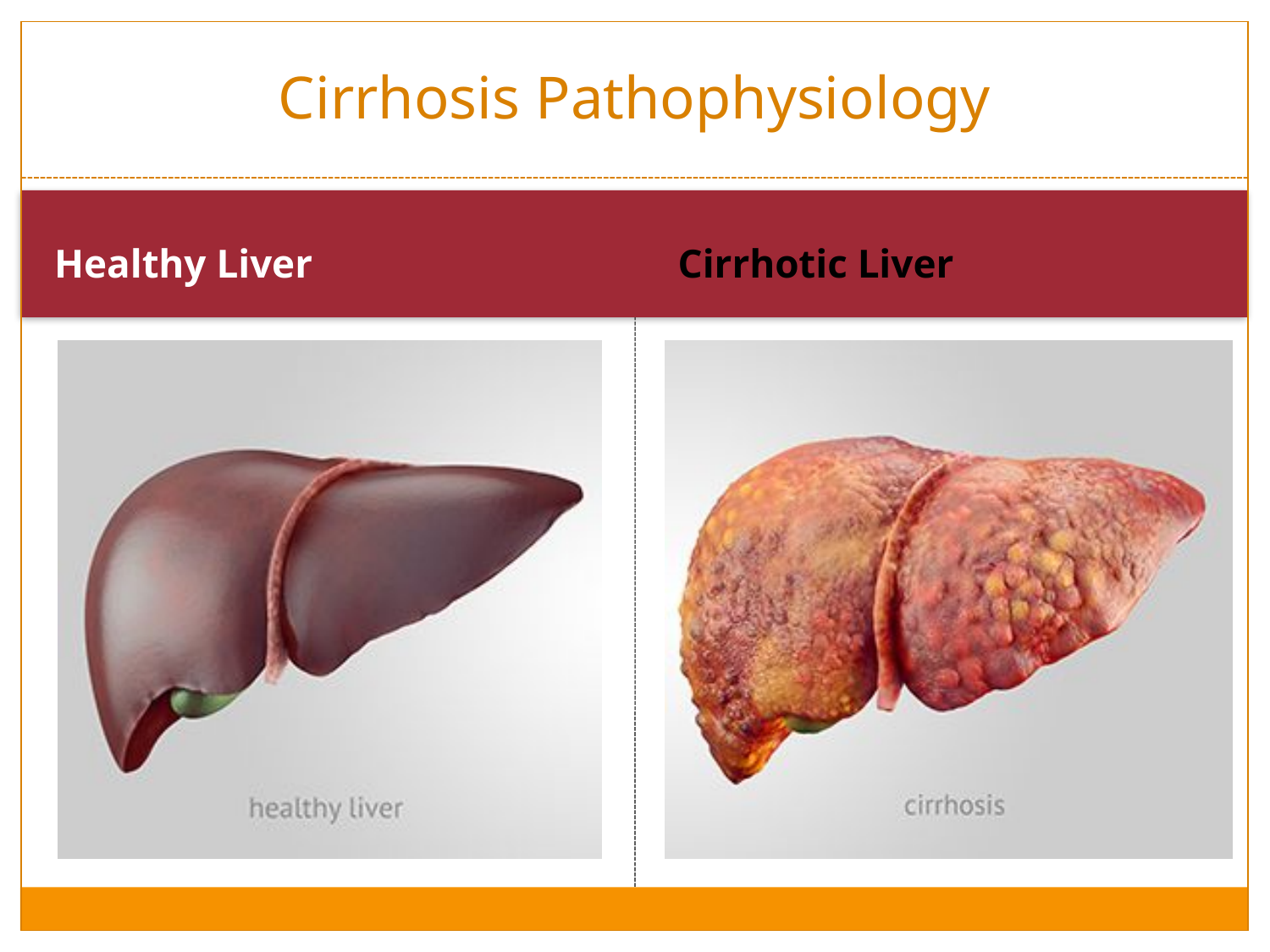

# Cirrhosis Pathophysiology
Healthy Liver
Cirrhotic Liver

## Slide 32
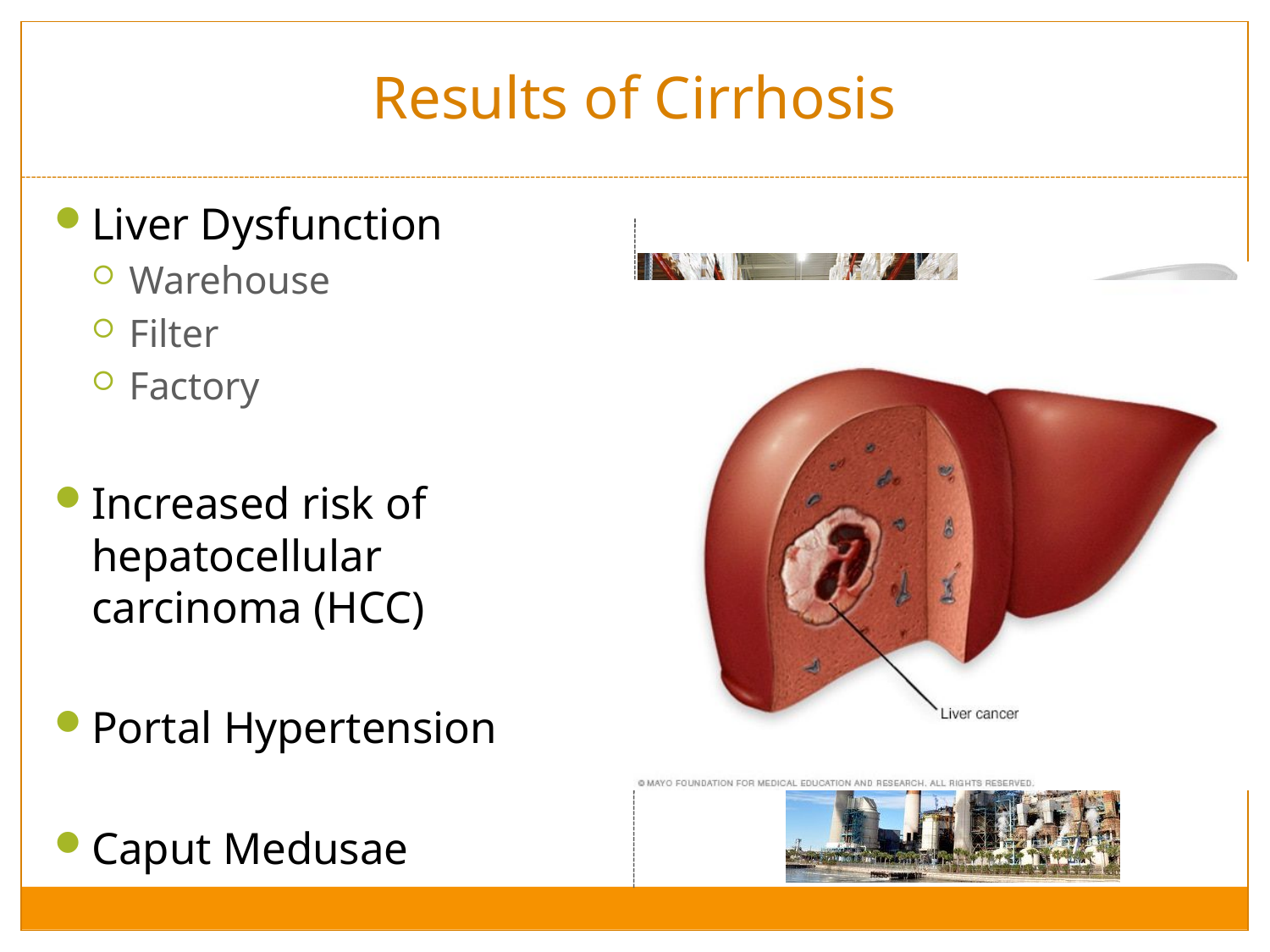

# Results of Cirrhosis
Liver Dysfunction
Warehouse
Filter
Factory
Increased risk of hepatocellular carcinoma (HCC)
Portal Hypertension
Caput Medusae

## Slide 33
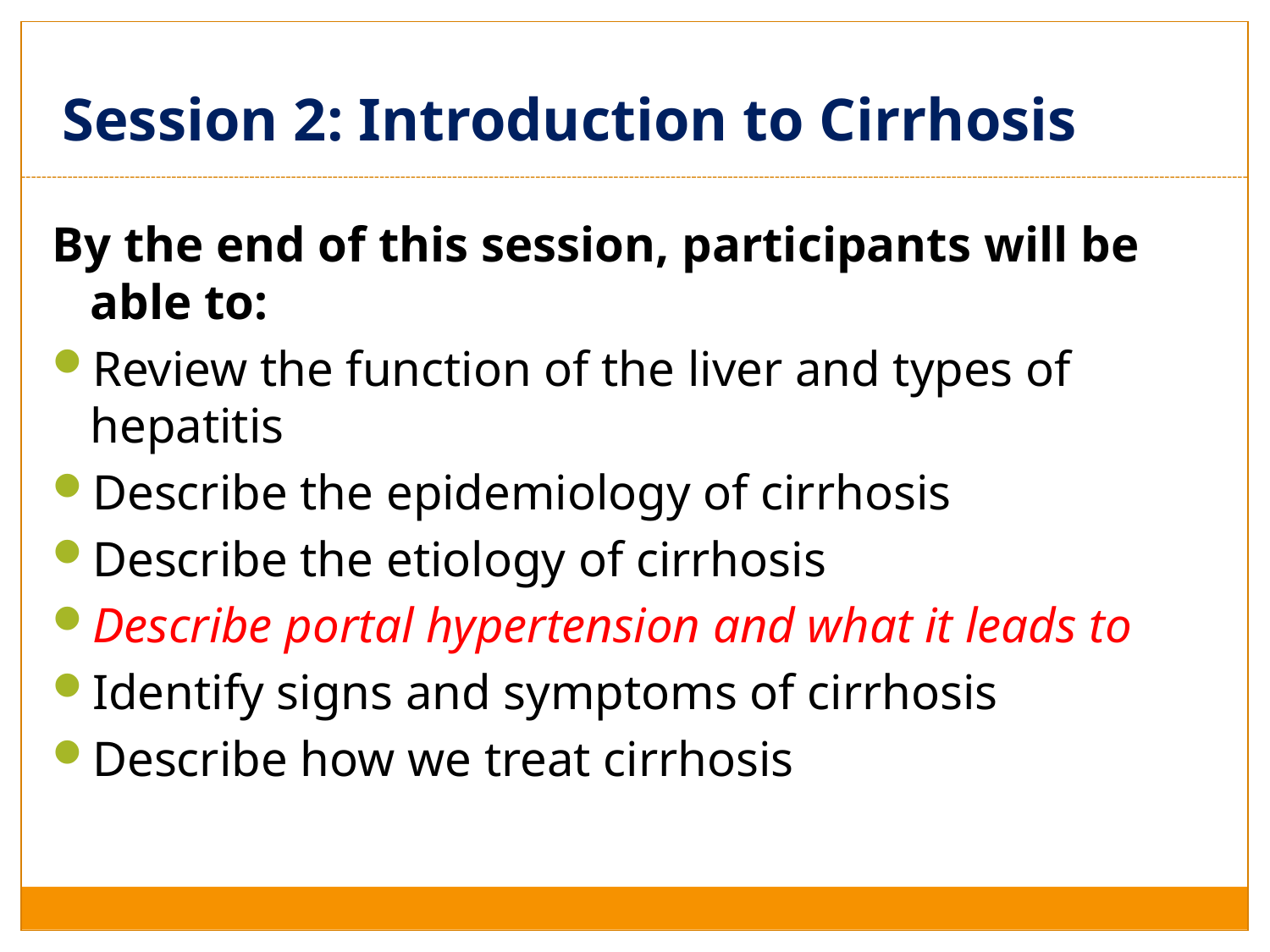

# Session 2: Introduction to Cirrhosis
By the end of this session, participants will be able to:
Review the function of the liver and types of hepatitis
Describe the epidemiology of cirrhosis
Describe the etiology of cirrhosis
Describe portal hypertension and what it leads to
Identify signs and symptoms of cirrhosis
Describe how we treat cirrhosis

## Slide 34
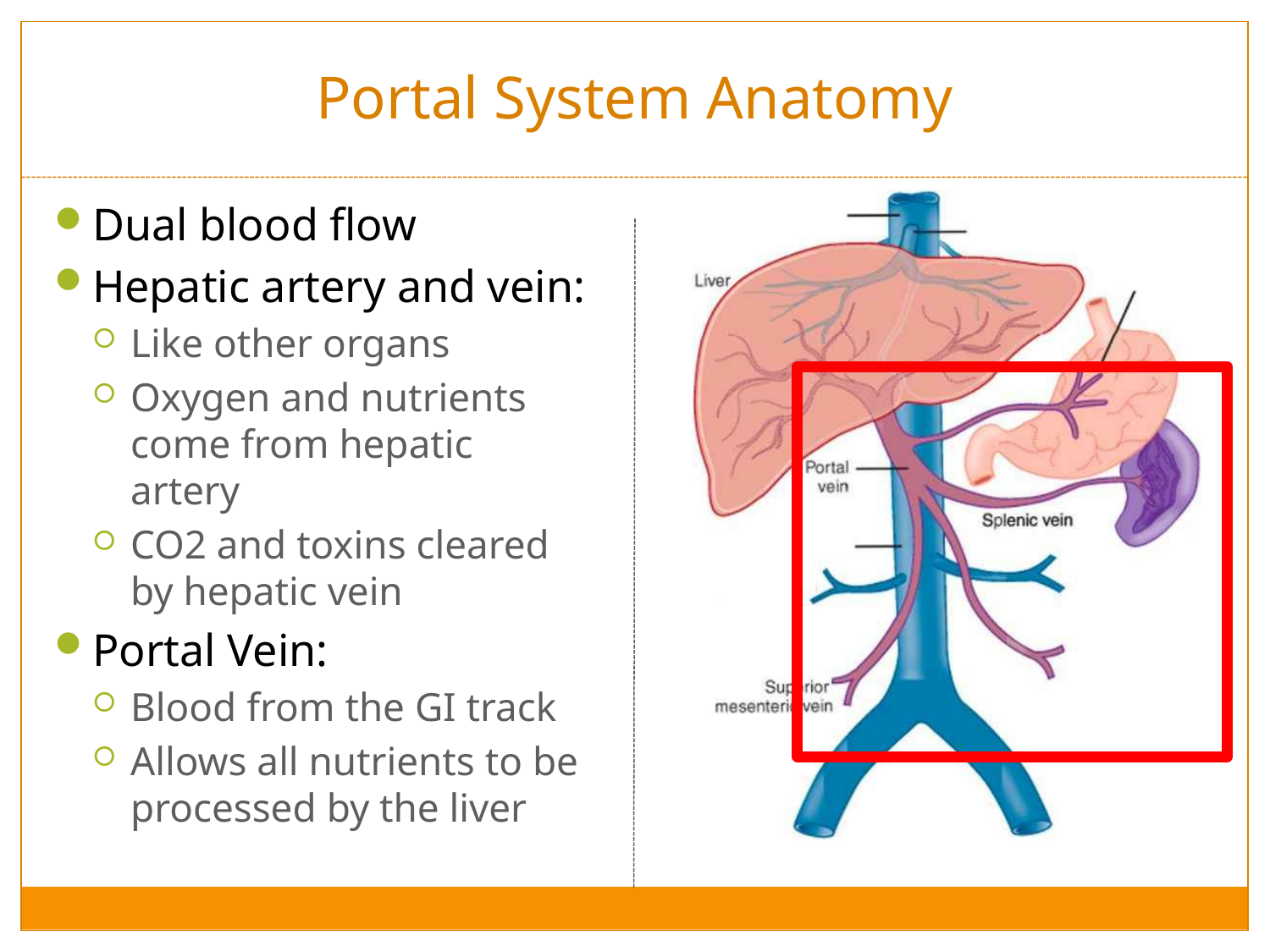

# Portal System Anatomy
Dual blood flow
Hepatic artery and vein:
Like other organs
Oxygen and nutrients come from hepatic artery
CO2 and toxins cleared by hepatic vein
Portal Vein:
Blood from the GI track
Allows all nutrients to be processed by the liver

## Slide 35
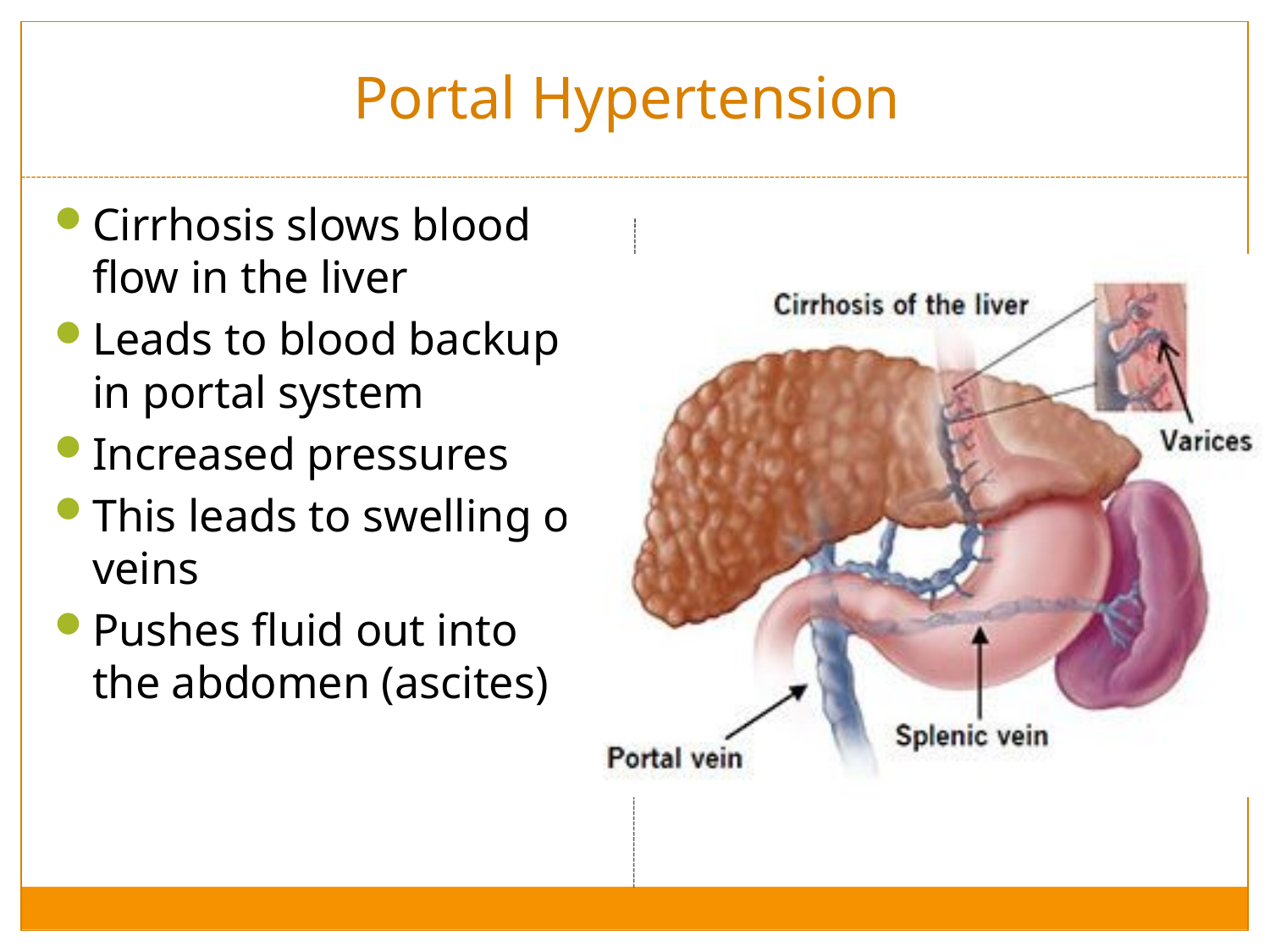

# Portal Hypertension
Cirrhosis slows blood flow in the liver
Leads to blood backup in portal system
Increased pressures
This leads to swelling of veins
Pushes fluid out into the abdomen (ascites)

## Slide 36
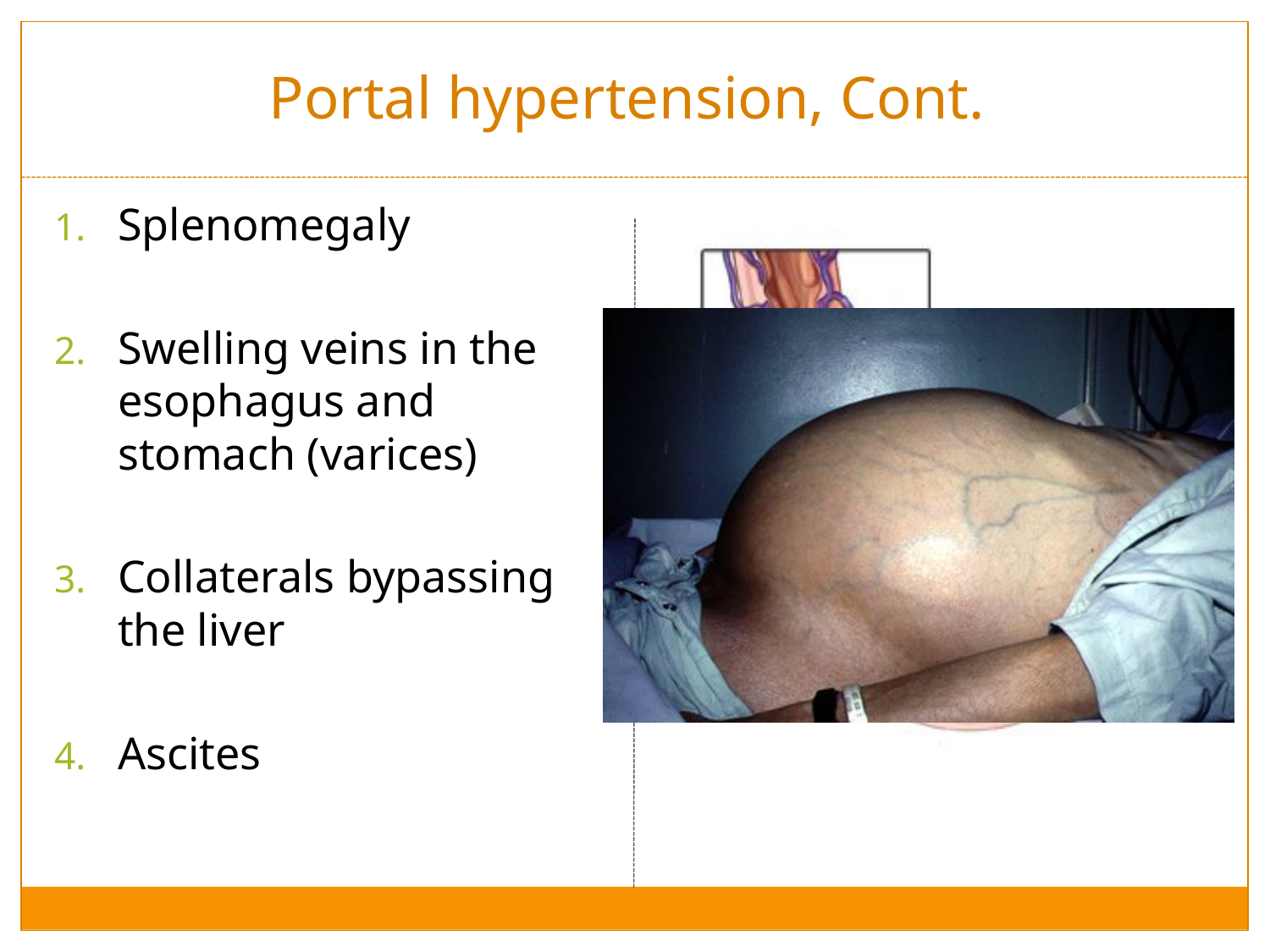

# Portal hypertension, Cont.
Splenomegaly
Swelling veins in the esophagus and stomach (varices)
Collaterals bypassing the liver
Ascites

## Slide 37
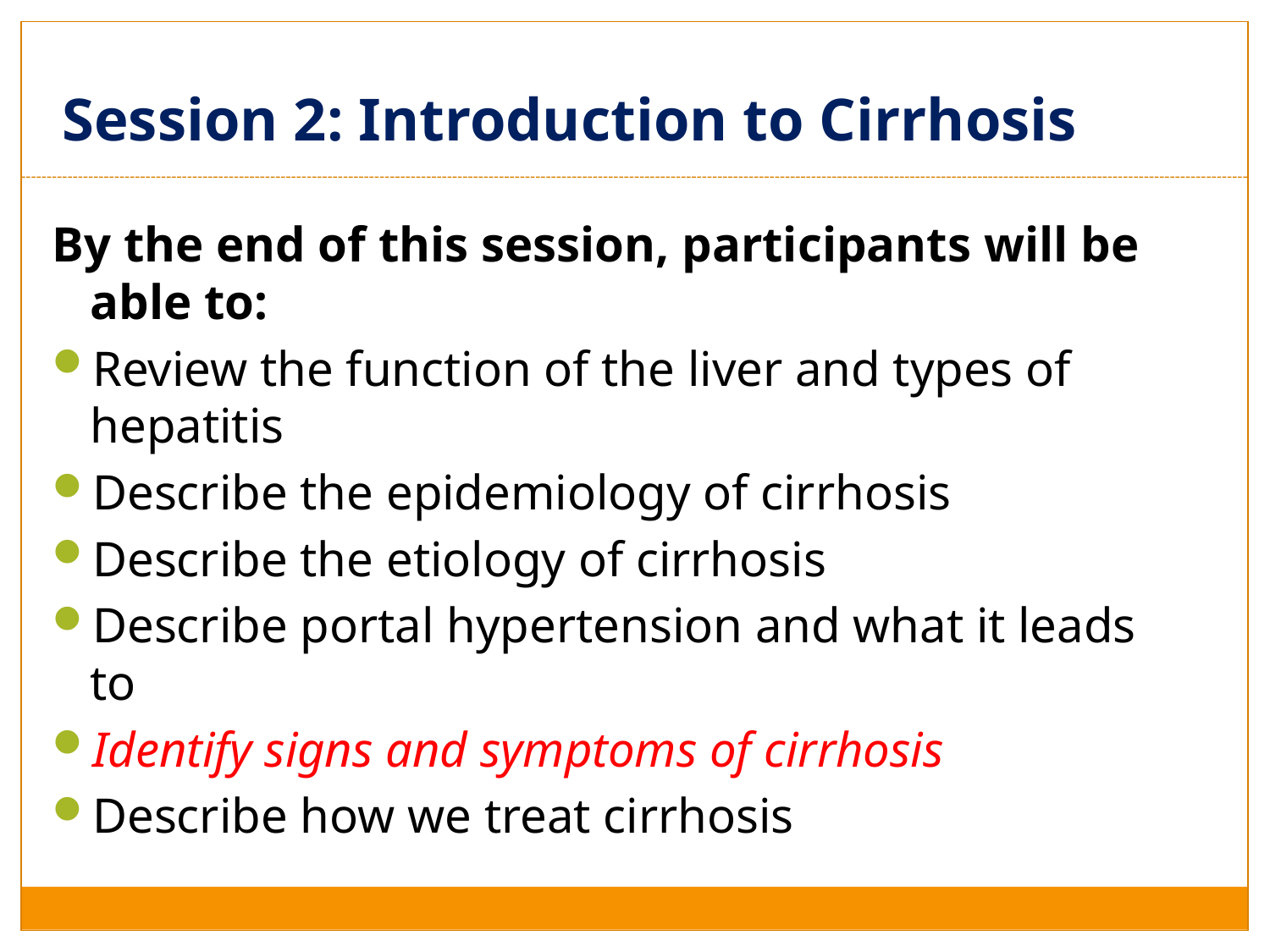

# Session 2: Introduction to Cirrhosis
By the end of this session, participants will be able to:
Review the function of the liver and types of hepatitis
Describe the epidemiology of cirrhosis
Describe the etiology of cirrhosis
Describe portal hypertension and what it leads to
Identify signs and symptoms of cirrhosis
Describe how we treat cirrhosis

## Slide 38
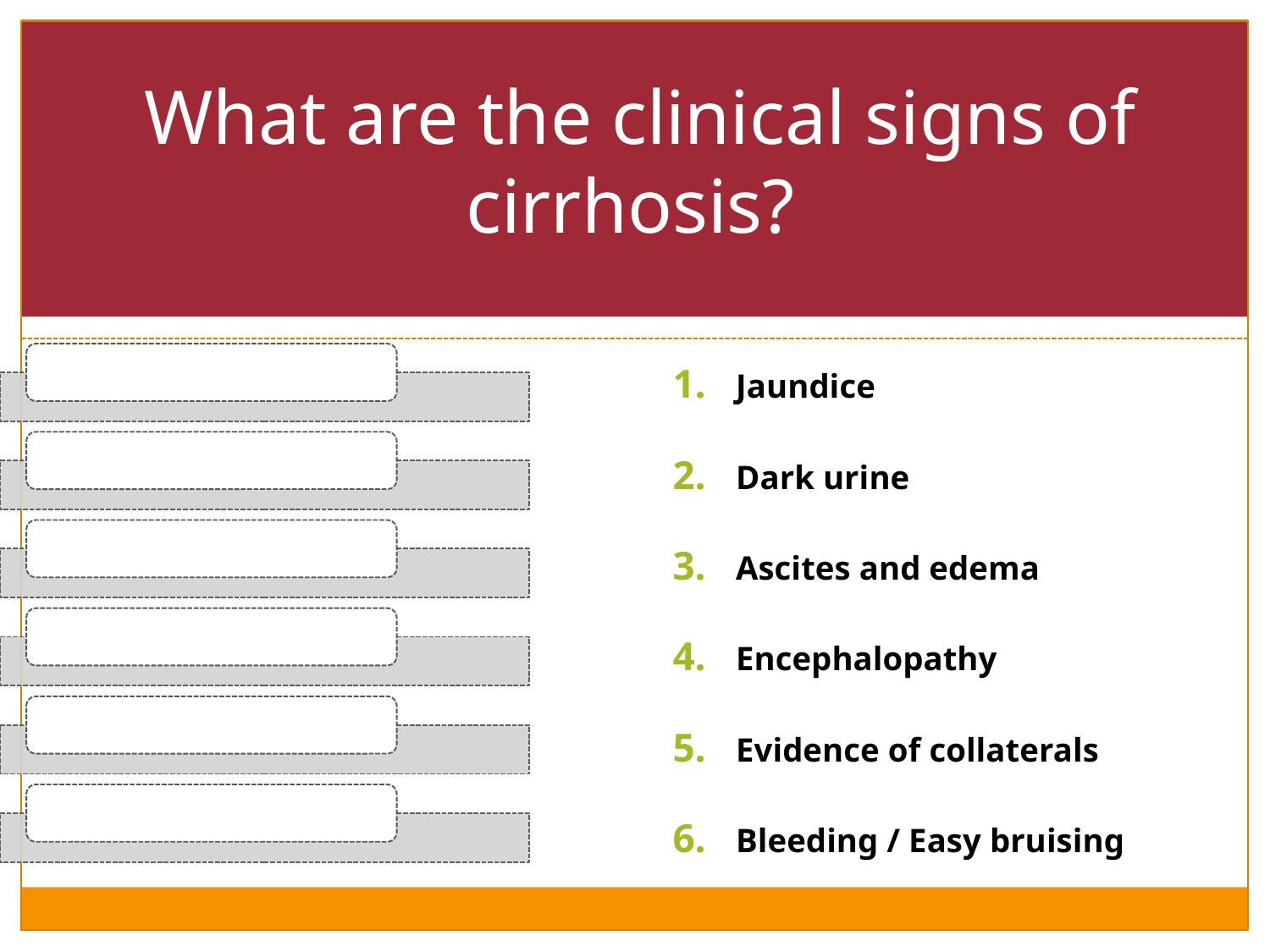

# What are the clinical signs of cirrhosis?
Jaundice
Dark urine
Ascites and edema
Encephalopathy
Evidence of collaterals
Bleeding / Easy bruising

## Slide 39
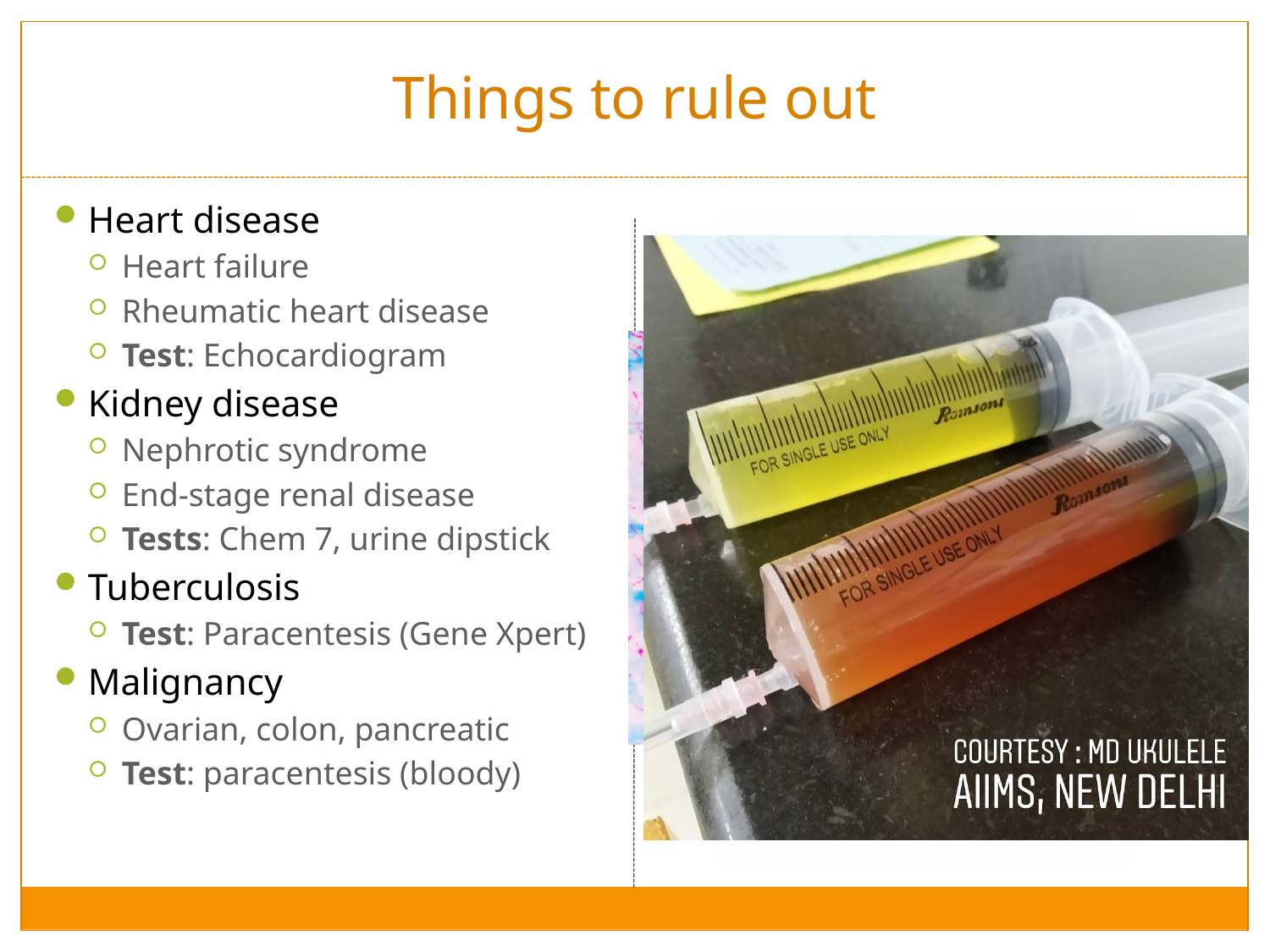

# Things to rule out
Heart disease
Heart failure
Rheumatic heart disease
Test: Echocardiogram
Kidney disease
Nephrotic syndrome
End-stage renal disease
Tests: Chem 7, urine dipstick
Tuberculosis
Test: Paracentesis (Gene Xpert)
Malignancy
Ovarian, colon, pancreatic
Test: paracentesis (bloody)

## Slide 40
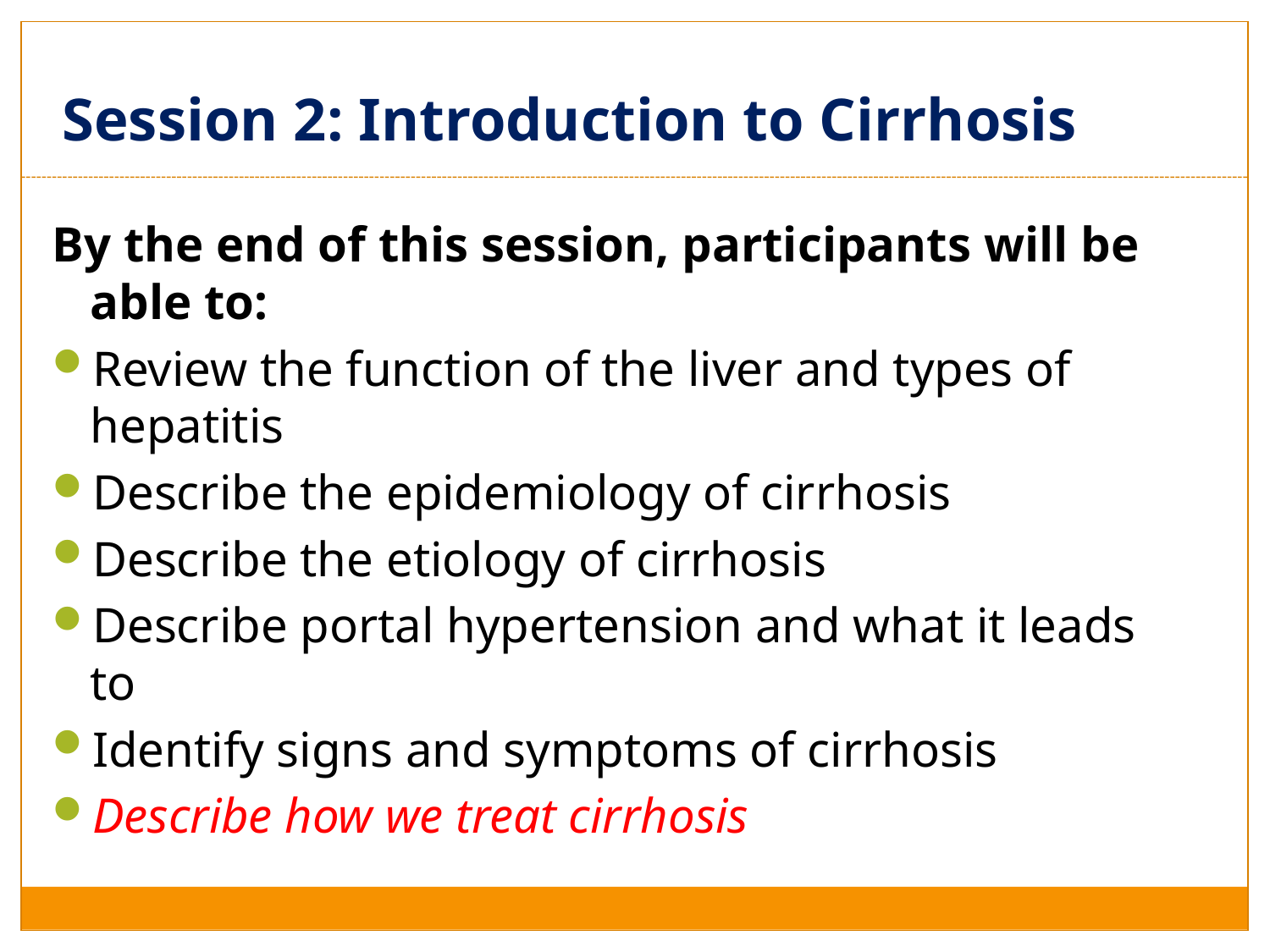

# Session 2: Introduction to Cirrhosis
By the end of this session, participants will be able to:
Review the function of the liver and types of hepatitis
Describe the epidemiology of cirrhosis
Describe the etiology of cirrhosis
Describe portal hypertension and what it leads to
Identify signs and symptoms of cirrhosis
Describe how we treat cirrhosis

## Slide 41
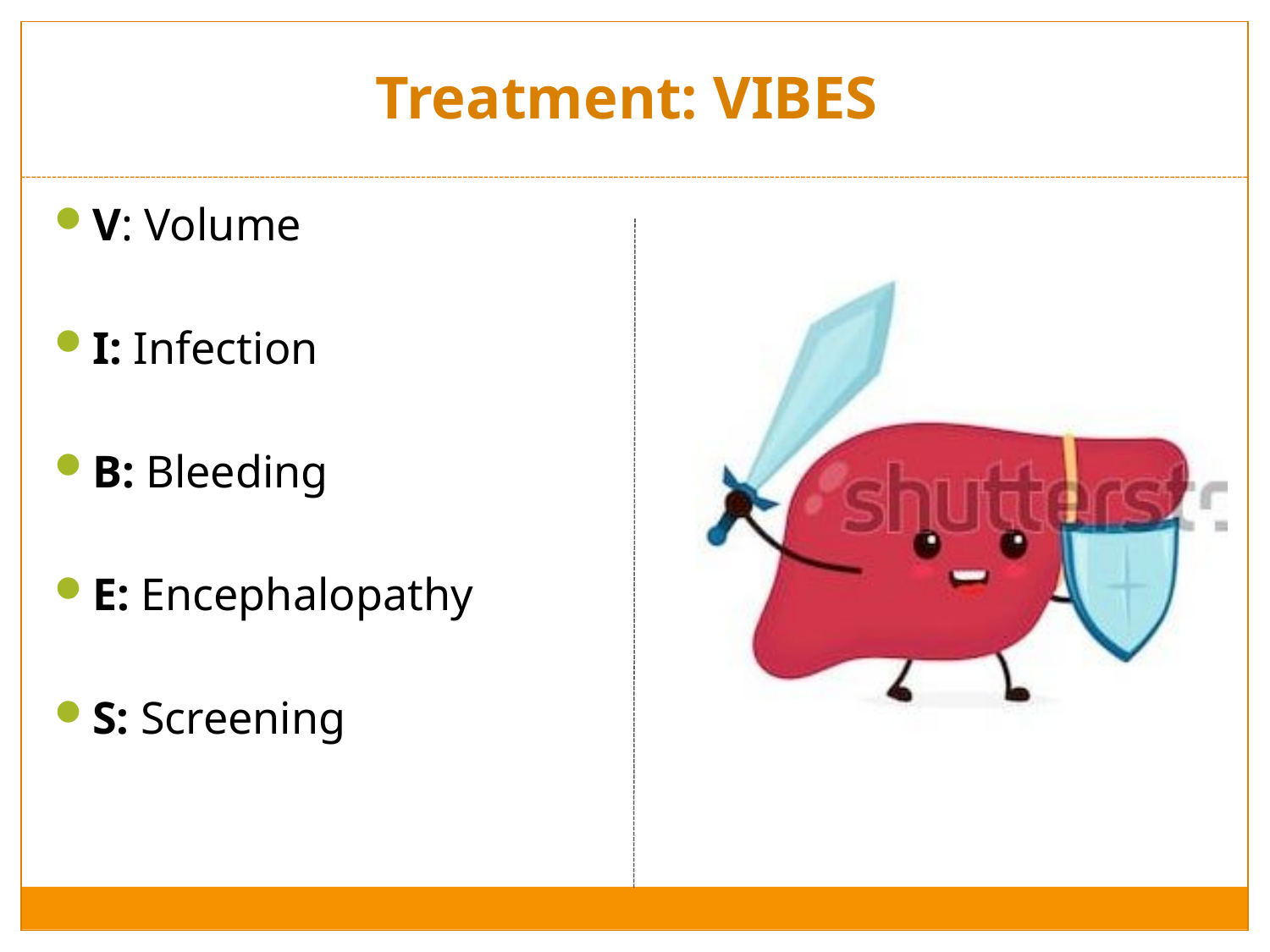

# Treatment: VIBES
V: Volume
I: Infection
B: Bleeding
E: Encephalopathy
S: Screening

## Slide 42
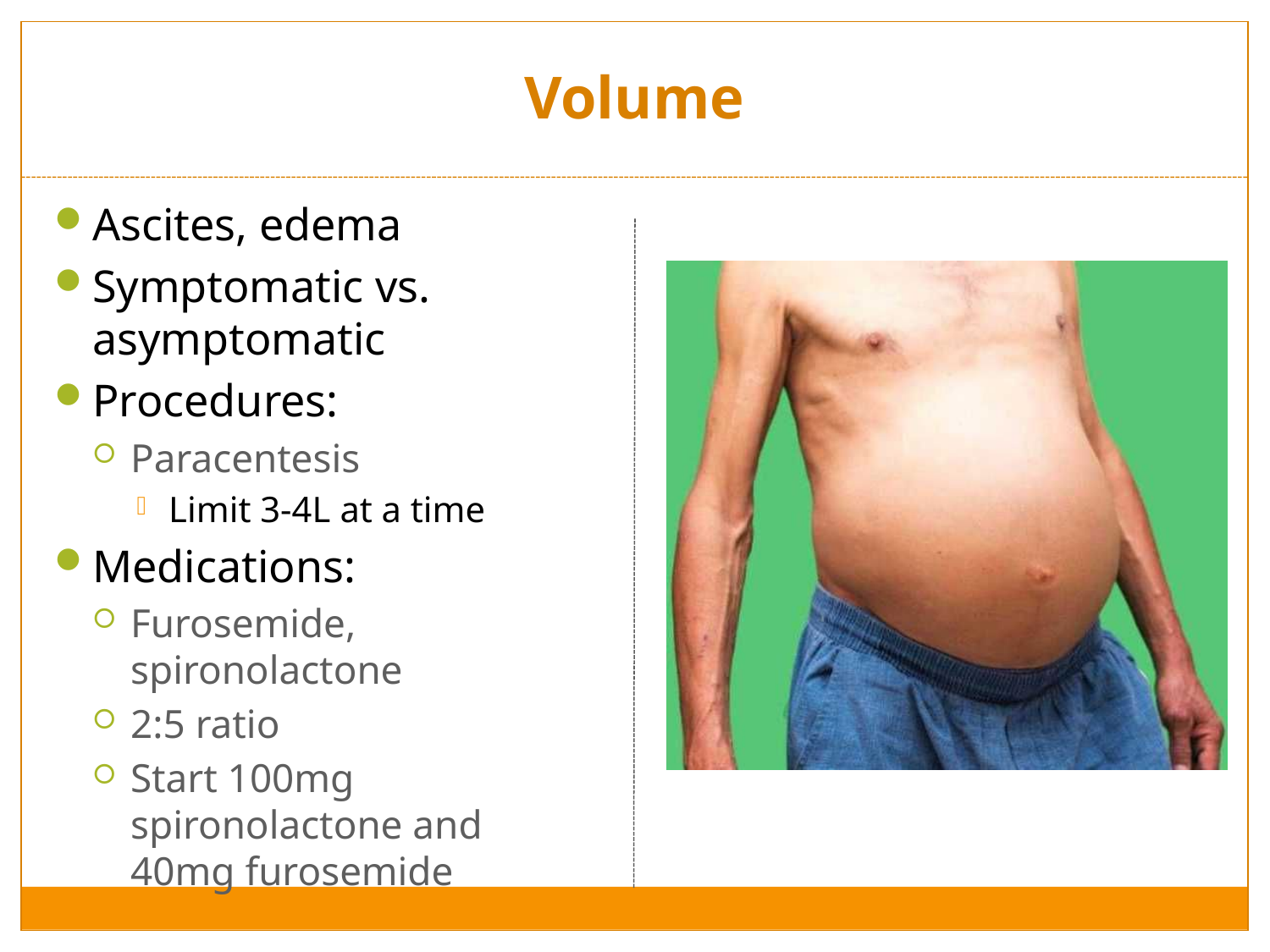

# Volume
Ascites, edema
Symptomatic vs. asymptomatic
Procedures:
Paracentesis
Limit 3-4L at a time
Medications:
Furosemide, spironolactone
2:5 ratio
Start 100mg spironolactone and 40mg furosemide

## Slide 43
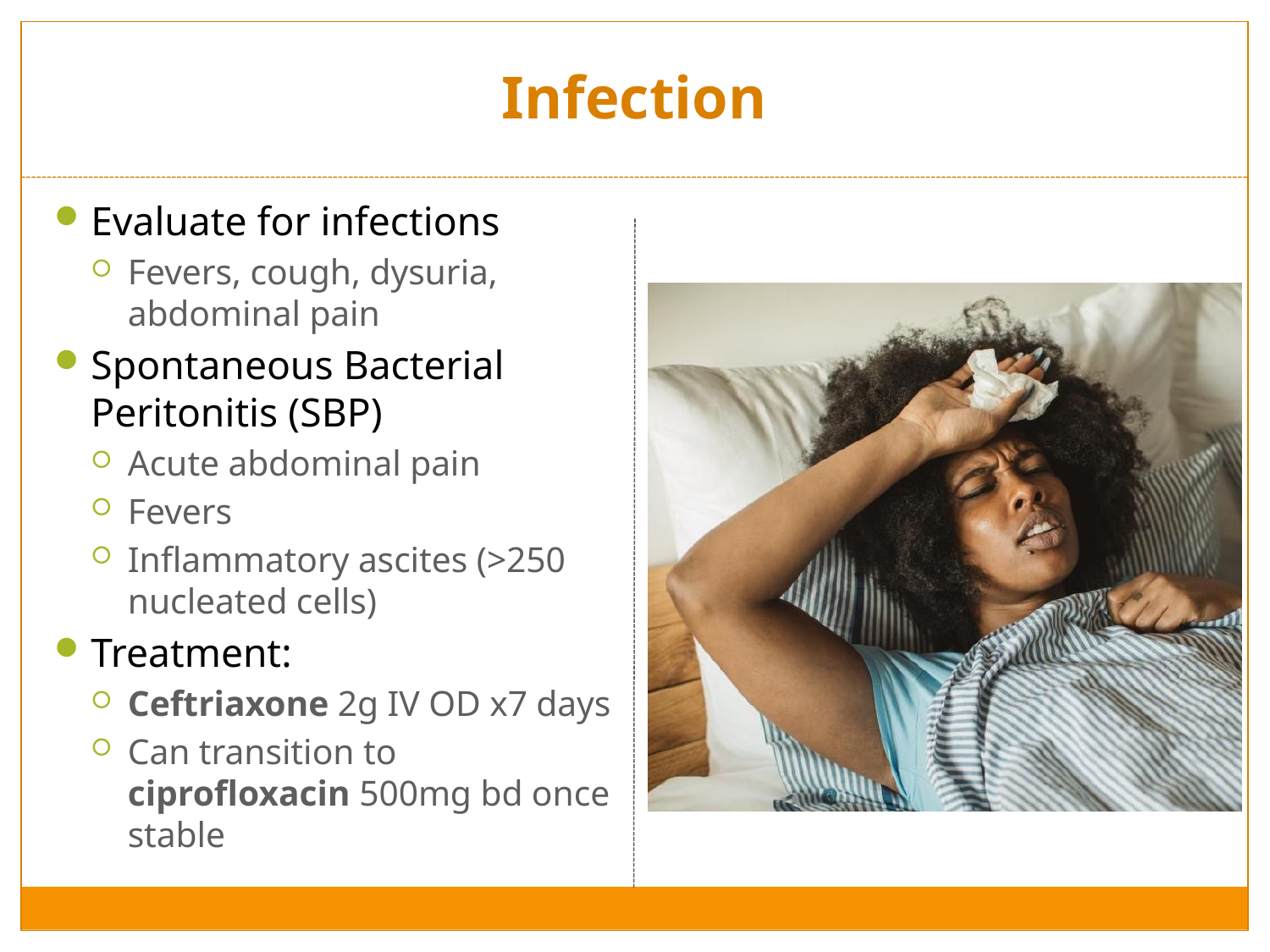

# Infection
Evaluate for infections
Fevers, cough, dysuria, abdominal pain
Spontaneous Bacterial Peritonitis (SBP)
Acute abdominal pain
Fevers
Inflammatory ascites (>250 nucleated cells)
Treatment:
Ceftriaxone 2g IV OD x7 days
Can transition to ciprofloxacin 500mg bd once stable

## Slide 44
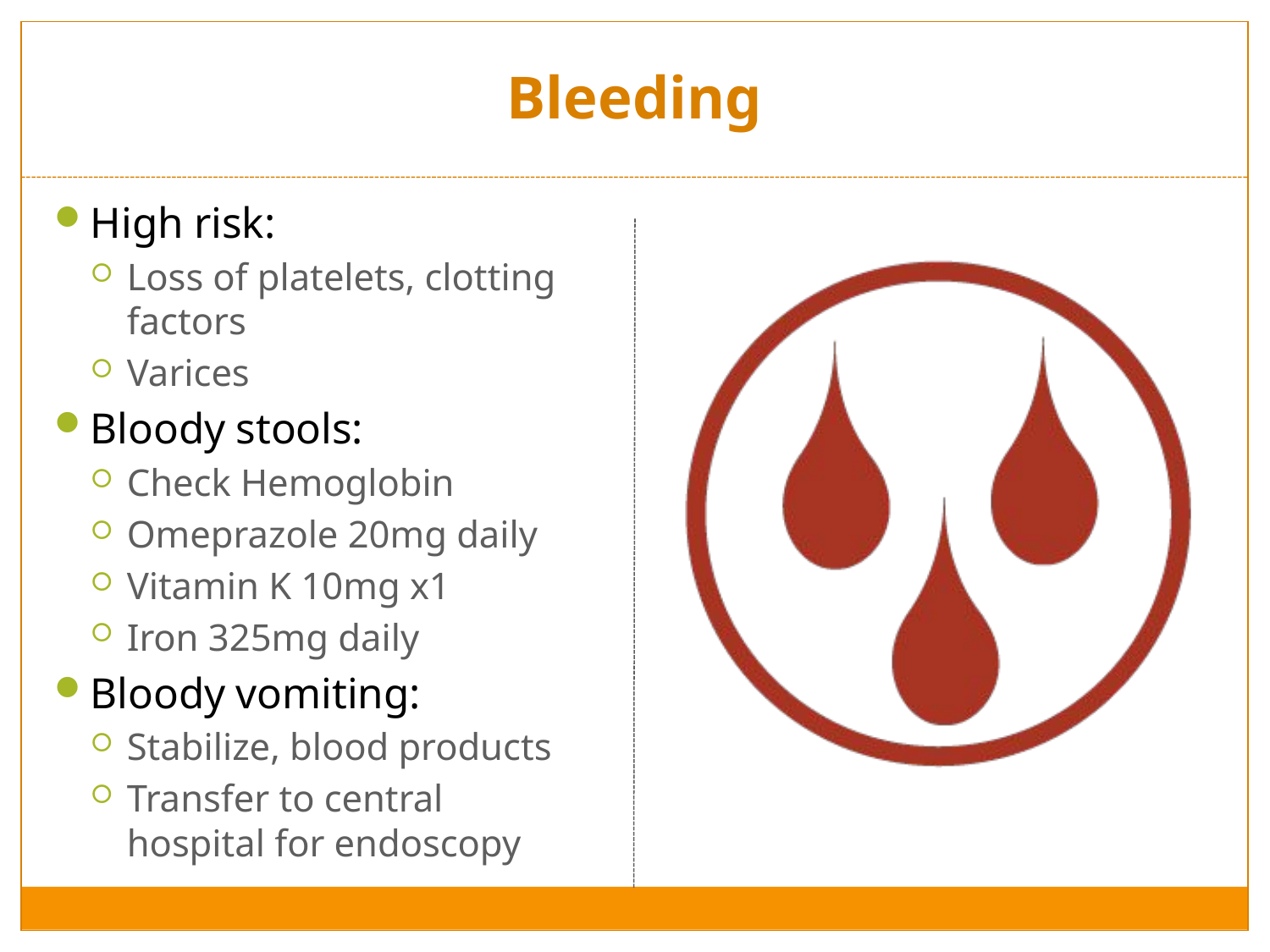

# Bleeding
High risk:
Loss of platelets, clotting factors
Varices
Bloody stools:
Check Hemoglobin
Omeprazole 20mg daily
Vitamin K 10mg x1
Iron 325mg daily
Bloody vomiting:
Stabilize, blood products
Transfer to central hospital for endoscopy

## Slide 45
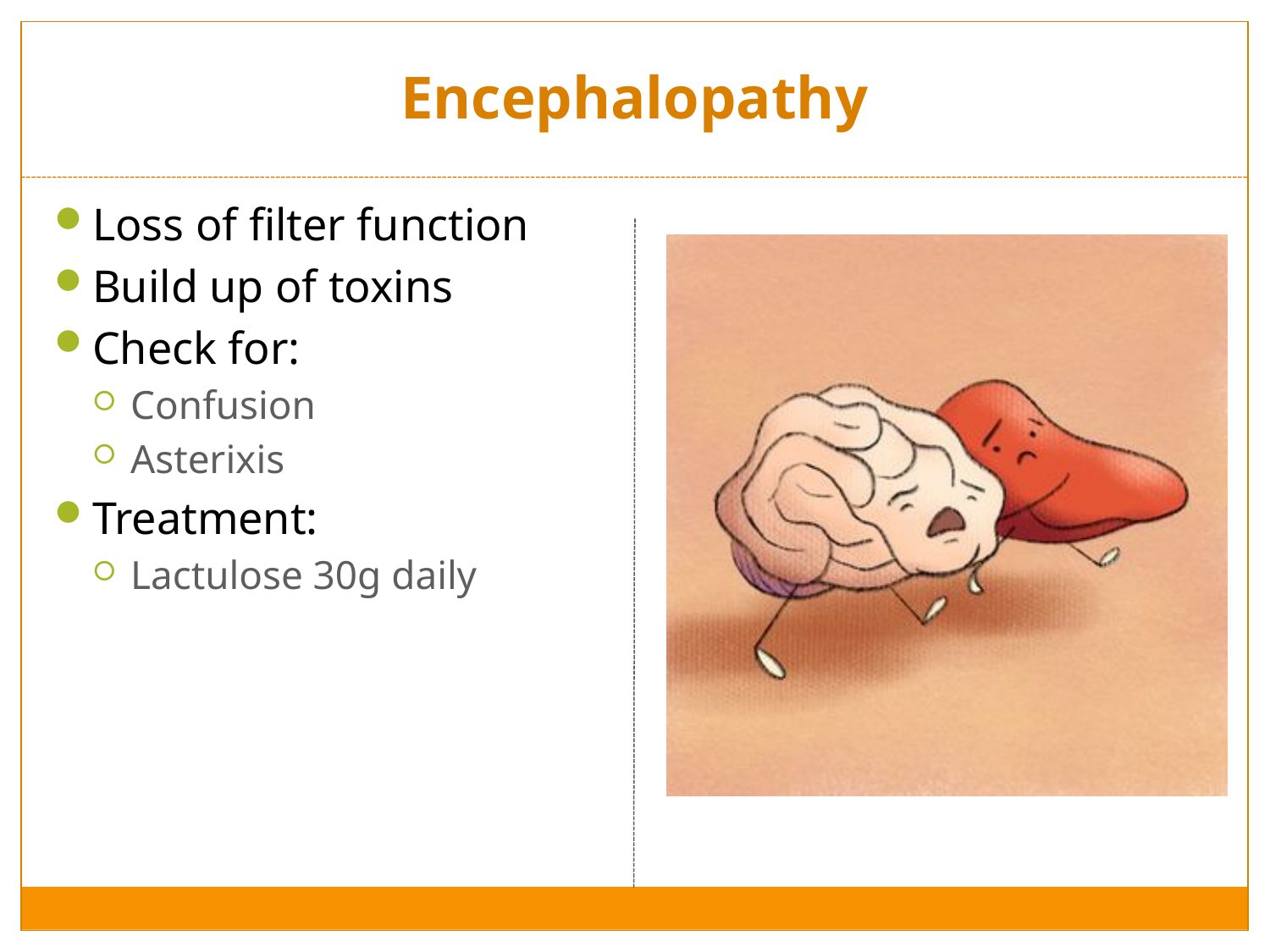

# Encephalopathy
Loss of filter function
Build up of toxins
Check for:
Confusion
Asterixis
Treatment:
Lactulose 30g daily

## Slide 46
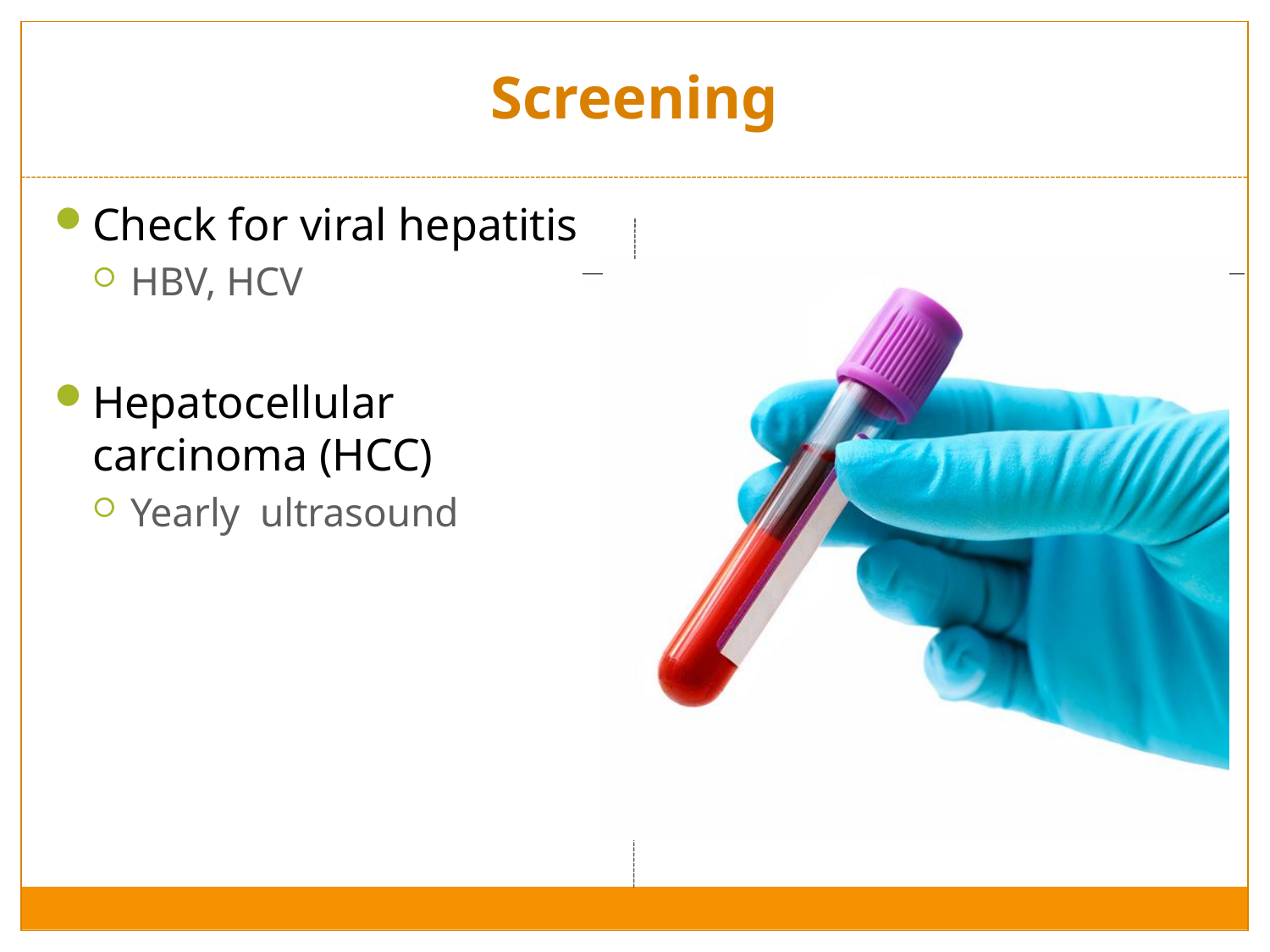

# Screening
Check for viral hepatitis
HBV, HCV
Hepatocellular carcinoma (HCC)
Yearly ultrasound

## Slide 47
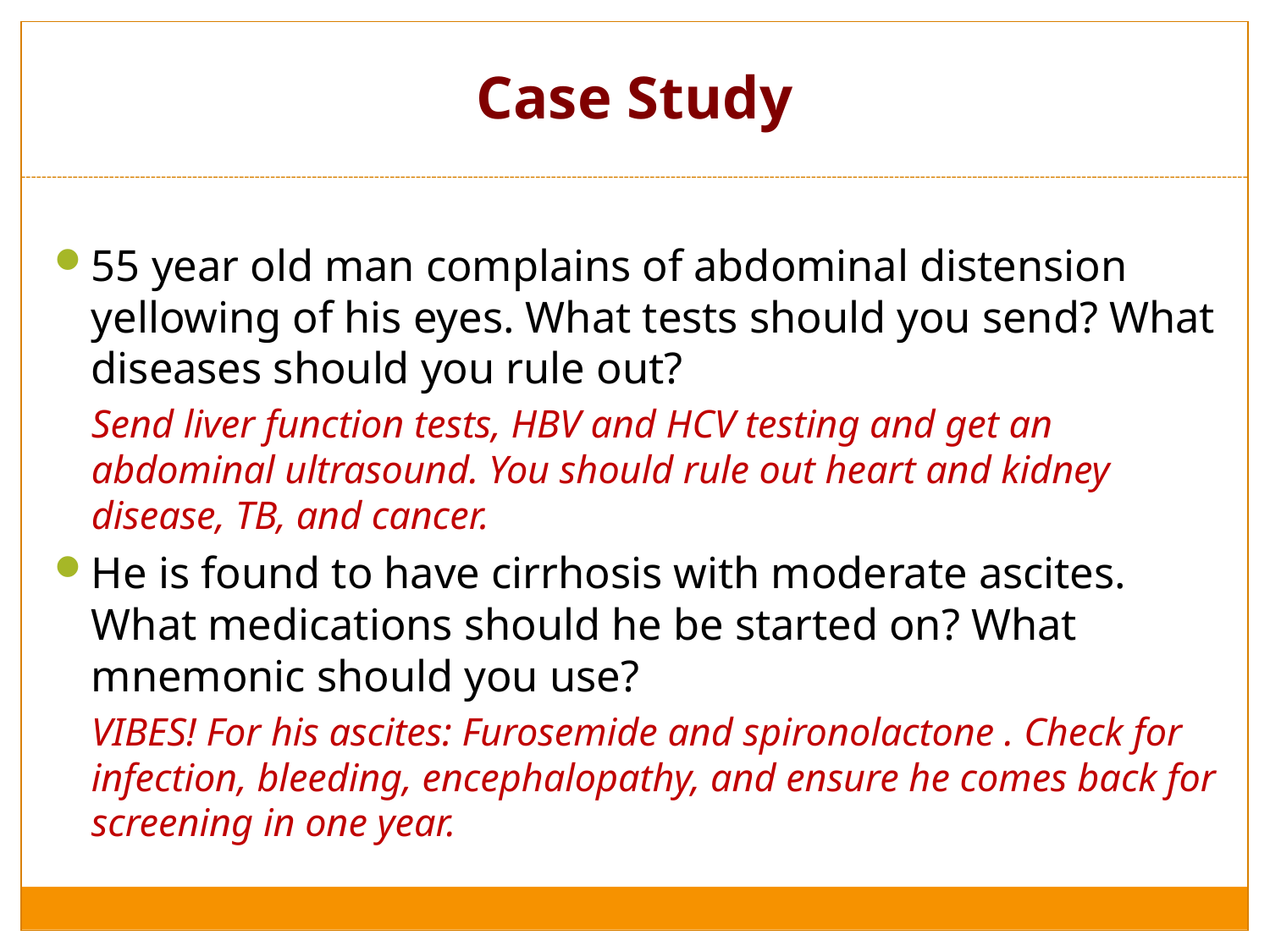

# Case Study
55 year old man complains of abdominal distension yellowing of his eyes. What tests should you send? What diseases should you rule out?
Send liver function tests, HBV and HCV testing and get an abdominal ultrasound. You should rule out heart and kidney disease, TB, and cancer.
He is found to have cirrhosis with moderate ascites. What medications should he be started on? What mnemonic should you use?
VIBES! For his ascites: Furosemide and spironolactone . Check for infection, bleeding, encephalopathy, and ensure he comes back for screening in one year.

## Slide 48
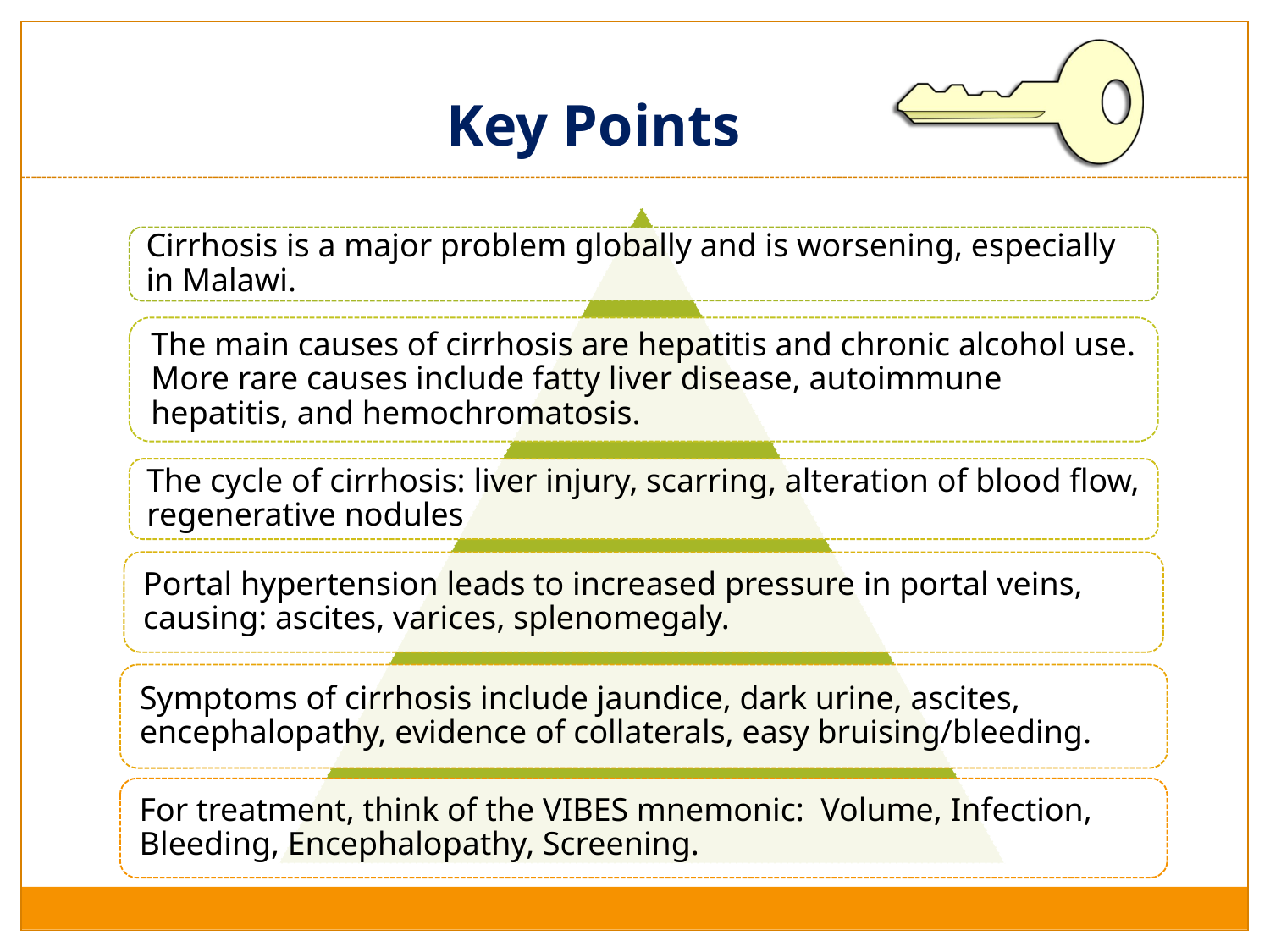

# Key Points

## Slide 49
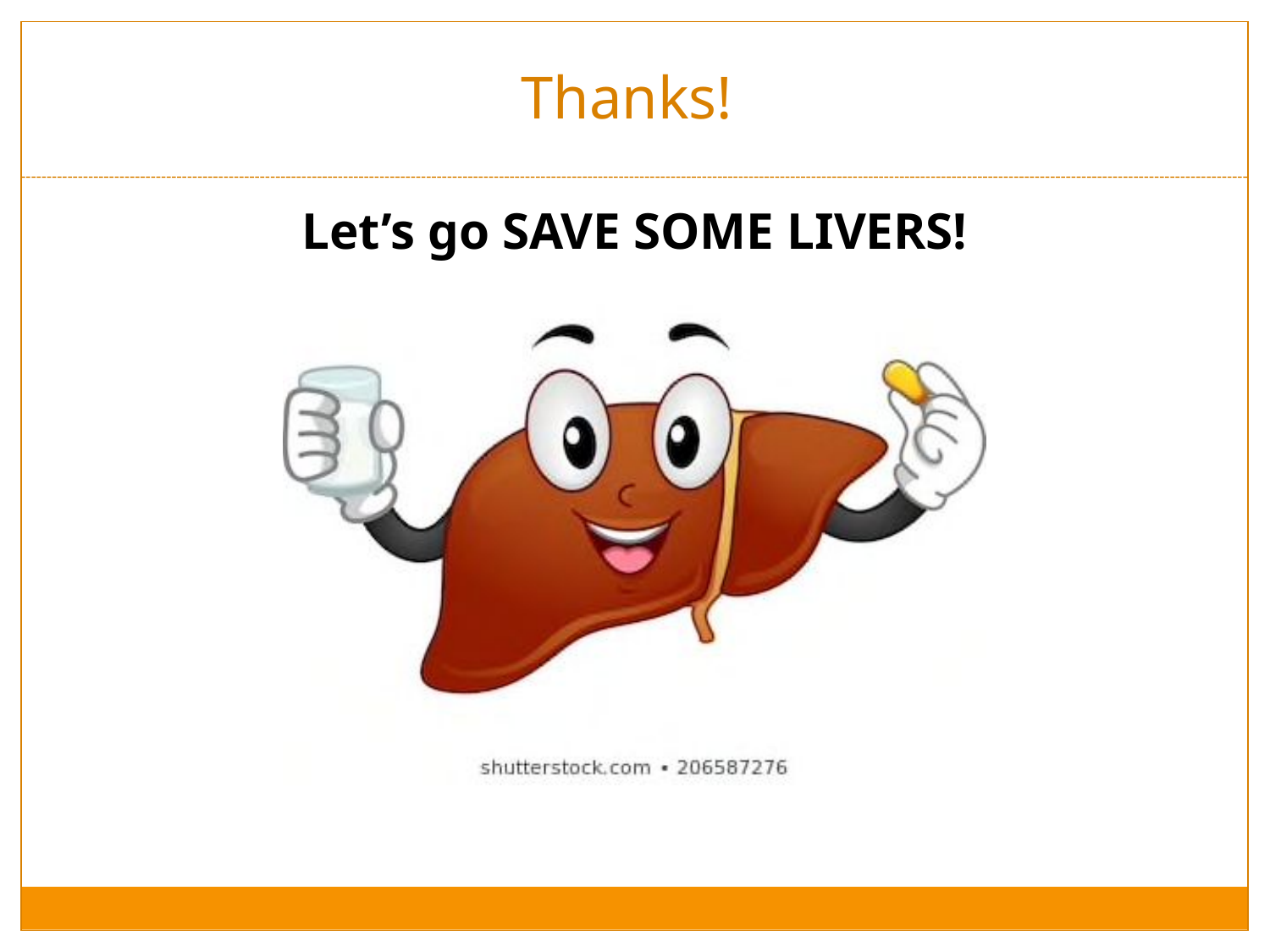

# Thanks!
Let’s go SAVE SOME LIVERS!
