# Supplementary material for: Training Mid-Level Providers to Treat Severe Non-Communicable Diseases in Neno, Malawi through PEN-Plus Strategies
Source: Ann Glob Health. 2022 Aug 11;88(1):69. doi: 10.5334/aogh.3750 (PMC9389951; doi:10.5334/aogh.3750)
Supplement: Didactic Materials. — The supplementary materials contain a suggested didactic training schedule and the PowerPoint presentations used for PEN-Plus training in Neno, Malawi. These materials have been reviewed and accepted by the Malawi Ministry of Health for future PEN-Plus trainings in Malawi. [file agh-88-1-3750-s2.zip › Didactic_Materials/H_Sickle Cell_edited 10.2.22[81] - Read-Only.pptx]

## Slide 1
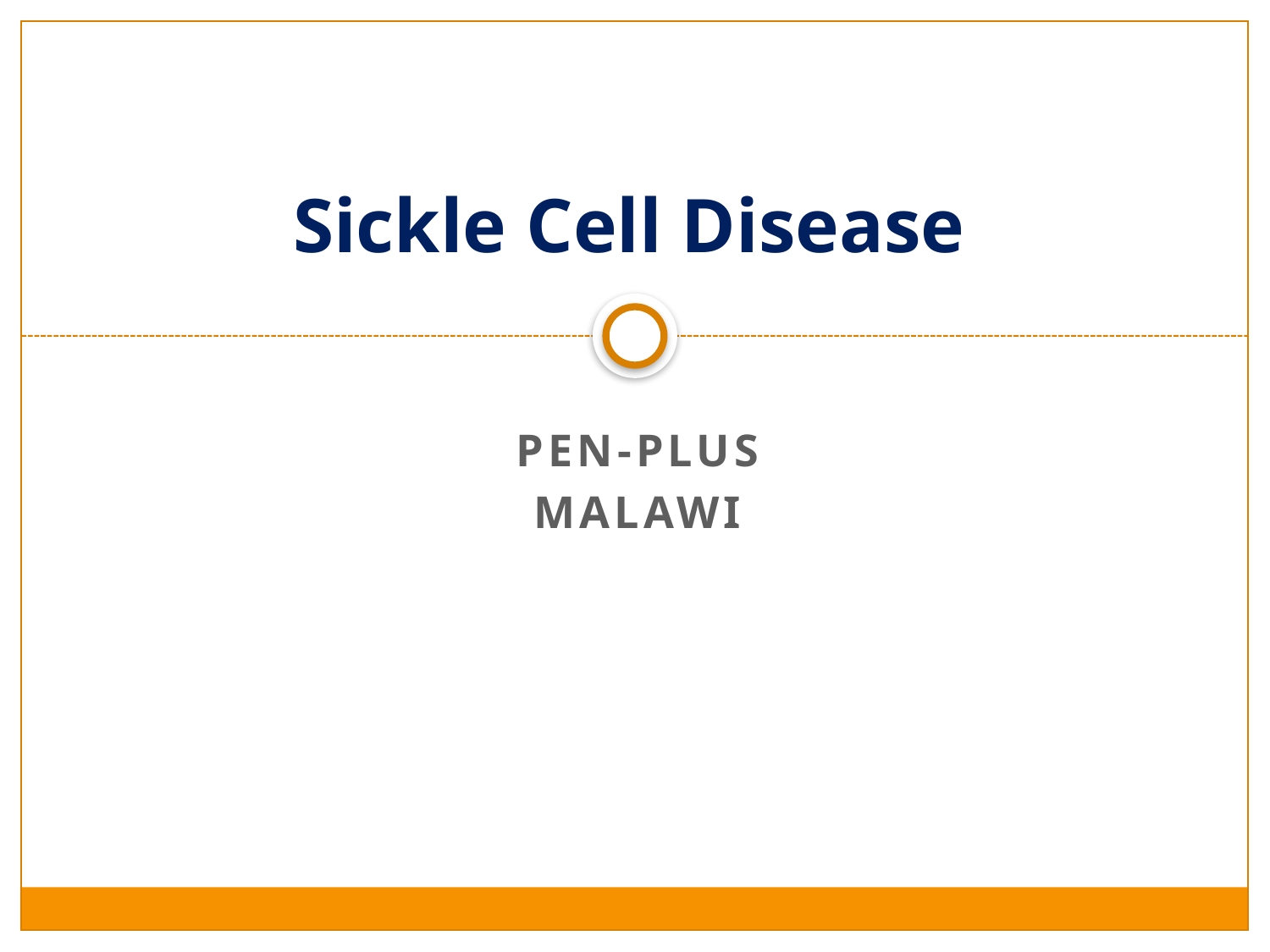

# Sickle Cell Disease
PEN-Plus
Malawi

## Slide 2
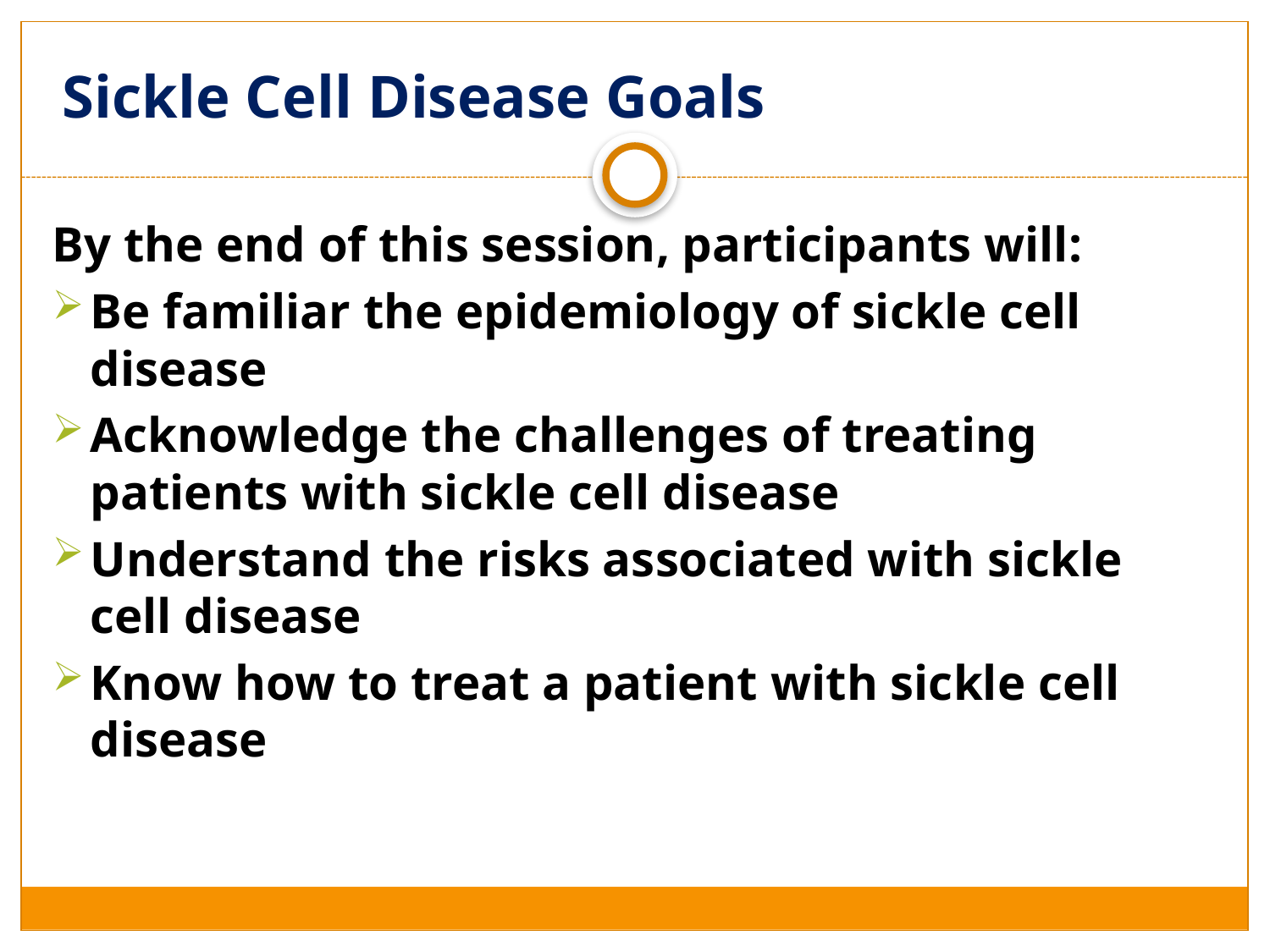

# Sickle Cell Disease Goals
By the end of this session, participants will:
Be familiar the epidemiology of sickle cell disease
Acknowledge the challenges of treating patients with sickle cell disease
Understand the risks associated with sickle cell disease
Know how to treat a patient with sickle cell disease

## Slide 3
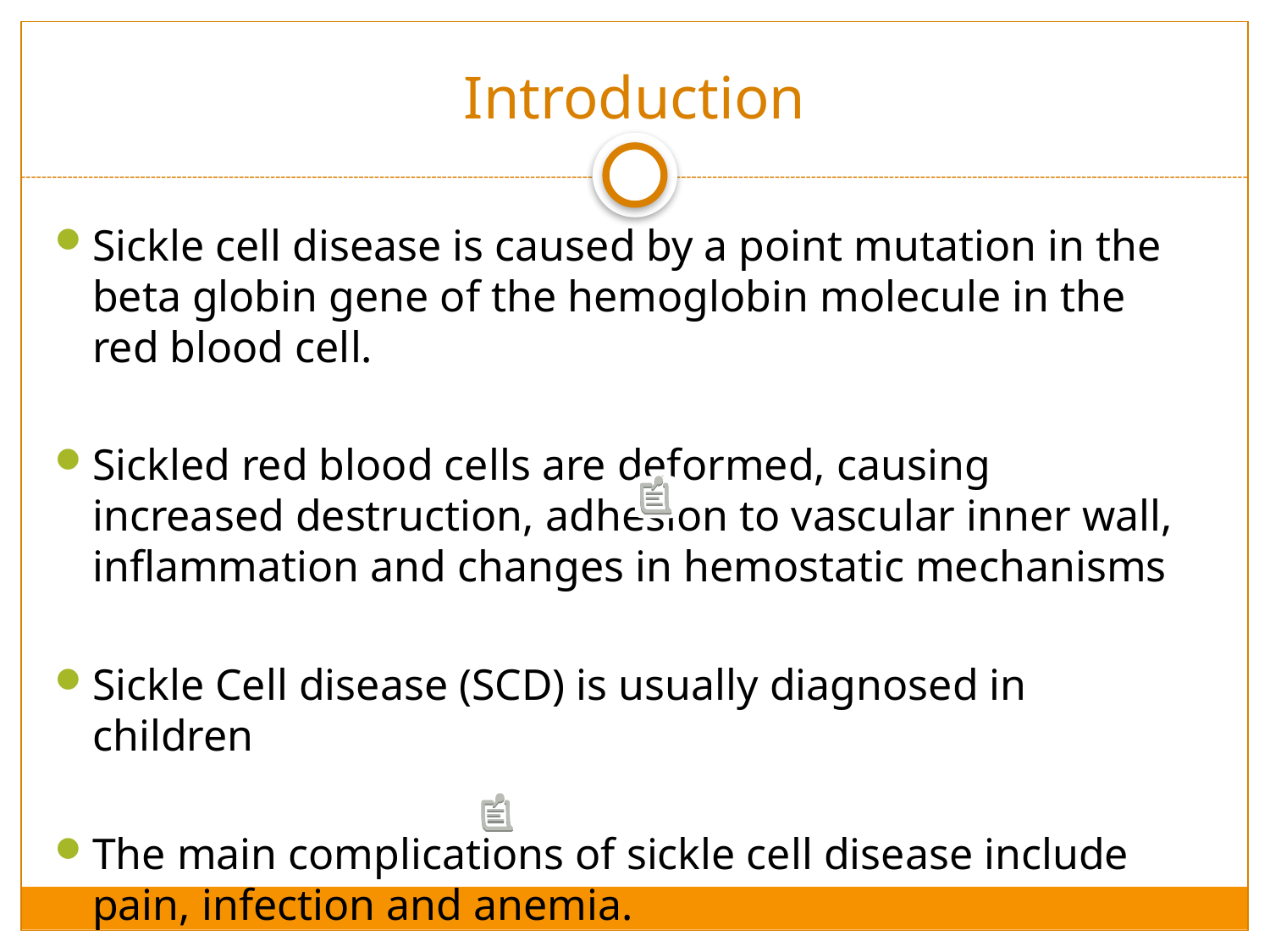

# Introduction
Sickle cell disease is caused by a point mutation in the beta globin gene of the hemoglobin molecule in the red blood cell.
Sickled red blood cells are deformed, causing increased destruction, adhesion to vascular inner wall, inflammation and changes in hemostatic mechanisms
Sickle Cell disease (SCD) is usually diagnosed in children
The main complications of sickle cell disease include pain, infection and anemia.

## Slide 4
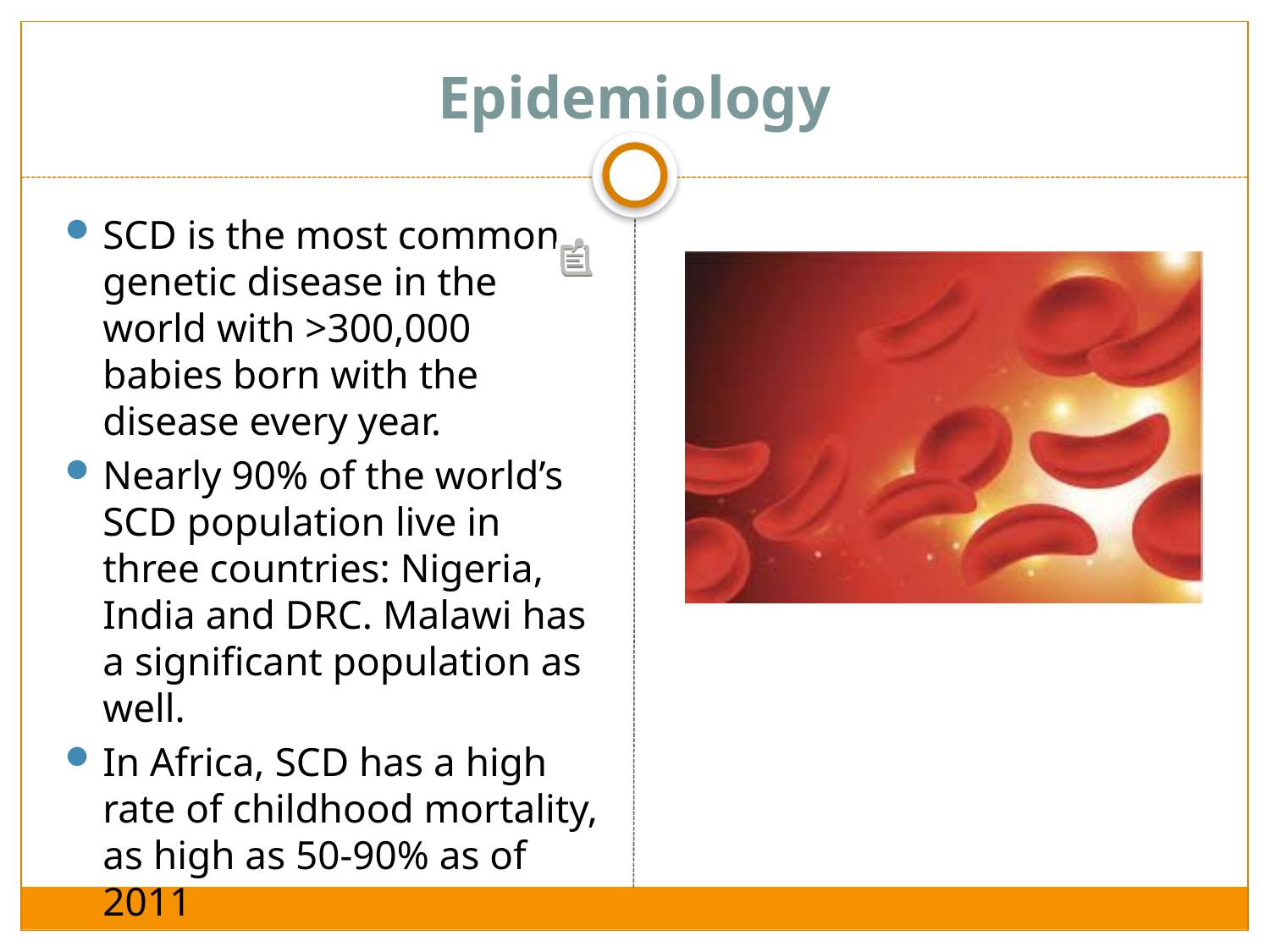

# Epidemiology
SCD is the most common genetic disease in the world with >300,000 babies born with the disease every year.
Nearly 90% of the world’s SCD population live in three countries: Nigeria, India and DRC. Malawi has a significant population as well.
In Africa, SCD has a high rate of childhood mortality, as high as 50-90% as of 2011

## Slide 5
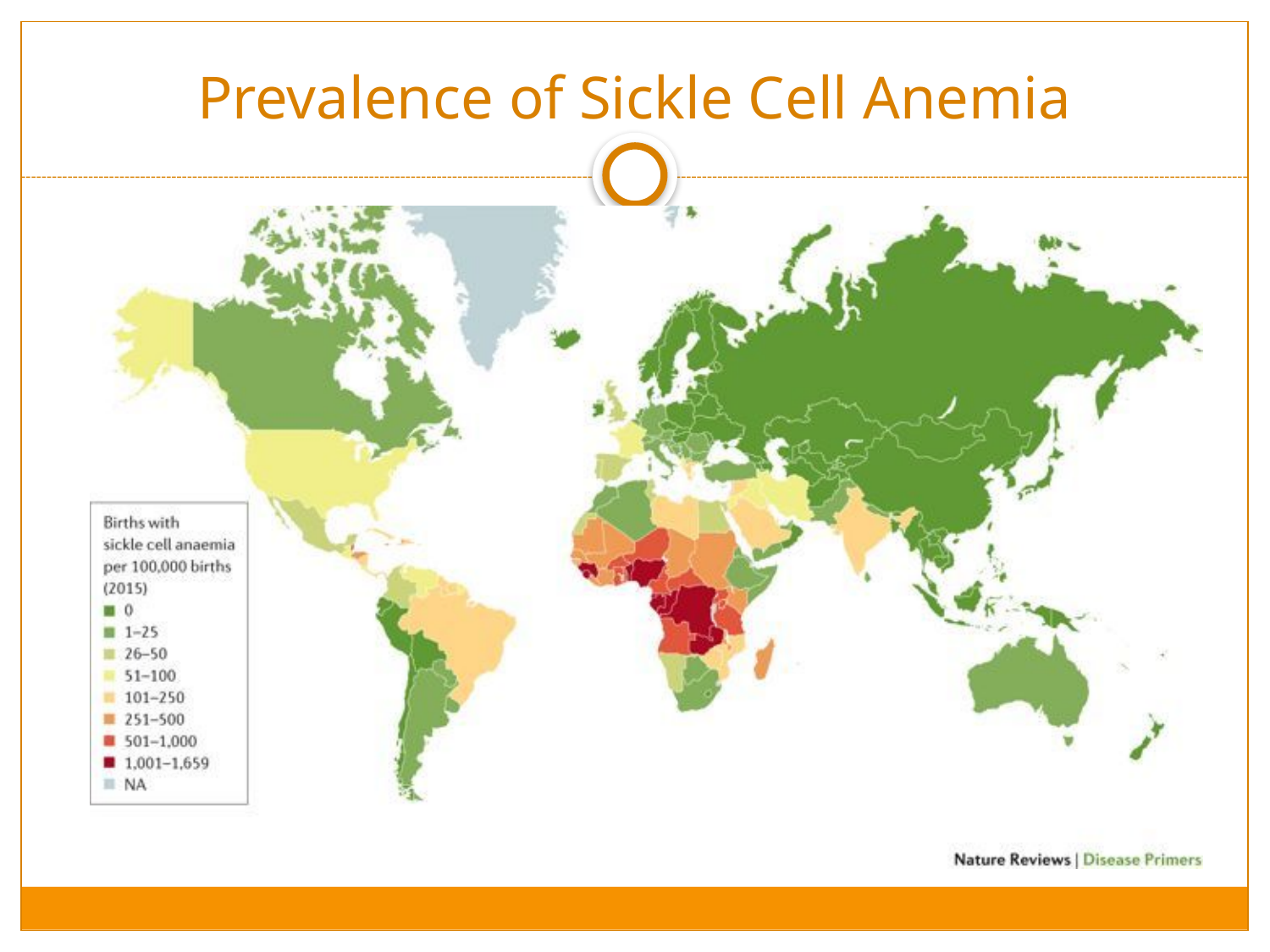

# Prevalence of Sickle Cell Anemia

## Slide 6
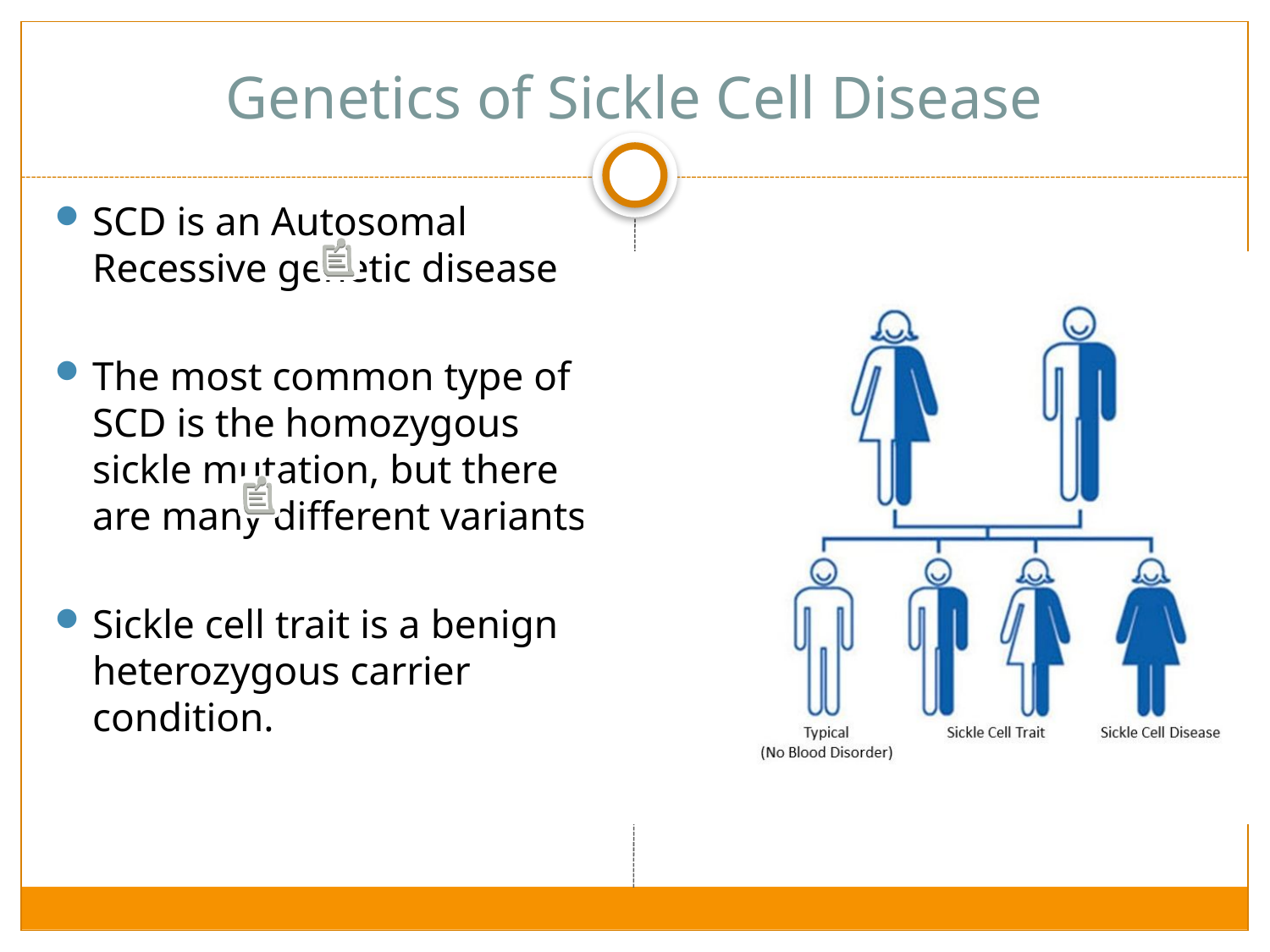

# Genetics of Sickle Cell Disease
SCD is an Autosomal Recessive genetic disease
The most common type of SCD is the homozygous sickle mutation, but there are many different variants
Sickle cell trait is a benign heterozygous carrier condition.

## Slide 7
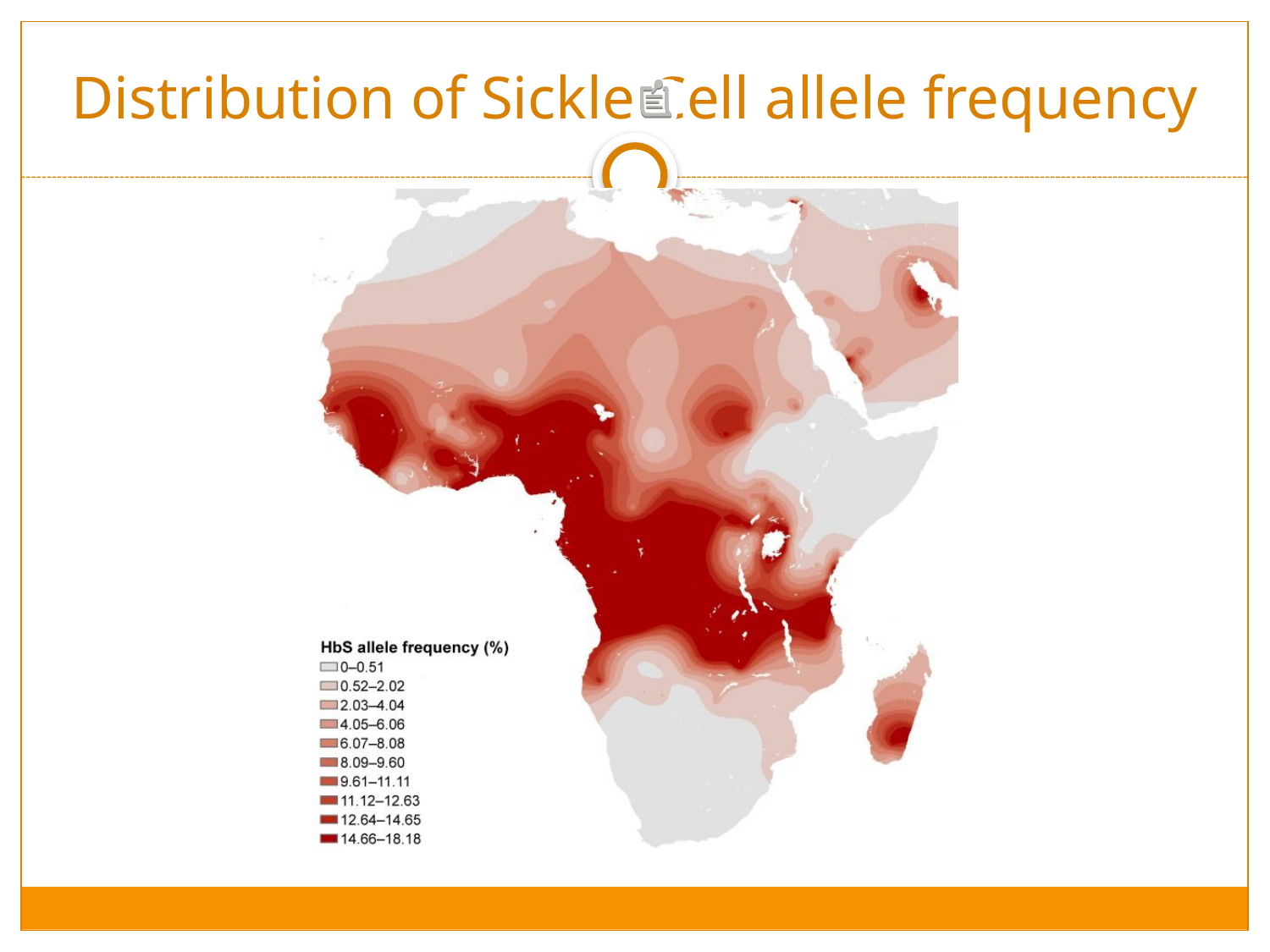

# Distribution of Sickle Cell allele frequency

## Slide 8
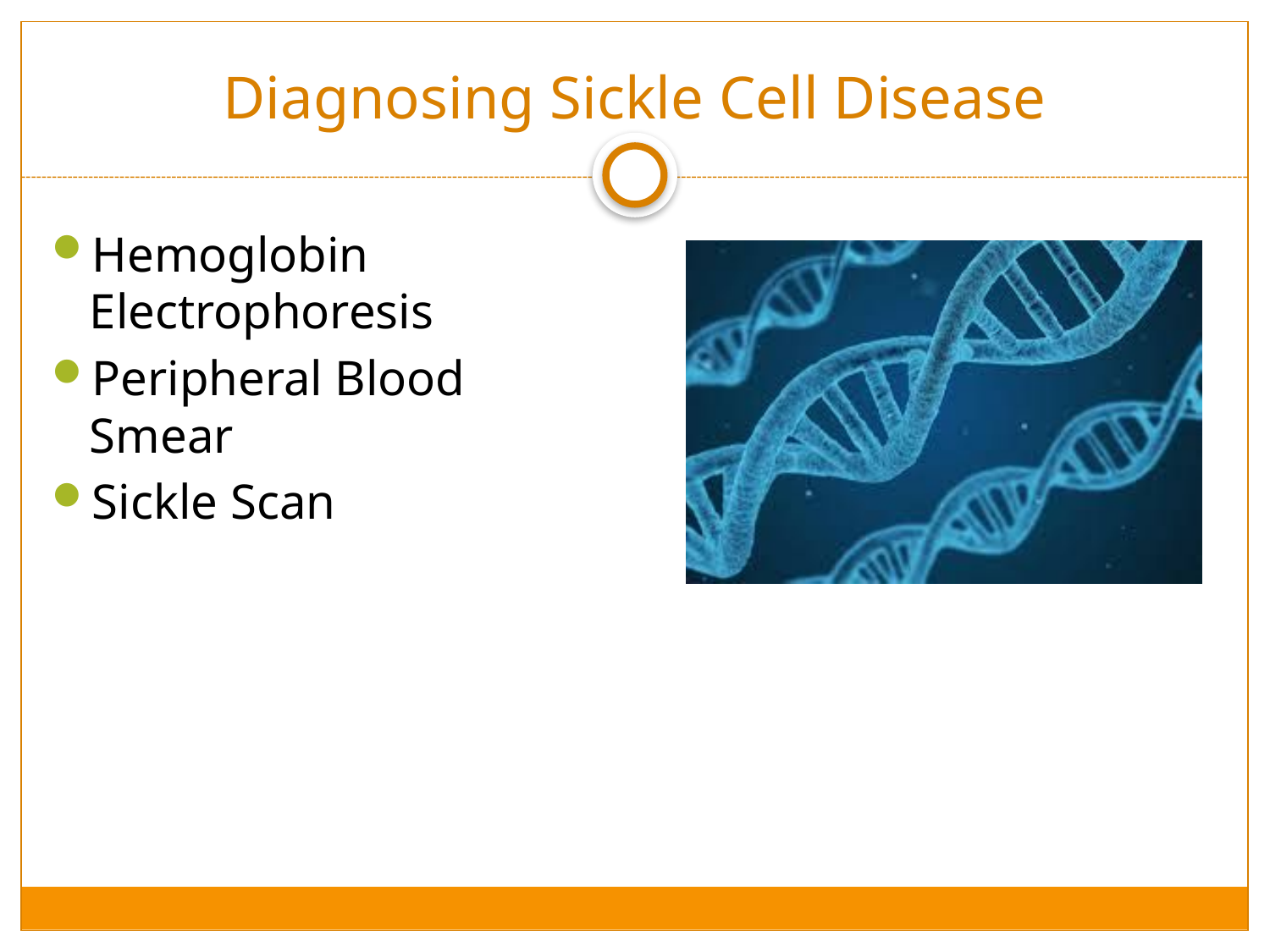

# Diagnosing Sickle Cell Disease
Hemoglobin Electrophoresis
Peripheral Blood Smear
Sickle Scan

## Slide 9
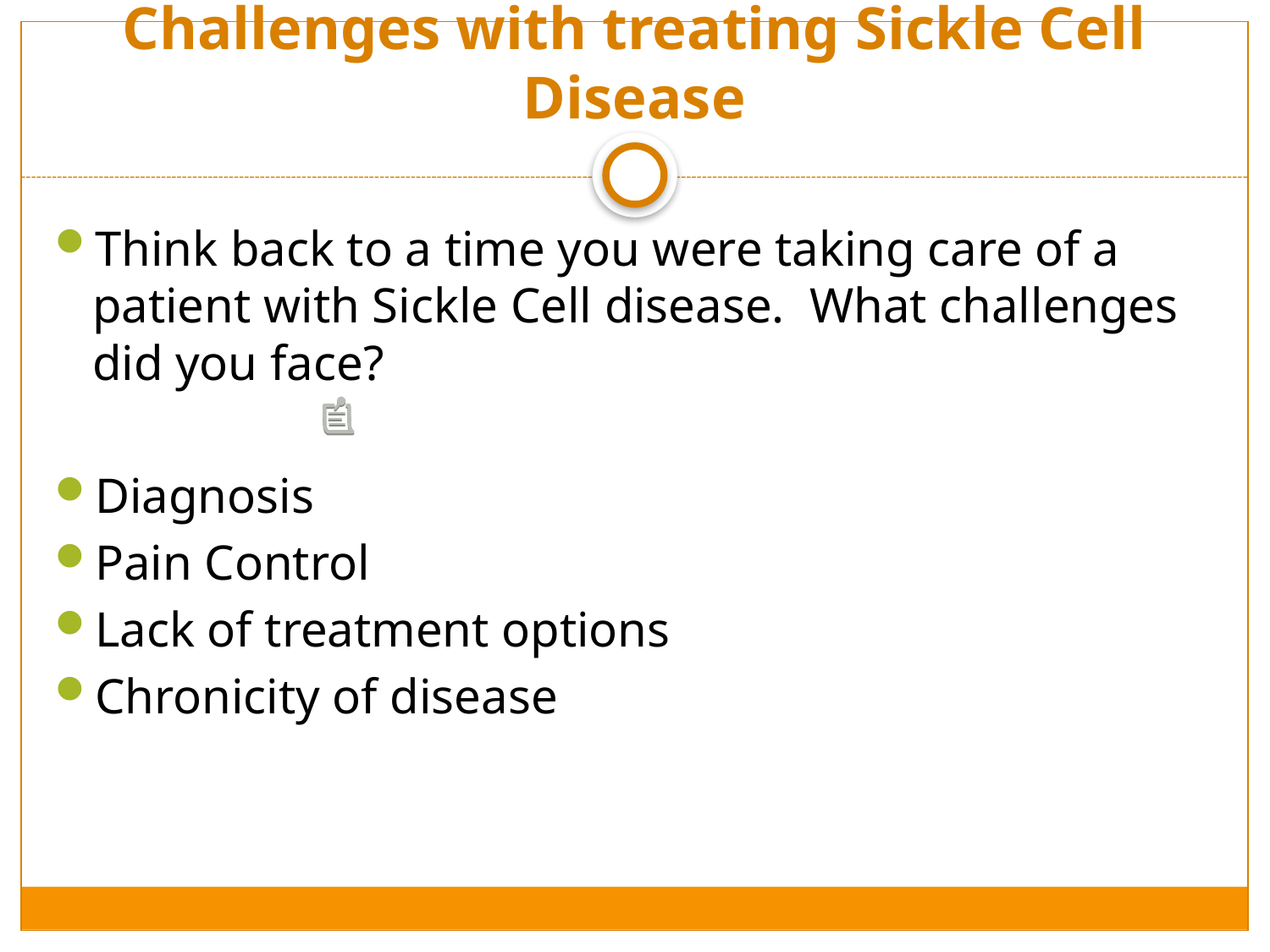

# Challenges with treating Sickle Cell Disease
Think back to a time you were taking care of a patient with Sickle Cell disease. What challenges did you face?
Diagnosis
Pain Control
Lack of treatment options
Chronicity of disease

## Slide 10
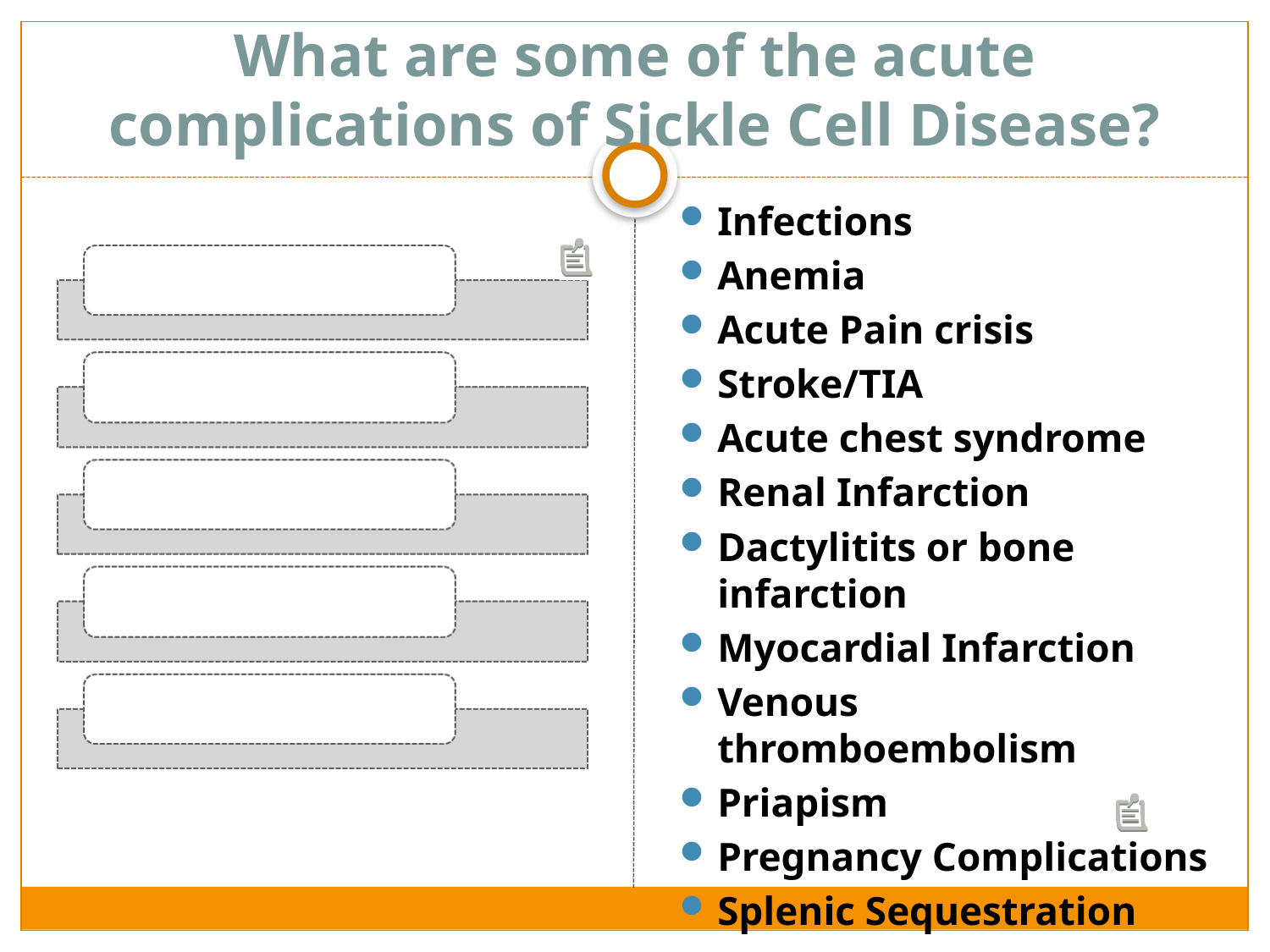

# What are some of the acute complications of Sickle Cell Disease?
Infections
Anemia
Acute Pain crisis
Stroke/TIA
Acute chest syndrome
Renal Infarction
Dactylitits or bone infarction
Myocardial Infarction
Venous thromboembolism
Priapism
Pregnancy Complications
Splenic Sequestration

## Slide 11
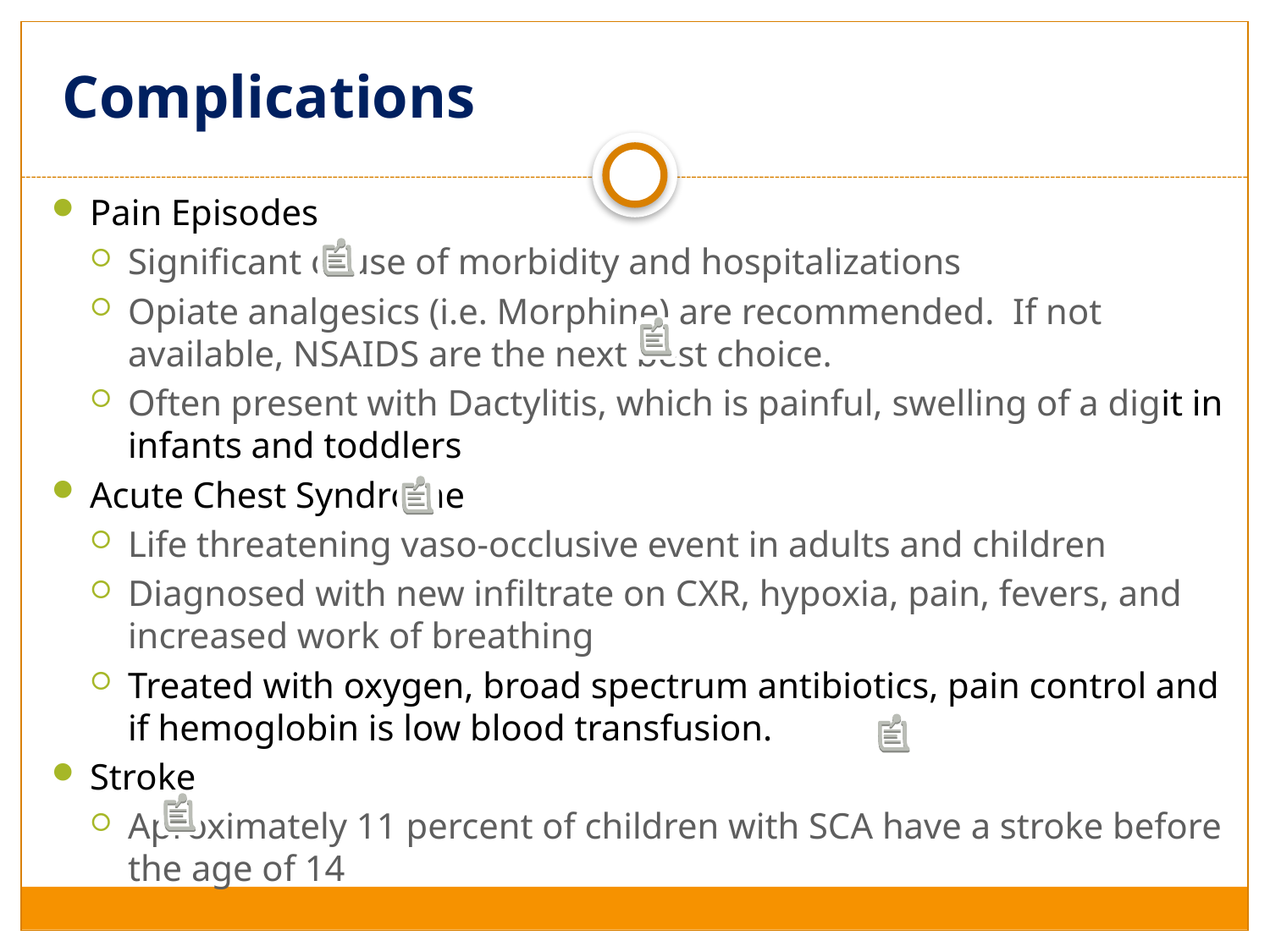

# Complications
Pain Episodes
Significant cause of morbidity and hospitalizations
Opiate analgesics (i.e. Morphine) are recommended. If not available, NSAIDS are the next best choice.
Often present with Dactylitis, which is painful, swelling of a digit in infants and toddlers
Acute Chest Syndrome
Life threatening vaso-occlusive event in adults and children
Diagnosed with new infiltrate on CXR, hypoxia, pain, fevers, and increased work of breathing
Treated with oxygen, broad spectrum antibiotics, pain control and if hemoglobin is low blood transfusion.
Stroke
Aproximately 11 percent of children with SCA have a stroke before the age of 14

## Slide 12
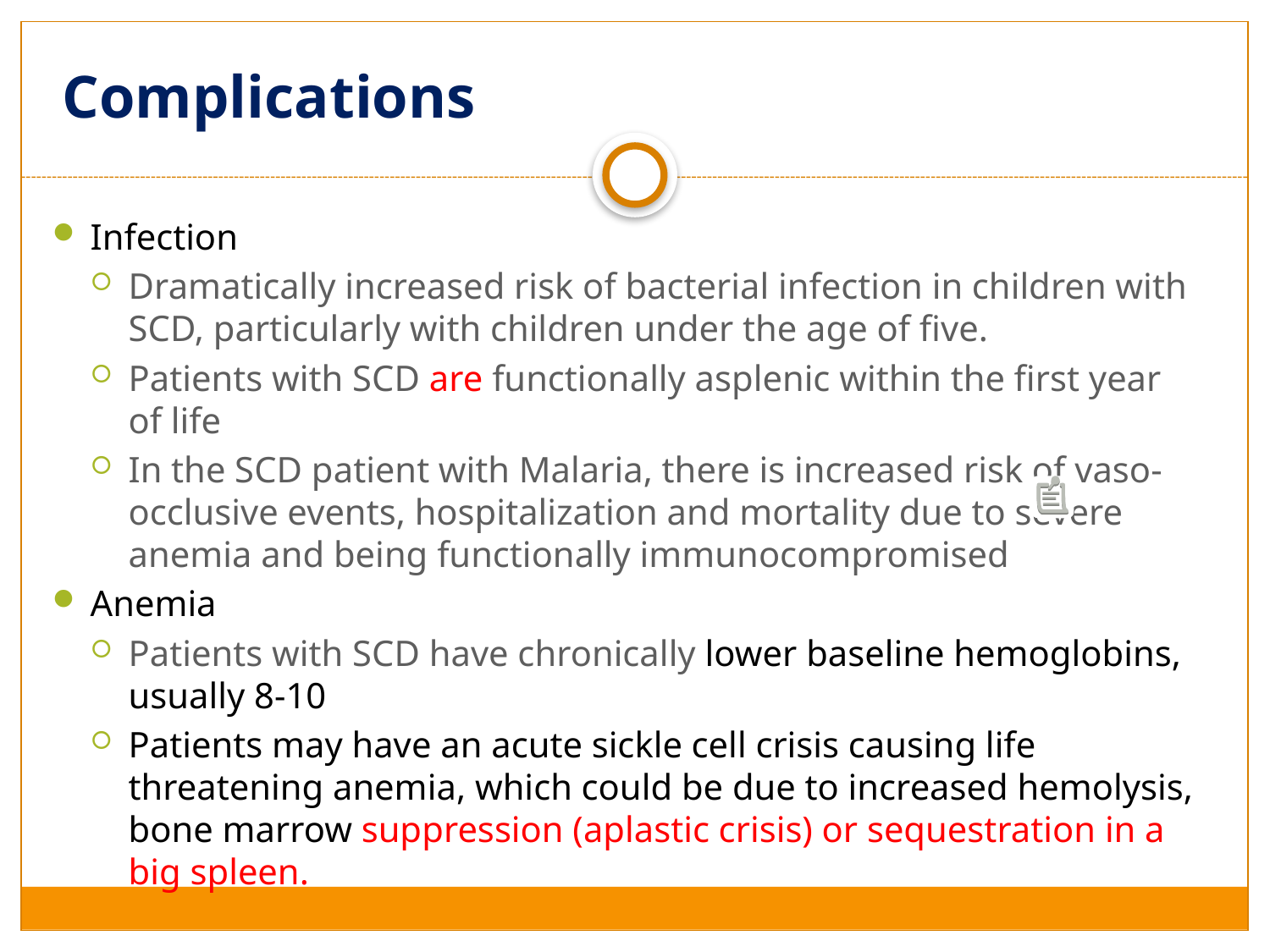

# Complications
Infection
Dramatically increased risk of bacterial infection in children with SCD, particularly with children under the age of five.
Patients with SCD are functionally asplenic within the first year of life
In the SCD patient with Malaria, there is increased risk of vaso-occlusive events, hospitalization and mortality due to severe anemia and being functionally immunocompromised
Anemia
Patients with SCD have chronically lower baseline hemoglobins, usually 8-10
Patients may have an acute sickle cell crisis causing life threatening anemia, which could be due to increased hemolysis, bone marrow suppression (aplastic crisis) or sequestration in a big spleen.

## Slide 13
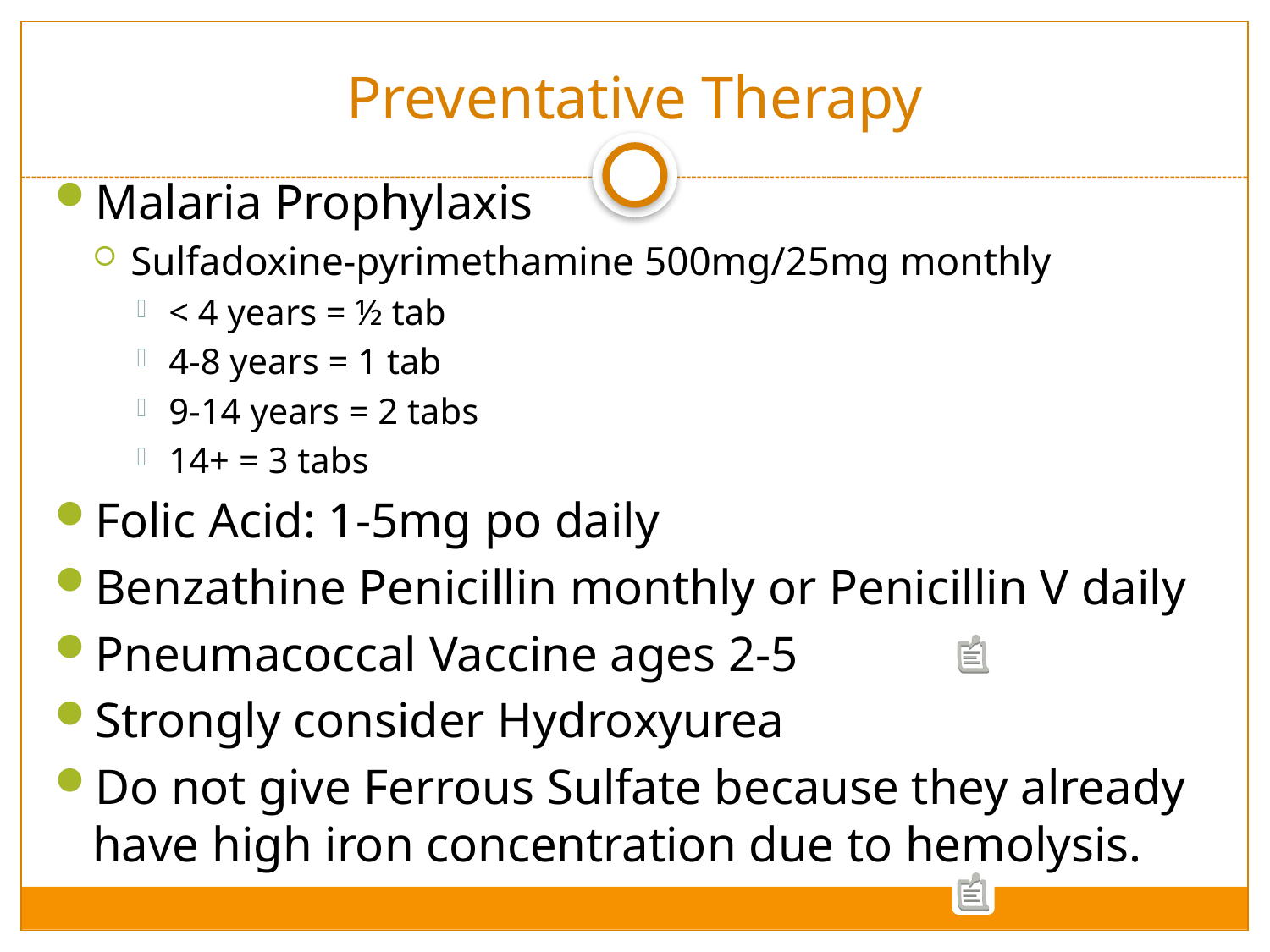

# Preventative Therapy
Malaria Prophylaxis
Sulfadoxine-pyrimethamine 500mg/25mg monthly
< 4 years = ½ tab
4-8 years = 1 tab
9-14 years = 2 tabs
14+ = 3 tabs
Folic Acid: 1-5mg po daily
Benzathine Penicillin monthly or Penicillin V daily
Pneumacoccal Vaccine ages 2-5
Strongly consider Hydroxyurea
Do not give Ferrous Sulfate because they already have high iron concentration due to hemolysis.

## Slide 14
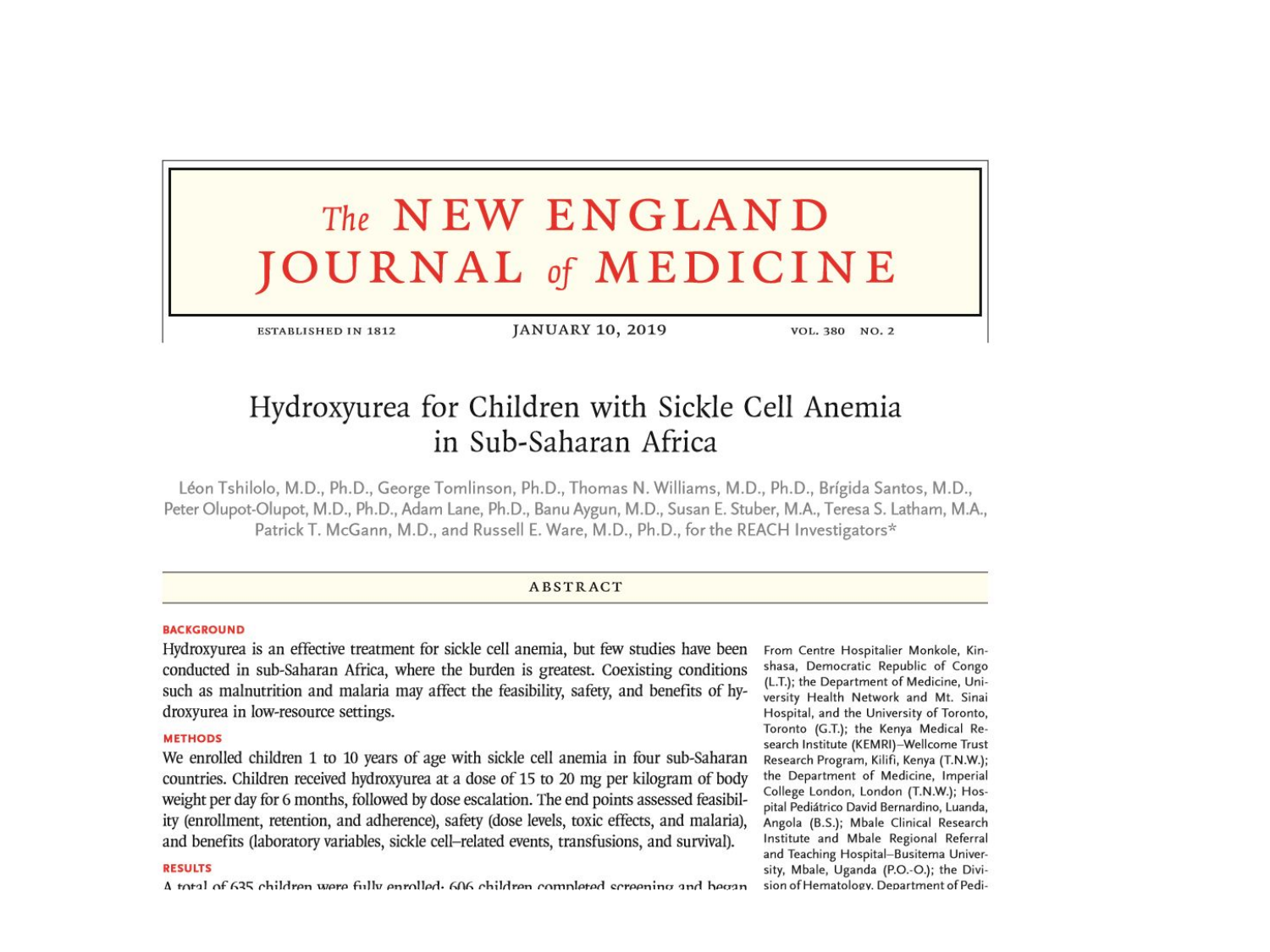

# Medications

## Slide 15
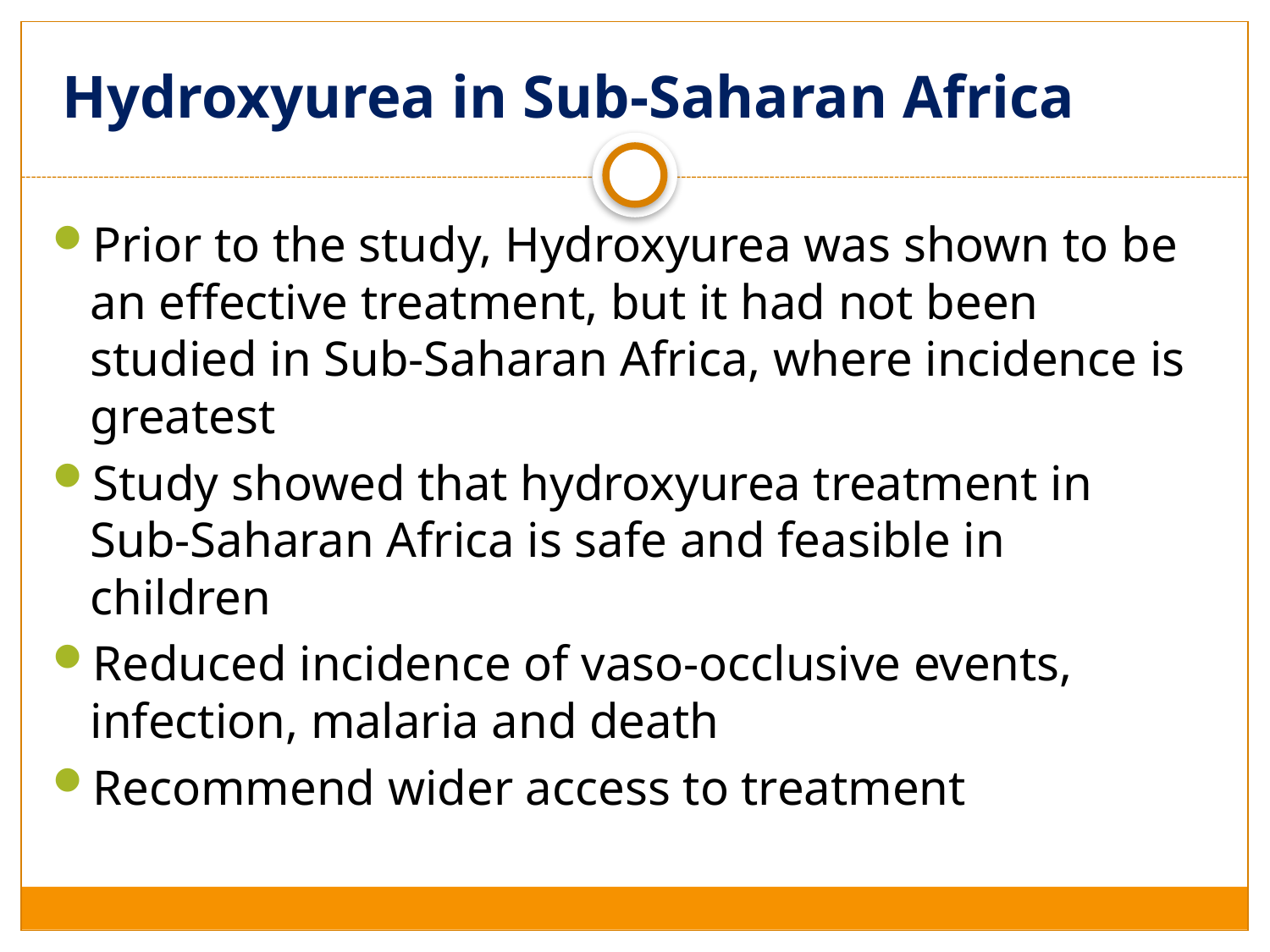

# Hydroxyurea in Sub-Saharan Africa
Prior to the study, Hydroxyurea was shown to be an effective treatment, but it had not been studied in Sub-Saharan Africa, where incidence is greatest
Study showed that hydroxyurea treatment in Sub-Saharan Africa is safe and feasible in children
Reduced incidence of vaso-occlusive events, infection, malaria and death
Recommend wider access to treatment

## Slide 16
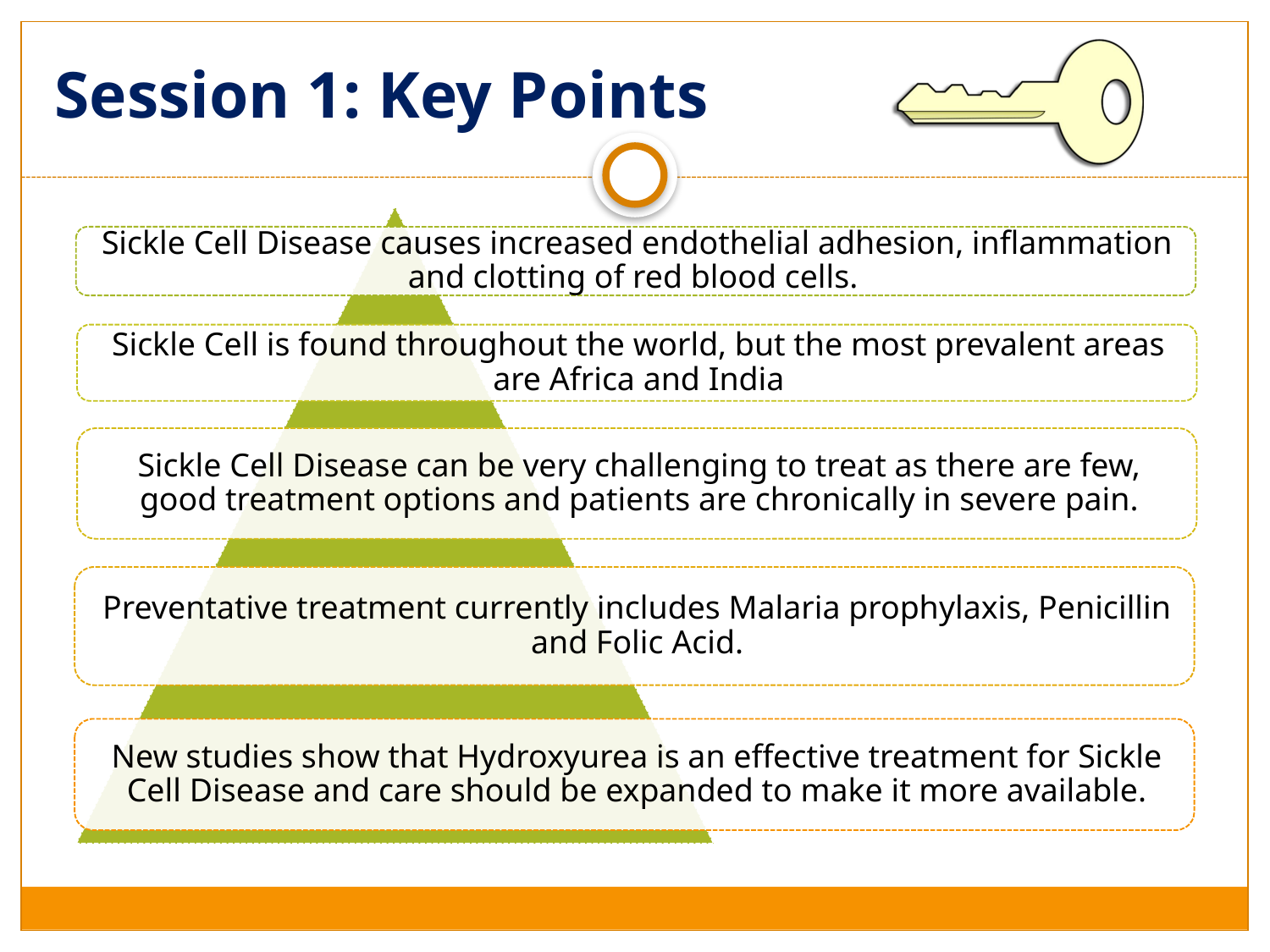

# Session 1: Key Points
