# Supplementary material for: Training Mid-Level Providers to Treat Severe Non-Communicable Diseases in Neno, Malawi through PEN-Plus Strategies
Source: Ann Glob Health. 2022 Aug 11;88(1):69. doi: 10.5334/aogh.3750 (PMC9389951; doi:10.5334/aogh.3750)
Supplement: Didactic Materials. — The supplementary materials contain a suggested didactic training schedule and the PowerPoint presentations used for PEN-Plus training in Neno, Malawi. These materials have been reviewed and accepted by the Malawi Ministry of Health for future PEN-Plus trainings in Malawi. [file agh-88-1-3750-s2.zip › Didactic_Materials/N_Epilepsy.pptx]

## Slide 1
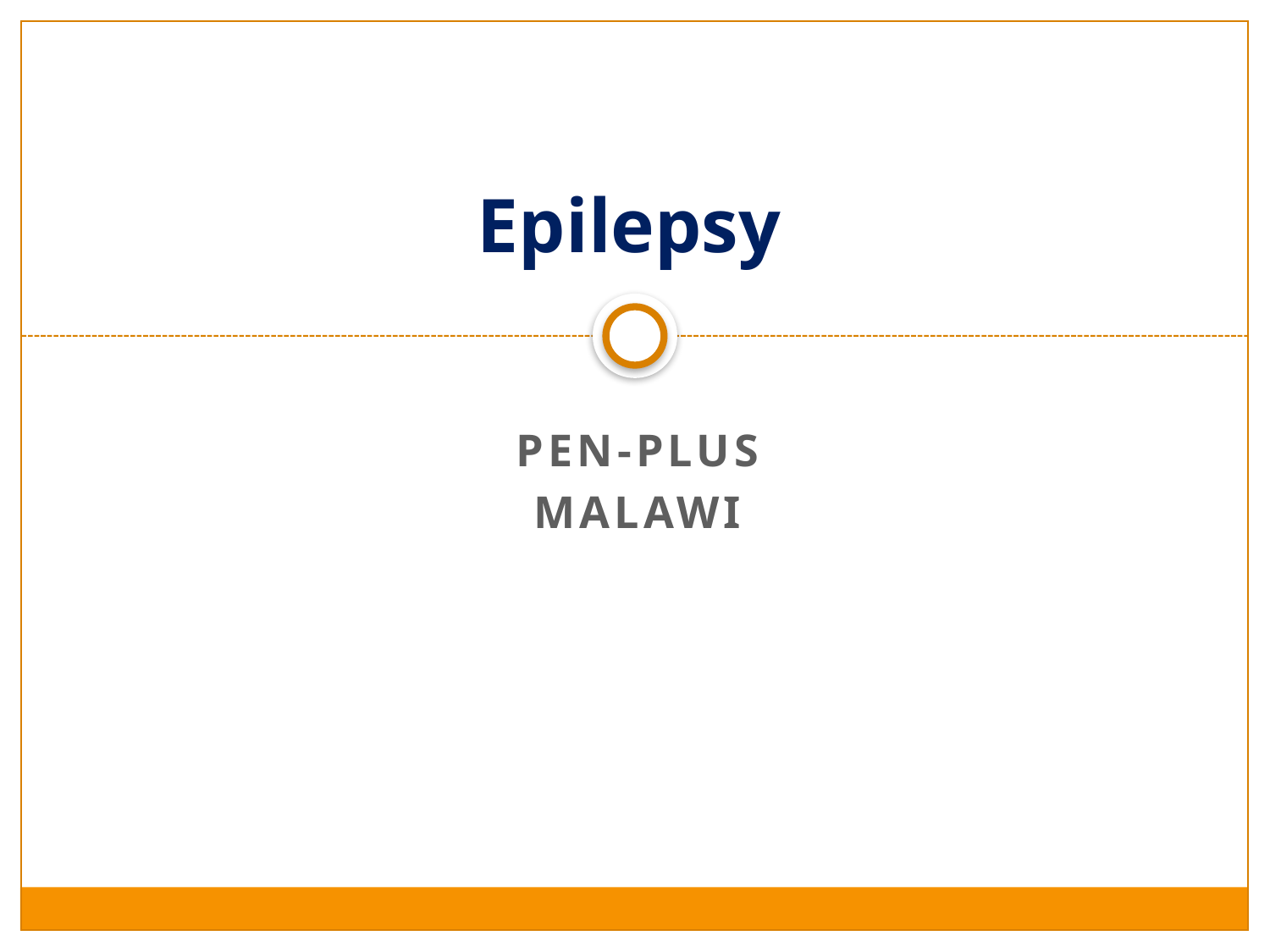

# Epilepsy
PEN-Plus
Malawi

## Slide 2
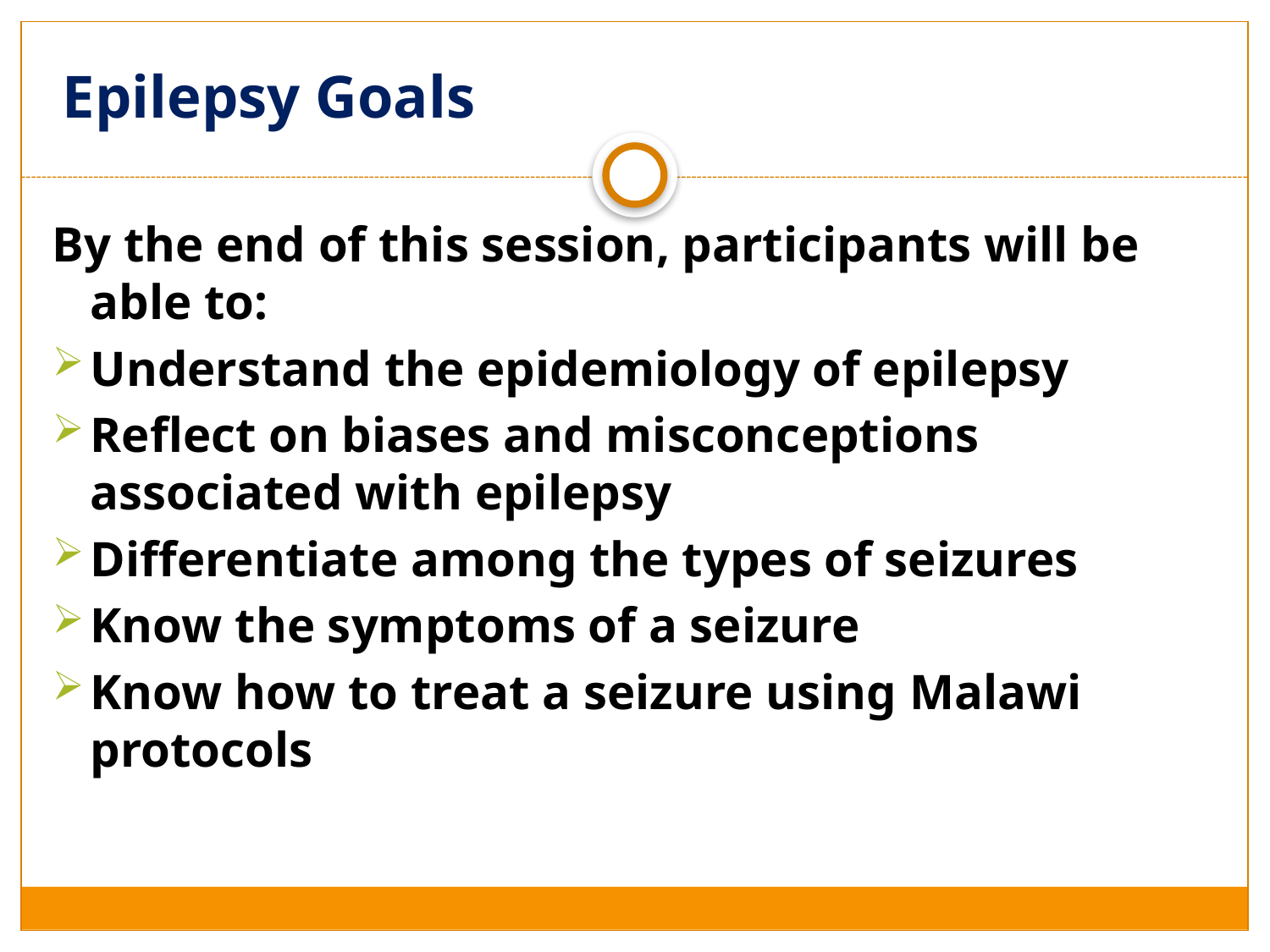

# Epilepsy Goals
By the end of this session, participants will be able to:
Understand the epidemiology of epilepsy
Reflect on biases and misconceptions associated with epilepsy
Differentiate among the types of seizures
Know the symptoms of a seizure
Know how to treat a seizure using Malawi protocols

## Slide 3
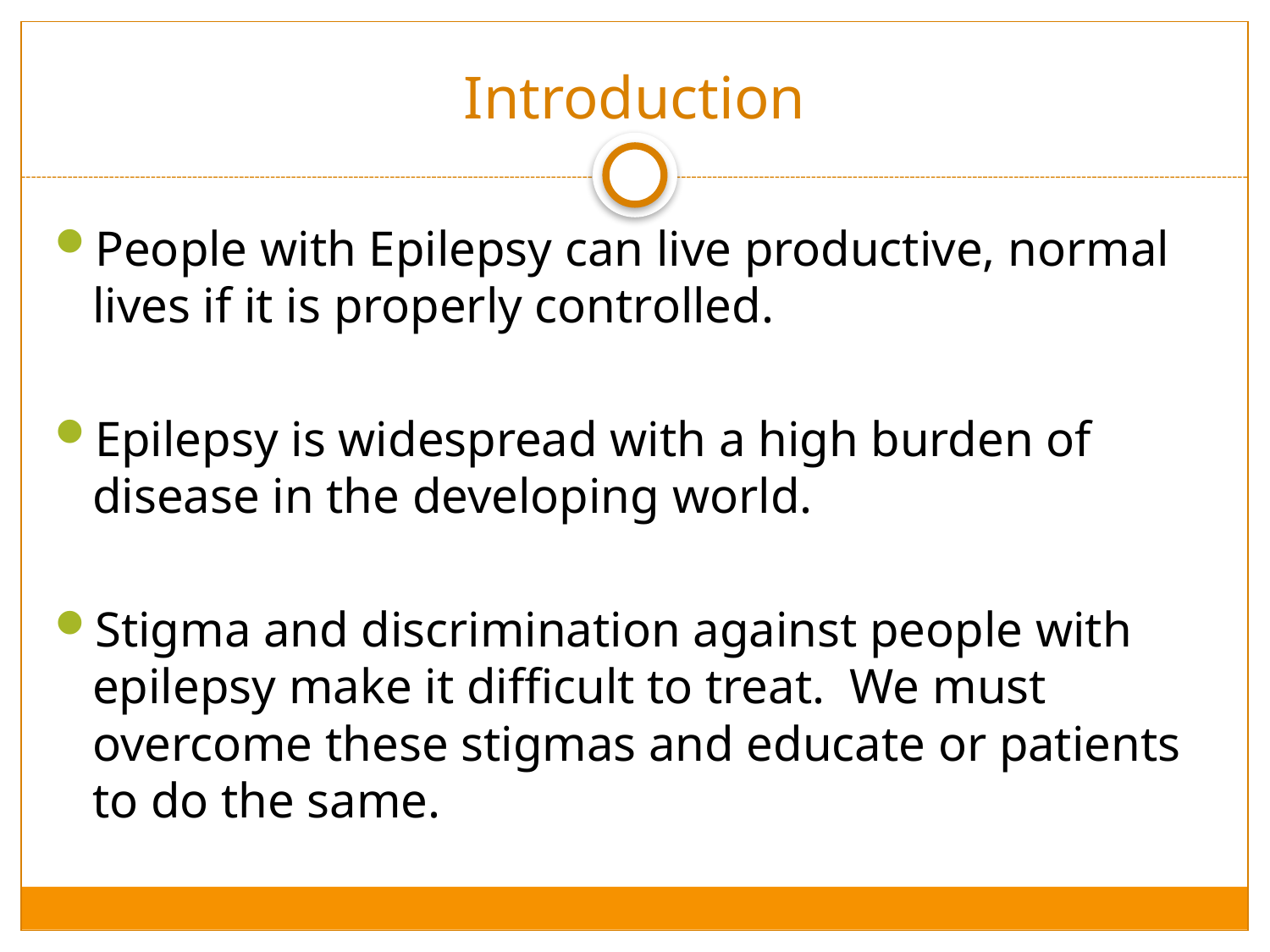

# Introduction
People with Epilepsy can live productive, normal lives if it is properly controlled.
Epilepsy is widespread with a high burden of disease in the developing world.
Stigma and discrimination against people with epilepsy make it difficult to treat. We must overcome these stigmas and educate or patients to do the same.

## Slide 4
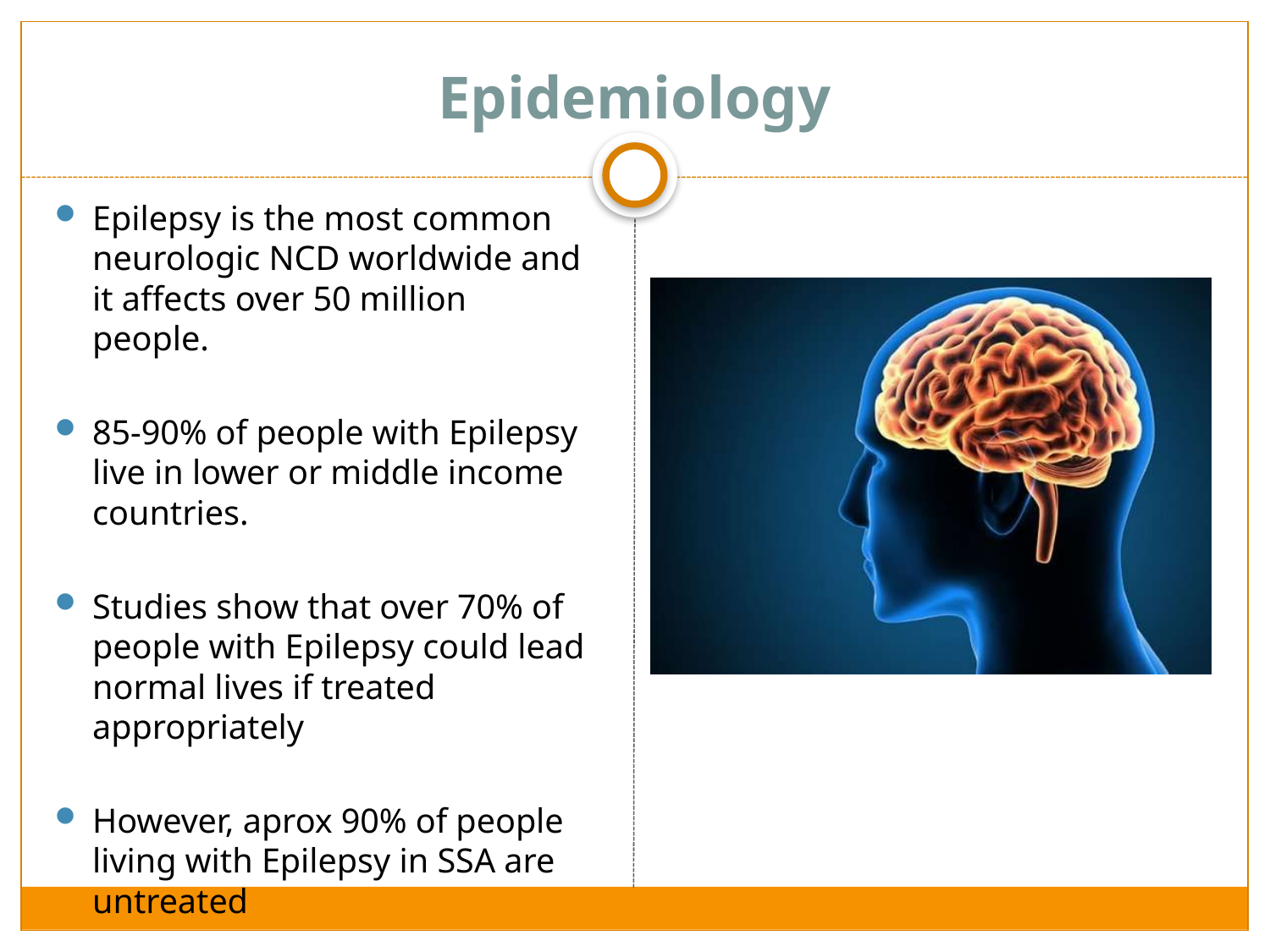

# Epidemiology
Epilepsy is the most common neurologic NCD worldwide and it affects over 50 million people.
85-90% of people with Epilepsy live in lower or middle income countries.
Studies show that over 70% of people with Epilepsy could lead normal lives if treated appropriately
However, aprox 90% of people living with Epilepsy in SSA are untreated

## Slide 5
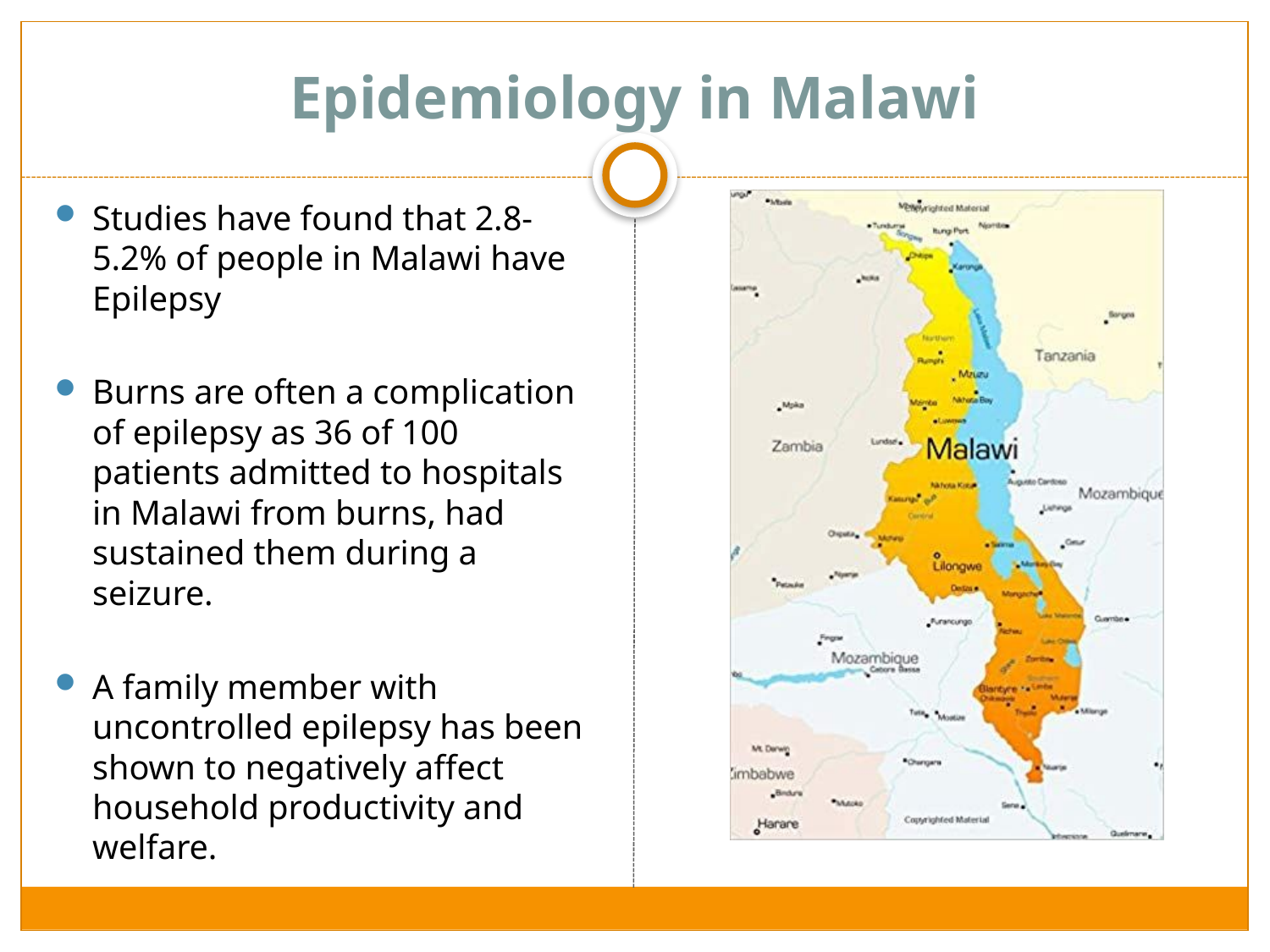

# Epidemiology in Malawi
Studies have found that 2.8-5.2% of people in Malawi have Epilepsy
Burns are often a complication of epilepsy as 36 of 100 patients admitted to hospitals in Malawi from burns, had sustained them during a seizure.
A family member with uncontrolled epilepsy has been shown to negatively affect household productivity and welfare.

## Slide 6
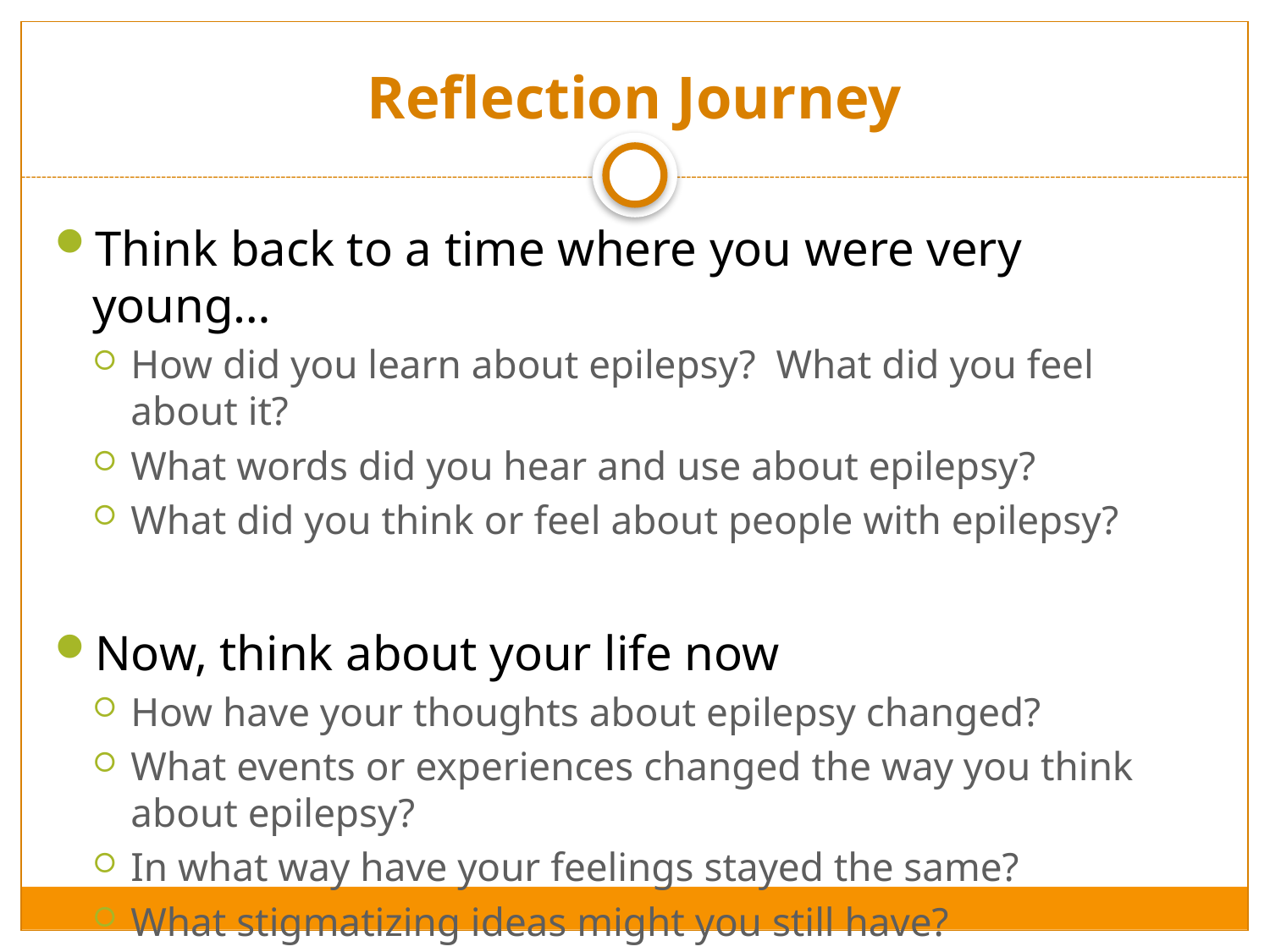

# Reflection Journey
Think back to a time where you were very young…
How did you learn about epilepsy? What did you feel about it?
What words did you hear and use about epilepsy?
What did you think or feel about people with epilepsy?
Now, think about your life now
How have your thoughts about epilepsy changed?
What events or experiences changed the way you think about epilepsy?
In what way have your feelings stayed the same?
What stigmatizing ideas might you still have?

## Slide 7
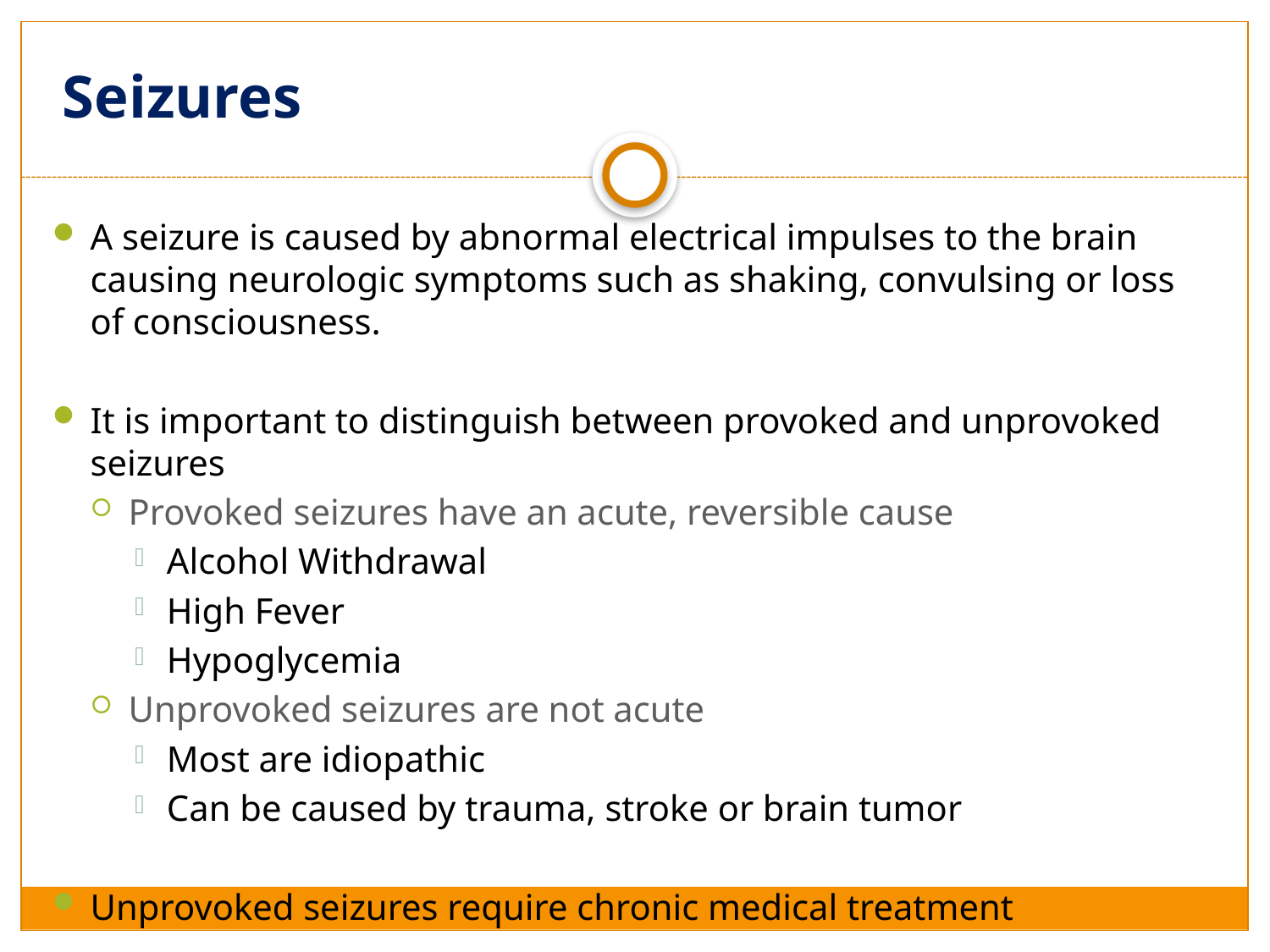

# Seizures
A seizure is caused by abnormal electrical impulses to the brain causing neurologic symptoms such as shaking, convulsing or loss of consciousness.
It is important to distinguish between provoked and unprovoked seizures
Provoked seizures have an acute, reversible cause
Alcohol Withdrawal
High Fever
Hypoglycemia
Unprovoked seizures are not acute
Most are idiopathic
Can be caused by trauma, stroke or brain tumor
Unprovoked seizures require chronic medical treatment

## Slide 8
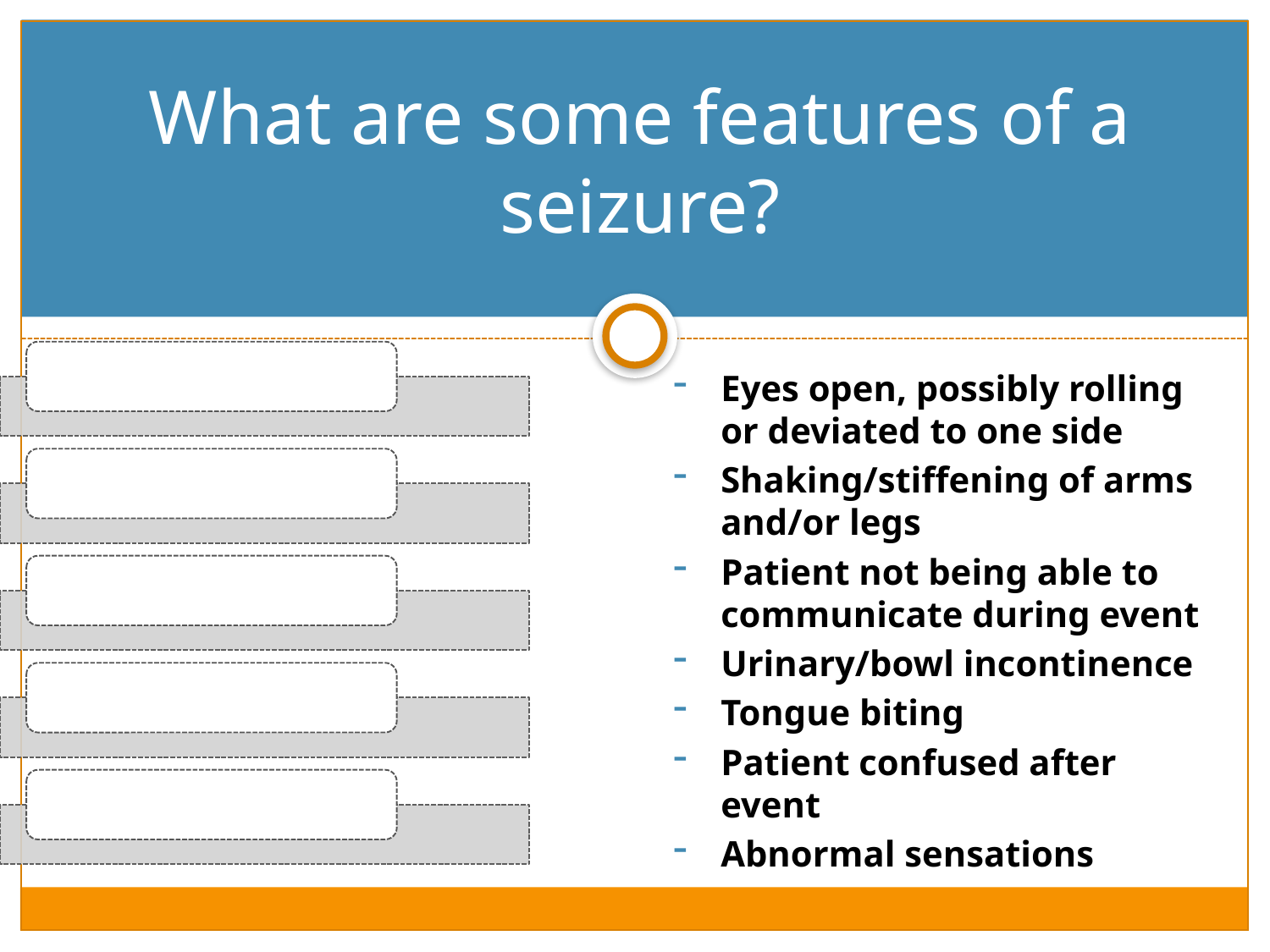

# What are some features of a seizure?
Eyes open, possibly rolling or deviated to one side
Shaking/stiffening of arms and/or legs
Patient not being able to communicate during event
Urinary/bowl incontinence
Tongue biting
Patient confused after event
Abnormal sensations

## Slide 9
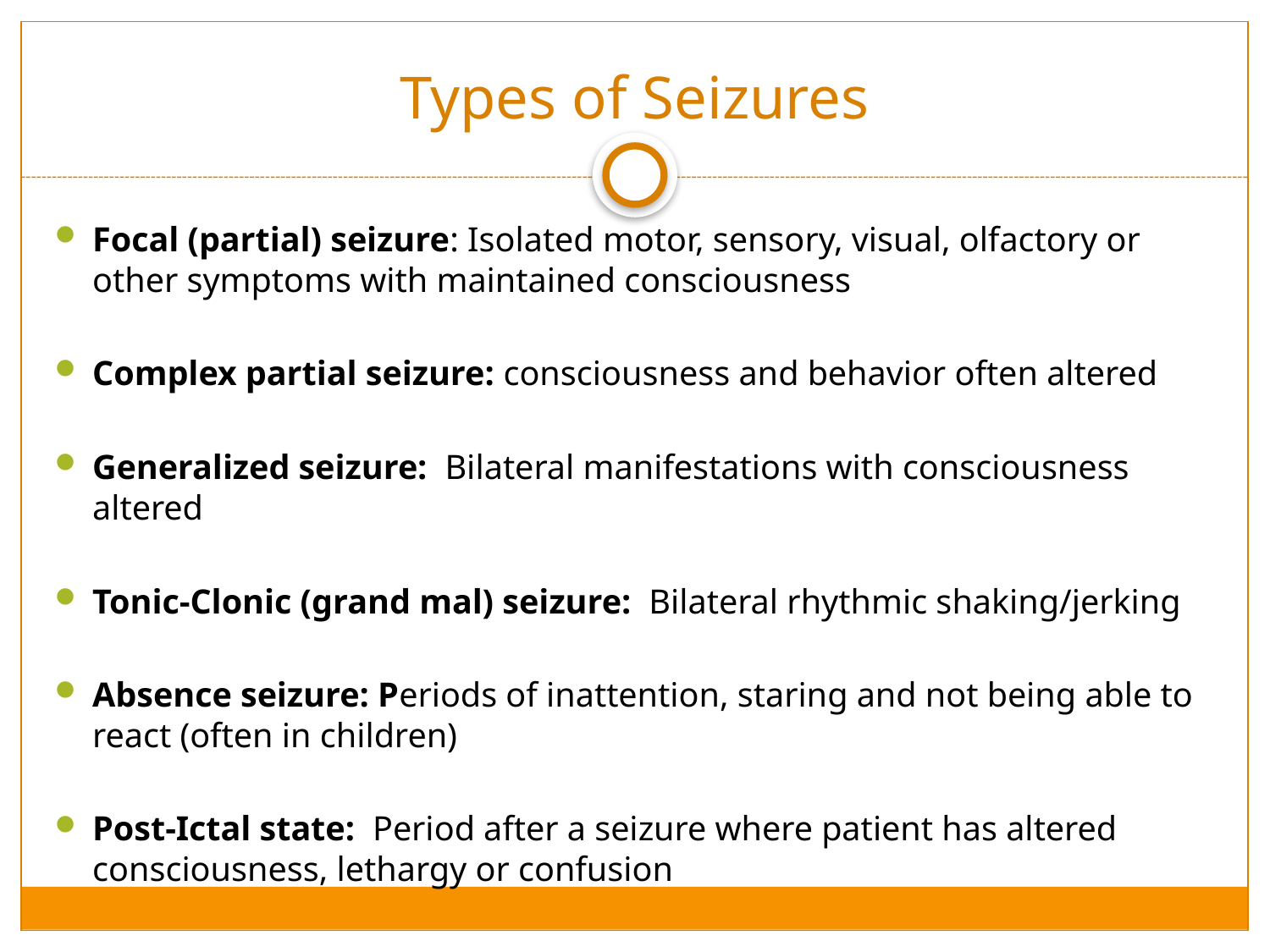

# Types of Seizures
Focal (partial) seizure: Isolated motor, sensory, visual, olfactory or other symptoms with maintained consciousness
Complex partial seizure: consciousness and behavior often altered
Generalized seizure: Bilateral manifestations with consciousness altered
Tonic-Clonic (grand mal) seizure: Bilateral rhythmic shaking/jerking
Absence seizure: Periods of inattention, staring and not being able to react (often in children)
Post-Ictal state: Period after a seizure where patient has altered consciousness, lethargy or confusion

## Slide 10
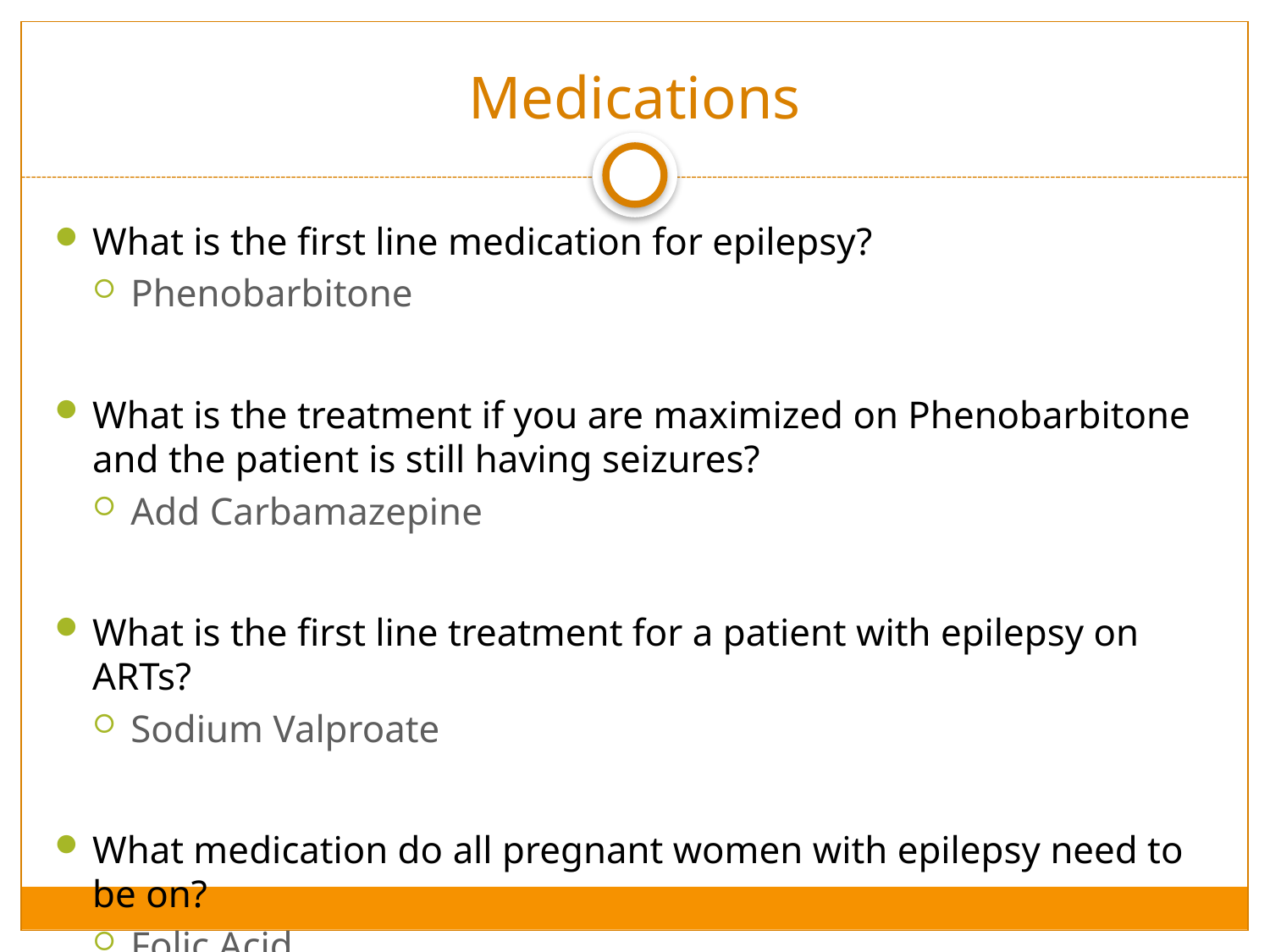

# Medications
What is the first line medication for epilepsy?
Phenobarbitone
What is the treatment if you are maximized on Phenobarbitone and the patient is still having seizures?
Add Carbamazepine
What is the first line treatment for a patient with epilepsy on ARTs?
Sodium Valproate
What medication do all pregnant women with epilepsy need to be on?
Folic Acid

## Slide 11
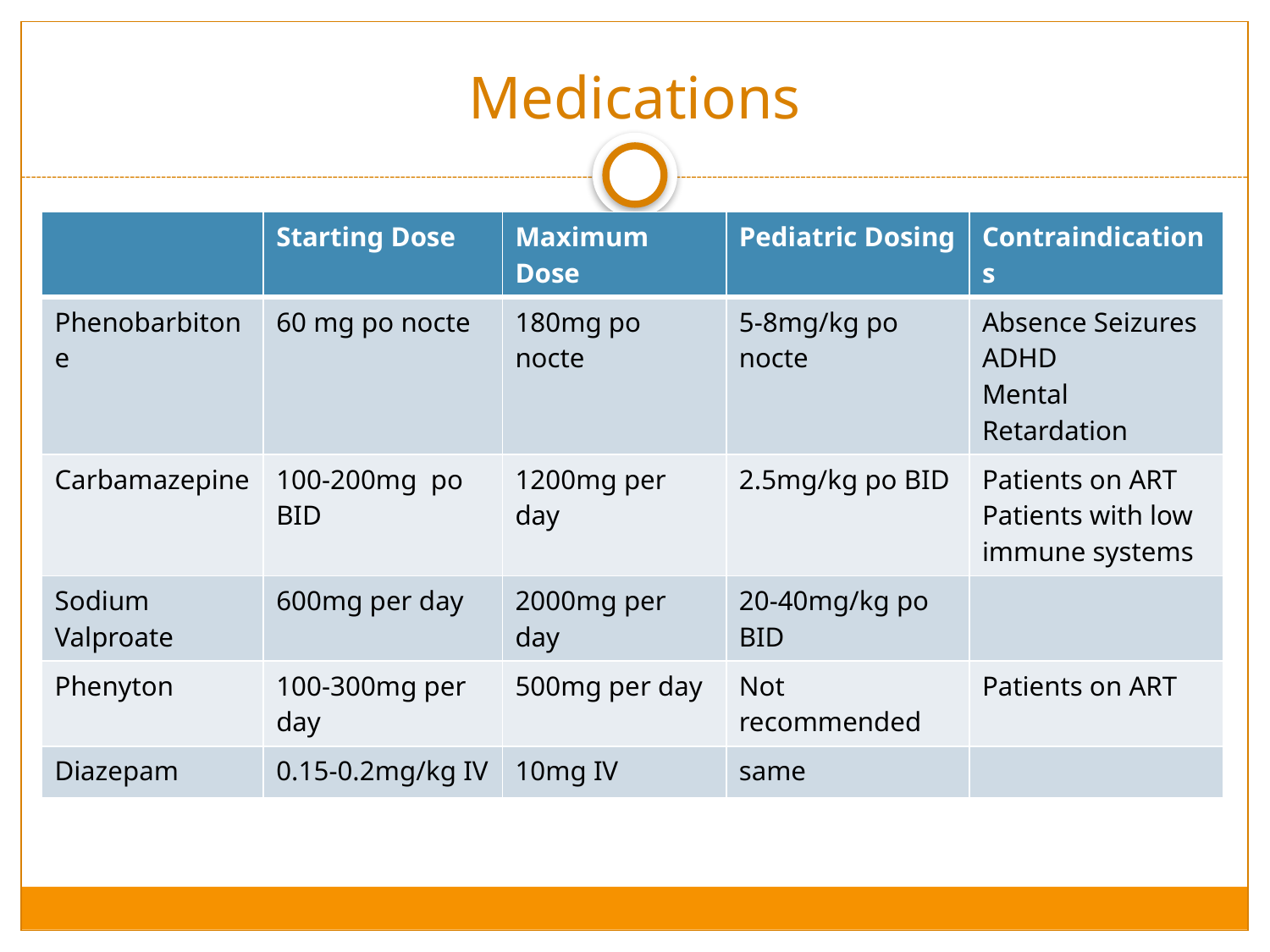

# Medications
| | Starting Dose | Maximum Dose | Pediatric Dosing | Contraindications |
| --- | --- | --- | --- | --- |
| Phenobarbitone | 60 mg po nocte | 180mg po nocte | 5-8mg/kg po nocte | Absence Seizures ADHD Mental Retardation |
| Carbamazepine | 100-200mg po BID | 1200mg per day | 2.5mg/kg po BID | Patients on ART Patients with low immune systems |
| Sodium Valproate | 600mg per day | 2000mg per day | 20-40mg/kg po BID | |
| Phenyton | 100-300mg per day | 500mg per day | Not recommended | Patients on ART |
| Diazepam | 0.15-0.2mg/kg IV | 10mg IV | same | |

## Slide 12
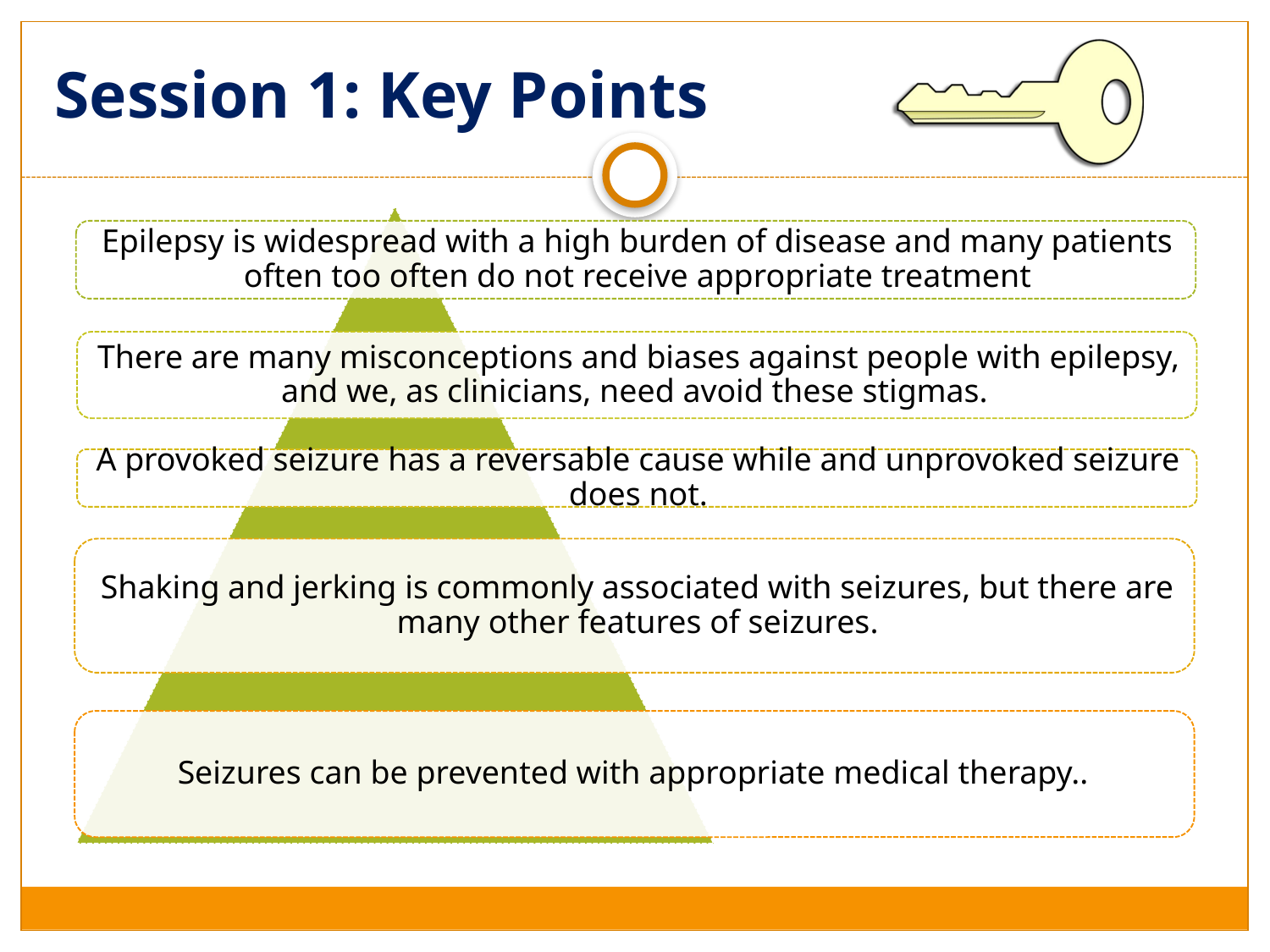

# Session 1: Key Points
