# Supplementary material for: Training Mid-Level Providers to Treat Severe Non-Communicable Diseases in Neno, Malawi through PEN-Plus Strategies
Source: Ann Glob Health. 2022 Aug 11;88(1):69. doi: 10.5334/aogh.3750 (PMC9389951; doi:10.5334/aogh.3750)
Supplement: Didactic Materials. — The supplementary materials contain a suggested didactic training schedule and the PowerPoint presentations used for PEN-Plus training in Neno, Malawi. These materials have been reviewed and accepted by the Malawi Ministry of Health for future PEN-Plus trainings in Malawi. [file agh-88-1-3750-s2.zip › Didactic_Materials/NCD Pre-post test.docx]

**NCD Follow up Exam**

1. What is the effect of insulin on the blood glucose level?
   1. Causes a decrease in the blood glucose level.
   2. Causes an increase in the blood glucose level.
   3. Does not affect the blood glucose level.
2. A 25-year-old man presents to the OPD with weight loss and getting up at night often to urinate. You suspect diabetes. What test/s can you use to diagnose diabetes?
   1. Urinalysis
   2. Random blood glucose
   3. Hemoglobin A1c test
   4. Sputum sample
   5. Both B and C
3. A 45-year-old was referred from SHARC the previous week. On routine screening by SHARC team her BMI was 30 kg/m2 and blood pressure is 150/90. She denies any symptoms of diabetes. Her finger stick blood sugar (random) was 210 mg/dl (11.7 mmol/L). Does this patient have diabetes?
   1. Yes
   2. No
   3. Maybe, but only if we repeat her random glucose and it’s ≥ 200 mg/dl.
   4. Maybe, but it depends on her weight
4. A 70-year-old woman with long-standing diabetes comes to the integrated chronic care clinic. She takes metformin 1000 mg twice daily, and enalapril 20 mg daily. She has no current complaints. She gets an I-STAT and you see that her Creatinine is 2.8. What changes, if any do you make to her medications?
   1. None, her metformin protect her kidneys
   2. Stop the enalapril
   3. Stop the metformin
   4. Stop both the metformin and enalapril
5. A 30-year-old girl with Type I Diabetes presents to the OPD. She has been on insulin therapy since her diagnosis at age 25. She is recovering from a diarrheal illness and has not been eating much. She awoke this morning feeling very anxious, weak, and dizzy. She has a heart rate of 110 bpm, blood pressure 110/70, oxygen saturation 99%. Malaria test is negative. What is your next step?
   1. Give her diazepam for panic attack
   2. Check I-STAT for kidney failure
   3. Check random blood glucose for hypoglycemia
   4. Give amoxycillin for suspected pneumonia
6. A 54-year-old woman with diabetes presents to OPD with diarrhea and is confused and has difficulty staying awake. Blood pressure is 110/78, heart rate is 100, oxygen saturation is 99%. Blood glucose is taken and returns at 560 mg/dl. You prepare for regular glucose checks and insulin administration. What other parts of treatment of HONK are critical here?
   1. Normal saline boluses and potassium repletion
   2. Lisinopril and metformin
   3. Aspirin and amlodipine
   4. Diazepam
7. A 78-year-old man presents to the chronic care clinic. He has been receiving treatment for her diabetes and hypertension. He takes metformin 1000 g twice daily, enalapril 20mg daily, and amlodipine 5mg daily. He smokes cigarettes daily. You calculate that he has a 30-40% cardiovascular risk. After you advise him on quitting smoking, what is your next step?
   1. Start aspirin and simvastatin
   2. Start furosemide
   3. Increase metformin to 2g twice daily
   4. No medication changes
8. A 45-year-old woman with type 2 diabetes presents to the chronic care clinic for regular follow-up. She was last seen 6 months ago and has had good control of her sugar levels on metformin 500mg twice daily. She has a heart rate of 80, blood pressure of 160/94. Random glucose measurement is 130. What medication would you like to start?
   1. Increase metformin to 1g twice daily
   2. Amlodipine 5mg daily
   3. Enalapril 10mg daily
   4. Propranolol 40mg twic daily
9. A 56-year-old man has recently been diagnosed with type 2 diabetes. Today, his heart rate is 70, his blood pressure is 120/80, and his BMI is 32. What things can you discuss with him that will improve his diabetes?
   1. Increasing the amount of daily exercise
   2. Advise on eating more vegetables
   3. Tell him there is nothing he can do for his diabetes
   4. Tell him to take his medications regularly
   5. Choices A, B, and D
10. Which statement is true
    1. If symptomatic, a single FBS **≥ 126 mg/dL** is diagnostic of diabetes
    2. If asymptomaitic, a single FBS **≥ 126 mg/dL** is diagnostic of diabetes
    3. If asymptomatic a single RBS of **≥ 200 mg/dL** is diagnostic of diabetes
    4. If symptomatic a single RBS of **≥ 126 mg/dL** is diagnostic of diabetes
11. One of the following values for the ejection fraction (EF) does NOT typically represent systolic heart failure
    1. 60%
    2. 35%
    3. 15%
    4. 5%
    5. None of the Above
12. Which of the following is a cause of a heart failure exacerbation?
    1. Non-adherence to medications
    2. Change in diet
    3. Acute Illness
    4. Anemia
    5. All of the Above
13. A 32-year old woman comes to the clinic. She was doing well until 2 months ago, when she started experiencing some difficulty breathing while going up the hill. She can perform her daily activities. She was pregnant and gave birth to her son 3 months ago. You diagnose her with postpartum cardiomyopathy. Her NYHA classification is
    1. Class I/II
    2. Class III
    3. Class IV
    4. Class V
14. The possible symptoms of hypertension are (mark only ONE answer)
    1. Headache
    2. Blurred vision
    3. Hematruia
    4. Dyspnea
    5. No symptoms
    6. All of the above
15. The complication of hypertension are (mark only ONE answer)
    1. Stroke
    2. Heart attack
    3. Heat failure
    4. Kidney disase
    5. All of the above
16. Patients with hypertension and which of the following conditions should be started on an ACE inhibitor (if there are no contraindications for ACE inhibitor)
    1. Diabetes
    2. Proteniuria
    3. Pregnancy
    4. Heart Failure
    5. A, b, & c
    6. A, B & d
    7. A, C & d
17. Which of the following findings is NOT associated with a stroke
    1. Facial droop
    2. Slurred Speech
    3. Hyperreflexia
    4. Arm drift
18. Which of the following is TRUE
    1. HIV does not increase your risk of cardiovascular disease
    2. Starting ARTs soon can decrease your risk of cardiovascular disease
    3. HAART does NOT have cardiac side effects
    4. HIV reduces coronary plaque development
19. Which of the following medications is first line in Pre-Eclampsia
    1. Methyldopa
    2. HCTZ
    3. Nifedipine
    4. Enalopril
20. In addition to an ACE inhibitor, which medication is a FIRST line treatment for Congestive Heart Failure?
    1. Atenolol
    2. HCTZ
    3. Spironolactone
    4. Digoxin
21. A 15 year old patient is being seen at the IC3 clinic. He was diagnosed with asthma and started on treatment. He states that he wakes up coughing 3 nights per week and has to use his salbutamol inhaler once per day. He has difficulty playing football, but can still to go school. Today he is having a lot of difficulty breathing. What should be checked in this patient?
    1. Respiratory rate
    2. Oxygen saturation
    3. Pulmonary auscultation
    4. Evaluate his response to salbutamol
    5. All of the above
22. Which of the following asthma medications can cause tachycardia and palpitations?
    1. Salbutamol
    2. Beclomethasone
    3. Aminophylline
    4. Prednisolone
23. Which of the following medications can cause hyperglycemia?
    1. Salbutamol
    2. Beclomethasone
    3. Aminophylline
    4. Prednisolone
24. A 6 year old boy with a past medical history of multiple admissions to the hospital for “difficulty breathing” presents for follow up in the clinic. On exam you note that he has difficulty speaking in full sentences, severe intercostal retractions and diffuse wheezing on auscultation. Vital signs are: RR 35, HR 112, SaO2 84% on room air. He had difficulty breathing for the last 3 days, not responding to home salbutamol use (1 puff 3x/day). Weight is 22 kg. What is your immediate treatment plan?
    1. Oxygen alone
    2. Oxygen and oral salbutamol
    3. Oxygen and inhaled salbutamol
    4. Oxygen, inhaled salbutamol and prednisone
25. Which of the following are risk factors for developing COPD?
    1. Smoking
    2. Hypertension
    3. Diabetes
    4. Cooking indoors with charcoal
    5. A&D only
    6. B&C only
    7. All of the above
26. Which of the following are common causes of renal failure?
    1. HIV
    2. Hypertension
    3. Diabetes
    4. All of the above
    5. None of the above
27. A GFR of 20 corresponds to what degree of CKD?
    1. CKD stage 2
    2. CKD stage 3
    3. CKD stage 4
    4. CKD stage 5
28. Which of the following statements is true of ACE inhibitors in chronic kidney disease
    1. ACE inhibitors should be avoided in all stages of CKD
    2. ACE inhibitors are safe in all stages of CKD
    3. ACE inhibitors should be stopped as CKD progresses to CKD 3-4
    4. ACE inhibitors have no effect on renal function
29. A sodium of 118 is consistent with which of the following diagnoses?
    1. Hypernatremia
    2. Hyponatremia
    3. Hyperkalemia
    4. Hypokalemia
30. Which of the following electrolyte abnormalities is concerning for arrythmia?
    1. Hyponatremia
    2. Hyperkalemia
    3. Hyperchloremia
    4. Hypocarbia
31. What type of seizure is characterized by staring off into space, inattention and lack of reaction?
    1. Grand Mal Seizure
    2. Tonic Clonic Sizure
    3. Absence Seizure
    4. Focal (partial) Seizure
32. What is the first line medication for a patient with Epilepsy on ARTs?
    1. Phenobarbatone
    2. Carbamazepime
    3. Sodium Valproate
    4. Phenytoin
33. What medication should pregnant women with epilepsy be on?
    1. Aspirin
    2. HCTZ
    3. Vitamin C
    4. Folic Acid
34. Which of the following is true about sickle cell disease
    1. Sickle cell disease is rare in children
    2. Complications of sickle cell disease include anemia, infection and vaco-occlusive phenomena
    3. Sickle cell disease is commonly found in Western Europe and is rare in Africa
    4. Sickle cell disease is acquired from mosquito bites
35. In pain crises from sickle cell disease…
    1. Morphine is the best medication for pain control
    2. Patients often present with Dactylitis, which is painful swelling of a joint
    3. There is high morbidity and mortality in pain crises from sickle cell disease
    4. All of the above
36. Which medication is NOT recommended in a patient with sickle cell disease?
    1. Sulfadoxine-pyrimethamine
    2. Folic Acid
    3. Benzathine Penicillin
    4. Ferrous Sulfate
37. Which type of viral hepatitis can be obtained from a contaminated water source?
    1. Hepatitis A
    2. Hepatitis B
    3. Hepatitis C
    4. Hepatitis D
38. Which of the following are signs and symptoms of hepatitis?
    1. Jaundice
    2. Abdominal Pain
    3. Dark Urine
    4. Confusion
    5. All of the above
39. Which medication is given to patients for chronic hepatic encephalopathy?
    1. Ciprofloxacin
    2. Lactulose
    3. Spironolactone
    4. Omeprazole
40. What is the term for fluid accumulation around the liver?
    1. Hepatitis
    2. Cirrhosis
    3. Ascites
    4. Crohns Disease
