# Supplementary material for: Training Mid-Level Providers to Treat Severe Non-Communicable Diseases in Neno, Malawi through PEN-Plus Strategies
Source: Ann Glob Health. 2022 Aug 11;88(1):69. doi: 10.5334/aogh.3750 (PMC9389951; doi:10.5334/aogh.3750)
Supplement: Didactic Materials. — The supplementary materials contain a suggested didactic training schedule and the PowerPoint presentations used for PEN-Plus training in Neno, Malawi. These materials have been reviewed and accepted by the Malawi Ministry of Health for future PEN-Plus trainings in Malawi. [file agh-88-1-3750-s2.zip › Didactic_Materials/P_ Pulmonary.pptx]

## Slide 1
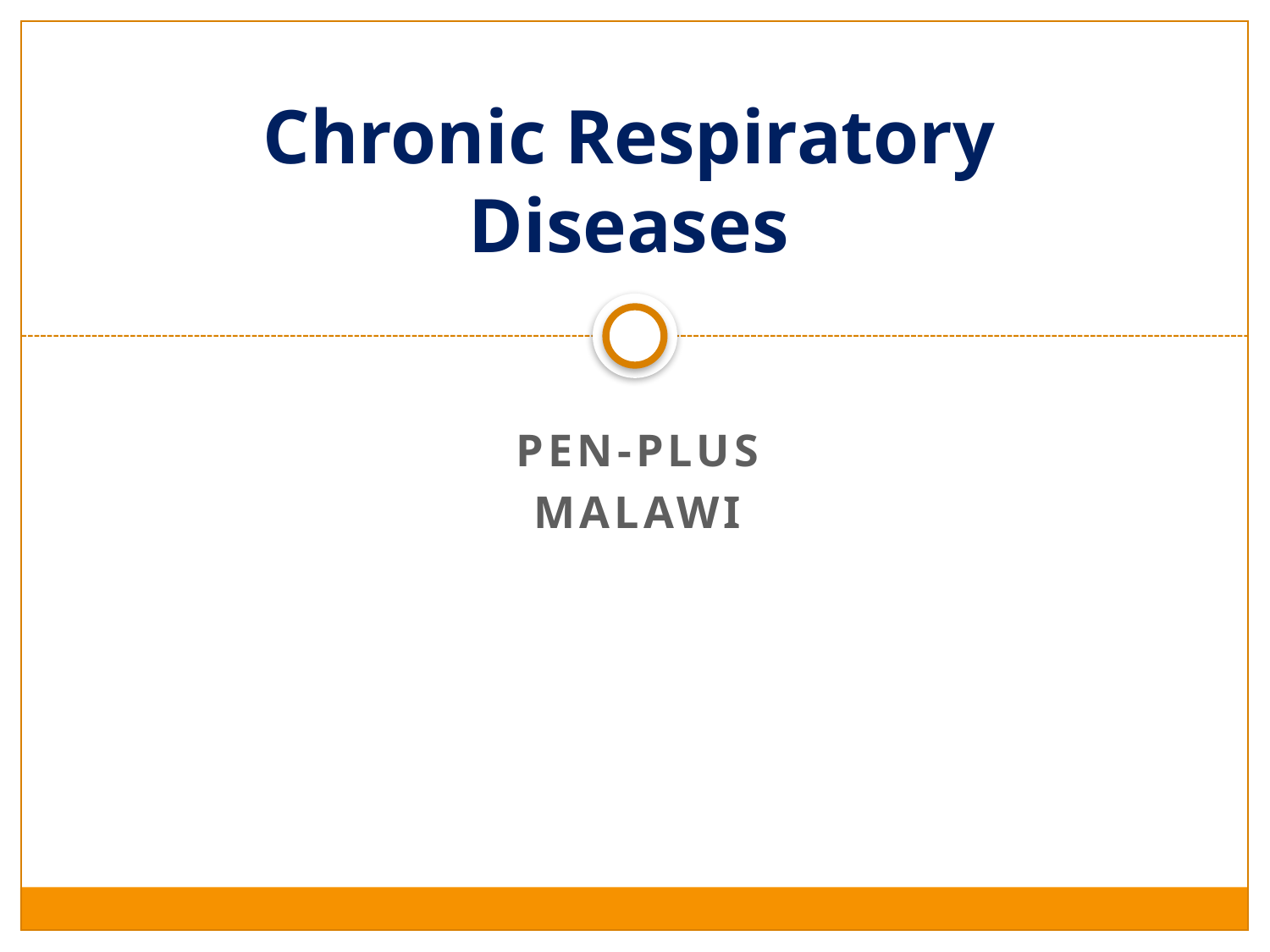

# Chronic Respiratory Diseases
PEN-Plus
Malawi

## Slide 2
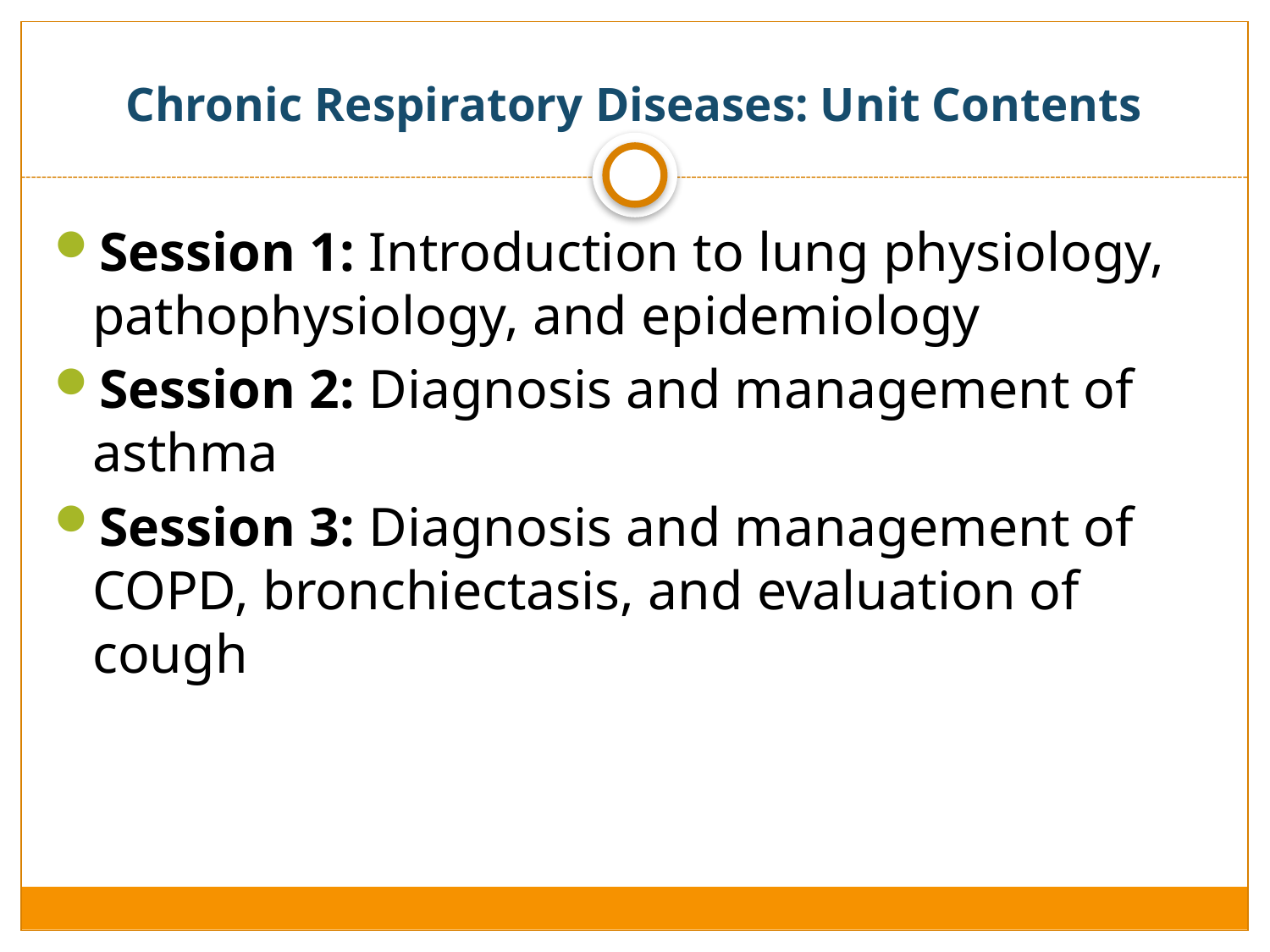

# Chronic Respiratory Diseases: Unit Contents
Session 1: Introduction to lung physiology, pathophysiology, and epidemiology
Session 2: Diagnosis and management of asthma
Session 3: Diagnosis and management of COPD, bronchiectasis, and evaluation of cough

## Slide 3
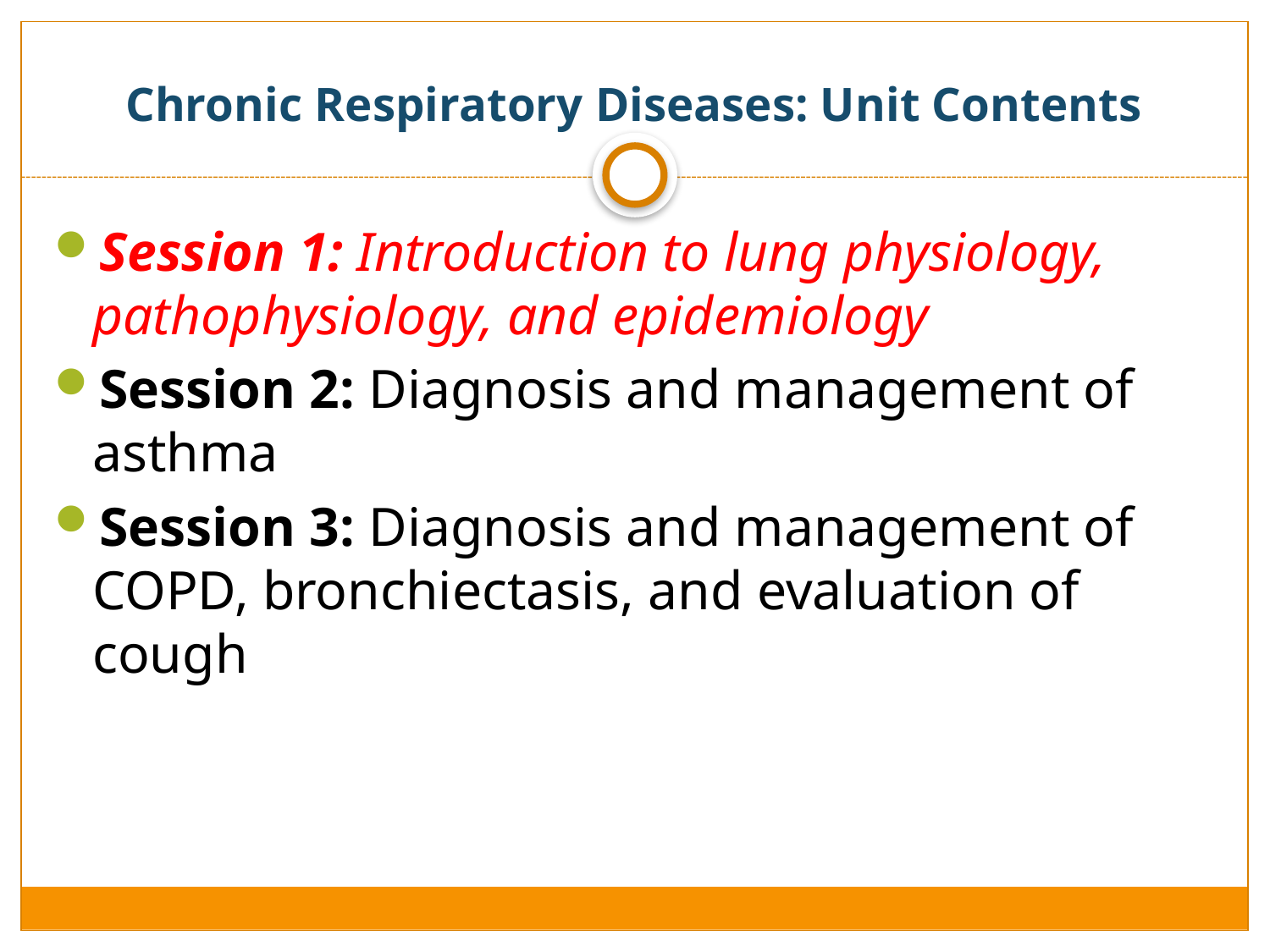

# Chronic Respiratory Diseases: Unit Contents
Session 1: Introduction to lung physiology, pathophysiology, and epidemiology
Session 2: Diagnosis and management of asthma
Session 3: Diagnosis and management of COPD, bronchiectasis, and evaluation of cough

## Slide 4
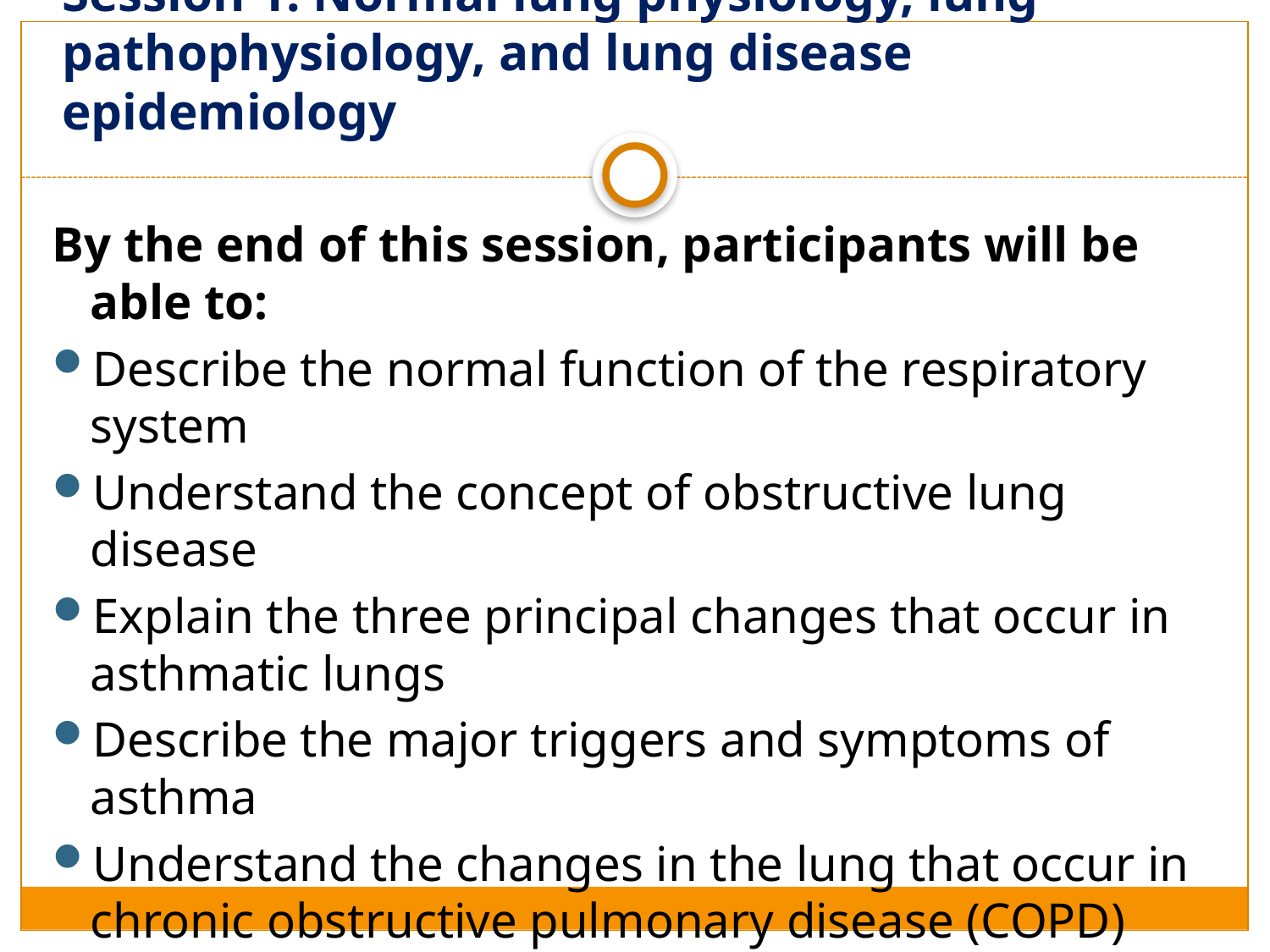

# Session 1: Normal lung physiology, lung pathophysiology, and lung disease epidemiology
By the end of this session, participants will be able to:
Describe the normal function of the respiratory system
Understand the concept of obstructive lung disease
Explain the three principal changes that occur in asthmatic lungs
Describe the major triggers and symptoms of asthma
Understand the changes in the lung that occur in chronic obstructive pulmonary disease (COPD)
Describe the epidemiology of asthma and COPD

## Slide 5
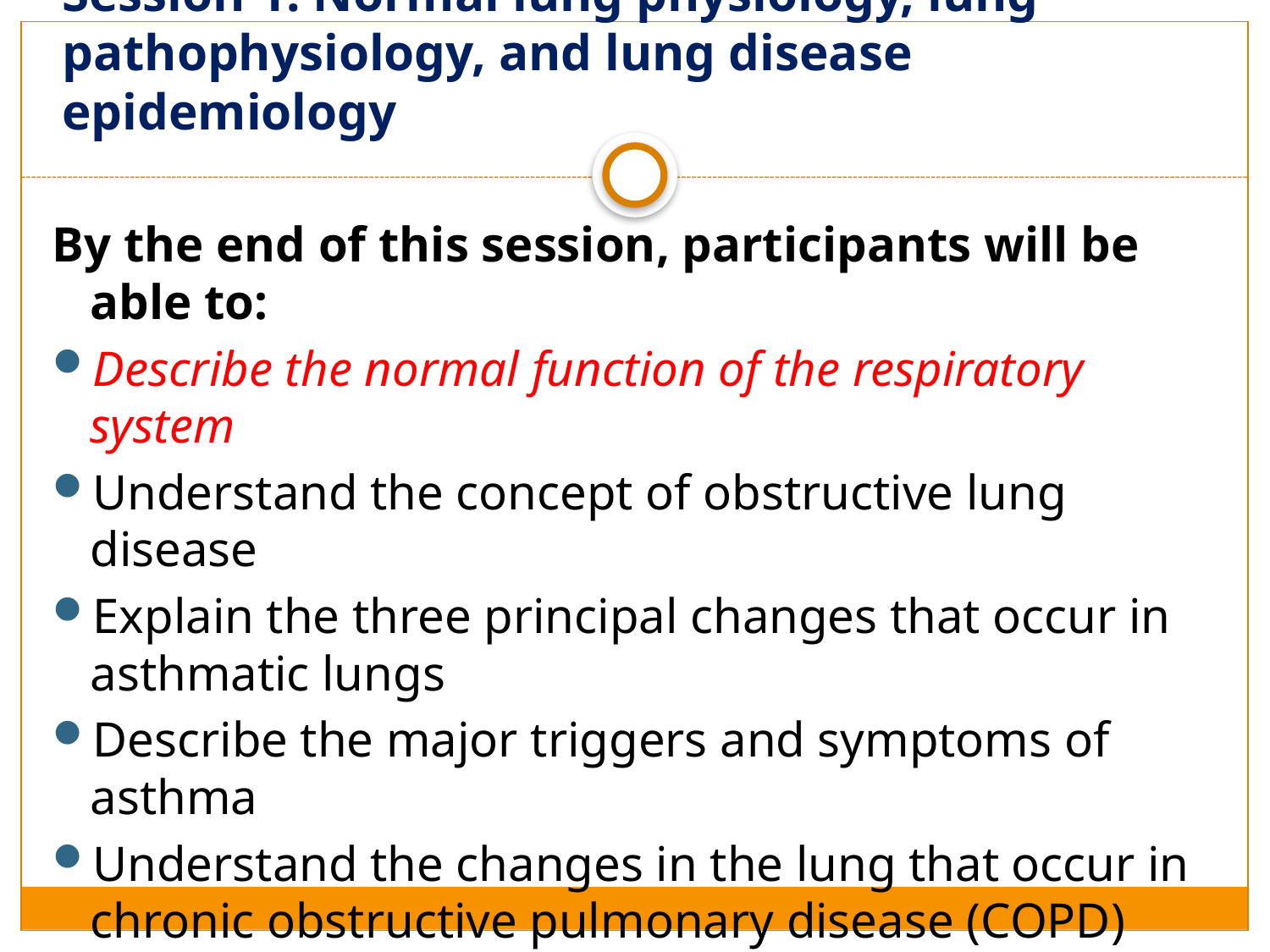

# Session 1: Normal lung physiology, lung pathophysiology, and lung disease epidemiology
By the end of this session, participants will be able to:
Describe the normal function of the respiratory system
Understand the concept of obstructive lung disease
Explain the three principal changes that occur in asthmatic lungs
Describe the major triggers and symptoms of asthma
Understand the changes in the lung that occur in chronic obstructive pulmonary disease (COPD)
Describe the epidemiology of asthma and COPD

## Slide 6
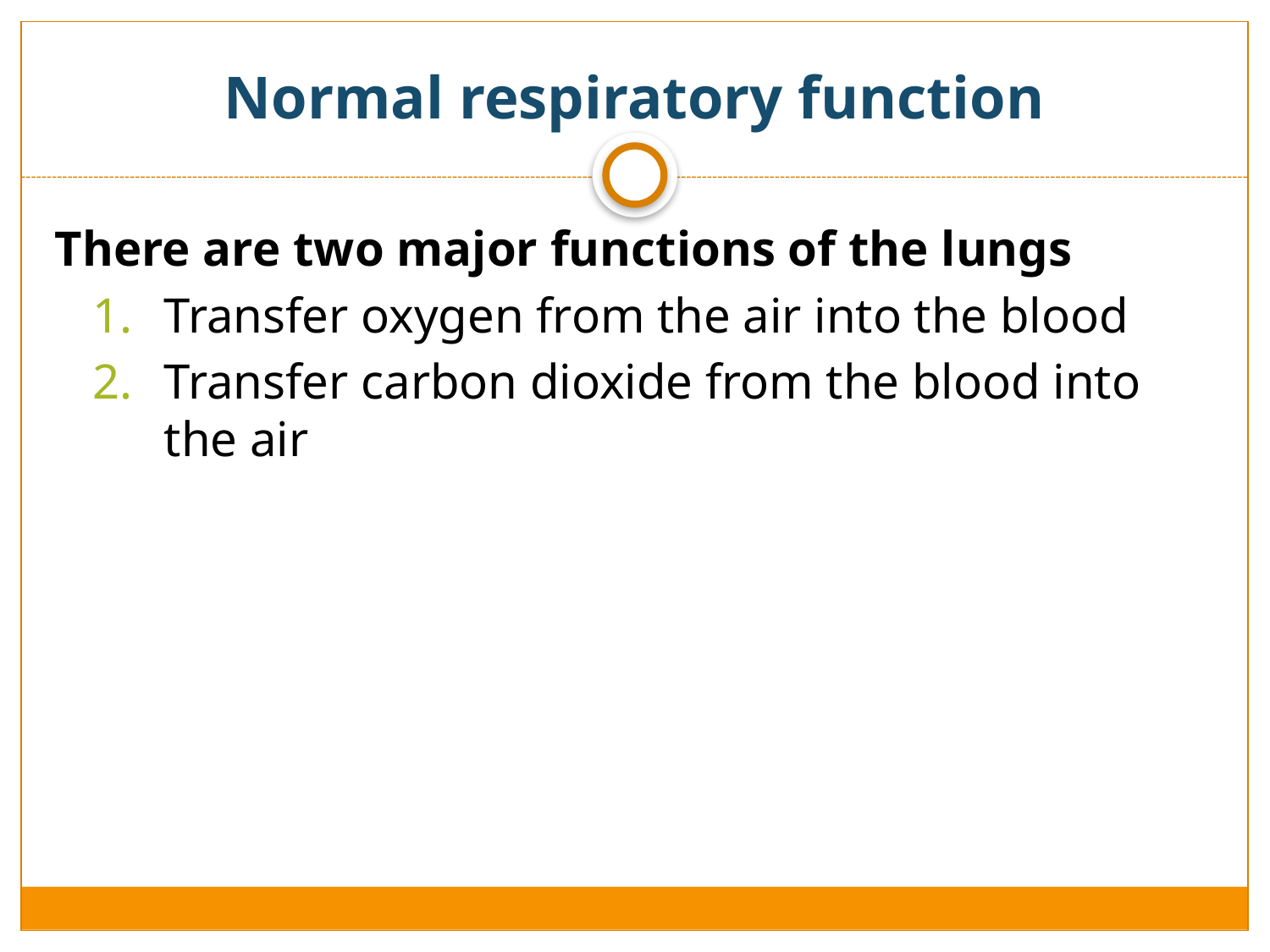

# Normal respiratory function
There are two major functions of the lungs
Transfer oxygen from the air into the blood
Transfer carbon dioxide from the blood into the air

## Slide 7
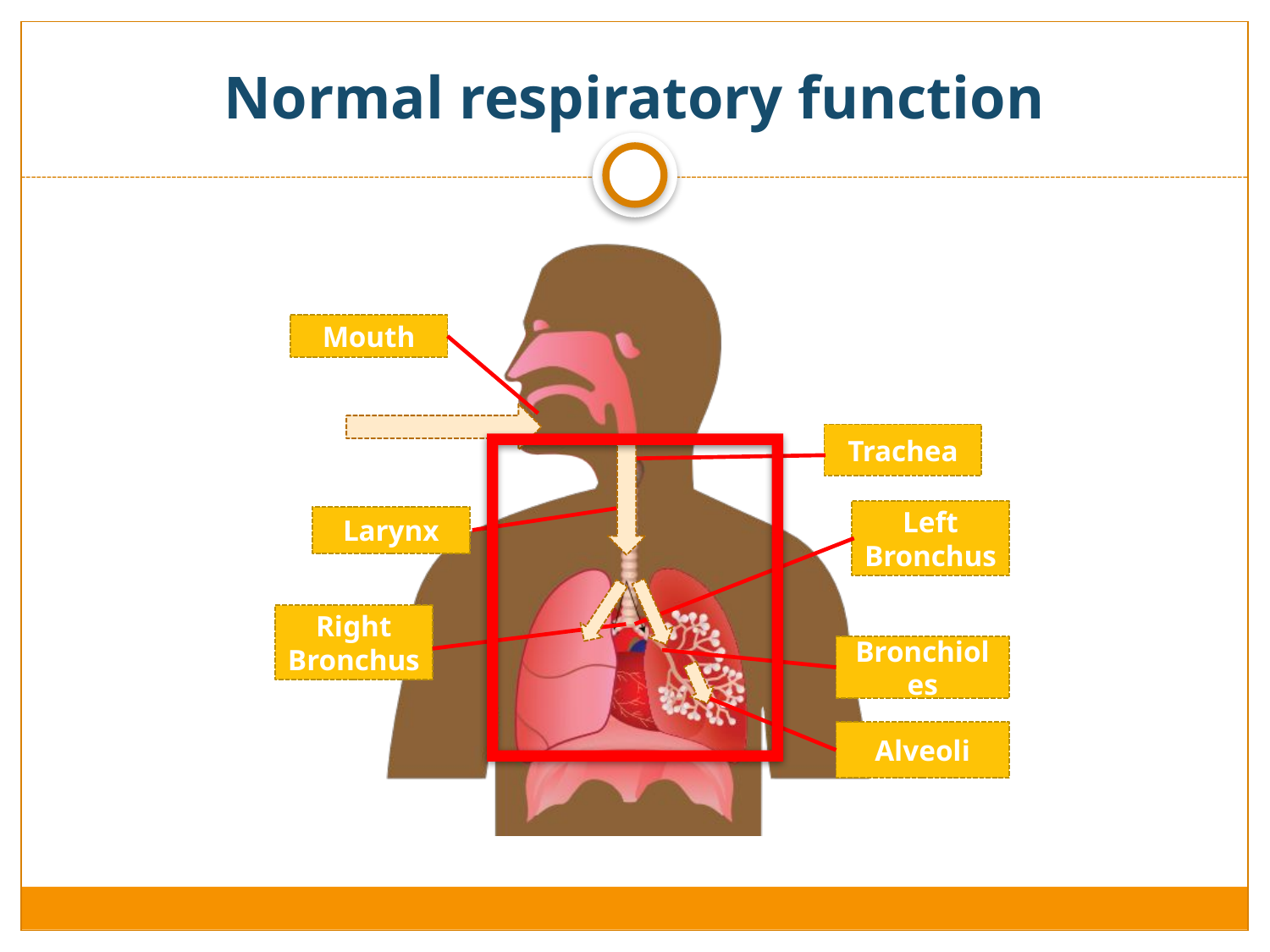

# Normal respiratory function
Mouth
Trachea
Left Bronchus
Larynx
Right Bronchus
Bronchioles
Alveoli

## Slide 8
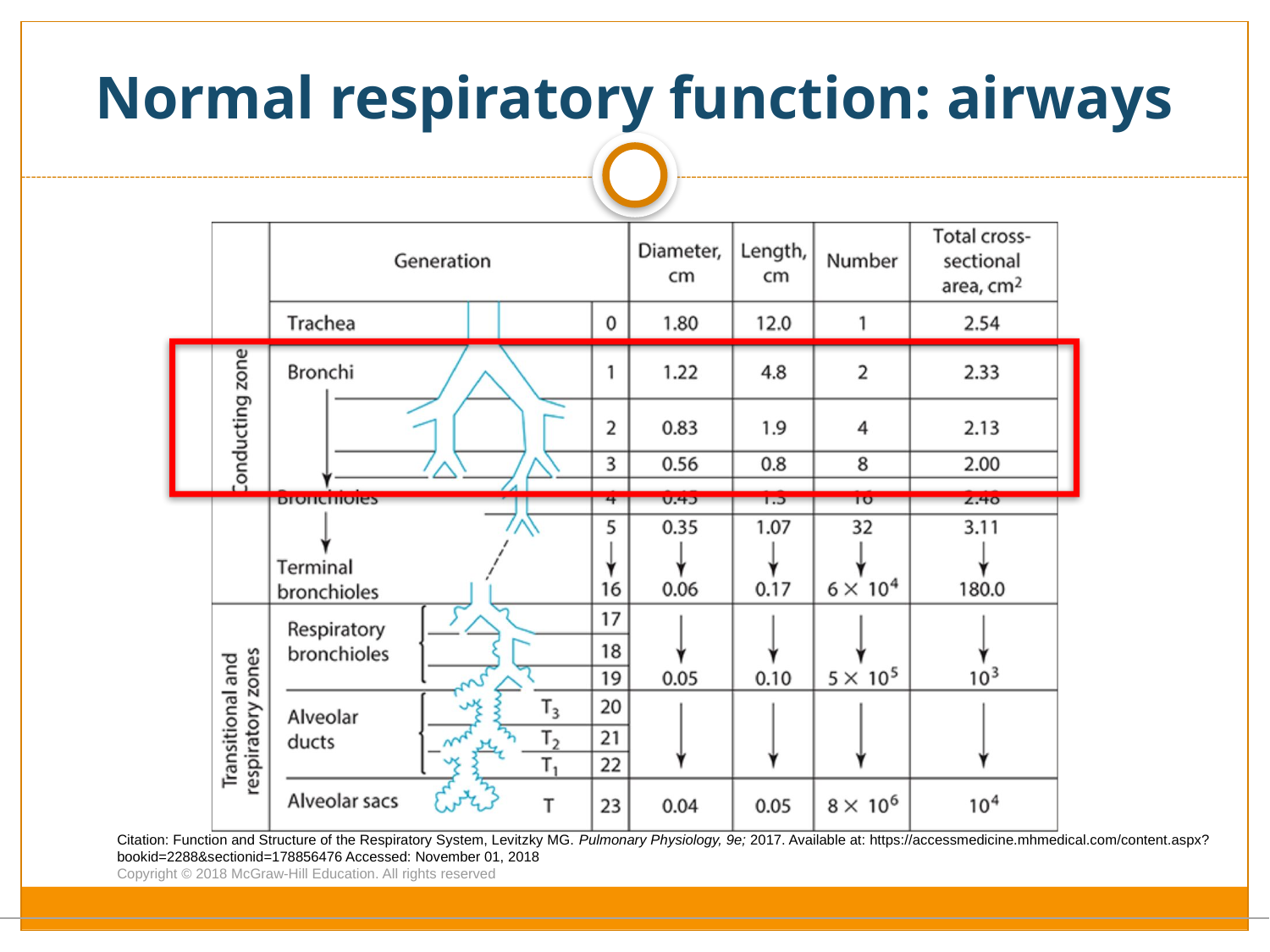

# Normal respiratory function: airways
Citation: Function and Structure of the Respiratory System, Levitzky MG. Pulmonary Physiology, 9e; 2017. Available at: https://accessmedicine.mhmedical.com/content.aspx?bookid=2288&sectionid=178856476 Accessed: November 01, 2018
Copyright © 2018 McGraw-Hill Education. All rights reserved

## Slide 9
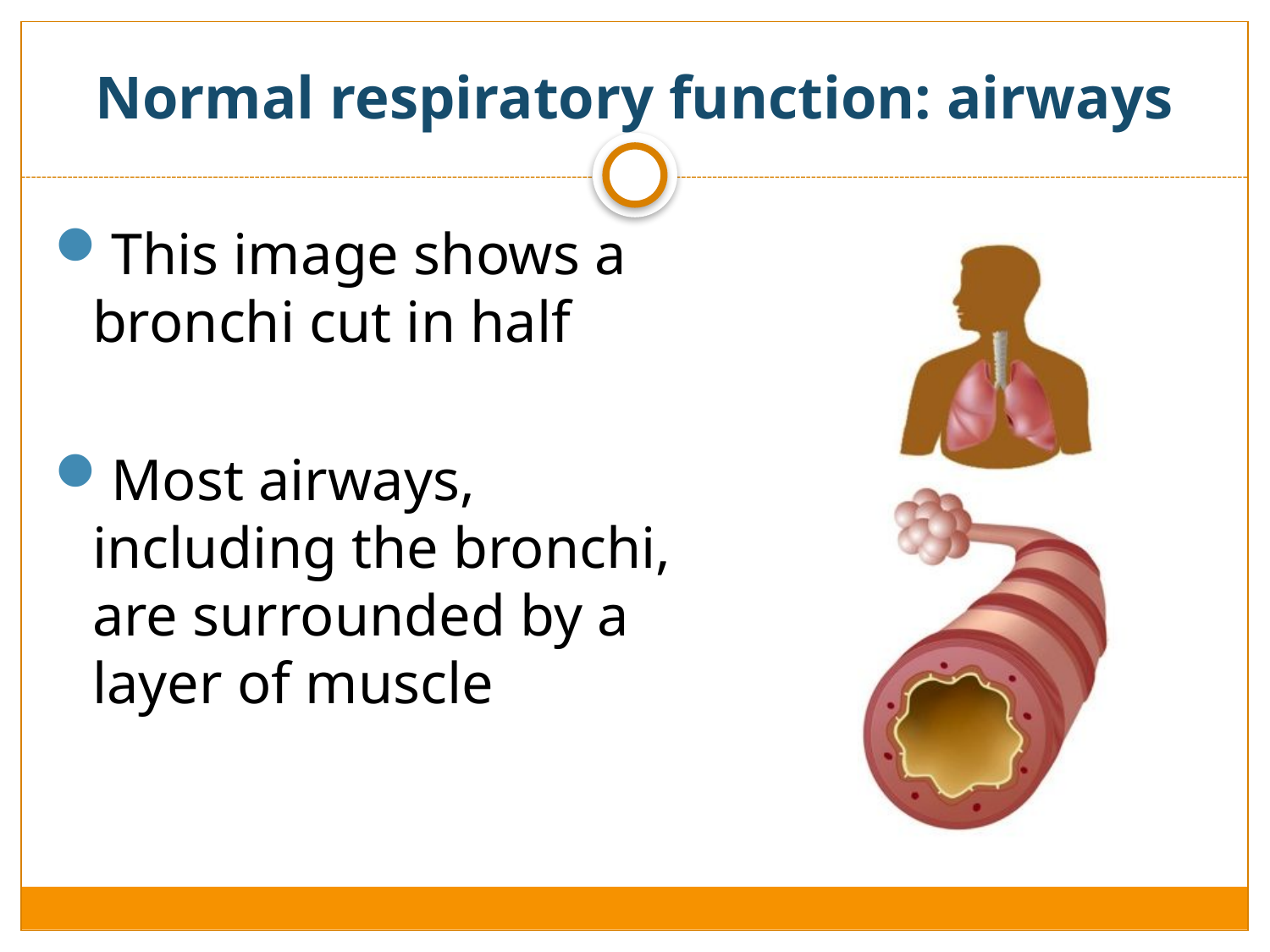

# Normal respiratory function: airways
This image shows a bronchi cut in half
Most airways, including the bronchi, are surrounded by a layer of muscle

## Slide 10
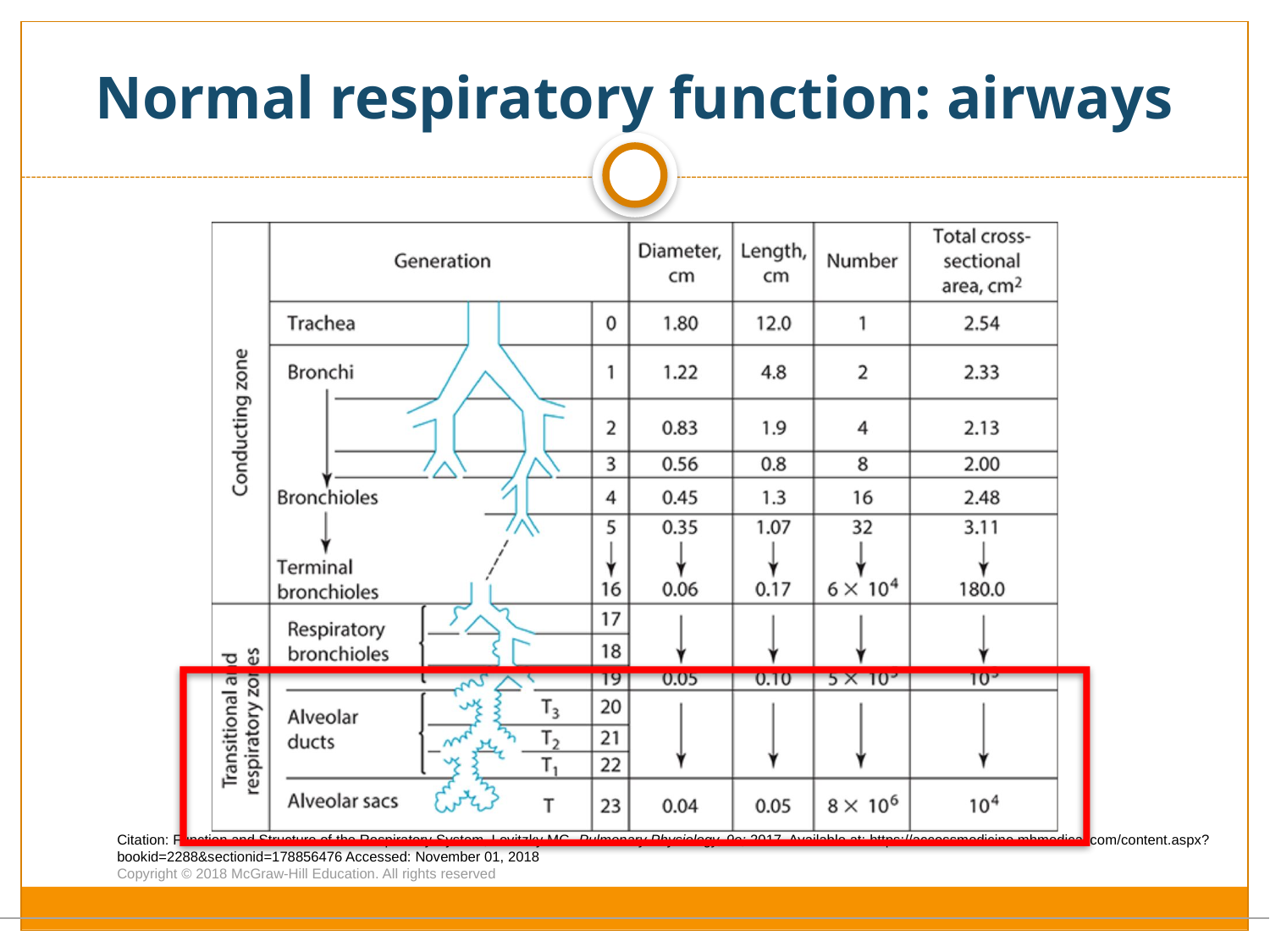

# Normal respiratory function: airways
Citation: Function and Structure of the Respiratory System, Levitzky MG. Pulmonary Physiology, 9e; 2017. Available at: https://accessmedicine.mhmedical.com/content.aspx?bookid=2288&sectionid=178856476 Accessed: November 01, 2018
Copyright © 2018 McGraw-Hill Education. All rights reserved

## Slide 11
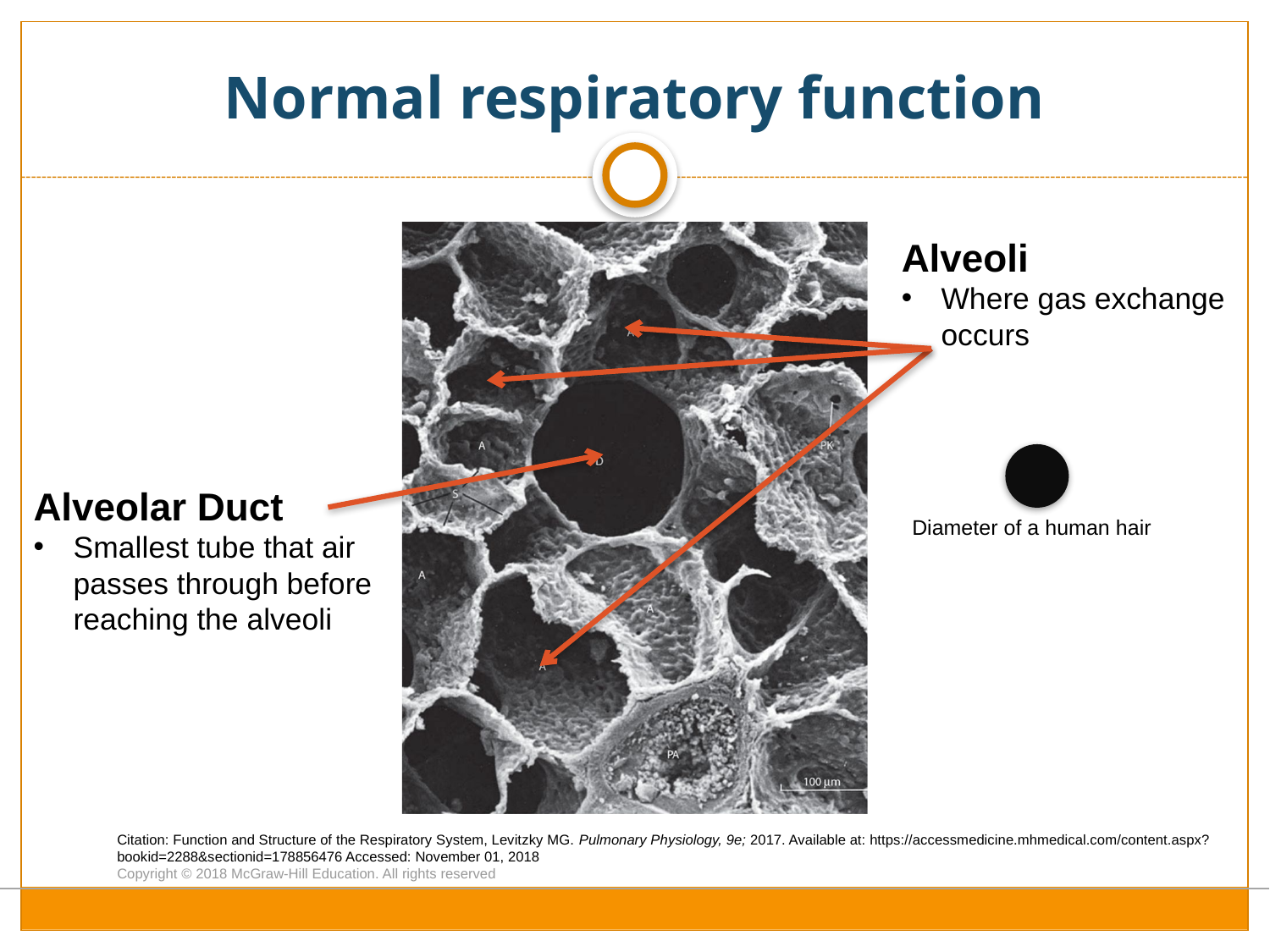

# Normal respiratory function
Alveoli
Where gas exchange occurs
Alveolar Duct
Smallest tube that air passes through before reaching the alveoli
Diameter of a human hair
Citation: Function and Structure of the Respiratory System, Levitzky MG. Pulmonary Physiology, 9e; 2017. Available at: https://accessmedicine.mhmedical.com/content.aspx?bookid=2288&sectionid=178856476 Accessed: November 01, 2018
Copyright © 2018 McGraw-Hill Education. All rights reserved

## Slide 12
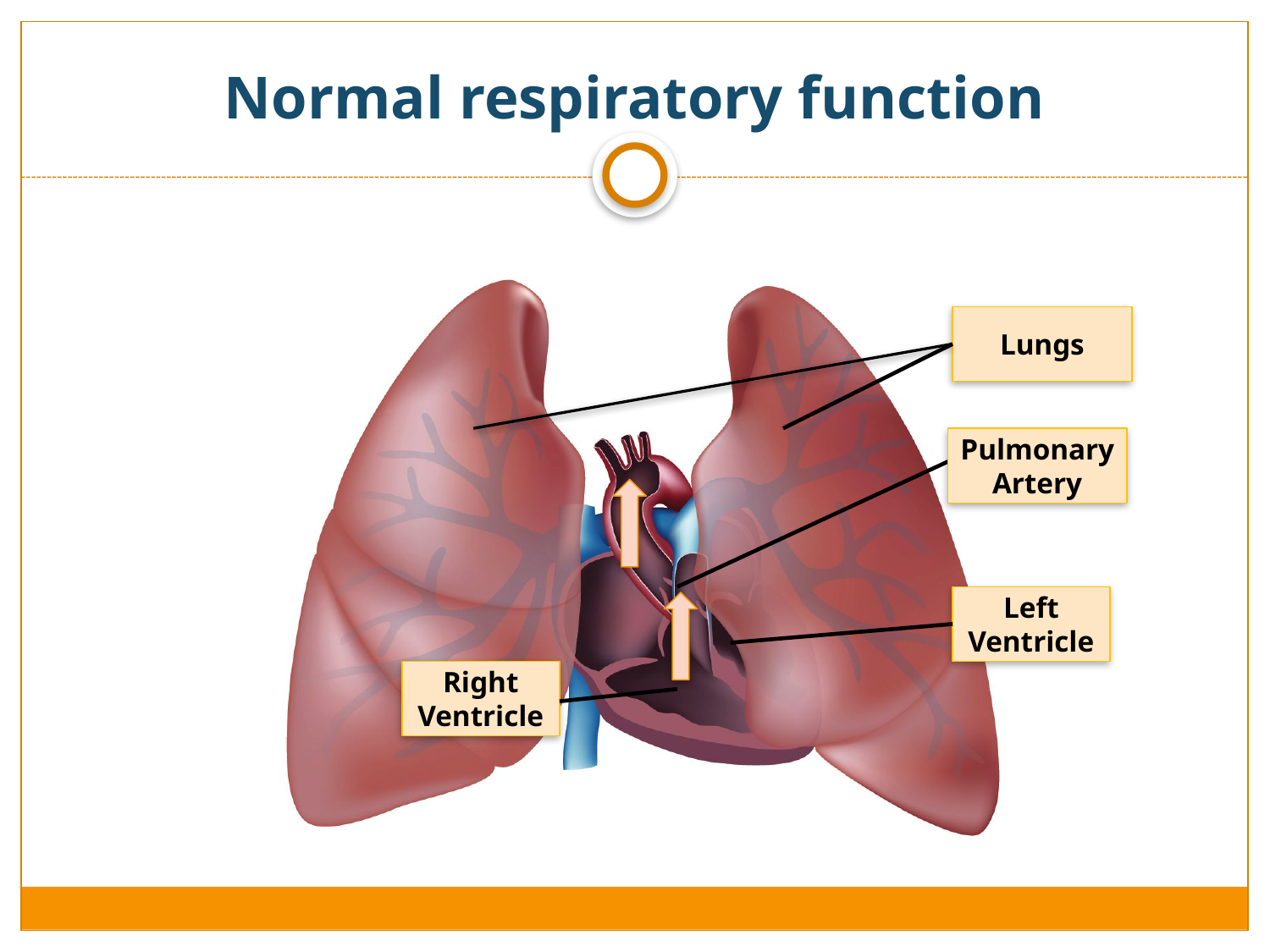

# Normal respiratory function
Lungs
Pulmonary Artery
Left Ventricle
Right Ventricle

## Slide 13
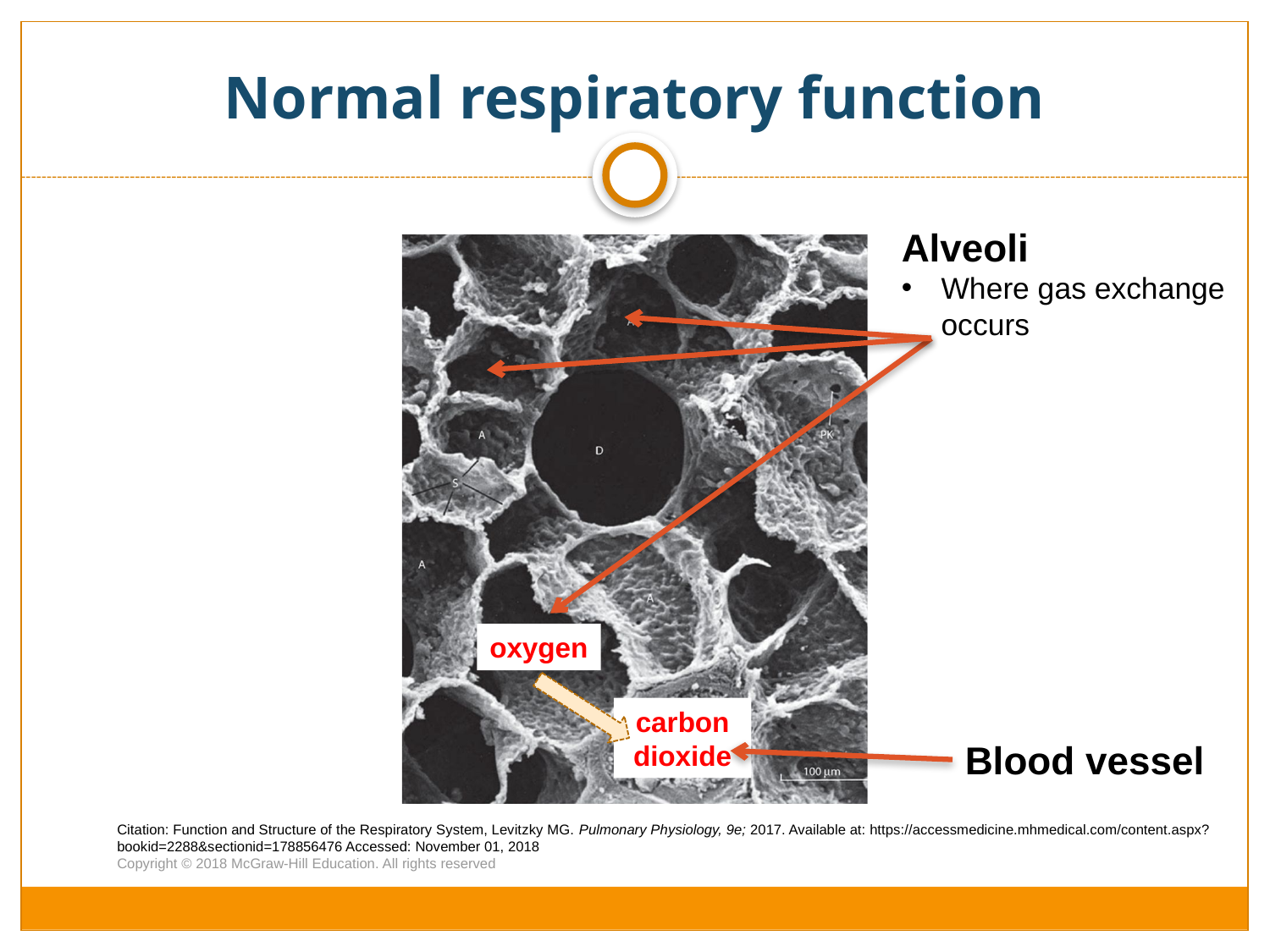

# Normal respiratory function
Alveoli
Where gas exchange occurs
oxygen
carbon dioxide
Blood vessel
Citation: Function and Structure of the Respiratory System, Levitzky MG. Pulmonary Physiology, 9e; 2017. Available at: https://accessmedicine.mhmedical.com/content.aspx?bookid=2288&sectionid=178856476 Accessed: November 01, 2018
Copyright © 2018 McGraw-Hill Education. All rights reserved

## Slide 14
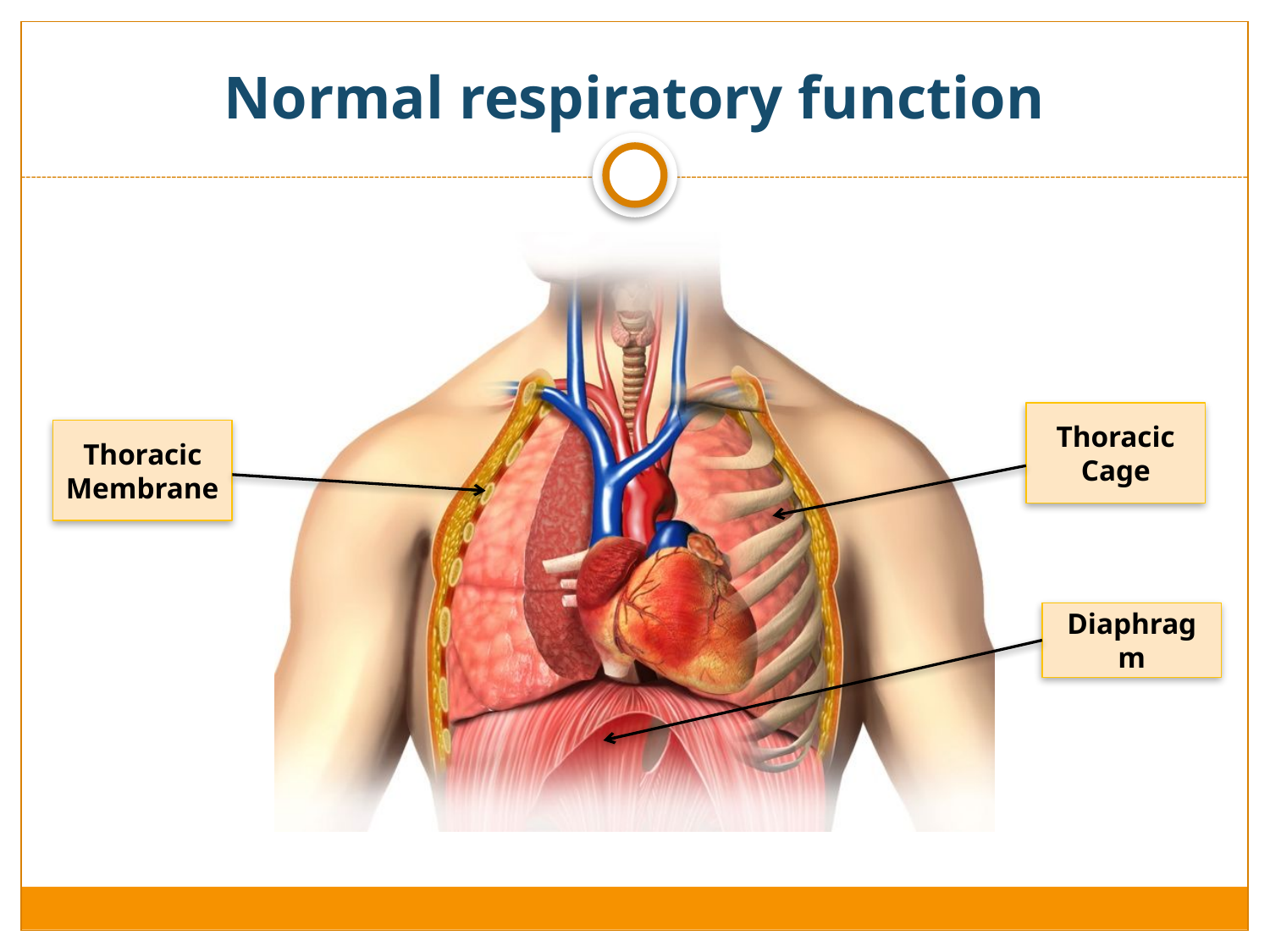

# Normal respiratory function
Thoracic Cage
Thoracic Membrane
Diaphragm

## Slide 15
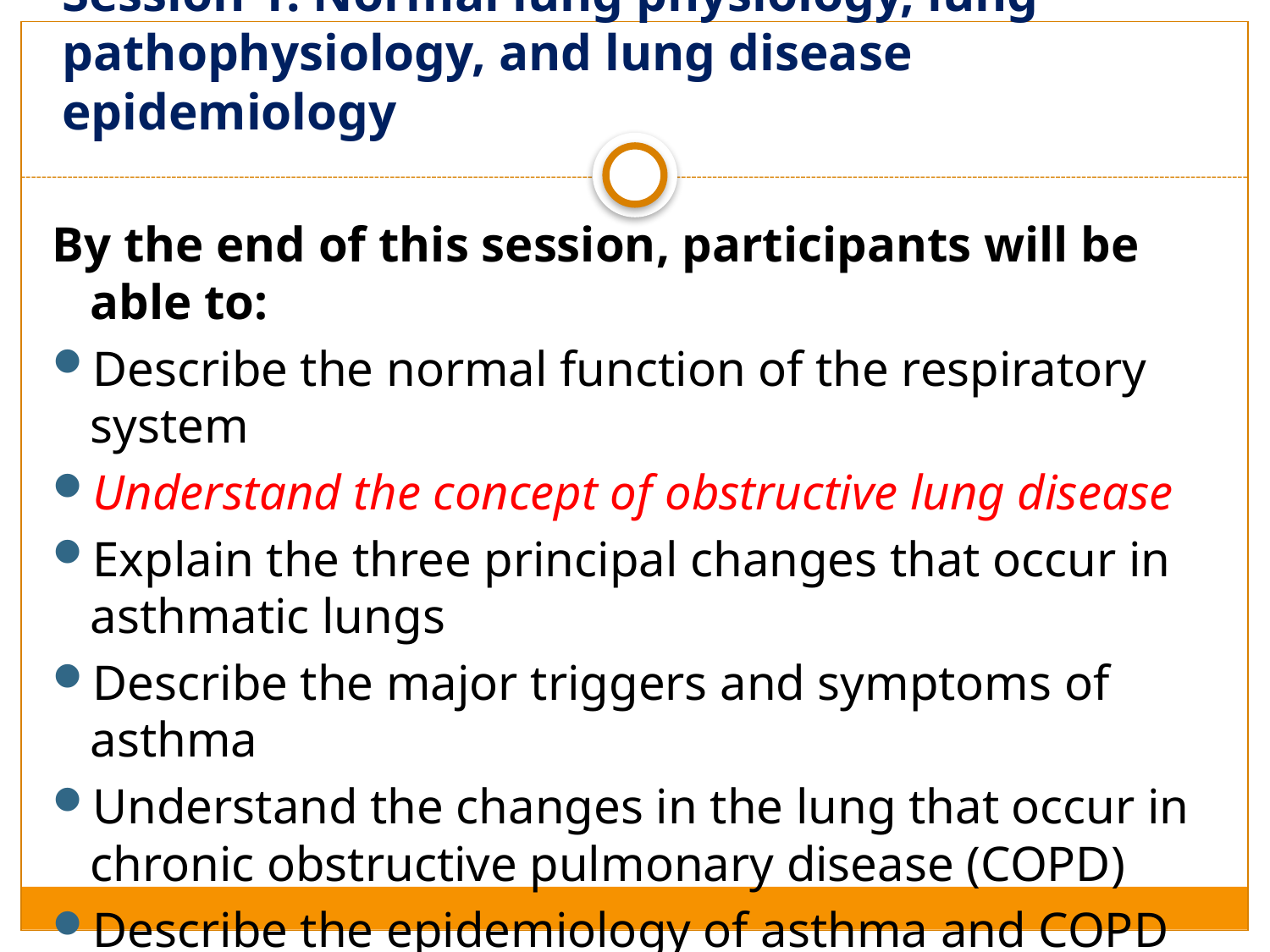

# Session 1: Normal lung physiology, lung pathophysiology, and lung disease epidemiology
By the end of this session, participants will be able to:
Describe the normal function of the respiratory system
Understand the concept of obstructive lung disease
Explain the three principal changes that occur in asthmatic lungs
Describe the major triggers and symptoms of asthma
Understand the changes in the lung that occur in chronic obstructive pulmonary disease (COPD)
Describe the epidemiology of asthma and COPD

## Slide 16
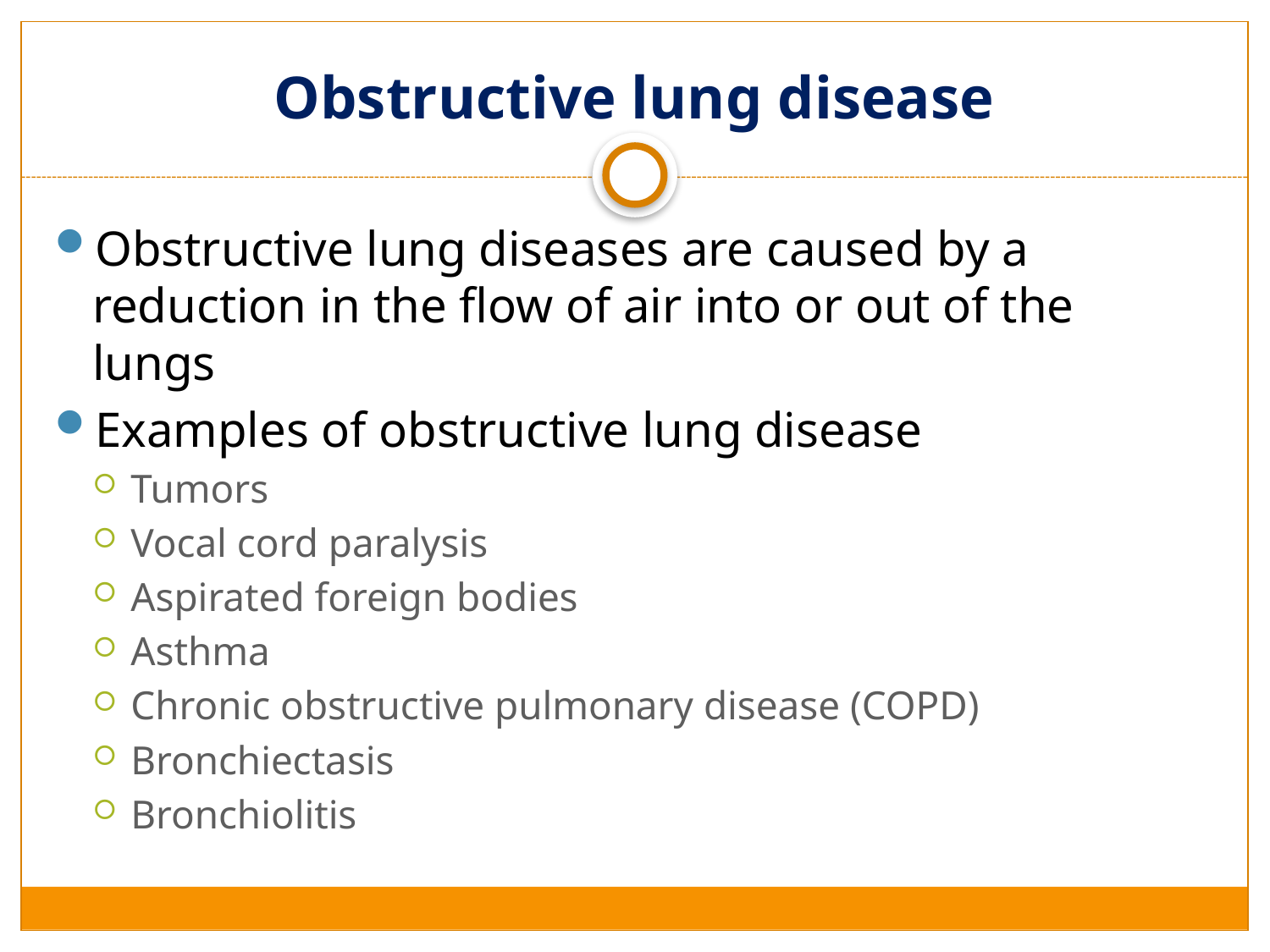

# Obstructive lung disease
Obstructive lung diseases are caused by a reduction in the flow of air into or out of the lungs
Examples of obstructive lung disease
Tumors
Vocal cord paralysis
Aspirated foreign bodies
Asthma
Chronic obstructive pulmonary disease (COPD)
Bronchiectasis
Bronchiolitis

## Slide 17
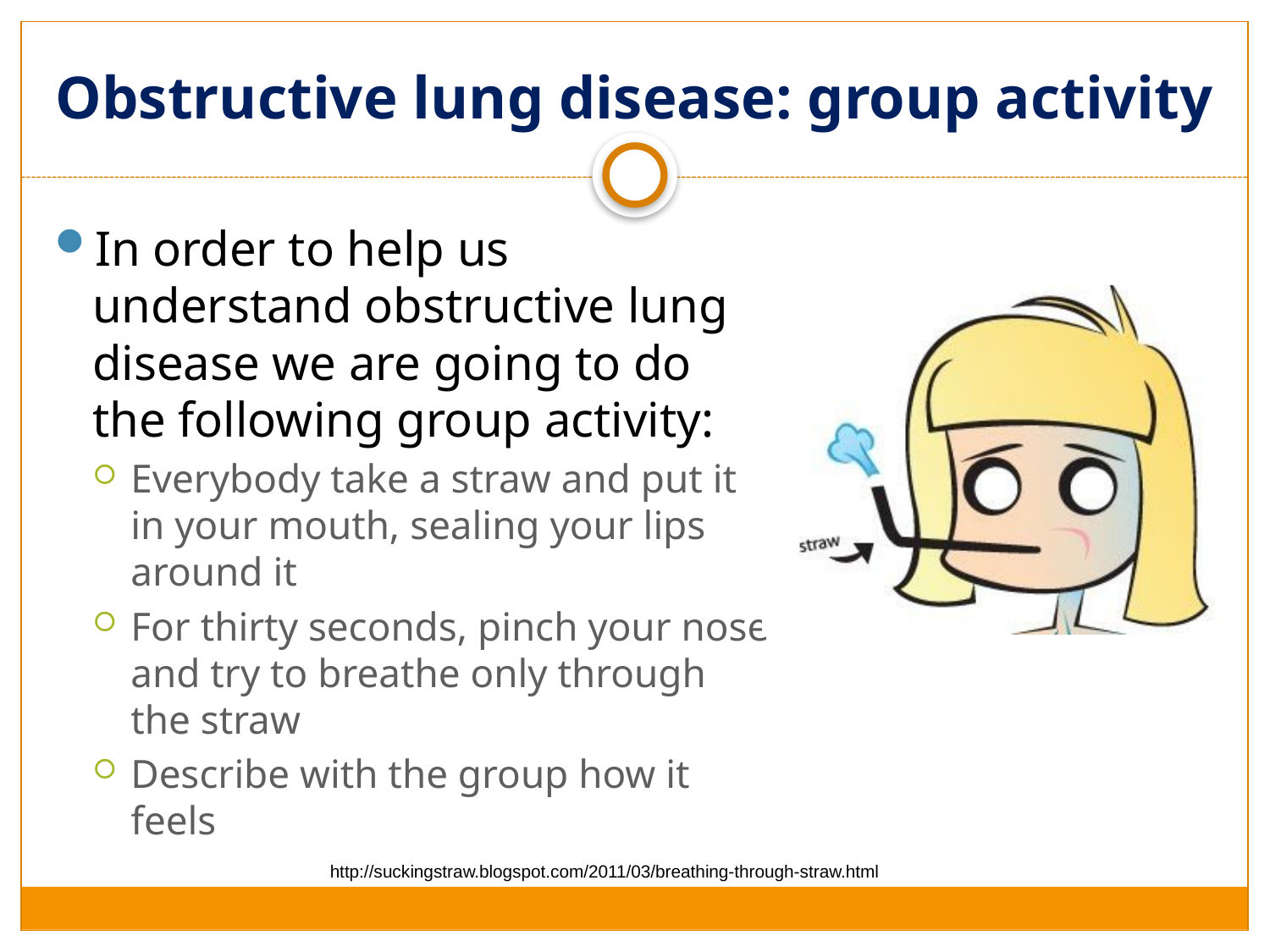

# Obstructive lung disease: group activity
In order to help us understand obstructive lung disease we are going to do the following group activity:
Everybody take a straw and put it in your mouth, sealing your lips around it
For thirty seconds, pinch your nose and try to breathe only through the straw
Describe with the group how it feels
http://suckingstraw.blogspot.com/2011/03/breathing-through-straw.html

## Slide 18
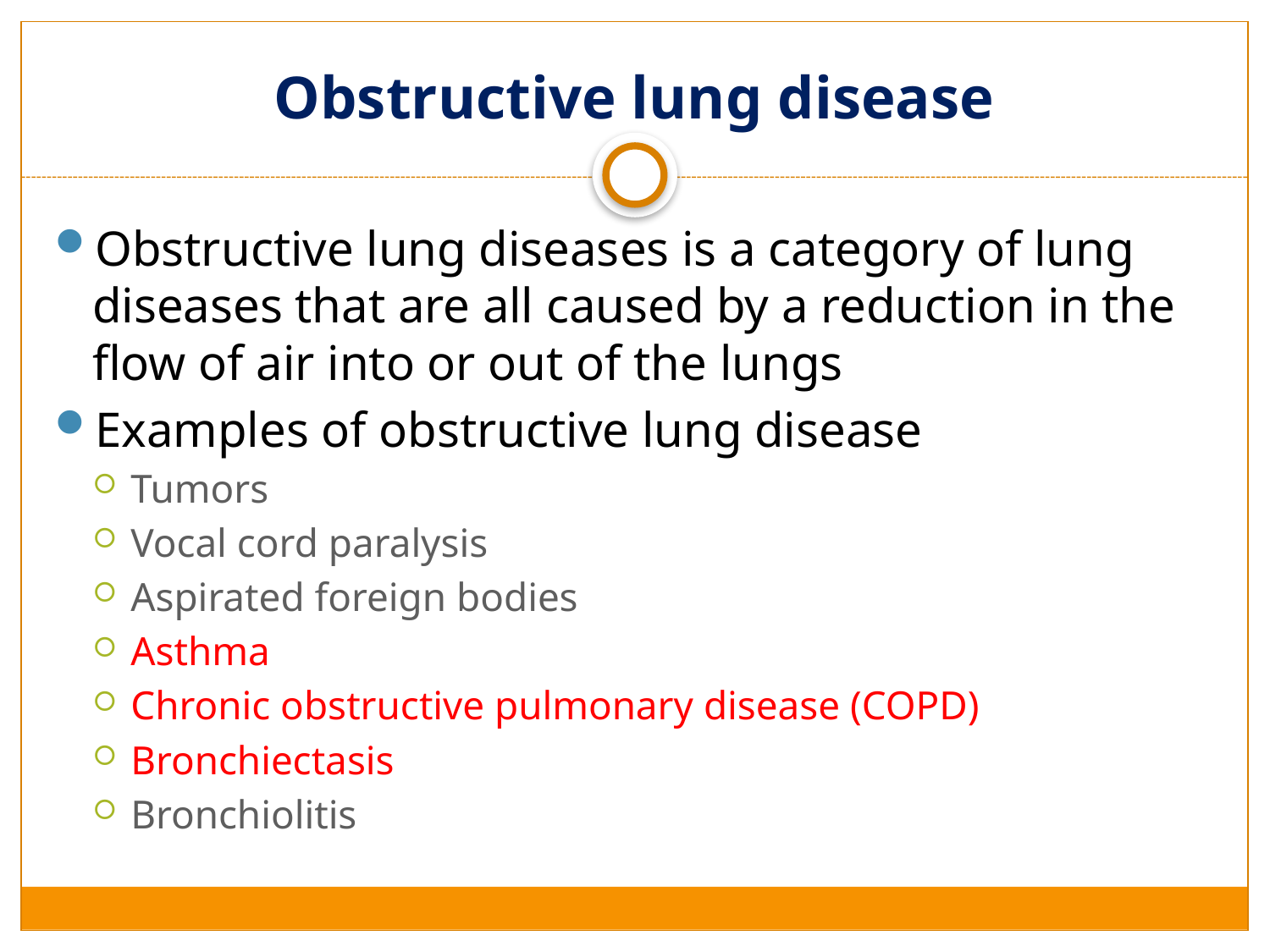

# Obstructive lung disease
Obstructive lung diseases is a category of lung diseases that are all caused by a reduction in the flow of air into or out of the lungs
Examples of obstructive lung disease
Tumors
Vocal cord paralysis
Aspirated foreign bodies
Asthma
Chronic obstructive pulmonary disease (COPD)
Bronchiectasis
Bronchiolitis

## Slide 19
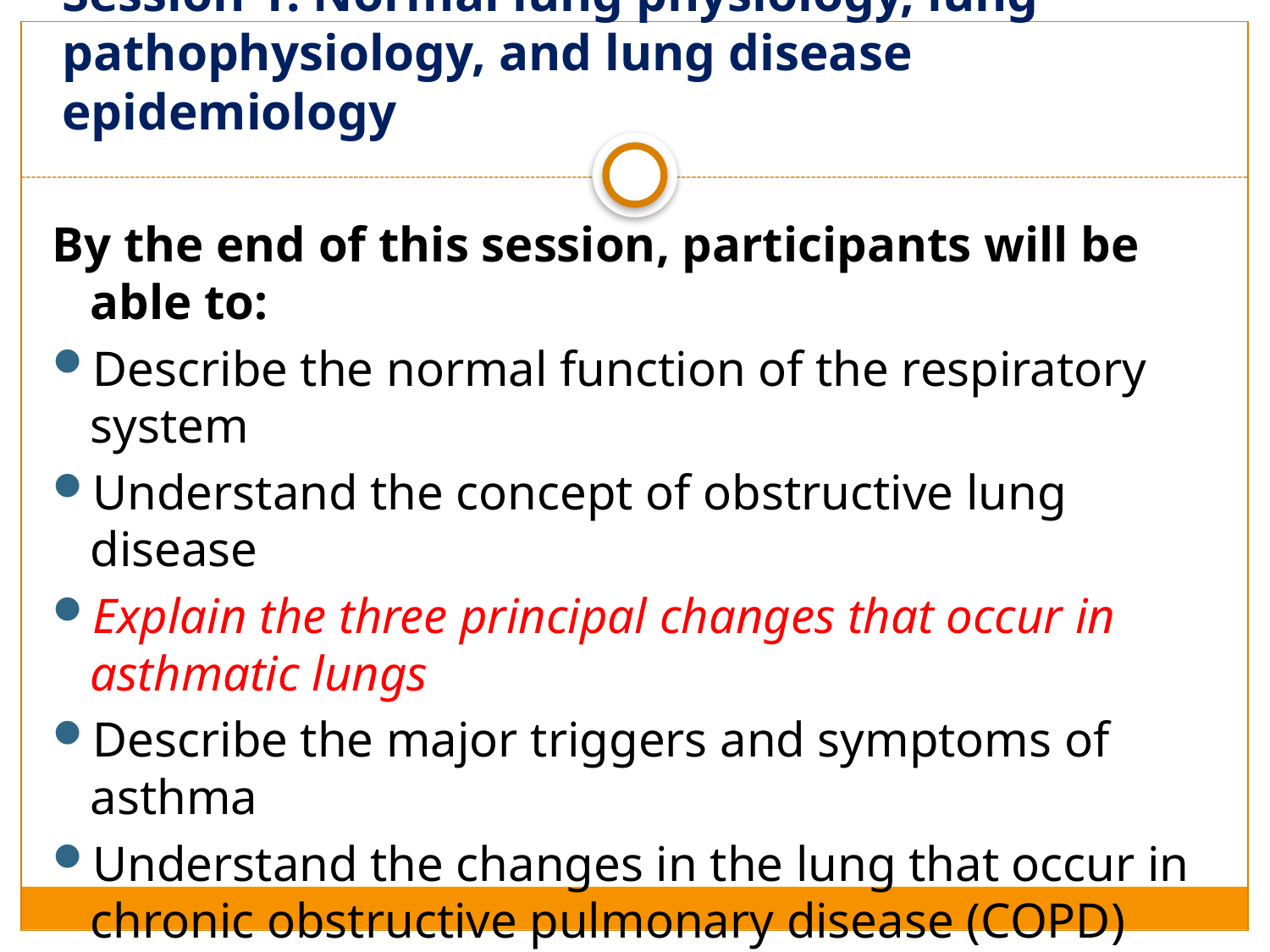

# Session 1: Normal lung physiology, lung pathophysiology, and lung disease epidemiology
By the end of this session, participants will be able to:
Describe the normal function of the respiratory system
Understand the concept of obstructive lung disease
Explain the three principal changes that occur in asthmatic lungs
Describe the major triggers and symptoms of asthma
Understand the changes in the lung that occur in chronic obstructive pulmonary disease (COPD)
Describe the epidemiology of asthma and COPD

## Slide 20
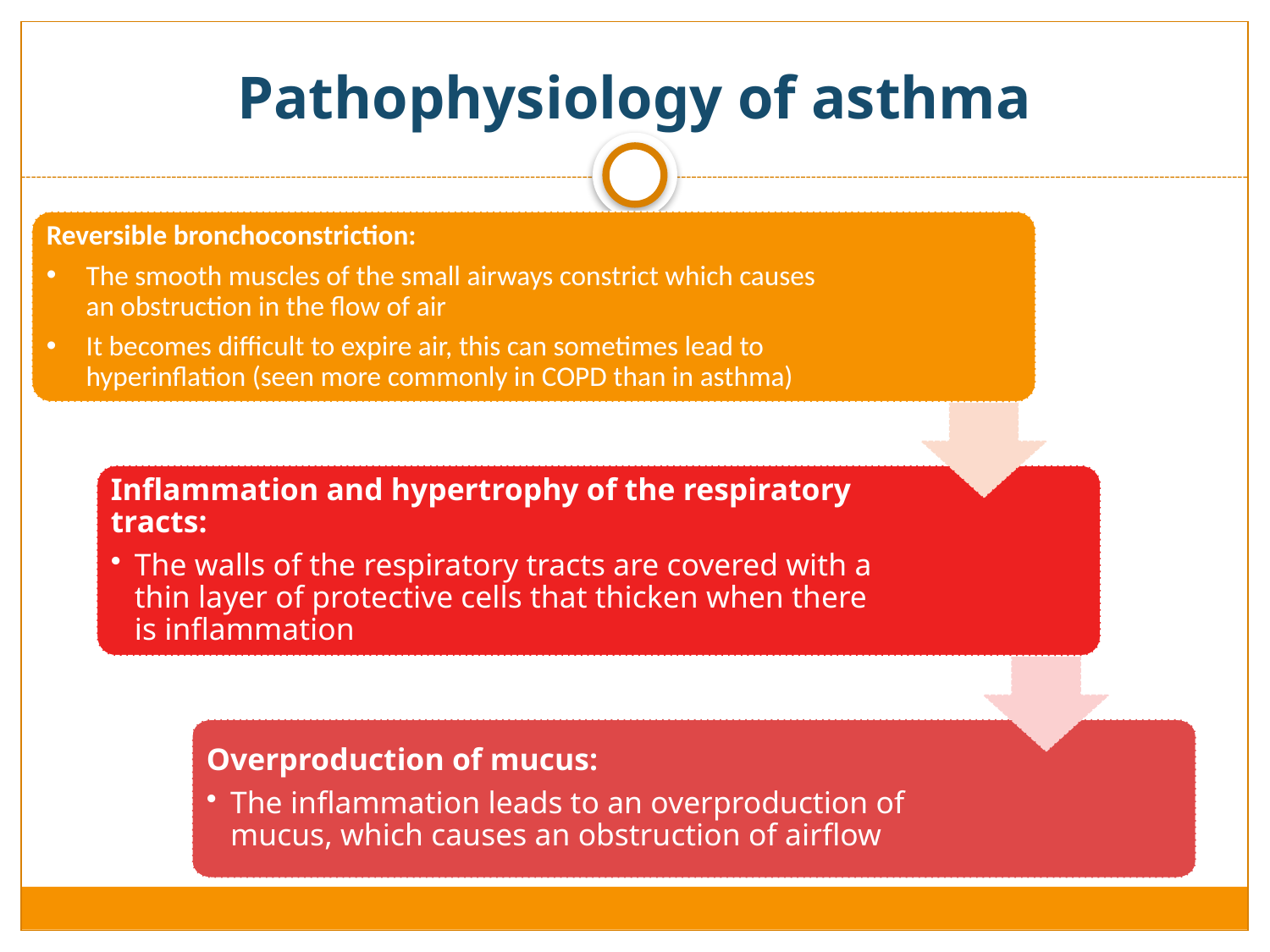

# Pathophysiology of asthma
Reversible bronchoconstriction:
The smooth muscles of the small airways constrict which causes an obstruction in the flow of air
It becomes difficult to expire air, this can sometimes lead to hyperinflation (seen more commonly in COPD than in asthma)
Inflammation and hypertrophy of the respiratory tracts:
The walls of the respiratory tracts are covered with a thin layer of protective cells that thicken when there is inflammation
Overproduction of mucus:
The inflammation leads to an overproduction of mucus, which causes an obstruction of airflow

## Slide 21
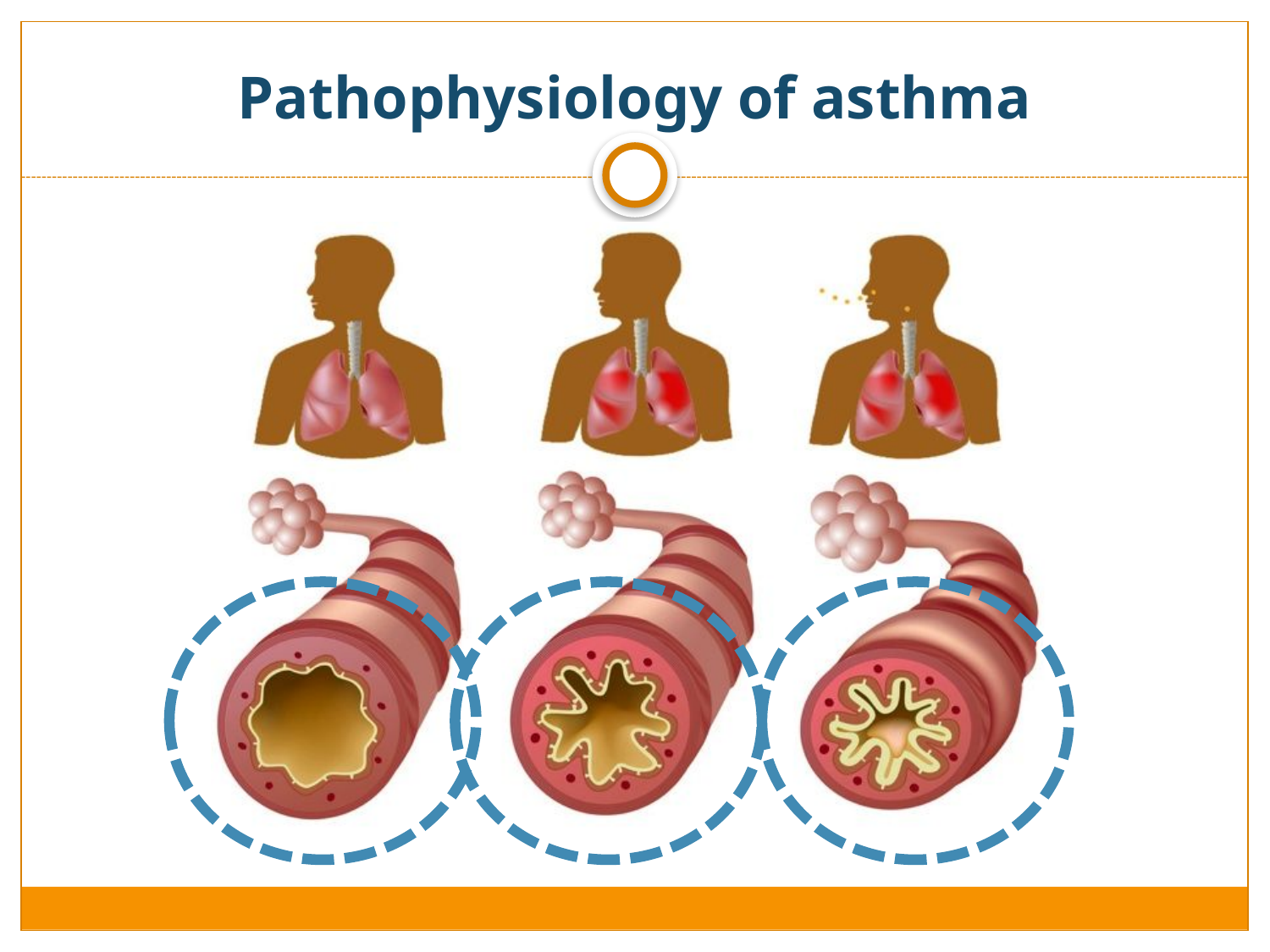

# Pathophysiology of asthma

## Slide 22
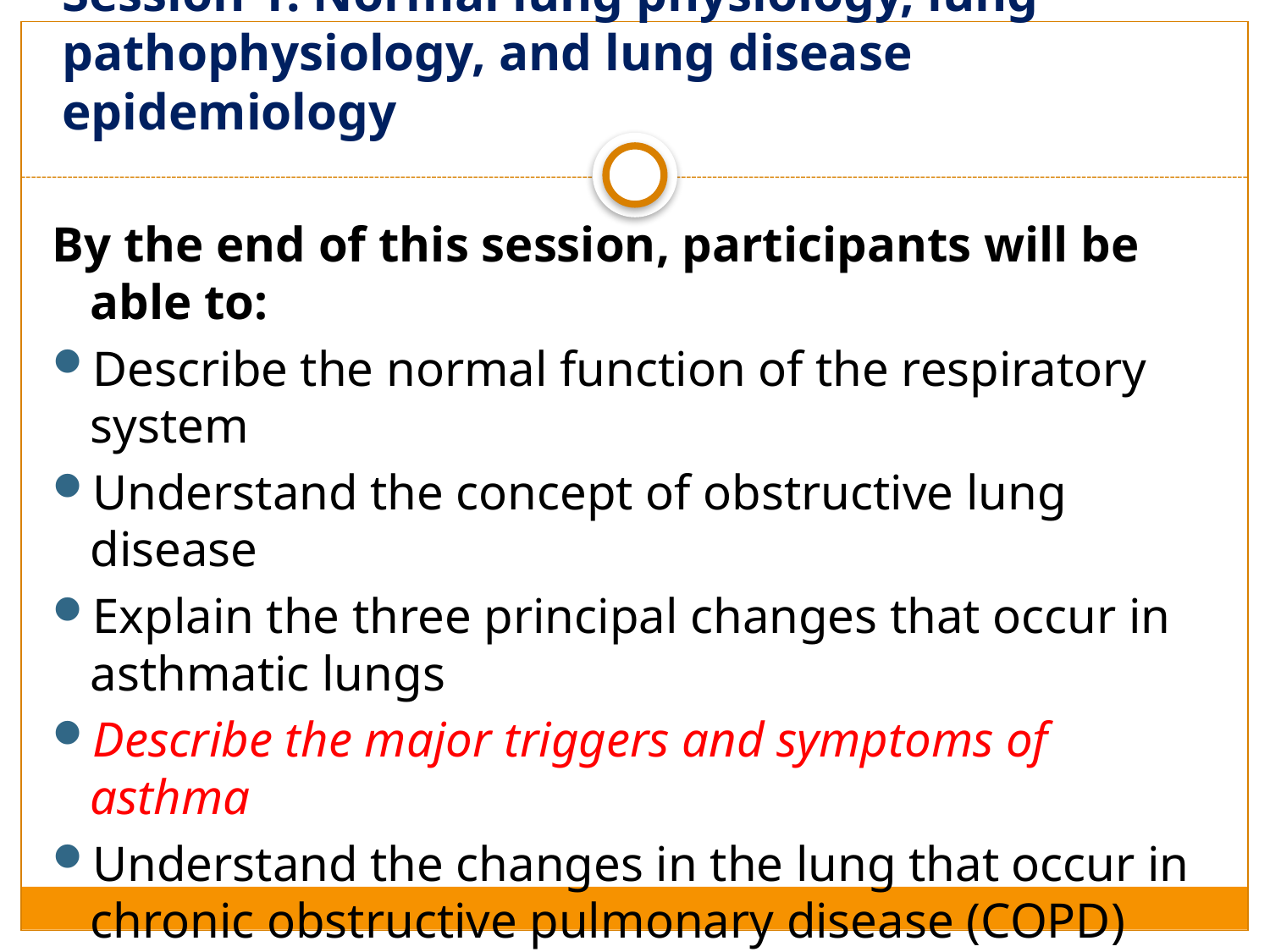

# Session 1: Normal lung physiology, lung pathophysiology, and lung disease epidemiology
By the end of this session, participants will be able to:
Describe the normal function of the respiratory system
Understand the concept of obstructive lung disease
Explain the three principal changes that occur in asthmatic lungs
Describe the major triggers and symptoms of asthma
Understand the changes in the lung that occur in chronic obstructive pulmonary disease (COPD)
Describe the epidemiology of asthma and COPD

## Slide 23
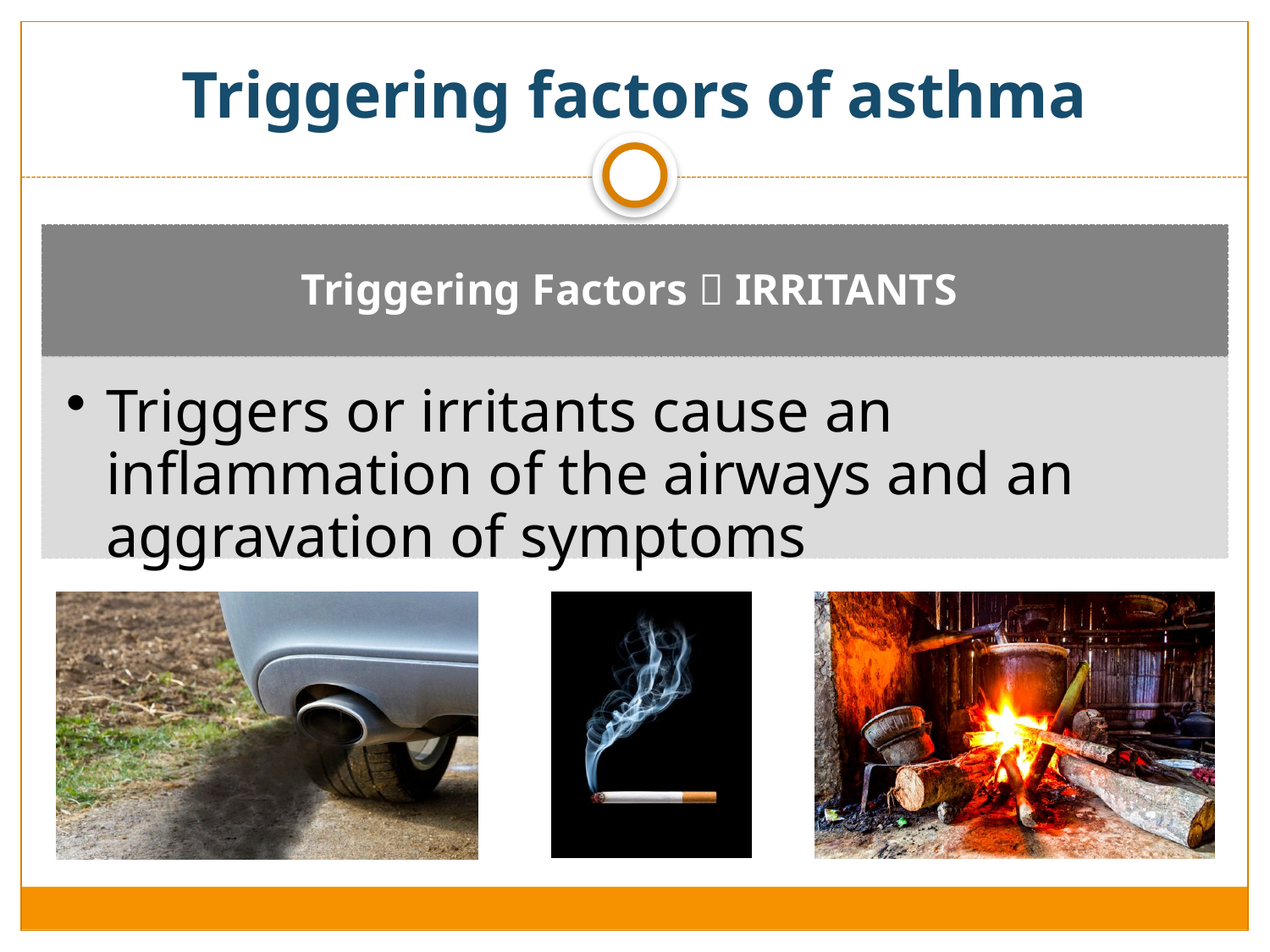

# Triggering factors of asthma

## Slide 24
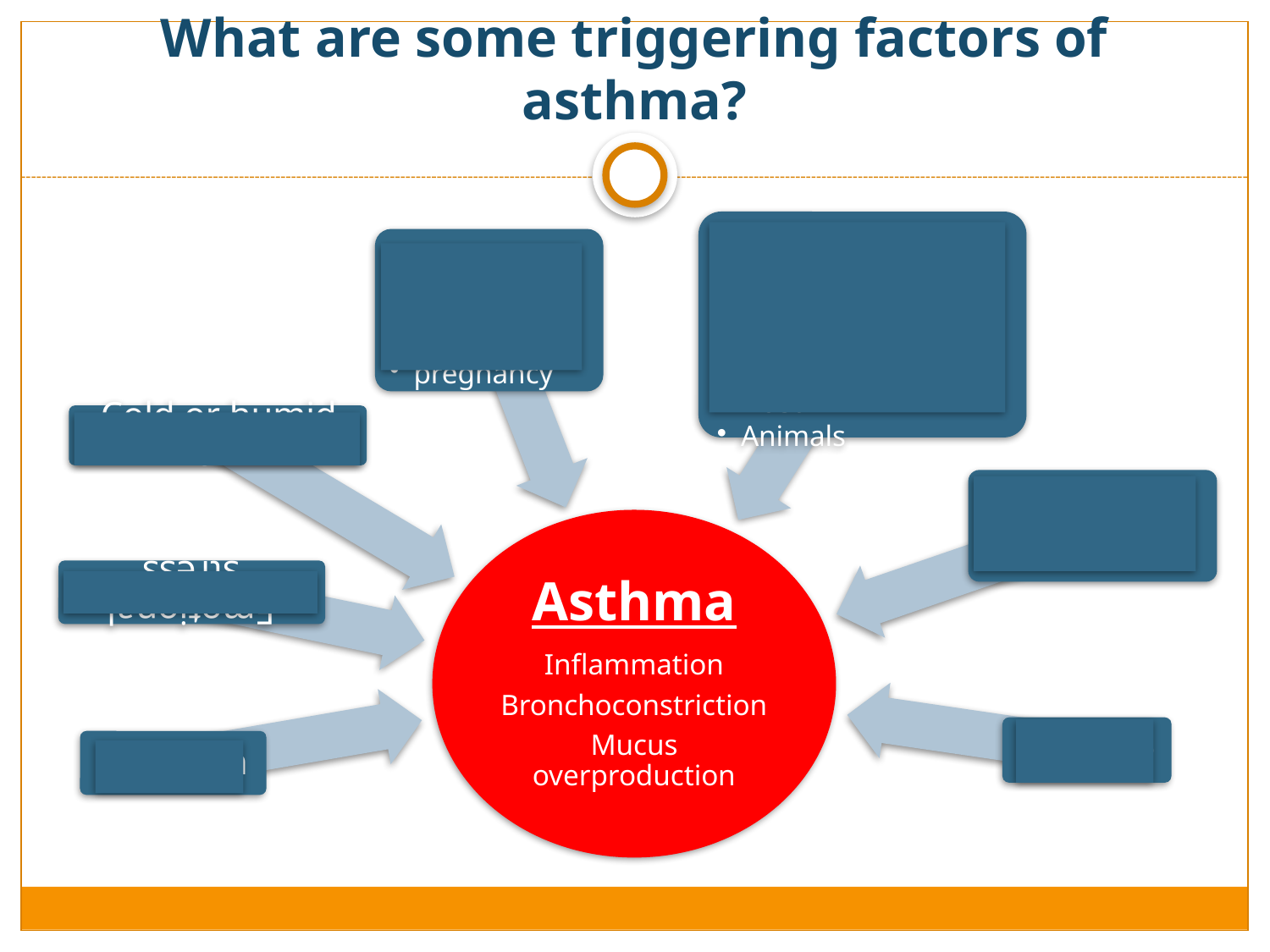

# What are some triggering factors of asthma?

## Slide 25
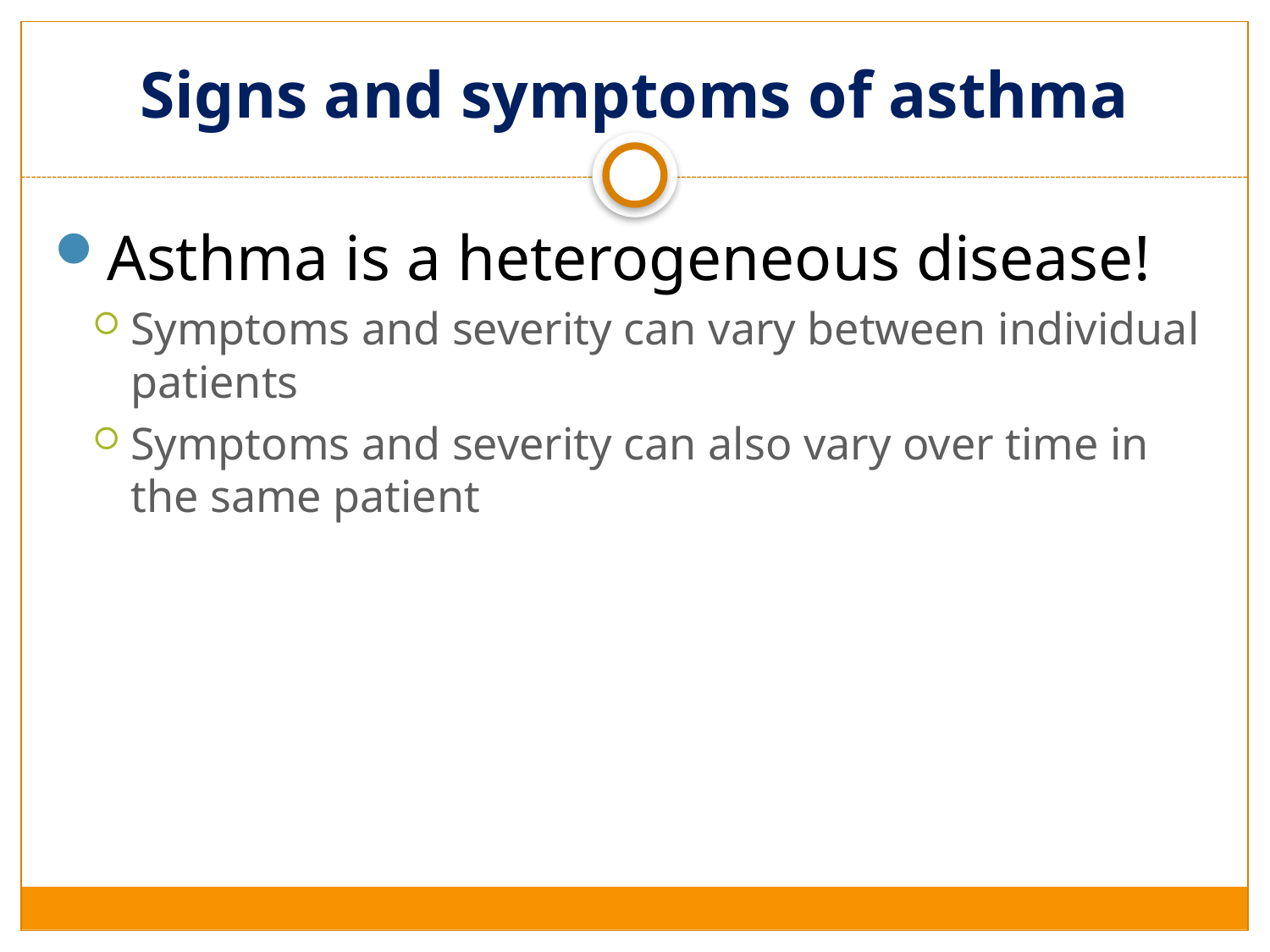

# Signs and symptoms of asthma
Asthma is a heterogeneous disease!
Symptoms and severity can vary between individual patients
Symptoms and severity can also vary over time in the same patient

## Slide 26
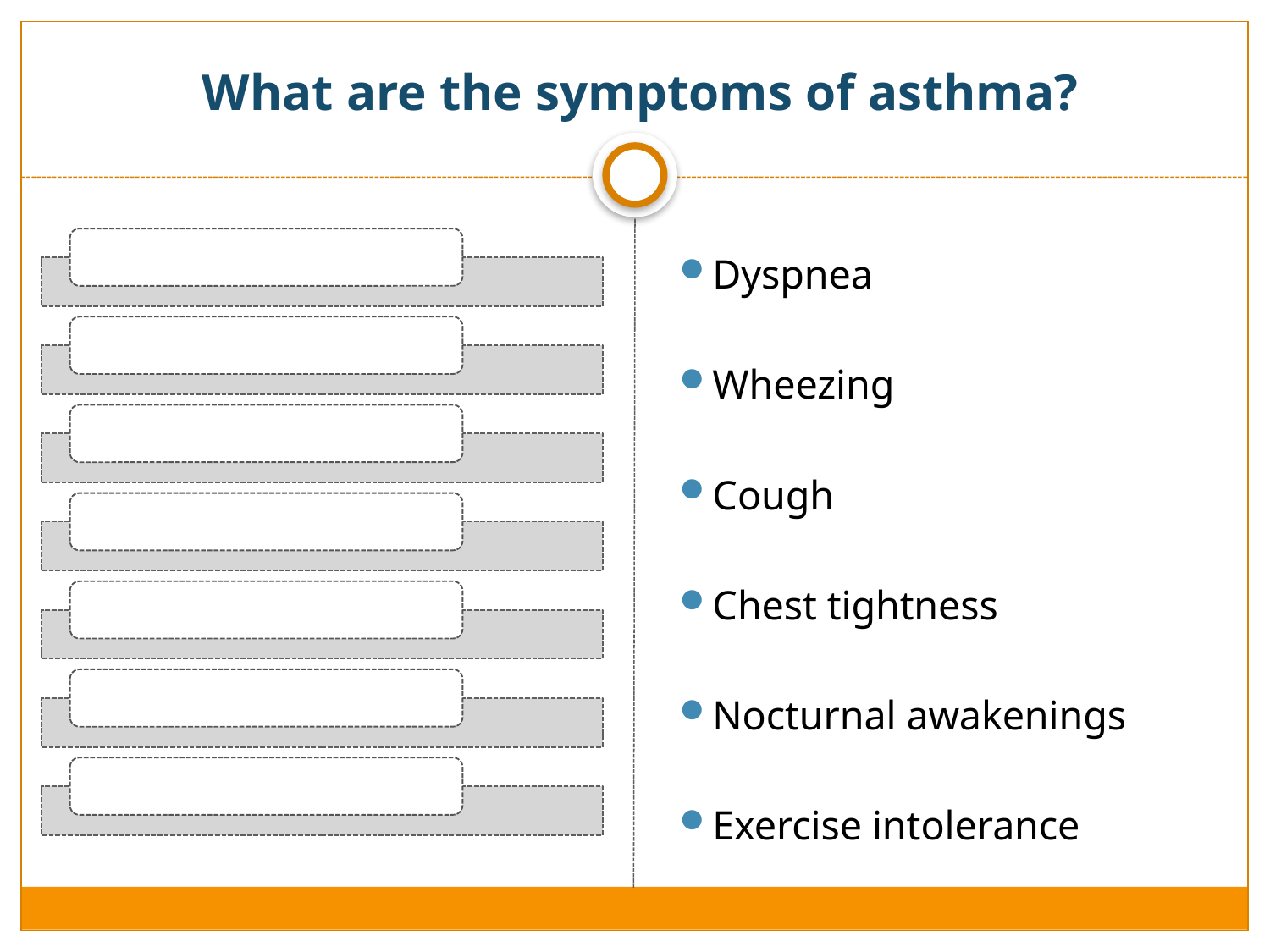

# What are the symptoms of asthma?
Dyspnea
Wheezing
Cough
Chest tightness
Nocturnal awakenings
Exercise intolerance

## Slide 27
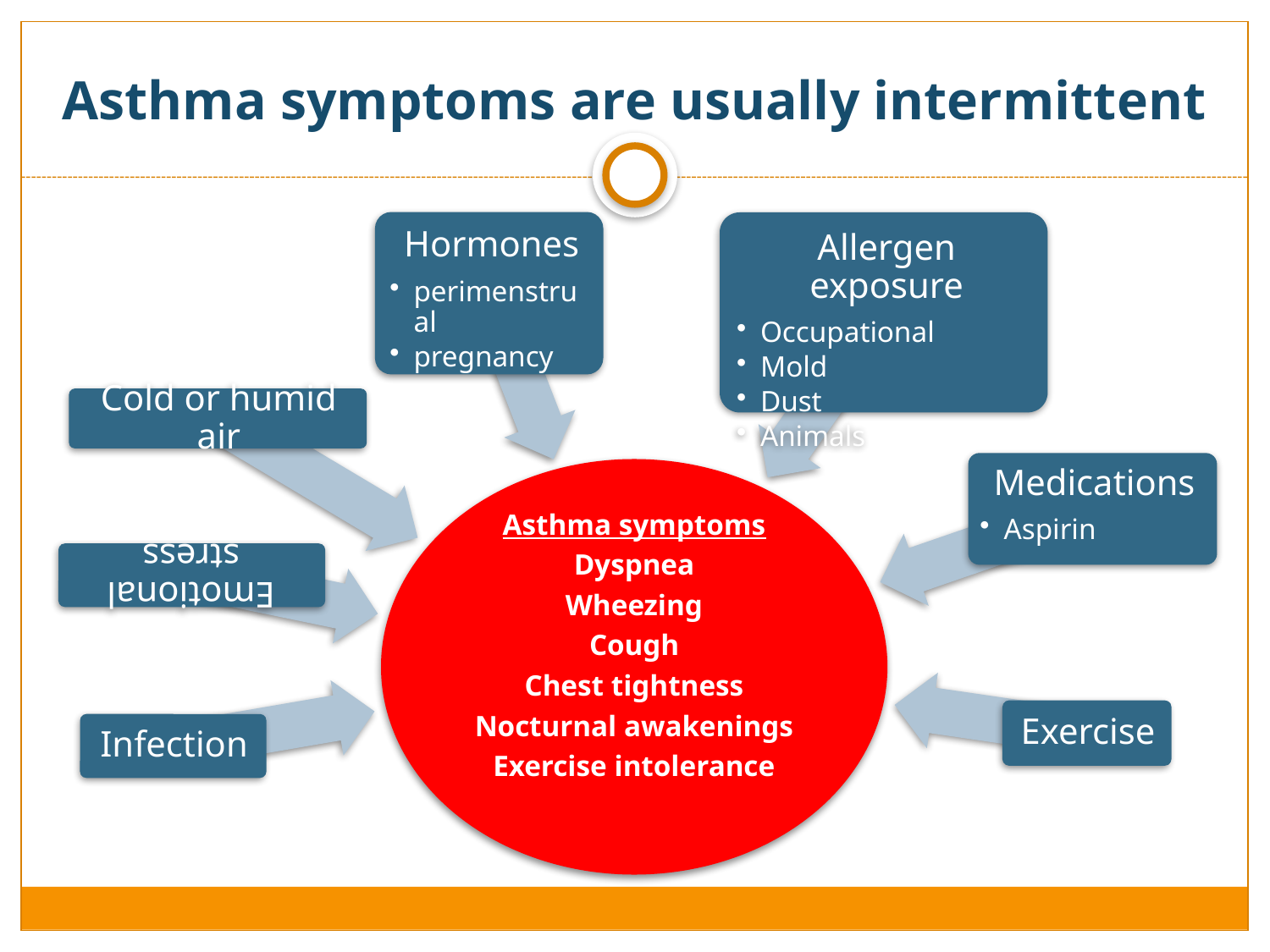

# Asthma symptoms are usually intermittent

## Slide 28
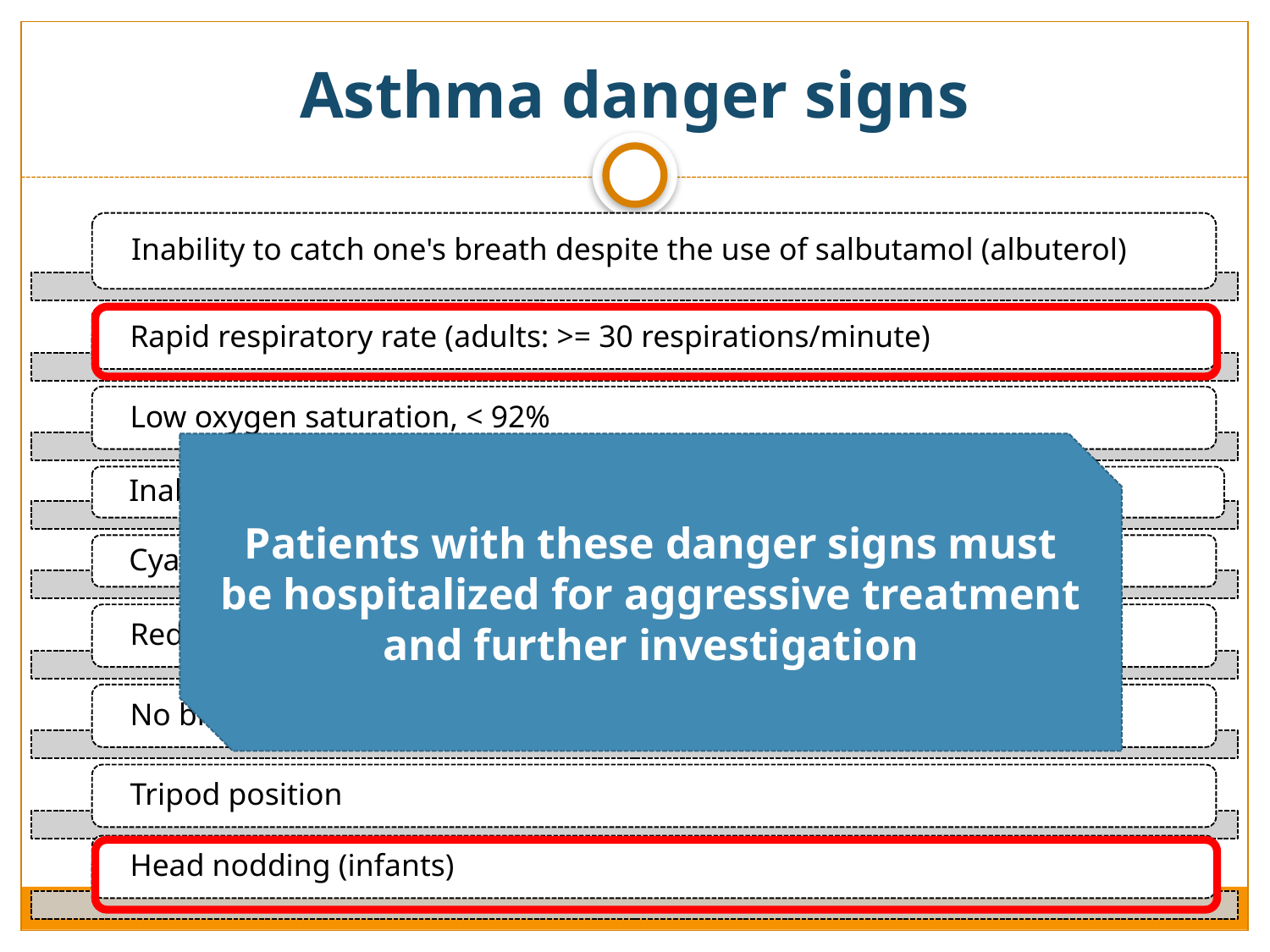

# Asthma danger signs
Patients with these danger signs must be hospitalized for aggressive treatment and further investigation

## Slide 29
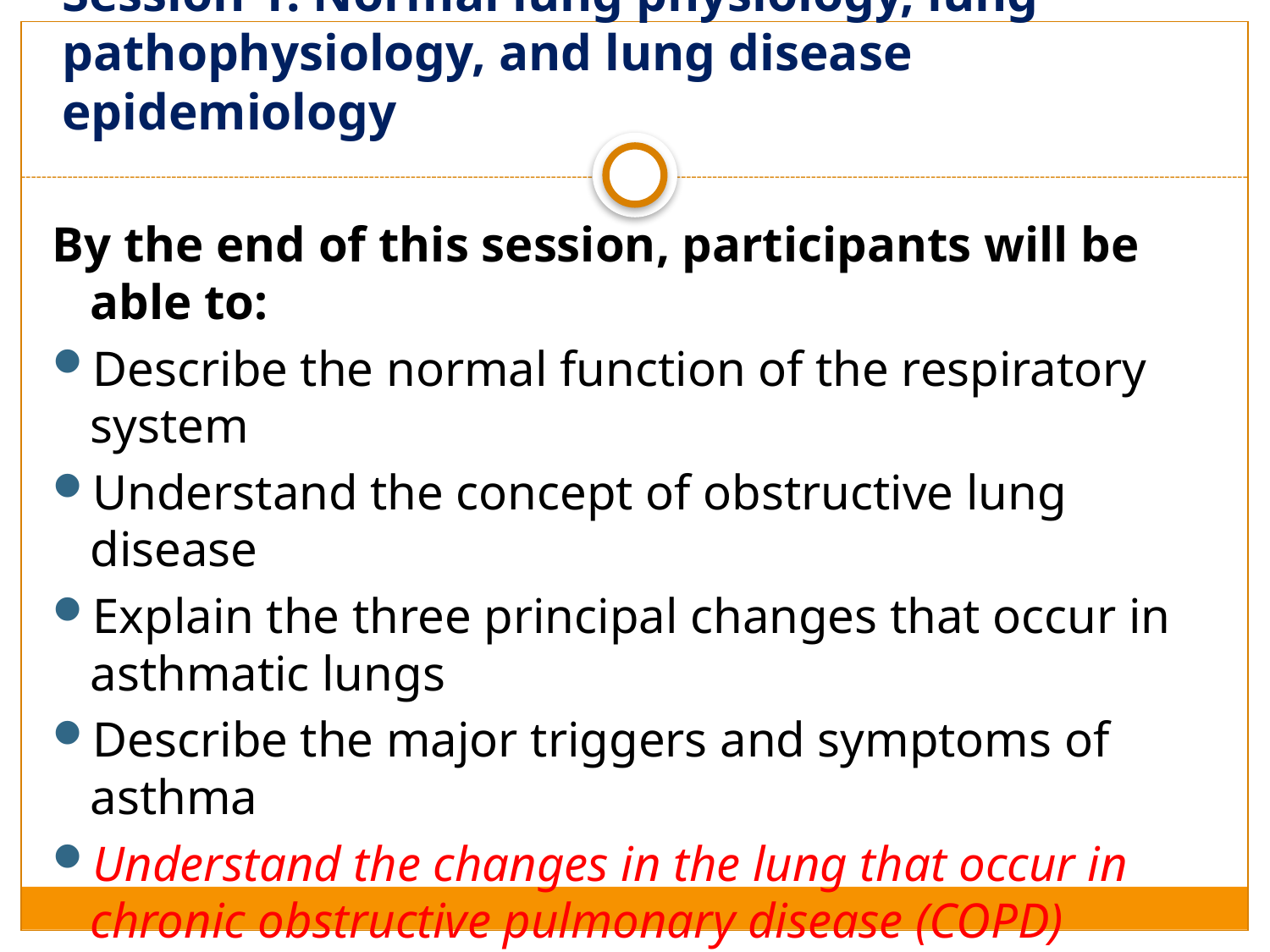

# Session 1: Normal lung physiology, lung pathophysiology, and lung disease epidemiology
By the end of this session, participants will be able to:
Describe the normal function of the respiratory system
Understand the concept of obstructive lung disease
Explain the three principal changes that occur in asthmatic lungs
Describe the major triggers and symptoms of asthma
Understand the changes in the lung that occur in chronic obstructive pulmonary disease (COPD)
Describe the epidemiology of asthma and COPD

## Slide 30
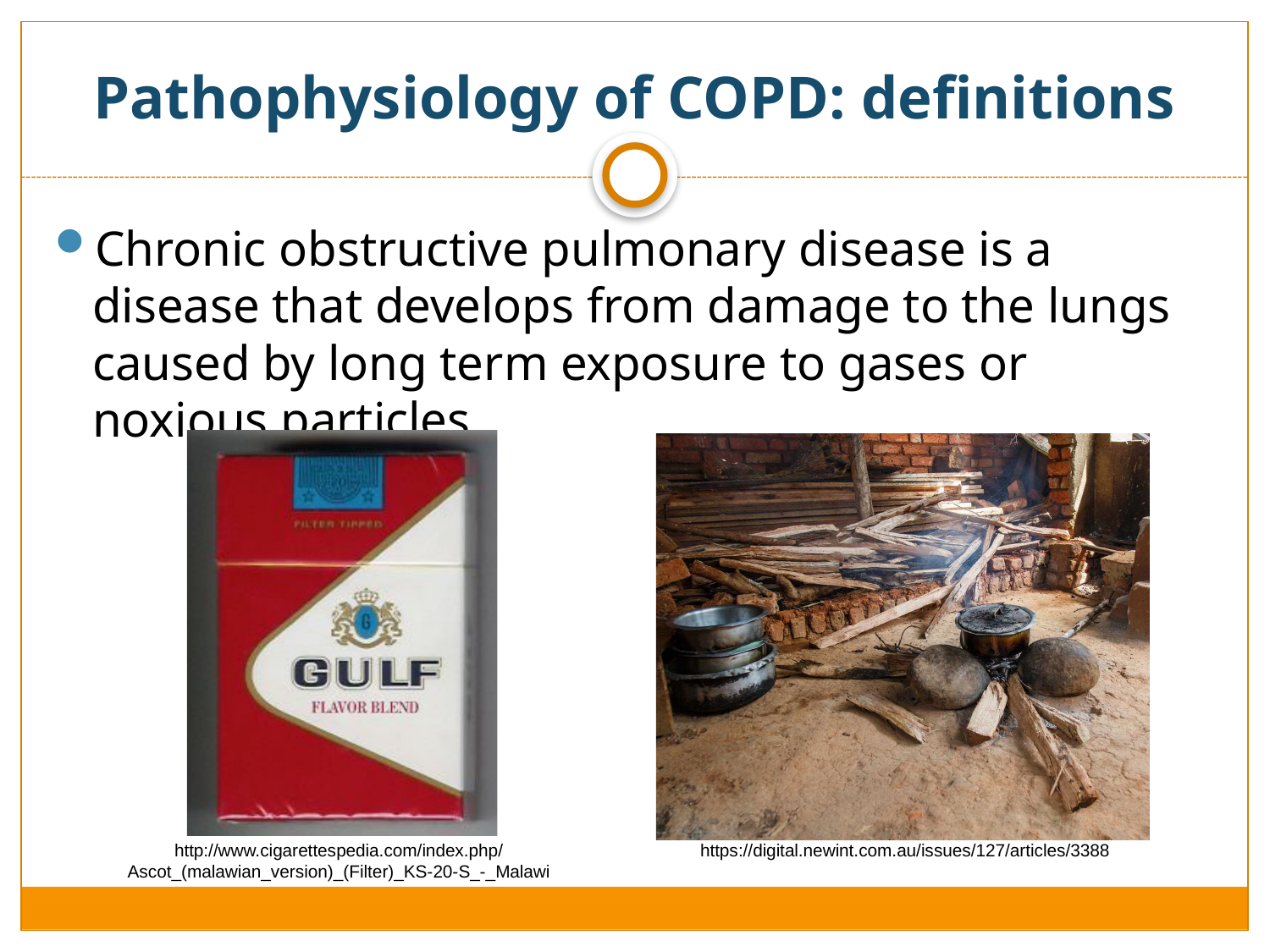

# Pathophysiology of COPD: definitions
Chronic obstructive pulmonary disease is a disease that develops from damage to the lungs caused by long term exposure to gases or noxious particles
http://www.cigarettespedia.com/index.php/Ascot_(malawian_version)_(Filter)_KS-20-S_-_Malawi
https://digital.newint.com.au/issues/127/articles/3388

## Slide 31
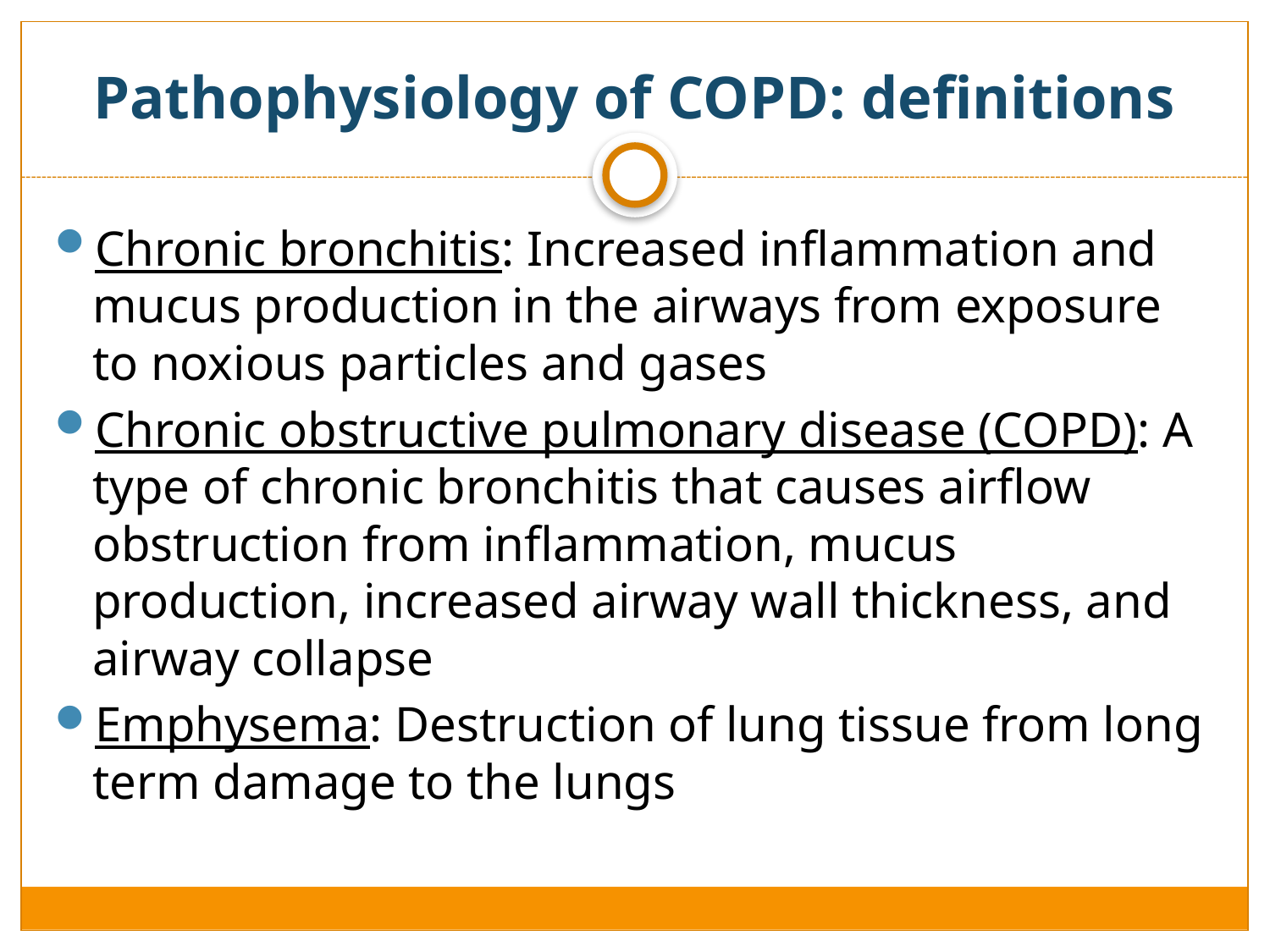

# Pathophysiology of COPD: definitions
Chronic bronchitis: Increased inflammation and mucus production in the airways from exposure to noxious particles and gases
Chronic obstructive pulmonary disease (COPD): A type of chronic bronchitis that causes airflow obstruction from inflammation, mucus production, increased airway wall thickness, and airway collapse
Emphysema: Destruction of lung tissue from long term damage to the lungs

## Slide 32
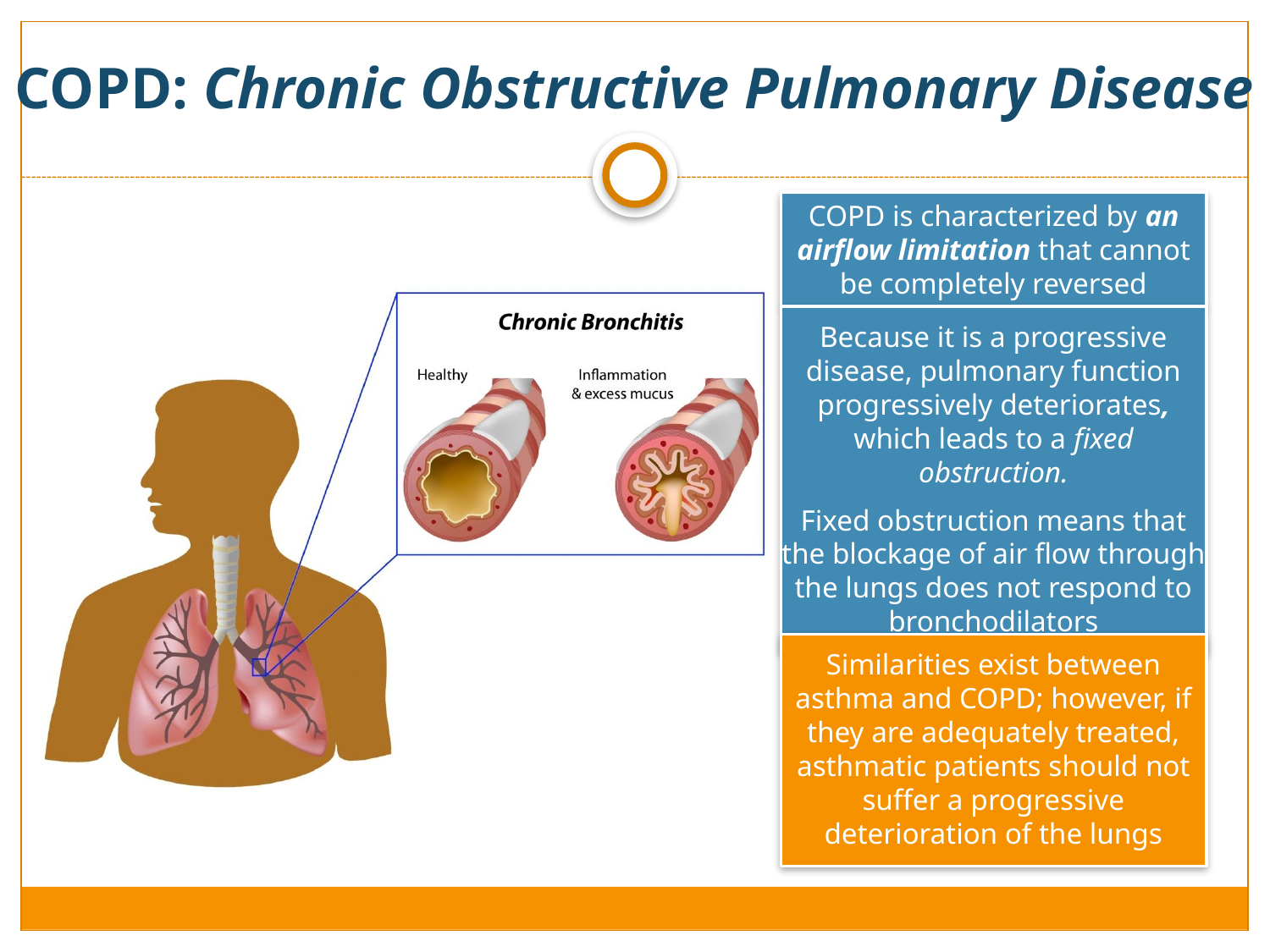

# COPD: Chronic Obstructive Pulmonary Disease
COPD is characterized by an airflow limitation that cannot be completely reversed
Because it is a progressive disease, pulmonary function progressively deteriorates, which leads to a fixed obstruction.
Fixed obstruction means that the blockage of air flow through the lungs does not respond to bronchodilators
Similarities exist between asthma and COPD; however, if they are adequately treated, asthmatic patients should not suffer a progressive deterioration of the lungs

## Slide 33
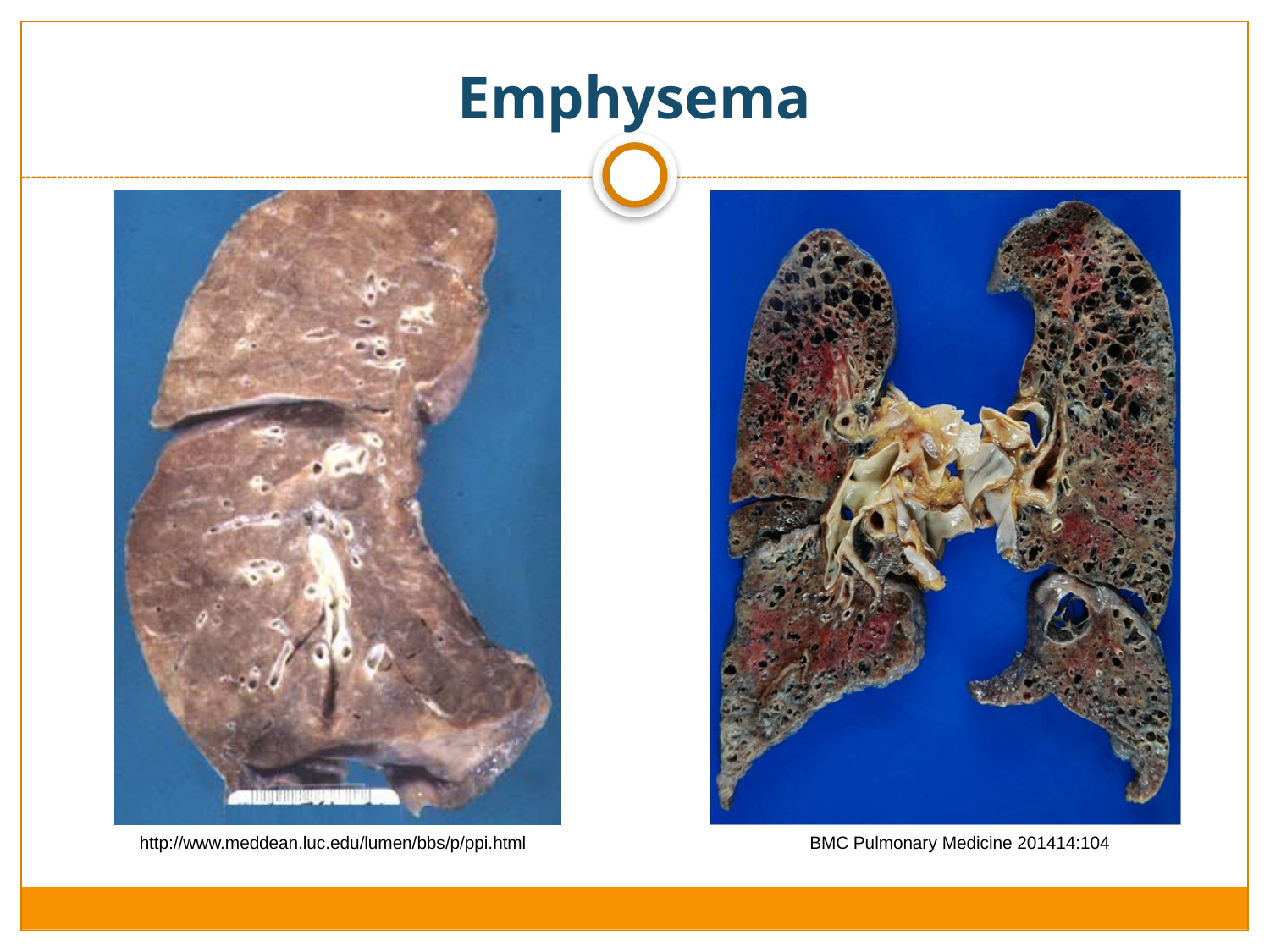

# Emphysema
http://www.meddean.luc.edu/lumen/bbs/p/ppi.html
BMC Pulmonary Medicine 201414:104

## Slide 34
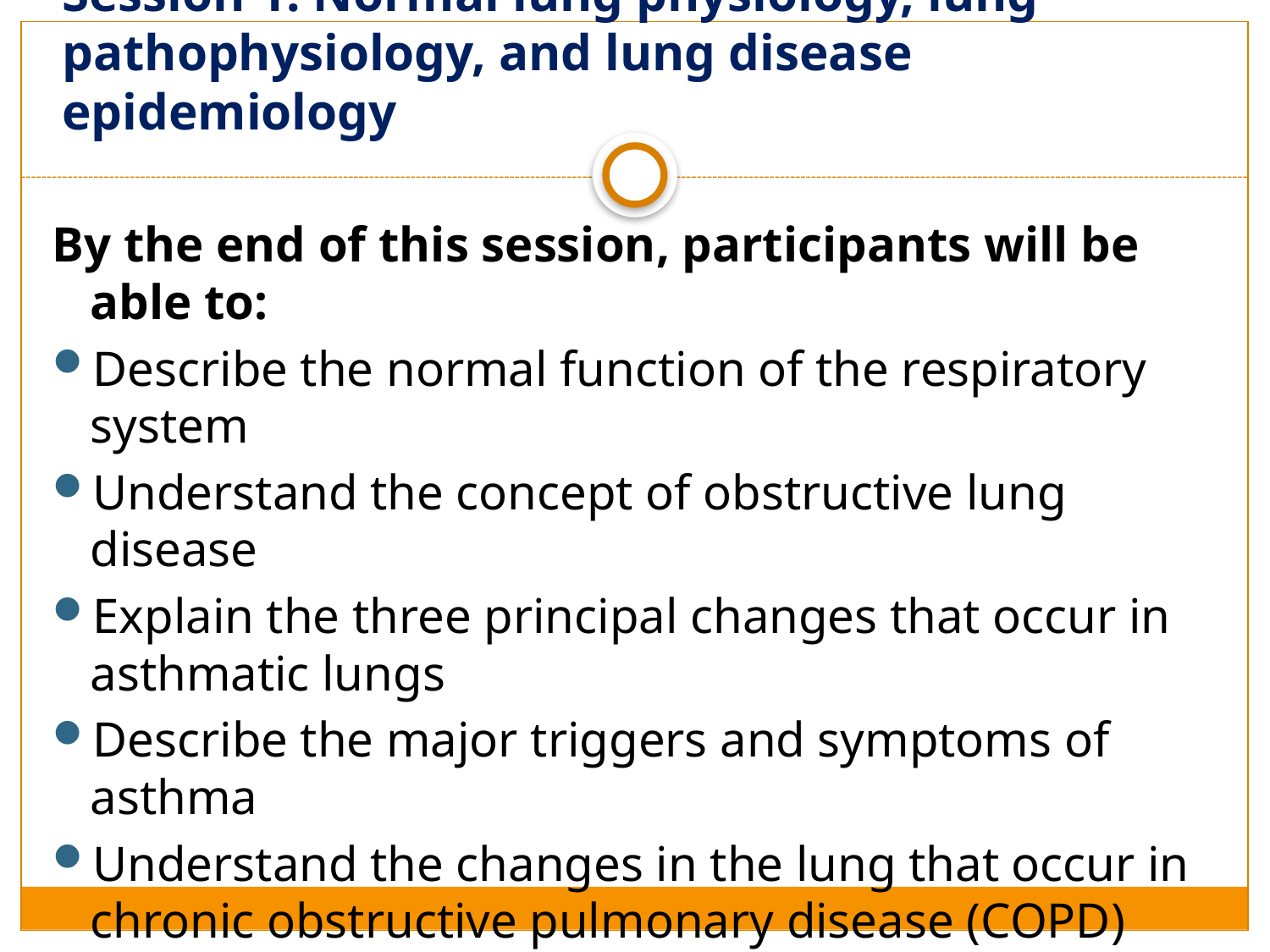

# Session 1: Normal lung physiology, lung pathophysiology, and lung disease epidemiology
By the end of this session, participants will be able to:
Describe the normal function of the respiratory system
Understand the concept of obstructive lung disease
Explain the three principal changes that occur in asthmatic lungs
Describe the major triggers and symptoms of asthma
Understand the changes in the lung that occur in chronic obstructive pulmonary disease (COPD)
Describe the epidemiology of asthma and COPD

## Slide 35
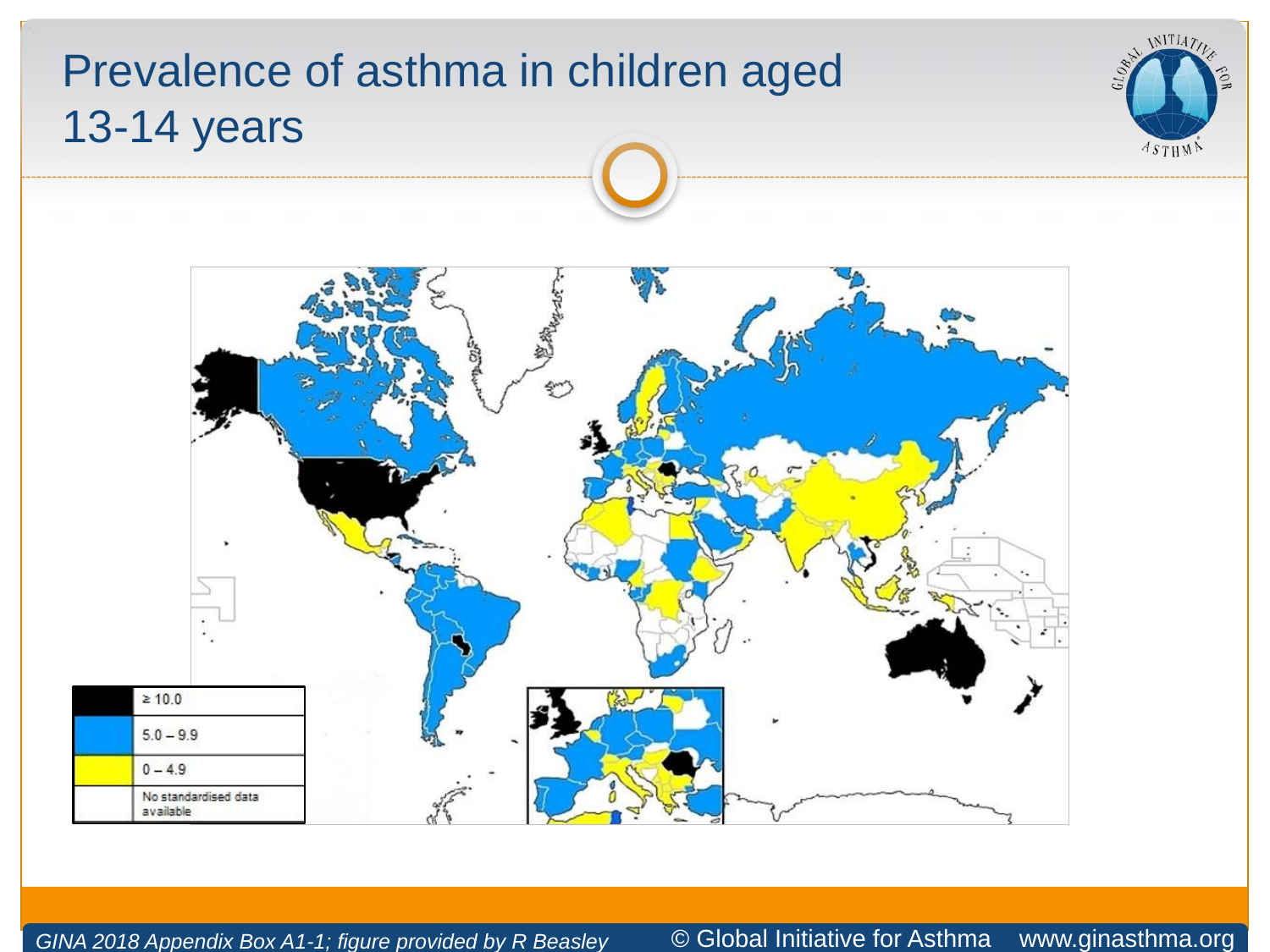

# Prevalence of asthma in children aged 13-14 years
GINA 2018 Appendix Box A1-1; figure provided by R Beasley

## Slide 36
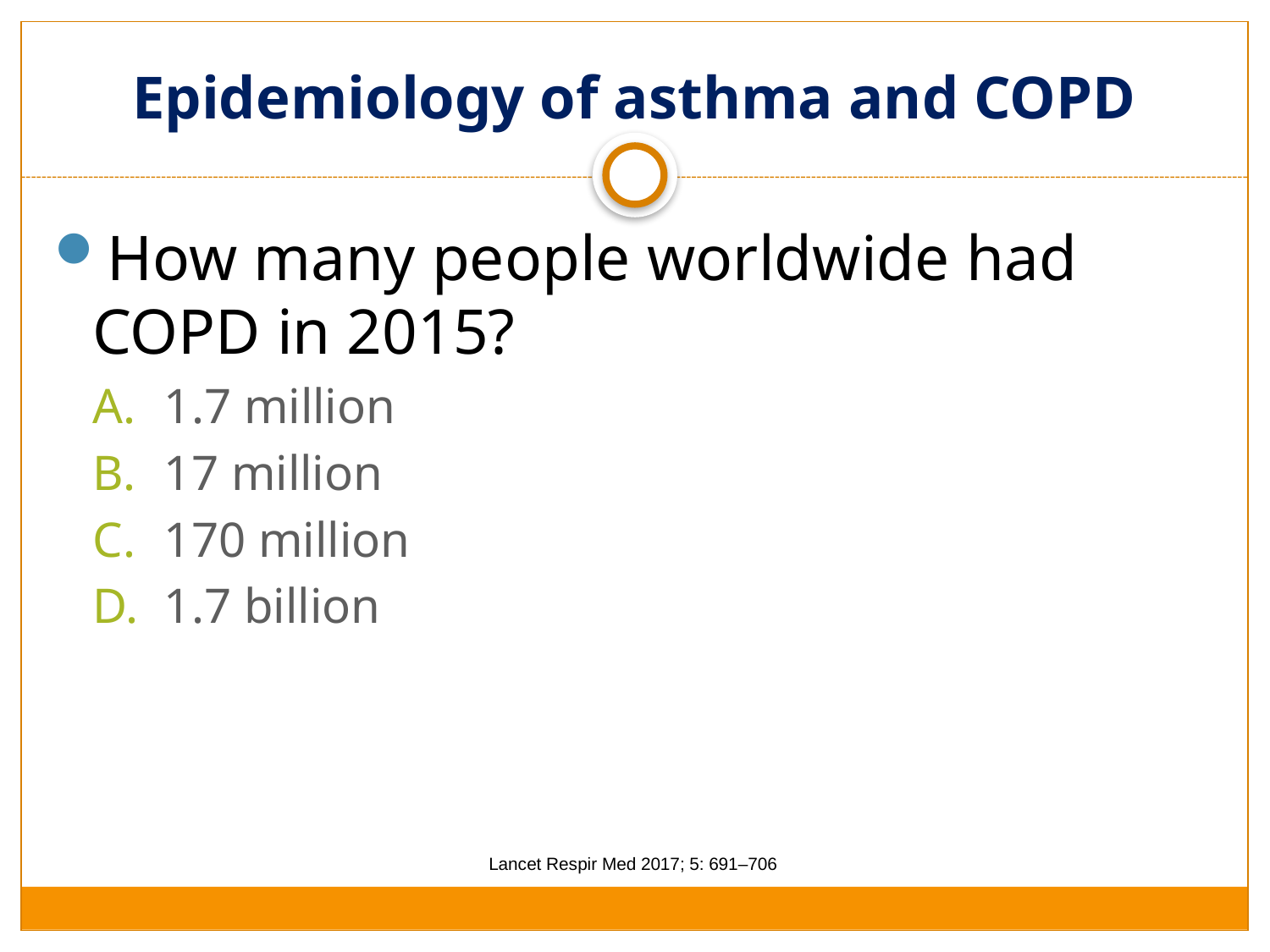

# Epidemiology of asthma and COPD
How many people worldwide had COPD in 2015?
1.7 million
17 million
170 million
1.7 billion
Lancet Respir Med 2017; 5: 691–706

## Slide 37
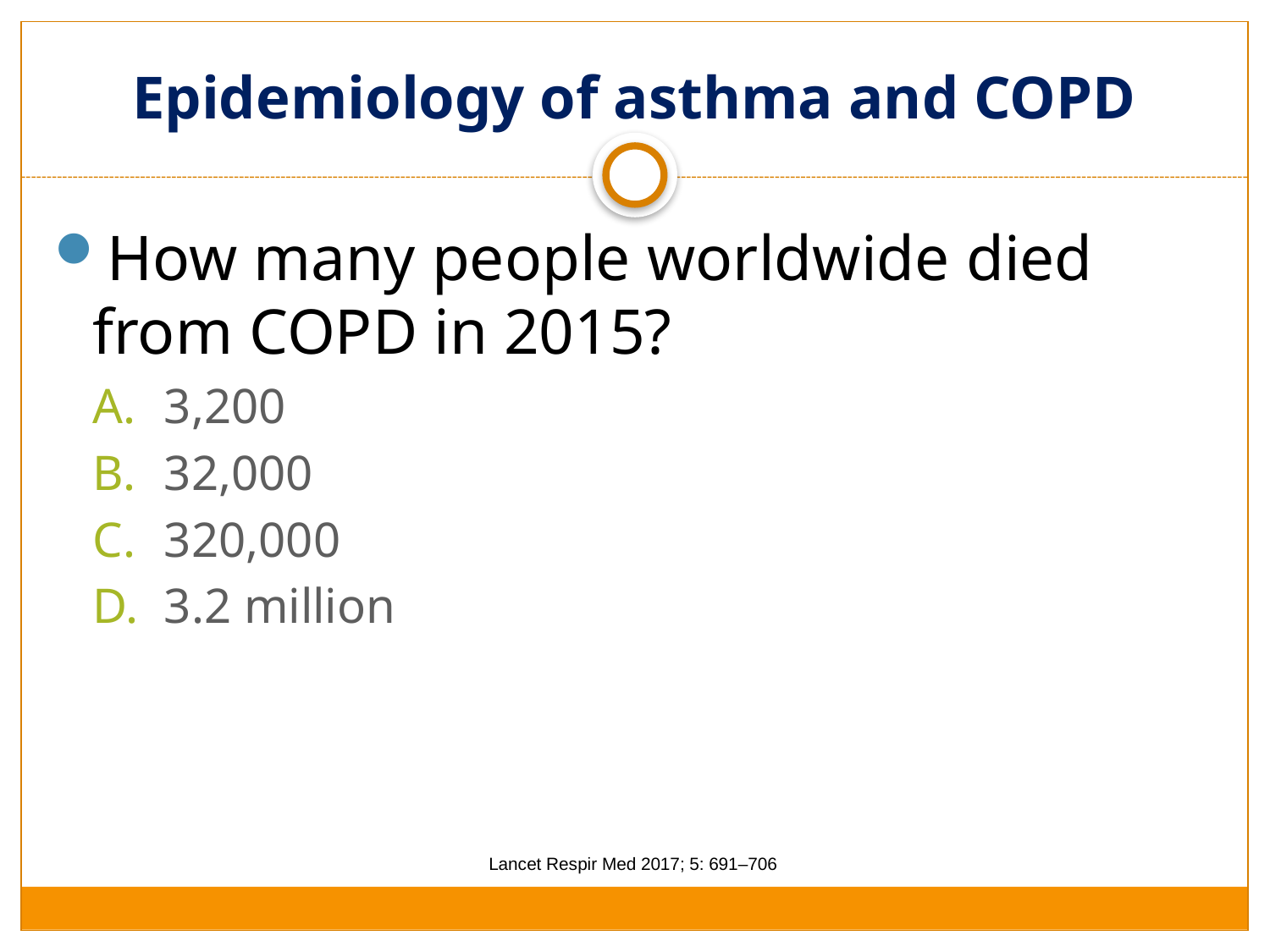

# Epidemiology of asthma and COPD
How many people worldwide died from COPD in 2015?
3,200
32,000
320,000
3.2 million
Lancet Respir Med 2017; 5: 691–706

## Slide 38
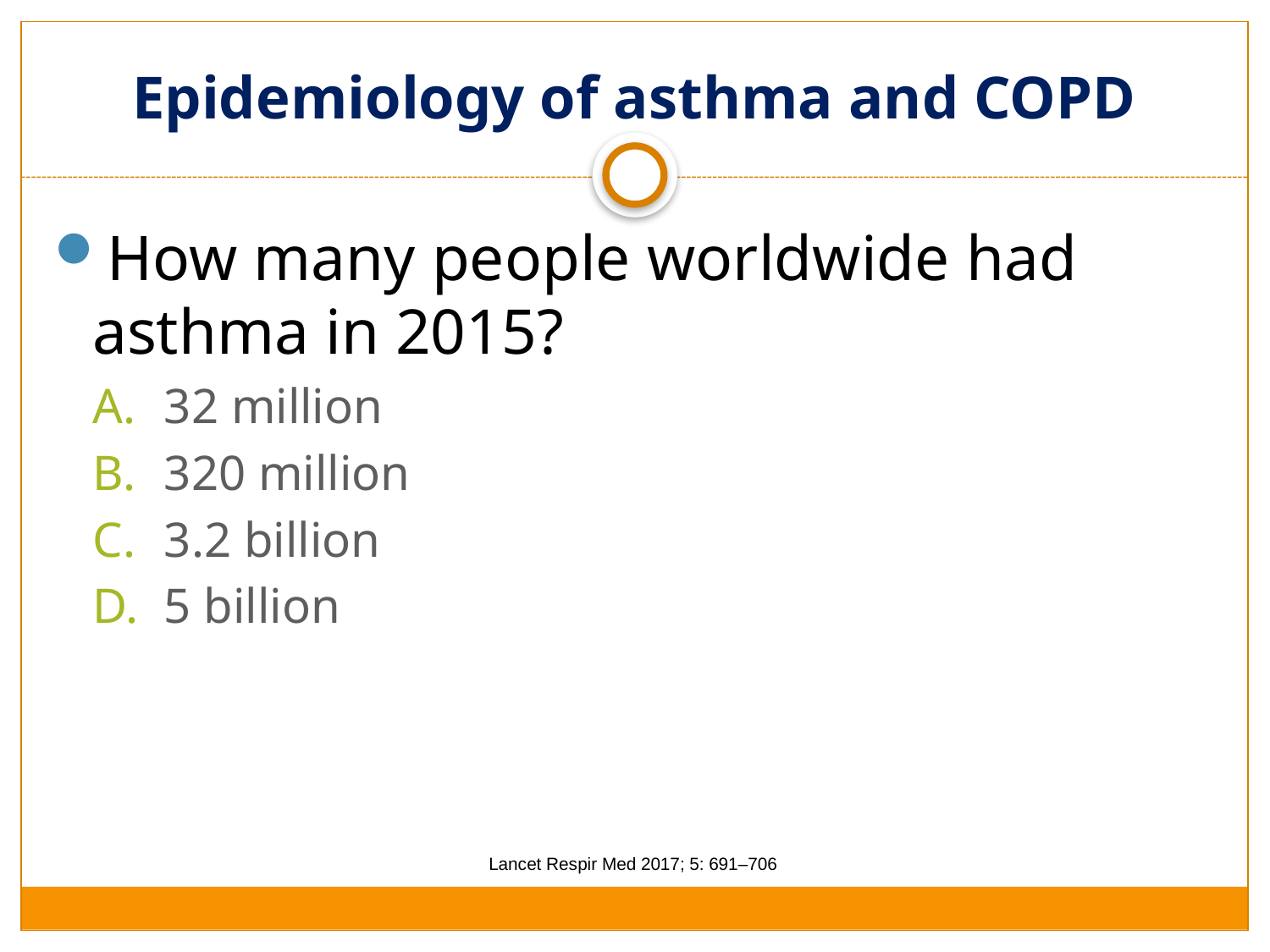

# Epidemiology of asthma and COPD
How many people worldwide had asthma in 2015?
32 million
320 million
3.2 billion
5 billion
Lancet Respir Med 2017; 5: 691–706

## Slide 39
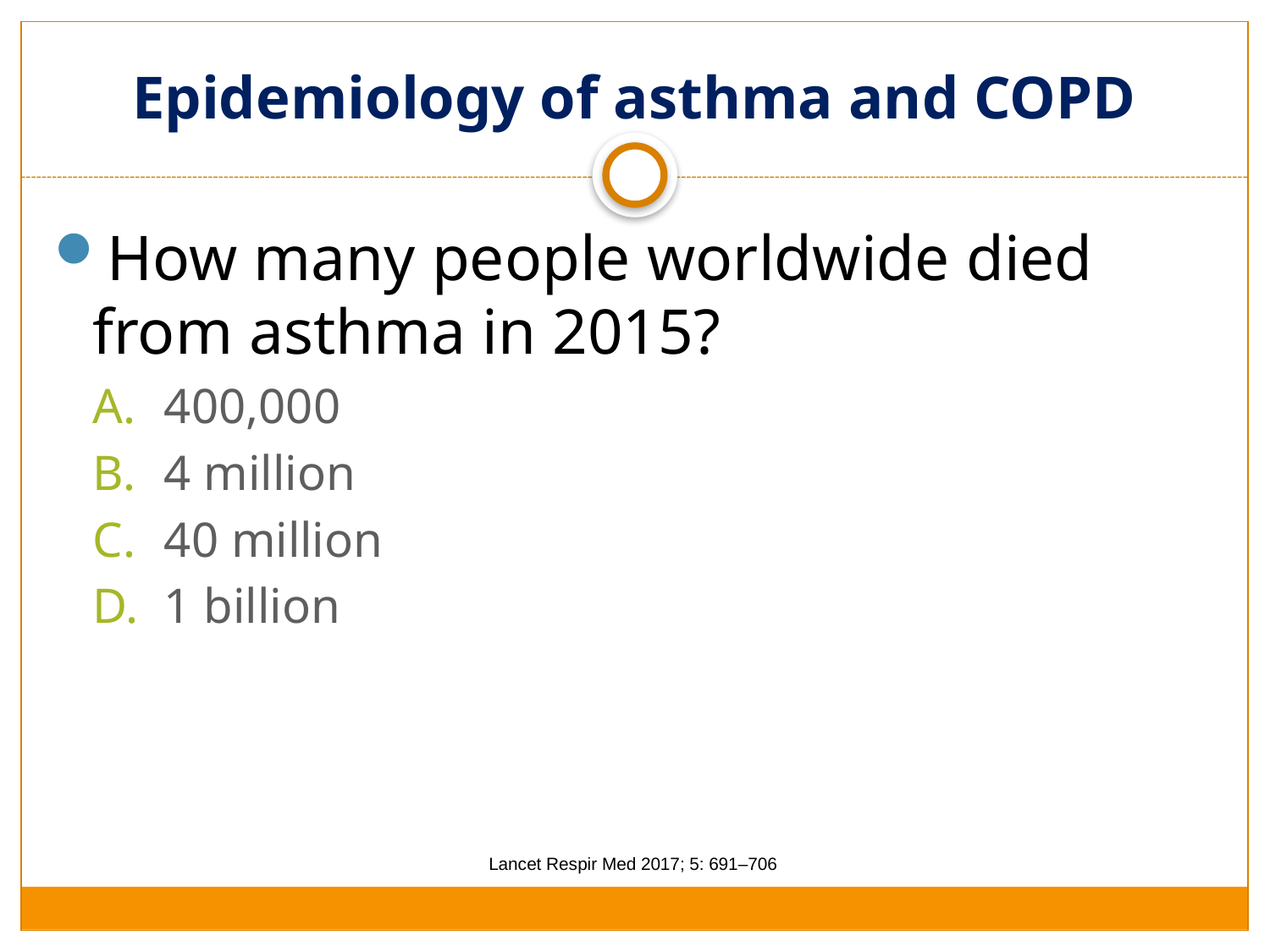

# Epidemiology of asthma and COPD
How many people worldwide died from asthma in 2015?
400,000
4 million
40 million
1 billion
Lancet Respir Med 2017; 5: 691–706

## Slide 40
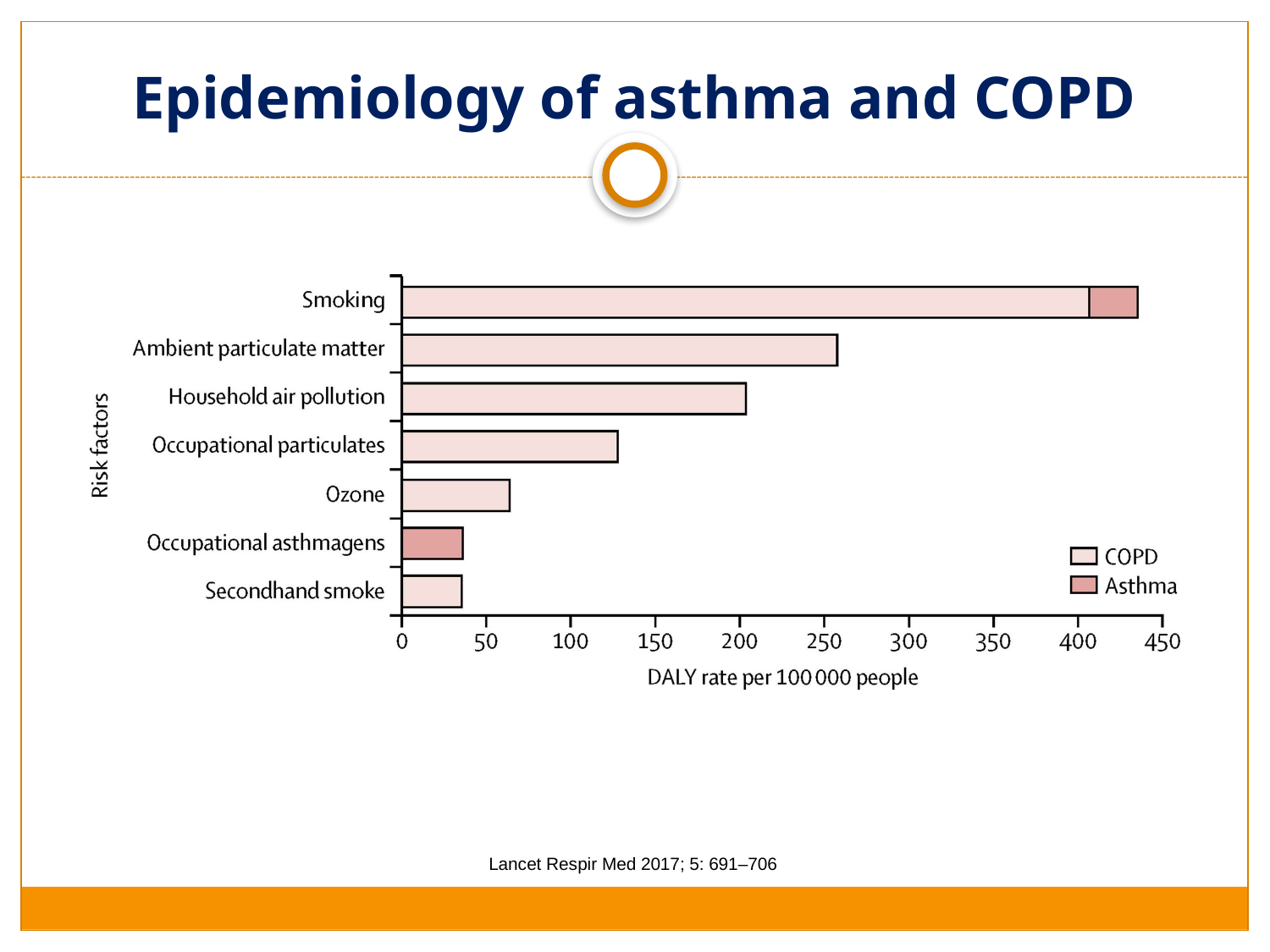

# Epidemiology of asthma and COPD
Lancet Respir Med 2017; 5: 691–706

## Slide 41
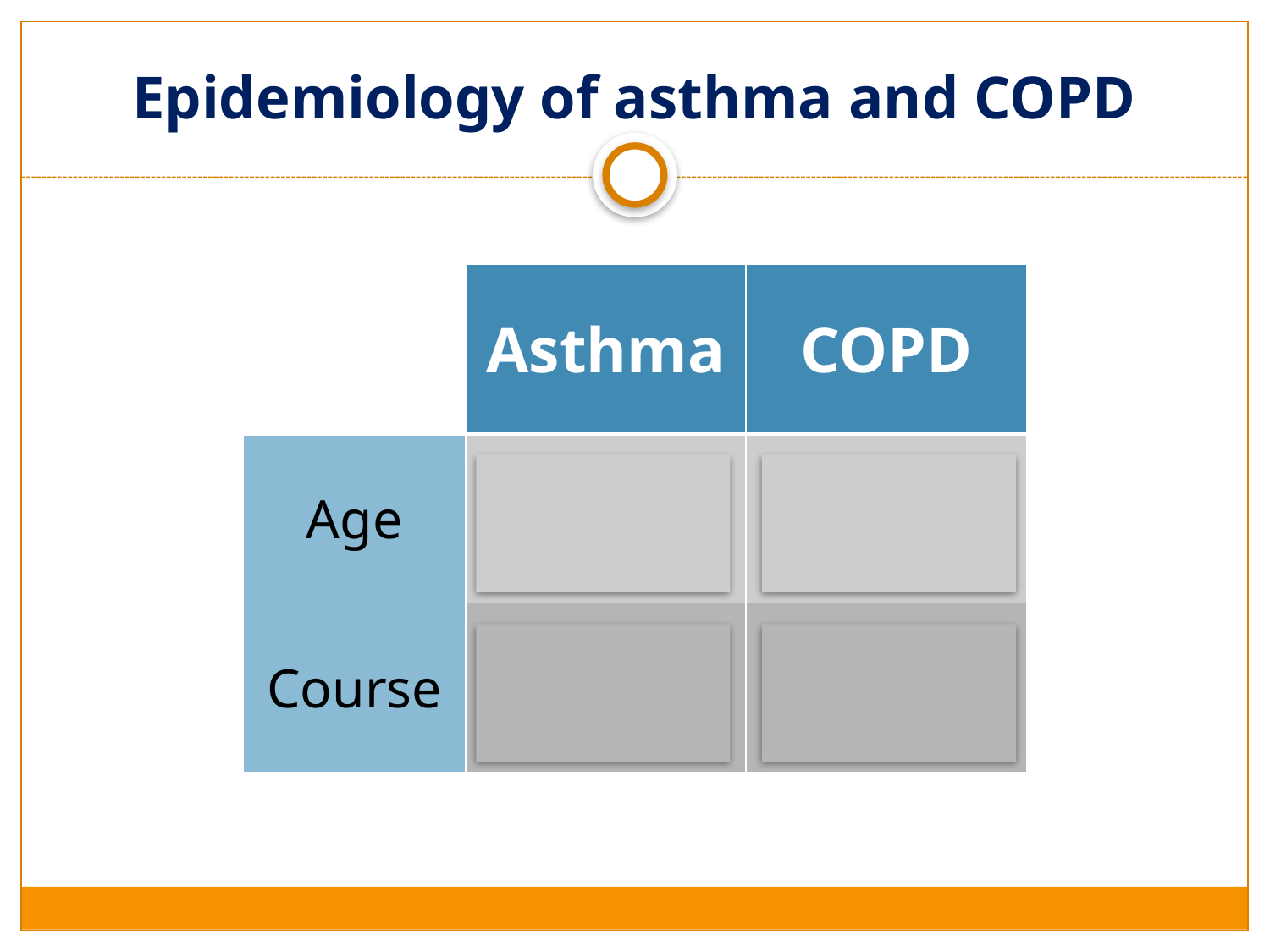

# Epidemiology of asthma and COPD
| | Asthma | COPD |
| --- | --- | --- |
| Age | Can develop at any age | Usually develops later in life |
| Course | Can wax and wane over time | Progressive over time |

## Slide 42
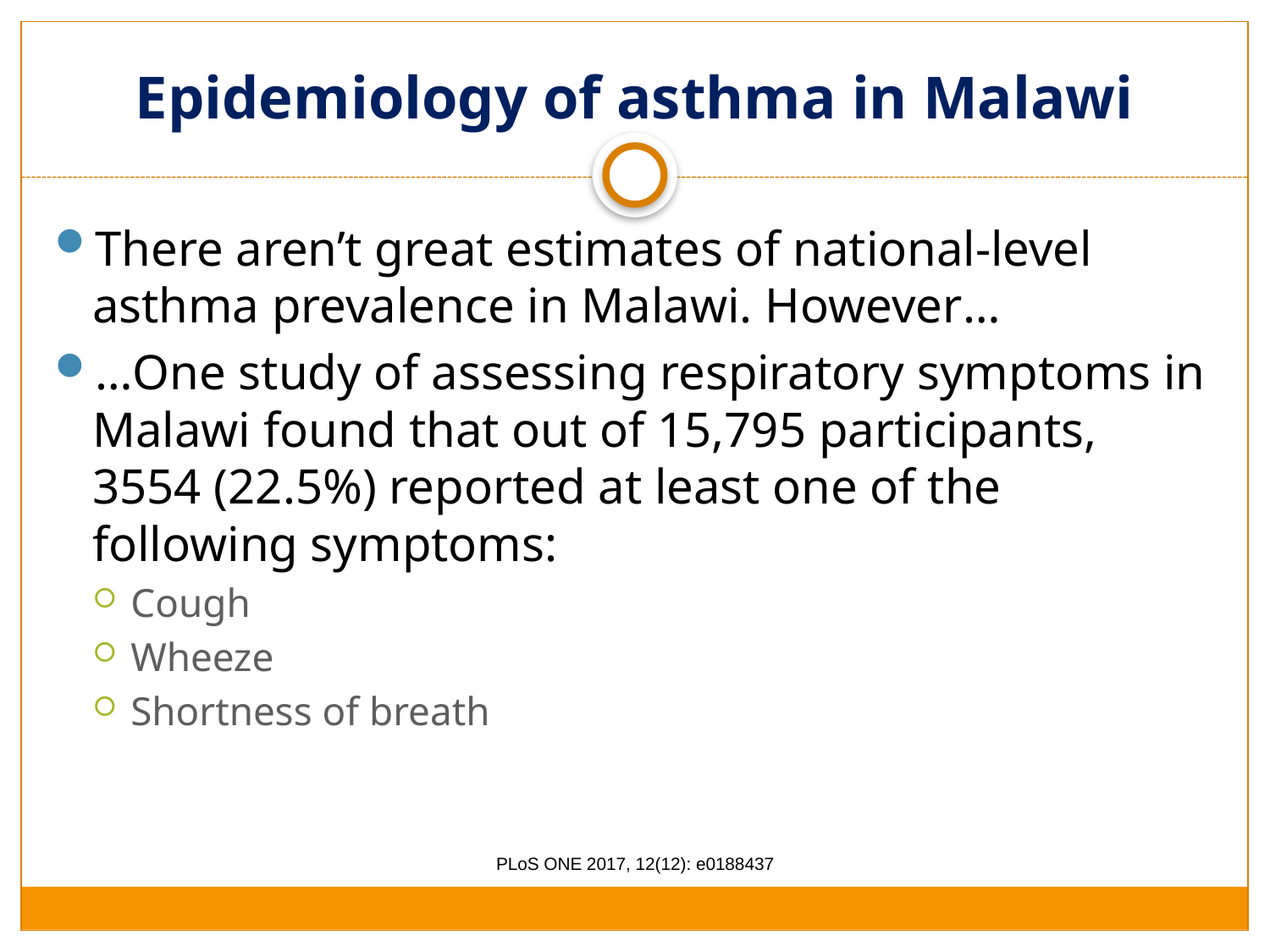

# Epidemiology of asthma in Malawi
There aren’t great estimates of national-level asthma prevalence in Malawi. However…
…One study of assessing respiratory symptoms in Malawi found that out of 15,795 participants, 3554 (22.5%) reported at least one of the following symptoms:
Cough
Wheeze
Shortness of breath
PLoS ONE 2017, 12(12): e0188437

## Slide 43
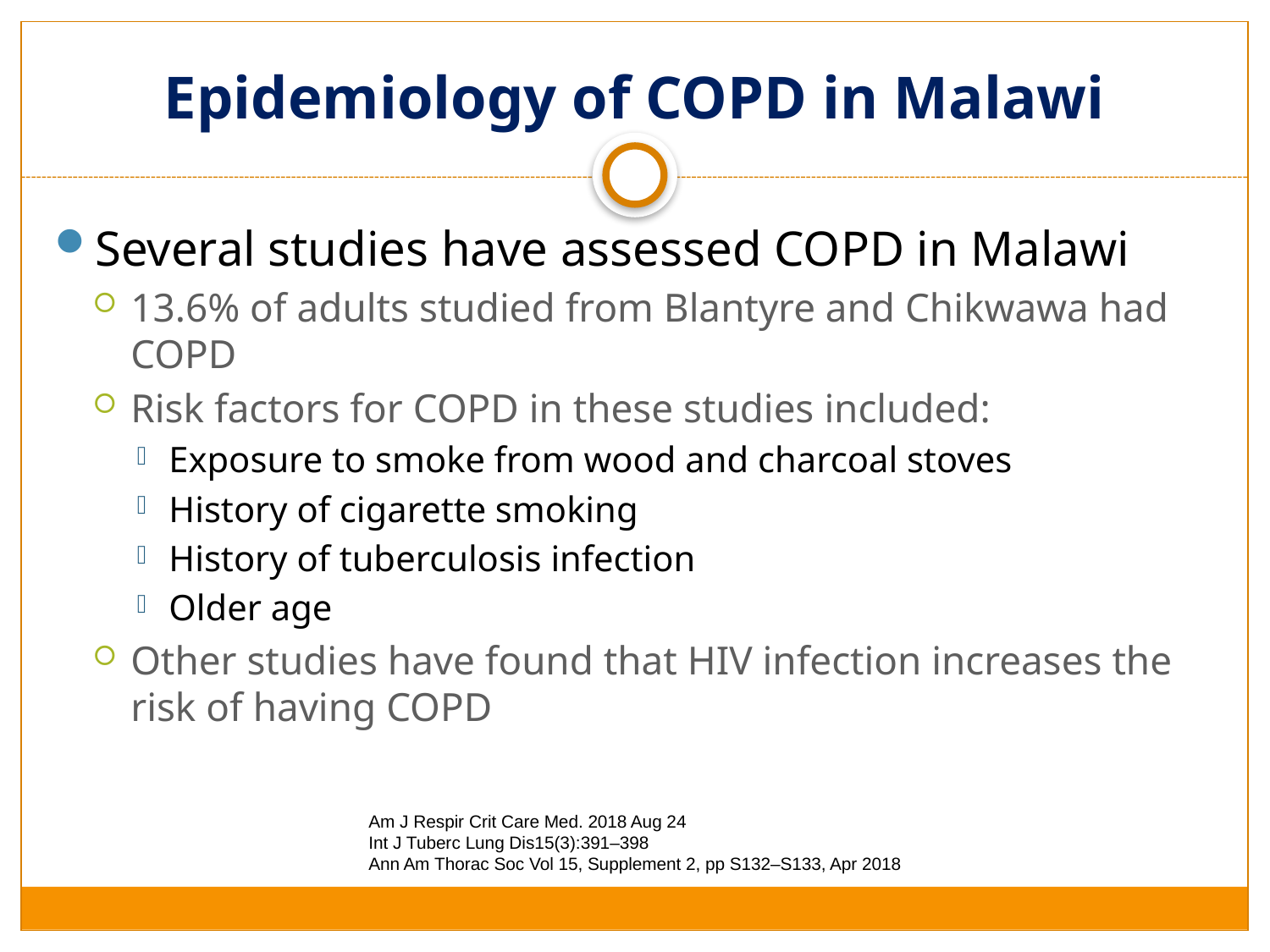

# Epidemiology of COPD in Malawi
Several studies have assessed COPD in Malawi
13.6% of adults studied from Blantyre and Chikwawa had COPD
Risk factors for COPD in these studies included:
Exposure to smoke from wood and charcoal stoves
History of cigarette smoking
History of tuberculosis infection
Older age
Other studies have found that HIV infection increases the risk of having COPD
Am J Respir Crit Care Med. 2018 Aug 24
Int J Tuberc Lung Dis15(3):391–398
Ann Am Thorac Soc Vol 15, Supplement 2, pp S132–S133, Apr 2018

## Slide 44
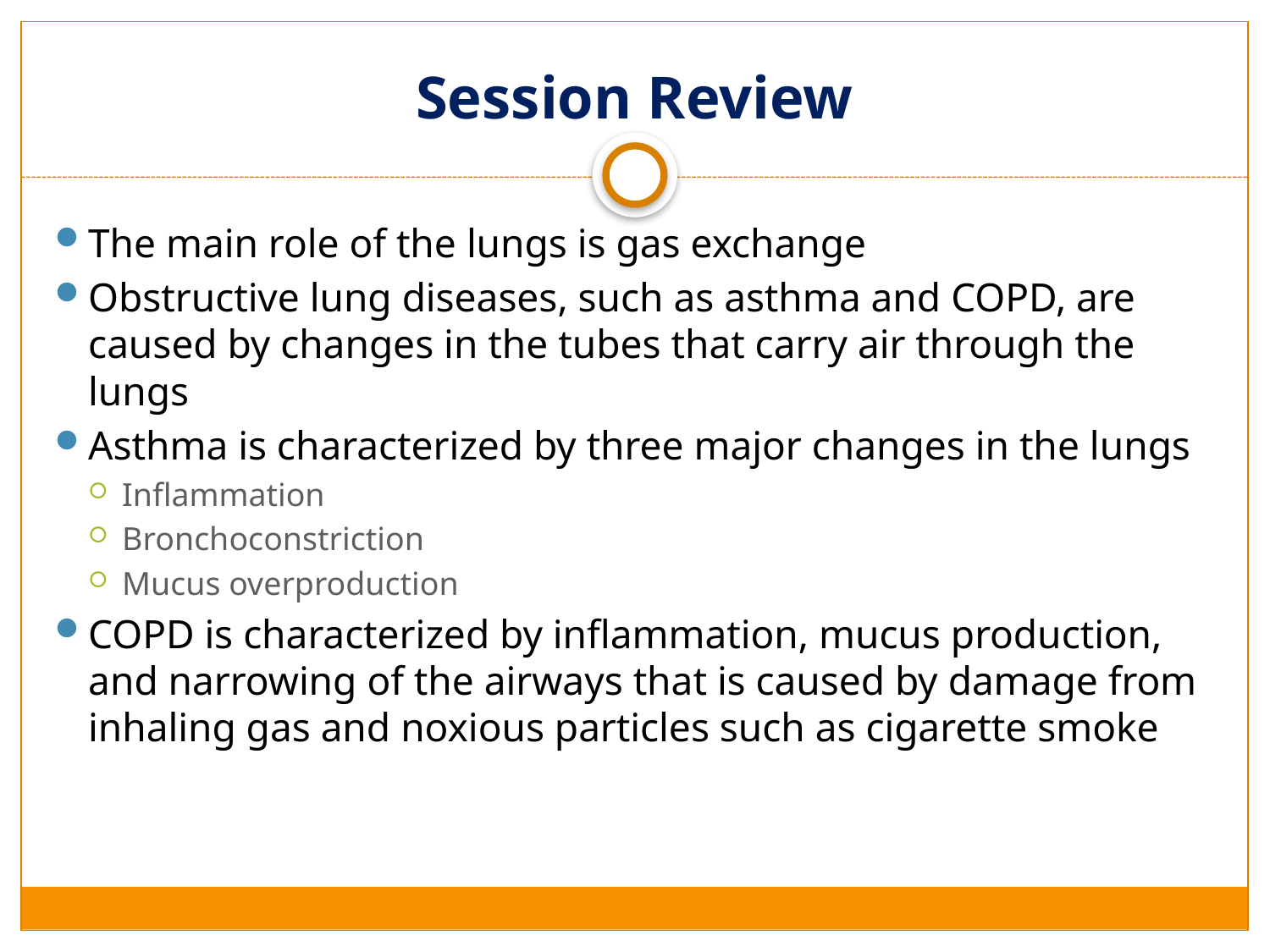

# Session Review
The main role of the lungs is gas exchange
Obstructive lung diseases, such as asthma and COPD, are caused by changes in the tubes that carry air through the lungs
Asthma is characterized by three major changes in the lungs
Inflammation
Bronchoconstriction
Mucus overproduction
COPD is characterized by inflammation, mucus production, and narrowing of the airways that is caused by damage from inhaling gas and noxious particles such as cigarette smoke

## Slide 45
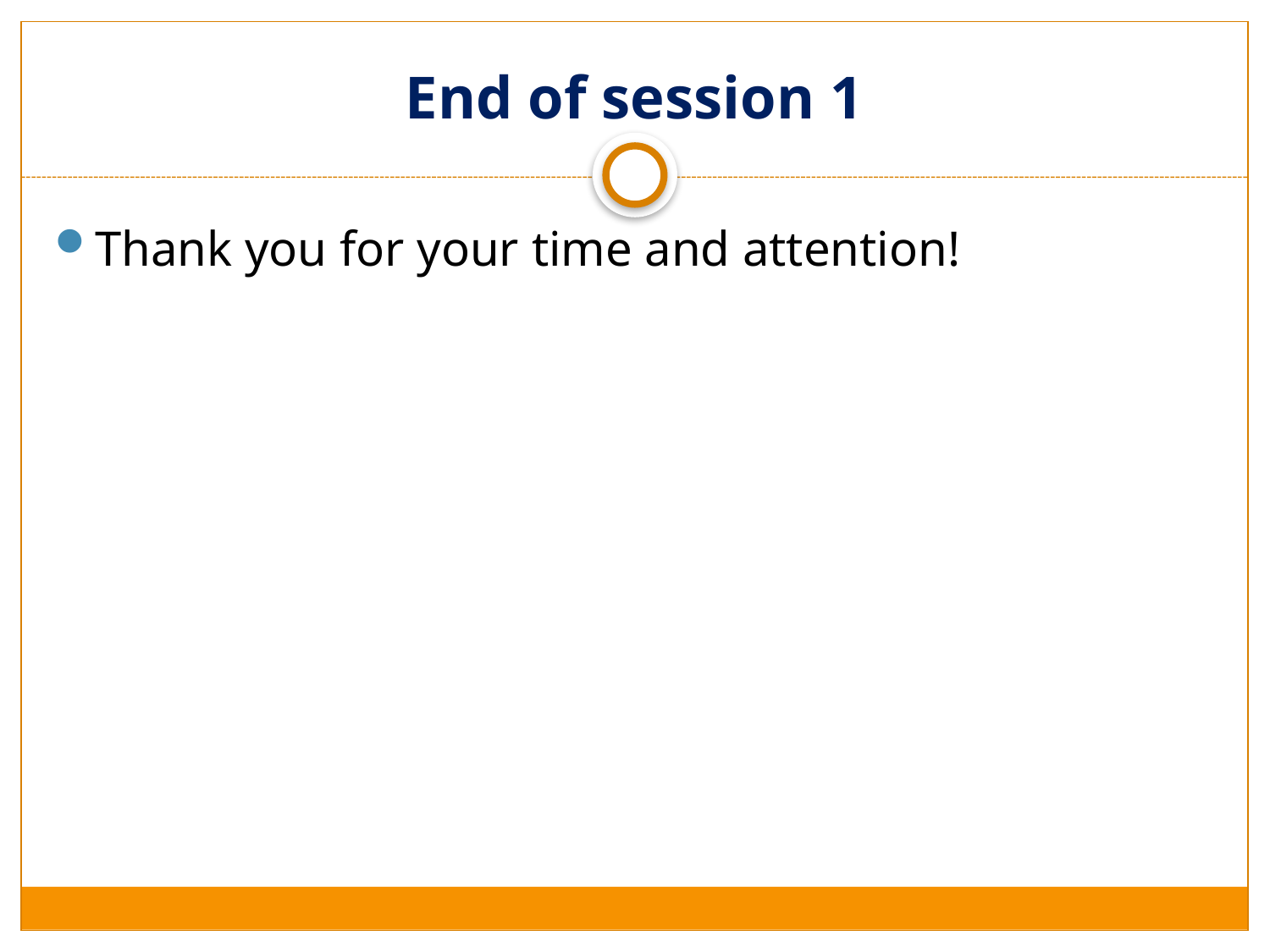

# End of session 1
Thank you for your time and attention!

## Slide 46
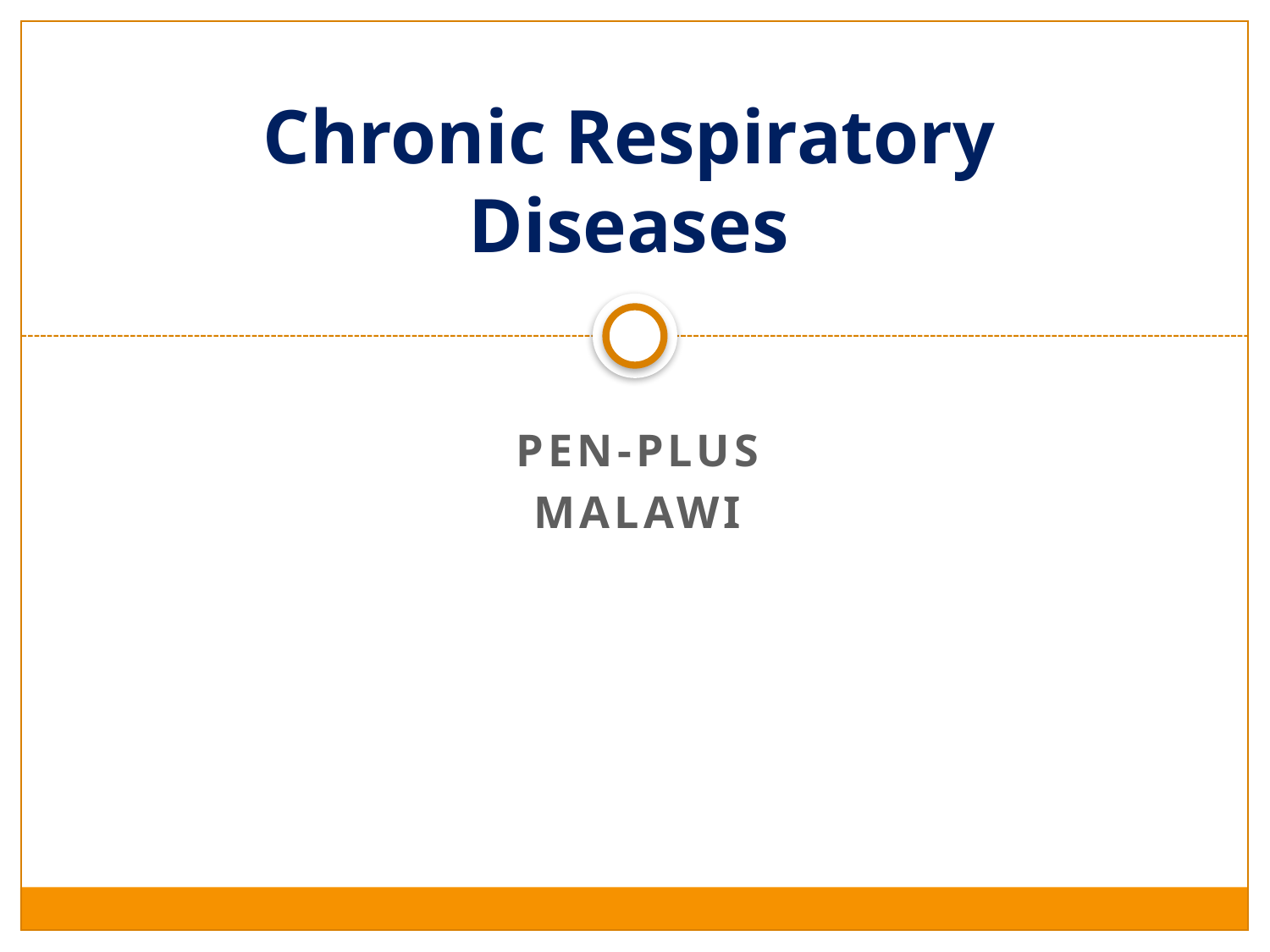

# Chronic Respiratory Diseases
PEN-Plus
Malawi

## Slide 47
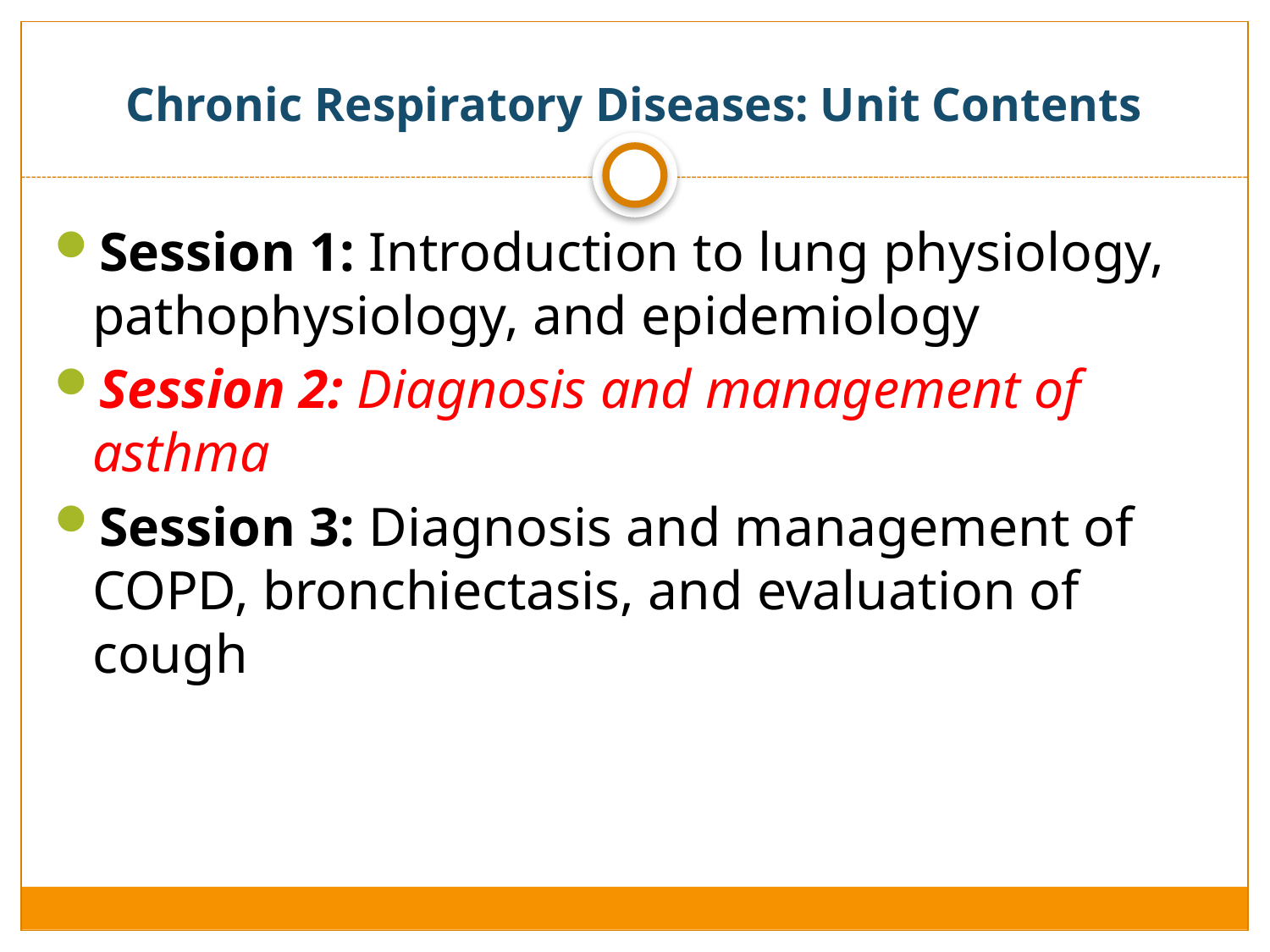

# Chronic Respiratory Diseases: Unit Contents
Session 1: Introduction to lung physiology, pathophysiology, and epidemiology
Session 2: Diagnosis and management of asthma
Session 3: Diagnosis and management of COPD, bronchiectasis, and evaluation of cough

## Slide 48
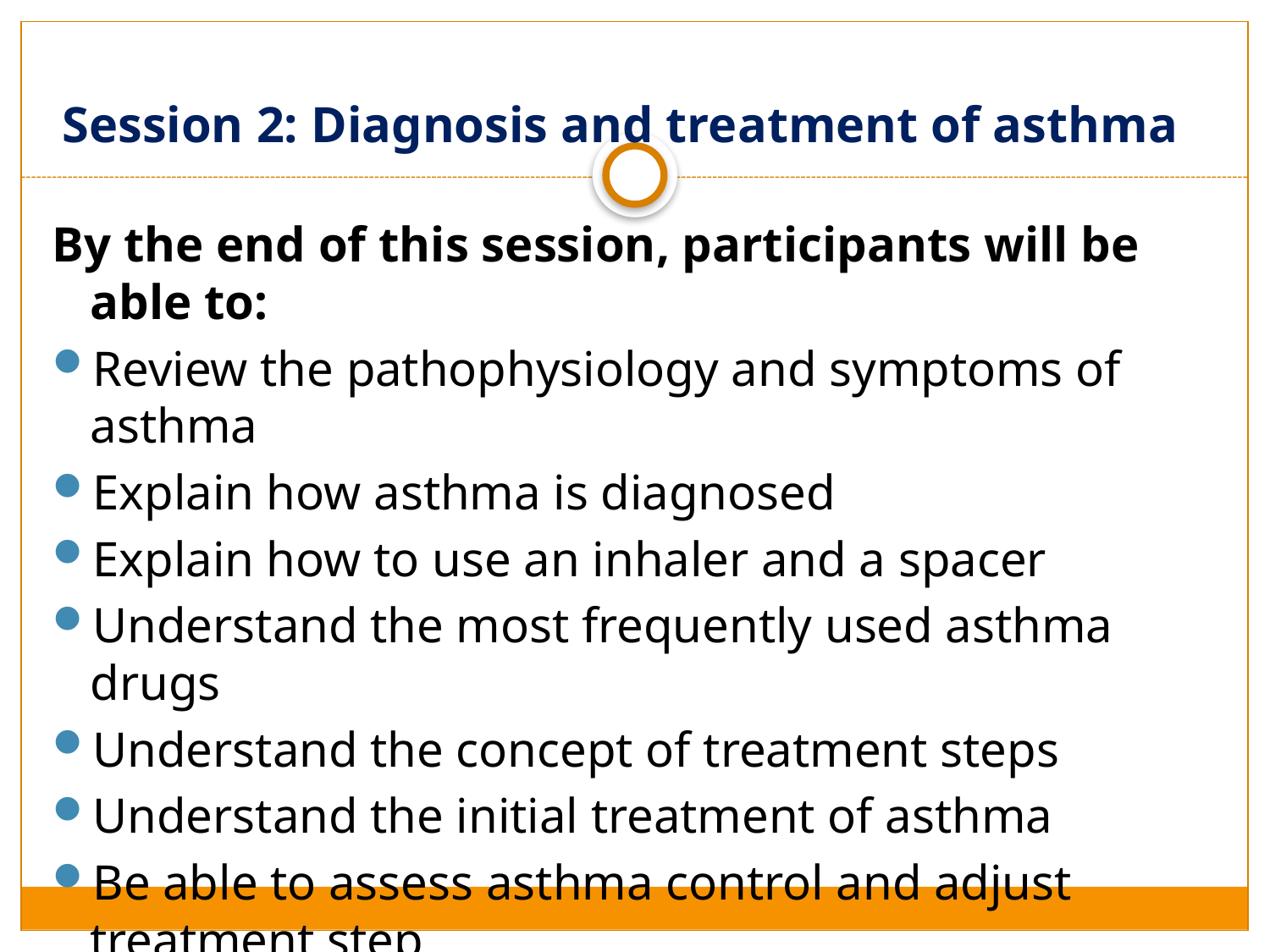

# Session 2: Diagnosis and treatment of asthma
By the end of this session, participants will be able to:
Review the pathophysiology and symptoms of asthma
Explain how asthma is diagnosed
Explain how to use an inhaler and a spacer
Understand the most frequently used asthma drugs
Understand the concept of treatment steps
Understand the initial treatment of asthma
Be able to assess asthma control and adjust treatment step
Be able to assess asthma attacks

## Slide 49
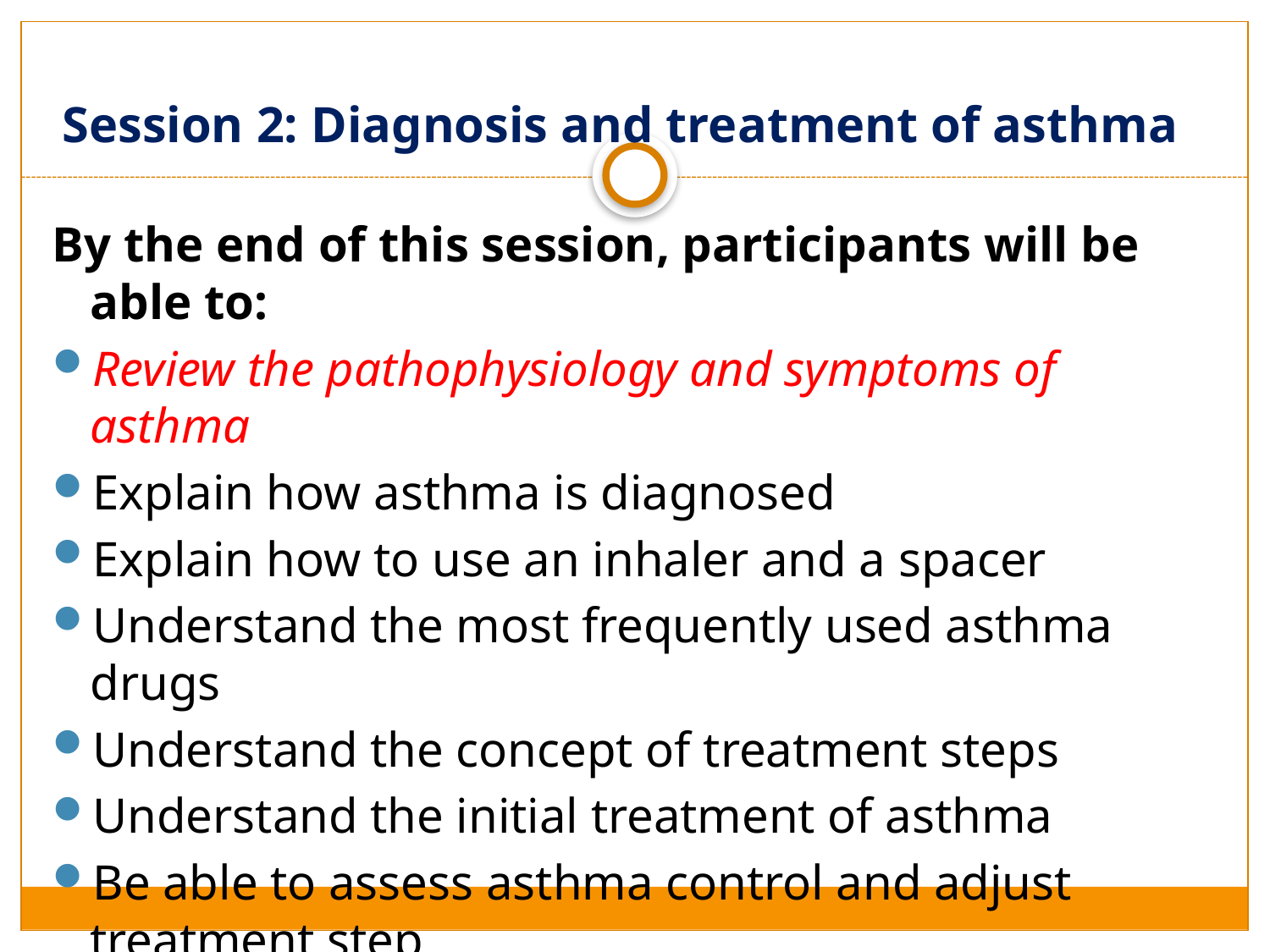

# Session 2: Diagnosis and treatment of asthma
By the end of this session, participants will be able to:
Review the pathophysiology and symptoms of asthma
Explain how asthma is diagnosed
Explain how to use an inhaler and a spacer
Understand the most frequently used asthma drugs
Understand the concept of treatment steps
Understand the initial treatment of asthma
Be able to assess asthma control and adjust treatment step
Be able to assess asthma attacks

## Slide 50
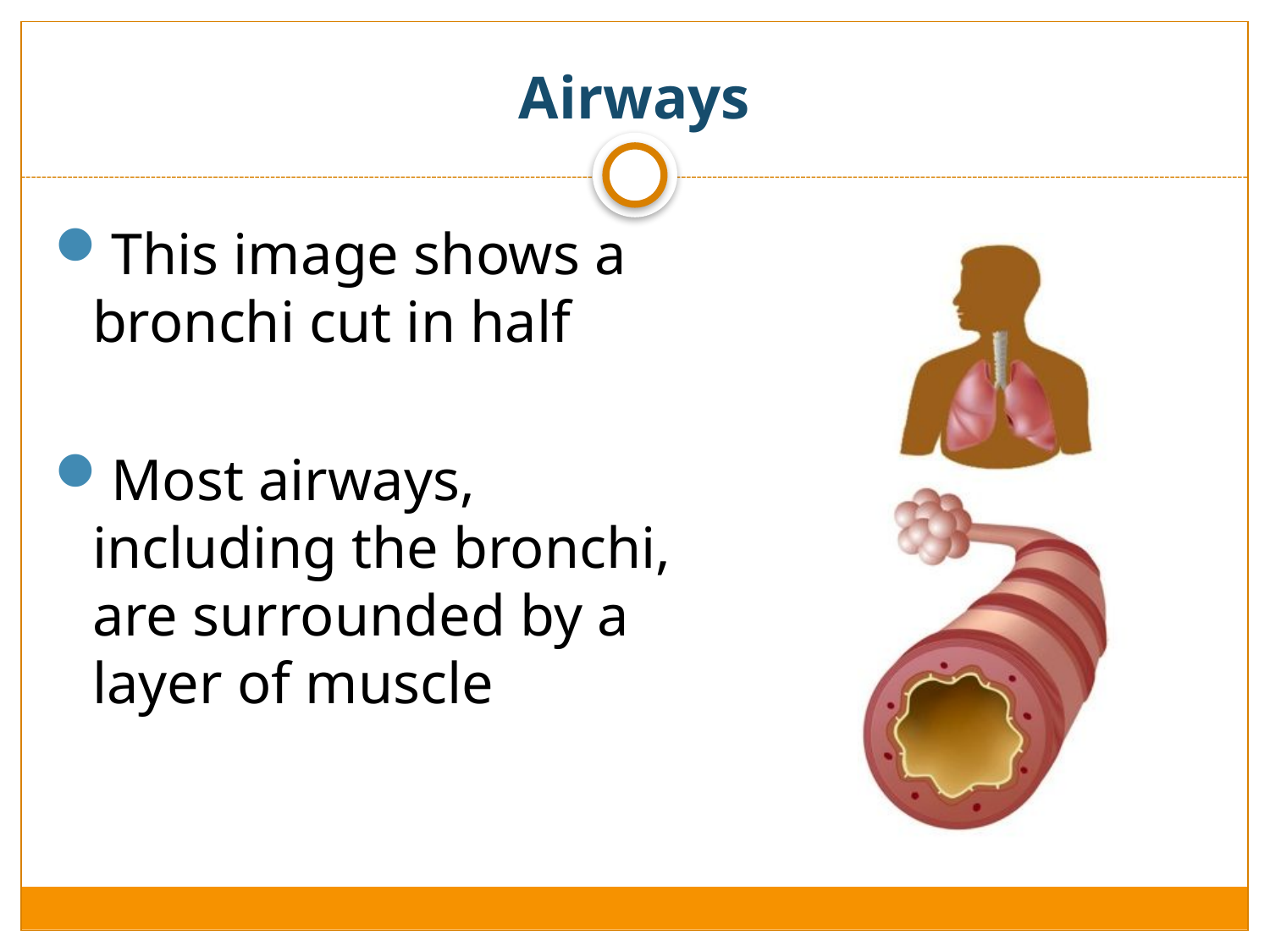

# Airways
This image shows a bronchi cut in half
Most airways, including the bronchi, are surrounded by a layer of muscle

## Slide 51
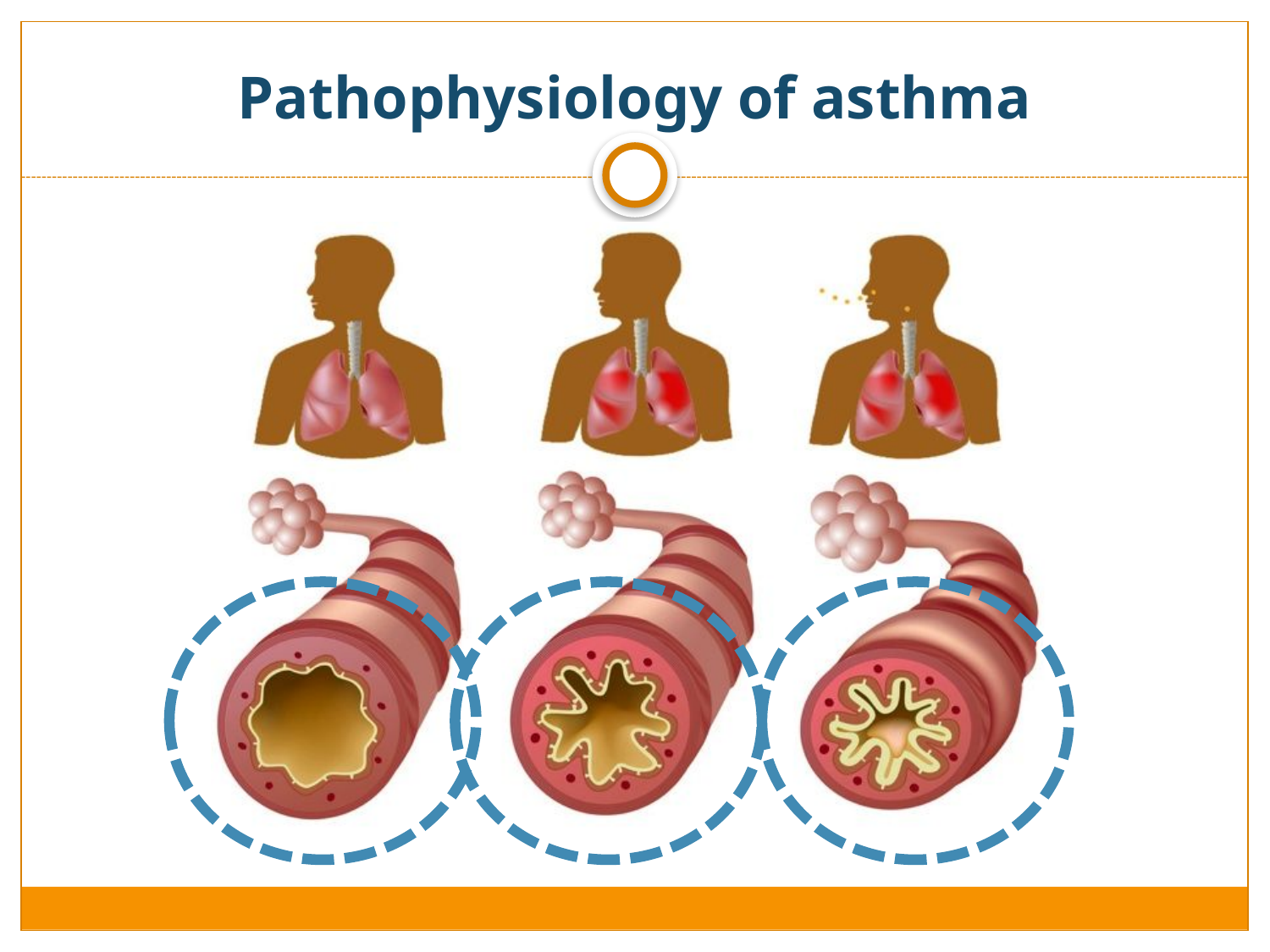

# Pathophysiology of asthma

## Slide 52
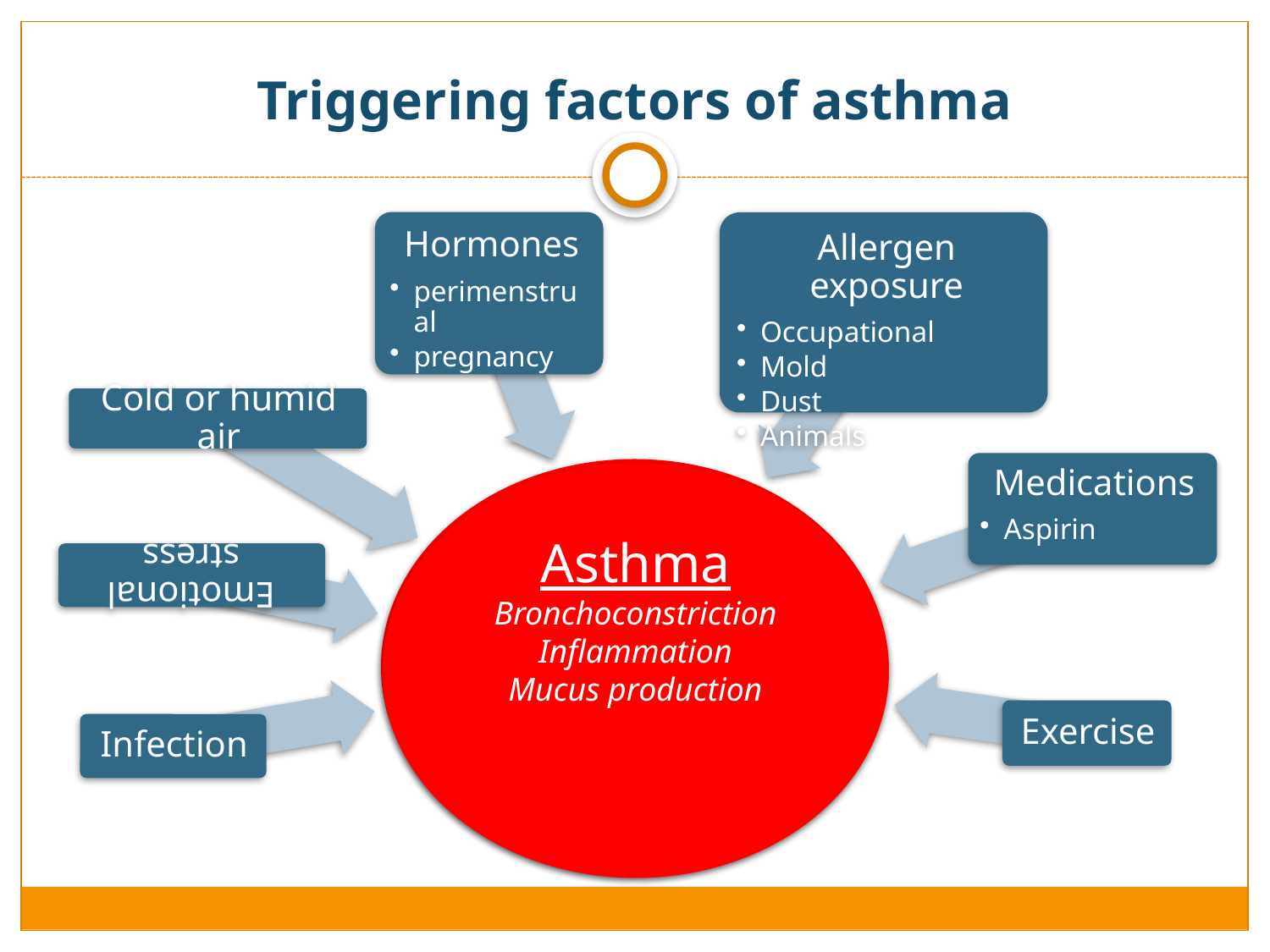

# Triggering factors of asthma
Asthma
Bronchoconstriction
Inflammation
Mucus production

## Slide 53
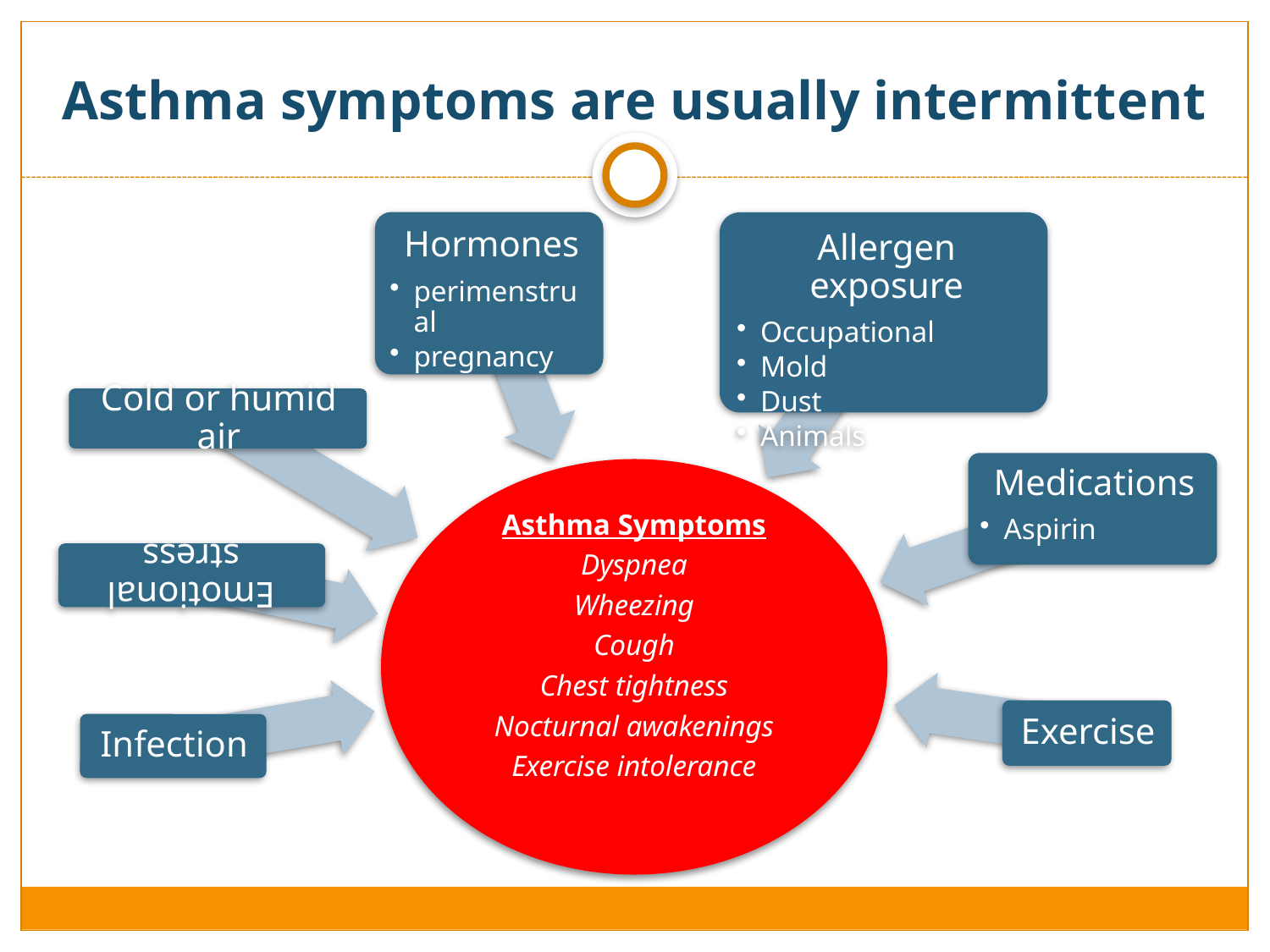

# Asthma symptoms are usually intermittent

## Slide 54
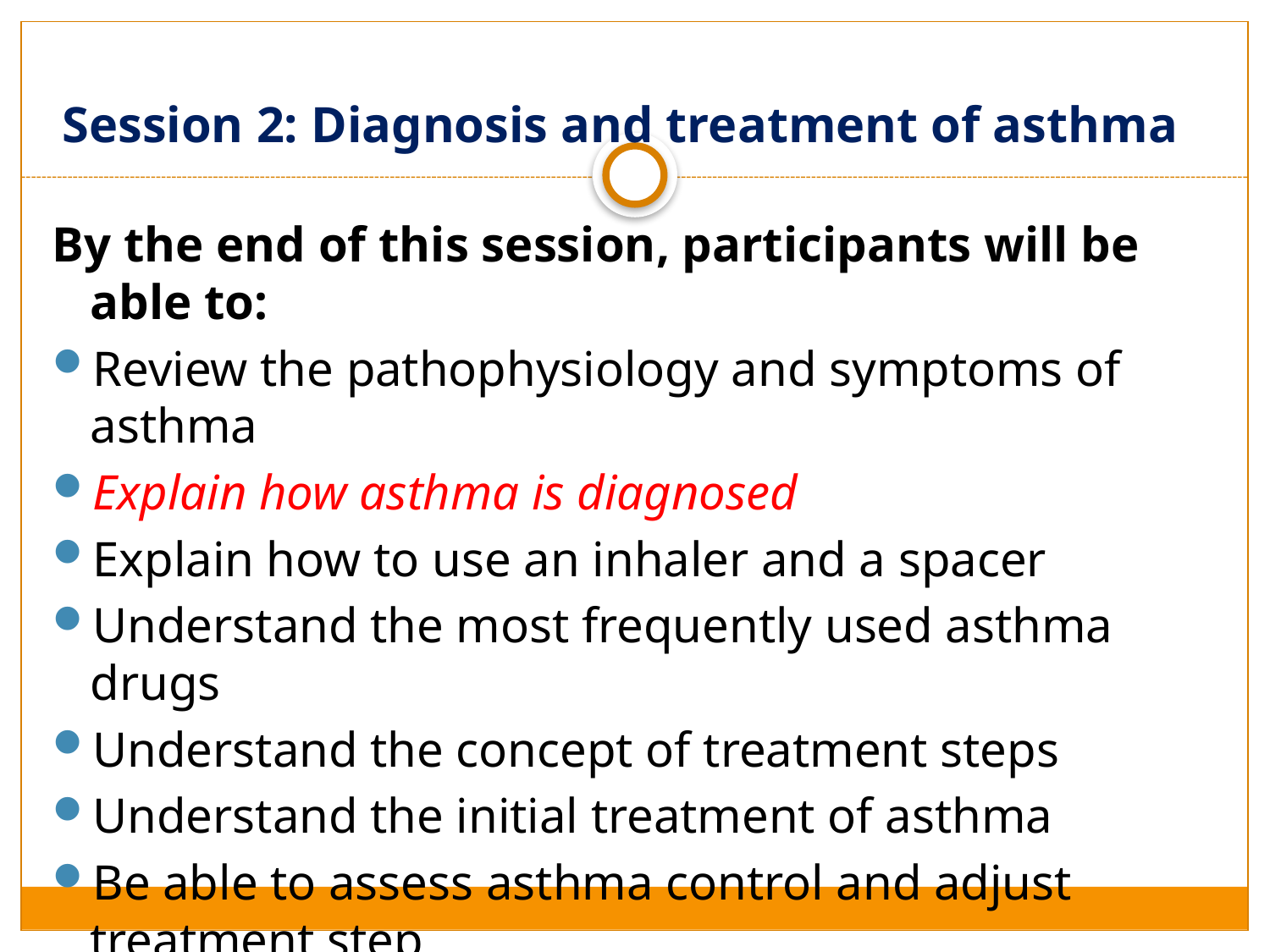

# Session 2: Diagnosis and treatment of asthma
By the end of this session, participants will be able to:
Review the pathophysiology and symptoms of asthma
Explain how asthma is diagnosed
Explain how to use an inhaler and a spacer
Understand the most frequently used asthma drugs
Understand the concept of treatment steps
Understand the initial treatment of asthma
Be able to assess asthma control and adjust treatment step
Be able to assess asthma attacks

## Slide 55
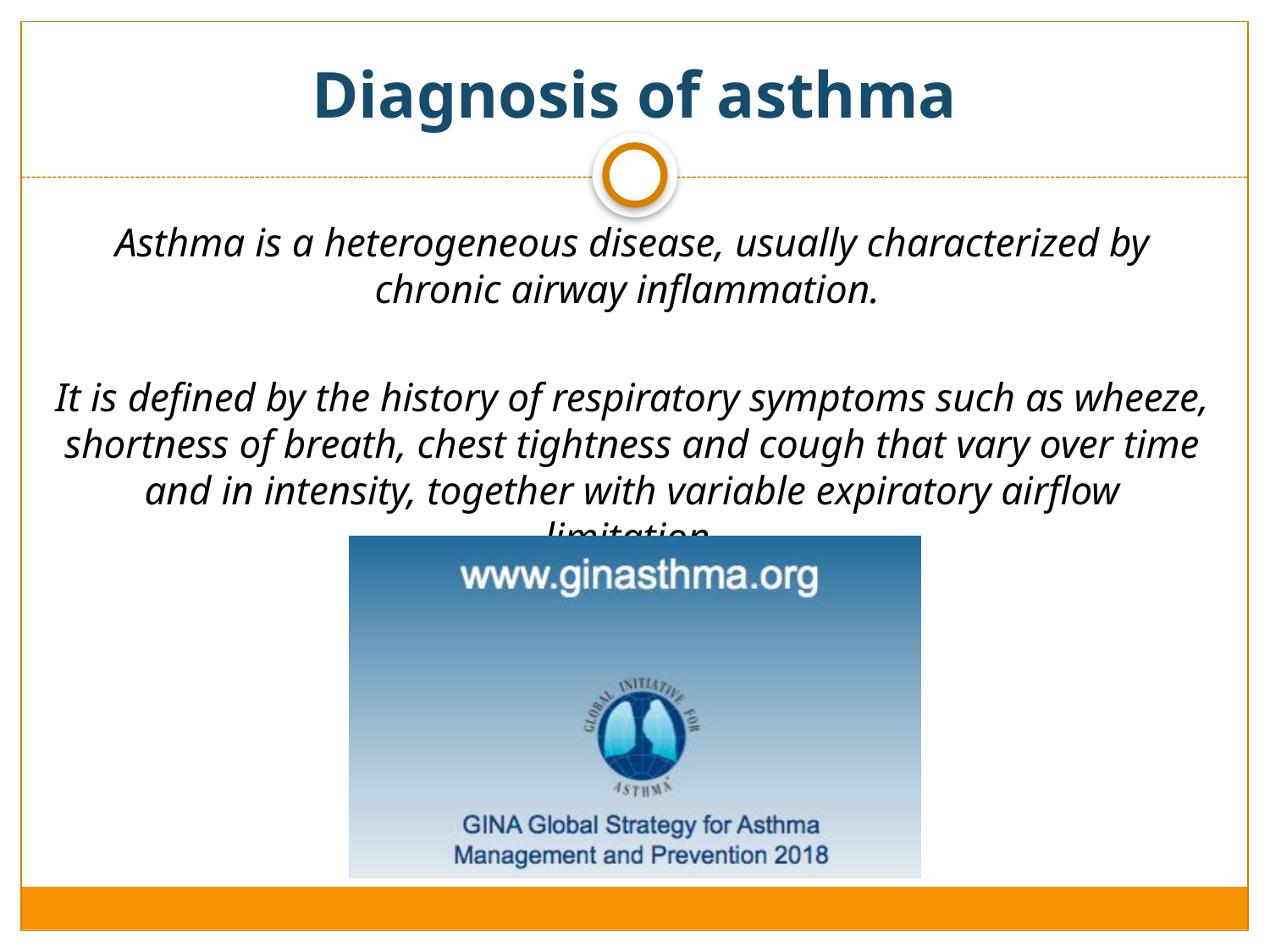

# Diagnosis of asthma
Asthma is a heterogeneous disease, usually characterized by chronic airway inflammation.
It is defined by the history of respiratory symptoms such as wheeze, shortness of breath, chest tightness and cough that vary over time and in intensity, together with variable expiratory airflow limitation.

## Slide 56
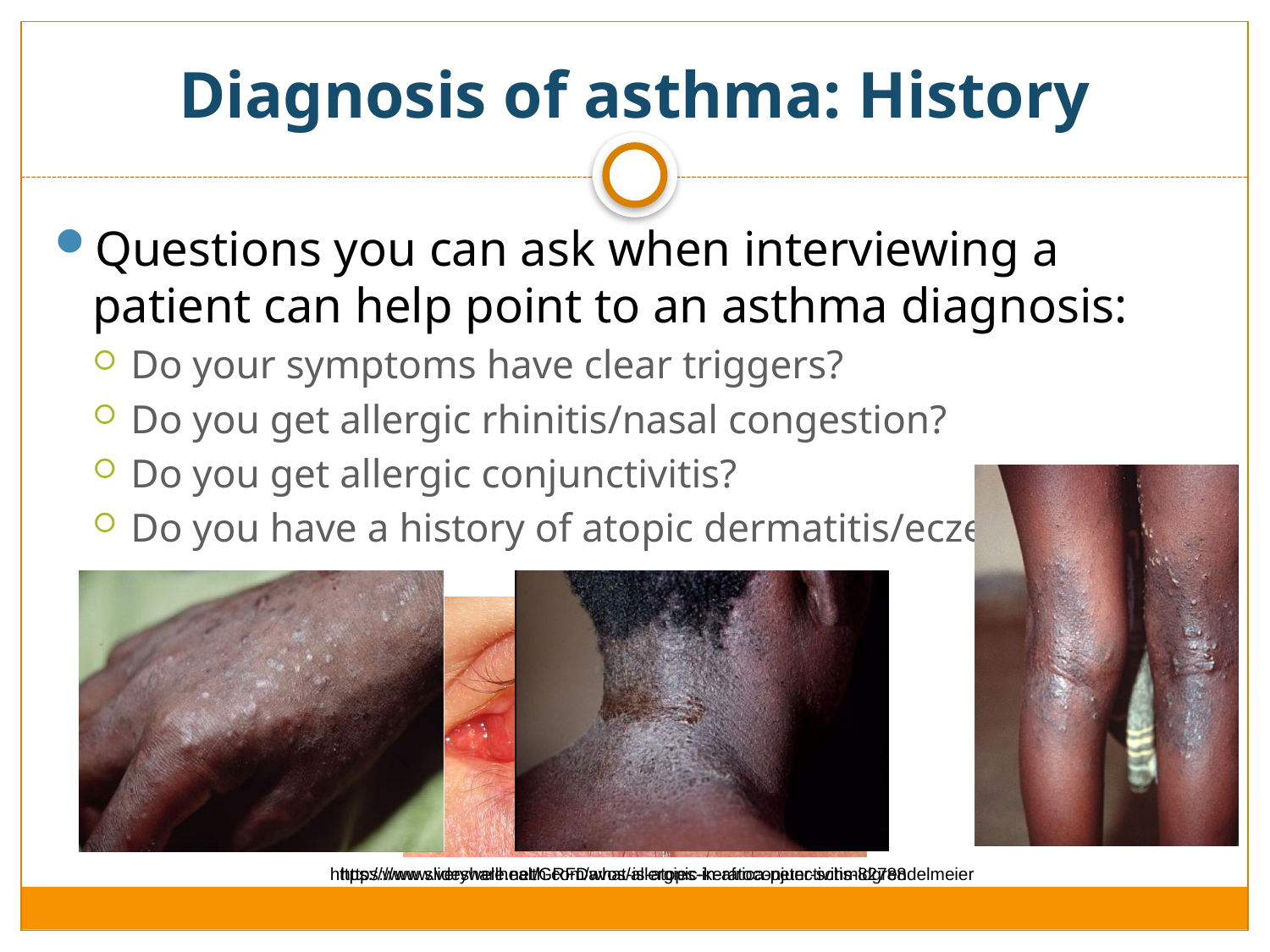

# Diagnosis of asthma: History
Questions you can ask when interviewing a patient can help point to an asthma diagnosis:
Do your symptoms have clear triggers?
Do you get allergic rhinitis/nasal congestion?
Do you get allergic conjunctivitis?
Do you have a history of atopic dermatitis/eczema?
https://www.slideshare.net/GRFDavos/allergies-in-africa-peter-schmidgrendelmeier
https://www.verywellhealth.com/what-is-atopic-keratoconjunctivitis-82788

## Slide 57
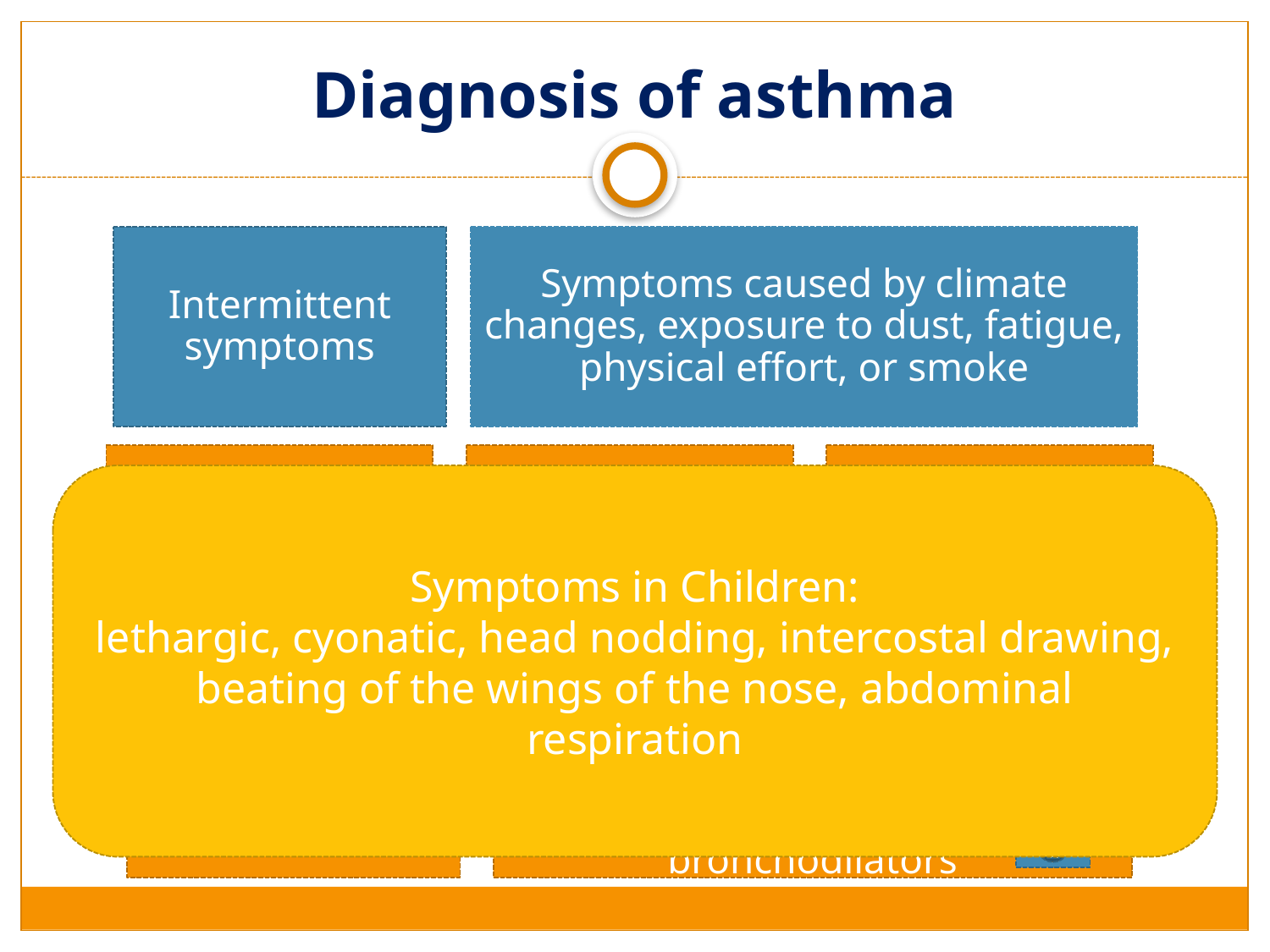

# Diagnosis of asthma
Intermittent symptoms
Symptoms caused by climate changes, exposure to dust, fatigue, physical effort, or smoke
Dyspnea
Wheezing
Dry cough, especially at night
Symptoms in Children:
lethargic, cyonatic, head nodding, intercostal drawing, beating of the wings of the nose, abdominal respiration
Chest tightness
Lowered Peak Expiratory Flow (PEF) in the presence of symptoms and with a 20% improvement under bronchodilators

## Slide 58
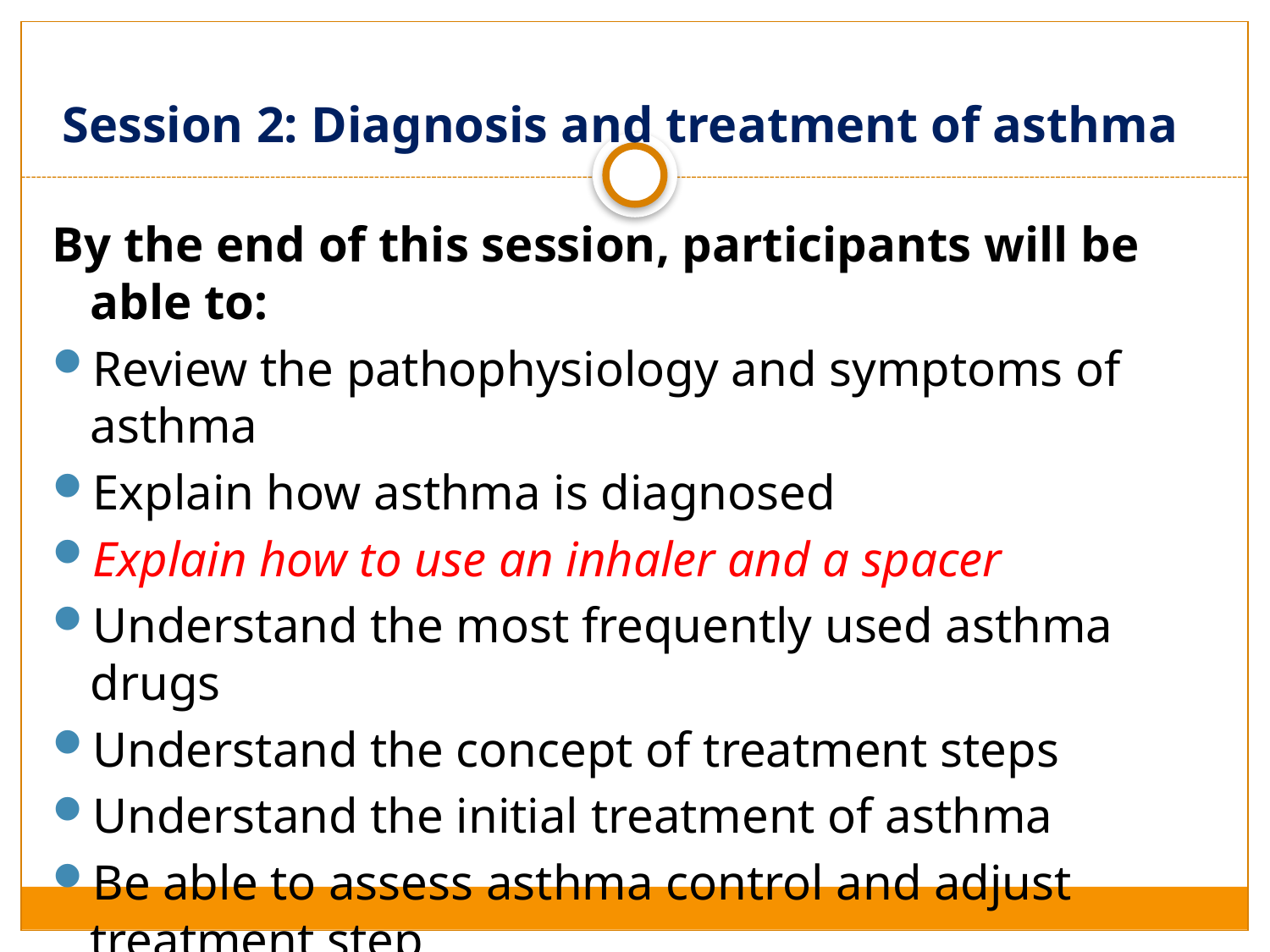

# Session 2: Diagnosis and treatment of asthma
By the end of this session, participants will be able to:
Review the pathophysiology and symptoms of asthma
Explain how asthma is diagnosed
Explain how to use an inhaler and a spacer
Understand the most frequently used asthma drugs
Understand the concept of treatment steps
Understand the initial treatment of asthma
Be able to assess asthma control and adjust treatment step
Be able to assess asthma attacks

## Slide 59
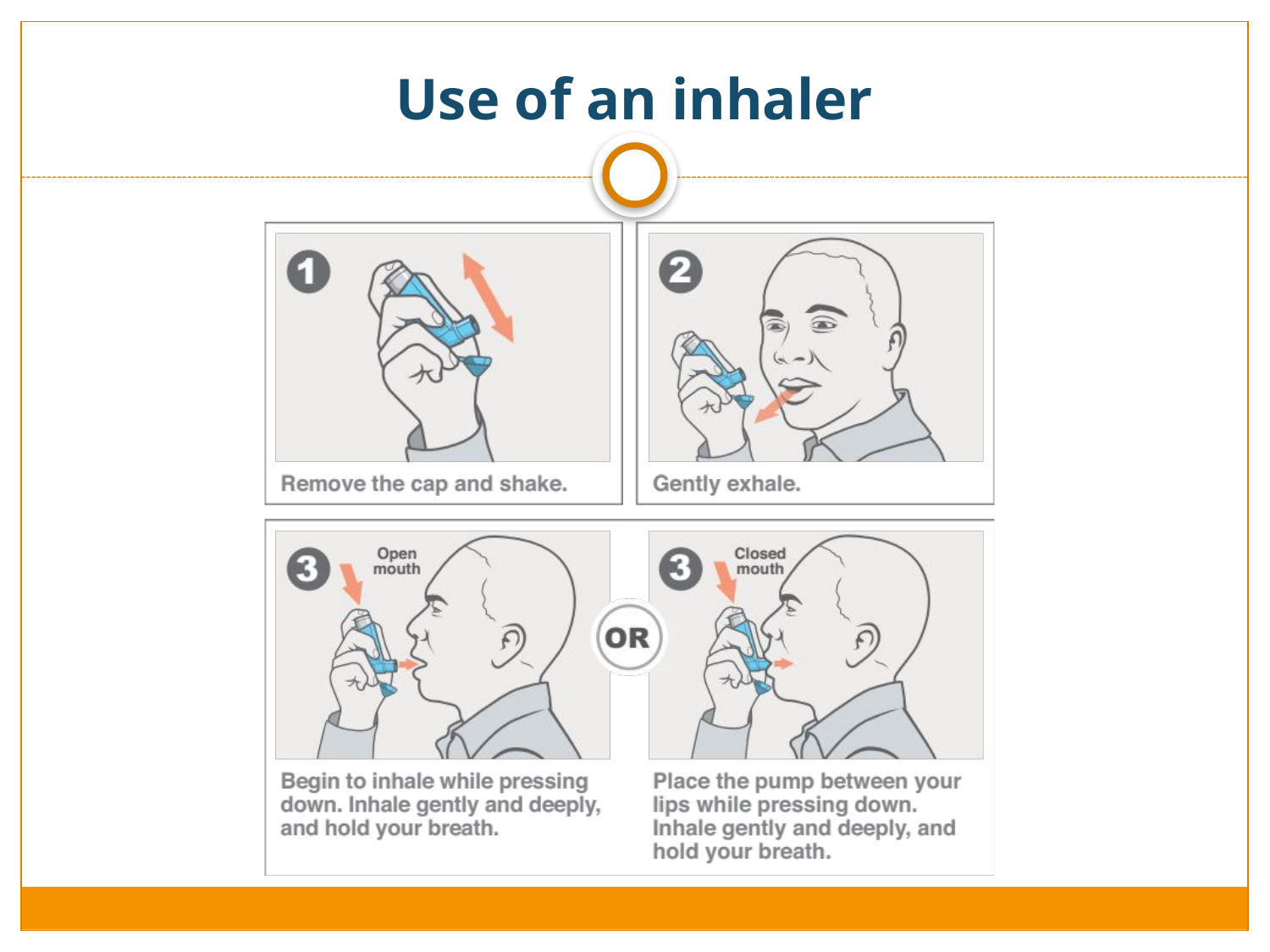

# Use of an inhaler

## Slide 60
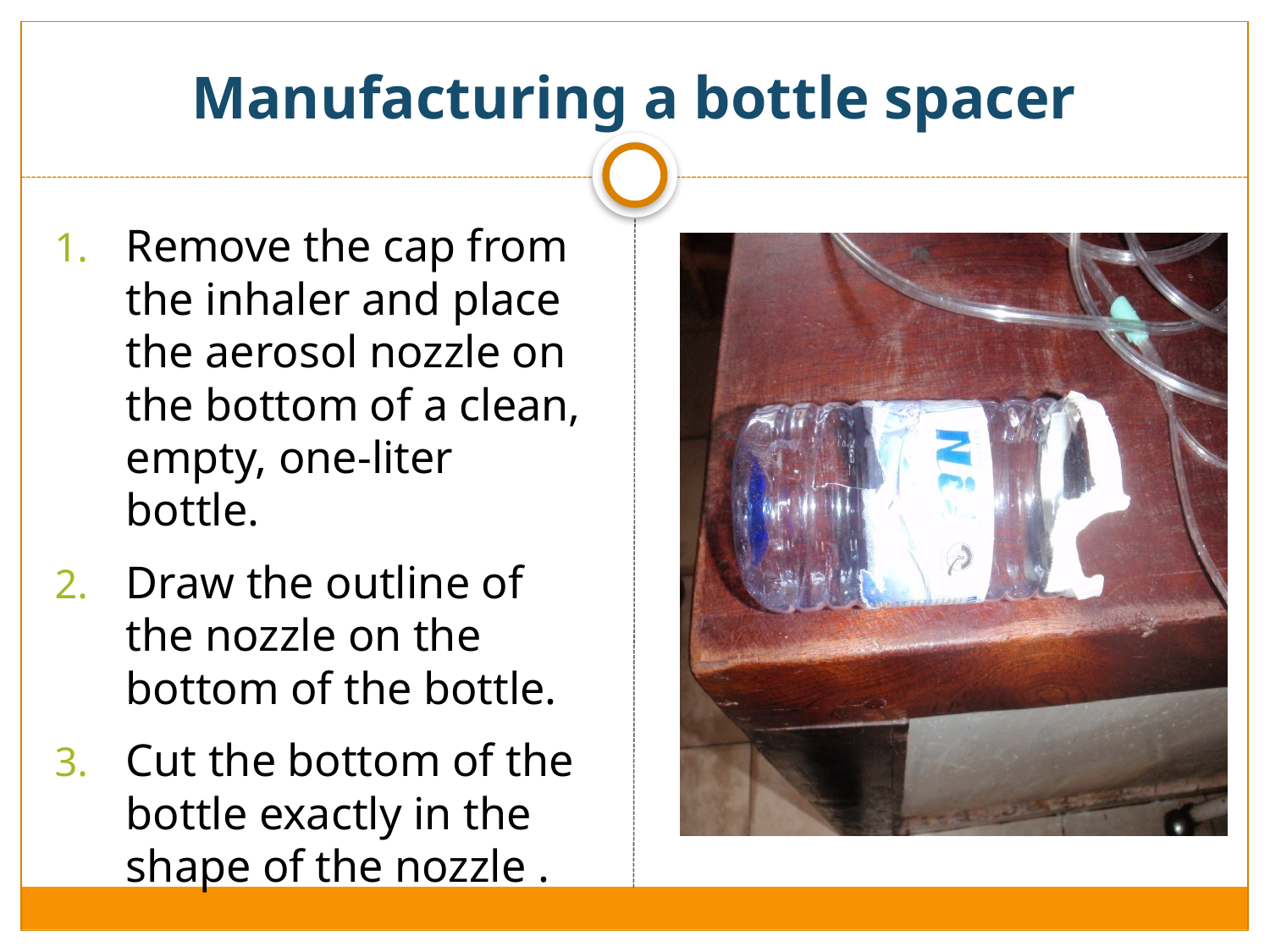

# Manufacturing a bottle spacer
Remove the cap from the inhaler and place the aerosol nozzle on the bottom of a clean, empty, one-liter bottle.
Draw the outline of the nozzle on the bottom of the bottle.
Cut the bottom of the bottle exactly in the shape of the nozzle .

## Slide 61
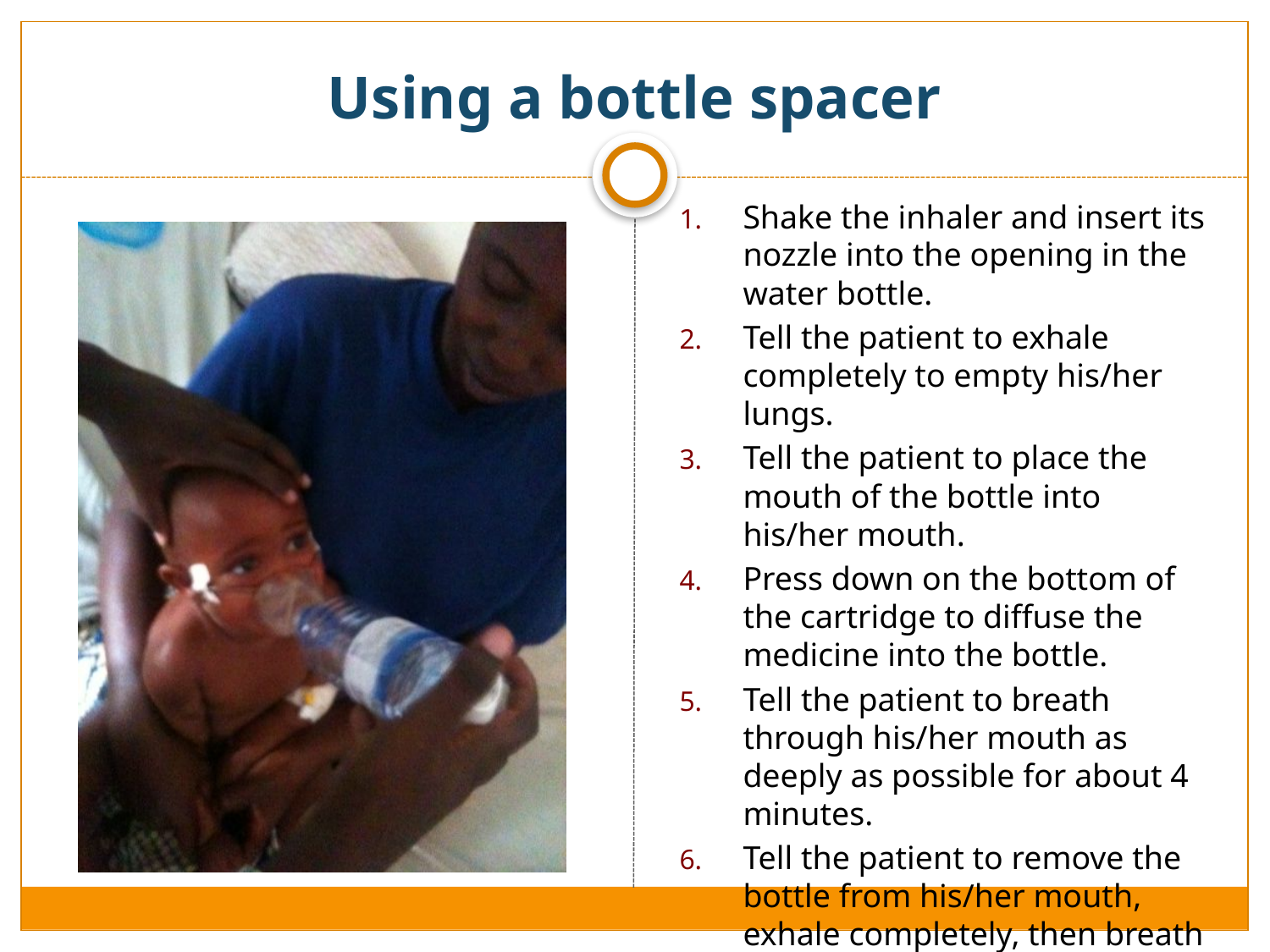

# Using a bottle spacer
Shake the inhaler and insert its nozzle into the opening in the water bottle.
Tell the patient to exhale completely to empty his/her lungs.
Tell the patient to place the mouth of the bottle into his/her mouth.
Press down on the bottom of the cartridge to diffuse the medicine into the bottle.
Tell the patient to breath through his/her mouth as deeply as possible for about 4 minutes.
Tell the patient to remove the bottle from his/her mouth, exhale completely, then breath normally.

## Slide 62
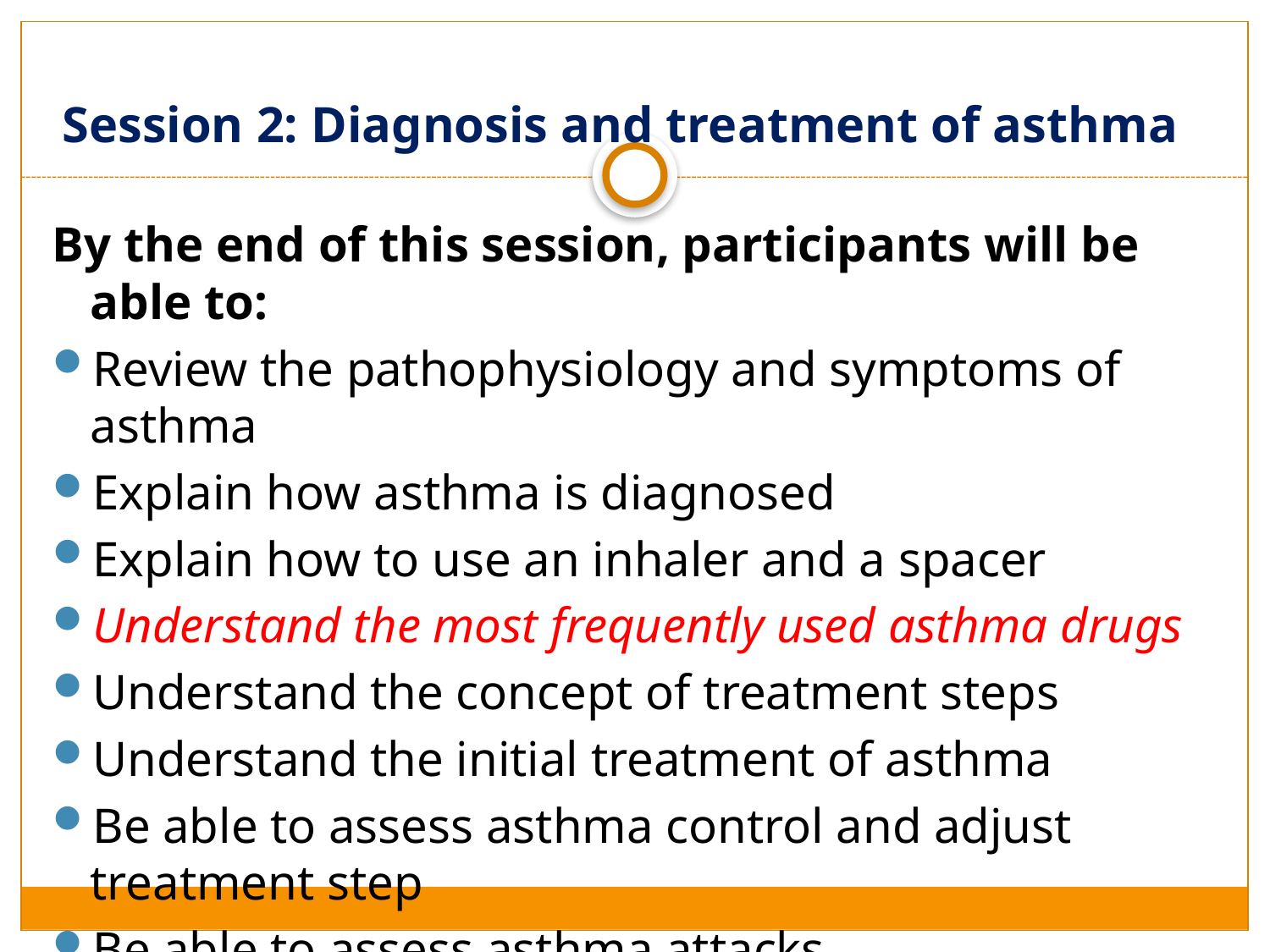

# Session 2: Diagnosis and treatment of asthma
By the end of this session, participants will be able to:
Review the pathophysiology and symptoms of asthma
Explain how asthma is diagnosed
Explain how to use an inhaler and a spacer
Understand the most frequently used asthma drugs
Understand the concept of treatment steps
Understand the initial treatment of asthma
Be able to assess asthma control and adjust treatment step
Be able to assess asthma attacks

## Slide 63
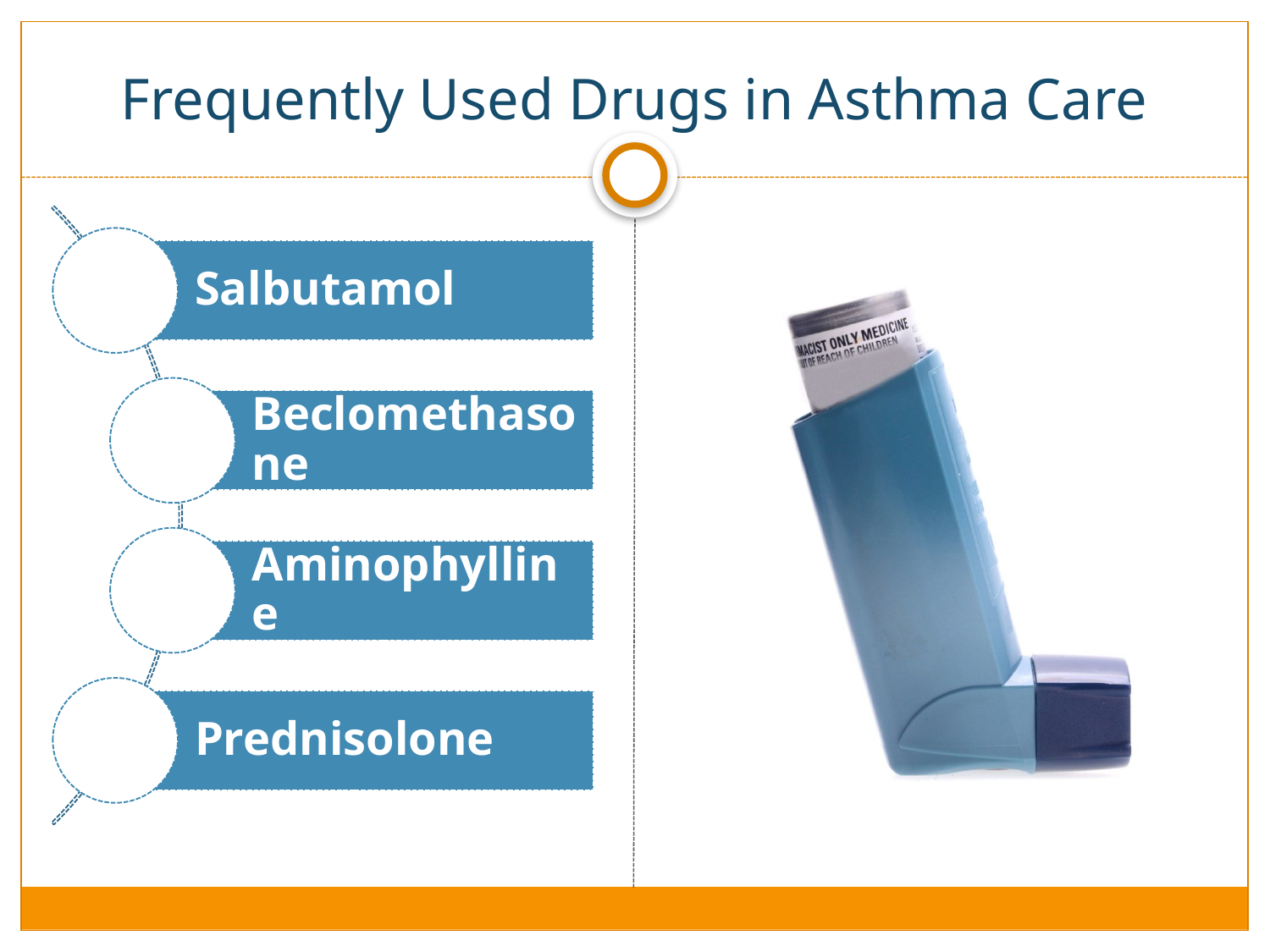

# Frequently Used Drugs in Asthma Care

## Slide 64
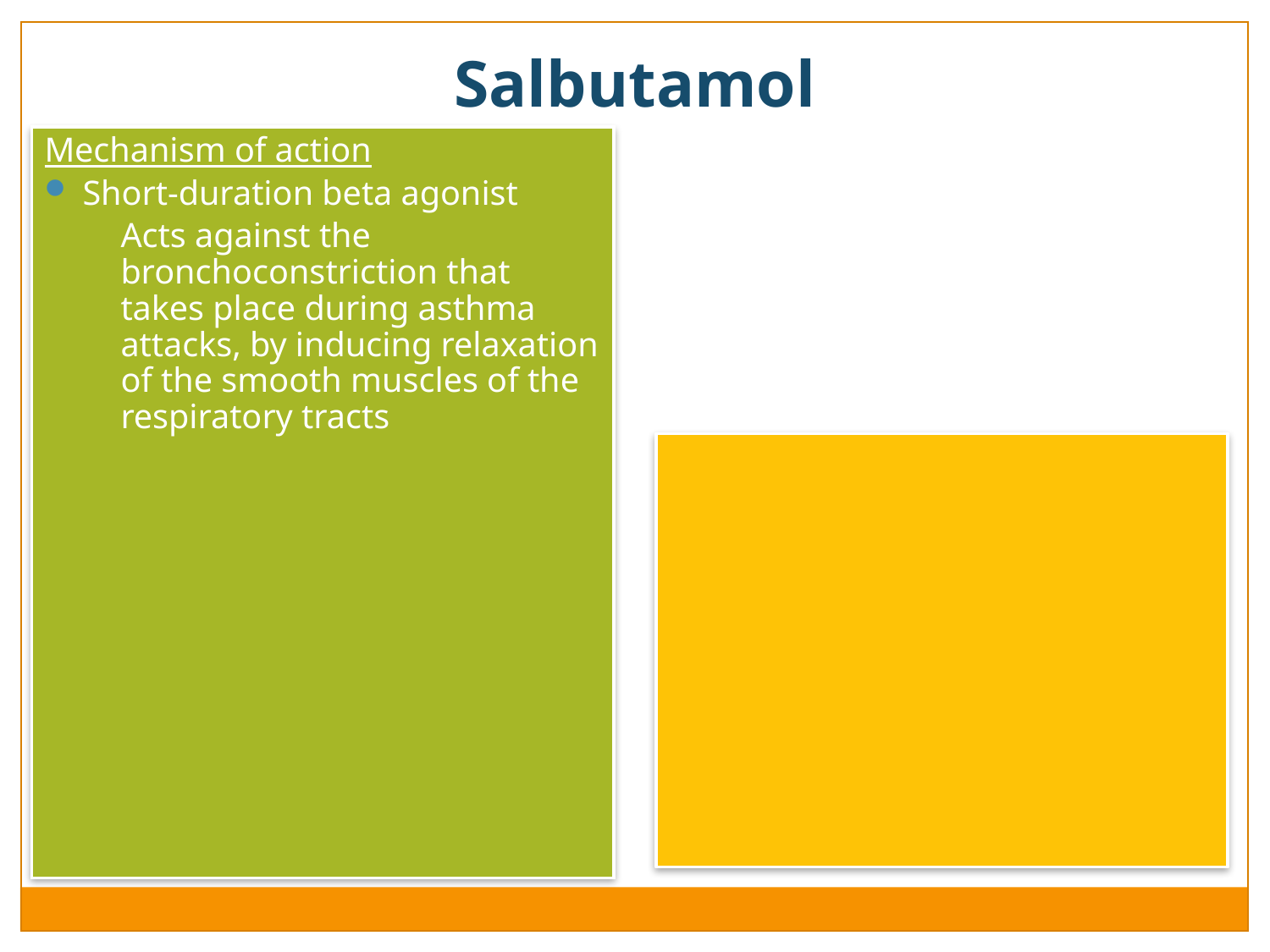

Salbutamol
Mechanism of action
Short-duration beta agonist
Acts against the bronchoconstriction that takes place during asthma attacks, by inducing relaxation of the smooth muscles of the respiratory tracts

## Slide 65
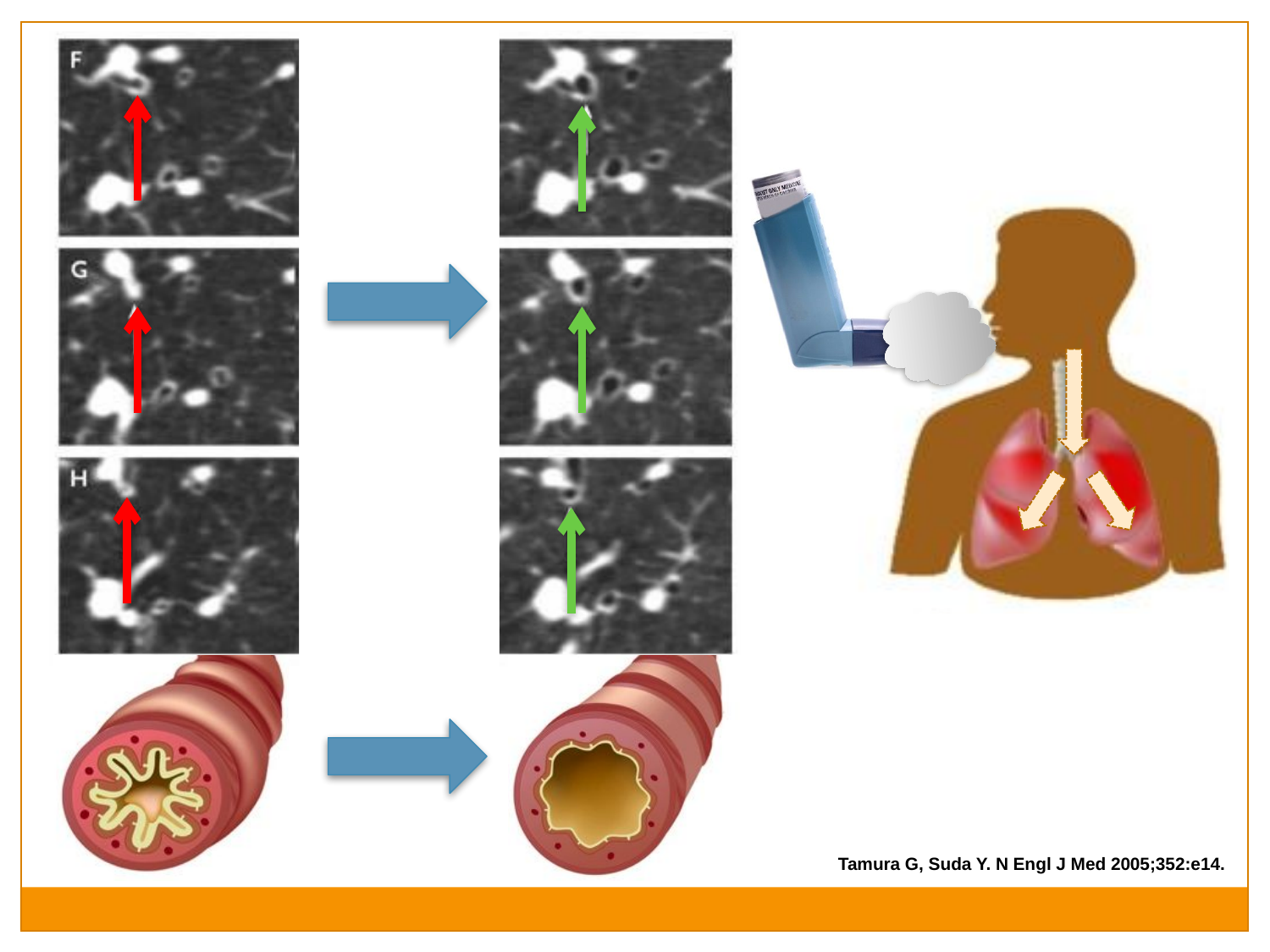

Tamura G, Suda Y. N Engl J Med 2005;352:e14.

## Slide 66
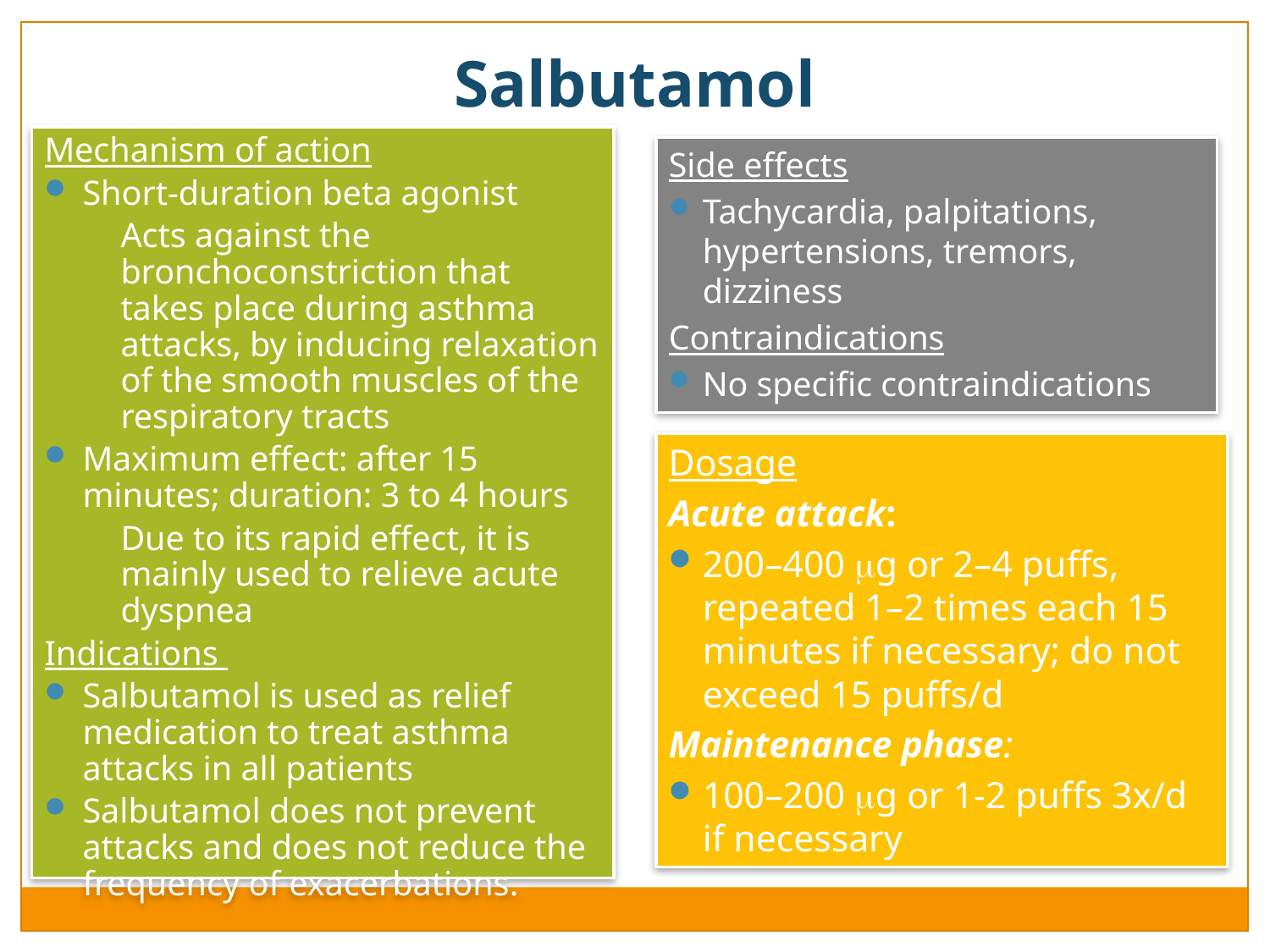

Salbutamol
Mechanism of action
Short-duration beta agonist
Acts against the bronchoconstriction that takes place during asthma attacks, by inducing relaxation of the smooth muscles of the respiratory tracts
Maximum effect: after 15 minutes; duration: 3 to 4 hours
Due to its rapid effect, it is mainly used to relieve acute dyspnea
Indications
Salbutamol is used as relief medication to treat asthma attacks in all patients
Salbutamol does not prevent attacks and does not reduce the frequency of exacerbations.
Side effects
Tachycardia, palpitations, hypertensions, tremors, dizziness
Contraindications
No specific contraindications
Dosage
Acute attack:
200–400 μg or 2–4 puffs, repeated 1–2 times each 15 minutes if necessary; do not exceed 15 puffs/d
Maintenance phase:
100–200 μg or 1-2 puffs 3x/d if necessary

## Slide 67
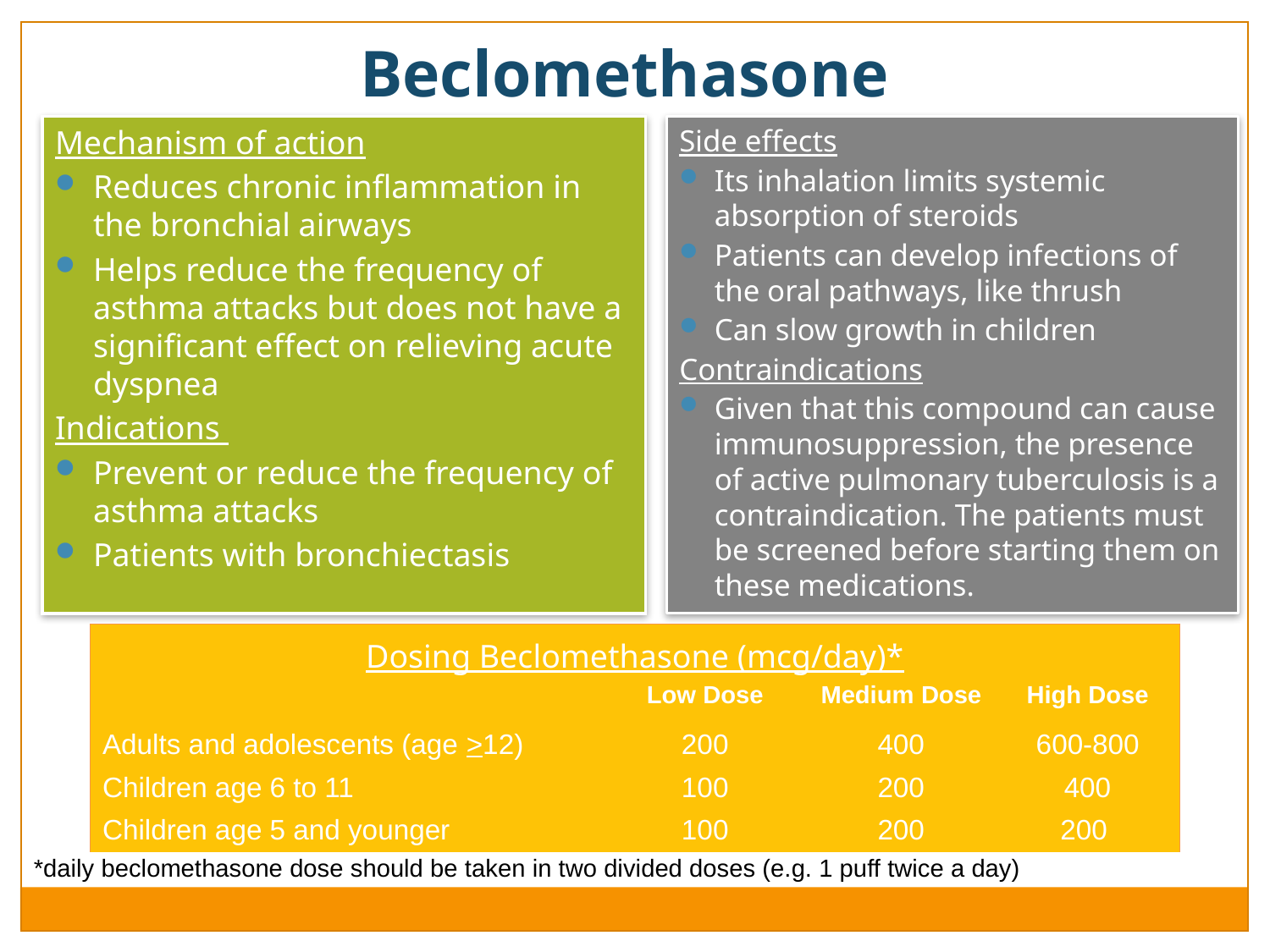

Beclomethasone
Mechanism of action
Reduces chronic inflammation in the bronchial airways
Helps reduce the frequency of asthma attacks but does not have a significant effect on relieving acute dyspnea
Indications
Prevent or reduce the frequency of asthma attacks
Patients with bronchiectasis
Side effects
Its inhalation limits systemic absorption of steroids
Patients can develop infections of the oral pathways, like thrush
Can slow growth in children
Contraindications
Given that this compound can cause immunosuppression, the presence of active pulmonary tuberculosis is a contraindication. The patients must be screened before starting them on these medications.
| Dosing Beclomethasone (mcg/day)\* | | | |
| --- | --- | --- | --- |
| | Low Dose | Medium Dose | High Dose |
| Adults and adolescents (age >12) | 200 | 400 | 600-800 |
| Children age 6 to 11 | 100 | 200 | 400 |
| Children age 5 and younger | 100 | 200 | 200 |
*daily beclomethasone dose should be taken in two divided doses (e.g. 1 puff twice a day)

## Slide 68
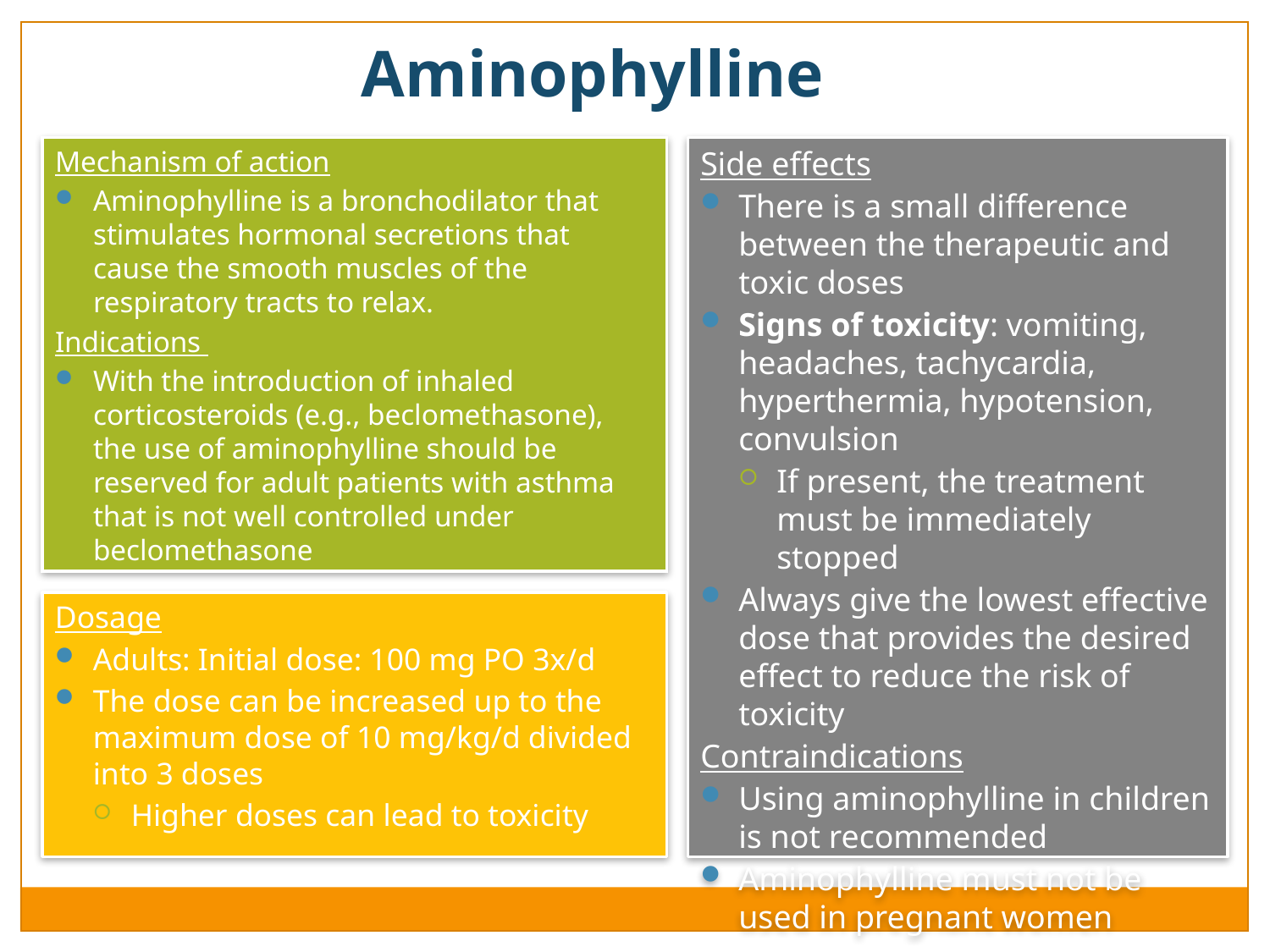

Aminophylline
Mechanism of action
Aminophylline is a bronchodilator that stimulates hormonal secretions that cause the smooth muscles of the respiratory tracts to relax.
Indications
With the introduction of inhaled corticosteroids (e.g., beclomethasone), the use of aminophylline should be reserved for adult patients with asthma that is not well controlled under beclomethasone
Side effects
There is a small difference between the therapeutic and toxic doses
Signs of toxicity: vomiting, headaches, tachycardia, hyperthermia, hypotension, convulsion
If present, the treatment must be immediately stopped
Always give the lowest effective dose that provides the desired effect to reduce the risk of toxicity
Contraindications
Using aminophylline in children is not recommended
Aminophylline must not be used in pregnant women
Dosage
Adults: Initial dose: 100 mg PO 3x/d
The dose can be increased up to the maximum dose of 10 mg/kg/d divided into 3 doses
Higher doses can lead to toxicity

## Slide 69
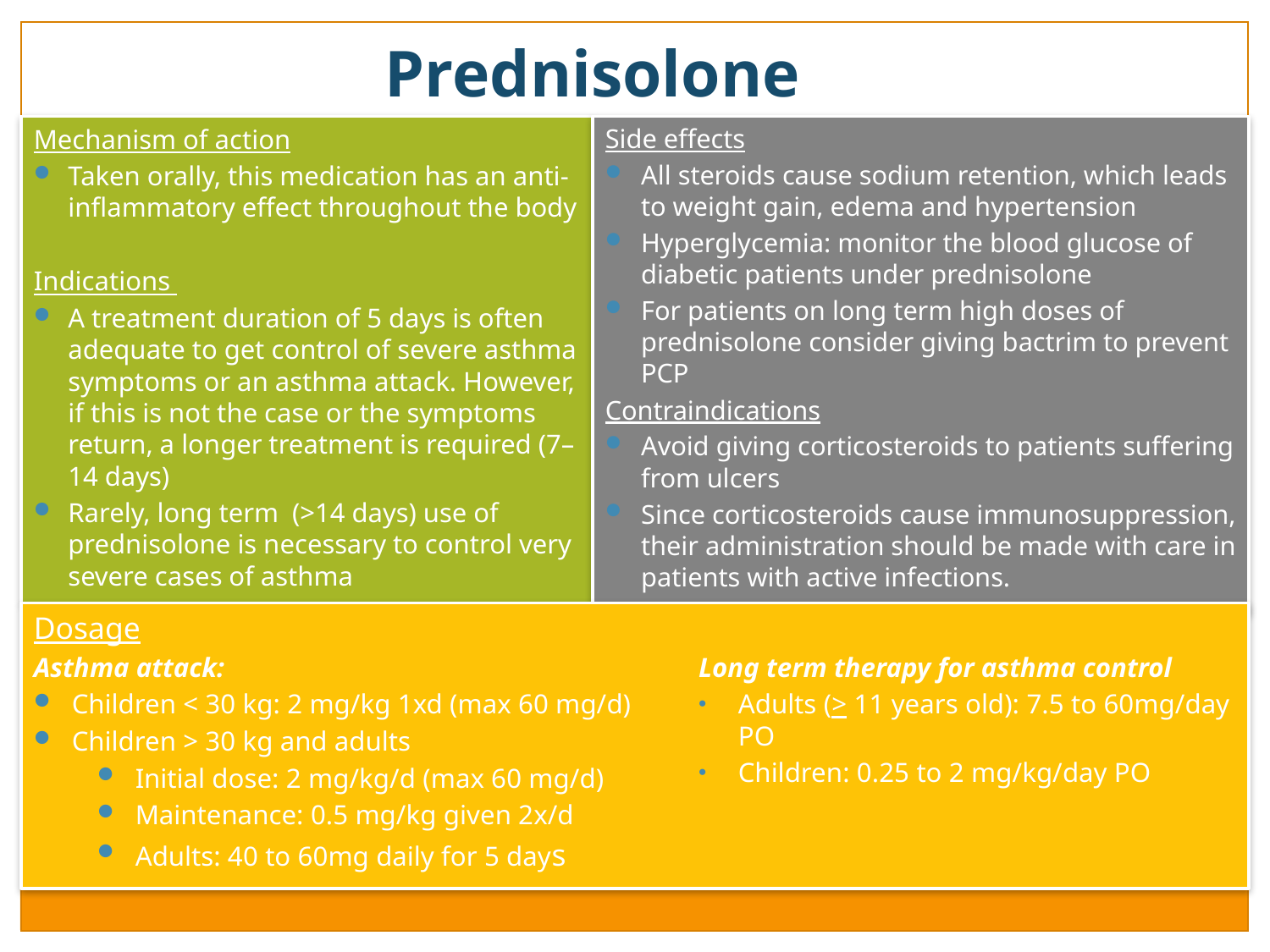

Prednisolone
Mechanism of action
Taken orally, this medication has an anti-inflammatory effect throughout the body
Indications
A treatment duration of 5 days is often adequate to get control of severe asthma symptoms or an asthma attack. However, if this is not the case or the symptoms return, a longer treatment is required (7–14 days)
Rarely, long term (>14 days) use of prednisolone is necessary to control very severe cases of asthma
Side effects
All steroids cause sodium retention, which leads to weight gain, edema and hypertension
Hyperglycemia: monitor the blood glucose of diabetic patients under prednisolone
For patients on long term high doses of prednisolone consider giving bactrim to prevent PCP
Contraindications
Avoid giving corticosteroids to patients suffering from ulcers
Since corticosteroids cause immunosuppression, their administration should be made with care in patients with active infections.
Dosage
Asthma attack:
Children < 30 kg: 2 mg/kg 1xd (max 60 mg/d)
Children > 30 kg and adults
Initial dose: 2 mg/kg/d (max 60 mg/d)
Maintenance: 0.5 mg/kg given 2x/d
Adults: 40 to 60mg daily for 5 days
Long term therapy for asthma control
Adults (> 11 years old): 7.5 to 60mg/day PO
Children: 0.25 to 2 mg/kg/day PO

## Slide 70
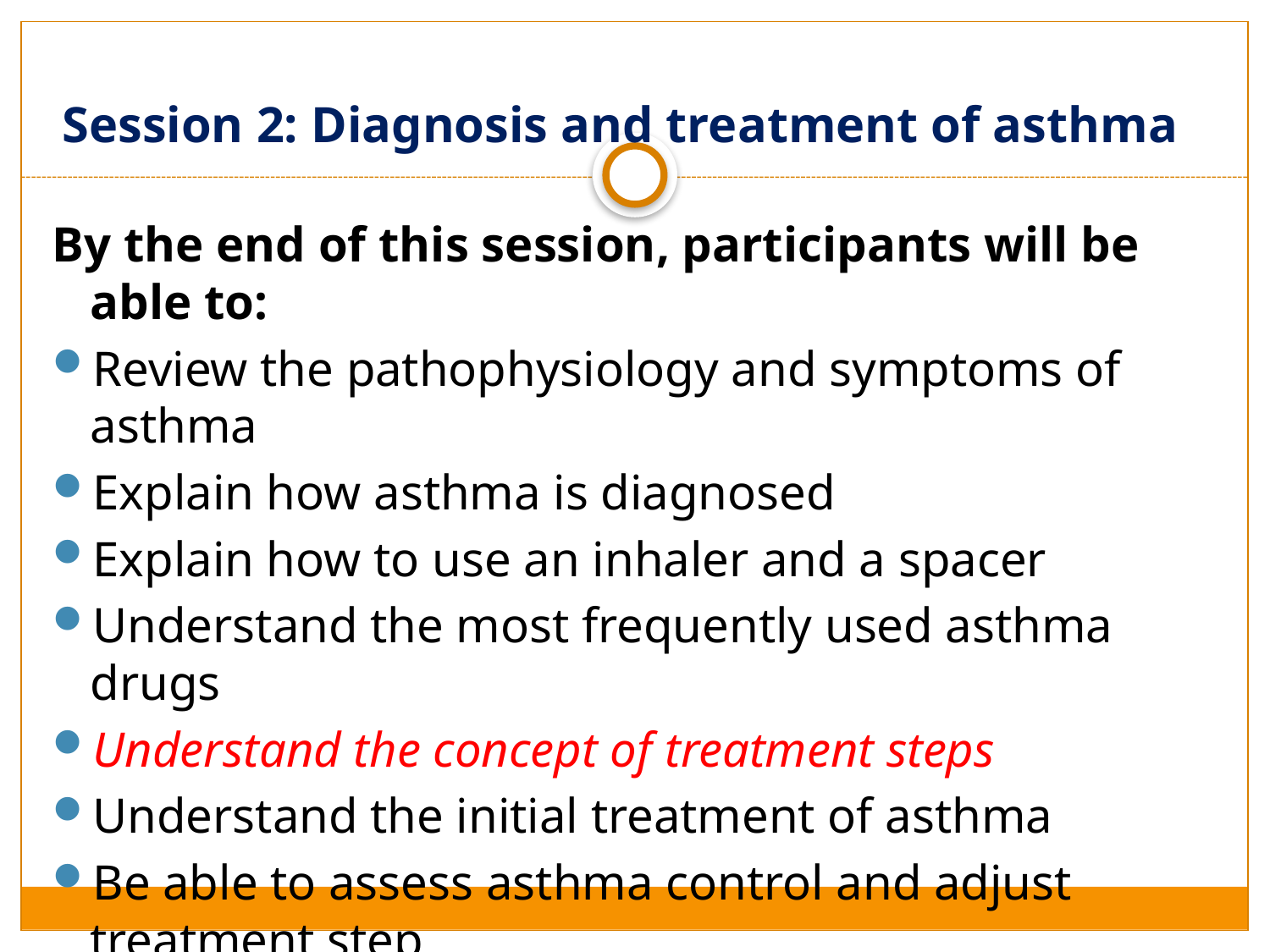

# Session 2: Diagnosis and treatment of asthma
By the end of this session, participants will be able to:
Review the pathophysiology and symptoms of asthma
Explain how asthma is diagnosed
Explain how to use an inhaler and a spacer
Understand the most frequently used asthma drugs
Understand the concept of treatment steps
Understand the initial treatment of asthma
Be able to assess asthma control and adjust treatment step
Be able to assess asthma attacks

## Slide 71
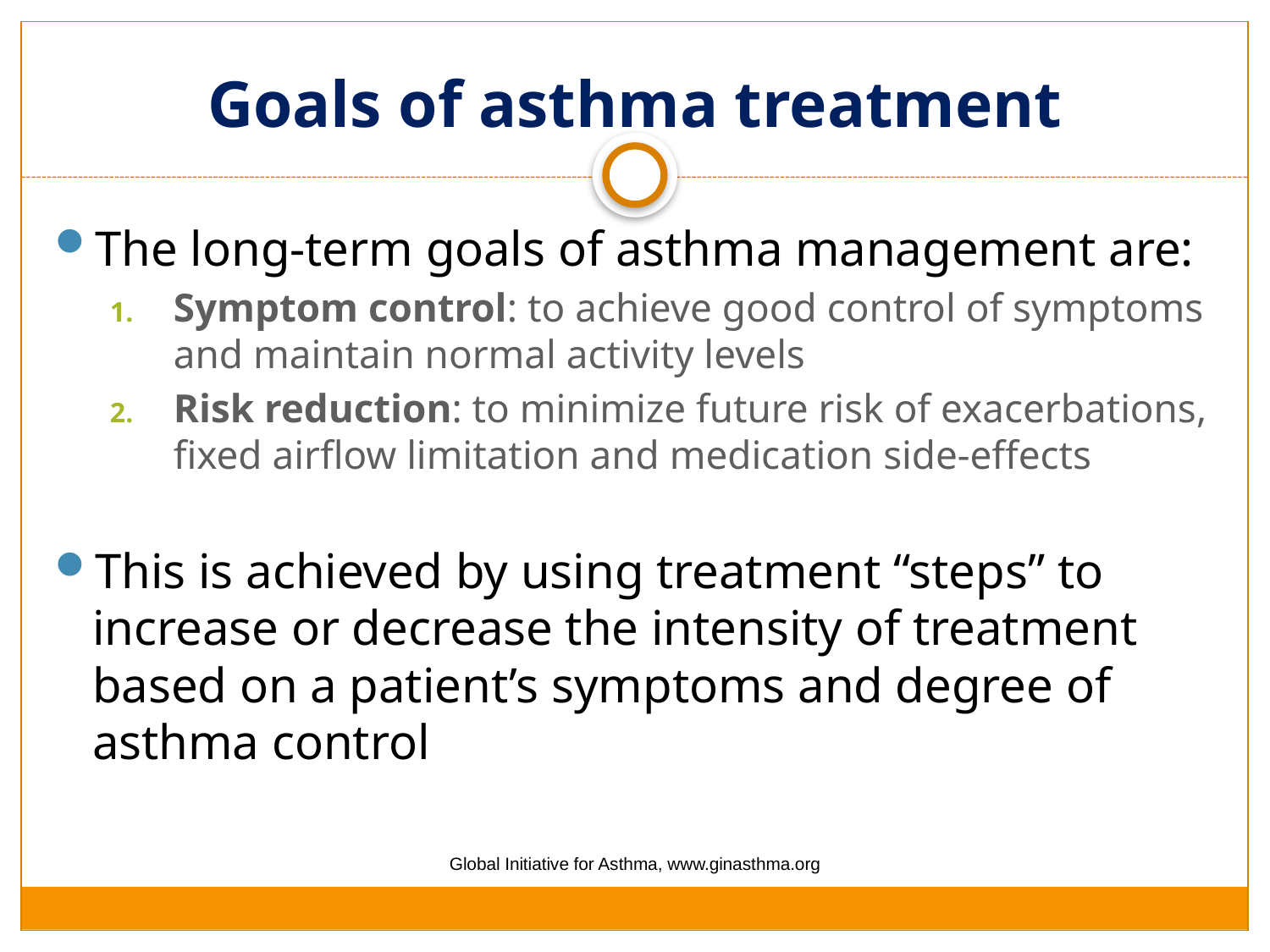

Goals of asthma treatment
The long-term goals of asthma management are:
Symptom control: to achieve good control of symptoms and maintain normal activity levels
Risk reduction: to minimize future risk of exacerbations, fixed airflow limitation and medication side-effects
This is achieved by using treatment “steps” to increase or decrease the intensity of treatment based on a patient’s symptoms and degree of asthma control
Global Initiative for Asthma, www.ginasthma.org
GINA 2018

## Slide 72
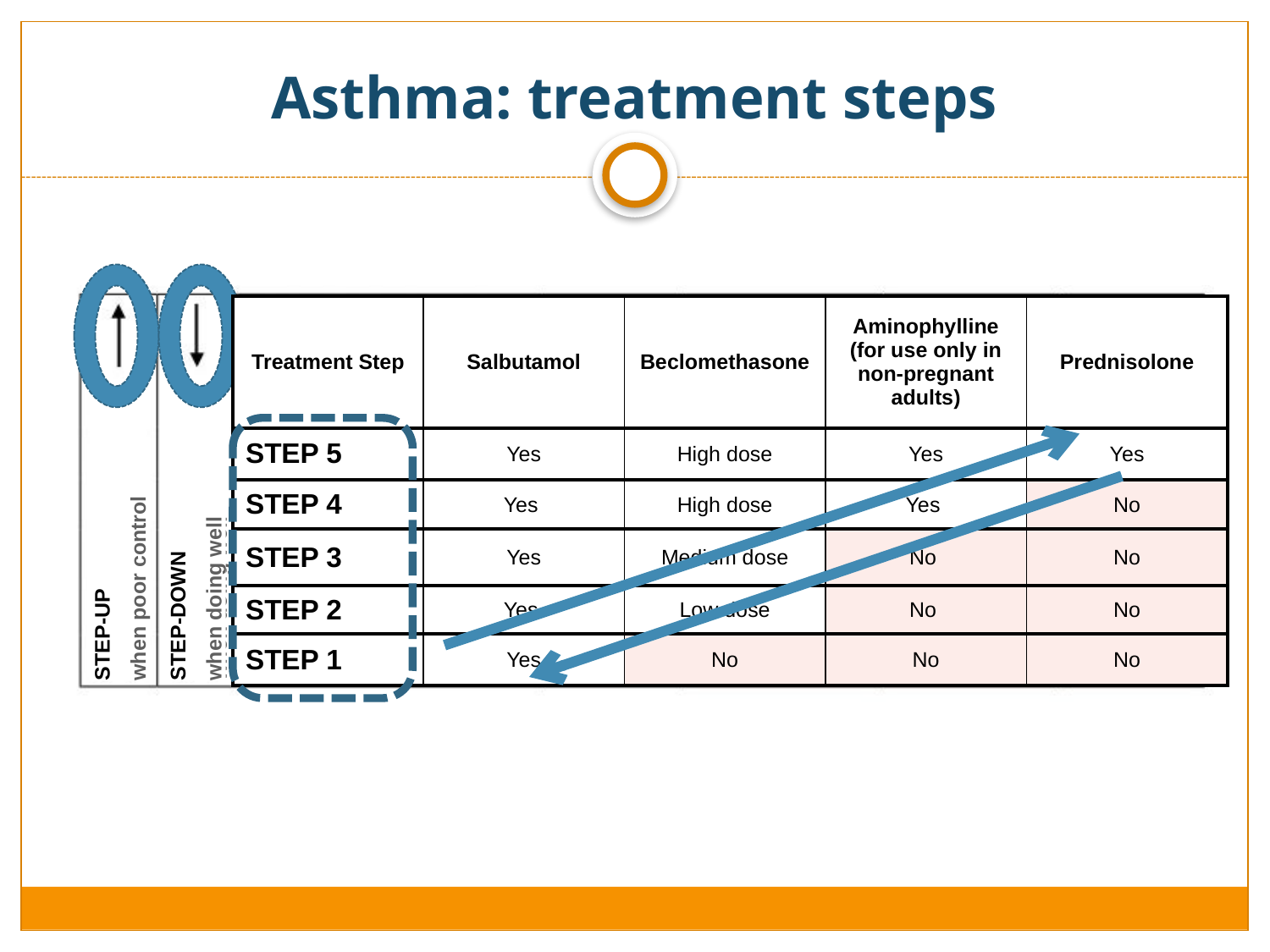

# Asthma: treatment steps
| Treatment Step | Salbutamol | Beclomethasone | Aminophylline (for use only in non-pregnant adults) | Prednisolone |
| --- | --- | --- | --- | --- |
| STEP 5 | Yes | High dose | Yes | Yes |
| STEP 4 | Yes | High dose | Yes | No |
| STEP 3 | Yes | Medium dose | No | No |
| STEP 2 | Yes | Low dose | No | No |
| STEP 1 | Yes | No | No | No |
STEP-UP
when poor control
STEP-UP
when poor control
STEP-DOWN
when doing well
STEP-DOWN
when doing well

## Slide 73
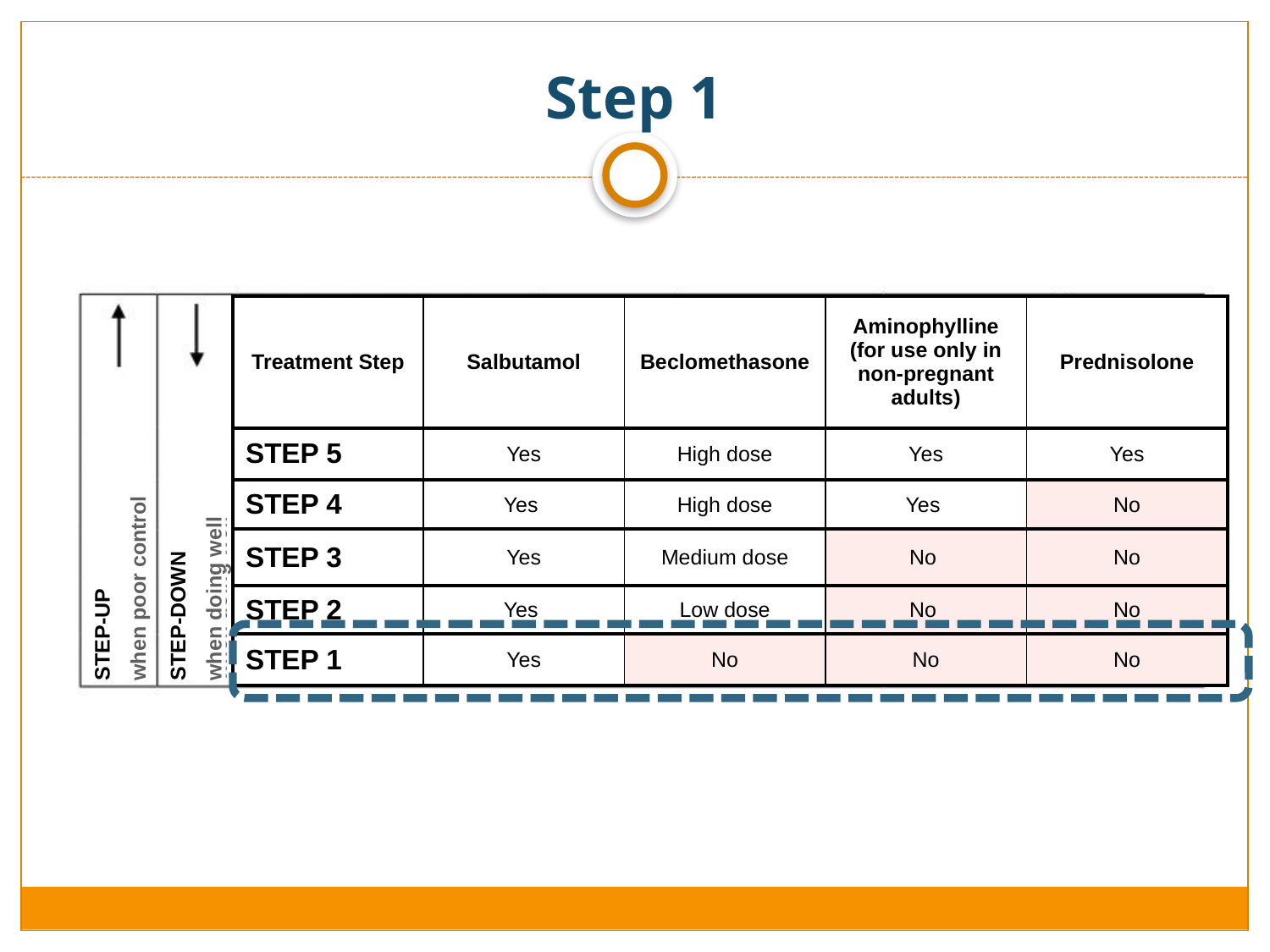

# Step 1
| Treatment Step | Salbutamol | Beclomethasone | Aminophylline (for use only in non-pregnant adults) | Prednisolone |
| --- | --- | --- | --- | --- |
| STEP 5 | Yes | High dose | Yes | Yes |
| STEP 4 | Yes | High dose | Yes | No |
| STEP 3 | Yes | Medium dose | No | No |
| STEP 2 | Yes | Low dose | No | No |
| STEP 1 | Yes | No | No | No |
STEP-UP
when poor control
STEP-UP
when poor control
STEP-DOWN
when doing well
STEP-DOWN
when doing well

## Slide 74
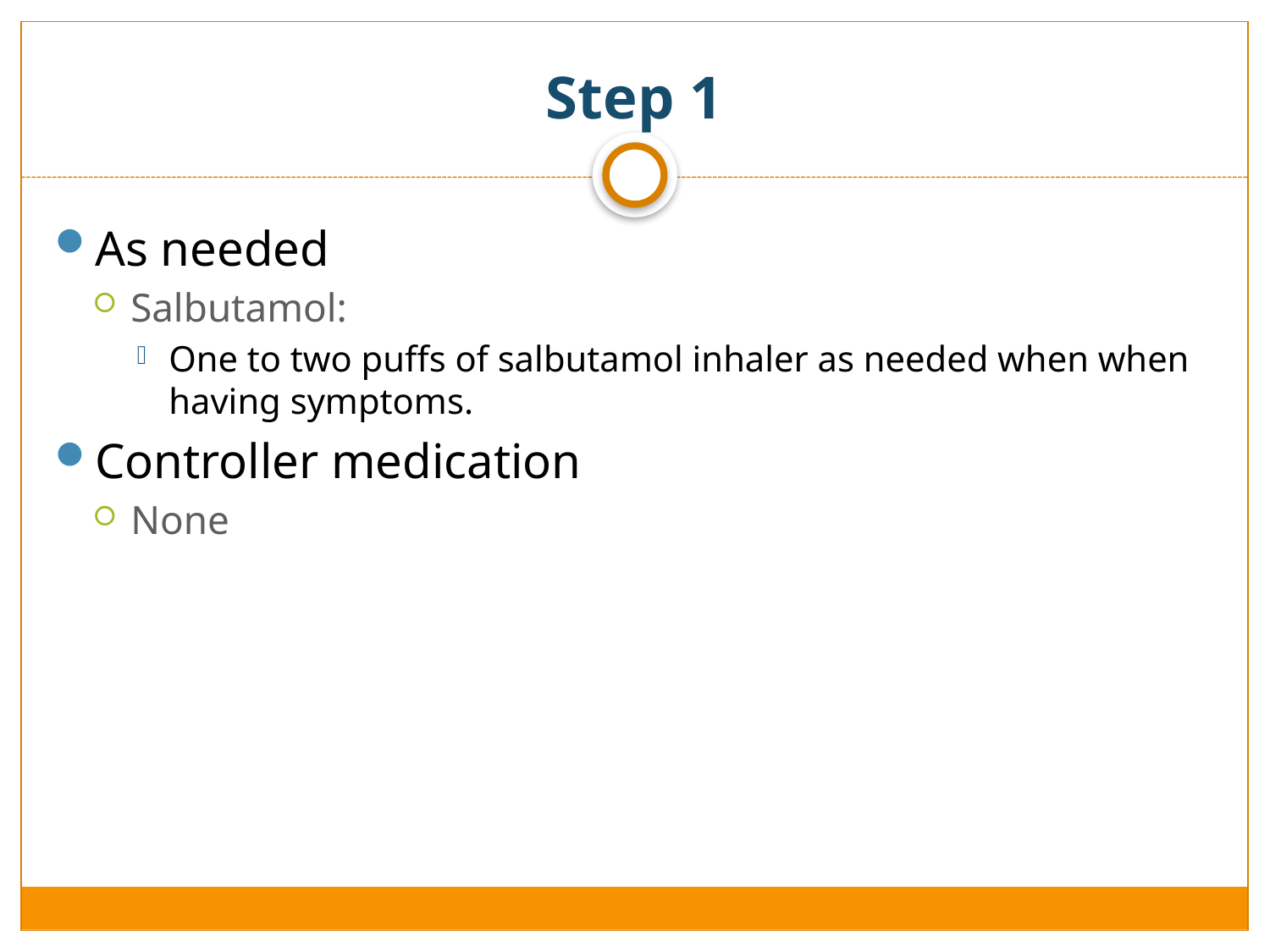

# Step 1
As needed
Salbutamol:
One to two puffs of salbutamol inhaler as needed when when having symptoms.
Controller medication
None

## Slide 75
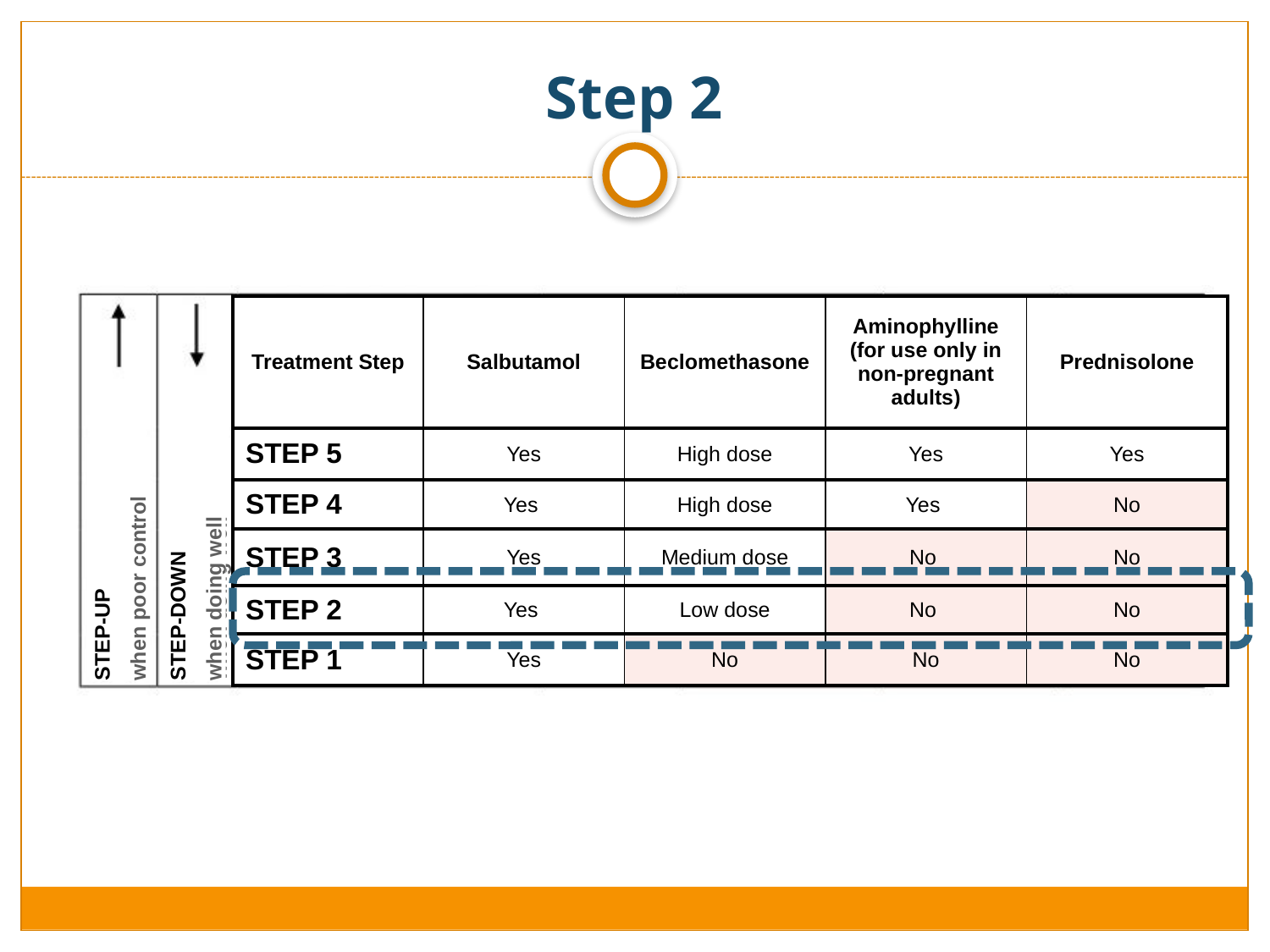

# Step 2
| Treatment Step | Salbutamol | Beclomethasone | Aminophylline (for use only in non-pregnant adults) | Prednisolone |
| --- | --- | --- | --- | --- |
| STEP 5 | Yes | High dose | Yes | Yes |
| STEP 4 | Yes | High dose | Yes | No |
| STEP 3 | Yes | Medium dose | No | No |
| STEP 2 | Yes | Low dose | No | No |
| STEP 1 | Yes | No | No | No |
STEP-UP
when poor control
STEP-UP
when poor control
STEP-DOWN
when doing well
STEP-DOWN
when doing well

## Slide 76
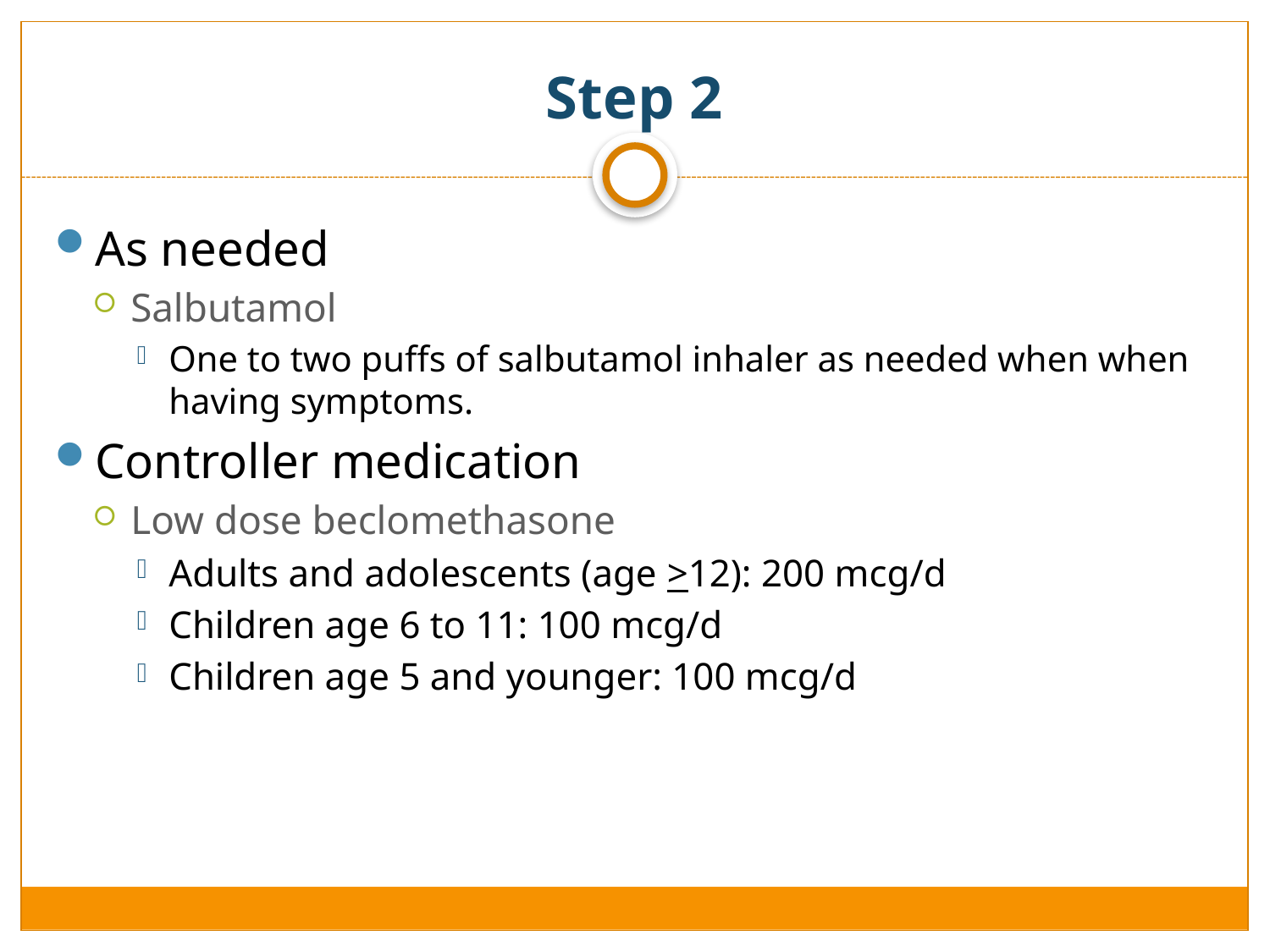

# Step 2
As needed
Salbutamol
One to two puffs of salbutamol inhaler as needed when when having symptoms.
Controller medication
Low dose beclomethasone
Adults and adolescents (age >12): 200 mcg/d
Children age 6 to 11: 100 mcg/d
Children age 5 and younger: 100 mcg/d

## Slide 77
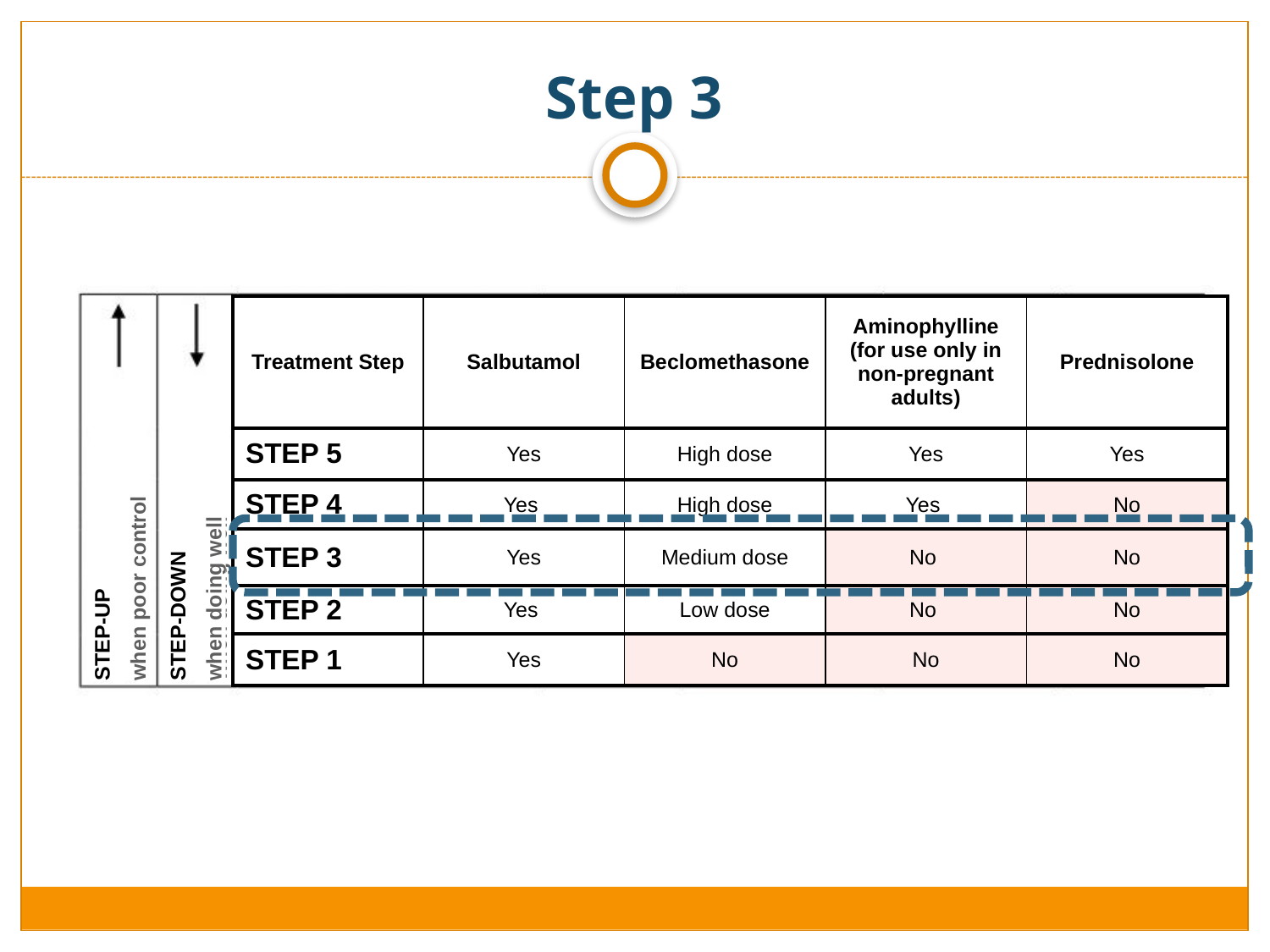

# Step 3
| Treatment Step | Salbutamol | Beclomethasone | Aminophylline (for use only in non-pregnant adults) | Prednisolone |
| --- | --- | --- | --- | --- |
| STEP 5 | Yes | High dose | Yes | Yes |
| STEP 4 | Yes | High dose | Yes | No |
| STEP 3 | Yes | Medium dose | No | No |
| STEP 2 | Yes | Low dose | No | No |
| STEP 1 | Yes | No | No | No |
STEP-UP
when poor control
STEP-UP
when poor control
STEP-DOWN
when doing well
STEP-DOWN
when doing well

## Slide 78
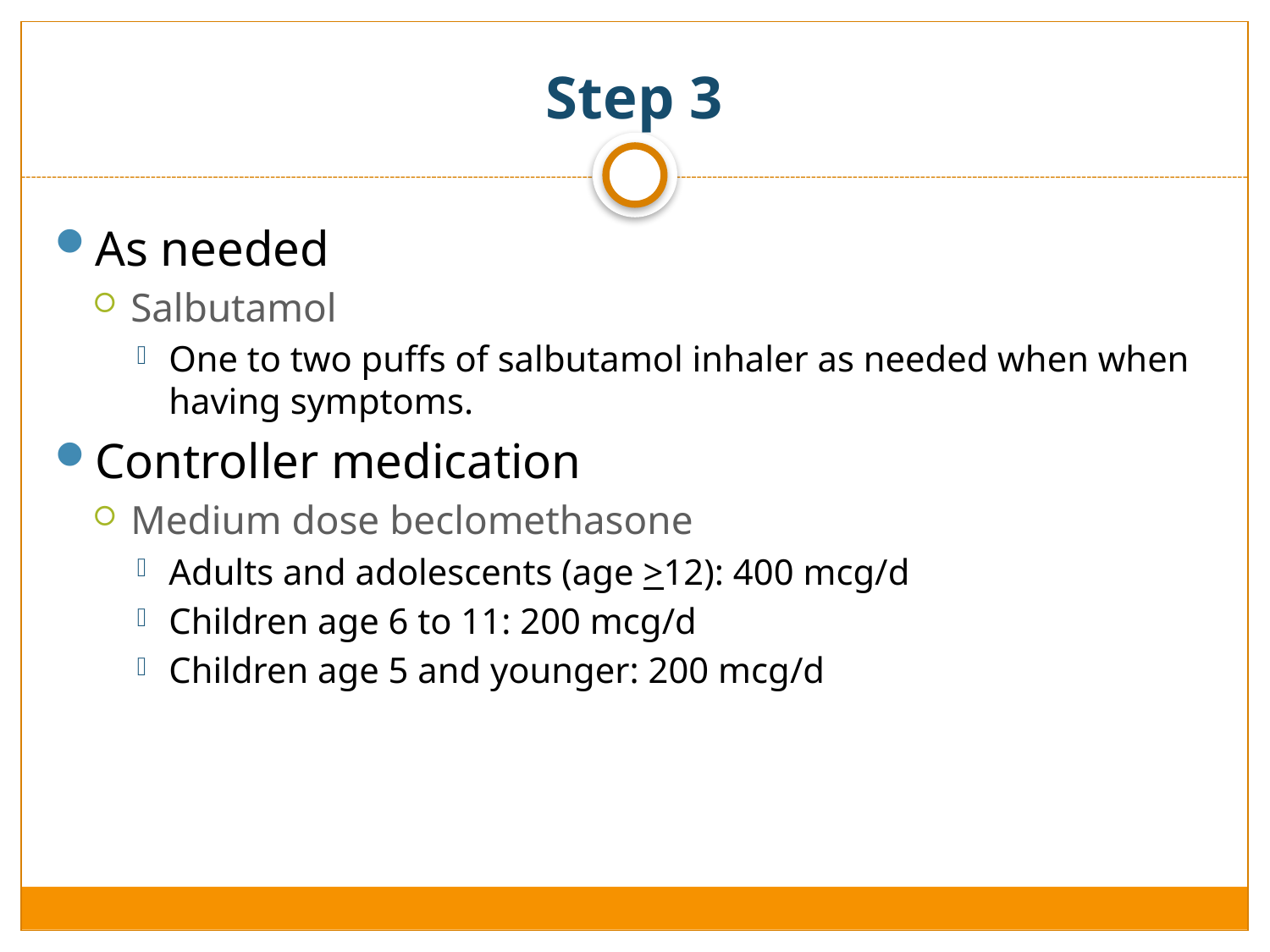

# Step 3
As needed
Salbutamol
One to two puffs of salbutamol inhaler as needed when when having symptoms.
Controller medication
Medium dose beclomethasone
Adults and adolescents (age >12): 400 mcg/d
Children age 6 to 11: 200 mcg/d
Children age 5 and younger: 200 mcg/d

## Slide 79
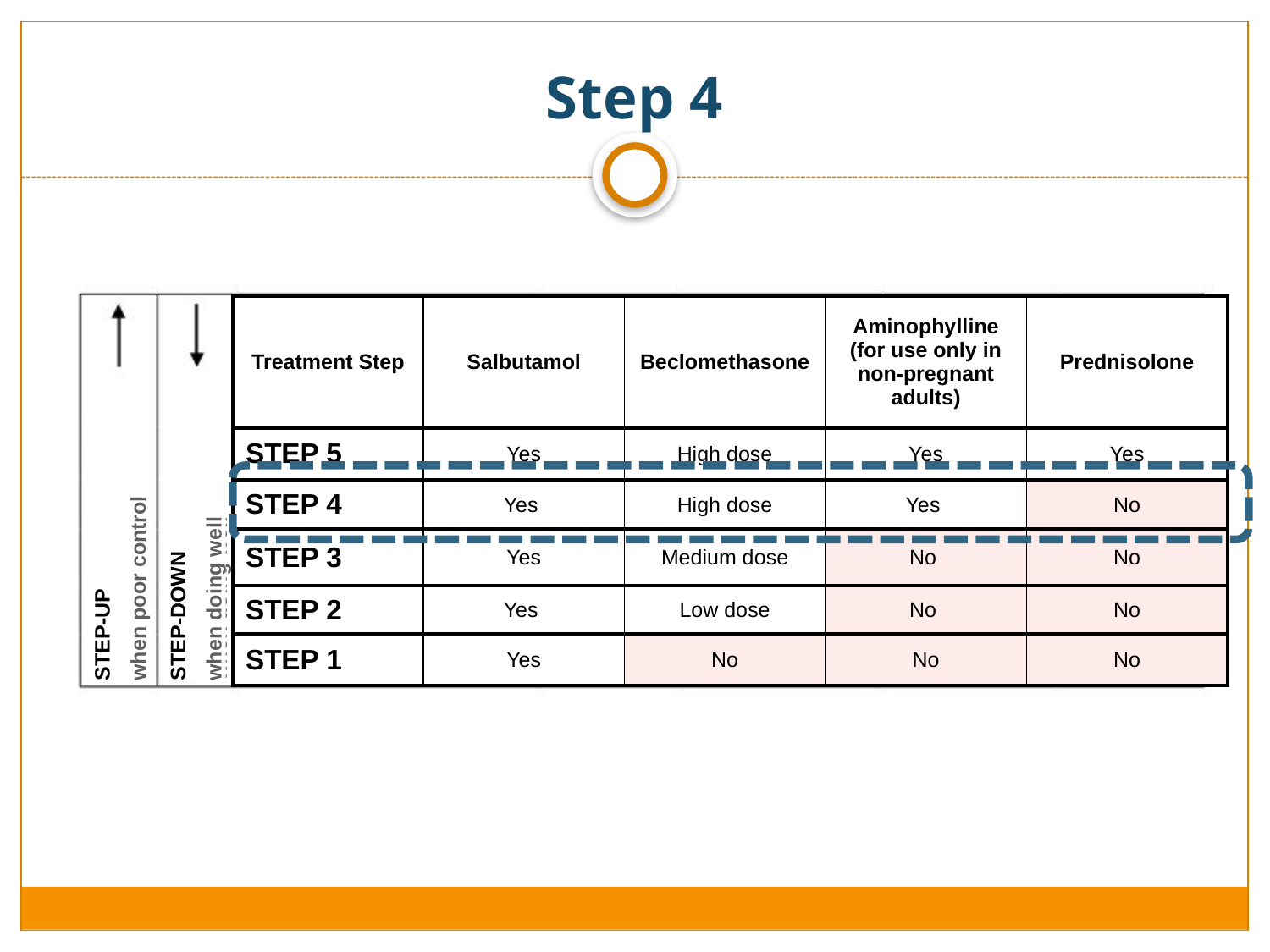

# Step 4
| Treatment Step | Salbutamol | Beclomethasone | Aminophylline (for use only in non-pregnant adults) | Prednisolone |
| --- | --- | --- | --- | --- |
| STEP 5 | Yes | High dose | Yes | Yes |
| STEP 4 | Yes | High dose | Yes | No |
| STEP 3 | Yes | Medium dose | No | No |
| STEP 2 | Yes | Low dose | No | No |
| STEP 1 | Yes | No | No | No |
STEP-UP
when poor control
STEP-UP
when poor control
STEP-DOWN
when doing well
STEP-DOWN
when doing well

## Slide 80
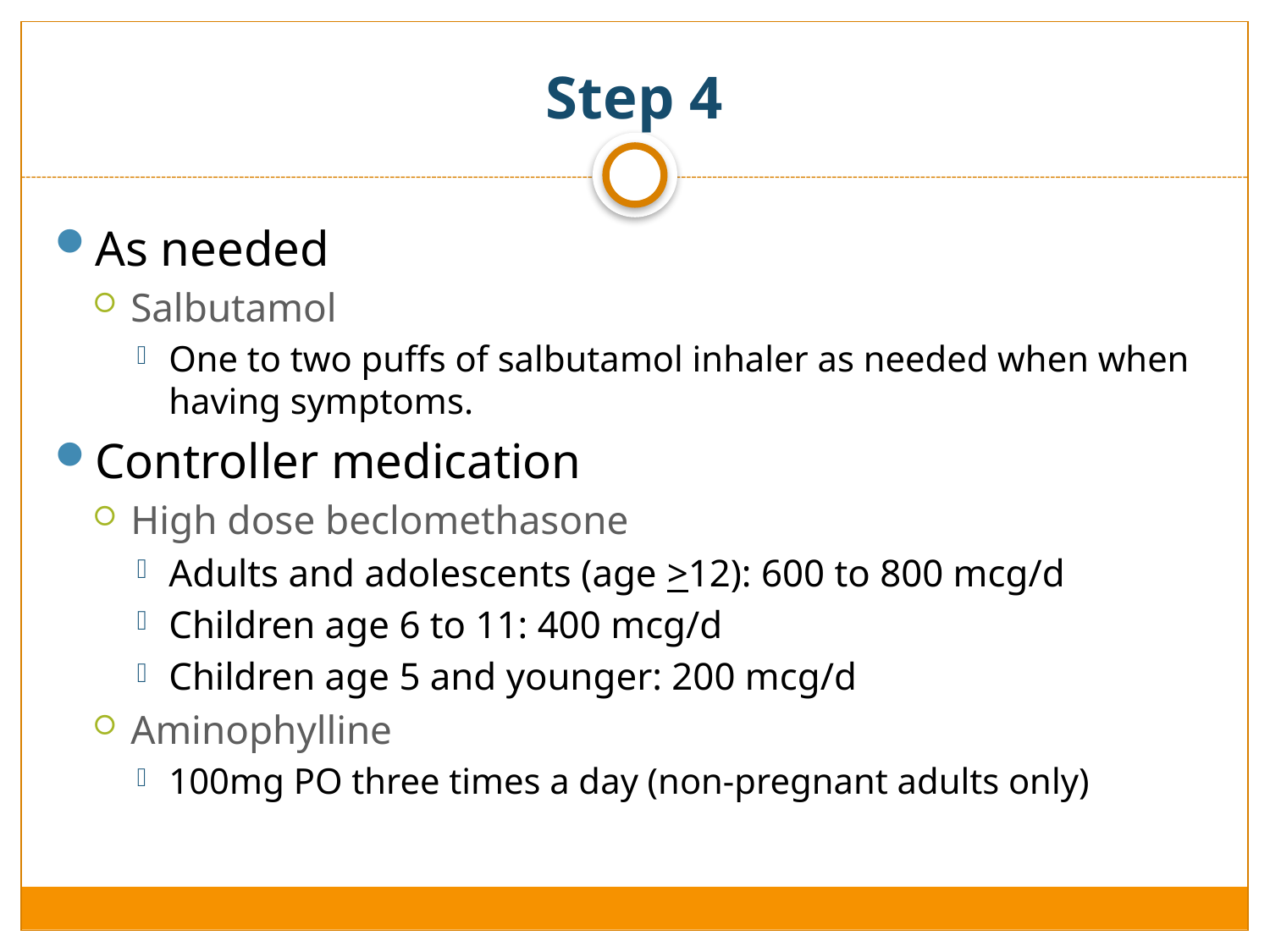

# Step 4
As needed
Salbutamol
One to two puffs of salbutamol inhaler as needed when when having symptoms.
Controller medication
High dose beclomethasone
Adults and adolescents (age >12): 600 to 800 mcg/d
Children age 6 to 11: 400 mcg/d
Children age 5 and younger: 200 mcg/d
Aminophylline
100mg PO three times a day (non-pregnant adults only)

## Slide 81
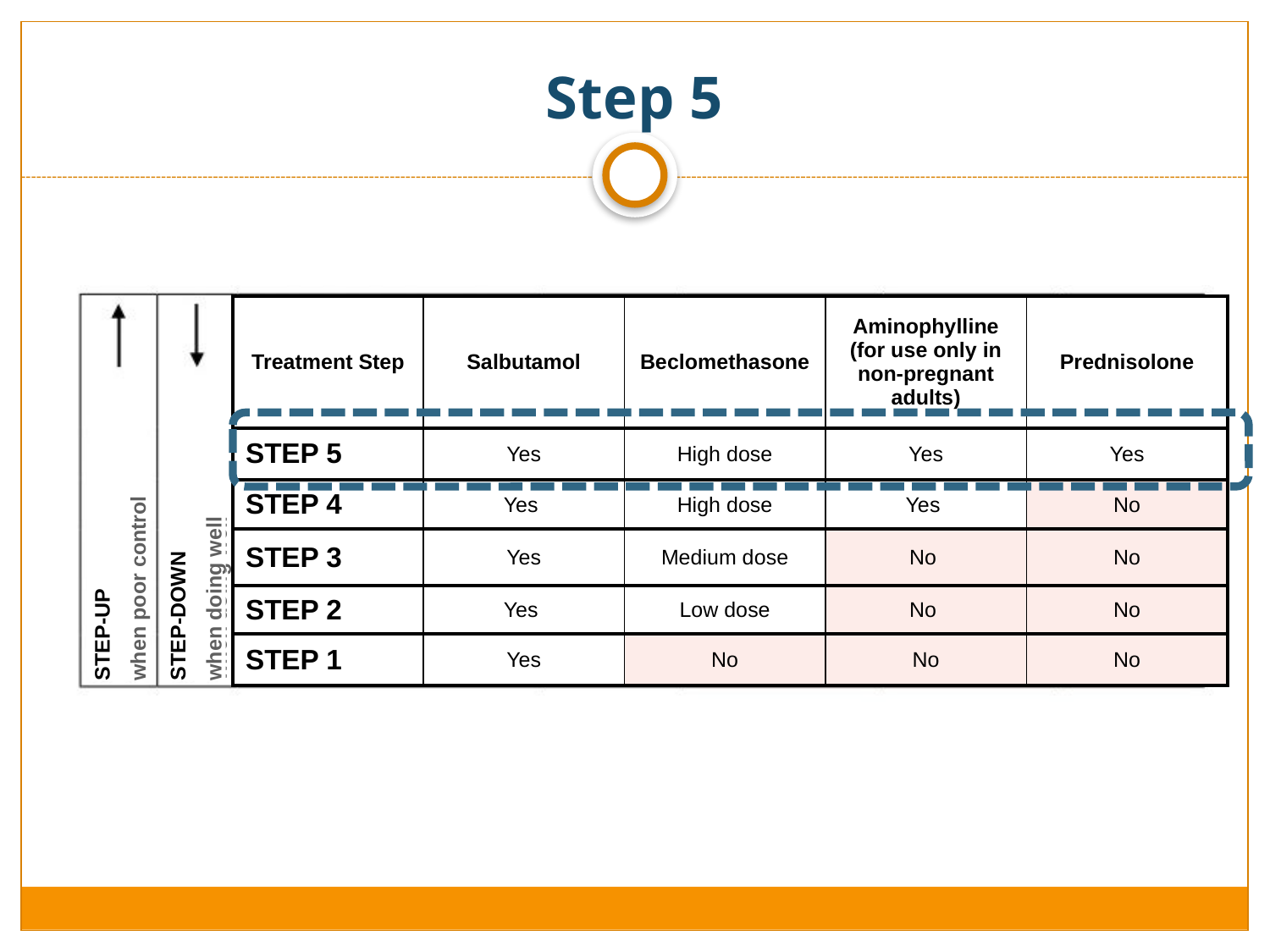

# Step 5
| Treatment Step | Salbutamol | Beclomethasone | Aminophylline (for use only in non-pregnant adults) | Prednisolone |
| --- | --- | --- | --- | --- |
| STEP 5 | Yes | High dose | Yes | Yes |
| STEP 4 | Yes | High dose | Yes | No |
| STEP 3 | Yes | Medium dose | No | No |
| STEP 2 | Yes | Low dose | No | No |
| STEP 1 | Yes | No | No | No |
STEP-UP
when poor control
STEP-UP
when poor control
STEP-DOWN
when doing well
STEP-DOWN
when doing well

## Slide 82
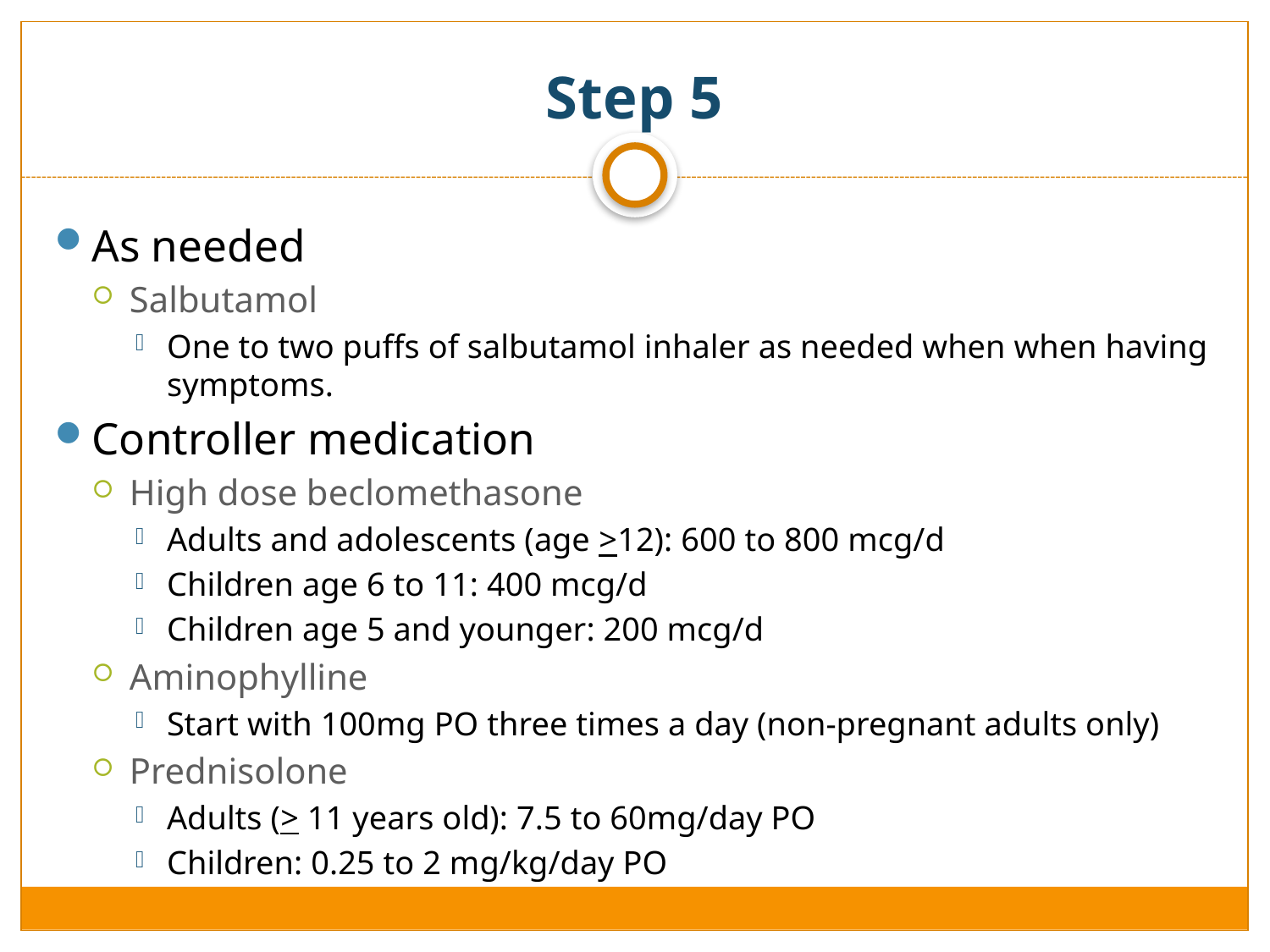

# Step 5
As needed
Salbutamol
One to two puffs of salbutamol inhaler as needed when when having symptoms.
Controller medication
High dose beclomethasone
Adults and adolescents (age >12): 600 to 800 mcg/d
Children age 6 to 11: 400 mcg/d
Children age 5 and younger: 200 mcg/d
Aminophylline
Start with 100mg PO three times a day (non-pregnant adults only)
Prednisolone
Adults (> 11 years old): 7.5 to 60mg/day PO
Children: 0.25 to 2 mg/kg/day PO

## Slide 83
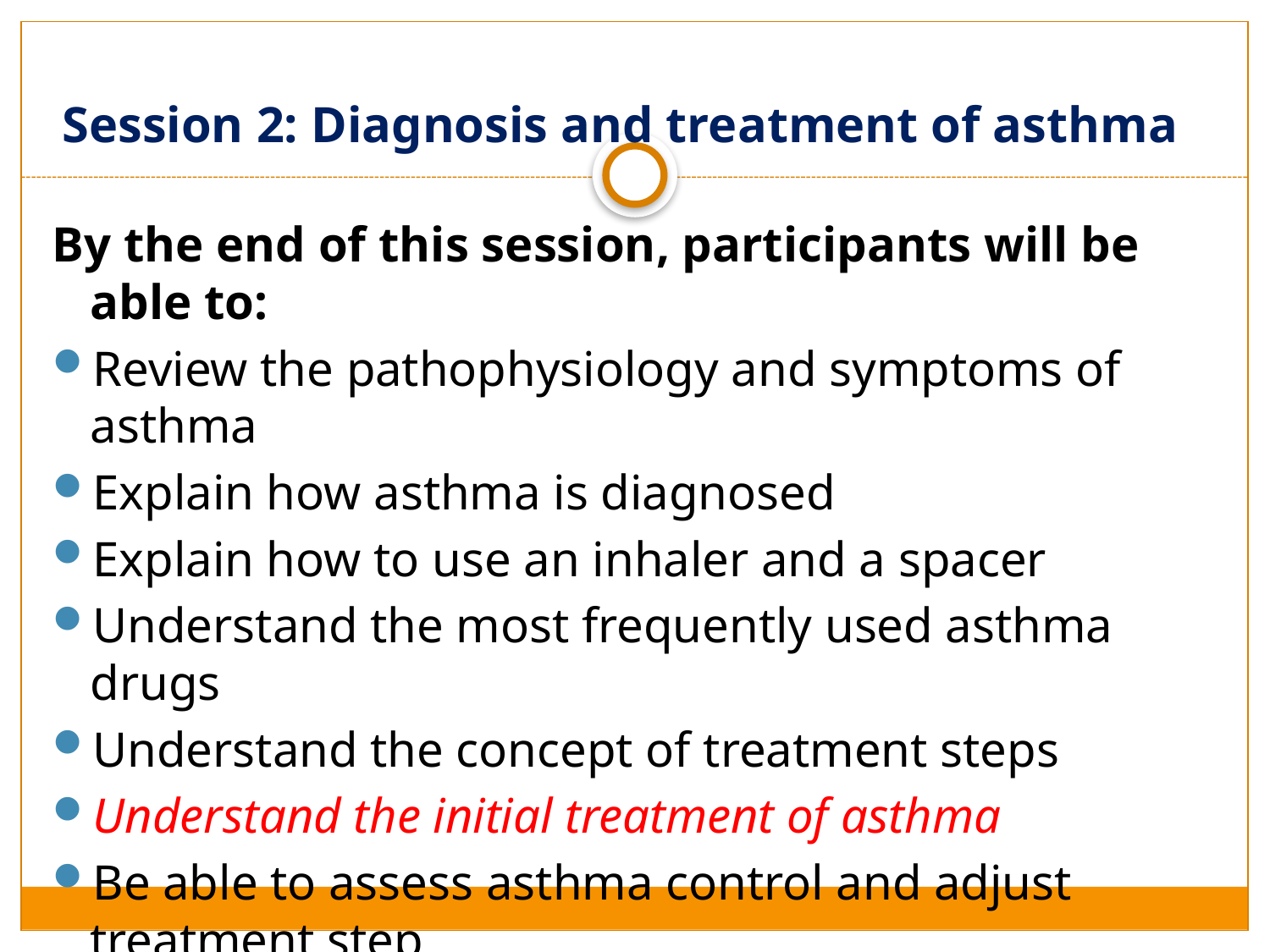

# Session 2: Diagnosis and treatment of asthma
By the end of this session, participants will be able to:
Review the pathophysiology and symptoms of asthma
Explain how asthma is diagnosed
Explain how to use an inhaler and a spacer
Understand the most frequently used asthma drugs
Understand the concept of treatment steps
Understand the initial treatment of asthma
Be able to assess asthma control and adjust treatment step
Be able to assess asthma attacks

## Slide 84
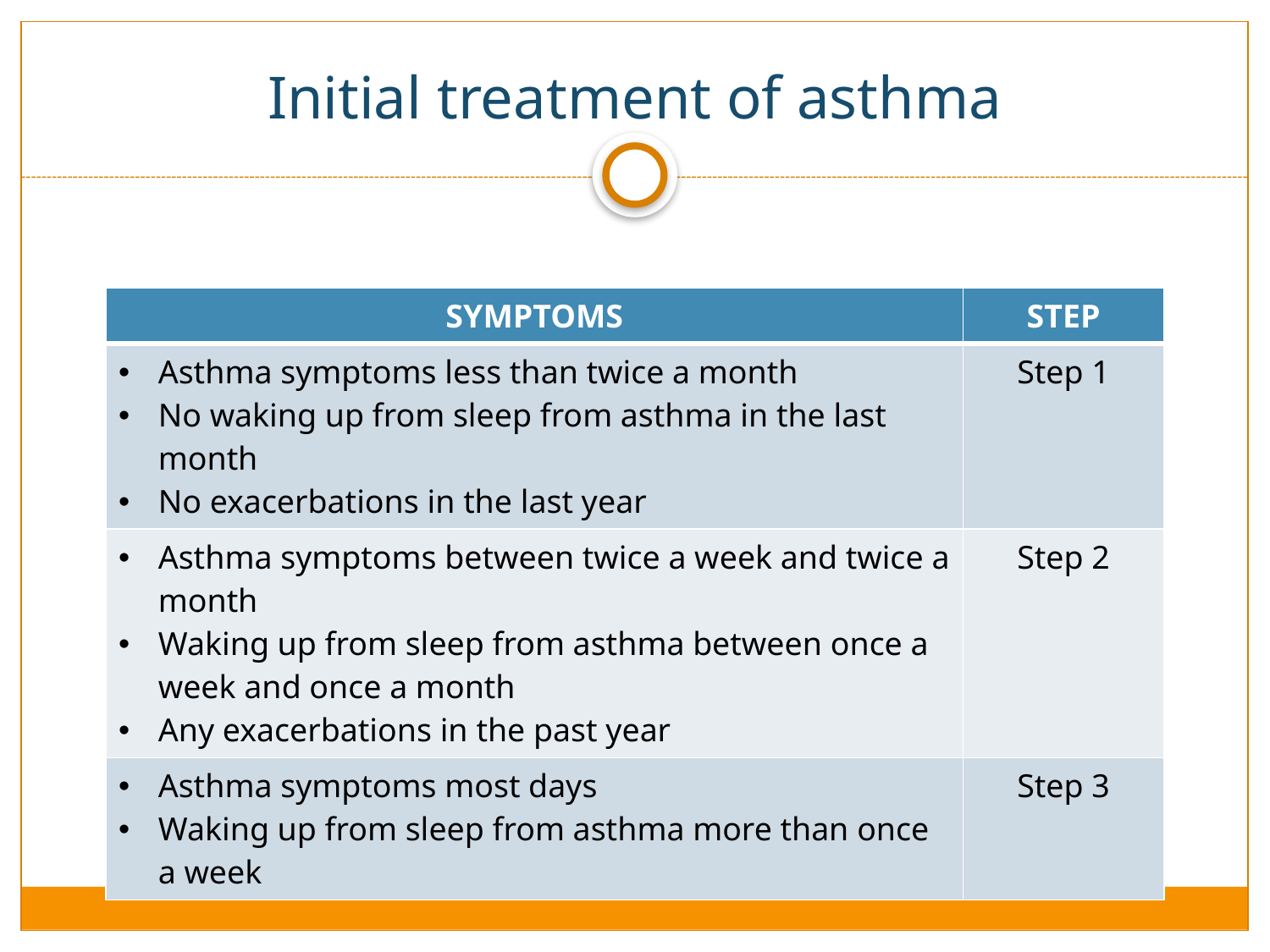

# Initial treatment of asthma
| SYMPTOMS | STEP |
| --- | --- |
| Asthma symptoms less than twice a month No waking up from sleep from asthma in the last month No exacerbations in the last year | Step 1 |
| Asthma symptoms between twice a week and twice a month Waking up from sleep from asthma between once a week and once a month Any exacerbations in the past year | Step 2 |
| Asthma symptoms most days Waking up from sleep from asthma more than once a week | Step 3 |

## Slide 85
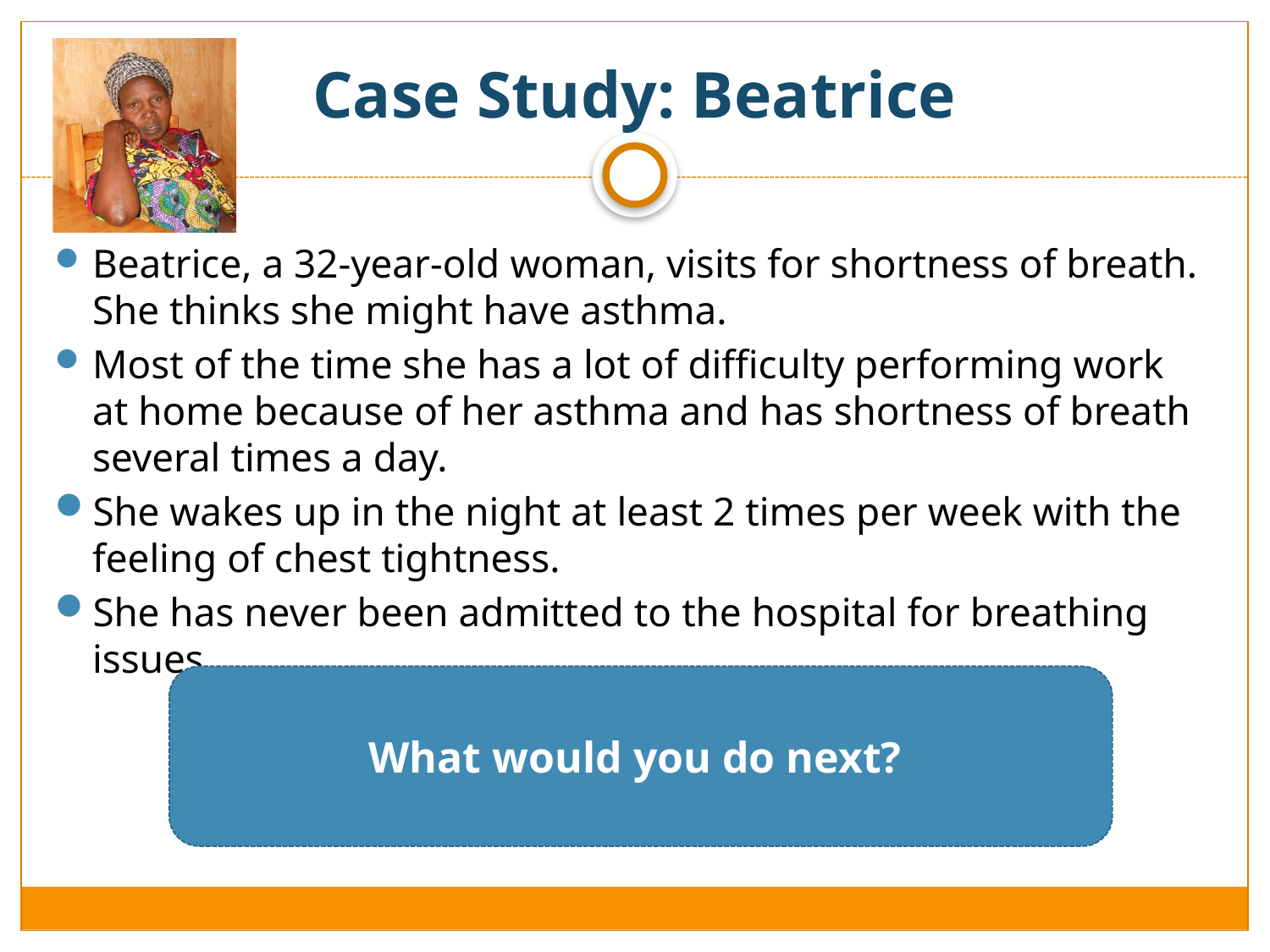

# Case Study: Beatrice
Beatrice, a 32-year-old woman, visits for shortness of breath. She thinks she might have asthma.
Most of the time she has a lot of difficulty performing work at home because of her asthma and has shortness of breath several times a day.
She wakes up in the night at least 2 times per week with the feeling of chest tightness.
She has never been admitted to the hospital for breathing issues
What would you do next?

## Slide 86
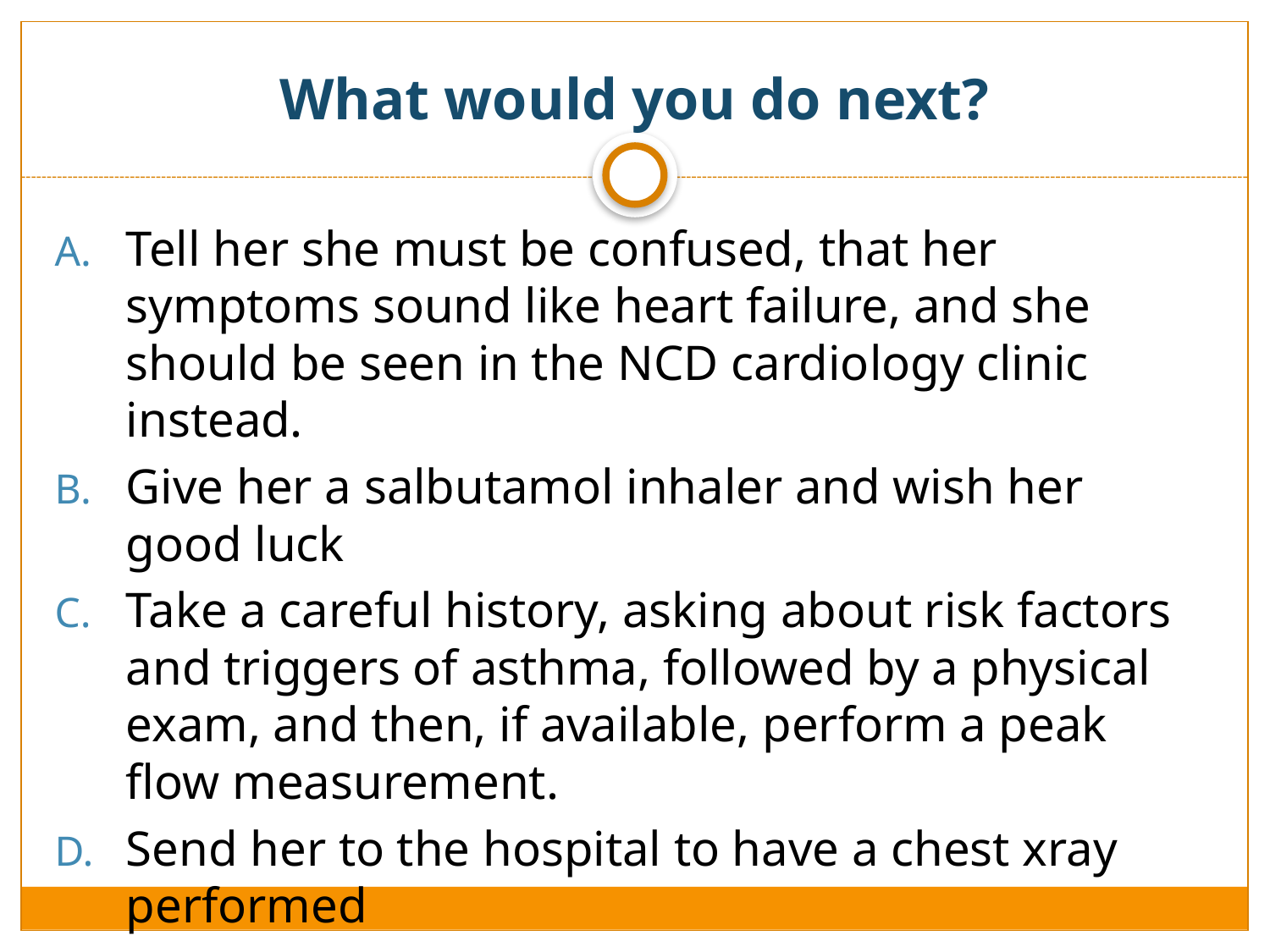

# What would you do next?
Tell her she must be confused, that her symptoms sound like heart failure, and she should be seen in the NCD cardiology clinic instead.
Give her a salbutamol inhaler and wish her good luck
Take a careful history, asking about risk factors and triggers of asthma, followed by a physical exam, and then, if available, perform a peak flow measurement.
Send her to the hospital to have a chest xray performed

## Slide 87
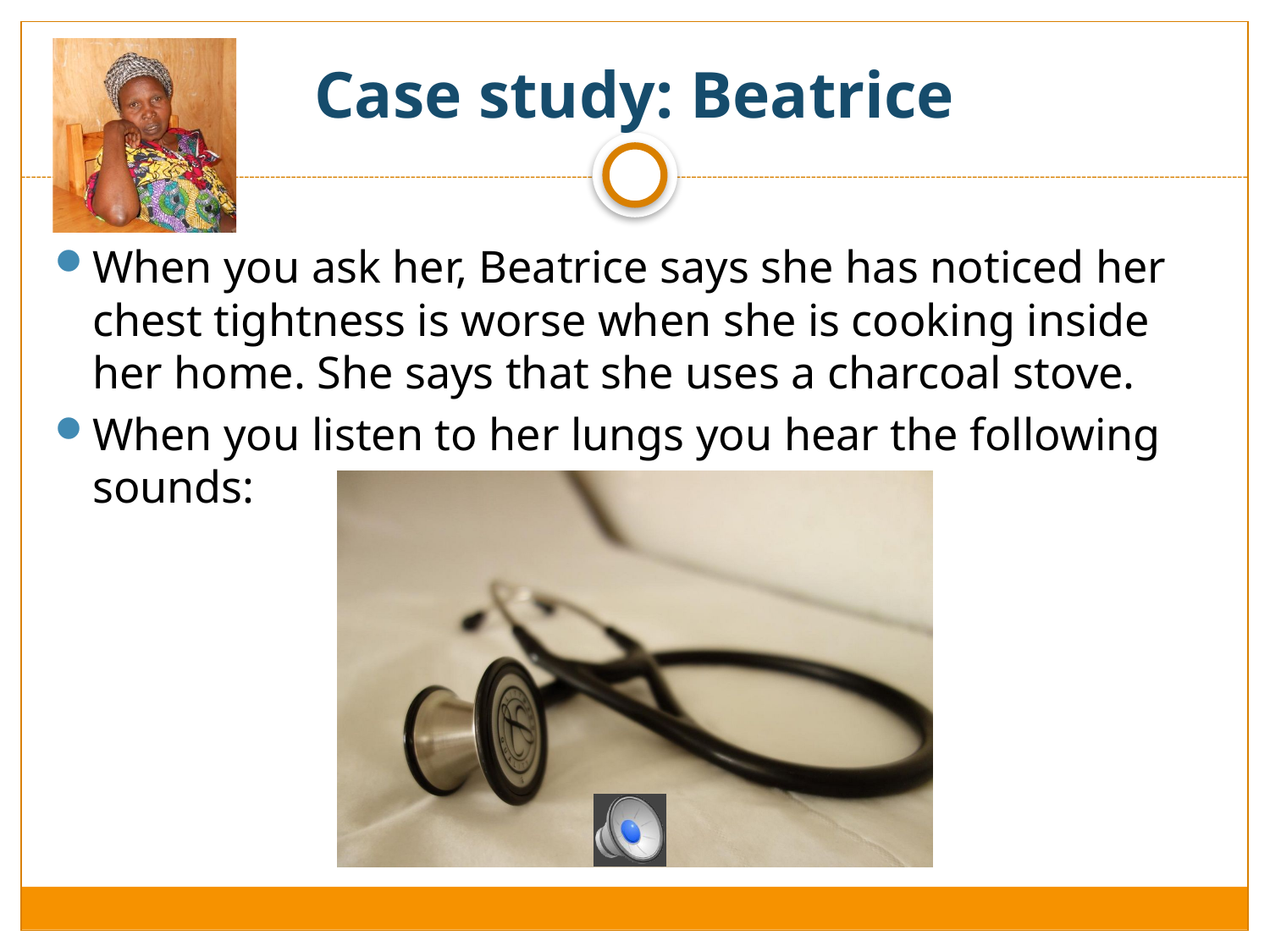

# Case study: Beatrice
When you ask her, Beatrice says she has noticed her chest tightness is worse when she is cooking inside her home. She says that she uses a charcoal stove.
When you listen to her lungs you hear the following sounds:

## Slide 88
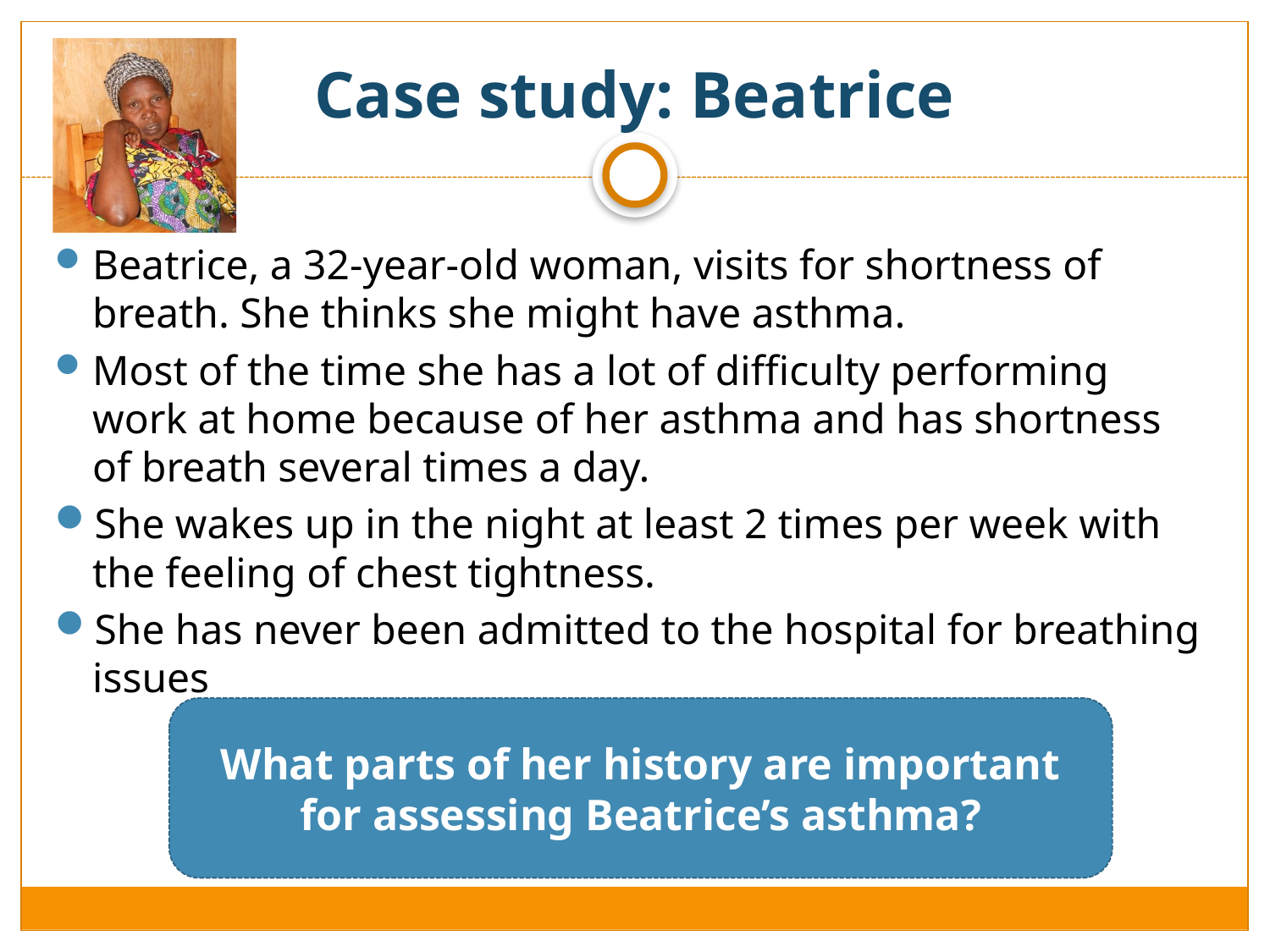

# Case study: Beatrice
Beatrice, a 32-year-old woman, visits for shortness of breath. She thinks she might have asthma.
Most of the time she has a lot of difficulty performing work at home because of her asthma and has shortness of breath several times a day.
She wakes up in the night at least 2 times per week with the feeling of chest tightness.
She has never been admitted to the hospital for breathing issues
What parts of her history are important for assessing Beatrice’s asthma?

## Slide 89
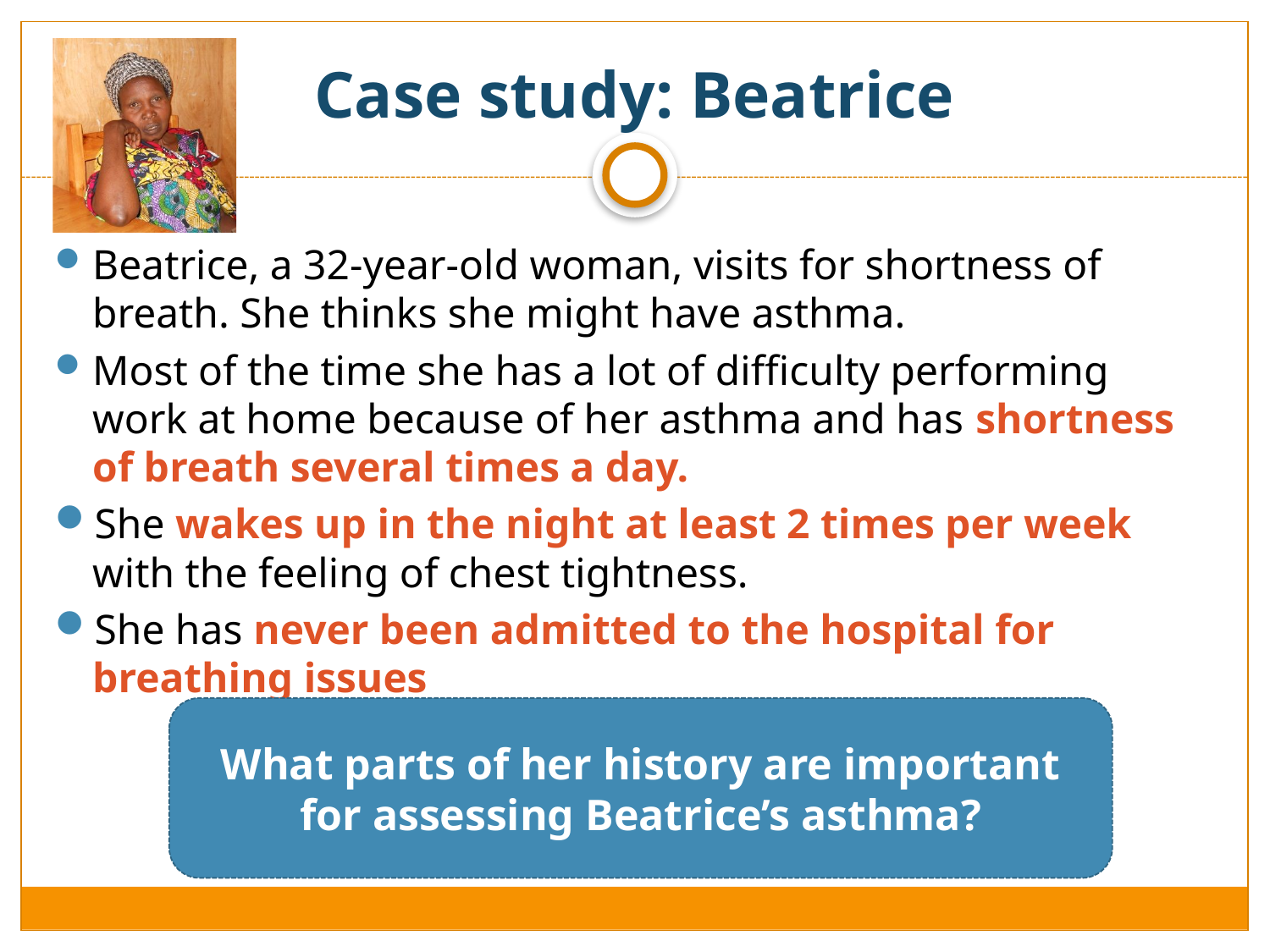

# Case study: Beatrice
Beatrice, a 32-year-old woman, visits for shortness of breath. She thinks she might have asthma.
Most of the time she has a lot of difficulty performing work at home because of her asthma and has shortness of breath several times a day.
She wakes up in the night at least 2 times per week with the feeling of chest tightness.
She has never been admitted to the hospital for breathing issues
What parts of her history are important for assessing Beatrice’s asthma?

## Slide 90
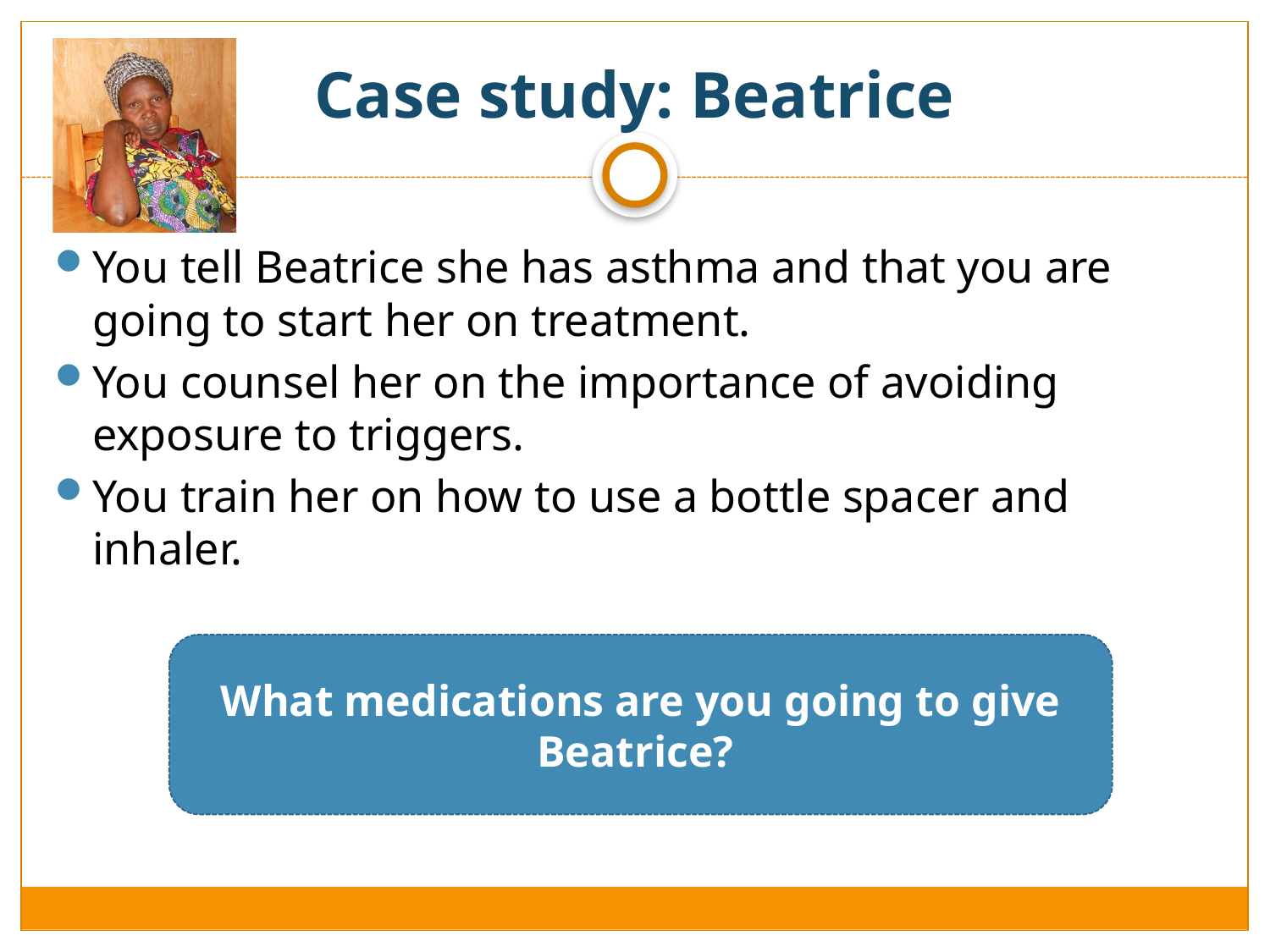

# Case study: Beatrice
You tell Beatrice she has asthma and that you are going to start her on treatment.
You counsel her on the importance of avoiding exposure to triggers.
You train her on how to use a bottle spacer and inhaler.
What medications are you going to give Beatrice?

## Slide 91
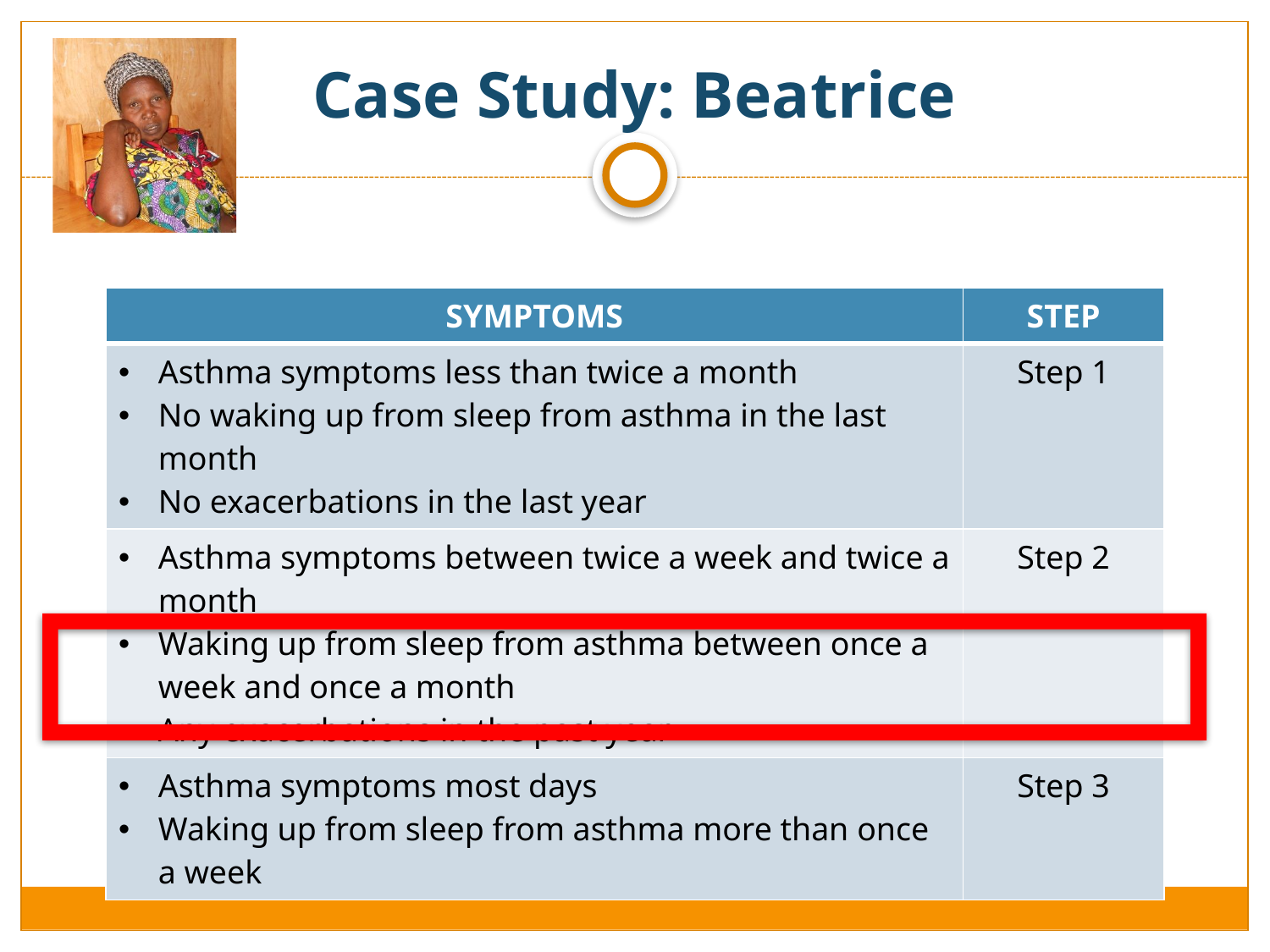

# Case Study: Beatrice
| SYMPTOMS | STEP |
| --- | --- |
| Asthma symptoms less than twice a month No waking up from sleep from asthma in the last month No exacerbations in the last year | Step 1 |
| Asthma symptoms between twice a week and twice a month Waking up from sleep from asthma between once a week and once a month Any exacerbations in the past year | Step 2 |
| Asthma symptoms most days Waking up from sleep from asthma more than once a week | Step 3 |

## Slide 92
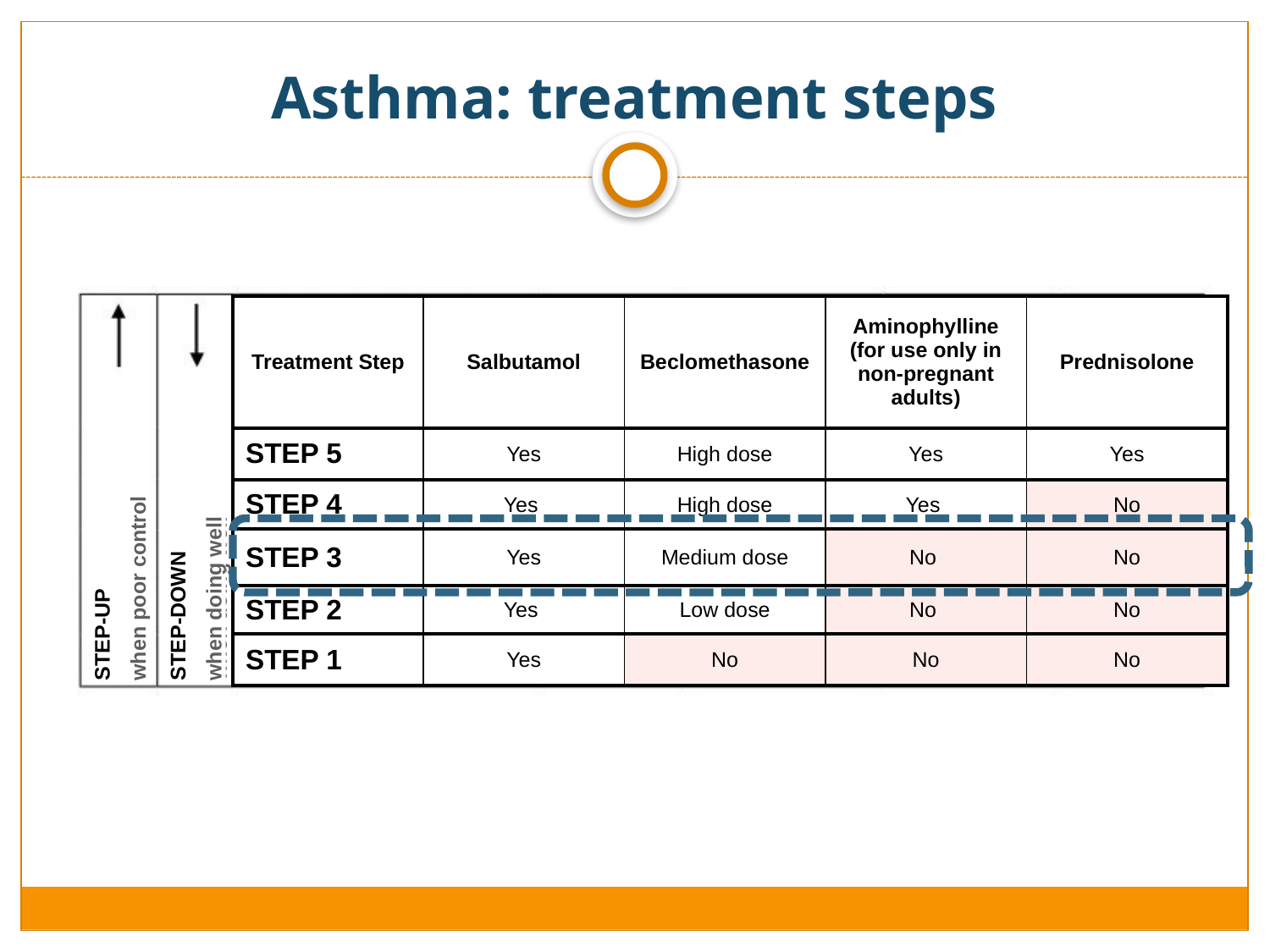

# Asthma: treatment steps
| Treatment Step | Salbutamol | Beclomethasone | Aminophylline (for use only in non-pregnant adults) | Prednisolone |
| --- | --- | --- | --- | --- |
| STEP 5 | Yes | High dose | Yes | Yes |
| STEP 4 | Yes | High dose | Yes | No |
| STEP 3 | Yes | Medium dose | No | No |
| STEP 2 | Yes | Low dose | No | No |
| STEP 1 | Yes | No | No | No |
STEP-UP
when poor control
STEP-UP
when poor control
STEP-DOWN
when doing well
STEP-DOWN
when doing well

## Slide 93
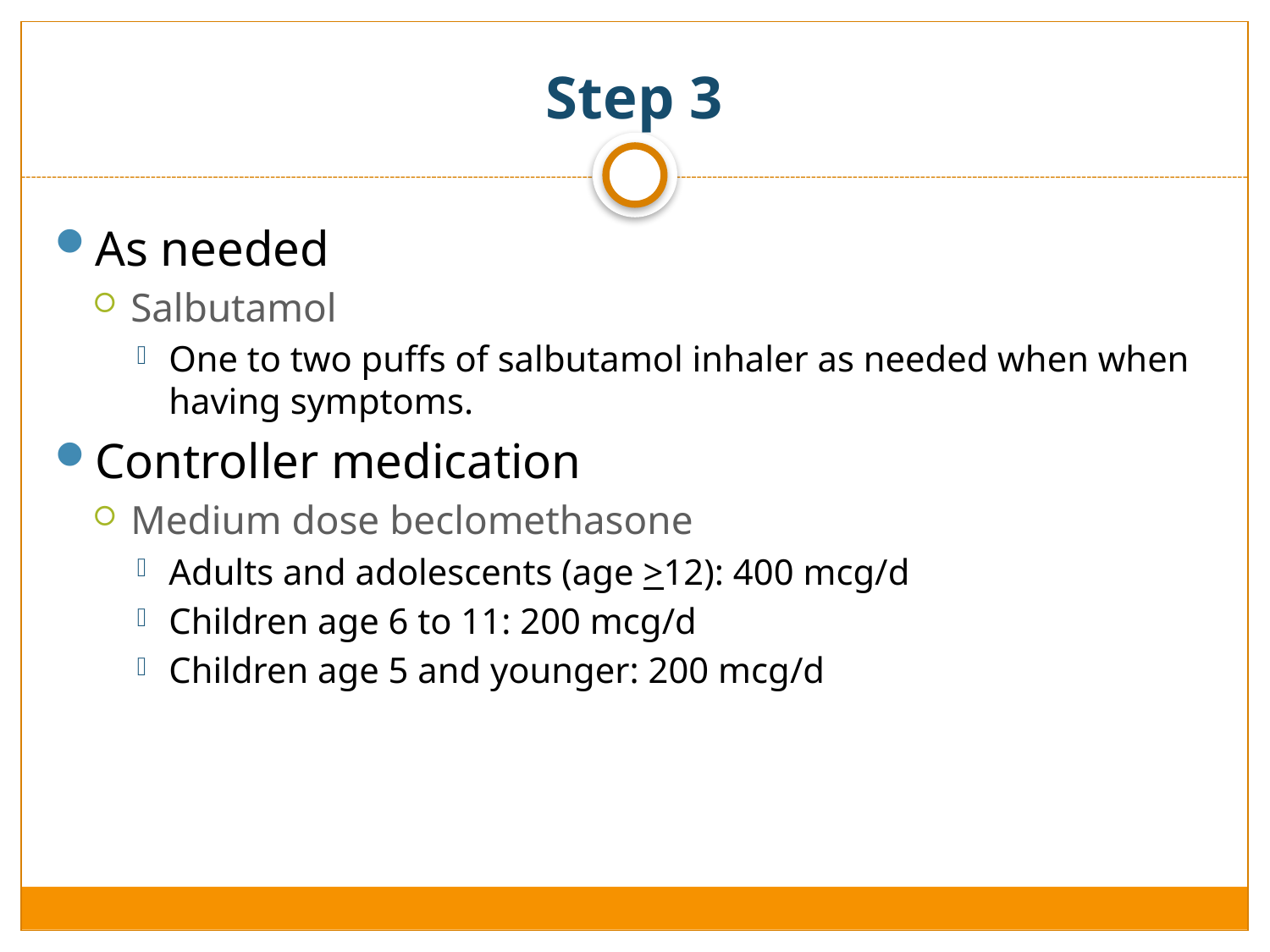

# Step 3
As needed
Salbutamol
One to two puffs of salbutamol inhaler as needed when when having symptoms.
Controller medication
Medium dose beclomethasone
Adults and adolescents (age >12): 400 mcg/d
Children age 6 to 11: 200 mcg/d
Children age 5 and younger: 200 mcg/d

## Slide 94
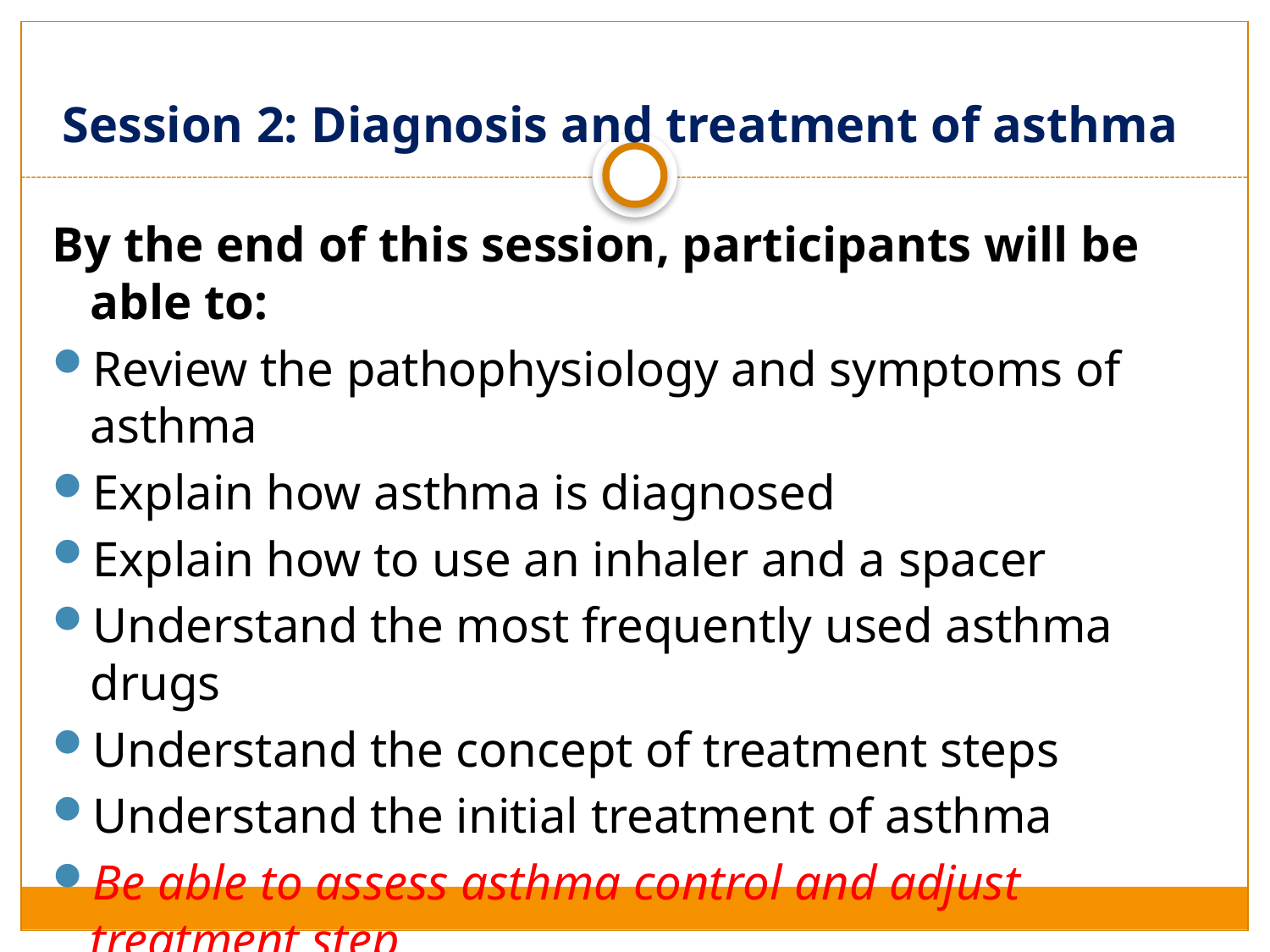

# Session 2: Diagnosis and treatment of asthma
By the end of this session, participants will be able to:
Review the pathophysiology and symptoms of asthma
Explain how asthma is diagnosed
Explain how to use an inhaler and a spacer
Understand the most frequently used asthma drugs
Understand the concept of treatment steps
Understand the initial treatment of asthma
Be able to assess asthma control and adjust treatment step
Be able to assess asthma attacks

## Slide 95
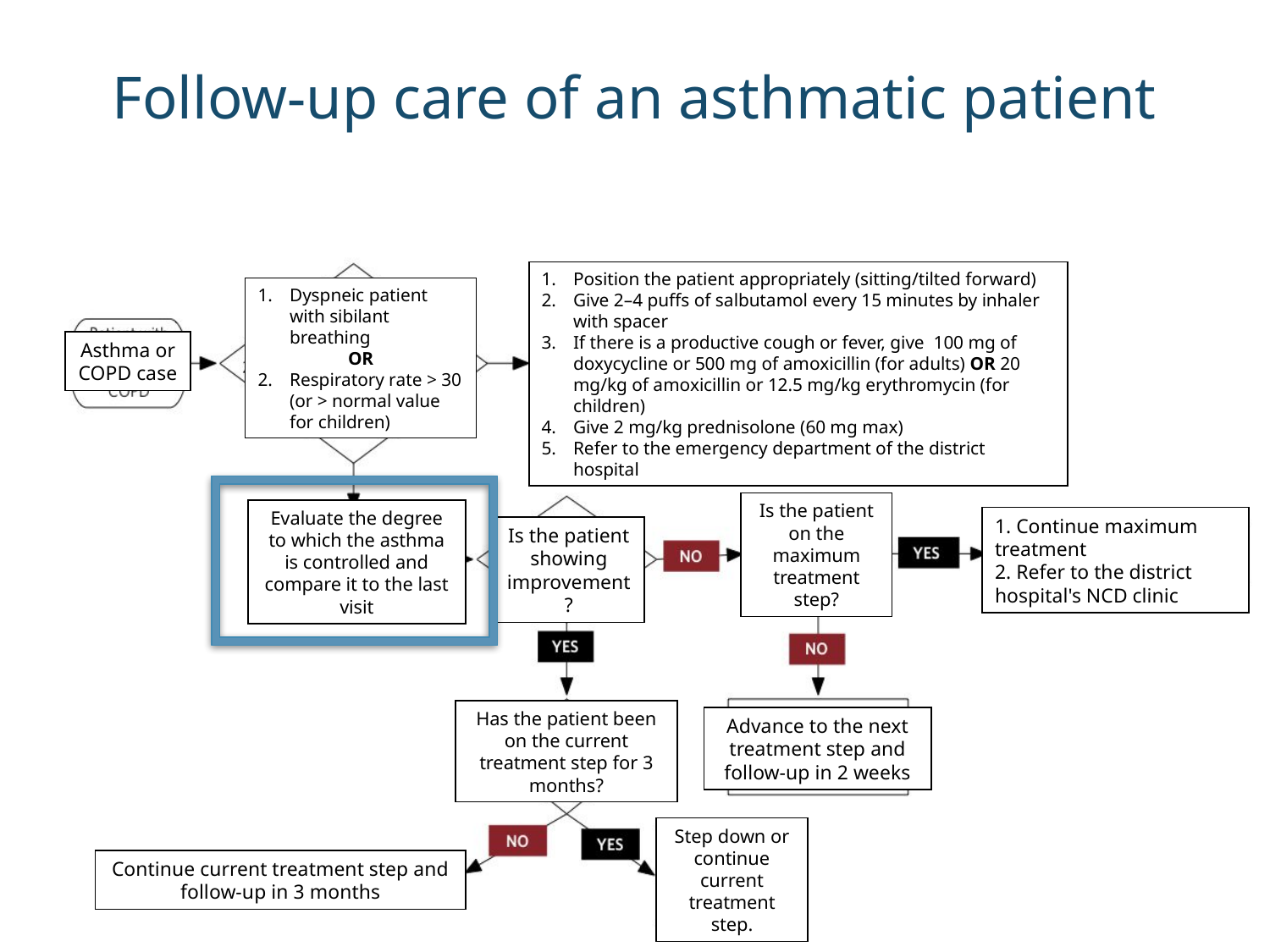

# Follow-up care of an asthmatic patient
Position the patient appropriately (sitting/tilted forward)
Give 2–4 puffs of salbutamol every 15 minutes by inhaler with spacer
If there is a productive cough or fever, give 100 mg of doxycycline or 500 mg of amoxicillin (for adults) OR 20 mg/kg of amoxicillin or 12.5 mg/kg erythromycin (for children)
Give 2 mg/kg prednisolone (60 mg max)
Refer to the emergency department of the district hospital
Dyspneic patient with sibilant breathing
OR
Respiratory rate > 30 (or > normal value for children)
Asthma or COPD case
Is the patient on the maximum treatment step?
1. Continue maximum treatment
2. Refer to the district hospital's NCD clinic
Evaluate the degree to which the asthma is controlled and compare it to the last visit
Is the patient showing improvement?
Advance to the next treatment step and follow-up in 2 weeks
Has the patient been on the current treatment step for 3 months?
Step down or continue current treatment step.
Continue current treatment step and follow-up in 3 months

## Slide 96
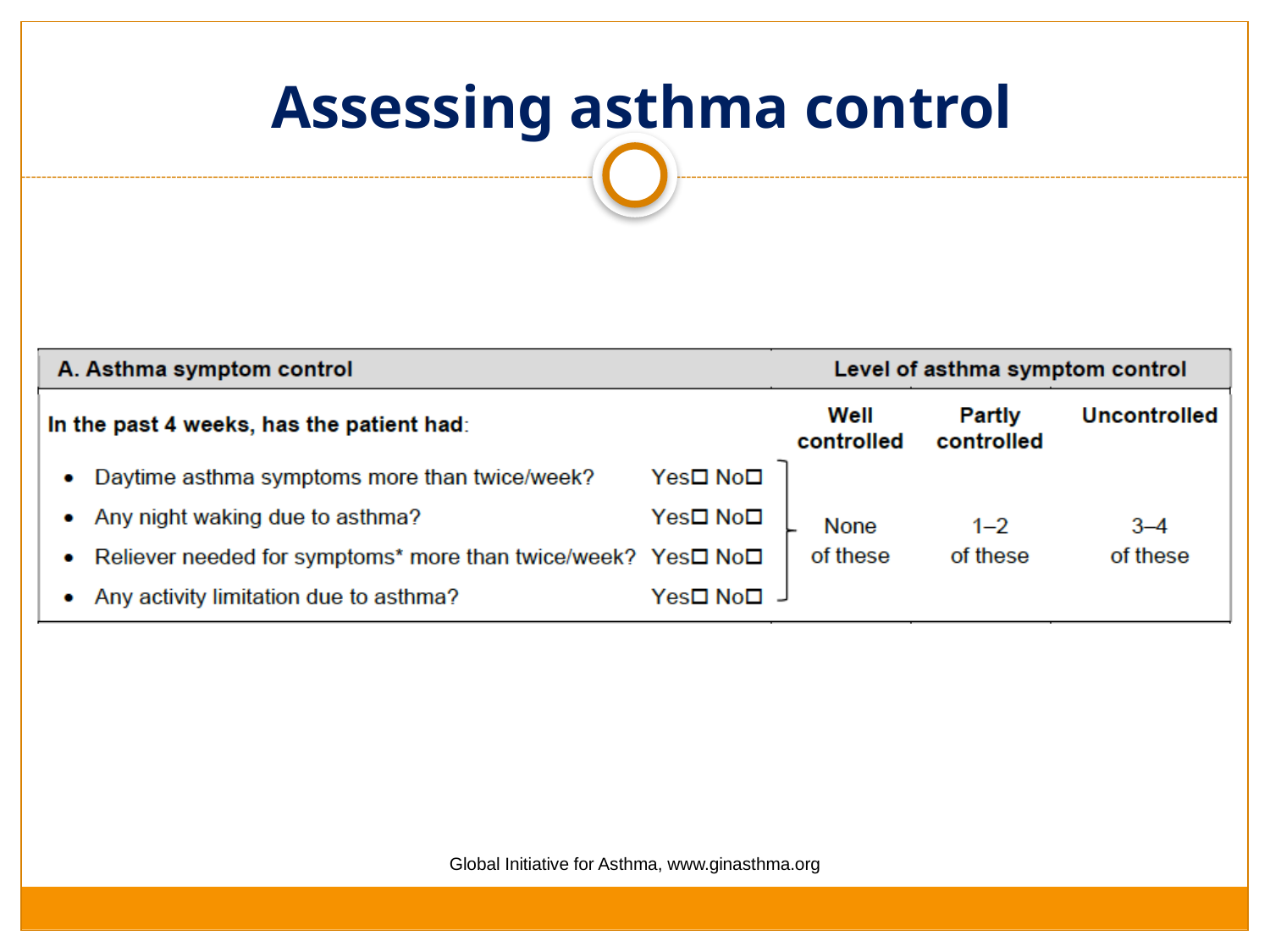

# Assessing asthma control
Global Initiative for Asthma, www.ginasthma.org

## Slide 97
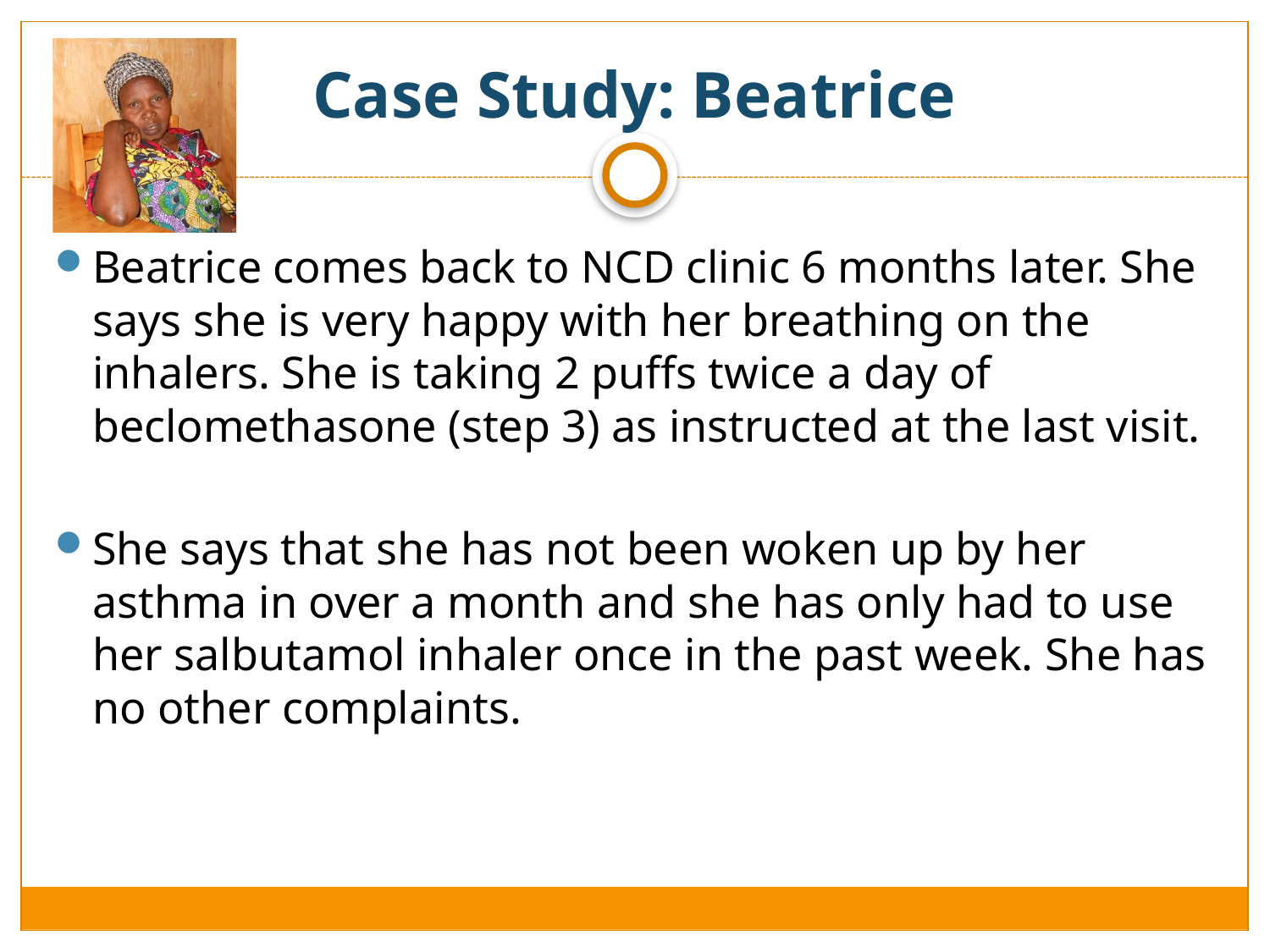

# Case Study: Beatrice
Beatrice comes back to NCD clinic 6 months later. She says she is very happy with her breathing on the inhalers. She is taking 2 puffs twice a day of beclomethasone (step 3) as instructed at the last visit.
She says that she has not been woken up by her asthma in over a month and she has only had to use her salbutamol inhaler once in the past week. She has no other complaints.

## Slide 98
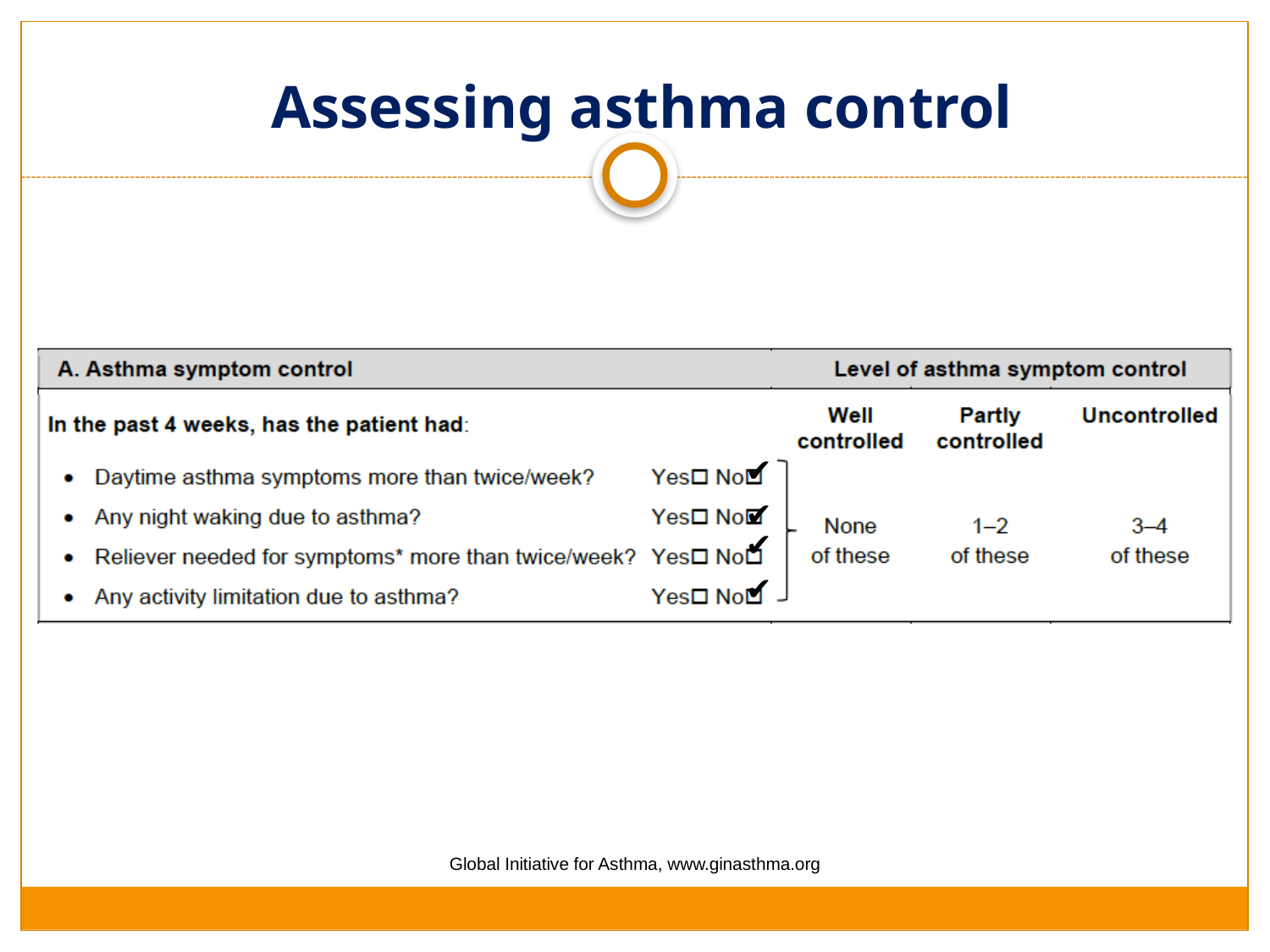

# Assessing asthma control
✔
✔
✔
✔
Global Initiative for Asthma, www.ginasthma.org

## Slide 99
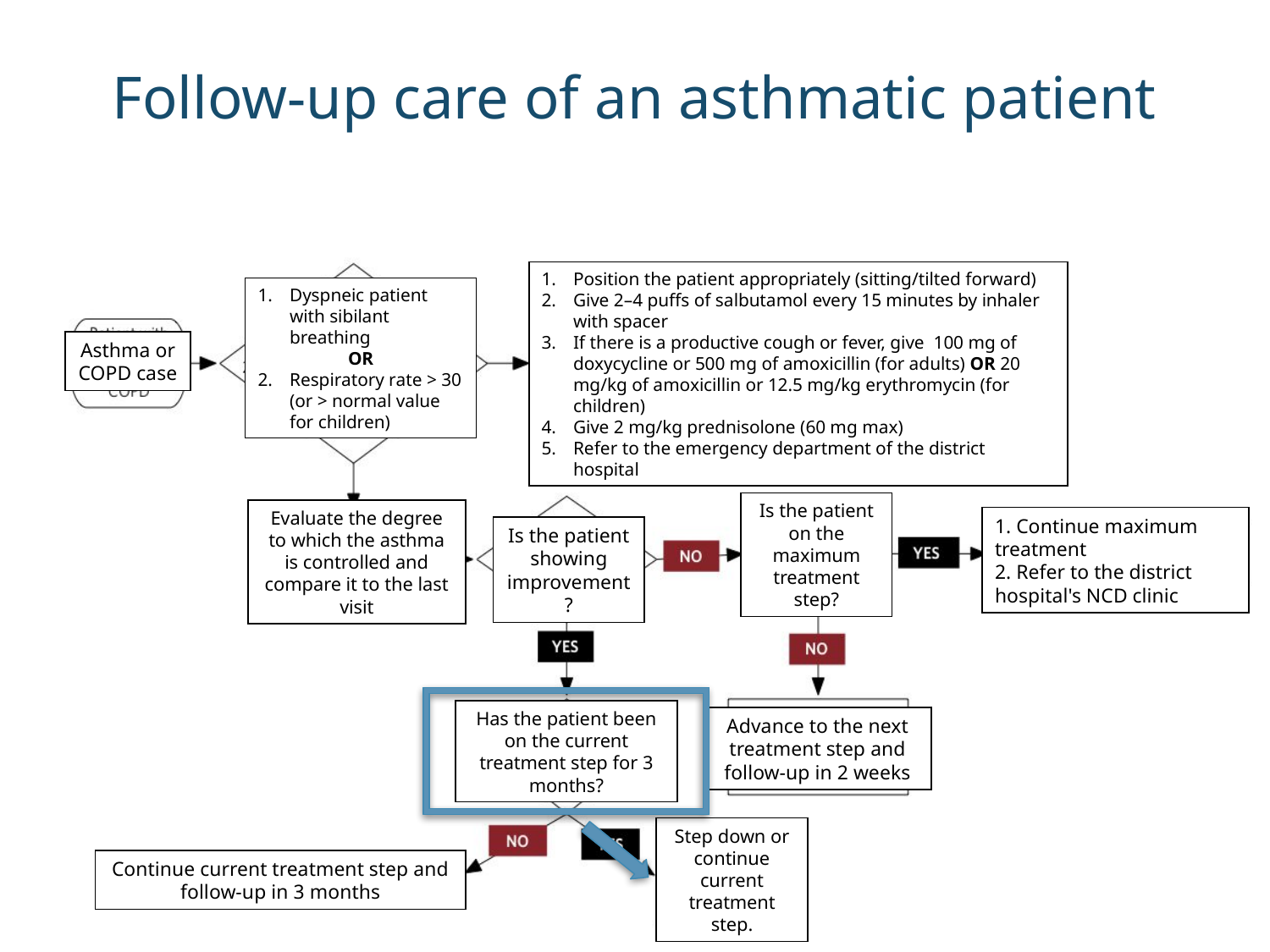

# Follow-up care of an asthmatic patient
Position the patient appropriately (sitting/tilted forward)
Give 2–4 puffs of salbutamol every 15 minutes by inhaler with spacer
If there is a productive cough or fever, give 100 mg of doxycycline or 500 mg of amoxicillin (for adults) OR 20 mg/kg of amoxicillin or 12.5 mg/kg erythromycin (for children)
Give 2 mg/kg prednisolone (60 mg max)
Refer to the emergency department of the district hospital
Dyspneic patient with sibilant breathing
OR
Respiratory rate > 30 (or > normal value for children)
Asthma or COPD case
Is the patient on the maximum treatment step?
1. Continue maximum treatment
2. Refer to the district hospital's NCD clinic
Evaluate the degree to which the asthma is controlled and compare it to the last visit
Is the patient showing improvement?
Advance to the next treatment step and follow-up in 2 weeks
Has the patient been on the current treatment step for 3 months?
Step down or continue current treatment step.
Continue current treatment step and follow-up in 3 months

## Slide 100
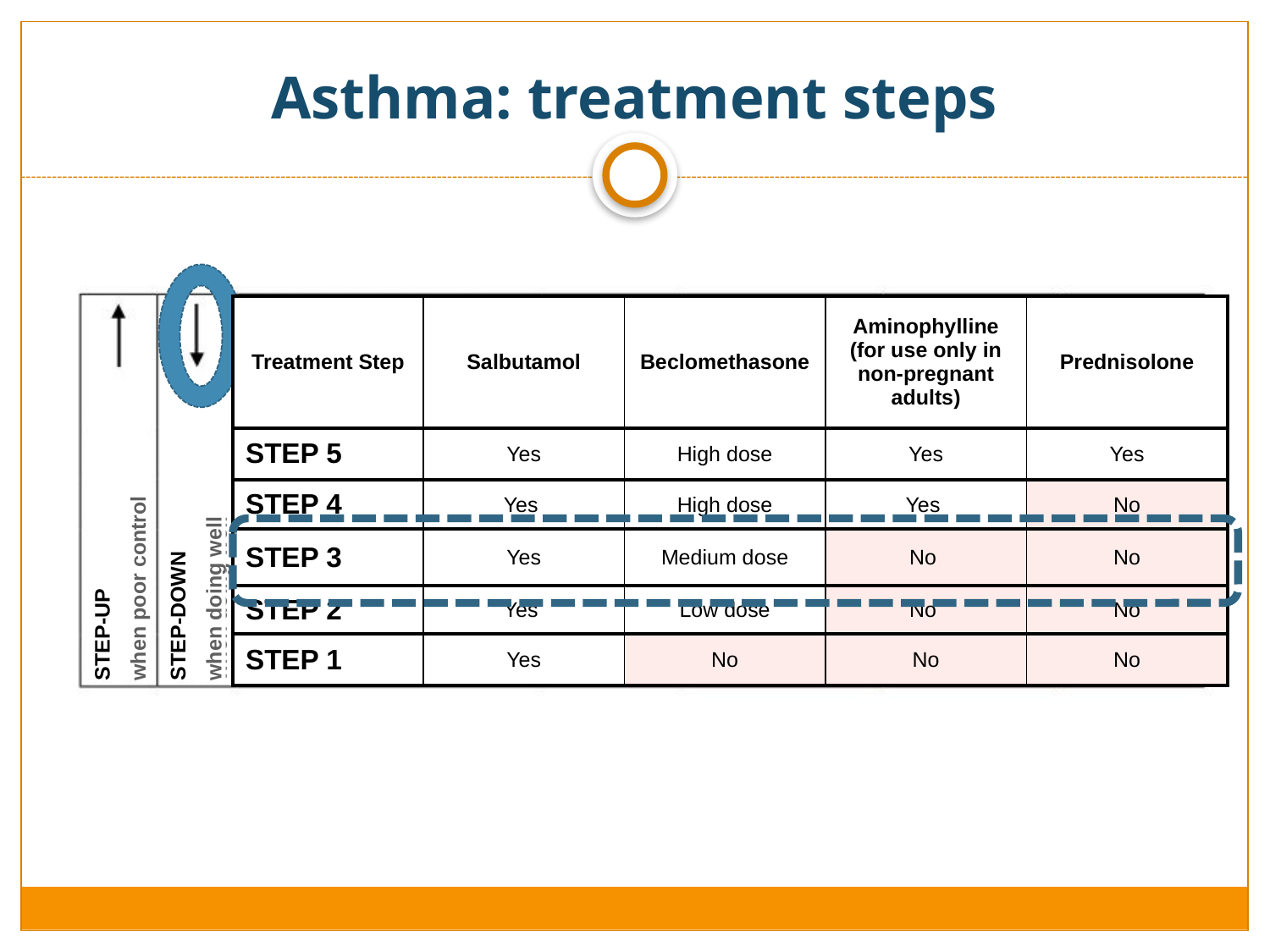

# Asthma: treatment steps
| Treatment Step | Salbutamol | Beclomethasone | Aminophylline (for use only in non-pregnant adults) | Prednisolone |
| --- | --- | --- | --- | --- |
| STEP 5 | Yes | High dose | Yes | Yes |
| STEP 4 | Yes | High dose | Yes | No |
| STEP 3 | Yes | Medium dose | No | No |
| STEP 2 | Yes | Low dose | No | No |
| STEP 1 | Yes | No | No | No |
STEP-UP
when poor control
STEP-UP
when poor control
STEP-DOWN
when doing well
STEP-DOWN
when doing well

## Slide 101
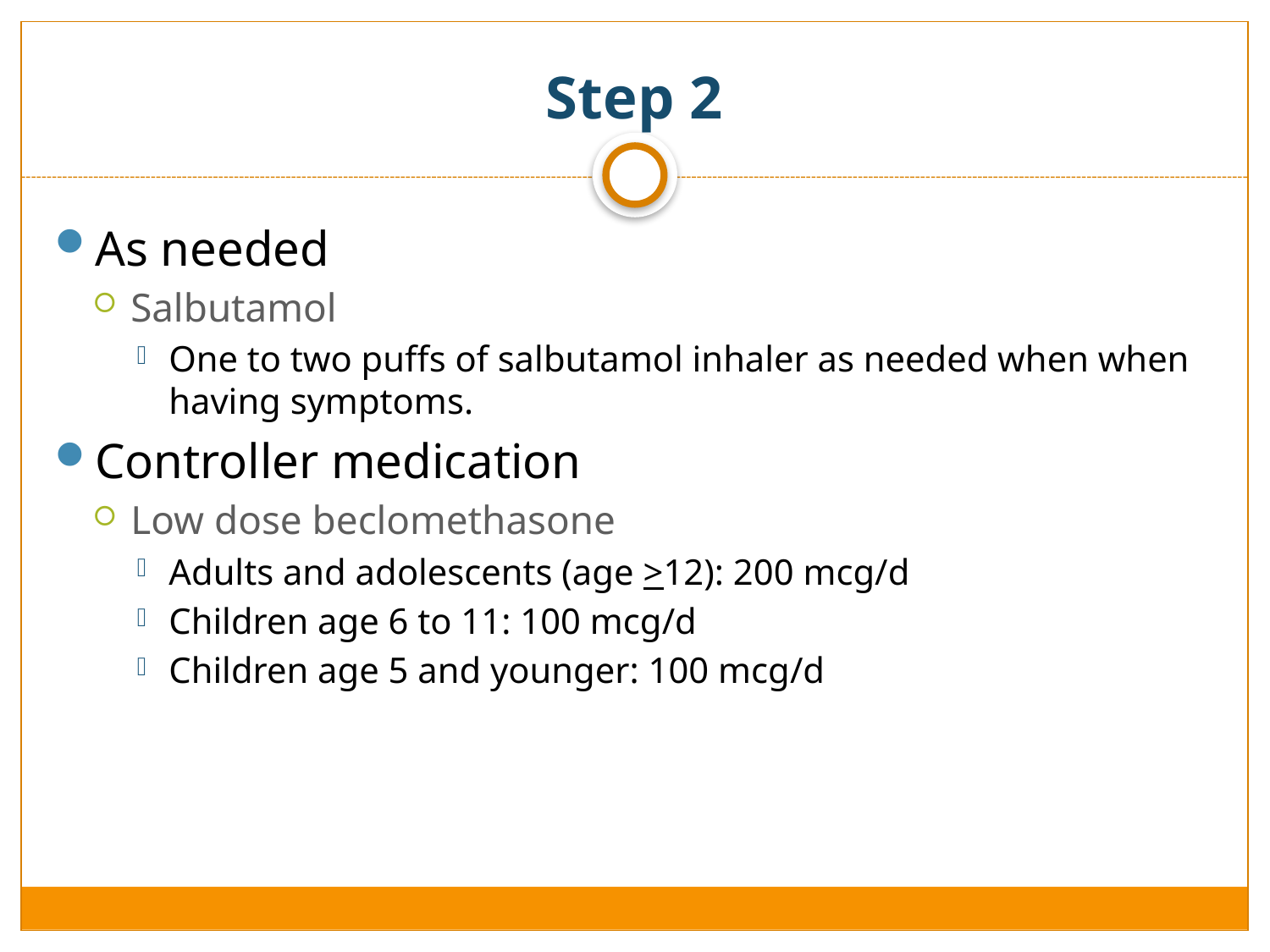

# Step 2
As needed
Salbutamol
One to two puffs of salbutamol inhaler as needed when when having symptoms.
Controller medication
Low dose beclomethasone
Adults and adolescents (age >12): 200 mcg/d
Children age 6 to 11: 100 mcg/d
Children age 5 and younger: 100 mcg/d

## Slide 102
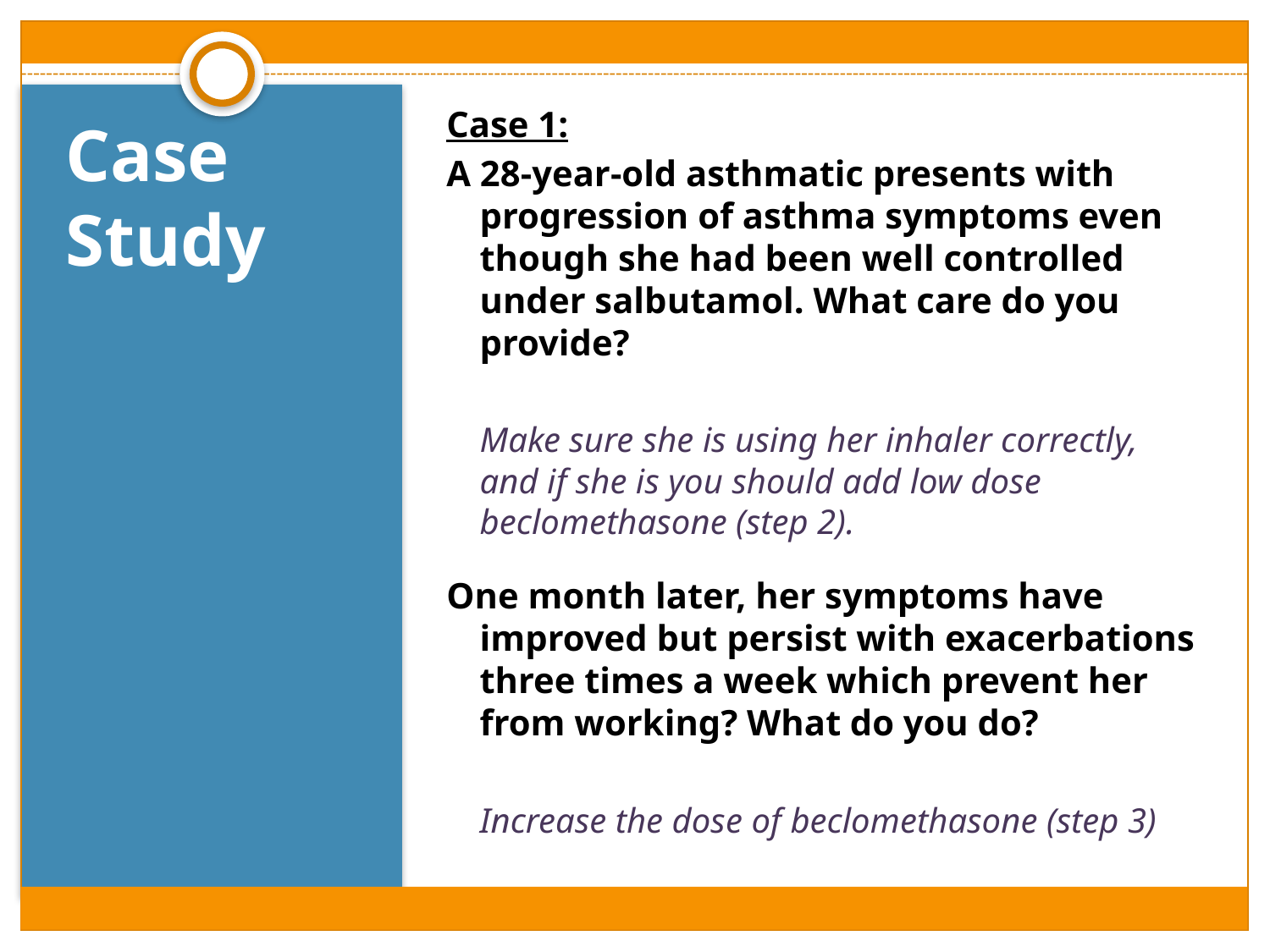

Case 1:
A 28-year-old asthmatic presents with progression of asthma symptoms even though she had been well controlled under salbutamol. What care do you provide?
	Make sure she is using her inhaler correctly, and if she is you should add low dose beclomethasone (step 2).
One month later, her symptoms have improved but persist with exacerbations three times a week which prevent her from working? What do you do?
	Increase the dose of beclomethasone (step 3)
# Case Study

## Slide 103
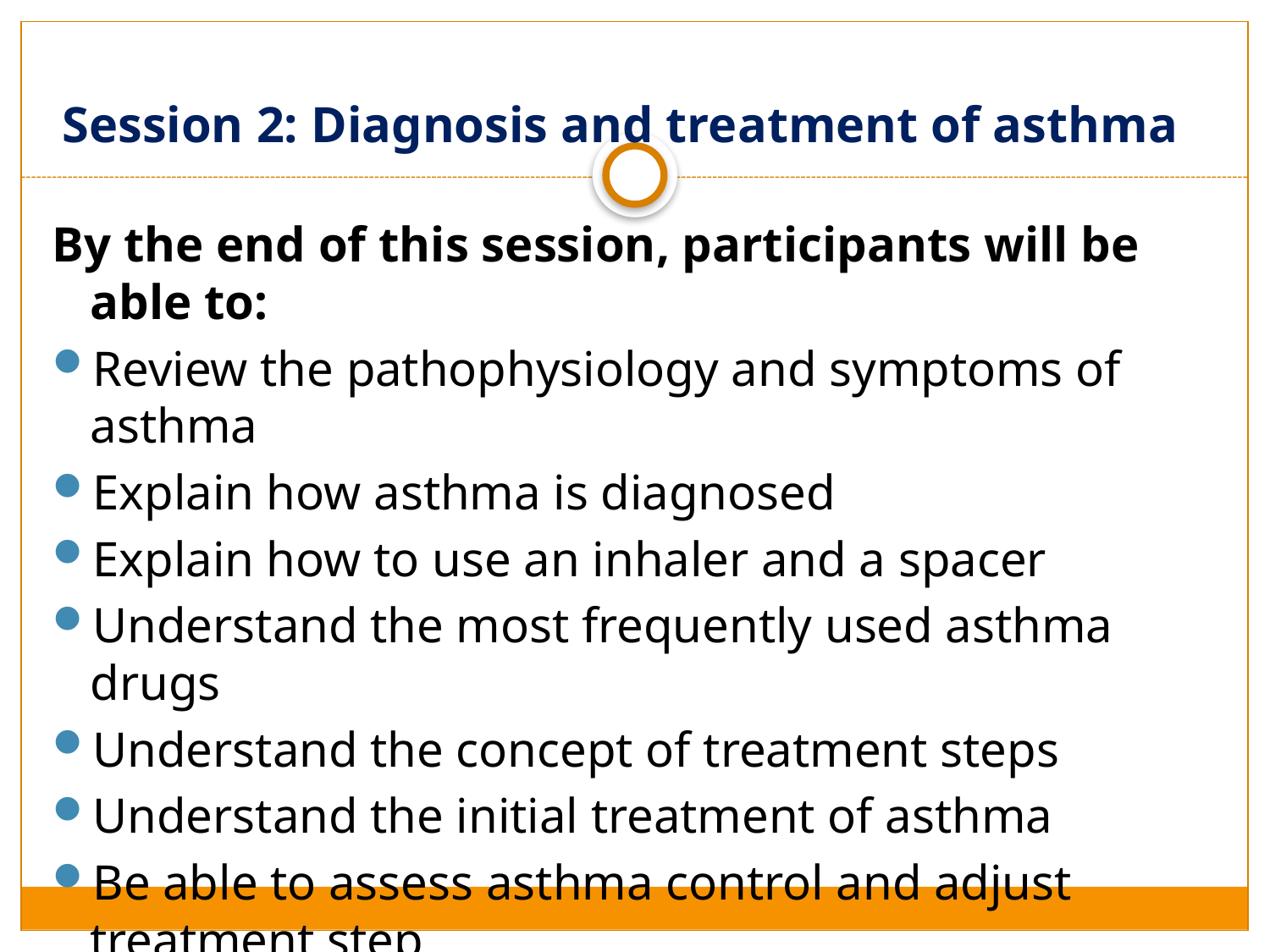

# Session 2: Diagnosis and treatment of asthma
By the end of this session, participants will be able to:
Review the pathophysiology and symptoms of asthma
Explain how asthma is diagnosed
Explain how to use an inhaler and a spacer
Understand the most frequently used asthma drugs
Understand the concept of treatment steps
Understand the initial treatment of asthma
Be able to assess asthma control and adjust treatment step
Be able to assess asthma attacks

## Slide 104
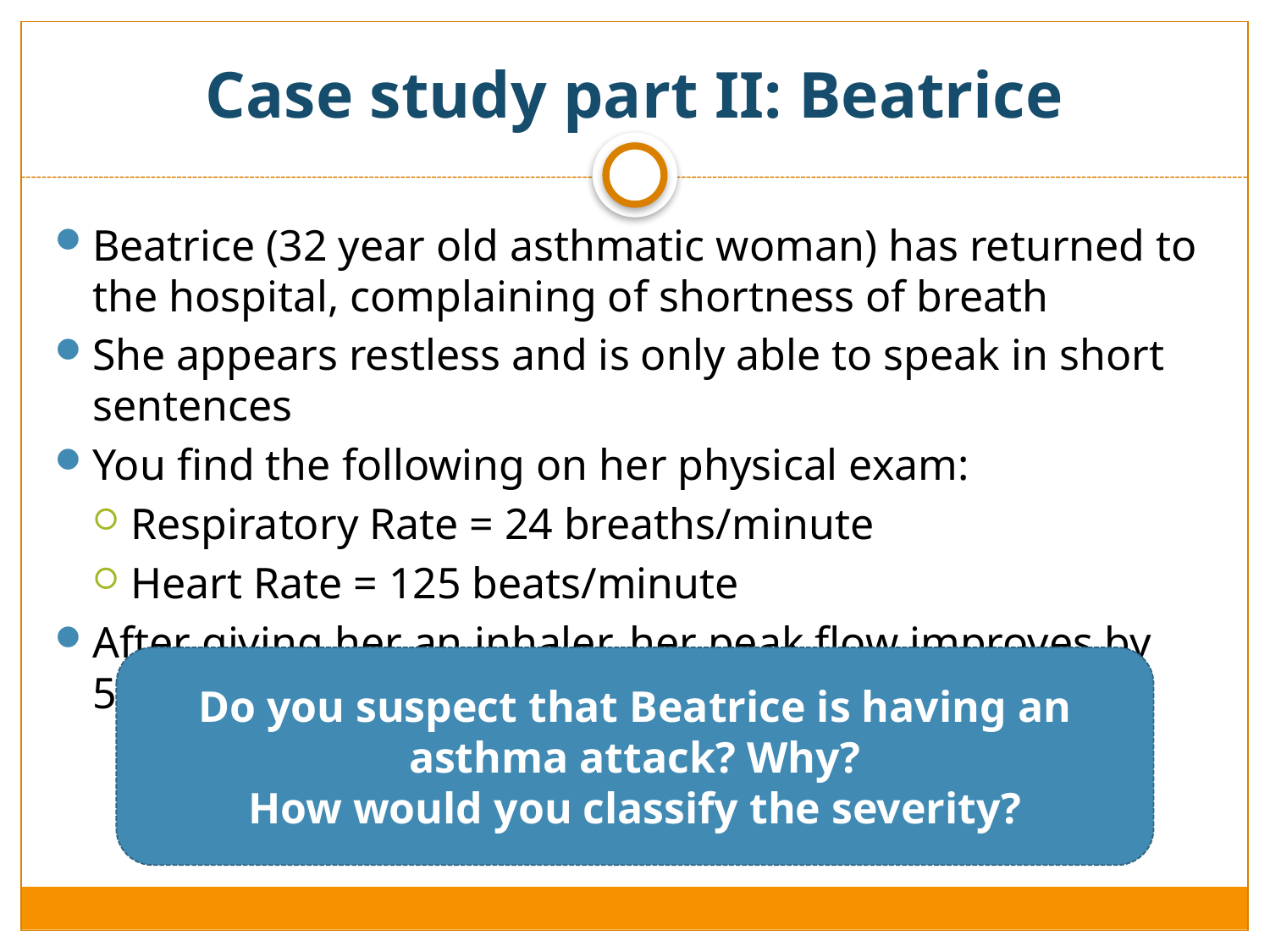

# Case study part II: Beatrice
Beatrice (32 year old asthmatic woman) has returned to the hospital, complaining of shortness of breath
She appears restless and is only able to speak in short sentences
You find the following on her physical exam:
Respiratory Rate = 24 breaths/minute
Heart Rate = 125 beats/minute
After giving her an inhaler, her peak flow improves by 50%
Do you suspect that Beatrice is having an asthma attack? Why?
How would you classify the severity?

## Slide 105
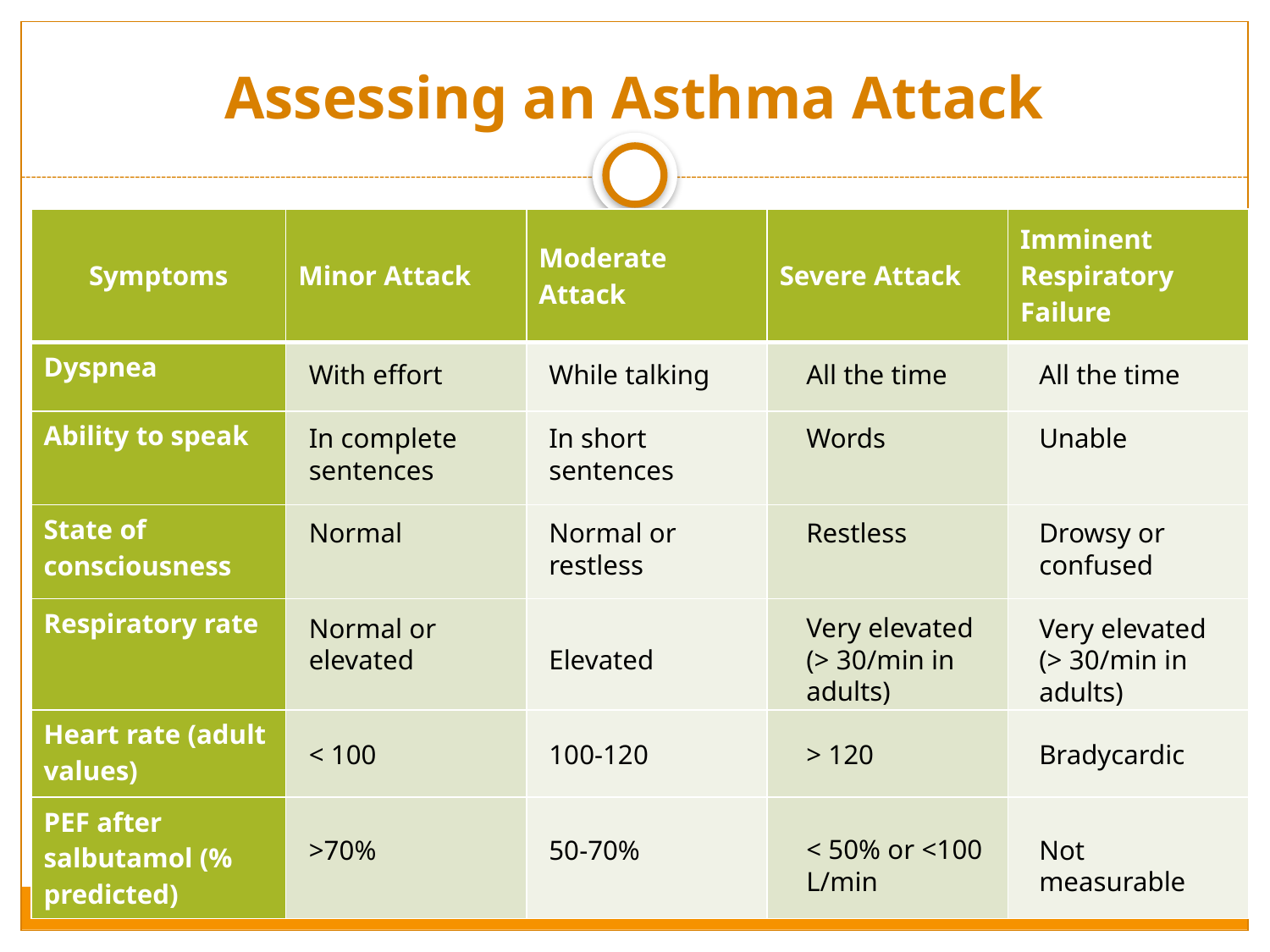

# Assessing an Asthma Attack
| Symptoms | Minor Attack | Moderate Attack | Severe Attack | Imminent Respiratory Failure |
| --- | --- | --- | --- | --- |
| Dyspnea | | | | |
| Ability to speak | | | | |
| State of consciousness | | | | |
| Respiratory rate | | | | |
| Heart rate (adult values) | | | | |
| PEF after salbutamol (% predicted) | | | | |
All the time
Words
Restless
Very elevated (> 30/min in adults)
> 120
< 50% or <100 L/min
With effort
In complete sentences
Normal
Normal or elevated
< 100
>70%
While talking
In short sentences
Normal or restless
Elevated
100-120
50-70%
All the time
Unable
Drowsy or confused
Very elevated (> 30/min in adults)
Bradycardic
Not measurable

## Slide 106
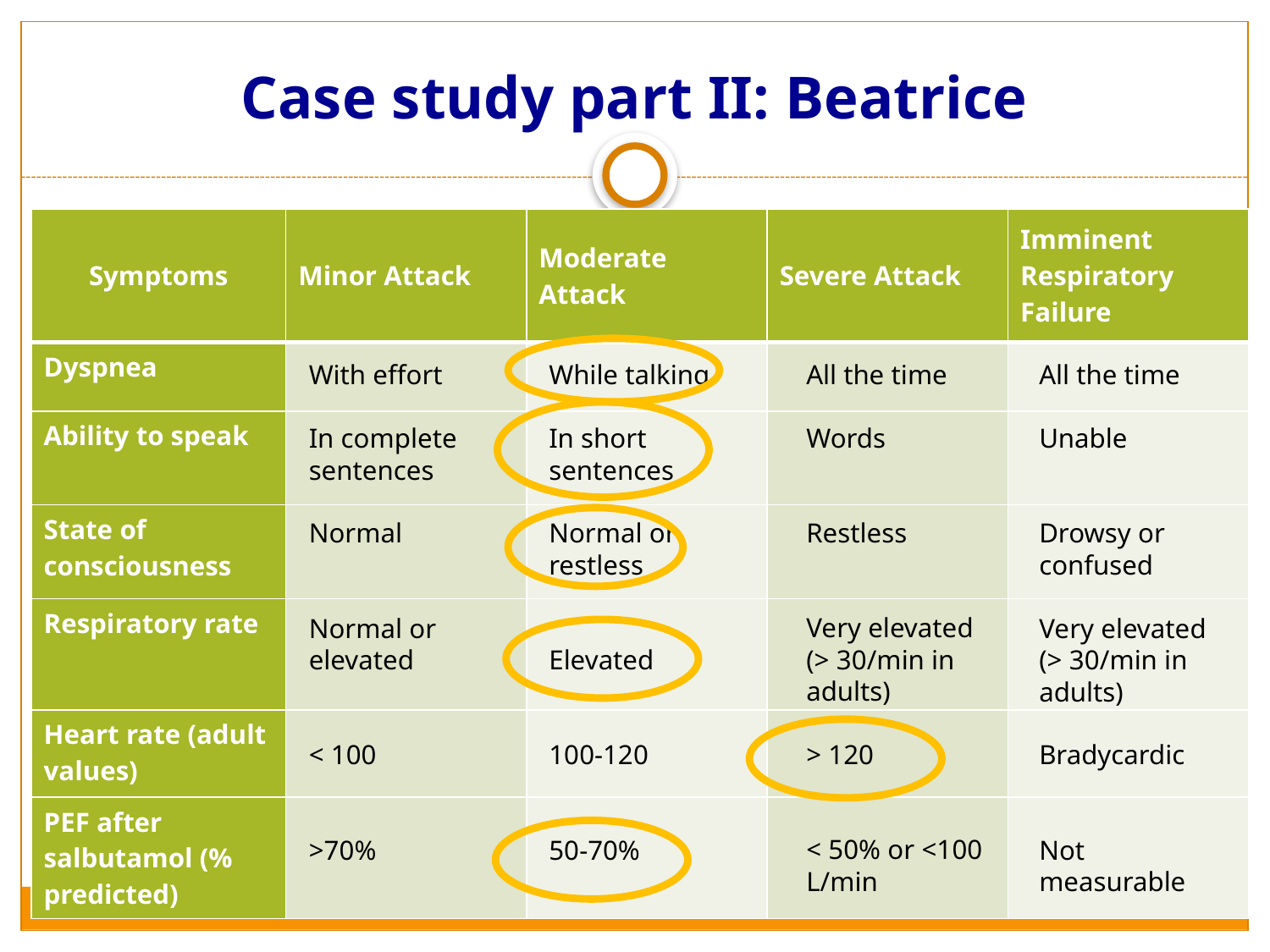

# Case study part II: Beatrice
| Symptoms | Minor Attack | Moderate Attack | Severe Attack | Imminent Respiratory Failure |
| --- | --- | --- | --- | --- |
| Dyspnea | | | | |
| Ability to speak | | | | |
| State of consciousness | | | | |
| Respiratory rate | | | | |
| Heart rate (adult values) | | | | |
| PEF after salbutamol (% predicted) | | | | |
All the time
Words
Restless
Very elevated (> 30/min in adults)
> 120
< 50% or <100 L/min
With effort
In complete sentences
Normal
Normal or elevated
< 100
>70%
While talking
In short sentences
Normal or restless
Elevated
100-120
50-70%
All the time
Unable
Drowsy or confused
Very elevated (> 30/min in adults)
Bradycardic
Not measurable

## Slide 107
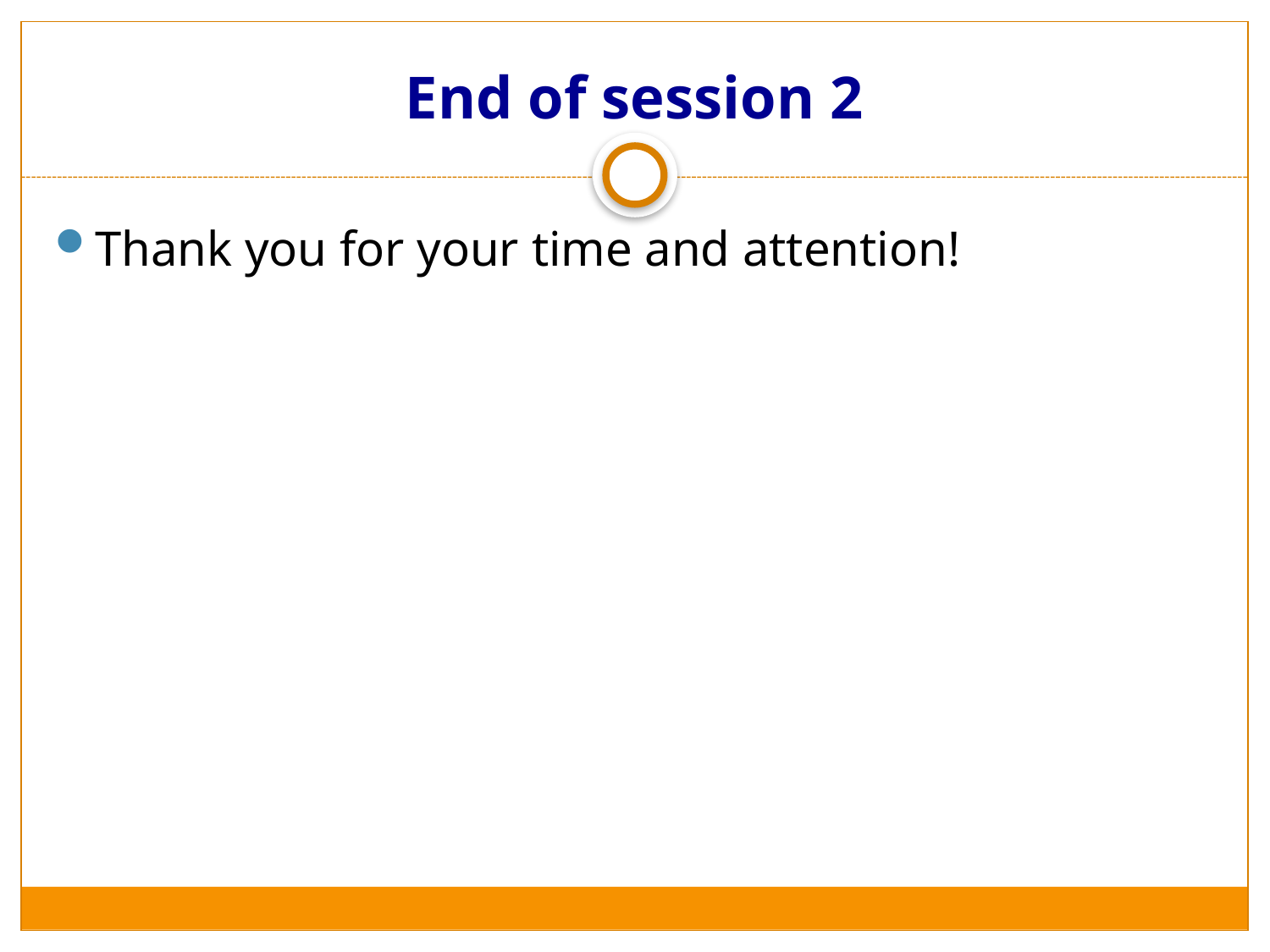

# End of session 2
Thank you for your time and attention!

## Slide 108
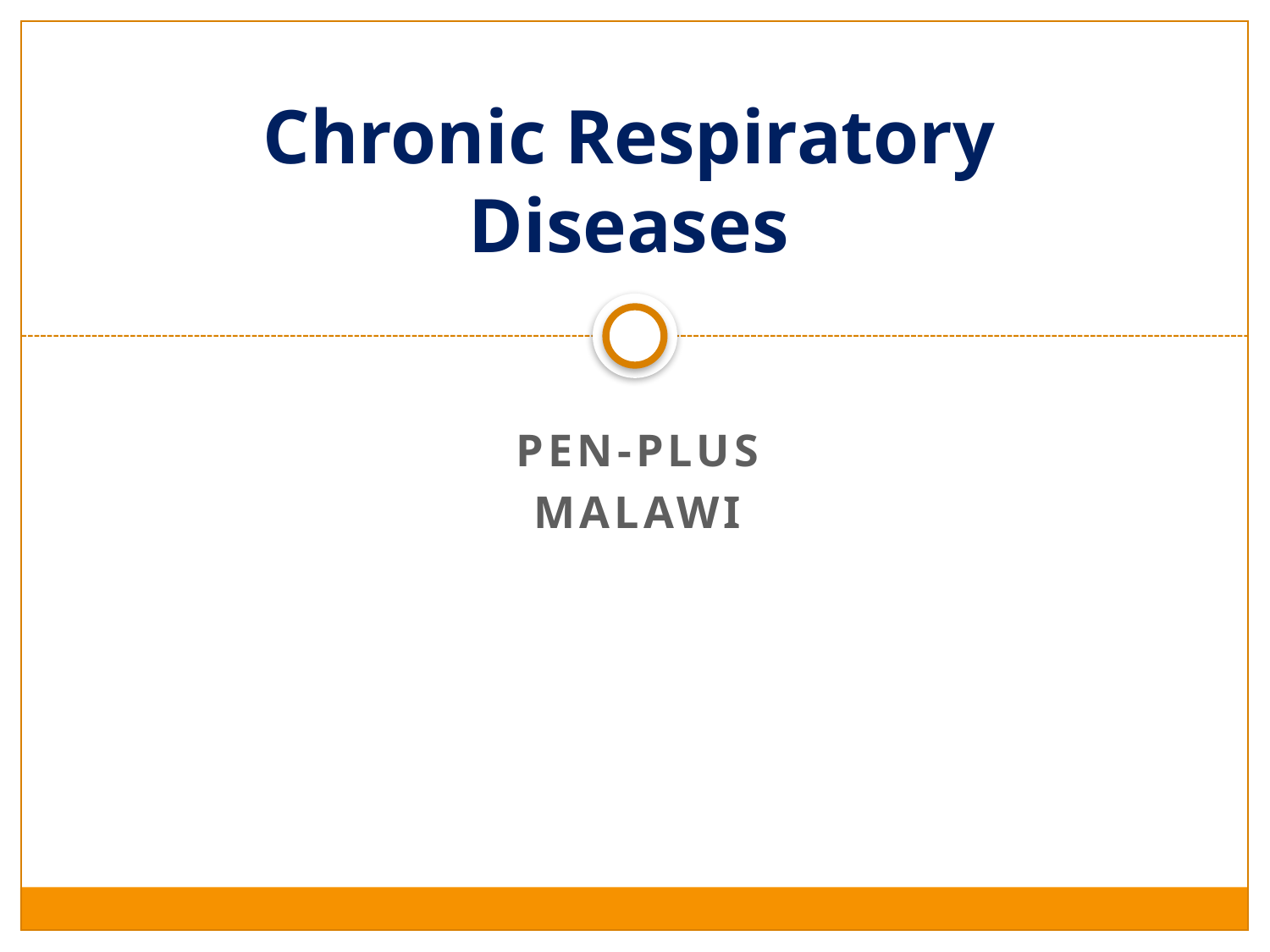

# Chronic Respiratory Diseases
PeN-Plus
Malawi

## Slide 109
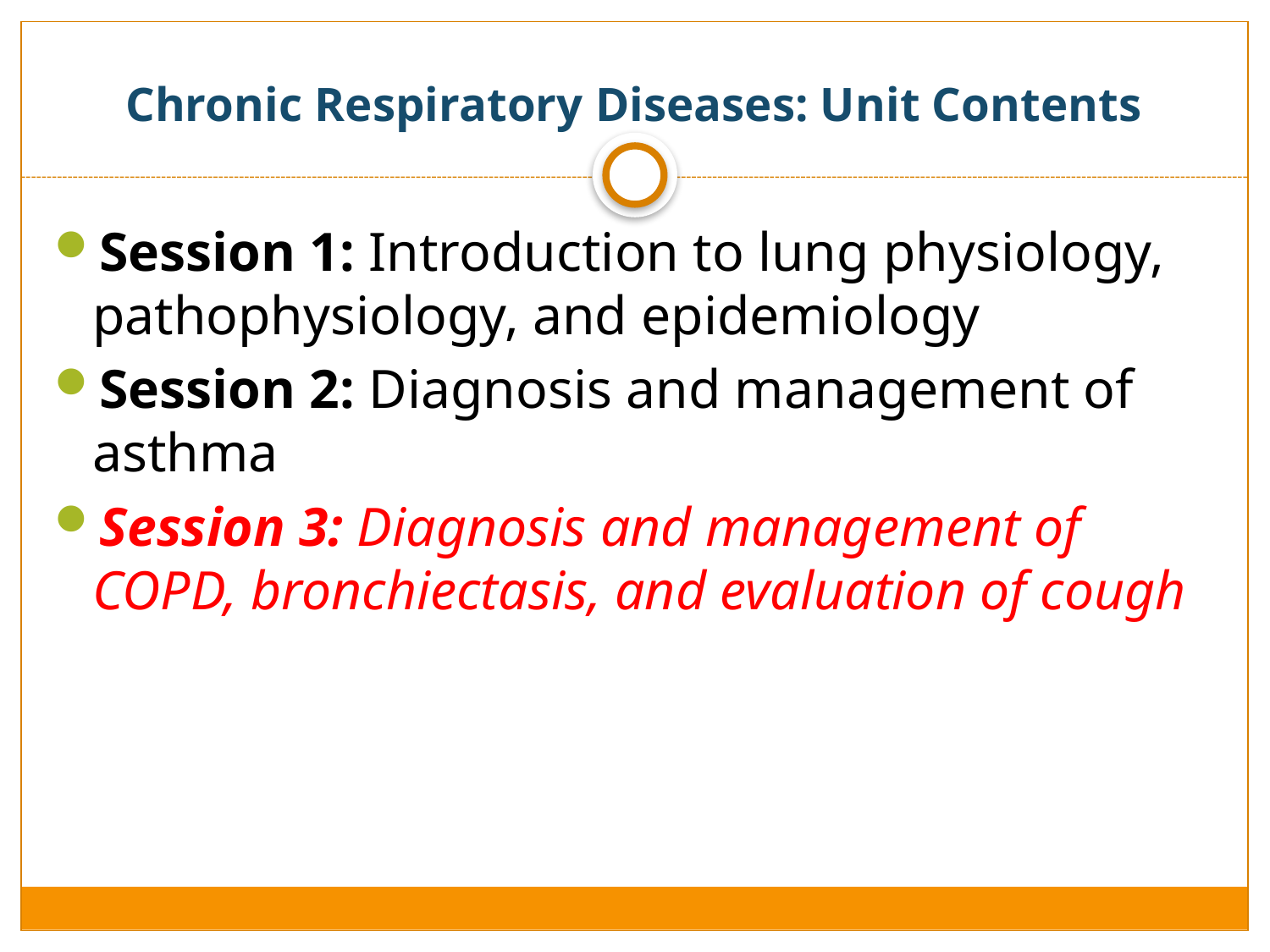

# Chronic Respiratory Diseases: Unit Contents
Session 1: Introduction to lung physiology, pathophysiology, and epidemiology
Session 2: Diagnosis and management of asthma
Session 3: Diagnosis and management of COPD, bronchiectasis, and evaluation of cough

## Slide 110
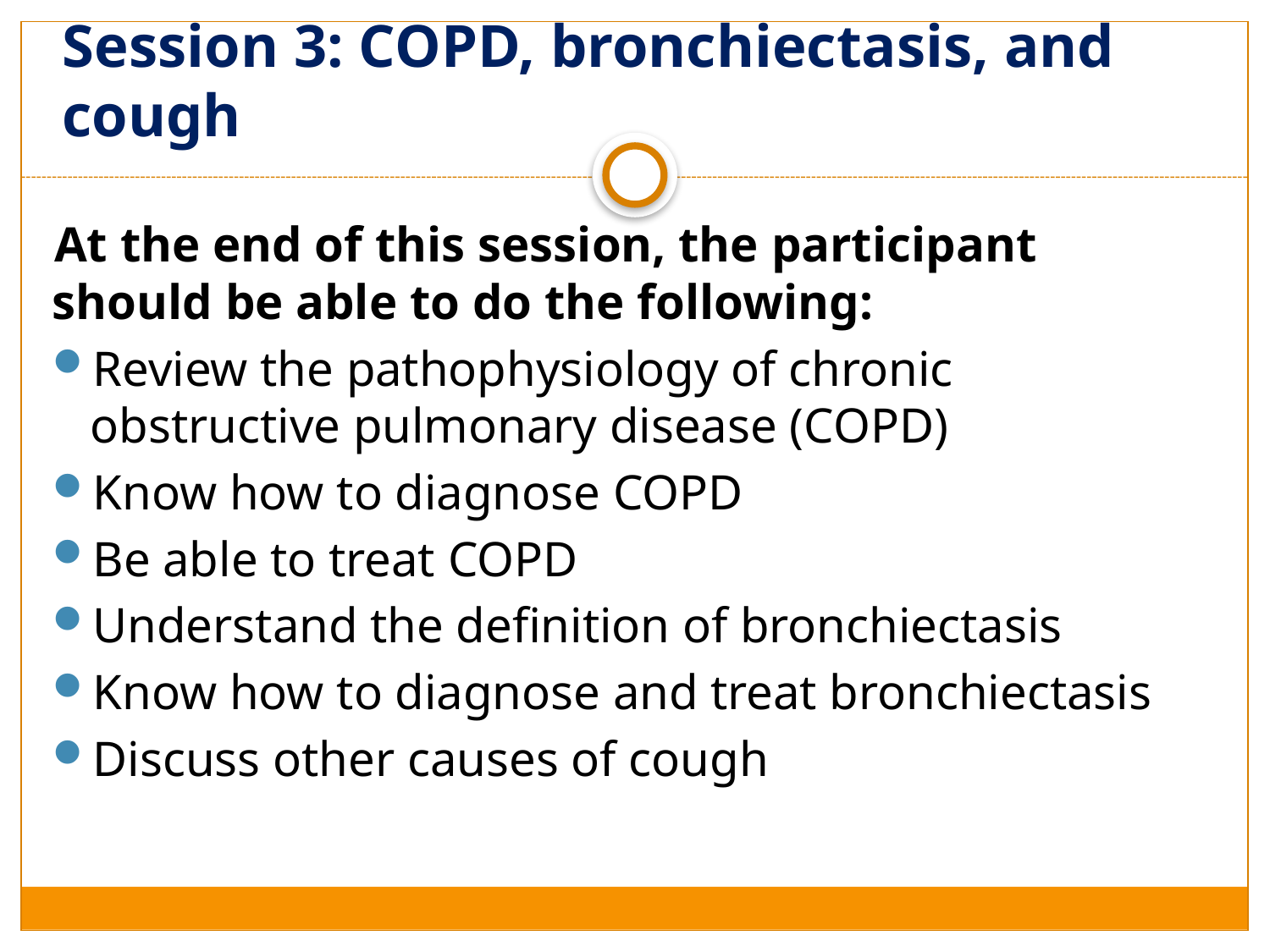

# Session 3: COPD, bronchiectasis, and cough
At the end of this session, the participant should be able to do the following:
Review the pathophysiology of chronic obstructive pulmonary disease (COPD)
Know how to diagnose COPD
Be able to treat COPD
Understand the definition of bronchiectasis
Know how to diagnose and treat bronchiectasis
Discuss other causes of cough

## Slide 111
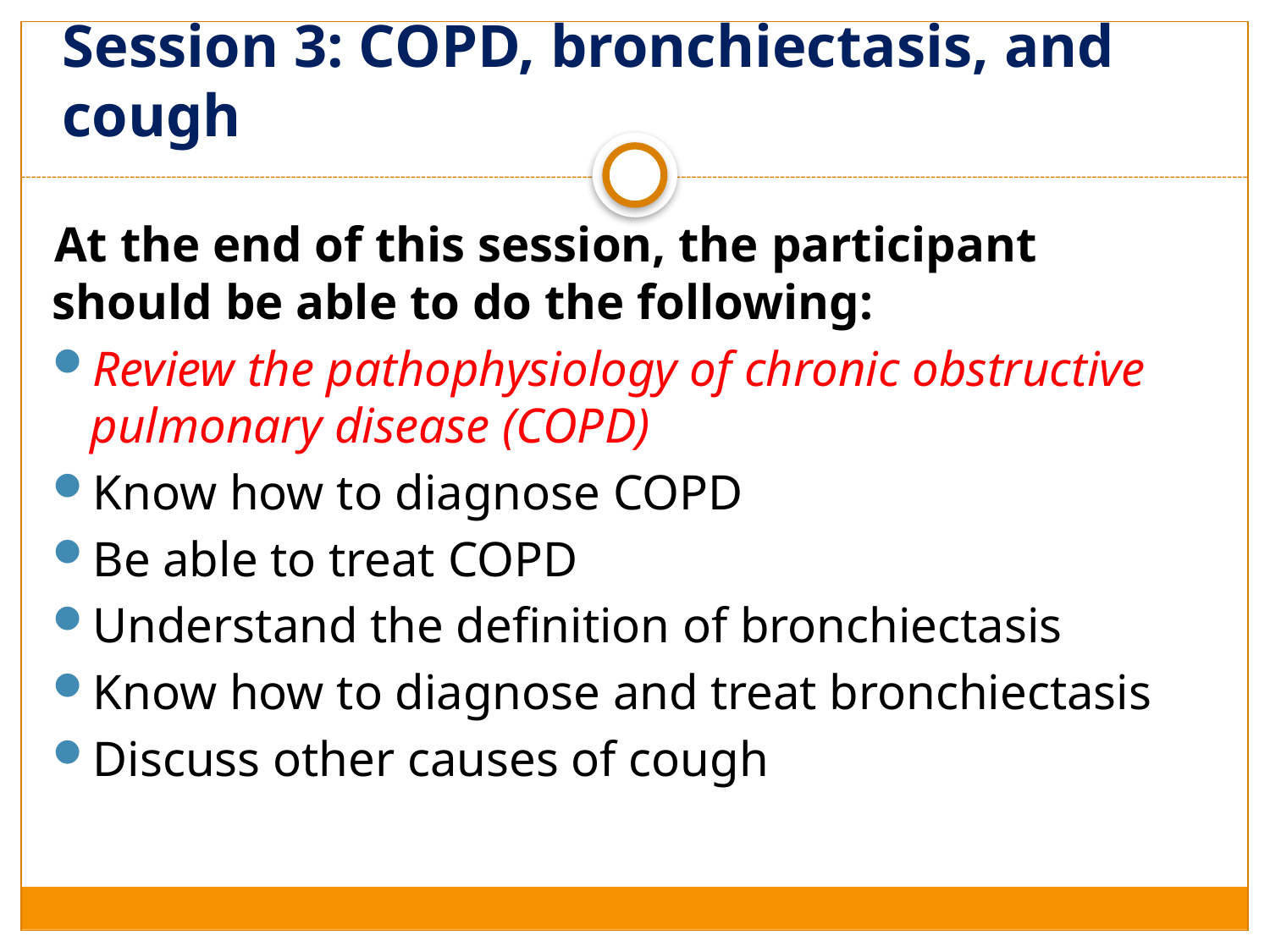

# Session 3: COPD, bronchiectasis, and cough
At the end of this session, the participant should be able to do the following:
Review the pathophysiology of chronic obstructive pulmonary disease (COPD)
Know how to diagnose COPD
Be able to treat COPD
Understand the definition of bronchiectasis
Know how to diagnose and treat bronchiectasis
Discuss other causes of cough

## Slide 112
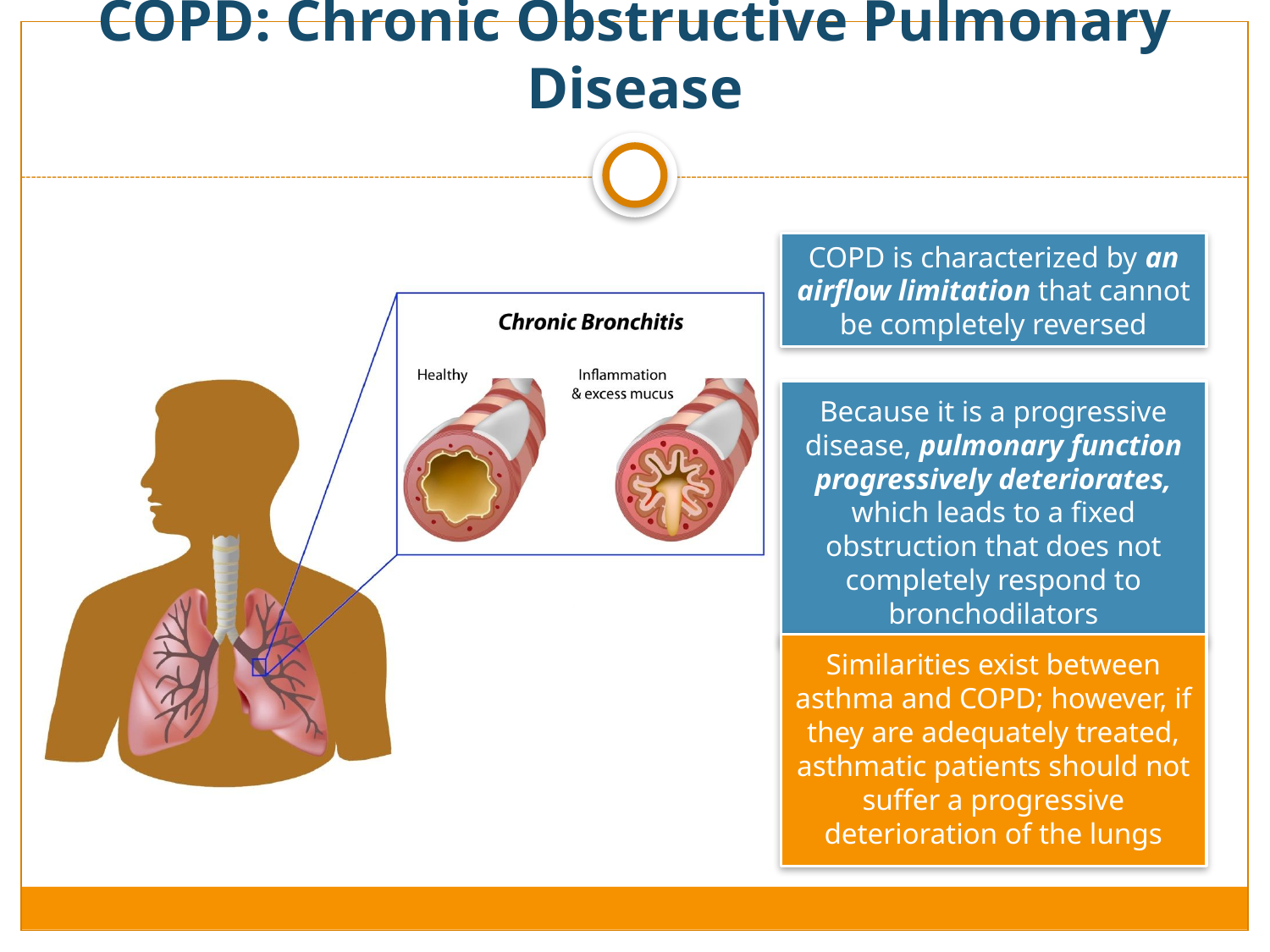

# COPD: Chronic Obstructive Pulmonary Disease
COPD is characterized by an airflow limitation that cannot be completely reversed
Because it is a progressive disease, pulmonary function progressively deteriorates, which leads to a fixed obstruction that does not completely respond to bronchodilators
Similarities exist between asthma and COPD; however, if they are adequately treated, asthmatic patients should not suffer a progressive deterioration of the lungs

## Slide 113
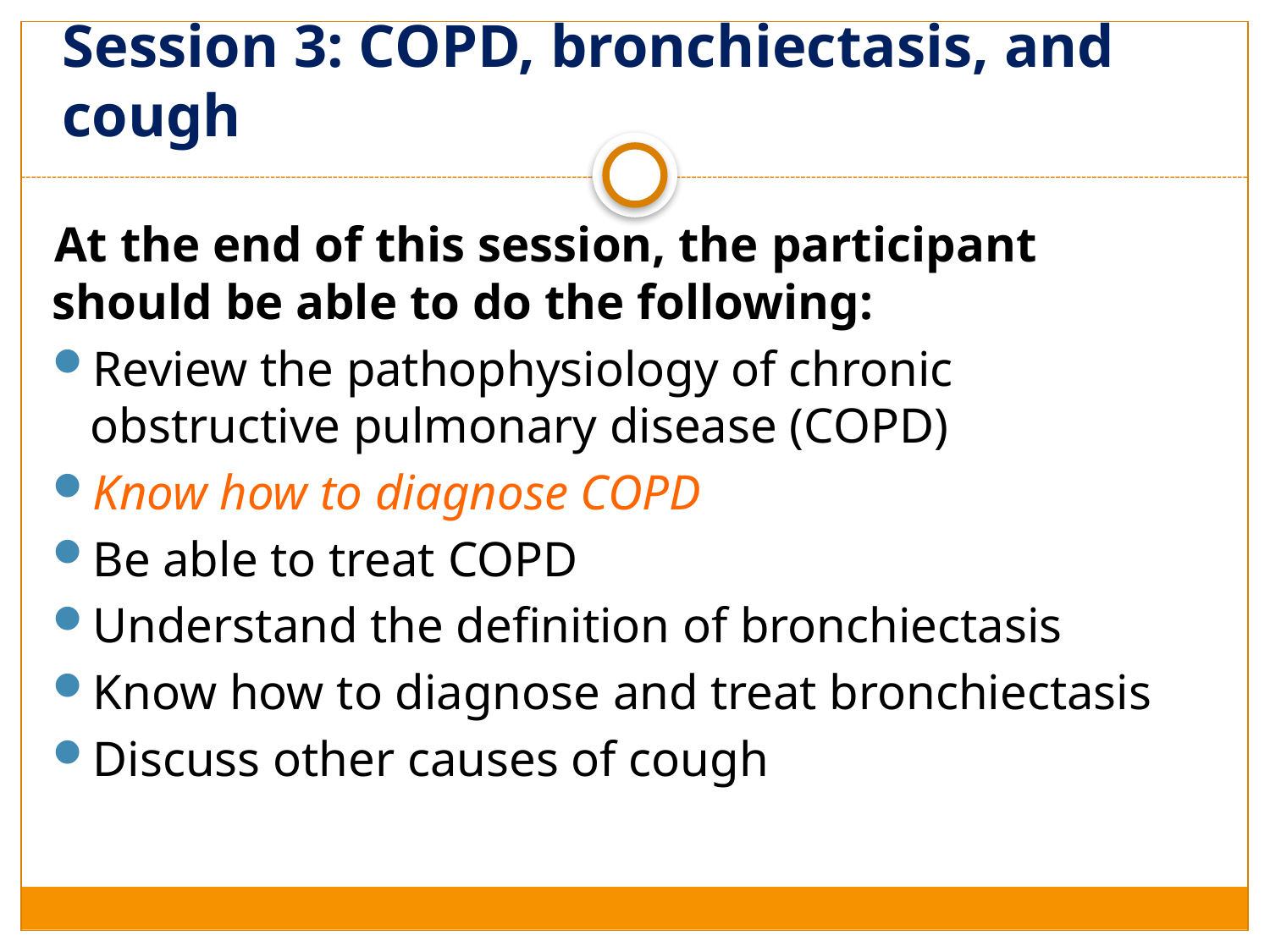

# Session 3: COPD, bronchiectasis, and cough
At the end of this session, the participant should be able to do the following:
Review the pathophysiology of chronic obstructive pulmonary disease (COPD)
Know how to diagnose COPD
Be able to treat COPD
Understand the definition of bronchiectasis
Know how to diagnose and treat bronchiectasis
Discuss other causes of cough

## Slide 114
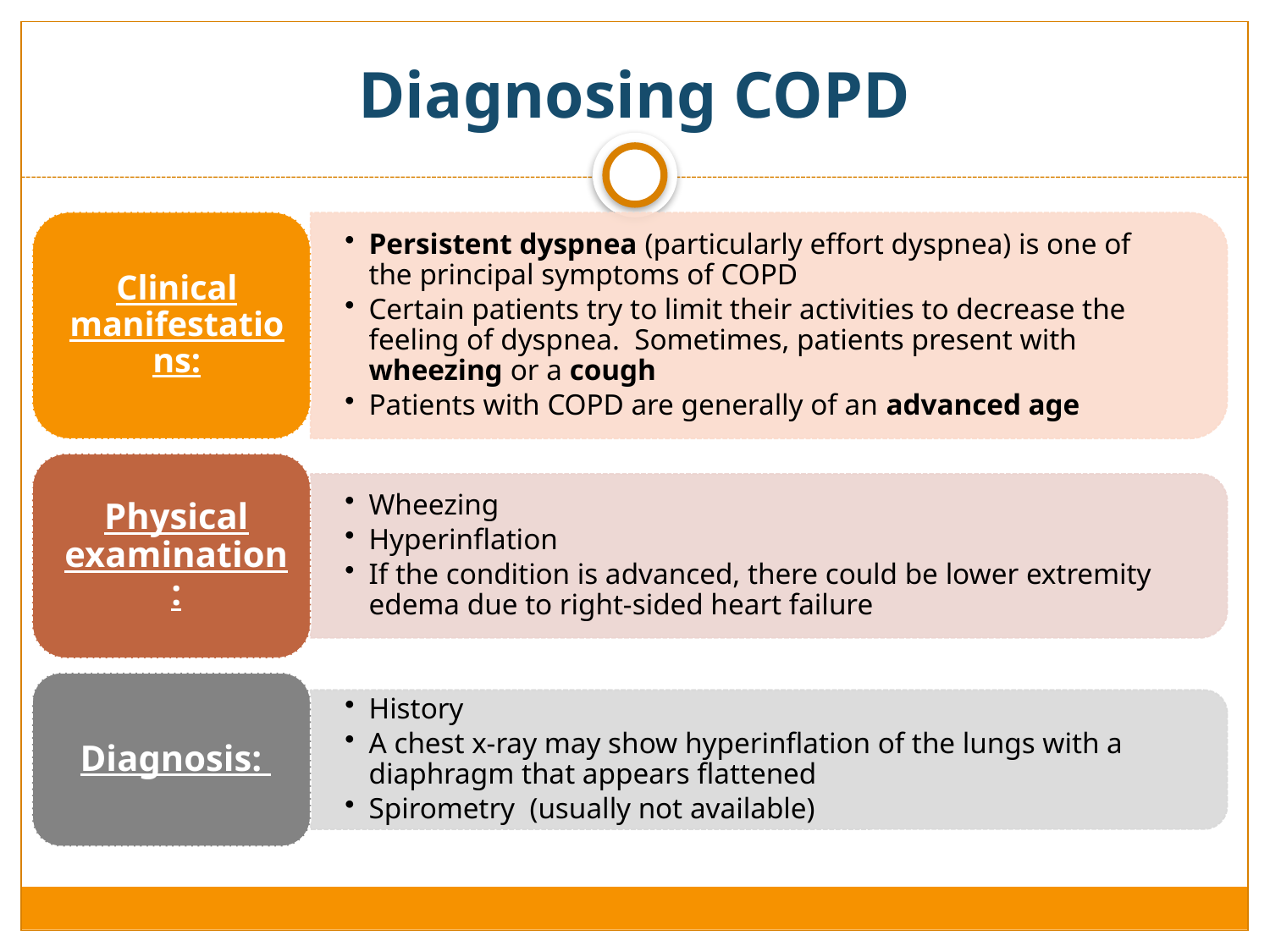

# Diagnosing COPD

## Slide 115
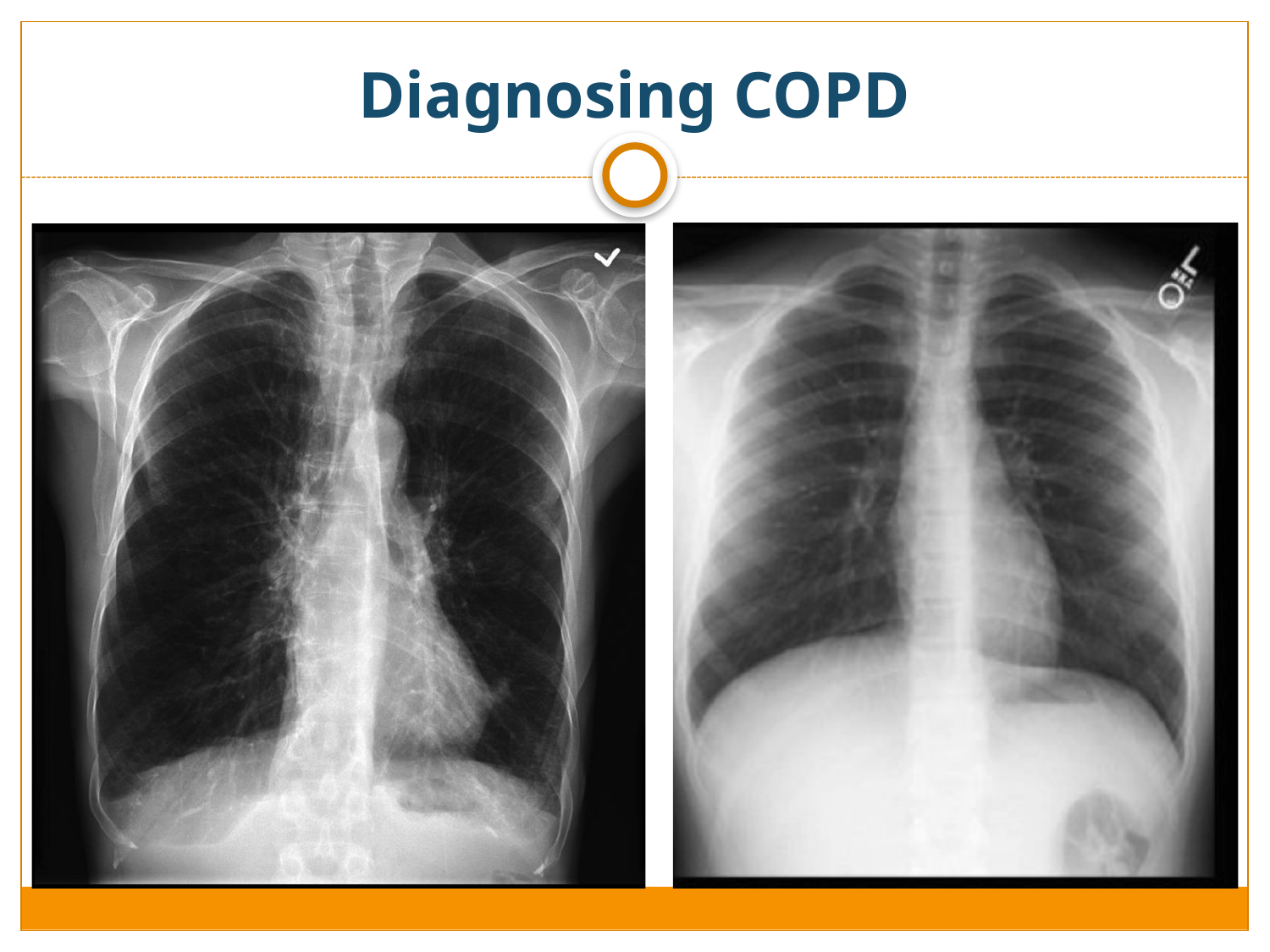

# Diagnosing COPD

## Slide 116
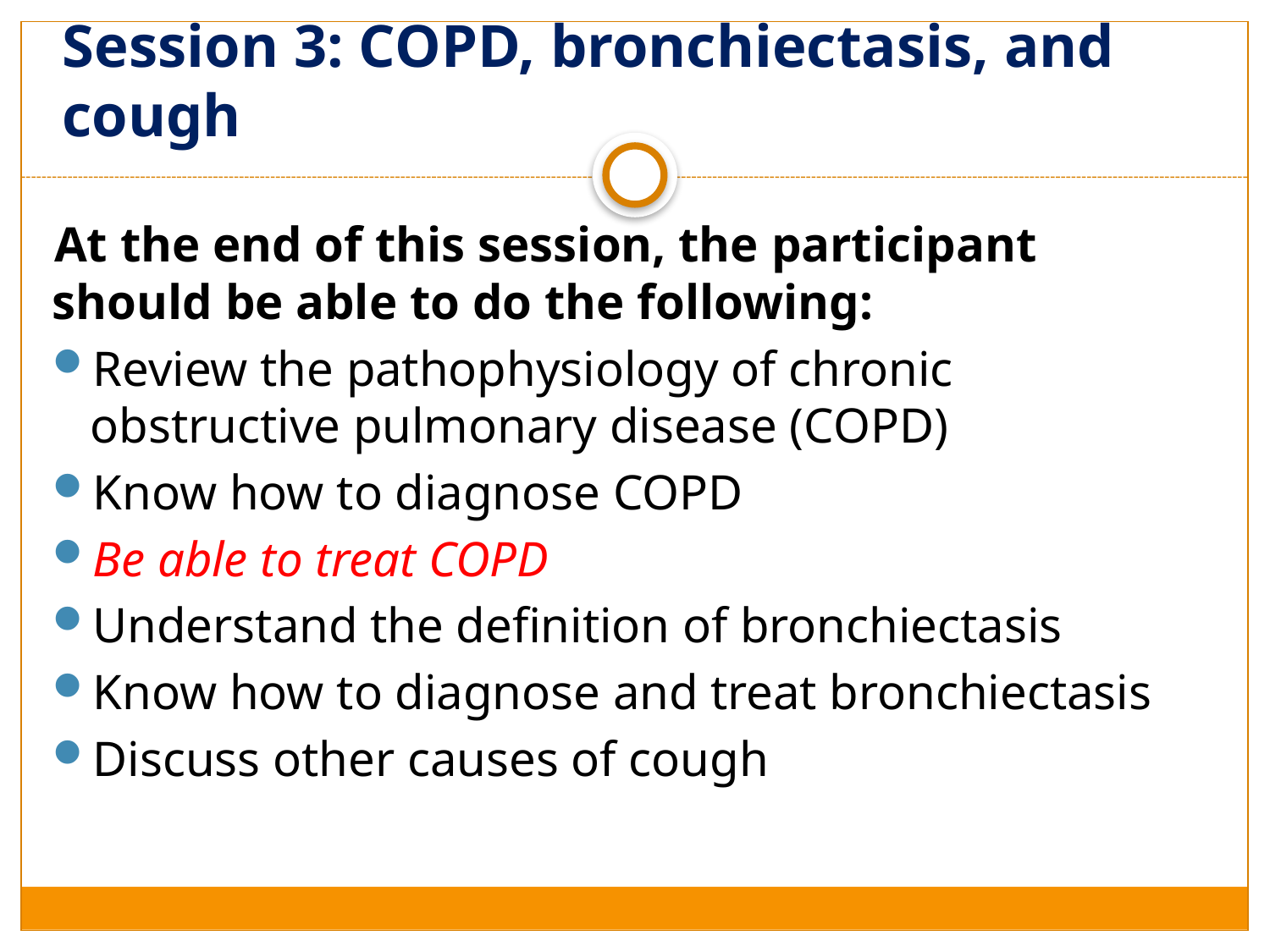

# Session 3: COPD, bronchiectasis, and cough
At the end of this session, the participant should be able to do the following:
Review the pathophysiology of chronic obstructive pulmonary disease (COPD)
Know how to diagnose COPD
Be able to treat COPD
Understand the definition of bronchiectasis
Know how to diagnose and treat bronchiectasis
Discuss other causes of cough

## Slide 117
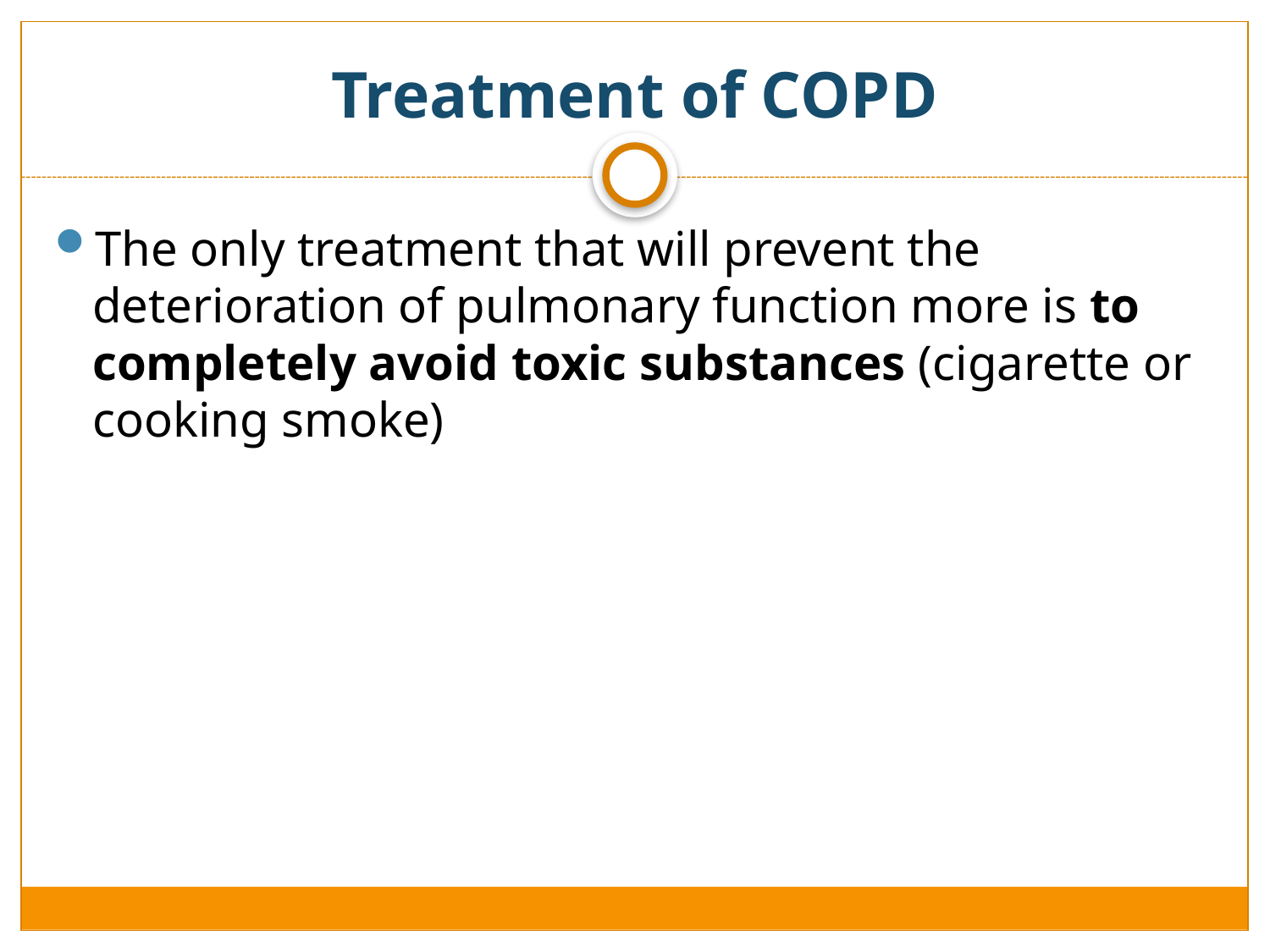

# Treatment of COPD
The only treatment that will prevent the deterioration of pulmonary function more is to completely avoid toxic substances (cigarette or cooking smoke)

## Slide 118
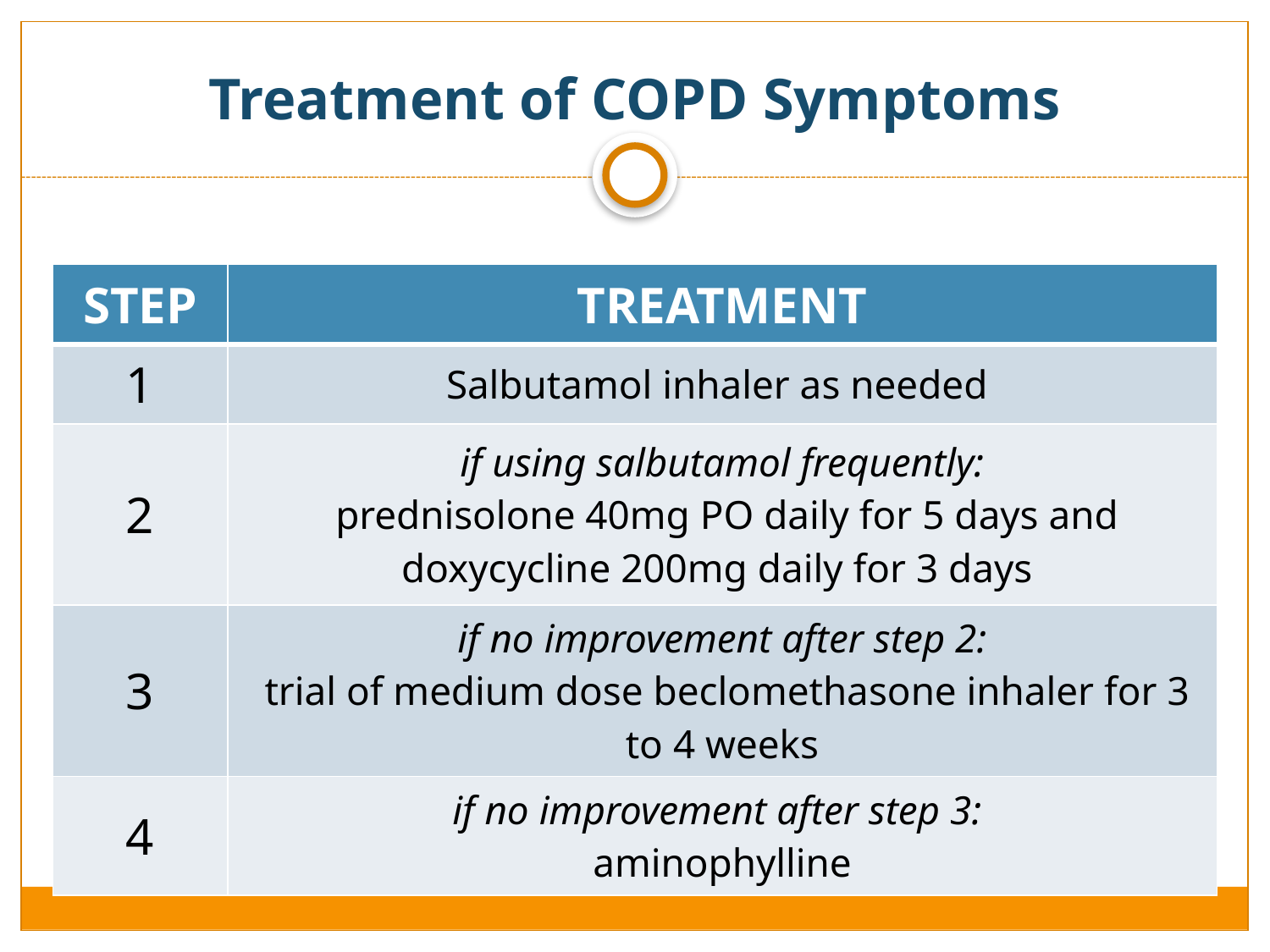

# Treatment of COPD Symptoms
| STEP | TREATMENT |
| --- | --- |
| 1 | Salbutamol inhaler as needed |
| 2 | if using salbutamol frequently: prednisolone 40mg PO daily for 5 days and doxycycline 200mg daily for 3 days |
| 3 | if no improvement after step 2: trial of medium dose beclomethasone inhaler for 3 to 4 weeks |
| 4 | if no improvement after step 3: aminophylline |

## Slide 119
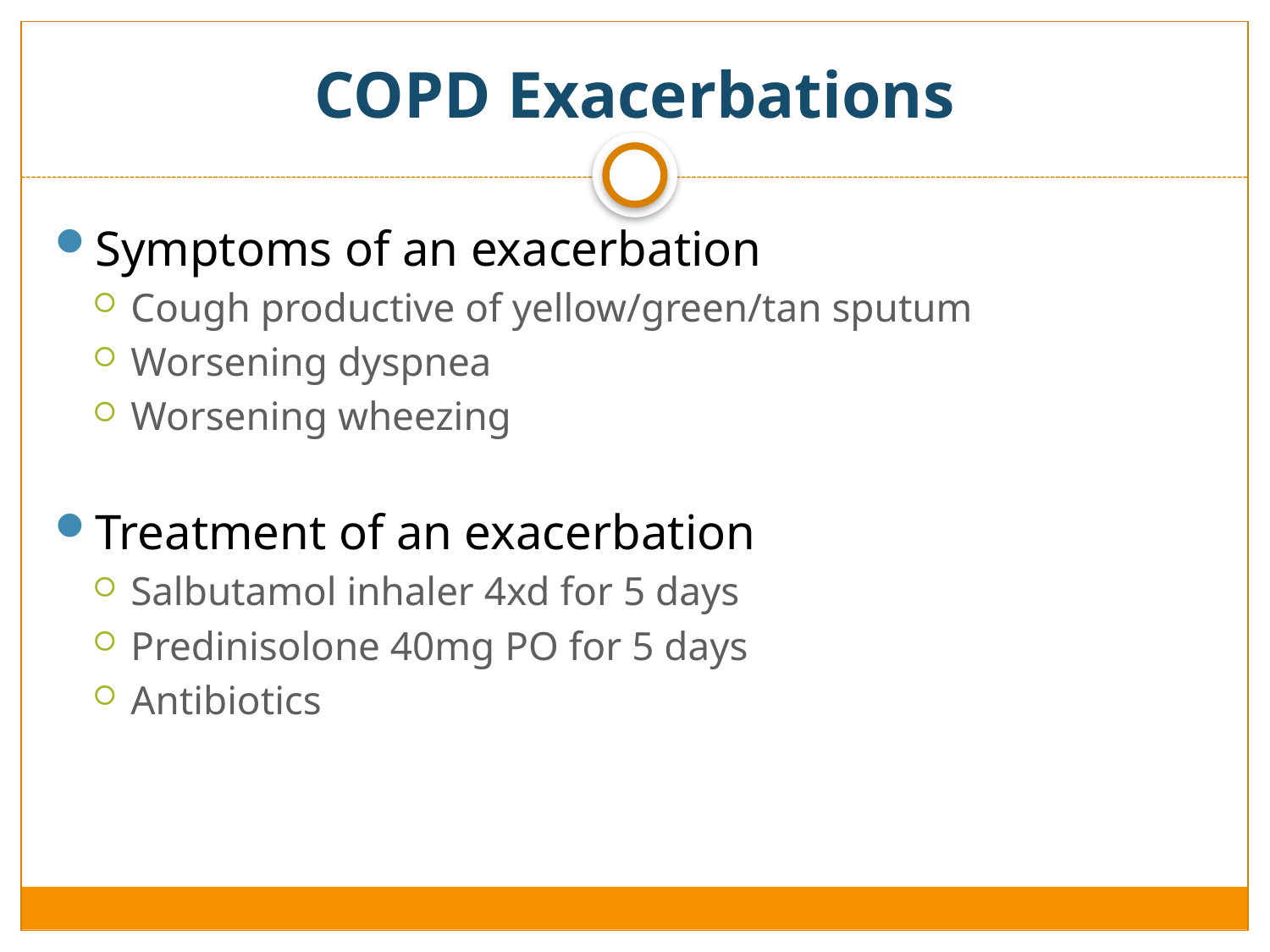

# COPD Exacerbations
Symptoms of an exacerbation
Cough productive of yellow/green/tan sputum
Worsening dyspnea
Worsening wheezing
Treatment of an exacerbation
Salbutamol inhaler 4xd for 5 days
Predinisolone 40mg PO for 5 days
Antibiotics

## Slide 120
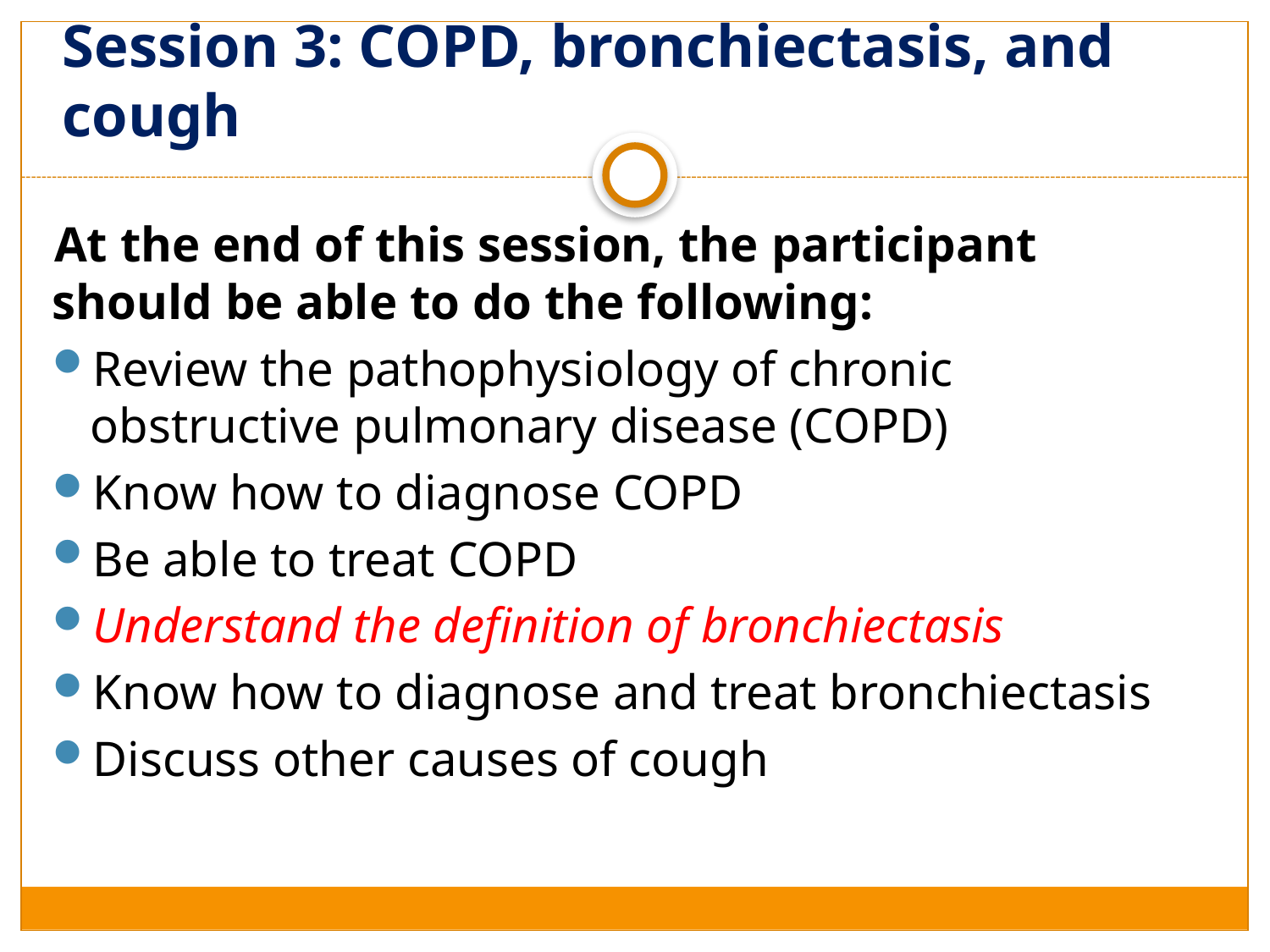

# Session 3: COPD, bronchiectasis, and cough
At the end of this session, the participant should be able to do the following:
Review the pathophysiology of chronic obstructive pulmonary disease (COPD)
Know how to diagnose COPD
Be able to treat COPD
Understand the definition of bronchiectasis
Know how to diagnose and treat bronchiectasis
Discuss other causes of cough

## Slide 121
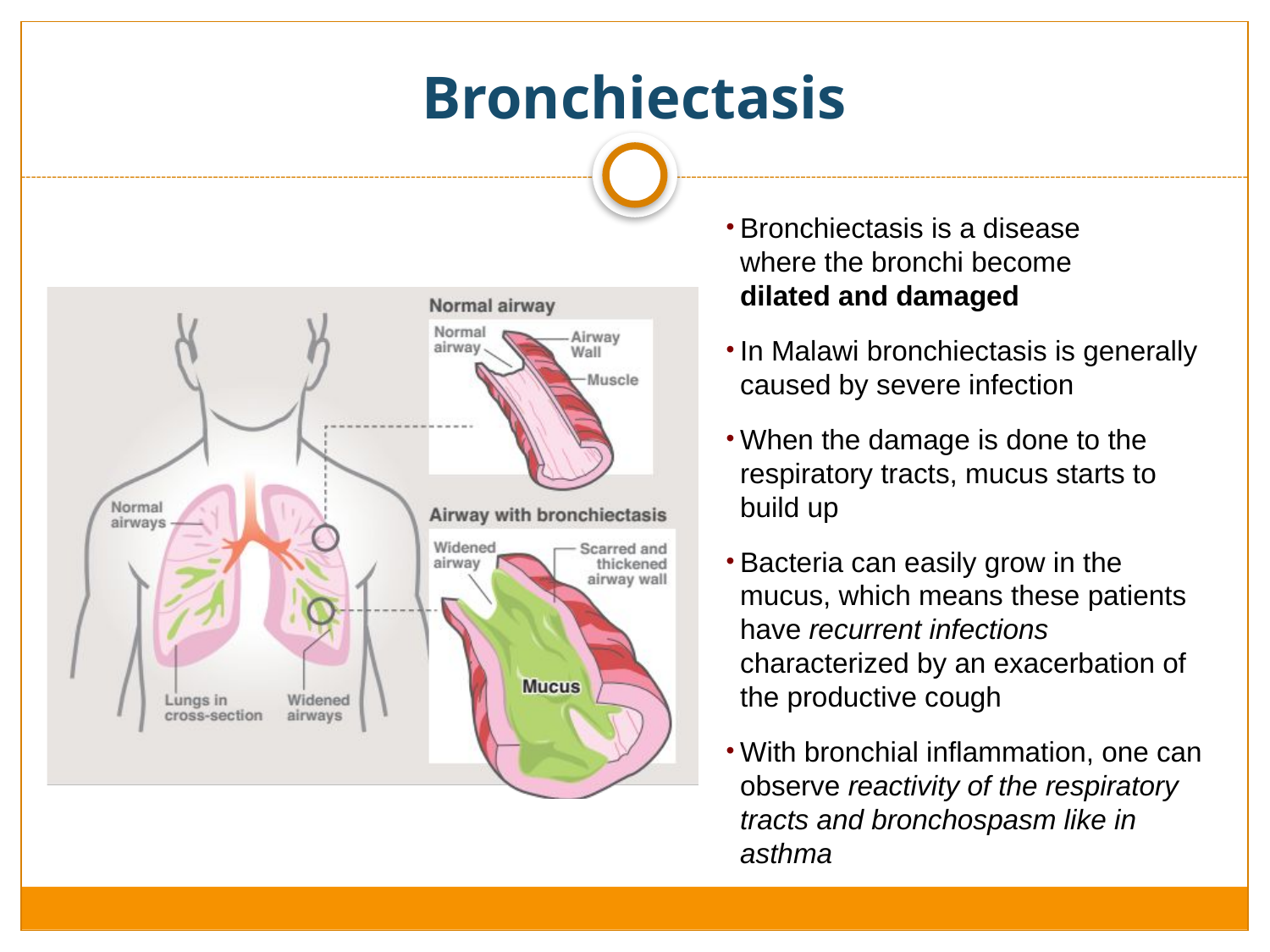

# Bronchiectasis
Bronchiectasis is a disease
where the bronchi become
dilated and damaged
In Malawi bronchiectasis is generally caused by severe infection
When the damage is done to the respiratory tracts, mucus starts to build up
Bacteria can easily grow in the mucus, which means these patients have recurrent infections characterized by an exacerbation of the productive cough
With bronchial inflammation, one can observe reactivity of the respiratory tracts and bronchospasm like in asthma

## Slide 122
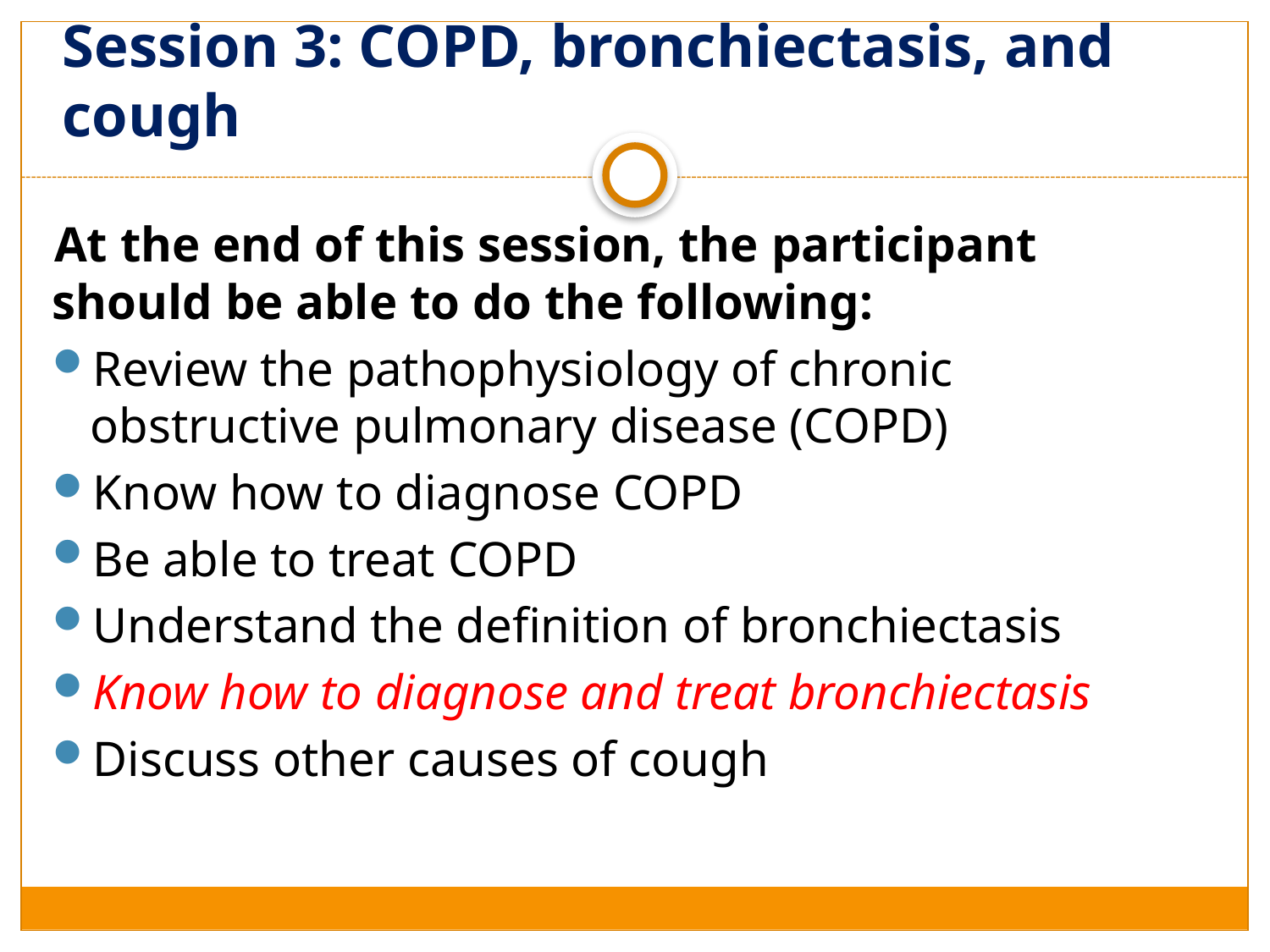

# Session 3: COPD, bronchiectasis, and cough
At the end of this session, the participant should be able to do the following:
Review the pathophysiology of chronic obstructive pulmonary disease (COPD)
Know how to diagnose COPD
Be able to treat COPD
Understand the definition of bronchiectasis
Know how to diagnose and treat bronchiectasis
Discuss other causes of cough

## Slide 123
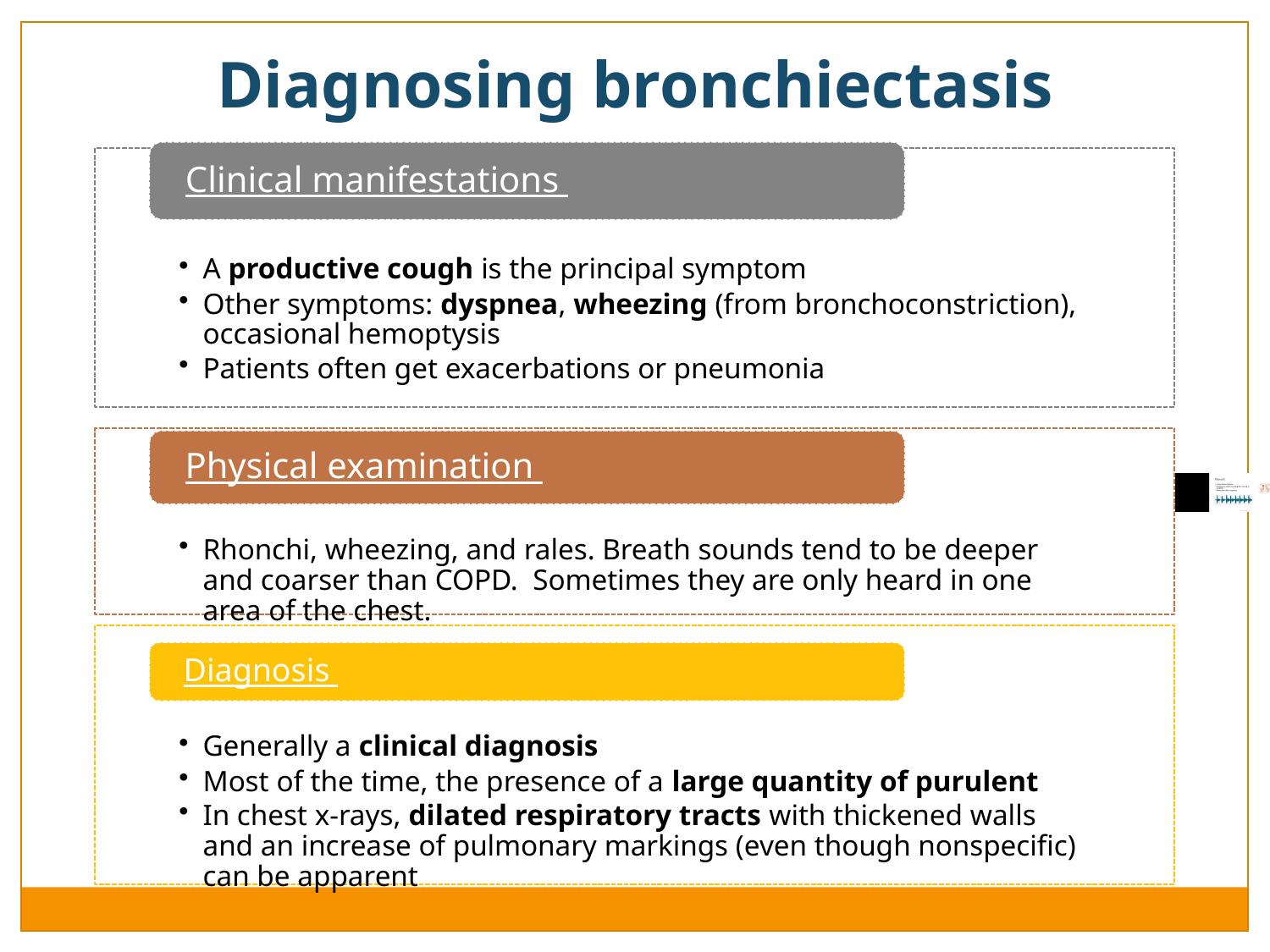

Diagnosing bronchiectasis

## Slide 124
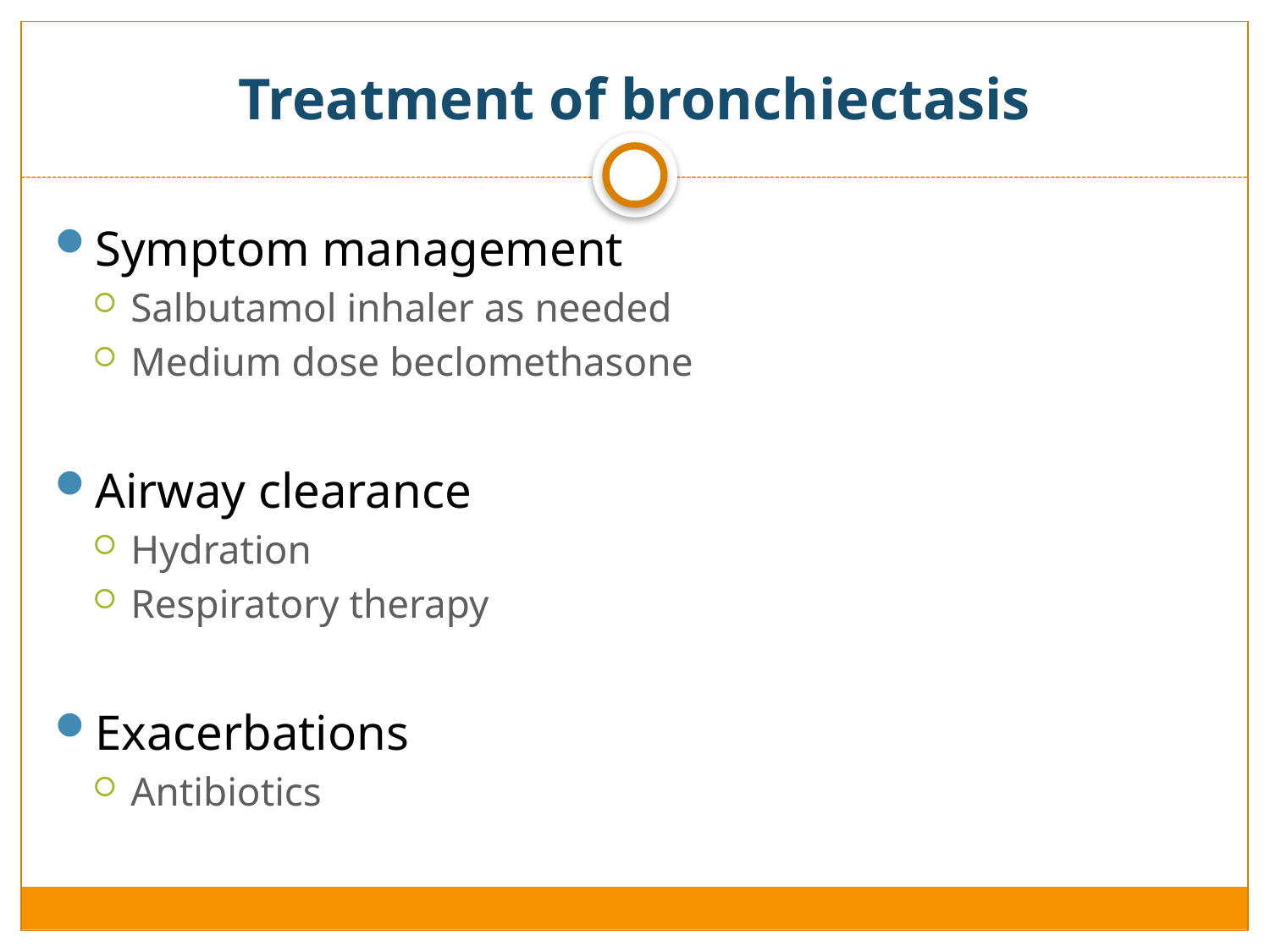

# Treatment of bronchiectasis
Symptom management
Salbutamol inhaler as needed
Medium dose beclomethasone
Airway clearance
Hydration
Respiratory therapy
Exacerbations
Antibiotics

## Slide 125
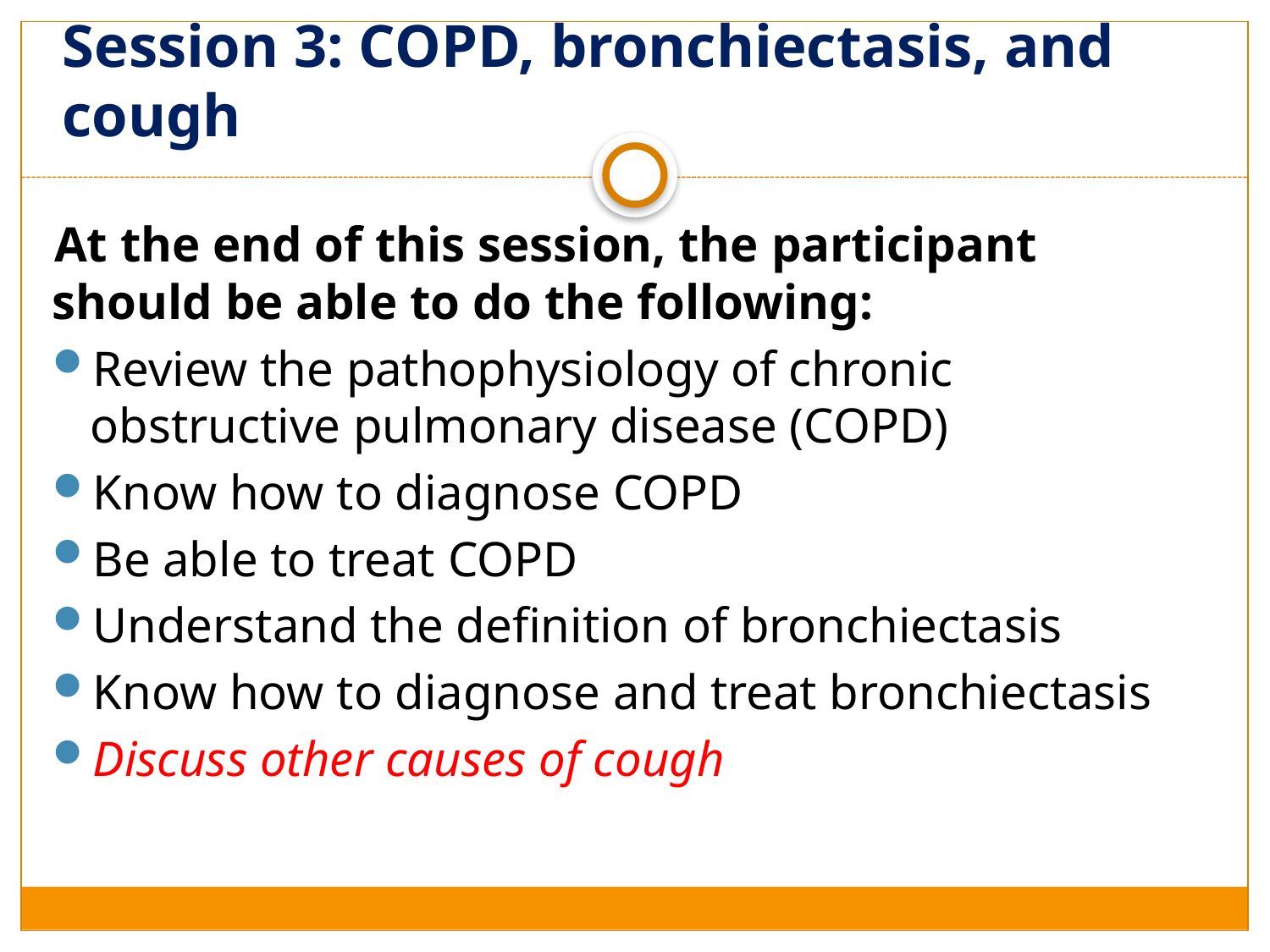

# Session 3: COPD, bronchiectasis, and cough
At the end of this session, the participant should be able to do the following:
Review the pathophysiology of chronic obstructive pulmonary disease (COPD)
Know how to diagnose COPD
Be able to treat COPD
Understand the definition of bronchiectasis
Know how to diagnose and treat bronchiectasis
Discuss other causes of cough

## Slide 126
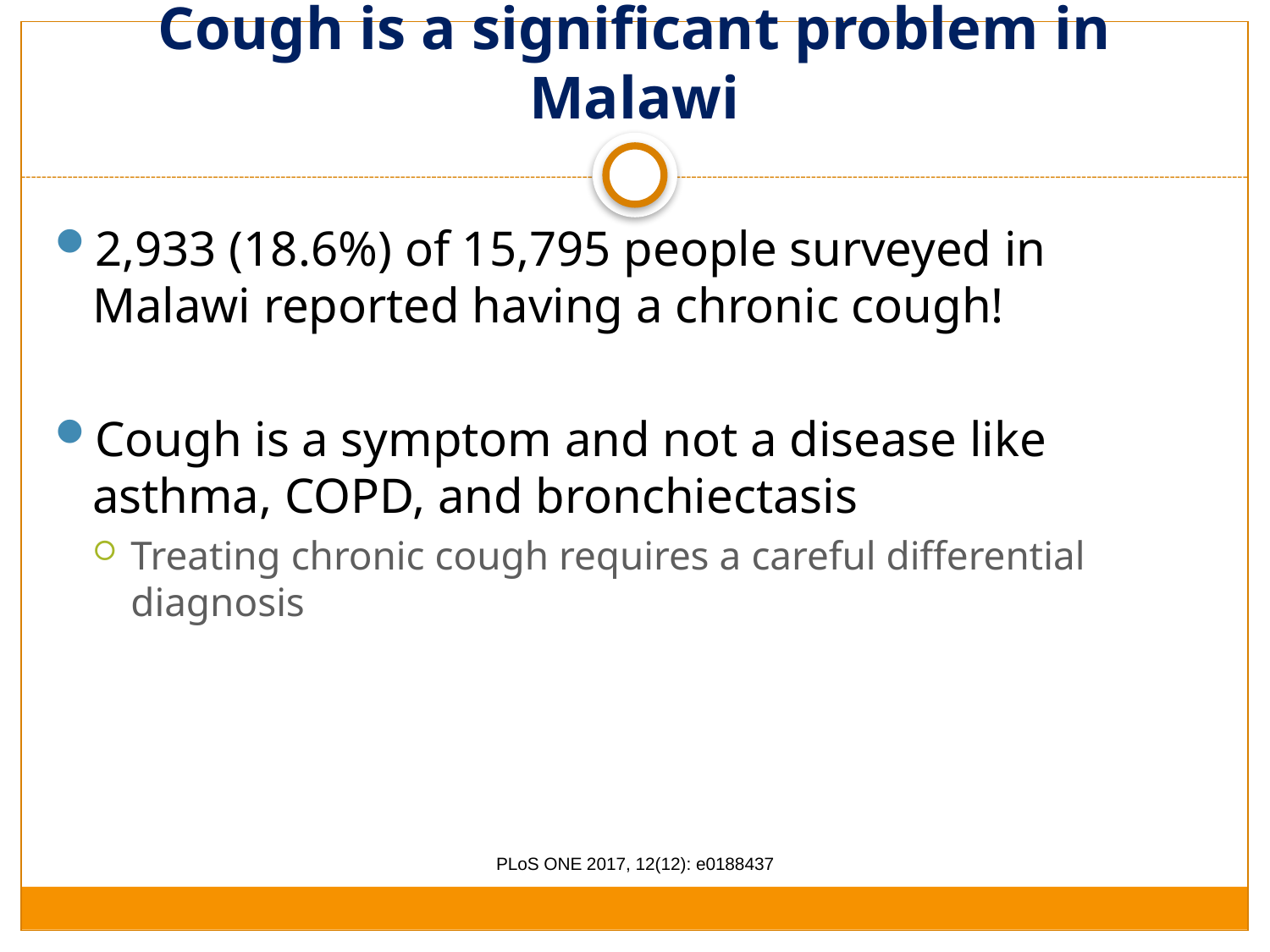

# Cough is a significant problem in Malawi
2,933 (18.6%) of 15,795 people surveyed in Malawi reported having a chronic cough!
Cough is a symptom and not a disease like asthma, COPD, and bronchiectasis
Treating chronic cough requires a careful differential diagnosis
PLoS ONE 2017, 12(12): e0188437

## Slide 127
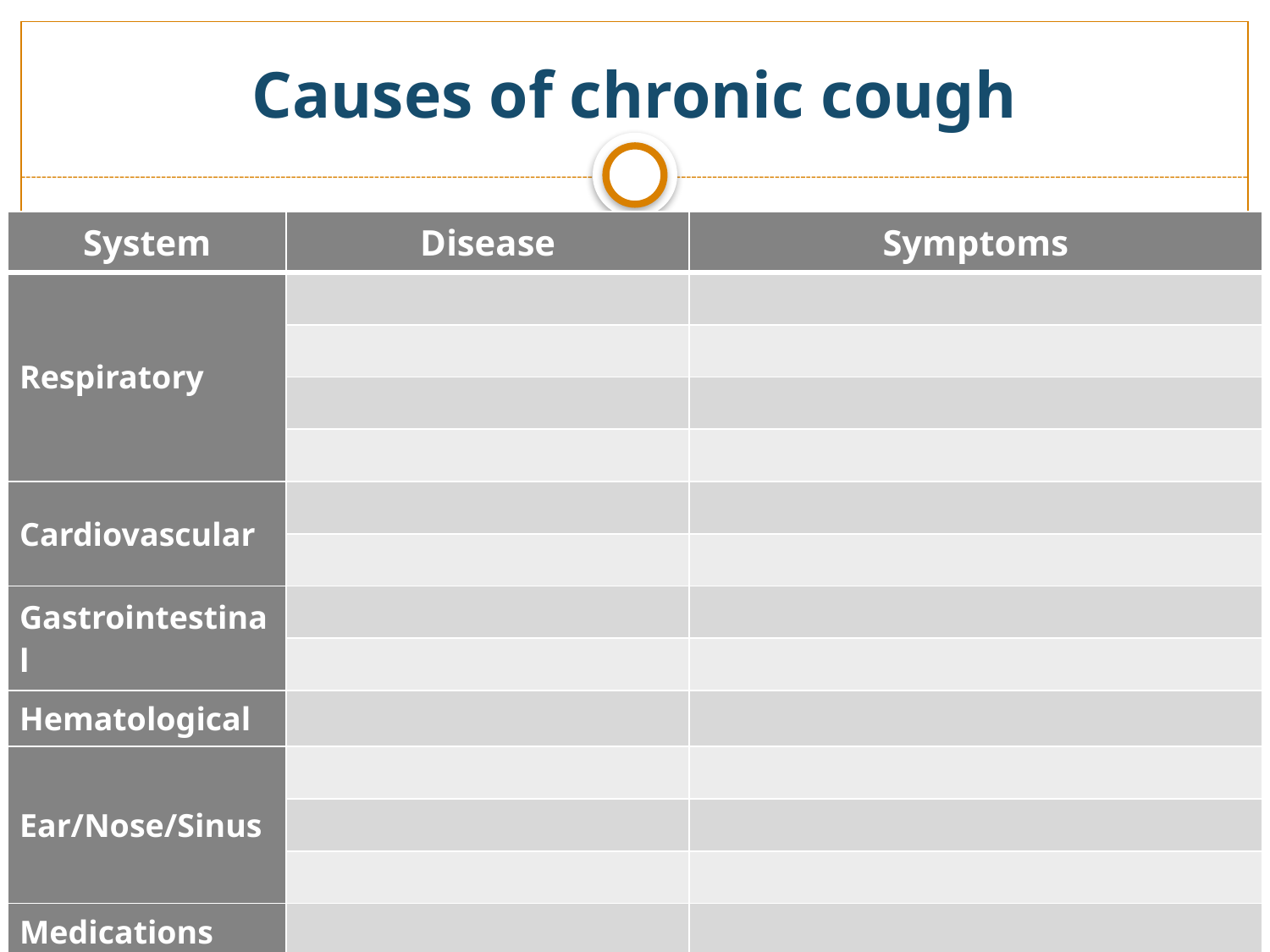

# Causes of chronic cough
| System | Disease | Symptoms |
| --- | --- | --- |
| Respiratory | | |
| | | |
| | | |
| | | |
| Cardiovascular | | |
| | | |
| Gastrointestinal | | |
| | | |
| Hematological | | |
| Ear/Nose/Sinus | | |
| | | |
| | | |
| Medications | | |

## Slide 128
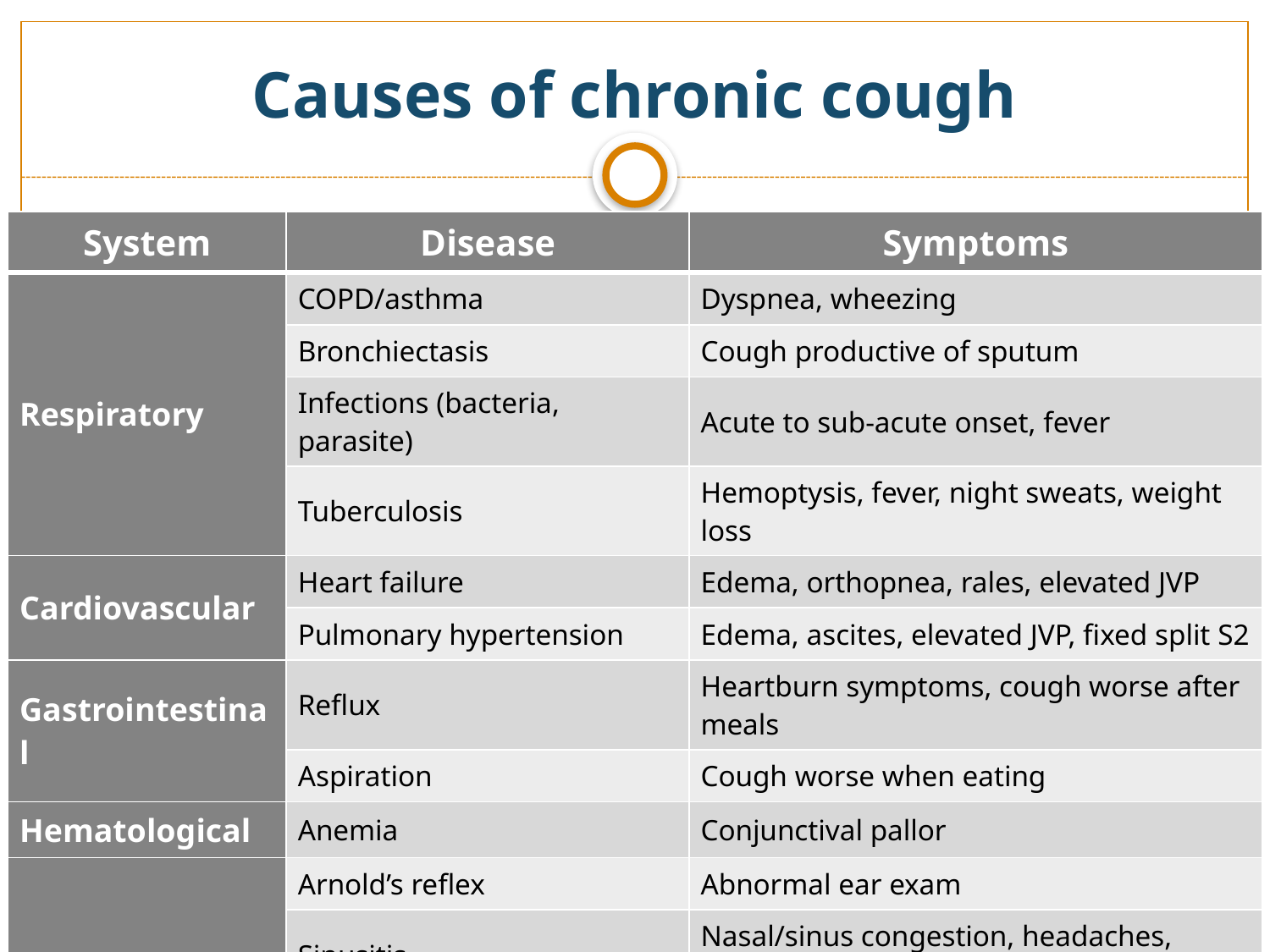

# Causes of chronic cough
| System | Disease | Symptoms |
| --- | --- | --- |
| Respiratory | COPD/asthma | Dyspnea, wheezing |
| | Bronchiectasis | Cough productive of sputum |
| | Infections (bacteria, parasite) | Acute to sub-acute onset, fever |
| | Tuberculosis | Hemoptysis, fever, night sweats, weight loss |
| Cardiovascular | Heart failure | Edema, orthopnea, rales, elevated JVP |
| | Pulmonary hypertension | Edema, ascites, elevated JVP, fixed split S2 |
| Gastrointestinal | Reflux | Heartburn symptoms, cough worse after meals |
| | Aspiration | Cough worse when eating |
| Hematological | Anemia | Conjunctival pallor |
| Ear/Nose/Sinus | Arnold’s reflex | Abnormal ear exam |
| | Sinusitis | Nasal/sinus congestion, headaches, rhinorrhea |
| | Rhinitis | Rhinorrhea, post-nasal drip, worse lying down |
| Medications | Ace inhibitors | Dry persistent cough |

## Slide 129
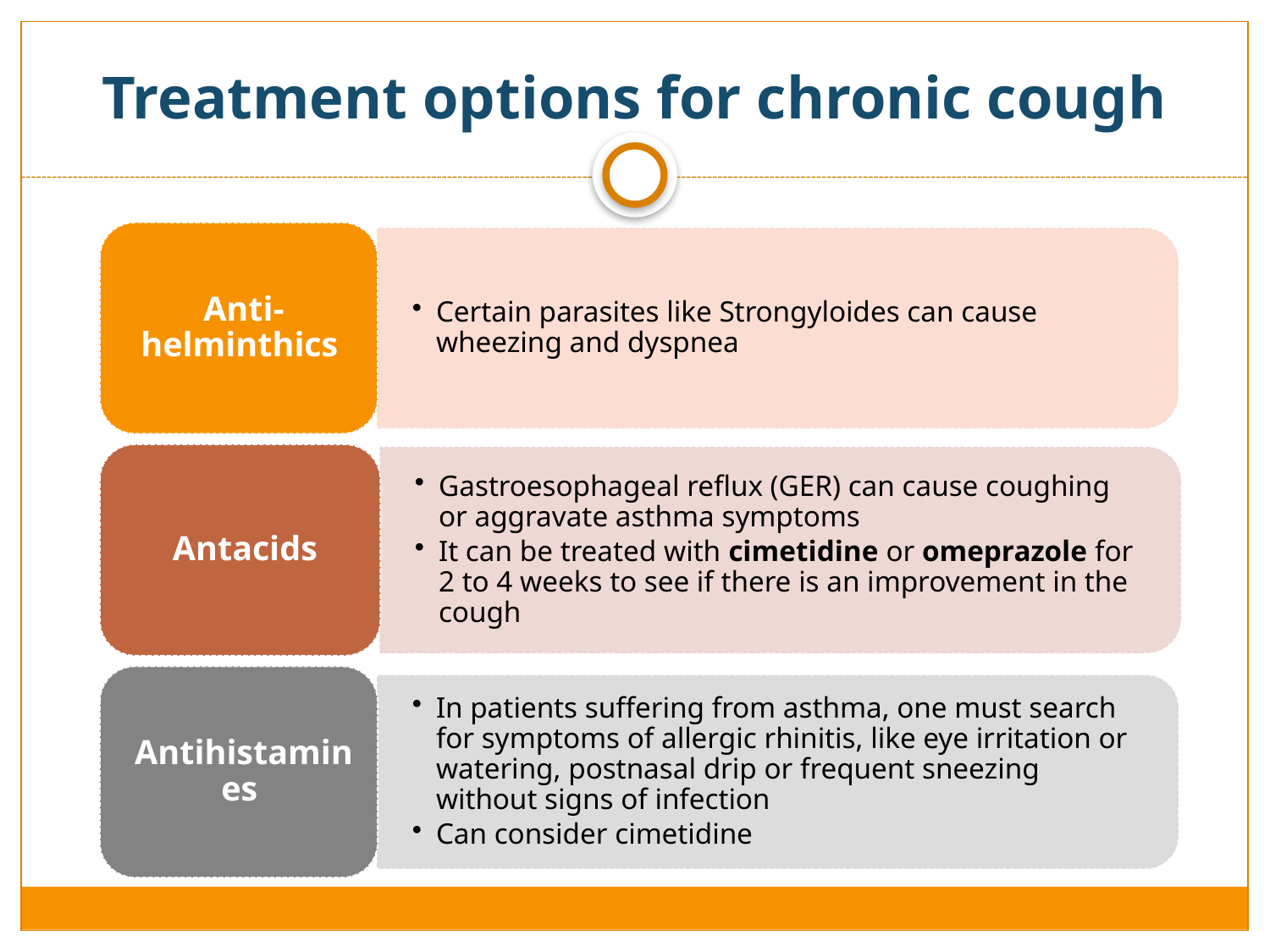

# Treatment options for chronic cough

## Slide 130
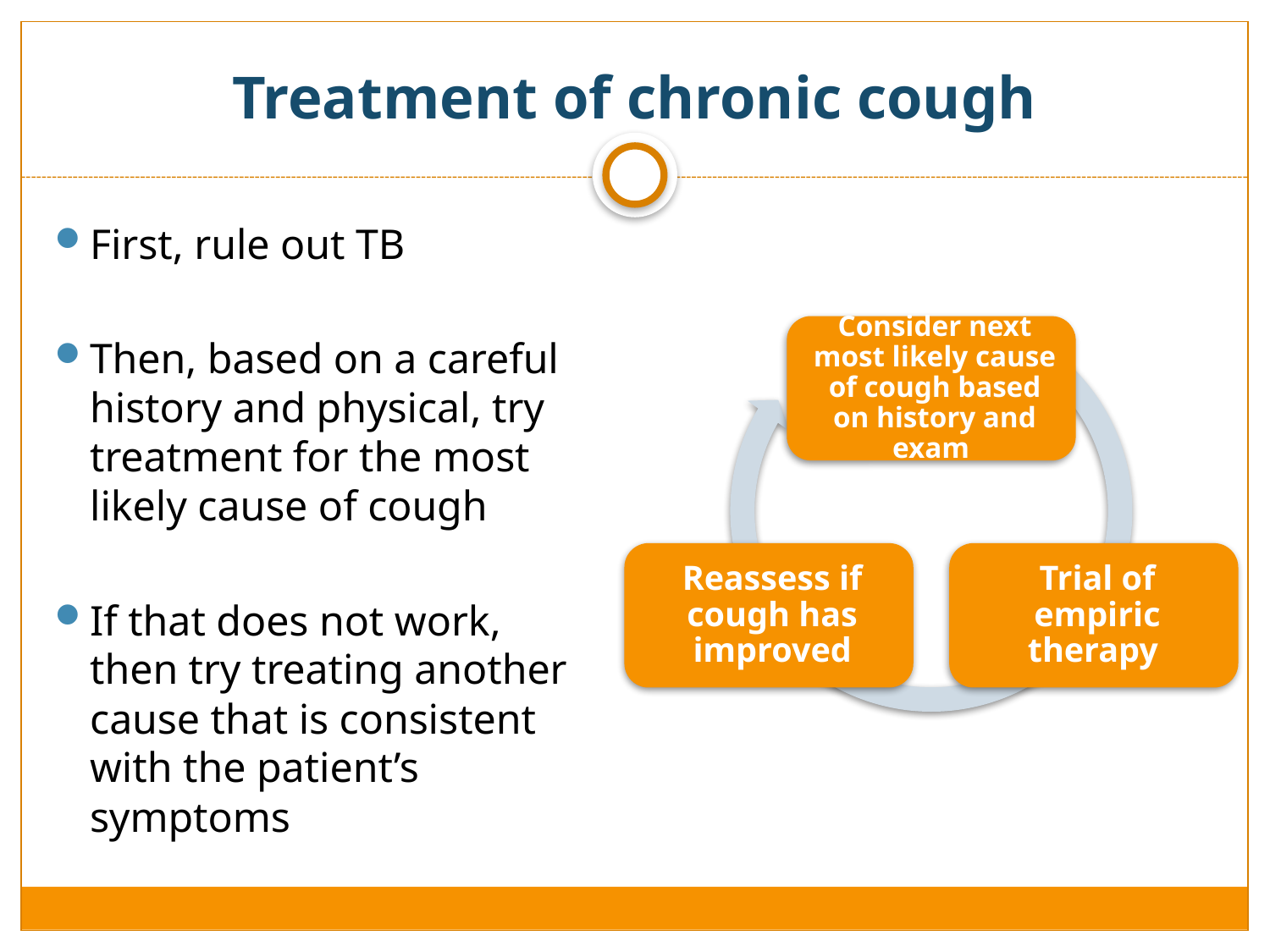

# Treatment of chronic cough
First, rule out TB
Then, based on a careful history and physical, try treatment for the most likely cause of cough
If that does not work, then try treating another cause that is consistent with the patient’s symptoms

## Slide 131
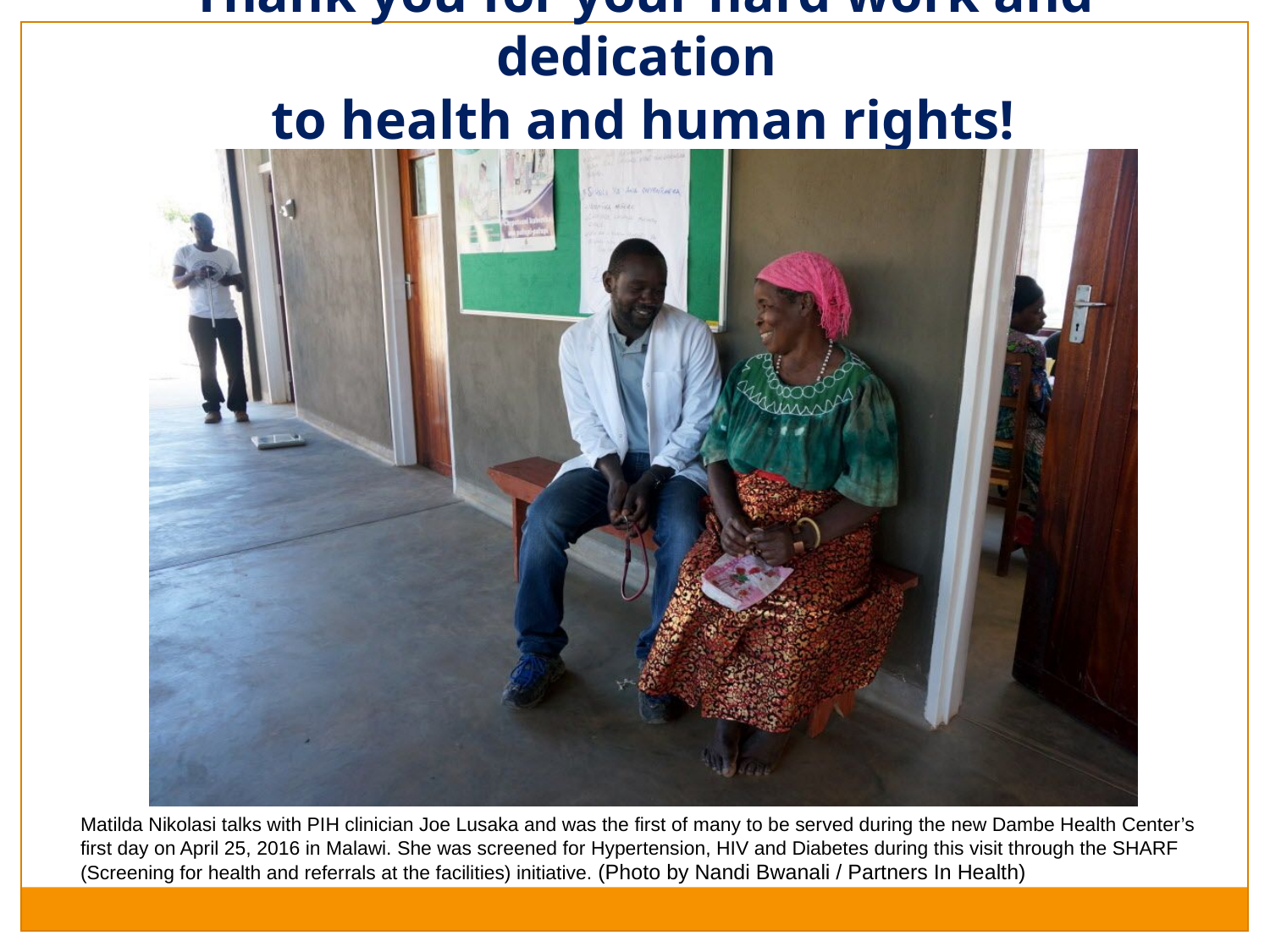

Thank you for your hard work and dedication
to health and human rights!
Matilda Nikolasi talks with PIH clinician Joe Lusaka and was the first of many to be served during the new Dambe Health Center’s first day on April 25, 2016 in Malawi. She was screened for Hypertension, HIV and Diabetes during this visit through the SHARF (Screening for health and referrals at the facilities) initiative. (Photo by Nandi Bwanali / Partners In Health)
