# Supplementary material for: Training Mid-Level Providers to Treat Severe Non-Communicable Diseases in Neno, Malawi through PEN-Plus Strategies
Source: Ann Glob Health. 2022 Aug 11;88(1):69. doi: 10.5334/aogh.3750 (PMC9389951; doi:10.5334/aogh.3750)
Supplement: Didactic Materials. — The supplementary materials contain a suggested didactic training schedule and the PowerPoint presentations used for PEN-Plus training in Neno, Malawi. These materials have been reviewed and accepted by the Malawi Ministry of Health for future PEN-Plus trainings in Malawi. [file agh-88-1-3750-s2.zip › Didactic_Materials/R_CKD.pptx]

## Slide 1
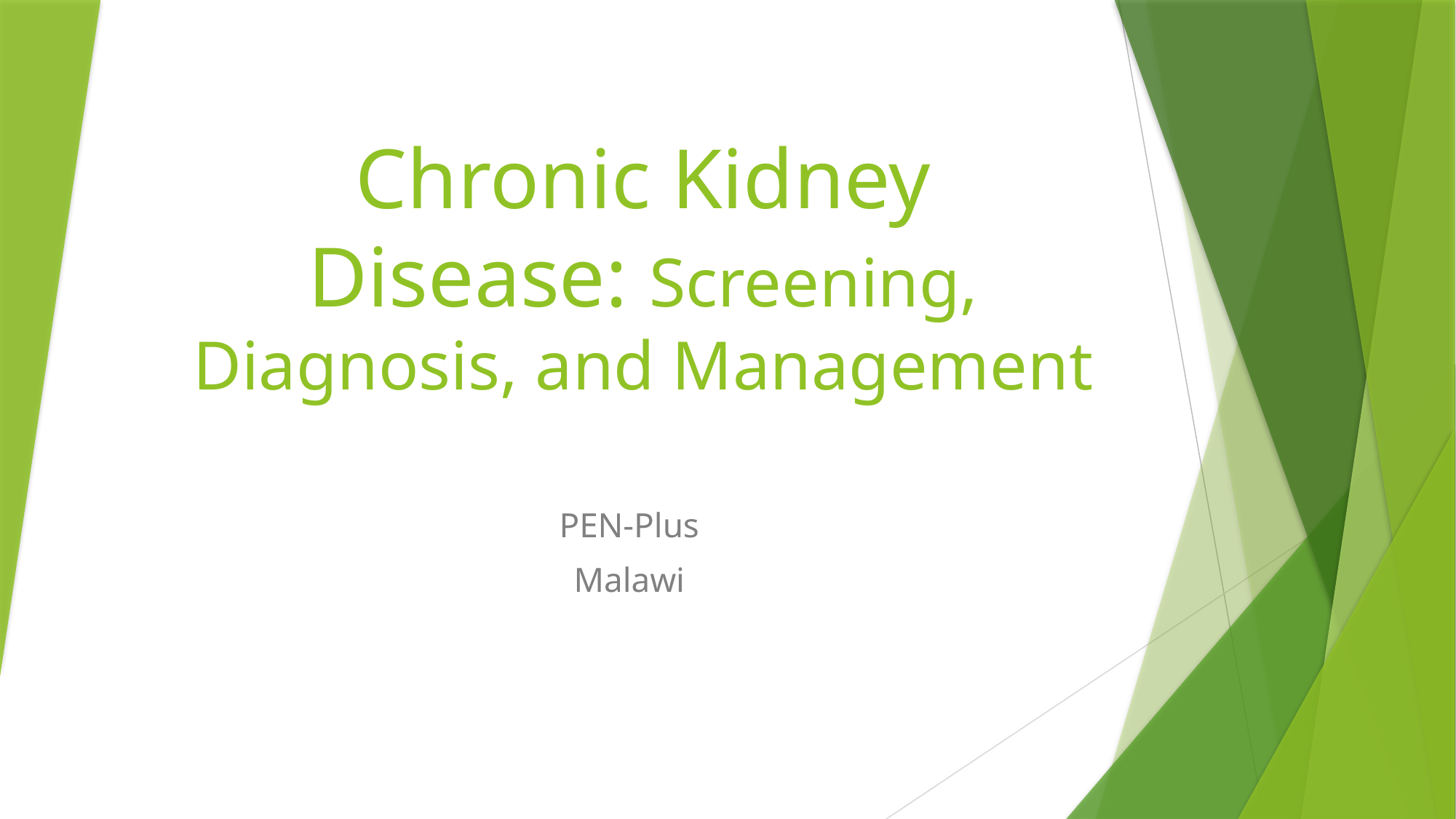

# Chronic Kidney Disease: Screening, Diagnosis, and Management
PEN-Plus
Malawi

## Slide 2
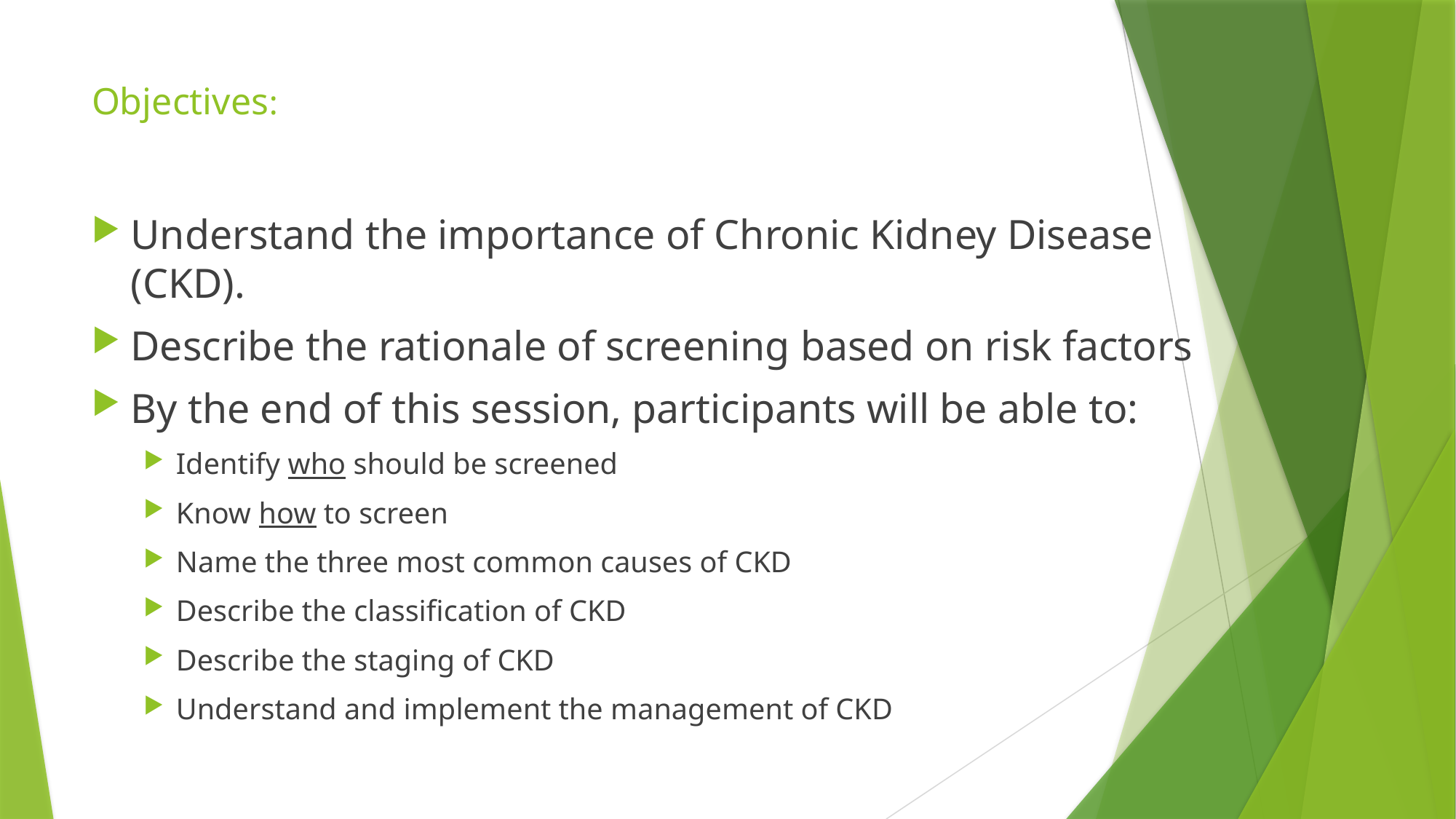

# Objectives:
Understand the importance of Chronic Kidney Disease (CKD).
Describe the rationale of screening based on risk factors
By the end of this session, participants will be able to:
Identify who should be screened
Know how to screen
Name the three most common causes of CKD
Describe the classification of CKD
Describe the staging of CKD
Understand and implement the management of CKD

## Slide 3
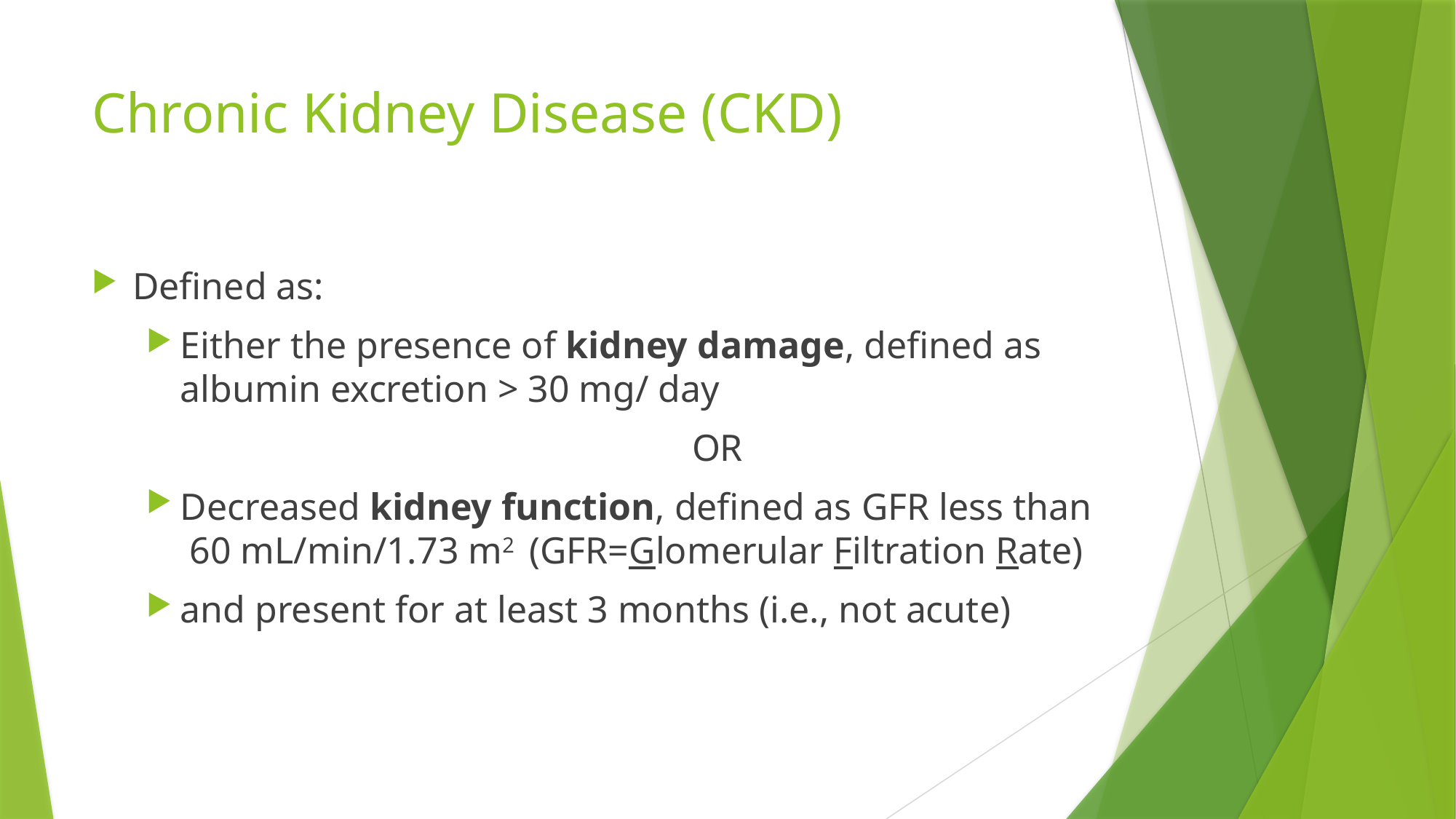

# Chronic Kidney Disease (CKD)
Defined as:
Either the presence of kidney damage, defined as albumin excretion > 30 mg/ day
					OR
Decreased kidney function, defined as GFR less than 60 mL/min/1.73 m2 (GFR=Glomerular Filtration Rate)
and present for at least 3 months (i.e., not acute)

## Slide 4
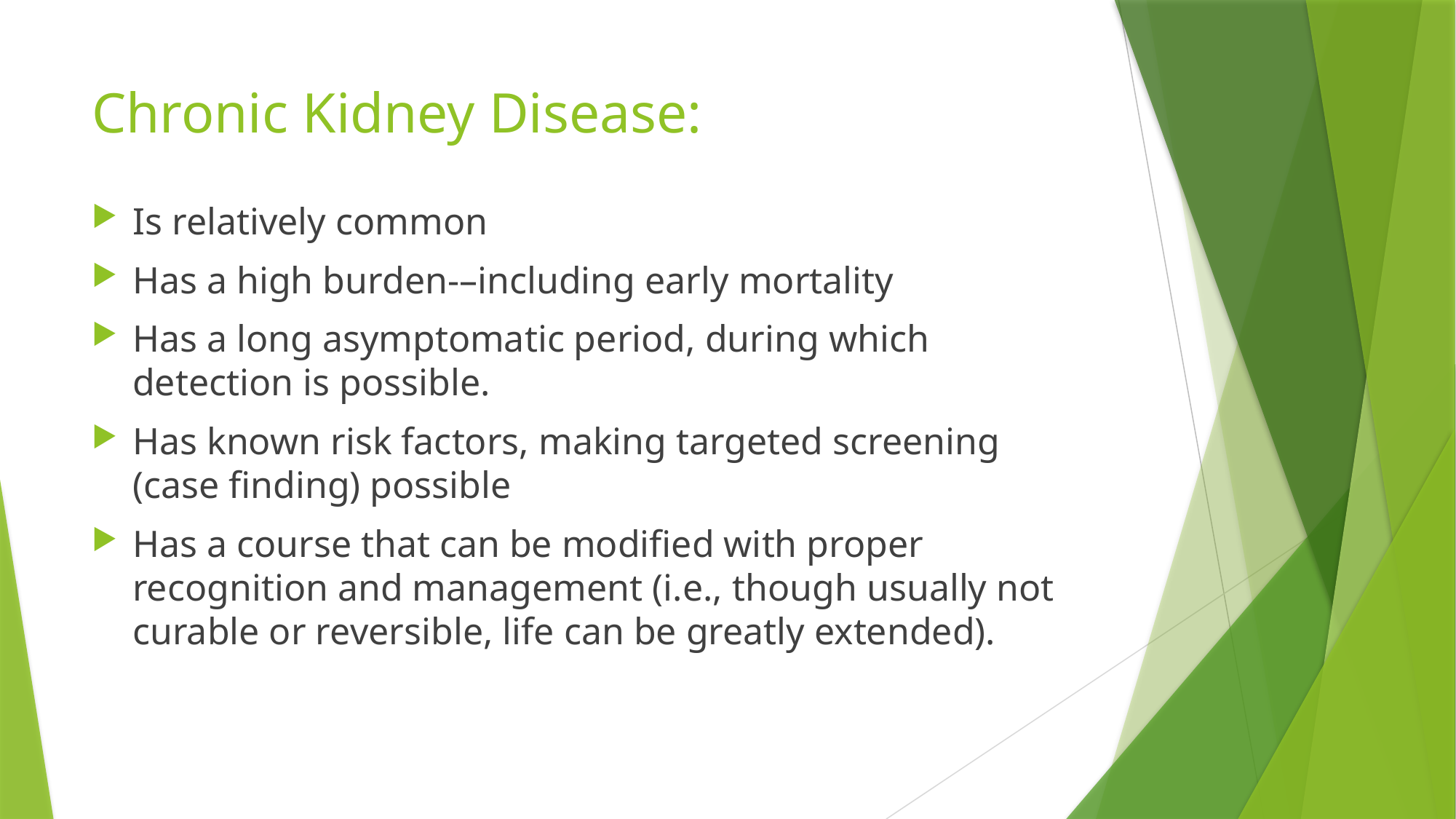

# Chronic Kidney Disease:
Is relatively common
Has a high burden-–including early mortality
Has a long asymptomatic period, during which detection is possible.
Has known risk factors, making targeted screening (case finding) possible
Has a course that can be modified with proper recognition and management (i.e., though usually not curable or reversible, life can be greatly extended).

## Slide 5
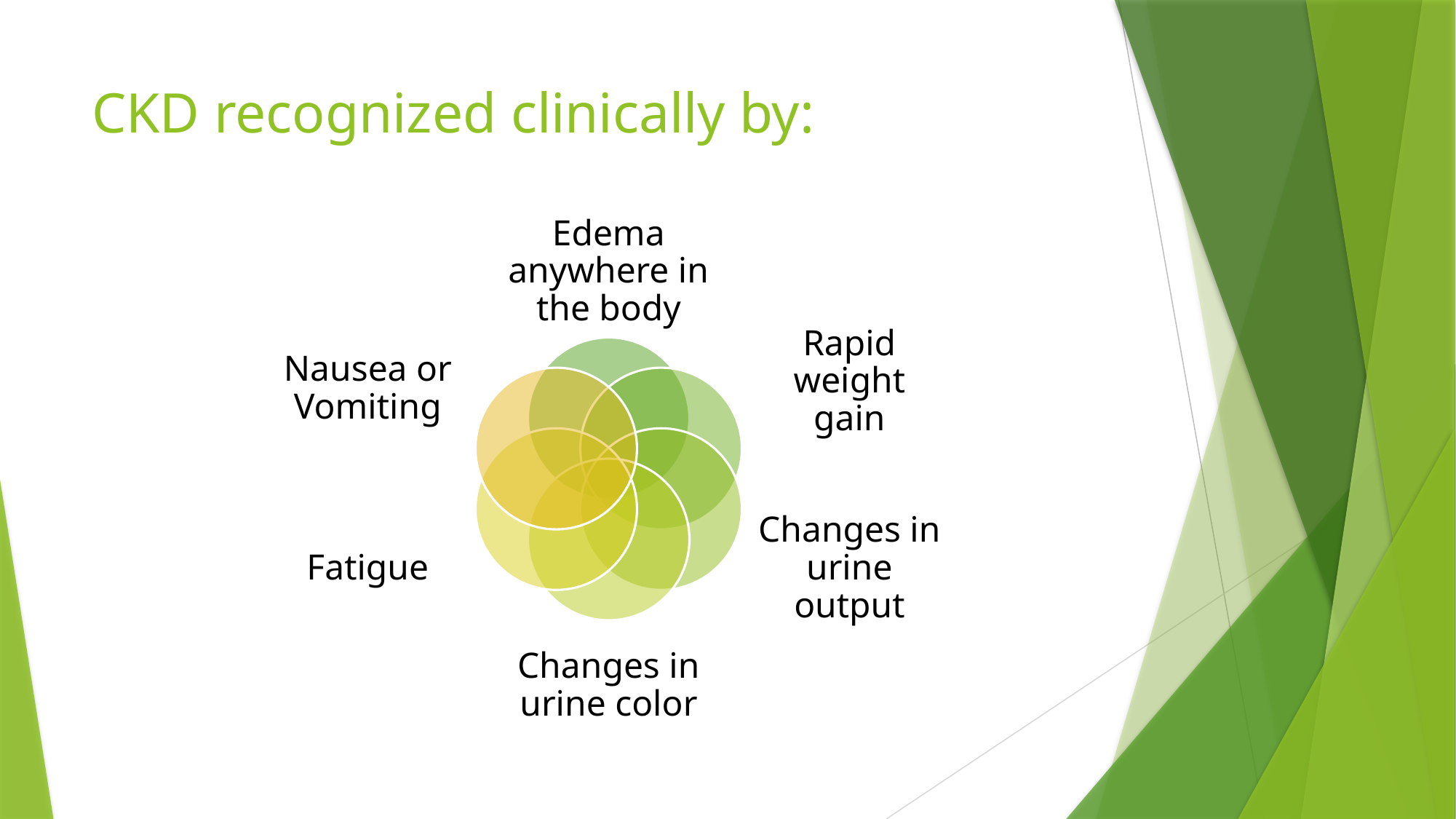

# CKD recognized clinically by:

## Slide 6
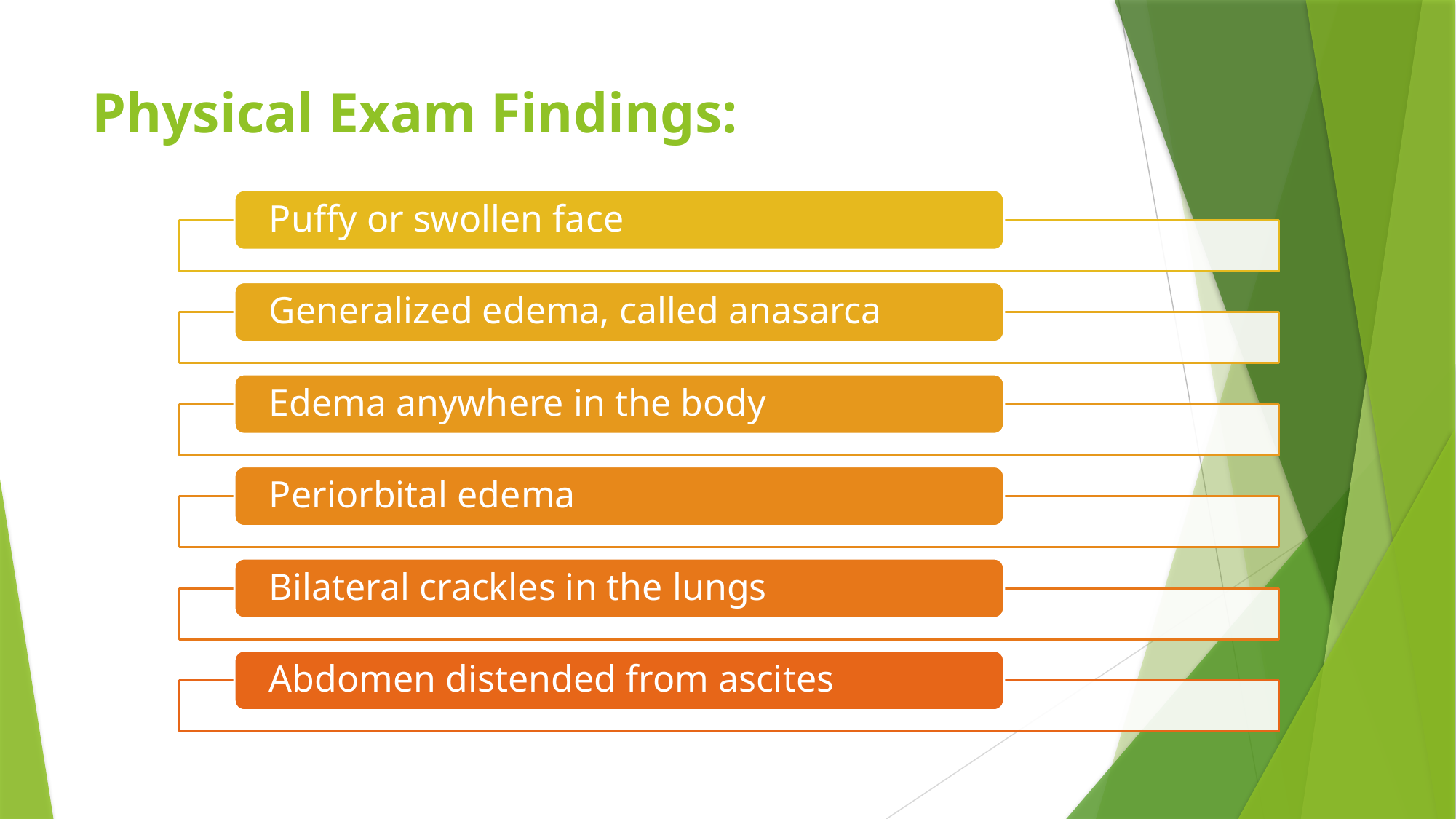

# Physical Exam Findings:

## Slide 7
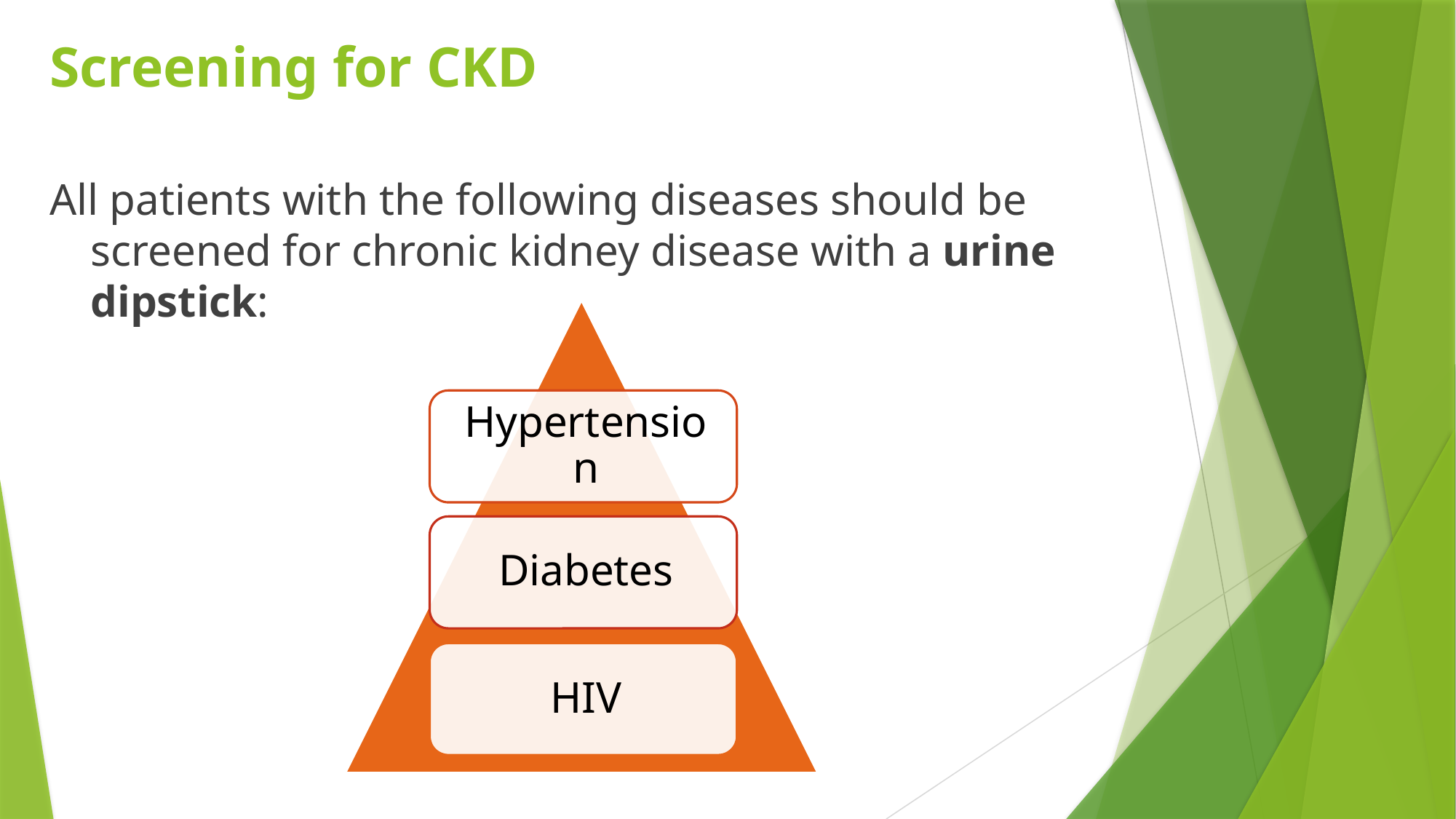

Screening for CKD
All patients with the following diseases should be screened for chronic kidney disease with a urine dipstick:

## Slide 8
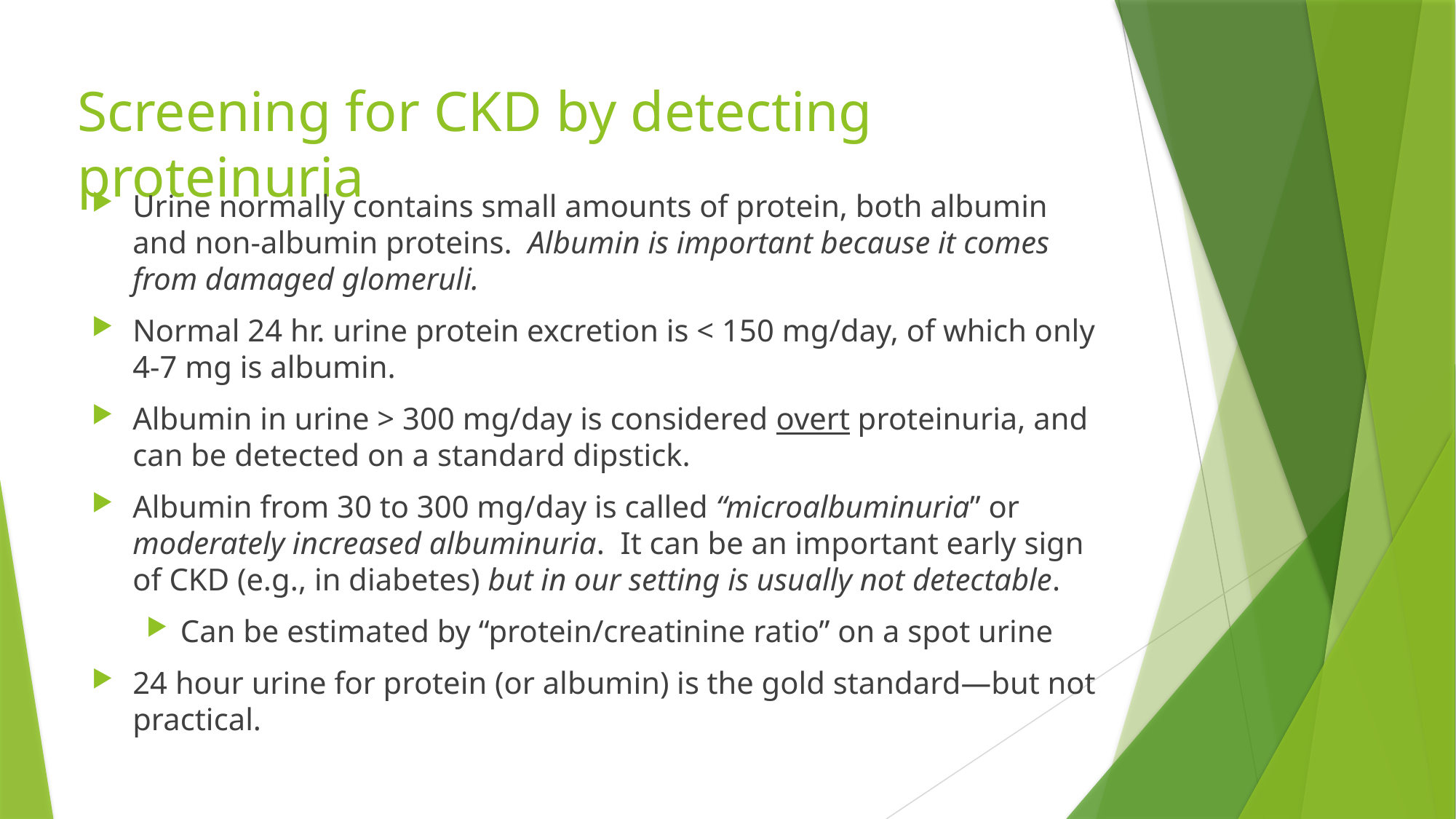

# Screening for CKD by detecting proteinuria
Urine normally contains small amounts of protein, both albumin and non-albumin proteins. Albumin is important because it comes from damaged glomeruli.
Normal 24 hr. urine protein excretion is < 150 mg/day, of which only 4-7 mg is albumin.
Albumin in urine > 300 mg/day is considered overt proteinuria, and can be detected on a standard dipstick.
Albumin from 30 to 300 mg/day is called “microalbuminuria” or moderately increased albuminuria. It can be an important early sign of CKD (e.g., in diabetes) but in our setting is usually not detectable.
Can be estimated by “protein/creatinine ratio” on a spot urine
24 hour urine for protein (or albumin) is the gold standard—but not practical.

## Slide 9
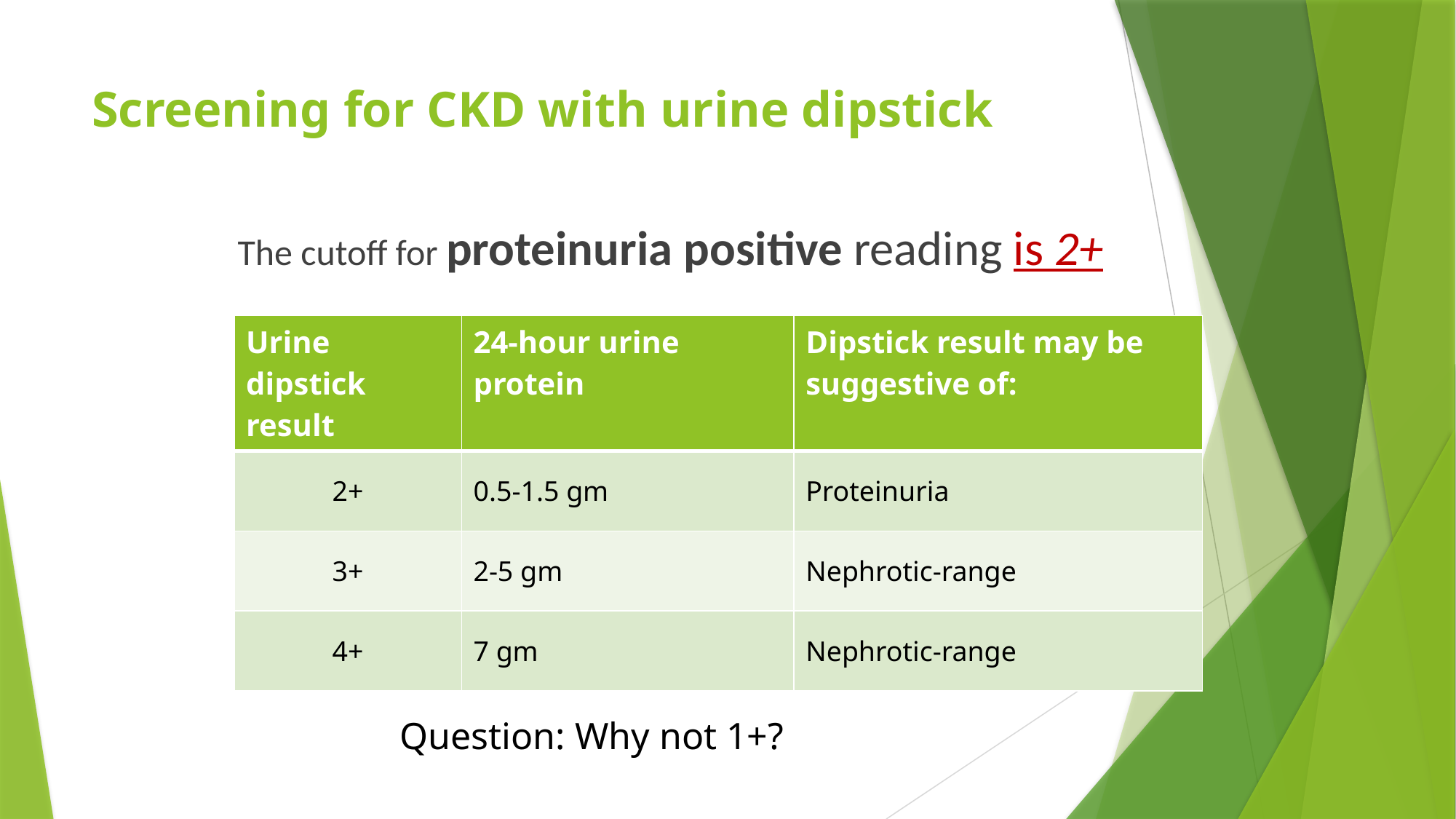

# Screening for CKD with urine dipstick
The cutoff for proteinuria positive reading is 2+
| Urine dipstick result | 24-hour urine protein | Dipstick result may be suggestive of: |
| --- | --- | --- |
| 2+ | 0.5-1.5 gm | Proteinuria |
| 3+ | 2-5 gm | Nephrotic-range |
| 4+ | 7 gm | Nephrotic-range |
 Question: Why not 1+?

## Slide 10
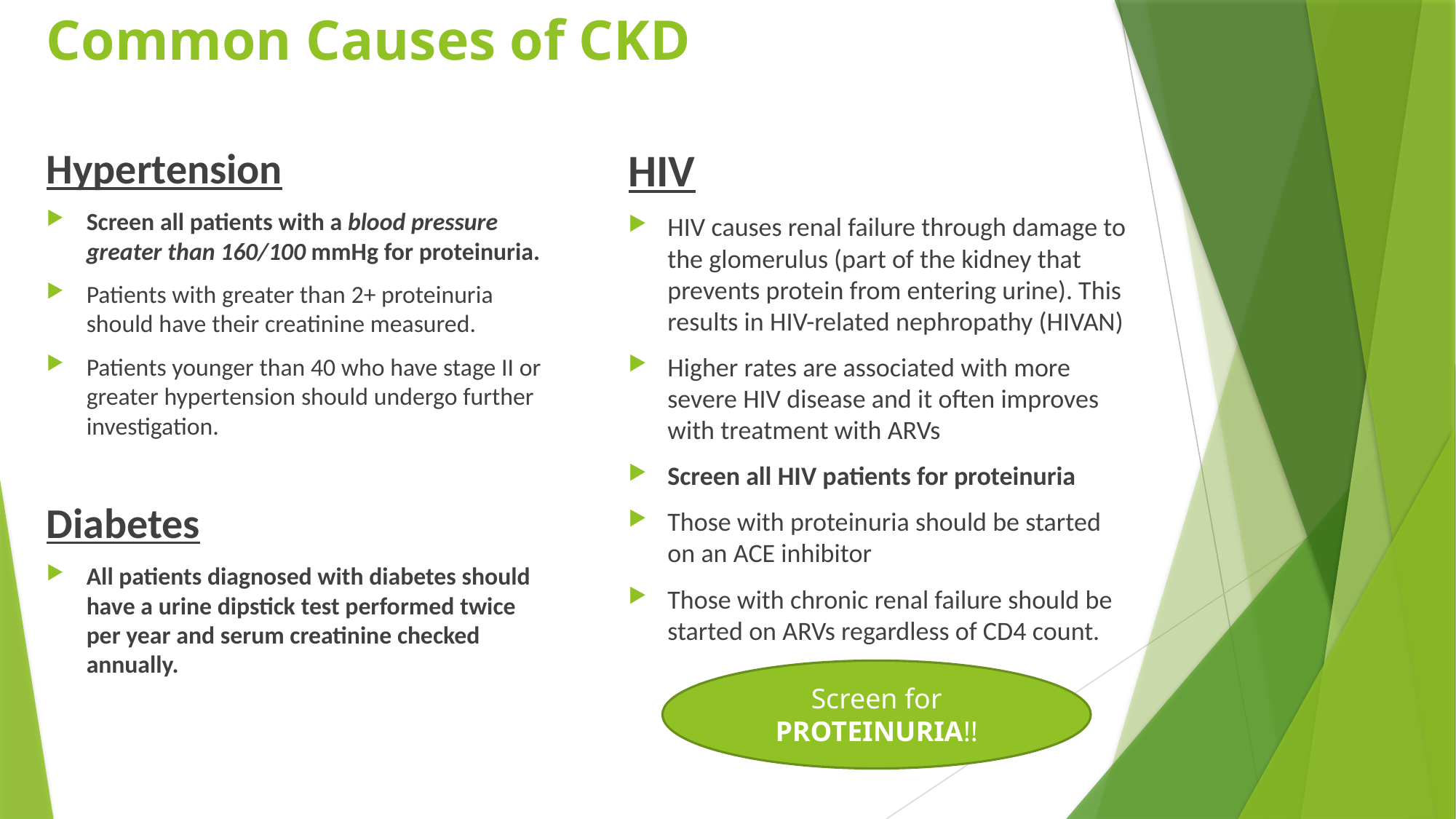

Common Causes of CKD
Hypertension
Screen all patients with a blood pressure greater than 160/100 mmHg for proteinuria.
Patients with greater than 2+ proteinuria should have their creatinine measured.
Patients younger than 40 who have stage II or greater hypertension should undergo further investigation.
Diabetes
All patients diagnosed with diabetes should have a urine dipstick test performed twice per year and serum creatinine checked annually.
HIV
HIV causes renal failure through damage to the glomerulus (part of the kidney that prevents protein from entering urine). This results in HIV-related nephropathy (HIVAN)
Higher rates are associated with more severe HIV disease and it often improves with treatment with ARVs
Screen all HIV patients for proteinuria
Those with proteinuria should be started on an ACE inhibitor
Those with chronic renal failure should be started on ARVs regardless of CD4 count.
Screen for PROTEINURIA!!

## Slide 11
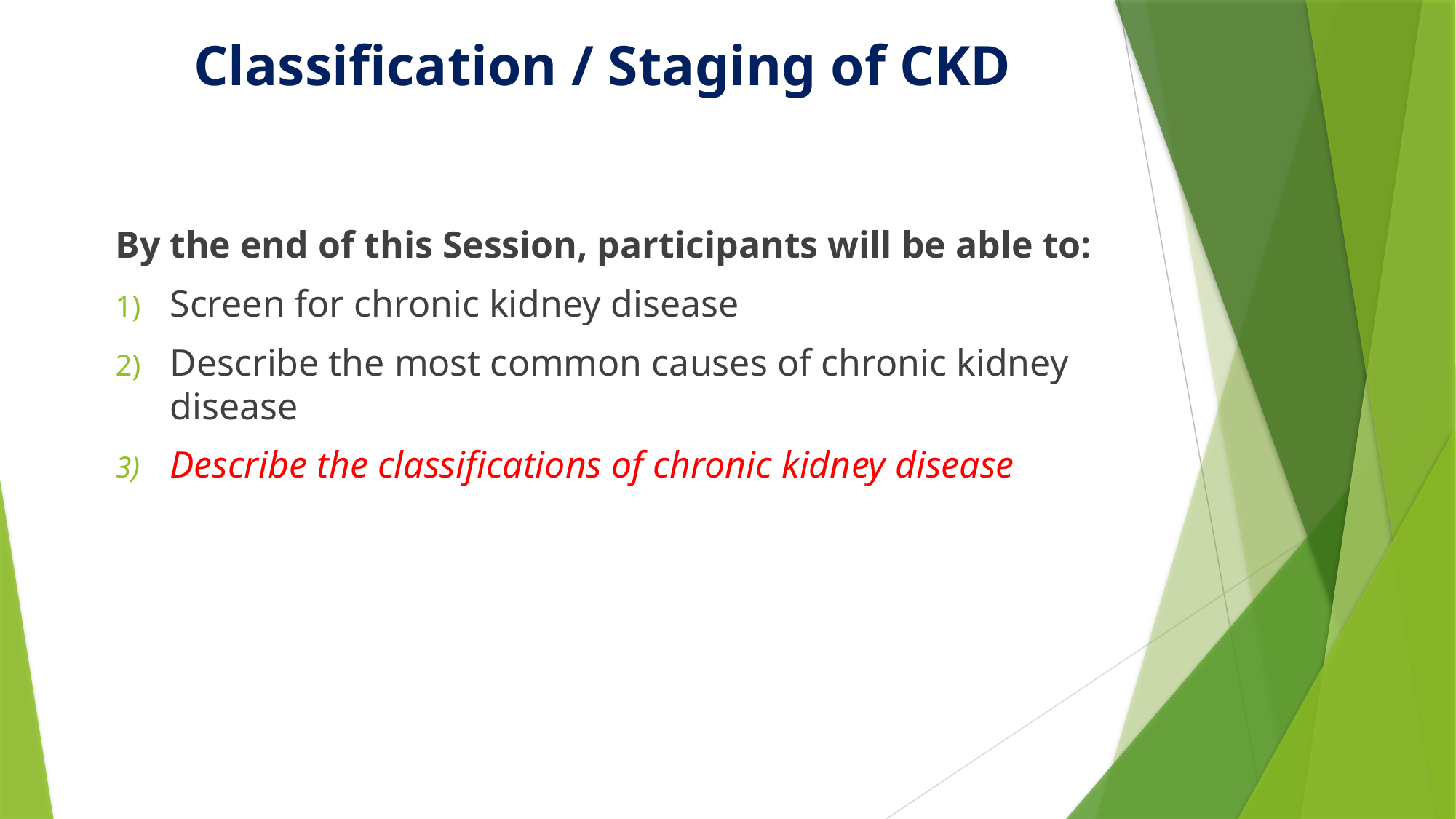

# Classification / Staging of CKD
By the end of this Session, participants will be able to:
Screen for chronic kidney disease
Describe the most common causes of chronic kidney disease
Describe the classifications of chronic kidney disease

## Slide 12
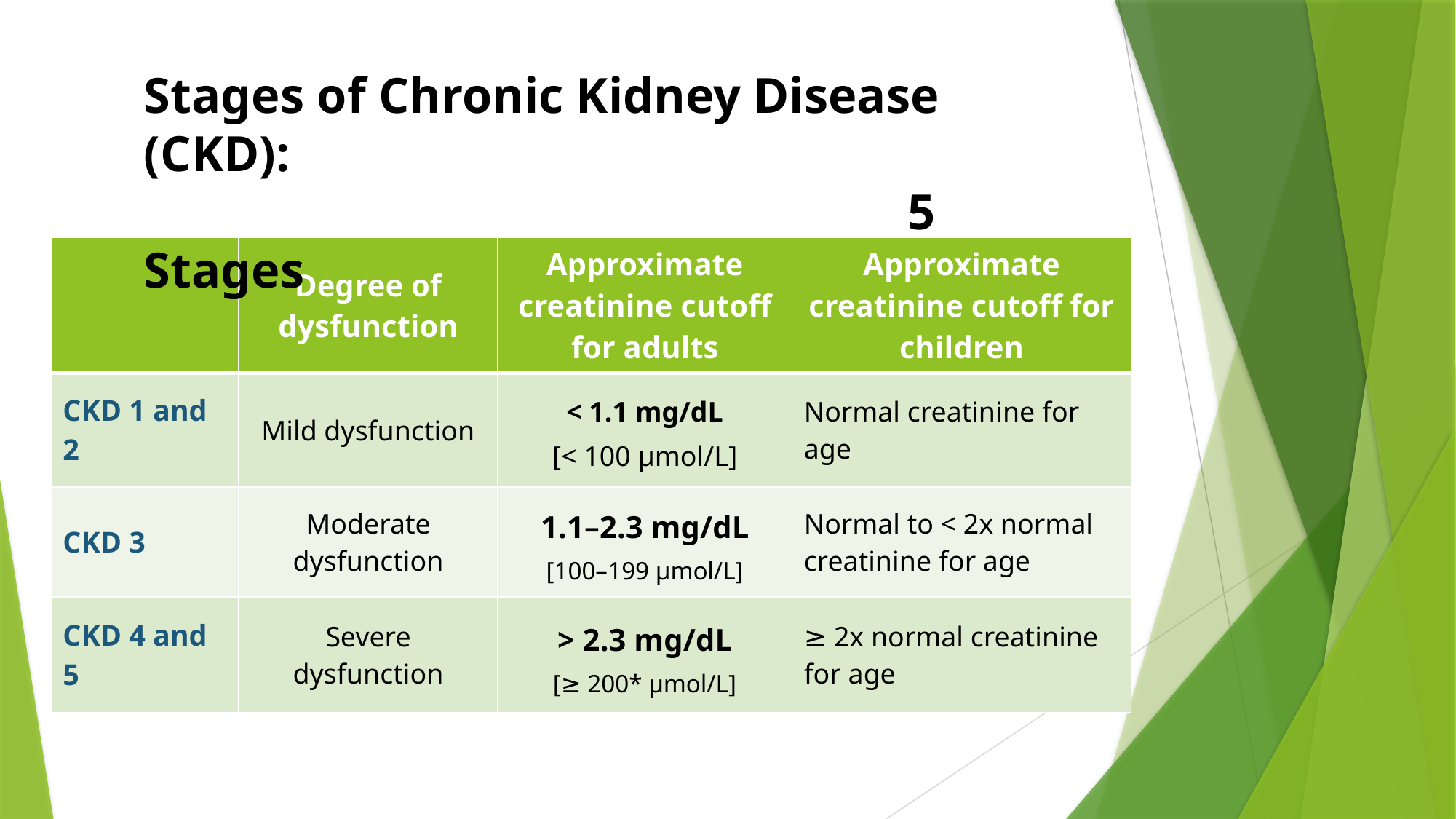

Stages of Chronic Kidney Disease (CKD):
							5 Stages
| | Degree of dysfunction | Approximate creatinine cutoff for adults | Approximate creatinine cutoff for children |
| --- | --- | --- | --- |
| CKD 1 and 2 | Mild dysfunction | < 1.1 mg/dL [< 100 µmol/L] | Normal creatinine for age |
| CKD 3 | Moderate dysfunction | 1.1–2.3 mg/dL [100–199 µmol/L] | Normal to < 2x normal creatinine for age |
| CKD 4 and 5 | Severe dysfunction | > 2.3 mg/dL [≥ 200\* µmol/L] | ≥ 2x normal creatinine for age |

## Slide 13
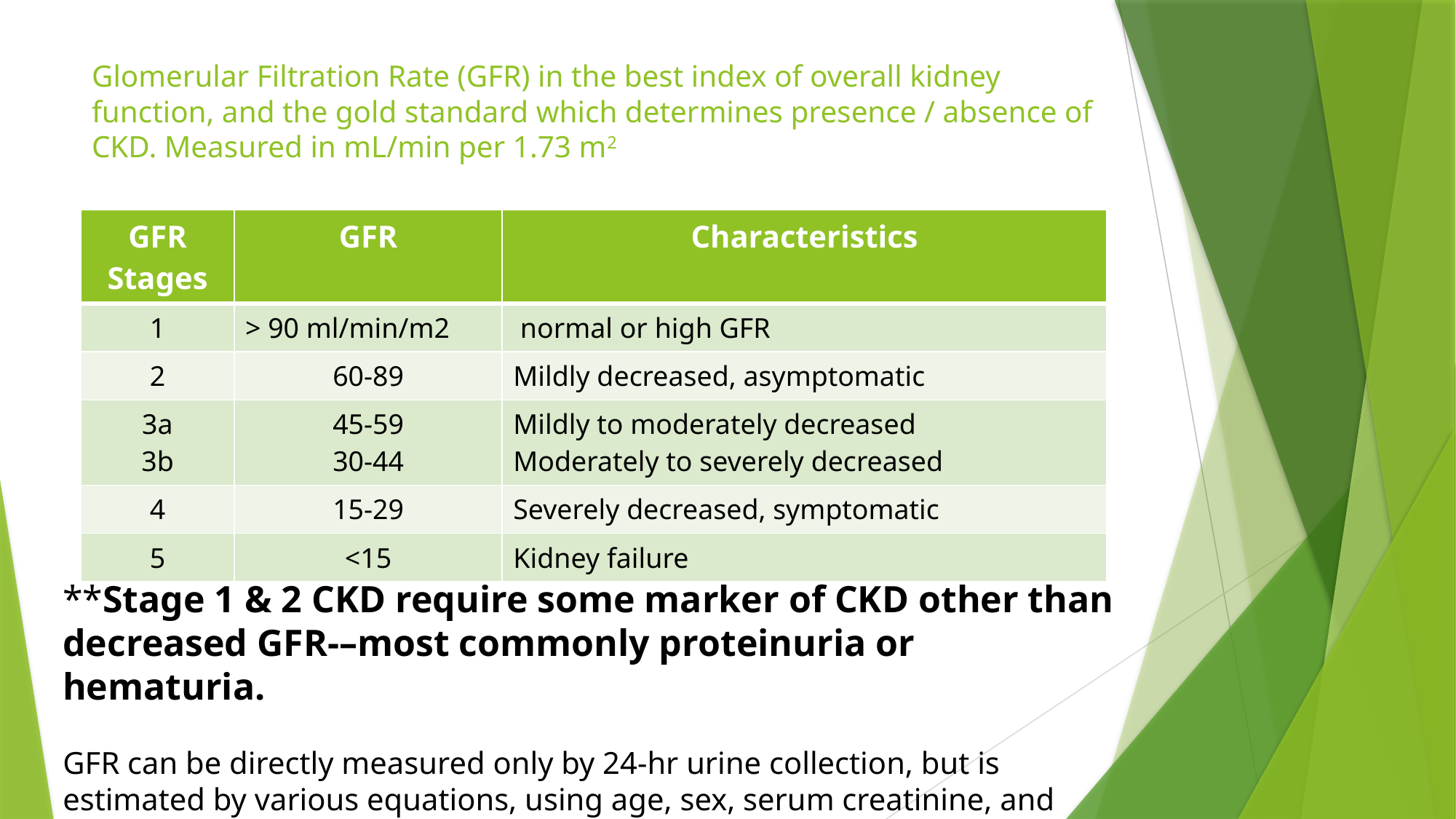

# Glomerular Filtration Rate (GFR) in the best index of overall kidney function, and the gold standard which determines presence / absence of CKD. Measured in mL/min per 1.73 m2
| GFR Stages | GFR | Characteristics |
| --- | --- | --- |
| 1 | > 90 ml/min/m2 | normal or high GFR |
| 2 | 60-89 | Mildly decreased, asymptomatic |
| 3a 3b | 45-59 30-44 | Mildly to moderately decreased Moderately to severely decreased |
| 4 | 15-29 | Severely decreased, symptomatic |
| 5 | <15 | Kidney failure |
**Stage 1 & 2 CKD require some marker of CKD other than decreased GFR-–most commonly proteinuria or hematuria.
GFR can be directly measured only by 24-hr urine collection, but is estimated by various equations, using age, sex, serum creatinine, and body weight.

## Slide 14
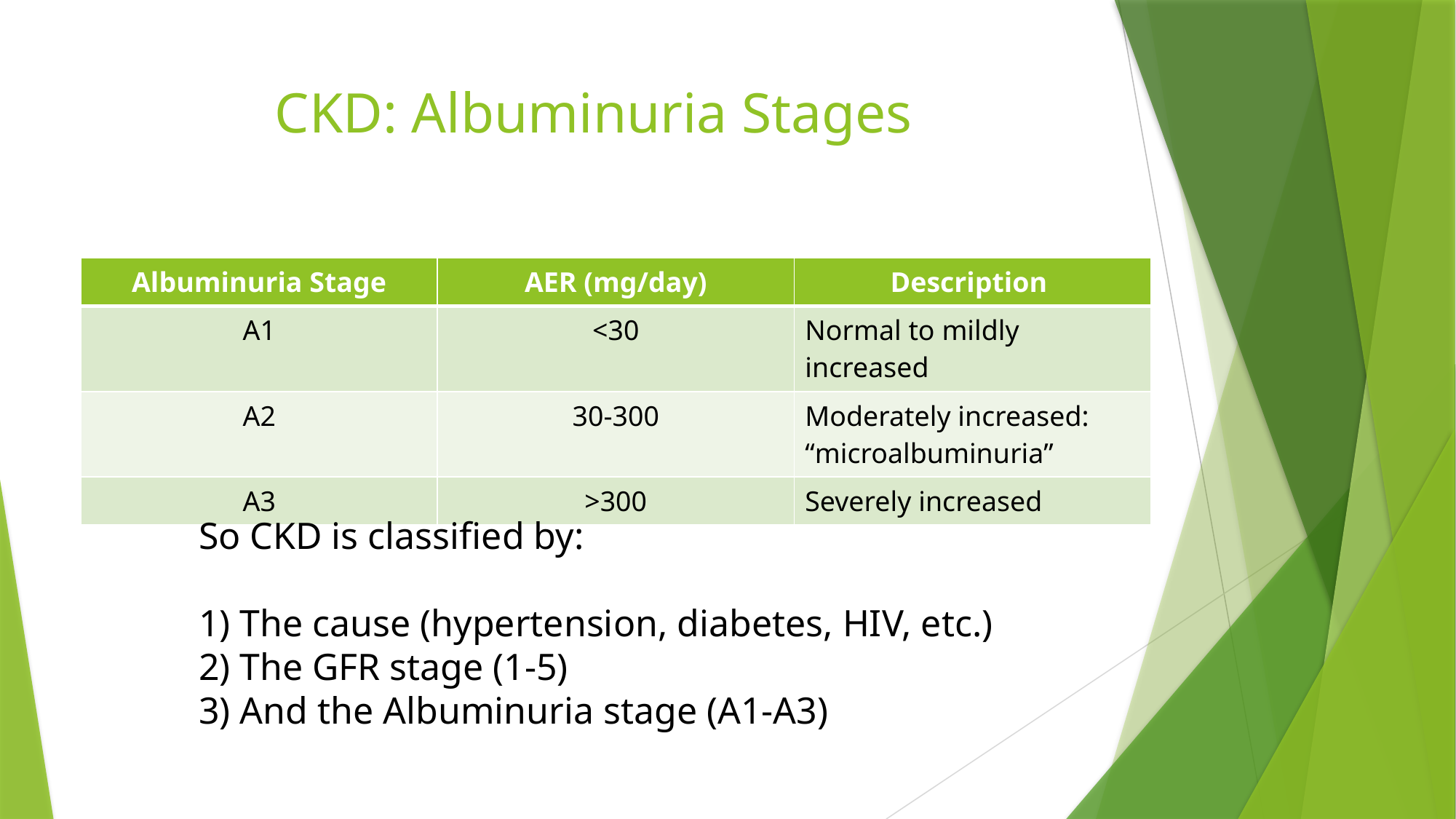

# CKD: Albuminuria Stages
| Albuminuria Stage | AER (mg/day) | Description |
| --- | --- | --- |
| A1 | <30 | Normal to mildly increased |
| A2 | 30-300 | Moderately increased: “microalbuminuria” |
| A3 | >300 | Severely increased |
So CKD is classified by:
The cause (hypertension, diabetes, HIV, etc.)
The GFR stage (1-5)
And the Albuminuria stage (A1-A3)

## Slide 15
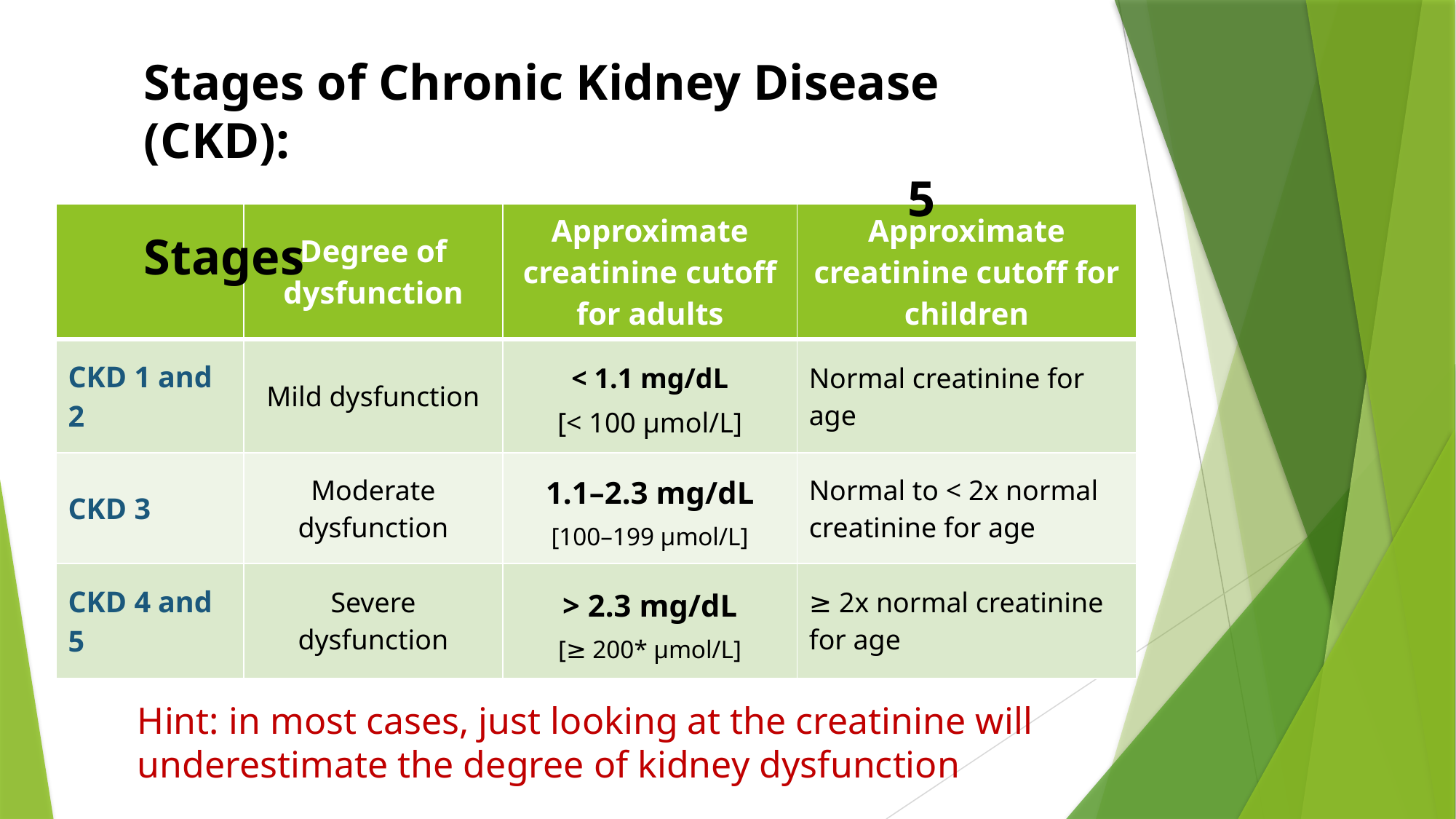

Stages of Chronic Kidney Disease (CKD):
							5 Stages
| | Degree of dysfunction | Approximate creatinine cutoff for adults | Approximate creatinine cutoff for children |
| --- | --- | --- | --- |
| CKD 1 and 2 | Mild dysfunction | < 1.1 mg/dL [< 100 µmol/L] | Normal creatinine for age |
| CKD 3 | Moderate dysfunction | 1.1–2.3 mg/dL [100–199 µmol/L] | Normal to < 2x normal creatinine for age |
| CKD 4 and 5 | Severe dysfunction | > 2.3 mg/dL [≥ 200\* µmol/L] | ≥ 2x normal creatinine for age |
Hint: in most cases, just looking at the creatinine will underestimate the degree of kidney dysfunction

## Slide 16
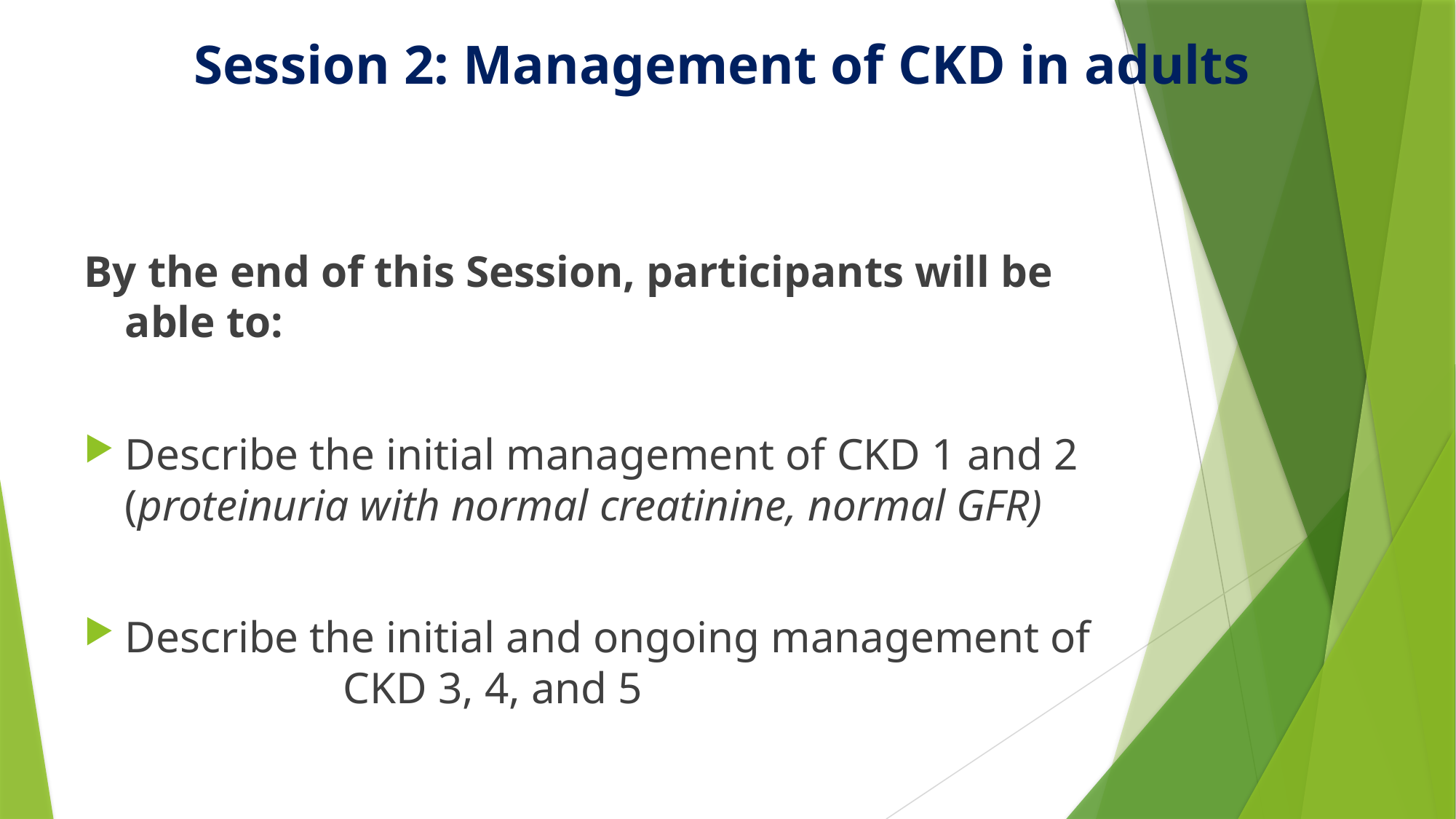

# Session 2: Management of CKD in adults
By the end of this Session, participants will be able to:
Describe the initial management of CKD 1 and 2 (proteinuria with normal creatinine, normal GFR)
Describe the initial and ongoing management of 	CKD 3, 4, and 5

## Slide 17
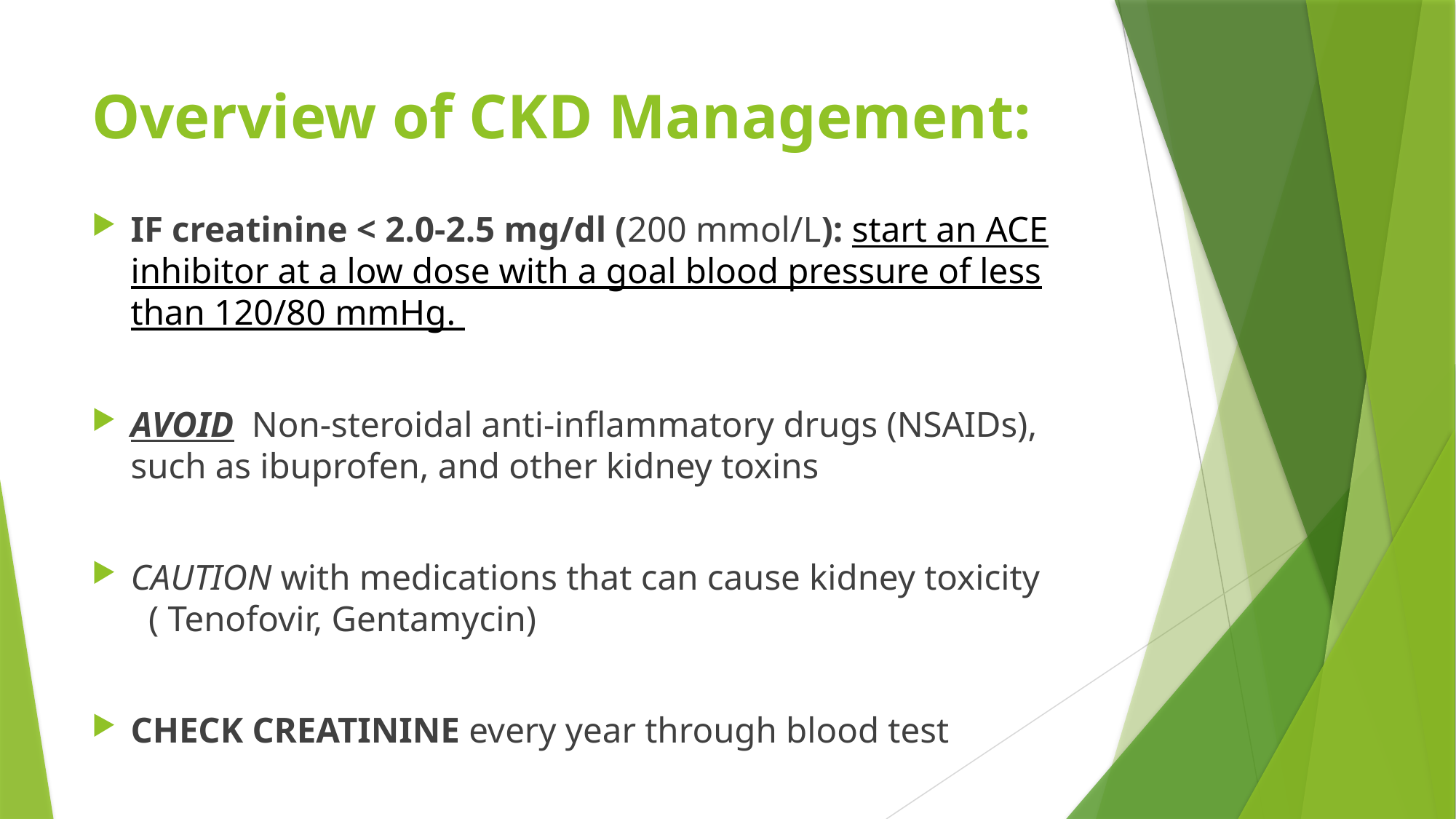

# Overview of CKD Management:
IF creatinine < 2.0-2.5 mg/dl (200 mmol/L): start an ACE inhibitor at a low dose with a goal blood pressure of less than 120/80 mmHg.
AVOID Non-steroidal anti-inflammatory drugs (NSAIDs), such as ibuprofen, and other kidney toxins
CAUTION with medications that can cause kidney toxicity ( Tenofovir, Gentamycin)
CHECK CREATININE every year through blood test

## Slide 18
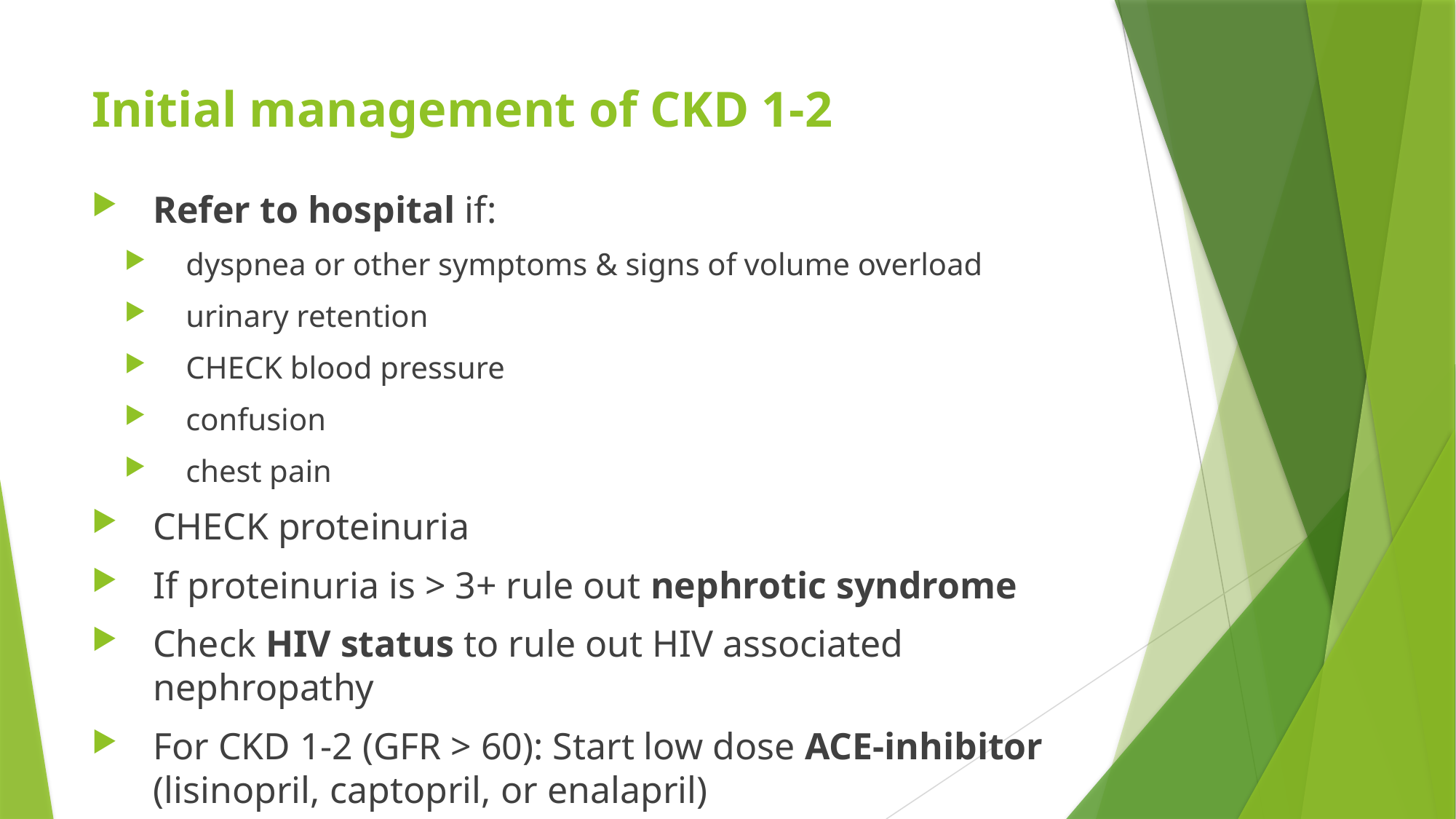

# Initial management of CKD 1-2
Refer to hospital if:
dyspnea or other symptoms & signs of volume overload
urinary retention
CHECK blood pressure
confusion
chest pain
CHECK proteinuria
If proteinuria is > 3+ rule out nephrotic syndrome
Check HIV status to rule out HIV associated nephropathy
For CKD 1-2 (GFR > 60): Start low dose ACE-inhibitor (lisinopril, captopril, or enalapril)

## Slide 19
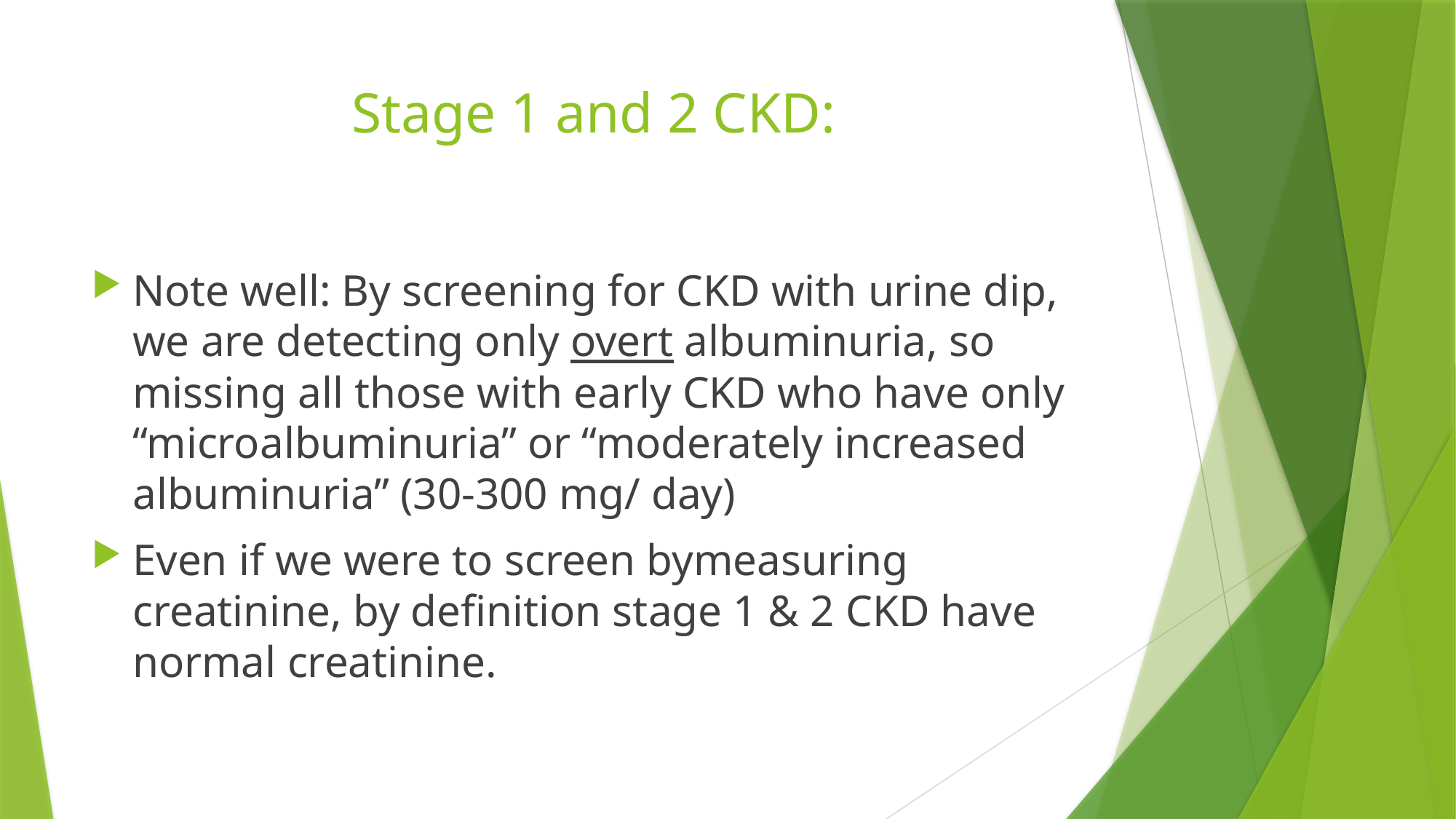

# Stage 1 and 2 CKD:
Note well: By screening for CKD with urine dip, we are detecting only overt albuminuria, so missing all those with early CKD who have only “microalbuminuria” or “moderately increased albuminuria” (30-300 mg/ day)
Even if we were to screen bymeasuring creatinine, by definition stage 1 & 2 CKD have normal creatinine.

## Slide 20
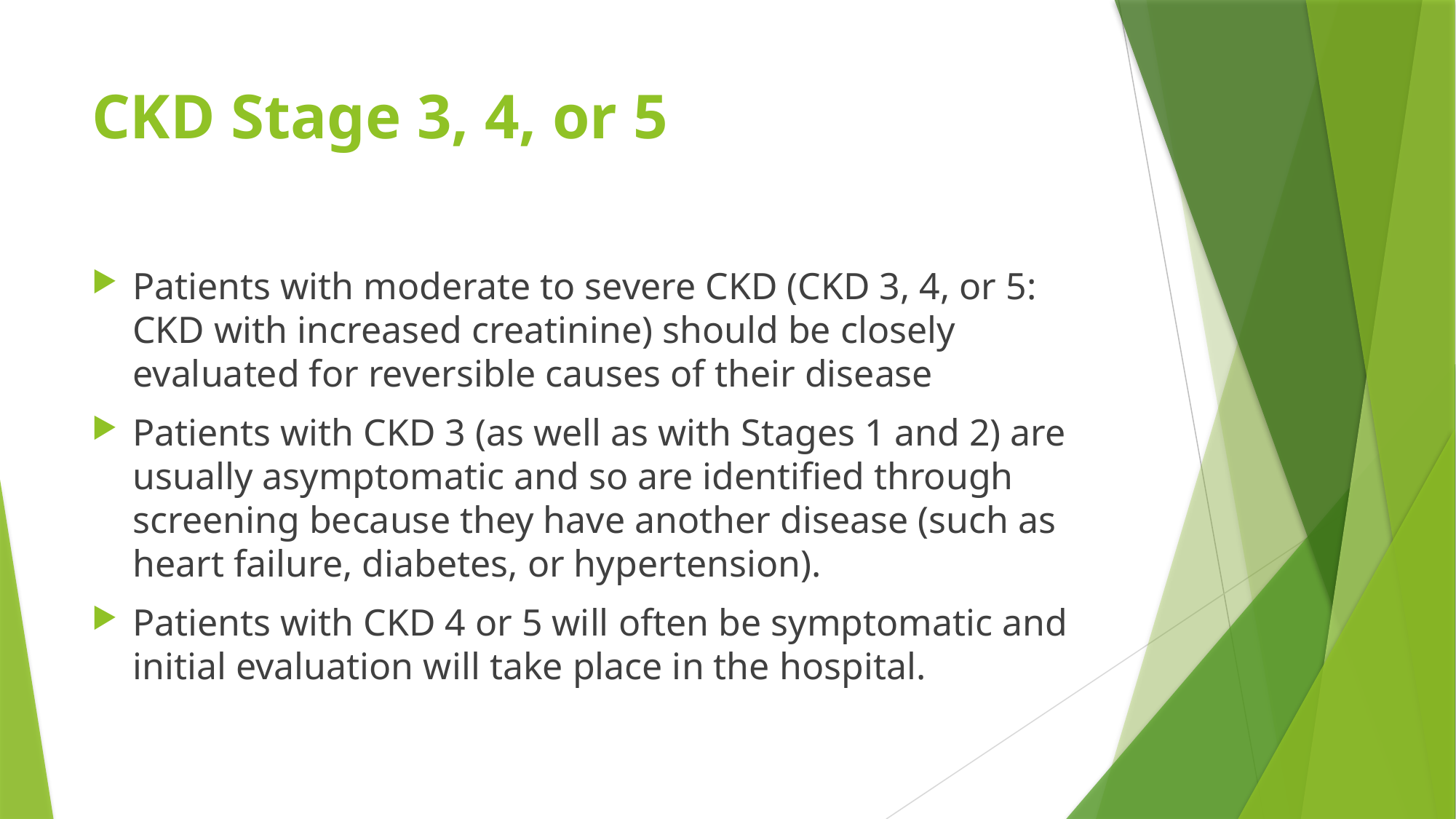

# CKD Stage 3, 4, or 5
Patients with moderate to severe CKD (CKD 3, 4, or 5: CKD with increased creatinine) should be closely evaluated for reversible causes of their disease
Patients with CKD 3 (as well as with Stages 1 and 2) are usually asymptomatic and so are identified through screening because they have another disease (such as heart failure, diabetes, or hypertension).
Patients with CKD 4 or 5 will often be symptomatic and initial evaluation will take place in the hospital.

## Slide 21
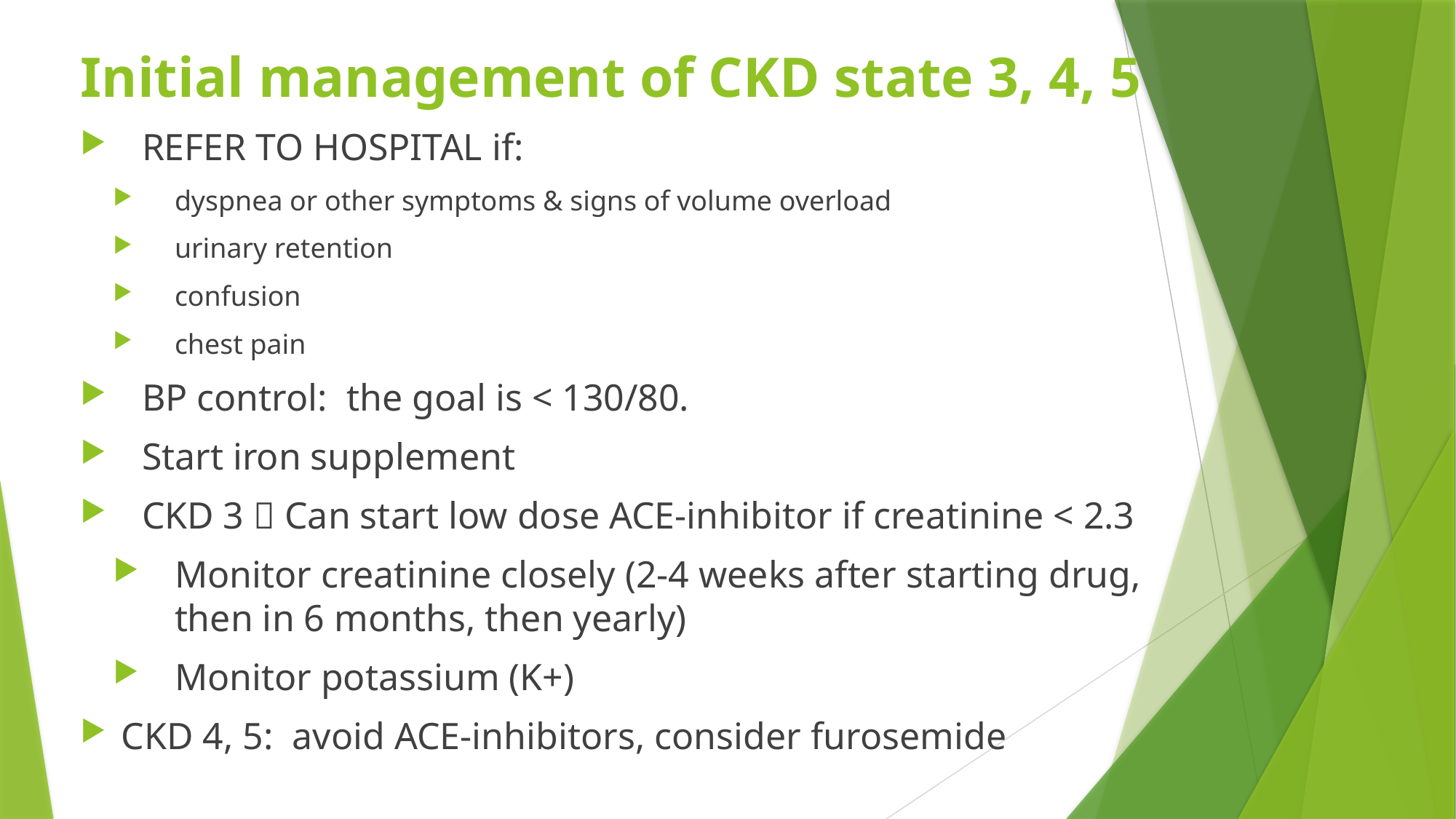

# Initial management of CKD state 3, 4, 5
REFER TO HOSPITAL if:
dyspnea or other symptoms & signs of volume overload
urinary retention
confusion
chest pain
BP control: the goal is < 130/80.
Start iron supplement
CKD 3  Can start low dose ACE-inhibitor if creatinine < 2.3
Monitor creatinine closely (2-4 weeks after starting drug, then in 6 months, then yearly)
Monitor potassium (K+)
CKD 4, 5: avoid ACE-inhibitors, consider furosemide

## Slide 22
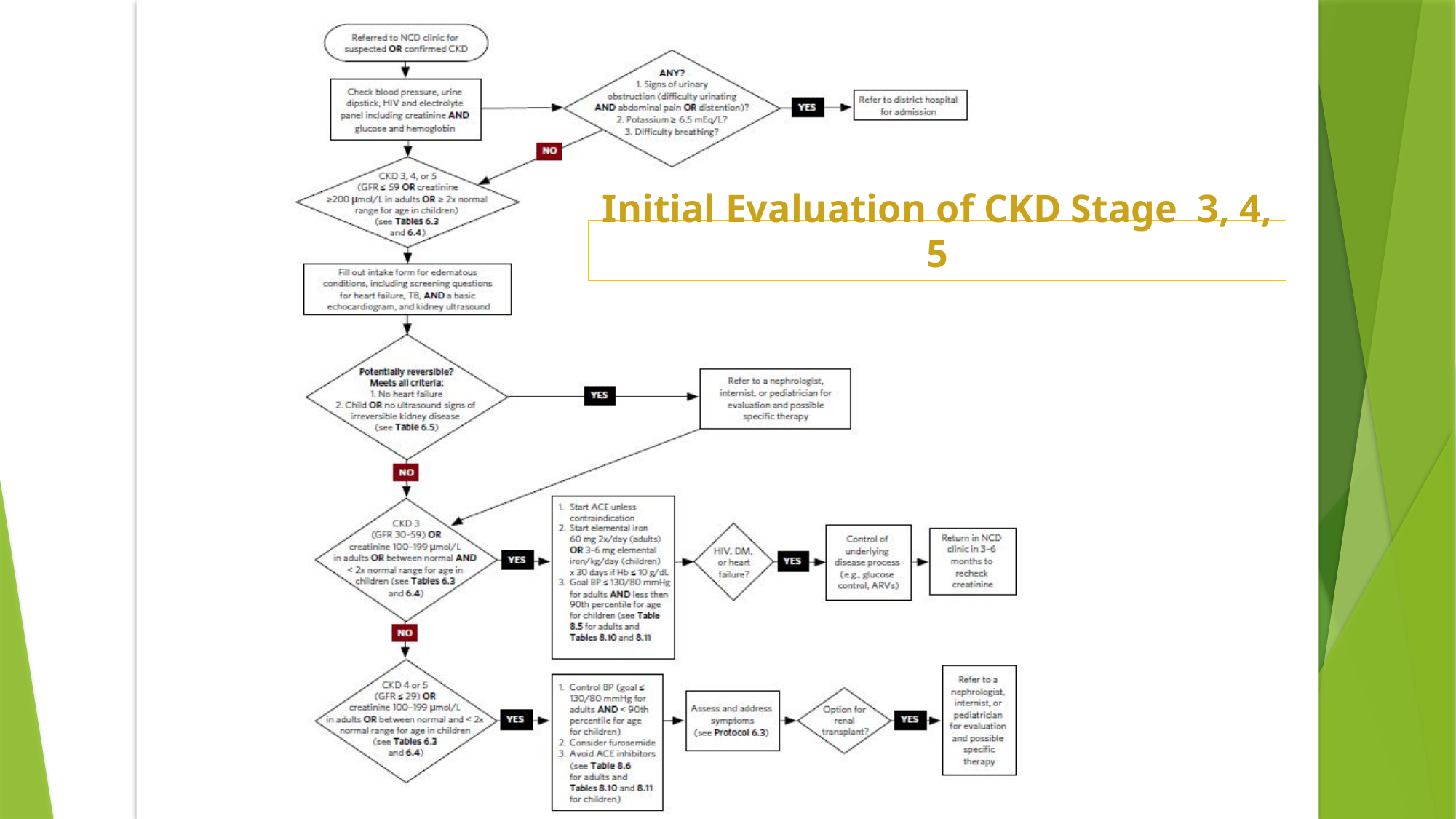

Initial Evaluation of CKD Stage 3, 4, 5

## Slide 23
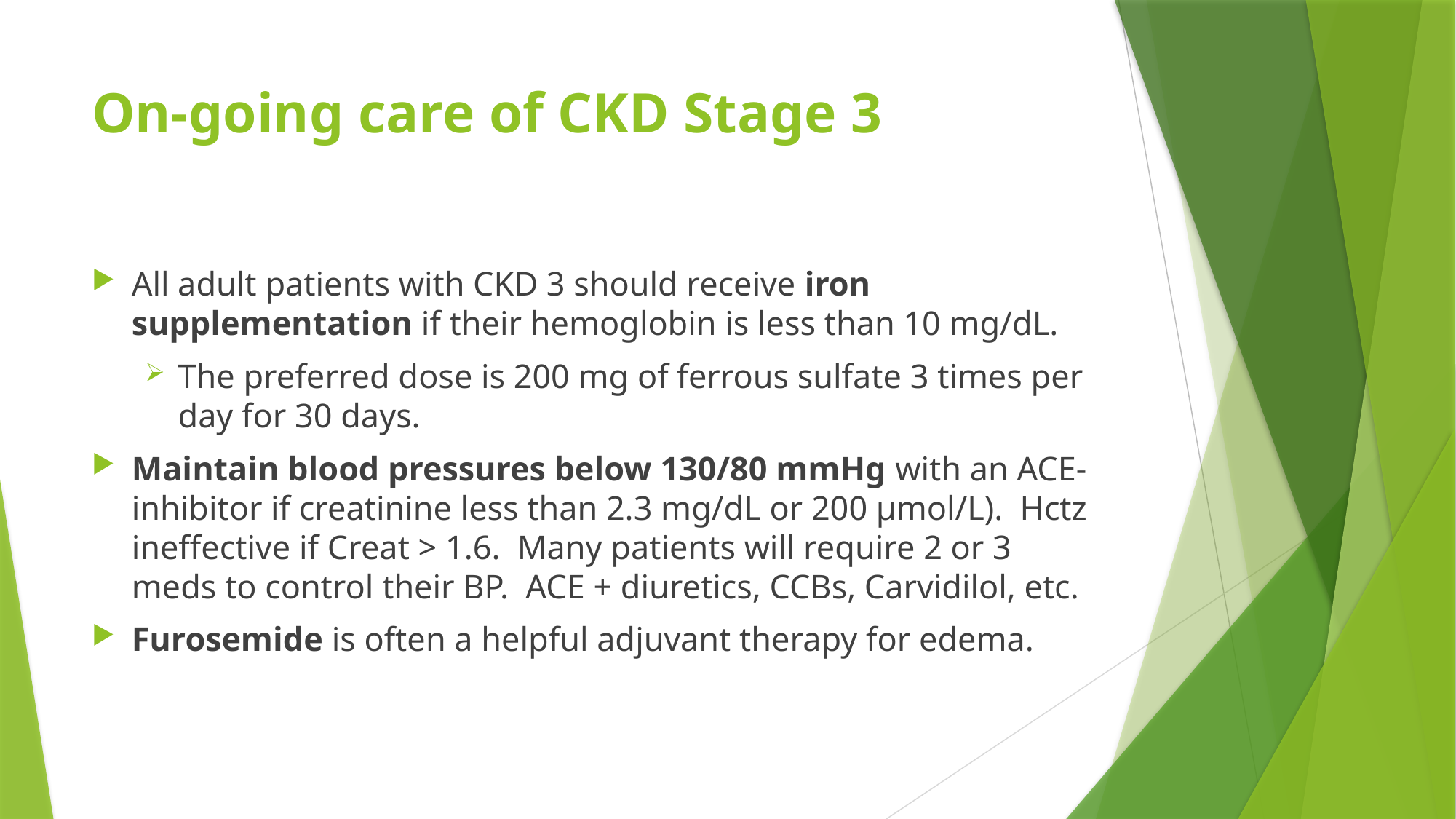

# On-going care of CKD Stage 3
All adult patients with CKD 3 should receive iron supplementation if their hemoglobin is less than 10 mg/dL.
The preferred dose is 200 mg of ferrous sulfate 3 times per day for 30 days.
Maintain blood pressures below 130/80 mmHg with an ACE-inhibitor if creatinine less than 2.3 mg/dL or 200 µmol/L). Hctz ineffective if Creat > 1.6. Many patients will require 2 or 3 meds to control their BP. ACE + diuretics, CCBs, Carvidilol, etc.
Furosemide is often a helpful adjuvant therapy for edema.

## Slide 24
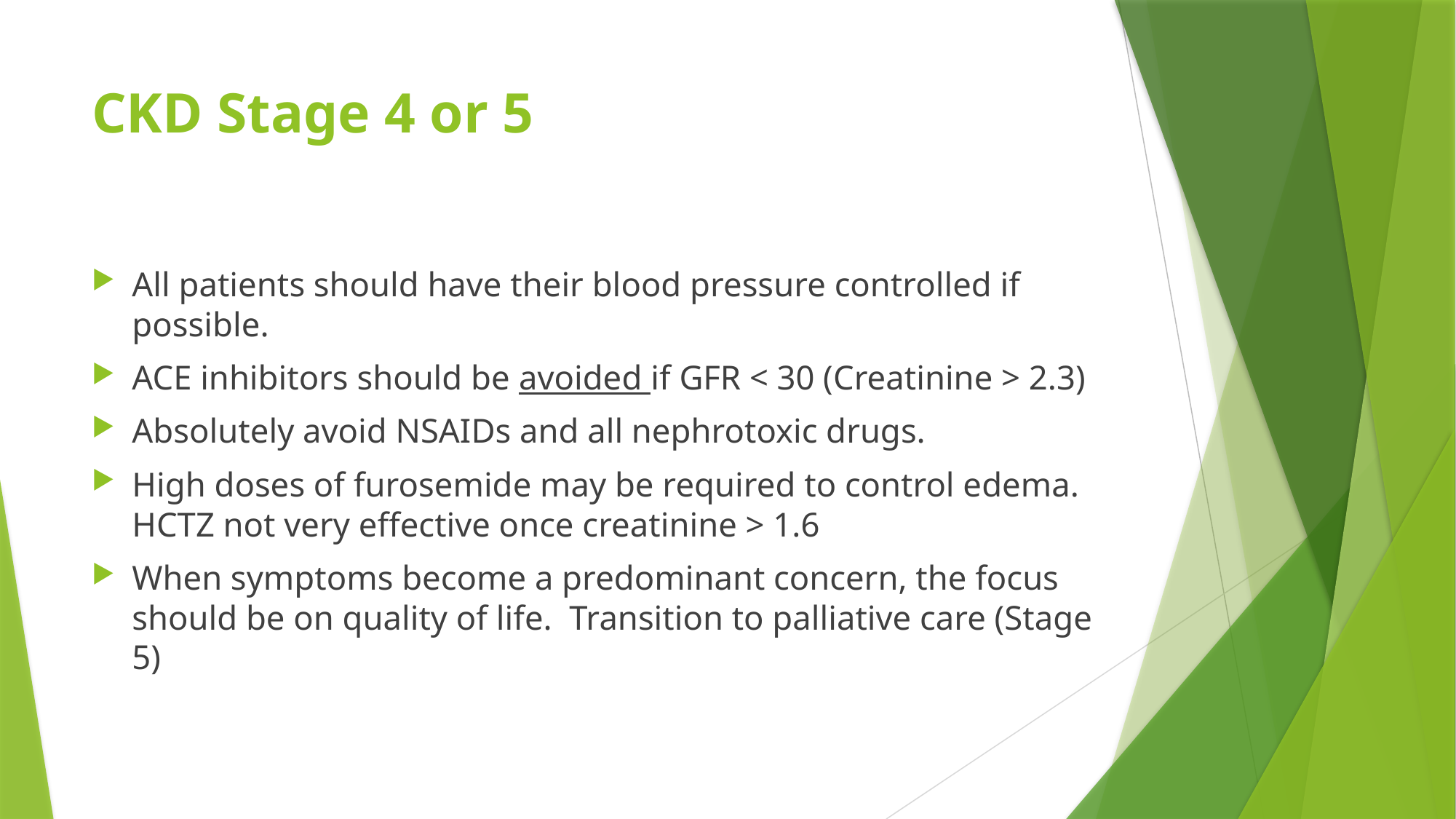

# CKD Stage 4 or 5
All patients should have their blood pressure controlled if possible.
ACE inhibitors should be avoided if GFR < 30 (Creatinine > 2.3)
Absolutely avoid NSAIDs and all nephrotoxic drugs.
High doses of furosemide may be required to control edema. HCTZ not very effective once creatinine > 1.6
When symptoms become a predominant concern, the focus should be on quality of life. Transition to palliative care (Stage 5)

## Slide 25
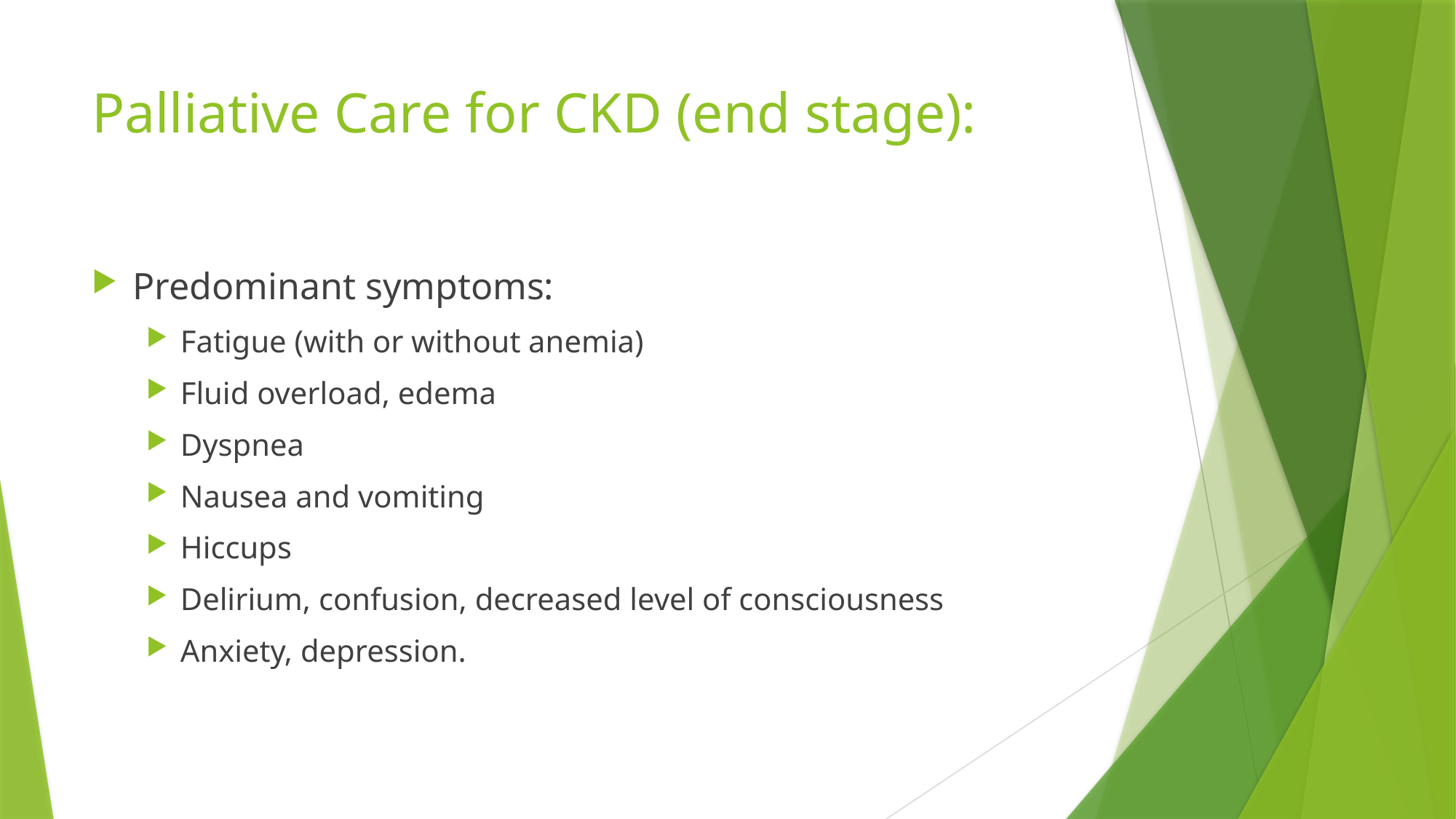

# Palliative Care for CKD (end stage):
Predominant symptoms:
Fatigue (with or without anemia)
Fluid overload, edema
Dyspnea
Nausea and vomiting
Hiccups
Delirium, confusion, decreased level of consciousness
Anxiety, depression.

## Slide 26
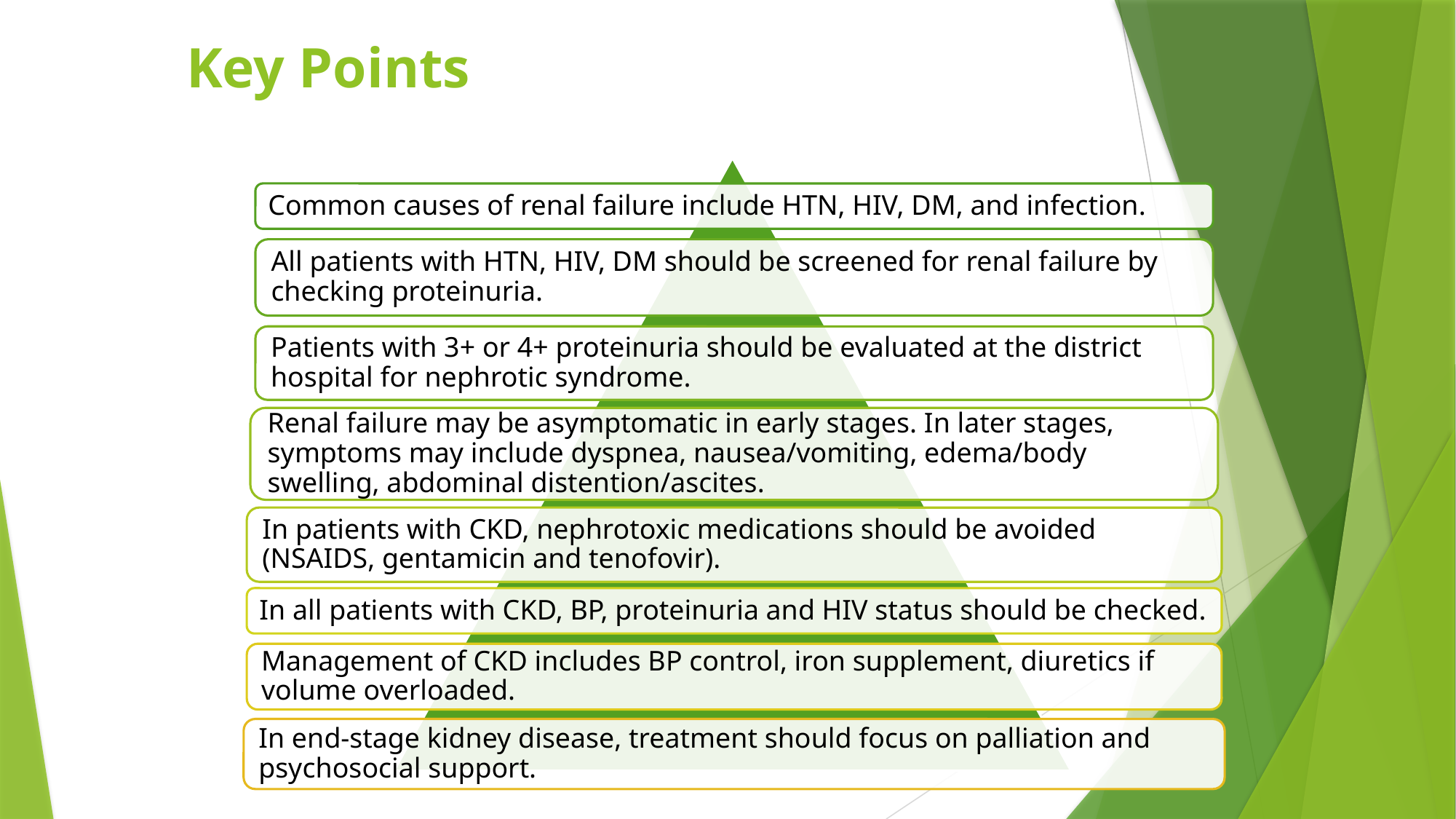

# Key Points

## Slide 27
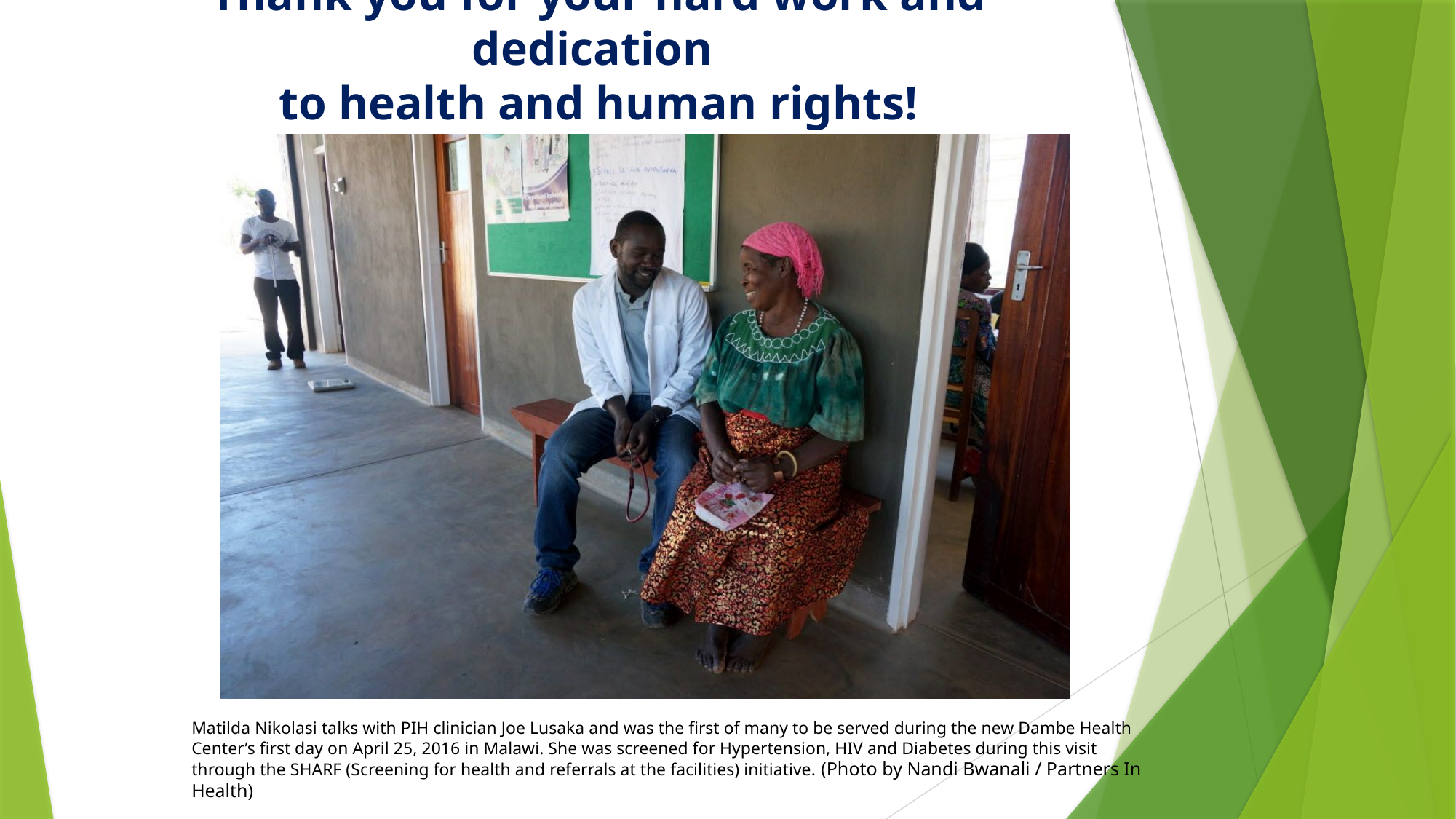

Thank you for your hard work and dedication
to health and human rights!
Matilda Nikolasi talks with PIH clinician Joe Lusaka and was the first of many to be served during the new Dambe Health Center’s first day on April 25, 2016 in Malawi. She was screened for Hypertension, HIV and Diabetes during this visit through the SHARF (Screening for health and referrals at the facilities) initiative. (Photo by Nandi Bwanali / Partners In Health)
