# Supplementary material for: Training Mid-Level Providers to Treat Severe Non-Communicable Diseases in Neno, Malawi through PEN-Plus Strategies
Source: Ann Glob Health. 2022 Aug 11;88(1):69. doi: 10.5334/aogh.3750 (PMC9389951; doi:10.5334/aogh.3750)
Supplement: Didactic Materials. — The supplementary materials contain a suggested didactic training schedule and the PowerPoint presentations used for PEN-Plus training in Neno, Malawi. These materials have been reviewed and accepted by the Malawi Ministry of Health for future PEN-Plus trainings in Malawi. [file agh-88-1-3750-s2.zip › Didactic_Materials/R_Electrolytes.pptx]

## Slide 1
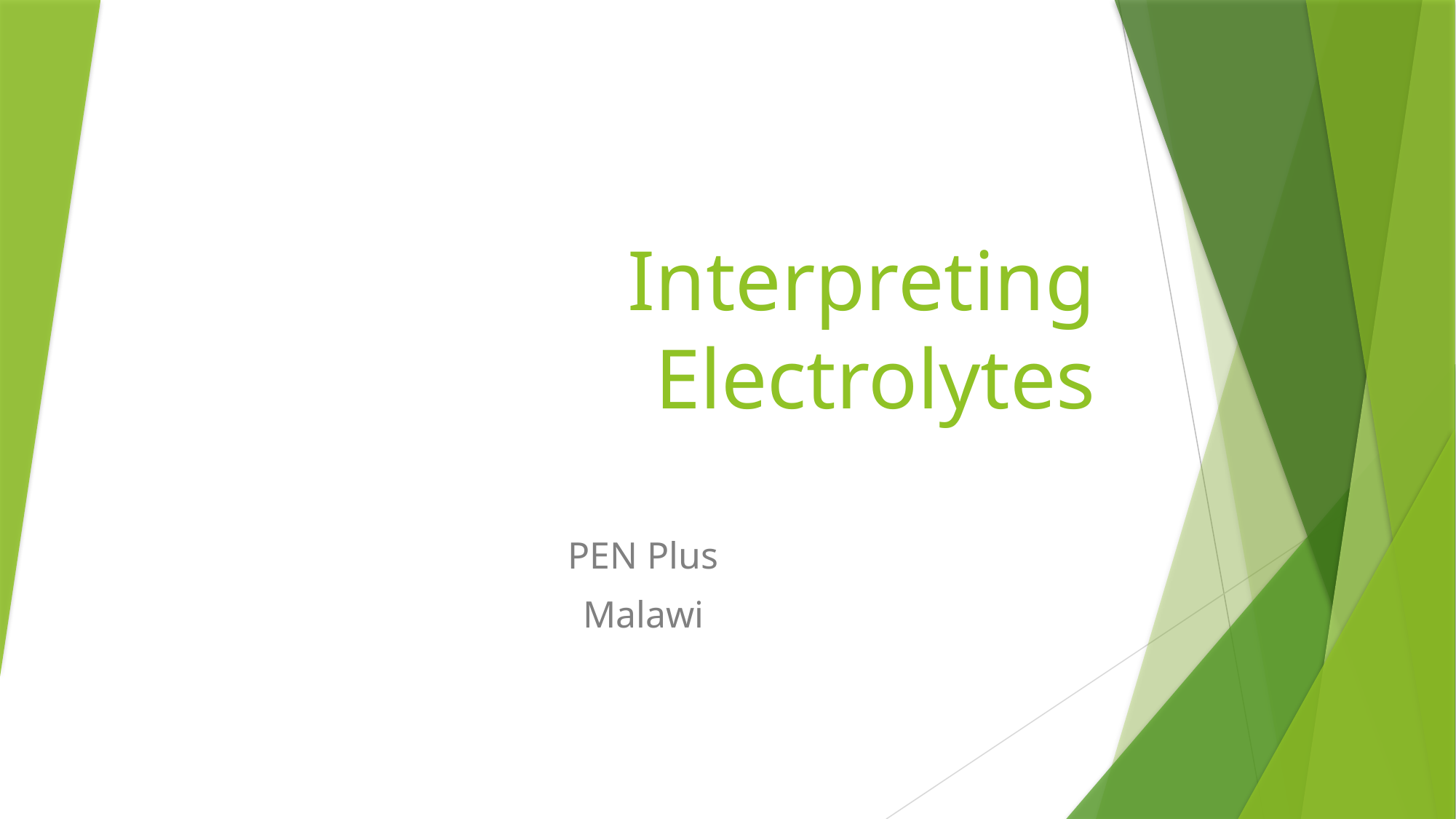

# Interpreting Electrolytes
PEN Plus
Malawi

## Slide 2
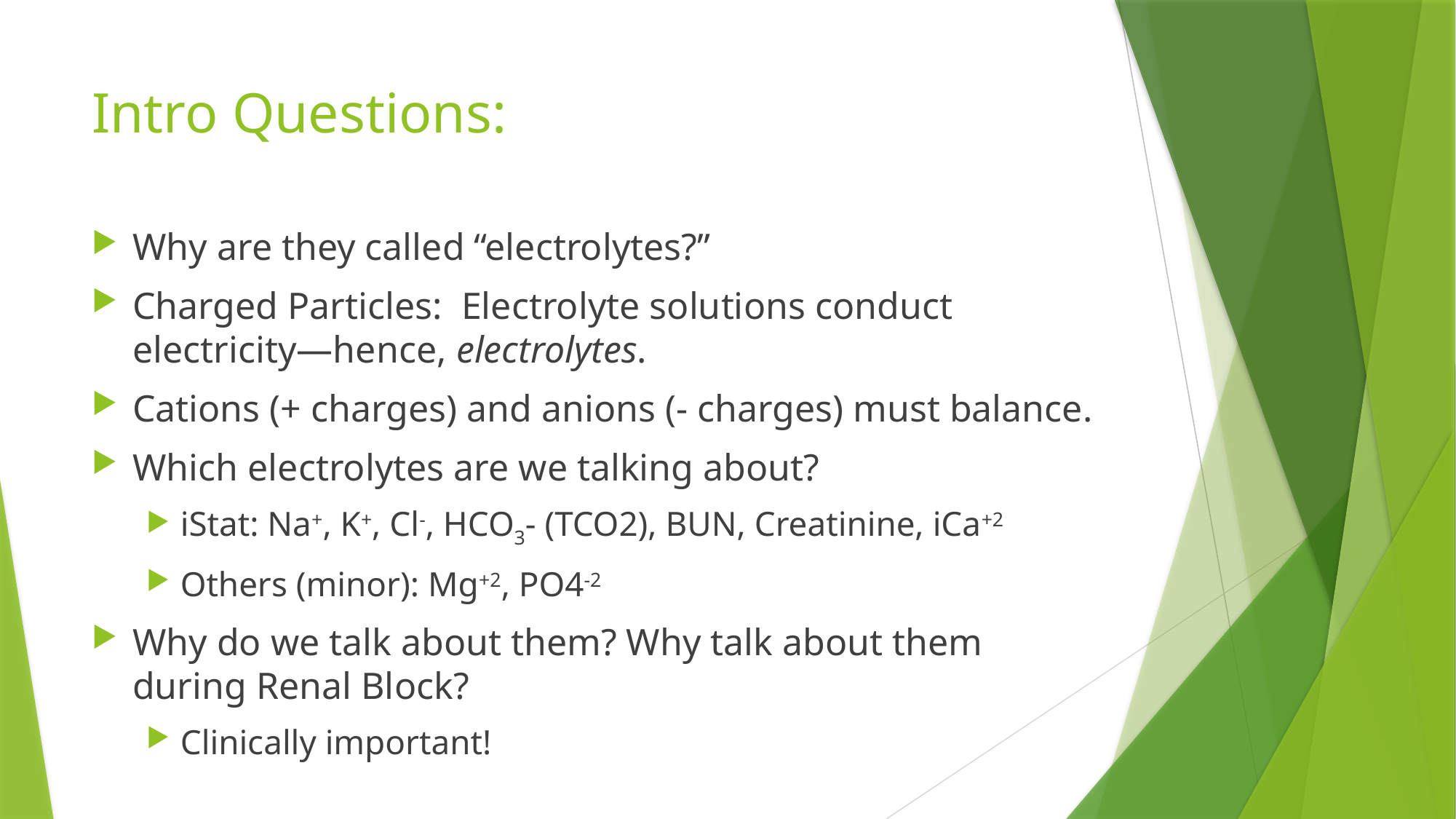

# Intro Questions:
Why are they called “electrolytes?”
Charged Particles: Electrolyte solutions conduct electricity—hence, electrolytes.
Cations (+ charges) and anions (- charges) must balance.
Which electrolytes are we talking about?
iStat: Na+, K+, Cl-, HCO3- (TCO2), BUN, Creatinine, iCa+2
Others (minor): Mg+2, PO4-2
Why do we talk about them? Why talk about them during Renal Block?
Clinically important!

## Slide 3
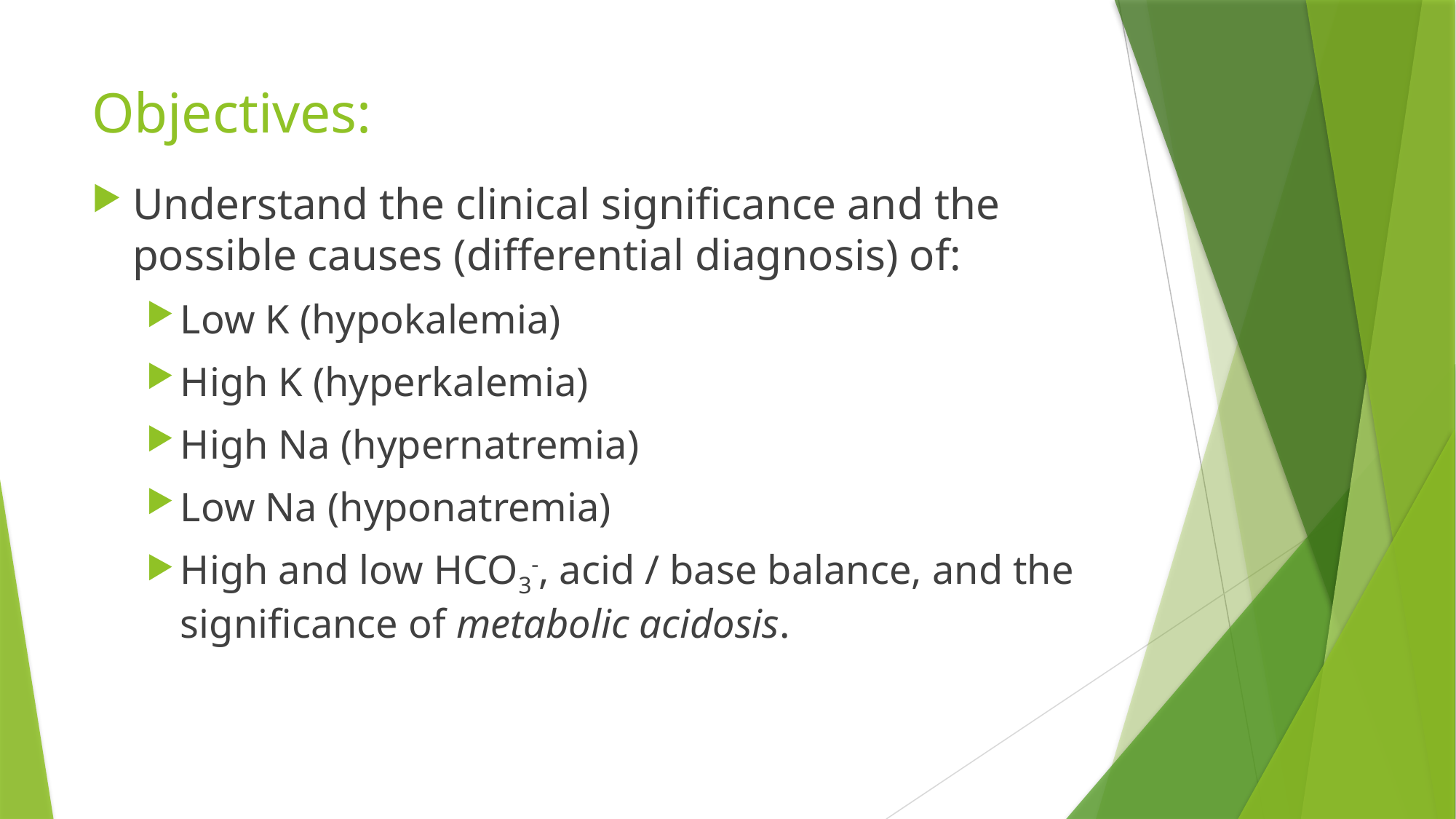

# Objectives:
Understand the clinical significance and the possible causes (differential diagnosis) of:
Low K (hypokalemia)
High K (hyperkalemia)
High Na (hypernatremia)
Low Na (hyponatremia)
High and low HCO3-, acid / base balance, and the significance of metabolic acidosis.

## Slide 4
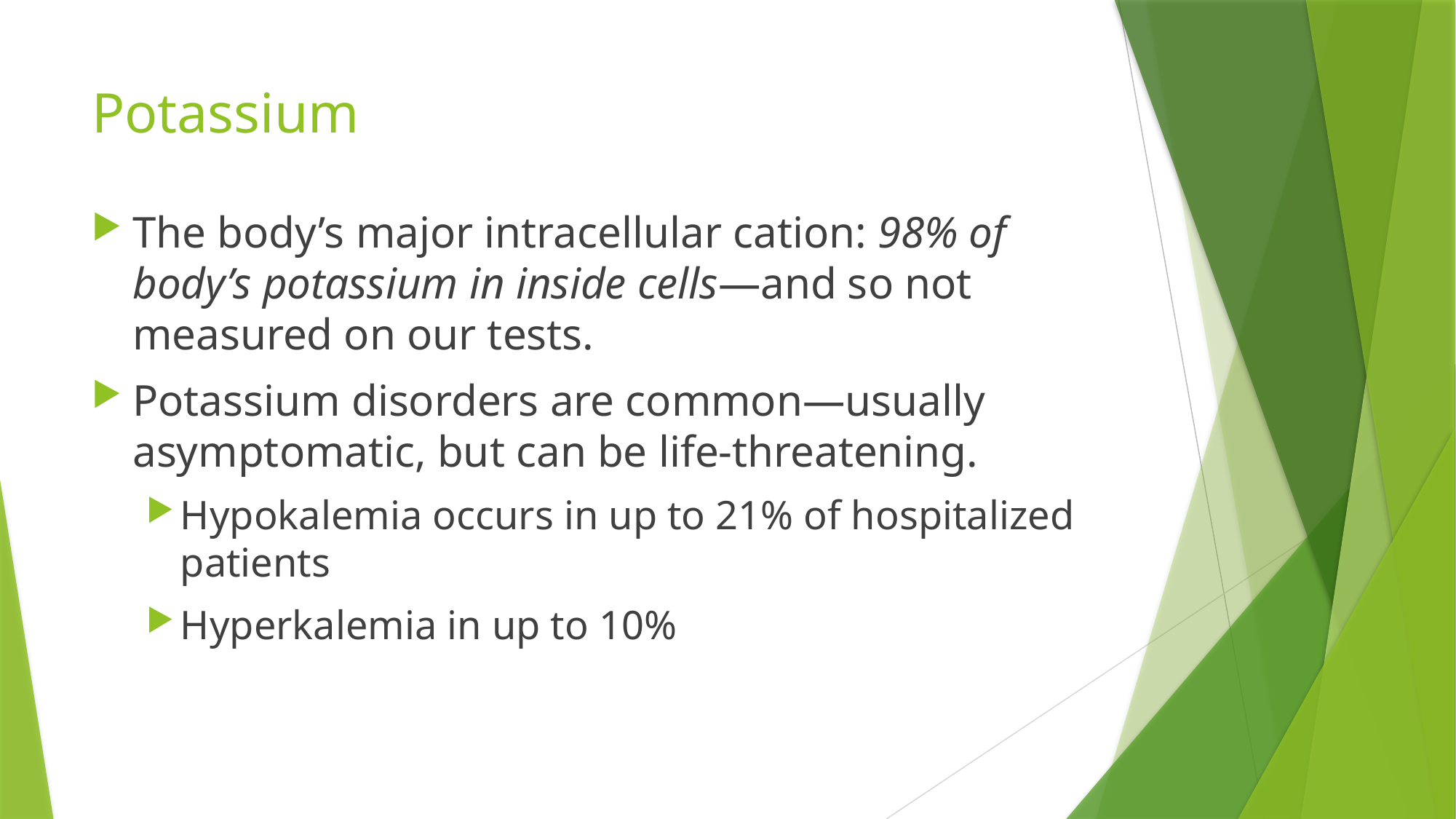

# Potassium
The body’s major intracellular cation: 98% of body’s potassium in inside cells—and so not measured on our tests.
Potassium disorders are common—usually asymptomatic, but can be life-threatening.
Hypokalemia occurs in up to 21% of hospitalized patients
Hyperkalemia in up to 10%

## Slide 5
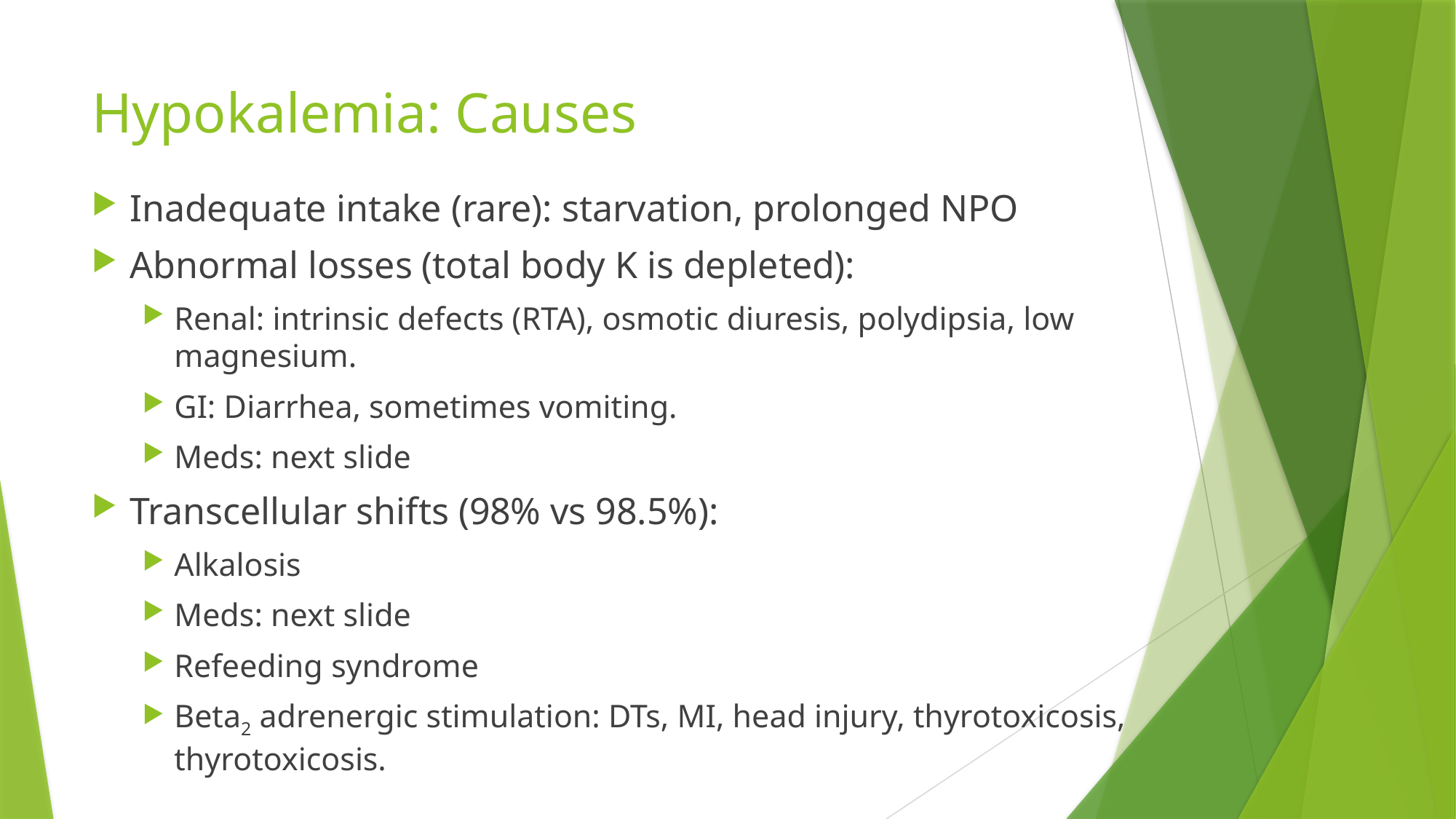

# Hypokalemia: Causes
Inadequate intake (rare): starvation, prolonged NPO
Abnormal losses (total body K is depleted):
Renal: intrinsic defects (RTA), osmotic diuresis, polydipsia, low magnesium.
GI: Diarrhea, sometimes vomiting.
Meds: next slide
Transcellular shifts (98% vs 98.5%):
Alkalosis
Meds: next slide
Refeeding syndrome
Beta2 adrenergic stimulation: DTs, MI, head injury, thyrotoxicosis, thyrotoxicosis.

## Slide 6
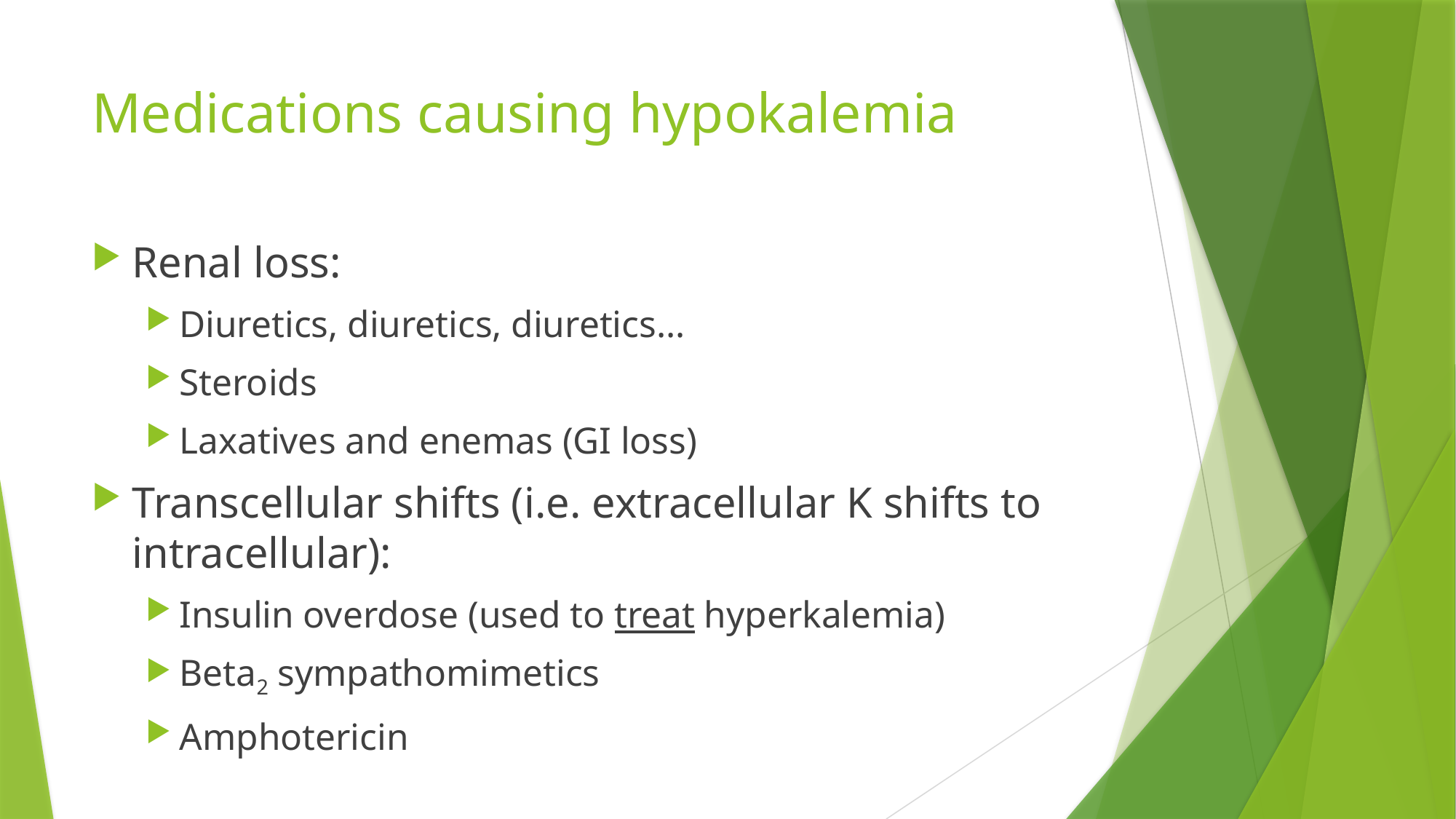

# Medications causing hypokalemia
Renal loss:
Diuretics, diuretics, diuretics…
Steroids
Laxatives and enemas (GI loss)
Transcellular shifts (i.e. extracellular K shifts to intracellular):
Insulin overdose (used to treat hyperkalemia)
Beta2 sympathomimetics
Amphotericin

## Slide 7
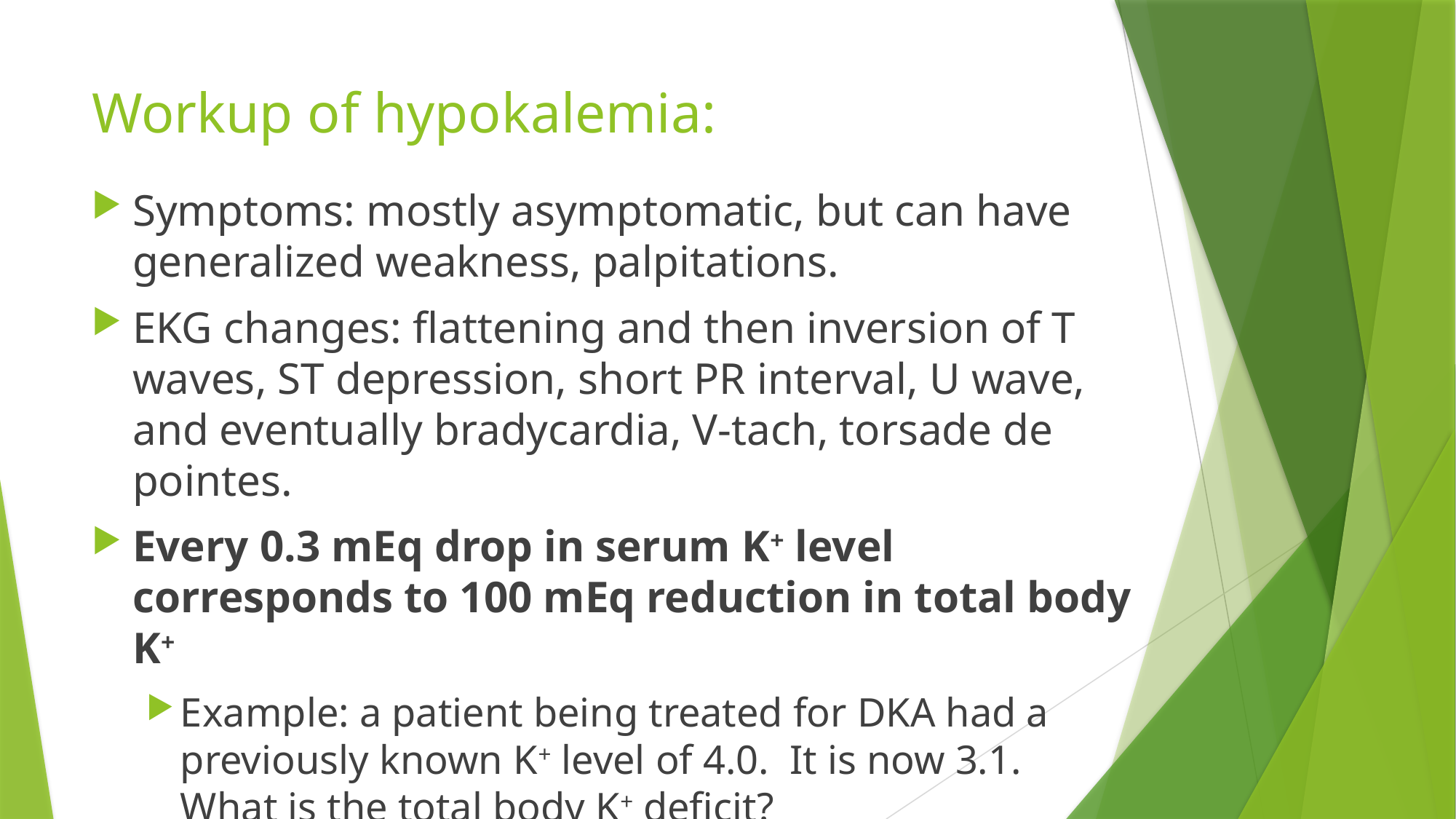

# Workup of hypokalemia:
Symptoms: mostly asymptomatic, but can have generalized weakness, palpitations.
EKG changes: flattening and then inversion of T waves, ST depression, short PR interval, U wave, and eventually bradycardia, V-tach, torsade de pointes.
Every 0.3 mEq drop in serum K+ level corresponds to 100 mEq reduction in total body K+
Example: a patient being treated for DKA had a previously known K+ level of 4.0. It is now 3.1. What is the total body K+ deficit?

## Slide 8
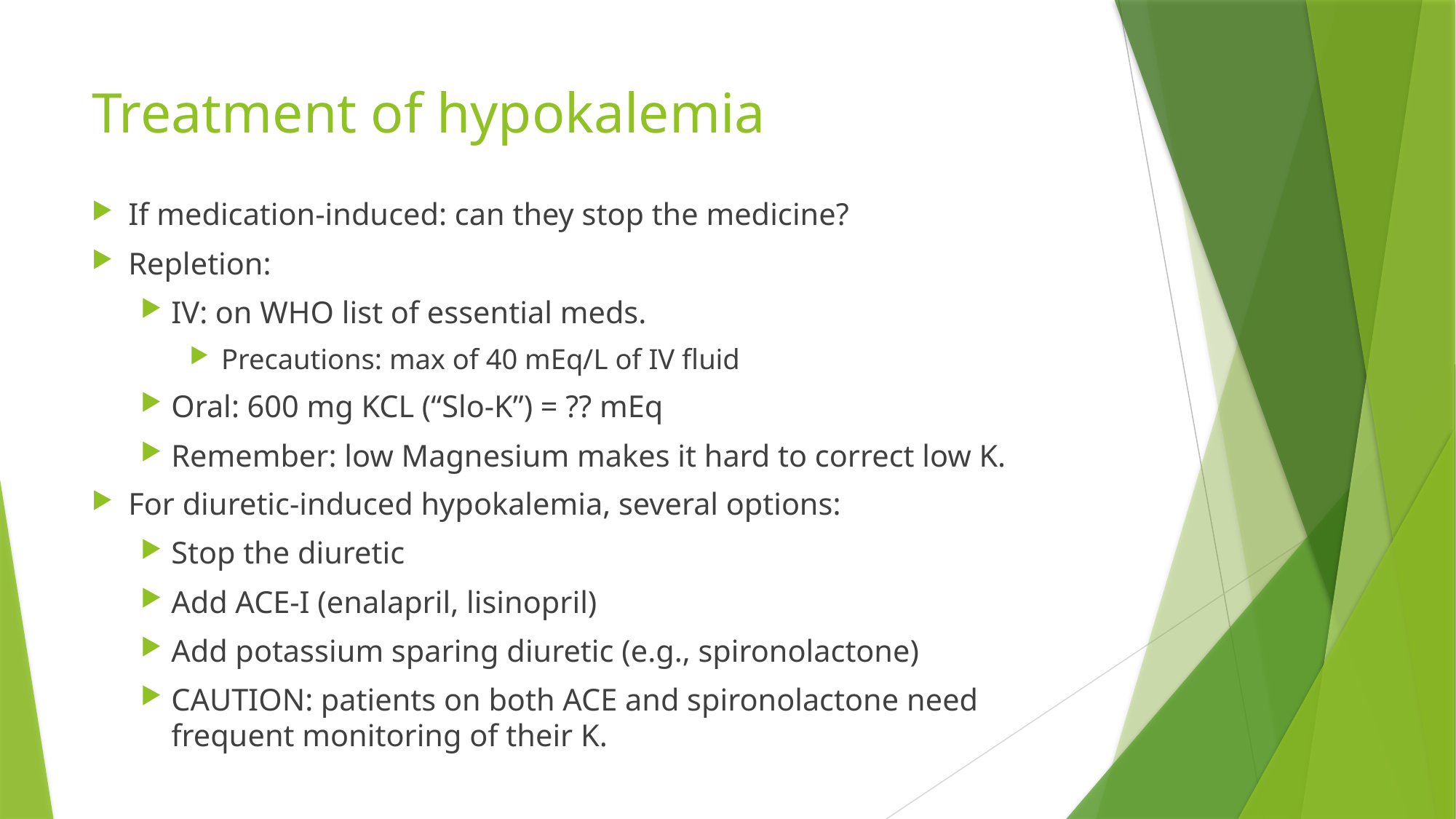

# Treatment of hypokalemia
If medication-induced: can they stop the medicine?
Repletion:
IV: on WHO list of essential meds.
 Precautions: max of 40 mEq/L of IV fluid
Oral: 600 mg KCL (“Slo-K”) = ?? mEq
Remember: low Magnesium makes it hard to correct low K.
For diuretic-induced hypokalemia, several options:
Stop the diuretic
Add ACE-I (enalapril, lisinopril)
Add potassium sparing diuretic (e.g., spironolactone)
CAUTION: patients on both ACE and spironolactone need frequent monitoring of their K.

## Slide 9
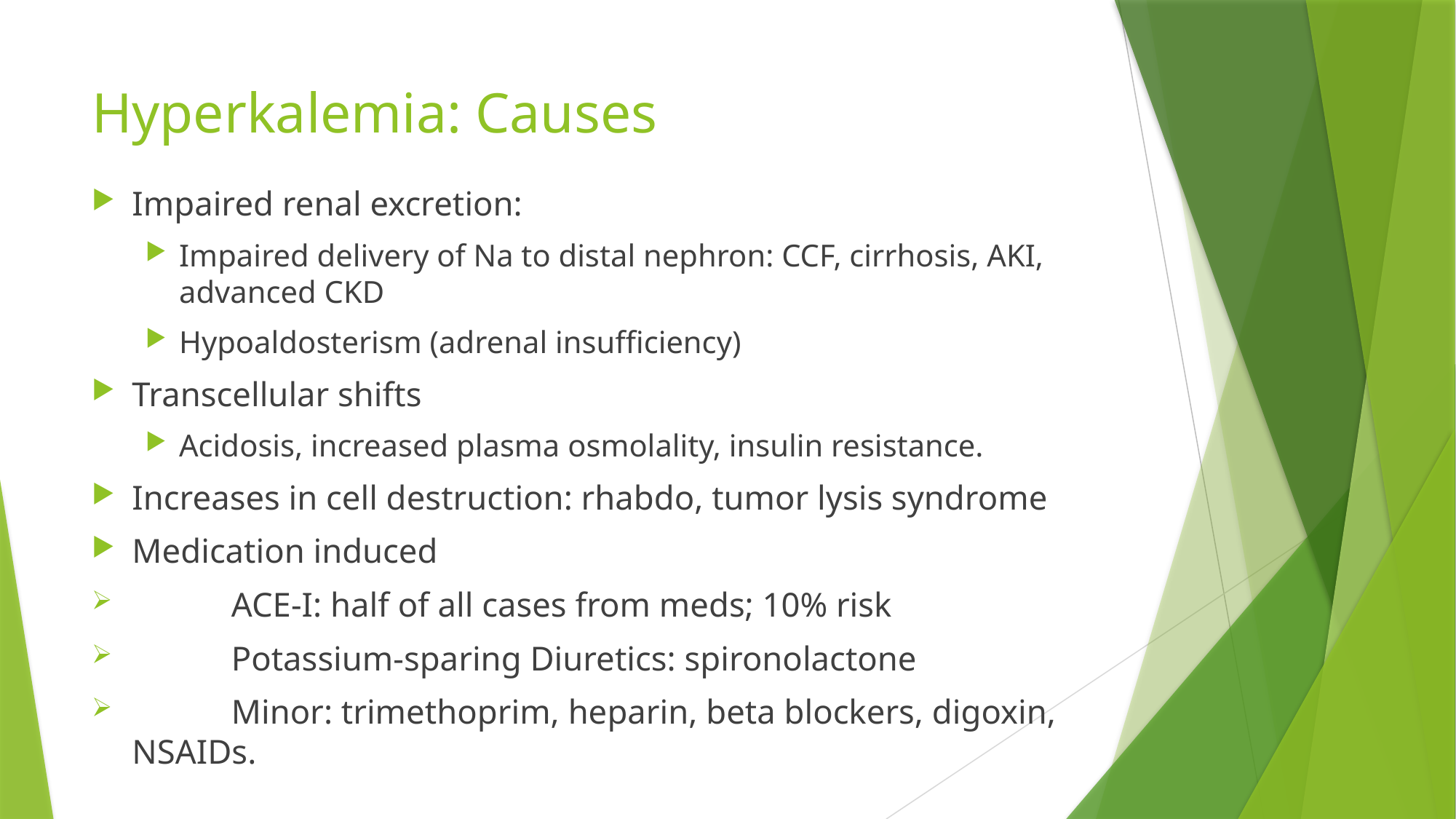

# Hyperkalemia: Causes
Impaired renal excretion:
Impaired delivery of Na to distal nephron: CCF, cirrhosis, AKI, advanced CKD
Hypoaldosterism (adrenal insufficiency)
Transcellular shifts
Acidosis, increased plasma osmolality, insulin resistance.
Increases in cell destruction: rhabdo, tumor lysis syndrome
Medication induced
	ACE-I: half of all cases from meds; 10% risk
	Potassium-sparing Diuretics: spironolactone
	Minor: trimethoprim, heparin, beta blockers, digoxin, NSAIDs.

## Slide 10
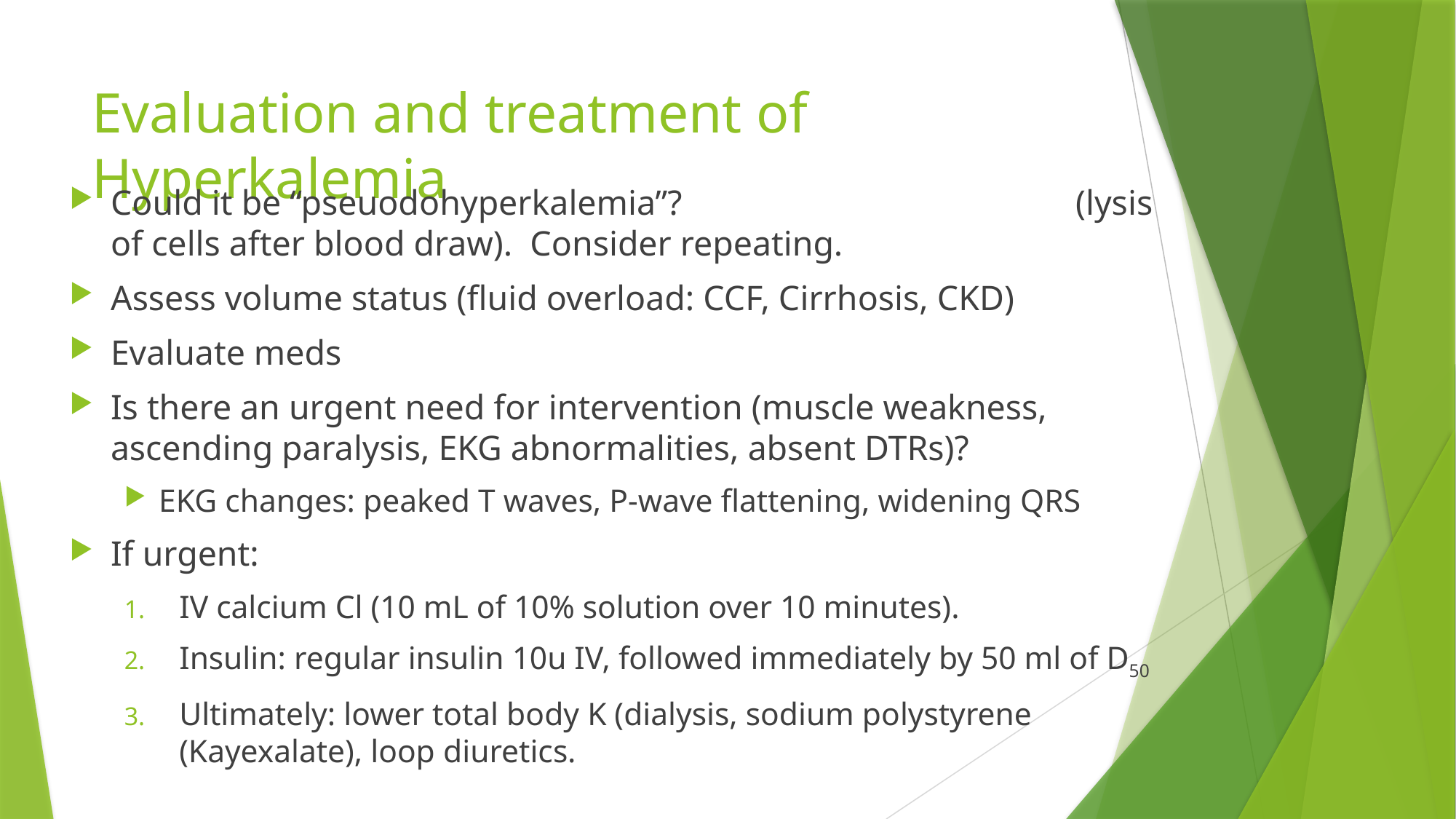

# Evaluation and treatment of Hyperkalemia
Could it be “pseuodohyperkalemia”? (lysis of cells after blood draw). Consider repeating.
Assess volume status (fluid overload: CCF, Cirrhosis, CKD)
Evaluate meds
Is there an urgent need for intervention (muscle weakness, ascending paralysis, EKG abnormalities, absent DTRs)?
EKG changes: peaked T waves, P-wave flattening, widening QRS
If urgent:
IV calcium Cl (10 mL of 10% solution over 10 minutes).
Insulin: regular insulin 10u IV, followed immediately by 50 ml of D50
Ultimately: lower total body K (dialysis, sodium polystyrene (Kayexalate), loop diuretics.

## Slide 11
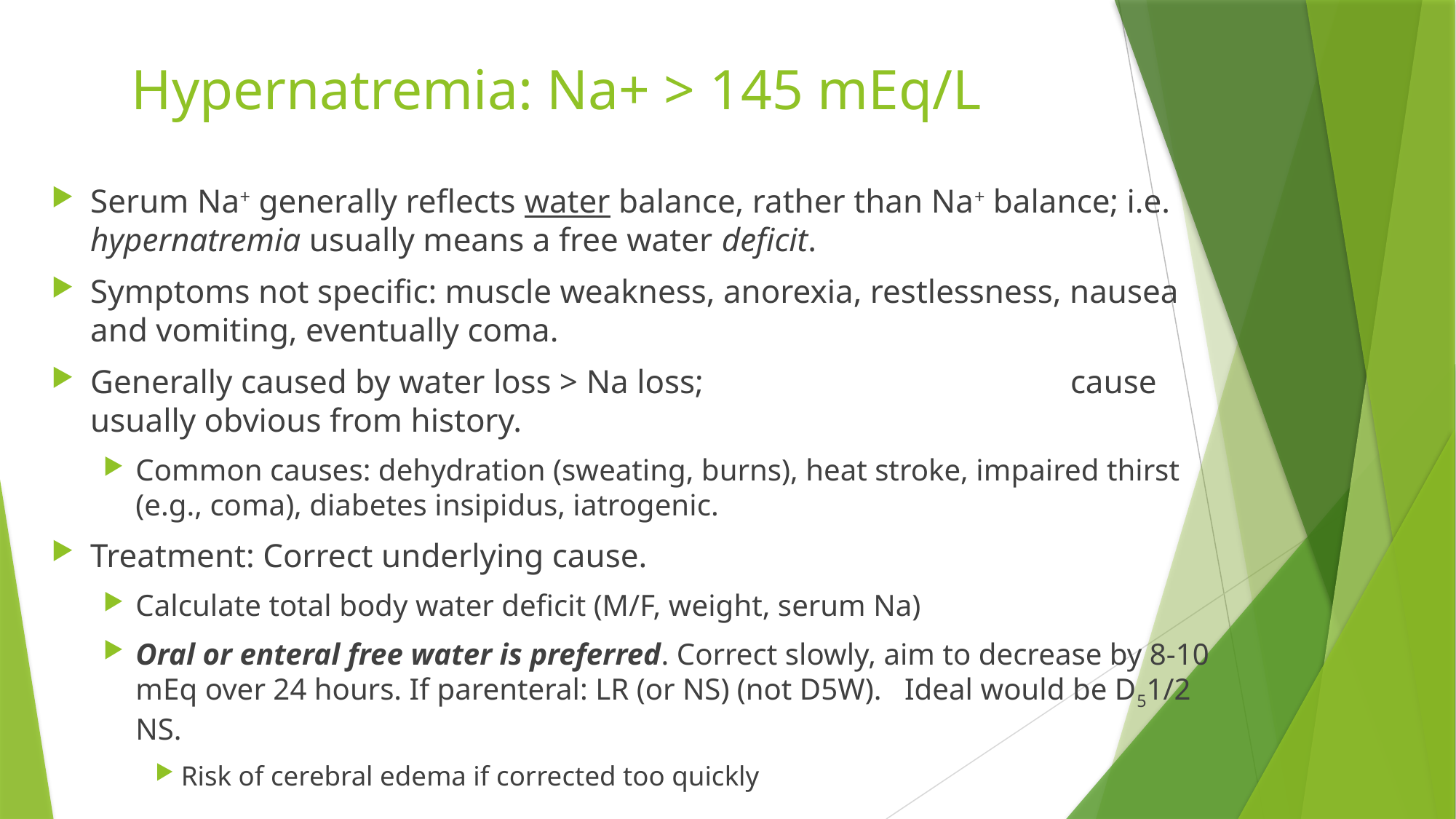

# Hypernatremia: Na+ > 145 mEq/L
Serum Na+ generally reflects water balance, rather than Na+ balance; i.e. hypernatremia usually means a free water deficit.
Symptoms not specific: muscle weakness, anorexia, restlessness, nausea and vomiting, eventually coma.
Generally caused by water loss > Na loss; cause usually obvious from history.
Common causes: dehydration (sweating, burns), heat stroke, impaired thirst (e.g., coma), diabetes insipidus, iatrogenic.
Treatment: Correct underlying cause.
Calculate total body water deficit (M/F, weight, serum Na)
Oral or enteral free water is preferred. Correct slowly, aim to decrease by 8-10 mEq over 24 hours. If parenteral: LR (or NS) (not D5W). Ideal would be D51/2 NS.
Risk of cerebral edema if corrected too quickly

## Slide 12
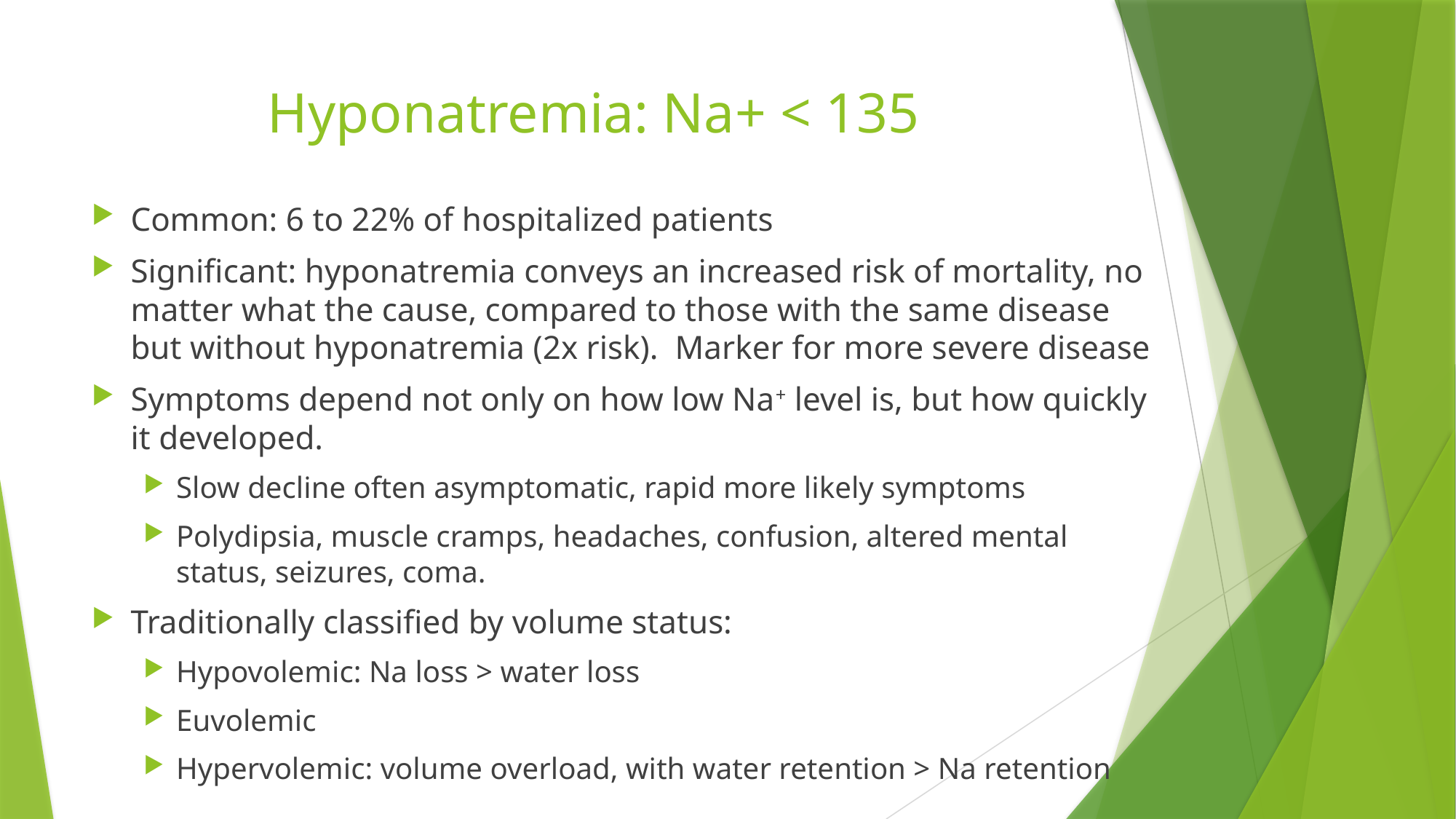

# Hyponatremia: Na+ < 135
Common: 6 to 22% of hospitalized patients
Significant: hyponatremia conveys an increased risk of mortality, no matter what the cause, compared to those with the same disease but without hyponatremia (2x risk). Marker for more severe disease
Symptoms depend not only on how low Na+ level is, but how quickly it developed.
Slow decline often asymptomatic, rapid more likely symptoms
Polydipsia, muscle cramps, headaches, confusion, altered mental status, seizures, coma.
Traditionally classified by volume status:
Hypovolemic: Na loss > water loss
Euvolemic
Hypervolemic: volume overload, with water retention > Na retention

## Slide 13
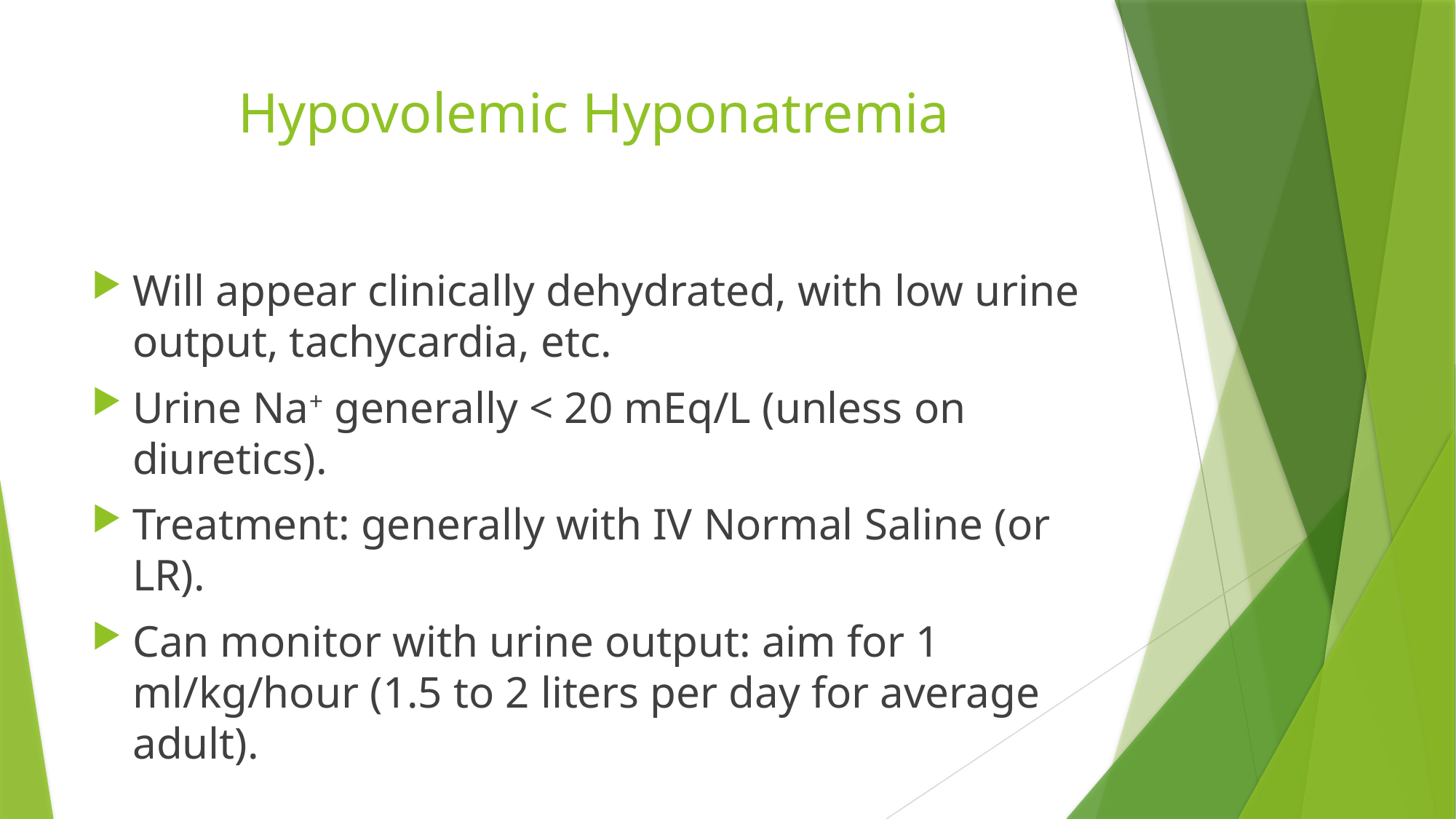

# Hypovolemic Hyponatremia
Will appear clinically dehydrated, with low urine output, tachycardia, etc.
Urine Na+ generally < 20 mEq/L (unless on diuretics).
Treatment: generally with IV Normal Saline (or LR).
Can monitor with urine output: aim for 1 ml/kg/hour (1.5 to 2 liters per day for average adult).

## Slide 14
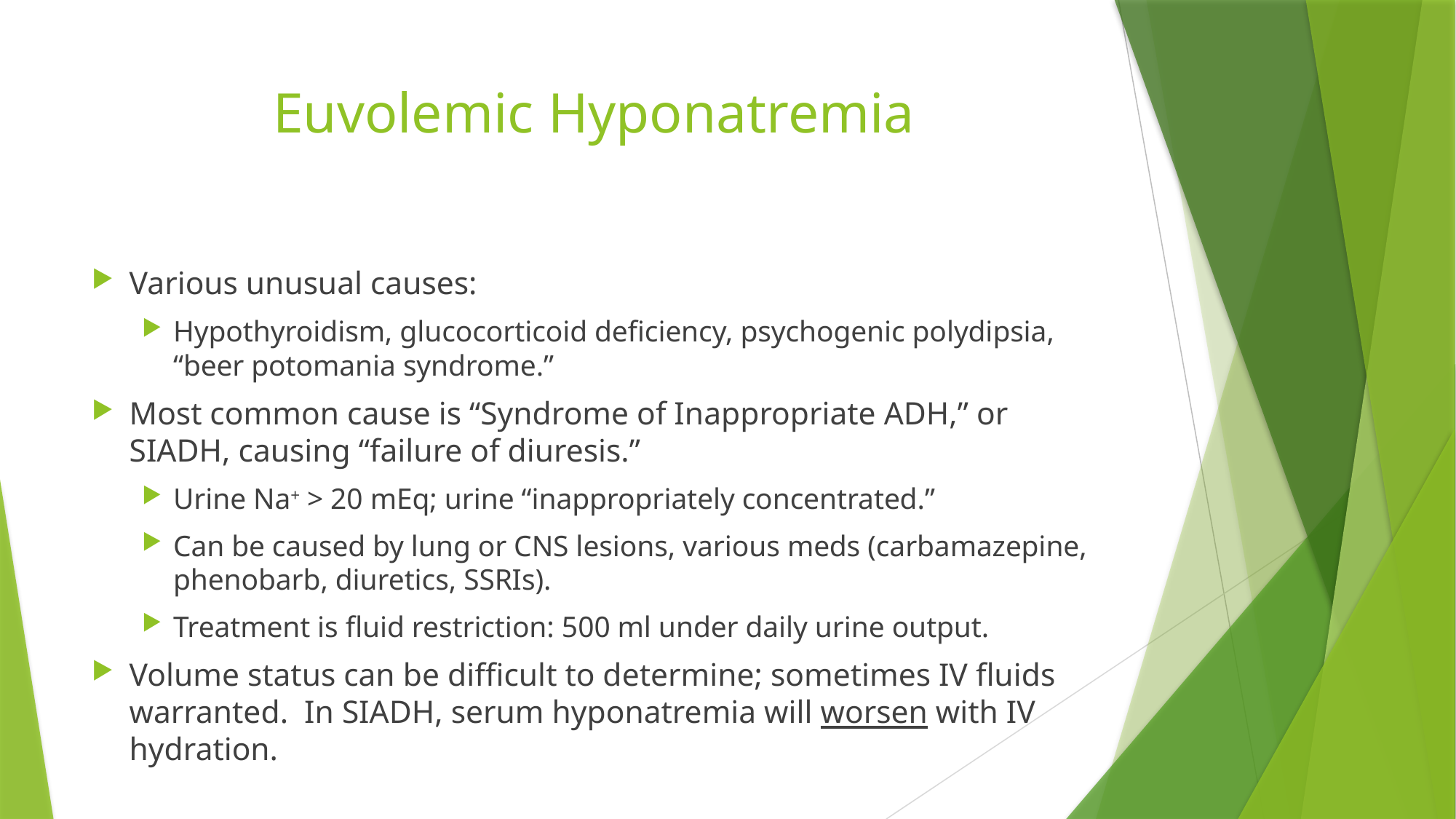

# Euvolemic Hyponatremia
Various unusual causes:
Hypothyroidism, glucocorticoid deficiency, psychogenic polydipsia, “beer potomania syndrome.”
Most common cause is “Syndrome of Inappropriate ADH,” or SIADH, causing “failure of diuresis.”
Urine Na+ > 20 mEq; urine “inappropriately concentrated.”
Can be caused by lung or CNS lesions, various meds (carbamazepine, phenobarb, diuretics, SSRIs).
Treatment is fluid restriction: 500 ml under daily urine output.
Volume status can be difficult to determine; sometimes IV fluids warranted. In SIADH, serum hyponatremia will worsen with IV hydration.

## Slide 15
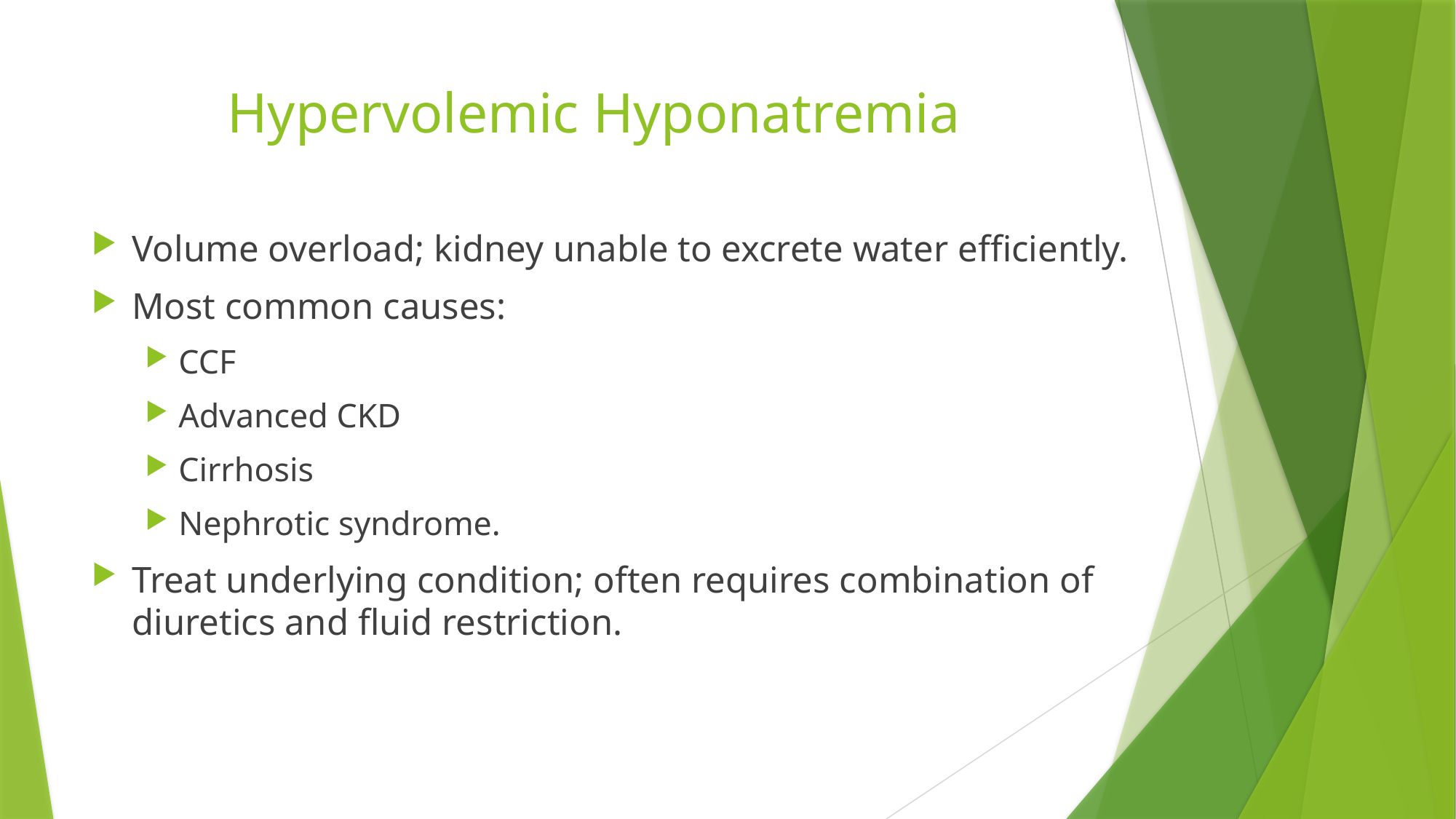

# Hypervolemic Hyponatremia
Volume overload; kidney unable to excrete water efficiently.
Most common causes:
CCF
Advanced CKD
Cirrhosis
Nephrotic syndrome.
Treat underlying condition; often requires combination of diuretics and fluid restriction.

## Slide 16
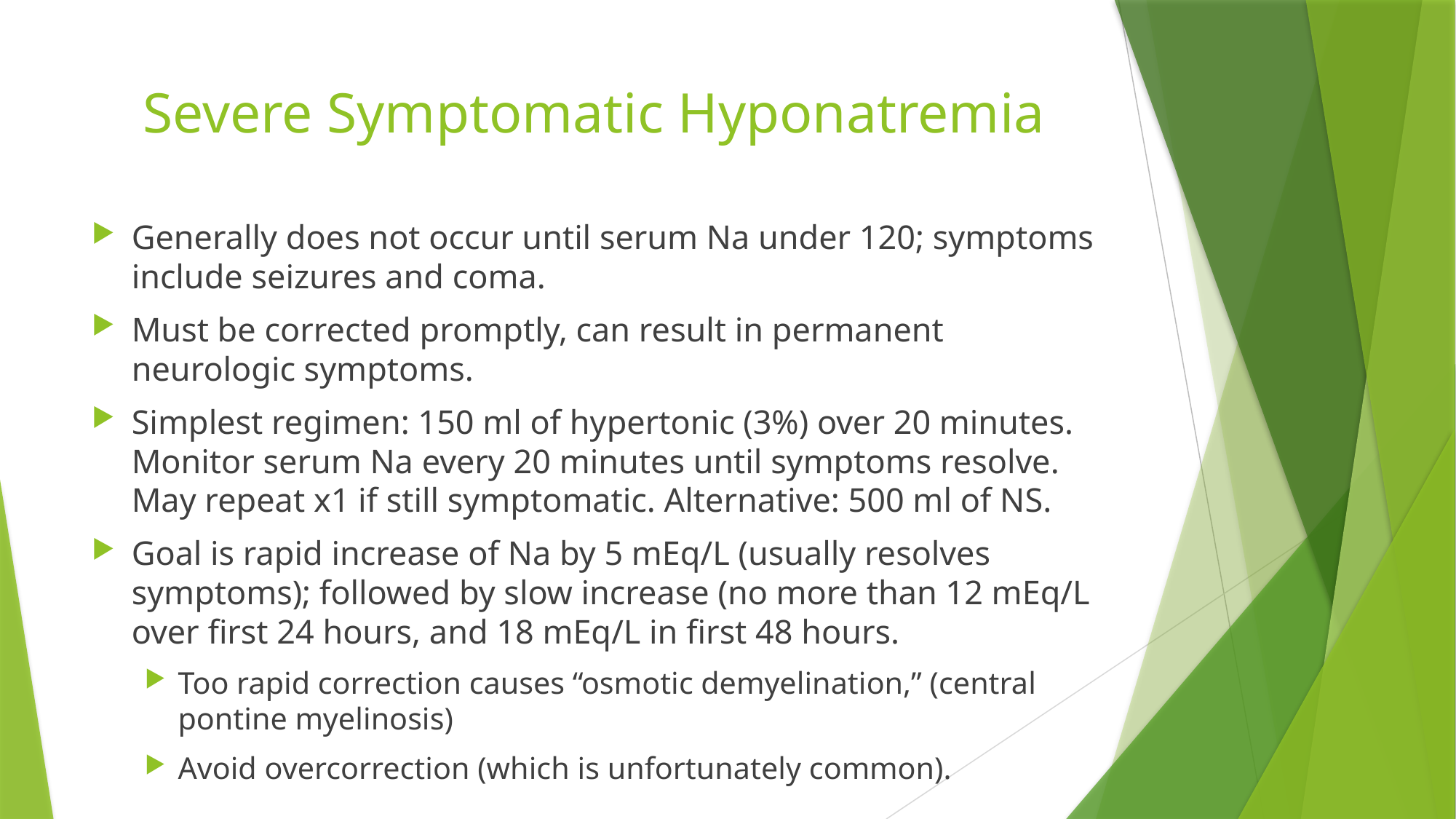

# Severe Symptomatic Hyponatremia
Generally does not occur until serum Na under 120; symptoms include seizures and coma.
Must be corrected promptly, can result in permanent neurologic symptoms.
Simplest regimen: 150 ml of hypertonic (3%) over 20 minutes. Monitor serum Na every 20 minutes until symptoms resolve. May repeat x1 if still symptomatic. Alternative: 500 ml of NS.
Goal is rapid increase of Na by 5 mEq/L (usually resolves symptoms); followed by slow increase (no more than 12 mEq/L over first 24 hours, and 18 mEq/L in first 48 hours.
Too rapid correction causes “osmotic demyelination,” (central pontine myelinosis)
Avoid overcorrection (which is unfortunately common).

## Slide 17
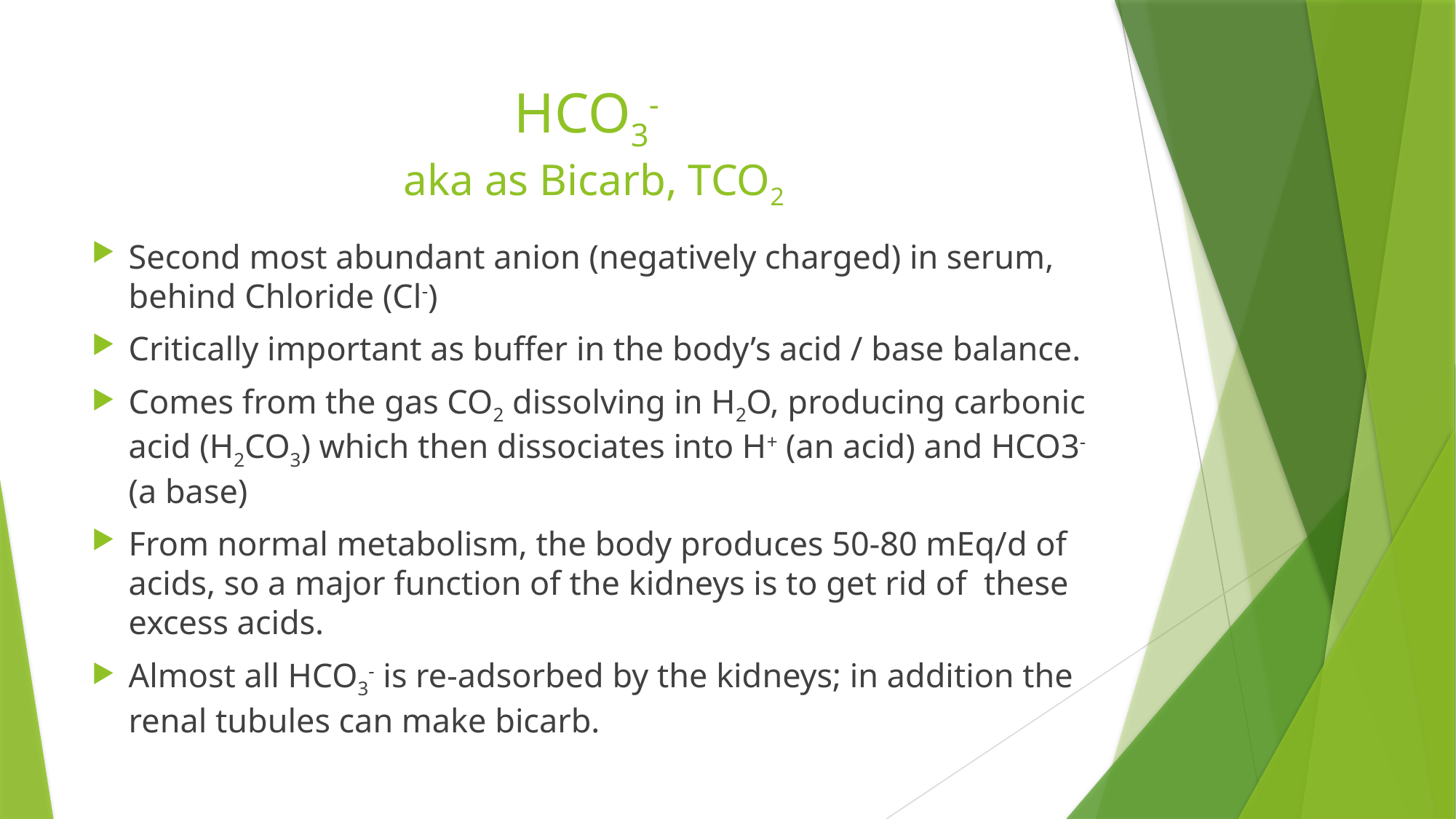

# HCO3- aka as Bicarb, TCO2
Second most abundant anion (negatively charged) in serum, behind Chloride (Cl-)
Critically important as buffer in the body’s acid / base balance.
Comes from the gas CO2 dissolving in H2O, producing carbonic acid (H2CO3) which then dissociates into H+ (an acid) and HCO3- (a base)
From normal metabolism, the body produces 50-80 mEq/d of acids, so a major function of the kidneys is to get rid of these excess acids.
Almost all HCO3- is re-adsorbed by the kidneys; in addition the renal tubules can make bicarb.

## Slide 18
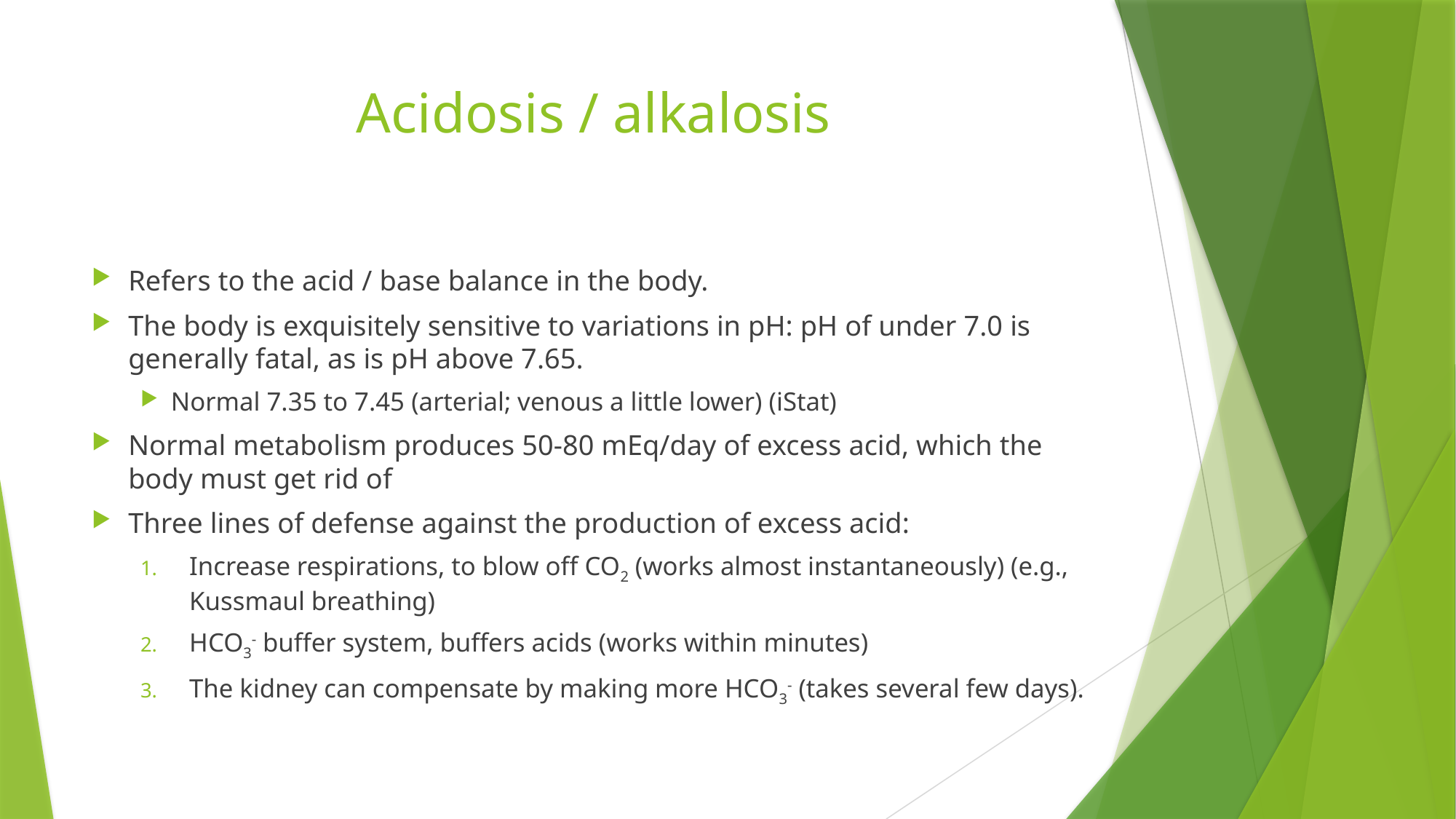

# Acidosis / alkalosis
Refers to the acid / base balance in the body.
The body is exquisitely sensitive to variations in pH: pH of under 7.0 is generally fatal, as is pH above 7.65.
Normal 7.35 to 7.45 (arterial; venous a little lower) (iStat)
Normal metabolism produces 50-80 mEq/day of excess acid, which the body must get rid of
Three lines of defense against the production of excess acid:
Increase respirations, to blow off CO2 (works almost instantaneously) (e.g., Kussmaul breathing)
HCO3- buffer system, buffers acids (works within minutes)
The kidney can compensate by making more HCO3- (takes several few days).

## Slide 19
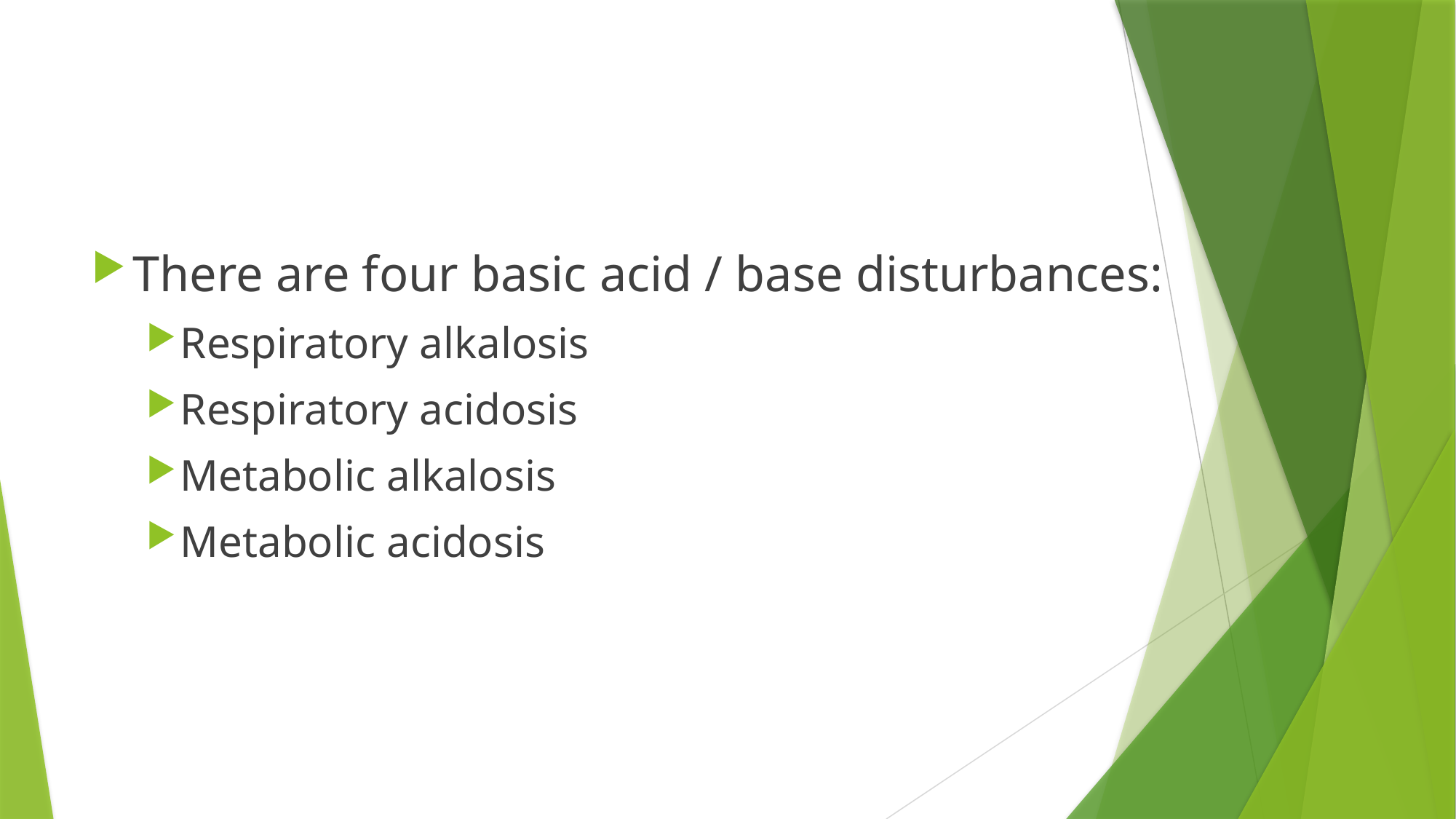

#
There are four basic acid / base disturbances:
Respiratory alkalosis
Respiratory acidosis
Metabolic alkalosis
Metabolic acidosis

## Slide 20
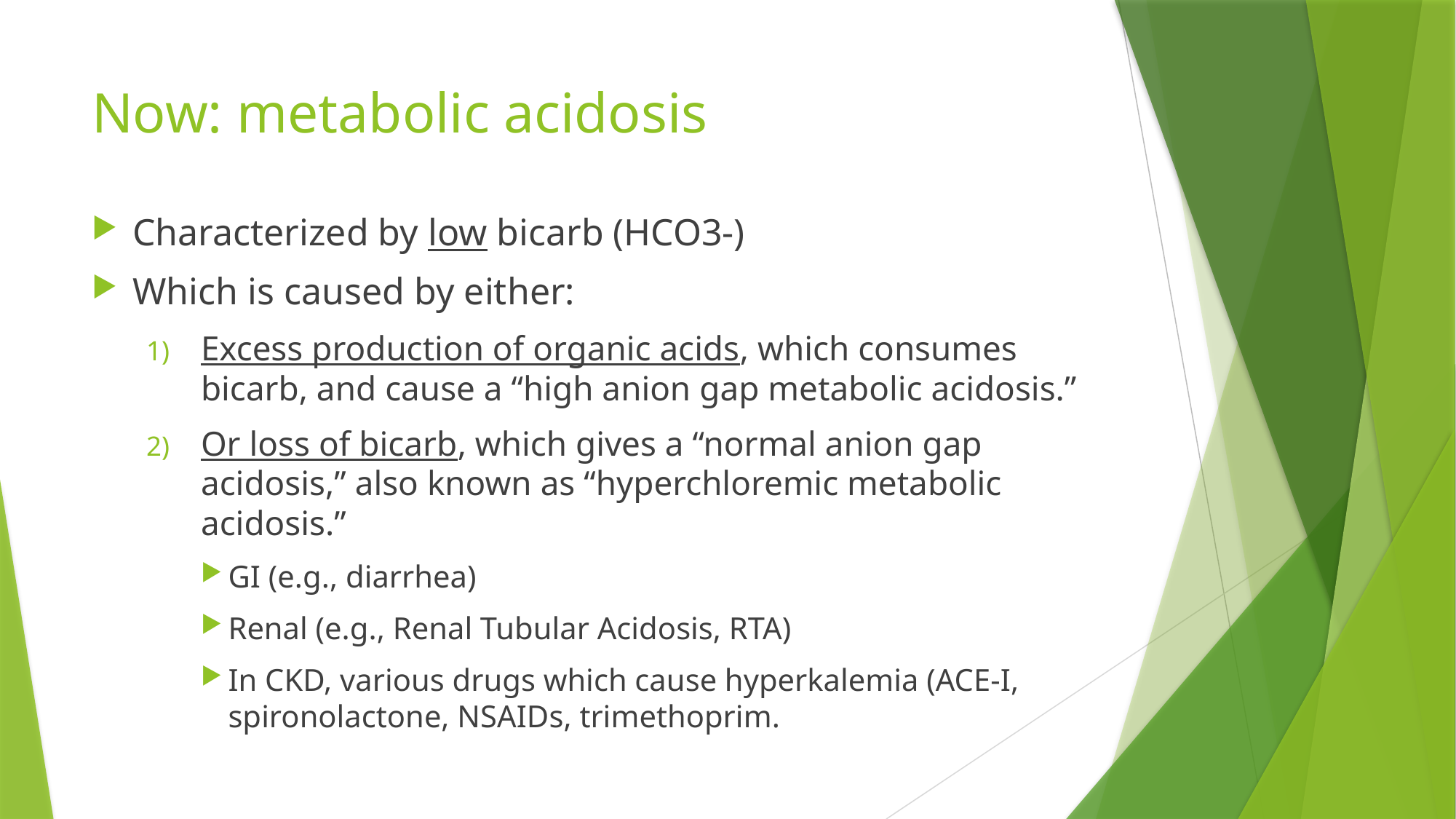

# Now: metabolic acidosis
Characterized by low bicarb (HCO3-)
Which is caused by either:
Excess production of organic acids, which consumes bicarb, and cause a “high anion gap metabolic acidosis.”
Or loss of bicarb, which gives a “normal anion gap acidosis,” also known as “hyperchloremic metabolic acidosis.”
GI (e.g., diarrhea)
Renal (e.g., Renal Tubular Acidosis, RTA)
In CKD, various drugs which cause hyperkalemia (ACE-I, spironolactone, NSAIDs, trimethoprim.

## Slide 21
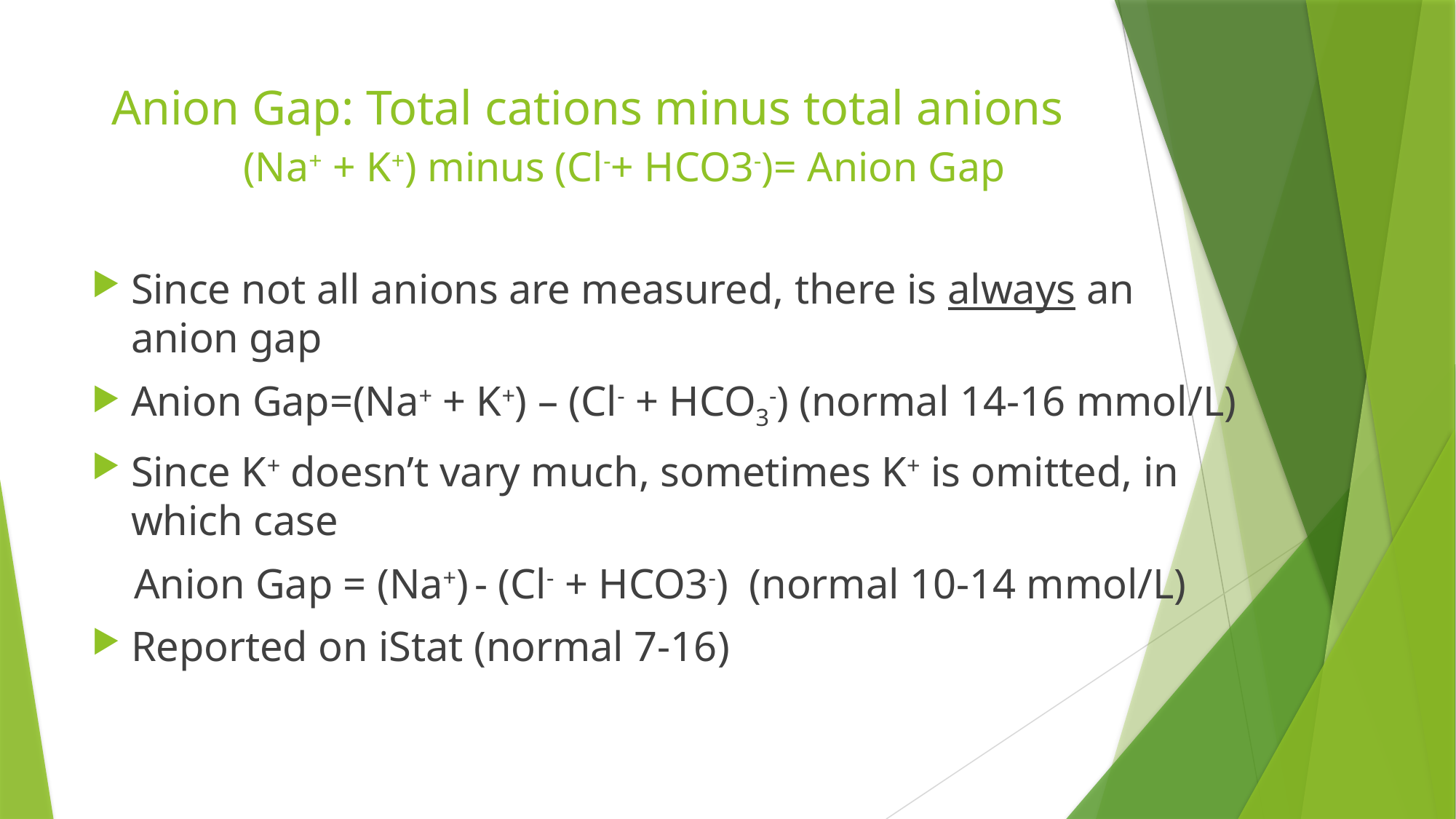

# Anion Gap: Total cations minus total anions (Na+ + K+) minus (Cl-+ HCO3-)= Anion Gap
Since not all anions are measured, there is always an anion gap
Anion Gap=(Na+ + K+) – (Cl- + HCO3-) (normal 14-16 mmol/L)
Since K+ doesn’t vary much, sometimes K+ is omitted, in which case
 Anion Gap = (Na+) - (Cl- + HCO3-) (normal 10-14 mmol/L)
Reported on iStat (normal 7-16)

## Slide 22
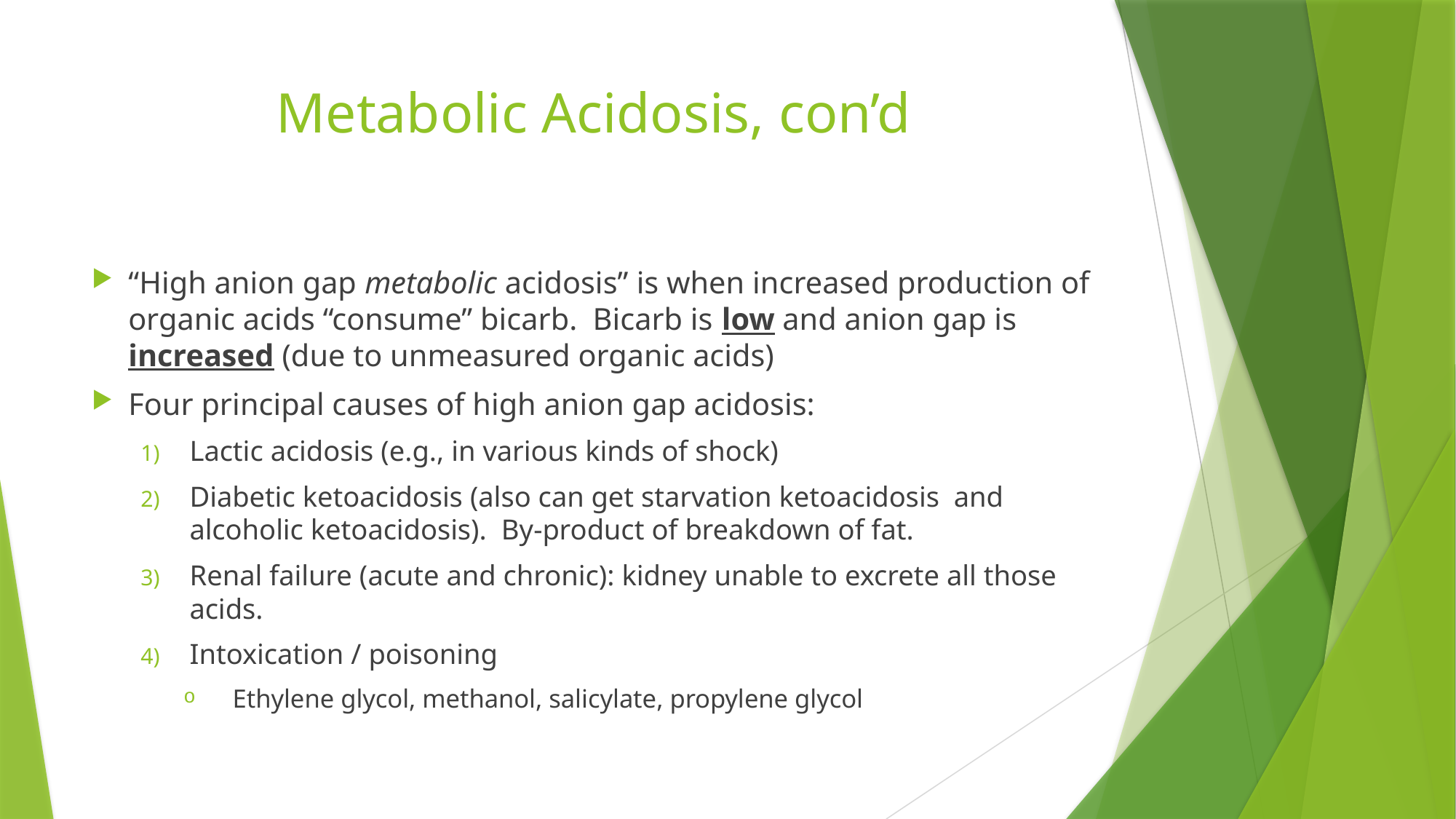

# Metabolic Acidosis, con’d
“High anion gap metabolic acidosis” is when increased production of organic acids “consume” bicarb. Bicarb is low and anion gap is increased (due to unmeasured organic acids)
Four principal causes of high anion gap acidosis:
Lactic acidosis (e.g., in various kinds of shock)
Diabetic ketoacidosis (also can get starvation ketoacidosis and alcoholic ketoacidosis). By-product of breakdown of fat.
Renal failure (acute and chronic): kidney unable to excrete all those acids.
Intoxication / poisoning
Ethylene glycol, methanol, salicylate, propylene glycol

## Slide 23
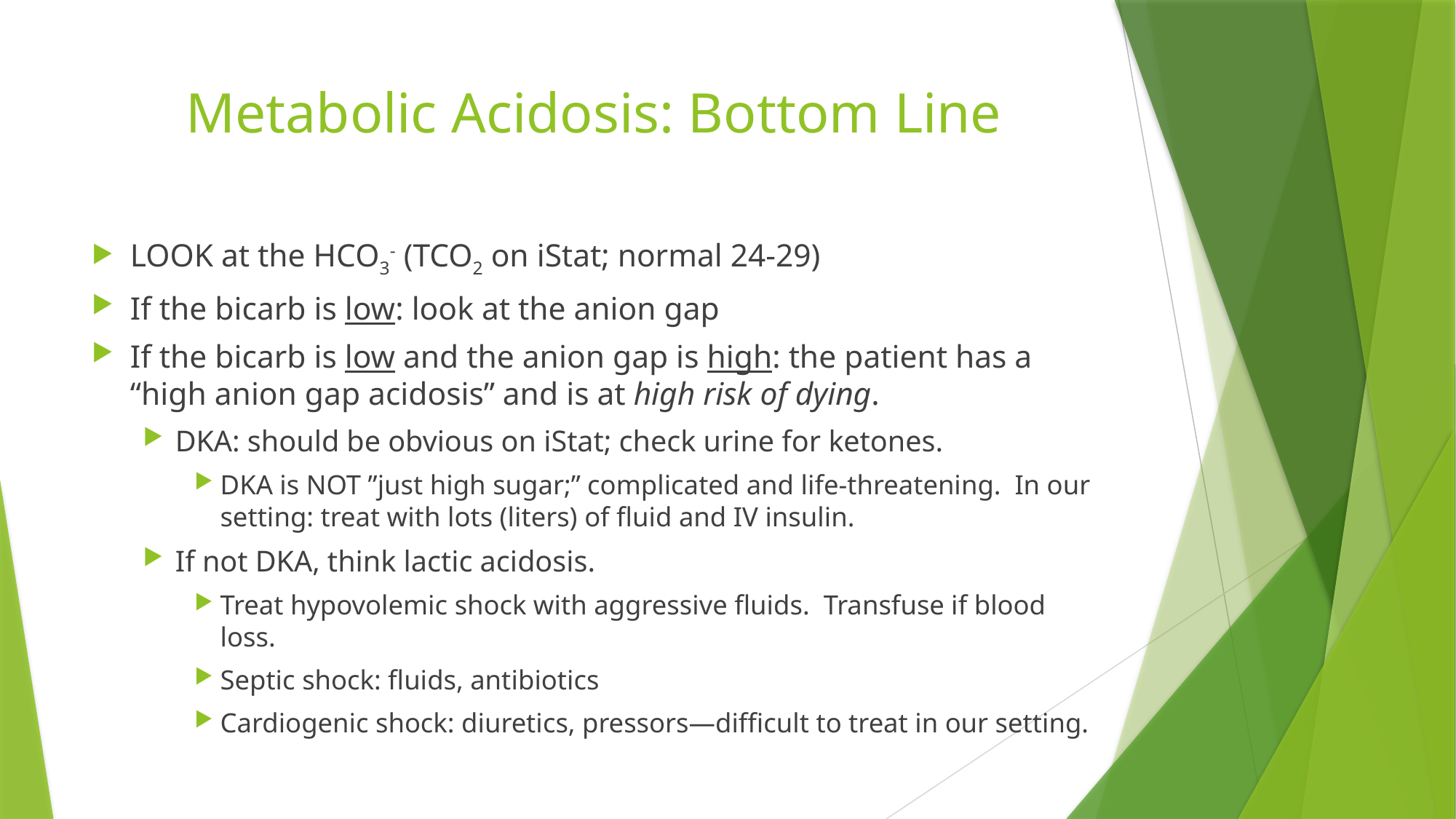

# Metabolic Acidosis: Bottom Line
LOOK at the HCO3- (TCO2 on iStat; normal 24-29)
If the bicarb is low: look at the anion gap
If the bicarb is low and the anion gap is high: the patient has a “high anion gap acidosis” and is at high risk of dying.
DKA: should be obvious on iStat; check urine for ketones.
DKA is NOT ”just high sugar;” complicated and life-threatening. In our setting: treat with lots (liters) of fluid and IV insulin.
If not DKA, think lactic acidosis.
Treat hypovolemic shock with aggressive fluids. Transfuse if blood loss.
Septic shock: fluids, antibiotics
Cardiogenic shock: diuretics, pressors—difficult to treat in our setting.

## Slide 24
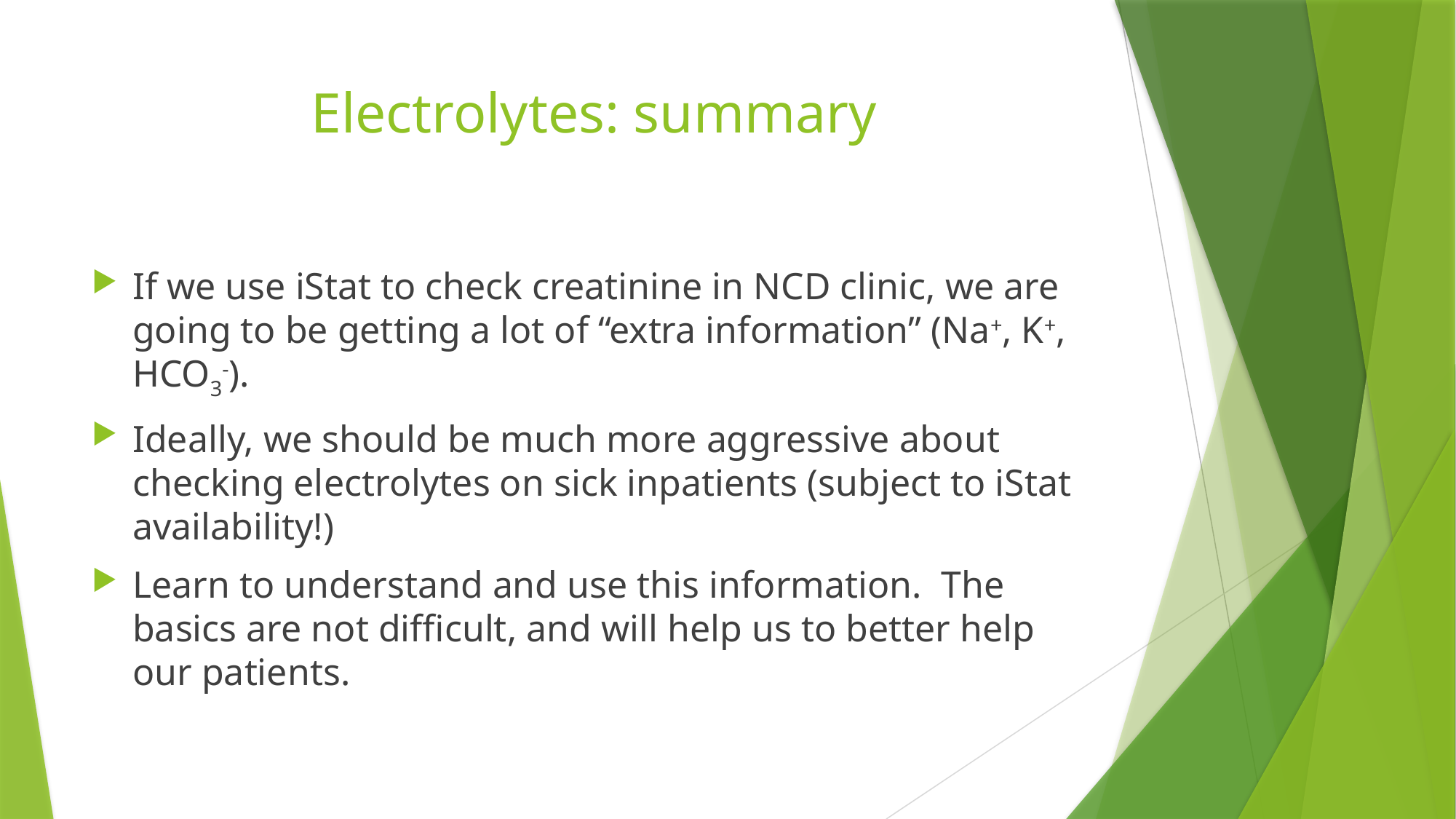

# Electrolytes: summary
If we use iStat to check creatinine in NCD clinic, we are going to be getting a lot of “extra information” (Na+, K+, HCO3-).
Ideally, we should be much more aggressive about checking electrolytes on sick inpatients (subject to iStat availability!)
Learn to understand and use this information. The basics are not difficult, and will help us to better help our patients.
