# Supplementary material for: Training Mid-Level Providers to Treat Severe Non-Communicable Diseases in Neno, Malawi through PEN-Plus Strategies
Source: Ann Glob Health. 2022 Aug 11;88(1):69. doi: 10.5334/aogh.3750 (PMC9389951; doi:10.5334/aogh.3750)
Supplement: Didactic Materials. — The supplementary materials contain a suggested didactic training schedule and the PowerPoint presentations used for PEN-Plus training in Neno, Malawi. These materials have been reviewed and accepted by the Malawi Ministry of Health for future PEN-Plus trainings in Malawi. [file agh-88-1-3750-s2.zip › Didactic_Materials/R_Epi and Path.pptx]

## Slide 1
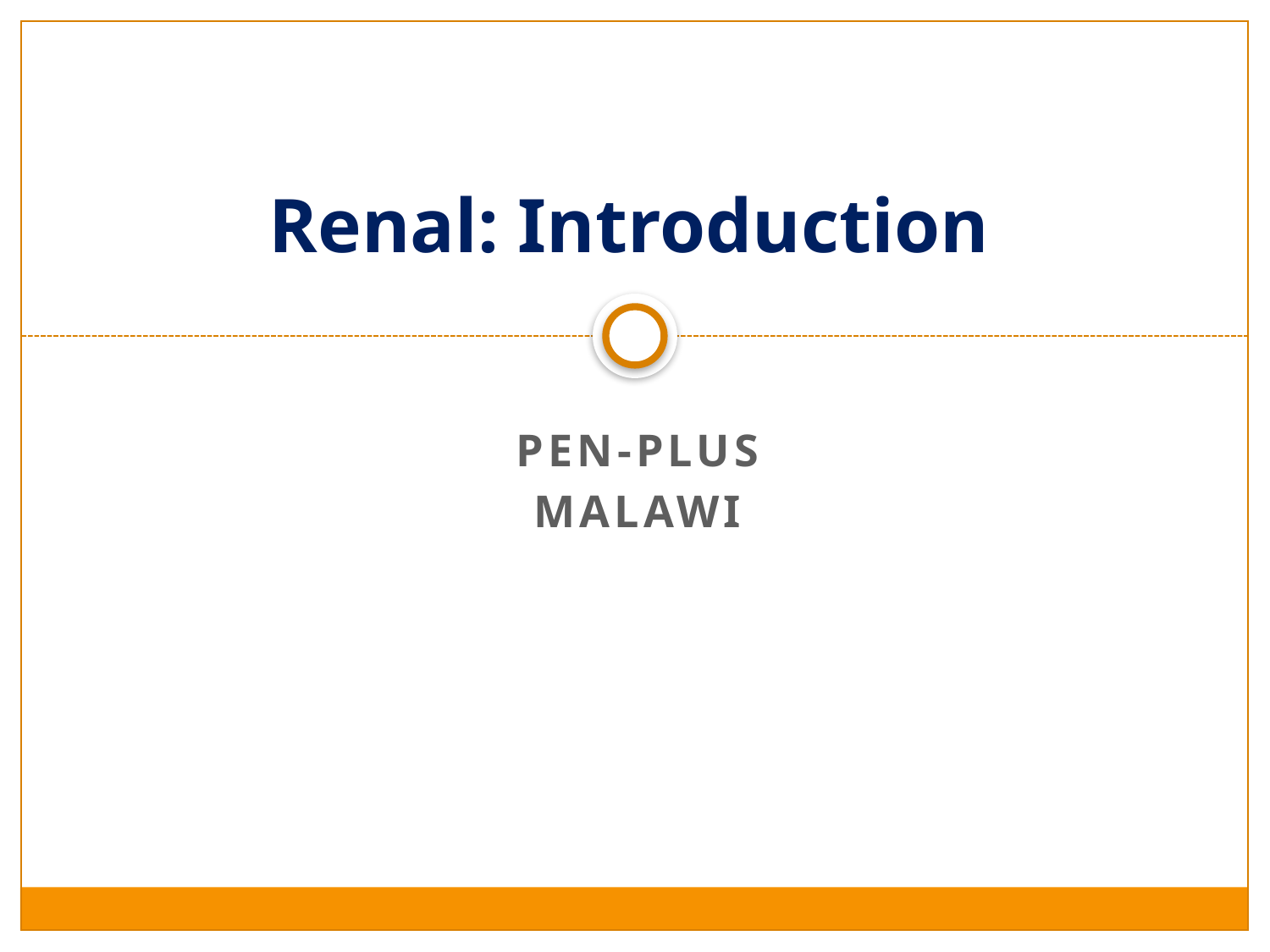

# Renal: Introduction
PEN-Plus
Malawi

## Slide 2
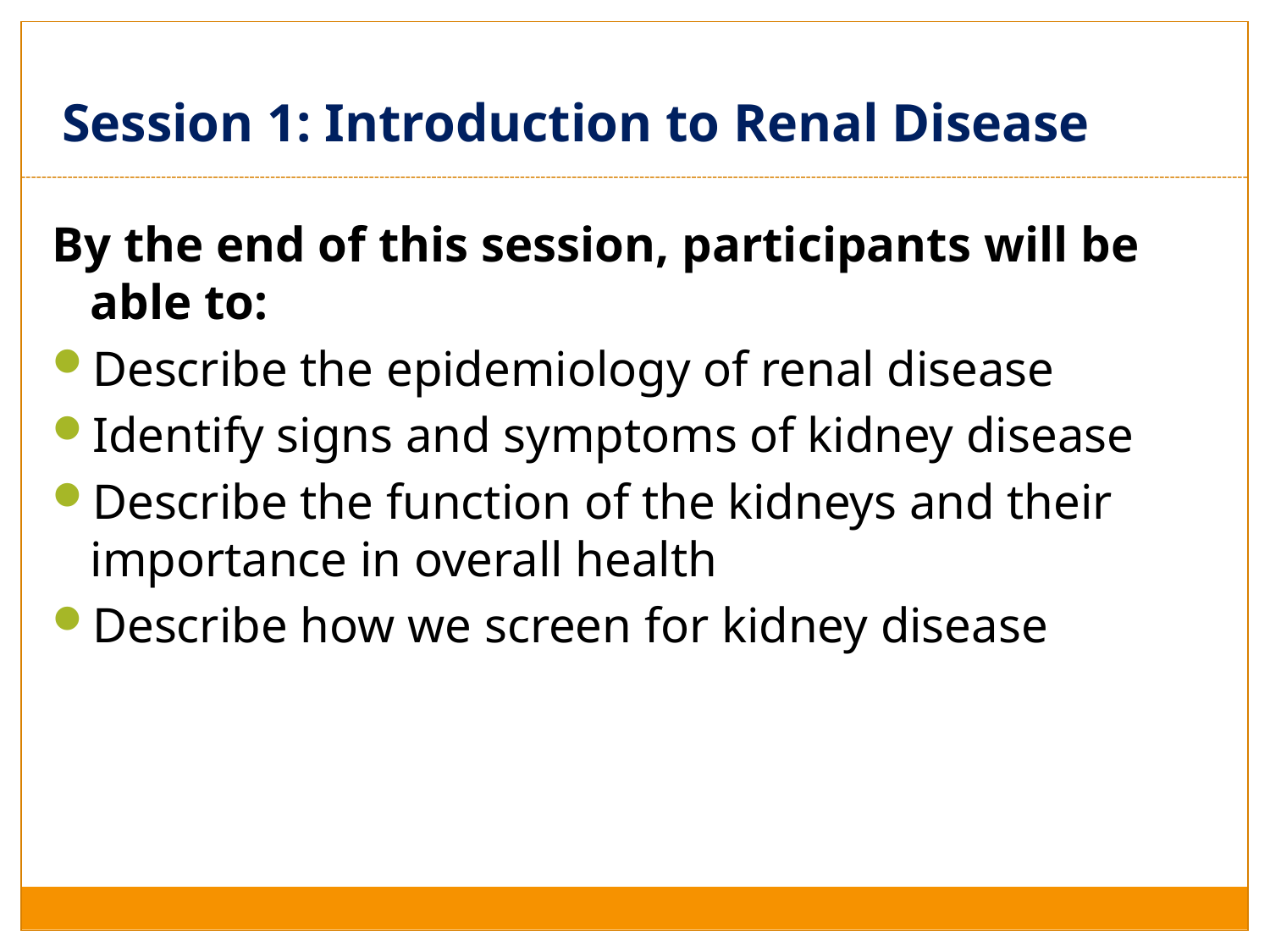

# Session 1: Introduction to Renal Disease
By the end of this session, participants will be able to:
Describe the epidemiology of renal disease
Identify signs and symptoms of kidney disease
Describe the function of the kidneys and their importance in overall health
Describe how we screen for kidney disease

## Slide 3
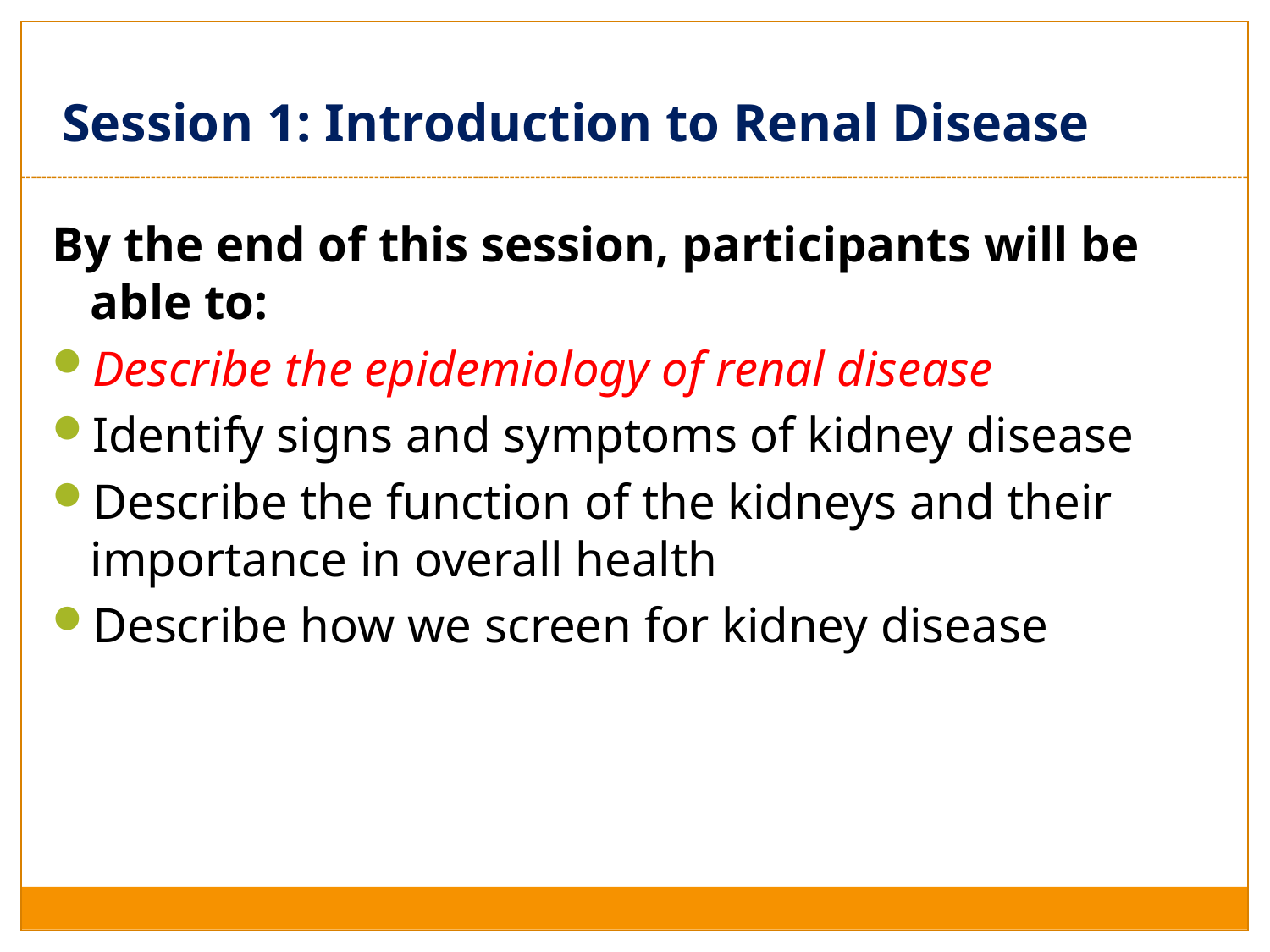

# Session 1: Introduction to Renal Disease
By the end of this session, participants will be able to:
Describe the epidemiology of renal disease
Identify signs and symptoms of kidney disease
Describe the function of the kidneys and their importance in overall health
Describe how we screen for kidney disease

## Slide 4
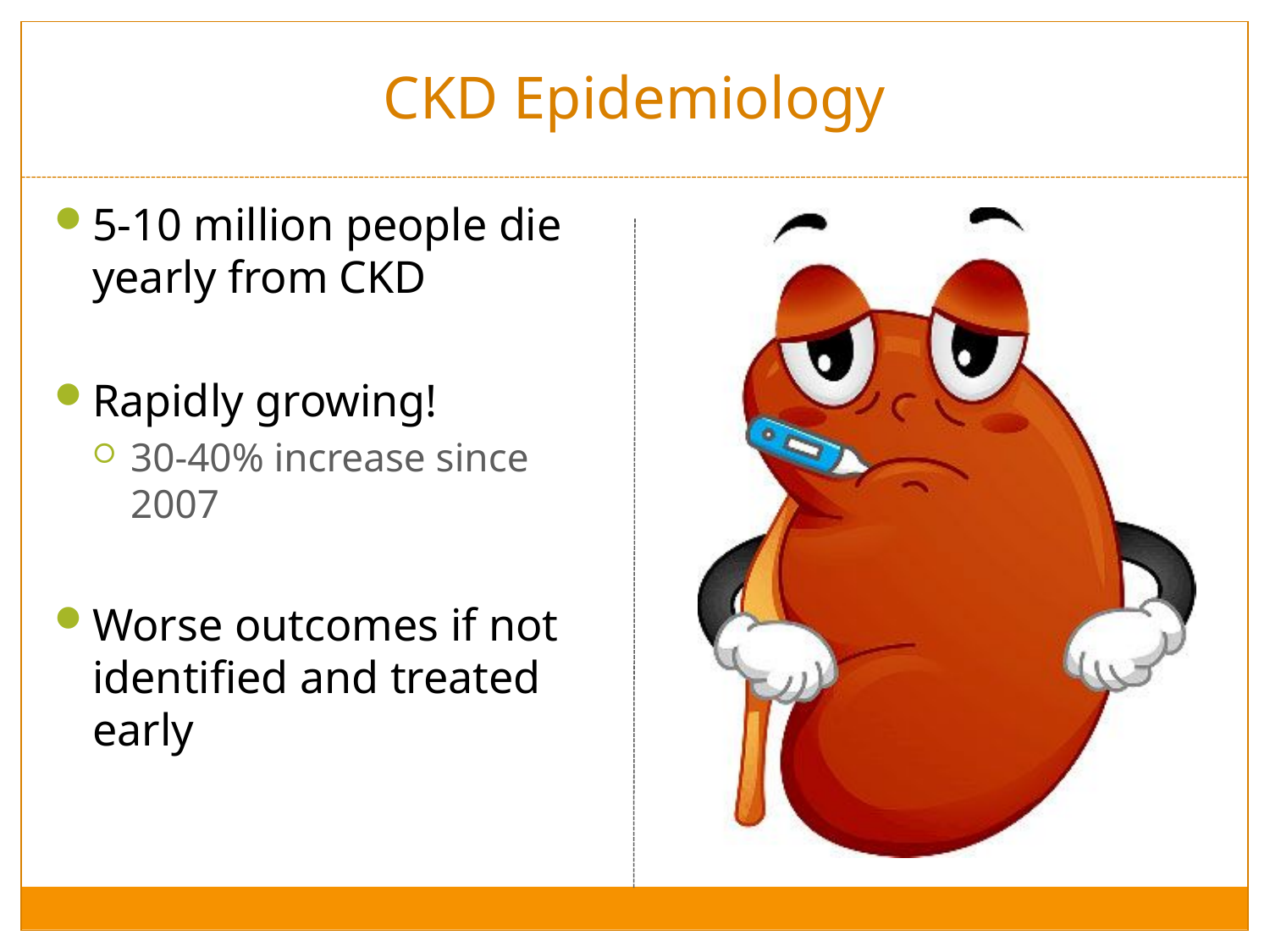

# CKD Epidemiology
5-10 million people die yearly from CKD
Rapidly growing!
30-40% increase since 2007
Worse outcomes if not identified and treated early

## Slide 5
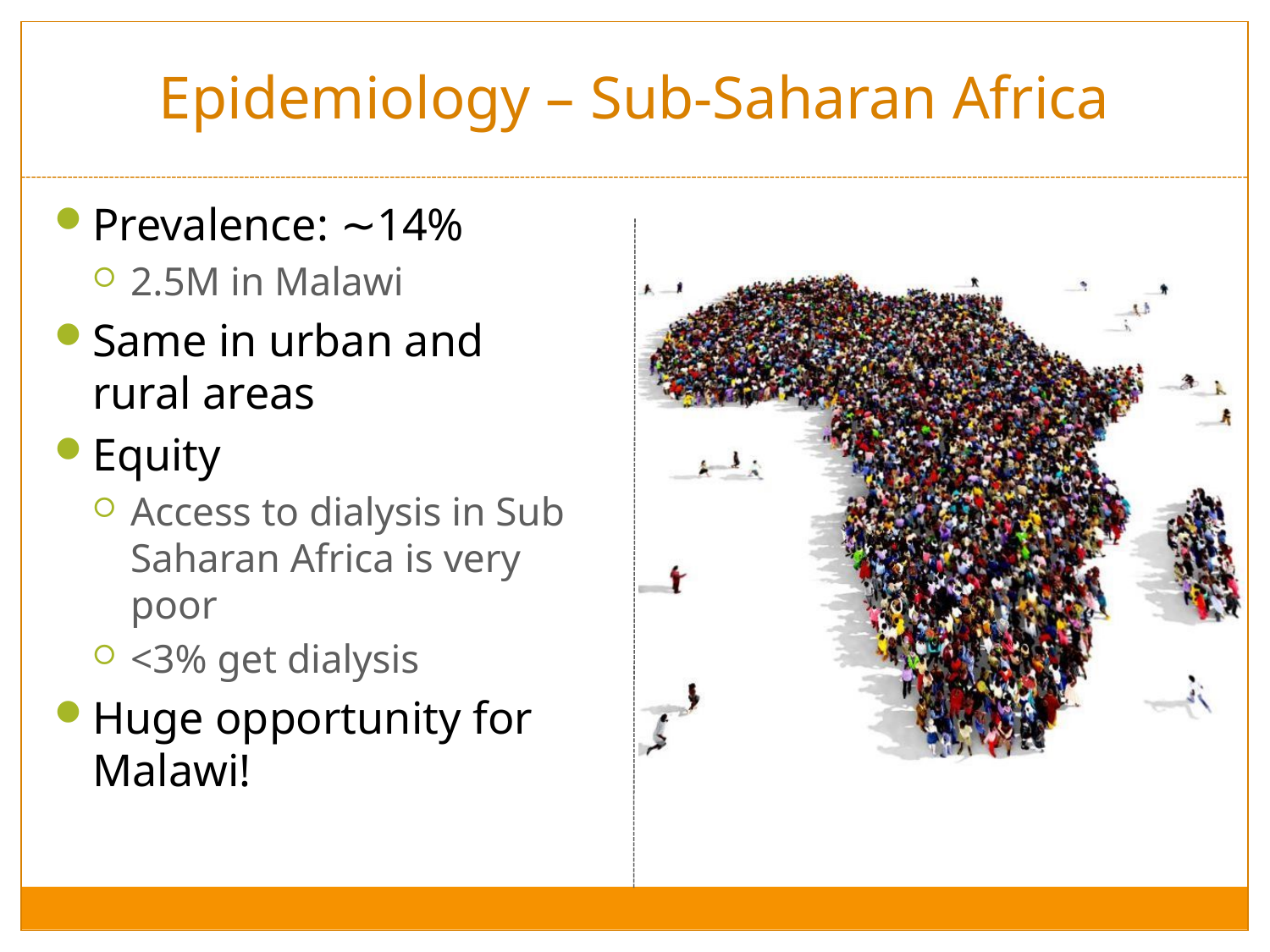

# Epidemiology – Sub-Saharan Africa
Prevalence: ∼14%
2.5M in Malawi
Same in urban and rural areas
Equity
Access to dialysis in Sub Saharan Africa is very poor
<3% get dialysis
Huge opportunity for Malawi!

## Slide 6
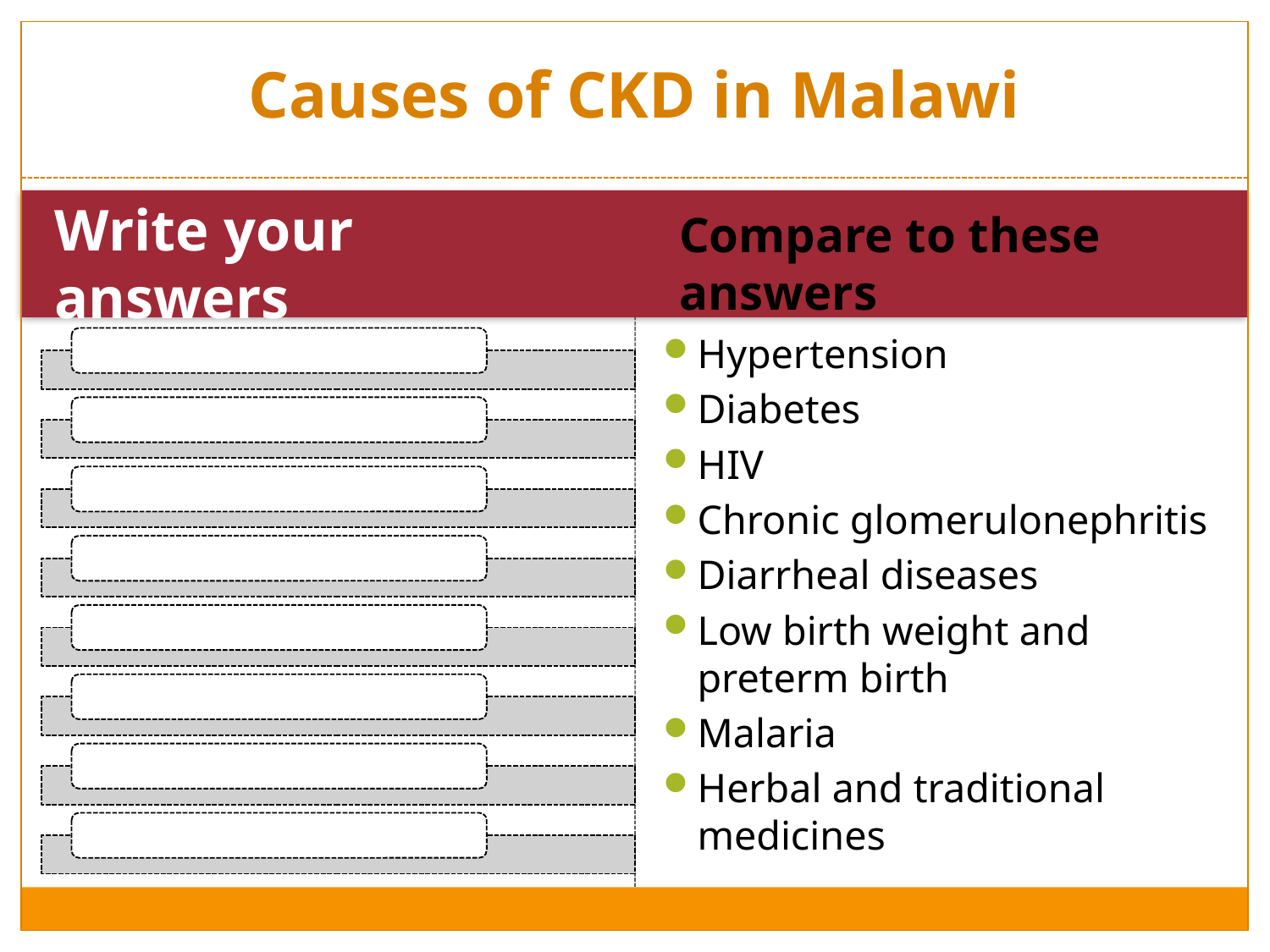

# Causes of CKD in Malawi
Write your answers
Compare to these answers
Hypertension
Diabetes
HIV
Chronic glomerulonephritis
Diarrheal diseases
Low birth weight and preterm birth
Malaria
Herbal and traditional medicines

## Slide 7
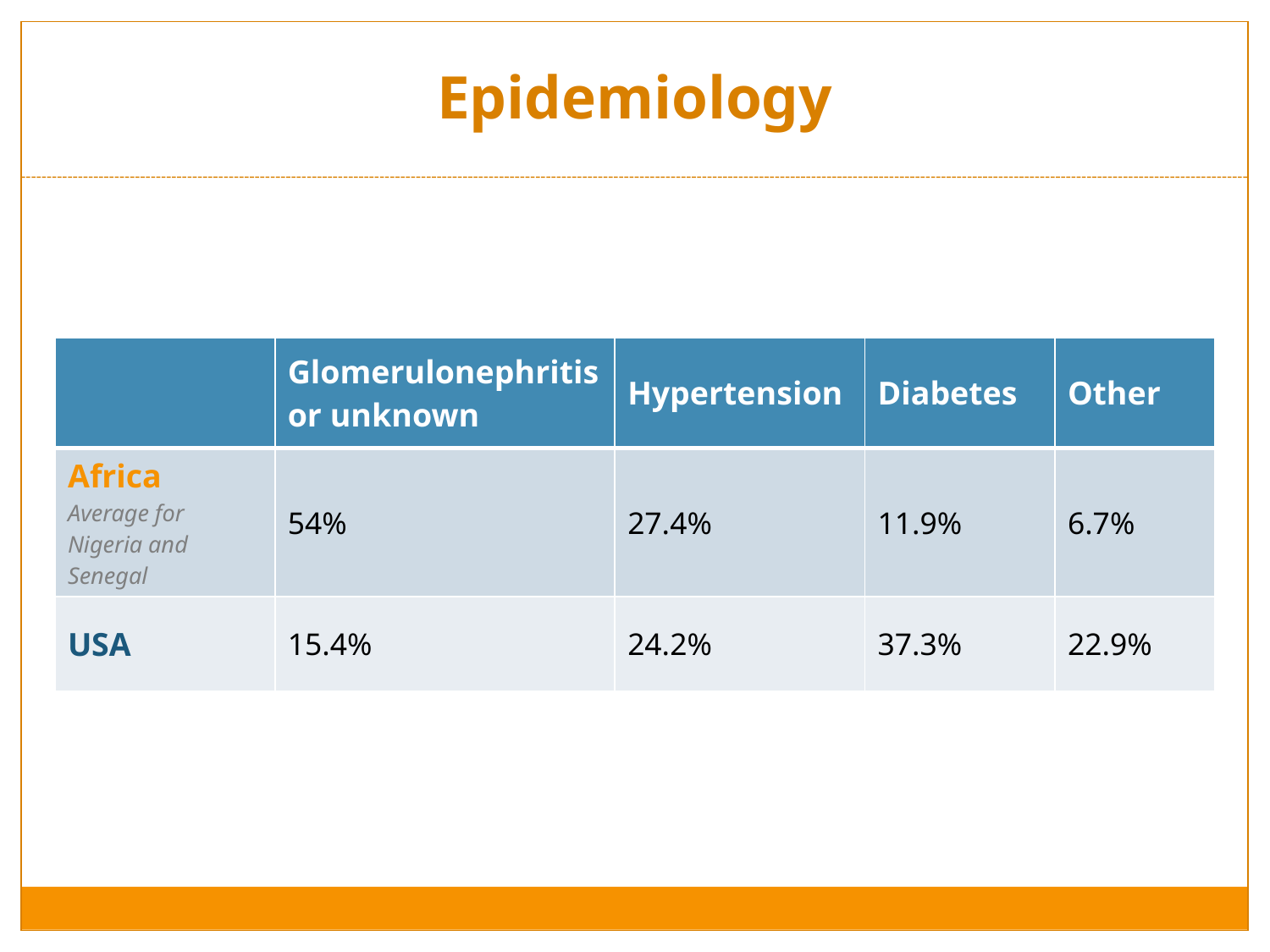

# Epidemiology
| | Glomerulonephritis or unknown | Hypertension | Diabetes | Other |
| --- | --- | --- | --- | --- |
| Africa Average for Nigeria and Senegal | 54% | 27.4% | 11.9% | 6.7% |
| USA | 15.4% | 24.2% | 37.3% | 22.9% |

## Slide 8
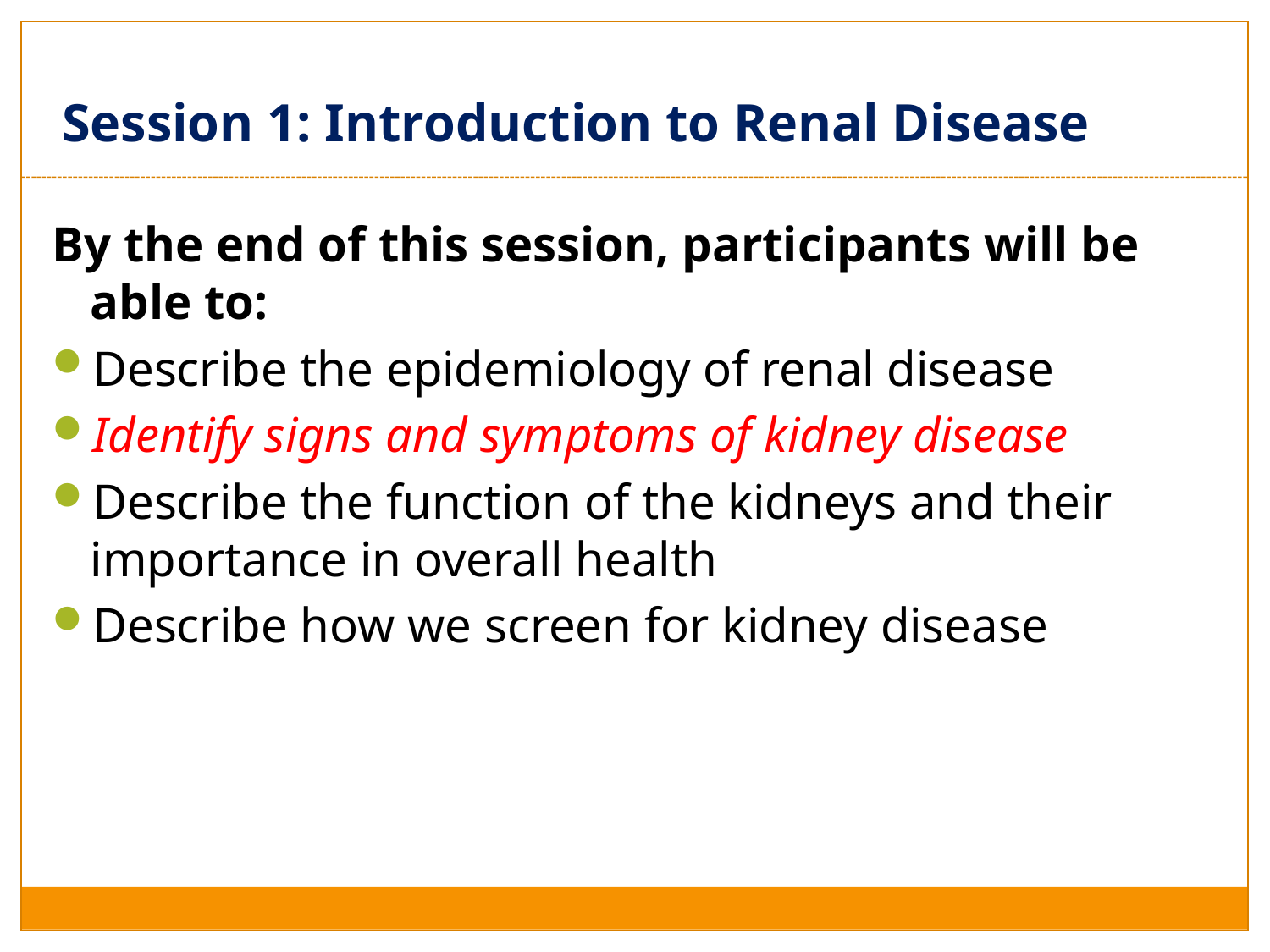

# Session 1: Introduction to Renal Disease
By the end of this session, participants will be able to:
Describe the epidemiology of renal disease
Identify signs and symptoms of kidney disease
Describe the function of the kidneys and their importance in overall health
Describe how we screen for kidney disease

## Slide 9
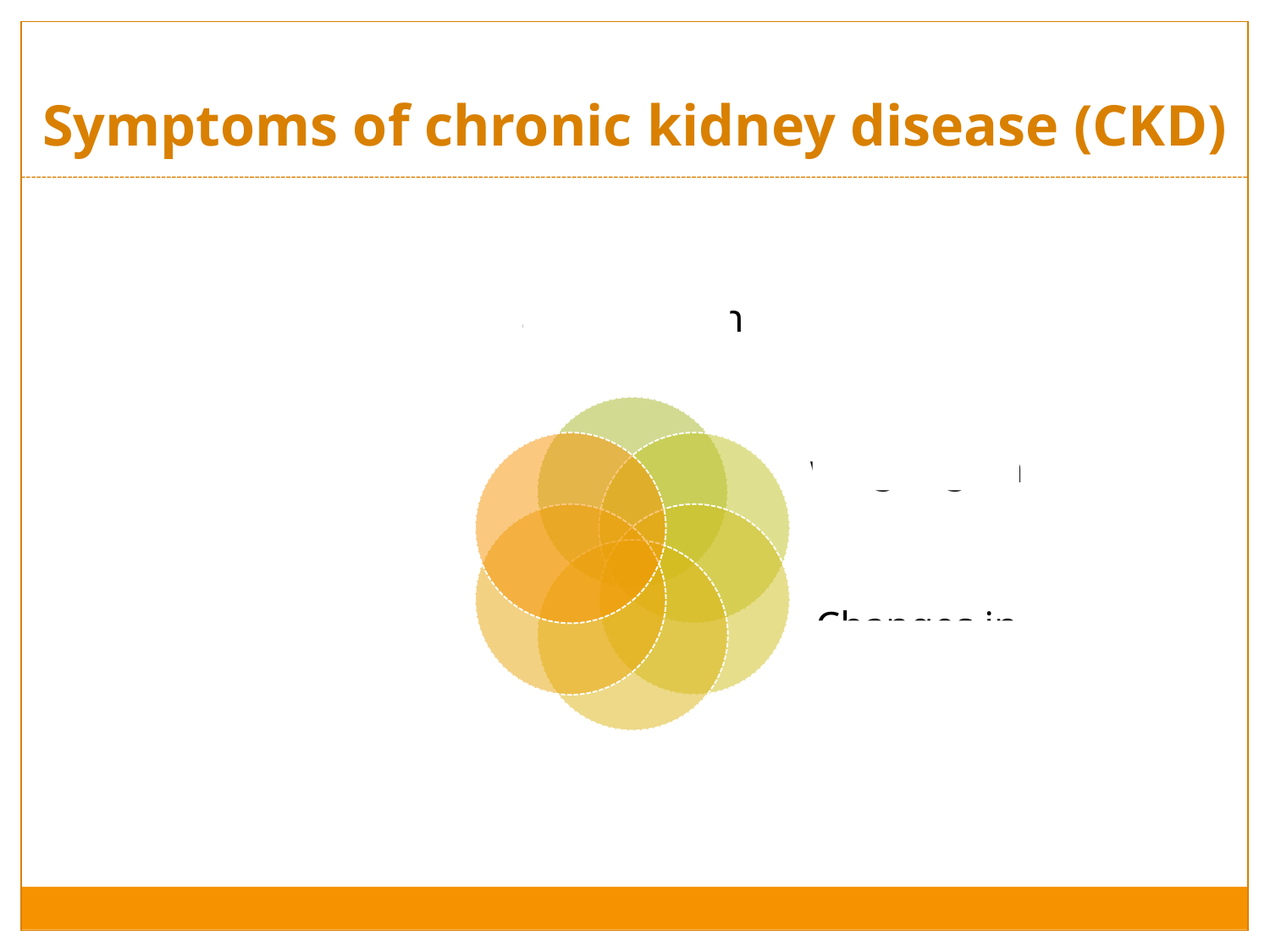

# Symptoms of chronic kidney disease (CKD)

## Slide 10
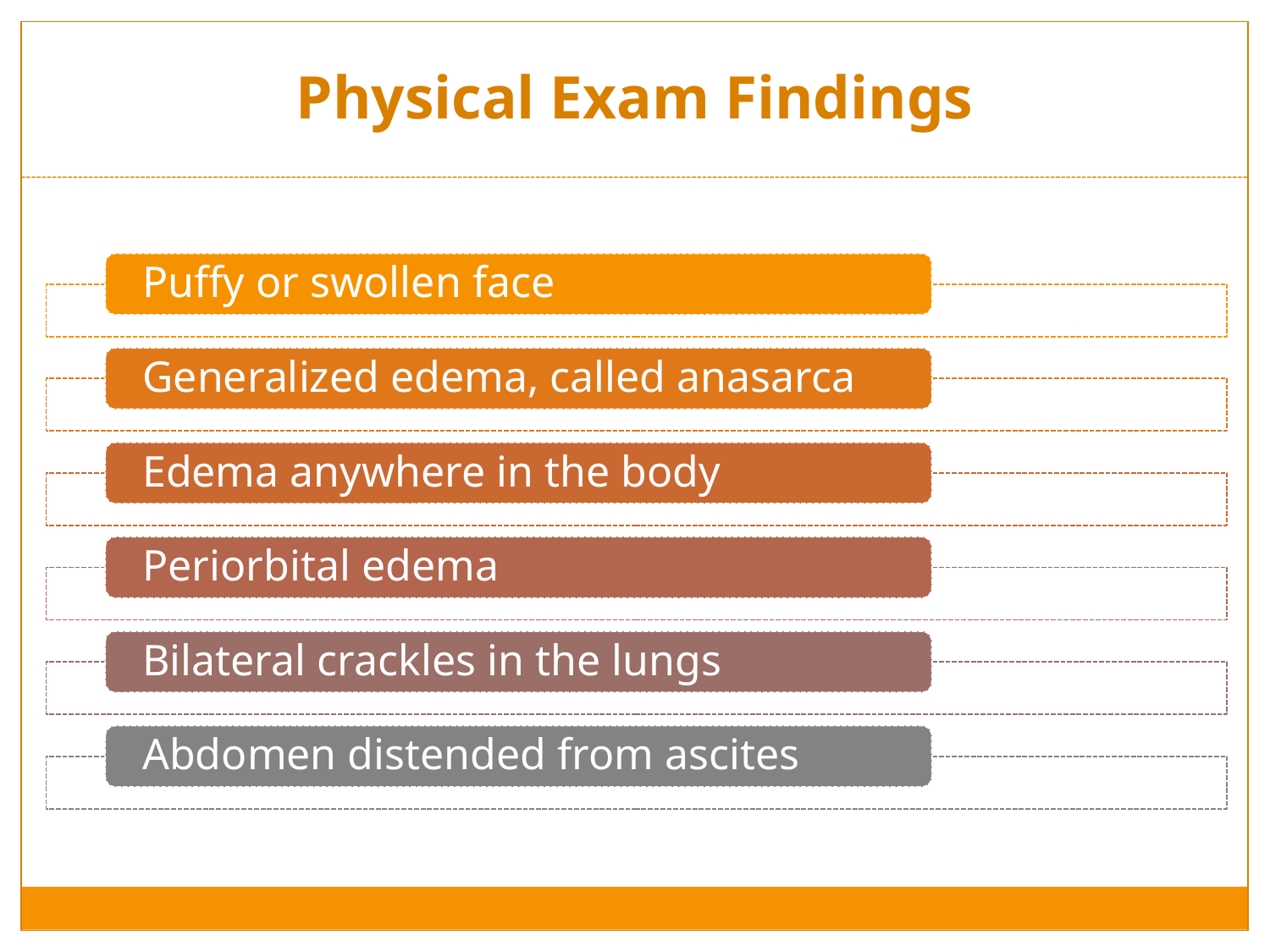

# Physical Exam Findings

## Slide 11
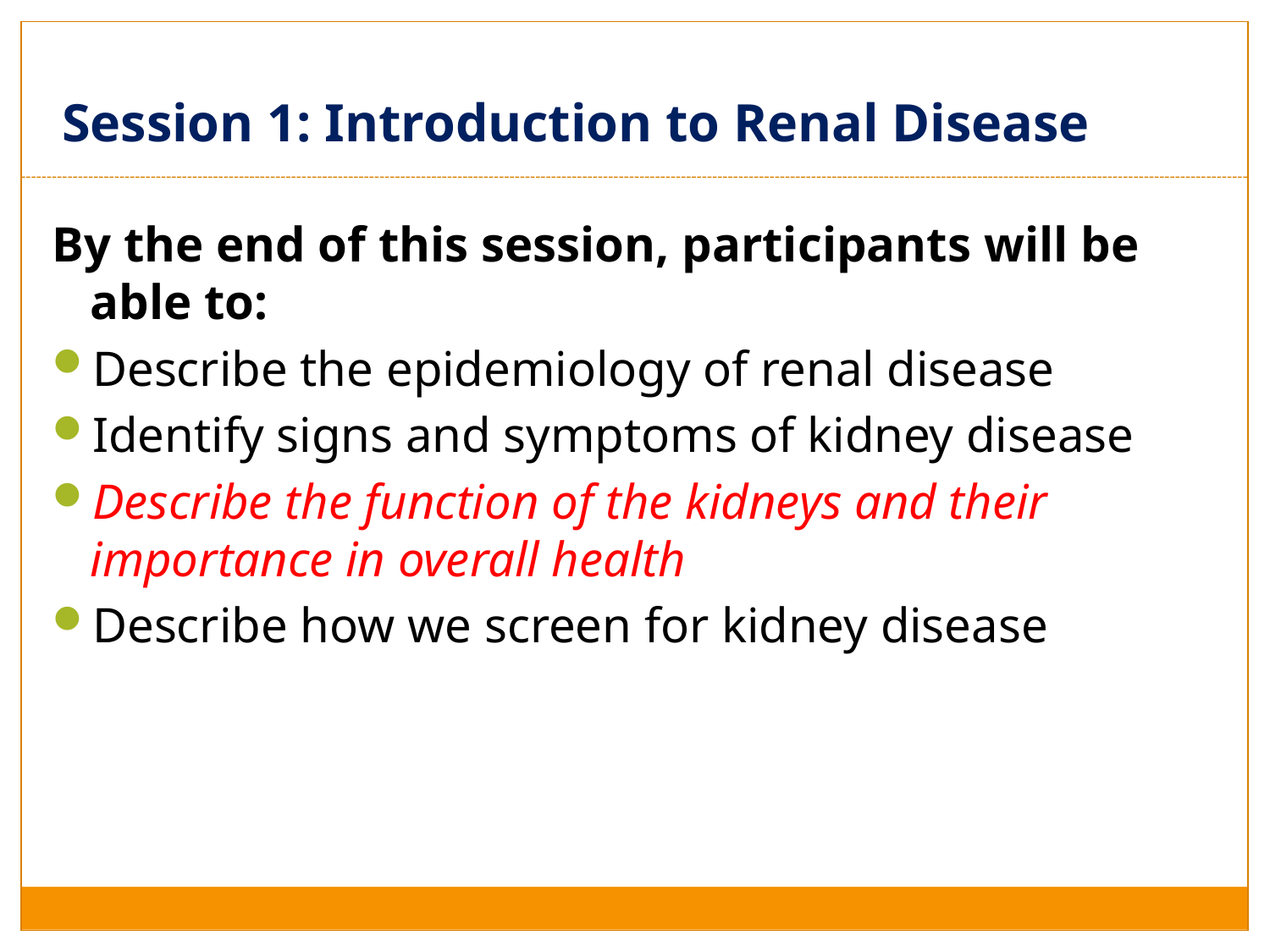

# Session 1: Introduction to Renal Disease
By the end of this session, participants will be able to:
Describe the epidemiology of renal disease
Identify signs and symptoms of kidney disease
Describe the function of the kidneys and their importance in overall health
Describe how we screen for kidney disease

## Slide 12
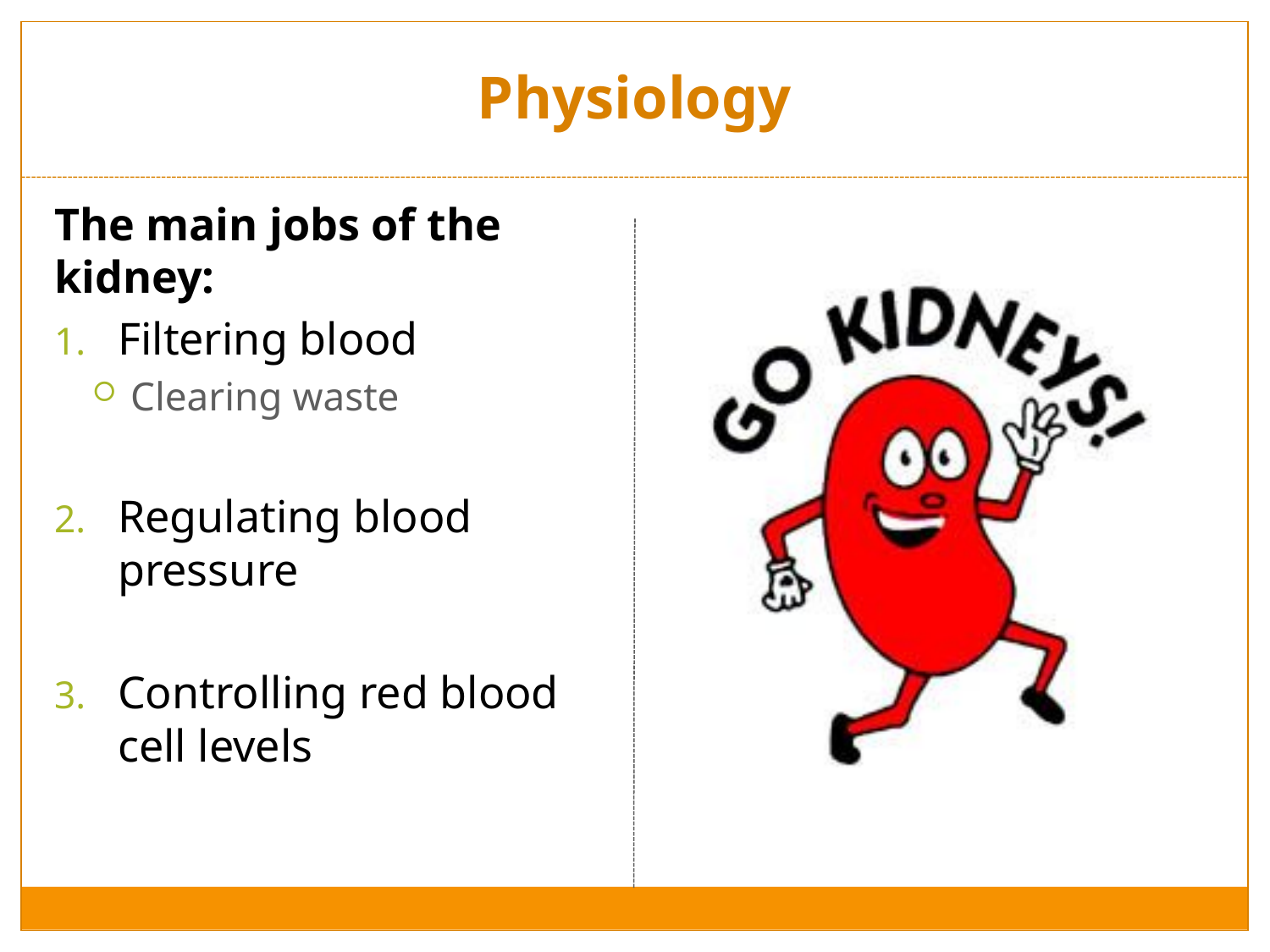

# Physiology
The main jobs of the kidney:
Filtering blood
Clearing waste
Regulating blood pressure
Controlling red blood cell levels

## Slide 13
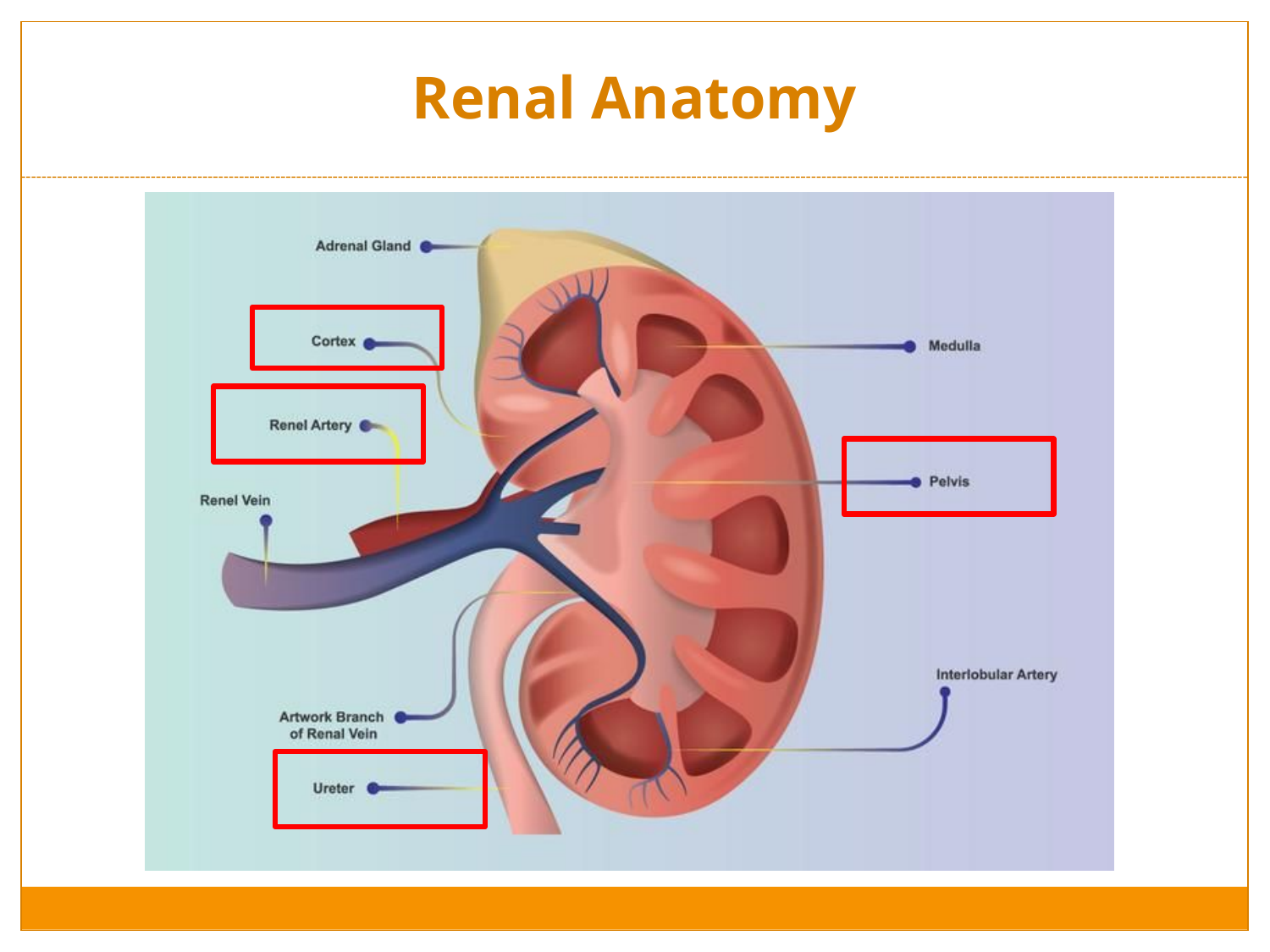

# Renal Anatomy

## Slide 14
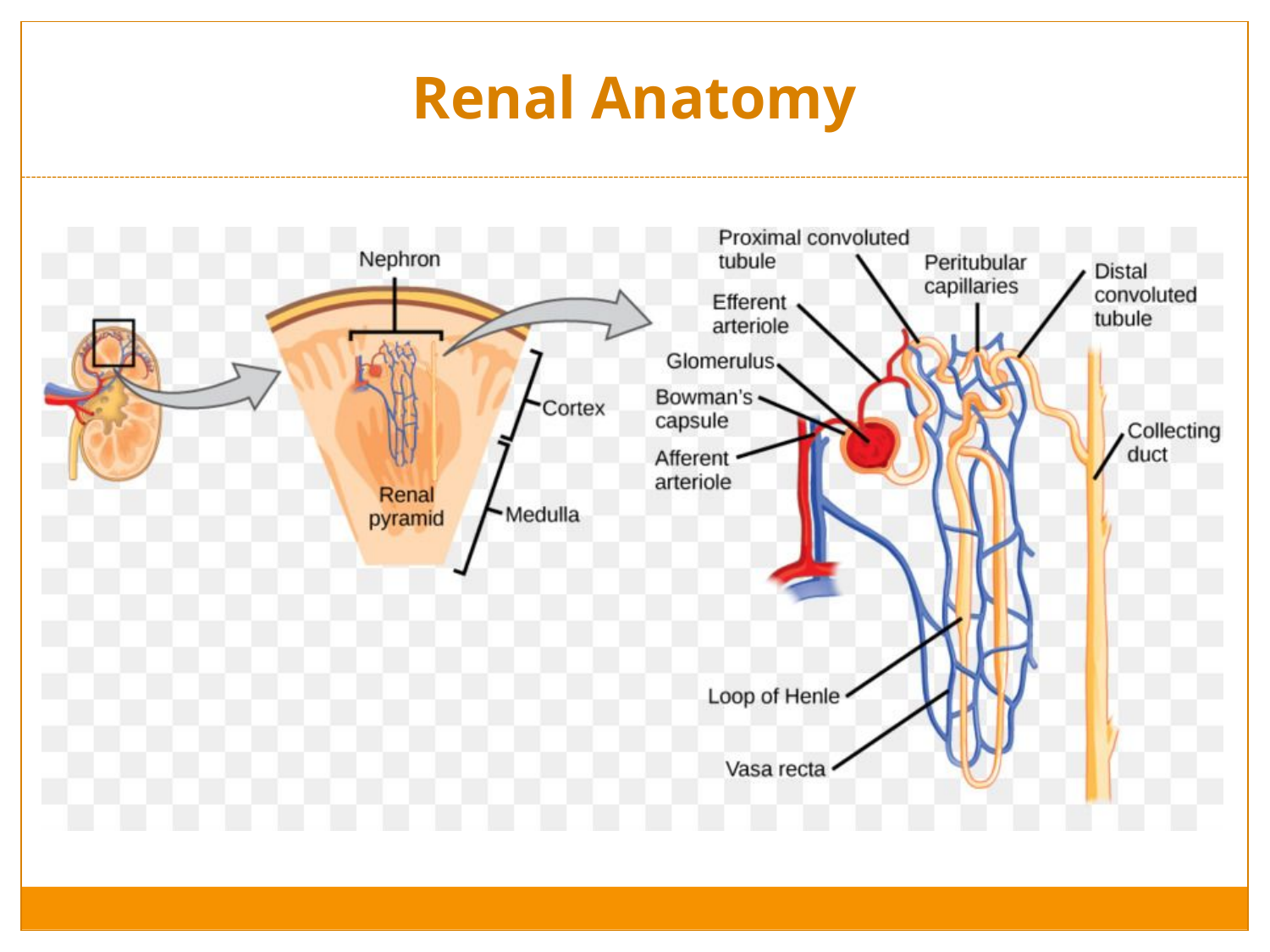

# Renal Anatomy

## Slide 15
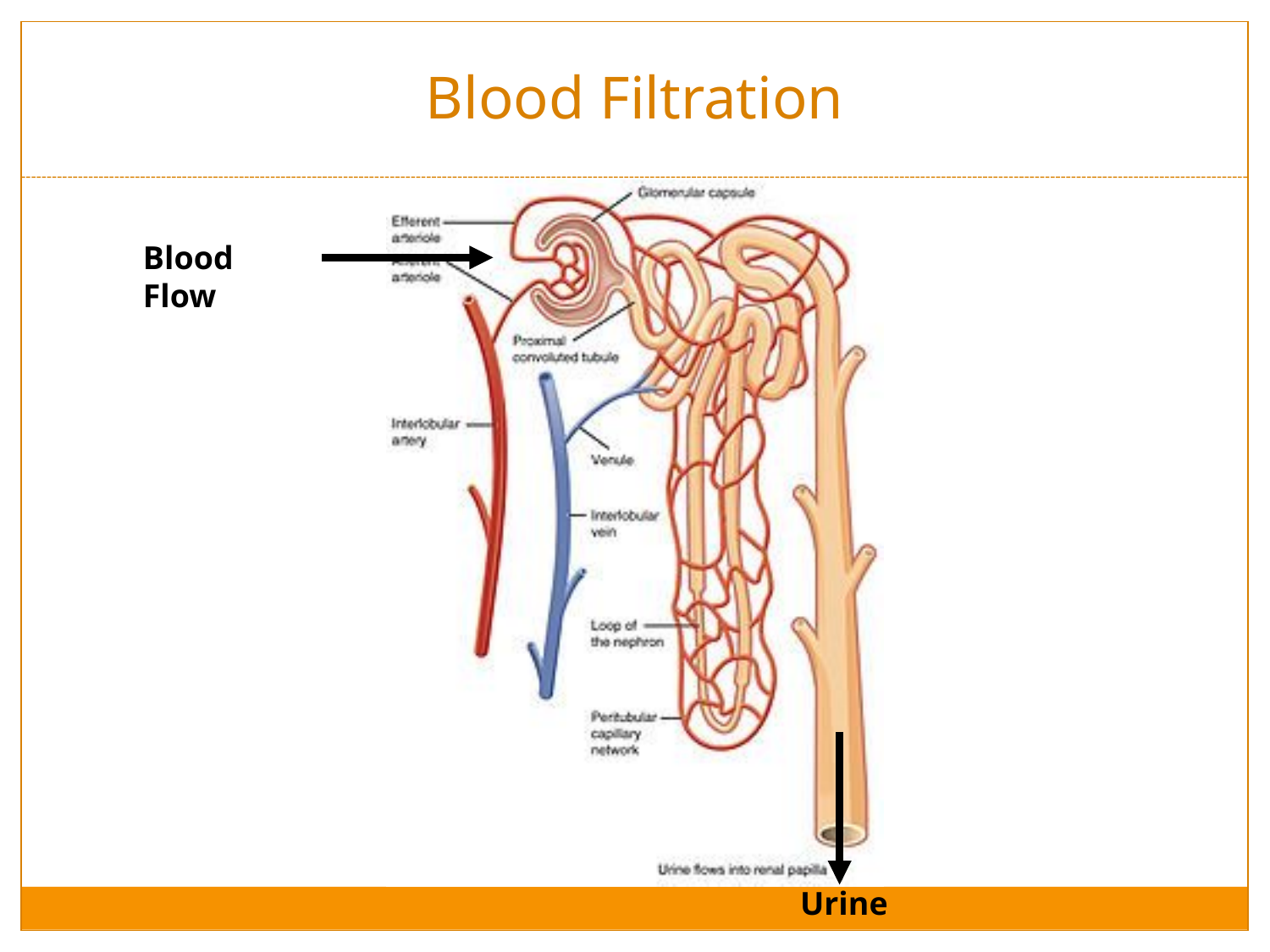

# Blood Filtration
Blood Flow
Urine

## Slide 16
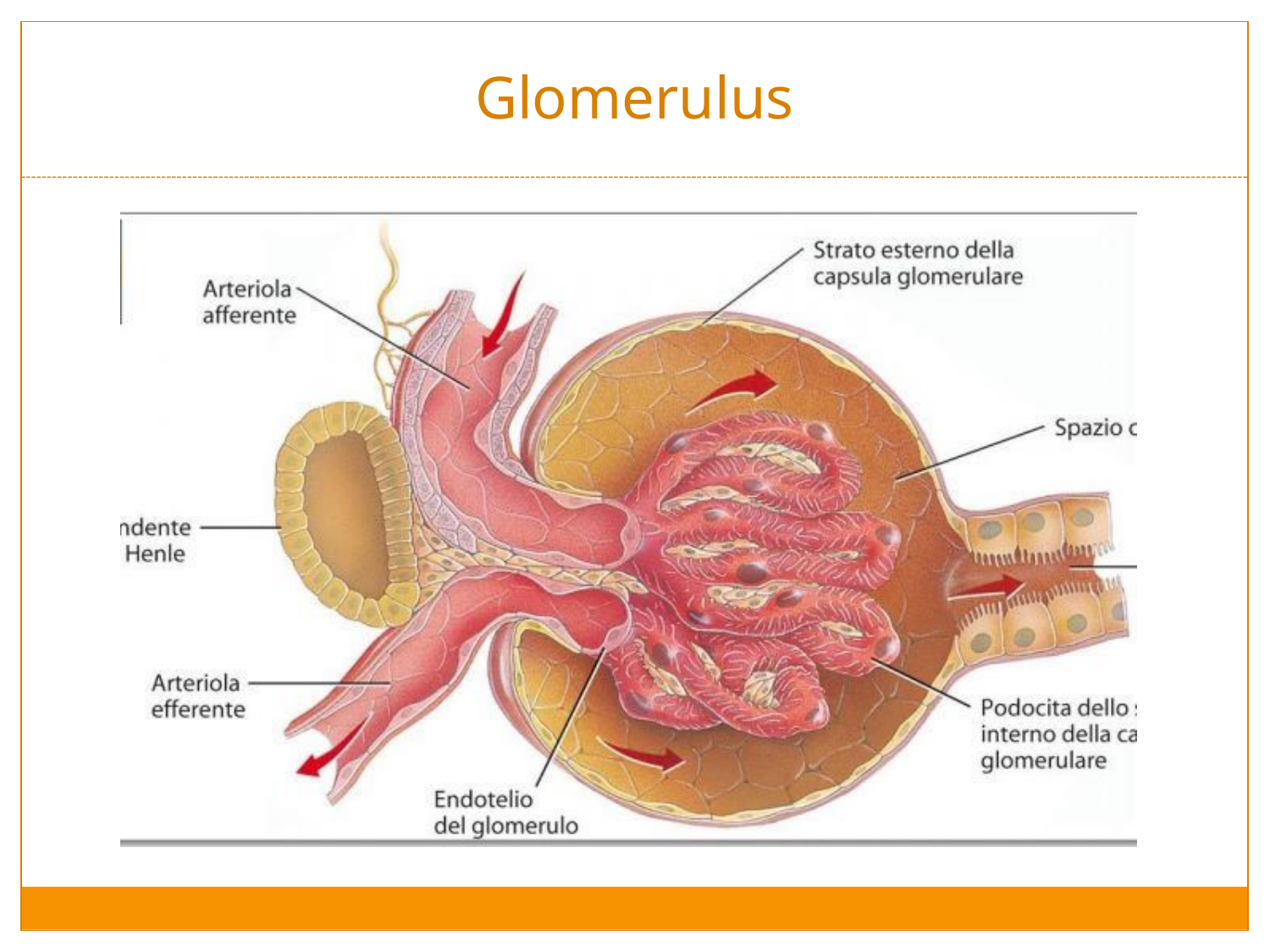

# Glomerulus

## Slide 17
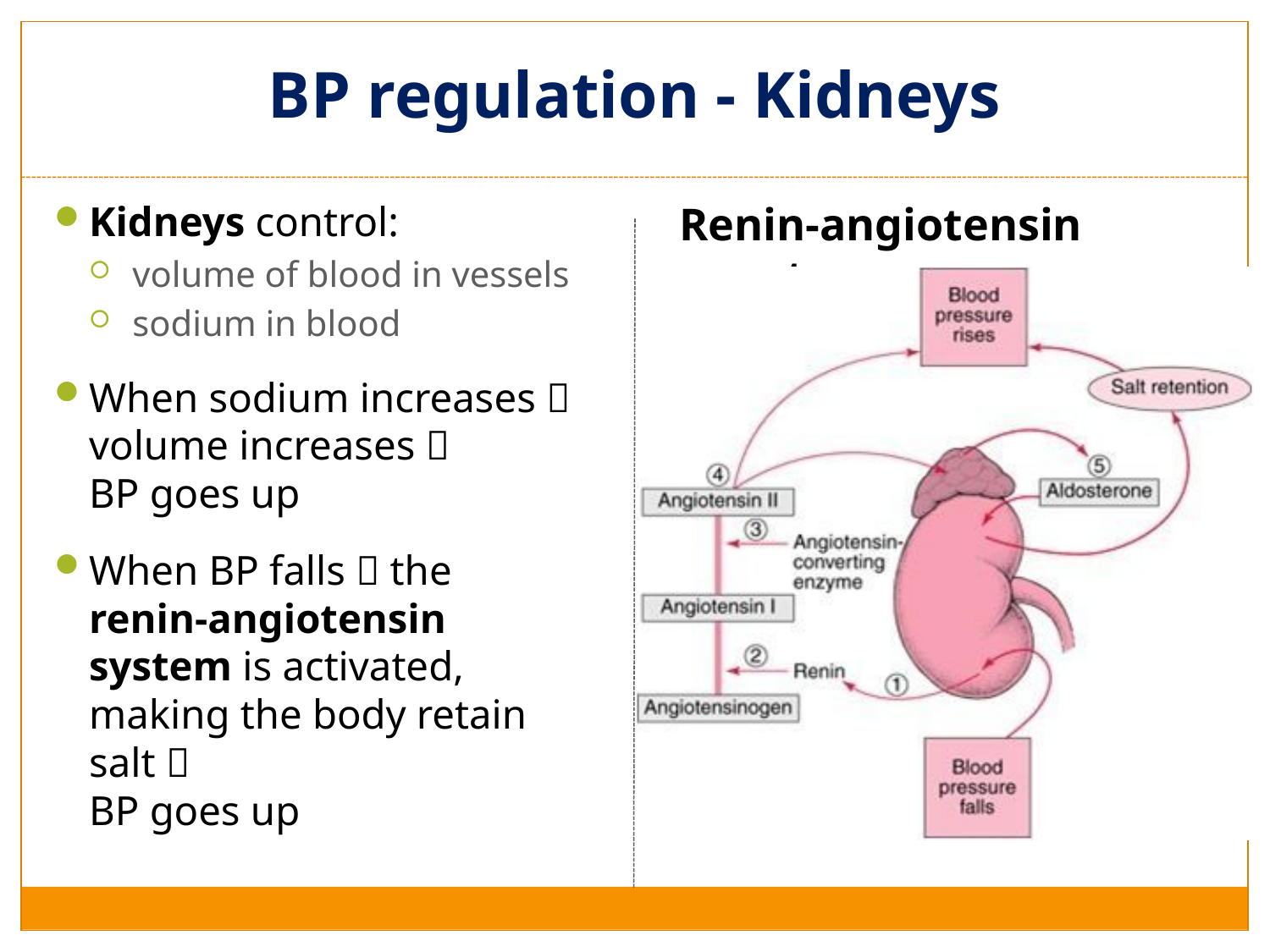

# BP regulation - Kidneys
Kidneys control:
volume of blood in vessels
sodium in blood
When sodium increases  volume increases  BP goes up
When BP falls  the
renin-angiotensin system is activated, making the body retain salt 
BP goes up
Renin-angiotensin system

## Slide 18
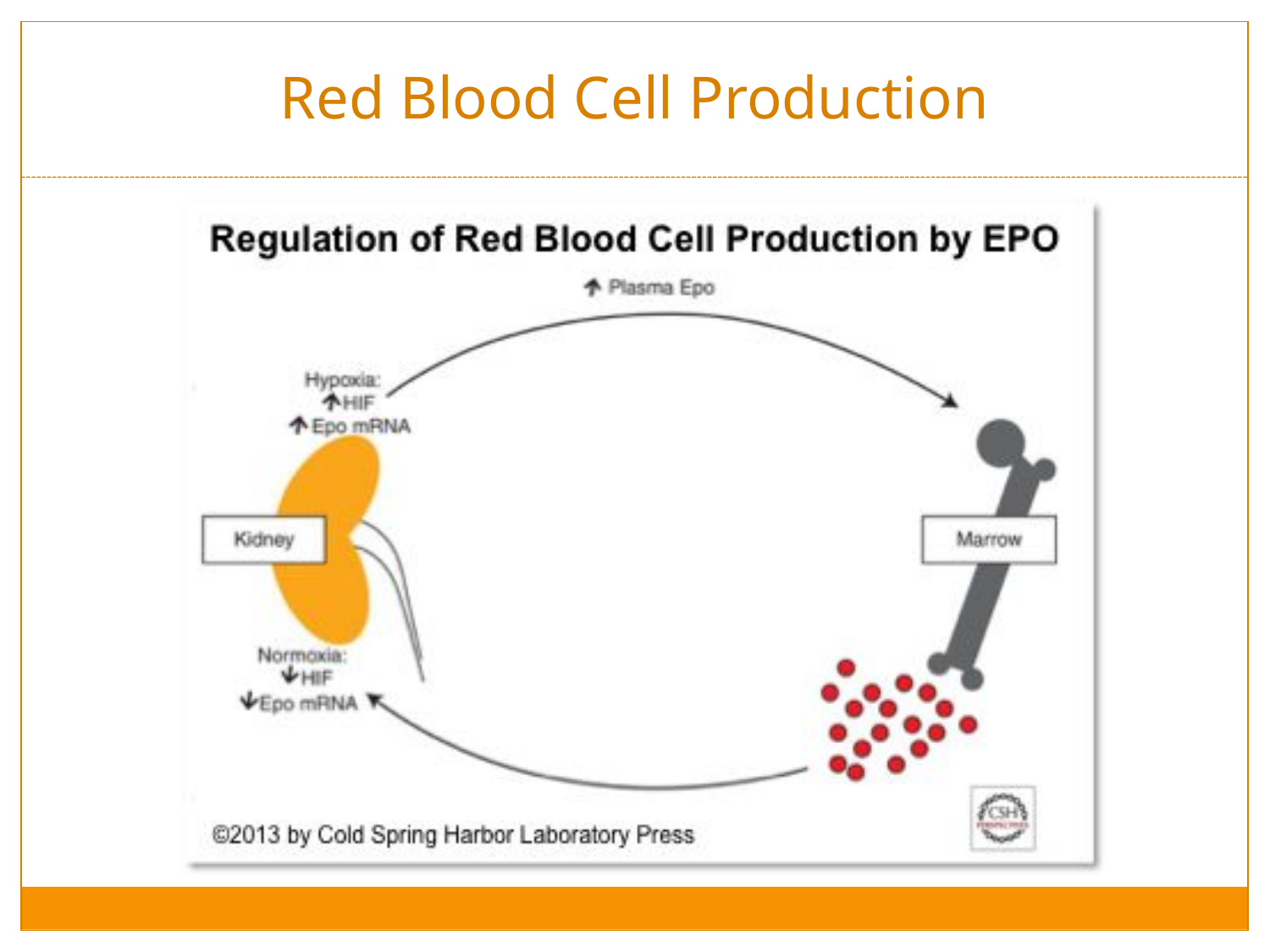

# Red Blood Cell Production

## Slide 19
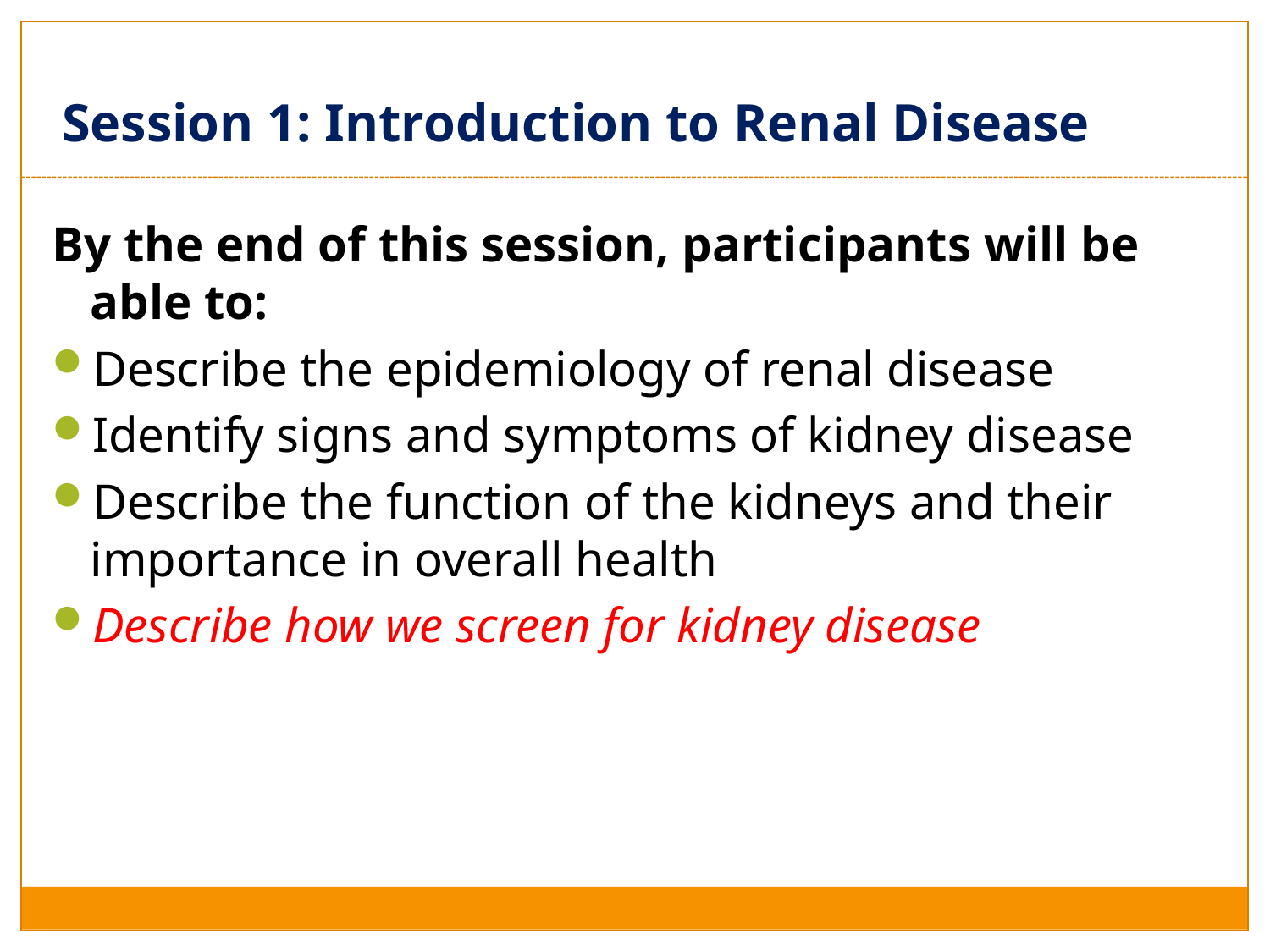

# Session 1: Introduction to Renal Disease
By the end of this session, participants will be able to:
Describe the epidemiology of renal disease
Identify signs and symptoms of kidney disease
Describe the function of the kidneys and their importance in overall health
Describe how we screen for kidney disease

## Slide 20
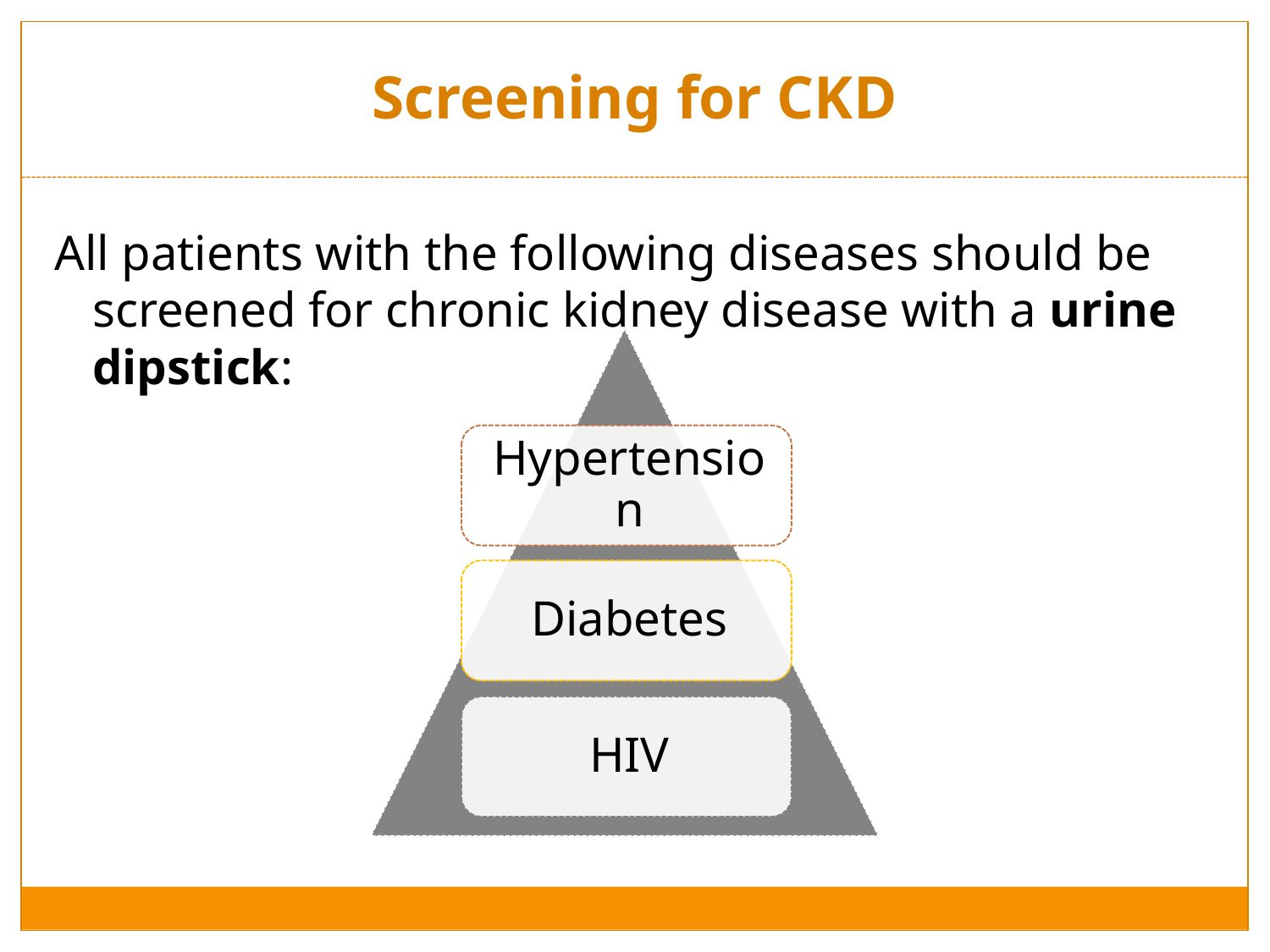

# Screening for CKD
All patients with the following diseases should be screened for chronic kidney disease with a urine dipstick:

## Slide 21
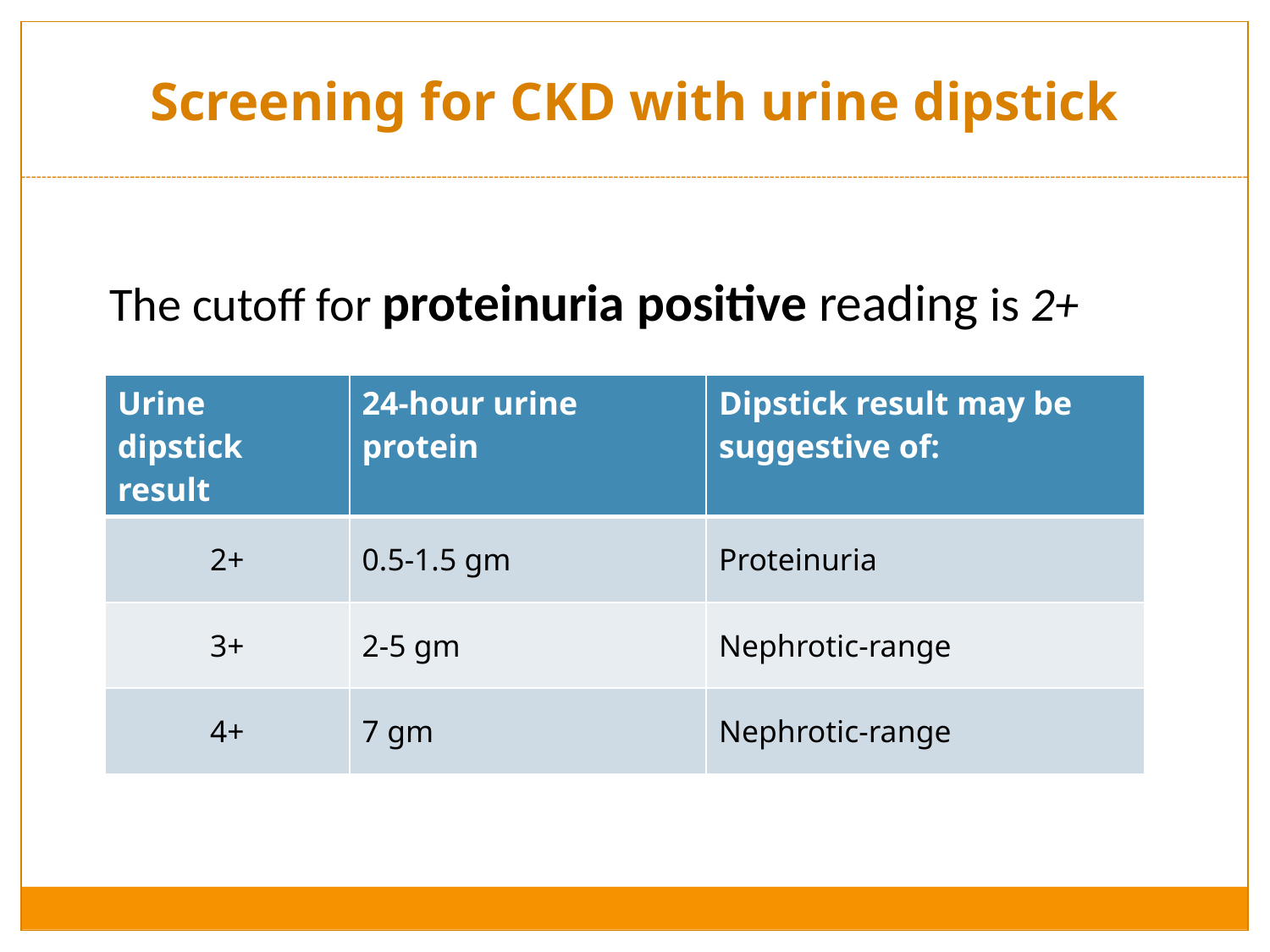

# Screening for CKD with urine dipstick
The cutoff for proteinuria positive reading is 2+
| Urine dipstick result | 24-hour urine protein | Dipstick result may be suggestive of: |
| --- | --- | --- |
| 2+ | 0.5-1.5 gm | Proteinuria |
| 3+ | 2-5 gm | Nephrotic-range |
| 4+ | 7 gm | Nephrotic-range |

## Slide 22
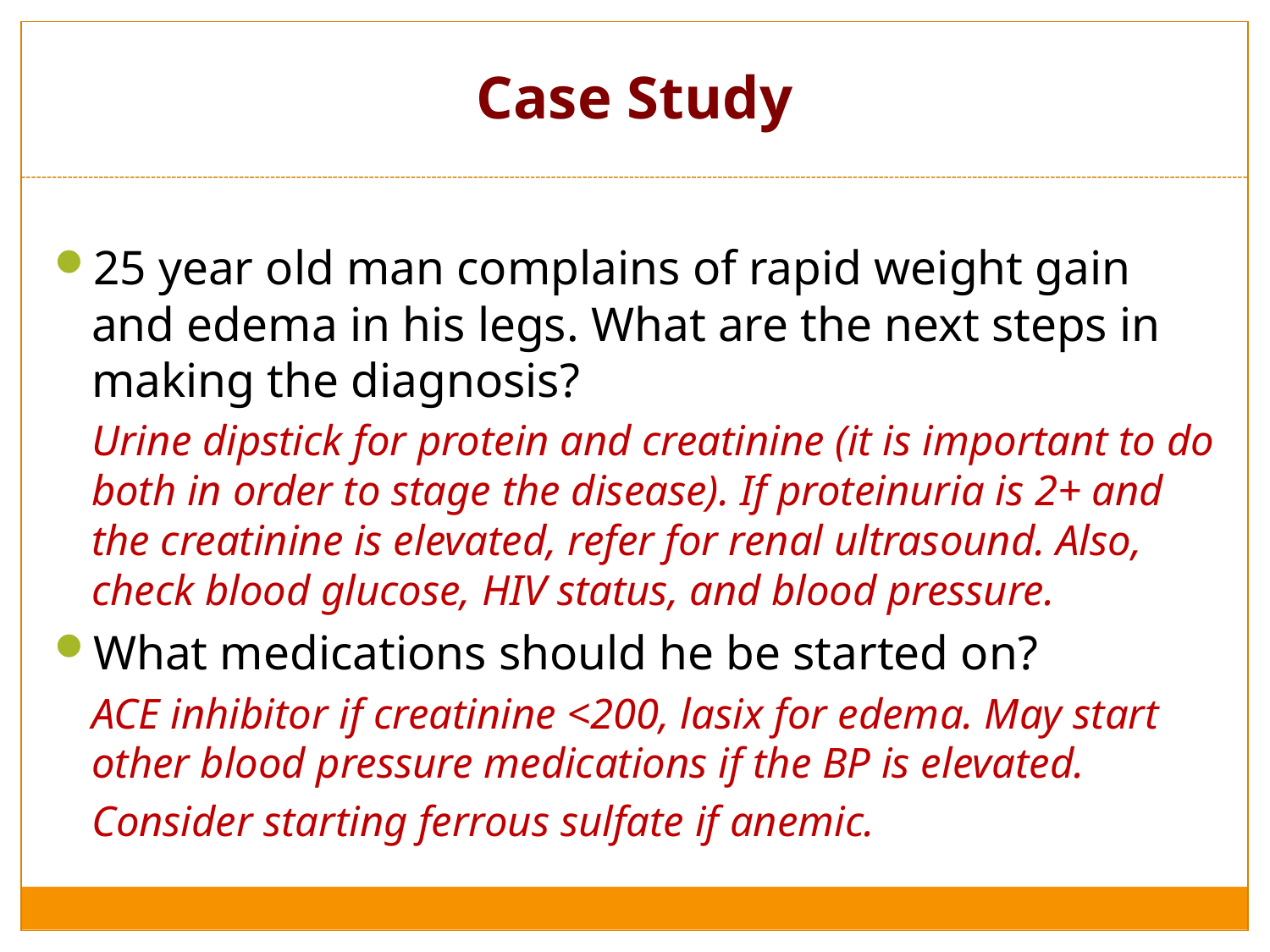

# Case Study
25 year old man complains of rapid weight gain and edema in his legs. What are the next steps in making the diagnosis?
Urine dipstick for protein and creatinine (it is important to do both in order to stage the disease). If proteinuria is 2+ and the creatinine is elevated, refer for renal ultrasound. Also, check blood glucose, HIV status, and blood pressure.
What medications should he be started on?
ACE inhibitor if creatinine <200, lasix for edema. May start other blood pressure medications if the BP is elevated.
Consider starting ferrous sulfate if anemic.

## Slide 23
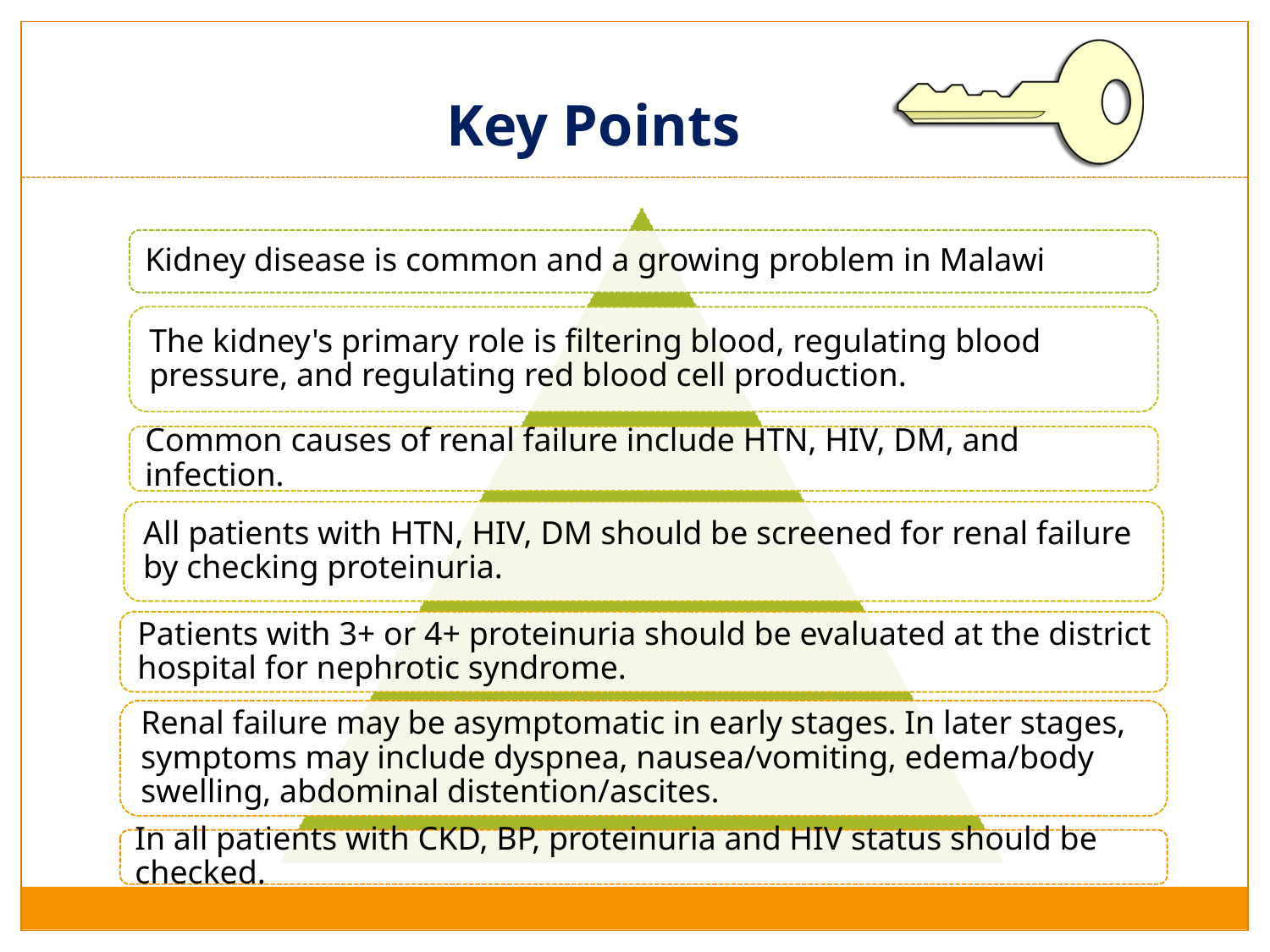

# Key Points

## Slide 24
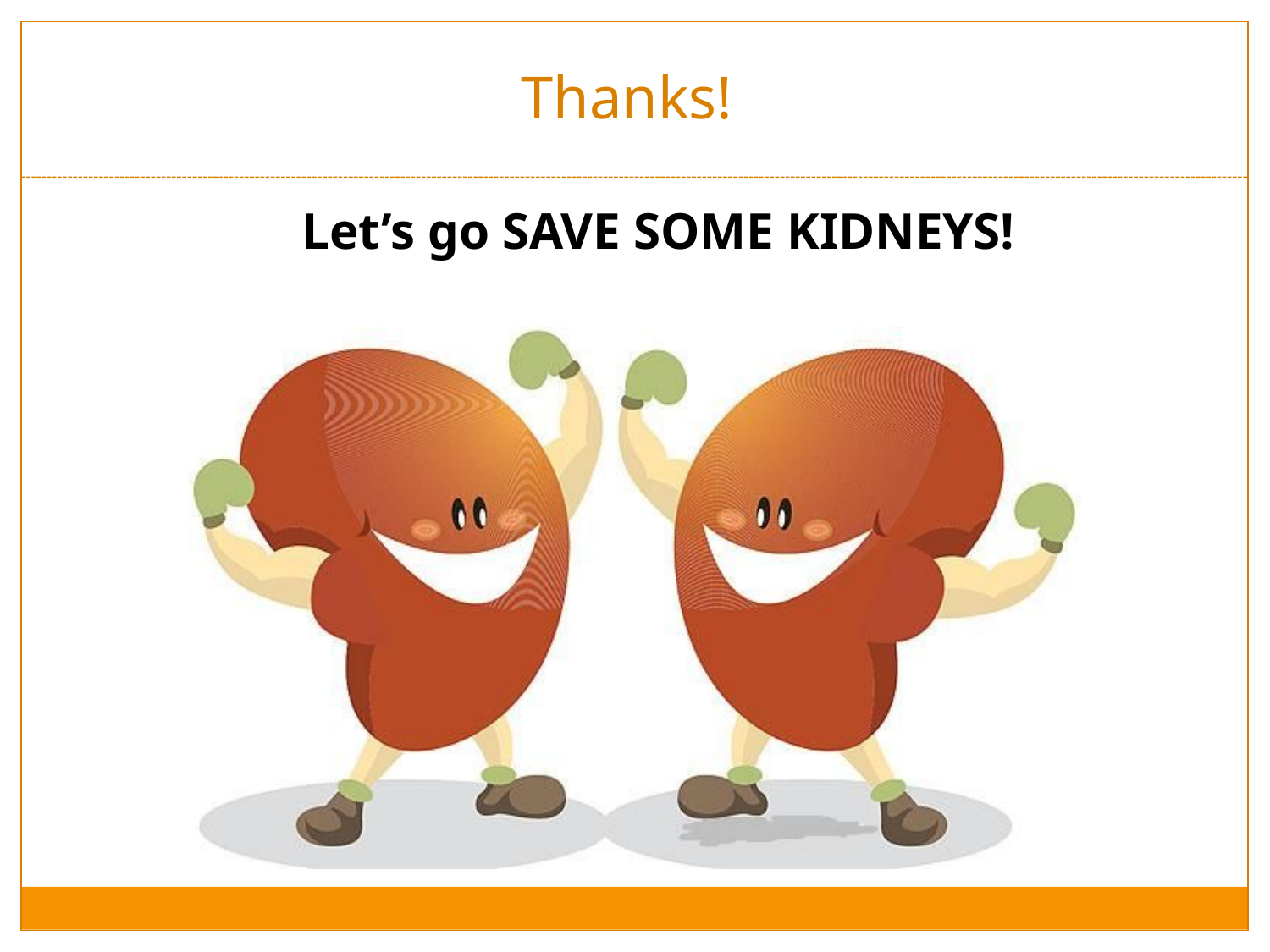

# Thanks!
Let’s go SAVE SOME KIDNEYS!
